# Venomics of Remipede Crustaceans Reveals Novel Peptide Diversity and Illuminates the Venom’s Biological Role

**DOI:** 10.3390/toxins9080234

**Published:** 2017-07-26

**Authors:** Björn M. von Reumont, Eivind A. B. Undheim, Robin-Tobias Jauss, Ronald A. Jenner

**Affiliations:** 1 Molecular Evolution and Systematics of Animals, Institute for Biology, University of Leipzig, Leipzig 04103, Germany; RJauss@gmx.net; 2 Department of Life Sciences, Natural History Museum, Cromwell Road, London SW7 5BD, UK; 3 Centre for Advanced Imaging, University of Queensland, St. Lucia 4072, QLD, Australia

**Keywords:** venomics, Remipedia, crustaceans, ICK, venom, arthropods, anchialine caves

## Abstract

We report the first integrated proteomic and transcriptomic investigation of a crustacean venom. Remipede crustaceans are the venomous sister group of hexapods, and the venom glands of the remipede *Xibalbanus tulumensis* express a considerably more complex cocktail of proteins and peptides than previously thought. We identified 32 venom protein families, including 13 novel peptide families that we name xibalbins, four of which lack similarities to any known structural class. Our proteomic data confirm the presence in the venom of 19 of the 32 families. The most highly expressed venom components are serine peptidases, chitinase and six of the xibalbins. The xibalbins represent Inhibitory Cystine Knot peptides (ICK), a double ICK peptide, peptides with a putative Cystine-stabilized α-helix/β-sheet motif, a peptide similar to hairpin-like β-sheet forming antimicrobial peptides, two peptides related to different hormone families, and four peptides with unique structural motifs. Remipede venom components represent the full range of evolutionary recruitment frequencies, from families that have been recruited into many animal venoms (serine peptidases, ICKs), to those having a very narrow taxonomic range (double ICKs), to those unique for remipedes. We discuss the most highly expressed venom components to shed light on their possible functional significance in the predatory and defensive use of remipede venom, and to provide testable ideas for any future bioactivity studies.

## 1. Introduction

Remipedes are superficially centipede-like predatory crustaceans that exclusively inhabit marine cave systems [1]. There are currently 29 described species [2], and their first description in 1981 [3] rekindled the interest of zoologists to attempt to reconstruct the evolutionary origin of crustaceans. Carcinologists were quick to interpret remipedes’ long segmented bodies, their lack of segment specialization, their lack of a carapace, and their serially homonomous, biramous, and paddle-like trunk limbs as evidence that they could well be the earliest diverging lineage of extant crustaceans with the most primitive body plan [4,5]. Since then molecular evidence has painted a rather different picture. Phylogenomic analyses suggest that remipedes are nested deeply within pancrustaceans (the clade comprising insects and crustaceans), as the living sister group of insects [6,7,8,9]. This has drawn remipedes into debates about the origin of the most successful group of animals on Earth [10], but it also diminishes their relevance for understanding early crustacean evolution. At the same time the molecular evidence refocuses attention on the remipedes’ most strikingly unique trait: their venom system.

The vast majority of venomous species on Earth are arthropods. Three of the four main groups of arthropods—chelicerates, myriapods and insects—together represent more than 150,000 venomous species, and there are venomic studies for multiple species within each of these groups. In sharp contrast, although putatively venomous crustaceans exist, in particular several parasitic species of copepods, malacostracans and branchiurans [11], the venom system of only a single crustacean species has been the subject of a transcriptomic study [12]. This showed that the remipede *Xibalbanus tulumensis* (previously *Speleonectes tulumensis*) [13] is the first known venomous predatory crustacean. Remipede biology and the environment in which they live provide some clues as to why remipedes have evolved venom.

With the exception of two species that occur in fully marine, sub-seafloor caves [2], remipedes are known only from anchialine caves, which are subterranean caves that merge with the sea at the coast and groundwater inland. They contain a layer of fresh or brackish water that overlays a layer of seawater separated by a halocline. Remipedes live exclusively in the oxygen poor salt water zones of these caves, where prey abundance is low. Remipedes lack eyes, but have well-developed olfactory pathways [14], and they rely on olfactory cues to navigate their environment. They are active predators of cave crustaceans, but are reported as normally being relatively slow swimmers that are likely to be outmanoeuvred by potential prey [15,16]. Under these conditions it is advantageous to possess venom that can rapidly debilitate prey. Remipedes may therefore have evolved a venom system concomitant with a move into the anchialine environment, and the adoption, or elaboration, of a predatory lifestyle that primarily targets other crustaceans that inhabit the same cave systems.

In our previous study of *X. tulumensis* we discovered that its venom apparatus is morphologically more complex than expected, and can function as a sophisticated venom injection mechanism [12]. Their venom glands express a cocktail of transcripts that code for homologues of known venom toxins in other taxa, with enzymes being the dominant component. The transcripts that are the most highly expressed as well as the most diverse code for serine peptidases of the S1 family and chitinases. The most highly expressed transcripts for non-enzymatic proteins code for cysteine-rich peptides with a putative inhibitor cystine knot (ICK) motif, with sequence similarities to spider β/δ agatoxins.

The striking differences between this transcriptomic profile and the venom composition of other venomous arthropophagous arthropods are intriguing. The venoms of predatory arthropods, such as centipedes, scorpions, spiders, as well as several hymenopterans, are typically rich in peptides [17,18,19,20,21,22,23,24], with larger proteins and enzymes being less abundant. Most of these peptides are thought to have a role in subduing prey, and many act as paralytic neurotoxins. The dearth of transcripts coding for peptides in the venom glands of *X. tulumensis* was therefore surprising. This apparent difference in venom composition could be real, and reflect differences in the evolutionary history and biology of the different venoms, but it could also be an artefact resulting from the methodological strategy of our original study. Bioinformatically identifying peptides in de novo assembled transcriptome data from an unstudied organism in the absence of proteomic data is likely to underestimate the true diversity of venom peptides. Moreover, our transcriptome data were generated on the 454 FLX platform sequencing technology, which does not provide the greatest possible sequencing depth.

To test if this apparent uniqueness of remipede venom composition is real or due to inevitable methodological limitations of our initial study we: (1) conducted a proteomic analysis of the venom of the remipede *X. tulumensis*; (2) produced a new transcriptome for the venom glands of *X. tulumensis* using Illumina MiSeq technology based on the same RNA material used for our previous 454 FLX analysis; and (3) re-sequenced the whole animal transcriptome library of *X. tulumensis* with Illumina HiSeq technology.

## 2. Results and Discussion

### 2.1. The Effect of Transcriptome Sequencing Platforms and Assembly Strategies 

#### 2.1.1. Transcriptome Assembly Strategy

Contig sequences generated with SOAPdenovo-Trans and Trinity were generally identical, although for some protein classes the different assembly methods generated slightly different contig numbers. However, a comparison of these assembly strategies revealed that, on average, Trinity assemblies contain more sequences that BLAST to secreted and putative toxin proteins, as well as generally longer sequences (see Supplementary Figure S1) than the SOAPdenovo-Trans assemblies (see Table 1). Consequently, we decided to use the Trinity assembled data for all further downstream analyses (see also Section 4.2).

#### 2.1.2. Comparison of 454 FLX and Illumina MiSeq and HiSeq Transcriptomes

**Figure 1:**
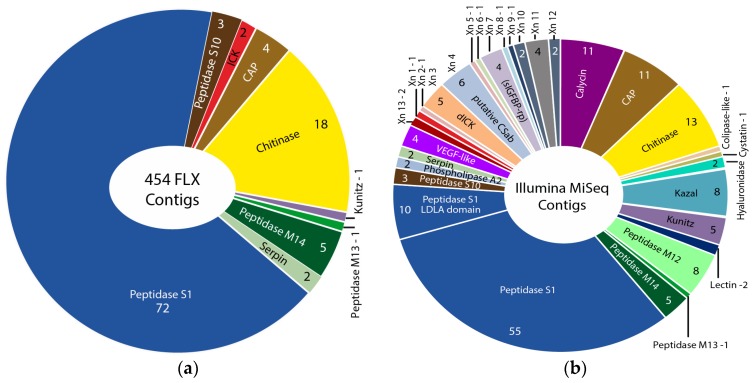
Comparison of contig numbers and the diversity of putative toxin families identified in the 454 FLX Titanium (**a**) and Illumina MiSeq (**b**) venom gland transcriptomes of *Xibalbanus tulumensis* sequenced from the same RNA sample. Abbreviations: CAP: Cysteine-rich (CRISP), Antigen 5 (Ag5), and Pathogenesis-related (Pr-1) proteins; CSαβ: Cystine-stabilized α-helix/β-sheet motif; ICK: Inhibitory Cystine Knot motif; dICK: double ICK peptide; LDLA: low density lipoprotein receptor class A domain; sIGFBP-rp: single Insulin-like Growth Factor Binding Protein-domain related peptide; VEGF: Vascular Endothelial Growth Factor; Xn: xibalbins, which are new peptides identified in this study (see Table 2).

**Figure 2:**
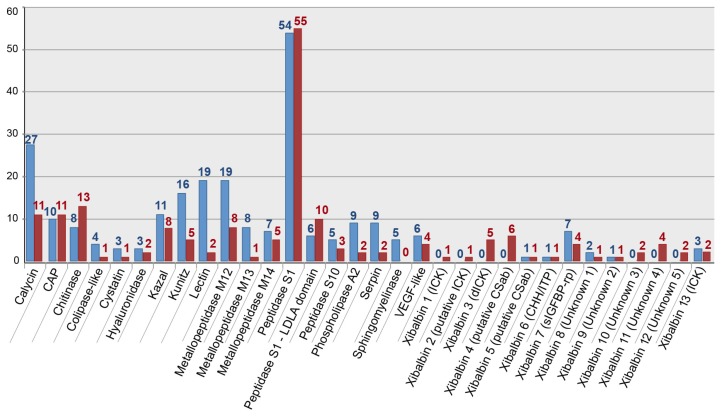
Bar chart showing the 32 identified protein families and the diversity of contigs that passed the expression level threshold (see Materials and Methods) for the venom gland (red) and whole body transcriptomes (blue). See also Supplementary Tables S2 and S3 for further details.

**Figure 3:**
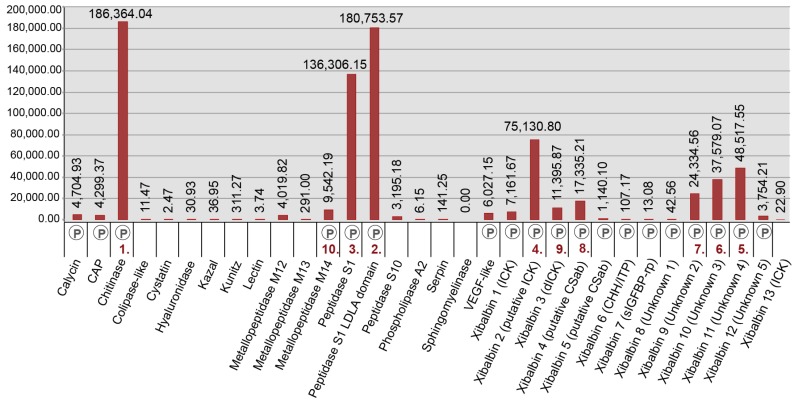
Bar chart showing the relative expression levels (FPKM values) of protein families in the venom gland transcriptome. Presence in the proteome is indicated by the P below the columns; see also Figure 5 and Supplementary Table S1. The 10 most abundantly expressed transcripts are highlighted by the rank numbers below the bars. For more details see Supplementary Table S2.

**Figure 4:**
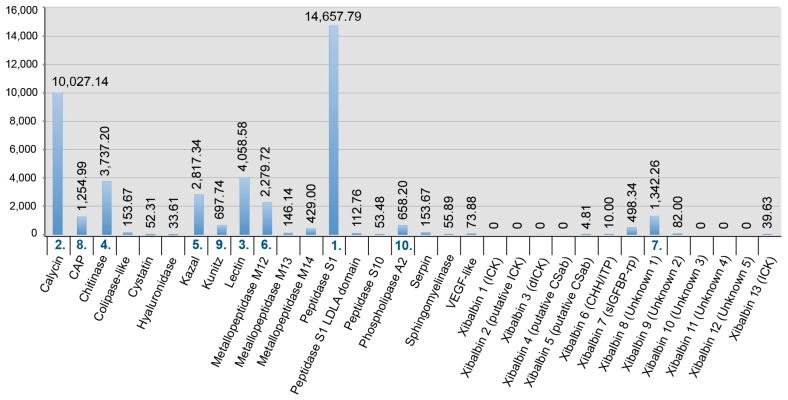
Bar chart showing the relative expression levels (FPKM values) of protein families in the whole body transcriptome. The 10 most abundantly expressed transcripts are highlighted by the rank numbers below the bars. For more details see Supplementary Table S3.

**Figure 5:**
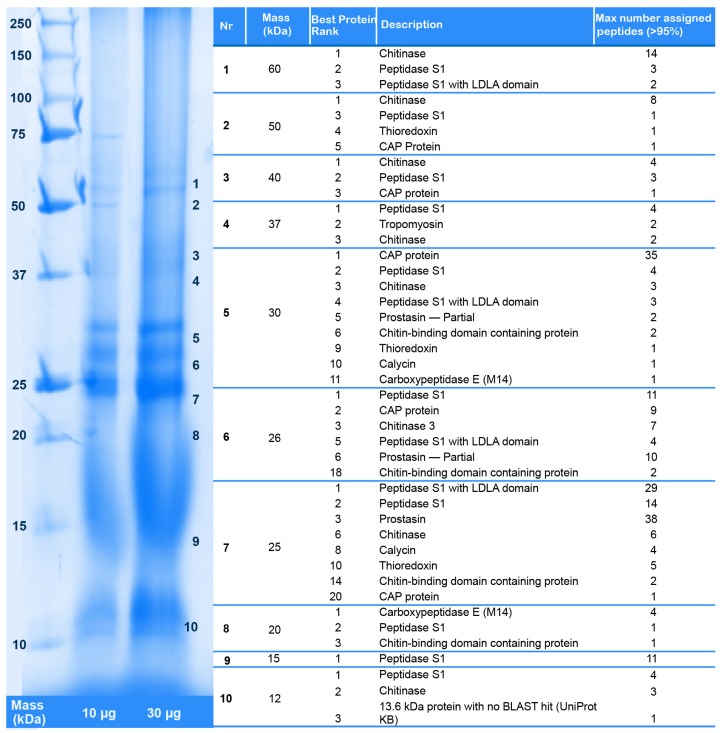
SDS-PAGE gel of crude venom of *X. tulumensis* stained with Coomassie blue (left), and the identified venom proteins (right). The excised bands are indicated with numbers on the right side of the gel. See Supplementary Table S1 for the identity of matching contigs in the transcriptome.

The Illumina MiSeq venom gland transcriptome produced 105 times more sequence reads than the 454 FLX transcriptome in our original study [12] (27,421,129 versus 260,172 reads), which were assembled into 157 times more contigs in the preferred Trinity assembly (197,240 versus 1052 contigs). The Illumina HiSeq whole body transcriptome produced nine times more sequence reads than the original 454 FLX transcriptome (9,165,598 versus 1,000,000 reads), and yielded slightly less than one and a half times more assembled contigs in the preferred Trinity assembly (161,100 versus 115,038 contigs). The greater sequencing depth of the Illumina transcriptomes, informed by the results of the proteomic analysis (see below), allowed us to identify transcripts for 32 venom protein families expressed in the remipede venom glands, 23 of which were not previously reported (Figure 1, Figure 2, Figure 3 and Figure 4). Twelve of these novel putative venom toxins were also identified in the venom proteome (Figure 3 and Figure 5, Table 2, and Supplementary Table S1). All protein families that we previously identified in the 454 data were also found in the Illumina data, and sequences from both transcriptomes are included in the alignments in the Supplementary Material.

Several studies have used both 454 and Illumina sequencing for the transcriptomic profiling of venom glands, either to improve the assembly of Illumina data with 454 data [25], to create a more deeply sequenced transcriptome [26], or to compare the performance of the techniques in recovering venom protein families [27,28]. We know of only one other venomics study that directly compared these sequencing platforms to assess the diversity of venom protein families in transcriptome libraries derived from the same RNA material. Barghi et al. [29] found that although 454 sequencing was better able to identify longer peptide sequences, 10 of the 30 conopeptide superfamilies expressed in the venom glands of the cone snail *Conus tribblei* were only present in the Illumina data. Our results agree with those of Barghi et al. that Illumina sequencing can provide deeper insights into venom gland gene expression than 454 sequencing. However, the results of the two sequencing technologies differ the most for the least expressed transcripts. Only Illumina sequencing managed to pick up the least expressed proteins, such as lectin, kazal domain proteins, hyaluronidase, cystatin, colipase-like proteins, and calycin.

### 2.2. Revising the Venomic Profile of *X. Tulumensis*

The three most highly expressed venom protein families (fragments per kilobase million (FPKM) >100,000) in the venom gland are the enzymes chitinase, peptidase S1 with LDLa domains, and peptidase S1 without LDLa domains. The next six most highly expressed protein families (FPKM > 10,000) are six new peptide families (xibalbins 2–4, 9–11) that represent different peptide scaffolds, including a putative ICK peptide, a double ICK peptide, a peptide with a putative CSαβ fold, two cysteine-rich peptides with unknown structural folds, and two peptides without cysteines. Of the 23 remaining venom protein families, nine are expressed at FPKM values between 1000 and 10,000, while 14 are present only at much lower expression levels (FPKM ≤ 311).

Our proteomic analyses confirmed the presence in the venom of 19 of the 32 venom protein families identified in the transcriptome, including the 12 most highly expressed families (Figure 3 and Figure 5, Table 2 and Supplementary Table S1). The only two protein families that were expressed in the venom gland transcriptome at moderately high levels (FPKM between 1000 and 10,000) that were not observed in the venom proteome are metallopeptidase M12 and peptidase S10. With the exception of xibalbin 1, which we identified on the basis of transcriptome data in our previous study as a peptide with similarities to ß/δ agatoxins, all novel peptides reported here were identified with the help of our proteomic analyses. Interestingly, with the exception of the very lowly expressed peptides xibalbin 7 and 8, all venom peptides could also be identified in the 454 FLX venom gland transcriptome of our previous study [12].

The gene expression profile of the whole body transcriptome is strikingly different from that of the venom glands (Figure 3 and Figure 4). Only two protein families are ranked in the top ten most highly expressed families of both datasets: chitinase and peptidase S1 without LDLa domains. Eight venom protein families, including five of the ten most highly expressed venom protein families in the venom gland (xibalbins 2–4, 10, 11), are not detected at all in the whole body transcriptome. These may therefore represent venom gland specific proteins. Conversely, six of the ten most highly expressed protein families in the whole body transcriptome (calycin, kazal, kunitz, lectin, phospholipase A_2_ and xibalbin 8) are expressed only at moderately to extremely low levels in the venom glands. Interestingly, eight of the ten venom protein families most highly expressed in the venom glands also have higher contig diversity than in the whole body transcriptome, which suggests tissue-specific regulation of contig expression (Figure 2).

A recent study [32] provided a manual approach to deal with the problem that widely used assembly software, such as Trinity, may erroneously assemble highly similar but distinct reads into chimeric contigs, and that a failure to recognize this may underestimate the true diversity of contigs. One consequence would be the overinflation of the expression levels of such chimeric contigs. This could potentially affect our results. However, performing a manual assessment of this issue following the strategy of Macrander and colleagues [32] is unfeasible given the enormous number of reads in each assembly. Moreover, our venom gland transciptome was generated from the pooled venom systems of 25 individuals. Therefore, given the lack of additional data concerning gene copy numbers and intra-population toxin gene diversity, we consider selectively splitting and readjusting expression levels for venom-encoding contigs to see if they might represent multi-copy genes an inappropriate strategy. Consequently, our results provide conservative estimates of the contig diversity within protein families, and expression levels of individual contigs, venom protein-encoding or not, may in some cases be overestimates. However, when the expression levels of all contigs within given protein families are taken together, they provide accurate estimates of the relative expression levels of those families (Figure 3 and Figure 4).

Our new insights into the venom of *X. tulumensis* necessitate revising the previous hypothesis that remipede venom is dominated by enzymes [12]. Although chitinase and peptidase S1 enzymes are indeed the most abundant venom components, a diversity of peptides that are highly or uniquely expressed in the venom glands, notably xibalbins 1–4, and xibalbins 9–11, show that remipede venom is more complex than previously thought. The presence of these newly discovered venom peptides alongside the high molecular weight proteins, more closely aligns remipede venom composition to those of the venoms of other predatory arthropods such as spiders, scorpions and centipedes [17,18,23,24,33,34].

### 2.3. New Venomic Profile Provides Insights into Putative Function of Venom

In this section we discuss in more detail the novel and the most highly expressed venom protein families identified in this study, as well as their possible functional significance. For a discussion of the other venom protein families we refer readers to our previous work [12].

#### 2.3.1. Enzymes

The most abundantly expressed proteins in the proteome and transcriptome are peptidase S1 (PS1) enzymes (Figure 3, Supplementary Table S2 and Figure S3). Likewise, PS1 enzymes are represented by the highest transcript diversity of all venom proteins, a result that corresponds closely to our initial study [12]. Serine peptidases are present in a broad range of venoms, but the extraordinarily high level of expression in remipede venom glands most closely parallels the expression found in the venom glands of some predators such as vipers, helodermatid lizards, and cephalopods [35,36,37,38,39]. PS1s could play a variety of roles in remipede venom. Reptile PS1s (kallikreins) often act on the blood vascular system, activating blood clotting, lowering blood pressure, and increasing vascular permeability [38]. However, since remipedes are predators of invertebrate prey, particularly crustaceans, it is unlikely that their venom has evolved to target the vertebrate circulatory system. Cephalopods provide a closer functional analogue for the possible roles of serine peptidases in remipede venom.

Crustaceans are important prey for octopus, squid and cuttlefish, and among cephalopods the predatory role of venom is best understood for octopuses. Octopuses drill tiny holes into the shells of molluscs and the exoskeleton of crustaceans through which they inject their venom [40,41,42]. The venom paralyses and kills the prey, but it also facilitates feeding through pre-digestion of the prey’s soft tissues. Octopus venom proteases specifically target muscle attachment sites in crabs, which allows the octopus to easily extract their muscle tissue [43,44,45]. In accordance with this venom role serine protease transcripts are the most highly expressed venom protein transcripts in both octopus and cuttlefish posterior salivary glands [35,36,46,47]. A similarly high level of PS1 expression in remipede venom glands is compatible with the idea that they too use their venom to detach the soft tissue of their prey from the exoskeleton, helping with prey ingestion and pre-digestion. However, the diversity of venom PS1s also suggests a functional radiation may have taken place and that this protein family may play additional roles in remipede venom.

The diversity of PS1s expressed in remipede venom glands includes forms with and without low density lipoprotein receptor class A (LDLa) domains. Interestingly, proteins with LDL domains are relatively rare in venoms, and to our knowledge have only been found in arthropod venoms. Beyond centipede venoms [32], which are rich in proteins with LDLa domains, proteins with LDLa domains have been described from the venom of the parasitoid wasps *Nasonia vitripennis* [48] and *Pteromalus puparum* [49], from the skin and bristles of the caterpillar of *Lonomia obliqua* [50], from the venoms of the widow spiders *Latrodectus hesperus*, *L. geometricus* and *Steatoda grossa* [51,52], from the venom of the kissing bug *Rhodnius neglectus* [53], and from the venom of the scorpion *Hadrurus spadix* [34]. The biological roles of these proteins in these taxa remains unknown. However, the structure of the LDLa venom proteins in remipedes in the context of their diet provide clues to a possible role.

The LDLa domain plays a central role in cholesterol metabolism in mammals by binding to lipoproteins, which facilitates their transport across cell membranes via endocytosis, and LDL domains in insects play similar roles in lipid transport [54,55,56]. The structure of the remipede venom LDLa proteins is suggestive of a role in lipid metabolism as well. Compared to the proteins with LDL domains in the venoms of the other taxa listed above, the remipede sequences are unique in containing one, four, five, or six LDLa domains followed by a PS1 domain. PS1s play a crucial role in protein digestion in invertebrates [57]. The unique structure of these remipede venom proteins suggests that the LDLa domains confer affinity for lipoproteins, while the PS1 domain facilitates their digestion by hydrolysing their peptide bonds. Lipoproteins are abundantly present in crustaceans and perform a variety of functions, including lipid transport, hemolymph clotting, defence against microorganisms, and lipid storage in eggs [58]. The highly expressed LDLa domain enzymes present in remipede venom may allow them to effectively digest and absorb the lipoproteins present in their crustacean prey. It may be noteworthy that the cave system where *X. tulumensis* was collected houses a dense population of atyid shrimp, *Typhlatya pearsi*. Although they predominantly inhabit the brackish water above the halocline, they do penetrate the underlying marine water, where remipedes can prey upon them. Since a very high percentage of these shrimp were observed to be gravid [59], it makes them a particularly lipid-rich meal.

Chitinases are the second most abundantly expressed venom protein, as we found previously [12]. We recovered 13 chitinase contigs in our venom gland data that satisfied the expression level cut-off, and 8 in the body tissue transcriptome, which suggests that chitinases exclusively expressed in the venom glands may be undergoing a diversification that could underpin a functional radiation. In support of this hypothesis, the residues crucial for chitinase activity were conserved in all seven body tissue contigs that were of sufficient length to include the catalytic domain, but not in four of the venom gland contigs. While three of these venom gland contigs (c30447_g1_i1; c93564_g1_i1; c137531_g1_i1) are expressed at such low expression levels that they failed our expression level cut-off, the fourth contig (c27030_g1_i1) is the third most highly expressed chitinase contig in the venom glands and was detected in the venom proteome. Interestingly, the chitinase encoded by this contig has substituted the critical catalytic glutamic acid for glycine in alignment position 341. This likely abolishes chitinolytic activity as glutamic acid is the proton donor necessary for the enzyme’s activity. But given its high level of expression it may play another role in the venom, enzymatic or otherwise.

Our phylogenetic analysis shows that remipede chitinases group together in several distinct parts of the chitinase tree (see Supplementary Figure S2). Interestingly, all chitinases found in the venom gland proteome and the top seven most highly expressed chitinase transcripts (expressed one to three orders of magnitude more highly than the others) all cluster together in a single clade. The two chitinase contigs that are most highly expressed in the whole body transcriptome are placed in two different clades elsewhere in the tree.

Chitinases are known from a number of animal venoms, mostly from the venoms of arthropod predators such as glycerid polychaetes, cephalopods, centipedes, scorpions, spiders, cnidarians, and hymenopterans [32,33,35,36,47,52,60,61,62,63]. But chitinase expression in remipede venom glands is probably unparalleled. Chitinase transcript c29839_g1_i1 is the single most highly expressed transcript in the venom glands of the remipede. The expression of this one transcript is higher than the expression of all transcripts combined within each of the other venom protein families, except PS1s with LDLa domains.

Cephalopods, especially octopuses, again provide an instructive functional analogue for the probale role of this venom protein in remipedes. Octopuses, like remipedes, are expert crustacean predators, and chitinase is expressed at relatively high levels in their posterior salivary glands [35,36,43,64]. Octopuses probably do not use their venom chitinase to derive significant nutritional benefit from eating their prey’s chitinous exoskeleton; they are careful not to ingest too much exoskeleton [64]. There is instead suggestive evidence that octopuses use their venom chitinases as a crustacean can opener. Crustacean muscles insert on their chitinous endocuticle, and by weakening this attachment, chitinases can assist PS1s in retrieving and digesting the prey’s muscle [64]. In one published experiment crabs were taken from an octopus a minute and a half after it captured them. Half an hour later the crab’s leg muscles slid out of their exoskeleton “like a string of sausages” [45] (pp. 443–444). We suspect that remipedes deploy their venom chitinase in a similar manner. In one of the few published field observations [65], a remipede was seen to feed on crustacean prey, after which it released an empty exoskeletal husk. Although an additional role of chitinases as a spreading factor for remipede venom is possible as well, we suspect that its main function is disintegrating the bodily integrity of crustacean prey.

#### 2.3.2. Non-Enzymatic Proteins

The most highly expressed non-enzymatic, non-peptidic protein family in the venom of *X. tulumensis* is vascular endothelial growth factor (VEGF)-like protein. It is present in the venom proteome and is predominantly represented by a single highly expressed contig (c29360_g3_i1; see Supplementary Table S2). VEGF-like proteins have been detected in the venoms of viperid, elapid and colubrid snakes, as well as anguimorph lizards, the platypus, and hymenopteran venoms [19,37,38,39,66,67,68,69,70]. VEGF is a potent inducer of vascular permeability, and can thus act as a spreading factor for the venom, as well as assist in prey capture by producing rapid hypotension and shock. However, VEGF-like proteins can be expressed in many tissues, and may be lowly expressed in venom, such as in colubrid snakes, suggesting instead an endophysiological role [66]. The relatively high expression level of VEGF-like protein in the remipede venom glands, compared to its low level of expression in the whole body transcriptome, suggests that it may play a role in remipede venom. Since remipedes have not been seen to prey on vertebrates (although a possible sporadic influx of larval open water fish into their cave system [59] does not preclude the possibility that remipedes could occasionally prey on tiny fish), this venom protein probably doesn’t play a role in predation. It has been proposed that in hymenopterans with cytolytic venoms, VEGF may help maintain an intact venom gland by promoting cell growth [19,70]. Although a similar role is conceivable in remipedes (xibalbin 10 is a possible candidate cytolytic toxin; see Section 2.3.3), VEGF might also play a role in the defensive use of venom. When manipulating collected remipedes with forceps, they attack, grab and audibly bite it with their venom delivering maxillules. It is possible that when taken in the mouth by cave fish, *X. tulumensis* may deliver a defensive bite, with VEGF-like protein targeting the fish’s circulatory system to cause a rapid drop in blood pressure. The effectiveness of hypotensive venom peptides in faciliting escape from fish predators has recently been demonstrated for fangblennies [71], and remipedes might rely on a similar strategy.

#### 2.3.3. Peptides

Contrary to the previous transcriptome-based perception of remipede venom as being composed almost exclusively of enzymes, our revised venom profile of *X. tulumensis* shows that it also contains several novel putative neurotoxin-like peptides. Although the contig diversity is low compared to other predatory arthropod venoms, the peptides we identified in the venom of *X. tulumensis* comprise a significant 13 families spanning at least nine unique structural scaffolds, four of which lack significant similarity to any known structural class. These peptides also include some of the most highly expressed venom components identified—the transcript encoding the putative ICK U-xibalbin2-Xtu1a is the second highest expressed contig in the venom gland transcriptome (c29772_g1_i1, FPKM 75,130.8), second only to a presumably enzymatically functional chitinase isoform (c29839_g1_i1, FPKM 178,137.36). Peptides, and especially cysteine-rich peptides, are therefore likely to play a greater role in the venom of remipedes than previously thought, most likely as primarily neurotoxic components.

ICK peptides are among the most abundant cysteine-rich peptides known, and are found in an exceptionally wide range of organisms where they most likely act as defense molecules against pathogens (“defensins”) [72]. ICK peptides have also been identified in venoms from a number of animal lineages, and confirming their presence in the venom of *X. tulumensis* therefore came as no surprise. However, in addition to the previously reported xibalbin 1 and the extremely highly expressed xibalbin 2, we also identified a highly unusual family of double ICK domain (dICK) peptides, xibalbin 3. In stark contrast to the ubiquity of single ICK domain peptides, dICKs have previously only been described from venoms from the mygalomorph spiders *Haplopelma schmidtii* (tau-TRTX-Hs1a, henceforth Hs1a) [73] and *Hadronyche infensa* (pi-HXTX-Hi1a, henceforth Hi1a) [74] as well as the araneomorph spider *Cheiracanthium punctorium* (delta-MGTX-Cp1, henceforth Cp1) [75]. In the cases of the mygalomorph toxins Hi1a and Hs1a, this unique double neurotoxin domain architecture allows the toxins to act as bivalent ligands that bind virtually irreversibly to their molecular target [73,74]. The dICK architecture of the cytolytic peptide Cp1 also likely enables a bivalent mechanism of action. Cp1s are the primary insecticidal peptides in the venom of *C. punctorium* and have LD_50_ values that are up to several orders of magnitude more potent than what is typical for cytolytic toxins [75]. The presence of two homologous domains may lend Cp1 a targeted cytolytic activity that results in increased potency as a paralytic and lethal insecticidal toxin. Although the significance of the double domain architecture of remipede dICKs remains unknown, the lack of long amphipathic N- or C-terminal tails typical of cytolytic toxins such as Cp1 suggests that their function is likely to be neurotoxin-related.

**Figure 6:**
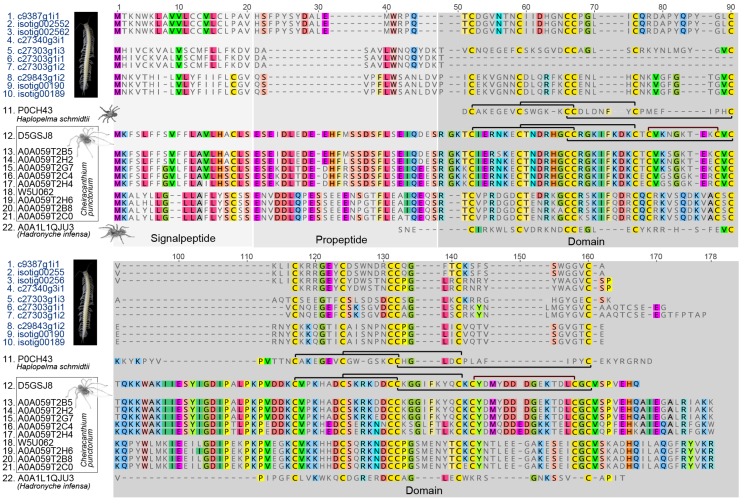
Structural alignment of remipede and all known spider venom dICK sequences. The alignment shows the full peptide length, including signalpeptide, propetide and domain. Disulfide connectivity is shown in black brackets. Amino acid residues are coloured according to the RasMol scheme used in Geneious version 6.

**Figure 7:**
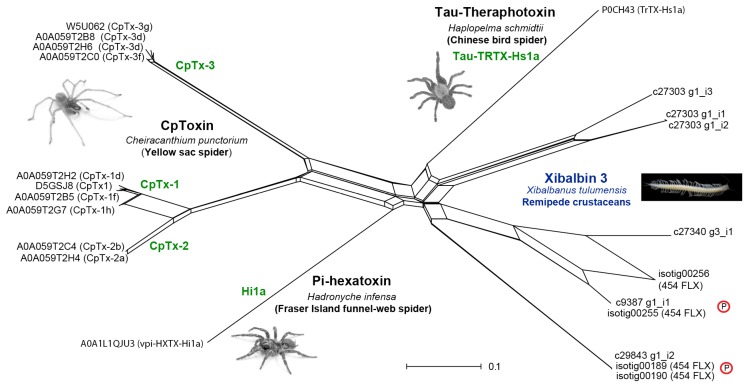
NeighborNet network, reconstructed in SplitsTree 4 [76], based on the structural alignment of Figure 6 of remipede and spider venom dICK sequences. The remipede dICKs group together in three clusters, two of which are represented in the proteomic data (red circled Ps).

A network reconstructed for an alignment of the remipede and all other known venom dICK sequences (Figure 6) shows that the remipede dICKs group together in three distinct clusters (Figure 7). Two of these clusters are present in the venom proteome, and are represented by contigs that are hundreds of times more highly expressed than the contigs in the third cluster. Interestingly, the remipede dICKs cluster more closely together with the mygalomorph dICKs than the latter do with the araneomorph dICKs. Although this does not mean that the remipede and mygalomorph dICKs share a unique common ancestry, it does indicate that these independently evolved venom peptides share some unique similarities that possibly have functional significance, as alluded to above. The mature dICK peptides of the remipede and mygalomorph spiders are much shorter than those of the araneomorph spider, and they lack the C-terminal tails that follow both ICK domains of the araneomorph dICKs.

The relatively low sequence similarity of the two ICK domains of xibalbin 3 and the presence of three additional peptide families in the venom of *X. tulumensis* containing single ICK domains (xibalbin 1, 2 and 13) raises questions as to their evolutionary relationships as structurally homologous putative toxin families. In order to address this question, we constructed an alignment of putatively homologues sequences obtained by BLAST and hmm searches of arthropod sequences in UniProtKB and a custom sequence database designed to improve the taxonomic sampling of arthropod taxa. The two ICK domains of xibalbin 3 were aligned as separate sequences, and the alignment was used to construct a phylogenetic tree and a network (Supplementary Figures S4 and S5). The tree resolves the remipede ICKs and dICKS into four clades separated by sequences from other arthropod taxa. Given there is proteomic evidence for the presence of xibalbins 1–3 in the venom, this suggests that ICK-type venom peptide families may have been recruited into the venom of remipedes three times. The grouping together of the two ICK domains of xibalbin 3 indicates that they likely originated from a duplication of a single non-toxin ICK domain. This is consistent with the lack of detectable single domain ICK venom peptides that, like the xibalbin 3 peptides but unlike xibalbin 1 and 2, lack the fourth pair of cysteines that probably form a β-sheet-stabilising inter-strand disulfide bond in loop 4 [72]. However, these interpretations should be approached with caution given the very low clade support values of the tree and the clustering of the xibalbin 2 and 3 sequences in the same part of the network.

The CSαβ fold is another ubiquitous defensin fold that has been recruited into numerous venoms, and is particularly abundant in scorpion venoms where it comprises the vast majority of known neurotoxins. As the name suggests, the CSαβ fold is characterised by an α-helix joined to a C-terminally positioned β-sheet by two disulfide bonds as well as the presence of additional stabilising disulfide bonds elsewhere in the structure. While the CSαβ cysteine pattern is less well-defined than for the ICK fold, the directional requirements of the disulfide bonds for the stabilisation of the α-helix/β-sheet results in a characteristic y_n_CxxxCy_n_CxCy_n_ motif, where x denotes any amino acid except C and y denotes any amino acid. While neither xilbalbin 4 nor 5 were initially identified as CSαβ peptides by our bioinformatic pipeline, the aforementioned motif is present in both of these families. In addition, the cysteine pattern of xibalbin 4 is very similar to that of a putative toxin identified in the venom gland transcriptome of the lesser brown scorpion, *Isometrus maculatus*, which is predicted to assume a CSαβ fold (UniProt accession A0A0U1S870). Thus, we hypothesise that xibalbin 4 and 5 are likely to adopt novel structural versions of this widely distributed and pharmacologically important peptide fold. It is also interesting to note that xibalbin 4 was identified only in the venom gland transcriptome, where it was highly expressed (c18306_g1_i1, FPKM 17,073.49), suggesting a venom-specific role, perhaps as a neurotoxin.

While the ICK and CSαβ folds represent two defensin folds that have been recruited to a toxic or putatively toxic role in several animal lineages, we also identified a possible third defensin fold that has to our knowledge not previously been reported from any venom. Although xibalbin 10 shows no significant overall sequence similarity to any peptide, its cysteine pattern and spacing is identical to that of several hairpin-like β-sheet forming antimicrobial peptides (AMPs) such as the pancrustacean arasins [77] and mammalian protegrins [78]. In the arasins, the antimicrobial activity is contained in a cysteine-free proline- and arginine-rich N-terminal domain [79] that is not present in xibalbin 10. This domain is also absent in protegrins, which instead exert their antimicrobial activity by assembling into multimeric transmembrane β-barrel pores [80]. However, the antimicrobial activity of protegrins is heavily dependent on a high number of positively charged residues and the presence of an amphipathic structure [81], neither of which is present in xibalbin 10. It is also interesting to note that xibalbin 10 is highly expressed in the venom glands and absent from the whole body transcriptome, which would be unexpected for an AMP family. Together these observations suggest that xibalbin 10 is a novel family of defensin-derived peptides with a venom-specific role, perhaps as pore-forming cytotoxins or neurotoxins.

Defensins have probably been an important source of putative peptide toxins in remipedes. However, we also identified two hormone peptide families in the venom of *X. tulumensis*, namely ion transport peptide and crustacean hyperglycemic hormones (ITP/CHH: xibalbin 6) and insulin-like growth factor binding protein-related proteins (IGFBP-rp: xibalbin 7). Both these families are taxonomically widespread—ITP/CHH within Ecdysozoa and IGFBP-rp within Bilateria—where they perform diverse functions such as glucose metabolism, osmoregulation, neurosignalling, and developmental control [82,83]. Both families also include examples of recruitment into animal venoms, namely the venom insulins that are found in a wide range of cone snails [84] and the helical arthropod neuropeptide-derived (HAND) toxins that are weaponised CHH/ITP peptides found in the venoms of some spiders and centipedes [85]. The functional signifance of these peptides, however, is uncertain. The transcript expression levels of xibalbin 6 and xibalbin 7 are moderate and very low, respectively. Moreover, the body transcriptome contains IGFBP-rp encoding contigs that are identical to those identified in the venom glands. And while the xibalbin 6 peptide identified in the venom is encoded by a contig unique to the venom gland, it has retained the ancestral C-terminal tail that forms a fifth helix, the loss of which appears to be crucial for the switch from a primarily hormonal or toxin-chaperone function to that of a bona fide toxin [85]. Thus, although we cannot rule out that xibalbin 6 and 7 contribute to envenomation, our data suggest they are probably not integral to the toxicity of the venom.

In addition to venom peptide families where their likely origin provides clues as to their possible venom function, we identified an additional four peptide families in the venom for which we were unable to identify potential homologues outside *X. tulumensis*. Two of these are cysteine-rich peptide families (xibalbin 8 and 9) that could represent entirely novel structural folds, while two consist of putative linear peptide toxins that are encoded as multiple mature domains on their respective transcripts (xibalbin 11 and 12). Of these, xibalbin 9 and 11 are particularly interesting given their high expression levels in the venom gland (FPKM > 20,000) versus low expression level (xibalbin 09, FPKM 82) or complete absence (xibalbin 11) from the whole body transcriptome. Although the potential activities of these venom peptides remain even more elusive and speculative than the peptide families described above, it is interesting to note that xibalbin 11 encodes two domains that are both proline-rich, which is a common feature among many bioactive non-cysteine-rich peptides.

## 3. Conclusions

**Figure 8:**
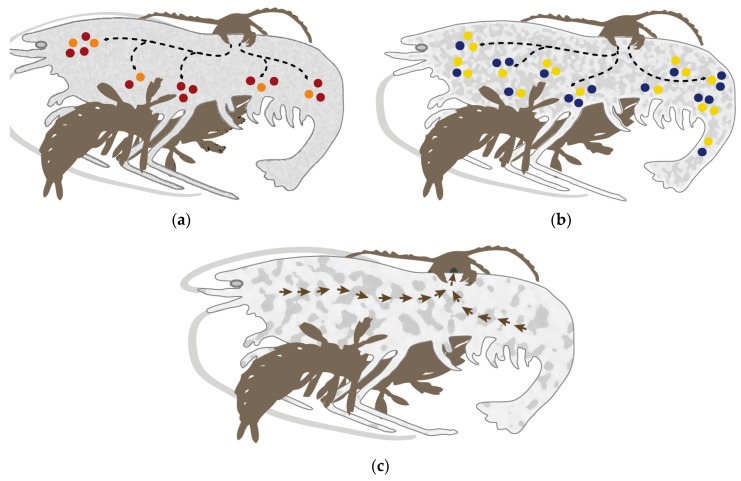
Speculative illustration of the prey capture and the venom injection process of remipedes. (**a**) Crustacean prey (here a shrimp) is immobilized by injection (red and orange circles) of the putative neurotoxic peptides (xibalbins 1–4: ICKs, dICKs and CSαβ). (**b**) Peptidases and chitinase (blue and yellow dots) break down the structural integrity of internal soft tissue, and dissociate the muscles from their anchoring points on the exoskeleton, as well as enhance paralysis effects by allowing the venom to spread further through the body. (**c**) The liquefied prey tissue is sucked up by the remipede as illustrated by the arrows.

Our study reveals the power of a combined transcriptomic and proteomic investigation of remipede venom. The proteomic results confirm and extend our previous findings, and allowed us to identify a diversity of novel venom peptide families, four of which are currently without known homologues outside remipedes. The new results show that remipede venom is considerably more complex than previously thought, consisting of a mixture dominated by peptidases, chitinase and a diversity of peptides we have named xibalbins. We have used these new insights to speculate on the possible roles of the dominent venom proteins and peptides to provide testable ideas that can inform the design of any future studies on the bioactivity of the venom (Figure 8).

Molecular clock estimates suggest that the origin of the remipede lineage may lie as far back as the Ordovician [8] or Cambrian [9]. Only two known fossil species from the Carboniferous have been assigned to the remipede lineage, but there remains serious doubt about the validity of these taxonomic assignments [1,59], and what is known about these fossils does not illuminate the origin of the remipede venom system. However, if a recent estimate that puts the origin of crown-group remipedes somewhere in the Cretaceous is accurate [9], it would be very interesting to explore the venoms of the other known species of remipedes as they would represent a chemical weapon that has been uniquely and exclusively honed in disjunct marine cave environments for 70 odd million years.

## 4. Materials and Methods 

### 4.1. Species Collection, Dissection and Preservation

**Figure 9:**
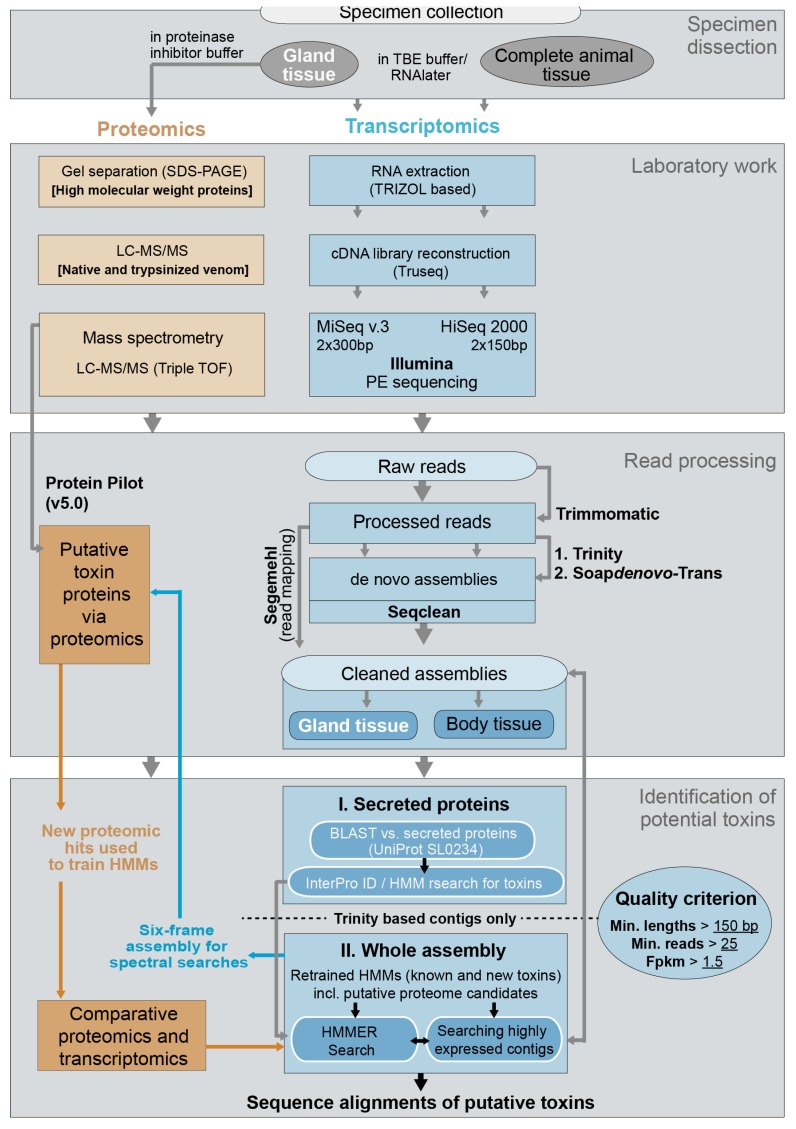
Workflow of comparative proteomics and transcriptomics. PE sequencing is paired end sequencing.

Specimens of *X. tulumensis* were collected in 2012 and 2014 for transcriptomic and proteomic analyses in cave diving expeditions to the Yucatan, Mexico, see also [12]. Several complete individuals were dissected in TBE buffer and preserved in RNAlater to sequence a body transcriptome (BodyT) on the Illumina HiSeq platform (San Diego, USA). The venom delivery systems of 25 specimens (including venom gland, venom duct and venom reservoir) were dissected and preserved in RNAlater to generate the venom gland transcriptome (VgT) on the MiSeq Illumina platform (San Diego, CA, USA). For proteomic work venom delivery systems of 25 specimens were dissected in sterile PBS buffer and preserved in a protease inhibitor solution using cOmpleteUltra Mini tablets (Roche, Basel, Switzerland) dissolved in PBS and RNAse free HCP water following the manufacturer’s protocol, see Figure 9 for complete work flow.

### 4.2. Identification of Putative Toxins via Transcriptomics

#### 4.2.1. RNA Extraction and Library Construction

Total RNA of venom gland tissue (VgT) was extracted at LGC Genomics Berlin using the Trizol-GTI-LiCl method. Synthesis of cDNA and amplification was conducted with the Mint kit (Evrogen, Moscow, Russia). After cDNA digestion, fragments were size selected on an LMP agarose gel. To include shorter toxin sequences the size of selected fragments was lowered to 200 bp. Purified fragments (MinElute Gel extraction kit, Qiagen, Hilden, Germany) were ligated to a pDNR-lib vector (Clontech-Takara Bio USA, Mountain View, CA, USA) using the Fast Ligation Kit (New England Biolabs, Ipswich, UK). Inserts were LMP agarose gel purified (MinElute Gel extraction kit) and ligated to high-molecular weight DNA using a proprietary Sfi-linker (see also [12]). The sequencing library was generated using the Illumina MiSeq V3 kit following the manufacturer’s protocol. Paired end sequencing was performed in 600 cycles (2 × 300 bp) at the sequencing facility in the Natural History Museum, London, UK. Total RNA of the body tissue from complete specimens (BodyT) was isolated applying TRIzol according to the manufacturer’s instructions (Invitrogen, Grant Island, NY, USA). Messenger RNA was purified using the Dynabeads mRNA Purification Kit (Invitrogen, Grand Island, NY, USA) and sheared applying the RNA fragmentation reagent (Ambion, Austin, TX, USA). First strands were transcribed using SuperScript™ II Reverse Transcriptase (Invitrogen, Grand Island, NY, USA) and random N6 primer (IDT). Second strand cDNA synthesis was performed using RNase H (Invitrogen, Grand Island, NY, USA) and DNA polymerase I (New England BioLabs, Ipswich, MA, USA). After end repair, adapter ligation and size selection on agarose gels (250 ± 20 bp) and indexing the library was paired end sequenced with 150 bp on Illumina HiSeq platform following the manufacturer’s protocol at the Beijing Genomics Institution, Beijing, China.

#### 4.2.2. Pre-Processing of RNA Sequence Reads

All reads from both libraries, representing the Venom gland transcriptome (VgT) and the body transcriptome (BodyT) were pre-processed and quality checked before assembly. First, all reads were visually checked for overrepresented sequences and general quality using FastQC v.0.11.2 [86]. Then Trimmomatic v.0.33 [87] was run to exclude all reads below a quality phred value of 20 (sliding window size 4). A modified file of all available adapter and vector sequences was used to screen and to clip sequences for adapter and vector contamination. All reads with lengths of less than 60 bp were excluded. After this first step FastQC was used again to check all reads for each library. In a second step Prinseq v.020.4 [88] was applied to trim putative poly-A and T contamination and to exclude possible homopolymer sequences. Again reads shorter than 60 bp were excluded. Surviving singleton reads from the paired end reads that matched all quality criteria were written into separate fastq files for each sequencing direction and were included into the downstream analyses.

The MiSeq Illumina data for the VgT is available in NCBI via the bioproject PRJNA203251, biosample SAMN02146300 and the SRA accession number SRR5483223. The BodyT Illumina HiSeq data can be accessed via the bioproject PRJNA254312, biosample SAMN03142473, SRA accession number SRS744741 and TSA entry GCBC01000000. Transcriptome assemblies, supplementary figures and tables, including spreadsheet versions of Supplementary Tables S5–S8, and alignments of all venom proteins are accessible via the Natural History Museum’s Data Portal at [89].

#### 4.2.3. Comparative Read Assembly Strategy Using Trinity and SOAPdenovo-Trans

All pre-processed reads from both libraries were assembled to contigs using the assembly software Trinity v.2.0.2 [90] with standard settings applying a minimum length of 101 bp. To test two different assembly methods SOAPde novo-Trans v.1.0.3 [91] was also used to generate three assemblies with kmer sizes of 31, 47 and 65 for the VgT library. After assembly all contig sequences were checked again for vector, linker and adapter sequences performing a local VecScreen against a local UniVec [92] and Emvec database [93]. All sequences that matched a possible contamination were automatically excluded. Finally, all surviving contigs with a minimum length of 138 bp were kept for subsequent analyses. The SOAP assembly with kmer size 47 showed the highest number of contigs (197,240), and was kept for subsequent analyses and comparison to the Trinity based assemblies (see also Table 1).

#### 4.2.4. Read Mapping to Assess Expression Levels of Identified Putative Toxin Contigs

To assess expression levels of identified putative toxins all reads were mapped with 95% accuracy against the assemblies using the software Segemehl 0.2.0 [94]. The resulting SAM files were converted to sorted BAM and BAMindex files in Samtools v. 1.2 [95]. The read mapping results were visually inspected with the program Tablet 1.14.11.07 [96] based on the sorted BAM files and extracted as table format. All resulting read numbers for each contig are shown in the Supplementary Tables S5–S8 for Trinity and SOAP de novo based assembly strategies. It is important to keep in mind that a read mapping after a kmer based assembly does not reflect the true and precise read distribution per contig, but represents an approximation estimating the reads by a posteriori alignment approaches.

#### 4.2.5. Identification of Putative Venom Toxins in Secreted Proteins of Assembly and Selection of Optimal Assembly Strategy

To identify putative toxin transcripts all contigs were processed in a first step with the pipeline developed in [97] (this step is labelled ‘I. Secreted proteins’ in Figure 9). In this pipeline all nucleotide transcripts are translated in all six reading frames. Contigs are then blasted with an e-value of 0.0001 in a second step using BLASTP of the BLAST 2.2.30+ suite [98] against a database file including all secreted cellular component proteins from UniProt (Term: SL0243 including all known 93,736 canonical sequences and isoforms from secreted proteins and isoforms, April 2015). Matching sequences were extracted from our libraries in the last pipeline step. Finally, sets of sequences of secreted proteins were constructed, excluding duplicate sequences created by the six-frame translation. For all of these contigs a local search for signal peptides was performed using SignalP 4.1 [99] with more sensitive settings of SignalP 3.0 (0.34 for both D-cutoff values).

Putative toxin homologues were then identified in a two-fold approach: (1) applying a customized script following the procedure described in [97] to search the results based on the InterPro ID’s of the InterProscan 5 search [100] integrated in Blast2GO 2.8 [101]. We searched for the following IDs for 21 known toxin protein families and terms related to toxins: IPR009104, IPR001304, IPR014044, IPR001223, IPR000010, IPR002223, IPR000566, IPR000566, M12, IPR001254, IPR001563, IPR001211, IPR000215, IPR003582, IPR017766, IPR005853, Stonu, Gigan, Agat, toxin, venom; (2) Hidden Markov models were trained using the version v3.1b2 of hmmer3 [102] based on alignments of 27 known, annotated toxin sequences from UniProt (August 2016).

After analysing the results of the first toxin identification subsequent analysis steps were conducted only with Trinity assembled data. A comparison of the results of SOAPdenovo-Trans and Trinity assembled data showed that the SOAP assemblies yielded fewer matching sequences (see Supplementary Tables S5–S8 and Figure S1). Furthermore, a comparison of alignments of putative toxin contigs showed that SOAP-assembled contigs were generally much shorter than Trinity-based contigs.

#### 4.2.6. Identification of Putative Venom Toxins in the Complete (Secreted and Non-Secreted Proteins) Assembled Data

Next, the complete six-frame translated assembled data for venom glands and body tissue were searched with hidden Markov models that were retrained with (a) putative venom toxins identified above with the search restricted to secreted proteins and (b) venom proteins identified via mass spectrometry. Additionally, highly expressed transcripts were searched separately by hand if they were recovered by the hmm searches and checked via BLAST.

For every contig included in the final analyses the FPKM value was calculated, normalizing the number of mapped fragments for the sequencing depth of each library and the length of each contig [103,104]. This is crucial to facilitate comparisons of expression levels of contigs across protein families within transcriptome libraries. All contigs below a threshold of FPKM < 1.5, a minimal length of 150 bp, and a minimum of 25 reads, were excluded to ensure further analyses were conservative and to prevent over-interpreting the results.

### 4.3. Identification of Putative Toxins via Proteomics

Lyophilized venom was dissolved in ultrapure water to a concentration of 5 mg/mL prior to proteomic analyses. To fractionate and visualize high-molecular weight proteins present in the venom of *X. tulumensis*, 50 μg crude venom was separated by SDS-PAGE using a 12.5% Tris-Glycine gel. Bands were visualized by staining with colloidal Coomassie followed by destaining of the gel with 1% acetic acid. Individual bands were dissected, digested with trypsin, and tryptic peptides eluted as described previously [32]. Proteins were then identified by analysing the tryptic peptides by LC-ESI-MS/MS and matching the resulting fragment spectra with the venom gland and body transcriptomes translated to all six reading frames using ProteinPilot v5.0 (ABSciex, Framingham, MA, USA) as described below; see also Figure 9. LC-MS/MS experiments were carried out on a ABSciex 5600 TripleTOF mass spectrometer as described below, but using a 30 min gradient of 2–45% solvent B (0.1% formic acid (FA), 90% acetonitrile (ACN)) in 0.1% FA.

To further identify proteins and peptides found in the venom of *X. tulumensis* we also analysed both native and trypsinized venom by LC-MS/MS; see Figure 9 for the processing flow. For digestion by trypsin, 10 μg crude venom was first resuspended in 4 M urea 10% ACN 100 mM ammonium bicarbonate, pH 8. Cystines were reduced by incubating with 5 mM dithiothreitol at 70 °C for 5 min and alkylated with 10 mM iodoacetamide at 37 °C for 90 min. Reduced and alkylated venom was then digested by incubating with 30 μg/μL trypsin overnight at 37 °C in 2 M urea 10% ACN 100 mM ammonium bicarbonate, pH 8, at a final substrate to enzyme ratio of 100:1. The digested sample was desalted using a C18 ZipTip (Thermo Fisher, Waltham, FL, USA) and dried using a vacuum centrifuge. For LC-MS/MS analyses, native or digested venom was dissolved in 0.5% FA and 2 μg analysed on an AB Sciex 5600 TripleTOF equipped with a Turbo-V source heated to 550 °C, and an AB Sciex 5600 TripleTOF equipped with a nano electrospray ion source. For analysis on the turbo source equipped 5600 mass spectrometer, venom was fractionated on a Shimadzu (Kyoto, Japan) Nexera UHPLC with an Agilent Zorbax stable-bond C18 column (Santa Clara, CA, USA) (2.1 mm × 100 mm, 1.8 μm particle size, 300 Å pore size), using a flow rate of 180 μL/min and a gradient of 1–40% solvent B (90% ACN, 0.1% FA) in 0.1% FA over 60 min. For analysis on the nano source equipped 5600 mass spectrometer, venom was fractionated on a nano HPLC–MS–MS/MS on a Shimadzu Prominence Nano HPLC with a Agilent C18 column (1 μm × 100 mm, 3 μm particle size, 90 Å pore size) over the same gradient as above but at a flow of 500 nL/min.

MS1 spectra were acquired at 300–1800 *m*/*z* with an accumulation time of 250 ms, and selecting the 20 most intense ions for MS2 scans acquired at 80–1400 *m*/*z* with an accumulation time of 100 ms and optimized for high resolution. Precursor ions with a charge of +2 to +5 and an intensity of at least 120 counts/s were selected, with a unit mass precursor ion inclusion window of ±0.7 Da, and excluding isotopes within ±2 Da for MS/MS. MS/MS spectra were searched against venom gland and body transcriptomes translated to all six reading frames using ProteinPilot v5.0 (ABSciex) using thorough search. Amino acid substitutions and biological modifications were allowed in order to identify potential post translational modifications but also to minimize the effect of false negatives due to the inherent variability of venom toxins, isoform mismatch with transcriptome data, and to account for chemical modifications due to experimental artefacts. Decoy-based false discovery rates (FDR) was estimated by ProteinPilot v5.0, and only protein identifications with a corresponding local FDR of <0.5% were considered significant.

### 4.4. Final Comparative Toxin Identification and Analyses

#### 4.4.1. Sequence Alignments, Phylogenetic Tree and Network Reconstructions

Sequence alignments were constructed for all identified putative toxin classes by including known toxin proteins from the UniProt database and NCBI GenBank (Supplementary Materials). After testing the best fitting substitution model with Prottest 3.4.2 [105], phylogenetic trees were computed for selected proteins discussed in the text with RAxML 8.2 [106], applying the Maximum Likelihood criterion (–f a and 1000 or 10,000 bootstraps; see figure legends of phylogenetic trees). Alignments of venom proteins show often conserved domain regions in combination with highly divergent sequence regions such as signalpeptide and propetide that are almost impossible to align. For that reason we cut the alignments manually based on information of manually curated toxins for domain and propeptide information to eliminate unalignable regions before reconstructing trees. Tree files from RAxML were imported into Archaeopteryx version 0.9901 [107] to visualize the topology, and they were edited in Adobe Illustrator (CS 5, Adobe Systems Software Ireland Ltd, Dublin, Ireland). Additionally, neighbour joining networks were reconstructed in SplitsTree 4 [108] to identify conflicting signals in our alignments. Adobe Illustrator was used for final graphics editing.

#### 4.4.2. Assessing the Evolution of ICK Sequences by Extensive Data Mining in NCBI and UniProt 

In 2014 [12] we described the first ICK scaffold-based putative remipede toxin (then referred to as agatoxin-like) that we rename xibalbin 1 in this study. To understand the evolution of xibalbin 1 and other putative toxins with ICK cysteine scaffolds (xibalbins 2, 3, 13) within pancrustaceans we used contig c29168 as a query in a BLAST search (e-value 0.001) against NCBI (nr) and UniProt for possible homologues within invertebrates. Additionally, available sequences of arthropods and other invertebrates were included from TSA (Transcriptome Sequencing Archive) and SRA (Sequencing Read Archive). Publicly available assemblies from next generation sequencing (NGS) in TSA were used to search for putative ICK scaffold peptides via trained hidden Markov models (HMMs) for this protein (e-value 0.001). The HMMs were also applied to search for putative ICK contigs in eight new assemblies of crustaceans from the SRA representing several major crustacean lineages (see Table 3). Raw data was processed with the same programs and settings that were used to generate the remipede transcriptome data, with the exception of a quality threshold of phred 30 for Trimmomatic.

## Figures and Tables

**Table 1:** Overview of transcriptome libraries and assembly sizes (reads and numbers of contig sequences) for different assembly strategies. Underlined numbers highlight the assembly strategies that were used to identify putative toxins for downstream analyses.

Assembly Strategy	Raw Data	Clipped Data	Cleaned Data	Number of Secreted Proteins
	“Raw” Contigs	Contigs > 137 nuc	Trimmomatic Seqclean	BLASTX vs. UniProt (SL0243)
**Venom gland transcriptome (library: 27,421,129 reads, paired end)**				
SOAPdenovo-Trans (k-mer 31)	294,931	177,668	176,408	-
SOAPdenovo-Trans (k-mer 47)	247,453	198,075	197,240	1799 ^1^
SOAPdenovo-Trans (k-mer 65)	203.964	179,168	178,835	-
Trinity (read length > 101)	191,255	166,309	165,333	1943 ^1^
**Body transcriptome (library: 9,165,598 reads, paired end)**				
SOAPdenovo-Trans (k-mer 47)	151,399	123,676	123,241	2376 ^1^
Trinity (read length > 101)	203,113	161,511	161,100	3004 ^1^

^1^ See also methods Section 4.2.3.

**Table 2:** Peptide families identified by proteomic analyses of the venom. Canonical cysteines and sequence motifs are marked in red where known, while brackets in the cysteine pattern of the double ICK domain peptide denote length of the inter-domain linker. Predicted mature length refers to the predicted length of the mature peptide rather than of the contig. Contigs VG specifies the number of contigs matching the mass spec fragments for each peptide. FPKM VG records the cumulative FPKM values for the contigs with a matching mass spectrometry fragment. See Supplementary Tables S2 and S3 for additional contigs for these peptides that lack matching mass spectrometry fragments. See Supplementary Table S4 for the names of the individual peptides, named according to the rational nomenclature for venom peptide toxins developed in [30,31]. Abbreviations: ITP/CHH: Ion Transport Peptide/Crustacean Hyperglycermic Hormone; all other abbreviations as in Figure 1. ^1^ A proteomic hit for xibalbin 12 was found only for a contig from the 454 data, for which FPKM values are not available.

Peptide Family	Structural Fold	Scaffold	Predicted Mature Length	Contigs VG	FPKM VG
Xibalbin 1	ICK	xCx_6_Cx_6_CCx_4_CxCx_6_CxCx	49	1	7138.77
Xibalbin 2	Putative ICK	Cx_5_Cx_5_Cx_6_Cx_7_CCx_4_CxCx_8_CxCx	50	1	75,130.80
Xibalbin 3	Double ICK	xCx_6_Cx_6_CCx_4_Cx_9–11_C [x_4_] Cx_6_Cx_6_CCx_4_Cx_9_Cx	74–79	2	11,366.77
Xibalbin 4	Putative CSαβ	xCx_12_Cx_3_Cx_5_Cx_5_Cx_5_CxCxC	61	1	17,073.49
Xibalbin 5	Putative CSαβ	xCx_14_Cx_8_CxCx_3_Cx_17_CxCx_4_Cx_10_CCx	87	1	1140.10
Xibalbin 6	ITP/CHH	xCx_15_Cx_2_Cx_12_Cx_3_Cx_8_Cx	82	1	107.17
Xibalbin 7	sIGFBP-rp	Cx_2_Cx_4_Cx_5_Cx_8_Cx_4_Cx_7_Cx_9_Cx_5_CxCx_2_Cx_2_Cx_6_Cx_4_Cx	77	2	1.96
Xibalbin 8	Unknown	xCx_10_Cx_28_CCx_4_Cx_7_Cx_10_Cx_21_Cx	105	1	42.56
Xibalbin 9	Unknown	xCx_5_CxCx_2_Cx_21_Cx_3_Cx_3_Cx_2_Cx_>50_	114	1	24,334.56
Xibalbin 10	Unknown	xCxCx_4_CxCx	21	2	37,579.07
Xibalbin 11	Unknown	No cysteines; two P-rich domains	18/32	2	46,289.60
Xibalbin 12	Unknown	No cysteines; multiple ‘SIFQK’/‘FIFPK’ domains	5	2	0 ^1^

**Table 3:** Overview of data for pancrustacean taxa mined from next generation sequencing archives in NCBI, TSA (assemblies) and SRA (raw data); databases were last accessed in April 2016. All possible crustacean sequences were included as well as new data from early hexapod lineages. For the two venomous parasitic crustaceans *Caligus rogercresseyi* and *Lepeoptheirus salmonis* and six other crustaceans the SRA data was newly assembled either due to lack of information about the assembly or to improve on the quality and quantity of available sequences.

Pancrustacean Group	Major Group	Species	TSA	SRA	No Matching Sequence
**Major crustacean lineages**	Thecostraca (Cirripedia)	*Tetraclita japonica* (OA)		SRR426837	x
	Copepoda	*Calanus finmarchicus*	x		
		*Tigriopus californicus*	x		
		*Caligus rogercresseyi* (OA)		SRR1232138	
		*Caligus rogercresseyi* (bad data)	x		
		*Lepeophtheirus salmonis* (OA)		ERR262962	
		*Lepeophtheirus salmonis* (bad data)	x		x
	Branchiura	*Argulus siamensis* (OA)		SRR514120	
		*Argulus foliaceus* (OA)		SRR3183279	
	Decapoda	*Procambarus clarkii*	x		
		*Astacus astacus*	x		x
		*Carcinus maenas*	x		
		*Eriocheir sinsensis*	x		
		*Euphausia crystallorophias* (OA)		ERR264582	
	Amphipoda	*Ligia exotica (OA)*		DRR054553	
		*Asellus aquaticus*	x		
		*Armadillidium vulgare* (OA)		SRR1324800	
		*Hyalella azteca*	x		
	Branchiopoda	*Triops newberryi*	x		
**Early hexapod lineages**	Protura	*Acerentomon* sp. *AD-2013*	x		
	Diplura	*Campodea augens*	x		
		*Occasjapyx japonicus*	x		
	Collembola	*Tetrodontophora bielanensis*	x		
		*Anurida maritima*	x		
		*Folsomia candida*	x		x
		*Sminthurus viridis*	x		
		*Pogonognathellus* sp. *AD-2013*	x		
	Archaeognatha	*Machilis hrabei*	x		
	Zygentoma	*Thermobia domestica*	x		
	Odonata	*Calopteryx splendens*	x		x

## Supplementary Materials

The following are available online at www.mdpi.com/2072-6651/9/8/234/s1, Figure S1: Comparison of the assembled transcripts from the two different assembly strategies Soap *denovo*–Tran and Trinity, which match secreted proteins after translation to amino acids, Figure S2: Phylogenetic tree reconstructed of all chitinase sequences, Figure S3: Phylogenetic tree reconstructed from an alignment of all peptidase S1 sequences, Figure S4: Phylogenetic tree reconstructed from an alignment of all dICK and ICK sequences, Figure S5: Neighbour joining network reconstructed from an alignment of all dICK and ICK sequences, Table S1: Venom gland proteomics, Table S2: Venom gland tissue transcriptomics, Table S3: Whole body transcriptomics, Table S4: Novel remipede venom peptide families and peptides named according to the rational nomenclature for venom peptide toxins proposed by King et al, Table S5: Venom gland tissue transcriptome (SOAP *denovo*-Tran contigs that match against secreted proteins [UniProtSL0243]), Table S6: Whole body transcriptome (SOAP *denovo*-Tran contigs that match against secreted proteins [UniProtSL0243]), Table S7: Whole body tissue transcriptome (Trinity contigs that match against secreted proteins [UniProtSL0243]), Table S8: Venom gland tissue transcriptome (Trinity contigs that match against secreted proteins [UniProtSL0243]).
