# Supplementary material for: Venomics of Remipede Crustaceans Reveals Novel Peptide Diversity and Illuminates the Venom’s Biological Role
Source: Toxins (Basel). 2017 Jul 26;9(8):234. doi: 10.3390/toxins9080234 (PMC5577568; doi:10.3390/toxins9080234)
Supplement: Supplementary file 1 [file toxins-09-00234-s001.zip › Supplementary Figure.pdf]

Supplementary Materials: Venomics of Remipede Crustaceans Reveals Novel Peptide Diversity and Illuminates the Venom's Biological Role

Björn M. von Reumont, Eivind A. B. Undheim, Robin-Tobias Jauss and Ronald A. Jenner

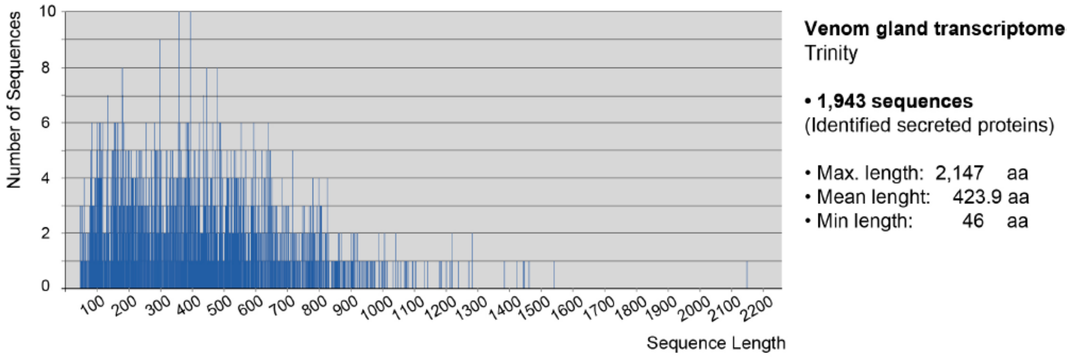

(a)

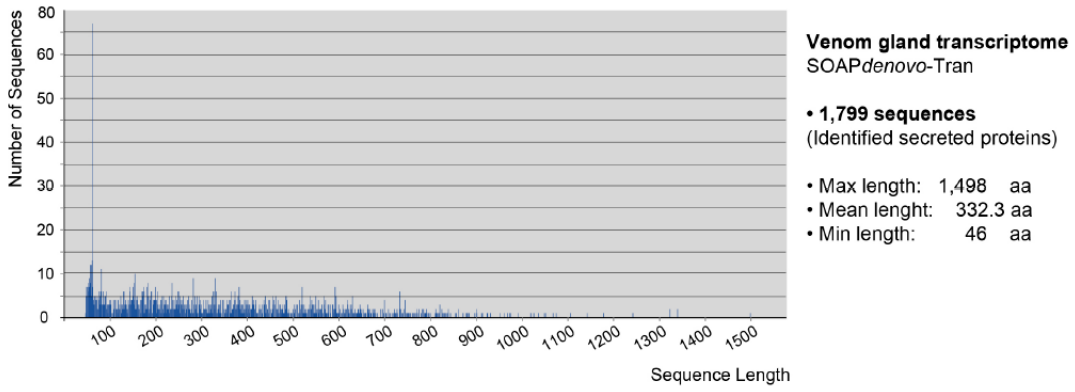

(b)

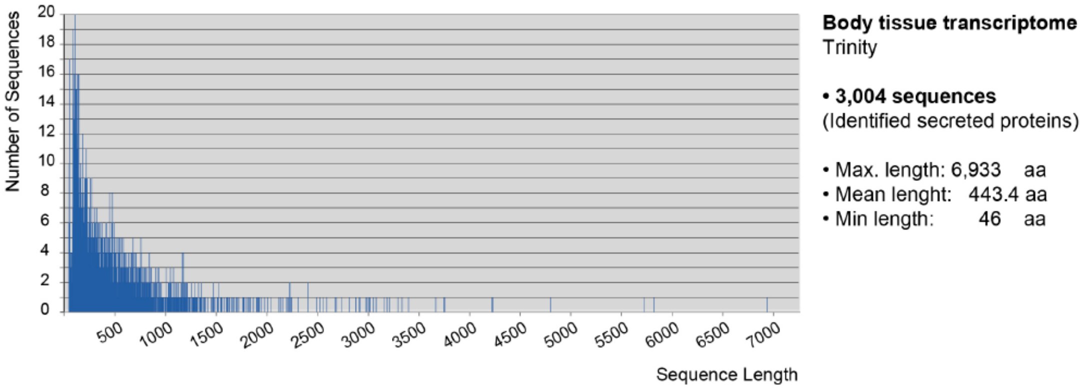

(c)

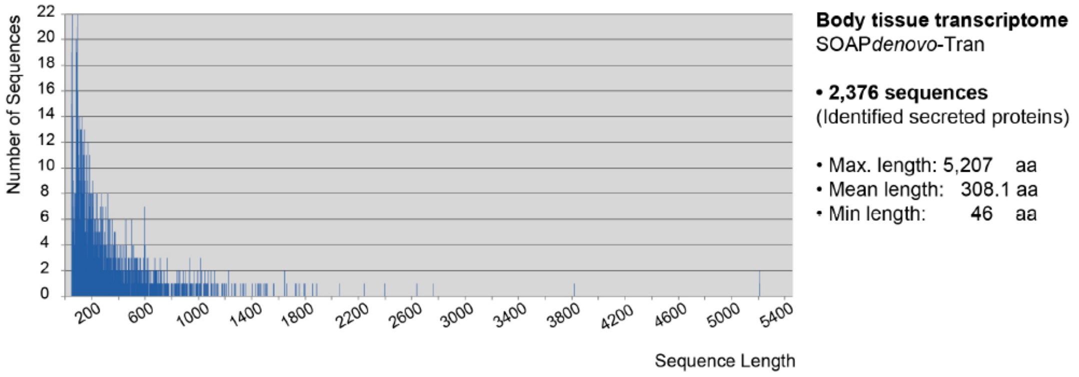

(d)

Figure S1. Comparison of the assembled transcripts from the two different assembly strategies Soap *denovo*–Tran (a, c) and Trinity (b, d), which match secreted proteins after translation to amino acids.

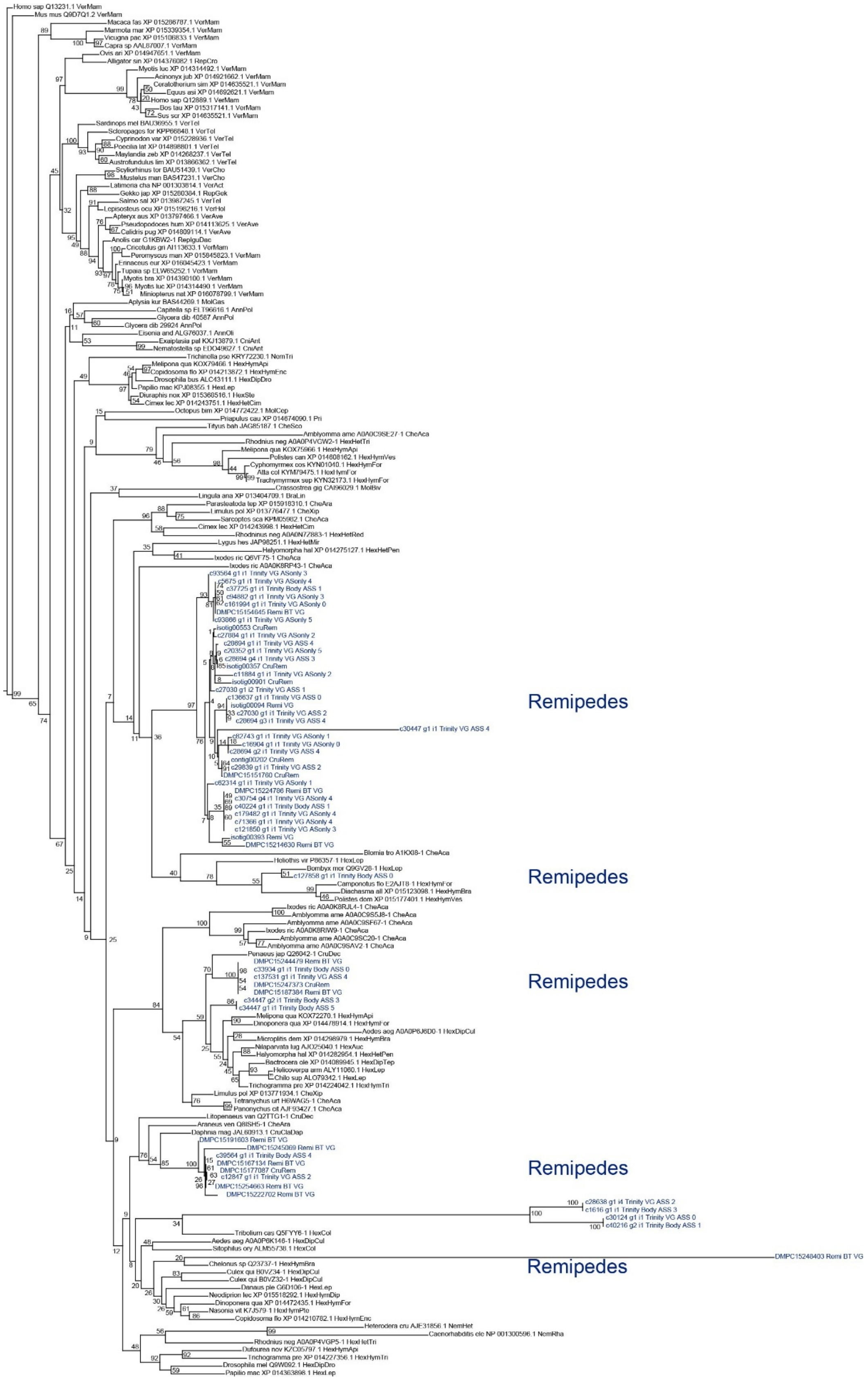

Figure S2. Phylogenetic tree reconstructed of all chitinase sequences. Only regions of the Domain were used unalignable signalpeptide and propetide regions were manually deleted. Tree was reconstructed using RAxML v8, 1000 BS, –f, a and applying the maximum likelihood criterion with the best fitting substitution model tested in Prottest (see material and methods). Please see additional supplementary files for all alignments.

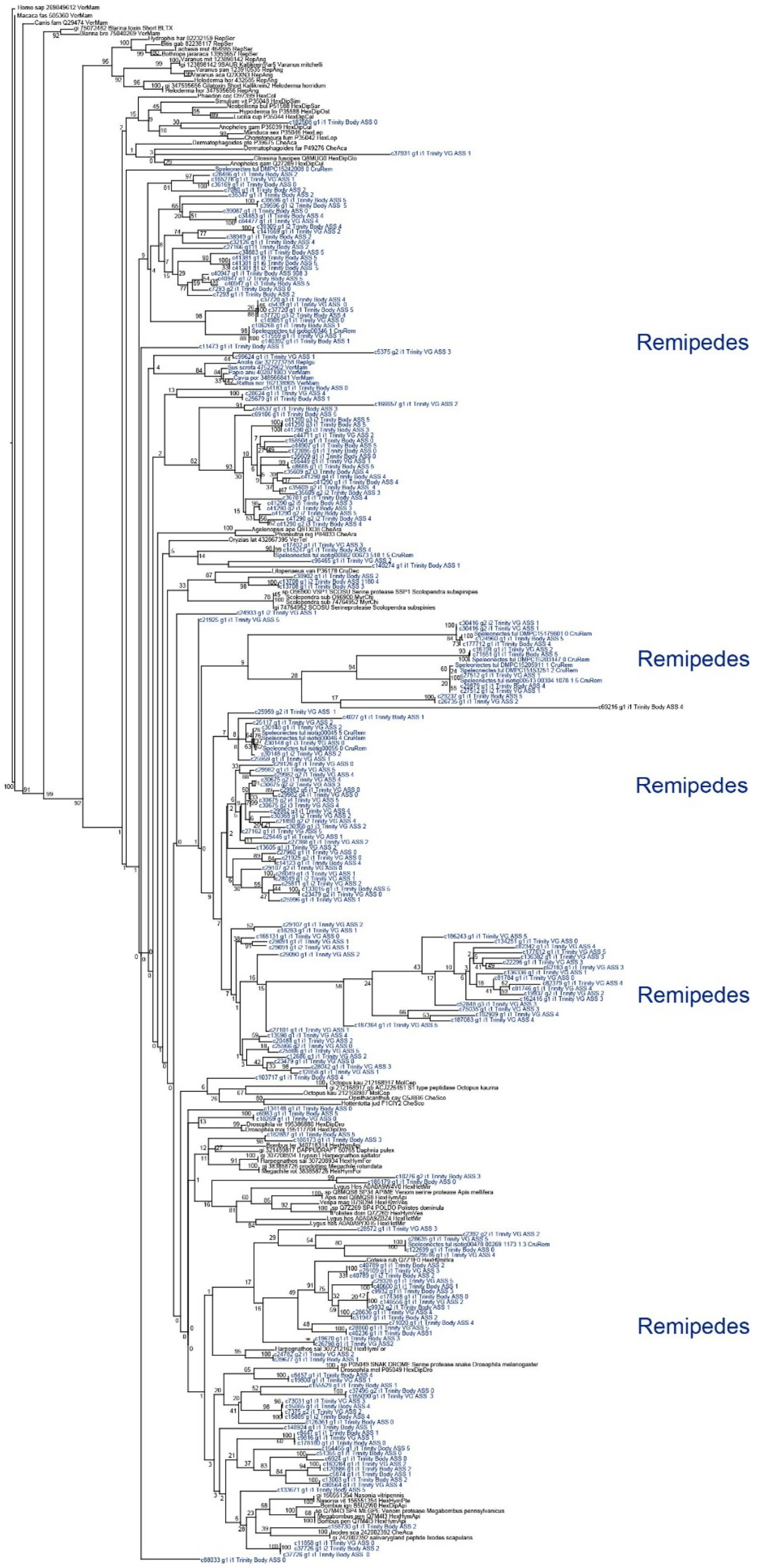

Figure S3. Phylogenetic tree reconstructed from an alignment of all peptidase S1 sequences. Only regions of the Domain were used and unalignable signalpeptide and propetide regions were manually deleted. Tree was reconstructed using RAxML v8, 1000 BS, –f a, applying the maximum likelihood criterion with the best fitting substitution model tested in Prottest (see material and methods). Please see additional supplementary files for all alignments.

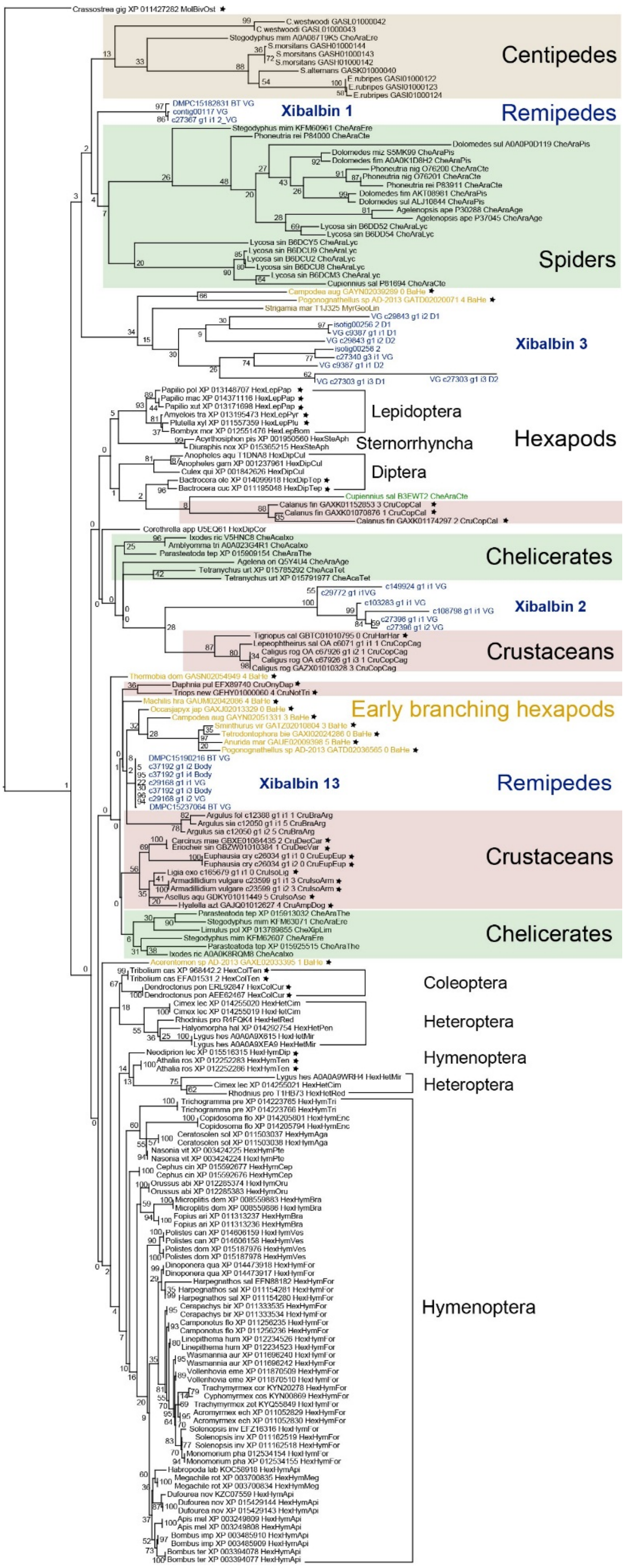

Figure S4. Phylogenetic tree reconstructed from an alignment of all dICK and ICK sequences. Only regions of the Domain were used and unalignable signalpeptide and propetide regions were manually deleted. Tree was reconstructed using RAxML v8, 1000 BS, –f a, applying the maximum likelihood criterion with the best fitting substitution model tested in Prottest (see material and methods). Please see additional supplementary files for all alignments.

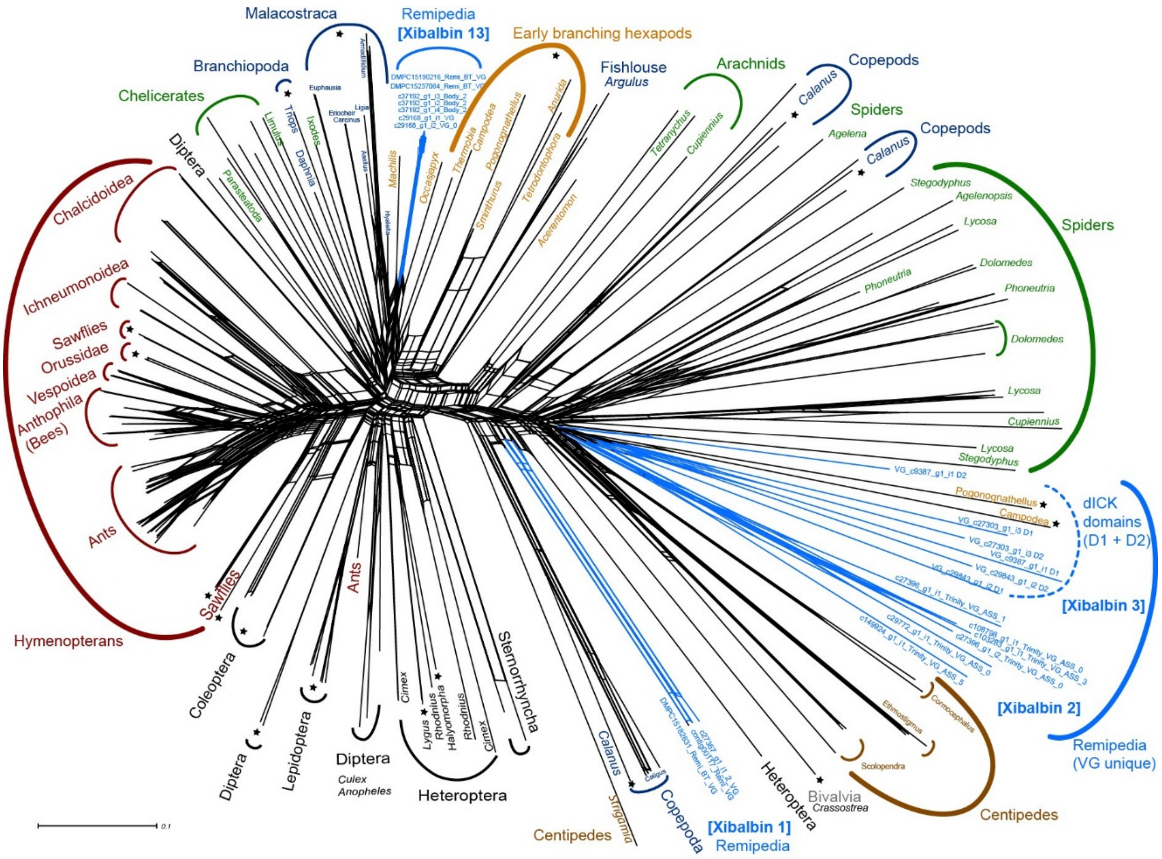

Figure S5. Neighbour joining network reconstructed from an alignment of all dICK and ICK sequences. Only regions of the Domain were used and unalignable signalpeptide and propetide regions were manually deleted. Network was reconstructed with SplitsTree. Please see additional supplementary files for all alignments.
