# Supplementary material for: Venomics of Remipede Crustaceans Reveals Novel Peptide Diversity and Illuminates the Venom’s Biological Role
Source: Toxins (Basel). 2017 Jul 26;9(8):234. doi: 10.3390/toxins9080234 (PMC5577568; doi:10.3390/toxins9080234)
Supplement: Supplementary file 1 [file Supplementary Tables.pdf › Supplementary Tables.pdf]

Supplementary Table 1: Venom gland proteomics

Gel Band	Mass (kDa)	Protein	Accession	Translation frame	Protein ID	SignalP	Peptides (>95%)	Expression (reads)	Coverage (%)	ProteinPilot Score (Total)
**1**	**60**	1	c29839_g1_i1	3	Chitinase	N	14	7378101	28.30	21.55
		2	c30148_g1_i2	3	Peptidase S1	Y	3	139510	14.18	5.37
		2	c30148_g1_i3	1	Peptidase S1	N	3	87362	18.18	5.37
		2	c30148_g1_i1	3	Peptidase S1	Y	3	364870	13.99	5.37
		2	c25117_g1_i1	3	Peptidase S1	N	2	301	29.10	4.03
		3	c30368_g1_i2	3	Peptidase S1 with LDLA domain	Y	2	995917	4.19	3.39
		3	c30675_g2_i2	4	Peptidase S1 with LDLA domain	Y	1	1228052	2.16	2
		3	c30675_g2_i1	5	Peptidase S1 with LDLA domain	N	1	434416	2.59	2
		3	c30675_g2_i4	6	Peptidase S1 with LDLA domain	N	1	933783	1.38	2
		3	c30675_g2_i3	5	Peptidase S1 with LDLA domain	Y	1	1612752	1.16	2
		3	c30368_g1_i3	3	Peptidase S1 with LDLA domain	Y	1	615193	1.39	2
		3	c29982_g3_i1	5	Peptidase S1 with LDLA domain	Y	1	381583	1.71	2
		3	c25446_g1_i4	2	Peptidase S1 with LDLA domain	N	1	14	7.29	2
**2**	**50**	1	c29839_g1_i1	3	Chitinase	N	8	7378101	21.44	10.24
		2	c27884_g1_i1	3	Chitinase	N	3	17222	18.18	3.76
		2	c28694_g4_i1	4	Chitinase	N	3	2471	13.30	3.33
		3	c30148_g1_i3	1	Peptidase S1	N	1	87362	12.46	3.23
		3	c30148_g1_i2	3	Peptidase S1	Y	1	139510	9.37	3.23
		3	c30148_g1_i1	3	Peptidase S1	Y	1	364870	9.58	3.23
		4	c57873_g1_i1	5	Thioredoxin	N	1	12	9.20	2.66
		5	c27020_g1_i1	4	CAP Protein	Y	1	67718	10.57	1.92
**3**	**40**	1	c29839_g1_i1	3	Chitinase	N	4	7378101	10.12	8.92
		2	c30148_g1_i3	1	Peptidase S1	N	3	87362	12.46	6
		2	c30148_g1_i2	3	Peptidase S1	Y	3	139510	9.37	6
		2	c30148_g1_i1	3	Peptidase S1	Y	3	364870	9.58	6
		2	c25117_g1_i1	3	Peptidase S1	N	2	301	14.93	4
		3	c27020_g1_i1	4	CAP Protein	Y	1	67718	4.57	2
**4**	**37**	1	c30148_g1_i2	3	Peptidase S1	Y	4	139510	13.67	8
		1	c30148_g1_i1	3	Peptidase S1	Y	4	364870	13.99	8
		1	c30148_g1_i3	1	Peptidase S1	N	3	87362	15.15	6
		2	c27577_g1_i3	3	Tropomyosin	N	2		10.21	4.62
		2	c27577_g1_i4	3	Tropomyosin	N	2		11.11	4.62
		3	c29839_g1_i1	3	Chitinase	N	2	7378101	3.09	3.12
		3	c28694_g3_i1	5	Chitinase	N	1	23414	7.58	1.4
		3	c28694_g2_i1	5	Chitinase	N	1	467	12.66	1.4
		3	c16904_g1_i1	1	Chitinase	N	1	0	13.33	1.4
**5**	**30**	1	c27020_g1_i1	4	CAP Protein	Y	35	67718	53.43	39.7
		2	c30148_g1_i3	1	Peptidase S1	N	4	87362	18.52	8.01
		2	c30148_g1_i2	3	Peptidase S1	Y	4	139510	11.39	8.01
		2	c30148_g1_i1	3	Peptidase S1	Y	4	364870	11.66	8.01
		3	c29839_g1_i1	3	Chitinase	N	3	7378101	5.49	4.01
		4	c30368_g1_i2	3	Peptidase S1 with LDLA domain	Y	3	995917	4.04	6
		4	c30675_g2_i3	5	Peptidase S1 with LDLA domain	Y	2	1612752	4.46	4
		4	c30675_g2_i2	4	Peptidase S1 with LDLA domain	Y	1	1228052	2.16	2
		4	c30675_g2_i1	5	Peptidase S1 with LDLA domain	N	1	434416	2.59	2
		4	c30368_g1_i3	3	Peptidase S1 with LDLA domain	Y	1	615193	1.39	2
		4	c29982_g3_i1	5	Peptidase S1 with LDLA domain	Y	1	381583	1.71	2
		4	c25446_g1_i4	2	Peptidase S1 with LDLA domain	N	1	14	7.29	2
		5	c18283_g1_i1	2	Prostasin	N	2	286704	8.79	4
		6	c29585_g1_i1	1	Chitin-binding domain containing protein	Y	2		3.28	3.48
		7	c27960_g1_i1	1	Peptidase S1	Y	3	316580	8.42	2.12
		8	c30675_g2_i4	6	Peptidase S1 with LDLA domain	N	2	933783	5.32	4
		8	c30675_g2_i3	5	Peptidase S1 with LDLA domain	Y	2	1612752	4.46	4
		9	c57873_g1_i1	5	Thioredoxin	N	1	12	4.73	2
		10	c26920_g1_i1	5	Calycin	Y	1		2.48	2
		11	c24110_g1_i1	2	Carboxypeptidase M14A	Y	1	189506	2.46	2
**6**	**26**	1	c30148_g1_i1	3	Peptidase S1	Y	11	364870	22.80	19.13
		2	c27020_g1_i1	4	CAP protein	Y	9	67718	27.43	17.56
		3	c29839_g1_i1	3	Chitinase	N	7	7378101	19.90	14.51
		4	c29126_g1_i1	1	Peptidase S1	Y	6	72458	27.89	8.43
		5	c25996_g1_i1	2	Peptidase S1 with LDLA domain	Y	4	488359	13.24	7.13
		6	c18283_g1_i1	2	Prostasin	N	10	286704	33.85	6.83
		7	c27388_g1_i1	3	Peptidase S1 with LDLA domain	Y	3	228907	8.82	5.89
		8	c30368_g1_i2	3	Peptidase S1 with LDLA domain	Y	8	995917	14.44	9.27
		9	c29090_g1_i1	3	Peptidase S1	Y	3	140303	9.45	4.96
		10	c30675_g2_i3	5	Peptidase S1 with LDLA domain	Y	6	1612752	17.02	5.93
		10	c30675_g2_i4	6	Peptidase S1 with LDLA domain	N	6	933783	19.13	5.93
		10	c30675_g2_i2	4	Peptidase S1 with LDLA domain	Y	5	1228052	13.46	3.68

		10	c30675_g2_i1	5	Peptidase S1 with LDLA domain	N	5	434416	14.97	3.68
		11	c12686_g1_i1	3	Peptidase S1	N	3	103249	10.67	4.79
		12	c29091_g1_i2	2	Peptidase S1	Y	2	216342	12.86	4
		12	c29091_g1_i1	2	Peptidase S1	Y	1	75244	4.68	2
		13	c11081_g1_i1	5	Peptidase S1	N	2		41.51	3.44
		14	c29982_g5_i1	1	Peptidase S1	N	2	29390	7.28	3.24
		14	c29982_g4_i1	1	Peptidase S1	N	2	118055	5.85	3.24
		15	c29982_g1_i1	6	Peptidase S1	N	2	196885	14.04	3.1
		16	c27101_g1_i1	2	Peptidase S1	N	3	140921	17.19	3.64
		17	c29982_g2_i1	5	Peptidase S1 with LDLA domain	Y	4	191528	11.97	2.73
		18	c29585_g1_i1	1	Chitin-binding domain containing protein	Y	2		7.29	2.37
		19	c28694_g4_i1	4	Chitinase	N	1	2471	13.83	2
		19	c27884_g1_i1	3	Chitinase	N	1	17222	14.69	2
		20	c27960_g1_i1	1	Peptidase S1	Y	3	316580	8.42	2
		21	c23479_g2_i1	1	Peptidase S1	N	2	68533	8.99	2
**7**	**25**	1	c30675_g2_i4	6	Peptidase S1 with LDLA domain	0.109	29	933783	26.82	28
		1	c30675_g2_i3	5	Peptidase S1 with LDLA domain	0.440 Y	29	1612752	22.48	28
		2	c30148_g1_i1	3	Peptidase S1	0.604 Y	14	364870	28.24	22.1
		2	c30148_g1_i2	3	Peptidase S1	0.602 Y	13	139510	25.82	22.1
		3	c18283_g1_i1	2	Prostasin	0.173	38	286704	39.79	24
		4	c30368_g1_i2	3	Peptidase S1 with LDLA domain	0.391 Y	20	995917	18.94	20.03
		5	c29982_g1_i1	6	Peptidase S1	0.098	7	196885	47.19	12
		6	c29839_g1_i1	3	Chitinase	0.21	6	7378101	9.61	12
		7	c29585_g1_i1	1	Chitin-binding domain containing protein	0.450 Y	5		9.84	10
		8	c27057_g1_i2	1	Calycin	0.882 Y	4		13.73	8.05
		8	c27057_g1_i1	1	Calycin	0.882 Y	4		13.48	8.05
		9	c27960_g1_i1	1	Peptidase S1	0.444 Y	10	316580	27.55	10.04
		10	c57873_g1_i1	5	Thioredoxin	0.183	5	12	13.93	8
		11	c13598_g1_i1	5	Peptidase S1	0.438 Y	5	775049	19.06	8.01
		12	c25996_g1_i1	2	Peptidase S1 with LDLA domain	0.731 Y	2	488359	16.81	4.01
		13	c27884_g1_i1	3	Chitinase	0.274	2	17222	14.69	4
		13	c28694_g4_i1	4	Chitinase	0.327	1	2471	6.38	2
		14	c136843_g1_i1	2	Chitin-binding domain containing protein	N	2		36.92	4
		15	c30148_g1_i3	1	Peptidase S1	0.328	11	87362	26.60	18.06
		16	c27030_g1_i2	2	Chitinase	0.107	4	4118	43.00	8
		16	c27030_g1_i1	3	Chitinase	0.112	3	18858	62.02	6
		17	c29982_g3_i1	5	Peptidase S1 with LDLA domain	0.791 Y	2	381583	7.58	4
		17	c29982_g2_i2	5	Peptidase S1 with LDLA domain	0.800 Y	1	88749	4.71	2
		18	c25446_g1_i4	2	Peptidase S1 with LDLA domain	0.126	3	14	16.67	4
		18	c30675_g2_i3	5	Peptidase S1 with LDLA domain	0.440 Y	29	1612752	22.48	28
		18	c30675_g2_i2	4	Peptidase S1 with LDLA domain	0.754 Y	20	1228052	14.45	20.03
		18	c30675_g2_i1	5	Peptidase S1 with LDLA domain	0.102	20	434416	17.37	20.03
		18	c25446_g1_i2	2	Peptidase S1 with LDLA domain	0.126	2	37	5.14	2
		18	c25446_g1_i1	2	Peptidase S1 with LDLA domain	0.126	2	39	6.62	2
		19	c27162_g1_i1	6	Peptidase S1	0.102	4	21533	20.95	4
		20	c27020_g1_i1	4	CAP protein	0.450 Y	1	67718	2.57	2
**8**	**20**	1	c29585_g1_i1	1	Chitin-binding domain containing protein	0.450 Y	4		13.66	5.1
		2	c30148_g1_i2	3	Peptidase S1	0.602 Y	1	139510	15.44	3.15
		2	c30148_g1_i3	1	Peptidase S1	0.328	1	87362	18.18	3.15
		2	c30148_g1_i1	3	Peptidase S1	0.604 Y	1	364870	13.99	3.15
		2	c25117_g1_i1	3	Peptidase S1	0.104	0	301	27.61	1.17
		3	c136843_g1_i1	2	Chitin-binding domain containing protein		1		46.15	2.11
**9**	**15**	1	c30148_g1_i1	3	Peptidase S1	0.602 Y	11	139510	16.96	9.5
		1	c30148_g1_i2	3	Peptidase S1	0.604 Y	11	364870	17.36	9.5
		1	c30148_g1_i3	1	Peptidase S1	0.328	11	87362	18.18	7.1
**10**	**12**	1	c30148_g1_i2	3	Peptidase S1	0.602 Y	4	139510	16.96	9.5
		1	c30148_g1_i1	3	Peptidase S1	0.604 Y	4	364870	17.36	9.5
		1	c30148_g1_i3	1	Peptidase S1	0.328	3	87362	18.18	7.1
		2	c29839_g1_i1	3	Chitinase	0.21	2	7378101	3.087	3.09
		2	c28694_g3_i1	5	Chitinase	0.107	1	23414	7.576	2
		2	c28694_g2_i1	5	Chitinase	0.112	1	467	12.66	2
		2	c16904_g1_i1	1	Chitinase	0.114	1	0	13.33	2
		3	c13428_g1_i2	3	13.6 kDa protein with no BLAST hits (UniProt KB)	0.506 Y	1		2.896	1.59
		3	c13428_g1_i1	3	13.6 kDa protein with no BLAST hits (UniProt KB)	0.506 Y	1		2.694	1.59

Supplementary Table 2: Venom gland tissue transcriptomics

Name of toxin protein family	ID of putative toxin contigs	Match via proteomics	Signal Peptide	Number of high-support contigs (reads > 25)	Lengths of contigs	Number of reads	FPKM
C1-like domain	c173255_g1_i1		yes	0 / 1	919	21	0.94
Calycin	c26920_g1_i1	yes	yes		1,158	81,138	2,880.98
	c27057_g1_i1	yes	yes		1,890	45,640	992.91
	c27057_g1_i2	yes	yes		1,847	35,449	789.15
	c23125_g1_i1	-	yes		1,018	297	12.00
	c27565_g1_i1	-	yes		1,017	206	8.33
	c24433_g1_i2	-	-		989	152	6.32
	c28078_g2_i1	-	yes		1,161	108	3.82
	c30135_g4_i1	-	yes		1,782	147	3.39
	c24433_g1_i3	-	-		594	45	3.11
	c1253_g1_i1	-	yes		1,246	78	2.57
	c28078_g1_i1	-	yes		2,080	119	2.35
	c83371_g1_i1	-	-		751	24	1.31
	c64218_g1_i1	-	-		595	11	0.76
	c179908_g1_i1	-	-		1,097	19	0.71
	c154025_g1_i1	-	-		383	1	0.11
Total (high-support contigs)				**11** / 15		**163,379**	**4,704.93**
CAP	c27020_g1_i1	yes	yes		1,048	67,718	2,656.86
	c17634_g1_i1	-	yes		1,032	25,221	1,004.86
	c30341_g1_i1	-	yes		1,218	17,300	584.01
	c136857_g1_i1	-	yes		1,159	580	20.58
	c30174_g1_i3	-	-		1,663	434	10.73
	c23191_g1_i1	-	yes		1,692	313	7.61
	c27364_g1_i1	-	-		1,296	215	6.82
	c35027_g1_i1	-	yes		1,689	98	2.39
	c29302_g2_i1	-	yes		1,937	104	2.21
	c7719_g1_i1	-	yes		646	28	1.78
	c1310_g1_i2	-	yes		1,641	61	1.53
	c29302_g1_i1	-	yes		1,580	57	1.48
	c22705_g2_i1	-	yes		450	4	0.37
	c169637_g1_i1	-	-		278	0	0.00
Total (high-support contigs)				**11** / 14		**112,072**	**4,299.37**
Chitinase	c29839_g1_i1	yes	yes		1,703	7,378,101	178,137.36
	c28694_g3_i1	yes	-		396	23,414	2,431.11
	c27030_g1_i1	yes	-		388	18,858	1,998.43
	c27884_g1_i1	yes	yes		424	17,222	1,670.10
	c136637_g1_i1	-	yes		311	9,301	1,229.69
	c27030_g1_i2	yes	-		300	4,118	564.40
	c28694_g4_i1	yes	yes		563	2,471	180.46
	c28694_g2_i1	yes	-		238	467	80.68
	c82743_g1_i1	-	-		169	144	35.03
	c28638_g1_i4	-	yes		1,750	821	19.29
	c11884_g1_i1	-	yes		440	85	7.94
	c30124_g1_i1	-	-		1,796	320	7.33
	c12847_g1_i1	-	-		1,341	72	2.21
	c28694_g1_i1	-	-		233	12	2.12
	c5675_g1_i1	-	-		1,109	40	1.48
	c137531_g1_i1	-	-		1,898	39	0.84
	c62314_g1_i1	-	-		203	4	0.81

	c121850_g1_i1	-	yes		633	8	0.52
	c94882_g1_i1	-	yes		326	4	0.50
	c30447_g1_i1	-	-		1,206	3	0.10
	c20352_g1_i1	-	yes		157	0	0.00
	c179482_g1_i1	-	-		262	0	0.00
	c71366_g1_i1	-	-		142	0	0.00
	c30754_g4_i1	-	-		246	0	0.00
	c161994_g1_i1	-	-		173	0	0.00
	c93866_g1_i1	-	-		163	0	0.00
	c16904_g1_i1	yes	-		225	0	0.00
	c93564_g1_i1	-	-		185	0	0.00
Total (high-support contigs)				**13** / 28		**7,455,394**	**186,364.04**
**Colipase-like**	c26403_g1_i1	-	yes		1,523	425	11.47
	c83533_g1_i1	-	-		632	6	0.39
	c69394_g1_i1	-	-		255	0	0.00
Total (high-support contigs)				**1** / 3		425	11.47
**Cystatin**	c30119_g1_i1	-	yes	**1**	2,699	**162**	**2.47**
**Hyaluronidase**	c28000_g2_i2	-	yes		1,299	630	19.94
	c28000_g2_i1	-	yes		1,299	347	10.98
	c26537_g1_i1	-	-		1,057	22	0.86
	c99311_g1_i1	-	-		491	8	0.67
	c26537_g1_i2	-	yes		477	7	0.60
	c84753_g1_i1	-	-		587	7	0.49
	c139045_g1_i1	-	yes		196	0	0.00
Total (high-support contigs)				**2** / 7		**977**	**30.93**
**Kazal**	c28644_g2_i2	-	yes		930	190	8.40
	c28848_g1_i2	-	-		1,797	333	7.62
	c28644_g2_i1	-	yes		539	68	5.19
	c28699_g2_i1	-	-		1,988	203	4.20
	c28699_g2_i2	-	-		1,973	200	4.17
	c30818_g8_i1	-	-		2,855	238	3.43
	c10145_g1_i1	-	-		1,333	65	2.00
	c30818_g8_i2	-	-		1,667	79	1.95
	c60227_g1_i1	-	yes		960	33	1.41
	c8461_g2_i1	-	-		1,381	40	1.19
	c22300_g1_i1	-	-		696	9	0.53
	c36870_g1_i1	-	-		447	3	0.28
Total (high-support contigs)				**8** /12		**1,376**	**36.95**
**Kunitz**	c26909_g1_i1	-	-		2,790	19,756	291.15
	c26026_g2_i2	-	-		1,815	482	10.92
	c21419_g1_i1	-	-		1,209	120	4.08
	c2037_g1_i1	-	-		1,527	111	2.99
	c115833_g1_i1	-	-		1,255	65	2.13
	c2789_g1_i1	-	yes		548	23	1.73
	c92205_g1_i1	-	-		712	10	0.58
	c112161_g1_i1	-	-		745	9	0.50
Total (high-support contigs)				**5** / 8		**20,534**	**311.27**
**Lectin**	c72_g1_i1	-	-		1,469	82	2.30
	c4773_g1_i1	-	-		916	32	1.44
	c71342_g1_i1	-	-		471	15	1.31
	c31264_g1_i1	-	yes		1,355	32	0.97
	c43104_g1_i1	-	-		1,067	7	0.27
	c111380_g1_i1	-	-		411	2	0.20

Total (high-support contigs)				**2** / 6		**114**	**3,73**
**Metallopeptidase M12**	c27520_g1_i1	-	yes		1,507	145,668	3,974.44
	c28947_g1_i1	-	yes		2,374	836	14.48
	c28592_g1_i2	-	yes		1,231	311	10.39
	c28592_g1_i1	-	-		1,276	298	9.60
	c16469_g1_i2	-	-		1,868	173	3.81
	c16469_g1_i1	-	-		1,917	174	3.73
	c37943_g1_i1	-	-		1,061	47	1.82
	c23132_g1_i1	-	-		1,012	38	1.54
	c140847_g1_i1	-	-		1,232	26	0.87
	c61570_g1_i1	-	-		547	7	0.53
	c61957_g1_i1	-	yes		432	5	0.48
	c46764_g1_i1	-	-		290	1	0.14
Total (high-support contigs)				**8** / 12		**147,545**	**4,019.82**
**Metallopeptidase M13**	c27666_g1_i1	-	-		2,487	17,601	291.00
	c20542_g2_i1	-	-		1,700	37	0.89
	c89912_g1_i1	-	-		688	13	0.78
	c90181_g1_i1	-	-		815	14	0.71
	c112272_g1_i1	-	-		1,408	23	0.67
	c48921_g1_i1	-	-		156	2	0.53
Total (high-support contigs)				**1** / 6		**17,601**	**291.00**
**Metallopeptidase M14**	c24110_g1_i1	yes	-		1,447	189,506	5,384.92
	c27520_g1_i1	-	-		1,507	145,668	3,974.44
	c27844_g2_i1	-	-		492	1,312	109.65
	c27232_g1_i1	-	yes		398	686	70.87
	c8150_g1_i1	-	yes		2,436	137	2.31
	c28686_g2_i1	-	-		1,401	47	1.38
	c12373_g1_i1	-	-		152	4	1.08
	c88597_g1_i1	-	-		220	4	0.75
	c137888_g1_i1	-	-		170	0	0.00
	c159732_g1_i1	-	-		159	0	0.00
Total (high-support contigs)				**5** / 10		**337,309**	**9,542.19**
**Peptidase S1**	c13598_g1_i1	yes	yes		1,205	775,049	26,446.44
	c29982_g1_i1	yes	-		534	196,885	15,159.90
	c30148_g1_i1	yes	yes		1,140	364,870	13,160.07
	c27960_g1_i1	yes	yes		1,144	316,580	11,378.43
	c18283_g1_i1	yes	-		1,179	286,704	9,998.73
	c29982_g4_i1	yes	-		563	118,055	8,621.86
	c29091_g1_i2	yes	yes		1,100	216,342	8,086.73
	c27101_g1_i1	yes	-		1,141	140,921	5,078.26
	c30148_g1_i2	yes	yes		1,147	139,510	5,001.12
	c30148_g1_i3	yes	-		883	87,362	4,068.06
	c12686_g1_i1	yes	-		1,200	103,249	3,537.77
	c28042_g1_i1	yes	yes		1,155	84,621	3,012.46
	c27162_g1_i1	yes	-		313	21,533	2,828.69
	c29091_g1_i1	yes	yes		1,102	75,244	2,807.47
	c29982_g5_i1	yes	-		447	29,390	2,703.44
	c23479_g2_i1	yes	-		1,060	68,533	2,658.39
	c29090_g1_i1	yes	yes		2,238	140,303	2,577.70
	c29126_g1_i1	yes	yes		1,184	72,458	2,516.28
	c30416_g2_i1	-	yes		1,898	82,423	1,785.57
	c30416_g2_i2	-	yes		3,809	121,765	1,314.43
	c23479_g1_i1	-	-		453	9,314	845.40
	c29107_g2_i1	-	-		2,214	32,715	607.57

c13605_g1_i1	-	-	1,660	18,211	451.08
c17559_g1_i1	-	-	1,939	12,556	266.26
c21925_g2_i1	-	-	459	2,417	216.52
c25966_g1_i1	-	-	831	3,766	186.34
c20484_g1_i1	-	-	656	2,519	157.89
c21925_g1_i1	-	-	323	857	109.09
c28635_g1_i1	-	-	1,203	3,080	105.27
c25966_g2_i1	-	-	438	1,015	95.28
c29107_g1_i1	-	-	631	1,109	72.26
c17402_g1_i1	-	-	2,191	2,822	52.96
c26798_g1_i1	-	-	1,501	1,589	43.53
c165131_g1_i1	-	-	369	310	34.54
c25959_g2_i1	-	yes	522	424	33.40
c25117_g1_i1	yes	-	403	301	30.71
c27512_g1_i1	-	-	1,535	1,124	30.11
c27512_g1_i2	-	-	1,525	1,088	29.33
c29516_g1_i1	-	-	1,570	1,069	28.00
c163284_g1_i1	-	-	1,774	1,156	26.79
c18269_g1_i1	-	-	1,960	1,099	23.06
c19800_g1_i1	-	-	1,609	857	21.90
c25959_g1_i1	-	-	390	161	16.97
c28636_g1_i1	-	-	1,626	511	12.92
c26735_g1_i1	-	-	1,274	312	10.07
c28049_g1_i1	-	-	1,051	235	9.19
c21890_g2_i2	-	-	365	63	7.10
c28624_g1_i1	-	-	1,964	301	6.30
c24782_g2_i1	-	-	2,510	370	6.06
c28572_g1_i1	-	-	1,648	242	6.04
c28860_g1_i1	-	-	1,745	229	5.40
c28049_g1_i2	-	-	1,280	165	5.30
c29109_g1_i1	-	-	2,327	261	4.61
c29328_g1_i1	-	-	2,864	279	4.01
c16194_g1_i1	-	-	1,473	110	3.07
c12858_g1_i1	-	-	241	13	2.22
c25811_g1_i2	-	-	491	24	2.01
c5375_g2_i1	-	-	738	26	1.45
c165278_g1_i1	-	-	1,159	39	1.38
c24933_g1_i2	-	-	1,751	56	1.32
c90564_g1_i1	-	-	990	29	1.20
c7375_g2_i1	-	-	1,534	32	0.86
c5439_g1_i1	-	-	697	14	0.83
c141669_g1_i1	-	-	767	13	0.70
c9816_g1_i1	-	-	1,297	20	0.63
c44711_g1_i1	-	-	458	7	0.63
c99624_g1_i1	-	-	378	5	0.54
c165090_g1_i1	-	-	1,079	13	0.50
c37931_g1_i1	-	-	425	4	0.39
c56449_g1_i1	-	-	861	8	0.38
c166657_g1_i1	-	-	705	6	0.35
c2392_g2_i1	-	-	486	4	0.34
c11858_g1_i1	-	-	740	6	0.33
c148556_g1_i1	-	-	686	5	0.30
c96465_g1_i1	-	-	562	3	0.22
c187364_g1_i1	-	-	314	1	0.13

	c19937_g2_i1	-	yes		336	1	0.12
	c134251_g1_i1	-	-		313	0	0.00
	c136336_g1_i1	-	-		193	0	0.00
	c136382_g1_i1	-	yes		249	0	0.00
	c149051_g1_i1	-	yes		256	0	0.00
	c162416_g1_i1	-	yes		179	0	0.00
	c177612_g1_i1	-	yes		326	0	0.00
	c182909_g1_i1	-	-		289	0	0.00
	c186243_g1_i1	-	-		141	0	0.00
	c187083_g1_i1	-	-		381	0	0.00
	c22296_g1_i1	-	-		404	0	0.00
	c52848_g3_i1	-	yes		204	0	0.00
	c62183_g1_i1	-	-		368	0	0.00
	c73031_g1_i1	-	-		153	0	0.00
	c75035_g1_i1	-	-		168	0	0.00
	c81746_g1_i1	-	-		179	0	0.00
	c81784_g1_i1	-	-		159	0	0.00
	c82342_g1_i1	-	-		220	0	0.00
	c82379_g1_i1	-	-		340	0	0.00
	c84477_g1_i1	-	-		245	0	0.00
Total (high-support contigs)				**55** / 96		**3,544,434**	**136,306.15**
**Peptidase S1 - LDLA domain**	c30675_g2_i3	yes	yes		1,795	1,612,752	36,942.66
	c30675_g2_i2	yes	yes		1,752	1,228,052	28,820.91
	c30675_g2_i4	yes	-		1,508	933,783	25,460.66
	c30368_g1_i2	yes	yes		1,910	995,917	21,439.51
	c30368_g1_i3	yes	yes		1,480	615,193	17,091.29
	c25996_g1_i1	yes	yes		1,410	488,359	14,241.15
	c29982_g3_i1	yes	yes		1,222	381,583	12,839.34
	c30675_g2_i1	yes	yes		1,456	434,416	12,267.88
	c29982_g2_i1	yes	yes		1,128	191,528	6,981.49
	c27388_g1_i1	yes	yes		2,016	228,907	4,668.67
	c25446_g1_i4	yes	-		288	14	2.00
Total (high-support contigs)				**10** / 11		**7,110,490**	**180,753.57**
**Peptidase S10**	c29986_g1_i1	-	-		2,346	181,822	3,186.72
	c29849_g1_i1	-	-		3,440	512	6.12
	c29473_g5_i1	-	-		1,877	107	2.34
	c103962_g1_i1	-	-		183	2	0.45
Total (high-support contigs)				**3** / 4		**182,441**	**3,195.18**
**Phospholipase A2**	c25267_g1_i1	-	yes		1,787	152	3.50
	c26550_g1_i1	-	-		932	60	2.65
	c17764_g1_i1	-	yes		1,612	38	0.97
	c1144_g1_i1	-	-		1,014	23	0.93
	c37684_g1_i1	-	-		259	0	0.00
Total (high-support contigs)				**2** / 5		**212**	**6.15**
**Serpin**	c26358_g2_i1	-	yes		1,506	5,106	139.41
	c21812_g1_i1	-	yes		1,612	72	1.84
	c17839_g1_i2	-	-		1,367	31	0.93
	c17839_g1_i1	-	-		1,821	38	0.86
	c156108_g1_i1	-	-		146	0	0.00
Total (high-support contigs)				**4** / 5		**5,178**	**141.25**
**Sphingomyelinase**	c60968_g1_i1	-	yes		1,224	33	1.11
	c88623_g1_i1	-	-		851	22	1.06
	c88354_g1_i1	-	-		837	3	0.15

Total (high-support contigs)				**0** / 3		**33**	**1.11**
**VEGF-like**	c29360_g3_i1	yes	yes		1,609	234,789	5,999.94
	c26353_g1_i1	-	yes		2,323	1,095	19.38
	c28217_g1_i2	-	yes		2,457	234	3.92
	c28217_g1_i1	-	yes		2,481	236	3.91
	c17785_g1_i2	-	yes		1,041	37	1.46
	c113445_g1_i1	-	-		968	22	0.93
	c90144_g1_i1	-	yes		972	13	0.55
	c182771_g1_i1	-	-		260	0	0.00
Total (high-support contigs)				**4** / 8		**236,354**	**6,027.15**
**Xibalbin 1 (ICK)**	c27367_g1_i1	yes	yes		728	126,395	7,138.77
Total (high-support contigs)				**1** / 1		**126,395**	**7,138.77**
**Xibalbin 2 (putative ICK)**	c29772_g1_i1	yes	yes		773	1,412,448	75,130.80
	c149924_g1_i1	-	yes		584	12	0.84
	c27396_g1_i1	-	yes		354	0	0.00
	c27396_g1_i2	-	-		293	0	0.00
	c103283_g1_i1	-	-		158	0	0.00
	c108798_g1_i1	-	-		181	0	0.00
Total (high-support contigs)				**1** / 6		**1,412,448**	**75,130.80**
**Xibalbin 3 (dICK)**	c9387_g1_i1	yes	yes		790	145,444	7,569.96
	c29843_g1_i2	yes	yes		1,640	151,439	3,796.81
	c27340_g3_i1	-	-		328	116	14.54
	c27303_g1_i2	-	yes		588	124	8.67
	c27303_g1_i3	-	yes		909	130	5.88
	c27303_g1_i1	-	yes		328	11	1.38
Total (high-support contigs)				**5** / 6		**297,253**	**11,395.87**
**Xibalbin 4 (putative CSab)**	c18306_g1_i1	yes	yes		781	324,301	17,073.49
	c26085_g1_i1	-	yes		1070	3,633	139.61
	c26882_g2_i1	-	yes		1,062	2,458	95.17
	c30070_g1_i1	-	yes		737	288	16.07
	c30070_g1_i2	-	yes		1,535	258	6.91
	c5499_g1_i1	-	yes		818	79	3.97
Total (high-support contigs)				**6** / 6		**331,017**	**17,335.21**
**Xibalbin 5 (putative CSab)**	c28491_g1_i1	yes	yes		647	17,940	1,140.10
	c74570_g1_i1	-	-		297	2	0.28
Total (high-support contigs)				**1** / 2		**17,940**	**1,140.10**
**Xibalbin 6 (CHH/ITP)**	c13591_g1_i1	yes	-	**1**	1,321	**3,443**	**107.17**
**Xibalbin 7 (sIGFBP-rp)**	c13811_g1_i2	-	yes		632	55	3.58
	c25812_g1_i1	-	yes		1,601	131	3.36
	c13811_g1_i1	-	yes		601	46	3.15
	c26493_g1_i3	-	yes		1,692	123	2.99
	c23977_g2_i2	yes	yes		665	23	1.42
	c26493_g1_i2	-	yes		890	22	1.02
	c23977_g2_i1	yes	yes		687	9	0.54
Total (high-support contigs)				**4** / 7		**355**	**13.08**
**Xibalbin 8 (Unknown 1)**	c23843_g2_i1	yes	yes		684	708	42.56
	c77249_g1_i1	-	yes		301	1	0.14
Total (high-support contigs)				**1** / 2		**708**	**42.56**
**Xibalbin 09 (Unknown 2)**	c23666_g1_i1	yes	yes	**1**	887	**524,955**	**24,334.56**
**Xibalbin 10 (Unknown 3)**	c25771_g1_i2	yes	yes		566	329,600	23,943.95
	c25771_g1_i1	-	yes		545	180,730	13,635.11
Total (high-support contigs)				**2** / 2		**510,330**	**37,579.07**

**Xibalbin 11 (putative linear two-domain transcript)**	c29407_g1_i2	yes	yes		715	564,966	32,489.37
	c29407_g1_i1	yes	yes		1,028	345,028	13,800.23
	c27729_g1_i1	-	yes		698	32,169	1,894.99
	c27729_g1_i2	-	yes		778	6,300	332.96
Total (high-support contigs)				**4** / **4**		**948,463**	**48,517.55**
**Xibalbin 12 (Unknown)**	c27075_g1_i1	-	yes		411	20,417	2,042.56
	c27075_g1_i2	-	yes		339	14,112	1,711.65
Total (high-support contigs)				**2** / **2**		**34,529**	**3,754.21**
**Xibalbin 13 (ICK)**	c29168_g1_i2	-	yes		1,966	597	12.49
	c29168_g1_i1	-	yes		2,050	519	10.41
Total (high-support contigs)				**2** / **2**	4,016	**1,116**	**22.90**

Supplementary Table 3: Whole body transcriptomics

Name of toxin protein family	ID of putative toxin contigs	Signal Peptide	Number of high-support contigs (reads > 25)	Lengths of contigs	Number of reads	FPKM
**C1-like domain**	c68643_g1_i1	yes		1,071	84	8.64
	c31704_g1_i1	yes		1,198	46	4.23
	c156732_g1_i1	yes		537	16	3.28
Total			**2** / 3		**130**	**12.86**
**Calycin**	c42271_g1_i1	yes		733	25,283	3,797.90
	c39523_g1_i2	yes		1,110	10,189	1,010.71
	c37020_g1_i1	yes		906	8,303	1,009.08
	c41349_g4_i2	yes		1,570	10,951	768.02
	c39523_g1_i1	yes		938	5,923	695.28
	c1872_g1_i1	yes		2,642	12,459	519.24
	c40613_g2_i1	yes		775	3,498	496.98
	c14409_g1_i1	yes		741	2,921	434.04
	c39556_g2_i2	yes		881	1,665	208.09
	c1964_g1_i1	yes		1,001	1,794	197.34
	c38227_g1_i1	yes		1,544	2,316	165.16
	c40613_g1_i1	yes		1,435	1,960	150.39
	c29444_g2_i2	yes		656	829	139.15
	c38916_g1_i1	yes		2,725	1,960	79.20
	c38227_g1_i4	yes		390	276	77.92
	c30222_g1_i2	yes		1,169	534	50.30
	c40953_g3_i1	yes		1,971	855	47.76
	c39950_g3_i1	yes		980	268	30.11
	c30968_g2_i1	yes		1,153	309	29.51
	c38227_g1_i3	-		1,031	276	29.48
	c33342_g1_i1	yes		986	260	29.03
	c28551_g1_i1	yes		784	118	16.57
	c39950_g4_i1	yes		2,120	314	16.31
	c43200_g1_i1	yes		659	59	9.86
	c43013_g1_i1	yes		1,112	89	8.81
	c33878_g1_i1	yes		616	32	5.72
	c6754_g1_i1	-		637	30	5.19
	c56731_g1_i1	-		267	9	3.71
	c163658_g1_i1	-		284	8	3.10
	c98630_g1_i1	yes		762	20	2.89
	c153149_g1_i1	yes		276	6	2.39
	c79065_g1_i1	-		273	4	1.61
Total			**27** / 32		**93,471**	**10,027.14**
**CAP**	c39648_g1_i1	yes		1,398	8,621	679.00
	c39316_g1_i1	yes		2,531	7,693	334.67
	c36696_g1_i1	yes		1,533	946	67.95
	c19356_g1_i1	yes		1,588	829	57.48
	c40495_g1_i2	-		1,427	609	46.99
	c35351_g1_i1	yes		967	290	33.02
	c30140_g1_i2	yes		1,371	139	11.16
	c29338_g1_i1	-		871	77	9.73

	c39513_g1_i1	yes		1,743	147	9.29
	c105183_g1_i1	-		948	49	5.69
	c120579_g1_i1	yes		674	20	3.27
	c179430_g1_i1	-		286	8	3.08
	c185465_g1_i1	-		286	5	1.92
	c101573_g1_i1	-		495	6	1.33
Total			**10** / **14**		**19,400**	**1,254.99**
**Chitinase**	c39564_g1_i1	yes		1,962	47,087	2,642.53
	c33934_g1_i1	yes		2,060	18,169	971.14
	c40224_g1_i1	yes		2,314	801	38.11
	c40216_g2_i1	yes		1,607	466	31.93
	c37725_g1_i1	yes		2,184	484	24.40
	c1616_g1_i1	yes		1,652	224	14.93
	c34447_g2_i1	-		661	56	9.33
	c34447_g1_i1	-		937	41	4.82
	c127858_g1_i1	-		299	8	2.95
Total			**8** / **9**		**67,328**	**3,737.20**
**Colipase-like**	c96280_g1_i1	yes		926	1,144	136.03
	c126351_g1_i1	yes		834	60	7.92
	c98001_g1_i1	yes		1,359	69	5.59
	c127271_g1_i1	yes		694	26	4.13
Total			**4 / 4**		**1,299**	**153.67**
**Cystatin**	c37574_g1_i1	-		2,700	1,065	43.43
	c8661_g1_i1	-		2,206	105	5.24
	c99564_g1_i1	-		1,091	36	3.63
	c77886_g1_i1	-		150	0	0.00
Total			**3** / **4**		**1,206**	**52.31**
**Hyaluronidase**	c40379_g1_i2	yes		1,184	151	14.04
	c31967_g2_i1	yes		1,211	119	10.82
	c40379_g1_i1	yes		1,284	102	8.75
	c28820_g1_i1	yes		264	9	3.75
	c47494_g1_i1	-		366	8	2.41
	c43222_g1_i1	-		400	6	1.65
	c111275_g1_i1	-		332	4	1.33
Total			**3** / **7**		**372**	**33.61**
**Kazal**	c40841_g1_i1	-		652	5,084	858.57
	c40841_g2_i1	-		1,681	12,103	792.76
	c40674_g1_i1	-		546	2,965	597.93
	c73313_g1_i1	yes		774	1,436	204.28
	c38149_g1_i1	yes		1,001	1,409	154.99
	c38149_g1_i2	yes		957	833	95.84
	c37585_g1_i2	-		1,534	549	39.41
	c40913_g3_i1	-		3,085	755	26.95
	c40913_g3_i2	-		2,595	509	21.60
	c29394_g1_i1	yes		1,365	201	16.21
	c151081_g1_i1	-		826	66	8.80
	c113609_g1_i1	yes		344	8	2.56
	c13750_g2_i1	-		618	13	2.32
	c8253_g2_i1	-		717	13	2.00
	c95379_g1_i1	yes		407	6	1.62
	c69644_g1_i1	-		293	3	1.13

Total			**11** / **16**		**25,910**	**2,817.34**
**Kunitz**	c41394_g2_i1	-		6,022	13,902	254.19
	c39736_g1_i1	-		2,724	2,103	85.01
	c36647_g1_i1	-		1,175	901	84.43
	c40229_g1_i1	-		3,064	1,703	61.20
	c39884_g2_i1	-		2,615	942	39.66
	c40838_g1_i2	-		3,230	1,016	34.63
	c40838_g1_i1	-		3,240	1,002	34.05
	c40913_g3_i1	-		3,085	755	26.95
	c40883_g1_i1	-		3,576	741	22.82
	c40913_g3_i2	-		2,595	509	21.60
	c37109_g1_i2	-		2,559	206	8.86
	c6455_g1_i1	-		1,089	82	8.29
	c17588_g1_i1	yes		2,247	120	5.88
	c99819_g1_i1	-		680	23	3.72
	c43688_g1_i1	-		1,297	43	3.65
	c175170_g1_i1	-		906	27	3.28
	c149176_g1_i1	-		1,191	35	3.24
	c31027_g3_i1	-		534	11	2.27
	c124181_g1_i1	yes		454	5	1.21
Total			**16** / **19**		**24,087**	**697.74**
**Lectin**	c3306_g1_i1	yes		1,020	11,353	1,225.54
	c38034_g1_i2	yes		903	7,367	898.30
	c38034_g1_i1	-		720	5,185	792.93
	c41421_g5_i2	-		2,744	14,360	576.22
	c33884_g1_i1	-		1,899	6,806	394.63
	c121397_g1_i1	-		1,474	770	57.52
	c39626_g1_i1	yes		1,482	261	19.39
	c41669_g1_i1	-		1,665	238	15.74
	c40169_g2_i1	-		1,149	142	13.61
	c34169_g1_i1	-		914	95	11.44
	c146039_g1_i1	-		1,111	110	10.90
	c173595_g1_i1	-		3,140	202	7.08
	c40169_g1_i1	-		1,082	60	6.11
	c23063_g1_i2	-		769	41	5.87
	c69970_g1_i1	yes		1,261	64	5.59
	c38285_g1_i1	-		1,897	92	5.34
	c174585_g1_i1	-		481	22	5.04
	c6021_g1_i1	-		457	20	4.82
	c80582_g1_i1	-		466	20	4.73
	c31371_g3_i1	-		405	17	4.62
	c44412_g1_i1	-		687	27	4.33
	c45488_g1_i1	-		695	27	4.28
	c31371_g1_i1	-		417	15	3.96
	c146790_g1_i1	yes		582	20	3.78
	c23063_g1_i1	-		732	25	3.76
	c46549_g1_i1	-		431	14	3.58
	c121773_g1_i1	-		436	14	3.54
	c31738_g2_i1	-		287	9	3.45
	c32349_g1_i1	-		417	13	3.43
	c95417_g1_i1	-		882	22	2.75

	c31371_g2_i1	-		322	8	2.74
	c122717_g1_i1	-		728	17	2.57
	c174091_g1_i1	-		372	8	2.37
	c147606_g1_i1	-		1,008	21	2.29
	c12041_g1_i1	-		918	19	2.28
	c12041_g1_i1	-		918	19	2.28
	c32349_g2_i1	-		306	6	2.16
	c46354_g1_i1	-		469	9	2.11
	c111370_g1_i1	yes		433	8	2.03
	c121716_g1_i1	-		715	13	2.00
	c114781_g1_i1	-		351	6	1.88
	c148179_g1_i1	-		247	4	1.78
	c55614_g1_i1	-		262	4	1.68
	c183385_g1_i1	-		334	5	1.65
	c125283_g1_i1	-		271	4	1.63
	c105897_g1_i1	-		475	7	1.62
	c95339_g1_i1	-		277	4	1.59
	c25755_g1_i1	-		208	3	1.59
	c15023_g1_i1	-		169	2	1.30
	c100372_g1_i1	-		234	2	0.94
	c18393_g1_i1	-		187	0	0.00
Total			**19 / 51**		**47,225**	**4,058.58**
**Metallopeptidase M12**	c38819_g1_i1	yes		1,853	11,265	669.38
	c40821_g1_i3	yes		1,859	4,370	258.83
	c40821_g1_i2	yes		1,829	4,281	257.72
	c40821_g1_i1	yes		1,790	4,056	249.50
	c39128_g1_i1	yes		2,799	5,379	211.60
	c38245_g1_i1	yes		1,518	1,950	141.44
	c38587_g1_i1	yes			834	106.90
	c36638_g2_i1	-		672	596	97.66
	c29881_g1_i1	yes		1,101	626	62.60
	c39901_g1_i1	yes		3,065	1,633	58.66
	c29881_g1_i2	-		1,245	622	55.01
	c7262_g1_i1	yes		1,914	595	34.23
	c38943_g1_i3	-		4,882	1,201	27.09
	c38943_g1_i2	-		4,894	1,149	25.85
	c38192_g1_i1	-		2,134	131	6.76
	c125508_g1_i1	yes		1,258	69	6.04
	c20701_g1_i1	-		787	30	4.20
	c6166_g1_i1	-		1,485	54	4.00
	c5983_g1_i1	-		589	12	2.24
	c38192_g2_i1	yes		624	11	1.94
	c134572_g1_i1	-		479	8	1.84
	c48691_g1_i1	-		360	6	1.84
	c156237_g1_i1	-		434	7	1.78
	c51615_g1_i1	yes		400	6	1.65
	c143471_g1_i1	-		215	3	1.54
Total			**19 / 25**		**38,853**	**2,279.72**
**Metallopeptidase M13**	c40941_g3_i1	yes		3,871	2,596	73.84
	c38812_g2_i1	-		3,652	434	13.09
	c38809_g1_i1	-		747	129	19.01

	c38809_g2_i1	-		2,276	320	15.48
	c36771_g1_i1	-		2,751	219	8.77
	c96701_g1_i1	-		1,994	147	8.12
	c28676_g1_i1	-		2,181	108	5.45
	c158946_g1_i1	-		243	7	3.17
	c120779_g1_i1	-		709	18	2.80
	c121332_g1_i1	-		643	16	2.74
	c122940_g1_i1	-		600	13	2.39
	c80464_g1_i1	-		505	9	1.96
	c29581_g3_i1	-		408	7	1.89
	c140065_g1_i1	-		464	7	1.66
	c137305_g1_i1	-		272	4	1.62
	c11088_g1_i1	-		445	6	1.48
	c147180_g1_i1	-		310	4	1.42
	c89109_g1_i1	-		286	3	1.15
	c22820_g1_i1	-		151	1	0.73
	c29581_g2_i1	-		196	1	0.56
	c22820_g2_i1	-		198	1	0.56
Total			**8** / 21		**3,966**	**146.14**
**Metallopeptidase M14**	c39004_g1_i2	-		1,365	2,573	207.55
	c96470_g1_i1	-		1,804	2,962	180.79
	c39046_g1_i1	-		4,531	622	15.12
	c34863_g1_i2	yes		3,109	229	8.11
	c29585_g1_i1	-		832	54	7.15
	c125508_g1_i1	-		1,258	69	6.04
	c74890_g1_i1	-		725	28	4.25
	c7622_g1_i1	-		317	10	3.47
	c151706_g1_i1	-		260	7	2.96
	c72727_g1_i1	-		351	9	2.82
	c85284_g1_i1	-		253	6	2.61
	c43522_g1_i1	-		344	6	1.92
	c48691_g1_i1	-		360	6	1.84
	c69660_g1_i1	-		399	6	1.66
	c107555_g1_i1	yes		203	0	0.00
Total			**7** / 15		**6,537**	**429.00**
**Peptidase S1**	c40947_g1_i1	-		908	26,544	3,218.84
	c40947_g1_i2	-		916	20,101	2,416.24
	c35347_g1_i1	-		981	17,078	1,916.84
	c40947_g1_i3	-		1,324	17,711	1,472.90
	c34883_g1_i1	-		994	13,011	1,441.26
	c36169_g1_i1	-		1,422	16,211	1,255.25
	c29879_g1_i1	-		1,522	6,271	453.67
	c39087_g1_i1	-		1,009	3,208	350.08
	c6983_g1_i1	-		1,657	3,013	200.21
	c40600_g1_i1	-		3,335	5,274	174.13
	c41381_g1_i2	-		2,480	3,839	170.45
	c41381_g1_i6	-		2,521	3,764	164.40
	c41381_g1_i9	-		2,484	3,685	163.34
	c28486_g1_i1	-		1,098	1,517	152.13
	c40236_g1_i1	-		1,698	1,860	120.61
	c29237_g1_i1	-		1,203	1,309	119.81

c40789_g1_i2	-	2,474	1,759	78.29
c40789_g1_i1	-	2,516	1,698	74.31
c38949_g1_i1	-	1,486	932	69.06
c13708_g1_i2	-	1,180	678	63.27
c7080_g1_i1	-	1,076	482	49.32
c13708_g1_i1	-	1,305	549	46.32
c39677_g1_i1	-	2,028	828	44.96
c31947_g1_i1	-	1,492	472	34.83
c27166_g1_i1	-	1,619	509	34.62
c38902_g1_i1	-	1,516	411	29.85
c39596_g1_i2	-	2,554	596	25.69
c120886_g1_i1	-	1,683	363	23.75
c8457_g1_i1	-	1,469	316	23.69
c39309_g1_i2	-	1,098	230	23.06
c41290_g3_i2	-	3,125	642	22.62
c37720_g1_i1	-	598	116	21.36
c39596_g1_i1	-	2,347	441	20.69
c34483_g1_i1	-	958	172	19.77
c37726_g1_i1	-	1,490	260	19.21
c37726_g1_i2	-	1,660	286	18.97
c37720_g3_i1	-	861	115	14.71
c41290_g2_i3	-	2,285	252	12.14
c7293_g1_i1	-	665	71	11.76
c37720_g3_i2	-	1,131	116	11.29
c8665_g1_i1	-	933	88	10.39
c32126_g1_i1	-	1,186	71	6.59
c7293_g2_i1	-	219	13	6.54
c25679_g1_i1	-	1,196	67	6.17
c15885_g1_i2	-	1,440	76	5.81
c15885_g1_i1	-	1,404	70	5.49
c6924_g1_i1	-	828	40	5.32
c134148_g1_i1	-	562	25	4.90
c41290_g1_i1	-	452	20	4.87
c145247_g1_i1	-	1,424	63	4.87
c37495_g2_i1	-	389	17	4.81
c10276_g2_i1	-	507	22	4.78
c35609_g2_i1	-	595	25	4.63
c122699_g1_i1	-	765	31	4.46
c14123_g1_i1	-	374	15	4.42
c44907_g1_i1	-	824	33	4.41
c9932_g2_i1	-	433	17	4.32
c148924_g1_i1	-	318	12	4.16
c36781_g1_i1	-	717	27	4.15
c35609_g1_i1	-	772	26	3.71
c69106_g1_i1	-	331	11	3.66
c158730_g1_i1	-	638	21	3.62
c106268_g1_i1	-	465	14	3.32
c35609_g2_i3	-	574	17	3.26
c154455_g1_i1	-	410	12	3.22
c124960_g1_i1	-	1,131	33	3.21
c126361_g1_i1	-	343	10	3.21

	c51355_g1_i1	-		279	8	3.16
	c5874_g1_i1	-		532	15	3.10
	c44537_g1_i1	-		836	23	3.03
	c103717_g1_i1	-		342	9	2.90
	c54183_g1_i1	-		687	18	2.88
	c11473_g1_i1	-		325	8	2.71
	c35609_g2_i2	-		501	12	2.64
	c177712_g1_i1	-		350	8	2.52
	c13003_g1_i1	-		396	9	2.50
	c71551_g1_i1	-		269	6	2.46
	c8447_g1_i1	-		866	19	2.42
	c140392_g1_i1	-		289	6	2.29
	c9932_g1_i1	-		167	3	1.98
	c155529_g1_i1	-		587	10	1.88
	c71020_g1_i1	-		369	6	1.79
	c133671_g1_i1	-		393	6	1.68
	c178180_g1_i1	-		344	5	1.60
	c174348_g1_i1	-		826	12	1.60
	c69216_g1_i1	-		696	10	1.58
	c123895_g1_i1	yes		372	5	1.48
	c88033_g1_i1	-		150	2	1.47
	c182508_g1_i1	-		150	2	1.47
	c4027_g1_i1	-		154	2	1.43
	c182887_g1_i1	-		315	4	1.40
	c158504_g1_i1	-		402	5	1.37
	c133015_g1_i1	-		251	3	1.32
	c188173_g1_i1	-		221	2	1.00
	c140274_g1_i1	-		142	1	0.78
	c185179_g1_i1	-		319	2	0.69
Total			**54** / 96		**157,365**	**14,657.79**
**Peptidase S1 - LDLA domain**	c19670_g1_i1	-		1,506	460	33.63
	c41290_g2_i7	-		1,986	174	9.65
	c41290_g2_i5	-		4,402	420	10.51
	c41290_g2_i1	-		2,407	246	11.25
	c41290_g3_i1	-		2,939	670	25.10
	c41290_g3_i2	-		3,125	642	22.62
Total			**6** / 6		**2,612**	**112.76**
**Peptidase S10**	c36911_g1_i1	-		3,284	720	24.14
	c171170_g1_i1	-		332	39	12.93
	c122754_g1_i1	-		1,082	65	6.61
	c170269_g1_i1	-		693	36	5.72
	c121851_g1_i1	-		1,327	49	4.07
	c12279_g1_i1	-		409	14	3.77
Total			**5** / 6		**909**	**53.48**
**Phospholipase A2**	c40783_g1_i2	-		3,136	5,734	201.33
	c40783_g1_i1	-		3,133	5,149	180.96
	c36194_g1_i1	yes		958	754	86.66
	c35152_g1_i1	-		965	603	68.80
	c41269_g2_i1	yes		1,066	441	45.55
	c38299_g1_i2	yes		1,238	408	36.29
	c38299_g1_i1	yes		610	165	29.78

	c14022_g1_i1	yes		1,074	56	5.74
	c44309_g1_i1	yes		589	23	4.30
	c3302_g1_i1	-		926	26	3.09
	c3302_g1_i2	-		926	22	2.62
	c147416_g1_i1	-		573	12	2.31
Total			**9** / 12		**13,336**	**658.20**
**Serpin**	c38500_g1_i2	-		1,835	618	37.08
	c40151_g1_i1	yes		1,738	510	32.31
	c38500_g1_i1	-		1,375	250	20.02
	c44653_g1_i1	yes		578	76	14.48
	c40233_g1_i2	-		1,217	140	12.67
	c40233_g1_i1	-		1,357	152	12.33
	c41454_g5_i3	-		1,833	142	8.53
	c41454_g5_i1	yes		1,657	127	8.44
	c41454_g5_i4	-		1,761	125	7.82
Total			**9 / 9**		**2,140**	**153.67**
**Sphingomyelinase**	c37208_g1_i1	yes		1,978	479	26.66
	c42331_g1_i1	yes		2,029	196	10.64
	c95721_g1_i1	yes		2,631	206	8.62
	c122187_g1_i1	-		1,330	61	5.05
	c44135_g1_i1	-		649	29	4.92
	c142423_g1_i1	-		336	5	1.64
	c33214_g1_i1	-		213	0	0.00
Total			**5** / 7		**971**	**55.89**
**VEGF-like**	c29900_g1_i1	yes		2,348	575	26.96
	c32284_g1_i1	yes		877	184	23.10
	c8224_g1_i1	yes		1,142	85	8.20
	c102420_g1_i1	yes		1,144	66	6.35
	c123107_g1_i1	yes		346	16	5.09
	c29347_g1_i1	yes		764	34	4.90
	c88368_g1_i1	-		443	19	4.72
	c8330_g1_i1	yes		732	29	4.36
	c27914_g1_i1	-		545	21	4.24
	c30963_g3_i1	-		184	4	2.39
	c97173_g1_i1	-		175	1	0.63
Total			**6** / 11		**973**	**73.88**
**Xibalbin 5 (Putative CSab)**	c69533_g1_i1	yes	**1** / 1	802	35	4.81
**Xibalbin 6 (CHH/ITP)**	c170523_g1_i1	-	**1**	969	**88**	**10.00**
**Xibalbin 7 (sIGFBP-rp)**	c39061_g3_i1	yes		619	2,507	445.95
	c37504_g1_i1	yes		725	66	10.02
	c37504_g1_i2	yes		1,144	99	9.53
	c147484_g1_i1	yes		477	40	9.23
	c151081_g1_i1	yes		826	66	8.80
	c20493_g1_i1	yes		962	72	8.24
	c37504_g1_i3	yes		737	44	6.57
	c95379_g1_i1	yes		407	6	1.62
Total			**7** / 8		**2,894**	**498.34**
**Xibalbin 8 (Unknown 1)**	c122822_g1_i1	yes		741	5,312	789.33

	c38865_g1_i1	yes		600	3,013	552.93
	c27492_g1_i1	yes		417	7	1.85
Total			**2** / **3**		**8,325**	**1,342.26**
**Xibalbin 9 (Unknown 2)**	c11438_g1_i1	yes	**1**	727	**540**	**82**
**Xibalbin 13 (ICK)**	c37192_g1_i4	yes		984	124	13.88
	c37192_g1_i2	yes		927	116	13.78
	c37192_g1_i3	yes		1,011	110	11.98
Total			**3** / **3**		**350**	**39.63**

Supplementary Table 4:

Novel remipede venom peptide families and peptides named according to the rational nomenclature for venom peptide toxins proposed by King et al. (2008). Toxicon 52: 264-276, and modified by Undheim et al. (2014). Molecular Biology and Evolution 31: 2124-2148.* The expression level of these transcripts did not satisfy the cut-off level. See main text for explanation.

Peptide family	Peptide	Transcript
Xibalbin 1	U-xibalbin1-Xtu1a	c27367_g1_i1
Xibalbin 2	U-xibalbin2-Xtu1a	c29772_g1_i1
Xibalbin 3	U-xibalbin3-Xtu1a	c9387_g1_i1
	U-xibalbin3-Xtu1b	c29843_g1_i2
Xibalbin 4	U-xibalbin4-Xtu1a	c18306_g1_i1
Xibalbin 5	U-xibalbin5-Xtu1a	c28491_g1_i1
Xibalbin 6	U-xibalbin6-Xtu1a	c13591_g1_i1
Xibalbin 7	U-xibalbin7-Xtu1a	c23977_g2_i2*
	U-xibalbin7-Xtu1b	c23977_g2_i1*
Xibalbin 8	U-xibalbin8-Xtu1a	c23843_g2_i1
Xibalbin 9	U-xibalbin9-Xtu1a	c23666_g1_i1
Xibalbin 10	U-xibalbin10-Xtu1a	c25771_g1_i2
Xibalbin 11	U-xibalbin11-Xtu1a	c29407_g1_i2
	U-xibalbin11-Xtu1b	c29407_g1_i1

Supplementary Table 5: Venom gland tissue transcriptome (SOAP*denovo*-Tran contigs that match against secreted proteins [UniProtSL0243)]

Nr.	Contig name	Read Numbers	Blast result (evalue=10^{-5}, BlastP, Nr database)	Seq. length	min. evalue	mean similarity	SignalP	InterProScan
1799	98935_length_138_cvg_113.1_tip_1_0	71	serine protease 56	46	1.94E-08	71.70%	0.123	IPR001254 (PFAM); G3DSA:2.40.10.10 (GENE3D); PTHR24265 (PANTHER); IPR009003 (SUPERFAMILY)
1	101333_length_141_cvg_61.0_tip_1_5	60	chitinase 3	47	2.70E-10	67.50%	0.121	IPR013781 (G3DSA:3.20.20.GENE3D); IPR017853 (SUPERFAMILY)
2	102385_length_141_cvg_96.7_tip_0_4	59	serine protease like protein	47	6.55E-07	77.60%	0.174	IPR001254 (PFAM); G3DSA:2.40.10.10 (GENE3D); IPR018114 (PROSITE_PATTERNS); IPR009003 (SUPERFAMILY)
3	102473_length_141_cvg_63.0_tip_0_4	25	serine protease like protein	47	2.91E-07	77.60%	0.188	IPR001254 (PFAM); G3DSA:2.40.10.10 (GENE3D); IPR018114 (PROSITE_PATTERNS); IPR009003 (SUPERFAMILY)
4	102637_length_142_cvg_23.3_tip_1_5	0	zinc finger protein partial	47	6.80E-18	89.20%	0.107	IPR004457 (PFAM); PTHR10876 (PANTHER); PTHR10876:SF0 (PANTHER)
5	103713_length_142_cvg_7.4_tip_1_2	4	serine protease like protein	47	8.62E-07	74.90%	0.154	IPR001254 (PFAM); G3DSA:2.40.10.10 (GENE3D); IPR018114 (PROSITE_PATTERNS); IPR009003 (SUPERFAMILY)
6	104322_length_143_cvg_5.2_tip_1_0	0	bile salt-activated lipase	48	4.23E-09	73.40%	0.172	IPR029058 (G3DSA:3.40.50.GENE3D); IPR002018 (PFAM); PTHR11559 (PANTHER); PTHR11559:SF139 (PANTHER); IPR029058 (SUPERFAMILY)
7	104656_length_143_cvg_2.0_tip_1_1	0	insulin-degrading enzyme isoform x2	48	2.91E-11	80.20%	0.124	IPR011237 (G3DSA:3.30.830.GENE3D); IPR011249 (SUPERFAMILY)
8	105418_length_143_cvg_10.5_tip_1_3	0	stearoyl- desaturase partial	48	7.97E-14	83.70%	0.289	IPR015876 (PRINTS); PTHR11351 (PANTHER); CYTOPLASMIC_DOMAIN (PHOBIUS); NON_CYTOPLASMIC_DOMAIN (PHOBIUS); TRANSMEMBRANE (PHOBIUS); TMhelix (TMHMM)
9	106280_length_144_cvg_6.2_tip_1_2	0	venom serine carboxypeptidase	48	1.06E-13	81.00%	0.098	IPR001563 (PFAM); IPR029058 (G3DSA:3.40.50.GENE3D); PTHR11802:SF30 (PANTHER); IPR001563 (PANTHER); IPR029058 (SUPERFAMILY)
10	106336_length_144_cvg_6.0_tip_1_0	0	---NA---	48			0.113	IPR000716 (PFAM); IPR000716 (G3DSA:4.10.800.GENE3D); IPR000716 (PROSITE_PROFILES); IPR000716 (SUPERFAMILY)
11	107051_length_144_cvg_61.0_tip_1_1	47	mucin-22 isoform x4	48	4.61E-07	67.50%	0.1	IPR013781 (G3DSA:3.20.20.GENE3D); IPR001223 (PFAM); IPR017853 (SUPERFAMILY)
12	108305_length_145_cvg_2.0_tip_1_2	2	hypothetical protein EGK_12522, partial	48	6.34E-08	75.00%	0.759 Y	SIGNAL_PEPTIDE (PHOBIUS); NON_CYTOPLASMIC_DOMAIN (PHOBIUS); SIGNAL_PEPTIDE_C_REGION (PHOBIUS); SIGNAL_PEPTIDE_N_REGION (PHOBIUS); SIGNAL_PEPTIDE_H_REGION (PHOBIUS); SignalP-noTM (SIGNALP_EUK)
13	110726_length_146_cvg_3.0_tip_1_3	0	prophenoloxidase 1	49	2.55E-16	78.20%	0.109	IPR000896 (PFAM); IPR008922 (G3DSA:1.10.1280.GENE3D); IPR013788 (PANTHER); PTHR11511:SF24 (PANTHER); IPR008922 (SUPERFAMILY)
14	110738_length_146_cvg_2.0_tip_1_0	0	alanine dehydrogenase	49	1.75E-21	100.00%	0.16	IPR007698 (PFAM); IPR016040 (G3DSA:3.40.50.GENE3D); PTHR10160 (PANTHER); SSF51735 (SUPERFAMILY); TMhelix (TMHMM)
15	111644_length_147_cvg_60.0_tip_1_4	23	polycystic kidney disease protein 1-like 2-like	49	2.10E-06	70.50%	0.097	IPR002172 (SMART); IPR002172 (PFAM); IPR002172 (G3DSA:4.10.400.GENE3D); IPR023415 (PROSITE_PATTERNS); IPR002172 (PROSITE_PROFILES); IPR002172 (SUPERFAMILY)
16	111700_length_147_cvg_2.0_tip_1_1	2	mre11 meiotic recombination 11 homolog a (cerevisiae) isoform cra_b	49	7.16E-15	85.70%	0.217	PTHR22878 (PANTHER)
17	111700_length_147_cvg_2.0_tip_1_3	2	polymerase (dna directed) isoform cra_c	49	1.88E-19	85.80%	0.329	PR02045 (PRINTS); PTHR12138 (PANTHER)
18	111700_length_147_cvg_2.0_tip_1_5	2	tak1-binding protein isoform cra_b	49	8.43E-14	79.50%	0.175	PTHR16213 (PANTHER)
19	114208_length_148_cvg_10.4_tip_1_4	1	serine proteinase stubble-like	49	1.22E-07	66.70%	0.111	G3DSA:2.40.10.10 (GENE3D); IPR001254 (PFAM); IPR009003 (SUPERFAMILY)
20	114538_length_149_cvg_2.0_tip_1_0	0	histone h2b 1 2-like	50	1.81E-26	100.00%	0.111	IPR000558 (PRINTS); IPR000558 (SMART); IPR007125 (PFAM); IPR009072 (G3DSA:1.10.20.GENE3D); IPR000558 (PANTHER); IPR009072 (SUPERFAMILY)
21	115238_length_149_cvg_9.0_tip_1_0	2	chitinase 2	50	1.44E-10	68.50%	0.105	IPR001223 (PFAM); IPR013781 (G3DSA:3.20.20.GENE3D); PTHR11177:SF37 (PANTHER); PTHR11177 (PANTHER); IPR001579 (PROSITE_PATTERNS); IPR017853 (SUPERFAMILY)
22	116894_length_150_cvg_4.5_tip_1_5	1	atrial natriuretic peptide-converting enzyme-like	50	3.03E-06	67.50%	0.119	IPR002172 (SMART); IPR002172 (PFAM); IPR002172 (G3DSA:4.10.400.GENE3D); IPR002172 (PROSITE_PROFILES); IPR002172 (SUPERFAMILY)
24	118732_length_151_cvg_76.5_tip_0_2	76	secreted partial	50	2.34E-09	63.20%	0.102	IPR002557 (SMART); IPR002557 (PFAM); IPR002557 (G3DSA:2.170.140.GENE3D); PTHR23301 (PANTHER); IPR002557 (PROSITE_PROFILES); IPR002557 (SUPERFAMILY)
26	120362_length_152_cvg_14.7_tip_1_5	1	carboxypeptidase partial	50	1.02E-09	71.10%	0.097	G3DSA:3.40.630.10 (GENE3D); PTHR11705 (PANTHER); SSF53187 (SUPERFAMILY)
23	118192_length_151_cvg_60.8_tip_1_3	56	isoform h	51	4.58E-09	65.80%	0.106	IPR001223 (PFAM); IPR013781 (G3DSA:3.20.20.GENE3D); IPR017853 (SUPERFAMILY)
25	119748_length_152_cvg_35.3_tip_1_0	25	---NA---	51			0.098	IPR002172 (SMART); IPR002172 (PFAM); IPR002172 (G3DSA:4.10.400.GENE3D); IPR023415 (PROSITE_PATTERNS); IPR002172 (PROSITE_PROFILES); IPR002172 (SUPERFAMILY)

27	120494_length_152_cvg_9.4_tip_1_3	9	low-density lipoprotein receptor-related protein 6 isoform 2	51	2.72E-11	73.10%	0.102	IPR002172 (SMART); IPR002172 (PFAM); G3DSA:4.10.1220.10 (GENE3D); IPR002172 (G3DSA:4.10.400.GENE3D); PTHR10529 (PANTHER); IPR002172 (PROSITE_PROFILES); IPR002172 (SUPERFAMILY)
28	121382_length_153_cvg_75.8_tip_1_1	2	carboxypeptidase b-like	51	7.06E-12	77.80%	0.126	IPR000834 (PFAM); G3DSA:3.40.630.10 (GENE3D); PTHR11705 (PANTHER); SSF53187 (SUPERFAMILY)
29	121892_length_153_cvg_7.1_tip_1_3	2	low-density lipoprotein receptor-related protein 5	51	5.30E-08	63.22%	0.105	IPR002172 (SMART); IPR002172 (PFAM); IPR002172 (G3DSA:4.10.400.GENE3D); IPR023415 (PROSITE_PATTERNS); IPR002172 (PROSITE_PROFILES); IPR002172 (SUPERFAMILY)
30	122044_length_153_cvg_3.0_tip_1_5	0	gelsolin-like allergen der f 16	51	1.67E-14	69.10%	0.108	IPR029006 (G3DSA:3.40.20.GENE3D); IPR007123 (PFAM); IPR007122 (PANTHER); IPR030016 (PTHR11977:PANTHER); SSF55753 (SUPERFAMILY)
31	122840_length_154_cvg_6.7_tip_1_4	0	partial	51	1.38E-13	80.80%	0.106	Coil (COILS)
33	124508_length_155_cvg_2.0_tip_1_5	0	serine protease 45 isoform x5	51	6.33E-07	72.40%	0.281	G3DSA:2.40.10.10 (GENE3D); IPR001254 (PFAM); IPR009003 (SUPERFAMILY)
32	123884_length_155_cvg_5.0_tip_1_3	0	alpha-carbonic anhydrase	52	5.88E-15	77.10%	0.265	IPR001148 (PFAM); IPR001148 (G3DSA:3.10.200.GENE3D); PTHR18952:SF92 (PANTHER); IPR023561 (PANTHER); IPR001148 (PROSITE_PROFILES); IPR001148 (SUPERFAMILY)
34	124954_length_155_cvg_5.0_tip_1_3	3	venom allergen 3-like	52	1.73E-11	67.80%	0.129	IPR014044 (G3DSA:3.40.33.GENE3D); IPR014044 (SUPERFAMILY)
35	125338_length_156_cvg_34.9_tip_1_4	13	serine protease easter	52	3.26E-07	74.20%	0.183	IPR001254 (PFAM); G3DSA:2.40.10.10 (GENE3D); PTHR24275 (PANTHER); IPR009003 (SUPERFAMILY)
36	125508_length_156_cvg_14.0_tip_1_5	13	chitinase 3	52	4.42E-09	63.90%	0.102	IPR001223 (PFAM); IPR013781 (G3DSA:3.20.20.GENE3D); IPR017853 (SUPERFAMILY)
37	126180_length_156_cvg_41.2_tip_1_0	3	basement membrane-specific heparan sulfate proteoglycan core protein	52	3.37E-08	63.30%	0.1	IPR002172 (PRINTS); IPR002172 (SMART); IPR002172 (G3DSA:4.10.400.GENE3D); IPR002172 (PFAM); IPR023415 (PROSITE_PATTERNS); IPR002172 (PROSITE_PROFILES); IPR002172 (SUPERFAMILY)
38	127252_length_157_cvg_39.5_tip_1_2	124	proclotting enzyme-like	52	2.79E-10	72.00%	0.147	IPR001254 (PFAM); G3DSA:2.40.10.10 (GENE3D); PTHR24275 (PANTHER); IPR001254 (PROSITE_PROFILES); IPR009003 (SUPERFAMILY)
39	127822_length_157_cvg_45.2_tip_1_0	23	venom allergen 3	53	8.87E-09	78.33%	0.101	IPR014044 (G3DSA:3.40.33.GENE3D); IPR014044 (SUPERFAMILY)
40	128010_length_157_cvg_3.6_tip_1_3	1	---NA---	53			0.199	no IPS match
41	128474_length_158_cvg_20.8_tip_1_3	7	transmembrane protease serine 11f	53	1.56E-08	64.10%	0.235	G3DSA:2.40.10.10 (GENE3D); IPR001254 (PFAM); PTHR24250 (PANTHER); IPR009003 (SUPERFAMILY)
42	130338_length_159_cvg_16.0_tip_1_4	0	serine proteinase stubble-like	53	2.25E-09	72.70%	0.154	G3DSA:2.40.10.10 (GENE3D); IPR001254 (PFAM); PTHR24256 (PANTHER); IPR018114 (PROSITE_PATTERNS); IPR009003 (SUPERFAMILY)
43	130422_length_159_cvg_2.0_tip_1_4	0	serine endoprotease partial	53	3.99E-24	100.00%	0.107	IPR001940 (PRINTS); G3DSA:2.40.10.10 (GENE3D); IPR001254 (PFAM); PTHR22939 (PANTHER); IPR009003 (SUPERFAMILY)
44	130464_length_159_cvg_14.8_tip_1_0	1	carboxypeptidase b-like	53	1.15E-10	71.30%	0.142	G3DSA:3.40.630.10 (GENE3D); SSF53187 (SUPERFAMILY)
45	130948_length_159_cvg_63.0_tip_0_3	3	probable serine carboxypeptidase cpvl	53	1.59E-11	72.00%	0.12	IPR001563 (PFAM); IPR029058 (G3DSA:3.40.50.GENE3D); IPR001563 (PANTHER); IPR029058 (SUPERFAMILY)
46	133688_length_161_cvg_3.8_tip_1_4	0	alpha beta hydrolase domain-containing protein 17b-like	54	9.27E-30	99.00%	0.101	IPR029059 (PFAM); IPR029058 (G3DSA:3.40.50.GENE3D); PTHR12277:SF55 (PANTHER); PTHR12277 (PANTHER); IPR029058 (SUPERFAMILY)
47	133740_length_161_cvg_63.0_tip_0_0	2	low quality protein: glutathione peroxidase 1-like	54	1.53E-22	84.20%	0.106	IPR012336 (G3DSA:3.40.30.GENE3D); PTHR11592:SF24 (PANTHER); IPR000889 (PANTHER); IPR000889 (PROSITE_PROFILES); IPR012336 (SUPERFAMILY)
48	134146_length_162_cvg_3.5_tip_1_4	1	---NA---	54			0.105	IPR002172 (SMART); IPR002172 (PFAM); IPR002172 (G3DSA:4.10.400.GENE3D); IPR023415 (PROSITE_PATTERNS); IPR002172 (PROSITE_PROFILES); IPR002172 (SUPERFAMILY)
49	134236_length_162_cvg_55.7_tip_1_5	2	very low-density lipoprotein receptor	54	8.68E-07	63.67%	0.191	G3DSA:4.10.1220.10 (GENE3D); IPR002172 (G3DSA:4.10.400.GENE3D); IPR002172 (PFAM); IPR002172 (SUPERFAMILY)
50	134534_length_162_cvg_30.0_tip_1_5	3	serine protease 42-like	54	8.10E-07	71.40%	0.157	G3DSA:2.40.10.10 (GENE3D); IPR001254 (PFAM); IPR018114 (PROSITE_PATTERNS); IPR009003 (SUPERFAMILY)
51	134664_length_162_cvg_4.0_tip_1_1	4	st14a protein	54	3.02E-06	69.00%	0.1	IPR001254 (PFAM); G3DSA:2.40.10.10 (GENE3D); IPR009003 (SUPERFAMILY)
52	135072_length_162_cvg_8.5_tip_1_5	8	basement membrane-specific heparan sulfate proteoglycan core protein	54	2.27E-09	66.60%	0.103	IPR002172 (SMART); G3DSA:4.10.1220.10 (GENE3D); IPR002172 (PFAM); IPR002172 (G3DSA:4.10.400.GENE3D); IPR002172 (PROSITE_PROFILES); IPR002172 (SUPERFAMILY)
53	135316_length_162_cvg_2.0_tip_1_4	0	heat shock cognate 70	54	4.74E-20	85.80%	0.099	IPR029048 (G3DSA:1.20.1270.GENE3D); PTHR19375 (PANTHER); PTHR19375:SF142 (PANTHER); IPR029048 (SUPERFAMILY)
55	136372_length_163_cvg_2.0_tip_1_2	2	membrane alanyl aminopeptidase	54	2.73E-29	100.00%	0.168	IPR014782 (PRINTS); IPR014782 (PFAM); G3DSA:1.10.390.10 (GENE3D); PTHR11533:SF163 (PANTHER); IPR001930 (PANTHER); SSF55486 (SUPERFAMILY)
54	136174_length_163_cvg_2.2_tip_1_3	0	hypothetical protein Y032_0473g2095	55	1.01E-07	73.80%	0.122	no IPS match
56	136396_length_163_cvg_3.1_tip_1_0	0	heat shock cognate 71 kda protein	55	4.22E-27	93.40%	0.1	IPR013126 (PRINTS); G3DSA:3.30.30.30 (GENE3D); IPR013126 (PFAM); PTHR19375 (PANTHER); PTHR19375:SF155 (PANTHER); SSF53067 (SUPERFAMILY)
57	136832_length_163_cvg_2.0_tip_1_3	0	protein sda1 homolog	55	6.40E-20	91.00%	0.165	IPR027312 (PTHR12730:PANTHER); PTHR12730 (PANTHER)
58	136844_length_163_cvg_2.0_tip_1_0	0	elastin isoform x11	55	4.46E-08	66.14%	0.099	no IPS match
59	138652_length_165_cvg_2.1_tip_1_0	0	glyceraldehyde-3-phosphate dehydrogenase	55	2.64E-13	89.70%	0.123	IPR020831 (PANTHER); PTHR10836:SF31 (PANTHER)

60	139536_length_165_cvg_132.3_tip_0_1	473	low-density lipoprotein receptor-related protein 2-like	55	1.51E-06	71.50%	0.099	IPR002172 (SMART); IPR002172 (PFAM); IPR002172 (G3DSA:4.10.400.GENE3D); IPR023415 (PROSITE_PATTERNS); IPR002172 (PROSITE_PROFILES); IPR002172 (SUPERFAMILY)
63	141944_length_167_cvg_2.0_tip_1_2	0	partial	55	3.22E-19	85.00%	0.096	IPR013320 (G3DSA:2.60.120.GENE3D); IPR001580 (PFAM); PTHR11073:SF5 (PANTHER); IPR001580 (PANTHER); IPR013320 (SUPERFAMILY)
61	140504_length_166_cvg_78.5_tip_1_0	5	urokinase-type plasminogen activator	56	1.43E-08	73.30%	0.31	G3DSA:2.40.10.10 (GENE3D); IPR001254 (PFAM); PTHR24265 (PANTHER); IPR001254 (PROSITE_PROFILES); IPR009003 (SUPERFAMILY)
62	141658_length_167_cvg_63.0_tip_1_3	31	chymotrypsin-like elastase family member 1	56	1.73E-08	65.90%	0.118	G3DSA:2.40.10.10 (GENE3D); IPR001254 (PFAM); PTHR24268 (PANTHER); IPR018114 (PROSITE_PATTERNS); IPR009003 (SUPERFAMILY)
64	142817_length_168_cvg_109.6_tip_0_0	179	flocculation protein flo11 isoform x2	56	2.58E-07	76.80%	0.132	IPR001254 (PFAM); G3DSA:2.40.10.10 (GENE3D); IPR018114 (PROSITE_PATTERNS); IPR009003 (SUPERFAMILY)
65	142839_length_168_cvg_2.0_tip_1_0	0	c4b-binding protein beta chain	56	3.17E-19	78.00%	0.121	G3DSA:2.10.70.10 (GENE3D); IPR000436 (PFAM); PTHR19325:SF340 (PANTHER); PTHR19325 (PANTHER); IPR000436 (PROSITE_PROFILES); IPR000436 (SUPERFAMILY)
66	142999_length_168_cvg_4.6_tip_1_1	1	venom allergen 3- partial	56	8.58E-13	75.20%	0.104	IPR001283 (PRINTS); IPR002413 (PRINTS); IPR014044 (G3DSA:3.40.33.GENE3D); IPR014044 (PFAM); IPR018244 (PROSITE_PATTERNS); IPR014044 (SUPERFAMILY)
67	143039_length_168_cvg_7.0_tip_1_4	0	chitinase-related protein 1	56	1.89E-15	76.30%	0.114	IPR013781 (G3DSA:3.20.20.GENE3D); IPR001223 (PFAM); PTHR11177 (PANTHER); IPR017853 (SUPERFAMILY)
68	143301_length_168_cvg_103.7_tip_1_4	4	low-density lipoprotein receptor-related protein 2	56	2.61E-08	64.30%	0.1	IPR002172 (SMART); IPR002172 (PFAM); IPR002172 (G3DSA:4.10.400.GENE3D); G3DSA:4.10.1220.10 (GENE3D); PTHR10529 (PANTHER); IPR002172 (PROSITE_PROFILES); IPR002172 (SUPERFAMILY)
69	143319_length_168_cvg_10.3_tip_1_1	0	hemagglutinin amebocyte aggregation factor-like	56	2.36E-12	74.30%	0.108	PF14704 (PFAM); IPR026645 (PANTHER)
70	143575_length_168_cvg_32.3_tip_1_4	1	transmembrane protease serine 11f	56	3.72E-10	65.40%	0.105	IPR001254 (PFAM); G3DSA:2.40.10.10 (GENE3D); PTHR24275:SF50 (PANTHER); PTHR24275 (PANTHER); IPR001254 (PROSITE_PROFILES); IPR009003 (SUPERFAMILY)
71	143643_length_168_cvg_63.0_tip_0_3	170	flocculation protein flo11 isoform x1	56	3.63E-07	76.80%	0.133	G3DSA:2.40.10.10 (GENE3D); IPR001254 (PFAM); IPR018114 (PROSITE_PATTERNS); IPR009003 (SUPERFAMILY)
76	145147_length_169_cvg_2.4_tip_1_1	0	hypothetical protein Y032_0580g260	56	2.10E-09	66.30%	0.102	no IPS match
77	145155_length_169_cvg_17.3_tip_1_1	37	---NA---	56			0.101	G3DSA:2.40.10.10 (GENE3D); IPR009003 (SUPERFAMILY)
72	144515_length_169_cvg_31.0_tip_1_3	130	---NA---	57			0.101	G3DSA:4.10.1220.10 (GENE3D); IPR002172 (G3DSA:4.10.400.GENE3D); IPR002172 (PFAM); IPR002172 (PROSITE_PROFILES); IPR002172 (SUPERFAMILY)
73	144637_length_169_cvg_25.2_tip_1_3	2	agap000573-pb-like protein	57	7.35E-08	66.60%	0.11	G3DSA:2.40.10.10 (GENE3D); IPR001254 (PFAM); IPR009003 (SUPERFAMILY)
74	145045_length_169_cvg_3.2_tip_0_0	0	hyaluronidase ph-20-like	57	5.38E-08	62.90%	0.101	IPR018155 (PFAM); IPR013785 (G3DSA:3.20.20.GENE3D); IPR017853 (SUPERFAMILY)
75	145135_length_169_cvg_2.3_tip_0_3	0	hyaluronidase- partial	57	6.11E-08	63.20%	0.1	IPR013785 (G3DSA:3.20.20.GENE3D); IPR018155 (PFAM); IPR018155 (PANTHER); IPR017853 (SUPERFAMILY)
78	145391_length_170_cvg_61.9_tip_1_3	81	acidic mammalian chitinase-like	57	8.22E-10	65.30%	0.117	IPR001223 (PFAM); IPR013781 (G3DSA:3.20.20.GENE3D); IPR017853 (SUPERFAMILY)
79	146565_length_171_cvg_2.0_tip_1_1	0	cysteine-rich secretory protein 2	57	3.11E-26	85.60%	0.12	IPR014044 (G3DSA:3.40.33.GENE3D); IPR014044 (SUPERFAMILY)
80	147255_length_171_cvg_2.6_tip_1_1	0	neurogenic locus notch	57	2.42E-24	96.00%	0.105	PR00010 (PRINTS); IPR001881 (SMART); G3DSA:2.10.25.10 (GENE3D); IPR000742 (PFAM); G3DSA:2.10.25.10 (GENE3D); PTHR24033 (PANTHER); PTHR24033:SF36 (PANTHER); IPR018097 (PROSITE_PATTERNS); IPR000152 (PROSITE_PATTERNS); IPR013032 (PROSITE_PATTERNS); IPR000152 (PROSITE_PATTERNS); IPR000742 (PROSITE_PROFILES); IPR000742 (PROSITE_PROFILES); SSF57196 (SUPERFAMILY); SSF57196 (SUPERFAMILY)
84	148789_length_172_cvg_2.0_tip_1_2	0	isoform a	57	1.27E-06	65.50%	0.102	IPR002223 (PRINTS); IPR002223 (PFAM); IPR002223 (G3DSA:4.10.410.GENE3D); IPR020901 (PROSITE_PATTERNS); IPR002223 (PROSITE_PROFILES); IPR002223 (SUPERFAMILY)
81	147787_length_172_cvg_56.0_tip_1_0	357	sortilin-related receptor	58	2.67E-07	62.60%	0.1	IPR002172 (SMART); IPR002172 (PFAM); IPR002172 (G3DSA:4.10.400.GENE3D); PTHR10529 (PANTHER); PTHR10529:SF222 (PANTHER); IPR023415 (PROSITE_PATTERNS); IPR002172 (PROSITE_PROFILES); IPR002172 (SUPERFAMILY)
82	147841_length_172_cvg_8.0_tip_1_0	0	hemagglutinin amebocyte aggregation factor-like	58	3.77E-10	64.60%	0.102	PF14704 (PFAM); IPR026645 (PANTHER)
83	148351_length_172_cvg_4.5_tip_1_3	0	histidine-rich glyco	58	5.00E-08	63.90%	0.098	no IPS match
85	149541_length_173_cvg_93.8_tip_1_0	248	isoform a	58	3.71E-06	57.67%	0.11	IPR002172 (PRINTS); IPR002172 (SMART); IPR002172 (G3DSA:4.10.400.GENE3D); IPR002172 (PFAM); IPR002172 (PROSITE_PROFILES); IPR002172 (SUPERFAMILY)
86	149543_length_173_cvg_63.0_tip_1_0	132	isoform a	58	3.71E-06	57.67%	0.11	IPR002172 (PRINTS); IPR002172 (SMART); IPR002172 (G3DSA:4.10.400.GENE3D); IPR002172 (PFAM); IPR002172 (PROSITE_PROFILES); IPR002172 (SUPERFAMILY)

87	149725_length_173_cvg_115.5_tip_0_0	297	low-density lipoprotein receptor-related protein 1-like	58	1.28E-09	65.50%	0.099	IPR002172 (G3DSA:4.10.400.GENE3D); G3DSA:4.10.1220.10 (GENE3D); IPR002172 (PFAM); PTHR10529 (PANTHER); IPR023415 (PROSITE_PATTERNS); IPR002172 (PROSITE_PROFILES); IPR002172 (SUPERFAMILY); IPR002172 (SUPERFAMILY)
88	151099_length_174_cvg_42.2_tip_1_1	1	suppressor of tumorigenicity 14 protein homolog	58	8.20E-09	64.00%	0.101	IPR002172 (PRINTS); IPR002172 (PFAM); G3DSA:4.10.1220.10 (GENE3D); IPR002172 (G3DSA:4.10.400.GENE3D); PTHR10529 (PANTHER); IPR023415 (PROSITE_PATTERNS); IPR002172 (PROSITE_PROFILES); IPR002172 (SUPERFAMILY); IPR002172 (SUPERFAMILY)
89	151199_length_174_cvg_59.3_tip_1_1	390	st14a protein	58	1.48E-06	63.57%	0.101	G3DSA:2.40.10.10 (GENE3D); IPR001254 (PFAM); IPR009003 (SUPERFAMILY)
90	151427_length_174_cvg_2.6_tip_1_5	5	endo- -d-glucanase	58	5.15E-22	100.00%	0.12	IPR002037 (PFAM)
91	152010_length_175_cvg_21.0_tip_1_4	2	transmembrane protease serine 11a	58	8.67E-08	65.83%	0.105	G3DSA:2.40.10.10 (GENE3D); IPR001254 (PFAM); IPR009003 (SUPERFAMILY)
93	152778_length_176_cvg_88.1_tip_0_5	30	probable serine carboxypeptidase cpvl isoform x5	58	2.01E-15	72.50%	0.104	IPR029058 (G3DSA:3.40.50.GENE3D); IPR001563 (PFAM); IPR001563 (PANTHER); IPR029058 (SUPERFAMILY)
95	153190_length_176_cvg_2.0_tip_1_5	0	enolase partial	58	2.58E-23	79.70%	0.118	IPR029065 (G3DSA:3.20.20.GENE3D); IPR020810 (PFAM); IPR000941 (PANTHER); IPR029065 (SUPERFAMILY)
92	152440_length_175_cvg_22.6_tip_1_0	3	clipb6 protein	59	8.03E-12	66.70%	0.127	IPR001254 (PFAM); G3DSA:2.40.10.10 (GENE3D); PTHR24268:SF72 (PANTHER); PTHR24268 (PANTHER); IPR018114 (PROSITE_PATTERNS); IPR009003 (SUPERFAMILY)
94	152988_length_176_cvg_2.0_tip_1_3	1	ensangg00000017398 protein	59	2.37E-29	95.80%	0.099	IPR013126 (PRINTS); IPR029047 (G3DSA:2.60.34.GENE3D); IPR013126 (PFAM); PTHR19375 (PANTHER); PTHR19375:SF142 (PANTHER); SSF53067 (SUPERFAMILY)
96	154172_length_177_cvg_7.1_tip_1_1	0	granzyme a-like	59	6.05E-06	55.00%	0.1	G3DSA:2.40.10.10 (GENE3D); IPR001254 (PFAM); IPR009003 (SUPERFAMILY)
97	154674_length_177_cvg_12.6_tip_1_5	0	carboxypeptidase b-like	59	1.41E-15	83.00%	0.1	IPR000834 (PRINTS); G3DSA:3.40.630.10 (GENE3D); IPR000834 (PFAM); PTHR11705 (PANTHER); PTHR11705:SF61 (PANTHER); SSF53187 (SUPERFAMILY)
98	155286_length_178_cvg_2.5_tip_1_2	24	chitinase 2	59	1.01E-09	61.90%	0.103	IPR001223 (PFAM); IPR013781 (G3DSA:3.20.20.GENE3D); PTHR11177:SF45 (PANTHER); PTHR11177 (PANTHER); IPR017853 (SUPERFAMILY)
99	155822_length_178_cvg_5.8_tip_1_3	1	---NA---	60			0.186	IPR029058 (G3DSA:3.40.50.GENE3D); IPR001563 (PFAM); IPR029058 (SUPERFAMILY)
100	155898_length_178_cvg_60.3_tip_1_0	886	chitinase partial	60	3.69E-11	65.10%	0.103	IPR029070 (G3DSA:3.10.50.GENE3D); IPR029070 (SUPERFAMILY)
101	157098_length_179_cvg_3.8_tip_1_3	0	serine (or cysteine) proteinase clade b member	60	3.00E-09	65.60%	0.119	IPR023796 (PFAM); G3DSA:2.30.39.10 (GENE3D); IPR000215 (PANTHER); PTHR11461:SF52 (PANTHER); IPR023795 (PROSITE_PATTERNS); IPR023796 (SUPERFAMILY)
102	157254_length_179_cvg_84.3_tip_0_3	308	chymotrypsin-like elastase family member 1	60	8.06E-09	61.90%	0.113	IPR001254 (PFAM); G3DSA:2.40.10.10 (GENE3D); PTHR24268 (PANTHER); IPR018114 (PROSITE_PATTERNS); IPR009003 (SUPERFAMILY)
103	157808_length_179_cvg_35.0_tip_1_3	31	isoform a	60	5.71E-08	75.40%	0.227	G3DSA:2.40.10.10 (GENE3D); IPR001254 (PFAM); PTHR24275 (PANTHER); IPR009003 (SUPERFAMILY)
104	158144_length_180_cvg_9.0_tip_1_4	0	dermatopontin 3	60	6.99E-10	69.20%	0.103	PF14704 (PFAM)
105	158236_length_180_cvg_62.0_tip_1_4	2290	acidic mammalian chitinase-like isoform x1	60	1.65E-10	64.00%	0.133	IPR013781 (G3DSA:3.20.20.GENE3D); IPR029070 (G3DSA:3.10.50.GENE3D); IPR001223 (PFAM); PTHR11177:SF112 (PANTHER); PTHR11177 (PANTHER); IPR029070 (SUPERFAMILY); IPR017853 (SUPERFAMILY)
106	158352_length_180_cvg_16.3_tip_1_3	0	carboxypeptidase b-like	60	1.40E-16	75.70%	0.097	G3DSA:3.40.630.10 (GENE3D); IPR000834 (PFAM); PTHR11705 (PANTHER); SSF53187 (SUPERFAMILY)
107	158546_length_180_cvg_3.3_tip_1_2	0	low-density lipoprotein receptor-related protein 2-like	60	3.71E-07	58.10%	0.114	IPR002172 (SMART); IPR002172 (PFAM); IPR002172 (G3DSA:4.10.400.GENE3D); IPR002172 (G3DSA:4.10.400.GENE3D); IPR002172 (PROSITE_PROFILES); IPR002172 (SUPERFAMILY)
109	159385_length_181_cvg_2.0_tip_1_1	0	nitrate reductase catalytic partial	60	8.13E-35	100.00%	0.118	IPR006656 (PFAM); G3DSA:3.40.228.10 (GENE3D); PTHR11615 (PANTHER); PTHR11615:SF123 (PANTHER); SSF53706 (SUPERFAMILY)
110	159581_length_181_cvg_62.1_tip_1_4	70	isoform f	60	2.92E-08	74.70%	0.135	IPR001223 (PFAM); IPR013781 (G3DSA:3.20.20.GENE3D); IPR017853 (SUPERFAMILY)
111	160585_length_182_cvg_68.7_tip_1_5	85	chitinase	60	2.17E-06	71.00%	0.115	IPR013781 (G3DSA:3.20.20.GENE3D); IPR001223 (PFAM); IPR017853 (SUPERFAMILY)
113	161237_length_182_cvg_62.0_tip_1_2	48	chitinase partial	60	5.61E-09	71.00%	0.102	IPR013781 (G3DSA:3.20.20.GENE3D); IPR001223 (PFAM); IPR017853 (SUPERFAMILY)
108	159259_length_181_cvg_28.7_tip_1_3	9	low-density lipoprotein receptor-related protein 5	61	1.64E-07	65.30%	0.112	IPR002172 (SMART); IPR002172 (PFAM); IPR002172 (G3DSA:4.10.400.GENE3D); IPR002172 (PROSITE_PROFILES); IPR002172 (SUPERFAMILY)
112	160629_length_182_cvg_2.0_tip_1_1	0	protein kinase dc2	61	1.28E-33	97.40%	0.096	G3DSA:1.10.510.10 (GENE3D); IPR000719 (PFAM); PTHR24353 (PANTHER); IPR008271 (PROSITE_PATTERNS); IPR000719 (PROSITE_PROFILES); IPR011009 (SUPERFAMILY)
114	161279_length_182_cvg_2.0_tip_1_3	0	polypeptide n-acetylgalactosaminyltransferase 2	61	1.39E-22	84.80%	0.186	G3DSA:2.80.10.50 (GENE3D); IPR000772 (PFAM); IPR000772 (PROSITE_PROFILES); IPR000772 (SUPERFAMILY)

115	161283_length_182_cvg_60.6_tip_1_3	2113	chitinase partial	61	1.47E-12	66.00%	0.145	IPR029070 (G3DSA:3.10.50.GENE3D); IPR013781 (G3DSA:3.20.20.GENE3D); IPR001223 (PFAM); PTHR11177 (PANTHER); IPR029070 (SUPERFAMILY); IPR017853 (SUPERFAMILY)
116	161522_length_183_cvg_61.5_tip_1_0	11	af117899_1ldlr-fut fusion protein	61	3.81E-06	72.67%	0.217	IPR002172 (SMART); IPR002172 (G3DSA:4.10.400.GENE3D); IPR002172 (PFAM); IPR023415 (PROSITE_PATTERNS); IPR002172 (PROSITE_PROFILES); IPR002172 (SUPERFAMILY)
117	161528_length_183_cvg_28.8_tip_1_3	1	carboxypeptidase partial	61	2.42E-12	63.80%	0.304	IPR000834 (PFAM); G3DSA:3.40.630.10 (GENE3D); PTHR11705 (PANTHER); SSF53187 (SUPERFAMILY)
118	161538_length_183_cvg_56.0_tip_1_2	10	---NA---	61			0.182	IPR002172 (SMART); IPR002172 (PFAM); IPR002172 (G3DSA:4.10.400.GENE3D); IPR002172 (PROSITE_PROFILES); IPR002172 (SUPERFAMILY)
119	161564_length_183_cvg_2.0_tip_1_0	0	molecular chaperone partial	61	3.98E-35	99.80%	0.112	IPR013126 (PRINTS); IPR013126 (PFAM); G3DSA:3.30.420.40 (GENE3D); PTHR19375 (PANTHER); PTHR19375:SF173 (PANTHER); IPR018181 (PROSITE_PATTERNS); SSF53067 (SUPERFAMILY)
120	161578_length_183_cvg_53.2_tip_1_5	9	serine protease 57	61	3.31E-06	58.30%	0.103	IPR001254 (PFAM); G3DSA:2.40.10.10 (GENE3D); IPR018114 (PROSITE_PATTERNS); IPR009003 (SUPERFAMILY)
121	161584_length_183_cvg_19.2_tip_1_2	2	chymotrypsin-like elastase family member 2a	61	3.78E-06	63.50%	0.136	IPR001254 (PFAM); G3DSA:2.40.10.10 (GENE3D); IPR009003 (SUPERFAMILY)
122	161654_length_183_cvg_61.0_tip_1_0	59	chitinase partial	61	5.12E-12	61.30%	0.101	IPR013781 (G3DSA:3.20.20.GENE3D); IPR001223 (PFAM); IPR017853 (SUPERFAMILY)
123	161700_length_183_cvg_62.0_tip_1_1	49	chitinase 2	61	2.46E-11	62.80%	0.104	IPR001223 (PFAM); IPR013781 (G3DSA:3.20.20.GENE3D); IPR001579 (PROSITE_PATTERNS); IPR017853 (SUPERFAMILY)
124	161704_length_183_cvg_31.8_tip_1_4	8	secreted salivary gland	61	1.52E-10	68.70%	0.112	G3DSA:2.40.10.10 (GENE3D); IPR001254 (PFAM); PTHR24256 (PANTHER); IPR009003 (SUPERFAMILY)
125	161716_length_183_cvg_45.2_tip_1_1	21	low-density lipoprotein receptor-related protein 2-like	61	5.32E-08	61.10%	0.098	IPR002172 (PRINTS); IPR002172 (SMART); IPR002172 (G3DSA:4.10.400.GENE3D); IPR002172 (PFAM); IPR002172 (G3DSA:4.10.400.GENE3D); IPR023415 (PROSITE_PATTERNS); IPR002172 (PROSITE_PROFILES); IPR002172 (SUPERFAMILY)
126	161752_length_183_cvg_61.8_tip_1_1	59	chitotriosidase- partial	61	4.14E-09	61.60%	0.1	IPR013781 (G3DSA:3.20.20.GENE3D); IPR001223 (PFAM); IPR017853 (SUPERFAMILY)
127	161762_length_183_cvg_35.3_tip_1_1	260	st14a protein	61	5.27E-08	64.70%	0.112	G3DSA:2.40.10.10 (GENE3D); IPR001254 (PFAM); IPR009003 (SUPERFAMILY)
128	161782_length_183_cvg_61.0_tip_1_2	10	---NA---	61			0.21	G3DSA:2.40.10.10 (GENE3D); IPR001254 (PFAM); IPR009003 (SUPERFAMILY)
129	161822_length_183_cvg_61.0_tip_1_4	75	chitinase 3	61	2.04E-09	62.40%	0.119	IPR013781 (G3DSA:3.20.20.GENE3D); IPR001223 (PFAM); IPR017853 (SUPERFAMILY)
130	161826_length_183_cvg_28.4_tip_1_1	3	neurotrypsin- partial	61	1.19E-10	68.90%	0.134	IPR001254 (PFAM); G3DSA:2.40.10.10 (GENE3D); PTHR24256 (PANTHER); IPR001254 (PROSITE_PROFILES); IPR009003 (SUPERFAMILY)
131	161830_length_183_cvg_50.6_tip_1_5	216	low-density lipoprotein receptor-related protein 8	61	1.97E-11	65.00%	0.101	IPR002172 (SMART); IPR002172 (G3DSA:4.10.400.GENE3D); IPR002172 (PFAM); IPR002172 (G3DSA:4.10.400.GENE3D); PTHR10529 (PANTHER); IPR002172 (PROSITE_PROFILES); IPR002172 (SUPERFAMILY); IPR002172 (SUPERFAMILY)
132	161832_length_183_cvg_11.7_tip_1_5	2	low quality protein: serine protease 55	61	8.65E-08	67.20%	0.108	IPR001254 (PFAM); G3DSA:2.40.10.10 (GENE3D); IPR018114 (PROSITE_PATTERNS); IPR009003 (SUPERFAMILY)
133	161836_length_183_cvg_31.9_tip_1_4	159	low-density lipoprotein receptor-related protein 6 isoform x2	61	5.06E-10	68.30%	0.103	IPR002172 (SMART); IPR002172 (PFAM); IPR002172 (G3DSA:4.10.400.GENE3D); IPR002172 (G3DSA:4.10.400.GENE3D); PTHR10529 (PANTHER); IPR023415 (PROSITE_PATTERNS); IPR002172 (PROSITE_PROFILES); IPR002172 (SUPERFAMILY)
134	161868_length_183_cvg_38.4_tip_1_4	6	chitinase 1	61	1.02E-08	65.70%	0.174	IPR013781 (G3DSA:3.20.20.GENE3D); IPR017853 (SUPERFAMILY)
135	161874_length_183_cvg_45.0_tip_1_3	2	chymotrypsin-like elastase family member 2a	61	2.26E-08	59.70%	0.136	IPR001254 (PFAM); G3DSA:2.40.10.10 (GENE3D); PTHR24258:SF97 (PANTHER); PTHR24258 (PANTHER); IPR018114 (PROSITE_PATTERNS); IPR009003 (SUPERFAMILY)
136	161882_length_183_cvg_19.6_tip_1_3	2	zinc carboxypeptidase-like	61	1.05E-15	67.40%	0.117	G3DSA:3.40.630.10 (GENE3D); IPR000834 (PFAM); PTHR11705 (PANTHER); SSF53187 (SUPERFAMILY)
137	161896_length_183_cvg_28.4_tip_1_2	185	transmembrane protease serine 11b	61	4.84E-07	61.80%	0.106	G3DSA:2.40.10.10 (GENE3D); IPR001254 (PFAM); PTHR24268:SF72 (PANTHER); PTHR24268 (PANTHER); IPR009003 (SUPERFAMILY)
138	161916_length_183_cvg_31.2_tip_1_2	5	suppressor of tumorigenicity 14 protein	61	6.65E-12	66.00%	0.193	IPR001254 (PFAM); G3DSA:2.40.10.10 (GENE3D); PTHR24268 (PANTHER); IPR009003 (SUPERFAMILY)
139	161948_length_183_cvg_44.6_tip_1_5	10	---NA---	61			0.204	IPR001254 (PFAM); G3DSA:2.40.10.10 (GENE3D); IPR009003 (SUPERFAMILY)
140	161960_length_183_cvg_10.2_tip_1_2	2	---NA---	61			0.098	G3DSA:2.40.10.10 (GENE3D); IPR001254 (PFAM); IPR009003 (SUPERFAMILY)
141	161966_length_183_cvg_48.8_tip_1_1	2	transmembrane protease serine 11e	61	4.25E-10	68.30%	0.134	IPR001254 (PFAM); G3DSA:2.40.10.10 (GENE3D); PTHR24275 (PANTHER); IPR018114 (PROSITE_PATTERNS); IPR001254 (PROSITE_PROFILES); IPR009003 (SUPERFAMILY)
142	162006_length_183_cvg_34.0_tip_1_2	4	complement c1s subcomponent	61	1.27E-06	66.50%	0.106	IPR001254 (PFAM); G3DSA:2.40.10.10 (GENE3D); PTHR24265 (PANTHER); IPR009003 (SUPERFAMILY)
143	162010_length_183_cvg_39.7_tip_1_4	1	chymotrypsin-like elastase family member 2b	61	1.50E-09	66.70%	0.103	IPR001254 (PFAM); G3DSA:2.40.10.10 (GENE3D); PTHR24258:SF97 (PANTHER); PTHR24258 (PANTHER); IPR009003 (SUPERFAMILY)
144	162012_length_183_cvg_61.0_tip_1_1	54	chitinase 7 precursor	61	3.09E-08	61.60%	0.177	IPR013781 (G3DSA:3.20.20.GENE3D); IPR017853 (SUPERFAMILY)

145	162022_length_183_cvg_61.0_tip_1_5	48	chitinase partial	61	4.45E-08	68.00%	0.1	IPR013781 (G3DSA:3.20.20.GENE3D); IPR001223 (PFAM); IPR017853 (SUPERFAMILY)
146	162030_length_183_cvg_43.2_tip_1_1	204	isoform a	61	3.64E-07	64.20%	0.109	G3DSA:2.40.10.10 (GENE3D); IPR001254 (PFAM); IPR009003 (SUPERFAMILY)
147	162038_length_183_cvg_60.2_tip_1_1	43	chitinase partial	61	2.71E-12	68.60%	0.131	IPR001223 (PFAM); IPR013781 (G3DSA:3.20.20.GENE3D); IPR017853 (SUPERFAMILY)
148	162040_length_183_cvg_60.2_tip_1_1	40	chitinase partial	61	1.71E-11	61.50%	0.105	IPR013781 (G3DSA:3.20.20.GENE3D); IPR001223 (PFAM); PTHR11177:SF112 (PANTHER); PTHR11177 (PANTHER); IPR017853 (SUPERFAMILY)
149	162062_length_183_cvg_2.0_tip_1_2	0	zinc finger protein 2 homolog	61	5.30E-17	72.60%	0.162	IPR015880 (SMART); PF13465 (PFAM); IPR013087 (G3DSA:3.30.160.GENE3D); IPR013087 (G3DSA:3.30.160.GENE3D); IPR007087 (PFAM); IPR013087 (G3DSA:3.30.160.GENE3D); PTHR24409 (PANTHER); IPR007087 (PROSITE_PATTERNS); IPR007087 (PROSITE_PROFILES); SSF57667 (SUPERFAMILY)
150	162120_length_183_cvg_60.8_tip_1_4	53	chitinase partial	61	1.11E-10	70.00%	0.101	IPR013781 (G3DSA:3.20.20.GENE3D); IPR001223 (PFAM); IPR017853 (SUPERFAMILY)
151	162144_length_183_cvg_29.7_tip_1_3	11	serine proteinase stubble-like	61	6.27E-10	61.00%	0.1	IPR001254 (PFAM); G3DSA:2.40.10.10 (GENE3D); PTHR24256 (PANTHER); IPR009003 (SUPERFAMILY)
152	162178_length_183_cvg_60.0_tip_1_5	1107	chitinase isoform a	61	6.04E-16	63.30%	0.101	IPR001223 (PFAM); IPR013781 (G3DSA:3.20.20.GENE3D); PTHR11177 (PANTHER); PTHR11177:SF45 (PANTHER); IPR001579 (PROSITE_PATTERNS); IPR017853 (SUPERFAMILY)
153	162186_length_183_cvg_28.7_tip_1_2	0	venom serine carboxypeptidase-like	61	2.68E-17	68.00%	0.128	IPR029058 (G3DSA:3.40.50.GENE3D); IPR001563 (PFAM); IPR001563 (PANTHER); IPR029058 (SUPERFAMILY)
154	162190_length_183_cvg_53.6_tip_1_3	13	low-density lipoprotein receptor-related protein 6 isoform x2	61	2.55E-11	69.00%	0.102	IPR002172 (SMART); IPR002172 (PFAM); IPR002172 (G3DSA:4.10.400.GENE3D); PTHR10529 (PANTHER); IPR023415 (PROSITE_PATTERNS); IPR002172 (PROSITE_PROFILES); IPR002172 (SUPERFAMILY)
155	162220_length_183_cvg_61.0_tip_1_1	82	chitinase 2	61	1.69E-12	64.10%	0.101	IPR013781 (G3DSA:3.20.20.GENE3D); IPR001223 (PFAM); PTHR11177 (PANTHER); PTHR11177:SF45 (PANTHER); IPR001579 (PROSITE_PATTERNS); IPR017853 (SUPERFAMILY)
156	162266_length_183_cvg_31.0_tip_1_2	480	low-density lipoprotein receptor-related protein 2	61	1.85E-09	64.90%	0.099	IPR002172 (PRINTS); IPR002172 (SMART); G3DSA:4.10.1220.10 (GENE3D); IPR002172 (PFAM); IPR002172 (G3DSA:4.10.400.GENE3D); PTHR10529 (PANTHER); IPR002172 (PROSITE_PROFILES); IPR002172 (PROSITE_PROFILES); IPR002172 (SUPERFAMILY); IPR002172 (SUPERFAMILY)
157	162286_length_183_cvg_7.8_tip_1_1	0	---NA---	61			0.123	IPR014044 (G3DSA:3.40.33.GENE3D); IPR014044 (PFAM); IPR014044 (SUPERFAMILY)
158	162410_length_183_cvg_26.2_tip_1_3	26	sortilin-related receptor containing ldlr class a repeats preproprotein	61	1.43E-06	62.00%	0.1	IPR002172 (SMART); IPR002172 (G3DSA:4.10.400.GENE3D); IPR002172 (PFAM); IPR023415 (PROSITE_PATTERNS); IPR002172 (PROSITE_PROFILES); IPR002172 (SUPERFAMILY)
159	162414_length_183_cvg_33.6_tip_1_5	1	serine protease like protein	61	1.45E-12	64.50%	0.164	IPR001254 (PFAM); G3DSA:2.40.10.10 (GENE3D); PTHR24268 (PANTHER); PTHR24268:SF72 (PANTHER); IPR018114 (PROSITE_PATTERNS); IPR009003 (SUPERFAMILY)
160	162434_length_183_cvg_60.2_tip_1_3	54	chitinase partial	61	2.63E-09	59.30%	0.1	IPR013781 (G3DSA:3.20.20.GENE3D); IPR001223 (PFAM); IPR017853 (SUPERFAMILY)
161	162440_length_183_cvg_56.0_tip_1_4	11	granzyme a-like	61	5.72E-06	58.00%	0.112	IPR001254 (PFAM); G3DSA:2.40.10.10 (GENE3D); IPR009003 (SUPERFAMILY)
162	162452_length_183_cvg_61.0_tip_1_3	9	low-density lipoprotein receptor-related protein 2-like	61	5.69E-13	68.00%	0.11	IPR002172 (PRINTS); IPR002172 (SMART); IPR002172 (PFAM); IPR002172 (G3DSA:4.10.400.GENE3D); IPR002172 (G3DSA:4.10.400.GENE3D); PTHR10529 (PANTHER); PTHR10529:SF222 (PANTHER); IPR002172 (PROSITE_PROFILES); IPR002172 (SUPERFAMILY); IPR002172 (SUPERFAMILY)
163	162472_length_183_cvg_36.9_tip_1_0	0	chitinase partial	61	1.27E-16	71.70%	0.156	IPR001223 (PFAM); IPR029070 (G3DSA:3.10.50.GENE3D); IPR013781 (G3DSA:3.20.20.GENE3D); PTHR11177 (PANTHER); IPR017853 (SUPERFAMILY); IPR029070 (SUPERFAMILY)
164	162496_length_183_cvg_59.6_tip_1_1	53	isoform k	61	7.61E-13	65.40%	0.108	IPR013781 (G3DSA:3.20.20.GENE3D); IPR001223 (PFAM); PTHR11177:SF45 (PANTHER); PTHR11177 (PANTHER); IPR017853 (SUPERFAMILY)
165	162544_length_183_cvg_18.6_tip_1_1	3	carboxypeptidase b	61	1.36E-10	62.90%	0.139	G3DSA:3.40.630.10 (GENE3D); IPR000834 (PFAM); PTHR11705 (PANTHER); SSF53187 (SUPERFAMILY)
166	162546_length_183_cvg_60.0_tip_1_0	405	clip-domain serine protease	61	3.46E-06	77.33%	0.313	G3DSA:2.40.10.10 (GENE3D); IPR001254 (PFAM); PTHR24275 (PANTHER); IPR009003 (SUPERFAMILY)
167	162564_length_183_cvg_41.0_tip_1_0	266	transcription activator brg1	61	2.25E-10	66.10%	0.1	IPR002172 (SMART); IPR002172 (G3DSA:4.10.400.GENE3D); IPR002172 (PFAM); IPR002172 (G3DSA:4.10.400.GENE3D); IPR023415 (PROSITE_PATTERNS); IPR002172 (PROSITE_PROFILES); IPR002172 (SUPERFAMILY)
168	162574_length_183_cvg_41.7_tip_1_5	156	sortilin-related receptor- partial	61	3.67E-08	59.30%	0.101	IPR002172 (SMART); IPR002172 (PFAM); G3DSA:4.10.1220.10 (GENE3D); IPR002172 (G3DSA:4.10.400.GENE3D); IPR002172 (PROSITE_PROFILES); IPR002172 (SUPERFAMILY)

169	162596_length_183_cvg_26.1_tip_1_0	4	chymotrypsin-like elastase family member 1	61	1.52E-07	62.10%	0.145	G3DSA:2.40.10.10 (GENE3D); IPR001254 (PFAM); PTHR24258 (PANTHER); PTHR24258:SF97 (PANTHER); IPR018114 (PROSITE_PATTERNS); IPR009003 (SUPERFAMILY)
170	162598_length_183_cvg_31.2_tip_1_4	9	granzyme a-like	61	4.74E-08	60.90%	0.106	G3DSA:2.40.10.10 (GENE3D); IPR001254 (PFAM); IPR009003 (SUPERFAMILY)
171	162640_length_183_cvg_35.5_tip_1_3	3	plasma kallikrein	61	5.08E-08	61.60%	0.123	IPR001254 (PFAM); G3DSA:2.40.10.10 (GENE3D); PTHR24250 (PANTHER); PTHR24250:SF6 (PANTHER); IPR018114 (PROSITE_PATTERNS); IPR009003 (SUPERFAMILY)
172	162682_length_183_cvg_60.2_tip_1_1	36	chitinase partial	61	1.59E-12	65.20%	0.107	IPR001223 (PFAM); IPR013781 (G3DSA:3.20.20.GENE3D); PTHR11177:SF112 (PANTHER); PTHR11177 (PANTHER); IPR001579 (PROSITE_PATTERNS); IPR017853 (SUPERFAMILY)
173	162776_length_183_cvg_60.0_tip_1_5	40	chitinase partial	61	1.93E-10	71.00%	0.101	IPR013781 (G3DSA:3.20.20.GENE3D); IPR001223 (PFAM); IPR017853 (SUPERFAMILY)
174	162800_length_183_cvg_61.0_tip_1_5	11	suppressor of tumorigenicity 14 protein	61	5.42E-09	67.80%	0.13	G3DSA:2.40.10.10 (GENE3D); IPR001254 (PFAM); PTHR24256 (PANTHER); IPR009003 (SUPERFAMILY)
175	162816_length_183_cvg_60.0_tip_1_2	66	isoform h	61	2.01E-13	67.80%	0.12	IPR013781 (G3DSA:3.20.20.GENE3D); IPR001223 (PFAM); PTHR11177 (PANTHER); IPR017853 (SUPERFAMILY)
176	162826_length_183_cvg_42.6_tip_1_2	14	low-density lipoprotein receptor-related protein 2-partial	61	2.23E-10	63.60%	0.097	IPR002172 (PRINTS); G3DSA:4.10.1220.10 (GENE3D); IPR002172 (PFAM); IPR002172 (G3DSA:4.10.400.GENE3D); PTHR10529 (PANTHER); IPR002172 (PROSITE_PROFILES); IPR002172 (SUPERFAMILY); IPR002172 (SUPERFAMILY)
177	162828_length_183_cvg_59.3_tip_1_5	32	chitinase partial	61	2.48E-09	68.10%	0.118	IPR013781 (G3DSA:3.20.20.GENE3D); IPR017853 (SUPERFAMILY)
178	163819_length_184_cvg_84.4_tip_1_5	6	carboxypeptidase a2-like	61	4.06E-15	76.80%	0.106	IPR000834 (PFAM); G3DSA:3.40.630.10 (GENE3D); PTHR11705 (PANTHER); SSF53187 (SUPERFAMILY)
179	164537_length_185_cvg_62.0_tip_1_0	239	probable chitinase 3	62	4.12E-13	68.60%	0.144	IPR013781 (G3DSA:3.20.20.GENE3D); IPR001223 (PFAM); IPR017853 (SUPERFAMILY)
180	164579_length_185_cvg_14.9_tip_1_4	67	chitinase isoform a	62	1.38E-12	63.30%	0.106	IPR013781 (G3DSA:3.20.20.GENE3D); IPR001223 (PFAM); PTHR11177 (PANTHER); PTHR11177:SF45 (PANTHER); IPR017853 (SUPERFAMILY)
181	164733_length_185_cvg_59.0_tip_1_3	60	isoform h	62	1.09E-13	66.30%	0.116	IPR013781 (G3DSA:3.20.20.GENE3D); IPR001223 (PFAM); PTHR11177 (PANTHER); IPR017853 (SUPERFAMILY)
182	165047_length_185_cvg_58.7_tip_1_4	69	brain chitinase and chia	62	7.44E-09	63.50%	0.104	IPR001223 (PFAM); IPR013781 (G3DSA:3.20.20.GENE3D); PTHR11177:SF45 (PANTHER); PTHR11177 (PANTHER); IPR001579 (PROSITE_PATTERNS); IPR017853 (SUPERFAMILY)
183	165415_length_186_cvg_2.0_tip_1_4	2	outer membrane autotransporter barrel domain protein	62	1.21E-33	100.00%	0.111	IPR005546 (PFAM); IPR005546 (G3DSA:2.40.128.GENE3D); IPR005546 (SUPERFAMILY)
184	165911_length_186_cvg_36.8_tip_1_0	37	neurotrypsin- partial	62	7.20E-07	64.57%	0.138	G3DSA:2.40.10.10 (GENE3D); IPR001254 (PFAM); IPR018114 (PROSITE_PATTERNS); IPR009003 (SUPERFAMILY)
185	167307_length_187_cvg_56.7_tip_1_1	87	peptidase s1 and s6 chymotrypsin hap	62	1.51E-11	73.40%	0.163	G3DSA:2.40.10.10 (GENE3D); IPR001254 (PFAM); PTHR24275 (PANTHER); IPR018114 (PROSITE_PATTERNS); IPR001254 (PROSITE_PROFILES); IPR009003 (SUPERFAMILY)
186	168596_length_188_cvg_19.3_tip_1_4	2	low quality protein: probable chitinase 3	63	1.09E-13	70.50%	0.103	IPR013781 (G3DSA:3.20.20.GENE3D); IPR017853 (SUPERFAMILY)
187	168688_length_189_cvg_13.0_tip_1_4	0	leukocyte elastase inhibitor	63	2.20E-09	72.10%	0.133	G3DSA:2.30.39.10 (GENE3D); IPR023796 (PFAM); IPR000215 (PANTHER); IPR023795 (PROSITE_PATTERNS); IPR023796 (SUPERFAMILY)
188	170656_length_190_cvg_2.0_tip_1_4	0	hypothetical protein DAPPUDRAFT_317027	63	5.33E-08	62.00%	0.135	IPR015420 (PFAM); G3DSA:2.40.10.10 (GENE3D); IPR009003 (SUPERFAMILY)
189	171606_length_191_cvg_16.8_tip_1_5	1	---NA---	63			0.103	IPR002172 (SMART); IPR002172 (G3DSA:4.10.400.GENE3D); IPR002172 (PFAM); IPR002172 (PROSITE_PROFILES); IPR002172 (SUPERFAMILY)
190	172110_length_192_cvg_2.0_tip_1_1	0	nad h:quinone oxidoreductase	64	1.27E-35	99.20%	0.156	IPR029039 (G3DSA:3.40.50.GENE3D); PTHR30546 (PANTHER); IPR029039 (SUPERFAMILY)
192	174052_length_193_cvg_5.8_tip_1_4	0	suppressor of tumorigenicity 14 protein	64	3.11E-11	72.70%	0.134	G3DSA:2.40.10.10 (GENE3D); IPR001254 (PFAM); PTHR24256 (PANTHER); IPR009003 (SUPERFAMILY)
193	174474_length_194_cvg_2.0_tip_1_2	0	chitinase partial	64	2.02E-06	47.00%	0.166	IPR029070 (G3DSA:3.10.50.GENE3D)
191	173640_length_193_cvg_2.0_tip_1_0	0	lysyl-trna synthetase	65	1.04E-28	90.80%	0.102	IPR004364 (PFAM); G3DSA:3.30.930.10 (GENE3D); PTHR22594:SF25 (PANTHER); IPR018150 (PANTHER); IPR006195 (PROSITE_PROFILES); SSF55681 (SUPERFAMILY)
194	176070_length_195_cvg_63.0_tip_1_4	0	allergen 5	65	5.04E-13	74.80%	0.128	IPR002413 (PRINTS); IPR014044 (G3DSA:3.40.33.GENE3D); IPR014044 (SUPERFAMILY)
195	176198_length_195_cvg_56.2_tip_1_2	9	chymotrypsin-like elastase family member partial	65	2.17E-10	66.40%	0.259	IPR001254 (PFAM); G3DSA:2.40.10.10 (GENE3D); PTHR24275 (PANTHER); IPR018114 (PROSITE_PATTERNS); IPR009003 (SUPERFAMILY)
196	176208_length_195_cvg_7.3_tip_1_4	4	serine protease	65	2.59E-08	69.80%	0.119	IPR001254 (PFAM); G3DSA:2.40.10.10 (GENE3D); PTHR24268:SF81 (PANTHER); PTHR24268 (PANTHER); IPR018114 (PROSITE_PATTERNS); IPR009003 (SUPERFAMILY)
197	177798_length_197_cvg_3.1_tip_1_5	0	serine protease	65	2.50E-09	64.40%	0.104	IPR001254 (PFAM); G3DSA:2.40.10.10 (GENE3D); PTHR24268 (PANTHER); IPR018114 (PROSITE_PATTERNS); IPR009003 (SUPERFAMILY)

198	178412_length_197_cvg_84.0_tip_1_0	1163	trypsin-like protease	66	9.40E-08	61.70%	0.135	G3DSA:2.40.10.10 (GENE3D); IPR001254 (PFAM); PTHR24275 (PANTHER); IPR018114 (PROSITE_PATTERNS); IPR001254 (PROSITE_PROFILES); IPR009003 (SUPERFAMILY)
199	178612_length_198_cvg_63.8_tip_0_3	2	gamma-glutamyl hydrolase	66	4.32E-15	63.80%	0.098	IPR029062 (G3DSA:3.40.50.GENE3D); PTHR11315:SF0 (PANTHER); IPR015527 (PANTHER)
200	179098_length_198_cvg_2.0_tip_1_4	0	udp- c:polypeptide n-	66	5.24E-07	82.90%	0.112	no IPS match
201	181240_length_200_cvg_71.2_tip_0_3	698	proclotting enzyme	67	9.68E-14	69.40%	0.103	IPR001254 (PFAM); G3DSA:2.40.10.10 (GENE3D); PTHR24256 (PANTHER); IPR001254 (PROSITE_PROFILES); IPR009003 (SUPERFAMILY)
202	181262_length_200_cvg_2.4_tip_1_1	0	hypothetical protein Y032_0473g2095	67	1.99E-10	66.20%	0.109	no IPS match
203	181788_length_201_cvg_2.0_tip_1_1	0	alpha-amylase	67	1.55E-39	99.60%	0.158	IPR013781 (G3DSA:3.20.20.GENE3D); IPR017853 (SUPERFAMILY)
204	181916_length_201_cvg_60.9_tip_1_1	41	chitinase-3-like protein 1	67	9.48E-14	65.40%	0.12	IPR001223 (PFAM); IPR013781 (G3DSA:3.20.20.GENE3D); PTHR11177 (PANTHER); IPR017853 (SUPERFAMILY)
205	182700_length_202_cvg_2.0_tip_1_1	0	zinc finger protein 501-like	67	5.59E-09	69.60%	0.1	IPR015880 (SMART); PF13465 (PFAM); IPR013087 (G3DSA:3.30.160.GENE3D); PTHR24409 (PANTHER); PTHR24409:SF14 (PANTHER); IPR007087 (PROSITE_PATTERNS); IPR007087 (PROSITE_PATTERNS); IPR007087 (PROSITE_PROFILES); IPR007087 (PROSITE_PROFILES); SSF57667 (SUPERFAMILY)
206	183980_length_203_cvg_120.8_tip_1_3	15	venom allergen partial	68	3.67E-11	61.60%	0.117	IPR001283 (PRINTS); IPR014044 (PFAM); IPR014044 (G3DSA:3.40.33.GENE3D); IPR001283 (PANTHER); IPR018244 (PROSITE_PATTERNS); IPR014044 (SUPERFAMILY)
207	184866_length_204_cvg_85.6_tip_1_1	22	urokinase-type plasminogen activator	68	2.57E-12	69.00%	0.14	G3DSA:2.40.10.10 (GENE3D); IPR001254 (PFAM); PTHR24265:SF76 (PANTHER); PTHR24265 (PANTHER); IPR001254 (PROSITE_PROFILES); IPR009003 (SUPERFAMILY)
208	184962_length_204_cvg_2.0_tip_1_4	0	endothelin-converting enzyme 1-like	68	4.75E-20	83.60%	0.118	IPR024079 (G3DSA:3.40.390.GENE3D); IPR018497 (PFAM); PTHR11733:SF110 (PANTHER); IPR000718 (PANTHER); SSF55486 (SUPERFAMILY)
210	186520_length_205_cvg_61.0_tip_1_1	47	chitotriosidase- partial	68	1.31E-14	62.40%	0.104	IPR029070 (G3DSA:3.10.50.GENE3D); IPR001223 (PFAM); IPR013781 (G3DSA:3.20.20.GENE3D); PTHR11177 (PANTHER); PTHR11177:SF45 (PANTHER); IPR017853 (SUPERFAMILY)
209	186108_length_205_cvg_2.0_tip_1_0	0	serine threonine-protein kinase mark2-like isoform x10	69	6.33E-39	99.80%	0.109	G3DSA:3.30.200.20 (GENE3D); IPR000719 (PFAM); PTHR24346 (PANTHER); IPR008271 (PROSITE_PATTERNS); IPR000719 (PROSITE_PROFILES); IPR011009 (SUPERFAMILY)
211	190393_length_209_cvg_9.0_tip_1_2	0	lysosomal acid phosphatase	69	4.69E-14	71.00%	0.517 Y	IPR000560 (PFAM); IPR029033 (G3DSA:3.40.50.GENE3D); PTHR11567:SF26 (PANTHER); PTHR11567 (PANTHER); IPR000560 (PROSITE_PATTERNS); SignalP-noTM (SIGNALP_EUK); IPR029033 (SUPERFAMILY)
212	190901_length_209_cvg_2.0_tip_1_5	0	von willebrand factor type egf and pentraxin domain-containing protein 1 isoform x1	69	1.06E-19	77.50%	0.128	IPR011641 (PFAM); G3DSA:2.10.50.10 (GENE3D); PTHR24049:SF6 (PANTHER); PTHR24049 (PANTHER); IPR009030 (SUPERFAMILY)
346	288445_length_332_cvg_4.5_tip_1_0	1	chitinase 7 precursor	69	3.13E-09	61.90%	0.101	IPR013781 (G3DSA:3.20.20.GENE3D)
213	191679_length_210_cvg_104.4_tip_0_4	2	neutral protease	70	2.32E-14	68.10%	0.112	IPR013856 (PFAM); IPR013856 (G3DSA:3.10.170.GENE3D); SSF55486 (SUPERFAMILY)
214	192413_length_211_cvg_2.0_tip_1_2	0	glyceraldehyde-3-phosphate dehydrogenase c	70	1.94E-25	96.40%	0.105	IPR020828 (PFAM); IPR016040 (G3DSA:3.40.50.GENE3D); PTHR10836:SF31 (PANTHER); IPR020831 (PANTHER); SSF51735 (SUPERFAMILY)
215	193737_length_212_cvg_2.0_tip_1_1	0	n-acetylated-alpha-linked acidic dipeptidase	71	4.95E-31	85.70%	0.107	G3DSA:3.40.630.10 (GENE3D); IPR007484 (PFAM); PTHR10404 (PANTHER); SSF53187 (SUPERFAMILY)
216	194686_length_213_cvg_2.0_tip_1_5	0	protein partial	71	3.11E-16	80.90%	0.16	IPR019154 (PFAM); PTHR21357 (PANTHER)
217	195332_length_213_cvg_2.0_tip_1_4	0	tryptophan-specific transport protein	71	8.96E-40	100.00%	0.14	IPR018227 (PFAM); PTHR32195 (PANTHER); PTHR32195:SF11 (PANTHER); TRANSMEMBRANE (PHOBIUS); CYTOPLASMIC_DOMAIN (PHOBIUS); NON_CYTOPLASMIC_DOMAIN (PHOBIUS); TMhelix (TMHMM)
218	195482_length_213_cvg_2.0_tip_1_4	0	aael008198- partial	71	6.22E-17	66.30%	0.101	G3DSA:2.170.300.10 (GENE3D); PTHR24035 (PANTHER); IPR013032 (PROSITE_PATTERNS); IPR000742 (PROSITE_PROFILES)
219	195916_length_214_cvg_4.0_tip_1_3	0	dimethylaniline monooxygenase	72	9.16E-22	75.90%	0.111	IPR020946 (PFAM); G3DSA:3.50.50.60 (GENE3D); PTHR23023 (PANTHER); PTHR23023:SF4 (PANTHER); SSF51905 (SUPERFAMILY)
220	196852_length_215_cvg_2.0_tip_1_0	0	phosphoglucose partial	72	3.26E-45	98.80%	0.168	IPR001672 (PFAM); G3DSA:3.40.50.10490 (GENE3D); IPR001672 (PANTHER); PTHR11469:SF7 (PANTHER); IPR001672 (PROSITE_PROFILES); SSF53697 (SUPERFAMILY)
221	197384_length_215_cvg_2.0_tip_1_1	0	serine threonine-protein phosphatase 4 catalytic subunit	72	4.46E-43	98.00%	0.1	IPR006186 (PRINTS); IPR029052 (G3DSA:3.60.21.GENE3D); IPR004843 (PFAM); PTHR11668 (PANTHER); IPR029052 (SUPERFAMILY)
222	197766_length_216_cvg_87.7_tip_1_1	11	carboxypeptidase b-like	72	2.81E-21	76.10%	0.11	G3DSA:3.40.630.10 (GENE3D); IPR000834 (PFAM); PTHR11705 (PANTHER); IPR000834 (PROSITE_PATTERNS); SSF53187 (SUPERFAMILY)
223	198108_length_216_cvg_63.0_tip_1_1	11	carboxypeptidase b-like	72	2.00E-20	77.90%	0.128	IPR000834 (PFAM); G3DSA:3.40.630.10 (GENE3D); PTHR11705 (PANTHER); IPR000834 (PROSITE_PATTERNS); SSF53187 (SUPERFAMILY)

224	200410_length_218_cvg_2.0_tip_1_1	0	isoform g	73	1.93E-23	72.70%	0.14	G3DSA:2.10.70.10 (GENE3D); IPR000436 (PFAM); G3DSA:2.10.70.10 (GENE3D); PTHR19325:SF340 (PANTHER); PTHR19325 (PANTHER); IPR000436 (PROSITE_PROFILES); IPR000436 (PROSITE_PROFILES); IPR000436 (SUPERFAMILY); IPR000436 (SUPERFAMILY)
225	200875_length_219_cvg_3.8_tip_1_2	0	upf0669 protein c6orf120 homolog	73	3.07E-12	67.70%	0.809 Y	PTHR31703 (PANTHER); NON_CYTOPLASMIC_DOMAIN (PHOBIUS); SIGNAL_PEPTIDE_H_REGION (PHOBIUS); SIGNAL_PEPTIDE_N_REGION (PHOBIUS); SIGNAL_PEPTIDE_C_REGION (PHOBIUS); SIGNAL_PEPTIDE (PHOBIUS); SignalP-noTM (SIGNALP_EUK); SignalP-noTM (SIGNALP_GRAM_NEGATIVE)
226	203299_length_221_cvg_3.0_tip_1_3	2	xanthine dehydrogenase	74	3.27E-21	79.00%	0.103	IPR016169 (G3DSA:3.30.465.GENE3D); IPR002346 (PFAM); PTHR11908:SF32 (PANTHER); PTHR11908 (PANTHER); IPR016166 (SUPERFAMILY)
227	204205_length_222_cvg_2.0_tip_1_3	0	basement membrane-specific heparan sulfate proteoglycan core protein	74	1.25E-26	81.80%	0.109	IPR002049 (PFAM); G3DSA:2.10.25.10 (GENE3D); PTHR10574 (PANTHER); PTHR10574:SF252 (PANTHER); IPR002049 (PROSITE_PATTERNS); SSF57196 (SUPERFAMILY)
228	206453_length_224_cvg_2.0_tip_1_3	0	peptidyl-prolyl cis-trans rhodopsin-specific isozyme-like	75	1.03E-29	78.50%	0.134	IPR002130 (PRINTS); IPR002130 (PFAM); IPR029000 (G3DSA:2.40.100.GENE3D); IPR024936 (PANTHER); PTHR11071:SF11 (PANTHER); IPR002130 (PROSITE_PROFILES); IPR029000 (SUPERFAMILY)
229	206984_length_225_cvg_25.7_tip_1_2	0	xanthine dehydrogenase	75	3.94E-07	79.00%	0.226	IPR012675 (G3DSA:3.10.20.GENE3D)
230	209190_length_227_cvg_2.0_tip_1_5	0	proprotein convertase subtilisin kexin type 7	75	4.11E-31	85.10%	0.121	IPR000209 (PFAM); IPR000209 (G3DSA:3.40.50.GENE3D); PTHR10795:SF9 (PANTHER); IPR015500 (PANTHER); IPR023828 (PROSITE_PATTERNS); IPR000209 (SUPERFAMILY)
231	209490_length_227_cvg_2.5_tip_1_2	0	atp-binding component of a transport system	75	1.29E-40	98.10%	0.102	IPR012332 (G3DSA:2.160.20.GENE3D)
232	210110_length_228_cvg_4.4_tip_1_2	0	hypothetical protein Y032_0089g2326	76	5.56E-07	62.60%	0.106	no IPS match
233	210620_length_228_cvg_2.0_tip_1_4	0	zinc finger protein 676-like	76	6.78E-24	71.40%	0.119	IPR015880 (SMART); IPR013087 (G3DSA:3.30.160.GENE3D); IPR013087 (G3DSA:3.30.160.GENE3D); IPR013087 (G3DSA:3.30.160.GENE3D); PF13465 (PFAM); PTHR24409 (PANTHER); IPR007087 (PROSITE_PATTERNS); IPR007087 (PROSITE_PATTERNS); IPR007087 (PROSITE_PROFILES); IPR007087 (PROSITE_PROFILES); SSF57667 (SUPERFAMILY); SSF57667 (SUPERFAMILY)
235	211490_length_229_cvg_65.4_tip_1_1	115	chitinase 3	76	1.20E-17	64.90%	0.111	IPR001223 (PFAM); IPR013781 (G3DSA:3.20.20.GENE3D); PTHR11177 (PANTHER); IPR017853 (SUPERFAMILY)
236	211668_length_229_cvg_54.1_tip_1_5	6	26kda protease	76	2.73E-16	65.90%	0.124	G3DSA:2.40.10.10 (GENE3D); IPR001254 (PFAM); G3DSA:2.40.10.10 (GENE3D); PTHR24268 (PANTHER); IPR001254 (PROSITE_PROFILES); IPR009003 (SUPERFAMILY)
237	211962_length_229_cvg_47.2_tip_1_1	802	proclotting enzyme	76	2.09E-08	57.20%	0.106	IPR001254 (PFAM); G3DSA:2.40.10.10 (GENE3D); PTHR24268 (PANTHER); IPR009003 (SUPERFAMILY)
238	212778_length_230_cvg_2.0_tip_1_2	0	low quality protein: protein flightless-1 homolog	76	2.33E-27	85.90%	0.169	PR00019 (PRINTS); IPR003591 (SMART); G3DSA:3.80.10.10 (GENE3D); IPR001611 (PFAM); IPR007122 (PANTHER); IPR001611 (PROSITE_PROFILES); IPR001611 (PROSITE_PROFILES); IPR001611 (PROSITE_PROFILES); SSF52058 (SUPERFAMILY)
234	211482_length_229_cvg_59.4_tip_1_3	78	chitinase partial	77	2.37E-18	65.00%	0.122	IPR013781 (G3DSA:3.20.20.GENE3D); IPR001223 (PFAM); PTHR11177:SF112 (PANTHER); PTHR11177 (PANTHER); IPR017853 (SUPERFAMILY)
239	213100_length_231_cvg_60.0_tip_1_4	81	chitinase 7 precursor	77	8.32E-08	55.80%	0.135	IPR013781 (G3DSA:3.20.20.GENE3D); IPR017853 (SUPERFAMILY)
240	213684_length_231_cvg_2.0_tip_1_5	0	zinc finger protein 665-like isoform x6	77	5.69E-21	70.60%	0.108	IPR015880 (SMART); IPR013087 (G3DSA:3.30.160.GENE3D); PF13465 (PFAM); PTHR24409 (PANTHER); IPR007087 (PROSITE_PATTERNS); IPR007087 (PROSITE_PATTERNS); IPR007087 (PROSITE_PROFILES); IPR007087 (PROSITE_PROFILES); SSF57667 (SUPERFAMILY); SSF57667 (SUPERFAMILY)
241	214176_length_232_cvg_90.1_tip_0_3	426	low-density lipoprotein receptor-related protein 2-like	78	3.51E-15	63.70%	0.1	IPR002172 (PRINTS); IPR002172 (SMART); IPR002172 (PFAM); IPR002172 (G3DSA:4.10.400.GENE3D); G3DSA:4.10.1220.10 (GENE3D); PTHR10529 (PANTHER); IPR023415 (PROSITE_PATTERNS); IPR023415 (PROSITE_PATTERNS); IPR002172 (PROSITE_PROFILES); IPR002172 (PROSITE_PROFILES); IPR002172 (SUPERFAMILY); IPR002172 (SUPERFAMILY)
242	215360_length_233_cvg_63.0_tip_1_3	17	serine protease 27-like	78	1.20E-10	63.70%	0.104	IPR001314 (PRINTS); IPR001254 (PFAM); G3DSA:2.40.10.10 (GENE3D); PTHR24258:SF97 (PANTHER); PTHR24258 (PANTHER); IPR018114 (PROSITE_PATTERNS); IPR001254 (PROSITE_PROFILES); IPR009003 (SUPERFAMILY)
243	216616_length_234_cvg_4.7_tip_1_1	0	hemagglutinin	78	3.02E-15	68.60%	0.1	IPR013856 (G3DSA:3.10.170.GENE3D); IPR013856 (PFAM); SSF55486 (SUPERFAMILY)
244	216696_length_234_cvg_123.1_tip_0_4	2107	low-density lipoprotein receptor-related protein 2-partial	78	1.88E-14	64.40%	0.098	IPR002172 (PRINTS); IPR002172 (SMART); IPR002172 (G3DSA:4.10.400.GENE3D); IPR002172 (PFAM); IPR002172 (G3DSA:4.10.400.GENE3D); PTHR10529 (PANTHER); IPR023415 (PROSITE_PATTERNS); IPR002172 (PROSITE_PROFILES); IPR002172 (PROSITE_PROFILES); IPR002172 (SUPERFAMILY); IPR002172 (SUPERFAMILY)
246	217726_length_235_cvg_2.0_tip_1_2	2	coactosin-like protein isoform x1	78	7.41E-07	68.25%	0.143	no IPS match

245	217726_length_235_cvg_2.0_tip_1_0	2	neuronal thread protein ad7c-ntp	79	2.06E-19	62.80%	0.199		PF13900 (PFAM); PTHR16213 (PANTHER); PTHR16213:SF28 (PANTHER)
247	218528_length_236_cvg_4.1_tip_1_0	0	tab y 2 allergen	79	1.52E-08	65.20%	0.550	Y	IPR013785 (G3DSA:3.20.20.GENE3D); IPR018155 (PFAM); SIGNAL_PEPTIDE_C_REGION (PHOBIUS); SIGNAL_PEPTIDE_N_REGION (PHOBIUS); NON_CYTOPLASMIC_DOMAIN (PHOBIUS); SIGNAL_PEPTIDE (PHOBIUS); SIGNAL_PEPTIDE_H_REGION (PHOBIUS); SignalP-TM (SIGNALP_EUK); IPR017853 (SUPERFAMILY); TMhelix (TMHMM)
248	218614_length_236_cvg_3.7_tip_1_3	0	zinc finger protein 358	79	2.10E-17	64.40%	0.115		IPR015880 (SMART); IPR013087 (G3DSA:3.30.160.GENE3D); PF13465 (PFAM); IPR013087 (G3DSA:3.30.160.GENE3D); PTHR24409 (PANTHER); IPR007087 (PROSITE_PATTERNS); IPR007087 (PROSITE_PATTERNS); IPR007087 (PROSITE_PROFILES); IPR007087 (PROSITE_PROFILES); IPR007087 (PROSITE_PROFILES); SSF57667 (SUPERFAMILY); SSF57667 (SUPERFAMILY)
249	218837_length_237_cvg_5.2_tip_1_5	0	tyrosine-protein kinase wsck	79	6.35E-29	72.20%	0.469	Y	G3DSA:1.10.510.10 (GENE3D); IPR001245 (PFAM); PTHR24416 (PANTHER); IPR000719 (PROSITE_PROFILES); SignalP-noTM (SIGNALP_EUK); IPR011009 (SUPERFAMILY)
250	220301_length_238_cvg_61.5_tip_1_5	46	chitinase 8 precursor	79	7.14E-09	61.80%	0.108		IPR001223 (PFAM); IPR013781 (G3DSA:3.20.20.GENE3D); IPR017853 (SUPERFAMILY)
256	221489_length_239_cvg_2.0_tip_1_2	0	laminin subunit gamma-1-like	79	6.01E-06	69.67%	0.237		IPR002049 (SMART); IPR002049 (PFAM); G3DSA:2.10.25.10 (GENE3D); IPR002049 (PROSITE_PROFILES); SSF57196 (SUPERFAMILY)
251	220495_length_238_cvg_2.0_tip_1_3	0	transposase	80	2.33E-09	64.30%	0.122		no IPS match
252	220881_length_239_cvg_63.0_tip_0_3	0	14-3-3 protein zeta isoform x1	80	7.90E-46	95.40%	0.111		IPR000308 (PRINTS); IPR023410 (PFAM); G3DSA:1.20.190.20 (GENE3D); IPR000308 (PANTHER); IPR023410 (SUPERFAMILY)
253	220883_length_239_cvg_37.0_tip_0_3	0	14-3-3 protein zeta isoform x1	80	1.06E-43	91.60%	0.108		IPR000308 (PRINTS); IPR023410 (PFAM); G3DSA:1.20.190.20 (GENE3D); IPR000308 (PANTHER); IPR023410 (SUPERFAMILY)
254	221143_length_239_cvg_63.0_tip_0_4	0	carboxypeptidase b	80	5.25E-26	69.70%	0.108		IPR000834 (PFAM); G3DSA:3.40.630.10 (GENE3D); PTHR11705 (PANTHER); SSF53187 (SUPERFAMILY)
255	221301_length_239_cvg_96.7_tip_1_0	417	a chain crystal structure of porcine beta trypsin with % polydocanol	80	1.68E-13	67.80%	0.104		G3DSA:2.40.10.10 (GENE3D); G3DSA:2.40.10.10 (GENE3D); IPR001254 (PFAM); PTHR24265 (PANTHER); IPR018114 (PROSITE_PATTERNS); IPR001254 (PROSITE_PROFILES); IPR009003 (SUPERFAMILY)
257	221619_length_240_cvg_2.0_tip_1_1	0	gastrula zinc finger	80	1.33E-15	62.20%	0.114		IPR015880 (SMART); IPR013087 (G3DSA:3.30.160.GENE3D); PF13465 (PFAM); IPR013087 (G3DSA:3.30.160.GENE3D); PF13912 (PFAM); IPR013087 (G3DSA:3.30.160.GENE3D); PTHR24409 (PANTHER); IPR007087 (PROSITE_PATTERNS); IPR007087 (PROSITE_PATTERNS); IPR007087 (PROSITE_PROFILES); IPR007087 (PROSITE_PROFILES); SSF57667 (SUPERFAMILY); SSF57667 (SUPERFAMILY)
258	221775_length_240_cvg_20.0_tip_0_5	0	partial	80	6.76E-42	93.90%	0.1		IPR006689 (PFAM); IPR027417 (G3DSA:3.40.50.GENE3D); PTHR11711:SF146 (PANTHER); PTHR11711 (PANTHER); IPR024156 (PROSITE_PROFILES); IPR027417 (SUPERFAMILY)
259	222416_length_240_cvg_10.1_tip_1_2	0	odorranain-e1 antimicrobial peptide precursor	80	9.75E-06	61.00%	0.164		no IPS match
261	223378_length_241_cvg_2.0_tip_1_4	0	wd repeat domain isoform cra_c	80	8.19E-09	78.63%	0.127		no IPS match
262	223378_length_241_cvg_2.0_tip_1_5	0	low quality protein: lyr motif-containing protein partial	80	2.93E-12	79.60%	0.113		PF13900 (PFAM); PTHR16213 (PANTHER)
263	223830_length_242_cvg_2.0_tip_1_2	0	lysosomal alpha-mannosidase	80	4.46E-33	83.40%	0.11		IPR000602 (PFAM); IPR027291 (G3DSA:3.20.110.GENE3D); PTHR11607 (PANTHER); PTHR11607:SF3 (PANTHER); IPR011330 (SUPERFAMILY)
260	223378_length_241_cvg_2.0_tip_1_3	0	hypothetical calcrl-related pioneer protein	81	1.49E-09	72.00%	0.164		PTHR12138 (PANTHER)
264	224158_length_242_cvg_2.0_tip_1_1	0	peptidyl-prolyl cis-trans rhodopsin-specific isozyme	81	8.08E-31	78.70%	0.123		IPR002130 (PRINTS); IPR002130 (PFAM); IPR029000 (G3DSA:2.40.100.GENE3D); IPR024936 (PANTHER); IPR020892 (PROSITE_PATTERNS); IPR002130 (PROSITE_PROFILES); IPR029000 (SUPERFAMILY)
265	224530_length_243_cvg_2.0_tip_1_4	0	leukotriene a-4 partial	81	1.33E-35	83.50%	0.123		IPR014782 (PFAM); G3DSA:1.10.390.10 (GENE3D); PTHR11533:SF4 (PANTHER); IPR001930 (PANTHER); SSF55486 (SUPERFAMILY)
266	229941_length_249_cvg_3.4_tip_1_2	0	balbiani ring protein 3-like	83	4.91E-08	57.00%	0.101		IPR001506 (PRINTS); IPR024079 (G3DSA:3.40.390.GENE3D); IPR001506 (PFAM)
267	230539_length_250_cvg_2.0_tip_1_2	0	choline partial	83	3.68E-51	100.00%	0.103		G3DSA:3.30.560.10 (GENE3D); G3DSA:3.50.50.60 (GENE3D); IPR007867 (PFAM); PTHR11552 (PANTHER); SSF51905 (SUPERFAMILY)
270	231865_length_251_cvg_2.0_tip_1_5	0	carbohydrate sulfotransferase 11-like isoform x1	83	1.59E-24	71.70%	0.118		IPR005331 (PFAM); IPR018011 (PANTHER); PTHR12137:SF29 (PANTHER)
268	231227_length_250_cvg_5.5_tip_1_0	0	zinc finger protein 502-like	84	4.09E-15	65.30%	0.102		IPR015880 (SMART); IPR007087 (PFAM); PF13894 (PFAM); IPR013087 (G3DSA:3.30.160.GENE3D); IPR013087 (G3DSA:3.30.160.GENE3D); PTHR24409 (PANTHER); PTHR24409:SF14 (PANTHER); IPR007087 (PROSITE_PATTERNS); IPR007087 (PROSITE_PATTERNS); IPR007087 (PROSITE_PATTERNS); IPR007087 (PROSITE_PROFILES); IPR007087 (PROSITE_PROFILES); IPR007087 (PROSITE_PROFILES); SSF57667 (SUPERFAMILY); SSF57667 (SUPERFAMILY)
269	231647_length_251_cvg_2.0_tip_1_1	2	3-hydroxyisobutyrate dehydrogenase	84	5.28E-37	90.60%	0.121		IPR029154 (PFAM); IPR016040 (G3DSA:3.40.50.GENE3D); IPR006115 (PFAM); IPR013328 (G3DSA:1.10.1040.GENE3D); PTHR22981 (PANTHER); PTHR22981:SF7 (PANTHER); IPR008927 (SUPERFAMILY); SSF51735 (SUPERFAMILY)
271	233341_length_253_cvg_2.0_tip_1_1	0	adp-ribosylation factor 6	84	8.06E-30	98.90%	0.153		IPR006689 (PFAM); IPR027417 (G3DSA:3.40.50.GENE3D); PTHR11711:SF151 (PANTHER); PTHR11711 (PANTHER); IPR027417 (SUPERFAMILY)

272	234371_length_254_cvg_40.0_tip_1_0	0	adp-ribosylation factor	85	2.43E-09	88.90%	0.218		IPR006689 (PFAM); IPR027417 (G3DSA:3.40.50.GENE3D); PTHR11711 (PANTHER); PTHR11711:SF142 (PANTHER); IPR027417 (SUPERFAMILY)
273	234561_length_254_cvg_2.0_tip_1_3	0	tat (twin-arginine translocation) pathway signal sequence domain protein	85	6.66E-55	99.80%	0.101		IPR006963 (PFAM); G3DSA:3.40.50.740 (GENE3D); IPR006656 (PFAM); G3DSA:2.20.25.90 (GENE3D); PTHR11615 (PANTHER); PTHR11615:SF37 (PANTHER); IPR006963 (PROSITE_PROFILES); SSF53706 (SUPERFAMILY)
274	235091_length_255_cvg_12.0_tip_1_1	0	14-3-3 partial	85	3.61E-48	94.10%	0.106		IPR000308 (PRINTS); IPR023410 (SMART); G3DSA:1.20.190.20 (GENE3D); IPR023410 (PFAM); IPR000308 (PANTHER); IPR023410 (SUPERFAMILY)
275	235243_length_255_cvg_4.8_tip_1_5	0	nidogen- partial	85	3.23E-09	61.30%	0.157		IPR000716 (SMART); IPR000716 (G3DSA:4.10.800.GENE3D); IPR000716 (PFAM); IPR000716 (PROSITE_PATTERNS); IPR000716 (PROSITE_PROFILES); IPR000716 (SUPERFAMILY)
276	236013_length_256_cvg_2.0_tip_1_1	2	von willebrand factor a domain-containing protein 8	85	6.56E-13	99.20%	0.194		PTHR21610 (PANTHER); PTHR21610:SF9 (PANTHER); CYTOPLASMIC_DOMAIN (PHOBIUS); NON_CYTOPLASMIC_DOMAIN (PHOBIUS); TRANSMEMBRANE (PHOBIUS); TMhelix (TMHMM)
277	236653_length_257_cvg_2.0_tip_1_2	0	laminin subunit beta-1	85	6.24E-11	74.50%	0.1		IPR013015 (PROSITE_PROFILES)
278	237293_length_257_cvg_2.6_tip_1_3	0	inactive dipeptidyl	86	9.64E-27	80.50%	0.125		IPR029058 (G3DSA:3.40.50.GENE3D); IPR001375 (PFAM); PTHR11731:SF103 (PANTHER); PTHR11731 (PANTHER); IPR029058 (SUPERFAMILY)
279	237309_length_257_cvg_2.0_tip_1_4	0	cathepsin o-like	86	4.93E-33	83.60%	0.116		G3DSA:3.90.70.10 (GENE3D); IPR000668 (PFAM); PTHR12411:SF287 (PANTHER); IPR013128 (PANTHER); IPR025660 (PROSITE_PATTERNS); SSF54001 (SUPERFAMILY)
280	238113_length_258_cvg_2.0_tip_1_1	0	nidogen-2 isoform x1	86	8.02E-34	82.70%	0.102		IPR000033 (SMART); IPR011042 (G3DSA:2.120.10.GENE3D); IPR000033 (PFAM); PTHR10529 (PANTHER); IPR000033 (PROSITE_PROFILES); IPR000033 (PROSITE_PROFILES); IPR000033 (PROSITE_PROFILES); SSF63825 (SUPERFAMILY)
281	239445_length_260_cvg_63.0_tip_1_3	827	trypsin-1-like isoform x1	87	2.12E-16	66.10%	0.157		G3DSA:2.40.10.10 (GENE3D); IPR001254 (PFAM); PTHR24255 (PANTHER); IPR018114 (PROSITE_PATTERNS); IPR001254 (PROSITE_PROFILES); IPR009003 (SUPERFAMILY)
282	239589_length_260_cvg_16.0_tip_1_0	4	venom protease-like	87	1.19E-06	60.00%	0.630	Y	IPR001254 (PFAM); G3DSA:2.40.10.10 (GENE3D); SignalP-noTM (SIGNALP_EUK); IPR009003 (SUPERFAMILY)
283	240267_length_261_cvg_98.3_tip_0_5	1760	basement membrane-specific heparan sulfate proteoglycan core protein	87	5.78E-18	64.00%	0.103		IPR002172 (PRINTS); IPR002172 (SMART); IPR002172 (PFAM); IPR002172 (G3DSA:4.10.400.GENE3D); IPR002172 (G3DSA:4.10.400.GENE3D); PTHR10529 (PANTHER); IPR023415 (PROSITE_PATTERNS); IPR023415 (PROSITE_PATTERNS); IPR002172 (PROSITE_PROFILES); IPR002172 (PROSITE_PROFILES); IPR002172 (SUPERFAMILY); IPR002172 (SUPERFAMILY)
284	242763_length_264_cvg_4.0_tip_1_4	4	attractin-like protein 1	88	4.02E-23	70.70%	0.105		PF13854 (PFAM); IPR015915 (G3DSA:2.120.10.GENE3D); IPR002165 (PFAM); PTHR10574:SF223 (PANTHER); PTHR10574 (PANTHER); SSF117281 (SUPERFAMILY)
285	242967_length_264_cvg_5.9_tip_1_5	0	-like protein 3	88	4.10E-20	74.10%	0.114		G3DSA:3.30.70.1500 (GENE3D); IPR002634 (PFAM); PTHR12735 (PANTHER); PTHR12735:SF4 (PANTHER); IPR002634 (SUPERFAMILY)
286	243335_length_265_cvg_3.4_tip_1_4	0	hypothetical protein, partial	88	4.45E-06	50.00%	0.390	Y	SIGNAL_PEPTIDE_C_REGION (PHOBIUS); SIGNAL_PEPTIDE_H_REGION (PHOBIUS); SIGNAL_PEPTIDE (PHOBIUS); NON_CYTOPLASMIC_DOMAIN (PHOBIUS); SIGNAL_PEPTIDE_N_REGION (PHOBIUS); SignalP-TM (SIGNALP_GRAM_POSITIVE)
287	243351_length_265_cvg_2.0_tip_1_2	0	dnaj homolog subfamily c member 18	88	3.16E-31	87.30%	0.102		IPR001623 (PRINTS); IPR001623 (SMART); IPR001623 (G3DSA:1.10.287.GENE3D); IPR001623 (PFAM); PTHR24078:SF15 (PANTHER); PTHR24078 (PANTHER); IPR001623 (PROSITE_PROFILES); IPR001623 (SUPERFAMILY)
288	243423_length_265_cvg_81.6_tip_0_5	7	carboxypeptidase b-like	88	6.35E-29	75.60%	0.122		IPR000834 (PFAM); G3DSA:3.40.630.10 (GENE3D); PTHR11705 (PANTHER); SSF53187 (SUPERFAMILY)
289	244475_length_266_cvg_2.0_tip_1_0	0	hemagglutinin	89	1.13E-09	55.20%	0.134		G3DSA:1.10.390.10 (GENE3D)
290	244758_length_266_cvg_2.0_tip_1_4	0	serine protease	89	1.49E-07	56.10%	0.098		G3DSA:2.40.10.10 (GENE3D); IPR001254 (PFAM); IPR009003 (SUPERFAMILY)
291	246412_length_268_cvg_3.0_tip_1_4	0	6-phospho-beta- partial	89	2.41E-57	97.80%	0.148		IPR001360 (PFAM); IPR013781 (G3DSA:3.20.20.GENE3D); IPR001360 (PANTHER); PTHR10353:SF35 (PANTHER); IPR017853 (SUPERFAMILY)
292	246604_length_269_cvg_4.0_tip_0_2	0	upf0364 protein c6orf211 homolog	89	9.87E-21	70.50%	0.123		IPR002791 (PFAM); PTHR12260:SF1 (PANTHER); PTHR12260 (PANTHER); IPR002791 (SUPERFAMILY)
293	246606_length_269_cvg_8.5_tip_0_2	0	upf0364 protein c6orf211 homolog	89	3.03E-19	68.70%	0.141		IPR002791 (PFAM); PTHR12260 (PANTHER); PTHR12260:SF1 (PANTHER); IPR002791 (SUPERFAMILY)
294	247188_length_269_cvg_2.0_tip_1_1	0	down syndrome cell adhesion molecule-like protein dscam2	90	2.01E-22	71.00%	0.135		IPR013783 (G3DSA:2.60.40.GENE3D); IPR003961 (PFAM); PTHR10489 (PANTHER); PTHR10489:SF567 (PANTHER); IPR003961 (PROSITE_PROFILES); IPR003961 (SUPERFAMILY)
295	247890_length_270_cvg_2.0_tip_1_5	0	tryptophan partial	90	3.39E-53	99.80%	0.148		IPR018227 (PFAM); PTHR32195 (PANTHER); PTHR32195:SF11 (PANTHER); NON_CYTOPLASMIC_DOMAIN (PHOBIUS); CYTOPLASMIC_DOMAIN (PHOBIUS); NON_CYTOPLASMIC_DOMAIN (PHOBIUS); TRANSMEMBRANE (PHOBIUS); TRANSMEMBRANE (PHOBIUS); CYTOPLASMIC_DOMAIN (PHOBIUS); TRANSMEMBRANE (PHOBIUS); TMhelix (TMHMM); TMhelix (TMHMM)

298	249596_length_272_cvg_2.0_tip_1_2	0	gamma-aminobutyraldehyde dehydrogenase	90	1.10E-56	98.20%	0.102	IPR016163 (G3DSA:3.40.309.GENE3D); IPR015590 (PFAM); PTHR11699:SF109 (PANTHER); PTHR11699 (PANTHER); IPR016161 (SUPERFAMILY)
296	248344_length_271_cvg_4.9_tip_1_0	0	family partial	91	6.30E-57	100.00%	0.121	IPR006664 (PRINTS); IPR006665 (PFAM); IPR006665 (G3DSA:3.30.1330.GENE3D); PTHR30128:SF0 (PANTHER); PTHR30128 (PANTHER); IPR006690 (PROSITE_PATTERNS); IPR006665 (PROSITE_PROFILES); IPR006665 (SUPERFAMILY)
297	249594_length_272_cvg_16.9_tip_0_0	0	zinc finger protein 235-like	91	1.59E-12	56.90%	0.12	IPR015880 (SMART); IPR013087 (G3DSA:3.30.160.GENE3D); IPR007087 (PFAM); IPR013087 (G3DSA:3.30.160.GENE3D); PF13894 (PFAM); PTHR24409 (PANTHER); PTHR24409:SF14 (PANTHER); IPR007087 (PROSITE_PATTERNS); IPR007087 (PROSITE_PROFILES); IPR007087 (PROSITE_PROFILES); SSF57667 (SUPERFAMILY); SSF57667 (SUPERFAMILY)
299	250054_length_273_cvg_93.9_tip_0_0	2	annexin partial	91	1.50E-28	76.80%	0.11	Coil (COILS); IPR001464 (PRINTS); IPR018502 (SMART); IPR018502 (PFAM); IPR018502 (G3DSA:1.10.220.GENE3D); PTHR10502 (PANTHER); IPR018252 (PROSITE_PATTERNS); SSF47874 (SUPERFAMILY)
300	250320_length_273_cvg_84.9_tip_0_3	14	annexin partial	91	6.08E-29	78.60%	0.103	IPR001464 (PRINTS); IPR018502 (SMART); IPR018502 (G3DSA:1.10.220.GENE3D); IPR018502 (PFAM); PTHR10502 (PANTHER); IPR018252 (PROSITE_PATTERNS); SSF47874 (SUPERFAMILY)
301	250426_length_273_cvg_53.0_tip_1_0	0	isoform cra_b	91	1.38E-56	100.00%	0.128	IPR019956 (PRINTS); IPR000626 (SMART); G3DSA:3.10.20.90 (GENE3D); IPR000626 (PFAM); G3DSA:3.10.20.90 (GENE3D); PTHR10666 (PANTHER); IPR019954 (PROSITE_PATTERNS); IPR000626 (PROSITE_PROFILES); IPR029071 (SUPERFAMILY)
302	251628_length_275_cvg_57.7_tip_1_2	15	low-density lipoprotein receptor-related protein 2	91	2.30E-14	64.50%	0.131	IPR002172 (PRINTS); IPR002172 (SMART); IPR002172 (G3DSA:4.10.400.GENE3D); IPR002172 (PFAM); IPR002172 (G3DSA:4.10.400.GENE3D); PTHR10529 (PANTHER); IPR023415 (PROSITE_PATTERNS); IPR002172 (PROSITE_PROFILES); IPR002172 (PROSITE_PROFILES); IPR002172 (SUPERFAMILY); IPR002172 (SUPERFAMILY)
303	251756_length_275_cvg_60.0_tip_1_0	74	chitinase partial	92	1.24E-21	65.00%	0.104	IPR013781 (G3DSA:3.20.20.GENE3D); IPR001223 (PFAM); PTHR11177 (PANTHER); PTHR11177:SF45 (PANTHER); IPR001579 (PROSITE_PATTERNS); IPR017853 (SUPERFAMILY)
304	252826_length_277_cvg_50.0_tip_1_2	0	adp-ribosylation factor 2	92	2.27E-13	100.00%	0.111	IPR006689 (PFAM); IPR027417 (G3DSA:3.40.50.GENE3D); PTHR11711 (PANTHER); PTHR11711:SF145 (PANTHER); CYTOPLASMIC_DOMAIN (PHOBIUS); TRANSMEMBRANE (PHOBIUS); NON_CYTOPLASMIC_DOMAIN (PHOBIUS); IPR027417 (SUPERFAMILY); TMhelix (TMHMM)
305	254410_length_279_cvg_2.0_tip_1_2	0	plexin- partial	93	7.17E-41	84.80%	0.107	IPR001627 (PFAM); IPR015943 (G3DSA:2.130.10.GENE3D); PTHR22625 (PANTHER); PTHR22625:SF16 (PANTHER); IPR001627 (PROSITE_PROFILES); IPR001627 (SUPERFAMILY)
308	255670_length_280_cvg_2.8_tip_1_1	0	serine endoprotease partial	93	1.77E-57	100.00%	0.105	IPR001940 (PRINTS); PF13365 (PFAM); G3DSA:2.40.10.10 (GENE3D); PTHR22939 (PANTHER); PTHR22939:SF79 (PANTHER); IPR009003 (SUPERFAMILY)
306	255344_length_280_cvg_3.0_tip_1_0	2	ankyrin repeat-containing protein	94	2.55E-12	59.40%	0.119	IPR002110 (PRINTS); IPR002110 (SMART); IPR020683 (PFAM); IPR020683 (G3DSA:1.25.40.GENE3D); PTHR24198 (PANTHER); IPR002110 (PROSITE_PROFILES); IPR020683 (PROSITE_PROFILES); IPR002110 (PROSITE_PROFILES); IPR020683 (SUPERFAMILY)
307	255456_length_280_cvg_2.0_tip_1_0	0	hemocyte protein-glutamine gamma-glutamyltransferase-like	94	3.58E-35	79.70%	0.104	IPR013783 (G3DSA:2.60.40.GENE3D); IPR002931 (G3DSA:3.90.260.GENE3D); IPR023608 (PANTHER); PTHR11590:SF40 (PANTHER); SSF54001 (SUPERFAMILY)
309	257696_length_283_cvg_8.6_tip_1_2	0	laccase 1	94	3.20E-34	76.00%	0.121	IPR008972 (G3DSA:2.60.40.GENE3D); IPR011707 (PFAM); PTHR11709 (PANTHER); PTHR11709:SF33 (PANTHER); IPR008972 (SUPERFAMILY)
311	260749_length_287_cvg_2.0_tip_1_2	2	---NA---	95			0.151	IPR002172 (SMART); IPR002172 (PFAM); IPR002172 (G3DSA:4.10.400.GENE3D); IPR023415 (PROSITE_PATTERNS); IPR002172 (PROSITE_PROFILES); IPR002172 (SUPERFAMILY)
310	259772_length_286_cvg_2.0_tip_1_3	1	tyrosine-protein kinase-like 7	96	7.99E-25	71.70%	0.102	IPR003599 (SMART); IPR003598 (SMART); IPR013783 (G3DSA:2.60.40.GENE3D); IPR013098 (PFAM); IPR007110 (PROSITE_PROFILES); SSF48726 (SUPERFAMILY)
312	261405_length_288_cvg_2.0_tip_1_0	2	zinc c2h2 type domain-containing protein	96	3.05E-19	60.30%	0.102	IPR015880 (SMART); PF13465 (PFAM); PF13894 (PFAM); IPR013087 (G3DSA:3.30.160.GENE3D); IPR013087 (G3DSA:3.30.160.GENE3D); IPR013087 (G3DSA:3.30.160.GENE3D); PTHR24409 (PANTHER); IPR007087 (PROSITE_PATTERNS); IPR007087 (PROSITE_PATTERNS); IPR007087 (PROSITE_PATTERNS); IPR007087 (PROSITE_PROFILES); IPR007087 (PROSITE_PROFILES); IPR007087 (PROSITE_PROFILES); SSF57667 (SUPERFAMILY); SSF57667 (SUPERFAMILY)
313	261963_length_289_cvg_2.0_tip_1_5	0	n-acetylmuramoyl-l-alanine partial	96	5.98E-62	100.00%	0.117	IPR002508 (SMART); IPR002508 (PFAM); IPR002508 (G3DSA:3.40.630.GENE3D); PTHR30404:SF0 (PANTHER); PTHR30404 (PANTHER); SSF53187 (SUPERFAMILY)

314	262447_length_290_cvg_3.9_tip_1_3	2	zinc finger protein ozf-like	97	1.48E-24	64.50%	0.192	IPR015880 (SMART); IPR013087 (G3DSA:3.30.160.GENE3D); PF13465 (PFAM); IPR013087 (G3DSA:3.30.160.GENE3D); PTHR24409 (PANTHER); IPR007087 (PROSITE_PATTERNS); IPR007087 (PROSITE_PATTERNS); IPR007087 (PROSITE_PATTERNS); IPR007087 (PROSITE_PROFILES); IPR007087 (PROSITE_PROFILES); IPR007087 (PROSITE_PROFILES); SSF57667 (SUPERFAMILY); SSF57667 (SUPERFAMILY)
315	263625_length_291_cvg_3.0_tip_1_1	1	zinc finger protein	97	6.20E-18	63.00%	0.111	IPR015880 (SMART); IPR013087 (G3DSA:3.30.160.GENE3D); IPR013087 (G3DSA:3.30.160.GENE3D); PF13909 (PFAM); PF13465 (PFAM); PTHR24409:SF14 (PANTHER); PTHR24409 (PANTHER); IPR007087 (PROSITE_PATTERNS); IPR007087 (PROSITE_PATTERNS); IPR007087 (PROSITE_PATTERNS); IPR007087 (PROSITE_PROFILES); IPR007087 (PROSITE_PROFILES); IPR007087 (PROSITE_PROFILES); SSF57667 (SUPERFAMILY); SSF57667 (SUPERFAMILY)
316	263999_length_292_cvg_2.0_tip_1_1	1	polypeptide n-acetylgalactosaminyltransferase 9-like isoform x1	97	6.58E-41	91.40%	0.121	PTHR11675 (PANTHER)
317	265441_length_294_cvg_70.6_tip_1_4	92	serine (or cysteine) proteinase clade b member	98	5.69E-13	70.60%	0.113	G3DSA:2.30.39.10 (GENE3D); IPR023796 (PFAM); IPR000215 (PANTHER); PTHR11461:SF52 (PANTHER); IPR023795 (PROSITE_PATTERNS); IPR023796 (SUPERFAMILY)
318	265955_length_294_cvg_5.5_tip_1_2	0	zinc finger protein draculin	98	1.20E-12	57.00%	0.103	IPR015880 (SMART); IPR007087 (PFAM); PF13894 (PFAM); IPR013087 (G3DSA:3.30.160.GENE3D); IPR013087 (G3DSA:3.30.160.GENE3D); PTHR24409 (PANTHER); PTHR24409:SF14 (PANTHER); IPR007087 (PROSITE_PATTERNS); IPR007087 (PROSITE_PATTERNS); IPR007087 (PROSITE_PROFILES); IPR007087 (PROSITE_PROFILES); IPR007087 (PROSITE_PROFILES); SSF57667 (SUPERFAMILY); SSF57667 (SUPERFAMILY)
319	266815_length_295_cvg_4.0_tip_1_2	1	gamma-glutamyl hydrolase	98	6.47E-29	71.80%	0.113	IPR011697 (PFAM); IPR029062 (G3DSA:3.40.50.GENE3D); IPR015527 (PANTHER); IPR015527 (PROSITE_PROFILES); IPR029062 (SUPERFAMILY)
320	267525_length_296_cvg_2.2_tip_1_4	1	n-acetylmuramoyl-l-alanine amidase family partial	99	1.17E-51	100.00%	0.101	IPR002508 (SMART); IPR002508 (PFAM); IPR002508 (G3DSA:3.40.630.GENE3D); PTHR30404 (PANTHER); PTHR30404:SF0 (PANTHER); SSF53187 (SUPERFAMILY)
321	267961_length_297_cvg_2.0_tip_1_5	0	bifunctional chitinase lysozyme domain protein	99	4.63E-54	99.80%	0.107	CYTOPLASMIC_DOMAIN (PHOBIUS); NON_CYTOPLASMIC_DOMAIN (PHOBIUS); TRANSMEMBRANE (PHOBIUS); TMhelix (TMHMM)
322	268179_length_297_cvg_2.0_tip_1_5	1	agrin-like isoform x2	99	9.40E-20	59.40%	0.121	G3DSA:2.40.50.120 (GENE3D); IPR004850 (PFAM); IPR004850 (PROSITE_PROFILES); IPR008993 (SUPERFAMILY)
323	269203_length_299_cvg_3.0_tip_1_5	2	formate dehydrogenase h	99	8.09E-67	100.00%	0.102	G3DSA:2.20.25.90 (GENE3D); G3DSA:3.40.50.740 (GENE3D); IPR006656 (PFAM); PTHR11615:SF112 (PANTHER); PTHR11615 (PANTHER); IPR006963 (PROSITE_PROFILES); SSF53706 (SUPERFAMILY)
324	270615_length_300_cvg_4.0_tip_1_1	2	beta- -n-acetylgalactosaminyltransferase bre-4	100	1.02E-26	87.00%	0.105	IPR027791 (PFAM); IPR029044 (G3DSA:3.90.550.GENE3D); IPR003859 (PANTHER); PTHR19300:SF30 (PANTHER); IPR029044 (SUPERFAMILY)
325	270965_length_300_cvg_3.0_tip_1_3	2	protein sda1 homolog	100	4.89E-49	88.60%	0.121	IPR007949 (PFAM); PTHR12730 (PANTHER); IPR027312 (PTHR12730:PANTHER)
326	271585_length_300_cvg_3.3_tip_1_1	3	---NA---	100			0.151	TRANSMEMBRANE (PHOBIUS); NON_CYTOPLASMIC_DOMAIN (PHOBIUS); CYTOPLASMIC_DOMAIN (PHOBIUS); TMhelix (TMHMM)
327	273919_length_302_cvg_86.2_tip_0_3	586	venom allergen 3-like	101	8.31E-12	51.40%	0.119	IPR014044 (PFAM); IPR014044 (G3DSA:3.40.33.GENE3D); IPR001283 (PANTHER); IPR014044 (SUPERFAMILY)
328	274859_length_303_cvg_2.8_tip_1_1	2	histidine-rich glyco	101	1.33E-20	53.90%	0.113	no IPS match
329	275207_length_304_cvg_2.0_tip_1_2	1	serine protease 47 isoform x3	101	1.21E-13	56.10%	0.101	G3DSA:2.40.10.10 (GENE3D); G3DSA:2.40.10.10 (GENE3D); IPR001254 (PFAM); PTHR24275 (PANTHER); PTHR24275:SF50 (PANTHER); IPR001254 (PROSITE_PROFILES); IPR009003 (SUPERFAMILY)
330	275259_length_304_cvg_3.0_tip_1_5	1	glycerophosphoryl diester phosphodiesterase domain protein	101	3.41E-64	100.00%	0.566 Y	IPR030395 (PFAM); IPR017946 (G3DSA:3.20.20.GENE3D); PTHR23344:SF7 (PANTHER); IPR004129 (PANTHER); SIGNAL_PEPTIDE_C_REGION (PHOBIUS); SIGNAL_PEPTIDE (PHOBIUS); SIGNAL_PEPTIDE_N_REGION (PHOBIUS); SIGNAL_PEPTIDE_H_REGION (PHOBIUS); NON_CYTOPLASMIC_DOMAIN (PHOBIUS); IPR030395 (PROSITE_PROFILES); SignalP-TM (SIGNALP_GRAM_POSITIVE); SignalP-noTM (SIGNALP_EUK); SignalP-noTM (SIGNALP_GRAM_NEGATIVE); IPR017946 (SUPERFAMILY)
331	275481_length_305_cvg_2.0_tip_1_3	0	trna binding domain protein	102	1.31E-66	100.00%	0.096	IPR012340 (G3DSA:2.40.50.GENE3D); IPR002547 (PFAM); PTHR11586 (PANTHER); IPR002547 (PROSITE_PROFILES); IPR012340 (SUPERFAMILY)
332	275747_length_305_cvg_2.0_tip_1_0	0	hypothetical protein ZHAS_00020562	102	1.99E-26	67.50%	0.123	IPR016090 (G3DSA:1.20.90.GENE3D); IPR001211 (PFAM); PTHR12253 (PANTHER); IPR016090 (SUPERFAMILY)
333	275883_length_306_cvg_7.6_tip_0_1	1	bola-like protein 3	102	5.80E-12	90.70%	0.099	IPR002634 (PFAM); G3DSA:3.30.70.1500 (GENE3D); PTHR12735:SF4 (PANTHER); PTHR12735 (PANTHER); IPR002634 (SUPERFAMILY)

334	276413_length_307_cvg_7.9_tip_1_0	2	zinc finger partial	103	6.79E-21	60.00%	0.137	IPR015880 (SMART); IPR013087 (G3DSA:3.30.160.GENE3D); PF13465 (PFAM); IPR013087 (G3DSA:3.30.160.GENE3D); IPR013087 (G3DSA:3.30.160.GENE3D); PTHR24409:SF14 (PANTHER); PTHR24409 (PANTHER); IPR007087 (PROSITE_PATTERNS); IPR007087 (PROSITE_PATTERNS); IPR007087 (PROSITE_PROFILES); IPR007087 (PROSITE_PROFILES); IPR007087 (PROSITE_PROFILES); SSF57667 (SUPERFAMILY); SSF57667 (SUPERFAMILY)
335	281827_length_318_cvg_44.5_tip_1_3	6	sjchgc07951 protein	106	6.57E-06	55.50%	0.222	IPR002172 (SMART); IPR002172 (PFAM); IPR002172 (G3DSA:4.10.400.GENE3D); NON_CYTOPLASMIC_DOMAIN (PHOBIUS); CYTOPLASMIC_DOMAIN (PHOBIUS); TRANSMEMBRANE (PHOBIUS); IPR002172 (PROSITE_PROFILES); IPR002172 (SUPERFAMILY)
336	282343_length_319_cvg_3.9_tip_1_0	3	retinol dehydrogenase 13	107	8.96E-27	75.80%	0.134	IPR002198 (PFAM); IPR016040 (G3DSA:3.40.50.GENE3D); PTHR24320 (PANTHER); TRANSMEMBRANE (PHOBIUS); NON_CYTOPLASMIC_DOMAIN (PHOBIUS); CYTOPLASMIC_DOMAIN (PHOBIUS); SSF51735 (SUPERFAMILY); TMhelix (TMHMM)
337	283149_length_320_cvg_3.0_tip_1_4	2	zinc finger and scan domain-containing protein 2	107	2.65E-15	52.60%	0.1	IPR015880 (SMART); PF13465 (PFAM); IPR007087 (PFAM); IPR013087 (G3DSA:3.30.160.GENE3D); IPR013087 (G3DSA:3.30.160.GENE3D); IPR013087 (G3DSA:3.30.160.GENE3D); PTHR24409 (PANTHER); PTHR24409:SF13 (PANTHER); IPR007087 (PROSITE_PATTERNS); IPR007087 (PROSITE_PATTERNS); IPR007087 (PROSITE_PROFILES); IPR007087 (PROSITE_PROFILES); IPR007087 (PROSITE_PROFILES); SSF57667 (SUPERFAMILY); SSF57667 (SUPERFAMILY)
338	283595_length_321_cvg_95.8_tip_1_4	140	polycystic kidney disease protein 1-like 2-like	107	8.01E-10	57.60%	0.422 Y	IPR002172 (SMART); IPR002172 (G3DSA:4.10.400.GENE3D); IPR002172 (PFAM); IPR002172 (G3DSA:4.10.400.GENE3D); PTHR10529:SF222 (PANTHER); PTHR10529 (PANTHER); IPR023415 (PROSITE_PATTERNS); IPR002172 (PROSITE_PROFILES); IPR002172 (SUPERFAMILY)
339	283883_length_322_cvg_2.0_tip_1_5	2	lim domain	107	1.04E-26	87.40%	0.099	IPR001245 (PFAM); G3DSA:1.10.510.10 (GENE3D); PTHR23257:SF94 (PANTHER); PTHR23257 (PANTHER); IPR000719 (PROSITE_PROFILES); IPR011009 (SUPERFAMILY)
340	285435_length_326_cvg_81.6_tip_0_2	2688	low quality protein: transmembrane protease serine 11g-like	108	2.64E-15	58.60%	0.117	G3DSA:2.40.10.10 (GENE3D); IPR001254 (PFAM); PTHR24275 (PANTHER); IPR018114 (PROSITE_PATTERNS); IPR001254 (PROSITE_PROFILES); IPR009003 (SUPERFAMILY)
341	285673_length_326_cvg_3.0_tip_1_2	3	12 kda fk506-binding protein	108	9.28E-44	88.40%	0.251	G3DSA:3.10.50.40 (GENE3D); IPR001179 (PFAM); PTHR10516:SF272 (PANTHER); IPR023566 (PANTHER); IPR001179 (PROSITE_PROFILES); SSF54534 (SUPERFAMILY)
342	287179_length_329_cvg_6.0_tip_1_1	1	hemagglutinin amebocyte aggregation factor-like	110	3.64E-20	65.70%	0.111	PF14704 (PFAM); IPR026645 (PANTHER)
343	287459_length_330_cvg_2.4_tip_1_0	1	cell surface	110	5.39E-12	53.70%	0.153	no IPS match
344	287459_length_330_cvg_2.4_tip_1_4	1	collagen alpha type	110	3.84E-22	71.80%	0.12	IPR008160 (PFAM); PTHR24023:SF372 (PANTHER); PTHR24023 (PANTHER)
345	287467_length_330_cvg_2.0_tip_1_1	1	zinc finger protein 239	110	1.98E-19	61.80%	0.101	IPR015880 (SMART); IPR013087 (G3DSA:3.30.160.GENE3D); IPR013087 (G3DSA:3.30.160.GENE3D); IPR007087 (PFAM); PTHR24409 (PANTHER); PTHR24409:SF14 (PANTHER); IPR007087 (PROSITE_PATTERNS); IPR007087 (PROSITE_PATTERNS); IPR007087 (PROSITE_PROFILES); IPR007087 (PROSITE_PROFILES); SSF57667 (SUPERFAMILY); SSF57667 (SUPERFAMILY)
347	288873_length_333_cvg_2.0_tip_1_3	1	actin-depolymerizing factor	111	1.26E-18	66.80%	0.102	IPR007123 (PFAM); IPR029006 (G3DSA:3.40.20.GENE3D); IPR007122 (PANTHER); IPR030004 (PTHR11977:PANTHER); SSF55753 (SUPERFAMILY)
348	288899_length_333_cvg_4.0_tip_1_3	2	#NAME?	111	5.42E-74	100.00%	0.136	IPR018389 (PFAM)
349	288911_length_333_cvg_56.7_tip_1_4	11	dnaj homolog subfamily b member 6-b isoform x1	111	8.92E-28	92.80%	0.105	Coil (COILS); IPR001623 (PRINTS); IPR001623 (SMART); IPR001623 (PFAM); IPR001623 (G3DSA:1.10.287.GENE3D); PTHR24077 (PANTHER); PTHR24077:SF225 (PANTHER); IPR001623 (PROSITE_PROFILES); IPR001623 (SUPERFAMILY)
350	288981_length_333_cvg_84.3_tip_1_0	11554	proclotting enzyme-like isoform x1	111	1.57E-17	58.90%	0.149	IPR001254 (PFAM); G3DSA:2.40.10.10 (GENE3D); PTHR24272 (PANTHER); IPR018114 (PROSITE_PATTERNS); IPR001254 (PROSITE_PROFILES); IPR009003 (SUPERFAMILY)
351	289973_length_335_cvg_2.7_tip_1_0	2	zinc c2h2 type domain-containing protein	112	1.12E-07	50.90%	0.102	PF13465 (PFAM)
352	291251_length_338_cvg_2.0_tip_1_2	0	zinc c2h2 type domain-containing protein	112	7.59E-23	58.00%	0.099	IPR015880 (SMART); PF13465 (PFAM); IPR013087 (G3DSA:3.30.160.GENE3D); IPR013087 (G3DSA:3.30.160.GENE3D); IPR013087 (G3DSA:3.30.160.GENE3D); PTHR24409:SF14 (PANTHER); PTHR24409 (PANTHER); IPR007087 (PROSITE_PATTERNS); IPR007087 (PROSITE_PATTERNS); IPR007087 (PROSITE_PATTERNS); IPR007087 (PROSITE_PATTERNS); IPR007087 (PROSITE_PROFILES); IPR007087 (PROSITE_PROFILES); IPR007087 (PROSITE_PROFILES); IPR007087 (PROSITE_PROFILES); SSF57667 (SUPERFAMILY); SSF57667 (SUPERFAMILY)

353	291367_length_338_cvg_2.0_tip_1_1	2	12 kda fk506-binding protein	113	1.00E-32	97.60%	0.11	G3DSA:3.10.50.40 (GENE3D); IPR001179 (PFAM); PTHR10516:SF260 (PANTHER); IPR023566 (PANTHER); CYTOPLASMIC_DOMAIN (PHOBIUS); TRANSMEMBRANE (PHOBIUS); NON_CYTOPLASMIC_DOMAIN (PHOBIUS); IPR001179 (PROSITE_PROFILES); SSF54534 (SUPERFAMILY); TMhelix (TMHMM)
354	291931_length_340_cvg_2.0_tip_1_5	0	plasma alpha-l-fucosidase	113	6.88E-59	86.20%	0.117	IPR016286 (PRINTS); IPR000933 (SMART); IPR013781 (G3DSA:3.20.20.GENE3D); IPR000933 (PFAM); PTHR10030:SF26 (PANTHER); IPR000933 (PANTHER); IPR017853 (SUPERFAMILY)
355	292487_length_341_cvg_4.0_tip_1_3	1	flavin-containing monooxygenase 2-like	114	3.28E-20	74.70%	0.113	PR00419 (PRINTS); G3DSA:3.50.50.60 (GENE3D); PF13450 (PFAM); PTHR23023:SF4 (PANTHER); PTHR23023 (PANTHER); SSF51905 (SUPERFAMILY)
356	293107_length_342_cvg_13.3_tip_1_0	7	peptidase s8	114	2.26E-09	66.80%	0.103	no IPS match
357	293741_length_344_cvg_2.2_tip_1_1	0	family protein	115	1.47E-48	99.40%	0.125	IPR004629 (PFAM); IPR004629 (TIGRFAM); CYTOPLASMIC_DOMAIN (PHOBIUS); TRANSMEMBRANE (PHOBIUS); NON_CYTOPLASMIC_DOMAIN (PHOBIUS)
358	293975_length_344_cvg_4.6_tip_1_0	4	aldose reductase	115	8.19E-30	70.90%	0.151	IPR020471 (PRINTS); IPR023210 (G3DSA:3.20.20.GENE3D); IPR023210 (PFAM); PTHR11732:SF183 (PANTHER); IPR001395 (PANTHER); IPR023210 (SUPERFAMILY)
359	295365_length_347_cvg_2.0_tip_1_0	1	upf0378 protein kiaa0100	116	6.44E-48	83.70%	0.107	IPR019443 (PFAM); PTHR15678 (PANTHER); PTHR15678:SF6 (PANTHER)
360	296805_length_351_cvg_20.7_tip_0_0	10	low-density lipoprotein receptor 1	117	1.72E-08	51.70%	0.535 Y	IPR002172 (PRINTS); IPR002172 (G3DSA:4.10.400.GENE3D); IPR002172 (G3DSA:4.10.400.GENE3D); IPR002172 (PFAM); SignalP-noTM (SIGNALP_EUK); IPR002172 (SUPERFAMILY); IPR002172 (SUPERFAMILY); TMhelix (TMHMM)
361	296989_length_351_cvg_4.0_tip_1_5	4	protein p21-like	117	1.31E-33	95.80%	0.099	IPR001938 (SMART); IPR001938 (G3DSA:2.60.110.GENE3D); IPR001938 (PFAM); IPR001938 (PANTHER); IPR001938 (PROSITE_PROFILES); IPR001938 (SUPERFAMILY)
362	297115_length_351_cvg_37.4_tip_0_5	15	dnaj homolog subfamily c member 4 isoform x4	117	5.02E-18	72.80%	0.112	IPR001623 (PRINTS); IPR001623 (SMART); IPR001623 (G3DSA:1.10.287.GENE3D); IPR001623 (PFAM); PTHR24077 (PANTHER); IPR001623 (PROSITE_PROFILES); IPR001623 (SUPERFAMILY)
364	297227_length_352_cvg_63.0_tip_0_1	154	muscle m-line assembly protein unc-89-like	117	2.03E-49	83.10%	0.106	IPR003598 (SMART); IPR003599 (SMART); IPR013098 (PFAM); IPR013783 (G3DSA:2.60.40.GENE3D); PTHR19897 (PANTHER); PTHR19897:SF155 (PANTHER); IPR007110 (PROSITE_PROFILES); SSF48726 (SUPERFAMILY)
363	297201_length_352_cvg_2.4_tip_1_3	1	zinc finger protein 235	118	2.13E-22	58.00%	0.114	IPR015880 (SMART); PF13465 (PFAM); IPR013087 (G3DSA:3.30.160.GENE3D); IPR013087 (G3DSA:3.30.160.GENE3D); PTHR24409:SF14 (PANTHER); PTHR24409 (PANTHER); IPR007087 (PROSITE_PATTERNS); IPR007087 (PROSITE_PATTERNS); IPR007087 (PROSITE_PATTERNS); IPR007087 (PROSITE_PROFILES); IPR007087 (PROSITE_PROFILES); IPR007087 (PROSITE_PROFILES); SSF57667 (SUPERFAMILY); SSF57667 (SUPERFAMILY)
365	298537_length_355_cvg_27.5_tip_1_5	13	low quality protein: twitchin-like	118	7.25E-44	82.80%	0.239	IPR003598 (SMART); IPR003599 (SMART); IPR013783 (G3DSA:2.60.40.GENE3D); IPR013098 (PFAM); PTHR19897 (PANTHER); IPR007110 (PROSITE_PROFILES); SSF48726 (SUPERFAMILY)
366	299003_length_356_cvg_7.9_tip_1_1	6	cyclophilin 2	119	3.95E-34	56.00%	0.1	IPR029000 (G3DSA:2.40.100.GENE3D); IPR002130 (PFAM); PTHR11071:SF11 (PANTHER); IPR024936 (PANTHER); IPR002130 (PROSITE_PROFILES); IPR029000 (SUPERFAMILY)
367	299539_length_357_cvg_3.3_tip_1_2	4	adamts-like protein 5	119	6.09E-10	71.80%	0.148	IPR010294 (PFAM); PTHR13723 (PANTHER); CYTOPLASMIC_DOMAIN (PHOBIUS); NON_CYTOPLASMIC_DOMAIN (PHOBIUS); TRANSMEMBRANE (PHOBIUS)
368	300717_length_360_cvg_2.0_tip_1_5	1	partial	120	8.33E-81	100.00%	0.099	IPR001661 (PFAM); IPR001661 (PANTHER); PTHR23403:SF1 (PANTHER); IPR008928 (SUPERFAMILY)
369	300835_length_360_cvg_87.9_tip_0_3	66492	low-density lipoprotein receptor-related protein 5	120	6.96E-16	55.80%	0.103	IPR002172 (PRINTS); IPR002172 (SMART); IPR002172 (G3DSA:4.10.400.GENE3D); IPR002172 (PFAM); PTHR10529 (PANTHER); IPR023415 (PROSITE_PATTERNS); IPR023415 (PROSITE_PATTERNS); IPR002172 (PROSITE_PROFILES); IPR002172 (PROSITE_PROFILES); IPR002172 (SUPERFAMILY); IPR002172 (SUPERFAMILY)
370	301159_length_361_cvg_5.3_tip_1_5	4	heat shock protein 68-like	120	1.00E-28	70.70%	0.099	IPR029048 (G3DSA:1.20.1270.GENE3D); IPR013126 (PFAM); PTHR19375:SF155 (PANTHER); PTHR19375 (PANTHER); IPR029048 (SUPERFAMILY)
371	303032_length_365_cvg_2.0_tip_1_3	3	gamma-glutamyltranspeptidase 1	122	4.15E-13	76.10%	0.205	IPR000101 (PFAM); IPR000101 (PANTHER); PTHR11686:SF15 (PANTHER); IPR029055 (SUPERFAMILY)
372	303208_length_365_cvg_7.4_tip_1_0	1	allergen 5	122	1.73E-08	72.60%	0.206	IPR002413 (PRINTS); IPR014044 (G3DSA:3.40.33.GENE3D); IPR014044 (SUPERFAMILY)
373	304080_length_368_cvg_2.0_tip_1_1	2	zinc finger	123	3.57E-64	92.10%	0.103	IPR015880 (SMART); IPR013087 (G3DSA:3.30.160.GENE3D); PF13465 (PFAM); IPR013087 (G3DSA:3.30.160.GENE3D); PF13894 (PFAM); IPR013087 (G3DSA:3.30.160.GENE3D); PTHR24391:SF9 (PANTHER); PTHR24391 (PANTHER); IPR007087 (PROSITE_PATTERNS); IPR007087 (PROSITE_PATTERNS); IPR007087 (PROSITE_PROFILES); IPR007087 (PROSITE_PROFILES); IPR007087 (PROSITE_PROFILES); SSF57667 (SUPERFAMILY); SSF57667 (SUPERFAMILY)

374	304094_length_368_cvg_92.9_tip_1_3	76389	elastase 2 precursor	123	1.29E-21	61.70%	0.102	IPR001314 (PRINTS); IPR001254 (SMART); IPR001254 (PFAM); G3DSA:2.40.10.10 (GENE3D); G3DSA:2.40.10.10 (GENE3D); PTHR24265 (PANTHER); IPR018114 (PROSITE_PATTERNS); IPR001254 (PROSITE_PROFILES); IPR009003 (SUPERFAMILY)
375	304280_length_368_cvg_4.9_tip_1_1	4	fat-like cadherin-related tumor suppressor partial	123	1.38E-43	73.30%	0.114	IPR000742 (SMART); IPR001881 (SMART); G3DSA:2.10.25.10 (GENE3D); G3DSA:2.10.25.10 (GENE3D); IPR001881 (PFAM); PTHR24026:SF39 (PANTHER); PTHR24026 (PANTHER); IPR018097 (PROSITE_PATTERNS); IPR000152 (PROSITE_PATTERNS); TRANSMEMBRANE (PHOBIUS); CYTOPLASMIC_DOMAIN (PHOBIUS); NON_CYTOPLASMIC_DOMAIN (PHOBIUS); IPR000742 (PROSITE_PROFILES); IPR000742 (PROSITE_PROFILES); SSF57196 (SUPERFAMILY); TMhelix (TMHMM)
376	304798_length_369_cvg_2.0_tip_1_2	2	von willebrand factor type egf and pentraxin domain-containing protein 1-like	123	1.33E-09	63.20%	0.1	IPR000436 (SMART); G3DSA:2.10.70.10 (GENE3D); IPR000436 (PFAM); PTHR19325 (PANTHER); NON_CYTOPLASMIC_DOMAIN (PHOBIUS); CYTOPLASMIC_DOMAIN (PHOBIUS); TRANSMEMBRANE (PHOBIUS); IPR000436 (PROSITE_PROFILES); IPR000436 (SUPERFAMILY); TMhelix (TMHMM)
377	305630_length_371_cvg_30.8_tip_1_2	38	transmembrane protease serine 9-like	123	1.15E-08	57.50%	0.139	G3DSA:2.40.10.10 (GENE3D); IPR001254 (PFAM); IPR009003 (SUPERFAMILY)
378	305984_length_372_cvg_6.4_tip_1_0	2	heat shock cognate protein 70	124	1.15E-29	92.90%	0.305	IPR013126 (PFAM); IPR029048 (G3DSA:1.20.1270.GENE3D); PTHR19375:SF142 (PANTHER); PTHR19375 (PANTHER); CYTOPLASMIC_DOMAIN (PHOBIUS); NON_CYTOPLASMIC_DOMAIN (PHOBIUS); TRANSMEMBRANE (PHOBIUS); IPR029048 (SUPERFAMILY); TMhelix (TMHMM)
379	306170_length_373_cvg_51.4_tip_0_5	29	wnt inhibitory factor 1 precursor	124	2.28E-69	92.20%	0.123	IPR000742 (SMART); G3DSA:2.10.25.10 (GENE3D); IPR013032 (PFAM); IPR000742 (PFAM); PTHR24838:SF276 (PANTHER); PTHR24838 (PANTHER); IPR013032 (PROSITE_PATTERNS); IPR013032 (PROSITE_PATTERNS); IPR013032 (PROSITE_PATTERNS); IPR013032 (PROSITE_PATTERNS); IPR013032 (PROSITE_PATTERNS); IPR013032 (PROSITE_PATTERNS); IPR000742 (PROSITE_PROFILES); IPR000742 (PROSITE_PROFILES); SSF57196 (SUPERFAMILY)
380	306180_length_373_cvg_3.0_tip_1_1	4	cathepsin l	124	2.24E-29	71.30%	0.333	IPR013201 (SMART); G3DSA:3.90.70.10 (GENE3D); IPR013201 (PFAM); IPR013128 (PANTHER); PTHR12411:SF289 (PANTHER); CYTOPLASMIC_DOMAIN (PHOBIUS); TRANSMEMBRANE (PHOBIUS); NON_CYTOPLASMIC_DOMAIN (PHOBIUS); SSF54001 (SUPERFAMILY)
381	307714_length_376_cvg_2.0_tip_1_4	2	hemagglutinin amebocyte aggregation factor-like	125	6.46E-18	68.40%	0.131	PF14704 (PFAM); IPR026645 (PANTHER)
382	308036_length_377_cvg_2.0_tip_1_1	3	lipophorin receptor	126	8.25E-25	68.60%	0.099	G3DSA:2.10.25.10 (GENE3D); PF14670 (PFAM); PTHR10529:SF208 (PANTHER); PTHR10529 (PANTHER); SSF57196 (SUPERFAMILY)
383	309180_length_380_cvg_2.0_tip_1_5	3	carbonic anhydrase isoform a	126	6.23E-11	57.60%	0.629 Y	IPR001148 (G3DSA:3.10.200.GENE3D); IPR001148 (PFAM); IPR023561 (PANTHER); SIGNAL_PEPTIDE_N_REGION (PHOBIUS); SIGNAL_PEPTIDE_H_REGION (PHOBIUS); SIGNAL_PEPTIDE (PHOBIUS); NON_CYTOPLASMIC_DOMAIN (PHOBIUS); SIGNAL_PEPTIDE_C_REGION (PHOBIUS); IPR001148 (PROSITE_PROFILES); SignalP-noTM (SIGNALP_EUK); IPR001148 (SUPERFAMILY)
384	309426_length_380_cvg_4.6_tip_1_2	6	vesicle-fusing atpase 1	126	2.57E-70	91.70%	0.104	IPR027417 (G3DSA:3.40.50.GENE3D); IPR003959 (PFAM); PTHR23078 (PANTHER); IPR027417 (SUPERFAMILY)
385	310200_length_382_cvg_2.0_tip_1_1	2	poxb regulator partial	127	2.75E-90	100.00%	0.137	IPR018149 (PRINTS); IPR004364 (PFAM); G3DSA:3.30.930.10 (GENE3D); IPR018150 (PANTHER); IPR004525 (PTHR22594:PANTHER); SSF55681 (SUPERFAMILY)
386	310352_length_382_cvg_3.0_tip_1_5	3	partial	127	1.46E-14	62.30%	0.108	IPR005203 (G3DSA:2.60.40.GENE3D); IPR005203 (PFAM); IPR013788 (PANTHER); PTHR11511:SF24 (PANTHER); IPR014756 (SUPERFAMILY)
387	310426_length_383_cvg_2.5_tip_1_1	3	zinc finger protein	128	1.28E-21	57.60%	0.099	IPR015880 (SMART); IPR013087 (G3DSA:3.30.160.GENE3D); PF13465 (PFAM); PF13894 (PFAM); IPR013087 (G3DSA:3.30.160.GENE3D); IPR013087 (G3DSA:3.30.160.GENE3D); PTHR24409 (PANTHER); PTHR24409:SF14 (PANTHER); IPR007087 (PROSITE_PATTERNS); IPR007087 (PROSITE_PATTERNS); IPR007087 (PROSITE_PATTERNS); IPR007087 (PROSITE_PROFILES); IPR007087 (PROSITE_PROFILES); IPR007087 (PROSITE_PROFILES); IPR007087 (PROSITE_PROFILES); IPR007087 (PROSITE_PROFILES); SSF57667 (SUPERFAMILY); SSF57667 (SUPERFAMILY)
388	310984_length_384_cvg_2.9_tip_1_2	4	collagen alpha-1 chain-like	128	1.05E-18	61.40%	0.107	IPR008160 (PFAM); PTHR24023:SF379 (PANTHER); PTHR24023 (PANTHER)
389	310984_length_384_cvg_2.9_tip_1_5	4	collagen-like protein	128	7.74E-14	49.90%	0.147	no IPS match
390	311402_length_385_cvg_3.7_tip_1_2	4	adam 17-like protease	128	1.21E-32	67.10%	0.111	IPR024079 (G3DSA:3.40.390.GENE3D); PTHR11905:SF121 (PANTHER); PTHR11905 (PANTHER); CYTOPLASMIC_DOMAIN (PHOBIUS); NON_CYTOPLASMIC_DOMAIN (PHOBIUS); TRANSMEMBRANE (PHOBIUS); CYTOPLASMIC_DOMAIN (PHOBIUS); TRANSMEMBRANE (PHOBIUS); TMhelix (TMHMM)
392	312112_length_386_cvg_3.0_tip_1_2	3	c-ter truncated protein	128	1.21E-86	99.80%	0.117	G3DSA:3.80.10.10 (GENE3D); SSF52058 (SUPERFAMILY)

393	312136_length_386_cvg_9.2_tip_1_2	8	von willebrand factor	128	8.21E-07	48.20%	0.102	no IPS match
391	311666_length_385_cvg_3.2_tip_1_0	4	tyrosine-protein kinase ryk-like	129	9.12E-14	84.30%	0.117	IPR001245 (PFAM); G3DSA:1.10.510.10 (GENE3D); PTHR24416 (PANTHER); PTHR24416:SF256 (PANTHER); IPR008266 (PROSITE_PATTERNS); IPR000719 (PROSITE_PROFILES); IPR011009 (SUPERFAMILY)
424	326392_length_421_cvg_59.9_tip_1_0	206	hemagglutinin amebocyte aggregation factor-like	129	1.28E-14	66.90%	0.16	PF14704 (PFAM); IPR026645 (PANTHER); CYTOPLASMIC_DOMAIN (PHOBIUS); NON_CYTOPLASMIC_DOMAIN (PHOBIUS); CYTOPLASMIC_DOMAIN (PHOBIUS); TRANSMEMBRANE (PHOBIUS); TRANSMEMBRANE (PHOBIUS); TMhelix (TMHMM); TMhelix (TMHMM)
394	313369_length_389_cvg_3.1_tip_1_3	2	nedd4 family-interacting protein 1-like	130	6.49E-58	83.40%	0.126	IPR019325 (PFAM); PTHR13396 (PANTHER); TRANSMEMBRANE (PHOBIUS); NON_CYTOPLASMIC_DOMAIN (PHOBIUS); TRANSMEMBRANE (PHOBIUS); TRANSMEMBRANE (PHOBIUS); NON_CYTOPLASMIC_DOMAIN (PHOBIUS); CYTOPLASMIC_DOMAIN (PHOBIUS); NON_CYTOPLASMIC_DOMAIN (PHOBIUS); TRANSMEMBRANE (PHOBIUS); CYTOPLASMIC_DOMAIN (PHOBIUS); TMhelix (TMHMM); TMhelix (TMHMM); TMhelix (TMHMM)
395	313647_length_390_cvg_7.1_tip_1_0	8	peptidase s8	130	2.47E-12	68.30%	0.361 Y	SIGNAL_PEPTIDE_C_REGION (PHOBIUS); SIGNAL_PEPTIDE (PHOBIUS); NON_CYTOPLASMIC_DOMAIN (PHOBIUS); SIGNAL_PEPTIDE_N_REGION (PHOBIUS); SIGNAL_PEPTIDE_H_REGION (PHOBIUS)
396	313669_length_390_cvg_98.8_tip_1_0	768	carboxypeptidase a2-like	130	9.09E-11	57.90%	0.247	IPR003146 (PFAM); IPR003146 (G3DSA:3.30.70.GENE3D); PTHR11705 (PANTHER); TRANSMEMBRANE (PHOBIUS); NON_CYTOPLASMIC_DOMAIN (PHOBIUS); CYTOPLASMIC_DOMAIN (PHOBIUS); IPR009020 (SUPERFAMILY); TMhelix (TMHMM)
398	314219_length_391_cvg_2.6_tip_1_2	4	complement c1q-like protein 4 isoform x2	130	1.16E-06	52.50%	0.156	IPR001073 (PFAM); IPR008983 (G3DSA:2.60.120.GENE3D); IPR001073 (PROSITE_PROFILES); IPR008983 (SUPERFAMILY)
411	319977_length_405_cvg_12.5_tip_1_0	13	phospholipase a2 isozymes pa3a pa3b partial	130	7.38E-17	51.30%	0.104	IPR016090 (G3DSA:1.20.90.GENE3D); IPR001211 (PFAM); PTHR12253 (PANTHER); PTHR12253:SF8 (PANTHER); IPR016090 (SUPERFAMILY)
416	322165_length_410_cvg_54.8_tip_1_3	151	hemagglutinin amebocyte aggregation factor-like	130	4.32E-16	67.70%	0.177	PF14704 (PFAM); IPR026645 (PANTHER)
397	313973_length_391_cvg_65.6_tip_1_0	910	chitinase partial	131	3.19E-12	54.00%	0.105	IPR001223 (PFAM); IPR013781 (G3DSA:3.20.20.GENE3D); IPR017853 (SUPERFAMILY)
399	314601_length_392_cvg_3.7_tip_1_1	2	tyrosine-protein kinase dnt-like	131	1.58E-24	85.40%	0.113	IPR001245 (PFAM); G3DSA:1.10.510.10 (GENE3D); PTHR24416:SF302 (PANTHER); PTHR24416 (PANTHER); IPR011009 (SUPERFAMILY)
400	315893_length_395_cvg_2.0_tip_1_1	3	catalase-like isoform x1	132	1.93E-34	79.20%	0.32	IPR011614 (G3DSA:2.40.180.GENE3D); IPR011614 (PFAM); IPR018028 (PANTHER); IPR018028 (PROSITE_PROFILES); IPR020835 (SUPERFAMILY)
401	316635_length_397_cvg_16.8_tip_1_4	13	protein vein	132	1.40E-33	60.20%	0.126	IPR000742 (SMART); IPR013783 (G3DSA:2.60.40.GENE3D); G3DSA:2.10.25.10 (GENE3D); IPR000742 (PFAM); PTHR11100 (PANTHER); IPR013032 (PROSITE_PATTERNS); IPR013032 (PROSITE_PATTERNS); IPR007110 (PROSITE_PROFILES); IPR000742 (PROSITE_PROFILES); SSF57196 (SUPERFAMILY); SSF48726 (SUPERFAMILY)
403	316853_length_398_cvg_60.7_tip_0_5	46	PREDICTED: putative uncharacterized protein ART2-like, partial	132	2.01E-10	74.10%	0.114	no IPS match
402	316655_length_397_cvg_2.0_tip_1_3	2	venom serine carboxypeptidase-like	133	4.65E-27	69.40%	0.105	IPR029058 (G3DSA:3.40.50.GENE3D); IPR001563 (PFAM); IPR001563 (PANTHER); IPR018202 (PROSITE_PATTERNS); IPR029058 (SUPERFAMILY)
404	317037_length_398_cvg_2.0_tip_1_3	2	tryptophan partial	133	5.76E-28	100.00%	0.106	IPR018227 (PFAM); IPR015422 (G3DSA:3.90.1150.GENE3D); PTHR32195:SF11 (PANTHER); PTHR32195 (PANTHER); IPR013061 (PROSITE_PATTERNS); CYTOPLASMIC_DOMAIN (PHOBIUS); NON_CYTOPLASMIC_DOMAIN (PHOBIUS); TRANSMEMBRANE (PHOBIUS); CYTOPLASMIC_DOMAIN (PHOBIUS); TRANSMEMBRANE (PHOBIUS); IPR015424 (SUPERFAMILY); TMhelix (TMHMM); TMhelix (TMHMM)
406	318163_length_401_cvg_2.0_tip_1_5	1	gelsolin	133	4.59E-34	74.60%	0.215	IPR029006 (G3DSA:3.40.20.GENE3D); IPR030004 (PTHR11977:PANTHER); IPR007122 (PANTHER); SSF55753 (SUPERFAMILY)
405	318159_length_401_cvg_74.8_tip_0_0	91965	serine protease like protein	134	3.58E-22	57.30%	0.1	IPR001314 (PRINTS); IPR001254 (SMART); IPR001254 (PFAM); G3DSA:2.40.10.10 (GENE3D); G3DSA:2.40.10.10 (GENE3D); PTHR24268 (PANTHER); IPR018114 (PROSITE_PATTERNS); IPR001254 (PROSITE_PROFILES); IPR009003 (SUPERFAMILY)
407	318371_length_401_cvg_2.4_tip_1_3	3	mam and ldl-receptor class a domain-containing protein 2-like	134	7.87E-12	56.80%	0.151	IPR002172 (SMART); IPR002172 (G3DSA:4.10.400.GENE3D); NON_CYTOPLASMIC_DOMAIN (PHOBIUS); CYTOPLASMIC_DOMAIN (PHOBIUS); TRANSMEMBRANE (PHOBIUS); IPR002172 (SUPERFAMILY); TMhelix (TMHMM)
408	319001_length_403_cvg_63.0_tip_0_4	11756	ves g 5 allergen	134	2.94E-20	58.30%	0.105	IPR002413 (PRINTS); IPR001283 (PRINTS); IPR014044 (G3DSA:3.40.33.GENE3D); IPR014044 (PFAM); IPR001283 (PANTHER); IPR018244 (PROSITE_PATTERNS); IPR014044 (SUPERFAMILY)
409	319263_length_403_cvg_2.0_tip_1_5	4	ectonucleoside triphosphate diphosphohydrolase 4-like isoform x2	134	8.04E-47	73.30%	0.118	IPR000407 (PFAM); PTHR11782:SF37 (PANTHER); IPR000407 (PANTHER); IPR000407 (PROSITE_PATTERNS)

410	319671_length_404_cvg_2.0_tip_1_0	3	zinc finger and scan domain-containing protein 2-like	135	2.18E-23	61.00%	0.123		IPR015880 (SMART); IPR013087 (G3DSA:3.30.160.GENE3D); IPR013087 (G3DSA:3.30.160.GENE3D); PF13465 (PFAM); IPR007087 (PFAM); PTHR24409 (PANTHER); IPR007087 (PROSITE_PATTERNS); IPR007087 (PROSITE_PATTERNS); IPR007087 (PROSITE_PATTERNS); IPR007087 (PROSITE_PATTERNS); IPR007087 (PROSITE_PROFILES); IPR007087 (PROSITE_PROFILES); IPR007087 (PROSITE_PROFILES); IPR007087 (PROSITE_PROFILES); SSF57667 (SUPERFAMILY); SSF57667 (SUPERFAMILY); SSF57667 (SUPERFAMILY)
412	320001_length_405_cvg_3.0_tip_1_2	4	lipophorin receptor	135	1.55E-51	79.00%	0.109		IPR000033 (SMART); IPR000033 (PFAM); IPR011042 (G3DSA:2.120.10.GENE3D); PTHR10529:SF208 (PANTHER); PTHR10529 (PANTHER); IPR000033 (PROSITE_PROFILES); IPR000033 (PROSITE_PROFILES); IPR000033 (PROSITE_PROFILES); SSF63825 (SUPERFAMILY)
413	320151_length_405_cvg_3.6_tip_1_5	3	neurogenic locus notch partial	135	7.02E-60	86.90%	0.112		PR00010 (PRINTS); IPR001881 (SMART); IPR000742 (SMART); G3DSA:2.10.25.10 (GENE3D); IPR000742 (PFAM); G3DSA:2.10.25.10 (GENE3D); G3DSA:2.10.25.10 (GENE3D); PTHR24033:SF36 (PANTHER); PTHR24033 (PANTHER); IPR013032 (PROSITE_PATTERNS); IPR013032 (PROSITE_PATTERNS); IPR000152 (PROSITE_PATTERNS); IPR018097 (PROSITE_PATTERNS); IPR018097 (PROSITE_PATTERNS); IPR000152 (PROSITE_PATTERNS); IPR018097 (PROSITE_PATTERNS); IPR013032 (PROSITE_PATTERNS); IPR013032 (PROSITE_PATTERNS); IPR000152 (PROSITE_PATTERNS); IPR000742 (PROSITE_PROFILES); IPR000742 (PROSITE_PROFILES); IPR000742 (PROSITE_PROFILES); IPR009030 (SUPERFAMILY)
414	320827_length_407_cvg_3.2_tip_1_1	5	b-cell lymphoma	136	4.45E-56	91.20%	0.113		IPR015880 (SMART); IPR013087 (G3DSA:3.30.160.GENE3D); IPR013087 (G3DSA:3.30.160.GENE3D); PF13465 (PFAM); IPR007087 (PFAM); PTHR23233:SF1 (PANTHER); PTHR23233 (PANTHER); IPR007087 (PROSITE_PATTERNS); IPR007087 (PROSITE_PATTERNS); IPR007087 (PROSITE_PROFILES); IPR007087 (PROSITE_PROFILES); SSF57667 (SUPERFAMILY); SSF57667 (SUPERFAMILY)
415	321041_length_408_cvg_3.0_tip_1_5	5	xanthine dehydrogenase	136	4.06E-35	68.60%	0.102		IPR016167 (G3DSA:3.30.43.GENE3D); IPR002346 (PFAM); PTHR11908 (PANTHER); PTHR11908:SF32 (PANTHER); IPR016166 (SUPERFAMILY)
417	322195_length_410_cvg_2.0_tip_1_4	3	pa domain-containing protein	137	2.06E-44	77.70%	0.102		G3DSA:3.50.30.30 (GENE3D); IPR003137 (PFAM); PTHR22702 (PANTHER)
418	323039_length_412_cvg_3.0_tip_1_0	3	dermatopontin 2	138	8.63E-18	58.20%	0.247		PF14704 (PFAM); IPR026645 (PANTHER); TRANSMEMBRANE (PHOBIUS); CYTOPLASMIC_DOMAIN (PHOBIUS); NON_CYTOPLASMIC_DOMAIN (PHOBIUS); TMhelix (TMHMM)
419	324163_length_415_cvg_2.0_tip_1_2	5	microtubule-associated serine threonine-protein kinase 3 isoform x1	138	1.18E-82	94.00%	0.131		IPR002290 (SMART); G3DSA:1.10.510.10 (GENE3D); IPR000719 (PFAM); PTHR24356 (PANTHER); PTHR24356:SF36 (PANTHER); NON_CYTOPLASMIC_DOMAIN (PHOBIUS); TRANSMEMBRANE (PHOBIUS); CYTOPLASMIC_DOMAIN (PHOBIUS); IPR000719 (PROSITE_PROFILES); IPR000961 (PROSITE_PROFILES); IPR011009 (SUPERFAMILY)
420	324440_length_416_cvg_3.9_tip_1_3	4	7-alpha-hydroxysteroid dehydrogenase	139	6.64E-96	100.00%	0.103		IPR002198 (PRINTS); IPR002347 (PRINTS); IPR002198 (PFAM); IPR016040 (G3DSA:3.40.50.GENE3D); PTHR24322:SF166 (PANTHER); PTHR24322 (PANTHER); IPR020904 (PROSITE_PATTERNS); SSF51735 (SUPERFAMILY)
421	325038_length_417_cvg_2.0_tip_1_2	3	gamma-glutamyltransferase	139	1.28E-93	99.70%	0.105		IPR000101 (PRINTS); IPR000101 (PFAM); IPR000101 (PANTHER); PTHR11686:SF9 (PANTHER); IPR029055 (SUPERFAMILY)
422	325140_length_418_cvg_2.6_tip_1_1	4	gastrula zinc finger isoform x1	139	3.93E-46	71.30%	0.106		IPR015880 (SMART); IPR013087 (G3DSA:3.30.160.GENE3D); PF13465 (PFAM); IPR013087 (G3DSA:3.30.160.GENE3D); IPR013087 (G3DSA:3.30.160.GENE3D); IPR013087 (G3DSA:3.30.160.GENE3D); IPR013087 (G3DSA:3.30.160.GENE3D); PTHR24409 (PANTHER); IPR007087 (PROSITE_PATTERNS); IPR007087 (PROSITE_PATTERNS); IPR007087 (PROSITE_PATTERNS); IPR007087 (PROSITE_PROFILES); IPR007087 (PROSITE_PROFILES); IPR007087 (PROSITE_PROFILES); IPR007087 (PROSITE_PROFILES); IPR007087 (PROSITE_PROFILES); SSF57667 (SUPERFAMILY); SSF57667 (SUPERFAMILY); SSF57667 (SUPERFAMILY)
423	325200_length_418_cvg_3.0_tip_1_5	4	astacin-like metalloprotease partial	139	2.73E-10	50.40%	0.849	Y	IPR001506 (PFAM); IPR024079 (G3DSA:3.40.390.GENE3D); SIGNAL_PEPTIDE_H_REGION (PHOBIUS); SIGNAL_PEPTIDE_C_REGION (PHOBIUS); SIGNAL_PEPTIDE (PHOBIUS); SIGNAL_PEPTIDE_N_REGION (PHOBIUS); NON_CYTOPLASMIC_DOMAIN (PHOBIUS); SignalP-noTM (SIGNALP_EUK); SSF55486 (SUPERFAMILY); TMhelix (TMHMM)
425	327388_length_423_cvg_4.0_tip_1_5	4	hypothetical protein X975_25329, partial	141	4.14E-36	63.00%	0.099		PTHR23022 (PANTHER); PTHR23022:SF53 (PANTHER)
427	327842_length_424_cvg_2.0_tip_1_1	1	thioredoxin	141	2.31E-93	99.80%	0.343	Y	PR00421 (PRINTS); IPR012336 (G3DSA:3.40.30.GENE3D); IPR005746 (TIGRFAM); IPR013766 (PFAM); PTHR10438:SF253 (PANTHER); IPR005746 (PANTHER); IPR017937 (PROSITE_PATTERNS); IPR012336 (PROSITE_PROFILES); IPR012336 (SUPERFAMILY)

428	327904_length_424_cvg_6.1_tip_1_2	4	cytochrome p450 partial	141	4.12E-46	69.20%	0.1	IPR001128 (PRINTS); IPR002401 (PRINTS); IPR001128 (G3DSA:1.10.630.GENE3D); IPR001128 (PFAM); PTHR24291 (PANTHER); IPR017972 (PROSITE_PATTERNS); IPR001128 (SUPERFAMILY)
426	327634_length_424_cvg_7.7_tip_1_0	11	PREDICTED: uncharacterized protein C15orf61-like	142	5.96E-41	77.60%	0.339	IPR029245 (PFAM)
429	328084_length_425_cvg_4.0_tip_1_4	1	serine protease inhibitor dipetalogastin precursor	142	9.12E-17	51.20%	0.102	IPR002350 (SMART); IPR002350 (PFAM); G3DSA:3.30.60.30 (GENE3D); PTHR10913 (PANTHER); PTHR10913:SF46 (PANTHER); IPR002350 (PROSITE_PROFILES); IPR002350 (PROSITE_PROFILES); IPR002350 (PROSITE_PROFILES); SSF100895 (SUPERFAMILY); SSF100895 (SUPERFAMILY)
430	328158_length_425_cvg_102.8_tip_0_0	92927	clotting factor partial	142	9.89E-16	70.40%	0.119	G3DSA:2.40.10.10 (GENE3D); IPR001254 (PFAM); PTHR24275 (PANTHER); IPR018114 (PROSITE_PATTERNS); IPR001254 (PROSITE_PROFILES); IPR009003 (SUPERFAMILY)
431	328524_length_426_cvg_2.0_tip_1_2	3	protein flightless-1	142	7.70E-67	87.60%	0.131	IPR003591 (SMART); IPR001611 (PFAM); G3DSA:3.80.10.10 (GENE3D); IPR001611 (PFAM); IPR007122 (PANTHER); IPR001611 (PROSITE_PROFILES); IPR001611 (PROSITE_PROFILES); IPR001611 (PROSITE_PROFILES); IPR001611 (PROSITE_PROFILES); IPR001611 (PROSITE_PROFILES); SSF52058 (SUPERFAMILY)
432	328884_length_426_cvg_2.0_tip_1_0	2	kelch-like ech-associated protein 1 isoform x2	142	5.79E-80	86.40%	0.102	IPR011705 (SMART); IPR011705 (PFAM); PTHR24412 (PANTHER); PTHR24412:SF1 (PANTHER)
437	329452_length_428_cvg_2.0_tip_1_2	2	low-density lipoprotein receptor-related protein 1b-like	142	1.54E-14	61.70%	0.269	IPR002172 (PRINTS); IPR002172 (SMART); IPR002172 (G3DSA:4.10.400.GENE3D); IPR002172 (G3DSA:4.10.400.GENE3D); IPR002172 (PFAM); PTHR10529 (PANTHER); IPR023415 (PROSITE_PATTERNS); IPR023415 (PROSITE_PATTERNS); CYTOPLASMIC_DOMAIN (PHOBIUS); NON_CYTOPLASMIC_DOMAIN (PHOBIUS); TRANSMEMBRANE (PHOBIUS); IPR002172 (PROSITE_PROFILES); IPR002172 (PROSITE_PROFILES); IPR002172 (SUPERFAMILY); IPR002172 (SUPERFAMILY)
433	329172_length_427_cvg_63.9_tip_0_0	105834	isoform a	143	3.13E-27	63.20%	0.111	IPR001254 (SMART); G3DSA:2.40.10.10 (GENE3D); IPR001254 (PFAM); PTHR24268 (PANTHER); IPR018114 (PROSITE_PATTERNS); IPR001254 (PROSITE_PROFILES); IPR009003 (SUPERFAMILY)
434	329294_length_428_cvg_2.0_tip_1_4	2	aminoacylase-1-like isoform 1	143	4.33E-67	80.10%	0.138	IPR002933 (PFAM); G3DSA:3.40.630.10 (GENE3D); PTHR11014:SF8 (PANTHER); PTHR11014 (PANTHER); IPR001261 (PROSITE_PATTERNS); IPR001261 (PROSITE_PATTERNS); SSF53187 (SUPERFAMILY)
435	329356_length_428_cvg_2.0_tip_1_1	2	allatostatin precursor protein	143	3.55E-16	51.20%	0.098	no IPS match
436	329414_length_428_cvg_78.6_tip_0_3	104728	suppressor of tumorigenicity 14 protein isoform x3	143	7.80E-16	46.50%	0.098	IPR002172 (SMART); IPR002172 (PFAM); IPR001254 (PFAM); G3DSA:2.40.10.10 (GENE3D); IPR002172 (G3DSA:4.10.400.GENE3D); G3DSA:2.40.10.10 (GENE3D); PTHR24256:SF96 (PANTHER); PTHR24256 (PANTHER); IPR023415 (PROSITE_PATTERNS); IPR018114 (PROSITE_PATTERNS); IPR002172 (PROSITE_PROFILES); IPR002172 (SUPERFAMILY); IPR009003 (SUPERFAMILY)
438	329842_length_429_cvg_58.0_tip_1_0	84	zinc finger protein	143	4.11E-20	58.50%	0.104	IPR015880 (SMART); PF13465 (PFAM); IPR013087 (G3DSA:3.30.160.GENE3D); IPR007087 (PFAM); IPR013087 (G3DSA:3.30.160.GENE3D); PTHR24409 (PANTHER); IPR007087 (PROSITE_PATTERNS); IPR007087 (PROSITE_PATTERNS); IPR007087 (PROSITE_PATTERNS); IPR007087 (PROSITE_PROFILES); IPR007087 (PROSITE_PROFILES); IPR007087 (PROSITE_PROFILES); SSF57667 (SUPERFAMILY); SSF57667 (SUPERFAMILY)
439	330362_length_430_cvg_2.0_tip_1_2	1	leucine-rich repeat and guanylate kinase domain-containing partial	143	2.25E-13	68.10%	0.104	PR00019 (PRINTS); SM00365 (SMART); G3DSA:3.80.10.10 (GENE3D); IPR001611 (PFAM); IPR025875 (PFAM); PTHR10588 (PANTHER); TRANSMEMBRANE (PHOBIUS); NON_CYTOPLASMIC_DOMAIN (PHOBIUS); CYTOPLASMIC_DOMAIN (PHOBIUS); IPR001611 (PROSITE_PROFILES); IPR001611 (PROSITE_PROFILES); IPR001611 (PROSITE_PROFILES); IPR001611 (PROSITE_PROFILES); SSF52058 (SUPERFAMILY)
440	330716_length_431_cvg_12.6_tip_1_5	15	sterile alpha motif domain-containing protein 13	143	2.79E-12	85.30%	0.125	IPR013761 (G3DSA:1.10.150.GENE3D); IPR021129 (PFAM); PTHR12247 (PANTHER); PTHR12247:SF66 (PANTHER); IPR013761 (SUPERFAMILY)
441	330878_length_432_cvg_2.0_tip_1_1	3	retinol dehydrogenase 11-like	144	7.43E-47	71.00%	0.315	IPR002347 (PRINTS); IPR002198 (PFAM); IPR016040 (G3DSA:3.40.50.GENE3D); PTHR24316 (PANTHER); PTHR24316:SF305 (PANTHER); TRANSMEMBRANE (PHOBIUS); NON_CYTOPLASMIC_DOMAIN (PHOBIUS); CYTOPLASMIC_DOMAIN (PHOBIUS); SSF51735 (SUPERFAMILY); TMhelix (TMHMM)
442	331358_length_433_cvg_5.4_tip_1_5	6	phenoloxidase subunit a3-like	144	1.16E-31	60.40%	0.122	IPR005203 (PFAM); IPR005203 (G3DSA:2.60.40.GENE3D); PTHR11511:SF24 (PANTHER); IPR013788 (PANTHER); IPR014756 (SUPERFAMILY)
443	331514_length_433_cvg_3.0_tip_1_5	4	venom metalloproteinase 3-like	144	2.31E-09	47.70%	0.106	IPR006586 (SMART)
444	332144_length_435_cvg_2.0_tip_1_1	3	laminin beta 1	145	3.88E-60	73.70%	0.125	IPR002049 (SMART); IPR002049 (PFAM); G3DSA:2.10.25.10 (GENE3D); PTHR10574 (PANTHER); PTHR10574:SF238 (PANTHER); IPR002049 (PROSITE_PROFILES); IPR013015 (PROSITE_PROFILES); SSF57196 (SUPERFAMILY)

445	332198_length_435_cvg_2.0_tip_1_1	3	attractin-like protein 1 isoform x1	145	1.25E-37	80.70%	0.121		PTHR10574 (PANTHER); PTHR10574:SF3 (PANTHER); TRANSMEMBRANE (PHOBIUS); NON_CYTOPLASMIC_DOMAIN (PHOBIUS); CYTOPLASMIC_DOMAIN (PHOBIUS); CYTOPLASMIC_DOMAIN (PHOBIUS); TRANSMEMBRANE (PHOBIUS); TMhelix (TMHMM)
446	332482_length_436_cvg_7.1_tip_1_5	7	cysteine string isoform x1	145	5.38E-71	94.60%	0.162		IPR001623 (PRINTS); IPR001623 (SMART); IPR001623 (PFAM); IPR001623 (G3DSA:1.10.287.GENE3D); PTHR24078:SF5 (PANTHER); PTHR24078 (PANTHER); IPR018253 (PROSITE_PATTERNS); IPR001623 (PROSITE_PROFILES); IPR001623 (SUPERFAMILY)
447	332770_length_436_cvg_37.2_tip_1_1	21	low-density lipoprotein receptor-related protein 2	145	5.80E-13	67.00%	0.1		IPR002172 (SMART); IPR002172 (G3DSA:4.10.400.GENE3D); IPR002172 (PFAM); G3DSA:4.10.1220.10 (GENE3D); PTHR10529 (PANTHER); IPR023415 (PROSITE_PATTERNS); IPR002172 (PROSITE_PROFILES); IPR002172 (SUPERFAMILY)
448	333228_length_438_cvg_2.0_tip_1_5	3	cyclin dependent kinase 8	146	6.47E-95	97.30%	0.102		IPR002290 (SMART); IPR000719 (PFAM); G3DSA:1.10.510.10 (GENE3D); G3DSA:3.30.200.20 (GENE3D); PTHR24056 (PANTHER); PTHR24056:SF162 (PANTHER); IPR008271 (PROSITE_PATTERNS); IPR000719 (PROSITE_PROFILES); IPR011009 (SUPERFAMILY)
449	333868_length_439_cvg_2.9_tip_1_2	4	ejaculatory bulb-specific protein 3	146	7.83E-37	70.00%	0.554	Y	IPR005055 (G3DSA:1.10.2080.GENE3D); IPR005055 (PFAM); PTHR11257:SF4 (PANTHER); IPR005055 (PANTHER); CYTOPLASMIC_DOMAIN (PHOBIUS); NON_CYTOPLASMIC_DOMAIN (PHOBIUS); TRANSMEMBRANE (PHOBIUS); SignalP-TM (SIGNALP_EUK); IPR005055 (SUPERFAMILY); TMhelix (TMHMM)
465	338474_length_450_cvg_3.0_tip_1_2	4	prostatic acid phosphatase-like	146	6.38E-13	56.00%	0.097		IPR029033 (G3DSA:3.40.50.GENE3D); PTHR11567:SF26 (PANTHER); PTHR11567 (PANTHER); CYTOPLASMIC_DOMAIN (PHOBIUS); NON_CYTOPLASMIC_DOMAIN (PHOBIUS); TRANSMEMBRANE (PHOBIUS); IPR029033 (SUPERFAMILY); TMhelix (TMHMM)
450	334092_length_440_cvg_9.7_tip_1_1	10	er protein gp78	147	7.95E-70	89.50%	0.544	Y	IPR013126 (PRINTS); G3DSA:3.30.420.40 (GENE3D); IPR013126 (PFAM); G3DSA:3.30.30.30 (GENE3D); PTHR19375:SF157 (PANTHER); PTHR19375 (PANTHER); IPR018181 (PROSITE_PATTERNS); SIGNAL_PEPTIDE_N_REGION (PHOBIUS); SIGNAL_PEPTIDE (PHOBIUS); NON_CYTOPLASMIC_DOMAIN (PHOBIUS); SIGNAL_PEPTIDE_H_REGION (PHOBIUS); SIGNAL_PEPTIDE_C_REGION (PHOBIUS); SignalP-noTM (SIGNALP_EUK); SSF53067 (SUPERFAMILY)
451	334888_length_442_cvg_3.0_tip_1_4	6	gram negative bacteria binding protein 1	147	7.17E-43	67.10%	0.102		IPR000757 (PFAM); IPR013320 (G3DSA:2.60.120.GENE3D); PTHR10963 (PANTHER); PTHR10963:SF4 (PANTHER); IPR013320 (SUPERFAMILY)
452	335456_length_443_cvg_2.0_tip_1_5	4	furin-like protease isoform 1-crr isoform x2	147	3.27E-13	46.29%	0.127		G3DSA:2.10.220.10 (GENE3D); IPR009030 (SUPERFAMILY)
517	352819_length_487_cvg_61.3_tip_1_1	756	ribosomal protein s26	147	2.02E-59	89.70%	0.106		IPR000892 (PFAM); IPR000892 (PANTHER); IPR000892 (PROSITE_PATTERNS)
453	335742_length_444_cvg_112.5_tip_0_2	146869	low-density lipoprotein receptor-related protein 2	148	1.36E-19	63.90%	0.492	Y	IPR002172 (PRINTS); IPR002172 (SMART); IPR002172 (PFAM); IPR002172 (G3DSA:4.10.400.GENE3D); IPR002172 (G3DSA:4.10.400.GENE3D); PTHR10529 (PANTHER); IPR023415 (PROSITE_PATTERNS); IPR023415 (PROSITE_PATTERNS); SIGNAL_PEPTIDE_N_REGION (PHOBIUS); SIGNAL_PEPTIDE (PHOBIUS); SIGNAL_PEPTIDE_H_REGION (PHOBIUS); SIGNAL_PEPTIDE_C_REGION (PHOBIUS); NON_CYTOPLASMIC_DOMAIN (PHOBIUS); IPR002172 (PROSITE_PROFILES); IPR002172 (PROSITE_PROFILES); IPR002172 (SUPERFAMILY); IPR002172 (SUPERFAMILY)
454	335776_length_444_cvg_2.3_tip_1_1	3	#NAME?	148	1.29E-52	81.50%	0.137		G3DSA:3.40.50.2000 (GENE3D); IPR001830 (PFAM); PTHR10788:SF6 (PANTHER); PTHR10788 (PANTHER); SSF53756 (SUPERFAMILY)
455	336054_length_445_cvg_2.6_tip_1_5	4	gels_homam ame: full= cytoplasmic ame: full=actin-depolymerizing factor short=adf	148	1.67E-40	78.30%	0.2		IPR007122 (SMART); IPR007123 (PFAM); IPR029006 (G3DSA:3.40.20.GENE3D); IPR007122 (PANTHER); SSF55753 (SUPERFAMILY)
456	336572_length_446_cvg_63.0_tip_0_2	178	vitellogenin receptor	148	3.10E-17	52.00%	0.127		IPR002172 (PRINTS); IPR002172 (SMART); IPR002172 (G3DSA:4.10.400.GENE3D); IPR002172 (PFAM); IPR002172 (G3DSA:4.10.400.GENE3D); PTHR10529 (PANTHER); IPR023415 (PROSITE_PATTERNS); IPR002172 (PROSITE_PROFILES); IPR002172 (PROSITE_PROFILES); IPR002172 (SUPERFAMILY); IPR002172 (SUPERFAMILY); IPR002172 (SUPERFAMILY)
457	336872_length_447_cvg_2.0_tip_1_1	3	protein lap4 isoform x4	149	8.02E-82	92.90%	0.103		IPR003591 (SMART); G3DSA:3.80.10.10 (GENE3D); IPR025875 (PFAM); IPR001611 (PFAM); G3DSA:3.80.10.10 (GENE3D); PTHR23155 (PANTHER); PTHR23155:SF31 (PANTHER); IPR001611 (PROSITE_PROFILES); IPR001611 (PROSITE_PROFILES); IPR001611 (PROSITE_PROFILES); IPR001611 (PROSITE_PROFILES); IPR001611 (PROSITE_PROFILES); SSF52058 (SUPERFAMILY)
458	337166_length_447_cvg_7.7_tip_1_1	9	serine protease nudel	149	2.27E-20	55.10%	0.129		IPR002172 (PRINTS); IPR002172 (SMART); IPR002172 (G3DSA:4.10.400.GENE3D); IPR002172 (PFAM); IPR002172 (G3DSA:4.10.400.GENE3D); PTHR10529:SF205 (PANTHER); PTHR10529 (PANTHER); IPR023415 (PROSITE_PATTERNS); IPR023415 (PROSITE_PATTERNS); IPR002172 (PROSITE_PROFILES); IPR002172 (PROSITE_PROFILES); IPR002172 (SUPERFAMILY); IPR002172 (SUPERFAMILY)

459	337188_length_447_cvg_2.0_tip_1_5	4	retinol dehydrogenase 13-like	149	1.02E-44	75.10%	0.243		IPR016040 (G3DSA:3.40.50.GENE3D); IPR002198 (PFAM); PTHR24316:SF305 (PANTHER); PTHR24316 (PANTHER); NON_CYTOPLASMIC_DOMAIN (PHOBIUS); TRANSMEMBRANE (PHOBIUS); CYTOPLASMIC_DOMAIN (PHOBIUS); SSF51735 (SUPERFAMILY); TMhelix (TMHMM)
460	337236_length_447_cvg_7.3_tip_0_1	4	low quality protein: otogelin	149	8.75E-08	49.00%	0.104		no IPS match
461	337516_length_448_cvg_8.0_tip_1_1	3	dehydrogenase reductase sdr family member 11-like	149	4.17E-14	86.50%	0.224		IPR016040 (G3DSA:3.40.50.GENE3D); PTHR24322 (PANTHER); PTHR24322:SF310 (PANTHER); SSF51735 (SUPERFAMILY)
462	337528_length_448_cvg_2.0_tip_1_4	4	basement membrane-specific heparan sulfate proteoglycan core protein	149	1.34E-16	64.00%	0.14		IPR000742 (PFAM); G3DSA:2.10.25.10 (GENE3D); TRANSMEMBRANE (PHOBIUS); CYTOPLASMIC_DOMAIN (PHOBIUS); NON_CYTOPLASMIC_DOMAIN (PHOBIUS); IPR000742 (PROSITE_PROFILES); SSF57196 (SUPERFAMILY); TMhelix (TMHMM)
463	337658_length_448_cvg_89.7_tip_0_5	18805	low quality protein: probable chitinase 3	149	2.04E-35	71.30%	0.579	Y	IPR001223 (PFAM); IPR013781 (G3DSA:3.20.20.GENE3D); PTHR11177:SF112 (PANTHER); PTHR11177 (PANTHER); NON_CYTOPLASMIC_DOMAIN (PHOBIUS); SIGNAL_PEPTIDE_H_REGION (PHOBIUS); SIGNAL_PEPTIDE_C_REGION (PHOBIUS); SIGNAL_PEPTIDE_N_REGION (PHOBIUS); SIGNAL_PEPTIDE (PHOBIUS); SignalP-TM (SIGNALP_GRAM_POSITIVE); SignalP-TM (SIGNALP_EUK); IPR017853 (SUPERFAMILY); TMhelix (TMHMM)
464	338374_length_450_cvg_2.0_tip_1_0	2	calnexin isoform x2	150	1.35E-69	83.90%	0.106		IPR001580 (PRINTS); IPR009033 (G3DSA:2.10.250.GENE3D); IPR001580 (PFAM); IPR001580 (PANTHER); IPR018124 (PROSITE_PATTERNS); IPR018124 (PROSITE_PATTERNS); IPR018124 (PROSITE_PATTERNS); IPR009033 (SUPERFAMILY)
466	338802_length_451_cvg_2.0_tip_1_4	3	lachesin	150	1.12E-50	73.20%	0.104		IPR003599 (SMART); IPR013783 (G3DSA:2.60.40.GENE3D); IPR013106 (PFAM); IPR013783 (G3DSA:2.60.40.GENE3D); PTHR19831 (PANTHER); PTHR19831:SF43 (PANTHER); IPR007110 (PROSITE_PROFILES); SSF48726 (SUPERFAMILY); SSF48726 (SUPERFAMILY)
468	338966_length_452_cvg_4.9_tip_1_5	5	alcohol dehydrogenase	150	3.60E-31	74.80%	0.106		IPR023210 (PFAM); IPR023210 (G3DSA:3.20.20.GENE3D); IPR001395 (PANTHER); PTHR11732:SF183 (PANTHER); IPR018170 (PROSITE_PATTERNS); IPR023210 (SUPERFAMILY)
469	339140_length_452_cvg_82.0_tip_0_5	658959	probable chitinase 3	150	3.25E-32	69.20%	0.317		IPR013781 (G3DSA:3.20.20.GENE3D); IPR001223 (PFAM); PTHR11177:SF112 (PANTHER); PTHR11177 (PANTHER); SIGNAL_PEPTIDE_N_REGION (PHOBIUS); SIGNAL_PEPTIDE (PHOBIUS); SIGNAL_PEPTIDE_H_REGION (PHOBIUS); SIGNAL_PEPTIDE_C_REGION (PHOBIUS); NON_CYTOPLASMIC_DOMAIN (PHOBIUS); IPR017853 (SUPERFAMILY); TMhelix (TMHMM)
507	349207_length_478_cvg_3.0_tip_1_1	4	serine protease 52-like	150	2.03E-12	58.70%	0.212		G3DSA:2.40.10.10 (GENE3D); IPR001254 (PFAM); PTHR24260 (PANTHER); IPR018114 (PROSITE_PATTERNS); IPR001254 (PROSITE_PROFILES); IPR009003 (SUPERFAMILY)
467	338874_length_452_cvg_61.8_tip_1_0	43	agap012894-pa-like protein	151	5.72E-48	95.80%	0.113		IPR000558 (PRINTS); IPR000558 (SMART); IPR009072 (G3DSA:1.10.20.GENE3D); IPR007125 (PFAM); IPR000558 (PANTHER); IPR000558 (PROSITE_PATTERNS); IPR009072 (SUPERFAMILY)
470	339174_length_452_cvg_3.1_tip_1_1	4	14-3-3 protein	151	8.81E-31	90.20%	0.104		Coil (COILS); IPR000308 (PRINTS); G3DSA:1.20.190.20 (GENE3D); IPR023410 (PFAM); IPR000308 (PANTHER); TRANSMEMBRANE (PHOBIUS); NON_CYTOPLASMIC_DOMAIN (PHOBIUS); CYTOPLASMIC_DOMAIN (PHOBIUS); IPR023410 (SUPERFAMILY); TMhelix (TMHMM); TMhelix (TMHMM)
471	339250_length_453_cvg_3.2_tip_1_4	4	mite allergen der f 3-like	151	6.67E-08	47.60%	0.14		IPR001254 (PFAM); G3DSA:2.40.10.10 (GENE3D); PTHR24276 (PANTHER); IPR018114 (PROSITE_PATTERNS); IPR009003 (SUPERFAMILY)
472	339364_length_453_cvg_76.4_tip_1_3	67906	proclotting enzyme-like	151	2.07E-16	62.80%	0.116		G3DSA:2.40.10.10 (GENE3D); IPR001254 (PFAM); PTHR24275 (PANTHER); IPR018114 (PROSITE_PATTERNS); IPR001254 (PROSITE_PROFILES); IPR009003 (SUPERFAMILY)
473	339382_length_453_cvg_2.0_tip_1_1	4	3-hydroxy acid dehydrogenase	151	4.96E-40	99.50%	0.162		IPR002198 (PFAM); IPR016040 (G3DSA:3.40.50.GENE3D); PTHR24322 (PANTHER); SSF51735 (SUPERFAMILY)
474	339588_length_453_cvg_82.6_tip_1_3	34753	proclotting enzyme-like	151	2.01E-17	64.80%	0.119		G3DSA:2.40.10.10 (GENE3D); IPR001254 (PFAM); PTHR24265 (PANTHER); IPR018114 (PROSITE_PATTERNS); IPR001254 (PROSITE_PROFILES); IPR009003 (SUPERFAMILY)
475	339762_length_454_cvg_3.5_tip_1_1	2	maguk p55 subfamily member 6 isoform x1	151	6.31E-21	86.20%	0.269		IPR004172 (SMART); IPR014775 (PFAM); IPR004172 (PROSITE_PROFILES)
476	340068_length_455_cvg_5.5_tip_1_2	11	transcription factor e4f1-like	151	1.32E-36	60.30%	0.104		IPR015880 (SMART); PF13912 (PFAM); IPR013087 (G3DSA:3.30.160.GENE3D); PF13465 (PFAM); IPR013087 (G3DSA:3.30.160.GENE3D); PTHR24409 (PANTHER); IPR007087 (PROSITE_PATTERNS); IPR007087 (PROSITE_PATTERNS); IPR007087 (PROSITE_PROFILES); IPR007087 (PROSITE_PROFILES); IPR007087 (PROSITE_PROFILES); IPR007087 (PROSITE_PROFILES); SSF57667 (SUPERFAMILY); SSF57667 (SUPERFAMILY)

477	340320_length_455_cvg_5.0_tip_1_0	6	low quality protein: dnaj homolog subfamily c member 10-like	152	4.04E-17	70.40%	0.162	IPR013766 (PFAM); IPR012336 (G3DSA:3.40.30.GENE3D); PTHR24078:SF3 (PANTHER); PTHR24078 (PANTHER); IPR017937 (PROSITE_PATTERNS); CYTOPLASMIC_DOMAIN (PHOBIUS); TRANSMEMBRANE (PHOBIUS); NON_CYTOPLASMIC_DOMAIN (PHOBIUS); IPR012336 (SUPERFAMILY); TMhelix (TMHMM)
478	340492_length_456_cvg_2.0_tip_1_3	2	multiple epidermal growth factor-like domains protein 8	152	4.96E-33	66.70%	0.121	PTHR10574 (PANTHER); PTHR10574:SF3 (PANTHER); CYTOPLASMIC_DOMAIN (PHOBIUS); TRANSMEMBRANE (PHOBIUS); NON_CYTOPLASMIC_DOMAIN (PHOBIUS); TMhelix (TMHMM); TMhelix (TMHMM)
479	340650_length_456_cvg_4.3_tip_1_5	6	down syndrome cell adhesion molecule-like protein dscam2 isoform x5	152	2.38E-59	68.80%	0.11	IPR013783 (G3DSA:2.60.40.GENE3D); IPR013783 (G3DSA:2.60.40.GENE3D); PF13895 (PFAM); PTHR10489:SF567 (PANTHER); PTHR10489 (PANTHER); IPR007110 (PROSITE_PROFILES); IPR007110 (PROSITE_PROFILES); SSF48726 (SUPERFAMILY); SSF48726 (SUPERFAMILY)
568	368844_length_532_cvg_4.3_tip_1_5	6	zinc finger protein 502	152	1.29E-32	60.20%	0.1	IPR015880 (SMART); IPR013087 (G3DSA:3.30.160.GENE3D); IPR007087 (PFAM); IPR013087 (G3DSA:3.30.160.GENE3D); PF13465 (PFAM); IPR013087 (G3DSA:3.30.160.GENE3D); PTHR24409 (PANTHER); PTHR24409:SF13 (PANTHER); IPR007087 (PROSITE_PATTERNS); IPR007087 (PROSITE_PATTERNS); IPR007087 (PROSITE_PATTERNS); IPR007087 (PROSITE_PATTERNS); IPR007087 (PROSITE_PROFILES); IPR007087 (PROSITE_PROFILES); IPR007087 (PROSITE_PROFILES); IPR007087 (PROSITE_PROFILES); IPR007087 (PROSITE_PROFILES); SSF57667 (SUPERFAMILY); SSF57667 (SUPERFAMILY); SSF57667 (SUPERFAMILY)
480	341202_length_457_cvg_2.0_tip_1_3	3	serine threonine-protein phosphatase 6 regulatory ankyrin repeat subunit a- partial	153	1.40E-36	89.10%	0.105	IPR002110 (SMART); IPR020683 (G3DSA:1.25.40.GENE3D); IPR020683 (PFAM); PTHR24158:SF22 (PANTHER); PTHR24158 (PANTHER); IPR002110 (PROSITE_PROFILES); IPR020683 (PROSITE_PROFILES); IPR002110 (PROSITE_PROFILES); IPR020683 (SUPERFAMILY)
481	341662_length_459_cvg_2.0_tip_1_4	2	peripheral plasma membrane protein cask isoform x2	153	3.17E-93	94.60%	0.102	IPR001452 (SMART); G3DSA:2.30.30.40 (GENE3D); IPR011511 (PFAM); PTHR23122:SF7 (PANTHER); PTHR23122 (PANTHER); IPR001452 (PROSITE_PROFILES); IPR001452 (SUPERFAMILY)
482	341882_length_459_cvg_62.6_tip_1_4	422	selenium-dependent glutathione peroxidase	153	2.12E-57	78.40%	0.115	IPR000889 (PRINTS); IPR000889 (PIRSF); IPR012336 (G3DSA:3.40.30.GENE3D); IPR000889 (PFAM); PTHR11592:SF21 (PANTHER); IPR000889 (PANTHER); IPR000889 (PROSITE_PROFILES); IPR012336 (SUPERFAMILY)
483	342038_length_460_cvg_5.3_tip_1_3	10	uncharacterized aarf domain-containing protein kinase 1	154	9.00E-26	70.40%	0.135	PTHR10566:SF58 (PANTHER); PTHR10566 (PANTHER)
484	342814_length_462_cvg_2.0_tip_1_1	3	apical endosomal glycoprotein	154	2.44E-19	68.60%	0.11	IPR000998 (PFAM); PTHR23282 (PANTHER); IPR000998 (PROSITE_PROFILES); IPR013320 (SUPERFAMILY)
485	342928_length_462_cvg_3.0_tip_1_4	4	hypothetical protein MDA_GLEAN10014894	154	7.37E-08	45.75%	0.12	no IPS match
486	343018_length_462_cvg_2.5_tip_1_3	3	PREDICTED: hypothetical protein LOC100647042	154	9.20E-18	52.70%	0.233	IPR001314 (PRINTS); IPR001254 (SMART); G3DSA:2.40.10.10 (GENE3D); G3DSA:2.40.10.10 (GENE3D); IPR001254 (PFAM); PTHR24258:SF96 (PANTHER); PTHR24258 (PANTHER); IPR018114 (PROSITE_PATTERNS); IPR001254 (PROSITE_PROFILES); IPR009003 (SUPERFAMILY)
487	343032_length_462_cvg_2.1_tip_1_4	4	titin isoform x3	154	1.56E-31	63.60%	0.1	IPR003599 (SMART); IPR003598 (SMART); IPR013098 (PFAM); IPR013783 (G3DSA:2.60.40.GENE3D); PTHR19897 (PANTHER); IPR007110 (PROSITE_PROFILES); SSF48726 (SUPERFAMILY)
488	343066_length_462_cvg_106.9_tip_1_0	30526	chondroitin proteoglycan partial	154	7.25E-15	58.80%	0.1	IPR002557 (SMART); IPR002557 (G3DSA:2.170.140.GENE3D); IPR002557 (PFAM); PTHR23301 (PANTHER); IPR002557 (PROSITE_PROFILES); IPR002557 (SUPERFAMILY)
489	343076_length_462_cvg_4.8_tip_1_0	5	zinc finger protein	154	8.60E-29	55.60%	0.11	IPR015880 (SMART); PF13894 (PFAM); PF13465 (PFAM); IPR013087 (G3DSA:3.30.160.GENE3D); IPR013087 (G3DSA:3.30.160.GENE3D); IPR013087 (G3DSA:3.30.160.GENE3D); PTHR24409:SF14 (PANTHER); PTHR24409 (PANTHER); IPR007087 (PROSITE_PATTERNS); IPR007087 (PROSITE_PATTERNS); IPR007087 (PROSITE_PATTERNS); IPR007087 (PROSITE_PATTERNS); IPR007087 (PROSITE_PROFILES); IPR007087 (PROSITE_PROFILES); IPR007087 (PROSITE_PROFILES); IPR007087 (PROSITE_PROFILES); SSF57667 (SUPERFAMILY); SSF57667 (SUPERFAMILY)
490	343138_length_462_cvg_2.9_tip_1_0	6	lipase 3-like	154	3.20E-45	72.00%	0.183	IPR029058 (G3DSA:3.40.50.GENE3D); PTHR11005 (PANTHER); PTHR11005:SF6 (PANTHER); IPR029058 (SUPERFAMILY)
491	343498_length_463_cvg_63.7_tip_0_5	1043398	chitinase partial	154	2.74E-40	63.00%	0.101	IPR011583 (SMART); IPR013781 (G3DSA:3.20.20.GENE3D); IPR001223 (PFAM); PTHR11177 (PANTHER); PTHR11177:SF112 (PANTHER); IPR001579 (PROSITE_PATTERNS); IPR017853 (SUPERFAMILY)
516	351721_length_484_cvg_2.0_tip_1_4	5	leucine-rich repeat protein soc-2 isoform x1	154	8.82E-64	82.50%	0.119	G3DSA:3.80.10.10 (GENE3D); IPR001611 (PFAM); PTHR16083 (PANTHER); PTHR16083:SF2 (PANTHER); SSF52075 (SUPERFAMILY)
492	343990_length_464_cvg_63.0_tip_0_4	401	uncharacterized oxidoreductase	155	8.03E-28	72.30%	0.133	IPR016040 (G3DSA:3.40.50.GENE3D); PTHR24322:SF237 (PANTHER); PTHR24322 (PANTHER); SSF51735 (SUPERFAMILY)

512	350945_length_482_cvg_2.6_tip_1_2	5	odorant-binding protein 4	155	1.25E-44	72.00%	0.101		IPR006170 (PRINTS); IPR006170 (SMART); IPR023316 (G3DSA:1.10.238.GENE3D); IPR006170 (PFAM); PTHR11857 (PANTHER); PTHR11857:SF4 (PANTHER); CYTOPLASMIC_DOMAIN (PHOBIUS); TRANSMEMBRANE (PHOBIUS); NON_CYTOPLASMIC_DOMAIN (PHOBIUS); TRANSMEMBRANE (PHOBIUS); CYTOPLASMIC_DOMAIN (PHOBIUS); IPR023316 (SUPERFAMILY); TMhelix (TMHMM); TMhelix (TMHMM)
493	344822_length_467_cvg_4.0_tip_1_4	6	peptidase partial	156	6.71E-06	70.50%	0.241		G3DSA:2.40.10.10 (GENE3D); IPR009003 (SUPERFAMILY)
494	344840_length_467_cvg_9.1_tip_1_0	16	ankyrin repeat domain-containing protein 32-like	156	2.25E-25	57.50%	0.162		IPR002110 (SMART); IPR020683 (PFAM); IPR002110 (PFAM); IPR020683 (G3DSA:1.25.40.GENE3D); PTHR24171 (PANTHER); IPR020683 (PROSITE_PROFILES); IPR002110 (PROSITE_PROFILES); IPR020683 (SUPERFAMILY)
495	345792_length_469_cvg_2.4_tip_1_3	7	f379 retina specific protein	157	1.12E-08	70.00%	0.187		PR02045 (PRINTS); PTHR12138 (PANTHER)
496	345870_length_469_cvg_3.0_tip_1_3	7	dna-specific endonuclease partial	157	2.68E-48	99.80%	0.132		IPR007346 (PFAM); SSF54060 (SUPERFAMILY)
497	346016_length_470_cvg_90.8_tip_0_0	126651	low-density lipoprotein receptor-related protein 2	157	3.19E-32	61.10%	0.1		IPR002172 (PRINTS); IPR002172 (SMART); IPR002172 (PFAM); IPR002172 (G3DSA:4.10.400.GENE3D); IPR002172 (G3DSA:4.10.400.GENE3D); IPR002172 (G3DSA:4.10.400.GENE3D); G3DSA:4.10.1220.10 (GENE3D); PTHR10529 (PANTHER); IPR023415 (PROSITE_PATTERNS); IPR023415 (PROSITE_PATTERNS); IPR023415 (PROSITE_PATTERNS); IPR023415 (PROSITE_PATTERNS); IPR002172 (PROSITE_PROFILES); IPR002172 (PROSITE_PROFILES); IPR002172 (PROSITE_PROFILES); IPR002172 (PROSITE_PROFILES); IPR002172 (PROSITE_PROFILES); IPR002172 (SUPERFAMILY); IPR002172 (SUPERFAMILY); IPR002172 (SUPERFAMILY); IPR002172 (SUPERFAMILY)
498	346482_length_471_cvg_82.5_tip_1_3	1404	transmembrane protease serine 9-like	157	2.31E-27	58.50%	0.113		IPR001254 (SMART); IPR001254 (PFAM); G3DSA:2.40.10.10 (GENE3D); PTHR24260 (PANTHER); IPR018114 (PROSITE_PATTERNS); IPR001254 (PROSITE_PROFILES); IPR009003 (SUPERFAMILY)
499	346786_length_472_cvg_3.7_tip_1_3	5	upf0669 protein c6orf120 homolog	158	3.01E-23	63.00%	0.494	Y	PTHR31703 (PANTHER); CYTOPLASMIC_DOMAIN (PHOBIUS); NON_CYTOPLASMIC_DOMAIN (PHOBIUS); TRANSMEMBRANE (PHOBIUS); SignalP-noTM (SIGNALP_EUK); TMhelix (TMHMM)
500	347108_length_473_cvg_2.0_tip_1_0	4	acid sphingomyelinase-like phosphodiesterase partial	158	3.36E-14	54.60%	0.101		PTHR10340 (PANTHER)
501	347640_length_474_cvg_2.0_tip_1_3	3	serine protease snake	158	1.53E-39	58.20%	0.149		IPR001314 (PRINTS); IPR001254 (SMART); G3DSA:2.40.10.10 (GENE3D); G3DSA:2.40.10.10 (GENE3D); IPR001254 (PFAM); PTHR24260 (PANTHER); IPR018114 (PROSITE_PATTERNS); IPR001254 (PROSITE_PROFILES); IPR009003 (SUPERFAMILY)
502	347978_length_475_cvg_65.4_tip_1_4	108	peptidyl-prolyl cis-trans isomerase-like 1	158	1.12E-21	91.90%	0.115		IPR029000 (G3DSA:2.40.100.GENE3D); IPR002130 (PFAM); IPR024936 (PANTHER); IPR002130 (PROSITE_PROFILES); IPR029000 (SUPERFAMILY)
503	348527_length_476_cvg_4.0_tip_1_5	4	upf0364 protein c6orf211 homolog	158	4.43E-28	60.30%	0.103		IPR002791 (PFAM); PTHR12260:SF1 (PANTHER); PTHR12260 (PANTHER); IPR002791 (SUPERFAMILY)
504	348861_length_477_cvg_51.0_tip_1_2	87	bursicon alpha	159	4.24E-61	85.80%	0.607	Y	IPR004133 (PFAM); PTHR11339 (PANTHER); SIGNAL_PEPTIDE (PHOBIUS); SIGNAL_PEPTIDE_N_REGION (PHOBIUS); NON_CYTOPLASMIC_DOMAIN (PHOBIUS); SIGNAL_PEPTIDE_H_REGION (PHOBIUS); SIGNAL_PEPTIDE_C_REGION (PHOBIUS); IPR006207 (PROSITE_PROFILES); SignalP-noTM (SIGNALP_EUK)
505	348889_length_477_cvg_4.0_tip_1_3	5	calcium calmodulin-dependent protein kinase	159	6.57E-94	92.10%	0.101		IPR008145 (SMART); IPR027417 (G3DSA:3.40.50.GENE3D); G3DSA:3.30.63.10 (GENE3D); IPR008145 (PFAM); PTHR23122:SF7 (PANTHER); PTHR23122 (PANTHER); IPR020590 (PROSITE_PATTERNS); IPR008144 (PROSITE_PROFILES); IPR027417 (SUPERFAMILY)
506	348917_length_477_cvg_5.2_tip_1_5	7	glutamine-rich protein 2	159	1.62E-10	48.00%	0.197		SignalP-TM (SIGNALP_GRAM_POSITIVE)
508	349643_length_479_cvg_3.4_tip_1_2	5	zinc finger protein 271 (zinc finger protein 7) (zinc finger protein znfphex133) (epstein-barr virus-induced zinc finger protein) (znf-eb) (ct-zfp48) (zinc finger protein	159	2.82E-23	59.50%	0.097		IPR015880 (SMART); IPR013087 (G3DSA:3.30.160.GENE3D); PF13465 (PFAM); IPR003656 (PFAM); IPR013087 (G3DSA:3.30.160.GENE3D); PTHR24409 (PANTHER); PTHR24409:SF14 (PANTHER); IPR007087 (PROSITE_PATTERNS); IPR007087 (PROSITE_PATTERNS); IPR007087 (PROSITE_PATTERNS); IPR007087 (PROSITE_PATTERNS); IPR007087 (PROSITE_PROFILES); IPR007087 (PROSITE_PROFILES); IPR007087 (PROSITE_PROFILES); IPR007087 (PROSITE_PROFILES); SSF57667 (SUPERFAMILY); SSF57667 (SUPERFAMILY)
509	350293_length_481_cvg_4.1_tip_1_1	6	adam 17-like protease	160	1.60E-56	73.80%	0.663	Y	IPR002870 (PFAM); PTHR11905 (PANTHER); PTHR11905:SF121 (PANTHER); SIGNAL_PEPTIDE_C_REGION (PHOBIUS); SIGNAL_PEPTIDE (PHOBIUS); NON_CYTOPLASMIC_DOMAIN (PHOBIUS); SIGNAL_PEPTIDE_N_REGION (PHOBIUS); SIGNAL_PEPTIDE_H_REGION (PHOBIUS); SignalP-noTM (SIGNALP_EUK)
510	350301_length_481_cvg_45.9_tip_1_2	84	maguk p55 subfamily member 6 isoform x1	160	9.46E-21	88.00%	0.099		IPR027417 (G3DSA:3.40.50.GENE3D); PTHR23122 (PANTHER); IPR008144 (PROSITE_PROFILES); IPR027417 (SUPERFAMILY)
511	350393_length_481_cvg_2.0_tip_1_4	3	transcriptional regulator partial	160	7.47E-112	99.90%	0.188		no IPS match

513	351333_length_483_cvg_3.0_tip_0_4	2	heat shock 70 kda protein 14	161	2.46E-38	65.30%	0.128		IPR013126 (PRINTS); G3DSA:3.30.30.30 (GENE3D); IPR013126 (PFAM); G3DSA:3.30.420.40 (GENE3D); PTHR19375:SF158 (PANTHER); PTHR19375 (PANTHER); SSF53067 (SUPERFAMILY)
514	351351_length_483_cvg_2.7_tip_1_5	5	peptidyl-prolyl cis-trans isomerase a	161	1.20E-81	100.00%	0.109		IPR029000 (G3DSA:2.40.100.GENE3D); IPR002130 (PFAM); PTHR11071:SF195 (PANTHER); IPR024936 (PANTHER); IPR002130 (PROSITE_PROFILES); IPR029000 (SUPERFAMILY)
515	351653_length_484_cvg_67.9_tip_1_0	1559	chitinase 2	162	5.57E-42	74.40%	0.336		IPR001223 (PFAM); IPR013781 (G3DSA:3.20.20.GENE3D); PTHR11177 (PANTHER); PTHR11177:SF112 (PANTHER); SIGNAL_PEPTIDE_H_REGION (PHOBIUS); SIGNAL_PEPTIDE_N_REGION (PHOBIUS); SIGNAL_PEPTIDE_C_REGION (PHOBIUS); NON_CYTOPLASMIC_DOMAIN (PHOBIUS); SIGNAL_PEPTIDE (PHOBIUS); IPR017853 (SUPERFAMILY); TMhelix (TMHMM)
518	352853_length_487_cvg_4.0_tip_1_4	10	tyrosine-protein kinase transmembrane receptor ror2	162	7.78E-67	92.10%	0.115		IPR001245 (PRINTS); IPR020635 (SMART); G3DSA:1.10.510.10 (GENE3D); IPR001245 (PFAM); PTHR24416:SF63 (PANTHER); PTHR24416 (PANTHER); TRANSMEMBRANE (PHOBIUS); TRANSMEMBRANE (PHOBIUS); NON_CYTOPLASMIC_DOMAIN (PHOBIUS); NON_CYTOPLASMIC_DOMAIN (PHOBIUS); CYTOPLASMIC_DOMAIN (PHOBIUS); IPR000719 (PROSITE_PROFILES); IPR011009 (SUPERFAMILY); TMhelix (TMHMM)
519	353229_length_488_cvg_3.0_tip_1_2	5	neutral ceramidase-like	162	5.52E-59	70.70%	0.121		IPR006823 (PFAM); PTHR12670:SF1 (PANTHER); IPR006823 (PANTHER)
520	353415_length_489_cvg_3.0_tip_1_5	3	protein fem-1 homolog cg6966 isoform x1	163	4.51E-71	88.70%	0.166		IPR002110 (PRINTS); IPR002110 (SMART); IPR002110 (PFAM); IPR020683 (PFAM); IPR020683 (G3DSA:1.25.40.GENE3D); IPR020683 (G3DSA:1.25.40.GENE3D); PTHR24173 (PANTHER); PTHR24173:SF15 (PANTHER); IPR002110 (PROSITE_PROFILES); IPR002110 (PROSITE_PROFILES); IPR002110 (PROSITE_PROFILES); IPR020683 (PROSITE_PROFILES); IPR020683 (SUPERFAMILY)
521	353849_length_490_cvg_63.0_tip_0_5	47563	low-density lipoprotein receptor-related protein 5	163	6.72E-23	56.60%	0.885	Y	IPR002172 (PRINTS); IPR002172 (SMART); IPR002172 (PFAM); IPR002172 (G3DSA:4.10.400.GENE3D); IPR002172 (G3DSA:4.10.400.GENE3D); PTHR10529 (PANTHER); IPR023415 (PROSITE_PATTERNS); IPR023415 (PROSITE_PATTERNS); SIGNAL_PEPTIDE_H_REGION (PHOBIUS); SIGNAL_PEPTIDE_N_REGION (PHOBIUS); NON_CYTOPLASMIC_DOMAIN (PHOBIUS); SIGNAL_PEPTIDE (PHOBIUS); SIGNAL_PEPTIDE_C_REGION (PHOBIUS); IPR002172 (PROSITE_PROFILES); IPR002172 (PROSITE_PROFILES); IPR002172 (PROSITE_PROFILES); SignalP-noTM (SIGNALP_EUK); SignalP-TM (SIGNALP_GRAM_POSITIVE); IPR002172 (SUPERFAMILY); IPR002172 (SUPERFAMILY); IPR002172 (SUPERFAMILY)
522	355275_length_494_cvg_30.0_tip_1_0	36	polyubiquitin-b isoform x2	165	1.34E-110	99.00%	0.103		IPR019956 (PRINTS); IPR000626 (SMART); G3DSA:3.10.20.90 (GENE3D); G3DSA:3.10.20.90 (GENE3D); IPR000626 (PFAM); PTHR10666 (PANTHER); IPR019954 (PROSITE_PATTERNS); IPR019954 (PROSITE_PATTERNS); IPR000626 (PROSITE_PROFILES); IPR000626 (PROSITE_PROFILES); IPR000626 (PROSITE_PROFILES); IPR029071 (SUPERFAMILY); IPR029071 (SUPERFAMILY); IPR029071 (SUPERFAMILY)
523	355307_length_494_cvg_5.6_tip_1_1	8	peroxiredoxin 1	165	6.41E-60	91.90%	0.108		IPR019479 (PFAM); IPR000866 (PFAM); IPR012336 (G3DSA:3.40.30.GENE3D); PTHR10681 (PANTHER); PTHR10681:SF101 (PANTHER); IPR012336 (SUPERFAMILY)
524	356111_length_496_cvg_3.0_tip_1_4	3	neuropeptide f	165	8.16E-08	65.80%	0.209		IPR001955 (SMART); G3DSA:1.20.5.570 (GENE3D); IPR001955 (PFAM); CYTOPLASMIC_DOMAIN (PHOBIUS); NON_CYTOPLASMIC_DOMAIN (PHOBIUS); TRANSMEMBRANE (PHOBIUS); IPR001955 (PROSITE_PROFILES); TMhelix (TMHMM)
525	356545_length_497_cvg_2.0_tip_1_3	5	venom serine protease bi-vsp-like	166	1.30E-45	68.40%	0.102		IPR001314 (PRINTS); IPR001254 (SMART); G3DSA:2.40.10.10 (GENE3D); IPR001254 (PFAM); G3DSA:2.40.10.10 (GENE3D); PTHR24268:SF97 (PANTHER); PTHR24268 (PANTHER); IPR018114 (PROSITE_PATTERNS); IPR001254 (PROSITE_PROFILES); IPR009003 (SUPERFAMILY)
526	356687_length_497_cvg_2.9_tip_1_0	5	50-kda dystrophin-associated glycoprotein	166	4.86E-15	67.30%	0.117		IPR013783 (G3DSA:2.60.40.GENE3D); IPR016186 (G3DSA:3.10.100.GENE3D); IPR008908 (PFAM); IPR008908 (PANTHER); PTHR10132:SF14 (PANTHER); IPR016187 (SUPERFAMILY)
527	357067_length_498_cvg_69.0_tip_1_0	244	chymotrypsin-like elastase family member 2a	166	1.19E-13	55.60%	0.329		G3DSA:2.40.10.10 (GENE3D); G3DSA:2.40.10.10 (GENE3D); IPR001254 (PFAM); PTHR24275:SF45 (PANTHER); PTHR24275 (PANTHER); IPR018114 (PROSITE_PATTERNS); NON_CYTOPLASMIC_DOMAIN (PHOBIUS); CYTOPLASMIC_DOMAIN (PHOBIUS); TRANSMEMBRANE (PHOBIUS); IPR001254 (PROSITE_PROFILES); IPR009003 (SUPERFAMILY); TMhelix (TMHMM)
528	357261_length_499_cvg_2.3_tip_1_4	4	atp-dependent partial	166	6.85E-113	99.90%	0.162		G3DSA:1.10.8.60 (GENE3D); IPR003959 (PFAM); IPR027417 (G3DSA:3.40.50.GENE3D); PTHR23076:SF10 (PANTHER); PTHR23076 (PANTHER); IPR003960 (PROSITE_PATTERNS); IPR027417 (SUPERFAMILY)

529	358282_length_502_cvg_63.0_tip_0_4	376	inactive hydroxysteroid dehydrogenase-like protein 1	167	7.29E-68	78.40%	0.104		Coil (COILS); IPR002347 (PRINTS); IPR002198 (PRINTS); IPR016040 (G3DSA:3.40.50.GENE3D); IPR002198 (PFAM); PTHR24316:SF317 (PANTHER); PTHR24316 (PANTHER); IPR020904 (PROSITE_PATTERNS); SSF51735 (SUPERFAMILY)
530	359418_length_505_cvg_5.3_tip_1_1	9	3-ketoacyl-acp reductase	168	6.76E-86	99.20%	0.12		IPR002347 (PRINTS); IPR001227 (G3DSA:3.40.366.GENE3D); IPR016040 (G3DSA:3.40.50.GENE3D); IPR002198 (PFAM); PTHR24312 (PANTHER); PTHR24312:SF0 (PANTHER); IPR016035 (SUPERFAMILY); SSF51735 (SUPERFAMILY)
531	359528_length_505_cvg_2.0_tip_1_5	5	multiple epidermal growth factor-like domains protein 10 isoform x1	168	8.42E-34	69.00%	0.109		PR00011 (PRINTS); IPR002049 (SMART); G3DSA:2.170.300.10 (GENE3D); IPR013032 (PFAM); IPR002049 (PFAM); PTHR24035 (PANTHER); IPR013032 (PROSITE_PATTERNS); IPR013032 (PROSITE_PATTERNS)
534	359954_length_506_cvg_35.0_tip_0_2	66	histone h1	168	1.72E-40	76.70%	0.15		IPR005819 (PRINTS); IPR005818 (SMART); IPR011991 (G3DSA:1.10.10.GENE3D); IPR005818 (PFAM); PTHR11467 (PANTHER); IPR005818 (PROSITE_PROFILES); SSF46785 (SUPERFAMILY)
562	366934_length_526_cvg_2.7_tip_1_1	2	dscam splice variant	168	1.13E-26	70.00%	0.145		IPR003598 (SMART); IPR013783 (G3DSA:2.60.40.GENE3D); PF13895 (PFAM); PTHR10489 (PANTHER); IPR007110 (PROSITE_PROFILES); SSF48726 (SUPERFAMILY)
532	359746_length_506_cvg_2.0_tip_1_1	6	mre11 meiotic recombination 11 homolog a (cerevisiae) isoform cra_b	169	6.91E-21	72.40%	0.146		PTHR22878 (PANTHER)
533	359746_length_506_cvg_2.0_tip_1_4	6	5 -nucleotidase domain containing isoform cra_d	169	2.09E-23	67.00%	0.201		PF13900 (PFAM); PTHR16213 (PANTHER)
535	360270_length_507_cvg_2.0_tip_1_5	4	zinc finger protein 530-like	169	5.75E-22	60.90%	0.167		IPR015880 (SMART); IPR013087 (G3DSA:3.30.160.GENE3D); PF13465 (PFAM); IPR007087 (PFAM); IPR013087 (G3DSA:3.30.160.GENE3D); PTHR24409 (PANTHER); IPR007087 (PROSITE_PATTERNS); IPR007087 (PROSITE_PATTERNS); IPR007087 (PROSITE_PROFILES); IPR007087 (PROSITE_PROFILES); SSF57667 (SUPERFAMILY); SSF57667 (SUPERFAMILY)
536	360526_length_508_cvg_7.9_tip_1_2	12	mitochondrial import inner membrane translocase subunit tim8	169	2.92E-30	84.30%	0.107		IPR004217 (PFAM); IPR004217 (G3DSA:1.10.287.GENE3D); PTHR21535:SF25 (PANTHER); PTHR21535 (PANTHER); IPR004217 (SUPERFAMILY)
537	360562_length_508_cvg_2.0_tip_1_2	6	glucosidase 2 subunit beta	169	2.70E-63	70.70%	0.107		Coil (COILS); PF13015 (PFAM); IPR009011 (G3DSA:2.70.130.GENE3D); IPR026874 (PTHR12630:PANTHER); PTHR12630 (PANTHER); IPR009011 (SUPERFAMILY)
539	360628_length_508_cvg_2.5_tip_1_5	6	dnaj homolog subfamily c member 25 homolog	169	1.17E-63	81.20%	0.269		Coil (COILS); IPR001623 (PRINTS); IPR001623 (SMART); IPR001623 (G3DSA:1.10.287.GENE3D); IPR001623 (PFAM); PTHR24078:SF125 (PANTHER); PTHR24078 (PANTHER); IPR018253 (PROSITE_PATTERNS); NON_CYTOPLASMIC_DOMAIN (PHOBIUS); TRANSMEMBRANE (PHOBIUS); CYTOPLASMIC_DOMAIN (PHOBIUS); IPR001623 (PROSITE_PROFILES); IPR001623 (SUPERFAMILY); TMhelix (TMHMM)
538	360580_length_508_cvg_65.6_tip_1_0	245	chitinase 10 precursor	170	4.80E-41	69.20%	0.188		IPR013781 (G3DSA:3.20.20.GENE3D); IPR001223 (PFAM); PTHR11177 (PANTHER); PTHR11177:SF112 (PANTHER); IPR017853 (SUPERFAMILY); TMhelix (TMHMM)
540	361324_length_510_cvg_57.5_tip_1_0	276	projectin short variant	170	1.49E-44	86.80%	0.196		IPR003598 (SMART); IPR013783 (G3DSA:2.60.40.GENE3D); IPR013098 (PFAM); PTHR19897 (PANTHER); PTHR19897:SF155 (PANTHER); IPR007110 (PROSITE_PROFILES); SSF48726 (SUPERFAMILY)
541	361506_length_510_cvg_2.0_tip_1_2	3	sp6-9 protein	170	1.98E-57	84.80%	0.159		IPR015880 (SMART); PF13465 (PFAM); IPR013087 (G3DSA:3.30.160.GENE3D); IPR013087 (G3DSA:3.30.160.GENE3D); PTHR23223:SF134 (PANTHER); PTHR23223 (PANTHER); IPR007087 (PROSITE_PATTERNS); IPR007087 (PROSITE_PATTERNS); IPR007087 (PROSITE_PROFILES); IPR007087 (PROSITE_PROFILES); SSF57667 (SUPERFAMILY)
542	361666_length_511_cvg_91.9_tip_1_2	123141	low-density lipoprotein receptor-related protein 5	170	1.03E-23	59.80%	0.420	Y	IPR002172 (PRINTS); IPR002172 (SMART); IPR002172 (PFAM); IPR002172 (G3DSA:4.10.400.GENE3D); IPR002172 (G3DSA:4.10.400.GENE3D); IPR002172 (G3DSA:4.10.400.GENE3D); PTHR10529 (PANTHER); IPR023415 (PROSITE_PATTERNS); IPR023415 (PROSITE_PATTERNS); TRANSMEMBRANE (PHOBIUS); CYTOPLASMIC_DOMAIN (PHOBIUS); NON_CYTOPLASMIC_DOMAIN (PHOBIUS); IPR002172 (PROSITE_PROFILES); IPR002172 (PROSITE_PROFILES); IPR002172 (PROSITE_PROFILES); SignalP-TM (SIGNALP_GRAM_POSITIVE); IPR002172 (SUPERFAMILY); IPR002172 (SUPERFAMILY); IPR002172 (SUPERFAMILY); TMhelix (TMHMM)
543	361682_length_511_cvg_63.9_tip_0_2	462	gamma-glutamyl hydrolase-like	170	6.01E-48	72.40%	0.342	Y	IPR011697 (PFAM); IPR029062 (G3DSA:3.40.50.GENE3D); PTHR11315:SF2 (PANTHER); IPR015527 (PANTHER); NON_CYTOPLASMIC_DOMAIN (PHOBIUS); TRANSMEMBRANE (PHOBIUS); CYTOPLASMIC_DOMAIN (PHOBIUS); IPR015527 (PROSITE_PROFILES); IPR029062 (SUPERFAMILY); TMhelix (TMHMM)
544	361778_length_511_cvg_2.0_tip_1_0	2	serine threonine-protein kinase 17b-like	171	4.78E-91	85.50%	0.151		IPR002290 (SMART); G3DSA:3.30.200.20 (GENE3D); IPR000719 (PFAM); G3DSA:1.10.510.10 (GENE3D); PTHR24342 (PANTHER); IPR008271 (PROSITE_PATTERNS); IPR000719 (PROSITE_PROFILES); IPR011009 (SUPERFAMILY)

545	361938_length_512_cvg_4.0_tip_1_0	6	kunitz bovine pancreatic trypsin inhibitor domain protein	171	2.46E-40	60.10%	0.106		IPR002223 (PRINTS); IPR002223 (SMART); IPR002223 (G3DSA:4.10.410.GENE3D); IPR002223 (G3DSA:4.10.410.GENE3D); IPR002223 (PFAM); IPR002223 (G3DSA:4.10.410.GENE3D); PTHR10083 (PANTHER); IPR020901 (PROSITE_PATTERNS); IPR020901 (PROSITE_PATTERNS); IPR020901 (PROSITE_PATTERNS); IPR002223 (PROSITE_PROFILES); IPR002223 (PROSITE_PROFILES); IPR002223 (PROSITE_PROFILES); IPR002223 (SUPERFAMILY); IPR002223 (SUPERFAMILY); IPR002223 (SUPERFAMILY)
546	362090_length_512_cvg_3.1_tip_1_0	8	hemocyte protein-glutamine gamma-glutamyltransferase-like	171	4.93E-25	63.90%	0.166		IPR001102 (PFAM); IPR013783 (G3DSA:2.60.40.GENE3D); IPR023608 (PANTHER); PTHR11590:SF40 (PANTHER); IPR014756 (SUPERFAMILY)
547	362276_length_513_cvg_2.9_tip_1_5	4	von willebrand factor c and egf domain-containing protein	171	1.03E-06	54.33%	0.107		IPR001007 (SMART); G3DSA:2.10.70.10 (GENE3D); IPR001007 (PFAM); IPR001007 (PROSITE_PATTERNS); IPR001007 (PROSITE_PROFILES); SSF57603 (SUPERFAMILY)
548	362556_length_513_cvg_2.0_tip_1_3	5	lipophorin receptor	171	2.71E-91	85.50%	0.11		IPR000033 (SMART); IPR000033 (PFAM); IPR011042 (G3DSA:2.120.10.GENE3D); PTHR10529:SF208 (PANTHER); PTHR10529 (PANTHER); IPR000033 (PROSITE_PROFILES); IPR000033 (PROSITE_PROFILES); IPR000033 (PROSITE_PROFILES); IPR000033 (PROSITE_PROFILES); IPR000033 (PROSITE_PROFILES); SSF63825 (SUPERFAMILY)
549	362778_length_514_cvg_37.6_tip_1_5	49	vacuolar-sorting protein snf8-like	171	2.97E-71	90.40%	0.142		Coil (COILS); IPR007286 (PFAM); IPR011991 (G3DSA:1.10.10.GENE3D); IPR007286 (PANTHER); PTHR12806:SF0 (PANTHER); SSF46785 (SUPERFAMILY)
550	362968_length_515_cvg_3.4_tip_1_0	6	protein cbg27381	172	7.67E-44	61.50%	0.099		PF13358 (PFAM); PTHR23022:SF64 (PANTHER); PTHR23022 (PANTHER)
551	362996_length_515_cvg_12.8_tip_1_0	21	protein disulfide-isomerase a6	172	2.13E-52	74.30%	0.680	Y	PR00421 (PRINTS); IPR012336 (G3DSA:3.40.30.GENE3D); IPR013766 (PFAM); IPR005788 (TIGRFAM); PTHR18929 (PANTHER); PTHR18929:SF38 (PANTHER); IPR017937 (PROSITE_PATTERNS); SIGNAL_PEPTIDE_N_REGION (PHOBIUS); NON_CYTOPLASMIC_DOMAIN (PHOBIUS); SIGNAL_PEPTIDE (PHOBIUS); SIGNAL_PEPTIDE_H_REGION (PHOBIUS); SIGNAL_PEPTIDE_C_REGION (PHOBIUS); IPR012336 (PROSITE_PROFILES); SignalP-noTM (SIGNALP_EUK); IPR012336 (SUPERFAMILY); TMhelix (TMHMM)
552	363010_length_515_cvg_63.0_tip_0_0	173	---NA---	172			0.103		IPR008197 (PRINTS); IPR008197 (SMART); IPR008197 (PFAM); IPR008197 (G3DSA:4.10.75.GENE3D); IPR008197 (PROSITE_PROFILES); IPR008197 (PROSITE_PROFILES); IPR008197 (SUPERFAMILY)
553	363018_length_515_cvg_21.3_tip_0_3	40	prophenoloxidase activating factor	172	2.04E-57	69.10%	0.118		IPR001314 (PRINTS); IPR001254 (SMART); G3DSA:2.40.10.10 (GENE3D); IPR001254 (PFAM); G3DSA:2.40.10.10 (GENE3D); G3DSA:2.40.10.10 (GENE3D); PTHR24268:SF95 (PANTHER); PTHR24268 (PANTHER); IPR018114 (PROSITE_PATTERNS); IPR001254 (PROSITE_PROFILES); IPR009003 (SUPERFAMILY)
554	363678_length_517_cvg_8.5_tip_1_1	11	extracellular zinc metalloprotease	172	1.37E-08	44.50%	0.103		IPR011096 (PFAM); IPR025711 (PFAM)
555	363782_length_517_cvg_48.9_tip_1_5	81	serine protease	172	6.92E-09	64.90%	0.128		IPR001254 (PFAM); G3DSA:2.40.10.10 (GENE3D); PTHR24268 (PANTHER); IPR009003 (SUPERFAMILY)
587	373648_length_546_cvg_12.4_tip_1_3	27	retinal dehydrogenase 1	172	2.14E-68	89.50%	0.101		IPR015590 (PFAM); IPR016163 (G3DSA:3.40.309.GENE3D); PTHR11699:SF145 (PANTHER); PTHR11699 (PANTHER); IPR016161 (SUPERFAMILY)
556	364314_length_518_cvg_3.0_tip_1_1	8	penicillin-binding protein partial	173	3.26E-116	99.50%	0.141		IPR001264 (PFAM); G3DSA:1.10.3810.10 (GENE3D); PTHR32282:SF1 (PANTHER); PTHR32282 (PANTHER); IPR023346 (SUPERFAMILY)
557	364844_length_520_cvg_7.2_tip_1_0	6	lpxtg-motif protein cell wall anchor domain protein	174	5.03E-11	49.10%	0.219		no IPS match
558	365050_length_521_cvg_19.3_tip_1_0	32	protein d2-like isoform x1	174	6.90E-64	77.40%	0.168		IPR008914 (G3DSA:3.90.280.GENE3D); IPR008914 (PFAM); PTHR11362 (PANTHER); IPR008914 (SUPERFAMILY)
559	365084_length_521_cvg_64.3_tip_0_0	3120	chitinase partial	174	7.87E-50	62.30%	0.107		IPR011583 (SMART); IPR001223 (PFAM); IPR013781 (G3DSA:3.20.20.GENE3D); IPR029070 (G3DSA:3.10.50.GENE3D); PTHR11177 (PANTHER); PTHR11177:SF112 (PANTHER); IPR001579 (PROSITE_PATTERNS); IPR017853 (SUPERFAMILY); IPR029070 (SUPERFAMILY)
560	365278_length_521_cvg_93.5_tip_0_3	485	chitinase partial	174	3.31E-50	61.20%	0.11		IPR011583 (SMART); IPR029070 (G3DSA:3.10.50.GENE3D); IPR013781 (G3DSA:3.20.20.GENE3D); IPR001223 (PFAM); PTHR11177 (PANTHER); PTHR11177:SF112 (PANTHER); IPR001579 (PROSITE_PATTERNS); IPR029070 (SUPERFAMILY); IPR017853 (SUPERFAMILY)
561	366424_length_524_cvg_5.1_tip_1_5	10	adp-ribosylation factor 1	174	4.72E-113	100.00%	0.122		IPR006689 (PRINTS); IPR024156 (SMART); IPR006687 (SMART); IPR005225 (TIGRFAM); IPR006689 (PFAM); IPR027417 (G3DSA:3.40.50.GENE3D); PTHR11711 (PANTHER); PTHR11711:SF104 (PANTHER); IPR024156 (PROSITE_PROFILES); IPR027417 (SUPERFAMILY)
571	370778_length_537_cvg_28.6_tip_1_2	48	prohormone-1-like	174	4.86E-33	78.40%	0.113		NON_CYTOPLASMIC_DOMAIN (PHOBIUS); CYTOPLASMIC_DOMAIN (PHOBIUS); TRANSMEMBRANE (PHOBIUS); TMhelix (TMHMM)
563	367302_length_527_cvg_11.8_tip_0_2	11	heat shock cognate 71 kda protein	175	6.35E-29	75.20%	0.104		IPR029048 (G3DSA:1.20.1270.GENE3D); PTHR19375:SF142 (PANTHER); PTHR19375 (PANTHER); IPR029048 (SUPERFAMILY)

564	367396_length_527_cvg_9.2_tip_1_5	22	mucin core	175	2.02E-11	74.80%	0.108	IPR007947 (PFAM); IPR007947 (PANTHER); NON_CYTOPLASMIC_DOMAIN (PHOBIUS); CYTOPLASMIC_DOMAIN (PHOBIUS); TRANSMEMBRANE (PHOBIUS); TMhelix (TMHMM)
629	385356_length_583_cvg_51.5_tip_1_3	244	serine protease 115 isoform x1	175	1.27E-16	70.00%	0.112	IPR001254 (PFAM); G3DSA:2.40.10.10 (GENE3D); PTHR24275 (PANTHER); IPR018114 (PROSITE_PATTERNS); IPR001254 (PROSITE_PROFILES); IPR009003 (SUPERFAMILY)
565	367986_length_529_cvg_2.0_tip_1_4	4	basement membrane-specific heparan sulfate proteoglycan core protein	176	3.12E-52	77.20%	0.098	IPR001791 (SMART); IPR001791 (PFAM); IPR013320 (G3DSA:2.60.120.GENE3D); PTHR10574 (PANTHER); PTHR10574:SF247 (PANTHER); IPR001791 (PROSITE_PROFILES); IPR013320 (SUPERFAMILY)
566	368702_length_531_cvg_2.0_tip_1_1	4	lysine--trna partial	177	2.42E-125	98.90%	0.155	IPR018149 (PRINTS); G3DSA:3.30.930.10 (GENE3D); IPR012340 (G3DSA:2.40.50.GENE3D); IPR004365 (PFAM); IPR004364 (PFAM); IPR018150 (PANTHER); PTHR22594:SF25 (PANTHER); IPR006195 (PROSITE_PROFILES); SSF55681 (SUPERFAMILY); IPR012340 (SUPERFAMILY)
567	368816_length_531_cvg_5.8_tip_1_2	13	serine threonine-protein kinase mark2-like isoform x11	177	7.45E-39	90.00%	0.108	G3DSA:3.30.200.20 (GENE3D); IPR000719 (PFAM); PTHR24346:SF23 (PANTHER); PTHR24346 (PANTHER); IPR017441 (PROSITE_PATTERNS); IPR000719 (PROSITE_PROFILES); IPR011009 (SUPERFAMILY)
569	370324_length_536_cvg_18.1_tip_1_2	26	n-acetylated-alpha-linked acidic dipeptidase	178	9.85E-48	65.50%	0.108	G3DSA:3.40.630.10 (GENE3D); IPR003137 (PFAM); G3DSA:3.50.30.30 (GENE3D); PTHR10404 (PANTHER); SSF53187 (SUPERFAMILY); SSF52025 (SUPERFAMILY)
598	376840_length_556_cvg_3.5_tip_1_0	8	wnt8-like protein 1	178	3.31E-34	65.60%	0.1	IPR005817 (SMART); IPR005817 (PFAM); IPR026540 (PTHR12027:PANTHER); IPR005817 (PANTHER); TRANSMEMBRANE (PHOBIUS); CYTOPLASMIC_DOMAIN (PHOBIUS); NON_CYTOPLASMIC_DOMAIN (PHOBIUS)
607	378667_length_561_cvg_12.2_tip_1_3	26	40s ribosomal protein s26	178	2.70E-68	90.00%	0.108	IPR000892 (PFAM); IPR000892 (PANTHER); IPR000892 (PROSITE_PATTERNS)
570	370538_length_536_cvg_3.6_tip_1_4	8	peptidyl-prolyl cis-trans isomerase fkbp4-like isoform x2	179	2.89E-46	75.40%	0.122	IPR001179 (PFAM); G3DSA:3.10.50.40 (GENE3D); PTHR10516:SF274 (PANTHER); IPR023566 (PANTHER); IPR001179 (PROSITE_PROFILES); SSF54534 (SUPERFAMILY)
604	378194_length_560_cvg_6.7_tip_1_3	14	aurora kinase a-like	179	5.41E-25	96.80%	0.1	IPR002290 (SMART); IPR000719 (PFAM); G3DSA:1.10.510.10 (GENE3D); IPR030616 (PANTHER); IPR008271 (PROSITE_PATTERNS); IPR000719 (PROSITE_PROFILES); IPR011009 (SUPERFAMILY)
572	371384_length_539_cvg_2.0_tip_1_1	4	flocculation protein flo11-like	180	4.65E-09	47.00%	0.104	no IPS match
573	371414_length_539_cvg_2.2_tip_1_0	4	---NA---	180			0.103	no IPS match
574	371468_length_539_cvg_2.0_tip_1_3	3	50MGP	180	5.20E-15	50.10%	0.114	PTHR11008 (PANTHER); PTHR11008:SF13 (PANTHER); IPR020234 (PRODOM)
575	371790_length_540_cvg_2.4_tip_1_4	5	hth-type transcriptional regulator malt	180	4.62E-71	100.00%	0.137	no IPS match
576	371820_length_540_cvg_5.4_tip_1_0	9	zinc finger protein 420-like isoform x1	180	2.28E-33	59.40%	0.099	IPR015880 (SMART); IPR013087 (G3DSA:3.30.160.GENE3D); IPR013087 (G3DSA:3.30.160.GENE3D); PF13894 (PFAM); IPR007087 (PFAM); PF13465 (PFAM); PTHR24409:SF14 (PANTHER); PTHR24409 (PANTHER); IPR007087 (PROSITE_PATTERNS); IPR007087 (PROSITE_PATTERNS); IPR007087 (PROSITE_PATTERNS); IPR007087 (PROSITE_PATTERNS); IPR007087 (PROSITE_PATTERNS); IPR007087 (PROSITE_PATTERNS); IPR007087 (PROSITE_PROFILES); IPR007087 (PROSITE_PROFILES); IPR007087 (PROSITE_PROFILES); IPR007087 (PROSITE_PROFILES); IPR007087 (PROSITE_PROFILES); IPR007087 (PROSITE_PROFILES); SSF57667 (SUPERFAMILY); SSF57667 (SUPERFAMILY); SSF57667 (SUPERFAMILY); SSF57667 (SUPERFAMILY)
577	371826_length_540_cvg_20.5_tip_1_0	33	zinc finger protein 891	180	1.23E-31	62.60%	0.099	IPR015880 (SMART); PF13913 (PFAM); IPR013087 (G3DSA:3.30.160.GENE3D); IPR013087 (G3DSA:3.30.160.GENE3D); IPR013087 (G3DSA:3.30.160.GENE3D); PF13465 (PFAM); PTHR24409 (PANTHER); PTHR24409:SF13 (PANTHER); IPR007087 (PROSITE_PATTERNS); IPR007087 (PROSITE_PATTERNS); IPR007087 (PROSITE_PATTERNS); IPR007087 (PROSITE_PATTERNS); IPR007087 (PROSITE_PATTERNS); IPR007087 (PROSITE_PROFILES); IPR007087 (PROSITE_PROFILES); IPR007087 (PROSITE_PROFILES); IPR007087 (PROSITE_PROFILES); IPR007087 (PROSITE_PROFILES); IPR007087 (PROSITE_PROFILES); SSF57667 (SUPERFAMILY); SSF57667 (SUPERFAMILY); SSF57667 (SUPERFAMILY)
578	372152_length_541_cvg_63.0_tip_1_1	1338	clotting factor b-like	180	1.74E-28	55.10%	0.108	IPR001314 (PRINTS); IPR001254 (SMART); G3DSA:2.40.10.10 (GENE3D); G3DSA:2.40.10.10 (GENE3D); G3DSA:2.40.10.10 (GENE3D); IPR001254 (PFAM); PTHR24256 (PANTHER); IPR018114 (PROSITE_PATTERNS); IPR001254 (PROSITE_PROFILES); IPR009003 (SUPERFAMILY)
579	372280_length_542_cvg_2.0_tip_1_0	6	hemagglutinin amebocyte aggregation factor-like	181	2.66E-27	60.50%	0.249	PF14704 (PFAM); IPR026645 (PANTHER); NON_CYTOPLASMIC_DOMAIN (PHOBIUS); CYTOPLASMIC_DOMAIN (PHOBIUS); TRANSMEMBRANE (PHOBIUS); TMhelix (TMHMM)
580	372372_length_542_cvg_2.3_tip_1_3	3	aldehyde partial	181	2.57E-126	99.10%	0.114	IPR016162 (G3DSA:3.40.605.GENE3D); IPR015590 (PFAM); PTHR11699:SF97 (PANTHER); PTHR11699 (PANTHER); IPR029510 (PROSITE_PATTERNS); IPR016161 (SUPERFAMILY)

581	372778_length_543_cvg_2.3_tip_1_0	4	upf0528 protein cg10038	181	2.16E-25	67.50%	0.114	IPR029058 (G3DSA:3.40.50.GENE3D); PTHR21357:SF4 (PANTHER); PTHR21357 (PANTHER); IPR029058 (SUPERFAMILY)
582	372808_length_543_cvg_2.0_tip_1_5	3	zinc finger protein 678-like	181	4.74E-24	56.30%	0.14	IPR015880 (SMART); IPR013087 (G3DSA:3.30.160.GENE3D); IPR013087 (G3DSA:3.30.160.GENE3D); IPR013087 (G3DSA:3.30.160.GENE3D); PF13465 (PFAM); PTHR24409 (PANTHER); IPR007087 (PROSITE_PATTERNS); IPR007087 (PROSITE_PATTERNS); IPR007087 (PROSITE_PATTERNS); IPR007087 (PROSITE_PROFILES); IPR007087 (PROSITE_PROFILES); IPR007087 (PROSITE_PROFILES); SSF57667 (SUPERFAMILY); SSF57667 (SUPERFAMILY)
620	381064_length_569_cvg_4.2_tip_1_4	10	phosphoglycerate kinase	181	3.47E-66	92.40%	0.102	IPR001576 (PRINTS); IPR015901 (G3DSA:3.40.50.GENE3D); IPR001576 (PFAM); PTHR11406:SF6 (PANTHER); IPR001576 (PANTHER); NON_CYTOPLASMIC_DOMAIN (PHOBIUS); TRANSMEMBRANE (PHOBIUS); CYTOPLASMIC_DOMAIN (PHOBIUS); IPR001576 (SUPERFAMILY); TMhelix (TMHMM)
583	373132_length_544_cvg_4.7_tip_1_3	0	---NA---	182			0.222	IPR013781 (G3DSA:3.20.20.GENE3D); IPR017853 (SUPERFAMILY)
584	373244_length_544_cvg_12.0_tip_1_3	18	otogelin- partial	182	6.97E-11	51.20%	0.101	no IPS match
585	373548_length_545_cvg_2.0_tip_1_1	4	peptidyl-glycine alpha-amidating monooxygenase partial	182	1.61E-56	64.50%	0.307	IPR000720 (PRINTS); PF03712 (PFAM); IPR014784 (G3DSA:2.60.120.GENE3D); PTHR10680 (PANTHER); PTHR10680:SF13 (PANTHER); IPR014783 (PROSITE_PATTERNS); IPR008977 (SUPERFAMILY)
586	373602_length_546_cvg_4.5_tip_1_2	9	pacifastin light chain	182	1.15E-16	50.10%	0.128	IPR008037 (PFAM); NON_CYTOPLASMIC_DOMAIN (PHOBIUS); CYTOPLASMIC_DOMAIN (PHOBIUS); TRANSMEMBRANE (PHOBIUS); IPR020862 (PROSITE_PROFILES); IPR020862 (PROSITE_PROFILES); IPR020862 (PROSITE_PROFILES); IPR008037 (SUPERFAMILY); IPR008037 (SUPERFAMILY); IPR008037 (SUPERFAMILY); TMhelix (TMHMM)
588	373700_length_546_cvg_2.0_tip_1_2	2	calcium calmodulin-dependent protein kinase kinase 1-like isoform x2	182	1.73E-41	93.90%	0.125	IPR002290 (SMART); G3DSA:1.10.510.10 (GENE3D); IPR000719 (PFAM); PTHR24347:SF141 (PANTHER); IPR020636 (PANTHER); IPR008271 (PROSITE_PATTERNS); IPR000719 (PROSITE_PROFILES); IPR011009 (SUPERFAMILY)
589	373730_length_546_cvg_2.0_tip_1_0	2	hypothetical protein DAPPUDRAFT_240129	182	3.27E-47	64.50%	0.132	IPR019791 (PFAM); IPR019791 (G3DSA:1.10.640.GENE3D); PTHR11475 (PANTHER); IPR019791 (PROSITE_PROFILES); IPR010255 (SUPERFAMILY)
591	374124_length_547_cvg_4.4_tip_1_2	8	dbh-like monooxygenase protein 1	182	2.49E-30	57.40%	0.108	PF03712 (PFAM); IPR014784 (G3DSA:2.60.120.GENE3D); IPR000945 (PANTHER); IPR014783 (PROSITE_PATTERNS); IPR008977 (SUPERFAMILY)
592	374296_length_548_cvg_3.7_tip_1_2	13	apolipoprotein li-ii precursor	182	3.59E-17	50.50%	0.099	no IPS match
590	374088_length_547_cvg_2.0_tip_1_0	3	histone-lysine n-methyltransferase setmar isoform x2	183	1.85E-63	69.30%	0.109	IPR001888 (PFAM); IPR011991 (G3DSA:1.10.10.GENE3D); PTHR23016 (PANTHER); PTHR23016:SF3 (PANTHER)
593	374388_length_548_cvg_2.0_tip_1_3	6	short chain dehydrogenase reductase family protein	183	6.95E-132	100.00%	0.111	IPR002347 (PRINTS); IPR002198 (PFAM); IPR016040 (G3DSA:3.40.50.GENE3D); PTHR24322:SF68 (PANTHER); PTHR24322 (PANTHER); IPR020904 (PROSITE_PATTERNS); SSF51735 (SUPERFAMILY)
594	374820_length_549_cvg_2.0_tip_1_3	3	glycosyl hydrolases 31 family protein	183	8.08E-130	100.00%	0.1	IPR000322 (PFAM); IPR025887 (PFAM); PTHR22762:SF38 (PANTHER); PTHR22762 (PANTHER); IPR017853 (SUPERFAMILY); IPR011013 (SUPERFAMILY)
595	375008_length_550_cvg_66.0_tip_0_4	14274	venom serine carboxypeptidase-like	183	4.42E-42	73.50%	0.195	IPR001563 (PFAM); IPR029058 (G3DSA:3.40.50.GENE3D); IPR001563 (PANTHER); NON_CYTOPLASMIC_DOMAIN (PHOBIUS); CYTOPLASMIC_DOMAIN (PHOBIUS); TRANSMEMBRANE (PHOBIUS); IPR029058 (SUPERFAMILY); TMhelix (TMHMM)
596	375256_length_551_cvg_4.6_tip_1_2	7	myosin-binding protein fast-type-like	183	1.03E-37	65.00%	0.102	IPR003598 (SMART); IPR003599 (SMART); IPR013098 (PFAM); IPR013783 (G3DSA:2.60.40.GENE3D); IPR020675 (PANTHER); IPR007110 (PROSITE_PROFILES); SSF48726 (SUPERFAMILY)
597	375474_length_551_cvg_2.0_tip_1_3	9	membrane metallo-endopeptidase-like 1 isoform x2	184	2.18E-76	80.10%	0.125	IPR008753 (PFAM); IPR024079 (G3DSA:3.40.390.GENE3D); PTHR11733:SF113 (PANTHER); IPR000718 (PANTHER); SSF55486 (SUPERFAMILY)
653	390544_length_601_cvg_62.6_tip_1_0	573	complement component 1 q subcomponent-binding mitochondrial	184	8.76E-11	78.90%	0.105	IPR003428 (PFAM); IPR003428 (G3DSA:3.10.280.GENE3D); PTHR10826 (PANTHER); PTHR10826:SF1 (PANTHER); IPR003428 (SUPERFAMILY)
600	376886_length_556_cvg_6.1_tip_1_1	13	anti-lipopolysaccharide partial	185	1.63E-19	66.10%	0.104	IPR024509 (PFAM); CYTOPLASMIC_DOMAIN (PHOBIUS); NON_CYTOPLASMIC_DOMAIN (PHOBIUS); TRANSMEMBRANE (PHOBIUS); IPR024716 (PRODOM); TMhelix (TMHMM)
599	376866_length_556_cvg_5.5_tip_1_0	9	peroxisome biogenesis factor 10	186	2.76E-33	61.00%	0.127	IPR001841 (SMART); IPR013083 (G3DSA:3.30.40.GENE3D); PF13923 (PFAM); IPR025654 (PANTHER); IPR017907 (PROSITE_PATTERNS); CYTOPLASMIC_DOMAIN (PHOBIUS); TRANSMEMBRANE (PHOBIUS); NON_CYTOPLASMIC_DOMAIN (PHOBIUS); IPR001841 (PROSITE_PROFILES); SSF57850 (SUPERFAMILY)
601	377980_length_559_cvg_2.0_tip_1_2	3	glucose-6-phosphate partial	186	3.85E-114	100.00%	0.11	IPR001672 (PRINTS); IPR023096 (G3DSA:1.10.1390.GENE3D); G3DSA:3.40.50.10490 (GENE3D); IPR001672 (PFAM); PTHR11469:SF7 (PANTHER); IPR001672 (PANTHER); IPR018189 (PROSITE_PATTERNS); IPR001672 (PROSITE_PROFILES); SSF53697 (SUPERFAMILY)

603	378174_length_560_cvg_4.9_tip_1_5	6	fibroblast growth factor receptor-like 1	186	9.10E-27	64.00%	0.115		IPR003599 (SMART); IPR003598 (SMART); IPR013783 (G3DSA:2.60.40.GENE3D); IPR013098 (PFAM); PTHR19890 (PANTHER); PTHR19890:SF5 (PANTHER); IPR007110 (PROSITE_PROFILES); SSF48726 (SUPERFAMILY)
605	378428_length_560_cvg_3.0_tip_1_5	7	membrane metallo-endopeptidase-like 1	186	2.78E-22	86.20%	0.188		SSF55486 (SUPERFAMILY)
635	386754_length_588_cvg_57.6_tip_1_2	115	zinc finger protein zpr1	186	3.91E-73	80.30%	0.11		IPR004457 (SMART); IPR004457 (TIGRFAM); IPR004457 (PFAM); PTHR10876 (PANTHER); PTHR10876:SF0 (PANTHER)
602	378084_length_559_cvg_3.1_tip_1_3	6	hypothetical protein LOTGIDRAFT_201948	187	1.13E-71	74.30%	0.145		IPR007122 (PRINTS); IPR007122 (SMART); IPR029006 (G3DSA:3.40.20.GENE3D); IPR007123 (PFAM); IPR029006 (G3DSA:3.40.20.GENE3D); IPR015628 (PTHR11977:PANTHER); IPR007122 (PANTHER); SSF55753 (SUPERFAMILY); SSF55753 (SUPERFAMILY)
606	378535_length_561_cvg_17.8_tip_1_4	29	calponin transgelin	187	1.70E-74	89.10%	0.347	Y	IPR001997 (PRINTS); IPR003096 (PRINTS); IPR001715 (SMART); IPR001715 (PFAM); IPR001715 (G3DSA:1.10.418.GENE3D); PTHR18959:SF51 (PANTHER); PTHR18959 (PANTHER); IPR001715 (PROSITE_PROFILES); IPR001715 (SUPERFAMILY)
608	378975_length_562_cvg_2.8_tip_1_1	6	latrophilin- partial	187	8.95E-51	91.90%	0.354	Y	IPR000832 (PFAM); PTHR12011:SF258 (PANTHER); PTHR12011 (PANTHER); TRANSMEMBRANE (PHOBIUS); TRANSMEMBRANE (PHOBIUS); NON_CYTOPLASMIC_DOMAIN (PHOBIUS); TRANSMEMBRANE (PHOBIUS); NON_CYTOPLASMIC_DOMAIN (PHOBIUS); NON_CYTOPLASMIC_DOMAIN (PHOBIUS); TRANSMEMBRANE (PHOBIUS); CYTOPLASMIC_DOMAIN (PHOBIUS); CYTOPLASMIC_DOMAIN (PHOBIUS); IPR017981 (PROSITE_PROFILES); TMhelix (TMHMM); TMhelix (TMHMM); TMhelix (TMHMM); TMhelix (TMHMM)
609	378975_length_562_cvg_2.8_tip_1_2	6	latrophilin cirl-like isoform x1	187	3.09E-34	79.50%	0.176		IPR000832 (PFAM); PTHR12011:SF258 (PANTHER); PTHR12011 (PANTHER); NON_CYTOPLASMIC_DOMAIN (PHOBIUS); CYTOPLASMIC_DOMAIN (PHOBIUS); TRANSMEMBRANE (PHOBIUS); NON_CYTOPLASMIC_DOMAIN (PHOBIUS); TRANSMEMBRANE (PHOBIUS); TRANSMEMBRANE (PHOBIUS); CYTOPLASMIC_DOMAIN (PHOBIUS); TMhelix (TMHMM); TMhelix (TMHMM)
610	379319_length_563_cvg_5.8_tip_1_3	12	perq amino acid-rich with gyf domain-containing protein 2-like	188	3.46E-32	63.90%	0.112		IPR000884 (SMART); IPR000884 (PFAM); G3DSA:2.20.100.10 (GENE3D); PTHR11311 (PANTHER); IPR000884 (PROSITE_PROFILES); IPR000884 (PROSITE_PROFILES); IPR000884 (SUPERFAMILY); IPR000884 (SUPERFAMILY)
611	379831_length_565_cvg_56.3_tip_1_2	147	14-3-3 zeta	188	3.75E-88	95.00%	0.124		Coil (COILS); IPR000308 (PRINTS); IPR023410 (SMART); G3DSA:1.20.190.20 (GENE3D); IPR023410 (PFAM); IPR000308 (PANTHER); IPR023409 (PROSITE_PATTERNS); IPR023410 (SUPERFAMILY)
612	379955_length_565_cvg_2.0_tip_1_0	4	poly -specific endoribonuclease homolog	189	3.35E-48	62.40%	0.1		IPR018998 (PFAM); PTHR12439 (PANTHER); SSF142877 (SUPERFAMILY)
613	380374_length_567_cvg_2.0_tip_1_1	6	transposable element tc3 partial	189	3.24E-35	57.40%	0.106		PTHR23022:SF53 (PANTHER); PTHR23022 (PANTHER)
614	380470_length_567_cvg_68.8_tip_1_1	1460	fk506-binding protein 2 isoform x1	189	1.32E-72	81.70%	0.294		G3DSA:3.10.50.40 (GENE3D); IPR001179 (PFAM); IPR023566 (PANTHER); PTHR10516:SF252 (PANTHER); NON_CYTOPLASMIC_DOMAIN (PHOBIUS); SIGNAL_PEPTIDE_N_REGION (PHOBIUS); SIGNAL_PEPTIDE_H_REGION (PHOBIUS); SIGNAL_PEPTIDE (PHOBIUS); SIGNAL_PEPTIDE_C_REGION (PHOBIUS); IPR001179 (PROSITE_PROFILES); SSF54534 (SUPERFAMILY)
615	380582_length_567_cvg_19.0_tip_1_0	36	zinc finger protein 699-like	189	2.31E-20	55.90%	0.1		IPR015880 (SMART); IPR013087 (G3DSA:3.30.160.GENE3D); IPR013087 (G3DSA:3.30.160.GENE3D); PF13465 (PFAM); IPR013087 (G3DSA:3.30.160.GENE3D); PTHR24409 (PANTHER); IPR007087 (PROSITE_PATTERNS); IPR007087 (PROSITE_PATTERNS); IPR007087 (PROSITE_PATTERNS); IPR007087 (PROSITE_PROFILES); IPR007087 (PROSITE_PROFILES); IPR007087 (PROSITE_PROFILES); SSF57667 (SUPERFAMILY); SSF57667 (SUPERFAMILY)
616	380822_length_568_cvg_64.4_tip_0_2	40185	chitinase 2	189	9.95E-45	65.70%	0.162		IPR011583 (SMART); IPR013781 (G3DSA:3.20.20.GENE3D); IPR001223 (PFAM); PTHR11177 (PANTHER); PTHR11177:SF112 (PANTHER); TRANSMEMBRANE (PHOBIUS); CYTOPLASMIC_DOMAIN (PHOBIUS); NON_CYTOPLASMIC_DOMAIN (PHOBIUS); IPR017853 (SUPERFAMILY); TMhelix (TMHMM)
617	380840_length_568_cvg_2.0_tip_1_1	4	sonic hedgehog protein a	189	4.98E-36	56.40%	0.108		IPR001657 (PRINTS); IPR003586 (SMART); IPR001767 (PFAM); IPR028992 (G3DSA:2.170.16.GENE3D); PTHR11889 (PANTHER); IPR028992 (SUPERFAMILY)
618	380888_length_568_cvg_30.6_tip_1_3	71	low-density lipoprotein receptor domain class a	190	1.61E-16	61.20%	0.17		IPR002172 (PRINTS); IPR002172 (SMART); IPR002172 (G3DSA:4.10.400.GENE3D); IPR002172 (G3DSA:4.10.400.GENE3D); IPR002172 (PFAM); PTHR10529:SF208 (PANTHER); PTHR10529 (PANTHER); IPR023415 (PROSITE_PATTERNS); IPR023415 (PROSITE_PATTERNS); TRANSMEMBRANE (PHOBIUS); NON_CYTOPLASMIC_DOMAIN (PHOBIUS); CYTOPLASMIC_DOMAIN (PHOBIUS); IPR002172 (PROSITE_PROFILES); IPR002172 (PROSITE_PROFILES); IPR002172 (SUPERFAMILY); IPR002172 (SUPERFAMILY); TMhelix (TMHMM); TMhelix (TMHMM)

619	380938_length_568_cvg_2.0_tip_1_3	5	voltage-dependent calcium channel type a subunit alpha-1 isoform x3	190	2.41E-101	90.20%	0.176		IPR002077 (PRINTS); IPR005821 (PFAM); IPR005448 (PTHR10037:PANTHER); PTHR10037 (PANTHER); CYTOPLASMIC_DOMAIN (PHOBIUS); TRANSMEMBRANE (PHOBIUS); CYTOPLASMIC_DOMAIN (PHOBIUS); NON_CYTOPLASMIC_DOMAIN (PHOBIUS); TRANSMEMBRANE (PHOBIUS); SSF81324 (SUPERFAMILY); TMhelix (TMHMM); TMhelix (TMHMM); TMhelix (TMHMM)
621	381276_length_569_cvg_2.0_tip_1_4	2	lipase 3	190	1.33E-72	70.60%	0.161		IPR000073 (PFAM); IPR029058 (G3DSA:3.40.50.GENE3D); IPR006693 (PFAM); PTHR11005 (PANTHER); IPR029058 (SUPERFAMILY)
622	381888_length_571_cvg_2.0_tip_1_1	5	outer membrane autotransporter barrel domain protein	190	1.46E-133	100.00%	0.102		IPR005546 (G3DSA:2.40.128.GENE3D); IPR006315 (TIGRFAM); IPR005546 (PFAM); PTHR12338 (PANTHER); IPR005546 (PROSITE_PROFILES); IPR005546 (SUPERFAMILY)
623	382056_length_572_cvg_2.4_tip_1_2	5	metabotropic gaba-b receptor subtype isoform b	190	3.69E-21	96.10%	0.243		G3DSA:3.40.50.2300 (GENE3D); PTHR10519:SF3 (PANTHER); IPR002455 (PANTHER); TRANSMEMBRANE (PHOBIUS); CYTOPLASMIC_DOMAIN (PHOBIUS); NON_CYTOPLASMIC_DOMAIN (PHOBIUS); IPR028082 (SUPERFAMILY)
631	385762_length_584_cvg_3.4_tip_1_0	6	low quality protein: zinc finger protein 333	190	2.39E-22	65.10%	0.316		IPR015880 (SMART); PF13894 (PFAM); IPR007087 (PFAM); IPR013087 (G3DSA:3.30.160.GENE3D); IPR013087 (G3DSA:3.30.160.GENE3D); PTHR24409:SF13 (PANTHER); PTHR24409 (PANTHER); IPR007087 (PROSITE_PATTERNS); IPR007087 (PROSITE_PATTERNS); IPR007087 (PROSITE_PATTERNS); IPR007087 (PROSITE_PROFILES); IPR007087 (PROSITE_PROFILES); IPR007087 (PROSITE_PROFILES); IPR007087 (PROSITE_PROFILES); SSF57667 (SUPERFAMILY); SSF57667 (SUPERFAMILY)
624	382814_length_575_cvg_90.4_tip_1_4	27006	basement membrane-specific heparan sulfate proteoglycan core protein	192	1.15E-24	60.70%	0.535	Y	IPR002172 (PRINTS); IPR002172 (SMART); IPR002172 (PFAM); IPR002172 (G3DSA:4.10.400.GENE3D); IPR002172 (G3DSA:4.10.400.GENE3D); IPR002172 (G3DSA:4.10.400.GENE3D); PTHR10529 (PANTHER); IPR023415 (PROSITE_PATTERNS); IPR023415 (PROSITE_PATTERNS); IPR023415 (PROSITE_PATTERNS); IPR002172 (PROSITE_PROFILES); IPR002172 (PROSITE_PROFILES); IPR002172 (PROSITE_PROFILES); SignalP-noTM (SIGNALP_EUK); IPR002172 (SUPERFAMILY); IPR002172 (SUPERFAMILY); IPR002172 (SUPERFAMILY)
625	384012_length_579_cvg_2.9_tip_1_3	5	otopetrin-2 isoform x1	193	3.51E-62	76.70%	0.218		IPR004878 (PFAM); IPR004878 (PANTHER); PTHR21522:SF37 (PANTHER); TRANSMEMBRANE (PHOBIUS); TRANSMEMBRANE (PHOBIUS); CYTOPLASMIC_DOMAIN (PHOBIUS); TRANSMEMBRANE (PHOBIUS); NON_CYTOPLASMIC_DOMAIN (PHOBIUS); CYTOPLASMIC_DOMAIN (PHOBIUS); NON_CYTOPLASMIC_DOMAIN (PHOBIUS); TMhelix (TMHMM); TMhelix (TMHMM); TMhelix (TMHMM)
626	385008_length_582_cvg_18.6_tip_1_2	35	cytochrome p450 partial	194	1.61E-62	69.30%	0.148		IPR002401 (PRINTS); IPR001128 (PRINTS); IPR001128 (G3DSA:1.10.630.GENE3D); IPR001128 (PFAM); PTHR24305 (PANTHER); PTHR24305:SF49 (PANTHER); IPR017972 (PROSITE_PATTERNS); IPR001128 (SUPERFAMILY)
627	385244_length_583_cvg_12.0_tip_1_1	16	acetylcholine receptor subunit alpha-type acr-16	194	1.78E-40	59.70%	0.610	Y	IPR006201 (PRINTS); IPR002394 (PRINTS); IPR006202 (PFAM); IPR006202 (G3DSA:2.70.170.GENE3D); IPR006201 (PANTHER); PTHR18945:SF490 (PANTHER); SIGNAL_PEPTIDE_C_REGION (PHOBIUS); NON_CYTOPLASMIC_DOMAIN (PHOBIUS); SIGNAL_PEPTIDE_H_REGION (PHOBIUS); TRANSMEMBRANE (PHOBIUS); SIGNAL_PEPTIDE (PHOBIUS); CYTOPLASMIC_DOMAIN (PHOBIUS); SIGNAL_PEPTIDE_N_REGION (PHOBIUS); SignalP-TM (SIGNALP_EUK); IPR006202 (SUPERFAMILY)
628	385324_length_583_cvg_2.2_tip_1_2	6	alpha c-terminal all-beta domain protein	194	8.14E-137	98.00%	0.118		IPR017853 (SUPERFAMILY)
630	385556_length_584_cvg_3.0_tip_1_5	6	cysteine-rich with egf-like domain protein 2	194	3.31E-21	72.90%	0.112		IPR021852 (PFAM); PTHR24838:SF251 (PANTHER); PTHR24838 (PANTHER)
632	385970_length_585_cvg_2.0_tip_1_3	4	low quality protein: protein lap4-like	195	1.67E-90	82.60%	0.131		IPR001478 (SMART); IPR001478 (G3DSA:2.30.42.GENE3D); IPR001478 (G3DSA:2.30.42.GENE3D); IPR001478 (PFAM); IPR016313 (PANTHER); IPR001478 (PROSITE_PROFILES); IPR001478 (PROSITE_PROFILES); IPR001478 (SUPERFAMILY); IPR001478 (SUPERFAMILY)
633	386018_length_585_cvg_27.0_tip_1_2	59	dehydrogenase reductase sdr family member 11 isoform x1	195	8.03E-42	73.60%	0.105		IPR016040 (G3DSA:3.40.50.GENE3D); IPR002198 (PFAM); PTHR24322:SF310 (PANTHER); PTHR24322 (PANTHER); SSF51735 (SUPERFAMILY)

634	386026_length_585_cvg_13.4_tip_1_0	27	zinc finger protein 227	195	5.64E-53	70.00%	0.118		IPR015880 (SMART); PF13465 (PFAM); IPR013087 (G3DSA:3.30.160.GENE3D); IPR013087 (G3DSA:3.30.160.GENE3D); IPR013087 (G3DSA:3.30.160.GENE3D); PTHR24409 (PANTHER); IPR007087 (PROSITE_PATTERNS); IPR007087 (PROSITE_PATTERNS); IPR007087 (PROSITE_PATTERNS); IPR007087 (PROSITE_PATTERNS); IPR007087 (PROSITE_PATTERNS); TRANSMEMBRANE (PHOBIUS); CYTOPLASMIC_DOMAIN (PHOBIUS); NON_CYTOPLASMIC_DOMAIN (PHOBIUS); IPR007087 (PROSITE_PROFILES); IPR007087 (PROSITE_PROFILES); IPR007087 (PROSITE_PROFILES); IPR007087 (PROSITE_PROFILES); IPR007087 (PROSITE_PROFILES); SSF57667 (SUPERFAMILY); SSF57667 (SUPERFAMILY); SSF57667 (SUPERFAMILY); TMhelix (TMHMM)
636	387006_length_589_cvg_9.3_tip_1_1	21	kazal-type serine protease inhibitor domain-containing protein 1	196	3.51E-37	59.70%	0.430	Y	IPR002350 (SMART); IPR000867 (SMART); IPR002350 (PFAM); IPR000867 (PFAM); G3DSA:3.30.60.30 (GENE3D); PTHR14186:SF9 (PANTHER); IPR011390 (PANTHER); NON_CYTOPLASMIC_DOMAIN (PHOBIUS); SIGNAL_PEPTIDE (PHOBIUS); SIGNAL_PEPTIDE_H_REGION (PHOBIUS); SIGNAL_PEPTIDE_C_REGION (PHOBIUS); SIGNAL_PEPTIDE_N_REGION (PHOBIUS); IPR000867 (PROSITE_PROFILES); IPR002350 (PROSITE_PROFILES); SSF100895 (SUPERFAMILY); IPR009030 (SUPERFAMILY); TMhelix (TMHMM)
637	387016_length_589_cvg_4.4_tip_1_1	9	leukocyte elastase inhibitor isoform x2	196	1.31E-38	56.30%	0.101		IPR023796 (SMART); IPR023796 (PFAM); G3DSA:3.30.497.10 (GENE3D); G3DSA:2.30.39.10 (GENE3D); IPR000215 (PANTHER); IPR023796 (SUPERFAMILY)
639	387218_length_589_cvg_3.0_tip_1_5	5	otogelin- partial	196	2.32E-13	44.30%	0.12		no IPS match
667	393627_length_612_cvg_77.0_tip_1_0	6665	ubiquitin-60s ribosomal protein l40	196	1.58E-85	99.10%	0.104		IPR019956 (PRINTS); IPR000626 (SMART); IPR001975 (PFAM); G3DSA:3.10.20.90 (GENE3D); IPR000626 (PFAM); PTHR10666 (PANTHER); IPR019954 (PROSITE_PATTERNS); IPR000626 (PROSITE_PROFILES); IPR029071 (SUPERFAMILY); IPR011332 (SUPERFAMILY)
638	387142_length_589_cvg_2.7_tip_1_3	4	vitellogenin fused with superoxide dismutase	197	3.60E-07	52.00%	0.11		no IPS match
640	387262_length_589_cvg_2.7_tip_1_0	8	catalase peroxidase partial	197	1.36E-136	99.90%	0.104		IPR002016 (PRINTS); G3DSA:1.10.520.10 (GENE3D); G3DSA:1.10.420.10 (GENE3D); IPR002016 (PFAM); PTHR30555 (PANTHER); PTHR30555:SF0 (PANTHER); IPR019793 (PROSITE_PATTERNS); IPR010255 (SUPERFAMILY)
641	388034_length_592_cvg_2.8_tip_1_4	5	cyclin-dependent kinase 1 isoform x1	197	1.70E-110	94.50%	0.098		IPR002290 (SMART); G3DSA:1.10.510.10 (GENE3D); IPR000719 (PFAM); G3DSA:3.30.200.20 (GENE3D); PTHR24056 (PANTHER); IPR017441 (PROSITE_PATTERNS); IPR008271 (PROSITE_PATTERNS); IPR000719 (PROSITE_PROFILES); IPR011009 (SUPERFAMILY)
642	388178_length_592_cvg_2.0_tip_1_4	4	zinc finger protein	197	4.26E-38	57.20%	0.099		IPR015880 (SMART); IPR013087 (G3DSA:3.30.160.GENE3D); IPR013087 (G3DSA:3.30.160.GENE3D); IPR007087 (PFAM); IPR013087 (G3DSA:3.30.160.GENE3D); IPR013087 (G3DSA:3.30.160.GENE3D); PF13465 (PFAM); PF13894 (PFAM); PTHR24409:SF14 (PANTHER); PTHR24409 (PANTHER); IPR007087 (PROSITE_PATTERNS); IPR007087 (PROSITE_PATTERNS); IPR007087 (PROSITE_PATTERNS); IPR007087 (PROSITE_PATTERNS); IPR007087 (PROSITE_PATTERNS); IPR007087 (PROSITE_PATTERNS); IPR007087 (PROSITE_PROFILES); IPR007087 (PROSITE_PROFILES); IPR007087 (PROSITE_PROFILES); IPR007087 (PROSITE_PROFILES); IPR007087 (PROSITE_PROFILES); SSF57667 (SUPERFAMILY); SSF57667 (SUPERFAMILY); SSF57667 (SUPERFAMILY); SSF57667 (SUPERFAMILY)
643	388308_length_593_cvg_2.0_tip_1_0	6	low quality protein: mucin-19	198	1.73E-11	44.60%	0.165		SSF141571 (SUPERFAMILY); TMhelix (TMHMM); TMhelix (TMHMM); TMhelix (TMHMM)
644	388582_length_594_cvg_3.0_tip_1_1	5	serine protease	198	4.35E-13	65.30%	0.14		NON_CYTOPLASMIC_DOMAIN (PHOBIUS); NON_CYTOPLASMIC_DOMAIN (PHOBIUS); CYTOPLASMIC_DOMAIN (PHOBIUS); TRANSMEMBRANE (PHOBIUS); NON_CYTOPLASMIC_DOMAIN (PHOBIUS); TRANSMEMBRANE (PHOBIUS); TRANSMEMBRANE (PHOBIUS); CYTOPLASMIC_DOMAIN (PHOBIUS); TRANSMEMBRANE (PHOBIUS); TMhelix (TMHMM); TMhelix (TMHMM); TMhelix (TMHMM); TMhelix (TMHMM)
645	388582_length_594_cvg_3.0_tip_1_3	5	exosporium glycoprotein	198	7.47E-25	72.90%	0.297		TRANSMEMBRANE (PHOBIUS); CYTOPLASMIC_DOMAIN (PHOBIUS); NON_CYTOPLASMIC_DOMAIN (PHOBIUS); TMhelix (TMHMM); TMhelix (TMHMM); TMhelix (TMHMM); TMhelix (TMHMM)
646	388718_length_594_cvg_62.4_tip_1_1	1475	chymotrypsin-like protease ctrl-1	198	5.28E-16	56.40%	0.245		IPR001314 (PRINTS); IPR001254 (PFAM); G3DSA:2.40.10.10 (GENE3D); G3DSA:2.40.10.10 (GENE3D); PTHR24259 (PANTHER); IPR018114 (PROSITE_PATTERNS); IPR001254 (PROSITE_PROFILES); IPR009003 (SUPERFAMILY)
647	388978_length_595_cvg_6.0_tip_1_2	16	pc3-like endoprotease variant b-like	198	2.22E-38	63.00%	0.102		IPR015500 (PRINTS); IPR000209 (PFAM); IPR000209 (G3DSA:3.40.50.GENE3D); IPR015500 (PANTHER); PTHR10795:SF310 (PANTHER); IPR023827 (PROSITE_PATTERNS); IPR000209 (SUPERFAMILY)

648	389222_length_596_cvg_6.5_tip_1_2	17	secreted salivary gland	198	2.70E-10	70.80%	0.251		IPR001254 (PFAM); G3DSA:2.40.10.10 (GENE3D); PTHR24268 (PANTHER); PTHR24268:SF85 (PANTHER); CYTOPLASMIC_DOMAIN (PHOBIUS); TRANSMEMBRANE (PHOBIUS); NON_CYTOPLASMIC_DOMAIN (PHOBIUS); IPR001254 (PROSITE_PROFILES); IPR009003 (SUPERFAMILY)
673	396057_length_621_cvg_93.1_tip_1_0	2757	glyceraldehyde-3-phosphate dehydrogenase	198	3.19E-11	89.10%	0.121		IPR020831 (PANTHER); PTHR10836:SF31 (PANTHER); TRANSMEMBRANE (PHOBIUS); TRANSMEMBRANE (PHOBIUS); CYTOPLASMIC_DOMAIN (PHOBIUS); CYTOPLASMIC_DOMAIN (PHOBIUS); NON_CYTOPLASMIC_DOMAIN (PHOBIUS)
649	389278_length_596_cvg_3.6_tip_1_1	7	protein creg1	199	1.96E-57	76.60%	0.106		PF13883 (PFAM); IPR012349 (G3DSA:2.30.110.GENE3D); PTHR13343 (PANTHER); PTHR13343:SF12 (PANTHER); IPR012349 (SUPERFAMILY)
650	389306_length_596_cvg_5.4_tip_1_1	6	protein will die slowly	199	3.96E-141	98.90%	0.118		IPR020472 (PRINTS); IPR001680 (SMART); IPR001680 (PFAM); IPR015943 (G3DSA:2.130.10.GENE3D); PTHR22847:SF308 (PANTHER); PTHR22847 (PANTHER); IPR019775 (PROSITE_PATTERNS); IPR019775 (PROSITE_PATTERNS); IPR019775 (PROSITE_PATTERNS); IPR001680 (PROSITE_PROFILES); IPR001680 (PROSITE_PROFILES); IPR001680 (PROSITE_PROFILES); IPR001680 (PROSITE_PROFILES); IPR017986 (PROSITE_PROFILES); IPR001680 (PROSITE_PROFILES); IPR017986 (SUPERFAMILY)
651	389926_length_598_cvg_2.0_tip_1_5	6	tyrosine-protein phosphatase partial	199	5.91E-25	50.90%	0.106		IPR000242 (PRINTS); IPR000242 (SMART); IPR029021 (G3DSA:3.90.190.GENE3D); IPR000242 (PFAM); PTHR19134 (PANTHER); PTHR19134:SF81 (PANTHER); IPR000242 (PROSITE_PROFILES); IPR029021 (SUPERFAMILY)
682	399076_length_632_cvg_6.0_tip_1_5	10	mesencephalic astrocyte-derived neurotrophic factor homolog	199	6.92E-73	85.00%	0.166		IPR019345 (PFAM); IPR011001 (G3DSA:1.10.225.GENE3D); PTHR12990 (PANTHER); CYTOPLASMIC_DOMAIN (PHOBIUS); NON_CYTOPLASMIC_DOMAIN (PHOBIUS); TRANSMEMBRANE (PHOBIUS); SSF68906 (SUPERFAMILY); TMhelix (TMHMM)
652	390444_length_600_cvg_2.7_tip_1_4	10	annexin b10-like	200	2.63E-39	80.10%	0.107		IPR018502 (SMART); IPR018502 (PFAM); IPR018502 (G3DSA:1.10.220.GENE3D); PTHR10502 (PANTHER); SSF47874 (SUPERFAMILY)
654	391011_length_602_cvg_91.8_tip_0_3	1967	carboxypeptidase b-like	201	7.31E-83	68.70%	0.107		IPR000834 (SMART); G3DSA:3.40.630.10 (GENE3D); IPR000834 (PFAM); PTHR11705 (PANTHER); SSF53187 (SUPERFAMILY)
655	391067_length_603_cvg_62.5_tip_1_5	3082	ubiquitin-40s ribosomal protein s27a-like	201	4.35E-94	95.50%	0.241		IPR019956 (PRINTS); IPR000626 (SMART); IPR000626 (PFAM); G3DSA:3.10.20.90 (GENE3D); IPR002906 (PFAM); PTHR10666:SF99 (PANTHER); PTHR10666 (PANTHER); IPR019954 (PROSITE_PATTERNS); IPR000626 (PROSITE_PROFILES); IPR029071 (SUPERFAMILY); IPR011332 (SUPERFAMILY)
656	391107_length_603_cvg_5.8_tip_1_0	9	calcium calmodulin-dependent protein kinase type 1	201	1.88E-53	97.10%	0.1		IPR002290 (SMART); IPR000719 (PFAM); G3DSA:1.10.510.10 (GENE3D); PTHR24347:SF116 (PANTHER); IPR020636 (PANTHER); IPR008271 (PROSITE_PATTERNS); NON_CYTOPLASMIC_DOMAIN (PHOBIUS); TRANSMEMBRANE (PHOBIUS); CYTOPLASMIC_DOMAIN (PHOBIUS); NON_CYTOPLASMIC_DOMAIN (PHOBIUS); TRANSMEMBRANE (PHOBIUS); IPR000719 (PROSITE_PROFILES); IPR011009 (SUPERFAMILY); TMhelix (TMHMM); TMhelix (TMHMM)
657	391227_length_603_cvg_6.2_tip_1_5	13	neogenin isoform x3	201	9.99E-12	74.20%	0.109		IPR013783 (G3DSA:2.60.40.GENE3D); IPR013098 (PFAM); NON_CYTOPLASMIC_DOMAIN (PHOBIUS); NON_CYTOPLASMIC_DOMAIN (PHOBIUS); TRANSMEMBRANE (PHOBIUS); TRANSMEMBRANE (PHOBIUS); CYTOPLASMIC_DOMAIN (PHOBIUS); IPR007110 (PROSITE_PROFILES); SSF48726 (SUPERFAMILY)
658	391877_length_606_cvg_3.4_tip_1_1	6	proactivator polypeptide	202	3.01E-64	67.30%	0.127		IPR008373 (PRINTS); IPR003119 (SMART); IPR008139 (SMART); IPR011001 (G3DSA:1.10.225.GENE3D); IPR003119 (PFAM); IPR007856 (PFAM); IPR008138 (PFAM); PTHR11480 (PANTHER); PTHR11480:SF3 (PANTHER); IPR003119 (PROSITE_PROFILES); IPR008139 (PROSITE_PROFILES); IPR011001 (SUPERFAMILY)
660	392439_length_608_cvg_2.0_tip_1_2	6	phenoloxidase subunit a3	202	1.90E-62	65.80%	0.102		IPR000896 (PFAM); IPR008922 (G3DSA:1.10.1280.GENE3D); PTHR11511:SF24 (PANTHER); IPR013788 (PANTHER); IPR013788 (PROSITE_PATTERNS); IPR002227 (PROSITE_PATTERNS); IPR008922 (SUPERFAMILY)
659	392419_length_608_cvg_22.7_tip_1_4	55	vp302_lycmc ame: full=venom protein 302 flags: precursor	203	2.24E-14	55.80%	0.718	Y	PTHR14186:SF8 (PANTHER); IPR011390 (PANTHER); SIGNAL_PEPTIDE_N_REGION (PHOBIUS); NON_CYTOPLASMIC_DOMAIN (PHOBIUS); SIGNAL_PEPTIDE (PHOBIUS); SIGNAL_PEPTIDE_H_REGION (PHOBIUS); SIGNAL_PEPTIDE_C_REGION (PHOBIUS); IPR000867 (PROSITE_PROFILES); SignalP-noTM (SIGNALP_EUK); SignalP-TM (SIGNALP_GRAM_POSITIVE); IPR009030 (SUPERFAMILY)
661	392483_length_608_cvg_2.0_tip_1_4	4	lysine--trna partial	203	2.86E-146	100.00%	0.101		IPR018149 (PRINTS); IPR004365 (PFAM); G3DSA:3.30.930.10 (GENE3D); IPR012340 (G3DSA:2.40.50.GENE3D); IPR004364 (PFAM); IPR018150 (PANTHER); PTHR22594:SF25 (PANTHER); IPR006195 (PROSITE_PROFILES); SSF55681 (SUPERFAMILY); IPR012340 (SUPERFAMILY)

662	392707_length_609_cvg_24.5_tip_1_2	54	zinc finger and btb domain-containing protein 48	203	4.67E-19	53.00%	0.098	IPR015880 (SMART); PF13465 (PFAM); PF13894 (PFAM); IPR013087 (G3DSA:3.30.160.GENE3D); PTHR24409:SF13 (PANTHER); PTHR24409 (PANTHER); IPR007087 (PROSITE_PATTERNS); IPR007087 (PROSITE_PATTERNS); IPR007087 (PROSITE_PATTERNS); IPR007087 (PROSITE_PATTERNS); IPR007087 (PROSITE_PROFILES); IPR007087 (PROSITE_PROFILES); IPR007087 (PROSITE_PROFILES); IPR007087 (PROSITE_PROFILES); SSF57667 (SUPERFAMILY); SSF57667 (SUPERFAMILY)
663	392841_length_609_cvg_15.7_tip_1_0	44	bone morphogenetic protein 5 8 precursor	203	1.44E-42	91.20%	0.119	IPR001839 (SMART); IPR001839 (PFAM); IPR029034 (G3DSA:2.10.90.GENE3D); IPR015615 (PANTHER); PTHR11848:SF137 (PANTHER); NON_CYTOPLASMIC_DOMAIN (PHOBIUS); TRANSMEMBRANE (PHOBIUS); CYTOPLASMIC_DOMAIN (PHOBIUS); TRANSMEMBRANE (PHOBIUS); CYTOPLASMIC_DOMAIN (PHOBIUS); IPR001839 (PROSITE_PROFILES); IPR029034 (SUPERFAMILY); TMhelix (TMHMM); TMhelix (TMHMM)
664	393101_length_610_cvg_2.0_tip_1_1	6	protein sidekick-1	203	9.76E-12	50.40%	0.167	IPR013098 (PFAM); IPR013783 (G3DSA:2.60.40.GENE3D); IPR007110 (PROSITE_PROFILES); SSF48726 (SUPERFAMILY)
665	393355_length_611_cvg_3.2_tip_1_2	5	---NA---	203			0.149	CYTOPLASMIC_DOMAIN (PHOBIUS); NON_CYTOPLASMIC_DOMAIN (PHOBIUS); CYTOPLASMIC_DOMAIN (PHOBIUS); TRANSMEMBRANE (PHOBIUS); TRANSMEMBRANE (PHOBIUS)
666	393579_length_612_cvg_35.8_tip_0_3	63	serine protease nudel	204	1.29E-16	51.90%	0.1	IPR002172 (SMART); IPR001190 (PFAM); IPR002172 (PFAM); IPR002172 (G3DSA:4.10.400.GENE3D); IPR002172 (G3DSA:4.10.400.GENE3D); IPR017448 (G3DSA:3.10.250.GENE3D); IPR023415 (PROSITE_PATTERNS); IPR001190 (PROSITE_PROFILES); IPR002172 (PROSITE_PROFILES); IPR002172 (SUPERFAMILY); IPR017448 (SUPERFAMILY)
668	393999_length_614_cvg_69.2_tip_1_2	24117	venom allergen 3-like	204	1.27E-25	53.40%	0.305	IPR002413 (PRINTS); IPR014044 (SMART); IPR014044 (PFAM); IPR014044 (G3DSA:3.40.33.GENE3D); IPR001283 (PANTHER); TRANSMEMBRANE (PHOBIUS); NON_CYTOPLASMIC_DOMAIN (PHOBIUS); CYTOPLASMIC_DOMAIN (PHOBIUS); IPR014044 (SUPERFAMILY); TMhelix (TMHMM)
669	394141_length_614_cvg_2.7_tip_1_2	11	prostaglandin-endoperoxide synthase 2	204	2.37E-92	77.40%	0.106	IPR019791 (PRINTS); IPR019791 (PFAM); IPR019791 (G3DSA:1.10.640.GENE3D); PTHR11903:SF10 (PANTHER); PTHR11903 (PANTHER); IPR019791 (PROSITE_PROFILES); IPR010255 (SUPERFAMILY)
697	402288_length_644_cvg_55.1_tip_1_0	2371	peptidase s8	205	1.07E-17	61.00%	0.301	IPR007280 (PFAM); TRANSMEMBRANE (PHOBIUS); CYTOPLASMIC_DOMAIN (PHOBIUS); CYTOPLASMIC_DOMAIN (PHOBIUS); TRANSMEMBRANE (PHOBIUS); NON_CYTOPLASMIC_DOMAIN (PHOBIUS); TMhelix (TMHMM); TMhelix (TMHMM)
713	407048_length_663_cvg_2.9_tip_1_1	19	serine proteinase stubble- partial	205	2.27E-23	64.20%	0.104	IPR001254 (SMART); IPR001254 (PFAM); G3DSA:2.40.10.10 (GENE3D); PTHR24265 (PANTHER); IPR018114 (PROSITE_PATTERNS); IPR001254 (PROSITE_PROFILES); IPR009003 (SUPERFAMILY)
670	395019_length_617_cvg_3.5_tip_1_0	4	serpin i2	206	4.04E-09	66.40%	0.163	G3DSA:2.30.39.10 (GENE3D); IPR023796 (PFAM); IPR000215 (PANTHER); PTHR11461:SF52 (PANTHER); IPR023795 (PROSITE_PATTERNS); IPR023796 (SUPERFAMILY)
688	400532_length_637_cvg_6.5_tip_1_3	15	---NA---	206			0.153	IPR000742 (SMART); IPR023413 (G3DSA:2.40.155.GENE3D); IPR024731 (PFAM); IPR013032 (PROSITE_PATTERNS); IPR000152 (PROSITE_PATTERNS); CYTOPLASMIC_DOMAIN (PHOBIUS); NON_CYTOPLASMIC_DOMAIN (PHOBIUS); TRANSMEMBRANE (PHOBIUS); TRANSMEMBRANE (PHOBIUS); CYTOPLASMIC_DOMAIN (PHOBIUS); TRANSMEMBRANE (PHOBIUS); NON_CYTOPLASMIC_DOMAIN (PHOBIUS); IPR000742 (PROSITE_PROFILES); SSF57196 (SUPERFAMILY); TMhelix (TMHMM); TMhelix (TMHMM); TMhelix (TMHMM); TMhelix (TMHMM)
671	395833_length_620_cvg_2.6_tip_1_0	7	isocitrate dehydrogenase	207	3.28E-66	82.80%	0.187	IPR024084 (PFAM); IPR024084 (G3DSA:3.40.718.GENE3D); IPR001804 (PANTHER); PTHR11835:SF42 (PANTHER); SSF53659 (SUPERFAMILY); TMhelix (TMHMM)
672	395985_length_621_cvg_63.0_tip_1_4	339	probable chitinase 3	207	9.80E-54	65.30%	0.222	IPR011583 (SMART); IPR013781 (G3DSA:3.20.20.GENE3D); IPR001223 (PFAM); PTHR11177:SF112 (PANTHER); PTHR11177 (PANTHER); IPR001579 (PROSITE_PATTERNS); CYTOPLASMIC_DOMAIN (PHOBIUS); TRANSMEMBRANE (PHOBIUS); NON_CYTOPLASMIC_DOMAIN (PHOBIUS); IPR017853 (SUPERFAMILY); TMhelix (TMHMM)
674	396271_length_622_cvg_2.0_tip_1_2	4	glutathione peroxidase 6	207	2.79E-13	67.40%	0.107	IPR012336 (G3DSA:3.40.30.GENE3D); PTHR11592:SF21 (PANTHER); IPR000889 (PANTHER); IPR000889 (PROSITE_PROFILES); IPR012336 (SUPERFAMILY); TMhelix (TMHMM)

675	397317_length_626_cvg_2.0_tip_1_5	6	leucomyosuppressin precursor	208	4.44E-15	68.90%	0.768 Y	SIGNAL_PEPTIDE_N_REGION (PHOBIUS); SIGNAL_PEPTIDE (PHOBIUS); SIGNAL_PEPTIDE_H_REGION (PHOBIUS); CYTOPLASMIC_DOMAIN (PHOBIUS); NON_CYTOPLASMIC_DOMAIN (PHOBIUS); TRANSMEMBRANE (PHOBIUS); SIGNAL_PEPTIDE_C_REGION (PHOBIUS); NON_CYTOPLASMIC_DOMAIN (PHOBIUS); TRANSMEMBRANE (PHOBIUS); SignalP-TM (SIGNALP_GRAM_POSITIVE); SignalP-noTM (SIGNALP_GRAM_NEGATIVE); SignalP-noTM (SIGNALP_EUK); TMhelix (TMHMM); TMhelix (TMHMM); TMhelix (TMHMM)
696	402278_length_644_cvg_10.7_tip_1_5	24	transcription factor sp4-like	208	3.71E-72	84.20%	0.11	IPR015880 (SMART); PF13465 (PFAM); IPR013087 (G3DSA:3.30.160.GENE3D); IPR013087 (G3DSA:3.30.160.GENE3D); IPR030450 (PANTHER); IPR007087 (PROSITE_PATTERNS); IPR007087 (PROSITE_PATTERNS); IPR007087 (PROSITE_PATTERNS); IPR007087 (PROSITE_PROFILES); IPR007087 (PROSITE_PROFILES); IPR007087 (PROSITE_PROFILES); SSF57667 (SUPERFAMILY)
676	397615_length_627_cvg_3.4_tip_1_3	8	retinol dehydrogenase 13	209	6.33E-66	69.70%	0.126	IPR002198 (PRINTS); IPR002347 (PRINTS); IPR016040 (G3DSA:3.40.50.GENE3D); IPR002198 (PFAM); PTHR24320 (PANTHER); SSF51735 (SUPERFAMILY)
677	398396_length_630_cvg_2.0_tip_1_5	4	f-box wd repeat-containing protein 7	210	7.27E-111	96.90%	0.115	IPR020472 (PRINTS); IPR001680 (SMART); IPR001680 (PFAM); IPR015943 (G3DSA:2.130.10.GENE3D); PTHR22844 (PANTHER); PTHR22844:SF116 (PANTHER); IPR019775 (PROSITE_PATTERNS); IPR019775 (PROSITE_PATTERNS); IPR019775 (PROSITE_PATTERNS); IPR017986 (PROSITE_PROFILES); IPR001680 (PROSITE_PROFILES); IPR001680 (PROSITE_PROFILES); IPR001680 (PROSITE_PROFILES); IPR017986 (SUPERFAMILY)
678	398764_length_631_cvg_4.0_tip_1_5	8	hypothetical protein Y032_0655g1201	210	1.11E-45	56.20%	0.097	PF13358 (PFAM); PTHR23022:SF64 (PANTHER); PTHR23022 (PANTHER)
679	398852_length_632_cvg_3.0_tip_1_2	7	carcinolectin 5b partial	210	6.13E-28	72.60%	0.108	IPR002181 (SMART); IPR002181 (PFAM); IPR014715 (G3DSA:4.10.530.GENE3D); PTHR19143 (PANTHER); IPR020837 (PROSITE_PATTERNS); IPR002181 (PROSITE_PROFILES); IPR002181 (SUPERFAMILY)
681	399036_length_632_cvg_3.3_tip_1_2	12	---NA---	210			0.27	IPR029058 (G3DSA:3.40.50.GENE3D); NON_CYTOPLASMIC_DOMAIN (PHOBIUS); NON_CYTOPLASMIC_DOMAIN (PHOBIUS); TRANSMEMBRANE (PHOBIUS); CYTOPLASMIC_DOMAIN (PHOBIUS); TRANSMEMBRANE (PHOBIUS); TRANSMEMBRANE (PHOBIUS); NON_CYTOPLASMIC_DOMAIN (PHOBIUS); TRANSMEMBRANE (PHOBIUS); CYTOPLASMIC_DOMAIN (PHOBIUS); TMhelix (TMHMM); TMhelix (TMHMM); TMhelix (TMHMM); TMhelix (TMHMM)
680	398890_length_632_cvg_14.8_tip_1_0	23	protein shifted	211	4.47E-19	67.20%	0.105	IPR000742 (SMART); IPR013032 (PFAM); G3DSA:2.170.300.10 (GENE3D); PTHR24838 (PANTHER); PTHR24838:SF276 (PANTHER); IPR013032 (PROSITE_PATTERNS); IPR013032 (PROSITE_PATTERNS); IPR013032 (PROSITE_PATTERNS); IPR013032 (PROSITE_PATTERNS); IPR000742 (PROSITE_PROFILES); IPR000742 (PROSITE_PROFILES); IPR009030 (SUPERFAMILY)
683	399116_length_632_cvg_4.8_tip_1_4	13	hemocyte protein-glutamine gamma-glutamyltransferase-like	211	7.67E-43	71.00%	0.122	IPR013783 (G3DSA:2.60.40.GENE3D); IPR013783 (G3DSA:2.60.40.GENE3D); IPR008958 (PFAM); PTHR11590:SF40 (PANTHER); IPR023608 (PANTHER); IPR008958 (SUPERFAMILY); IPR008958 (SUPERFAMILY)
684	399290_length_633_cvg_2.0_tip_1_3	6	ankyrin repeat domain protein	211	7.44E-15	57.60%	0.107	IPR002110 (SMART); IPR020683 (PFAM); IPR020683 (G3DSA:1.25.40.GENE3D); PTHR24198 (PANTHER); IPR020683 (PROSITE_PROFILES); IPR002110 (PROSITE_PROFILES); IPR002110 (PROSITE_PROFILES); IPR002110 (PROSITE_PROFILES); IPR020683 (SUPERFAMILY)
711	406510_length_661_cvg_11.6_tip_1_4	32	xanthine dehydrogenase	211	1.16E-67	84.40%	0.101	IPR008274 (G3DSA:3.30.365.GENE3D); IPR008274 (PFAM); PTHR11908:SF32 (PANTHER); PTHR11908 (PANTHER); IPR008274 (SUPERFAMILY)
685	399988_length_636_cvg_6.7_tip_1_3	17	aldehyde oxidase	212	1.94E-17	80.90%	0.108	IPR002888 (PFAM); IPR002888 (G3DSA:1.10.150.GENE3D); PTHR11908 (PANTHER); PTHR11908:SF65 (PANTHER); TRANSMEMBRANE (PHOBIUS); NON_CYTOPLASMIC_DOMAIN (PHOBIUS); CYTOPLASMIC_DOMAIN (PHOBIUS); IPR002888 (SUPERFAMILY)
686	400146_length_636_cvg_5.2_tip_1_3	12	sodium channel protein type 5 subunit alpha	212	4.72E-09	72.80%	0.136	IPR028820 (PTHR10037:PANTHER); PTHR10037 (PANTHER); TRANSMEMBRANE (PHOBIUS); TRANSMEMBRANE (PHOBIUS); CYTOPLASMIC_DOMAIN (PHOBIUS); TRANSMEMBRANE (PHOBIUS); NON_CYTOPLASMIC_DOMAIN (PHOBIUS); NON_CYTOPLASMIC_DOMAIN (PHOBIUS); CYTOPLASMIC_DOMAIN (PHOBIUS); TMhelix (TMHMM); TMhelix (TMHMM); TMhelix (TMHMM)
689	400548_length_637_cvg_2.0_tip_1_2	6	sugar partial	212	1.42E-155	100.00%	0.103	IPR013781 (G3DSA:3.20.20.GENE3D); IPR006047 (PFAM); G3DSA:3.90.400.10 (GENE3D); IPR015902 (PANTHER); PTHR10357:SF132 (PANTHER); IPR017853 (SUPERFAMILY)

687	400354_length_637_cvg_56.6_tip_0_3	120	phenoloxidase subunit a3	213	2.76E-62	63.70%	0.138	IPR013788 (PRINTS); IPR005204 (PFAM); IPR008922 (G3DSA:1.10.1280.GENE3D); IPR005204 (G3DSA:1.20.1370.GENE3D); IPR000896 (PFAM); PTHR11511:SF4 (PANTHER); IPR013788 (PANTHER); IPR013788 (PROSITE_PATTERNS); IPR005204 (SUPERFAMILY); IPR008922 (SUPERFAMILY)
690	400566_length_638_cvg_2.6_tip_1_3	7	circumsporozoite protein	213	7.62E-30	45.70%	0.137	no IPS match
691	400566_length_638_cvg_2.6_tip_1_4	7	---NA---	213			0.104	no IPS match
692	400664_length_638_cvg_2.8_tip_1_0	7	protein cbg25835	213	3.25E-20	57.00%	0.113	PTHR23022:SF64 (PANTHER); PTHR23022 (PANTHER)
694	401352_length_641_cvg_2.0_tip_1_2	7	mitogen-activated protein kinase kinase kinase 7-like	213	4.40E-47	86.50%	0.374 Y	IPR001245 (PFAM); G3DSA:3.30.200.20 (GENE3D); PTHR23257 (PANTHER); IPR017441 (PROSITE_PATTERNS); SIGNAL_PEPTIDE_C_REGION (PHOBIUS); SIGNAL_PEPTIDE (PHOBIUS); SIGNAL_PEPTIDE_N_REGION (PHOBIUS); SIGNAL_PEPTIDE_H_REGION (PHOBIUS); NON_CYTOPLASMIC_DOMAIN (PHOBIUS); IPR000719 (PROSITE_PROFILES); SignalP-TM (SIGNALP_GRAM_POSITIVE); IPR011009 (SUPERFAMILY)
693	401270_length_640_cvg_17.4_tip_0_3	39	serine protease 44-like	214	4.82E-35	77.20%	0.245	Coil (COILS); G3DSA:2.40.10.10 (GENE3D); PF11600 (PFAM); IPR001254 (PFAM); PTHR24268:SF95 (PANTHER); PTHR24268 (PANTHER); IPR001254 (PROSITE_PROFILES); IPR009003 (SUPERFAMILY)
695	402128_length_644_cvg_2.2_tip_1_4	4	xanthine dehydrogenase	215	1.62E-22	90.80%	0.371 Y	IPR008274 (G3DSA:3.30.365.GENE3D); IPR008274 (PFAM); PTHR11908:SF32 (PANTHER); PTHR11908 (PANTHER); NON_CYTOPLASMIC_DOMAIN (PHOBIUS); CYTOPLASMIC_DOMAIN (PHOBIUS); TRANSMEMBRANE (PHOBIUS); NON_CYTOPLASMIC_DOMAIN (PHOBIUS); TRANSMEMBRANE (PHOBIUS); IPR008274 (SUPERFAMILY); TMhelix (TMHMM); TMhelix (TMHMM)
698	402434_length_645_cvg_2.6_tip_1_0	6	gastrula zinc finger	215	2.42E-24	61.30%	0.103	IPR015880 (SMART); IPR013087 (G3DSA:3.30.160.GENE3D); PF13465 (PFAM); IPR013087 (G3DSA:3.30.160.GENE3D); PF13894 (PFAM); PTHR24409 (PANTHER); IPR007087 (PROSITE_PATTERNS); IPR007087 (PROSITE_PATTERNS); IPR007087 (PROSITE_PATTERNS); IPR007087 (PROSITE_PATTERNS); IPR007087 (PROSITE_PROFILES); IPR007087 (PROSITE_PROFILES); IPR007087 (PROSITE_PROFILES); IPR007087 (PROSITE_PROFILES); SSF57667 (SUPERFAMILY); SSF57667 (SUPERFAMILY)
699	402960_length_647_cvg_4.1_tip_1_0	10	clotting factor partial	216	8.72E-25	63.90%	0.248	IPR001254 (SMART); G3DSA:2.40.10.10 (GENE3D); IPR001254 (PFAM); PTHR24265 (PANTHER); IPR001254 (PROSITE_PROFILES); IPR009003 (SUPERFAMILY)
700	403058_length_648_cvg_2.9_tip_1_3	7	hypothetical protein Y032_0001g415	216	2.20E-45	57.80%	0.114	PF13358 (PFAM); PTHR23022 (PANTHER); PTHR23022:SF64 (PANTHER)
701	403364_length_649_cvg_5.3_tip_1_2	6	alcohol dehydrogenase	216	2.67E-16	69.90%	0.128	IPR023210 (G3DSA:3.20.20.GENE3D); PTHR11732:SF183 (PANTHER); IPR001395 (PANTHER); NON_CYTOPLASMIC_DOMAIN (PHOBIUS); TRANSMEMBRANE (PHOBIUS); NON_CYTOPLASMIC_DOMAIN (PHOBIUS); CYTOPLASMIC_DOMAIN (PHOBIUS); TRANSMEMBRANE (PHOBIUS); IPR023210 (SUPERFAMILY); TMhelix (TMHMM)
702	403658_length_650_cvg_2.8_tip_1_5	7	glucose dehydrogenase	216	1.91E-51	64.10%	0.127	IPR000172 (PFAM); G3DSA:3.50.50.60 (GENE3D); PTHR11552:SF77 (PANTHER); PTHR11552 (PANTHER); IPR000172 (PROSITE_PATTERNS); SSF51905 (SUPERFAMILY); SSF54373 (SUPERFAMILY)
721	408426_length_669_cvg_7.5_tip_1_5	19	---NA---	216			0.111	G3DSA:2.40.10.10 (GENE3D); NON_CYTOPLASMIC_DOMAIN (PHOBIUS); TRANSMEMBRANE (PHOBIUS); TRANSMEMBRANE (PHOBIUS); CYTOPLASMIC_DOMAIN (PHOBIUS); CYTOPLASMIC_DOMAIN (PHOBIUS); IPR009003 (SUPERFAMILY); TMhelix (TMHMM)
703	404582_length_653_cvg_6.5_tip_1_5	21	multiple ankyrin repeats single kh domain	217	3.67E-74	90.60%	0.135	Coil (COILS); IPR002110 (SMART); IPR020683 (PFAM); IPR020683 (G3DSA:1.25.40.GENE3D); PTHR23206 (PANTHER); PTHR23206:SF4 (PANTHER); IPR020683 (PROSITE_PROFILES); IPR002110 (PROSITE_PROFILES); IPR020683 (SUPERFAMILY)
704	404856_length_654_cvg_4.3_tip_1_5	14	5 -nucleotidase	218	8.15E-72	82.80%	0.111	IPR029052 (G3DSA:3.60.21.GENE3D); PTHR11575:SF7 (PANTHER); IPR006179 (PANTHER); IPR029052 (SUPERFAMILY)
722	408538_length_669_cvg_91.4_tip_0_0	5878	c-type lectin	218	6.33E-33	51.70%	0.13	IPR002557 (SMART); IPR002557 (G3DSA:2.170.140.GENE3D); IPR013781 (G3DSA:3.20.20.GENE3D); IPR001223 (PFAM); IPR002557 (PFAM); PTHR11177 (PANTHER); PTHR11177:SF112 (PANTHER); IPR002557 (PROSITE_PROFILES); IPR002557 (SUPERFAMILY); IPR017853 (SUPERFAMILY)
734	412054_length_685_cvg_5.7_tip_1_3	17	protein disulfide	218	1.53E-109	79.70%	0.106	PR00421 (PRINTS); IPR013766 (PFAM); IPR012336 (G3DSA:3.40.30.GENE3D); IPR012336 (G3DSA:3.40.30.GENE3D); PTHR18929:SF85 (PANTHER); PTHR18929 (PANTHER); IPR012336 (PROSITE_PROFILES); IPR012336 (SUPERFAMILY); IPR012336 (SUPERFAMILY)
705	405384_length_656_cvg_81.8_tip_1_1	1287	hornerin isoform x2	219	1.47E-29	50.20%	0.123	IPR001314 (PRINTS); IPR001254 (SMART); G3DSA:2.40.10.10 (GENE3D); G3DSA:2.40.10.10 (GENE3D); IPR001254 (PFAM); G3DSA:2.40.10.10 (GENE3D); PTHR24268 (PANTHER); IPR001254 (PROSITE_PROFILES); IPR009003 (SUPERFAMILY)

706	405648_length_658_cvg_10.2_tip_1_4	28	gamma-interferon-inducible lysosomal thiol reductase	219	1.40E-19	58.20%	0.255		IPR004911 (PFAM); IPR004911 (PANTHER); PTHR13234:SF8 (PANTHER)
732	411764_length_684_cvg_5.3_tip_1_4	17	zinc finger protein partial	219	1.54E-23	59.20%	0.128		IPR015880 (SMART); IPR013087 (G3DSA:3.30.160.GENE3D); PF13465 (PFAM); IPR013087 (G3DSA:3.30.160.GENE3D); IPR013087 (G3DSA:3.30.160.GENE3D); PTHR24409 (PANTHER); PTHR24409:SF13 (PANTHER); IPR007087 (PROSITE_PATTERNS); IPR007087 (PROSITE_PATTERNS); IPR007087 (PROSITE_PATTERNS); IPR007087 (PROSITE_PROFILES); IPR007087 (PROSITE_PROFILES); IPR007087 (PROSITE_PROFILES); SSF57667 (SUPERFAMILY); SSF57667 (SUPERFAMILY)
707	406110_length_659_cvg_2.6_tip_1_0	7	hsp70 family protein	220	1.51E-157	99.90%	0.131		IPR013126 (PRINTS); IPR013126 (PFAM); G3DSA:3.30.420.40 (GENE3D); IPR029047 (G3DSA:2.60.34.GENE3D); PTHR19375:SF151 (PANTHER); PTHR19375 (PANTHER); IPR018181 (PROSITE_PATTERNS); IPR029047 (SUPERFAMILY); SSF53067 (SUPERFAMILY)
708	406298_length_660_cvg_2.0_tip_1_5	5	twitchin- partial	220	9.81E-99	80.80%	0.113		IPR003599 (SMART); IPR003598 (SMART); IPR013098 (PFAM); IPR003961 (PFAM); IPR013783 (G3DSA:2.60.40.GENE3D); IPR013783 (G3DSA:2.60.40.GENE3D); PTHR19897 (PANTHER); PTHR19897:SF155 (PANTHER); IPR007110 (PROSITE_PROFILES); IPR003961 (PROSITE_PROFILES); IPR003961 (SUPERFAMILY); SSF48726 (SUPERFAMILY)
710	406388_length_661_cvg_3.7_tip_1_2	12	zinc finger protein	220	8.53E-29	57.60%	0.098		IPR015880 (SMART); IPR013087 (G3DSA:3.30.160.GENE3D); IPR013087 (G3DSA:3.30.160.GENE3D); IPR013087 (G3DSA:3.30.160.GENE3D); IPR013087 (G3DSA:3.30.160.GENE3D); PF13465 (PFAM); PTHR24409 (PANTHER); PTHR24409:SF14 (PANTHER); IPR007087 (PROSITE_PATTERNS); IPR007087 (PROSITE_PATTERNS); IPR007087 (PROSITE_PATTERNS); IPR007087 (PROSITE_PATTERNS); IPR007087 (PROSITE_PATTERNS); IPR007087 (PROSITE_PROFILES); IPR007087 (PROSITE_PROFILES); IPR007087 (PROSITE_PROFILES); IPR007087 (PROSITE_PROFILES); IPR007087 (PROSITE_PROFILES); SSF57667 (SUPERFAMILY); SSF57667 (SUPERFAMILY); SSF57667 (SUPERFAMILY)
712	406790_length_662_cvg_2.0_tip_1_5	7	zinc finger and scan domain-containing protein 22	220	1.55E-32	55.20%	0.102		IPR015880 (SMART); IPR013087 (G3DSA:3.30.160.GENE3D); IPR013087 (G3DSA:3.30.160.GENE3D); PF13894 (PFAM); PF13465 (PFAM); PTHR24409 (PANTHER); PTHR24409:SF14 (PANTHER); IPR007087 (PROSITE_PATTERNS); IPR007087 (PROSITE_PATTERNS); IPR007087 (PROSITE_PATTERNS); IPR007087 (PROSITE_PROFILES); IPR007087 (PROSITE_PROFILES); IPR007087 (PROSITE_PROFILES); IPR007087 (PROSITE_PROFILES); SSF57667 (SUPERFAMILY); SSF57667 (SUPERFAMILY); SSF57667 (SUPERFAMILY)
709	406376_length_661_cvg_100.9_tip_0_3	160937	low-density lipoprotein receptor-related protein 2	221	8.70E-37	59.10%	0.109		IPR002172 (PRINTS); IPR002172 (SMART); IPR002172 (G3DSA:4.10.400.GENE3D); IPR002172 (PFAM); PTHR10529 (PANTHER); IPR023415 (PROSITE_PATTERNS); IPR023415 (PROSITE_PATTERNS); IPR023415 (PROSITE_PATTERNS); IPR023415 (PROSITE_PATTERNS); IPR002172 (PROSITE_PROFILES); IPR002172 (PROSITE_PROFILES); IPR002172 (PROSITE_PROFILES); IPR002172 (PROSITE_PROFILES); IPR009003 (SUPERFAMILY); IPR002172 (SUPERFAMILY); IPR002172 (SUPERFAMILY); IPR002172 (SUPERFAMILY); IPR002172 (SUPERFAMILY)
714	407216_length_664_cvg_2.0_tip_1_4	6	acyloxyacyl hydrolase	221	4.95E-70	64.30%	0.11		IPR001087 (PFAM); IPR013830 (G3DSA:3.40.50.GENE3D); PTHR15010 (PANTHER); IPR013830 (SUPERFAMILY)
716	407286_length_664_cvg_3.3_tip_1_4	11	semaphorin-1a isoform x1	221	1.37E-12	89.10%	0.356	Y	IPR015943 (G3DSA:2.130.10.GENE3D); IPR001627 (PFAM); IPR027231 (PANTHER); PTHR11036:SF66 (PANTHER); NON_CYTOPLASMIC_DOMAIN (PHOBIUS); CYTOPLASMIC_DOMAIN (PHOBIUS); CYTOPLASMIC_DOMAIN (PHOBIUS); TRANSMEMBRANE (PHOBIUS); TRANSMEMBRANE (PHOBIUS); IPR001627 (SUPERFAMILY); TMhelix (TMHMM); TMhelix (TMHMM)
715	407234_length_664_cvg_63.2_tip_1_3	347	macrophage migration inhibitory factor	222	2.37E-39	69.80%	0.135		IPR001398 (PFAM); G3DSA:3.30.429.10 (GENE3D); IPR001398 (PANTHER); PTHR11954:SF6 (PANTHER); IPR001398 (PRODOM); IPR014347 (SUPERFAMILY)
717	407458_length_665_cvg_10.9_tip_1_4	24	maguk p55 subfamily member partial	222	3.06E-23	87.60%	0.248		Coil (COILS); IPR027417 (G3DSA:3.40.50.GENE3D); PTHR23122:SF32 (PANTHER); PTHR23122 (PANTHER); CYTOPLASMIC_DOMAIN (PHOBIUS); TRANSMEMBRANE (PHOBIUS); TRANSMEMBRANE (PHOBIUS); TRANSMEMBRANE (PHOBIUS); CYTOPLASMIC_DOMAIN (PHOBIUS); NON_CYTOPLASMIC_DOMAIN (PHOBIUS); NON_CYTOPLASMIC_DOMAIN (PHOBIUS); IPR027417 (SUPERFAMILY); TMhelix (TMHMM)

718	407656_length_666_cvg_61.3_tip_1_1	706	polyubiquitin with 3 ub domains	222	1.84E-128	99.30%	0.424 Y	IPR019956 (PRINTS); IPR000626 (SMART); G3DSA:3.10.20.90 (GENE3D); IPR000626 (PFAM); G3DSA:3.10.20.90 (GENE3D); PTHR10666 (PANTHER); IPR019954 (PROSITE_PATTERNS); IPR019954 (PROSITE_PATTERNS); IPR000626 (PROSITE_PROFILES); IPR000626 (PROSITE_PROFILES); IPR000626 (PROSITE_PROFILES); IPR029071 (SUPERFAMILY); IPR029071 (SUPERFAMILY); IPR029071 (SUPERFAMILY)
719	407920_length_667_cvg_2.3_tip_1_2	6	beta-lactamase	222	9.42E-160	100.00%	0.104	IPR012338 (G3DSA:3.40.710.GENE3D); IPR001466 (PFAM); PTHR22935 (PANTHER); PTHR22935:SF58 (PANTHER); IPR001586 (PROSITE_PATTERNS); IPR012338 (SUPERFAMILY)
720	408346_length_669_cvg_2.9_tip_1_2	3	zinc finger protein 271 (zinc finger protein 7) (zinc finger protein znfphex133) (epstein-barr virus-induced zinc finger protein) (znf-eb) (ct-zfp48) (zinc finger protein	223	2.48E-10	61.50%	0.102	IPR015880 (SMART); IPR013087 (G3DSA:3.30.160.GENE3D); IPR007087 (PFAM); IPR013087 (G3DSA:3.30.160.GENE3D); PTHR24409 (PANTHER); IPR007087 (PROSITE_PATTERNS); IPR007087 (PROSITE_PATTERNS); NON_CYTOPLASMIC_DOMAIN (PHOBIUS); CYTOPLASMIC_DOMAIN (PHOBIUS); TRANSMEMBRANE (PHOBIUS); IPR007087 (PROSITE_PROFILES); IPR007087 (PROSITE_PROFILES); SSF57667 (SUPERFAMILY); TMhelix (TMHMM)
724	409466_length_673_cvg_2.6_tip_1_5	5	nicotinamide phosphoribosyltransferase	224	1.73E-30	79.70%	0.172	IPR007229 (PANTHER); IPR016471 (PTHR11098:PANTHER); TRANSMEMBRANE (PHOBIUS); NON_CYTOPLASMIC_DOMAIN (PHOBIUS); CYTOPLASMIC_DOMAIN (PHOBIUS); TMhelix (TMHMM); TMhelix (TMHMM)
725	409470_length_673_cvg_4.4_tip_1_4	10	phosphatidylethanolamine binding protein isoform 2	224	4.79E-25	85.10%	0.101	IPR008914 (G3DSA:3.90.280.GENE3D); PTHR11362 (PANTHER); IPR008914 (SUPERFAMILY)
723	409310_length_673_cvg_2.7_tip_1_3	7	gamma-glutamyl hydrolase-like	225	3.70E-10	76.90%	0.099	IPR029062 (G3DSA:3.40.50.GENE3D); IPR015527 (PANTHER); NON_CYTOPLASMIC_DOMAIN (PHOBIUS); TRANSMEMBRANE (PHOBIUS); TRANSMEMBRANE (PHOBIUS); NON_CYTOPLASMIC_DOMAIN (PHOBIUS); CYTOPLASMIC_DOMAIN (PHOBIUS); TRANSMEMBRANE (PHOBIUS); CYTOPLASMIC_DOMAIN (PHOBIUS); TMhelix (TMHMM); TMhelix (TMHMM); TMhelix (TMHMM)
726	409904_length_675_cvg_2.3_tip_1_5	6	alpha-amylase	225	2.71E-163	99.00%	0.103	IPR013781 (G3DSA:3.20.20.GENE3D); IPR006047 (PFAM); IPR017853 (SUPERFAMILY)
727	410162_length_676_cvg_2.8_tip_1_2	9	peptidyl-alpha-hydroxyglycine alpha-amidating lyase 1	225	1.10E-76	68.40%	0.115	IPR000720 (PRINTS); IPR011042 (G3DSA:2.120.10.GENE3D); IPR001258 (PFAM); PTHR10680 (PANTHER); PTHR10680:SF13 (PANTHER); IPR013017 (PROSITE_PROFILES); IPR013017 (PROSITE_PROFILES); IPR013017 (PROSITE_PROFILES); SSF63825 (SUPERFAMILY)
728	410290_length_677_cvg_3.4_tip_1_1	8	serine protease 52 isoform x2	226	2.45E-09	66.10%	0.132	IPR001254 (PFAM); G3DSA:2.40.10.10 (GENE3D); PTHR24268 (PANTHER); IPR009003 (SUPERFAMILY)
729	410484_length_678_cvg_31.1_tip_1_3	89	vacuolar-sorting protein snf8	226	3.10E-42	77.00%	0.129	IPR011991 (G3DSA:1.10.10.GENE3D); IPR011991 (G3DSA:1.10.10.GENE3D); IPR007286 (PFAM); PTHR12806:SF0 (PANTHER); IPR007286 (PANTHER); SSF46785 (SUPERFAMILY); SSF46785 (SUPERFAMILY)
750	415683_length_702_cvg_4.6_tip_1_3	15	ribonuclease oy	226	2.41E-31	65.00%	0.106	IPR001568 (PFAM); IPR001568 (G3DSA:3.90.730.GENE3D); IPR001568 (PANTHER); IPR018188 (PROSITE_PATTERNS); IPR001568 (SUPERFAMILY)
730	411116_length_681_cvg_8.0_tip_1_2	28	nidogen-2 isoform x5	227	6.98E-23	54.70%	0.429 Y	IPR000716 (SMART); IPR000716 (PFAM); IPR000716 (G3DSA:4.10.800.GENE3D); IPR022339 (PANTHER); IPR000716 (PROSITE_PATTERNS); IPR000716 (PROSITE_PATTERNS); IPR000716 (PROSITE_PROFILES); IPR000716 (PROSITE_PROFILES); IPR000716 (SUPERFAMILY); IPR000716 (SUPERFAMILY); TMhelix (TMHMM)
731	411392_length_682_cvg_3.0_tip_1_1	7	aael014742- partial	227	1.29E-49	58.60%	0.162	IPR002110 (PRINTS); IPR002110 (SMART); IPR020683 (G3DSA:1.25.40.GENE3D); IPR002110 (PFAM); IPR020683 (PFAM); IPR002110 (PFAM); IPR020683 (G3DSA:1.25.40.GENE3D); PTHR24198 (PANTHER); IPR002110 (PROSITE_PROFILES); IPR002110 (PROSITE_PROFILES); IPR002110 (PROSITE_PROFILES); IPR002110 (PROSITE_PROFILES); IPR002110 (PROSITE_PROFILES); IPR020683 (PROSITE_PROFILES); IPR002110 (PROSITE_PROFILES); IPR002110 (PROSITE_PROFILES); IPR020683 (SUPERFAMILY)
733	411928_length_684_cvg_3.3_tip_1_1	9	zinc finger protein 420-like	228	1.51E-28	64.00%	0.101	IPR015880 (SMART); IPR007087 (PFAM); IPR013087 (G3DSA:3.30.160.GENE3D); PF13465 (PFAM); IPR013087 (G3DSA:3.30.160.GENE3D); PTHR24409:SF13 (PANTHER); PTHR24409 (PANTHER); IPR007087 (PROSITE_PATTERNS); IPR007087 (PROSITE_PATTERNS); IPR007087 (PROSITE_PATTERNS); IPR007087 (PROSITE_PATTERNS); IPR007087 (PROSITE_PROFILES); IPR007087 (PROSITE_PROFILES); IPR007087 (PROSITE_PROFILES); SSF57667 (SUPERFAMILY); SSF57667 (SUPERFAMILY); SSF57667 (SUPERFAMILY)

735	412094_length_685_cvg_77.5_tip_1_3	813	brain chitinase and chia	229	1.65E-35	49.80%	0.13	IPR002557 (SMART); IPR002557 (PFAM); IPR002557 (G3DSA:2.170.140.GENE3D); IPR013781 (G3DSA:3.20.20.GENE3D); IPR001223 (PFAM); PTHR11177:SF112 (PANTHER); PTHR11177 (PANTHER); CYTOPLASMIC_DOMAIN (PHOBIUS); NON_CYTOPLASMIC_DOMAIN (PHOBIUS); TRANSMEMBRANE (PHOBIUS); IPR002557 (PROSITE_PROFILES); IPR017853 (SUPERFAMILY); IPR002557 (SUPERFAMILY)
736	412104_length_685_cvg_10.0_tip_1_3	28	apolipoprotein d-like	229	6.38E-10	44.40%	0.245	IPR012674 (G3DSA:2.40.128.GENE3D); IPR000566 (PFAM); PTHR10612:SF7 (PANTHER); PTHR10612 (PANTHER); IPR022272 (PROSITE_PATTERNS); IPR011038 (SUPERFAMILY)
737	412406_length_687_cvg_8.8_tip_1_4	20	ras-related protein rab-39b	229	7.71E-89	87.90%	0.123	IPR001806 (PRINTS); IPR003579 (SMART); IPR003578 (SMART); IPR020849 (SMART); IPR001806 (PFAM); IPR005225 (TIGRFAM); IPR027417 (G3DSA:3.40.50.GENE3D); PTHR24073 (PANTHER); PTHR24073:SF376 (PANTHER); NON_CYTOPLASMIC_DOMAIN (PHOBIUS); CYTOPLASMIC_DOMAIN (PHOBIUS); TRANSMEMBRANE (PHOBIUS); PS51419 (PROSITE_PROFILES); IPR027417 (SUPERFAMILY); TMhelix (TMHMM)
738	412926_length_689_cvg_63.0_tip_0_5	579	projectin short variant	229	7.03E-120	83.80%	0.102	IPR003598 (SMART); IPR003599 (SMART); IPR013098 (PFAM); IPR013783 (G3DSA:2.60.40.GENE3D); IPR013783 (G3DSA:2.60.40.GENE3D); PTHR19897:SF155 (PANTHER); PTHR19897 (PANTHER); IPR007110 (PROSITE_PROFILES); IPR007110 (PROSITE_PROFILES); SSF48726 (SUPERFAMILY); SSF48726 (SUPERFAMILY)
767	418535_length_716_cvg_28.0_tip_1_3	75	low quality protein: gastrula zinc finger	229	1.50E-20	61.60%	0.103	IPR015880 (SMART); IPR013087 (G3DSA:3.30.160.GENE3D); PF13465 (PFAM); IPR013087 (G3DSA:3.30.160.GENE3D); IPR013087 (G3DSA:3.30.160.GENE3D); PTHR24409 (PANTHER); IPR007087 (PROSITE_PATTERNS); IPR007087 (PROSITE_PATTERNS); IPR007087 (PROSITE_PATTERNS); IPR007087 (PROSITE_PROFILES); IPR007087 (PROSITE_PROFILES); IPR007087 (PROSITE_PROFILES); SSF57667 (SUPERFAMILY); SSF57667 (SUPERFAMILY)
739	413000_length_690_cvg_63.0_tip_0_2	538	secreted metalloprotease mcp02	230	4.04E-16	55.10%	0.098	IPR025711 (PFAM)
740	413462_length_692_cvg_7.7_tip_1_5	20	hemagglutinin amebocyte aggregation factor-like	230	1.61E-40	62.30%	0.630 Y	PF14704 (PFAM); IPR026645 (PANTHER); NON_CYTOPLASMIC_DOMAIN (PHOBIUS); CYTOPLASMIC_DOMAIN (PHOBIUS); TRANSMEMBRANE (PHOBIUS); SignalP-noTM (SIGNALP_EUK)
741	414004_length_695_cvg_6.6_tip_1_2	17	hypothetical protein LOTGIDRAFT_159529	231	3.07E-08	70.00%	0.133	IPR008197 (PRINTS); IPR008197 (SMART); IPR008197 (PFAM); IPR008197 (G3DSA:4.10.75.GENE3D); IPR008197 (PROSITE_PROFILES); IPR008197 (SUPERFAMILY)
742	414180_length_695_cvg_3.5_tip_1_2	11	amine oxidase	231	1.74E-61	66.90%	0.148	G3DSA:3.50.50.60 (GENE3D); G3DSA:1.10.405.10 (GENE3D); PF13450 (PFAM); PTHR10742:SF217 (PANTHER); PTHR10742 (PANTHER); SSF51905 (SUPERFAMILY)
743	414190_length_695_cvg_57.0_tip_1_3	200	prohormone-4	232	7.40E-71	86.30%	0.103	IPR002172 (SMART); IPR002172 (PFAM); IPR002172 (G3DSA:4.10.400.GENE3D); PTHR20967 (PANTHER); IPR023415 (PROSITE_PATTERNS); TRANSMEMBRANE (PHOBIUS); NON_CYTOPLASMIC_DOMAIN (PHOBIUS); TRANSMEMBRANE (PHOBIUS); NON_CYTOPLASMIC_DOMAIN (PHOBIUS); CYTOPLASMIC_DOMAIN (PHOBIUS); IPR002172 (PROSITE_PROFILES); IPR002172 (SUPERFAMILY); TMhelix (TMHMM); TMhelix (TMHMM)
745	414836_length_698_cvg_3.9_tip_1_5	11	protein disulfide-isomerase-like	232	2.81E-20	74.10%	0.159	IPR013766 (PFAM); IPR012336 (G3DSA:3.40.30.GENE3D); PTHR18929:SF56 (PANTHER); PTHR18929 (PANTHER); IPR012336 (PROSITE_PROFILES); IPR012336 (SUPERFAMILY)
744	414700_length_698_cvg_10.6_tip_1_0	28	peptidyl-prolyl cis-trans isomerase cyp11 isoform x1	233	8.09E-105	88.20%	0.125	IPR002130 (PRINTS); IPR029000 (G3DSA:2.40.100.GENE3D); IPR002130 (PFAM); IPR024936 (PANTHER); PTHR11071:SF229 (PANTHER); IPR020892 (PROSITE_PATTERNS); IPR002130 (PROSITE_PROFILES); IPR029000 (SUPERFAMILY)
746	414962_length_699_cvg_2.0_tip_1_2	8	zinc finger protein 345 isoform x3	233	6.87E-97	56.00%	0.101	IPR015880 (SMART); IPR013087 (G3DSA:3.30.160.GENE3D); PF13465 (PFAM); IPR007087 (PFAM); IPR013087 (G3DSA:3.30.160.GENE3D); IPR013087 (G3DSA:3.30.160.GENE3D); IPR013087 (G3DSA:3.30.160.GENE3D); PF13909 (PFAM); PTHR24409 (PANTHER); IPR007087 (PROSITE_PATTERNS); IPR007087 (PROSITE_PATTERNS); IPR007087 (PROSITE_PATTERNS); IPR007087 (PROSITE_PATTERNS); IPR007087 (PROSITE_PATTERNS); IPR007087 (PROSITE_PATTERNS); IPR007087 (PROSITE_PROFILES); IPR007087 (PROSITE_PROFILES); IPR007087 (PROSITE_PROFILES); IPR007087 (PROSITE_PROFILES); IPR007087 (PROSITE_PROFILES); IPR007087 (PROSITE_PROFILES); SSF57667 (SUPERFAMILY); SSF57667 (SUPERFAMILY); SSF57667 (SUPERFAMILY)
747	415343_length_701_cvg_5.4_tip_1_1	18	protein bv8	234	3.84E-20	59.60%	0.19	G3DSA:2.10.80.10 (GENE3D); TRANSMEMBRANE (PHOBIUS); CYTOPLASMIC_DOMAIN (PHOBIUS); NON_CYTOPLASMIC_DOMAIN (PHOBIUS); TMhelix (TMHMM)

748	415401_length_701_cvg_78.9_tip_1_3	812	myophilin-like isoform 2	234	5.07E-100	85.00%	0.103	IPR003096 (PRINTS); IPR001715 (SMART); IPR001715 (PFAM); IPR001715 (G3DSA:1.10.418.GENE3D); PTHR18959 (PANTHER); IPR000557 (PROSITE_PATTERNS); IPR000557 (PROSITE_PROFILES); IPR001715 (PROSITE_PROFILES); IPR001715 (SUPERFAMILY)
749	415499_length_702_cvg_47.8_tip_1_1	117	a-kinase anchor protein partial	234	1.01E-21	56.50%	0.098	Coil (COILS)
752	415927_length_704_cvg_3.0_tip_1_2	12	wnt10 protein	234	3.04E-34	80.60%	0.13	IPR005817 (PFAM); PTHR12027:SF89 (PANTHER); IPR005817 (PANTHER)
777	421213_length_729_cvg_3.2_tip_1_2	13	zinc finger protein	234	1.79E-31	57.30%	0.107	IPR015880 (SMART); IPR013087 (G3DSA:3.30.160.GENE3D); PF13465 (PFAM); PF13912 (PFAM); PF13894 (PFAM); PTHR24409 (PANTHER); PTHR24409:SF14 (PANTHER); IPR007087 (PROSITE_PATTERNS); IPR007087 (PROSITE_PATTERNS); IPR007087 (PROSITE_PATTERNS); IPR007087 (PROSITE_PATTERNS); IPR007087 (PROSITE_PATTERNS); IPR007087 (PROSITE_PROFILES); IPR007087 (PROSITE_PROFILES); IPR007087 (PROSITE_PROFILES); IPR007087 (PROSITE_PROFILES); IPR007087 (PROSITE_PROFILES); SSF57667 (SUPERFAMILY); SSF57667 (SUPERFAMILY); SSF57667 (SUPERFAMILY)
751	415791_length_703_cvg_8.1_tip_1_0	21	zinc finger protein	235	2.61E-54	69.70%	0.098	IPR015880 (SMART); IPR013087 (G3DSA:3.30.160.GENE3D); PF13465 (PFAM); IPR013087 (G3DSA:3.30.160.GENE3D); IPR013087 (G3DSA:3.30.160.GENE3D); IPR013087 (G3DSA:3.30.160.GENE3D); PTHR24409 (PANTHER); IPR007087 (PROSITE_PATTERNS); IPR007087 (PROSITE_PATTERNS); IPR007087 (PROSITE_PATTERNS); IPR007087 (PROSITE_PATTERNS); NON_CYTOPLASMIC_DOMAIN (PHOBIUS); CYTOPLASMIC_DOMAIN (PHOBIUS); TRANSMEMBRANE (PHOBIUS); IPR007087 (PROSITE_PROFILES); IPR007087 (PROSITE_PROFILES); IPR007087 (PROSITE_PROFILES); IPR007087 (PROSITE_PROFILES); IPR007087 (PROSITE_PROFILES); IPR007087 (PROSITE_PROFILES); SSF57667 (SUPERFAMILY); SSF57667 (SUPERFAMILY); SSF57667 (SUPERFAMILY)
753	415949_length_704_cvg_82.8_tip_1_1	946	serine protease 27-like	235	1.65E-43	54.80%	0.116	IPR001314 (PRINTS); IPR001254 (SMART); IPR001254 (PFAM); G3DSA:2.40.10.10 (GENE3D); G3DSA:2.40.10.10 (GENE3D); G3DSA:2.40.10.10 (GENE3D); PTHR24268 (PANTHER); IPR018114 (PROSITE_PATTERNS); IPR018114 (PROSITE_PATTERNS); IPR001254 (PROSITE_PROFILES); IPR009003 (SUPERFAMILY)
754	415953_length_704_cvg_2.7_tip_1_1	9	ganglioside-induced differentiation-associated protein 2	235	3.28E-56	70.70%	0.252	IPR002589 (SMART); IPR002589 (PFAM); G3DSA:3.40.220.10 (GENE3D); PTHR11106 (PANTHER); PTHR11106:SF59 (PANTHER); CYTOPLASMIC_DOMAIN (PHOBIUS); TRANSMEMBRANE (PHOBIUS); NON_CYTOPLASMIC_DOMAIN (PHOBIUS); IPR002589 (PROSITE_PROFILES); SSF52949 (SUPERFAMILY)
755	416033_length_704_cvg_5.7_tip_1_3	16	zinc finger protein 850-like	235	7.97E-41	59.00%	0.098	IPR015880 (SMART); IPR013087 (G3DSA:3.30.160.GENE3D); IPR013087 (G3DSA:3.30.160.GENE3D); IPR013087 (G3DSA:3.30.160.GENE3D); PF13465 (PFAM); IPR007087 (PFAM); IPR013087 (G3DSA:3.30.160.GENE3D); PF13894 (PFAM); PTHR24409 (PANTHER); PTHR24409:SF13 (PANTHER); IPR007087 (PROSITE_PATTERNS); IPR007087 (PROSITE_PATTERNS); IPR007087 (PROSITE_PATTERNS); IPR007087 (PROSITE_PATTERNS); IPR007087 (PROSITE_PATTERNS); IPR007087 (PROSITE_PATTERNS); IPR007087 (PROSITE_PATTERNS); IPR007087 (PROSITE_PATTERNS); IPR007087 (PROSITE_PATTERNS); IPR007087 (PROSITE_PROFILES); IPR007087 (PROSITE_PROFILES); IPR007087 (PROSITE_PROFILES); IPR007087 (PROSITE_PROFILES); IPR007087 (PROSITE_PROFILES); IPR007087 (PROSITE_PROFILES); IPR007087 (PROSITE_PROFILES); IPR007087 (PROSITE_PROFILES); IPR007087 (PROSITE_PROFILES); SSF57667 (SUPERFAMILY); SSF57667 (SUPERFAMILY); SSF57667 (SUPERFAMILY); SSF57667 (SUPERFAMILY)
756	416121_length_705_cvg_2.0_tip_1_4	6	adhesive serine protease	235	2.89E-26	67.30%	0.12	IPR001254 (SMART); G3DSA:2.40.10.10 (GENE3D); IPR001254 (PFAM); PTHR24256 (PANTHER); IPR018114 (PROSITE_PATTERNS); IPR001254 (PROSITE_PROFILES); IPR009003 (SUPERFAMILY)
757	416121_length_705_cvg_2.0_tip_1_5	6	trypsin- partial	235	5.87E-12	73.40%	0.15	G3DSA:2.40.10.10 (GENE3D); IPR001254 (PFAM); PTHR24258:SF4 (PANTHER); PTHR24258 (PANTHER); IPR009003 (SUPERFAMILY)
758	416133_length_705_cvg_68.3_tip_0_1	128445	chitinase partial	235	9.04E-60	63.30%	0.106	IPR011583 (SMART); IPR001223 (PFAM); IPR013781 (G3DSA:3.20.20.GENE3D); IPR029070 (G3DSA:3.10.50.GENE3D); PTHR11177 (PANTHER); IPR017853 (SUPERFAMILY); IPR029070 (SUPERFAMILY)

805	425325_length_749_cvg_4.2_tip_1_5	13	zinc finger protein 226-like	235	8.23E-33	60.20%	0.123	IPR015880 (SMART); PF13465 (PFAM); IPR013087 (G3DSA:3.30.160.GENE3D); IPR013087 (G3DSA:3.30.160.GENE3D); IPR007087 (PFAM); IPR013087 (G3DSA:3.30.160.GENE3D); IPR013087 (G3DSA:3.30.160.GENE3D); PTHR24409 (PANTHER); IPR007087 (PROSITE_PATTERNS); IPR007087 (PROSITE_PATTERNS); IPR007087 (PROSITE_PATTERNS); IPR007087 (PROSITE_PATTERNS); IPR007087 (PROSITE_PATTERNS); TRANSMEMBRANE (PHOBIUS); CYTOPLASMIC_DOMAIN (PHOBIUS); NON_CYTOPLASMIC_DOMAIN (PHOBIUS); IPR007087 (PROSITE_PROFILES); IPR007087 (PROSITE_PROFILES); IPR007087 (PROSITE_PROFILES); IPR007087 (PROSITE_PROFILES); IPR007087 (PROSITE_PROFILES); SSF57667 (SUPERFAMILY); SSF57667 (SUPERFAMILY); SSF57667 (SUPERFAMILY)
759	416639_length_707_cvg_2.0_tip_1_3	7	proclotting enzyme	236	3.44E-20	76.50%	0.122	IPR001254 (PFAM); G3DSA:2.40.10.10 (GENE3D); PTHR24265 (PANTHER); IPR018114 (PROSITE_PATTERNS); NON_CYTOPLASMIC_DOMAIN (PHOBIUS); TRANSMEMBRANE (PHOBIUS); CYTOPLASMIC_DOMAIN (PHOBIUS); IPR001254 (PROSITE_PROFILES); IPR009003 (SUPERFAMILY); TMhelix (TMHMM)
760	417027_length_709_cvg_4.0_tip_1_5	14	peptidyl-prolyl cis-trans isomerase-like 4	236	3.15E-76	84.20%	0.108	IPR002130 (PRINTS); IPR002130 (PFAM); IPR029000 (G3DSA:2.40.100.GENE3D); PTHR11071:SF156 (PANTHER); IPR024936 (PANTHER); IPR002130 (PROSITE_PROFILES); IPR029000 (SUPERFAMILY)
762	417143_length_709_cvg_2.2_tip_1_2	6	twitchin isoform x4	236	2.40E-116	85.60%	0.199	PR00014 (PRINTS); IPR003961 (SMART); IPR003961 (PFAM); IPR013783 (G3DSA:2.60.40.GENE3D); IPR013783 (G3DSA:2.60.40.GENE3D); IPR013783 (G3DSA:2.60.40.GENE3D); IPR013098 (PFAM); PTHR19897 (PANTHER); PTHR19897:SF142 (PANTHER); IPR003961 (PROSITE_PROFILES); SSF48726 (SUPERFAMILY); IPR003961 (SUPERFAMILY); SSF48726 (SUPERFAMILY)
763	417373_length_710_cvg_3.5_tip_1_2	13	trypsin 3	236	1.84E-32	63.40%	0.125	IPR001254 (SMART); G3DSA:2.40.10.10 (GENE3D); IPR001254 (PFAM); PTHR24260 (PANTHER); TRANSMEMBRANE (PHOBIUS); NON_CYTOPLASMIC_DOMAIN (PHOBIUS); TRANSMEMBRANE (PHOBIUS); CYTOPLASMIC_DOMAIN (PHOBIUS); CYTOPLASMIC_DOMAIN (PHOBIUS); IPR001254 (PROSITE_PROFILES); IPR009003 (SUPERFAMILY); TMhelix (TMHMM); TMhelix (TMHMM)
761	417119_length_709_cvg_2.5_tip_1_3	11	epidermal growth factor-like protein 7	237	1.29E-29	49.90%	0.117	Coil (COILS); Coil (COILS); IPR001881 (SMART); IPR000742 (SMART); IPR001881 (PFAM); G3DSA:2.10.25.10 (GENE3D); G3DSA:2.10.25.10 (GENE3D); IPR000742 (PFAM); PTHR14949 (PANTHER); PTHR14949:SF14 (PANTHER); IPR000152 (PROSITE_PATTERNS); IPR013032 (PROSITE_PATTERNS); IPR018097 (PROSITE_PATTERNS); IPR013032 (PROSITE_PATTERNS); IPR013032 (PROSITE_PATTERNS); IPR000742 (PROSITE_PROFILES); IPR000742 (PROSITE_PROFILES); SSF57196 (SUPERFAMILY); SSF57196 (SUPERFAMILY)
764	417581_length_711_cvg_4.3_tip_1_3	16	proprotein convertase subtilisin kexin type 7	237	4.37E-68	58.50%	0.645 Y	IPR002884 (PFAM); IPR008979 (G3DSA:2.60.120.GENE3D); PTHR10795:SF9 (PANTHER); IPR015500 (PANTHER); SignalP-noTM (SIGNALP_EUK); IPR008979 (SUPERFAMILY)
765	417847_length_712_cvg_4.3_tip_1_0	14	sortilin-related receptor	238	1.41E-17	80.70%	0.739 Y	G3DSA:2.130.10.140 (GENE3D); PTHR12106 (PANTHER); PTHR12106:SF7 (PANTHER); SIGNAL_PEPTIDE_N_REGION (PHOBIUS); SIGNAL_PEPTIDE_H_REGION (PHOBIUS); SIGNAL_PEPTIDE (PHOBIUS); NON_CYTOPLASMIC_DOMAIN (PHOBIUS); SIGNAL_PEPTIDE_C_REGION (PHOBIUS); SignalP-noTM (SIGNALP_EUK); SSF110296 (SUPERFAMILY); TMhelix (TMHMM)
766	418055_length_713_cvg_2.0_tip_1_4	6	tyrosine-protein kinase receptor tie-1-like isoform x1	238	2.03E-09	63.30%	0.114	G3DSA:2.10.25.10 (GENE3D); IPR013032 (PROSITE_PATTERNS); IPR013032 (PROSITE_PATTERNS); NON_CYTOPLASMIC_DOMAIN (PHOBIUS); TRANSMEMBRANE (PHOBIUS); CYTOPLASMIC_DOMAIN (PHOBIUS); IPR000742 (PROSITE_PROFILES)
768	418611_length_716_cvg_5.7_tip_1_1	15	probable nuclear transport factor 2 isoform x1	239	2.37E-63	81.90%	0.138	G3DSA:3.10.450.50 (GENE3D); IPR002075 (PFAM); PTHR12612 (PANTHER); IPR018222 (PROSITE_PROFILES); SSF54427 (SUPERFAMILY)
769	418797_length_717_cvg_2.2_tip_1_0	9	limbic system-associated membrane protein	239	3.71E-92	68.40%	0.1	IPR003961 (SMART); IPR003599 (SMART); IPR003598 (SMART); IPR013783 (G3DSA:2.60.40.GENE3D); IPR013783 (G3DSA:2.60.40.GENE3D); IPR003961 (PFAM); IPR013783 (G3DSA:2.60.40.GENE3D); IPR013098 (PFAM); PF13895 (PFAM); PTHR19831:SF40 (PANTHER); PTHR19831 (PANTHER); IPR003961 (PROSITE_PROFILES); IPR007110 (PROSITE_PROFILES); SSF48726 (SUPERFAMILY); SSF48726 (SUPERFAMILY); IPR003961 (SUPERFAMILY)
770	418907_length_717_cvg_63.6_tip_1_4	5047	granulins- partial	239	3.40E-36	53.10%	0.626 Y	IPR000118 (SMART); IPR000118 (PFAM); PTHR12274 (PANTHER); PTHR12274:SF1 (PANTHER); IPR000118 (PROSITE_PATTERNS); IPR000118 (PROSITE_PATTERNS); CYTOPLASMIC_DOMAIN (PHOBIUS); NON_CYTOPLASMIC_DOMAIN (PHOBIUS); TRANSMEMBRANE (PHOBIUS); SignalP-noTM (SIGNALP_EUK); SSF57277 (SUPERFAMILY); SSF57277 (SUPERFAMILY); TMhelix (TMHMM)

814	426739_length_757_cvg_63.0_tip_1_5	3024	virulence metalloprotease	239	6.88E-19	54.60%	0.191	IPR025711 (PFAM); IPR011096 (PFAM); CYTOPLASMIC_DOMAIN (PHOBIUS); TRANSMEMBRANE (PHOBIUS); NON_CYTOPLASMIC_DOMAIN (PHOBIUS); TMhelix (TMHMM)
771	419371_length_719_cvg_2.6_tip_1_3	10	transmembrane protease serine 12- partial	240	3.89E-23	50.20%	0.107	IPR001254 (SMART); G3DSA:2.40.10.10 (GENE3D); IPR001254 (PFAM); G3DSA:2.40.10.10 (GENE3D); PTHR24268 (PANTHER); IPR018114 (PROSITE_PATTERNS); IPR001254 (PROSITE_PROFILES); IPR009003 (SUPERFAMILY)
785	422351_length_735_cvg_7.7_tip_1_3	26	serine protease nudel	240	1.05E-34	75.10%	0.136	IPR002172 (SMART); IPR001254 (PFAM); IPR002172 (PFAM); IPR002172 (G3DSA:4.10.400.GENE3D); G3DSA:2.40.10.10 (GENE3D); PTHR24256:SF95 (PANTHER); PTHR24256 (PANTHER); IPR023415 (PROSITE_PATTERNS); IPR002172 (PROSITE_PROFILES); IPR002172 (SUPERFAMILY); IPR009003 (SUPERFAMILY)
772	420017_length_723_cvg_66.0_tip_0_2	165	secreted metalloprotease mcp02	241	2.08E-16	57.10%	0.1	IPR025711 (PFAM)
773	420109_length_723_cvg_14.6_tip_1_4	44	muscle m-line assembly protein unc-89	241	6.19E-83	70.80%	0.108	PR00014 (PRINTS); IPR003961 (SMART); IPR013783 (G3DSA:2.60.40.GENE3D); G3DSA:3.30.200.20 (GENE3D); IPR003961 (PFAM); IPR013783 (G3DSA:2.60.40.GENE3D); IPR020675 (PANTHER); PTHR22964:SF45 (PANTHER); IPR003961 (PROSITE_PROFILES); IPR011009 (SUPERFAMILY); IPR003961 (SUPERFAMILY)
774	420467_length_725_cvg_4.7_tip_1_3	14	adenosine monophosphate-protein transferase ficd homolog	242	1.98E-120	84.60%	0.103	IPR003812 (G3DSA:1.10.3290.GENE3D); IPR003812 (PFAM); PTHR13504:SF12 (PANTHER); PTHR13504 (PANTHER); IPR003812 (PROSITE_PROFILES); IPR003812 (SUPERFAMILY)
775	420561_length_725_cvg_63.3_tip_0_0	1327	gamma-glutamyltranspeptidase 1-like isoform x2	242	5.09E-64	70.70%	0.107	IPR000101 (PRINTS); IPR000101 (PFAM); IPR000101 (PANTHER); PTHR11686:SF15 (PANTHER); IPR029055 (SUPERFAMILY)
776	420991_length_728_cvg_9.2_tip_1_4	26	reticulon nogo	243	5.95E-60	64.80%	0.720 Y	IPR003591 (SMART); G3DSA:3.80.10.10 (GENE3D); IPR001611 (PFAM); IPR001611 (PFAM); PTHR24367 (PANTHER); SIGNAL_PEPTIDE_H_REGION (PHOBIUS); SIGNAL_PEPTIDE_C_REGION (PHOBIUS); NON_CYTOPLASMIC_DOMAIN (PHOBIUS); SIGNAL_PEPTIDE (PHOBIUS); SIGNAL_PEPTIDE_N_REGION (PHOBIUS); SignalP-noTM (SIGNALP_GRAM_NEGATIVE); SignalP-noTM (SIGNALP_EUK); SignalP-TM (SIGNALP_GRAM_POSITIVE); SSF52058 (SUPERFAMILY)
778	421299_length_729_cvg_2.0_tip_1_2	7	melanotransferrin	243	2.12E-66	77.40%	0.105	IPR001156 (PRINTS); IPR001156 (SMART); G3DSA:3.40.190.10 (GENE3D); IPR001156 (PFAM); G3DSA:3.40.190.10 (GENE3D); PTHR11485 (PANTHER); PTHR11485:SF18 (PANTHER); CYTOPLASMIC_DOMAIN (PHOBIUS); TRANSMEMBRANE (PHOBIUS); NON_CYTOPLASMIC_DOMAIN (PHOBIUS); IPR001156 (PROSITE_PROFILES); SSF53850 (SUPERFAMILY)
779	421389_length_729_cvg_6.4_tip_1_0	15	zinc finger protein	243	1.01E-35	54.80%	0.103	IPR015880 (SMART); IPR013087 (G3DSA:3.30.160.GENE3D); PF13894 (PFAM); PF13465 (PFAM); IPR013087 (G3DSA:3.30.160.GENE3D); IPR007087 (PFAM); PTHR24409 (PANTHER); PTHR24409:SF14 (PANTHER); IPR007087 (PROSITE_PATTERNS); IPR007087 (PROSITE_PATTERNS); IPR007087 (PROSITE_PATTERNS); IPR007087 (PROSITE_PATTERNS); IPR007087 (PROSITE_PATTERNS); IPR007087 (PROSITE_PATTERNS); IPR007087 (PROSITE_PROFILES); IPR007087 (PROSITE_PROFILES); IPR007087 (PROSITE_PROFILES); IPR007087 (PROSITE_PROFILES); IPR007087 (PROSITE_PROFILES); IPR007087 (PROSITE_PROFILES); SSF57667 (SUPERFAMILY); SSF57667 (SUPERFAMILY); SSF57667 (SUPERFAMILY); SSF57667 (SUPERFAMILY)
780	421481_length_730_cvg_2.0_tip_1_1	6	protein phosphatase 1 regulatory subunit 42	243	1.42E-52	70.40%	0.123	SM00365 (SMART); IPR025875 (PFAM); G3DSA:3.80.10.10 (GENE3D); IPR001611 (PFAM); PTHR10588:SF227 (PANTHER); PTHR10588 (PANTHER); IPR001611 (PROSITE_PROFILES); IPR001611 (PROSITE_PROFILES); IPR001611 (PROSITE_PROFILES); IPR001611 (PROSITE_PROFILES); IPR001611 (PROSITE_PROFILES); IPR001611 (PROSITE_PROFILES); SSF52058 (SUPERFAMILY)
810	426059_length_753_cvg_12.7_tip_1_0	43	adrenodoxin-like mitochondrial	243	8.15E-73	86.90%	0.161	IPR001055 (PRINTS); IPR001041 (PFAM); IPR012675 (G3DSA:3.10.20.GENE3D); PTHR23426 (PANTHER); PTHR23426:SF1 (PANTHER); IPR018298 (PROSITE_PATTERNS); IPR001041 (PROSITE_PROFILES); IPR001041 (SUPERFAMILY)
781	421845_length_732_cvg_3.2_tip_1_2	25	soma ferritin-like	244	1.25E-74	80.70%	0.101	IPR008331 (PFAM); IPR012347 (G3DSA:1.20.1260.GENE3D); IPR001519 (PANTHER); IPR009040 (PROSITE_PROFILES); IPR009078 (SUPERFAMILY)
782	421881_length_732_cvg_2.0_tip_1_1	3	protein twisted gastrulation	244	6.14E-63	75.10%	0.102	IPR006761 (PFAM); IPR006761 (PANTHER); PTHR12312:SF15 (PANTHER)
808	425791_length_752_cvg_8.0_tip_1_0	26	tachykinins isoform x2	244	7.60E-13	58.50%	0.099	TRANSMEMBRANE (PHOBIUS); CYTOPLASMIC_DOMAIN (PHOBIUS); NON_CYTOPLASMIC_DOMAIN (PHOBIUS); TMhelix (TMHMM)

783	422189_length_734_cvg_3.6_tip_1_1	17	transcription factor e4f1-like	245	4.47E-17	75.90%	0.103	IPR015880 (SMART); IPR013087 (G3DSA:3.30.160.GENE3D); PF13465 (PFAM); IPR013087 (G3DSA:3.30.160.GENE3D); PTHR24409 (PANTHER); IPR007087 (PROSITE_PATTERNS); IPR007087 (PROSITE_PATTERNS); IPR007087 (PROSITE_PROFILES); IPR007087 (PROSITE_PROFILES); SSF57667 (SUPERFAMILY)
784	422293_length_734_cvg_21.3_tip_1_0	69	gastrula zinc finger protein	245	1.25E-30	57.00%	0.105	IPR015880 (SMART); IPR013087 (G3DSA:3.30.160.GENE3D); IPR013087 (G3DSA:3.30.160.GENE3D); PF13465 (PFAM); PF13912 (PFAM); PTHR24409 (PANTHER); IPR007087 (PROSITE_PATTERNS); IPR007087 (PROSITE_PATTERNS); IPR007087 (PROSITE_PATTERNS); IPR007087 (PROSITE_PATTERNS); IPR007087 (PROSITE_PATTERNS); IPR007087 (PROSITE_PROFILES); IPR007087 (PROSITE_PROFILES); IPR007087 (PROSITE_PROFILES); IPR007087 (PROSITE_PROFILES); IPR007087 (PROSITE_PROFILES); SSF57667 (SUPERFAMILY); SSF57667 (SUPERFAMILY); SSF57667 (SUPERFAMILY)
786	422487_length_735_cvg_3.2_tip_1_4	14	down syndrome cell adhesion molecule-like protein dscam2	245	7.82E-20	63.90%	0.18	IPR003598 (SMART); IPR003599 (SMART); IPR013098 (PFAM); IPR013783 (G3DSA:2.60.40.GENE3D); PTHR10489 (PANTHER); PTHR10489:SF580 (PANTHER); NON_CYTOPLASMIC_DOMAIN (PHOBIUS); CYTOPLASMIC_DOMAIN (PHOBIUS); TRANSMEMBRANE (PHOBIUS); IPR007110 (PROSITE_PROFILES); SSF48726 (SUPERFAMILY); TMhelix (TMHMM)
787	422715_length_737_cvg_2.0_tip_1_2	7	protein kinase c-binding protein nell1-like isoform x2	245	1.05E-58	61.80%	0.107	IPR001007 (SMART); G3DSA:2.10.70.10 (GENE3D); IPR001007 (PFAM); PTHR24042 (PANTHER); PTHR24042:SF4 (PANTHER); IPR001007 (PROSITE_PATTERNS); IPR001007 (PROSITE_PROFILES); IPR001007 (PROSITE_PROFILES); SSF57603 (SUPERFAMILY); SSF57603 (SUPERFAMILY)
788	422727_length_737_cvg_4.2_tip_1_3	9	serrate partial	246	3.80E-28	77.60%	0.285	IPR000742 (SMART); IPR001881 (SMART); G3DSA:2.10.25.10 (GENE3D); IPR000742 (PFAM); G3DSA:2.10.25.10 (GENE3D); PTHR24044 (PANTHER); PTHR24044:SF222 (PANTHER); IPR018097 (PROSITE_PATTERNS); IPR013032 (PROSITE_PATTERNS); IPR000152 (PROSITE_PATTERNS); IPR013032 (PROSITE_PATTERNS); IPR013032 (PROSITE_PATTERNS); IPR013032 (PROSITE_PATTERNS); IPR000152 (PROSITE_PATTERNS); IPR000742 (PROSITE_PROFILES); IPR000742 (PROSITE_PROFILES); SSF57196 (SUPERFAMILY); SSF57196 (SUPERFAMILY)
789	423171_length_739_cvg_4.7_tip_1_5	14	upf0364 protein c6orf211 homolog	246	3.21E-74	69.10%	0.197	IPR002791 (PFAM); PTHR12260 (PANTHER); PTHR12260:SF1 (PANTHER); CYTOPLASMIC_DOMAIN (PHOBIUS); NON_CYTOPLASMIC_DOMAIN (PHOBIUS); TRANSMEMBRANE (PHOBIUS); IPR002791 (SUPERFAMILY)
790	423527_length_740_cvg_39.3_tip_1_5	150	marginal zone b- and b1-cell-specific	246	1.21E-44	56.40%	0.18	PTHR15881:SF1 (PANTHER); PTHR15881 (PANTHER)
791	423649_length_741_cvg_5.2_tip_1_4	14	prostaglandin f synthase-like isoform x4	247	1.39E-77	72.90%	0.102	IPR020471 (PRINTS); IPR023210 (G3DSA:3.20.20.GENE3D); IPR023210 (PFAM); IPR020471 (PIRSF); PTHR11732:SF133 (PANTHER); IPR001395 (PANTHER); IPR018170 (PROSITE_PATTERNS); IPR023210 (SUPERFAMILY)
792	423767_length_742_cvg_4.1_tip_1_4	12	protein cbg17465	247	1.09E-18	56.50%	0.206	PTHR23022 (PANTHER); PTHR23022:SF64 (PANTHER)
793	423843_length_742_cvg_2.2_tip_1_0	7	---NA---	248			0.131	NON_CYTOPLASMIC_DOMAIN (PHOBIUS); CYTOPLASMIC_DOMAIN (PHOBIUS); TRANSMEMBRANE (PHOBIUS); TMhelix (TMHMM)
794	423893_length_742_cvg_2.5_tip_1_3	8	alpha-l- partial	248	2.71E-06	68.29%	0.137	no IPS match
795	424183_length_743_cvg_2.0_tip_1_4	6	secreted frizzled-related protein 5-like	248	7.98E-19	77.60%	0.148	IPR020067 (G3DSA:1.10.2000.GENE3D); IPR015526 (PANTHER); CYTOPLASMIC_DOMAIN (PHOBIUS); TRANSMEMBRANE (PHOBIUS); NON_CYTOPLASMIC_DOMAIN (PHOBIUS); IPR020067 (SUPERFAMILY); TMhelix (TMHMM)
796	424227_length_744_cvg_8.4_tip_1_0	17	zinc metalloprotease	248	2.46E-10	59.20%	0.205	G3DSA:1.10.390.10 (GENE3D); TMhelix (TMHMM)
797	424569_length_745_cvg_7.7_tip_1_5	27	von willebrand factor like 1	248	1.50E-09	43.10%	0.101	no IPS match
817	426883_length_758_cvg_35.2_tip_1_1	148	lysine--trna ligase isoform x2	248	4.01E-79	94.30%	0.111	G3DSA:3.30.930.10 (GENE3D); IPR004364 (PFAM); IPR018150 (PANTHER); PTHR22594:SF4 (PANTHER); TRANSMEMBRANE (PHOBIUS); NON_CYTOPLASMIC_DOMAIN (PHOBIUS); NON_CYTOPLASMIC_DOMAIN (PHOBIUS); CYTOPLASMIC_DOMAIN (PHOBIUS); TRANSMEMBRANE (PHOBIUS); IPR006195 (PROSITE_PROFILES); SSF55681 (SUPERFAMILY); TMhelix (TMHMM); TMhelix (TMHMM)
798	424657_length_746_cvg_2.0_tip_1_0	6	pdz domain-containing protein 2	249	7.80E-16	82.20%	0.099	IPR001478 (PFAM); IPR001478 (G3DSA:2.30.42.GENE3D); PTHR19964 (PANTHER); IPR001478 (PROSITE_PROFILES); IPR001478 (SUPERFAMILY)
799	424833_length_747_cvg_3.9_tip_1_2	18	collagen alpha-1 partial	249	5.34E-23	52.60%	0.101	IPR008160 (PFAM); PTHR24023:SF379 (PANTHER); PTHR24023 (PANTHER)
800	424833_length_747_cvg_3.9_tip_1_5	18	collagen-like protein	249	1.82E-21	44.30%	0.218	no IPS match
801	425089_length_748_cvg_3.7_tip_1_4	11	protein partial	249	1.54E-16	57.40%	0.111	no IPS match
802	425111_length_748_cvg_6.4_tip_1_1	19	zinc metalloproteinase nas-13	249	3.85E-31	60.90%	0.169	IPR001506 (PRINTS); IPR024079 (G3DSA:3.40.390.GENE3D); IPR001506 (PFAM); PTHR10127 (PANTHER); SSF55486 (SUPERFAMILY)
803	425141_length_748_cvg_61.9_tip_1_4	210	hypothetical protein BRAFLDRAFT_71031	249	1.20E-15	50.50%	0.106	NON_CYTOPLASMIC_DOMAIN (PHOBIUS); CYTOPLASMIC_DOMAIN (PHOBIUS); TRANSMEMBRANE (PHOBIUS); TMhelix (TMHMM)

804	425317_length_749_cvg_2.4_tip_1_3	12	pt repeat family protein	250	1.50E-12	65.30%	0.638 Y	SignalP-noTM (SIGNALP_EUK)
806	425493_length_750_cvg_2.5_tip_1_1	9	zinc finger protein gfi-1b-like isoform x2	250	6.21E-23	72.40%	0.101	IPR015880 (SMART); IPR013087 (G3DSA:3.30.160.GENE3D); IPR007087 (PFAM); IPR013087 (G3DSA:3.30.160.GENE3D); PTHR24409 (PANTHER); IPR007087 (PROSITE_PATTERNS); IPR007087 (PROSITE_PATTERNS); NON_CYTOPLASMIC_DOMAIN (PHOBIUS); CYTOPLASMIC_DOMAIN (PHOBIUS); TRANSMEMBRANE (PHOBIUS); IPR007087 (PROSITE_PROFILES); IPR007087 (PROSITE_PROFILES); SSF57667 (SUPERFAMILY); TMhelix (TMHMM); TMhelix (TMHMM)
807	425591_length_750_cvg_2.4_tip_1_5	6	hemocyanin subunit	250	2.61E-11	54.70%	0.11	IPR005204 (G3DSA:1.20.1370.GENE3D); IPR005204 (PFAM); IPR005204 (SUPERFAMILY)
809	425989_length_753_cvg_64.9_tip_1_5	544	protein twin sister of ft	251	2.28E-24	59.50%	0.142	IPR008914 (G3DSA:3.90.280.GENE3D); IPR008914 (PFAM); PTHR11362 (PANTHER); IPR001858 (PROSITE_PATTERNS); CYTOPLASMIC_DOMAIN (PHOBIUS); TRANSMEMBRANE (PHOBIUS); NON_CYTOPLASMIC_DOMAIN (PHOBIUS); IPR008914 (SUPERFAMILY)
811	426635_length_756_cvg_4.6_tip_1_1	17	epidermal growth factor-like protein 8-like	252	1.20E-21	61.70%	0.26	IPR011489 (PFAM); NON_CYTOPLASMIC_DOMAIN (PHOBIUS); TRANSMEMBRANE (PHOBIUS); TRANSMEMBRANE (PHOBIUS); CYTOPLASMIC_DOMAIN (PHOBIUS); NON_CYTOPLASMIC_DOMAIN (PHOBIUS); IPR011489 (PROSITE_PROFILES); TMhelix (TMHMM); TMhelix (TMHMM)
812	426683_length_757_cvg_7.7_tip_1_2	26	phospholipase a-2-activating	252	4.29E-100	75.20%	0.11	IPR020472 (PRINTS); IPR001680 (SMART); IPR001680 (PFAM); IPR015943 (G3DSA:2.130.10.GENE3D); PTHR19849 (PANTHER); PTHR19849:SF0 (PANTHER); IPR017986 (PROSITE_PROFILES); IPR001680 (PROSITE_PROFILES); IPR001680 (PROSITE_PROFILES); IPR001680 (PROSITE_PROFILES); IPR017986 (SUPERFAMILY)
813	426729_length_757_cvg_6.3_tip_1_1	22	retinol dehydrogenase 13-like	252	3.71E-99	76.60%	0.109	IPR002198 (PRINTS); IPR002347 (PRINTS); IPR016040 (G3DSA:3.40.50.GENE3D); IPR002198 (PFAM); PTHR24320:SF55 (PANTHER); PTHR24320 (PANTHER); NON_CYTOPLASMIC_DOMAIN (PHOBIUS); CYTOPLASMIC_DOMAIN (PHOBIUS); TRANSMEMBRANE (PHOBIUS); SSF51735 (SUPERFAMILY); TMhelix (TMHMM)
815	426751_length_757_cvg_2.0_tip_1_2	6	zinc finger protein partial	252	5.53E-67	75.90%	0.107	IPR015880 (SMART); IPR013087 (G3DSA:3.30.160.GENE3D); IPR013087 (G3DSA:3.30.160.GENE3D); PF13465 (PFAM); PTHR24409 (PANTHER); IPR007087 (PROSITE_PATTERNS); IPR007087 (PROSITE_PROFILES); IPR007087 (PROSITE_PROFILES); IPR007087 (PROSITE_PROFILES); SSF57667 (SUPERFAMILY); SSF57667 (SUPERFAMILY)
841	430955_length_781_cvg_12.4_tip_1_4	40	peptidyl-prolyl cis-trans isomerase-like 6	252	6.44E-18	61.70%	0.103	IPR029000 (G3DSA:2.40.100.GENE3D); IPR002130 (PFAM); PTHR11071:SF210 (PANTHER); IPR024936 (PANTHER); IPR002130 (PROSITE_PROFILES); IPR029000 (SUPERFAMILY)
816	426761_length_757_cvg_30.1_tip_1_3	63	gastrula zinc finger isoform x1	253	1.92E-35	61.30%	0.101	IPR015880 (SMART); IPR013087 (G3DSA:3.30.160.GENE3D); IPR013087 (G3DSA:3.30.160.GENE3D); IPR013087 (G3DSA:3.30.160.GENE3D); IPR013087 (G3DSA:3.30.160.GENE3D); PF13912 (PFAM); PF13465 (PFAM); PTHR24409:SF14 (PANTHER); PTHR24409 (PANTHER); IPR007087 (PROSITE_PATTERNS); IPR007087 (PROSITE_PATTERNS); IPR007087 (PROSITE_PATTERNS); IPR007087 (PROSITE_PATTERNS); IPR007087 (PROSITE_PATTERNS); IPR007087 (PROSITE_PROFILES); IPR007087 (PROSITE_PROFILES); IPR007087 (PROSITE_PROFILES); IPR007087 (PROSITE_PROFILES); IPR007087 (PROSITE_PROFILES); SSF57667 (SUPERFAMILY); SSF57667 (SUPERFAMILY); SSF57667 (SUPERFAMILY)
818	427391_length_760_cvg_9.5_tip_1_3	29	peroxidase isoform x2	254	8.58E-14	65.10%	0.104	IPR019791 (G3DSA:1.10.640.GENE3D); PTHR11475 (PANTHER); IPR019791 (PROSITE_PROFILES); IPR010255 (SUPERFAMILY)
819	427557_length_761_cvg_4.4_tip_1_0	15	heat shock protein 70 a1-like	254	4.19E-161	95.50%	0.1	IPR013126 (PRINTS); G3DSA:3.30.420.40 (GENE3D); IPR013126 (PFAM); IPR029047 (G3DSA:2.60.34.GENE3D); G3DSA:3.90.640.10 (GENE3D); PTHR19375 (PANTHER); PTHR19375:SF142 (PANTHER); IPR029047 (SUPERFAMILY); SSF53067 (SUPERFAMILY)
820	427827_length_763_cvg_9.6_tip_1_4	23	adp-ribosylation factor-like protein 5b	254	2.15E-60	97.10%	0.152	IPR006689 (PRINTS); IPR024156 (SMART); IPR005225 (TIGRFAM); IPR027417 (G3DSA:3.40.50.GENE3D); IPR006689 (PFAM); PTHR11711 (PANTHER); PTHR11711:SF146 (PANTHER); IPR024156 (PROSITE_PROFILES); IPR027417 (SUPERFAMILY)
821	427873_length_763_cvg_6.5_tip_1_5	17	cre-ptr-16 protein	254	5.49E-22	56.80%	0.246	PTHR23022 (PANTHER); CYTOPLASMIC_DOMAIN (PHOBIUS); NON_CYTOPLASMIC_DOMAIN (PHOBIUS); TRANSMEMBRANE (PHOBIUS)
822	428007_length_764_cvg_3.4_tip_1_0	11	serine protease easter	255	3.04E-50	59.80%	0.15	IPR001314 (PRINTS); IPR001254 (SMART); G3DSA:2.40.10.10 (GENE3D); IPR001254 (PFAM); G3DSA:2.40.10.10 (GENE3D); PTHR24268 (PANTHER); PTHR24268:SF85 (PANTHER); IPR018114 (PROSITE_PATTERNS); IPR018114 (PROSITE_PATTERNS); IPR001254 (PROSITE_PROFILES); IPR009003 (SUPERFAMILY)

823	428181_length_765_cvg_28.7_tip_1_3	111	l-xylulose reductase	255	5.35E-90	80.60%	0.12	IPR002198 (PRINTS); IPR002347 (PRINTS); PF13561 (PFAM); IPR016040 (G3DSA:3.40.50.GENE3D); PTHR24311:SF6 (PANTHER); PTHR24311 (PANTHER); IPR020904 (PROSITE_PATTERNS); SSF51735 (SUPERFAMILY)
824	428377_length_766_cvg_14.2_tip_1_1	39	prenylcysteine oxidase-like	255	2.88E-61	64.20%	0.211	PF13450 (PFAM); IPR010795 (PFAM); G3DSA:3.50.50.60 (GENE3D); IPR017046 (PANTHER); SSF51905 (SUPERFAMILY)
825	428525_length_767_cvg_41.4_tip_1_0	95	suppressor of tumorigenicity 14 protein	256	5.71E-07	64.86%	0.155	IPR001254 (PFAM); G3DSA:2.40.10.10 (GENE3D); PTHR24256 (PANTHER); CYTOPLASMIC_DOMAIN (PHOBIUS); CYTOPLASMIC_DOMAIN (PHOBIUS); TRANSMEMBRANE (PHOBIUS); NON_CYTOPLASMIC_DOMAIN (PHOBIUS); TRANSMEMBRANE (PHOBIUS); IPR009003 (SUPERFAMILY); TMhelix (TMHMM); TMhelix (TMHMM)
826	428585_length_767_cvg_2.0_tip_1_1	9	penicillin-binding protein partial	256	0	99.40%	0.144	IPR012338 (G3DSA:3.40.710.GENE3D); IPR012340 (G3DSA:2.40.50.GENE3D); IPR001460 (PFAM); PTHR32282:SF5 (PANTHER); PTHR32282 (PANTHER); IPR012338 (SUPERFAMILY)
828	428969_length_769_cvg_4.8_tip_1_1	22	peptidyl-prolyl cis-trans	256	3.58E-57	71.20%	0.098	IPR029000 (G3DSA:2.40.100.GENE3D); IPR002130 (PFAM); IPR024936 (PANTHER); PTHR11071:SF193 (PANTHER); TRANSMEMBRANE (PHOBIUS); NON_CYTOPLASMIC_DOMAIN (PHOBIUS); CYTOPLASMIC_DOMAIN (PHOBIUS); IPR002130 (PROSITE_PROFILES); IPR029000 (SUPERFAMILY)
827	428943_length_769_cvg_9.5_tip_1_0	35	niemann-pick type c1 domain-containing	257	6.40E-58	72.50%	0.114	Coil (COILS); Coil (COILS); PTHR19237:SF20 (PANTHER); PTHR19237 (PANTHER); NON_CYTOPLASMIC_DOMAIN (PHOBIUS); TRANSMEMBRANE (PHOBIUS); CYTOPLASMIC_DOMAIN (PHOBIUS); TMhelix (TMHMM)
829	429347_length_772_cvg_6.0_tip_1_1	15	carbohydrate sulfotransferase 11-like	257	1.68E-43	68.50%	0.098	IPR005331 (PFAM); PTHR12137:SF29 (PANTHER); IPR018011 (PANTHER); TMhelix (TMHMM); TMhelix (TMHMM)
830	429715_length_774_cvg_6.7_tip_1_0	18	neurofilament medium polypeptide	258	8.48E-13	54.20%	0.096	no IPS match
831	429789_length_774_cvg_3.1_tip_1_0	11	mam and ldl-receptor class a domain-containing protein 2-like	258	8.70E-41	60.50%	0.149	IPR000998 (SMART); IPR000998 (PFAM); PTHR23282 (PANTHER); IPR000998 (PROSITE_PATTERNS); IPR000998 (PROSITE_PROFILES); IPR000998 (PROSITE_PROFILES); IPR013320 (SUPERFAMILY)
832	429789_length_774_cvg_3.1_tip_1_2	11	mlrp2_acrmi ame: full=mam and ldl-receptor class a domain-containing protein 2 ame: full=skeletal organic matrix mam and ldl-receptor 2 short=som mam and ldl-receptor partial	258	1.40E-07	55.86%	0.107	IPR000998 (PFAM); PTHR23282 (PANTHER); PTHR23282:SF75 (PANTHER); IPR000998 (PROSITE_PROFILES); IPR013320 (SUPERFAMILY)
833	429855_length_775_cvg_12.4_tip_1_5	34	esterase fe4	258	6.22E-91	70.60%	0.547 Y	IPR029058 (G3DSA:3.40.50.GENE3D); IPR002018 (PFAM); PTHR11559 (PANTHER); IPR019826 (PROSITE_PATTERNS); IPR019819 (PROSITE_PATTERNS); SignalP-noTM (SIGNALP_EUK); IPR029058 (SUPERFAMILY)
834	430241_length_777_cvg_3.5_tip_1_4	17	trafficking protein particle complex subunit 1	259	1.30E-62	86.10%	0.167	IPR007233 (PFAM); G3DSA:3.30.450.70 (GENE3D); PTHR23249:SF16 (PANTHER); IPR007233 (PANTHER); NON_CYTOPLASMIC_DOMAIN (PHOBIUS); TRANSMEMBRANE (PHOBIUS); CYTOPLASMIC_DOMAIN (PHOBIUS); IPR011012 (SUPERFAMILY); TMhelix (TMHMM)
835	430243_length_777_cvg_61.3_tip_1_0	817	superoxide dismutase	259	5.37E-78	85.20%	0.171	IPR001424 (PRINTS); IPR001424 (G3DSA:2.60.40.GENE3D); IPR001424 (PFAM); PTHR10003:SF33 (PANTHER); PTHR10003 (PANTHER); IPR018152 (PROSITE_PATTERNS); IPR018152 (PROSITE_PATTERNS); NON_CYTOPLASMIC_DOMAIN (PHOBIUS); TRANSMEMBRANE (PHOBIUS); CYTOPLASMIC_DOMAIN (PHOBIUS); IPR001424 (SUPERFAMILY); TMhelix (TMHMM)
836	430251_length_777_cvg_76.7_tip_0_1	2443	annexin b10-like	259	1.14E-83	69.70%	0.104	IPR001464 (PRINTS); IPR018502 (SMART); IPR018502 (G3DSA:1.10.220.GENE3D); IPR018502 (PFAM); IPR018502 (G3DSA:1.10.220.GENE3D); IPR018502 (G3DSA:1.10.220.GENE3D); PTHR10502 (PANTHER); IPR018252 (PROSITE_PATTERNS); SSF47874 (SUPERFAMILY)
837	430289_length_777_cvg_6.7_tip_1_3	22	gamma-glutamyl hydrolase-like	259	1.84E-57	68.00%	0.114	IPR011697 (PFAM); IPR029062 (G3DSA:3.40.50.GENE3D); IPR015527 (PANTHER); PTHR11315:SF2 (PANTHER); TRANSMEMBRANE (PHOBIUS); NON_CYTOPLASMIC_DOMAIN (PHOBIUS); CYTOPLASMIC_DOMAIN (PHOBIUS); IPR015527 (PROSITE_PROFILES); IPR029062 (SUPERFAMILY); TMhelix (TMHMM)
838	430315_length_777_cvg_102.3_tip_1_3	225214	secreted salivary gland	259	7.88E-34	54.40%	0.104	IPR001314 (PRINTS); IPR001254 (SMART); G3DSA:2.40.10.10 (GENE3D); G3DSA:2.40.10.10 (GENE3D); IPR001254 (PFAM); PTHR24265 (PANTHER); IPR018114 (PROSITE_PATTERNS); IPR001254 (PROSITE_PROFILES); IPR009003 (SUPERFAMILY)

839	430805_length_780_cvg_2.0_tip_1_2	9	vitellogenin receptor	260	6.85E-50	56.00%	0.12	IPR002172 (PRINTS); IPR001881 (SMART); IPR000742 (SMART); IPR002172 (SMART); G3DSA:2.10.25.10 (GENE3D); G3DSA:2.10.25.10 (GENE3D); IPR002172 (G3DSA:4.10.400.GENE3D); IPR002172 (PFAM); IPR002172 (G3DSA:4.10.400.GENE3D); IPR001881 (PFAM); PTHR10529 (PANTHER); PTHR10529:SF204 (PANTHER); IPR023415 (PROSITE_PATTERNS); IPR013032 (PROSITE_PATTERNS); IPR023415 (PROSITE_PATTERNS); IPR023415 (PROSITE_PATTERNS); IPR018097 (PROSITE_PATTERNS); IPR013032 (PROSITE_PATTERNS); IPR000152 (PROSITE_PATTERNS); IPR002172 (PROSITE_PROFILES); IPR002172 (PROSITE_PROFILES); IPR002172 (PROSITE_PROFILES); IPR002172 (SUPERFAMILY); IPR002172 (SUPERFAMILY); SSF57196 (SUPERFAMILY); SSF57196 (SUPERFAMILY)
840	430951_length_781_cvg_2.6_tip_1_4	11	hypothetical protein Y032_0322g2447	260	2.00E-24	52.50%	0.185	PTHR23022:SF64 (PANTHER); PTHR23022 (PANTHER)
842	431395_length_784_cvg_3.0_tip_1_1	9	low quality protein: zinc finger protein 233	261	2.79E-67	67.00%	0.116	IPR015880 (SMART); IPR007087 (PFAM); IPR013087 (G3DSA:3.30.160.GENE3D); IPR013087 (G3DSA:3.30.160.GENE3D); IPR013087 (G3DSA:3.30.160.GENE3D); IPR013087 (G3DSA:3.30.160.GENE3D); PF13465 (PFAM); PTHR24409 (PANTHER); IPR007087 (PROSITE_PATTERNS); IPR007087 (PROSITE_PATTERNS); IPR007087 (PROSITE_PATTERNS); IPR007087 (PROSITE_PATTERNS); IPR007087 (PROSITE_PATTERNS); IPR007087 (PROSITE_PATTERNS); IPR007087 (PROSITE_PATTERNS); IPR007087 (PROSITE_PATTERNS); IPR007087 (PROSITE_PROFILES); IPR007087 (PROSITE_PROFILES); IPR007087 (PROSITE_PROFILES); IPR007087 (PROSITE_PROFILES); IPR007087 (PROSITE_PROFILES); IPR007087 (PROSITE_PROFILES); IPR007087 (PROSITE_PROFILES); SSF57667 (SUPERFAMILY); SSF57667 (SUPERFAMILY); SSF57667 (SUPERFAMILY); SSF57667 (SUPERFAMILY)
843	431465_length_784_cvg_2.7_tip_1_5	7	leukotriene a-4 hydrolase	261	2.16E-76	75.60%	0.096	IPR014782 (PRINTS); IPR014782 (PFAM); IPR001930 (PANTHER); PTHR11533:SF4 (PANTHER); SSF63737 (SUPERFAMILY)
844	431471_length_784_cvg_2.5_tip_1_4	7	zinc finger protein 699-like isoform x1	261	3.66E-38	59.00%	0.103	IPR015880 (SMART); IPR013087 (G3DSA:3.30.160.GENE3D); IPR007087 (PFAM); PF13894 (PFAM); IPR013087 (G3DSA:3.30.160.GENE3D); PF13465 (PFAM); IPR013087 (G3DSA:3.30.160.GENE3D); PTHR24409 (PANTHER); PTHR24409:SF13 (PANTHER); IPR007087 (PROSITE_PATTERNS); IPR007087 (PROSITE_PATTERNS); IPR007087 (PROSITE_PATTERNS); IPR007087 (PROSITE_PATTERNS); IPR007087 (PROSITE_PATTERNS); IPR007087 (PROSITE_PATTERNS); IPR007087 (PROSITE_PATTERNS); IPR007087 (PROSITE_PROFILES); IPR007087 (PROSITE_PROFILES); IPR007087 (PROSITE_PROFILES); IPR007087 (PROSITE_PROFILES); IPR007087 (PROSITE_PROFILES); IPR007087 (PROSITE_PROFILES); SSF57667 (SUPERFAMILY); SSF57667 (SUPERFAMILY); SSF57667 (SUPERFAMILY); SSF57667 (SUPERFAMILY)
854	435732_length_810_cvg_21.3_tip_1_0	73	trafficking protein particle complex subunit 1 isoform x1	262	7.57E-07	71.00%	0.231	IPR007233 (PFAM)
860	436234_length_814_cvg_21.4_tip_1_2	66	spondin-2 isoform x1	262	9.11E-13	75.30%	0.166	IPR009465 (PFAM); CYTOPLASMIC_DOMAIN (PHOBIUS); TRANSMEMBRANE (PHOBIUS); NON_CYTOPLASMIC_DOMAIN (PHOBIUS); IPR009465 (PROSITE_PROFILES); TMhelix (TMHMM)
845	432175_length_788_cvg_8.0_tip_1_3	26	---NA---	263			0.104	CYTOPLASMIC_DOMAIN (PHOBIUS); NON_CYTOPLASMIC_DOMAIN (PHOBIUS); TRANSMEMBRANE (PHOBIUS); TRANSMEMBRANE (PHOBIUS); CYTOPLASMIC_DOMAIN (PHOBIUS)
846	432275_length_789_cvg_3.3_tip_1_3	9	repetitive proline-rich cell wall protein 2-like isoform x2	263	3.77E-28	51.90%	0.137	TRANSMEMBRANE (PHOBIUS); NON_CYTOPLASMIC_DOMAIN (PHOBIUS); CYTOPLASMIC_DOMAIN (PHOBIUS); SSF141571 (SUPERFAMILY)
855	435846_length_811_cvg_11.9_tip_1_2	32	hemagglutinin	263	1.75E-27	57.00%	0.135	IPR001570 (PFAM); IPR007280 (PFAM); G3DSA:1.10.390.10 (GENE3D); CYTOPLASMIC_DOMAIN (PHOBIUS); TRANSMEMBRANE (PHOBIUS); NON_CYTOPLASMIC_DOMAIN (PHOBIUS); SSF55486 (SUPERFAMILY); TMhelix (TMHMM)
847	433549_length_796_cvg_62.9_tip_1_2	1381	protein disulfide-isomerase a3	265	1.76E-102	76.90%	0.354 Y	PR00421 (PRINTS); IPR012336 (G3DSA:3.40.30.GENE3D); IPR012336 (G3DSA:3.40.30.GENE3D); PF13848 (PFAM); IPR013766 (PFAM); IPR005788 (TIGRFAM); PTHR18929:SF74 (PANTHER); PTHR18929 (PANTHER); IPR017937 (PROSITE_PATTERNS); CYTOPLASMIC_DOMAIN (PHOBIUS); TRANSMEMBRANE (PHOBIUS); NON_CYTOPLASMIC_DOMAIN (PHOBIUS); IPR012336 (PROSITE_PROFILES); IPR012336 (SUPERFAMILY); IPR012336 (SUPERFAMILY)
848	433855_length_798_cvg_4.7_tip_1_5	19	neutral ceramidase	266	5.14E-62	65.80%	0.108	IPR006823 (PFAM); IPR006823 (PANTHER); PTHR12670:SF1 (PANTHER)
869	437874_length_825_cvg_72.1_tip_1_2	666	protein them6	266	1.41E-25	54.90%	0.149	IPR029069 (G3DSA:3.10.129.GENE3D); PF13279 (PFAM); PTHR12475 (PANTHER); IPR029069 (SUPERFAMILY); TMhelix (TMHMM)

849	434079_length_800_cvg_4.1_tip_1_1	14	proactivator polypeptide	267	1.56E-10	71.90%	0.119	IPR008139 (SMART); IPR008138 (PFAM); IPR011001 (G3DSA:1.10.225.GENE3D); PTHR11480:SF3 (PANTHER); PTHR11480 (PANTHER); CYTOPLASMIC_DOMAIN (PHOBIUS); TRANSMEMBRANE (PHOBIUS); NON_CYTOPLASMIC_DOMAIN (PHOBIUS); NON_CYTOPLASMIC_DOMAIN (PHOBIUS); TRANSMEMBRANE (PHOBIUS); IPR008139 (PROSITE_PROFILES); IPR011001 (SUPERFAMILY); TMhelix (TMHMM); TMhelix (TMHMM)
871	438794_length_831_cvg_61.8_tip_1_1	782	hemagglutinin amebocyte aggregation factor-like	267	5.86E-38	57.90%	0.229	PF14704 (PFAM); IPR026645 (PANTHER); NON_CYTOPLASMIC_DOMAIN (PHOBIUS); TRANSMEMBRANE (PHOBIUS); CYTOPLASMIC_DOMAIN (PHOBIUS); TMhelix (TMHMM)
896	441963_length_853_cvg_16.5_tip_1_4	58	epididymal secretory protein e1	267	3.03E-23	58.80%	0.133	IPR003172 (SMART); IPR003172 (PFAM); IPR003172 (G3DSA:2.60.40.GENE3D); PTHR11306 (PANTHER); PTHR11306:SF3 (PANTHER); IPR014756 (SUPERFAMILY)
850	435057_length_806_cvg_2.5_tip_1_0	7	ligand of numb protein x 2	269	1.97E-41	67.40%	0.131	IPR001478 (SMART); IPR001478 (G3DSA:2.30.42.GENE3D); IPR001478 (PFAM); IPR001478 (G3DSA:2.30.42.GENE3D); PTHR19964 (PANTHER); IPR001478 (PROSITE_PROFILES); IPR001478 (PROSITE_PROFILES); IPR001478 (SUPERFAMILY); IPR001478 (SUPERFAMILY)
851	435088_length_806_cvg_11.4_tip_1_1	34	cysteine-rich motor neuron 1	269	3.23E-41	59.90%	0.141	IPR000867 (SMART); IPR000867 (PFAM); PTHR14186:SF8 (PANTHER); IPR011390 (PANTHER); CYTOPLASMIC_DOMAIN (PHOBIUS); TRANSMEMBRANE (PHOBIUS); CYTOPLASMIC_DOMAIN (PHOBIUS); NON_CYTOPLASMIC_DOMAIN (PHOBIUS); TRANSMEMBRANE (PHOBIUS); IPR000867 (PROSITE_PROFILES); IPR009030 (SUPERFAMILY); TMhelix (TMHMM); TMhelix (TMHMM)
852	435248_length_807_cvg_4.2_tip_1_1	15	low quality protein: zinc finger protein 233	269	1.56E-32	60.80%	0.099	IPR015880 (SMART); IPR013087 (G3DSA:3.30.160.GENE3D); IPR007087 (PFAM); IPR013087 (G3DSA:3.30.160.GENE3D); IPR013087 (G3DSA:3.30.160.GENE3D); IPR013087 (G3DSA:3.30.160.GENE3D); PF13465 (PFAM); PTHR24409 (PANTHER); IPR007087 (PROSITE_PATTERNS); IPR007087 (PROSITE_PATTERNS); IPR007087 (PROSITE_PATTERNS); IPR007087 (PROSITE_PATTERNS); IPR007087 (PROSITE_PATTERNS); IPR007087 (PROSITE_PATTERNS); IPR007087 (PROSITE_PROFILES); IPR007087 (PROSITE_PROFILES); IPR007087 (PROSITE_PROFILES); IPR007087 (PROSITE_PROFILES); IPR007087 (PROSITE_PROFILES); IPR007087 (PROSITE_PROFILES); SSF57667 (SUPERFAMILY); SSF57667 (SUPERFAMILY); SSF57667 (SUPERFAMILY); SSF57667 (SUPERFAMILY); TMhelix (TMHMM)
853	435330_length_808_cvg_2.0_tip_1_1	8	exostosin 1-like protein	269	2.96E-116	79.40%	0.113	IPR004263 (PFAM); PTHR11062 (PANTHER); PTHR11062:SF7 (PANTHER)
856	435974_length_812_cvg_5.4_tip_1_5	19	zinc finger protein 665-like	270	1.44E-58	62.60%	0.099	IPR015880 (SMART); IPR007087 (PFAM); IPR013087 (G3DSA:3.30.160.GENE3D); IPR013087 (G3DSA:3.30.160.GENE3D); IPR013087 (G3DSA:3.30.160.GENE3D); PF13465 (PFAM); PTHR24409 (PANTHER); IPR007087 (PROSITE_PATTERNS); IPR007087 (PROSITE_PATTERNS); IPR007087 (PROSITE_PATTERNS); IPR007087 (PROSITE_PATTERNS); IPR007087 (PROSITE_PATTERNS); IPR007087 (PROSITE_PROFILES); IPR007087 (PROSITE_PROFILES); IPR007087 (PROSITE_PROFILES); IPR007087 (PROSITE_PROFILES); IPR007087 (PROSITE_PROFILES); IPR007087 (PROSITE_PROFILES); IPR007087 (PROSITE_PROFILES); SSF57667 (SUPERFAMILY); SSF57667 (SUPERFAMILY); SSF57667 (SUPERFAMILY); SSF57667 (SUPERFAMILY)
878	439269_length_835_cvg_4.2_tip_1_4	16	developmental protein cactus	270	2.70E-25	70.70%	0.114	IPR002110 (SMART); IPR020683 (G3DSA:1.25.40.GENE3D); IPR020683 (PFAM); PTHR24139:SF26 (PANTHER); PTHR24139 (PANTHER); NON_CYTOPLASMIC_DOMAIN (PHOBIUS); TRANSMEMBRANE (PHOBIUS); CYTOPLASMIC_DOMAIN (PHOBIUS); IPR002110 (PROSITE_PROFILES); IPR002110 (PROSITE_PROFILES); IPR020683 (PROSITE_PROFILES); IPR020683 (SUPERFAMILY); TMhelix (TMHMM)
857	436060_length_813_cvg_2.6_tip_1_4	14	neprilysin 2 isoform x3	271	1.66E-104	72.00%	0.1	IPR018497 (PRINTS); IPR018497 (PFAM); IPR024079 (G3DSA:3.40.390.GENE3D); IPR000718 (PANTHER); PTHR11733:SF113 (PANTHER); SSF55486 (SUPERFAMILY)
858	436116_length_813_cvg_85.8_tip_0_3	2705088	isoform k	271	6.99E-42	48.30%	0.105	IPR002557 (SMART); IPR001223 (PFAM); IPR029070 (G3DSA:3.10.50.GENE3D); IPR013781 (G3DSA:3.20.20.GENE3D); IPR002557 (G3DSA:2.170.140.GENE3D); IPR002557 (PFAM); PTHR11177 (PANTHER); PTHR11177:SF133 (PANTHER); IPR002557 (PROSITE_PROFILES); IPR017853 (SUPERFAMILY); IPR029070 (SUPERFAMILY); IPR002557 (SUPERFAMILY)

859	436214_length_814_cvg_15.4_tip_1_1	56	moxd1 homolog 2- partial	271	1.84E-28	59.10%	0.882	Y	IPR005018 (SMART); IPR015920 (G3DSA:2.60.40.GENE3D); IPR005018 (PFAM); IPR000945 (PANTHER); PTHR10157:SF23 (PANTHER); SIGNAL_PEPTIDE_C_REGION (PHOBIUS); SIGNAL_PEPTIDE_N_REGION (PHOBIUS); NON_CYTOPLASMIC_DOMAIN (PHOBIUS); SIGNAL_PEPTIDE (PHOBIUS); SIGNAL_PEPTIDE_H_REGION (PHOBIUS); IPR005018 (PROSITE_PROFILES); SignalP-TM (SIGNALP_GRAM_POSITIVE); SignalP-noTM (SIGNALP_EUK); SignalP-noTM (SIGNALP_GRAM_NEGATIVE); SSF49344 (SUPERFAMILY)
881	440183_length_841_cvg_3.3_tip_1_1	14	hemocytin isoform x1	271	3.28E-14	47.00%	0.102		IPR006207 (PROSITE_PROFILES)
902	443531_length_864_cvg_73.1_tip_1_5	491	hemagglutinin amebocyte aggregation factor-like	271	4.52E-39	61.70%	0.318		PF14704 (PFAM); IPR026645 (PANTHER); TRANSMEMBRANE (PHOBIUS); NON_CYTOPLASMIC_DOMAIN (PHOBIUS); TRANSMEMBRANE (PHOBIUS); NON_CYTOPLASMIC_DOMAIN (PHOBIUS); CYTOPLASMIC_DOMAIN (PHOBIUS); TMhelix (TMHMM)
861	436516_length_816_cvg_6.6_tip_1_0	21	dbh-like monooxygenase protein 1-like protein	272	4.85E-15	67.20%	0.177		IPR005018 (PFAM); PTHR10157:SF23 (PANTHER); IPR000945 (PANTHER); TRANSMEMBRANE (PHOBIUS); NON_CYTOPLASMIC_DOMAIN (PHOBIUS); CYTOPLASMIC_DOMAIN (PHOBIUS); IPR005018 (PROSITE_PROFILES)
862	436770_length_817_cvg_2.4_tip_1_2	6	thioredoxin domain-containing protein 5	272	3.13E-95	70.90%	0.281		PR00421 (PRINTS); IPR012336 (G3DSA:3.40.30.GENE3D); IPR013766 (PFAM); IPR005788 (TIGRFAM); PTHR18929:SF52 (PANTHER); PTHR18929 (PANTHER); IPR017937 (PROSITE_PATTERNS); IPR017937 (PROSITE_PATTERNS); IPR012336 (PROSITE_PROFILES); IPR012336 (PROSITE_PROFILES); IPR012336 (SUPERFAMILY); IPR012336 (SUPERFAMILY)
864	436848_length_818_cvg_2.0_tip_1_5	7	fimbrial family protein	272	1.58E-128	99.80%	0.295		IPR000259 (PFAM); IPR000259 (G3DSA:2.60.40.GENE3D); NON_CYTOPLASMIC_DOMAIN (PHOBIUS); SIGNAL_PEPTIDE (PHOBIUS); SIGNAL_PEPTIDE_H_REGION (PHOBIUS); SIGNAL_PEPTIDE_N_REGION (PHOBIUS); SIGNAL_PEPTIDE_C_REGION (PHOBIUS); IPR008966 (SUPERFAMILY)
863	436822_length_818_cvg_7.2_tip_1_4	28	ankyrin repeat domain-containing protein 49-like	273	4.62E-61	68.80%	0.146		IPR002110 (PRINTS); IPR002110 (SMART); IPR020683 (G3DSA:1.25.40.GENE3D); IPR020683 (PFAM); PTHR24144 (PANTHER); IPR020683 (PROSITE_PROFILES); IPR002110 (PROSITE_PROFILES); IPR002110 (PROSITE_PROFILES); IPR002110 (PROSITE_PROFILES); IPR020683 (SUPERFAMILY)
865	437214_length_820_cvg_4.0_tip_1_4	12	lachesin isoform x1	273	4.76E-72	76.80%	0.112		IPR003598 (SMART); IPR013098 (PFAM); IPR013783 (G3DSA:2.60.40.GENE3D); PF13895 (PFAM); IPR013783 (G3DSA:2.60.40.GENE3D); PTHR19831:SF43 (PANTHER); PTHR19831 (PANTHER); CYTOPLASMIC_DOMAIN (PHOBIUS); CYTOPLASMIC_DOMAIN (PHOBIUS); NON_CYTOPLASMIC_DOMAIN (PHOBIUS); TRANSMEMBRANE (PHOBIUS); TRANSMEMBRANE (PHOBIUS); IPR007110 (PROSITE_PROFILES); SSF48726 (SUPERFAMILY); SSF48726 (SUPERFAMILY); TMhelix (TMHMM); TMhelix (TMHMM)
866	437600_length_823_cvg_65.8_tip_1_2	794	mitochondrial matrix protein p33	274	4.68E-75	67.90%	0.123		IPR003428 (PFAM); IPR003428 (G3DSA:3.10.280.GENE3D); PTHR10826 (PANTHER); PTHR10826:SF1 (PANTHER); IPR003428 (SUPERFAMILY)
867	437604_length_823_cvg_4.5_tip_1_5	17	protein 60a	274	9.14E-62	60.90%	0.1		IPR029034 (G3DSA:2.10.90.GENE3D); IPR001839 (PFAM); IPR001111 (PFAM); PTHR11848:SF54 (PANTHER); IPR015615 (PANTHER); IPR001839 (PROSITE_PROFILES); IPR029034 (SUPERFAMILY)
868	437722_length_824_cvg_5.7_tip_1_4	23	beta- -n-acetylgalactosaminyltransferase bre-4-like	275	1.01E-41	69.20%	0.123		IPR029044 (G3DSA:3.90.550.GENE3D); IPR027791 (PFAM); PTHR19300:SF30 (PANTHER); IPR003859 (PANTHER); IPR029044 (SUPERFAMILY)
870	438278_length_828_cvg_2.7_tip_1_5	12	otogelin-like protein	276	8.22E-10	47.50%	0.109		no IPS match
872	438810_length_832_cvg_7.1_tip_1_2	19	zinc finger protein 2 homolog	277	1.65E-154	76.80%	0.106		IPR015880 (SMART); PF13465 (PFAM); IPR013087 (G3DSA:3.30.160.GENE3D); IPR013087 (G3DSA:3.30.160.GENE3D); IPR007087 (PFAM); IPR013087 (G3DSA:3.30.160.GENE3D); PTHR24409 (PANTHER); IPR007087 (PROSITE_PATTERNS); IPR007087 (PROSITE_PATTERNS); IPR007087 (PROSITE_PATTERNS); IPR007087 (PROSITE_PATTERNS); IPR007087 (PROSITE_PATTERNS); IPR007087 (PROSITE_PROFILES); IPR007087 (PROSITE_PROFILES); IPR007087 (PROSITE_PROFILES); IPR007087 (PROSITE_PROFILES); IPR007087 (PROSITE_PROFILES); IPR007087 (PROSITE_PROFILES); IPR007087 (PROSITE_PROFILES); SSF57667 (SUPERFAMILY); SSF57667 (SUPERFAMILY); SSF57667 (SUPERFAMILY)
876	439087_length_833_cvg_2.9_tip_1_2	16	26s proteasome non-atpase regulatory subunit 10	277	4.14E-15	50.60%	0.103		IPR002110 (SMART); IPR020683 (PFAM); IPR020683 (G3DSA:1.25.40.GENE3D); IPR002110 (PFAM); PTHR24198 (PANTHER); IPR002110 (PROSITE_PROFILES); IPR002110 (PROSITE_PROFILES); IPR020683 (PROSITE_PROFILES); IPR002110 (PROSITE_PROFILES); IPR020683 (SUPERFAMILY)

873	438903_length_832_cvg_3.3_tip_1_3	13	krueppel-like factor 5	278	1.87E-46	96.20%	0.103	IPR015880 (SMART); PF13465 (PFAM); IPR013087 (G3DSA:3.30.160.GENE3D); IPR013087 (G3DSA:3.30.160.GENE3D); IPR013087 (G3DSA:3.30.160.GENE3D); PTHR23223 (PANTHER); IPR007087 (PROSITE_PATTERNS); IPR007087 (PROSITE_PATTERNS); IPR007087 (PROSITE_PROFILES); IPR007087 (PROSITE_PROFILES); IPR007087 (PROSITE_PROFILES); SSF57667 (SUPERFAMILY)
874	439057_length_833_cvg_6.3_tip_1_3	21	integral membrane protein 2c	278	1.20E-91	66.60%	0.14	IPR007084 (SMART); IPR007084 (PFAM); PTHR10962:SF1 (PANTHER); PTHR10962 (PANTHER); CYTOPLASMIC_DOMAIN (PHOBIUS); NON_CYTOPLASMIC_DOMAIN (PHOBIUS); TRANSMEMBRANE (PHOBIUS); IPR007084 (PROSITE_PROFILES); TMhelix (TMHMM)
875	439061_length_833_cvg_2.9_tip_1_1	11	neural cell adhesion molecule 1	278	1.63E-32	54.90%	0.477 Y	IPR003599 (SMART); IPR003598 (SMART); IPR013783 (G3DSA:2.60.40.GENE3D); PF13895 (PFAM); IPR013783 (G3DSA:2.60.40.GENE3D); IPR013098 (PFAM); PTHR19831:SF40 (PANTHER); PTHR19831 (PANTHER); IPR007110 (PROSITE_PROFILES); IPR007110 (PROSITE_PROFILES); SignalP-noTM (SIGNALP_EUK); SSF48726 (SUPERFAMILY); SSF48726 (SUPERFAMILY)
877	439197_length_834_cvg_2.6_tip_1_4	8	cathepsin partial	278	4.20E-30	83.60%	0.146	G3DSA:3.90.70.10 (GENE3D); IPR000668 (PFAM); IPR013128 (PANTHER); PTHR12411:SF289 (PANTHER); IPR025661 (PROSITE_PATTERNS); IPR025660 (PROSITE_PATTERNS); SSF54001 (SUPERFAMILY); TMhelix (TMHMM); TMhelix (TMHMM); TMhelix (TMHMM)
904	443595_length_865_cvg_48.1_tip_1_4	422	histone h2a-like	279	1.68E-79	96.80%	0.221	IPR002119 (PRINTS); IPR002119 (SMART); IPR009072 (G3DSA:1.10.20.GENE3D); IPR007125 (PFAM); PTHR23430:SF45 (PANTHER); PTHR23430 (PANTHER); IPR002119 (PROSITE_PATTERNS); TRANSMEMBRANE (PHOBIUS); CYTOPLASMIC_DOMAIN (PHOBIUS); NON_CYTOPLASMIC_DOMAIN (PHOBIUS); IPR009072 (SUPERFAMILY); TMhelix (TMHMM)
879	440019_length_840_cvg_2.9_tip_1_2	11	spondin-1	280	3.58E-94	68.50%	0.136	IPR002223 (PRINTS); IPR000884 (SMART); IPR002223 (SMART); IPR002223 (G3DSA:4.10.410.GENE3D); G3DSA:2.20.100.10 (GENE3D); G3DSA:2.20.100.10 (GENE3D); IPR000884 (PFAM); IPR002223 (PFAM); PTHR11311 (PANTHER); PTHR11311:SF11 (PANTHER); IPR020901 (PROSITE_PATTERNS); IPR002223 (PROSITE_PROFILES); IPR000884 (PROSITE_PROFILES); IPR000884 (PROSITE_PROFILES); IPR000884 (PROSITE_PROFILES); IPR002223 (SUPERFAMILY); IPR000884 (SUPERFAMILY); IPR000884 (SUPERFAMILY); IPR000884 (SUPERFAMILY)
880	440147_length_841_cvg_19.6_tip_1_3	75	Adrenodoxin, mitochondrial	281	2.89E-52	76.80%	0.148	IPR001055 (PRINTS); IPR012675 (G3DSA:3.10.20.GENE3D); IPR001041 (PFAM); PTHR23426:SF21 (PANTHER); PTHR23426 (PANTHER); IPR018298 (PROSITE_PATTERNS); IPR001041 (PROSITE_PROFILES); IPR001041 (SUPERFAMILY)
882	440359_length_842_cvg_11.8_tip_1_0	52	thioredoxin 2	281	1.56E-35	71.30%	0.284	PR00421 (PRINTS); IPR012336 (G3DSA:3.40.30.GENE3D); IPR013766 (PFAM); PTHR10438:SF235 (PANTHER); IPR005746 (PANTHER); TRANSMEMBRANE (PHOBIUS); NON_CYTOPLASMIC_DOMAIN (PHOBIUS); TRANSMEMBRANE (PHOBIUS); CYTOPLASMIC_DOMAIN (PHOBIUS); CYTOPLASMIC_DOMAIN (PHOBIUS); IPR012336 (PROSITE_PROFILES); IPR012336 (SUPERFAMILY); TMhelix (TMHMM); TMhelix (TMHMM)
883	440477_length_843_cvg_5.9_tip_1_2	18	zinc finger protein 226-like	281	6.58E-43	57.00%	0.099	IPR015880 (SMART); IPR013087 (G3DSA:3.30.160.GENE3D); IPR013087 (G3DSA:3.30.160.GENE3D); PF13465 (PFAM); IPR007087 (PFAM); PTHR24409 (PANTHER); IPR007087 (PROSITE_PATTERNS); IPR007087 (PROSITE_PATTERNS); IPR007087 (PROSITE_PATTERNS); IPR007087 (PROSITE_PATTERNS); IPR007087 (PROSITE_PATTERNS); IPR007087 (PROSITE_PATTERNS); IPR007087 (PROSITE_PROFILES); IPR007087 (PROSITE_PROFILES); IPR007087 (PROSITE_PROFILES); IPR007087 (PROSITE_PROFILES); IPR007087 (PROSITE_PROFILES); IPR007087 (PROSITE_PROFILES); IPR007087 (PROSITE_PROFILES); SSF57667 (SUPERFAMILY); SSF57667 (SUPERFAMILY); SSF57667 (SUPERFAMILY)
884	440519_length_843_cvg_59.8_tip_1_5	682	lipophorin receptor isoform c	281	3.94E-13	54.60%	0.476 Y	IPR002172 (PRINTS); IPR002172 (SMART); IPR002172 (G3DSA:4.10.400.GENE3D); IPR002172 (PFAM); IPR002172 (G3DSA:4.10.400.GENE3D); PTHR10529 (PANTHER); PTHR10529:SF208 (PANTHER); IPR023415 (PROSITE_PATTERNS); NON_CYTOPLASMIC_DOMAIN (PHOBIUS); TRANSMEMBRANE (PHOBIUS); CYTOPLASMIC_DOMAIN (PHOBIUS); IPR002172 (PROSITE_PROFILES); IPR002172 (PROSITE_PROFILES); IPR002172 (SUPERFAMILY); IPR002172 (SUPERFAMILY); TMhelix (TMHMM)

885	440573_length_844_cvg_6.8_tip_1_2	19	n-acetylglucosamine-6-sulfatase-like isoform x3	281	5.52E-98	71.90%	0.206	IPR017849 (G3DSA:3.40.720.GENE3D); IPR000917 (PFAM); PTHR10342:SF208 (PANTHER); PTHR10342 (PANTHER); IPR024607 (PROSITE_PATTERNS); NON_CYTOPLASMIC_DOMAIN (PHOBIUS); TRANSMEMBRANE (PHOBIUS); CYTOPLASMIC_DOMAIN (PHOBIUS); IPR017850 (SUPERFAMILY); TMhelix (TMHMM)
886	440625_length_844_cvg_4.9_tip_1_5	16	variable charge x-linked protein 3b	281	2.27E-10	58.10%	0.258	no IPS match
887	440641_length_844_cvg_36.4_tip_0_5	119	acyl- delta desaturase	281	7.07E-118	82.70%	0.108	IPR015876 (PRINTS); IPR005804 (PFAM); PTHR11351:SF28 (PANTHER); PTHR11351 (PANTHER); CYTOPLASMIC_DOMAIN (PHOBIUS); TRANSMEMBRANE (PHOBIUS); NON_CYTOPLASMIC_DOMAIN (PHOBIUS); NON_CYTOPLASMIC_DOMAIN (PHOBIUS); TRANSMEMBRANE (PHOBIUS); TRANSMEMBRANE (PHOBIUS); CYTOPLASMIC_DOMAIN (PHOBIUS); TMhelix (TMHMM); TMhelix (TMHMM); TMhelix (TMHMM)
888	440795_length_845_cvg_5.0_tip_1_5	18	cyclin-dependent kinase 6	281	3.82E-132	79.20%	0.096	IPR002290 (SMART); IPR000719 (PFAM); G3DSA:1.10.510.10 (GENE3D); G3DSA:3.30.200.20 (GENE3D); PTHR24056:SF180 (PANTHER); PTHR24056 (PANTHER); IPR008271 (PROSITE_PATTERNS); IPR017441 (PROSITE_PATTERNS); IPR000719 (PROSITE_PROFILES); IPR011009 (SUPERFAMILY)
905	443797_length_866_cvg_14.9_tip_1_2	61	otogelin-like protein	281	2.65E-09	44.10%	0.101	no IPS match
889	440897_length_846_cvg_4.1_tip_1_5	14	dehydrogenase reductase sdr family protein 7-like	282	1.09E-101	78.40%	0.293	IPR002347 (PRINTS); IPR002198 (PRINTS); IPR016040 (G3DSA:3.40.50.GENE3D); IPR002198 (PFAM); PTHR24322:SF325 (PANTHER); PTHR24322 (PANTHER); IPR020904 (PROSITE_PATTERNS); CYTOPLASMIC_DOMAIN (PHOBIUS); TRANSMEMBRANE (PHOBIUS); NON_CYTOPLASMIC_DOMAIN (PHOBIUS); SSF51735 (SUPERFAMILY); TMhelix (TMHMM)
890	441101_length_848_cvg_64.9_tip_0_0	226	zinc finger protein gfi-1b	283	2.38E-22	55.40%	0.104	IPR015880 (SMART); IPR007087 (PFAM); IPR013087 (G3DSA:3.30.160.GENE3D); IPR013087 (G3DSA:3.30.160.GENE3D); PF13465 (PFAM); PTHR24409:SF14 (PANTHER); PTHR24409 (PANTHER); IPR007087 (PROSITE_PATTERNS); IPR007087 (PROSITE_PATTERNS); IPR007087 (PROSITE_PATTERNS); IPR007087 (PROSITE_PROFILES); IPR007087 (PROSITE_PROFILES); IPR007087 (PROSITE_PROFILES); IPR007087 (PROSITE_PROFILES); SSF57667 (SUPERFAMILY); SSF57667 (SUPERFAMILY)
891	441257_length_849_cvg_4.0_tip_1_4	22	protein cbg17465	283	9.56E-07	55.33%	0.122	no IPS match
892	441267_length_849_cvg_4.5_tip_1_2	18	c4b-binding protein beta	283	1.31E-119	77.10%	0.11	IPR000436 (SMART); IPR000436 (PFAM); G3DSA:2.10.70.10 (GENE3D); G3DSA:2.10.70.10 (GENE3D); PTHR19325 (PANTHER); PTHR19325:SF312 (PANTHER); IPR000436 (PROSITE_PROFILES); IPR000436 (PROSITE_PROFILES); IPR000436 (PROSITE_PROFILES); IPR000436 (PROSITE_PROFILES); IPR000436 (SUPERFAMILY); IPR000436 (SUPERFAMILY); IPR000436 (SUPERFAMILY); IPR000436 (SUPERFAMILY)
893	441805_length_852_cvg_2.2_tip_1_5	13	serine partial	284	1.49E-36	63.10%	0.107	IPR001563 (PFAM); IPR029058 (G3DSA:3.40.50.GENE3D); IPR001563 (PANTHER); PTHR11802:SF30 (PANTHER); IPR029058 (SUPERFAMILY)
894	441891_length_853_cvg_2.0_tip_1_0	6	epsilon-sarcoglycan isoform x2	285	1.25E-60	65.50%	0.113	IPR001304 (PFAM); IPR008908 (PFAM); IPR016186 (G3DSA:3.10.100.GENE3D); IPR008908 (PANTHER); PTHR10132:SF14 (PANTHER); TRANSMEMBRANE (PHOBIUS); CYTOPLASMIC_DOMAIN (PHOBIUS); NON_CYTOPLASMIC_DOMAIN (PHOBIUS); IPR001304 (PROSITE_PROFILES); IPR016187 (SUPERFAMILY); TMhelix (TMHMM)
895	441955_length_853_cvg_3.8_tip_1_0	13	thioredoxin domain-containing protein 11	285	1.46E-38	54.80%	0.129	IPR012336 (G3DSA:3.40.30.GENE3D); IPR013766 (PFAM); PTHR18929:SF61 (PANTHER); PTHR18929 (PANTHER); IPR012336 (SUPERFAMILY); TMhelix (TMHMM)
897	442037_length_854_cvg_7.7_tip_1_0	19	chorion peroxidase-like	285	1.11E-101	68.50%	0.139	IPR019791 (PRINTS); IPR019791 (PFAM); IPR019791 (G3DSA:1.10.640.GENE3D); PTHR11475 (PANTHER); IPR019791 (PROSITE_PROFILES); IPR010255 (SUPERFAMILY)
898	442107_length_855_cvg_79.0_tip_0_2	212877	plasma kallikrein	285	4.96E-48	55.90%	0.124	IPR001314 (PRINTS); IPR001254 (SMART); IPR001254 (PFAM); G3DSA:2.40.10.10 (GENE3D); G3DSA:2.40.10.10 (GENE3D); PTHR24265 (PANTHER); IPR018114 (PROSITE_PATTERNS); IPR018114 (PROSITE_PATTERNS); IPR001254 (PROSITE_PROFILES); IPR009003 (SUPERFAMILY)
899	442159_length_855_cvg_4.3_tip_1_2	16	collagen alpha-1 chain-like	285	1.81E-87	81.60%	0.127	IPR000885 (SMART); IPR000885 (PFAM); PTHR24023 (PANTHER); PTHR24023:SF444 (PANTHER); TRANSMEMBRANE (PHOBIUS); NON_CYTOPLASMIC_DOMAIN (PHOBIUS); TRANSMEMBRANE (PHOBIUS); NON_CYTOPLASMIC_DOMAIN (PHOBIUS); CYTOPLASMIC_DOMAIN (PHOBIUS); CYTOPLASMIC_DOMAIN (PHOBIUS); TRANSMEMBRANE (PHOBIUS); IPR000885 (PRODOM); IPR000885 (PROSITE_PROFILES); TMhelix (TMHMM); TMhelix (TMHMM); TMhelix (TMHMM)
900	442625_length_858_cvg_8.1_tip_1_1	23	hypothetical protein L798_15262	286	4.89E-12	77.00%	0.334	IPR029245 (PFAM)

921	446473_length_886_cvg_62.8_tip_1_5	397	secreted metalloprotease mcp02	286	1.25E-09	48.20%	0.154	IPR025711 (PFAM); TRANSMEMBRANE (PHOBIUS); NON_CYTOPLASMIC_DOMAIN (PHOBIUS); CYTOPLASMIC_DOMAIN (PHOBIUS); TMhelix (TMHMM)
910	444735_length_873_cvg_3.7_tip_1_4	15	zinc finger protein	287	2.57E-16	51.90%	0.102	IPR015880 (SMART); PF13465 (PFAM); IPR013087 (G3DSA:3.30.160.GENE3D); IPR007087 (PFAM); IPR013087 (G3DSA:3.30.160.GENE3D); IPR013087 (G3DSA:3.30.160.GENE3D); PTHR24409 (PANTHER); PTHR24409:SF14 (PANTHER); IPR007087 (PROSITE_PATTERNS); IPR007087 (PROSITE_PATTERNS); IPR007087 (PROSITE_PROFILES); IPR007087 (PROSITE_PROFILES); IPR007087 (PROSITE_PROFILES); SSF57667 (SUPERFAMILY); SSF57667 (SUPERFAMILY)
919	446427_length_885_cvg_32.3_tip_1_0	132	collagen and calcium-binding egf domain-containing protein 1-like	287	6.59E-19	55.90%	0.103	IPR008160 (PFAM); PTHR24023 (PANTHER)
920	446427_length_885_cvg_32.3_tip_1_4	132	large adhesin	287	1.09E-10	41.25%	0.153	no IPS match
901	443253_length_863_cvg_7.6_tip_1_0	19	zinc finger protein 271-like	288	6.71E-56	64.90%	0.102	IPR015880 (SMART); PF13465 (PFAM); IPR013087 (G3DSA:3.30.160.GENE3D); IPR013087 (G3DSA:3.30.160.GENE3D); PF13912 (PFAM); IPR013087 (G3DSA:3.30.160.GENE3D); PTHR24409 (PANTHER); IPR007087 (PROSITE_PATTERNS); IPR007087 (PROSITE_PATTERNS); IPR007087 (PROSITE_PATTERNS); IPR007087 (PROSITE_PATTERNS); IPR007087 (PROSITE_PATTERNS); IPR007087 (PROSITE_PATTERNS); IPR007087 (PROSITE_PROFILES); IPR007087 (PROSITE_PROFILES); IPR007087 (PROSITE_PROFILES); IPR007087 (PROSITE_PROFILES); IPR007087 (PROSITE_PROFILES); IPR007087 (PROSITE_PROFILES); IPR007087 (PROSITE_PROFILES); SSF57667 (SUPERFAMILY); SSF57667 (SUPERFAMILY); SSF57667 (SUPERFAMILY)
903	443581_length_865_cvg_3.0_tip_1_4	9	balbiani ring protein 3	288	2.08E-18	49.90%	0.688 Y	IPR000072 (SMART); IPR004153 (PFAM); IPR029034 (G3DSA:2.10.90.GENE3D); IPR000072 (PFAM); PTHR11633 (PANTHER); PTHR11633:SF1 (PANTHER); NON_CYTOPLASMIC_DOMAIN (PHOBIUS); SIGNAL_PEPTIDE_N_REGION (PHOBIUS); TRANSMEMBRANE (PHOBIUS); SIGNAL_PEPTIDE_H_REGION (PHOBIUS); SIGNAL_PEPTIDE_C_REGION (PHOBIUS); SIGNAL_PEPTIDE (PHOBIUS); CYTOPLASMIC_DOMAIN (PHOBIUS); IPR000072 (PROSITE_PROFILES); SignalP-noTM (SIGNALP_EUK); IPR029034 (SUPERFAMILY); TMhelix (TMHMM)
906	444027_length_868_cvg_16.4_tip_1_5	67	pancreatic lipase-related protein 1	289	7.61E-42	52.10%	0.104	IPR029058 (G3DSA:3.40.50.GENE3D); IPR013818 (PFAM); PTHR11610:SF41 (PANTHER); IPR000734 (PANTHER); IPR029058 (SUPERFAMILY)
907	444051_length_868_cvg_16.7_tip_1_5	56	matrix metalloproteinase-15-like	289	4.92E-91	76.80%	0.109	IPR018487 (SMART); IPR000585 (G3DSA:2.110.10.GENE3D); IPR018487 (PFAM); PTHR10201:SF135 (PANTHER); PTHR10201 (PANTHER); NON_CYTOPLASMIC_DOMAIN (PHOBIUS); TRANSMEMBRANE (PHOBIUS); TRANSMEMBRANE (PHOBIUS); CYTOPLASMIC_DOMAIN (PHOBIUS); CYTOPLASMIC_DOMAIN (PHOBIUS); IPR018487 (PROSITE_PROFILES); IPR018487 (PROSITE_PROFILES); IPR018487 (PROSITE_PROFILES); IPR018487 (PROSITE_PROFILES); IPR000585 (SUPERFAMILY); TMhelix (TMHMM); TMhelix (TMHMM)
908	444123_length_868_cvg_3.0_tip_1_2	10	zinc finger protein 347 isoform x1	289	5.05E-61	61.40%	0.127	IPR015880 (SMART); IPR013087 (G3DSA:3.30.160.GENE3D); IPR013087 (G3DSA:3.30.160.GENE3D); IPR013087 (G3DSA:3.30.160.GENE3D); PF13465 (PFAM); PTHR24409 (PANTHER); IPR007087 (PROSITE_PATTERNS); IPR007087 (PROSITE_PATTERNS); IPR007087 (PROSITE_PATTERNS); IPR007087 (PROSITE_PATTERNS); IPR007087 (PROSITE_PATTERNS); IPR007087 (PROSITE_PATTERNS); IPR007087 (PROSITE_PATTERNS); IPR007087 (PROSITE_PATTERNS); CYTOPLASMIC_DOMAIN (PHOBIUS); NON_CYTOPLASMIC_DOMAIN (PHOBIUS); TRANSMEMBRANE (PHOBIUS); IPR007087 (PROSITE_PROFILES); IPR007087 (PROSITE_PROFILES); IPR007087 (PROSITE_PROFILES); IPR007087 (PROSITE_PROFILES); IPR007087 (PROSITE_PROFILES); IPR007087 (PROSITE_PROFILES); IPR007087 (PROSITE_PROFILES); IPR007087 (PROSITE_PROFILES); SSF57667 (SUPERFAMILY); SSF57667 (SUPERFAMILY); SSF57667 (SUPERFAMILY); SSF57667 (SUPERFAMILY); TMhelix (TMHMM)
929	447385_length_893_cvg_61.0_tip_1_3	781	secreted partial	289	7.98E-46	81.40%	0.107	IPR003585 (SMART); IPR027789 (PFAM); IPR001050 (PANTHER); PTHR10915:SF1 (PANTHER); IPR030479 (PROSITE_PATTERNS); NON_CYTOPLASMIC_DOMAIN (PHOBIUS); CYTOPLASMIC_DOMAIN (PHOBIUS); TRANSMEMBRANE (PHOBIUS); TMhelix (TMHMM)
930	447527_length_895_cvg_66.2_tip_1_5	629	projectin short variant	289	1.15E-32	54.30%	0.102	IPR003598 (SMART); IPR003599 (SMART); IPR013783 (G3DSA:2.60.40.GENE3D); IPR013783 (G3DSA:2.60.40.GENE3D); IPR013098 (PFAM); PTHR19897 (PANTHER); IPR007110 (PROSITE_PROFILES); IPR007110 (PROSITE_PROFILES); SSF48726 (SUPERFAMILY); SSF48726 (SUPERFAMILY)

909	444575_length_871_cvg_4.5_tip_1_2	21	calumenin	290	4.65E-14	85.10%	0.429 Y	IPR011992 (G3DSA:1.10.238.GENE3D); IPR018247 (PROSITE_PATTERNS); NON_CYTOPLASMIC_DOMAIN (PHOBIUS); TRANSMEMBRANE (PHOBIUS); NON_CYTOPLASMIC_DOMAIN (PHOBIUS); TRANSMEMBRANE (PHOBIUS); CYTOPLASMIC_DOMAIN (PHOBIUS); TRANSMEMBRANE (PHOBIUS); CYTOPLASMIC_DOMAIN (PHOBIUS); TMhelix (TMHMM)
911	444933_length_874_cvg_51.0_tip_1_3	145	adp-ribosylation factor-like protein 16	292	1.30E-41	67.80%	0.145	IPR006687 (SMART); IPR024156 (SMART); IPR027417 (G3DSA:3.40.50.GENE3D); IPR006689 (PFAM); PTHR11711 (PANTHER); PTHR11711:SF42 (PANTHER); IPR024156 (PROSITE_PROFILES); IPR027417 (SUPERFAMILY)
912	445613_length_879_cvg_4.8_tip_1_2	15	zinc finger protein	293	3.06E-42	54.50%	0.1	IPR015880 (SMART); IPR013087 (G3DSA:3.30.160.GENE3D); IPR013087 (G3DSA:3.30.160.GENE3D); PF13894 (PFAM); IPR007087 (PFAM); PF13465 (PFAM); IPR013087 (G3DSA:3.30.160.GENE3D); PTHR24409:SF14 (PANTHER); PTHR24409 (PANTHER); IPR007087 (PROSITE_PATTERNS); IPR007087 (PROSITE_PATTERNS); IPR007087 (PROSITE_PATTERNS); IPR007087 (PROSITE_PATTERNS); IPR007087 (PROSITE_PATTERNS); IPR007087 (PROSITE_PATTERNS); IPR007087 (PROSITE_PATTERNS); IPR007087 (PROSITE_PROFILES); IPR007087 (PROSITE_PROFILES); IPR007087 (PROSITE_PROFILES); IPR007087 (PROSITE_PROFILES); IPR007087 (PROSITE_PROFILES); IPR007087 (PROSITE_PROFILES); SSF57667 (SUPERFAMILY); SSF57667 (SUPERFAMILY); SSF57667 (SUPERFAMILY); SSF57667 (SUPERFAMILY)
913	445673_length_880_cvg_9.3_tip_1_2	39	hypothetical protein BRAFLDRAFT_125057	293	7.15E-09	46.00%	0.112	no IPS match
938	448897_length_906_cvg_9.8_tip_1_3	53	spermatogenesis-associated protein 5	293	4.49E-141	82.30%	0.104	Coil (COILS); IPR003593 (SMART); G3DSA:1.10.8.60 (GENE3D); IPR003959 (PFAM); IPR027417 (G3DSA:3.40.50.GENE3D); PTHR23077:SF27 (PANTHER); PTHR23077 (PANTHER); IPR003960 (PROSITE_PATTERNS); IPR027417 (SUPERFAMILY)
914	445711_length_880_cvg_56.5_tip_1_0	461	ecdysteroid-regulated 16 kda protein	294	1.42E-17	54.20%	0.393 Y	IPR003172 (SMART); IPR003172 (PFAM); IPR003172 (G3DSA:2.60.40.GENE3D); PTHR11306 (PANTHER); IPR014756 (SUPERFAMILY)
915	445811_length_881_cvg_6.8_tip_1_4	28	alcohol dehydrogenase	294	1.44E-58	65.90%	0.16	IPR020471 (PRINTS); IPR023210 (G3DSA:3.20.20.GENE3D); IPR023210 (PFAM); PTHR11732:SF183 (PANTHER); IPR001395 (PANTHER); IPR018170 (PROSITE_PATTERNS); IPR018170 (PROSITE_PATTERNS); TRANSMEMBRANE (PHOBIUS); CYTOPLASMIC_DOMAIN (PHOBIUS); NON_CYTOPLASMIC_DOMAIN (PHOBIUS); IPR023210 (SUPERFAMILY); TMhelix (TMHMM)
916	445989_length_882_cvg_17.7_tip_1_2	68	adp-ribosylation factor-like protein 2	294	4.78E-109	93.70%	0.551 Y	IPR006689 (PRINTS); IPR003579 (SMART); IPR006687 (SMART); IPR024156 (SMART); IPR005225 (TIGRFAM); IPR027417 (G3DSA:3.40.50.GENE3D); IPR006689 (PFAM); PTHR11711:SF26 (PANTHER); PTHR11711 (PANTHER); NON_CYTOPLASMIC_DOMAIN (PHOBIUS); TRANSMEMBRANE (PHOBIUS); CYTOPLASMIC_DOMAIN (PHOBIUS); IPR024156 (PROSITE_PROFILES); SignalP-TM (SIGNALP_EUK); IPR027417 (SUPERFAMILY); TMhelix (TMHMM)
936	448597_length_903_cvg_3.0_tip_1_1	14	striatin- partial	294	2.49E-140	88.60%	0.118	IPR001680 (SMART); IPR001680 (PFAM); IPR015943 (G3DSA:2.130.10.GENE3D); PTHR15653 (PANTHER); IPR019775 (PROSITE_PATTERNS); TRANSMEMBRANE (PHOBIUS); CYTOPLASMIC_DOMAIN (PHOBIUS); CYTOPLASMIC_DOMAIN (PHOBIUS); NON_CYTOPLASMIC_DOMAIN (PHOBIUS); TRANSMEMBRANE (PHOBIUS); IPR001680 (PROSITE_PROFILES); IPR017986 (PROSITE_PROFILES); IPR001680 (PROSITE_PROFILES); IPR017986 (SUPERFAMILY); TMhelix (TMHMM)
954	451101_length_924_cvg_63.3_tip_1_5	23025	translationally-controlled tumor protein	294	4.03E-71	79.40%	0.274	IPR018105 (PRINTS); IPR018105 (PFAM); IPR011323 (G3DSA:2.170.150.GENE3D); IPR018105 (PANTHER); IPR011057 (SUPERFAMILY)
917	446083_length_883_cvg_4.0_tip_1_3	14	lipophorin receptor	295	2.88E-28	71.80%	0.323	IPR002172 (PRINTS); IPR002172 (SMART); IPR002172 (PFAM); IPR002172 (G3DSA:4.10.400.GENE3D); PTHR10529 (PANTHER); IPR023415 (PROSITE_PATTERNS); IPR023415 (PROSITE_PATTERNS); IPR002172 (PROSITE_PROFILES); IPR002172 (PROSITE_PROFILES); IPR002172 (SUPERFAMILY); IPR002172 (SUPERFAMILY)
918	446161_length_883_cvg_4.3_tip_1_0	13	peptidyl-prolyl cis-trans isomerase-like 2	295	7.36E-141	79.50%	0.102	Coil (COILS); IPR002130 (PRINTS); IPR002130 (PFAM); IPR029000 (G3DSA:2.40.100.GENE3D); IPR024936 (PANTHER); PTHR11071:SF147 (PANTHER); IPR020892 (PROSITE_PATTERNS); IPR002130 (PROSITE_PROFILES); IPR029000 (SUPERFAMILY)
923	446629_length_887_cvg_7.0_tip_1_5	23	venom dipeptidyl peptidase 4 isoform x3	295	1.03E-26	71.40%	0.103	IPR029058 (G3DSA:3.40.50.GENE3D); IPR001375 (PFAM); PTHR11731:SF93 (PANTHER); PTHR11731 (PANTHER); CYTOPLASMIC_DOMAIN (PHOBIUS); TRANSMEMBRANE (PHOBIUS); NON_CYTOPLASMIC_DOMAIN (PHOBIUS); IPR029058 (SUPERFAMILY)
922	446575_length_886_cvg_5.2_tip_1_0	23	nucleobindin-2-like isoform x5	296	3.03E-54	74.10%	0.103	IPR011992 (PFAM); IPR011992 (G3DSA:1.10.238.GENE3D); PTHR19237 (PANTHER); PTHR19237:SF20 (PANTHER); IPR018247 (PROSITE_PATTERNS); IPR002048 (PROSITE_PROFILES); IPR002048 (PROSITE_PROFILES); SSF47473 (SUPERFAMILY)

924	446803_length_889_cvg_3.9_tip_1_4	19	immunoglobulin-like and fibronectin type iii domain containing 7	296	7.97E-18	55.90%	0.156	IPR026966 (PFAM); PTHR10489 (PANTHER); PTHR10489:SF553 (PANTHER); NON_CYTOPLASMIC_DOMAIN (PHOBIUS); CYTOPLASMIC_DOMAIN (PHOBIUS); CYTOPLASMIC_DOMAIN (PHOBIUS); TRANSMEMBRANE (PHOBIUS); TRANSMEMBRANE (PHOBIUS); TMhelix (TMHMM); TMhelix (TMHMM)
925	446805_length_889_cvg_16.9_tip_1_1	61	calnexin-like isoform x2	296	1.89E-91	78.50%	0.102	IPR001580 (PRINTS); IPR013320 (G3DSA:2.60.120.GENE3D); IPR001580 (PFAM); PTHR11073:SF1 (PANTHER); IPR001580 (PANTHER); IPR018124 (PROSITE_PATTERNS); IPR013320 (SUPERFAMILY)
927	446899_length_889_cvg_5.9_tip_1_2	32	tyrosine-protein kinase receptor tie-1	296	1.69E-18	57.90%	0.185	G3DSA:2.170.300.10 (GENE3D); PTHR24416:SF125 (PANTHER); PTHR24416 (PANTHER); CYTOPLASMIC_DOMAIN (PHOBIUS); TRANSMEMBRANE (PHOBIUS); NON_CYTOPLASMIC_DOMAIN (PHOBIUS); IPR003961 (SUPERFAMILY); TMhelix (TMHMM); TMhelix (TMHMM)
926	446897_length_889_cvg_18.1_tip_1_3	63	hemagglutinin amebocyte aggregation factor-like	297	1.29E-29	57.60%	0.269	PF14704 (PFAM); IPR026645 (PANTHER); TRANSMEMBRANE (PHOBIUS); TRANSMEMBRANE (PHOBIUS); NON_CYTOPLASMIC_DOMAIN (PHOBIUS); NON_CYTOPLASMIC_DOMAIN (PHOBIUS); CYTOPLASMIC_DOMAIN (PHOBIUS); CYTOPLASMIC_DOMAIN (PHOBIUS); TRANSMEMBRANE (PHOBIUS)
928	447037_length_890_cvg_4.7_tip_1_0	21	zinc finger protein 420-like	297	3.48E-41	56.80%	0.107	IPR015880 (SMART); IPR013087 (G3DSA:3.30.160.GENE3D); IPR013087 (G3DSA:3.30.160.GENE3D); IPR013087 (G3DSA:3.30.160.GENE3D); PF13465 (PFAM); PTHR24409:SF13 (PANTHER); PTHR24409 (PANTHER); IPR007087 (PROSITE_PATTERNS); IPR007087 (PROSITE_PATTERNS); IPR007087 (PROSITE_PATTERNS); IPR007087 (PROSITE_PATTERNS); IPR007087 (PROSITE_PATTERNS); IPR007087 (PROSITE_PATTERNS); IPR007087 (PROSITE_PATTERNS); IPR007087 (PROSITE_PROFILES); IPR007087 (PROSITE_PROFILES); IPR007087 (PROSITE_PROFILES); IPR007087 (PROSITE_PROFILES); IPR007087 (PROSITE_PROFILES); IPR007087 (PROSITE_PROFILES); IPR007087 (PROSITE_PROFILES); SSF57667 (SUPERFAMILY); SSF57667 (SUPERFAMILY); SSF57667 (SUPERFAMILY); SSF57667 (SUPERFAMILY)
931	447621_length_895_cvg_42.7_tip_1_1	131	protein shifted isoform x1	298	7.43E-81	87.70%	0.145	IPR013309 (PRINTS); IPR003306 (SMART); IPR003306 (PFAM); PTHR24838 (PANTHER); PTHR24838:SF276 (PANTHER); TRANSMEMBRANE (PHOBIUS); CYTOPLASMIC_DOMAIN (PHOBIUS); NON_CYTOPLASMIC_DOMAIN (PHOBIUS); IPR003306 (PRODOM); IPR003306 (PROSITE_PROFILES); TMhelix (TMHMM)
932	447653_length_895_cvg_59.9_tip_1_2	7269	plasma kallikrein	298	7.74E-38	54.10%	0.214	IPR001314 (PRINTS); IPR001254 (SMART); G3DSA:2.40.10.10 (GENE3D); G3DSA:2.40.10.10 (GENE3D); G3DSA:2.40.10.10 (GENE3D); IPR001254 (PFAM); PTHR24265 (PANTHER); IPR018114 (PROSITE_PATTERNS); IPR018114 (PROSITE_PATTERNS); TRANSMEMBRANE (PHOBIUS); NON_CYTOPLASMIC_DOMAIN (PHOBIUS); CYTOPLASMIC_DOMAIN (PHOBIUS); IPR001254 (PROSITE_PROFILES); IPR009003 (SUPERFAMILY)
933	447777_length_896_cvg_10.6_tip_1_5	36	stromal cell-derived factor 2	298	5.28E-95	75.50%	0.448 Y	IPR016093 (SMART); IPR016093 (PFAM); G3DSA:2.80.10.50 (GENE3D); IPR027005 (PANTHER); CYTOPLASMIC_DOMAIN (PHOBIUS); NON_CYTOPLASMIC_DOMAIN (PHOBIUS); TRANSMEMBRANE (PHOBIUS); IPR016093 (PROSITE_PROFILES); IPR016093 (PROSITE_PROFILES); IPR016093 (PROSITE_PROFILES); SignalP-TM (SIGNALP_GRAM_POSITIVE); IPR016093 (SUPERFAMILY)
949	450611_length_920_cvg_9.8_tip_1_2	36	nad h-hydrate epimerase-like	299	1.33E-109	77.00%	0.195	IPR004443 (G3DSA:3.40.50.GENE3D); IPR004443 (PFAM); IPR004443 (TIGRFAM); PTHR13232 (PANTHER); IPR004443 (PROSITE_PROFILES); IPR004443 (HAMAP); IPR004443 (SUPERFAMILY)
934	448147_length_899_cvg_5.0_tip_1_3	18	cathepsin l-associated protein	300	2.63E-62	59.40%	0.510 Y	IPR000782 (SMART); IPR000782 (G3DSA:2.30.180.GENE3D); IPR000782 (G3DSA:2.30.180.GENE3D); IPR000782 (PFAM); PTHR10900 (PANTHER); SIGNAL_PEPTIDE (PHOBIUS); SIGNAL_PEPTIDE_C_REGION (PHOBIUS); SIGNAL_PEPTIDE_H_REGION (PHOBIUS); NON_CYTOPLASMIC_DOMAIN (PHOBIUS); SIGNAL_PEPTIDE_N_REGION (PHOBIUS); IPR000782 (PROSITE_PROFILES); IPR000782 (PROSITE_PROFILES); SignalP-TM (SIGNALP_EUK); IPR000782 (SUPERFAMILY); IPR000782 (SUPERFAMILY); TMhelix (TMHMM)
935	448499_length_902_cvg_2.0_tip_1_4	7	glucose partial	301	0	100.00%	0.149	IPR018391 (SMART); IPR027295 (G3DSA:2.140.10.GENE3D); PF13360 (PFAM); IPR002372 (PFAM); PTHR32303 (PANTHER); PTHR32303:SF2 (PANTHER); IPR001479 (PROSITE_PATTERNS); IPR011047 (SUPERFAMILY)
937	448643_length_904_cvg_49.6_tip_1_0	191	serine protease inhibitor kazal-type 6-like isoform x1	302	4.25E-09	61.43%	0.614 Y	IPR002350 (SMART); G3DSA:3.30.60.30 (GENE3D); IPR002350 (PFAM); IPR002350 (PROSITE_PATTERNS); TRANSMEMBRANE (PHOBIUS); NON_CYTOPLASMIC_DOMAIN (PHOBIUS); TRANSMEMBRANE (PHOBIUS); CYTOPLASMIC_DOMAIN (PHOBIUS); CYTOPLASMIC_DOMAIN (PHOBIUS); NON_CYTOPLASMIC_DOMAIN (PHOBIUS); TRANSMEMBRANE (PHOBIUS); IPR002350 (PROSITE_PROFILES); SignalP-noTM (SIGNALP_EUK); SSF100895 (SUPERFAMILY); TMhelix (TMHMM); TMhelix (TMHMM)

939	449317_length_909_cvg_3.4_tip_1_2	19	serine proteinase	303	1.72E-40	50.70%	0.246	IPR001314 (PRINTS); IPR001254 (SMART); G3DSA:2.40.10.10 (GENE3D); G3DSA:2.40.10.10 (GENE3D); IPR001254 (PFAM); G3DSA:2.40.10.10 (GENE3D); PTHR24260 (PANTHER); IPR018114 (PROSITE_PATTERNS); NON_CYTOPLASMIC_DOMAIN (PHOBIUS); NON_CYTOPLASMIC_DOMAIN (PHOBIUS); TRANSMEMBRANE (PHOBIUS); CYTOPLASMIC_DOMAIN (PHOBIUS); TRANSMEMBRANE (PHOBIUS); IPR001254 (PROSITE_PROFILES); IPR009003 (SUPERFAMILY)
940	449371_length_910_cvg_2.6_tip_1_5	8	ecdysis triggering hormone receptor isoform a	303	8.02E-71	81.80%	0.1	IPR000276 (PRINTS); IPR000276 (PFAM); G3DSA:1.20.1070.10 (GENE3D); PTHR24243 (PANTHER); PTHR24243:SF102 (PANTHER); IPR000276 (PROSITE_PATTERNS); NON_CYTOPLASMIC_DOMAIN (PHOBIUS); NON_CYTOPLASMIC_DOMAIN (PHOBIUS); TRANSMEMBRANE (PHOBIUS); CYTOPLASMIC_DOMAIN (PHOBIUS); TRANSMEMBRANE (PHOBIUS); NON_CYTOPLASMIC_DOMAIN (PHOBIUS); TRANSMEMBRANE (PHOBIUS); CYTOPLASMIC_DOMAIN (PHOBIUS); TRANSMEMBRANE (PHOBIUS); IPR017452 (PROSITE_PROFILES); SSF81321 (SUPERFAMILY); TMhelix (TMHMM); TMhelix (TMHMM); TMhelix (TMHMM); TMhelix (TMHMM)
941	449375_length_910_cvg_3.0_tip_1_2	10	serine-aspartate repeat protein f	303	2.09E-18	42.80%	0.363 Y	no IPS match
943	449375_length_910_cvg_3.0_tip_1_5	10	collagen alpha-1 chain	303	3.84E-16	60.20%	0.114	IPR008160 (PFAM)
942	449375_length_910_cvg_3.0_tip_1_3	10	collagen alpha-1 chain	304	3.28E-20	58.60%	0.101	IPR008160 (PFAM); PTHR24023:SF372 (PANTHER); PTHR24023 (PANTHER)
944	449511_length_911_cvg_20.1_tip_1_0	77	zinc finger protein	304	4.50E-30	54.20%	0.113	IPR015880 (SMART); IPR013087 (G3DSA:3.30.160.GENE3D); IPR013087 (G3DSA:3.30.160.GENE3D); PF13465 (PFAM); PF13894 (PFAM); IPR007087 (PFAM); IPR013087 (G3DSA:3.30.160.GENE3D); PTHR24409 (PANTHER); PTHR24409:SF14 (PANTHER); IPR007087 (PROSITE_PATTERNS); IPR007087 (PROSITE_PATTERNS); IPR007087 (PROSITE_PATTERNS); IPR007087 (PROSITE_PATTERNS); IPR007087 (PROSITE_PATTERNS); IPR007087 (PROSITE_PROFILES); IPR007087 (PROSITE_PROFILES); IPR007087 (PROSITE_PROFILES); IPR007087 (PROSITE_PROFILES); IPR007087 (PROSITE_PROFILES); IPR007087 (PROSITE_PROFILES); SSF57667 (SUPERFAMILY); SSF57667 (SUPERFAMILY); SSF57667 (SUPERFAMILY)
945	449841_length_914_cvg_5.1_tip_1_1	25	carbonyl reductase	305	1.30E-123	74.60%	0.121	IPR002347 (PRINTS); IPR002198 (PFAM); IPR016040 (G3DSA:3.40.50.GENE3D); PTHR24322 (PANTHER); PTHR24322:SF58 (PANTHER); SSF51735 (SUPERFAMILY)
946	450099_length_916_cvg_64.1_tip_1_1	867	glutathione peroxidase 7	305	2.61E-75	71.00%	0.168	IPR000889 (PRINTS); IPR000889 (PFAM); IPR012336 (G3DSA:3.40.30.GENE3D); IPR000889 (PANTHER); PTHR11592:SF10 (PANTHER); IPR029759 (PROSITE_PATTERNS); IPR029760 (PROSITE_PATTERNS); CYTOPLASMIC_DOMAIN (PHOBIUS); NON_CYTOPLASMIC_DOMAIN (PHOBIUS); TRANSMEMBRANE (PHOBIUS); IPR000889 (PROSITE_PROFILES); IPR012336 (SUPERFAMILY); TMhelix (TMHMM)
947	450357_length_918_cvg_4.3_tip_1_2	16	retinol dehydrogenase 13-like	306	7.66E-32	74.60%	0.134	PTHR24320:SF55 (PANTHER); PTHR24320 (PANTHER); CYTOPLASMIC_DOMAIN (PHOBIUS); NON_CYTOPLASMIC_DOMAIN (PHOBIUS); TRANSMEMBRANE (PHOBIUS); TRANSMEMBRANE (PHOBIUS); NON_CYTOPLASMIC_DOMAIN (PHOBIUS); TMhelix (TMHMM); TMhelix (TMHMM)
948	450493_length_919_cvg_2.6_tip_1_1	10	zinc finger protein	306	3.51E-15	54.60%	0.108	IPR015880 (SMART); IPR013087 (G3DSA:3.30.160.GENE3D); PTHR24409:SF14 (PANTHER); PTHR24409 (PANTHER); IPR007087 (PROSITE_PATTERNS); IPR007087 (PROSITE_PATTERNS); IPR007087 (PROSITE_PATTERNS); IPR007087 (PROSITE_PROFILES); IPR007087 (PROSITE_PROFILES); IPR007087 (PROSITE_PROFILES); SSF57667 (SUPERFAMILY); SSF57667 (SUPERFAMILY)
950	450633_length_920_cvg_4.6_tip_1_0	25	carboxypeptidase q isoform x2	307	1.93E-93	74.80%	0.262	IPR003137 (PFAM); G3DSA:3.40.630.10 (GENE3D); PTHR12053 (PANTHER); TRANSMEMBRANE (PHOBIUS); NON_CYTOPLASMIC_DOMAIN (PHOBIUS); CYTOPLASMIC_DOMAIN (PHOBIUS); SSF53187 (SUPERFAMILY); TMhelix (TMHMM)
951	450693_length_921_cvg_11.6_tip_1_0	42	zinc finger protein 420-like	307	4.97E-18	44.30%	0.1	IPR015880 (SMART); IPR013087 (G3DSA:3.30.160.GENE3D); IPR013087 (G3DSA:3.30.160.GENE3D); PF13894 (PFAM); IPR007087 (PFAM); IPR013087 (G3DSA:3.30.160.GENE3D); PTHR24409 (PANTHER); PTHR24409:SF14 (PANTHER); IPR007087 (PROSITE_PATTERNS); IPR007087 (PROSITE_PATTERNS); IPR007087 (PROSITE_PATTERNS); IPR007087 (PROSITE_PROFILES); IPR007087 (PROSITE_PROFILES); IPR007087 (PROSITE_PROFILES); IPR007087 (PROSITE_PROFILES); IPR007087 (PROSITE_PROFILES); SSF57667 (SUPERFAMILY); SSF57667 (SUPERFAMILY); SSF57667 (SUPERFAMILY)

952	450895_length_922_cvg_62.0_tip_1_0	4149	thioredoxin peroxidase	308	5.19E-138	85.70%	0.269	IPR000866 (PFAM); IPR019479 (PFAM); IPR012336 (G3DSA:3.40.30.GENE3D); PTHR10681 (PANTHER); PTHR10681:SF101 (PANTHER); IPR012336 (PROSITE_PROFILES); IPR012336 (SUPERFAMILY); TMhelix (TMHMM); TMhelix (TMHMM)
953	450973_length_923_cvg_3.1_tip_1_1	11	zinc finger protein	308	6.11E-31	52.70%	0.1	IPR015880 (SMART); IPR007087 (PFAM); PF13894 (PFAM); IPR013087 (G3DSA:3.30.160.GENE3D); PF13465 (PFAM); IPR013087 (G3DSA:3.30.160.GENE3D); PTHR24375 (PANTHER); PTHR24375:SF106 (PANTHER); IPR007087 (PROSITE_PATTERNS); IPR007087 (PROSITE_PATTERNS); IPR007087 (PROSITE_PATTERNS); IPR007087 (PROSITE_PATTERNS); IPR007087 (PROSITE_PATTERNS); IPR007087 (PROSITE_PATTERNS); IPR007087 (PROSITE_PROFILES); IPR007087 (PROSITE_PROFILES); IPR007087 (PROSITE_PROFILES); IPR007087 (PROSITE_PROFILES); SSF57667 (SUPERFAMILY); SSF57667 (SUPERFAMILY); SSF57667 (SUPERFAMILY)
955	451379_length_926_cvg_25.2_tip_1_1	67	mite allergen lep d partial	309	5.14E-12	45.44%	0.195	PTHR11008:SF13 (PANTHER); PTHR11008 (PANTHER); IPR020234 (PRODOM)
956	451419_length_927_cvg_5.7_tip_1_2	21	extensin-like repeat protein	309	3.03E-12	58.10%	0.185	TRANSMEMBRANE (PHOBIUS); CYTOPLASMIC_DOMAIN (PHOBIUS); TRANSMEMBRANE (PHOBIUS); CYTOPLASMIC_DOMAIN (PHOBIUS); NON_CYTOPLASMIC_DOMAIN (PHOBIUS); TMhelix (TMHMM); TMhelix (TMHMM); TMhelix (TMHMM)
958	451699_length_929_cvg_4.1_tip_1_2	17	immunoglobulin g-binding protein partial	309	1.68E-14	80.00%	0.106	Coil (COILS); PTHR31750 (PANTHER)
977	454905_length_959_cvg_10.2_tip_1_5	41	mitochondrial import inner membrane translocase subunit tim8 a-like	309	4.25E-31	80.30%	0.229	IPR004217 (PFAM); IPR006966 (PFAM); IPR004217 (G3DSA:1.10.287.GENE3D); PTHR21535 (PANTHER); NON_CYTOPLASMIC_DOMAIN (PHOBIUS); TRANSMEMBRANE (PHOBIUS); NON_CYTOPLASMIC_DOMAIN (PHOBIUS); CYTOPLASMIC_DOMAIN (PHOBIUS); TRANSMEMBRANE (PHOBIUS); CYTOPLASMIC_DOMAIN (PHOBIUS); CYTOPLASMIC_DOMAIN (PHOBIUS); TRANSMEMBRANE (PHOBIUS); TRANSMEMBRANE (PHOBIUS); IPR004217 (SUPERFAMILY)
957	451699_length_929_cvg_4.1_tip_1_0	17	immunoglobulin g-binding protein partial	310	1.32E-10	81.20%	0.117	no IPS match
959	451753_length_929_cvg_15.6_tip_0_0	60	nicotinic acetylcholine receptor subunit alpha10	310	1.07E-64	54.80%	0.123	IPR006202 (PFAM); IPR006029 (PFAM); IPR006202 (G3DSA:2.70.170.GENE3D); IPR027361 (G3DSA:1.20.120.GENE3D); IPR006201 (PANTHER); PTHR18945:SF481 (PANTHER); TRANSMEMBRANE (PHOBIUS); TRANSMEMBRANE (PHOBIUS); CYTOPLASMIC_DOMAIN (PHOBIUS); NON_CYTOPLASMIC_DOMAIN (PHOBIUS); NON_CYTOPLASMIC_DOMAIN (PHOBIUS); TRANSMEMBRANE (PHOBIUS); NON_CYTOPLASMIC_DOMAIN (PHOBIUS); TRANSMEMBRANE (PHOBIUS); CYTOPLASMIC_DOMAIN (PHOBIUS); IPR006202 (SUPERFAMILY); IPR006029 (SUPERFAMILY); TMhelix (TMHMM); TMhelix (TMHMM); TMhelix (TMHMM); TMhelix (TMHMM)
960	452249_length_934_cvg_19.9_tip_1_1	99	pacifastin light chain	311	8.10E-14	53.50%	0.355 Y	IPR002223 (PRINTS); IPR002223 (SMART); IPR002223 (PFAM); IPR002223 (G3DSA:4.10.410.GENE3D); IPR008037 (PFAM); PTHR10083 (PANTHER); IPR020901 (PROSITE_PATTERNS); CYTOPLASMIC_DOMAIN (PHOBIUS); NON_CYTOPLASMIC_DOMAIN (PHOBIUS); CYTOPLASMIC_DOMAIN (PHOBIUS); TRANSMEMBRANE (PHOBIUS); TRANSMEMBRANE (PHOBIUS); TRANSMEMBRANE (PHOBIUS); NON_CYTOPLASMIC_DOMAIN (PHOBIUS); IPR020862 (PROSITE_PROFILES); IPR020862 (PROSITE_PROFILES); IPR020862 (PROSITE_PROFILES); IPR002223 (PROSITE_PROFILES); IPR008037 (SUPERFAMILY); IPR002223 (SUPERFAMILY); IPR008037 (SUPERFAMILY); IPR008037 (SUPERFAMILY); TMhelix (TMHMM); TMhelix (TMHMM); TMhelix (TMHMM)
961	452544_length_937_cvg_4.2_tip_1_5	15	beta-lactamase	312	0	99.70%	0.348 Y	IPR000871 (PRINTS); PF13354 (PFAM); IPR012338 (G3DSA:3.40.710.GENE3D); IPR023650 (PROSITE_PATTERNS); NON_CYTOPLASMIC_DOMAIN (PHOBIUS); SIGNAL_PEPTIDE_N_REGION (PHOBIUS); SIGNAL_PEPTIDE_C_REGION (PHOBIUS); SIGNAL_PEPTIDE_H_REGION (PHOBIUS); SIGNAL_PEPTIDE (PHOBIUS); SignalP-TM (SIGNALP_GRAM_POSITIVE); IPR012338 (SUPERFAMILY)
964	452924_length_941_cvg_10.9_tip_1_2	40	polypeptide n-acetylgalactosaminyltransferase 2	313	1.17E-12	51.00%	0.111	IPR000772 (SMART); IPR000772 (PFAM); G3DSA:2.80.10.50 (GENE3D); PTHR11675 (PANTHER); PTHR11675:SF24 (PANTHER); IPR000772 (PROSITE_PROFILES); IPR000772 (SUPERFAMILY)
962	452850_length_940_cvg_71.3_tip_1_0	115683	chitinase 2	314	5.01E-60	59.80%	0.133	IPR011583 (SMART); IPR029070 (G3DSA:3.10.50.GENE3D); IPR001223 (PFAM); IPR013781 (G3DSA:3.20.20.GENE3D); PTHR11177 (PANTHER); IPR029070 (SUPERFAMILY); IPR017853 (SUPERFAMILY)
963	452924_length_941_cvg_10.9_tip_1_1	40	polypeptide n-acetylgalactosaminyltransferase 14	314	2.65E-30	77.80%	0.102	PTHR11675 (PANTHER); NON_CYTOPLASMIC_DOMAIN (PHOBIUS); TRANSMEMBRANE (PHOBIUS); CYTOPLASMIC_DOMAIN (PHOBIUS); IPR029044 (SUPERFAMILY)

966	453228_length_943_cvg_5.4_tip_1_5	28	wnt1-inducible-signaling pathway protein 3	314	8.68E-15	50.30%	0.223		IPR000867 (SMART); IPR000867 (PFAM); PTHR11348 (PANTHER); IPR000867 (PROSITE_PROFILES); IPR009030 (SUPERFAMILY)
980	455475_length_964_cvg_12.4_tip_1_3	52	adiponectin receptor protein	314	3.97E-21	77.70%	0.101		IPR004254 (PFAM); PTHR20855:SF15 (PANTHER); IPR004254 (PANTHER); TRANSMEMBRANE (PHOBIUS); NON_CYTOPLASMIC_DOMAIN (PHOBIUS); TRANSMEMBRANE (PHOBIUS); CYTOPLASMIC_DOMAIN (PHOBIUS); CYTOPLASMIC_DOMAIN (PHOBIUS); TRANSMEMBRANE (PHOBIUS); NON_CYTOPLASMIC_DOMAIN (PHOBIUS); TRANSMEMBRANE (PHOBIUS); CYTOPLASMIC_DOMAIN (PHOBIUS); TMhelix (TMHMM); TMhelix (TMHMM); TMhelix (TMHMM); TMhelix (TMHMM)
965	453212_length_943_cvg_5.6_tip_1_3	32	low quality protein: titin-like	315	1.55E-177	87.90%	0.099		IPR003598 (SMART); IPR003599 (SMART); IPR013783 (G3DSA:2.60.40.GENE3D); IPR013783 (G3DSA:2.60.40.GENE3D); IPR010939 (PFAM); IPR013098 (PFAM); IPR013783 (G3DSA:2.60.40.GENE3D); PTHR19897:SF149 (PANTHER); PTHR19897 (PANTHER); IPR007110 (PROSITE_PROFILES); IPR007110 (PROSITE_PROFILES); SSF48726 (SUPERFAMILY); SSF48726 (SUPERFAMILY)
967	453368_length_944_cvg_7.0_tip_1_0	37	thioredoxin-dependent peroxide reductase	315	4.63E-127	84.70%	0.476	Y	IPR019479 (PFAM); IPR000866 (PFAM); IPR012336 (G3DSA:3.40.30.GENE3D); PTHR10681 (PANTHER); PTHR10681:SF101 (PANTHER); SIGNAL_PEPTIDE_C_REGION (PHOBIUS); SIGNAL_PEPTIDE_N_REGION (PHOBIUS); SIGNAL_PEPTIDE_H_REGION (PHOBIUS); SIGNAL_PEPTIDE (PHOBIUS); NON_CYTOPLASMIC_DOMAIN (PHOBIUS); IPR012336 (PROSITE_PROFILES); SignalP-noTM (SIGNALP_EUK); IPR012336 (SUPERFAMILY)
968	453592_length_946_cvg_17.0_tip_1_5	66	wd repeat domain 5	315	6.32E-53	86.40%	0.102		IPR001680 (SMART); IPR001680 (PFAM); IPR015943 (G3DSA:2.130.10.GENE3D); PTHR22847:SF308 (PANTHER); PTHR22847 (PANTHER); IPR019775 (PROSITE_PATTERNS); IPR001680 (PROSITE_PROFILES); IPR017986 (PROSITE_PROFILES); IPR001680 (PROSITE_PROFILES); IPR017986 (SUPERFAMILY)
969	453752_length_948_cvg_2.0_tip_1_3	10	tyrosine-protein kinase hopscotch	316	1.36E-93	66.50%	0.116		IPR001245 (PRINTS); IPR020635 (SMART); G3DSA:3.30.200.20 (GENE3D); G3DSA:1.10.510.10 (GENE3D); IPR016253 (PIRSF); IPR001245 (PFAM); PTHR24418 (PANTHER); IPR017441 (PROSITE_PATTERNS); IPR008266 (PROSITE_PATTERNS); IPR000719 (PROSITE_PROFILES); IPR011009 (SUPERFAMILY)
970	454160_length_951_cvg_5.2_tip_1_0	16	collagen triple helix repeat family protein	317	2.17E-11	52.90%	0.135		no IPS match
971	454160_length_951_cvg_5.2_tip_1_4	16	pneumococcal surface protein c	317	3.41E-10	48.90%	0.112		no IPS match
972	454174_length_951_cvg_40.2_tip_1_5	162	casein kinase ii subunit alpha	317	0	96.10%	0.098		IPR002290 (SMART); IPR000719 (PFAM); G3DSA:1.10.510.10 (GENE3D); G3DSA:3.30.200.20 (GENE3D); PTHR24054:SF28 (PANTHER); PTHR24054 (PANTHER); IPR017441 (PROSITE_PATTERNS); IPR008271 (PROSITE_PATTERNS); IPR000719 (PROSITE_PROFILES); IPR011009 (SUPERFAMILY)
973	454452_length_954_cvg_3.0_tip_1_5	19	collagen alpha 1 chain	318	9.92E-57	55.70%	0.125		IPR016186 (G3DSA:3.10.100.GENE3D); IPR010515 (PFAM); IPR026917 (PTHR24023:PANTHER); PTHR24023 (PANTHER); NON_CYTOPLASMIC_DOMAIN (PHOBIUS); CYTOPLASMIC_DOMAIN (PHOBIUS); TRANSMEMBRANE (PHOBIUS); IPR016187 (SUPERFAMILY); TMhelix (TMHMM)
974	454796_length_958_cvg_5.8_tip_1_5	23	tyrosine-protein phosphatase 69d-like	319	6.36E-140	80.00%	0.133		IPR000242 (PRINTS); IPR000242 (SMART); IPR003595 (SMART); IPR000242 (PFAM); IPR029021 (G3DSA:3.90.190.GENE3D); IPR029021 (G3DSA:3.90.190.GENE3D); PTHR19134:SF227 (PANTHER); PTHR19134 (PANTHER); IPR000242 (PROSITE_PROFILES); IPR000387 (PROSITE_PROFILES); IPR029021 (SUPERFAMILY)
975	454810_length_958_cvg_12.8_tip_1_4	77	stereocilin	319	5.83E-19	47.00%	0.1		IPR026061 (PTHR23412:PANTHER); IPR026664 (PANTHER); NON_CYTOPLASMIC_DOMAIN (PHOBIUS); TRANSMEMBRANE (PHOBIUS); CYTOPLASMIC_DOMAIN (PHOBIUS); TMhelix (TMHMM)
976	454903_length_959_cvg_4.2_tip_1_3	15	zinc finger protein	320	6.06E-45	57.40%	0.1		IPR015880 (SMART); IPR013087 (G3DSA:3.30.160.GENE3D); IPR007087 (PFAM); IPR013087 (G3DSA:3.30.160.GENE3D); IPR013087 (G3DSA:3.30.160.GENE3D); PF13894 (PFAM); PF13465 (PFAM); PTHR24409:SF14 (PANTHER); PTHR24409 (PANTHER); IPR007087 (PROSITE_PATTERNS); IPR007087 (PROSITE_PATTERNS); IPR007087 (PROSITE_PATTERNS); IPR007087 (PROSITE_PATTERNS); IPR007087 (PROSITE_PATTERNS); IPR007087 (PROSITE_PATTERNS); IPR007087 (PROSITE_PATTERNS); IPR007087 (PROSITE_PATTERNS); IPR007087 (PROSITE_PROFILES); IPR007087 (PROSITE_PROFILES); IPR007087 (PROSITE_PROFILES); IPR007087 (PROSITE_PROFILES); IPR007087 (PROSITE_PROFILES); IPR007087 (PROSITE_PROFILES); IPR007087 (PROSITE_PROFILES); IPR007087 (PROSITE_PROFILES); SSF57667 (SUPERFAMILY); SSF57667 (SUPERFAMILY); SSF57667 (SUPERFAMILY)

978	455123_length_961_cvg_6.8_tip_1_4	22	zinc finger protein 76	320	3.08E-89	67.60%	0.151		IPR015880 (SMART); IPR013087 (G3DSA:3.30.160.GENE3D); IPR013087 (G3DSA:3.30.160.GENE3D); PF13465 (PFAM); PTHR11389 (PANTHER); PTHR11389:SF448 (PANTHER); IPR007087 (PROSITE_PATTERNS); IPR007087 (PROSITE_PATTERNS); IPR007087 (PROSITE_PATTERNS); IPR007087 (PROSITE_PROFILES); IPR007087 (PROSITE_PROFILES); IPR007087 (PROSITE_PROFILES); SSF57667 (SUPERFAMILY); SSF57667 (SUPERFAMILY)
979	455197_length_961_cvg_10.9_tip_1_2	45	prenylcysteine oxidase-like	320	1.07E-61	57.20%	0.099		IPR010795 (PFAM); IPR017046 (PANTHER)
983	455595_length_965_cvg_4.0_tip_1_2	19	retinol dehydrogenase 13	321	2.41E-70	70.90%	0.114		IPR002198 (PRINTS); IPR002347 (PRINTS); IPR016040 (G3DSA:3.40.50.GENE3D); IPR002198 (PFAM); PTHR24320:SF55 (PANTHER); PTHR24320 (PANTHER); IPR020904 (PROSITE_PATTERNS); SSF51735 (SUPERFAMILY)
981	455539_length_965_cvg_4.0_tip_1_0	16	hypothetical protein BRAFLDRAFT_203178	322	2.71E-26	69.30%	0.099		IPR008979 (G3DSA:2.60.120.GENE3D); IPR006104 (PFAM); PTHR10066 (PANTHER); IPR028369 (PTHR10066:PANTHER); NON_CYTOPLASMIC_DOMAIN (PHOBIUS); TRANSMEMBRANE (PHOBIUS); CYTOPLASMIC_DOMAIN (PHOBIUS); CYTOPLASMIC_DOMAIN (PHOBIUS); TRANSMEMBRANE (PHOBIUS); TRANSMEMBRANE (PHOBIUS); NON_CYTOPLASMIC_DOMAIN (PHOBIUS); IPR008979 (SUPERFAMILY); TMhelix (TMHMM); TMhelix (TMHMM); TMhelix (TMHMM)
982	455559_length_965_cvg_2.7_tip_1_4	16	ser thr protein	322	1.46E-153	85.50%	0.129		IPR002290 (SMART); G3DSA:1.10.510.10 (GENE3D); G3DSA:3.30.200.20 (GENE3D); IPR000719 (PFAM); IPR020675 (PANTHER); IPR008271 (PROSITE_PATTERNS); IPR000719 (PROSITE_PROFILES); IPR011009 (SUPERFAMILY)
984	455709_length_966_cvg_2.0_tip_1_1	10	hypoxanthine partial	322	1.95E-147	100.00%	0.146		IPR008274 (G3DSA:3.30.365.GENE3D); IPR008274 (PFAM); IPR008274 (G3DSA:3.30.365.GENE3D); PTHR11908 (PANTHER); PTHR11908:SF64 (PANTHER); IPR008274 (SUPERFAMILY)
985	455769_length_967_cvg_3.2_tip_1_4	13	secreted salivary gland	322	9.03E-31	50.40%	0.107		IPR023307 (G3DSA:2.170.15.GENE3D); IPR004991 (PFAM); NON_CYTOPLASMIC_DOMAIN (PHOBIUS); CYTOPLASMIC_DOMAIN (PHOBIUS); TRANSMEMBRANE (PHOBIUS); SSF56973 (SUPERFAMILY)
1057	463991_length_1058_cvg_4.4_tip_1_0	23	high affinity nerve growth factor	322	9.00E-32	73.70%	0.175		IPR020635 (SMART); IPR001245 (PFAM); G3DSA:1.10.510.10 (GENE3D); PTHR24418 (PANTHER); PTHR24418:SF198 (PANTHER); IPR008266 (PROSITE_PATTERNS); TRANSMEMBRANE (PHOBIUS); CYTOPLASMIC_DOMAIN (PHOBIUS); NON_CYTOPLASMIC_DOMAIN (PHOBIUS); IPR011009 (SUPERFAMILY)
986	455925_length_969_cvg_2.0_tip_1_3	9	periplasmic endochitinase	323	0	99.70%	0.141		IPR003610 (SMART); IPR013781 (G3DSA:3.20.20.GENE3D); IPR003610 (PFAM); IPR003610 (G3DSA:2.10.10.GENE3D); IPR017853 (SUPERFAMILY); IPR003610 (SUPERFAMILY)
987	455925_length_969_cvg_2.0_tip_1_4	9	chi11_metan ame: full=endochitinase 11 ame: full=chitinase 11 flags: precursor	323	1.30E-06	77.00%	0.146		NON_CYTOPLASMIC_DOMAIN (PHOBIUS); TRANSMEMBRANE (PHOBIUS); CYTOPLASMIC_DOMAIN (PHOBIUS)
988	456087_length_970_cvg_11.0_tip_1_2	55	peroxisomal trans-2-enoyl- reductase-like	323	3.32E-128	74.70%	0.131		IPR002347 (PRINTS); PF13561 (PFAM); IPR016040 (G3DSA:3.40.50.GENE3D); PTHR24317 (PANTHER); SSF51735 (SUPERFAMILY)
989	456095_length_970_cvg_2.5_tip_1_3	13	neural ectodermal development factor imp-l2	324	3.74E-44	51.40%	0.809	Y	IPR003599 (SMART); IPR003598 (SMART); IPR013098 (PFAM); PF13895 (PFAM); IPR013783 (G3DSA:2.60.40.GENE3D); IPR013783 (G3DSA:2.60.40.GENE3D); PTHR10489 (PANTHER); NON_CYTOPLASMIC_DOMAIN (PHOBIUS); SIGNAL_PEPTIDE (PHOBIUS); SIGNAL_PEPTIDE_C_REGION (PHOBIUS); SIGNAL_PEPTIDE_H_REGION (PHOBIUS); SIGNAL_PEPTIDE_N_REGION (PHOBIUS); IPR007110 (PROSITE_PROFILES); IPR007110 (PROSITE_PROFILES); SignalP-noTM (SIGNALP_EUK); SSF48726 (SUPERFAMILY); SSF48726 (SUPERFAMILY)
990	456115_length_970_cvg_4.0_tip_1_3	14	zinc finger protein 347 isoform x1	324	1.26E-57	69.00%	0.101		IPR015880 (SMART); IPR013087 (G3DSA:3.30.160.GENE3D); IPR013087 (G3DSA:3.30.160.GENE3D); IPR013087 (G3DSA:3.30.160.GENE3D); PF13465 (PFAM); PTHR24409 (PANTHER); PTHR24409:SF13 (PANTHER); IPR007087 (PROSITE_PATTERNS); IPR007087 (PROSITE_PATTERNS); IPR007087 (PROSITE_PATTERNS); IPR007087 (PROSITE_PATTERNS); IPR007087 (PROSITE_PATTERNS); IPR007087 (PROSITE_PATTERNS); TRANSMEMBRANE (PHOBIUS); CYTOPLASMIC_DOMAIN (PHOBIUS); NON_CYTOPLASMIC_DOMAIN (PHOBIUS); IPR007087 (PROSITE_PROFILES); IPR007087 (PROSITE_PROFILES); IPR007087 (PROSITE_PROFILES); IPR007087 (PROSITE_PROFILES); IPR007087 (PROSITE_PROFILES); IPR007087 (PROSITE_PROFILES); SSF57667 (SUPERFAMILY); SSF57667 (SUPERFAMILY); SSF57667 (SUPERFAMILY); TMhelix (TMHMM)
991	456291_length_972_cvg_11.8_tip_1_5	53	bone morphogenetic protein 4	324	5.39E-14	46.40%	0.112		Coil (COILS); IPR001111 (PFAM); IPR015615 (PANTHER)
992	456497_length_974_cvg_47.1_tip_1_2	225	zinc finger protein zpr1	324	1.14E-127	73.80%	0.185		IPR004457 (SMART); IPR004457 (PFAM); IPR004457 (TIGRFAM); PTHR10876:SF0 (PANTHER); PTHR10876 (PANTHER)

993	456537_length_974_cvg_4.5_tip_1_5	24	gastrula zinc finger protein	324	1.25E-22	56.80%	0.104		IPR015880 (SMART); IPR013087 (G3DSA:3.30.160.GENE3D); PF13894 (PFAM); PF13465 (PFAM); IPR013087 (G3DSA:3.30.160.GENE3D); IPR013087 (G3DSA:3.30.160.GENE3D); IPR007087 (PFAM); PTHR24409 (PANTHER); PTHR24409:SF14 (PANTHER); IPR007087 (PROSITE_PATTERNS); IPR007087 (PROSITE_PATTERNS); IPR007087 (PROSITE_PATTERNS); IPR007087 (PROSITE_PATTERNS); IPR007087 (PROSITE_PROFILES); IPR007087 (PROSITE_PROFILES); IPR007087 (PROSITE_PROFILES); IPR007087 (PROSITE_PROFILES); SSF57667 (SUPERFAMILY); SSF57667 (SUPERFAMILY)
1022	458903_length_999_cvg_36.3_tip_1_5	147	subtilisin-like serine protease	324	8.29E-13	56.00%	0.510	Y	TRANSMEMBRANE (PHOBIUS); CYTOPLASMIC_DOMAIN (PHOBIUS); NON_CYTOPLASMIC_DOMAIN (PHOBIUS); TRANSMEMBRANE (PHOBIUS); NON_CYTOPLASMIC_DOMAIN (PHOBIUS); SignalP-TM (SIGNALP_EUK)
994	456555_length_975_cvg_70.9_tip_1_4	15826	anti-lipopolysaccharide factor	325	3.10E-33	68.60%	0.411	Y	IPR024509 (PFAM); CYTOPLASMIC_DOMAIN (PHOBIUS); CYTOPLASMIC_DOMAIN (PHOBIUS); TRANSMEMBRANE (PHOBIUS); NON_CYTOPLASMIC_DOMAIN (PHOBIUS); TRANSMEMBRANE (PHOBIUS); NON_CYTOPLASMIC_DOMAIN (PHOBIUS); TRANSMEMBRANE (PHOBIUS); IPR024716 (PRODOM); TMhelix (TMHMM); TMhelix (TMHMM)
995	456779_length_977_cvg_3.0_tip_1_3	13	zinc finger protein	326	7.98E-15	56.10%	0.113		IPR015880 (SMART); PF13465 (PFAM); IPR013087 (G3DSA:3.30.160.GENE3D); PF13894 (PFAM); PTHR24375:SF106 (PANTHER); PTHR24375 (PANTHER); IPR007087 (PROSITE_PATTERNS); IPR007087 (PROSITE_PATTERNS); IPR007087 (PROSITE_PATTERNS); IPR007087 (PROSITE_PROFILES); IPR007087 (PROSITE_PROFILES); IPR007087 (PROSITE_PROFILES); SSF57667 (SUPERFAMILY); SSF57667 (SUPERFAMILY)
996	456819_length_977_cvg_61.8_tip_1_0	376	adp-ribosylation factor-like protein 1	326	8.92E-118	95.90%	0.134		IPR006689 (PRINTS); IPR006687 (SMART); IPR024156 (SMART); IPR003579 (SMART); IPR027417 (G3DSA:3.40.50.GENE3D); IPR005225 (TIGRFAM); IPR006689 (PFAM); PTHR11711:SF41 (PANTHER); PTHR11711 (PANTHER); IPR024156 (PROSITE_PROFILES); IPR027417 (SUPERFAMILY)
997	456875_length_978_cvg_3.1_tip_1_4	18	zinc finger	326	1.22E-70	69.50%	0.1		IPR015880 (SMART); IPR013087 (G3DSA:3.30.160.GENE3D); IPR013087 (G3DSA:3.30.160.GENE3D); IPR013087 (G3DSA:3.30.160.GENE3D); PF13894 (PFAM); PF13465 (PFAM); PTHR24391 (PANTHER); PTHR24391:SF9 (PANTHER); IPR007087 (PROSITE_PATTERNS); IPR007087 (PROSITE_PATTERNS); IPR007087 (PROSITE_PATTERNS); IPR007087 (PROSITE_PATTERNS); IPR007087 (PROSITE_PROFILES); IPR007087 (PROSITE_PROFILES); IPR007087 (PROSITE_PROFILES); IPR007087 (PROSITE_PROFILES); SSF57667 (SUPERFAMILY); SSF57667 (SUPERFAMILY)
998	456899_length_978_cvg_22.4_tip_1_4	89	apolipoprotein d-like	326	2.80E-42	60.20%	0.246		IPR003057 (PRINTS); IPR000566 (PFAM); IPR012674 (G3DSA:2.40.128.GENE3D); PTHR10612:SF7 (PANTHER); PTHR10612 (PANTHER); CYTOPLASMIC_DOMAIN (PHOBIUS); TRANSMEMBRANE (PHOBIUS); NON_CYTOPLASMIC_DOMAIN (PHOBIUS); IPR011038 (SUPERFAMILY); TMhelix (TMHMM)
999	457017_length_979_cvg_3.6_tip_1_1	15	dnaj homolog subfamily c member 21-like	326	1.55E-140	81.50%	0.122		Coil (COILS); Coil (COILS); IPR001623 (PRINTS); IPR001623 (SMART); IPR001623 (G3DSA:1.10.287.GENE3D); IPR001623 (PFAM); PTHR24078 (PANTHER); PTHR24078:SF150 (PANTHER); IPR018253 (PROSITE_PATTERNS); IPR001623 (PROSITE_PROFILES); IPR001623 (SUPERFAMILY)
1000	457113_length_980_cvg_76.7_tip_1_5	62696	chymotrypsinogen 2-like	326	2.46E-46	56.50%	0.31		IPR001314 (PRINTS); IPR001254 (SMART); G3DSA:2.40.10.10 (GENE3D); G3DSA:2.40.10.10 (GENE3D); G3DSA:2.40.10.10 (GENE3D); IPR001254 (PFAM); PTHR24256 (PANTHER); IPR018114 (PROSITE_PATTERNS); IPR018114 (PROSITE_PATTERNS); NON_CYTOPLASMIC_DOMAIN (PHOBIUS); CYTOPLASMIC_DOMAIN (PHOBIUS); TRANSMEMBRANE (PHOBIUS); IPR001254 (PROSITE_PROFILES); IPR009003 (SUPERFAMILY); TMhelix (TMHMM)
1001	457123_length_980_cvg_5.8_tip_1_3	26	trypsin theta	327	4.33E-19	52.80%	0.107		IPR001254 (SMART); G3DSA:2.40.10.10 (GENE3D); IPR001254 (PFAM); G3DSA:2.40.10.10 (GENE3D); PTHR24276 (PANTHER); CYTOPLASMIC_DOMAIN (PHOBIUS); NON_CYTOPLASMIC_DOMAIN (PHOBIUS); TRANSMEMBRANE (PHOBIUS); IPR001254 (PROSITE_PROFILES); IPR009003 (SUPERFAMILY); TMhelix (TMHMM); TMhelix (TMHMM)
1002	457203_length_981_cvg_5.4_tip_1_2	26	immunoglobulin superfamily dcc subclass member 3-like	327	7.51E-40	65.00%	0.105		IPR003598 (SMART); IPR003599 (SMART); IPR013783 (G3DSA:2.60.40.GENE3D); IPR013783 (G3DSA:2.60.40.GENE3D); PF13895 (PFAM); IPR013098 (PFAM); IPR013783 (G3DSA:2.60.40.GENE3D); PTHR10489 (PANTHER); TRANSMEMBRANE (PHOBIUS); CYTOPLASMIC_DOMAIN (PHOBIUS); NON_CYTOPLASMIC_DOMAIN (PHOBIUS); CYTOPLASMIC_DOMAIN (PHOBIUS); TRANSMEMBRANE (PHOBIUS); IPR007110 (PROSITE_PROFILES); SSF48726 (SUPERFAMILY); SSF48726 (SUPERFAMILY); TMhelix (TMHMM); TMhelix (TMHMM)

1038	461487_length_1028_cvg_41.3_tip_1_5	168	transcriptional repressor protein yy1-like isoform x2	327	3.09E-65	86.80%	0.098	IPR015880 (SMART); PF13465 (PFAM); IPR013087 (G3DSA:3.30.160.GENE3D); IPR013087 (G3DSA:3.30.160.GENE3D); IPR013087 (G3DSA:3.30.160.GENE3D); PTHR14003:SF10 (PANTHER); PTHR14003 (PANTHER); IPR007087 (PROSITE_PATTERNS); IPR007087 (PROSITE_PATTERNS); TRANSMEMBRANE (PHOBIUS); CYTOPLASMIC_DOMAIN (PHOBIUS); NON_CYTOPLASMIC_DOMAIN (PHOBIUS); IPR007087 (PROSITE_PROFILES); IPR007087 (PROSITE_PROFILES); IPR007087 (PROSITE_PROFILES); SSF57667 (SUPERFAMILY); SSF57667 (SUPERFAMILY)
1003	457349_length_983_cvg_4.0_tip_1_0	13	cathepsin l	328	1.40E-103	82.50%	0.126	IPR000668 (SMART); IPR000668 (PFAM); G3DSA:3.90.70.10 (GENE3D); IPR013128 (PANTHER); PTHR12411:SF265 (PANTHER); IPR025660 (PROSITE_PATTERNS); IPR025661 (PROSITE_PATTERNS); SSF54001 (SUPERFAMILY)
1004	457537_length_985_cvg_6.3_tip_1_2	23	zinc finger protein 84-like	328	8.64E-80	61.40%	0.116	IPR015880 (SMART); PF13465 (PFAM); IPR013087 (G3DSA:3.30.160.GENE3D); IPR013087 (G3DSA:3.30.160.GENE3D); IPR013087 (G3DSA:3.30.160.GENE3D); PTHR24409 (PANTHER); IPR007087 (PROSITE_PATTERNS); IPR007087 (PROSITE_PATTERNS); IPR007087 (PROSITE_PATTERNS); IPR007087 (PROSITE_PATTERNS); IPR007087 (PROSITE_PATTERNS); IPR007087 (PROSITE_PATTERNS); IPR007087 (PROSITE_PATTERNS); IPR007087 (PROSITE_PATTERNS); IPR007087 (PROSITE_PROFILES); IPR007087 (PROSITE_PROFILES); IPR007087 (PROSITE_PROFILES); IPR007087 (PROSITE_PROFILES); IPR007087 (PROSITE_PROFILES); IPR007087 (PROSITE_PROFILES); IPR007087 (PROSITE_PROFILES); IPR007087 (PROSITE_PROFILES); SSF57667 (SUPERFAMILY); SSF57667 (SUPERFAMILY); SSF57667 (SUPERFAMILY); SSF57667 (SUPERFAMILY)
1005	457557_length_985_cvg_9.2_tip_1_0	36	otogelin- partial	329	9.04E-11	40.40%	0.098	IPR001007 (PROSITE_PATTERNS)
1006	457619_length_986_cvg_3.4_tip_1_3	20	zinc finger protein 271 (zinc finger protein 7) (zinc finger protein znfphex133) (epstein-barr virus-induced zinc finger protein) (znf-eb) (ct-zfp48) (zinc finger protein	329	7.95E-28	55.40%	0.1	IPR015880 (SMART); IPR007087 (PFAM); PF13894 (PFAM); IPR013087 (G3DSA:3.30.160.GENE3D); IPR013087 (G3DSA:3.30.160.GENE3D); IPR013087 (G3DSA:3.30.160.GENE3D); PTHR24409 (PANTHER); PTHR24409:SF14 (PANTHER); IPR007087 (PROSITE_PATTERNS); IPR007087 (PROSITE_PATTERNS); IPR007087 (PROSITE_PATTERNS); IPR007087 (PROSITE_PATTERNS); IPR007087 (PROSITE_PATTERNS); IPR007087 (PROSITE_PATTERNS); IPR007087 (PROSITE_PROFILES); IPR007087 (PROSITE_PROFILES); IPR007087 (PROSITE_PROFILES); IPR007087 (PROSITE_PROFILES); IPR007087 (PROSITE_PROFILES); IPR007087 (PROSITE_PROFILES); SSF57667 (SUPERFAMILY); SSF57667 (SUPERFAMILY); SSF57667 (SUPERFAMILY)
1007	457663_length_986_cvg_4.0_tip_1_3	18	cytolysin src-1-like	329	3.53E-26	52.00%	0.111	IPR009104 (PFAM); IPR015926 (G3DSA:2.60.270.GENE3D); IPR015926 (SUPERFAMILY)
1008	457665_length_986_cvg_2.8_tip_1_0	16	er degradation-enhancing alpha-mannosidase-like protein 3 isoform x1	329	0	85.50%	0.134	IPR001382 (PRINTS); IPR001382 (G3DSA:1.50.10.GENE3D); IPR001382 (PFAM); PTHR11742:SF38 (PANTHER); IPR001382 (PANTHER); IPR001382 (SUPERFAMILY)
1009	457671_length_986_cvg_2.0_tip_1_0	9	leucine-rich repeat and calponin homology domain-containing protein 2-like	329	5.55E-49	71.70%	0.103	IPR001715 (SMART); IPR001715 (PFAM); IPR001715 (G3DSA:1.10.418.GENE3D); NON_CYTOPLASMIC_DOMAIN (PHOBIUS); CYTOPLASMIC_DOMAIN (PHOBIUS); TRANSMEMBRANE (PHOBIUS); NON_CYTOPLASMIC_DOMAIN (PHOBIUS); TRANSMEMBRANE (PHOBIUS); IPR001715 (PROSITE_PROFILES); IPR001715 (SUPERFAMILY); TMhelix (TMHMM)
1010	457725_length_987_cvg_5.0_tip_1_0	23	kielin chordin-like protein	329	3.39E-08	56.50%	0.106	IPR001846 (PFAM); PTHR11339 (PANTHER); IPR001846 (PROSITE_PROFILES)
1011	457749_length_987_cvg_33.8_tip_1_4	133	ferritin 2 light chain homologue	329	3.33E-29	57.10%	0.116	IPR008331 (PFAM); IPR012347 (G3DSA:1.20.1260.GENE3D); PTHR11431:SF28 (PANTHER); IPR001519 (PANTHER); IPR009040 (PROSITE_PROFILES); IPR009078 (SUPERFAMILY)
1012	457881_length_988_cvg_2.0_tip_1_4	12	otogelin-like protein	329	2.19E-13	45.00%	0.105	no IPS match
1028	459987_length_1011_cvg_59.8_tip_1_3	595	mitochondrial manganese superoxide dismutase	329	2.20E-121	86.10%	0.114	Coil (COILS); IPR001189 (PRINTS); G3DSA:1.10.287.990 (GENE3D); IPR019831 (PFAM); IPR019832 (PFAM); PTHR11404:SF6 (PANTHER); IPR001189 (PANTHER); IPR019833 (PROSITE_PATTERNS); CYTOPLASMIC_DOMAIN (PHOBIUS); NON_CYTOPLASMIC_DOMAIN (PHOBIUS); TRANSMEMBRANE (PHOBIUS); IPR019831 (SUPERFAMILY); IPR019832 (SUPERFAMILY); TMhelix (TMHMM)
1013	457915_length_989_cvg_59.3_tip_1_3	6591	peptidyl-prolyl cis-trans isomerase	330	2.14E-101	93.10%	0.149	IPR002130 (PRINTS); IPR029000 (G3DSA:2.40.100.GENE3D); IPR002130 (PFAM); PTHR11071:SF116 (PANTHER); IPR024936 (PANTHER); IPR020892 (PROSITE_PATTERNS); IPR002130 (PROSITE_PROFILES); IPR029000 (SUPERFAMILY)
1014	458097_length_991_cvg_3.2_tip_1_1	19	alpha-n-acetylgalactosaminidase isoform x4	330	2.17E-170	88.30%	0.177	IPR002241 (PRINTS); IPR000111 (PFAM); IPR013785 (G3DSA:3.20.20.GENE3D); PTHR11452 (PANTHER); PTHR11452:SF23 (PANTHER); IPR000111 (PROSITE_PATTERNS); IPR017853 (SUPERFAMILY)

1023	459023_length_1001_cvg_29.5_tip_1_3	126	aminoacyl trna synthase complex-interacting multifunctional protein partial	330	3.46E-100	68.30%	0.101	Coil (COILS); IPR002547 (PFAM); IPR012340 (G3DSA:2.40.50.GENE3D); PTHR11586 (PANTHER); PTHR11586:SF1 (PANTHER); IPR002547 (PROSITE_PROFILES); IPR012340 (SUPERFAMILY)
1015	458115_length_991_cvg_4.7_tip_1_3	23	rac protein kinase drac-	331	3.92E-162	86.90%	0.121	IPR000961 (SMART); IPR002290 (SMART); G3DSA:1.10.510.10 (GENE3D); IPR000719 (PFAM); IPR017892 (PFAM); PTHR24351 (PANTHER); PTHR24351:SF64 (PANTHER); IPR008271 (PROSITE_PATTERNS); IPR000961 (PROSITE_PROFILES); IPR000719 (PROSITE_PROFILES); IPR011009 (SUPERFAMILY)
1016	458135_length_991_cvg_72.9_tip_1_3	247454	prostasin- partial	331	7.65E-47	55.70%	0.132	IPR001314 (PRINTS); IPR001254 (SMART); G3DSA:2.40.10.10 (GENE3D); G3DSA:2.40.10.10 (GENE3D); G3DSA:2.40.10.10 (GENE3D); IPR001254 (PFAM); PTHR24268 (PANTHER); IPR018114 (PROSITE_PATTERNS); IPR018114 (PROSITE_PATTERNS); IPR001254 (PROSITE_PROFILES); IPR009003 (SUPERFAMILY)
1017	458187_length_992_cvg_63.0_tip_0_0	596	14-3-3 partial	331	2.98E-28	100.00%	0.113	IPR023410 (PFAM); G3DSA:1.20.190.20 (GENE3D); IPR000308 (PANTHER); IPR023409 (PROSITE_PATTERNS); IPR023410 (SUPERFAMILY)
1018	458195_length_992_cvg_68.2_tip_1_1	2323	probable chitinase 3	331	1.66E-72	53.40%	0.098	IPR011583 (SMART); IPR002557 (SMART); IPR001223 (PFAM); IPR002557 (G3DSA:2.170.140.GENE3D); IPR029070 (G3DSA:3.10.50.GENE3D); IPR002557 (PFAM); IPR013781 (G3DSA:3.20.20.GENE3D); PTHR11177:SF112 (PANTHER); PTHR11177 (PANTHER); IPR002557 (PROSITE_PROFILES); IPR029070 (SUPERFAMILY); IPR017853 (SUPERFAMILY); IPR002557 (SUPERFAMILY)
1019	458399_length_994_cvg_16.1_tip_1_2	74	lysosomal acid phosphatase	331	2.34E-86	67.10%	0.099	IPR029033 (G3DSA:3.40.50.GENE3D); IPR000560 (PFAM); PTHR11567:SF26 (PANTHER); PTHR11567 (PANTHER); IPR000560 (PROSITE_PATTERNS); NON_CYTOPLASMIC_DOMAIN (PHOBIUS); TRANSMEMBRANE (PHOBIUS); CYTOPLASMIC_DOMAIN (PHOBIUS); CYTOPLASMIC_DOMAIN (PHOBIUS); TRANSMEMBRANE (PHOBIUS); IPR029033 (SUPERFAMILY)
1020	458575_length_995_cvg_9.3_tip_1_5	46	tolloid-like protein 2 isoform x1	331	3.03E-160	74.70%	0.106	IPR000859 (SMART); IPR000859 (G3DSA:2.60.120.GENE3D); IPR000859 (PFAM); IPR002172 (G3DSA:4.10.400.GENE3D); IPR000859 (G3DSA:2.60.120.GENE3D); PTHR10127 (PANTHER); PTHR10127:SF557 (PANTHER); IPR000859 (PROSITE_PROFILES); IPR000859 (PROSITE_PROFILES); IPR000859 (SUPERFAMILY); IPR000859 (SUPERFAMILY)
1021	458817_length_998_cvg_3.2_tip_1_2	15	histone h2a	332	4.40E-78	99.80%	0.131	IPR002119 (PRINTS); IPR002119 (SMART); IPR007125 (PFAM); IPR009072 (G3DSA:1.10.20.GENE3D); PTHR23430:SF45 (PANTHER); PTHR23430 (PANTHER); IPR002119 (PROSITE_PATTERNS); IPR009072 (SUPERFAMILY)
1034	460605_length_1018_cvg_9.7_tip_1_0	46	a disintegrin and metalloproteinase with thrombospondin motifs 2	333	5.91E-15	48.80%	0.104	IPR002870 (PFAM); PTHR13723 (PANTHER)
1024	459545_length_1006_cvg_2.5_tip_1_4	14	af425264_1lysozyme precursor	335	5.60E-42	71.80%	0.421 Y	IPR001916 (PRINTS); IPR001916 (SMART); G3DSA:1.10.530.10 (GENE3D); IPR001916 (PFAM); PTHR11407:SF4 (PANTHER); PTHR11407 (PANTHER); IPR019799 (PROSITE_PATTERNS); TRANSMEMBRANE (PHOBIUS); CYTOPLASMIC_DOMAIN (PHOBIUS); NON_CYTOPLASMIC_DOMAIN (PHOBIUS); TRANSMEMBRANE (PHOBIUS); NON_CYTOPLASMIC_DOMAIN (PHOBIUS); IPR001916 (PROSITE_PROFILES); IPR023346 (SUPERFAMILY); TMhelix (TMHMM); TMhelix (TMHMM)
1040	461805_length_1032_cvg_58.9_tip_1_2	317	alpha-aminoadipic semialdehyde dehydrogenase	335	7.06E-164	84.80%	0.117	IPR015590 (PFAM); IPR016163 (G3DSA:3.40.309.GENE3D); IPR016162 (G3DSA:3.40.605.GENE3D); PTHR11699:SF144 (PANTHER); PTHR11699 (PANTHER); IPR029510 (PROSITE_PATTERNS); IPR016161 (SUPERFAMILY)
1025	459615_length_1007_cvg_64.9_tip_1_0	761	torsin-1b-like isoform x1	336	2.01E-77	66.90%	0.253	IPR001270 (PRINTS); IPR010448 (PFAM); IPR027417 (G3DSA:3.40.50.GENE3D); IPR010448 (PANTHER); NON_CYTOPLASMIC_DOMAIN (PHOBIUS); CYTOPLASMIC_DOMAIN (PHOBIUS); NON_CYTOPLASMIC_DOMAIN (PHOBIUS); TRANSMEMBRANE (PHOBIUS); TRANSMEMBRANE (PHOBIUS); IPR027417 (SUPERFAMILY); TMhelix (TMHMM)
1026	459749_length_1008_cvg_3.5_tip_1_5	15	sortilin-related receptor-like	336	3.90E-52	55.30%	0.465 Y	IPR013783 (G3DSA:2.60.40.GENE3D); CYTOPLASMIC_DOMAIN (PHOBIUS); NON_CYTOPLASMIC_DOMAIN (PHOBIUS); TRANSMEMBRANE (PHOBIUS); SignalP-noTM (SIGNALP_EUK); IPR003961 (SUPERFAMILY); TMhelix (TMHMM)
1027	459809_length_1009_cvg_37.2_tip_1_2	2816	alkaline serine protease	336	3.87E-20	66.80%	0.236	Coil (COILS); IPR007280 (PFAM); CYTOPLASMIC_DOMAIN (PHOBIUS); CYTOPLASMIC_DOMAIN (PHOBIUS); TRANSMEMBRANE (PHOBIUS); TRANSMEMBRANE (PHOBIUS); NON_CYTOPLASMIC_DOMAIN (PHOBIUS); TRANSMEMBRANE (PHOBIUS); NON_CYTOPLASMIC_DOMAIN (PHOBIUS); TRANSMEMBRANE (PHOBIUS); NON_CYTOPLASMIC_DOMAIN (PHOBIUS); TMhelix (TMHMM); TMhelix (TMHMM); TMhelix (TMHMM)
1029	460111_length_1012_cvg_3.7_tip_1_4	18	hyaluronidase- partial	337	5.63E-57	61.50%	0.121	IPR018155 (PRINTS); IPR018155 (PFAM); IPR013785 (G3DSA:3.20.20.GENE3D); IPR018155 (PANTHER); PTHR11769:SF8 (PANTHER); IPR017853 (SUPERFAMILY)

1030	460119_length_1012_cvg_8.3_tip_1_1	38	prolow-density lipoprotein receptor-related protein 1-like	337	3.41E-31	54.70%	0.103	IPR002172 (PRINTS); IPR002172 (SMART); IPR002172 (G3DSA:4.10.400.GENE3D); IPR002172 (G3DSA:4.10.400.GENE3D); IPR002172 (PFAM); IPR002172 (G3DSA:4.10.400.GENE3D); PTHR10529 (PANTHER); IPR023415 (PROSITE_PATTERNS); CYTOPLASMIC_DOMAIN (PHOBIUS); TRANSMEMBRANE (PHOBIUS); TRANSMEMBRANE (PHOBIUS); NON_CYTOPLASMIC_DOMAIN (PHOBIUS); CYTOPLASMIC_DOMAIN (PHOBIUS); IPR002172 (PROSITE_PROFILES); IPR002172 (PROSITE_PROFILES); IPR002172 (PROSITE_PROFILES); IPR002172 (SUPERFAMILY); IPR002172 (SUPERFAMILY); IPR002172 (SUPERFAMILY); TMhelix (TMHMM); TMhelix (TMHMM)
1031	460391_length_1015_cvg_10.4_tip_1_4	48	zinc finger protein 271 (zinc finger protein 7) (zinc finger protein znfphex133) (epstein-barr virus-induced zinc finger protein) (znf-eb) (ct-zfp48) (zinc finger protein	338	2.59E-31	51.90%	0.1	IPR015880 (SMART); IPR013087 (G3DSA:3.30.160.GENE3D); IPR007087 (PFAM); PF13894 (PFAM); IPR013087 (G3DSA:3.30.160.GENE3D); IPR013087 (G3DSA:3.30.160.GENE3D); PTHR24375:SF106 (PANTHER); PTHR24375 (PANTHER); IPR007087 (PROSITE_PATTERNS); IPR007087 (PROSITE_PATTERNS); IPR007087 (PROSITE_PATTERNS); IPR007087 (PROSITE_PATTERNS); IPR007087 (PROSITE_PATTERNS); IPR007087 (PROSITE_PATTERNS); NON_CYTOPLASMIC_DOMAIN (PHOBIUS); TRANSMEMBRANE (PHOBIUS); CYTOPLASMIC_DOMAIN (PHOBIUS); IPR007087 (PROSITE_PROFILES); IPR007087 (PROSITE_PROFILES); IPR007087 (PROSITE_PROFILES); IPR007087 (PROSITE_PROFILES); IPR007087 (PROSITE_PROFILES); SSF57667 (SUPERFAMILY); SSF57667 (SUPERFAMILY); SSF57667 (SUPERFAMILY); SSF57667 (SUPERFAMILY); TMhelix (TMHMM)
1032	460507_length_1017_cvg_37.0_tip_1_2	62	ensangp00000031746-like partial	339	8.20E-78	92.80%	0.232	IPR002130 (PRINTS); IPR002130 (PFAM); IPR029000 (G3DSA:2.40.100.GENE3D); IPR024936 (PANTHER); IPR020892 (PROSITE_PATTERNS); IPR002130 (PROSITE_PROFILES); IPR029000 (SUPERFAMILY)
1033	460545_length_1017_cvg_65.4_tip_1_1	1025	---NA---	339			0.187	no IPS match
1062	464363_length_1063_cvg_54.0_tip_1_1	333	isoamyl acetate-hydrolyzing esterase 1 homolog	339	2.20E-73	65.70%	0.118	IPR013830 (G3DSA:3.40.50.GENE3D); IPR001087 (PFAM); PTHR14209 (PANTHER); IPR013830 (SUPERFAMILY)
1047	462625_length_1041_cvg_55.3_tip_1_3	339	beta-mannosidase	340	8.69E-70	60.20%	0.114	IPR013781 (G3DSA:3.20.20.GENE3D); IPR028369 (PTHR10066:PANTHER); PTHR10066 (PANTHER); NON_CYTOPLASMIC_DOMAIN (PHOBIUS); TRANSMEMBRANE (PHOBIUS); CYTOPLASMIC_DOMAIN (PHOBIUS); IPR006102 (SUPERFAMILY); IPR017853 (SUPERFAMILY); IPR006102 (SUPERFAMILY)
1035	461089_length_1023_cvg_2.4_tip_1_4	15	dnaj homolog dnj-5	341	4.36E-132	71.60%	0.208	IPR001623 (PRINTS); IPR001623 (SMART); PF14901 (PFAM); IPR001623 (G3DSA:1.10.287.GENE3D); IPR001623 (PFAM); PTHR24078:SF141 (PANTHER); PTHR24078 (PANTHER); IPR001623 (PROSITE_PROFILES); IPR001623 (SUPERFAMILY)
1036	461141_length_1024_cvg_3.7_tip_1_5	21	aael001398- partial	341	2.90E-111	76.30%	0.156	Coil (COILS); IPR001849 (SMART); IPR001452 (SMART); IPR011993 (G3DSA:2.30.29.GENE3D); IPR001452 (PFAM); G3DSA:2.30.30.40 (GENE3D); PTHR12845 (PANTHER); PTHR12845:SF5 (PANTHER); IPR001849 (PROSITE_PROFILES); IPR001452 (PROSITE_PROFILES); SSF50729 (SUPERFAMILY); IPR001452 (SUPERFAMILY)
1037	461185_length_1025_cvg_21.3_tip_1_5	66	otogelin-like protein	341	6.30E-09	47.10%	0.099	no IPS match
1050	462795_length_1043_cvg_7.3_tip_1_4	42	dynein heavy chain axonemal	341	4.47E-11	60.80%	0.27	no IPS match
1039	461571_length_1029_cvg_7.6_tip_1_2	46	peptidyl-prolyl cis-trans isomerase h	343	5.42E-125	95.40%	0.111	IPR002130 (PRINTS); IPR002130 (PFAM); IPR029000 (G3DSA:2.40.100.GENE3D); PTHR11071:SF58 (PANTHER); IPR024936 (PANTHER); IPR020892 (PROSITE_PATTERNS); TRANSMEMBRANE (PHOBIUS); NON_CYTOPLASMIC_DOMAIN (PHOBIUS); TRANSMEMBRANE (PHOBIUS); NON_CYTOPLASMIC_DOMAIN (PHOBIUS); CYTOPLASMIC_DOMAIN (PHOBIUS); TRANSMEMBRANE (PHOBIUS); CYTOPLASMIC_DOMAIN (PHOBIUS); TRANSMEMBRANE (PHOBIUS); NON_CYTOPLASMIC_DOMAIN (PHOBIUS); IPR002130 (PROSITE_PROFILES); IPR029000 (SUPERFAMILY); TMhelix (TMHMM); TMhelix (TMHMM); TMhelix (TMHMM)
1041	462245_length_1037_cvg_5.6_tip_1_0	22	adamts-like protein 4 isoform x1	346	1.24E-22	56.70%	0.205	IPR000884 (SMART); IPR000884 (PFAM); G3DSA:2.20.100.10 (GENE3D); PTHR13723 (PANTHER); NON_CYTOPLASMIC_DOMAIN (PHOBIUS); NON_CYTOPLASMIC_DOMAIN (PHOBIUS); CYTOPLASMIC_DOMAIN (PHOBIUS); TRANSMEMBRANE (PHOBIUS); TRANSMEMBRANE (PHOBIUS); IPR000884 (PROSITE_PROFILES); IPR000884 (SUPERFAMILY); TMhelix (TMHMM); TMhelix (TMHMM); TMhelix (TMHMM)
1042	462307_length_1038_cvg_50.6_tip_1_1	694	peptidyl-prolyl cis-trans isomerase-like 3	346	2.52E-96	91.00%	0.146	IPR002130 (PRINTS); IPR029000 (G3DSA:2.40.100.GENE3D); IPR002130 (PFAM); IPR024936 (PANTHER); IPR020892 (PROSITE_PATTERNS); IPR002130 (PROSITE_PROFILES); IPR029000 (SUPERFAMILY)

1043	462333_length_1038_cvg_5.6_tip_1_1	26	serpin 3	346	1.92E-75	62.00%	0.11		IPR023796 (SMART); G3DSA:2.30.39.10 (GENE3D); G3DSA:3.30.497.10 (GENE3D); IPR023796 (PFAM); PTHR11461:SF52 (PANTHER); IPR000215 (PANTHER); IPR023796 (SUPERFAMILY)
1044	462479_length_1039_cvg_6.7_tip_1_2	34	zinc finger protein 729-like	346	4.41E-59	67.90%	0.101		IPR015880 (SMART); IPR013087 (G3DSA:3.30.160.GENE3D); PF13465 (PFAM); IPR013087 (G3DSA:3.30.160.GENE3D); PTHR24409 (PANTHER); IPR007087 (PROSITE_PATTERNS); IPR007087 (PROSITE_PATTERNS); IPR007087 (PROSITE_PATTERNS); IPR007087 (PROSITE_PATTERNS); IPR007087 (PROSITE_PATTERNS); IPR007087 (PROSITE_PATTERNS); IPR007087 (PROSITE_PROFILES); IPR007087 (PROSITE_PROFILES); IPR007087 (PROSITE_PROFILES); IPR007087 (PROSITE_PROFILES); IPR017455 (PROSITE_PROFILES); IPR007087 (PROSITE_PROFILES); IPR007087 (PROSITE_PROFILES); SSF57667 (SUPERFAMILY); SSF57667 (SUPERFAMILY); SSF57667 (SUPERFAMILY)
1045	462483_length_1039_cvg_2.8_tip_1_3	16	serine threonine-protein kinase 11	347	6.95E-118	75.10%	0.151		IPR002290 (SMART); IPR000719 (PFAM); G3DSA:1.10.510.10 (GENE3D); PTHR24347:SF1 (PANTHER); IPR020636 (PANTHER); IPR008271 (PROSITE_PATTERNS); NON_CYTOPLASMIC_DOMAIN (PHOBIUS); CYTOPLASMIC_DOMAIN (PHOBIUS); TRANSMEMBRANE (PHOBIUS); IPR000719 (PROSITE_PROFILES); IPR011009 (SUPERFAMILY)
1046	462567_length_1041_cvg_20.7_tip_1_4	117	27 kda hemolymph	347	1.53E-30	49.50%	0.177		IPR009832 (PFAM); PTHR20997 (PANTHER); CYTOPLASMIC_DOMAIN (PHOBIUS); TRANSMEMBRANE (PHOBIUS); NON_CYTOPLASMIC_DOMAIN (PHOBIUS)
1048	462677_length_1042_cvg_12.7_tip_1_2	86	3-hydroxyisobutyrate dehydrogenase	347	1.94E-42	55.30%	0.297		IPR013328 (G3DSA:1.10.1040.GENE3D); IPR006115 (PFAM); IPR016040 (G3DSA:3.40.50.GENE3D); IPR029154 (PFAM); PTHR22981:SF7 (PANTHER); PTHR22981 (PANTHER); CYTOPLASMIC_DOMAIN (PHOBIUS); NON_CYTOPLASMIC_DOMAIN (PHOBIUS); TRANSMEMBRANE (PHOBIUS); NON_CYTOPLASMIC_DOMAIN (PHOBIUS); TRANSMEMBRANE (PHOBIUS); CYTOPLASMIC_DOMAIN (PHOBIUS); TRANSMEMBRANE (PHOBIUS); IPR008927 (SUPERFAMILY); SSF51735 (SUPERFAMILY)
1049	462701_length_1042_cvg_67.8_tip_1_2	19147	glyceraldehyde-3-phosphate dehydrogenase	347	0	90.30%	0.134		IPR020831 (PRINTS); IPR020828 (SMART); IPR016040 (G3DSA:3.40.50.GENE3D); IPR020831 (PIRSF); IPR006424 (TIGRFAM); G3DSA:3.30.360.10 (GENE3D); IPR020829 (PFAM); IPR020828 (PFAM); IPR020831 (PANTHER); IPR020830 (PROSITE_PATTERNS); SSF51735 (SUPERFAMILY); SSF55347 (SUPERFAMILY)
1051	463099_length_1047_cvg_8.0_tip_1_1	33	gamma-interferon-inducible lysosomal thiol reductase	349	4.29E-43	51.10%	0.102		IPR004911 (PFAM); PTHR13234:SF8 (PANTHER); IPR004911 (PANTHER)
1069	465175_length_1072_cvg_7.1_tip_1_4	36	transcriptional activator related to msn2p	349	8.89E-09	63.00%	0.101		IPR015880 (SMART); IPR013087 (G3DSA:3.30.160.GENE3D); PF13894 (PFAM); IPR007087 (PFAM); PTHR24409 (PANTHER); PTHR24409:SF14 (PANTHER); IPR007087 (PROSITE_PATTERNS); IPR007087 (PROSITE_PATTERNS); NON_CYTOPLASMIC_DOMAIN (PHOBIUS); TRANSMEMBRANE (PHOBIUS); CYTOPLASMIC_DOMAIN (PHOBIUS); CYTOPLASMIC_DOMAIN (PHOBIUS); CYTOPLASMIC_DOMAIN (PHOBIUS); TRANSMEMBRANE (PHOBIUS); NON_CYTOPLASMIC_DOMAIN (PHOBIUS); TRANSMEMBRANE (PHOBIUS); TRANSMEMBRANE (PHOBIUS); IPR007087 (PROSITE_PROFILES); IPR007087 (PROSITE_PROFILES); SSF57667 (SUPERFAMILY); TMhelix (TMHMM)
1052	463255_length_1049_cvg_22.2_tip_1_4	108	coiled-coil domain-containing protein 134-like	350	1.61E-53	65.10%	0.203		IPR026321 (PFAM); IPR026321 (PANTHER); CYTOPLASMIC_DOMAIN (PHOBIUS); NON_CYTOPLASMIC_DOMAIN (PHOBIUS); TRANSMEMBRANE (PHOBIUS)
1053	463479_length_1052_cvg_2.0_tip_1_1	10	penicillin-binding protein partial	351	0	99.00%	0.384	Y	G3DSA:1.10.3810.10 (GENE3D); IPR012338 (G3DSA:3.40.710.GENE3D); IPR001264 (PFAM); PTHR32282 (PANTHER); PTHR32282:SF5 (PANTHER); IPR012338 (SUPERFAMILY); IPR023346 (SUPERFAMILY)
1054	463689_length_1055_cvg_4.9_tip_1_0	33	hypothetical protein CRE_02778	352	1.53E-14	56.00%	0.102		PTHR23022:SF64 (PANTHER); PTHR23022 (PANTHER)
1055	463695_length_1055_cvg_3.1_tip_1_3	20	gastrula zinc finger protein - partial	352	1.17E-45	57.70%	0.101		IPR015880 (SMART); IPR007087 (PFAM); IPR013087 (G3DSA:3.30.160.GENE3D); IPR013087 (G3DSA:3.30.160.GENE3D); PF13894 (PFAM); IPR013087 (G3DSA:3.30.160.GENE3D); PF13465 (PFAM); PTHR24409 (PANTHER); PTHR24409:SF14 (PANTHER); IPR007087 (PROSITE_PATTERNS); IPR007087 (PROSITE_PATTERNS); IPR007087 (PROSITE_PATTERNS); IPR007087 (PROSITE_PATTERNS); IPR007087 (PROSITE_PATTERNS); IPR007087 (PROSITE_PATTERNS); IPR007087 (PROSITE_PATTERNS); IPR007087 (PROSITE_PATTERNS); IPR007087 (PROSITE_PROFILES); IPR007087 (PROSITE_PROFILES); IPR007087 (PROSITE_PROFILES); IPR007087 (PROSITE_PROFILES); IPR007087 (PROSITE_PROFILES); IPR007087 (PROSITE_PROFILES); IPR007087 (PROSITE_PROFILES); IPR007087 (PROSITE_PROFILES); SSF57667 (SUPERFAMILY); SSF57667 (SUPERFAMILY); SSF57667 (SUPERFAMILY); SSF57667 (SUPERFAMILY); SSF57667 (SUPERFAMILY)

1056	463949_length_1058_cvg_15.4_tip_1_3	53	fibroblast growth factor receptor partial	353	8.31E-16	67.40%	0.101	IPR013783 (G3DSA:2.60.40.GENE3D); IPR028174 (PTHR24416:PANTHER); PTHR24416 (PANTHER); SSF48726 (SUPERFAMILY)
1058	464049_length_1059_cvg_5.4_tip_1_5	24	tyrosine-protein phosphatase lar isoform x1	353	8.30E-28	74.50%	0.118	IPR003961 (SMART); IPR013783 (G3DSA:2.60.40.GENE3D); IPR003961 (PFAM); PTHR19134 (PANTHER); PTHR19134:SF199 (PANTHER); CYTOPLASMIC_DOMAIN (PHOBIUS); TRANSMEMBRANE (PHOBIUS); NON_CYTOPLASMIC_DOMAIN (PHOBIUS); TRANSMEMBRANE (PHOBIUS); TRANSMEMBRANE (PHOBIUS); NON_CYTOPLASMIC_DOMAIN (PHOBIUS); CYTOPLASMIC_DOMAIN (PHOBIUS); IPR003961 (PROSITE_PROFILES); IPR003961 (SUPERFAMILY); TMhelix (TMHMM); TMhelix (TMHMM)
1059	464191_length_1061_cvg_6.4_tip_1_3	34	aromatic hydrocarbon receptor	354	3.40E-13	62.50%	0.172	NON_CYTOPLASMIC_DOMAIN (PHOBIUS); CYTOPLASMIC_DOMAIN (PHOBIUS); TRANSMEMBRANE (PHOBIUS); TMhelix (TMHMM)
1060	464303_length_1062_cvg_2.5_tip_1_3	13	zinc finger protein 235-like isoform x1	354	1.12E-31	61.20%	0.285	IPR015880 (SMART); IPR013087 (G3DSA:3.30.160.GENE3D); PF13465 (PFAM); IPR013087 (G3DSA:3.30.160.GENE3D); PTHR24409 (PANTHER); PTHR24409:SF13 (PANTHER); IPR007087 (PROSITE_PATTERNS); IPR007087 (PROSITE_PATTERNS); IPR007087 (PROSITE_PROFILES); IPR007087 (PROSITE_PROFILES); IPR007087 (PROSITE_PROFILES); SSF57667 (SUPERFAMILY); SSF57667 (SUPERFAMILY); SSF57667 (SUPERFAMILY)
1061	464335_length_1062_cvg_2.1_tip_1_5	17	xanthine dehydrogenase oxidase	354	1.95E-23	80.40%	0.623 Y	IPR008274 (PFAM); IPR008274 (G3DSA:3.30.365.GENE3D); PTHR11908:SF32 (PANTHER); PTHR11908 (PANTHER); SignalP-noTM (SIGNALP_EUK); IPR008274 (SUPERFAMILY)
1080	466451_length_1089_cvg_68.9_tip_1_0	537	glycosyl-phosphatidylinositol-linked carbonic anhydrase	354	3.78E-85	63.40%	0.167	IPR001148 (SMART); IPR001148 (PFAM); IPR001148 (G3DSA:3.10.200.GENE3D); IPR023561 (PANTHER); IPR018338 (PROSITE_PATTERNS); IPR001148 (PROSITE_PROFILES); IPR001148 (SUPERFAMILY)
1063	464691_length_1067_cvg_19.6_tip_1_5	85	insulin-like growth factor-binding protein 7 precursor	355	6.42E-37	64.90%	0.162	IPR003599 (SMART); IPR003598 (SMART); IPR013098 (PFAM); IPR013783 (G3DSA:2.60.40.GENE3D); IPR011390 (PANTHER); PTHR14186:SF9 (PANTHER); TRANSMEMBRANE (PHOBIUS); NON_CYTOPLASMIC_DOMAIN (PHOBIUS); NON_CYTOPLASMIC_DOMAIN (PHOBIUS); TRANSMEMBRANE (PHOBIUS); CYTOPLASMIC_DOMAIN (PHOBIUS); NON_CYTOPLASMIC_DOMAIN (PHOBIUS); CYTOPLASMIC_DOMAIN (PHOBIUS); TRANSMEMBRANE (PHOBIUS); TRANSMEMBRANE (PHOBIUS); IPR007110 (PROSITE_PROFILES); SSF48726 (SUPERFAMILY); TMhelix (TMHMM); TMhelix (TMHMM)
1064	464757_length_1068_cvg_7.4_tip_1_2	39	5-hydroxyisourate hydrolase	356	1.90E-33	68.20%	0.1	IPR000895 (PRINTS); IPR023416 (SMART); IPR023416 (G3DSA:2.60.40.GENE3D); IPR023416 (PFAM); IPR014306 (TIGRFAM); PTHR10395:SF8 (PANTHER); PTHR10395 (PANTHER); IPR023418 (PROSITE_PATTERNS); IPR023416 (SUPERFAMILY)
1065	464981_length_1070_cvg_7.4_tip_1_5	40	zinc finger protein 699-like	356	8.56E-34	55.70%	0.106	IPR015880 (SMART); PF13465 (PFAM); IPR013087 (G3DSA:3.30.160.GENE3D); IPR013087 (G3DSA:3.30.160.GENE3D); PF13894 (PFAM); IPR007087 (PFAM); PTHR24409 (PANTHER); PTHR24409:SF14 (PANTHER); IPR007087 (PROSITE_PATTERNS); IPR007087 (PROSITE_PATTERNS); IPR007087 (PROSITE_PATTERNS); IPR007087 (PROSITE_PATTERNS); IPR007087 (PROSITE_PATTERNS); IPR007087 (PROSITE_PATTERNS); IPR007087 (PROSITE_PATTERNS); IPR007087 (PROSITE_PROFILES); IPR007087 (PROSITE_PROFILES); IPR007087 (PROSITE_PROFILES); IPR007087 (PROSITE_PROFILES); IPR007087 (PROSITE_PROFILES); IPR007087 (PROSITE_PROFILES); IPR007087 (PROSITE_PROFILES); SSF57667 (SUPERFAMILY); SSF57667 (SUPERFAMILY); SSF57667 (SUPERFAMILY); SSF57667 (SUPERFAMILY)
1089	467121_length_1098_cvg_12.2_tip_1_5	70	protein spaetzle	356	8.86E-28	51.90%	0.141	PTHR23199 (PANTHER); NON_CYTOPLASMIC_DOMAIN (PHOBIUS); CYTOPLASMIC_DOMAIN (PHOBIUS); TRANSMEMBRANE (PHOBIUS); IPR029034 (SUPERFAMILY); TMhelix (TMHMM)
1066	465025_length_1070_cvg_3.0_tip_1_3	15	otogelin	357	1.29E-14	44.40%	0.11	no IPS match
1067	465069_length_1071_cvg_62.7_tip_1_1	782	thioredoxin 1	357	4.84E-49	83.30%	0.254	PR00421 (PRINTS); IPR012336 (G3DSA:3.40.30.GENE3D); IPR005746 (TIGRFAM); IPR013766 (PFAM); IPR005746 (PANTHER); IPR017937 (PROSITE_PATTERNS); TRANSMEMBRANE (PHOBIUS); TRANSMEMBRANE (PHOBIUS); TRANSMEMBRANE (PHOBIUS); NON_CYTOPLASMIC_DOMAIN (PHOBIUS); NON_CYTOPLASMIC_DOMAIN (PHOBIUS); TRANSMEMBRANE (PHOBIUS); TRANSMEMBRANE (PHOBIUS); NON_CYTOPLASMIC_DOMAIN (PHOBIUS); CYTOPLASMIC_DOMAIN (PHOBIUS); CYTOPLASMIC_DOMAIN (PHOBIUS); CYTOPLASMIC_DOMAIN (PHOBIUS); IPR012336 (PROSITE_PROFILES); IPR012336 (SUPERFAMILY); TMhelix (TMHMM); TMhelix (TMHMM); TMhelix (TMHMM); TMhelix (TMHMM); TMhelix (TMHMM)

1068	465169_length_1072_cvg_107.3_tip_0_4	61379	proclotting enzyme-like	357	4.08E-50	55.20%	0.860 Y	IPR001314 (PRINTS); IPR001254 (SMART); G3DSA:2.40.10.10 (GENE3D); G3DSA:2.40.10.10 (GENE3D); G3DSA:2.40.10.10 (GENE3D); IPR001254 (PFAM); PTHR24268 (PANTHER); IPR018114 (PROSITE_PATTERNS); IPR018114 (PROSITE_PATTERNS); SIGNAL_PEPTIDE_H_REGION (PHOBIUS); SIGNAL_PEPTIDE_C_REGION (PHOBIUS); NON_CYTOPLASMIC_DOMAIN (PHOBIUS); SIGNAL_PEPTIDE_N_REGION (PHOBIUS); SIGNAL_PEPTIDE (PHOBIUS); IPR001254 (PROSITE_PROFILES); SignalP-noTM (SIGNALP_EUK); IPR009003 (SUPERFAMILY)
1070	465191_length_1073_cvg_32.3_tip_1_3	153	peroxiredoxin partial	358	8.00E-116	83.50%	0.143	IPR000866 (PFAM); IPR012336 (G3DSA:3.40.30.GENE3D); G3DSA:3.30.1020.10 (GENE3D); IPR019479 (PFAM); PTHR10681 (PANTHER); PTHR10681:SF77 (PANTHER); TRANSMEMBRANE (PHOBIUS); NON_CYTOPLASMIC_DOMAIN (PHOBIUS); CYTOPLASMIC_DOMAIN (PHOBIUS); IPR012336 (PROSITE_PROFILES); IPR012336 (SUPERFAMILY); TMhelix (TMHMM)
1071	465395_length_1075_cvg_3.5_tip_1_1	17	galectin partial	358	8.14E-70	67.20%	0.099	IPR001079 (SMART); IPR001079 (SMART); IPR013320 (G3DSA:2.60.120.GENE3D); IPR001079 (PFAM); PTHR11346 (PANTHER); NON_CYTOPLASMIC_DOMAIN (PHOBIUS); TRANSMEMBRANE (PHOBIUS); CYTOPLASMIC_DOMAIN (PHOBIUS); TRANSMEMBRANE (PHOBIUS); NON_CYTOPLASMIC_DOMAIN (PHOBIUS); IPR001079 (PROSITE_PROFILES); IPR013320 (SUPERFAMILY); TMhelix (TMHMM)
1072	465495_length_1076_cvg_4.1_tip_1_5	25	scp-like extracellular domain containing protein 1	358	1.92E-47	52.30%	0.574 Y	IPR002413 (PRINTS); IPR001283 (PRINTS); IPR002172 (SMART); IPR014044 (SMART); IPR002172 (PFAM); IPR002172 (G3DSA:4.10.400.GENE3D); IPR014044 (PFAM); IPR014044 (G3DSA:3.40.33.GENE3D); IPR001283 (PANTHER); IPR018244 (PROSITE_PATTERNS); IPR023415 (PROSITE_PATTERNS); IPR018244 (PROSITE_PATTERNS); IPR002172 (PROSITE_PROFILES); SignalP-noTM (SIGNALP_EUK); IPR014044 (SUPERFAMILY); IPR002172 (SUPERFAMILY)
1073	465689_length_1079_cvg_7.3_tip_1_0	36	transposase	360	1.12E-42	57.50%	0.205	no IPS match
1074	465743_length_1079_cvg_53.0_tip_1_3	383	peptidoglycan recognition protein 3 short class	360	4.51E-58	65.10%	0.810 Y	IPR002502 (SMART); IPR006619 (SMART); IPR002502 (G3DSA:3.40.80.GENE3D); IPR002502 (PFAM); IPR015510 (PANTHER); SIGNAL_PEPTIDE_N_REGION (PHOBIUS); SIGNAL_PEPTIDE (PHOBIUS); NON_CYTOPLASMIC_DOMAIN (PHOBIUS); SIGNAL_PEPTIDE_C_REGION (PHOBIUS); SIGNAL_PEPTIDE_H_REGION (PHOBIUS); SignalP-noTM (SIGNALP_EUK); IPR002502 (SUPERFAMILY); TMhelix (TMHMM); TMhelix (TMHMM); TMhelix (TMHMM)
1075	465757_length_1080_cvg_6.3_tip_1_0	29	beta-mannosidase	360	7.42E-130	69.90%	0.121	IPR013781 (G3DSA:3.20.20.GENE3D); IPR006103 (PFAM); IPR013812 (G3DSA:2.60.40.GENE3D); PTHR10066 (PANTHER); IPR028369 (PTHR10066:PANTHER); IPR017853 (SUPERFAMILY); IPR006102 (SUPERFAMILY)
1117	469523_length_1132_cvg_61.6_tip_1_1	3070	eukaryotic translation initiation factor 3 subunit i-like	360	0	84.20%	0.173	IPR001680 (SMART); IPR015943 (G3DSA:2.130.10.GENE3D); IPR001680 (PFAM); PTHR19877 (PANTHER); IPR019775 (PROSITE_PATTERNS); IPR001680 (PROSITE_PROFILES); IPR017986 (PROSITE_PROFILES); IPR027525 (HAMAP); IPR001680 (PROSITE_PROFILES); IPR001680 (PROSITE_PROFILES); IPR001680 (PROSITE_PROFILES); IPR017986 (PROSITE_PROFILES); IPR017986 (SUPERFAMILY)
1076	465869_length_1081_cvg_3.6_tip_1_0	20	xanthine dehydrogenase partial	361	2.63E-180	81.50%	0.145	IPR008274 (G3DSA:3.30.365.GENE3D); IPR008274 (G3DSA:3.30.365.GENE3D); IPR008274 (G3DSA:3.30.365.GENE3D); IPR008274 (PFAM); IPR008274 (G3DSA:3.30.365.GENE3D); PTHR11908 (PANTHER); PTHR11908:SF32 (PANTHER); IPR008274 (SUPERFAMILY)
1077	465995_length_1083_cvg_9.0_tip_1_1	30	syntaxin-16 isoform x4	361	1.40E-104	70.80%	0.155	IPR000727 (SMART); IPR006011 (PFAM); G3DSA:1.20.58.70 (GENE3D); IPR000727 (PFAM); PTHR19957:SF83 (PANTHER); PTHR19957 (PANTHER); IPR006012 (PROSITE_PATTERNS); NON_CYTOPLASMIC_DOMAIN (PHOBIUS); TRANSMEMBRANE (PHOBIUS); NON_CYTOPLASMIC_DOMAIN (PHOBIUS); TRANSMEMBRANE (PHOBIUS); CYTOPLASMIC_DOMAIN (PHOBIUS); IPR000727 (PROSITE_PROFILES); IPR010989 (SUPERFAMILY); TMhelix (TMHMM); TMhelix (TMHMM)
1078	466097_length_1084_cvg_3.1_tip_1_5	14	clip-domain serine family d	361	1.02E-08	75.60%	0.12	G3DSA:2.40.10.10 (GENE3D); IPR001254 (PFAM); PTHR24264:SF9 (PANTHER); PTHR24264 (PANTHER); IPR009003 (SUPERFAMILY)
1105	468399_length_1115_cvg_68.0_tip_1_5	673	scp-like extracellular domain containing protein 2	361	1.97E-06	65.25%	0.789 Y	IPR002413 (PRINTS); IPR014044 (G3DSA:3.40.33.GENE3D); SIGNAL_PEPTIDE (PHOBIUS); SIGNAL_PEPTIDE_H_REGION (PHOBIUS); NON_CYTOPLASMIC_DOMAIN (PHOBIUS); SIGNAL_PEPTIDE_C_REGION (PHOBIUS); SIGNAL_PEPTIDE_N_REGION (PHOBIUS); SignalP-noTM (SIGNALP_EUK)

1079	466227_length_1086_cvg_8.2_tip_1_5	41	venom serine carboxypeptidase-like	362	1.32E-12	65.30%	0.119		IPR029058 (G3DSA:3.40.50.GENE3D); IPR001563 (PFAM); IPR001563 (PANTHER); TRANSMEMBRANE (PHOBIUS); CYTOPLASMIC_DOMAIN (PHOBIUS); TRANSMEMBRANE (PHOBIUS); NON_CYTOPLASMIC_DOMAIN (PHOBIUS); NON_CYTOPLASMIC_DOMAIN (PHOBIUS); IPR029058 (SUPERFAMILY); TMhelix (TMHMM); TMhelix (TMHMM)
1081	466557_length_1090_cvg_4.1_tip_1_1	24	chondroitin sulfate synthase 1	363	2.88E-111	64.50%	0.101		IPR029044 (G3DSA:3.90.550.GENE3D); IPR008428 (PFAM); PTHR12369 (PANTHER); PTHR12369:SF11 (PANTHER); IPR029044 (SUPERFAMILY)
1102	468229_length_1113_cvg_3.2_tip_1_4	16	zinc finger protein 239- partial	364	1.18E-49	57.20%	0.13		IPR015880 (SMART); IPR013087 (G3DSA:3.30.160.GENE3D); IPR013087 (G3DSA:3.30.160.GENE3D); PF13465 (PFAM); IPR013087 (G3DSA:3.30.160.GENE3D); IPR007087 (PFAM); IPR013087 (G3DSA:3.30.160.GENE3D); IPR013087 (G3DSA:3.30.160.GENE3D); PTHR24409 (PANTHER); IPR007087 (PROSITE_PATTERNS); IPR007087 (PROSITE_PATTERNS); IPR007087 (PROSITE_PATTERNS); IPR007087 (PROSITE_PATTERNS); IPR007087 (PROSITE_PATTERNS); IPR007087 (PROSITE_PATTERNS); IPR007087 (PROSITE_PROFILES); IPR007087 (PROSITE_PROFILES); IPR007087 (PROSITE_PROFILES); IPR007087 (PROSITE_PROFILES); IPR007087 (PROSITE_PROFILES); IPR007087 (PROSITE_PROFILES); IPR007087 (PROSITE_PROFILES); IPR007087 (PROSITE_PROFILES); SSF57667 (SUPERFAMILY); SSF57667 (SUPERFAMILY); SSF57667 (SUPERFAMILY); SSF57667 (SUPERFAMILY)
1082	466729_length_1093_cvg_2.0_tip_1_0	14	tissue factor pathway inhibitor	365	1.88E-55	50.10%	0.794	Y	IPR002223 (PRINTS); IPR002223 (SMART); IPR002223 (PFAM); IPR002223 (G3DSA:4.10.410.GENE3D); IPR002223 (G3DSA:4.10.410.GENE3D); PTHR10083 (PANTHER); IPR020901 (PROSITE_PATTERNS); IPR020901 (PROSITE_PATTERNS); IPR020901 (PROSITE_PATTERNS); NON_CYTOPLASMIC_DOMAIN (PHOBIUS); SIGNAL_PEPTIDE_N_REGION (PHOBIUS); SIGNAL_PEPTIDE (PHOBIUS); SIGNAL_PEPTIDE_H_REGION (PHOBIUS); SIGNAL_PEPTIDE_C_REGION (PHOBIUS); IPR002223 (PROSITE_PROFILES); IPR002223 (PROSITE_PROFILES); IPR002223 (PROSITE_PROFILES); IPR002223 (PROSITE_PROFILES); SignalP-noTM (SIGNALP_EUK); IPR002223 (SUPERFAMILY); IPR002223 (SUPERFAMILY); IPR002223 (SUPERFAMILY); IPR002223 (SUPERFAMILY)
1083	466769_length_1094_cvg_99.5_tip_1_0	213697	isoform a	365	2.63E-44	53.50%	0.776	Y	IPR001314 (PRINTS); IPR001254 (SMART); G3DSA:2.40.10.10 (GENE3D); IPR001254 (PFAM); G3DSA:2.40.10.10 (GENE3D); G3DSA:2.40.10.10 (GENE3D); PTHR24268 (PANTHER); IPR018114 (PROSITE_PATTERNS); IPR018114 (PROSITE_PATTERNS); SIGNAL_PEPTIDE_N_REGION (PHOBIUS); NON_CYTOPLASMIC_DOMAIN (PHOBIUS); SIGNAL_PEPTIDE (PHOBIUS); SIGNAL_PEPTIDE_H_REGION (PHOBIUS); SIGNAL_PEPTIDE_C_REGION (PHOBIUS); IPR001254 (PROSITE_PROFILES); SignalP-noTM (SIGNALP_EUK); IPR009003 (SUPERFAMILY)
1084	466899_length_1095_cvg_6.2_tip_1_3	27	neuronal cell adhesion	365	1.34E-111	70.20%	0.138		PR00014 (PRINTS); IPR003961 (SMART); IPR013783 (G3DSA:2.60.40.GENE3D); IPR003961 (PFAM); IPR013783 (G3DSA:2.60.40.GENE3D); IPR013783 (G3DSA:2.60.40.GENE3D); PTHR10489:SF553 (PANTHER); PTHR10489 (PANTHER); IPR003961 (PROSITE_PROFILES); IPR003961 (PROSITE_PROFILES); IPR003961 (PROSITE_PROFILES); IPR003961 (SUPERFAMILY); IPR003961 (SUPERFAMILY)
1085	466909_length_1095_cvg_2.0_tip_1_3	14	u4 u6 small nuclear ribonucleoprotein prp4	365	0	84.20%	0.122		IPR020472 (PRINTS); IPR001680 (SMART); IPR015943 (G3DSA:2.130.10.GENE3D); IPR001680 (PFAM); IPR027106 (PANTHER); IPR019775 (PROSITE_PATTERNS); IPR019775 (PROSITE_PATTERNS); IPR001680 (PROSITE_PROFILES); IPR001680 (PROSITE_PROFILES); IPR001680 (PROSITE_PROFILES); IPR001680 (PROSITE_PROFILES); IPR001680 (PROSITE_PROFILES); IPR001680 (PROSITE_PROFILES); IPR001680 (PROSITE_PROFILES); IPR017986 (PROSITE_PROFILES); IPR001680 (PROSITE_PROFILES); IPR017986 (SUPERFAMILY)
1086	466923_length_1096_cvg_58.6_tip_1_2	355	aldose reductase-like	365	4.20E-137	77.50%	0.101		IPR020471 (PRINTS); IPR023210 (G3DSA:3.20.20.GENE3D); IPR023210 (PFAM); IPR001395 (PANTHER); PTHR11732:SF202 (PANTHER); IPR018170 (PROSITE_PATTERNS); IPR018170 (PROSITE_PATTERNS); IPR023210 (SUPERFAMILY)
1087	466951_length_1096_cvg_3.3_tip_1_1	22	5 -nucleotidase	365	1.04E-125	70.80%	0.106		IPR008334 (PFAM); IPR008334 (G3DSA:3.90.780.GENE3D); IPR006179 (PANTHER); PTHR11575:SF7 (PANTHER); IPR008334 (SUPERFAMILY)
1088	467011_length_1097_cvg_4.5_tip_1_1	26	ves g 5 allergen	366	1.01E-46	53.20%	0.104		IPR001283 (PRINTS); IPR014044 (SMART); IPR014044 (PFAM); IPR014044 (G3DSA:3.40.33.GENE3D); IPR001283 (PANTHER); IPR018244 (PROSITE_PATTERNS); IPR014044 (SUPERFAMILY)

1115	469269_length_1128_cvg_63.0_tip_1_1	85847	transmembrane serine protease	366	9.92E-45	53.60%	0.593 Y	IPR001314 (PRINTS); IPR001254 (SMART); IPR001254 (PFAM); G3DSA:2.40.10.10 (GENE3D); G3DSA:2.40.10.10 (GENE3D); G3DSA:2.40.10.10 (GENE3D); PTHR24268 (PANTHER); IPR018114 (PROSITE_PATTERNS); IPR018114 (PROSITE_PATTERNS); SIGNAL_PEPTIDE (PHOBIUS); SIGNAL_PEPTIDE_C_REGION (PHOBIUS); NON_CYTOPLASMIC_DOMAIN (PHOBIUS); SIGNAL_PEPTIDE_N_REGION (PHOBIUS); SIGNAL_PEPTIDE_H_REGION (PHOBIUS); IPR001254 (PROSITE_PROFILES); SignalP-noTM (SIGNALP_EUK); IPR009003 (SUPERFAMILY)
1090	467319_length_1101_cvg_30.2_tip_1_5	145	estradiol 17-beta-dehydrogenase 8	367	1.75E-89	72.70%	0.204	IPR002347 (PRINTS); IPR002198 (PRINTS); IPR013968 (SMART); IPR016040 (G3DSA:3.40.50.GENE3D); IPR002198 (PFAM); PTHR24316 (PANTHER); PTHR24316:SF254 (PANTHER); IPR020904 (PROSITE_PATTERNS); SSF51735 (SUPERFAMILY)
1091	467333_length_1101_cvg_4.4_tip_1_3	26	ecto-nox disulfide-thiol exchanger 2	367	7.99E-113	77.00%	0.114	Coil (COILS); Coil (COILS); PTHR16001 (PANTHER)
1092	467565_length_1104_cvg_2.7_tip_1_4	13	17-beta-hydroxysteroid dehydrogenase 13-like	368	2.19E-34	73.50%	0.123	IPR002347 (PRINTS); IPR016040 (G3DSA:3.40.50.GENE3D); IPR002198 (PFAM); PTHR24316 (PANTHER); PTHR24316:SF214 (PANTHER); CYTOPLASMIC_DOMAIN (PHOBIUS); TRANSMEMBRANE (PHOBIUS); CYTOPLASMIC_DOMAIN (PHOBIUS); NON_CYTOPLASMIC_DOMAIN (PHOBIUS); TRANSMEMBRANE (PHOBIUS); NON_CYTOPLASMIC_DOMAIN (PHOBIUS); TRANSMEMBRANE (PHOBIUS); SSF51735 (SUPERFAMILY); TMhelix (TMHMM); TMhelix (TMHMM); TMhelix (TMHMM)
1093	467597_length_1104_cvg_4.2_tip_1_5	27	glucose 1-	368	3.25E-168	79.00%	0.139	IPR002347 (PRINTS); IPR016040 (G3DSA:3.40.50.GENE3D); IPR002198 (PFAM); PTHR24322:SF273 (PANTHER); PTHR24322 (PANTHER); SSF51735 (SUPERFAMILY)
1094	467807_length_1107_cvg_76.0_tip_1_5	98652	serine proteinase stubble	369	4.29E-40	52.30%	0.743 Y	IPR001314 (PRINTS); IPR001254 (SMART); G3DSA:2.40.10.10 (GENE3D); IPR001254 (PFAM); G3DSA:2.40.10.10 (GENE3D); G3DSA:2.40.10.10 (GENE3D); PTHR24268 (PANTHER); IPR018114 (PROSITE_PATTERNS); IPR018114 (PROSITE_PATTERNS); NON_CYTOPLASMIC_DOMAIN (PHOBIUS); SIGNAL_PEPTIDE (PHOBIUS); SIGNAL_PEPTIDE_H_REGION (PHOBIUS); SIGNAL_PEPTIDE_N_REGION (PHOBIUS); SIGNAL_PEPTIDE_C_REGION (PHOBIUS); IPR001254 (PROSITE_PROFILES); SignalP-noTM (SIGNALP_EUK); IPR009003 (SUPERFAMILY)
1095	467877_length_1108_cvg_10.9_tip_1_1	64	zinc finger protein	369	2.24E-37	55.80%	0.118	IPR015880 (SMART); IPR013087 (G3DSA:3.30.160.GENE3D); IPR013087 (G3DSA:3.30.160.GENE3D); IPR013087 (G3DSA:3.30.160.GENE3D); PF13894 (PFAM); PF13465 (PFAM); PTHR24409 (PANTHER); PTHR24409:SF14 (PANTHER); IPR007087 (PROSITE_PATTERNS); IPR007087 (PROSITE_PATTERNS); IPR007087 (PROSITE_PATTERNS); IPR007087 (PROSITE_PATTERNS); IPR007087 (PROSITE_PATTERNS); IPR007087 (PROSITE_PATTERNS); IPR007087 (PROSITE_PATTERNS); NON_CYTOPLASMIC_DOMAIN (PHOBIUS); CYTOPLASMIC_DOMAIN (PHOBIUS); TRANSMEMBRANE (PHOBIUS); IPR007087 (PROSITE_PROFILES); IPR007087 (PROSITE_PROFILES); IPR007087 (PROSITE_PROFILES); IPR007087 (PROSITE_PROFILES); IPR007087 (PROSITE_PROFILES); IPR007087 (PROSITE_PROFILES); IPR007087 (PROSITE_PROFILES); IPR007087 (PROSITE_PROFILES); SSF57667 (SUPERFAMILY); SSF57667 (SUPERFAMILY); SSF57667 (SUPERFAMILY); SSF57667 (SUPERFAMILY); TMhelix (TMHMM)
1096	467965_length_1109_cvg_48.5_tip_1_2	311	26s proteasome non-atpase regulatory subunit 10	369	4.16E-78	71.40%	0.151	IPR002110 (PRINTS); IPR002110 (SMART); IPR020683 (PFAM); IPR020683 (G3DSA:1.25.40.GENE3D); IPR020683 (G3DSA:1.25.40.GENE3D); PTHR24199 (PANTHER); TRANSMEMBRANE (PHOBIUS); CYTOPLASMIC_DOMAIN (PHOBIUS); TRANSMEMBRANE (PHOBIUS); CYTOPLASMIC_DOMAIN (PHOBIUS); NON_CYTOPLASMIC_DOMAIN (PHOBIUS); IPR002110 (PROSITE_PROFILES); IPR002110 (PROSITE_PROFILES); IPR002110 (PROSITE_PROFILES); IPR002110 (PROSITE_PROFILES); IPR002110 (PROSITE_PROFILES); IPR020683 (PROSITE_PROFILES); IPR020683 (SUPERFAMILY); TMhelix (TMHMM); TMhelix (TMHMM)
1097	468007_length_1110_cvg_3.9_tip_1_0	18	ras-related protein rab-35	370	7.92E-129	94.70%	0.115	IPR001806 (PRINTS); IPR020849 (SMART); IPR003578 (SMART); IPR002041 (SMART); IPR024156 (SMART); IPR003579 (SMART); IPR001806 (PFAM); IPR005225 (TIGRFAM); IPR027417 (G3DSA:3.40.50.GENE3D); PTHR24073:SF428 (PANTHER); PTHR24073 (PANTHER); PS51419 (PROSITE_PROFILES); IPR027417 (SUPERFAMILY)

1098	468075_length_1111_cvg_5.0_tip_1_4	30	adp-ribosylation factor-like protein 3	370	1.32E-109	93.90%	0.227	IPR006689 (PRINTS); IPR024156 (SMART); IPR003579 (SMART); IPR006687 (SMART); IPR005225 (TIGRFAM); IPR027417 (G3DSA:3.40.50.GENE3D); IPR006689 (PFAM); PTHR11711 (PANTHER); PTHR11711:SF124 (PANTHER); CYTOPLASMIC_DOMAIN (PHOBIUS); TRANSMEMBRANE (PHOBIUS); TRANSMEMBRANE (PHOBIUS); NON_CYTOPLASMIC_DOMAIN (PHOBIUS); NON_CYTOPLASMIC_DOMAIN (PHOBIUS); IPR024156 (PROSITE_PROFILES); IPR027417 (SUPERFAMILY); TMhelix (TMHMM)
1100	468141_length_1112_cvg_8.4_tip_1_5	37	gastrula zinc finger	370	7.04E-31	57.40%	0.109	IPR015880 (SMART); PF13894 (PFAM); PF13465 (PFAM); IPR013087 (G3DSA:3.30.160.GENE3D); IPR013087 (G3DSA:3.30.160.GENE3D); IPR013087 (G3DSA:3.30.160.GENE3D); PTHR24409 (PANTHER); IPR007087 (PROSITE_PATTERNS); IPR007087 (PROSITE_PATTERNS); IPR007087 (PROSITE_PATTERNS); IPR007087 (PROSITE_PATTERNS); IPR007087 (PROSITE_PATTERNS); TRANSMEMBRANE (PHOBIUS); NON_CYTOPLASMIC_DOMAIN (PHOBIUS); CYTOPLASMIC_DOMAIN (PHOBIUS); IPR007087 (PROSITE_PROFILES); IPR007087 (PROSITE_PROFILES); IPR007087 (PROSITE_PROFILES); IPR007087 (PROSITE_PROFILES); IPR007087 (PROSITE_PROFILES); IPR007087 (PROSITE_PROFILES); SSF57667 (SUPERFAMILY); SSF57667 (SUPERFAMILY); SSF57667 (SUPERFAMILY); TMhelix (TMHMM)
1099	468097_length_1111_cvg_3.4_tip_1_0	21	ankyrin repeat domain-containing protein 5-like	371	2.04E-18	47.80%	0.1	PTHR23022:SF64 (PANTHER); PTHR23022 (PANTHER)
1101	468191_length_1112_cvg_76.6_tip_1_3	4330	glyceraldehyde 3-phosphate dehydrogenase	371	1.36E-11	90.90%	0.128	IPR020831 (PANTHER); TRANSMEMBRANE (PHOBIUS); TRANSMEMBRANE (PHOBIUS); TRANSMEMBRANE (PHOBIUS); CYTOPLASMIC_DOMAIN (PHOBIUS); NON_CYTOPLASMIC_DOMAIN (PHOBIUS); CYTOPLASMIC_DOMAIN (PHOBIUS); TRANSMEMBRANE (PHOBIUS); CYTOPLASMIC_DOMAIN (PHOBIUS); NON_CYTOPLASMIC_DOMAIN (PHOBIUS); TMhelix (TMHMM); TMhelix (TMHMM); TMhelix (TMHMM)
1103	468245_length_1113_cvg_2.7_tip_1_1	17	metallophosphoesterase domain-containing protein 1	371	1.59E-116	81.20%	0.107	IPR004843 (PFAM); IPR029052 (G3DSA:3.60.21.GENE3D); PTHR12905 (PANTHER); IPR029052 (SUPERFAMILY)
1124	469853_length_1137_cvg_10.9_tip_1_4	72	chitinase partial	371	1.46E-66	67.10%	0.144	IPR002557 (SMART); IPR029070 (G3DSA:3.10.50.GENE3D); IPR002557 (G3DSA:2.170.140.GENE3D); IPR002557 (PFAM); IPR013781 (G3DSA:3.20.20.GENE3D); IPR001223 (PFAM); PTHR11177:SF112 (PANTHER); PTHR11177 (PANTHER); IPR002557 (PROSITE_PROFILES); IPR002557 (SUPERFAMILY); IPR017853 (SUPERFAMILY); IPR029070 (SUPERFAMILY)
1104	468297_length_1114_cvg_2.7_tip_1_0	12	upf0364 protein c6orf211 homolog	372	9.35E-96	60.50%	0.101	Coil (COILS); IPR002791 (PFAM); PTHR12260:SF1 (PANTHER); PTHR12260 (PANTHER); IPR002791 (SUPERFAMILY)
1106	468431_length_1115_cvg_6.3_tip_1_1	36	prepronuropeptide f i	372	1.58E-17	63.75%	0.142	IPR020392 (PROSITE_PATTERNS); TRANSMEMBRANE (PHOBIUS); NON_CYTOPLASMIC_DOMAIN (PHOBIUS); CYTOPLASMIC_DOMAIN (PHOBIUS); IPR001955 (PROSITE_PROFILES); TMhelix (TMHMM); TMhelix (TMHMM); TMhelix (TMHMM)
1107	468461_length_1116_cvg_3.8_tip_1_2	24	cysteine-rich with egf-like domain protein 2	372	3.58E-48	67.30%	0.112	IPR006212 (SMART); PTHR24838:SF13 (PANTHER); PTHR24838 (PANTHER); IPR018097 (PROSITE_PATTERNS); TRANSMEMBRANE (PHOBIUS); TRANSMEMBRANE (PHOBIUS); NON_CYTOPLASMIC_DOMAIN (PHOBIUS); CYTOPLASMIC_DOMAIN (PHOBIUS); CYTOPLASMIC_DOMAIN (PHOBIUS); TRANSMEMBRANE (PHOBIUS); NON_CYTOPLASMIC_DOMAIN (PHOBIUS); TRANSMEMBRANE (PHOBIUS); NON_CYTOPLASMIC_DOMAIN (PHOBIUS); TRANSMEMBRANE (PHOBIUS); CYTOPLASMIC_DOMAIN (PHOBIUS); IPR009030 (SUPERFAMILY); TMhelix (TMHMM); TMhelix (TMHMM)
1109	468545_length_1117_cvg_25.3_tip_1_1	109	thioredoxin 2	372	2.69E-34	70.70%	0.14	IPR013766 (PFAM); IPR012336 (G3DSA:3.40.30.GENE3D); PTHR10438:SF235 (PANTHER); IPR005746 (PANTHER); NON_CYTOPLASMIC_DOMAIN (PHOBIUS); CYTOPLASMIC_DOMAIN (PHOBIUS); TRANSMEMBRANE (PHOBIUS); IPR012336 (PROSITE_PROFILES); IPR012336 (SUPERFAMILY); TMhelix (TMHMM)
1133	470195_length_1143_cvg_20.4_tip_1_0	127	dehydrogenase reductase sdr family member 11-like	372	1.90E-42	73.30%	0.109	IPR002198 (PRINTS); IPR002347 (PRINTS); IPR016040 (G3DSA:3.40.50.GENE3D); PTHR24322:SF310 (PANTHER); PTHR24322 (PANTHER); SSF51735 (SUPERFAMILY)
1146	471119_length_1158_cvg_69.2_tip_1_2	1338	guanine nucleotide-binding protein subunit beta-like protein	372	0	93.20%	0.136	IPR020472 (PRINTS); IPR001680 (SMART); IPR015943 (G3DSA:2.130.10.GENE3D); IPR001680 (PFAM); IPR015943 (G3DSA:2.130.10.GENE3D); PTHR19868:SF0 (PANTHER); PTHR19868 (PANTHER); IPR019775 (PROSITE_PATTERNS); IPR019775 (PROSITE_PATTERNS); IPR019775 (PROSITE_PATTERNS); IPR019775 (PROSITE_PATTERNS); IPR001680 (PROSITE_PROFILES); IPR001680 (PROSITE_PROFILES); IPR001680 (PROSITE_PROFILES); IPR001680 (PROSITE_PROFILES); IPR001680 (PROSITE_PROFILES); IPR017986 (PROSITE_PROFILES); IPR001680 (PROSITE_PROFILES); IPR017986 (SUPERFAMILY)

1108	468519_length_1117_cvg_12.9_tip_1_0	66	zinc finger protein	373	1.24E-35	56.90%	0.101		IPR015880 (SMART); IPR013087 (G3DSA:3.30.160.GENE3D); IPR013087 (G3DSA:3.30.160.GENE3D); PF13894 (PFAM); PF13465 (PFAM); IPR013087 (G3DSA:3.30.160.GENE3D); PTHR24409 (PANTHER); IPR007087 (PROSITE_PATTERNS); IPR007087 (PROSITE_PATTERNS); IPR007087 (PROSITE_PATTERNS); IPR007087 (PROSITE_PATTERNS); IPR007087 (PROSITE_PATTERNS); IPR007087 (PROSITE_PATTERNS); TRANSMEMBRANE (PHOBIUS); CYTOPLASMIC_DOMAIN (PHOBIUS); NON_CYTOPLASMIC_DOMAIN (PHOBIUS); IPR007087 (PROSITE_PROFILES); IPR007087 (PROSITE_PROFILES); IPR007087 (PROSITE_PROFILES); IPR007087 (PROSITE_PROFILES); IPR007087 (PROSITE_PROFILES); IPR007087 (PROSITE_PROFILES); IPR007087 (PROSITE_PROFILES); SSF57667 (SUPERFAMILY); SSF57667 (SUPERFAMILY); SSF57667 (SUPERFAMILY); SSF57667 (SUPERFAMILY); TMhelix (TMHMM)
1110	468657_length_1118_cvg_4.0_tip_1_4	21	zinc finger protein 420-like	373	1.80E-37	61.10%	0.098		IPR015880 (SMART); PF13894 (PFAM); IPR013087 (G3DSA:3.30.160.GENE3D); PF13465 (PFAM); IPR013087 (G3DSA:3.30.160.GENE3D); PTHR24409:SF13 (PANTHER); PTHR24409 (PANTHER); IPR007087 (PROSITE_PATTERNS); IPR007087 (PROSITE_PATTERNS); IPR007087 (PROSITE_PATTERNS); IPR007087 (PROSITE_PATTERNS); IPR007087 (PROSITE_PATTERNS); IPR007087 (PROSITE_PATTERNS); CYTOPLASMIC_DOMAIN (PHOBIUS); TRANSMEMBRANE (PHOBIUS); TRANSMEMBRANE (PHOBIUS); NON_CYTOPLASMIC_DOMAIN (PHOBIUS); NON_CYTOPLASMIC_DOMAIN (PHOBIUS); IPR007087 (PROSITE_PROFILES); IPR007087 (PROSITE_PROFILES); IPR007087 (PROSITE_PROFILES); IPR007087 (PROSITE_PROFILES); IPR007087 (PROSITE_PROFILES); IPR007087 (PROSITE_PROFILES); SSF57667 (SUPERFAMILY); SSF57667 (SUPERFAMILY); SSF57667 (SUPERFAMILY); TMhelix (TMHMM); TMhelix (TMHMM)
1119	469579_length_1133_cvg_22.4_tip_1_4	109	e3 ubiquitin-protein ligase rnf185-like isoform x2	373	1.31E-86	81.00%	0.1		IPR001841 (SMART); PF13923 (PFAM); IPR013083 (G3DSA:3.30.40.GENE3D); PTHR12313:SF3 (PANTHER); PTHR12313 (PANTHER); IPR017907 (PROSITE_PATTERNS); CYTOPLASMIC_DOMAIN (PHOBIUS); TRANSMEMBRANE (PHOBIUS); CYTOPLASMIC_DOMAIN (PHOBIUS); TRANSMEMBRANE (PHOBIUS); CYTOPLASMIC_DOMAIN (PHOBIUS); NON_CYTOPLASMIC_DOMAIN (PHOBIUS); TRANSMEMBRANE (PHOBIUS); TRANSMEMBRANE (PHOBIUS); NON_CYTOPLASMIC_DOMAIN (PHOBIUS); IPR001841 (PROSITE_PROFILES); SSF57850 (SUPERFAMILY); TMhelix (TMHMM); TMhelix (TMHMM); TMhelix (TMHMM); TMhelix (TMHMM); TMhelix (TMHMM)
1156	471717_length_1167_cvg_82.4_tip_1_4	24389	venom allergen 3-like	373	5.77E-45	50.10%	0.174		IPR002413 (PRINTS); IPR001283 (PRINTS); IPR014044 (SMART); IPR014044 (G3DSA:3.40.33.GENE3D); IPR014044 (PFAM); IPR001283 (PANTHER); CYTOPLASMIC_DOMAIN (PHOBIUS); NON_CYTOPLASMIC_DOMAIN (PHOBIUS); TRANSMEMBRANE (PHOBIUS); IPR014044 (SUPERFAMILY); TMhelix (TMHMM)
1111	469029_length_1124_cvg_73.7_tip_1_2	2389	gamma-glutamyl hydrolase	374	3.14E-30	68.00%	0.1		IPR029062 (G3DSA:3.40.50.GENE3D); IPR015527 (PANTHER); IPR015527 (PROSITE_PROFILES); IPR029062 (SUPERFAMILY)
1151	471559_length_1164_cvg_6.2_tip_1_1	39	dentin matrix protein 4-like	374	0	85.00%	0.099		IPR009581 (PFAM); IPR024869 (PANTHER); PTHR12450:SF8 (PANTHER)
1112	469049_length_1124_cvg_63.0_tip_0_3	4759	protein disulfide-isomerase a3	375	1.13E-113	82.50%	0.119		PR00421 (PRINTS); IPR005788 (TIGRFAM); IPR012336 (G3DSA:3.40.30.GENE3D); PF13848 (PFAM); IPR013766 (PFAM); PTHR18929:SF60 (PANTHER); PTHR18929 (PANTHER); IPR017937 (PROSITE_PATTERNS); IPR012336 (PROSITE_PROFILES); IPR012336 (SUPERFAMILY)
1127	469959_length_1138_cvg_12.7_tip_1_0	78	thioredoxin-related transmembrane protein 1	375	7.31E-102	71.20%	0.462	Y	IPR012336 (G3DSA:3.40.30.GENE3D); IPR013766 (PFAM); PTHR18929:SF81 (PANTHER); PTHR18929 (PANTHER); IPR017937 (PROSITE_PATTERNS); SIGNAL_PEPTIDE_C_REGION (PHOBIUS); SIGNAL_PEPTIDE (PHOBIUS); TRANSMEMBRANE (PHOBIUS); SIGNAL_PEPTIDE_H_REGION (PHOBIUS); SIGNAL_PEPTIDE_N_REGION (PHOBIUS); CYTOPLASMIC_DOMAIN (PHOBIUS); NON_CYTOPLASMIC_DOMAIN (PHOBIUS); IPR012336 (PROSITE_PROFILES); IPR012336 (SUPERFAMILY); TMhelix (TMHMM); TMhelix (TMHMM)
1113	469171_length_1126_cvg_95.8_tip_1_0	9527	low quality protein: galectin-4	376	5.64E-51	50.50%	0.410	Y	IPR001079 (SMART); IPR001079 (SMART); IPR013320 (G3DSA:2.60.120.GENE3D); IPR001079 (PFAM); IPR013320 (G3DSA:2.60.120.GENE3D); PTHR11346 (PANTHER); IPR001079 (PROSITE_PROFILES); IPR001079 (PROSITE_PROFILES); IPR013320 (SUPERFAMILY); IPR013320 (SUPERFAMILY)
1114	469195_length_1127_cvg_4.8_tip_1_1	25	membrane metallo-endopeptidase-like 1-like	376	1.01E-91	84.90%	0.148		IPR018497 (PRINTS); IPR024079 (G3DSA:3.40.390.GENE3D); IPR018497 (PFAM); IPR000718 (PANTHER); PTHR11733:SF113 (PANTHER); TRANSMEMBRANE (PHOBIUS); NON_CYTOPLASMIC_DOMAIN (PHOBIUS); CYTOPLASMIC_DOMAIN (PHOBIUS); TRANSMEMBRANE (PHOBIUS); NON_CYTOPLASMIC_DOMAIN (PHOBIUS); TRANSMEMBRANE (PHOBIUS); CYTOPLASMIC_DOMAIN (PHOBIUS); SSF55486 (SUPERFAMILY); TMhelix (TMHMM); TMhelix (TMHMM)

1165	472193_length_1174_cvg_60.5_tip_1_2	602	mitochondrial acyl carrier protein 1 isoform b	376	1.40E-42	85.30%	0.115	IPR009081 (PFAM); IPR009081 (G3DSA:1.10.1200.GENE3D); PTHR20863 (PANTHER); PTHR20863:SF5 (PANTHER); NON_CYTOPLASMIC_DOMAIN (PHOBIUS); NON_CYTOPLASMIC_DOMAIN (PHOBIUS); TRANSMEMBRANE (PHOBIUS); CYTOPLASMIC_DOMAIN (PHOBIUS); TRANSMEMBRANE (PHOBIUS); IPR003231 (PRODOM); IPR003231 (HAMAP); IPR009081 (PROSITE_PROFILES); IPR009081 (SUPERFAMILY); TMhelix (TMHMM); TMhelix (TMHMM)
1116	469515_length_1132_cvg_4.5_tip_1_1	30	zinc finger protein	377	2.12E-57	66.20%	0.177	IPR015880 (SMART); IPR007087 (PFAM); IPR013087 (G3DSA:3.30.160.GENE3D); PF13465 (PFAM); IPR013087 (G3DSA:3.30.160.GENE3D); IPR013087 (G3DSA:3.30.160.GENE3D); PTHR24409 (PANTHER); IPR007087 (PROSITE_PATTERNS); IPR007087 (PROSITE_PATTERNS); IPR007087 (PROSITE_PATTERNS); IPR007087 (PROSITE_PATTERNS); IPR007087 (PROSITE_PATTERNS); IPR007087 (PROSITE_PATTERNS); TRANSMEMBRANE (PHOBIUS); NON_CYTOPLASMIC_DOMAIN (PHOBIUS); CYTOPLASMIC_DOMAIN (PHOBIUS); IPR007087 (PROSITE_PROFILES); IPR007087 (PROSITE_PROFILES); IPR007087 (PROSITE_PROFILES); IPR007087 (PROSITE_PROFILES); IPR007087 (PROSITE_PROFILES); IPR007087 (PROSITE_PROFILES); SSF57667 (SUPERFAMILY); SSF57667 (SUPERFAMILY); SSF57667 (SUPERFAMILY); SSF57667 (SUPERFAMILY)
1149	471387_length_1162_cvg_66.7_tip_1_2	2739	thioredoxin peroxidase	377	1.67E-118	88.20%	0.107	IPR012336 (G3DSA:3.40.30.GENE3D); IPR019479 (PFAM); IPR000866 (PFAM); PTHR10681 (PANTHER); PTHR10681:SF101 (PANTHER); IPR012336 (PROSITE_PROFILES); IPR012336 (SUPERFAMILY)
1118	469563_length_1133_cvg_5.5_tip_1_3	29	heat shock 70 kda protein 14	378	1.80E-105	61.60%	0.163	IPR013126 (PRINTS); IPR029047 (G3DSA:2.60.34.GENE3D); IPR013126 (PFAM); G3DSA:3.30.420.40 (GENE3D); G3DSA:3.30.420.40 (GENE3D); G3DSA:3.90.640.10 (GENE3D); PTHR19375 (PANTHER); PTHR19375:SF158 (PANTHER); IPR018181 (PROSITE_PATTERNS); SSF53067 (SUPERFAMILY); IPR029047 (SUPERFAMILY)
1120	469627_length_1133_cvg_4.0_tip_1_1	26	fasciclin-3 isoform x2	378	7.93E-29	46.00%	0.241	IPR003599 (SMART); IPR013783 (G3DSA:2.60.40.GENE3D); IPR013783 (G3DSA:2.60.40.GENE3D); IPR013098 (PFAM); IPR013162 (PFAM); PTHR11640 (PANTHER); PTHR11640:SF52 (PANTHER); IPR007110 (PROSITE_PROFILES); IPR007110 (PROSITE_PROFILES); SSF48726 (SUPERFAMILY); SSF48726 (SUPERFAMILY)
1145	471109_length_1158_cvg_52.1_tip_1_5	305	pacifastin light chain	378	3.33E-16	51.40%	0.102	IPR008037 (PFAM); NON_CYTOPLASMIC_DOMAIN (PHOBIUS); NON_CYTOPLASMIC_DOMAIN (PHOBIUS); TRANSMEMBRANE (PHOBIUS); CYTOPLASMIC_DOMAIN (PHOBIUS); TRANSMEMBRANE (PHOBIUS); IPR020862 (PROSITE_PROFILES); IPR020862 (PROSITE_PROFILES); IPR020862 (PROSITE_PROFILES); IPR008037 (SUPERFAMILY); IPR008037 (SUPERFAMILY); IPR008037 (SUPERFAMILY); TMhelix (TMHMM); TMhelix (TMHMM)
1121	469707_length_1135_cvg_7.6_tip_1_0	53	transcription initiation factor tfiid subunit 5	379	1.35E-126	84.70%	0.303	IPR020472 (PRINTS); IPR001680 (SMART); IPR001680 (PFAM); IPR015943 (G3DSA:2.130.10.GENE3D); PTHR19879 (PANTHER); PTHR19879:SF1 (PANTHER); IPR019775 (PROSITE_PATTERNS); IPR019775 (PROSITE_PATTERNS); CYTOPLASMIC_DOMAIN (PHOBIUS); TRANSMEMBRANE (PHOBIUS); TRANSMEMBRANE (PHOBIUS); NON_CYTOPLASMIC_DOMAIN (PHOBIUS); NON_CYTOPLASMIC_DOMAIN (PHOBIUS); IPR001680 (PROSITE_PROFILES); IPR017986 (PROSITE_PROFILES); IPR001680 (PROSITE_PROFILES); IPR001680 (PROSITE_PROFILES); IPR001680 (PROSITE_PROFILES); IPR017986 (SUPERFAMILY); TMhelix (TMHMM); TMhelix (TMHMM)
1122	469837_length_1137_cvg_6.4_tip_1_1	49	kettin protein	379	8.76E-167	75.20%	0.104	IPR003599 (SMART); IPR003598 (SMART); IPR013783 (G3DSA:2.60.40.GENE3D); IPR013098 (PFAM); IPR013783 (G3DSA:2.60.40.GENE3D); IPR013783 (G3DSA:2.60.40.GENE3D); IPR013783 (G3DSA:2.60.40.GENE3D); PTHR19897:SF149 (PANTHER); PTHR19897 (PANTHER); IPR007110 (PROSITE_PROFILES); IPR007110 (PROSITE_PROFILES); IPR007110 (PROSITE_PROFILES); SSF48726 (SUPERFAMILY); SSF48726 (SUPERFAMILY); SSF48726 (SUPERFAMILY); SSF48726 (SUPERFAMILY)

1123	469847_length_1137_cvg_4.1_tip_1_4	32	gastrula zinc finger	379	2.51E-74	56.20%	0.1	IPR015880 (SMART); IPR013087 (G3DSA:3.30.160.GENE3D); IPR013087 (G3DSA:3.30.160.GENE3D); IPR013087 (G3DSA:3.30.160.GENE3D); IPR013087 (G3DSA:3.30.160.GENE3D); IPR013087 (G3DSA:3.30.160.GENE3D); IPR007087 (PFAM); PF13465 (PFAM); PTHR24409 (PANTHER); IPR007087 (PROSITE_PATTERNS); IPR007087 (PROSITE_PATTERNS); IPR007087 (PROSITE_PATTERNS); IPR007087 (PROSITE_PATTERNS); IPR007087 (PROSITE_PATTERNS); IPR007087 (PROSITE_PATTERNS); IPR007087 (PROSITE_PATTERNS); IPR007087 (PROSITE_PATTERNS); IPR007087 (PROSITE_PATTERNS); IPR007087 (PROSITE_PATTERNS); NON_CYTOPLASMIC_DOMAIN (PHOBIUS); TRANSMEMBRANE (PHOBIUS); CYTOPLASMIC_DOMAIN (PHOBIUS); IPR007087 (PROSITE_PROFILES); IPR007087 (PROSITE_PROFILES); IPR007087 (PROSITE_PROFILES); IPR007087 (PROSITE_PROFILES); IPR007087 (PROSITE_PROFILES); IPR007087 (PROSITE_PROFILES); IPR007087 (PROSITE_PROFILES); IPR007087 (PROSITE_PROFILES); IPR007087 (PROSITE_PROFILES); IPR007087 (PROSITE_PROFILES); SSF57667 (SUPERFAMILY); SSF57667 (SUPERFAMILY); SSF57667 (SUPERFAMILY); SSF57667 (SUPERFAMILY); SSF57667 (SUPERFAMILY); SSF57667 (SUPERFAMILY); TMhelix (TMHMM)
1125	469883_length_1137_cvg_5.4_tip_1_0	32	endo-b- -glucanase	379	2.12E-108	72.30%	0.155	IPR012341 (G3DSA:1.50.10.GENE3D); IPR001701 (PFAM); PTHR22298:SF17 (PANTHER); PTHR22298 (PANTHER); IPR018221 (PROSITE_PATTERNS); IPR008928 (SUPERFAMILY)
1126	469947_length_1138_cvg_51.6_tip_1_1	633	cytochrome c oxidase assembly factor 7 homolog	379	3.20E-71	65.70%	0.14	Coil (COILS); IPR006597 (SMART); IPR011990 (G3DSA:1.25.40.GENE3D); IPR006597 (PFAM); PTHR13891 (PANTHER); PTHR13891:SF1 (PANTHER); SSF81901 (SUPERFAMILY)
1128	469983_length_1139_cvg_62.5_tip_1_3	569	scp-like extracellular domain containing protein 1	380	1.63E-43	50.60%	0.244	IPR001283 (PRINTS); IPR014044 (SMART); IPR014044 (G3DSA:3.40.33.GENE3D); IPR014044 (PFAM); IPR001283 (PANTHER); IPR018244 (PROSITE_PATTERNS); NON_CYTOPLASMIC_DOMAIN (PHOBIUS); TRANSMEMBRANE (PHOBIUS); CYTOPLASMIC_DOMAIN (PHOBIUS); IPR014044 (SUPERFAMILY)
1129	470007_length_1139_cvg_4.3_tip_1_1	25	zinc finger protein 282	380	9.42E-15	60.40%	0.103	IPR015880 (SMART); IPR013087 (G3DSA:3.30.160.GENE3D); IPR007087 (PFAM); IPR013087 (G3DSA:3.30.160.GENE3D); PTHR24409 (PANTHER); PTHR24409:SF14 (PANTHER); IPR007087 (PROSITE_PATTERNS); IPR007087 (PROSITE_PATTERNS); IPR007087 (PROSITE_PATTERNS); NON_CYTOPLASMIC_DOMAIN (PHOBIUS); TRANSMEMBRANE (PHOBIUS); CYTOPLASMIC_DOMAIN (PHOBIUS); TRANSMEMBRANE (PHOBIUS); NON_CYTOPLASMIC_DOMAIN (PHOBIUS); TRANSMEMBRANE (PHOBIUS); CYTOPLASMIC_DOMAIN (PHOBIUS); IPR007087 (PROSITE_PROFILES); IPR007087 (PROSITE_PROFILES); IPR007087 (PROSITE_PROFILES); SSF57667 (SUPERFAMILY)
1131	470151_length_1142_cvg_4.2_tip_1_5	25	spondin-2 isoform x1	380	6.56E-137	81.50%	0.116	IPR009465 (PFAM); PTHR11311 (PANTHER); TRANSMEMBRANE (PHOBIUS); NON_CYTOPLASMIC_DOMAIN (PHOBIUS); CYTOPLASMIC_DOMAIN (PHOBIUS); NON_CYTOPLASMIC_DOMAIN (PHOBIUS); TRANSMEMBRANE (PHOBIUS); IPR009465 (PROSITE_PROFILES)
1130	470137_length_1142_cvg_7.2_tip_1_0	35	perlucin-like protein	381	5.40E-22	53.50%	0.940 Y	Coil (COILS); Coil (COILS); IPR001304 (SMART); IPR016186 (G3DSA:3.10.100.GENE3D); IPR001304 (PFAM); PTHR22802 (PANTHER); IPR018378 (PROSITE_PATTERNS); SIGNAL_PEPTIDE_N_REGION (PHOBIUS); SIGNAL_PEPTIDE_H_REGION (PHOBIUS); SIGNAL_PEPTIDE_C_REGION (PHOBIUS); SIGNAL_PEPTIDE (PHOBIUS); NON_CYTOPLASMIC_DOMAIN (PHOBIUS); IPR001304 (PROSITE_PROFILES); SignalP-TM (SIGNALP_GRAM_POSITIVE); SignalP-noTM (SIGNALP_EUK); SignalP-noTM (SIGNALP_GRAM_NEGATIVE); IPR016187 (SUPERFAMILY)
1132	470191_length_1143_cvg_63.0_tip_1_2	2327	steroid dehydrogenase	381	7.55E-116	68.60%	0.25	Coil (COILS); IPR002347 (PRINTS); IPR002198 (PRINTS); IPR002198 (PFAM); IPR016040 (G3DSA:3.40.50.GENE3D); PTHR24316 (PANTHER); PTHR24316:SF68 (PANTHER); CYTOPLASMIC_DOMAIN (PHOBIUS); SIGNAL_PEPTIDE_N_REGION (PHOBIUS); SIGNAL_PEPTIDE_H_REGION (PHOBIUS); TRANSMEMBRANE (PHOBIUS); SIGNAL_PEPTIDE_C_REGION (PHOBIUS); NON_CYTOPLASMIC_DOMAIN (PHOBIUS); TRANSMEMBRANE (PHOBIUS); TRANSMEMBRANE (PHOBIUS); CYTOPLASMIC_DOMAIN (PHOBIUS); SIGNAL_PEPTIDE (PHOBIUS); NON_CYTOPLASMIC_DOMAIN (PHOBIUS); SSF51735 (SUPERFAMILY); TMhelix (TMHMM)

1134	470201_length_1143_cvg_64.3_tip_1_1	3385	ion transport peptide isoform x2	381	1.74E-30	77.70%	0.106	IPR000346 (PRINTS); IPR001166 (PRINTS); IPR001166 (G3DSA:1.10.2010.GENE3D); IPR001166 (PFAM); IPR018251 (PROSITE_PATTERNS); TRANSMEMBRANE (PHOBIUS); TRANSMEMBRANE (PHOBIUS); CYTOPLASMIC_DOMAIN (PHOBIUS); TRANSMEMBRANE (PHOBIUS); CYTOPLASMIC_DOMAIN (PHOBIUS); NON_CYTOPLASMIC_DOMAIN (PHOBIUS); NON_CYTOPLASMIC_DOMAIN (PHOBIUS); SignalP-TM (SIGNALP_GRAM_POSITIVE); IPR001166 (SUPERFAMILY); TMhelix (TMHMM); TMhelix (TMHMM); TMhelix (TMHMM)
1135	470241_length_1143_cvg_4.7_tip_1_3	18	multiple pdz domain	381	3.47E-110	75.80%	0.113	IPR001478 (SMART); IPR001478 (G3DSA:2.30.42.GENE3D); IPR001478 (PFAM); IPR001478 (G3DSA:2.30.42.GENE3D); PTHR19964 (PANTHER); PTHR19964:SF34 (PANTHER); IPR001478 (PROSITE_PROFILES); IPR001478 (PROSITE_PROFILES); IPR001478 (SUPERFAMILY); IPR001478 (SUPERFAMILY)
1136	470241_length_1143_cvg_4.7_tip_1_5	18	multiple pdz domain	381	4.62E-31	91.70%	0.182	IPR001478 (SMART); IPR001478 (G3DSA:2.30.42.GENE3D); IPR001478 (PFAM); PTHR19964 (PANTHER); PTHR19964:SF10 (PANTHER); IPR001478 (PROSITE_PROFILES); IPR001478 (SUPERFAMILY)
1155	471657_length_1166_cvg_8.8_tip_1_3	54	basement membrane-specific heparan sulfate proteoglycan core isoform x4	381	1.10E-86	77.00%	0.14	IPR013106 (SMART); IPR003599 (SMART); IPR003598 (SMART); IPR013783 (G3DSA:2.60.40.GENE3D); IPR013098 (PFAM); IPR013106 (PFAM); IPR013783 (G3DSA:2.60.40.GENE3D); PTHR23279 (PANTHER); TRANSMEMBRANE (PHOBIUS); NON_CYTOPLASMIC_DOMAIN (PHOBIUS); CYTOPLASMIC_DOMAIN (PHOBIUS); IPR007110 (PROSITE_PROFILES); IPR007110 (PROSITE_PROFILES); SSF48726 (SUPERFAMILY); SSF48726 (SUPERFAMILY)
1157	471857_length_1169_cvg_64.4_tip_1_0	651	histone -like	381	2.50E-80	99.20%	0.174	IPR002119 (PRINTS); IPR002119 (SMART); IPR007125 (PFAM); IPR009072 (G3DSA:1.10.20.GENE3D); PTHR23430:SF34 (PANTHER); PTHR23430 (PANTHER); IPR002119 (PROSITE_PATTERNS); IPR009072 (SUPERFAMILY)
1137	470347_length_1145_cvg_9.5_tip_1_0	42	dnaj homolog subfamily c member 30-like	382	1.10E-26	51.20%	0.123	IPR001623 (PRINTS); IPR001623 (SMART); IPR001623 (PFAM); IPR001623 (G3DSA:1.10.287.GENE3D); PTHR24078 (PANTHER); PTHR24078:SF171 (PANTHER); IPR018253 (PROSITE_PATTERNS); CYTOPLASMIC_DOMAIN (PHOBIUS); TRANSMEMBRANE (PHOBIUS); TRANSMEMBRANE (PHOBIUS); NON_CYTOPLASMIC_DOMAIN (PHOBIUS); CYTOPLASMIC_DOMAIN (PHOBIUS); IPR001623 (PROSITE_PROFILES); IPR001623 (SUPERFAMILY); TMhelix (TMHMM); TMhelix (TMHMM)
1166	472231_length_1175_cvg_23.1_tip_1_0	141	i-type lysozyme	382	1.86E-35	64.50%	0.117	IPR008597 (PFAM); IPR008597 (PANTHER); IPR018247 (PROSITE_PATTERNS); CYTOPLASMIC_DOMAIN (PHOBIUS); NON_CYTOPLASMIC_DOMAIN (PHOBIUS); TRANSMEMBRANE (PHOBIUS); TMhelix (TMHMM); TMhelix (TMHMM)
1138	470457_length_1147_cvg_6.5_tip_1_3	26	zinc c2h2 type domain-containing protein	383	6.69E-19	57.90%	0.101	IPR015880 (SMART); IPR007087 (PFAM); IPR013087 (G3DSA:3.30.160.GENE3D); PF13465 (PFAM); PTHR24409:SF14 (PANTHER); PTHR24409 (PANTHER); IPR007087 (PROSITE_PATTERNS); IPR007087 (PROSITE_PATTERNS); IPR007087 (PROSITE_PATTERNS); IPR007087 (PROSITE_PATTERNS); IPR007087 (PROSITE_PROFILES); IPR007087 (PROSITE_PROFILES); IPR007087 (PROSITE_PROFILES); IPR007087 (PROSITE_PROFILES); SSF57667 (SUPERFAMILY); SSF57667 (SUPERFAMILY)
1139	470533_length_1148_cvg_4.8_tip_1_1	25	zinc finger protein 585a-like isoform x1	383	5.02E-23	48.90%	0.098	IPR015880 (SMART); IPR007087 (PFAM); IPR013087 (G3DSA:3.30.160.GENE3D); PF13894 (PFAM); IPR013087 (G3DSA:3.30.160.GENE3D); IPR013087 (G3DSA:3.30.160.GENE3D); PTHR24409 (PANTHER); IPR007087 (PROSITE_PATTERNS); IPR007087 (PROSITE_PATTERNS); IPR007087 (PROSITE_PATTERNS); IPR007087 (PROSITE_PATTERNS); IPR007087 (PROSITE_PROFILES); IPR007087 (PROSITE_PROFILES); IPR007087 (PROSITE_PROFILES); IPR007087 (PROSITE_PROFILES); IPR007087 (PROSITE_PROFILES); SSF57667 (SUPERFAMILY); SSF57667 (SUPERFAMILY); SSF57667 (SUPERFAMILY)
1140	470587_length_1149_cvg_62.8_tip_0_1	725	prolyl 3-hydroxylase 1-like isoform x2	383	1.14E-112	68.80%	0.098	IPR011990 (G3DSA:1.25.40.GENE3D); PTHR13986 (PANTHER); PTHR13986:SF8 (PANTHER)
1141	470639_length_1150_cvg_61.9_tip_1_4	528	nucleoporin seh1 isoform x2	383	0	85.20%	0.339	IPR020472 (PRINTS); IPR001680 (SMART); IPR001680 (PFAM); IPR015943 (G3DSA:2.130.10.GENE3D); PTHR11024:SF3 (PANTHER); PTHR11024 (PANTHER); IPR017986 (PROSITE_PROFILES); IPR001680 (PROSITE_PROFILES); IPR001680 (PROSITE_PROFILES); IPR017986 (PROSITE_PROFILES); IPR017986 (SUPERFAMILY)

1142	470717_length_1151_cvg_3.0_tip_1_4	15	leucine rich repeat protein	384	1.51E-25	54.80%	0.274	PR00019 (PRINTS); SM00365 (SMART); IPR003591 (SMART); SM00364 (SMART); IPR001611 (PFAM); G3DSA:3.80.10.10 (GENE3D); IPR001611 (PFAM); PTHR24365 (PANTHER); IPR001611 (PROSITE_PROFILES); IPR001611 (PROSITE_PROFILES); IPR001611 (PROSITE_PROFILES); IPR001611 (PROSITE_PROFILES); IPR001611 (PROSITE_PROFILES); IPR001611 (PROSITE_PROFILES); IPR001611 (PROSITE_PROFILES); IPR001611 (PROSITE_PROFILES); IPR001611 (PROSITE_PROFILES); IPR001611 (PROSITE_PROFILES); SSF52058 (SUPERFAMILY); TMhelix (TMHMM)
1163	472107_length_1173_cvg_50.8_tip_1_5	357	leucine-rich repeat-containing protein 57	384	7.97E-93	75.70%	0.116	PR00019 (PRINTS); IPR003591 (SMART); IPR025875 (PFAM); IPR001611 (PFAM); G3DSA:3.80.10.10 (GENE3D); G3DSA:3.80.10.10 (GENE3D); PTHR23155 (PANTHER); TRANSMEMBRANE (PHOBIUS); NON_CYTOPLASMIC_DOMAIN (PHOBIUS); CYTOPLASMIC_DOMAIN (PHOBIUS); IPR001611 (PROSITE_PROFILES); IPR001611 (PROSITE_PROFILES); IPR001611 (PROSITE_PROFILES); IPR001611 (PROSITE_PROFILES); IPR001611 (PROSITE_PROFILES); IPR001611 (PROSITE_PROFILES); IPR001611 (PROSITE_PROFILES); SSF52058 (SUPERFAMILY); TMhelix (TMHMM)
1143	470847_length_1154_cvg_3.5_tip_1_0	20	vacuolar protein sorting-associated protein 4a-like	385	0	89.30%	0.121	IPR007330 (SMART); IPR003593 (SMART); IPR007330 (G3DSA:1.20.58.GENE3D); IPR003959 (PFAM); G3DSA:1.10.8.60 (GENE3D); IPR007330 (PFAM); IPR027417 (G3DSA:3.40.50.GENE3D); PTHR23074 (PANTHER); PTHR23074:SF72 (PANTHER); IPR003960 (PROSITE_PATTERNS); IPR007330 (SUPERFAMILY); IPR027417 (SUPERFAMILY)
1144	471095_length_1158_cvg_7.2_tip_1_5	36	von willebrand factor like 2	386	4.31E-09	42.20%	0.107	no IPS match
1147	471201_length_1159_cvg_8.3_tip_1_1	39	trypsin-like serine proteinase 2	386	1.88E-61	56.70%	0.824 Y	IPR001314 (PRINTS); IPR001254 (SMART); G3DSA:2.40.10.10 (GENE3D); G3DSA:2.40.10.10 (GENE3D); IPR001254 (PFAM); G3DSA:2.40.10.10 (GENE3D); PTHR24276 (PANTHER); IPR018114 (PROSITE_PATTERNS); IPR018114 (PROSITE_PATTERNS); TRANSMEMBRANE (PHOBIUS); NON_CYTOPLASMIC_DOMAIN (PHOBIUS); CYTOPLASMIC_DOMAIN (PHOBIUS); SIGNAL_PEPTIDE_H_REGION (PHOBIUS); SIGNAL_PEPTIDE_N_REGION (PHOBIUS); SIGNAL_PEPTIDE (PHOBIUS); SIGNAL_PEPTIDE_C_REGION (PHOBIUS); IPR001254 (PROSITE_PROFILES); SignalP-noTM (SIGNALP_EUK); IPR009003 (SUPERFAMILY); TMhelix (TMHMM)
1148	471203_length_1159_cvg_24.0_tip_1_2	139	wd repeat domain-containing protein 83	386	4.26E-100	78.70%	0.139	IPR020472 (PRINTS); IPR001680 (SMART); IPR015943 (G3DSA:2.130.10.GENE3D); IPR001680 (PFAM); PTHR22842 (PANTHER); IPR019775 (PROSITE_PATTERNS); CYTOPLASMIC_DOMAIN (PHOBIUS); TRANSMEMBRANE (PHOBIUS); TRANSMEMBRANE (PHOBIUS); CYTOPLASMIC_DOMAIN (PHOBIUS); NON_CYTOPLASMIC_DOMAIN (PHOBIUS); NON_CYTOPLASMIC_DOMAIN (PHOBIUS); TRANSMEMBRANE (PHOBIUS); IPR001680 (PROSITE_PROFILES); IPR001680 (PROSITE_PROFILES); IPR017986 (PROSITE_PROFILES); IPR017986 (SUPERFAMILY); TMhelix (TMHMM); TMhelix (TMHMM)
1162	472025_length_1172_cvg_67.6_tip_1_3	557	glutaminyl-peptide cyclotransferase-like	387	2.60E-116	68.90%	0.645 Y	G3DSA:3.40.630.10 (GENE3D); IPR007484 (PFAM); PTHR12283 (PANTHER); NON_CYTOPLASMIC_DOMAIN (PHOBIUS); TRANSMEMBRANE (PHOBIUS); CYTOPLASMIC_DOMAIN (PHOBIUS); SignalP-noTM (SIGNALP_EUK); SSF53187 (SUPERFAMILY)
1150	471555_length_1164_cvg_4.6_tip_1_0	33	sphingomyelin phosphodiesterase	388	7.06E-101	65.60%	0.261	IPR008139 (SMART); IPR011001 (G3DSA:1.10.225.GENE3D); IPR029052 (G3DSA:3.60.21.GENE3D); PTHR10340:SF13 (PANTHER); PTHR10340 (PANTHER); NON_CYTOPLASMIC_DOMAIN (PHOBIUS); CYTOPLASMIC_DOMAIN (PHOBIUS); TRANSMEMBRANE (PHOBIUS); IPR008139 (PROSITE_PROFILES); IPR011001 (SUPERFAMILY); IPR029052 (SUPERFAMILY); TMhelix (TMHMM)
1153	471609_length_1165_cvg_3.6_tip_1_2	16	partial	388	1.29E-146	99.50%	0.101	IPR000917 (PFAM); IPR017849 (G3DSA:3.40.720.GENE3D); PTHR10342 (PANTHER); PTHR10342:SF22 (PANTHER); IPR024607 (PROSITE_PATTERNS); IPR024607 (PROSITE_PATTERNS); IPR017850 (SUPERFAMILY)
1154	471641_length_1166_cvg_3.0_tip_1_2	22	hyaluronidase precursor	388	9.01E-83	59.90%	0.108	Coil (COILS); IPR001329 (PRINTS); IPR018155 (PRINTS); IPR013785 (G3DSA:3.20.20.GENE3D); IPR018155 (PFAM); PTHR11769:SF8 (PANTHER); IPR018155 (PANTHER); IPR017853 (SUPERFAMILY)
1152	471607_length_1165_cvg_58.7_tip_1_0	516	tropinone reductase 2-like	389	2.77E-122	76.40%	0.103	IPR002347 (PRINTS); IPR002198 (PRINTS); IPR013968 (SMART); IPR002198 (PFAM); IPR016040 (G3DSA:3.40.50.GENE3D); PTHR24322:SF111 (PANTHER); PTHR24322 (PANTHER); IPR020904 (PROSITE_PATTERNS); NON_CYTOPLASMIC_DOMAIN (PHOBIUS); CYTOPLASMIC_DOMAIN (PHOBIUS); TRANSMEMBRANE (PHOBIUS); SSF51735 (SUPERFAMILY)
1158	471887_length_1170_cvg_13.1_tip_1_0	57	alpha-mannosidase partial	390	3.00E-156	72.90%	0.109	G3DSA:2.70.98.30 (GENE3D); IPR011682 (PFAM); PTHR11607:SF22 (PANTHER); PTHR11607 (PANTHER); IPR011013 (SUPERFAMILY)

1159	471903_length_1170_cvg_13.8_tip_1_3	59	fk506-binding protein 2	390	3.52E-66	84.70%	0.247		G3DSA:3.10.50.40 (GENE3D); IPR001179 (PFAM); PTHR10516:SF134 (PANTHER); IPR023566 (PANTHER); CYTOPLASMIC_DOMAIN (PHOBIUS); NON_CYTOPLASMIC_DOMAIN (PHOBIUS); CYTOPLASMIC_DOMAIN (PHOBIUS); NON_CYTOPLASMIC_DOMAIN (PHOBIUS); TRANSMEMBRANE (PHOBIUS); CYTOPLASMIC_DOMAIN (PHOBIUS); NON_CYTOPLASMIC_DOMAIN (PHOBIUS); TRANSMEMBRANE (PHOBIUS); TRANSMEMBRANE (PHOBIUS); TRANSMEMBRANE (PHOBIUS); TRANSMEMBRANE (PHOBIUS); IPR001179 (PROSITE_PROFILES); SSF54534 (SUPERFAMILY); TMhelix (TMHMM); TMhelix (TMHMM); TMhelix (TMHMM); TMhelix (TMHMM)
1160	471913_length_1170_cvg_5.8_tip_1_1	32	dipeptidyl peptidase 9	390	0	82.30%	0.126		IPR002469 (PFAM); IPR029058 (G3DSA:3.40.50.GENE3D); IPR001375 (PFAM); IPR002469 (G3DSA:2.140.10.GENE3D); PTHR11731 (PANTHER); PTHR11731:SF110 (PANTHER); IPR029058 (SUPERFAMILY); SSF82171 (SUPERFAMILY)
1161	472003_length_1172_cvg_23.2_tip_1_3	72	sparc	391	1.62E-115	77.40%	0.206		Coil (COILS); Coil (COILS); IPR003645 (SMART); G3DSA:3.30.60.30 (GENE3D); IPR019577 (PFAM); IPR011992 (G3DSA:1.10.238.GENE3D); PTHR13866:SF14 (PANTHER); PTHR13866 (PANTHER); IPR001999 (PROSITE_PATTERNS); IPR018247 (PROSITE_PATTERNS); TRANSMEMBRANE (PHOBIUS); NON_CYTOPLASMIC_DOMAIN (PHOBIUS); CYTOPLASMIC_DOMAIN (PHOBIUS); SSF47473 (SUPERFAMILY); SSF100895 (SUPERFAMILY); TMhelix (TMHMM)
1164	472175_length_1174_cvg_4.6_tip_0_1	25	zinc finger protein 2 homolog	391	3.69E-143	63.60%	0.107		IPR015880 (SMART); IPR013087 (G3DSA:3.30.160.GENE3D); IPR013087 (G3DSA:3.30.160.GENE3D); PF13465 (PFAM); IPR013087 (G3DSA:3.30.160.GENE3D); IPR013087 (G3DSA:3.30.160.GENE3D); IPR013087 (G3DSA:3.30.160.GENE3D); IPR007087 (PFAM); PTHR24409 (PANTHER); IPR007087 (PROSITE_PATTERNS); IPR007087 (PROSITE_PATTERNS); IPR007087 (PROSITE_PATTERNS); IPR007087 (PROSITE_PATTERNS); IPR007087 (PROSITE_PATTERNS); IPR007087 (PROSITE_PROFILES); IPR007087 (PROSITE_PROFILES); IPR007087 (PROSITE_PROFILES); IPR007087 (PROSITE_PROFILES); IPR007087 (PROSITE_PROFILES); IPR007087 (PROSITE_PROFILES); IPR007087 (PROSITE_PROFILES); IPR007087 (PROSITE_PROFILES); SSF57667 (SUPERFAMILY); SSF57667 (SUPERFAMILY); SSF57667 (SUPERFAMILY); SSF57667 (SUPERFAMILY)
1167	472299_length_1176_cvg_12.0_tip_1_3	68	calbindin- partial	392	1.90E-56	82.50%	0.142		IPR002048 (SMART); IPR011992 (G3DSA:1.10.238.GENE3D); IPR002048 (PFAM); IPR014710 (G3DSA:2.60.120.GENE3D); PTHR19972 (PANTHER); IPR018247 (PROSITE_PATTERNS); IPR018247 (PROSITE_PATTERNS); TRANSMEMBRANE (PHOBIUS); NON_CYTOPLASMIC_DOMAIN (PHOBIUS); CYTOPLASMIC_DOMAIN (PHOBIUS); IPR002048 (PROSITE_PROFILES); IPR002048 (PROSITE_PROFILES); SSF47473 (SUPERFAMILY); TMhelix (TMHMM)
1168	472397_length_1178_cvg_10.0_tip_1_0	27	fas1_scham ame: full=fasciclin-1 ame: full=fasciclin i short=fas i short=fcn flags: precursor	393	5.52E-104	63.60%	0.105		IPR000782 (SMART); IPR000782 (G3DSA:2.30.180.GENE3D); IPR000782 (PFAM); PTHR10900 (PANTHER); PTHR10900:SF73 (PANTHER); IPR000782 (PROSITE_PROFILES); IPR000782 (PROSITE_PROFILES); IPR000782 (SUPERFAMILY); IPR000782 (SUPERFAMILY)
1169	472399_length_1178_cvg_63.8_tip_1_0	750127	serine protease 27-like	393	4.81E-42	55.00%	0.484	Y	IPR001314 (PRINTS); IPR001254 (SMART); IPR001254 (PFAM); G3DSA:2.40.10.10 (GENE3D); G3DSA:2.40.10.10 (GENE3D); PTHR24268 (PANTHER); IPR018114 (PROSITE_PATTERNS); IPR018114 (PROSITE_PATTERNS); NON_CYTOPLASMIC_DOMAIN (PHOBIUS); SIGNAL_PEPTIDE_C_REGION (PHOBIUS); SIGNAL_PEPTIDE (PHOBIUS); SIGNAL_PEPTIDE_H_REGION (PHOBIUS); SIGNAL_PEPTIDE_N_REGION (PHOBIUS); IPR001254 (PROSITE_PROFILES); SignalP-noTM (SIGNALP_EUK); IPR009003 (SUPERFAMILY)
1170	472427_length_1179_cvg_50.4_tip_1_4	321	meprin a subunit beta	393	3.44E-51	58.50%	0.196		IPR001506 (PRINTS); IPR006026 (SMART); IPR001506 (PFAM); IPR024079 (G3DSA:3.40.390.GENE3D); PTHR10127 (PANTHER); SSF55486 (SUPERFAMILY); TMhelix (TMHMM); TMhelix (TMHMM)
1171	472541_length_1181_cvg_3.0_tip_1_2	18	plasma alpha-l-fucosidase	393	7.44E-63	81.40%	0.768	Y	IPR016286 (PRINTS); IPR000933 (SMART); IPR000933 (PFAM); IPR013781 (G3DSA:3.20.20.GENE3D); IPR000933 (PANTHER); PTHR10030:SF26 (PANTHER); TRANSMEMBRANE (PHOBIUS); CYTOPLASMIC_DOMAIN (PHOBIUS); SIGNAL_PEPTIDE_C_REGION (PHOBIUS); NON_CYTOPLASMIC_DOMAIN (PHOBIUS); SIGNAL_PEPTIDE_N_REGION (PHOBIUS); SIGNAL_PEPTIDE (PHOBIUS); SIGNAL_PEPTIDE_H_REGION (PHOBIUS); SignalP-TM (SIGNALP_EUK); IPR017853 (SUPERFAMILY)

1172	472661_length_1183_cvg_5.2_tip_1_4	32	krueppel-like factor 6 isoform x1	394	1.78E-109	63.30%	0.105		IPR015880 (SMART); IPR013087 (G3DSA:3.30.160.GENE3D); IPR013087 (G3DSA:3.30.160.GENE3D); PF13465 (PFAM); PTHR23223:SF132 (PANTHER); PTHR23223 (PANTHER); IPR007087 (PROSITE_PATTERNS); IPR007087 (PROSITE_PATTERNS); IPR007087 (PROSITE_PATTERNS); IPR007087 (PROSITE_PROFILES); IPR007087 (PROSITE_PROFILES); IPR007087 (PROSITE_PROFILES); SSF57667 (SUPERFAMILY); SSF57667 (SUPERFAMILY)
1173	472693_length_1184_cvg_12.7_tip_1_5	85	zinc finger protein	394	1.38E-41	54.30%	0.113		IPR015880 (SMART); IPR007087 (PFAM); IPR013087 (G3DSA:3.30.160.GENE3D); PF13894 (PFAM); PF13465 (PFAM); IPR013087 (G3DSA:3.30.160.GENE3D); IPR013087 (G3DSA:3.30.160.GENE3D); PTHR24409 (PANTHER); PTHR24409:SF14 (PANTHER); IPR007087 (PROSITE_PATTERNS); IPR007087 (PROSITE_PATTERNS); IPR007087 (PROSITE_PATTERNS); IPR007087 (PROSITE_PATTERNS); IPR007087 (PROSITE_PATTERNS); IPR007087 (PROSITE_PATTERNS); IPR007087 (PROSITE_PATTERNS); IPR007087 (PROSITE_PATTERNS); TRANSMEMBRANE (PHOBIUS); NON_CYTOPLASMIC_DOMAIN (PHOBIUS); CYTOPLASMIC_DOMAIN (PHOBIUS); IPR007087 (PROSITE_PROFILES); IPR007087 (PROSITE_PROFILES); IPR007087 (PROSITE_PROFILES); IPR007087 (PROSITE_PROFILES); IPR007087 (PROSITE_PROFILES); IPR007087 (PROSITE_PROFILES); IPR007087 (PROSITE_PROFILES); IPR007087 (PROSITE_PROFILES); SSF57667 (SUPERFAMILY); SSF57667 (SUPERFAMILY); SSF57667 (SUPERFAMILY); SSF57667 (SUPERFAMILY); SSF57667 (SUPERFAMILY)
1175	472839_length_1186_cvg_5.8_tip_1_5	30	accumulation-associated protein	395	1.64E-11	64.40%	0.109		CYTOPLASMIC_DOMAIN (PHOBIUS); NON_CYTOPLASMIC_DOMAIN (PHOBIUS); TRANSMEMBRANE (PHOBIUS)
1174	472839_length_1186_cvg_5.8_tip_1_0	30	collagen alpha-1 chain	396	1.61E-14	69.40%	0.436	Y	IPR008160 (PFAM); TRANSMEMBRANE (PHOBIUS); NON_CYTOPLASMIC_DOMAIN (PHOBIUS); NON_CYTOPLASMIC_DOMAIN (PHOBIUS); TRANSMEMBRANE (PHOBIUS); CYTOPLASMIC_DOMAIN (PHOBIUS); NON_CYTOPLASMIC_DOMAIN (PHOBIUS); TRANSMEMBRANE (PHOBIUS); TRANSMEMBRANE (PHOBIUS); CYTOPLASMIC_DOMAIN (PHOBIUS); TMhelix (TMHMM); TMhelix (TMHMM); TMhelix (TMHMM)
1176	472871_length_1187_cvg_63.3_tip_0_1	2559	universal minicircle sequence binding protein	396	4.61E-26	52.70%	0.1		Coil (COILS); Coil (COILS); IPR001878 (SMART); IPR001878 (PFAM); IPR001878 (G3DSA:4.10.60.GENE3D); IPR001878 (G3DSA:4.10.60.GENE3D); PTHR23002 (PANTHER); IPR001878 (PROSITE_PROFILES); IPR001878 (PROSITE_PROFILES); IPR001878 (PROSITE_PROFILES); IPR001878 (PROSITE_PROFILES); IPR001878 (PROSITE_PROFILES); IPR001878 (PROSITE_PROFILES); IPR001878 (SUPERFAMILY); IPR001878 (SUPERFAMILY); IPR001878 (SUPERFAMILY)
1194	474337_length_1214_cvg_4.9_tip_1_1	35	transforming growth factor-beta-induced protein ig-h3-like	396	6.18E-41	52.60%	0.796	Y	IPR000782 (SMART); IPR000782 (PFAM); IPR000782 (G3DSA:2.30.180.GENE3D); IPR000782 (G3DSA:2.30.180.GENE3D); PTHR10900 (PANTHER); NON_CYTOPLASMIC_DOMAIN (PHOBIUS); NON_CYTOPLASMIC_DOMAIN (PHOBIUS); SIGNAL_PEPTIDE_H_REGION (PHOBIUS); SIGNAL_PEPTIDE (PHOBIUS); CYTOPLASMIC_DOMAIN (PHOBIUS); SIGNAL_PEPTIDE_N_REGION (PHOBIUS); TRANSMEMBRANE (PHOBIUS); SIGNAL_PEPTIDE_C_REGION (PHOBIUS); TRANSMEMBRANE (PHOBIUS); IPR000782 (PROSITE_PROFILES); IPR000782 (PROSITE_PROFILES); SignalP-noTM (SIGNALP_EUK); IPR000782 (SUPERFAMILY); IPR000782 (SUPERFAMILY); TMhelix (TMHMM)
1208	475311_length_1233_cvg_80.6_tip_1_0	5683	gamma-glutamyltranspeptidase 1	396	6.05E-114	67.50%	0.1		IPR000101 (PRINTS); IPR000101 (PFAM); PTHR11686:SF15 (PANTHER); IPR000101 (PANTHER); IPR029055 (SUPERFAMILY)
1177	473185_length_1192_cvg_4.3_tip_1_1	27	hypothetical protein DAPPUDRAFT_300634	397	8.75E-47	63.20%	0.265		IPR001916 (PRINTS); IPR001916 (SMART); IPR001916 (PFAM); G3DSA:1.10.530.10 (GENE3D); PTHR11407 (PANTHER); IPR019799 (PROSITE_PATTERNS); NON_CYTOPLASMIC_DOMAIN (PHOBIUS); TRANSMEMBRANE (PHOBIUS); CYTOPLASMIC_DOMAIN (PHOBIUS); IPR001916 (PROSITE_PROFILES); IPR023346 (SUPERFAMILY)
1192	474239_length_1212_cvg_47.7_tip_1_2	302	thioredoxin-like protein 1	397	9.20E-154	81.30%	0.133		PR00421 (PRINTS); IPR013766 (PFAM); IPR012336 (G3DSA:3.40.30.GENE3D); IPR010400 (PFAM); IPR010400 (G3DSA:2.60.120.GENE3D); IPR005746 (PANTHER); IPR017937 (PROSITE_PATTERNS); IPR010400 (PROSITE_PROFILES); IPR012336 (PROSITE_PROFILES); IPR008979 (SUPERFAMILY); IPR012336 (SUPERFAMILY)
1178	473259_length_1193_cvg_6.6_tip_1_0	37	protein spaetzle	398	1.39E-28	54.40%	0.169		PTHR23199 (PANTHER); IPR029034 (SUPERFAMILY)
1179	473299_length_1194_cvg_2.0_tip_1_3	12	sel1 repeat family protein	398	1.38E-144	98.70%	0.109		IPR006597 (SMART); IPR011990 (G3DSA:1.25.40.GENE3D); IPR006597 (PFAM); PTHR11102 (PANTHER); NON_CYTOPLASMIC_DOMAIN (PHOBIUS); TRANSMEMBRANE (PHOBIUS); CYTOPLASMIC_DOMAIN (PHOBIUS); SSF81901 (SUPERFAMILY)

1180	473371_length_1195_cvg_14.7_tip_1_5	79	low quality protein: agrin	398	8.47E-40	52.10%	0.400	Y	IPR002350 (SMART); G3DSA:2.40.50.120 (GENE3D); IPR004850 (PFAM); IPR002350 (PFAM); G3DSA:3.30.60.30 (GENE3D); PTHR10913 (PANTHER); PTHR10913:SF45 (PANTHER); IPR002350 (PROSITE_PROFILES); IPR004850 (PROSITE_PROFILES); SSF100895 (SUPERFAMILY); IPR008993 (SUPERFAMILY); TMhelix (TMHMM)
1181	473465_length_1197_cvg_8.2_tip_1_5	53	endonuclease mitochondrial	399	9.97E-127	84.00%	0.156		IPR020821 (SMART); IPR001604 (SMART); IPR020821 (G3DSA:3.40.570.GENE3D); IPR001604 (PFAM); PTHR13966 (PANTHER); IPR018524 (PROSITE_PATTERNS); SSF54060 (SUPERFAMILY)
1182	473507_length_1198_cvg_57.2_tip_1_3	584	neuferricin -like protein	400	3.09E-81	67.30%	0.326		IPR001199 (PFAM); IPR001199 (G3DSA:3.10.120.GENE3D); PTHR10281:SF4 (PANTHER); PTHR10281 (PANTHER); NON_CYTOPLASMIC_DOMAIN (PHOBIUS); TRANSMEMBRANE (PHOBIUS); CYTOPLASMIC_DOMAIN (PHOBIUS); NON_CYTOPLASMIC_DOMAIN (PHOBIUS); TRANSMEMBRANE (PHOBIUS); IPR001199 (SUPERFAMILY)
1183	473565_length_1199_cvg_5.7_tip_1_3	24	alpha- -mannosyl-glycoprotein 4-beta-n-acetylglucosaminyltransferase partial	400	6.77E-41	55.30%	0.164		IPR006759 (PFAM); PTHR12062:SF5 (PANTHER); IPR006759 (PANTHER); CYTOPLASMIC_DOMAIN (PHOBIUS); TRANSMEMBRANE (PHOBIUS); NON_CYTOPLASMIC_DOMAIN (PHOBIUS); NON_CYTOPLASMIC_DOMAIN (PHOBIUS); TRANSMEMBRANE (PHOBIUS); TMhelix (TMHMM)
1184	473571_length_1199_cvg_63.0_tip_1_0	15319	calreticulin	400	0	83.70%	0.323		Coil (COILS); IPR001580 (PRINTS); IPR009169 (PIRSF); IPR013320 (G3DSA:2.60.120.GENE3D); IPR001580 (PFAM); PTHR11073:SF8 (PANTHER); IPR001580 (PANTHER); IPR018124 (PROSITE_PATTERNS); IPR018124 (PROSITE_PATTERNS); IPR018124 (PROSITE_PATTERNS); IPR018124 (PROSITE_PATTERNS); IPR018124 (PROSITE_PATTERNS); NON_CYTOPLASMIC_DOMAIN (PHOBIUS); TRANSMEMBRANE (PHOBIUS); CYTOPLASMIC_DOMAIN (PHOBIUS); IPR009033 (SUPERFAMILY); IPR013320 (SUPERFAMILY); TMhelix (TMHMM)
1185	473741_length_1202_cvg_3.1_tip_1_5	19	early growth response	400	4.58E-83	70.40%	0.099		IPR015880 (SMART); IPR013087 (G3DSA:3.30.160.GENE3D); PF13465 (PFAM); IPR013087 (G3DSA:3.30.160.GENE3D); PTHR23223:SF80 (PANTHER); PTHR23223 (PANTHER); IPR007087 (PROSITE_PATTERNS); IPR007087 (PROSITE_PATTERNS); IPR007087 (PROSITE_PATTERNS); IPR007087 (PROSITE_PROFILES); IPR007087 (PROSITE_PROFILES); IPR007087 (PROSITE_PROFILES); SSF57667 (SUPERFAMILY); SSF57667 (SUPERFAMILY)
1186	473803_length_1203_cvg_85.9_tip_1_0	98515	transmembrane protease serine 4	401	1.78E-41	52.40%	0.496	Y	IPR001314 (PRINTS); IPR001254 (SMART); G3DSA:2.40.10.10 (GENE3D); G3DSA:2.40.10.10 (GENE3D); IPR001254 (PFAM); G3DSA:2.40.10.10 (GENE3D); PTHR24260 (PANTHER); IPR018114 (PROSITE_PATTERNS); IPR018114 (PROSITE_PATTERNS); SIGNAL_PEPTIDE_H_REGION (PHOBIUS); SIGNAL_PEPTIDE_N_REGION (PHOBIUS); SIGNAL_PEPTIDE (PHOBIUS); NON_CYTOPLASMIC_DOMAIN (PHOBIUS); SIGNAL_PEPTIDE_C_REGION (PHOBIUS); IPR001254 (PROSITE_PROFILES); IPR009003 (SUPERFAMILY)
1187	473853_length_1205_cvg_4.7_tip_1_2	27	melanopsin-like isoform x1	401	1.04E-74	60.00%	0.106		IPR000276 (PRINTS); IPR000276 (PFAM); G3DSA:1.20.1070.10 (GENE3D); PTHR24242:SF154 (PANTHER); PTHR24242 (PANTHER); TRANSMEMBRANE (PHOBIUS); NON_CYTOPLASMIC_DOMAIN (PHOBIUS); NON_CYTOPLASMIC_DOMAIN (PHOBIUS); TRANSMEMBRANE (PHOBIUS); TRANSMEMBRANE (PHOBIUS); NON_CYTOPLASMIC_DOMAIN (PHOBIUS); TRANSMEMBRANE (PHOBIUS); TRANSMEMBRANE (PHOBIUS); CYTOPLASMIC_DOMAIN (PHOBIUS); CYTOPLASMIC_DOMAIN (PHOBIUS); CYTOPLASMIC_DOMAIN (PHOBIUS); IPR017452 (PROSITE_PROFILES); SSF81321 (SUPERFAMILY); TMhelix (TMHMM); TMhelix (TMHMM); TMhelix (TMHMM); TMhelix (TMHMM); TMhelix (TMHMM)
1188	473919_length_1206_cvg_6.4_tip_1_0	32	nardilysin-like isoform x2	402	5.11E-98	66.10%	0.15		IPR011237 (G3DSA:3.30.830.GENE3D); IPR007863 (PFAM); IPR011237 (G3DSA:3.30.830.GENE3D); PTHR11851 (PANTHER); PTHR11851:SF108 (PANTHER); IPR011249 (SUPERFAMILY); IPR011249 (SUPERFAMILY)
1189	473961_length_1206_cvg_6.6_tip_1_1	49	endoplasmic reticulum resident protein 44 isoform x2	402	0	81.40%	0.154		IPR012336 (G3DSA:3.40.30.GENE3D); IPR013766 (PFAM); IPR012336 (G3DSA:3.40.30.GENE3D); PF13848 (PFAM); PTHR18929 (PANTHER); PTHR18929:SF49 (PANTHER); IPR012336 (PROSITE_PROFILES); IPR012336 (SUPERFAMILY); IPR012336 (SUPERFAMILY); IPR012336 (SUPERFAMILY)
1190	473987_length_1207_cvg_7.3_tip_1_4	38	transcription termination factor rho	402	3.22E-09	62.90%	0.108		TRANSMEMBRANE (PHOBIUS); CYTOPLASMIC_DOMAIN (PHOBIUS); TRANSMEMBRANE (PHOBIUS); NON_CYTOPLASMIC_DOMAIN (PHOBIUS); CYTOPLASMIC_DOMAIN (PHOBIUS)

1214	475455_length_1236_cvg_8.2_tip_1_4	49	ras-related protein rab-4b	403	5.25E-135	94.30%	0.236	IPR001806 (PRINTS); IPR003579 (SMART); IPR024156 (SMART); IPR003578 (SMART); IPR020849 (SMART); IPR002041 (SMART); IPR001806 (PFAM); IPR027417 (G3DSA:3.40.50.GENE3D); IPR005225 (TIGRFAM); PTHR24073 (PANTHER); PTHR24073:SF387 (PANTHER); TRANSMEMBRANE (PHOBIUS); CYTOPLASMIC_DOMAIN (PHOBIUS); TRANSMEMBRANE (PHOBIUS); NON_CYTOPLASMIC_DOMAIN (PHOBIUS); NON_CYTOPLASMIC_DOMAIN (PHOBIUS); CYTOPLASMIC_DOMAIN (PHOBIUS); TRANSMEMBRANE (PHOBIUS); PS51419 (PROSITE_PROFILES); IPR027417 (SUPERFAMILY); TMhelix (TMHMM)
1191	474127_length_1210_cvg_90.4_tip_1_0	414780	suppressor of tumorigenicity 14 protein homolog	404	4.56E-43	50.50%	0.316	IPR001314 (PRINTS); IPR002172 (SMART); IPR001254 (SMART); G3DSA:2.40.10.10 (GENE3D); G3DSA:2.40.10.10 (GENE3D); IPR002172 (PFAM); IPR002172 (G3DSA:4.10.400.GENE3D); IPR001254 (PFAM); G3DSA:2.40.10.10 (GENE3D); PTHR24256 (PANTHER); IPR018114 (PROSITE_PATTERNS); IPR023415 (PROSITE_PATTERNS); NON_CYTOPLASMIC_DOMAIN (PHOBIUS); CYTOPLASMIC_DOMAIN (PHOBIUS); TRANSMEMBRANE (PHOBIUS); IPR001254 (PROSITE_PROFILES); IPR002172 (PROSITE_PROFILES); IPR009003 (SUPERFAMILY); IPR002172 (SUPERFAMILY)
1193	474273_length_1212_cvg_15.5_tip_1_4	61	39s ribosomal protein mitochondrial	404	2.93E-101	70.30%	0.312	IPR008914 (G3DSA:3.90.280.GENE3D); IPR008914 (PFAM); PTHR11362:SF16 (PANTHER); PTHR11362 (PANTHER); IPR008914 (SUPERFAMILY)
1207	475245_length_1231_cvg_16.6_tip_1_3	70	dehydrogenase reductase sdr family member 7	404	7.23E-109	67.50%	0.141	IPR002347 (PRINTS); IPR002198 (PRINTS); IPR002198 (PFAM); IPR016040 (G3DSA:3.40.50.GENE3D); PTHR24322:SF31 (PANTHER); PTHR24322 (PANTHER); TRANSMEMBRANE (PHOBIUS); CYTOPLASMIC_DOMAIN (PHOBIUS); CYTOPLASMIC_DOMAIN (PHOBIUS); NON_CYTOPLASMIC_DOMAIN (PHOBIUS); TRANSMEMBRANE (PHOBIUS); SSF51735 (SUPERFAMILY); TMhelix (TMHMM); TMhelix (TMHMM)
1195	474493_length_1217_cvg_3.4_tip_1_2	29	krueppel-like factor 13	405	1.46E-50	84.30%	0.247	IPR015880 (SMART); IPR013087 (G3DSA:3.30.160.GENE3D); PF13465 (PFAM); IPR013087 (G3DSA:3.30.160.GENE3D); PTHR23223 (PANTHER); IPR007087 (PROSITE_PATTERNS); IPR007087 (PROSITE_PATTERNS); CYTOPLASMIC_DOMAIN (PHOBIUS); TRANSMEMBRANE (PHOBIUS); NON_CYTOPLASMIC_DOMAIN (PHOBIUS); IPR007087 (PROSITE_PROFILES); IPR007087 (PROSITE_PROFILES); SSF57667 (SUPERFAMILY)
1200	474927_length_1225_cvg_62.8_tip_1_1	1386	glutaredoxin- partial	405	3.20E-143	78.20%	0.238	IPR012336 (G3DSA:3.40.30.GENE3D); IPR013766 (PFAM); IPR002109 (PFAM); IPR012336 (G3DSA:3.40.30.GENE3D); IPR004480 (TIGRFAM); PTHR10293:SF40 (PANTHER); IPR004480 (PANTHER); IPR002109 (PROSITE_PROFILES); IPR002109 (PROSITE_PROFILES); IPR012336 (PROSITE_PROFILES); IPR012336 (SUPERFAMILY); IPR012336 (SUPERFAMILY); IPR012336 (SUPERFAMILY)
1196	474739_length_1222_cvg_3.4_tip_1_1	18	zinc finger protein 239-like	407	2.55E-29	47.40%	0.109	IPR015880 (SMART); IPR007087 (PFAM); IPR013087 (G3DSA:3.30.160.GENE3D); PF13465 (PFAM); IPR013087 (G3DSA:3.30.160.GENE3D); PF13894 (PFAM); IPR013087 (G3DSA:3.30.160.GENE3D); PTHR24409:SF14 (PANTHER); PTHR24409 (PANTHER); IPR007087 (PROSITE_PATTERNS); IPR007087 (PROSITE_PATTERNS); IPR007087 (PROSITE_PATTERNS); IPR007087 (PROSITE_PATTERNS); IPR007087 (PROSITE_PATTERNS); IPR007087 (PROSITE_PATTERNS); IPR007087 (PROSITE_PATTERNS); IPR007087 (PROSITE_PROFILES); IPR007087 (PROSITE_PROFILES); IPR007087 (PROSITE_PROFILES); IPR007087 (PROSITE_PROFILES); IPR007087 (PROSITE_PROFILES); IPR007087 (PROSITE_PROFILES); IPR007087 (PROSITE_PROFILES); SSF57667 (SUPERFAMILY); SSF57667 (SUPERFAMILY); SSF57667 (SUPERFAMILY); SSF57667 (SUPERFAMILY)
1197	474779_length_1223_cvg_12.2_tip_1_5	55	uv radiation resistance-associated gene protein	407	9.09E-40	66.40%	0.097	Coil (COILS); PTHR15157 (PANTHER)
1198	474875_length_1224_cvg_4.7_tip_1_0	30	von willebrand factor type egf and pentraxin domain-containing protein 1-like	408	4.39E-13	46.40%	0.101	IPR000436 (SMART); G3DSA:2.10.70.10 (GENE3D); IPR000436 (PFAM); G3DSA:2.10.70.10 (GENE3D); G3DSA:2.10.70.10 (GENE3D); G3DSA:2.10.70.10 (GENE3D); PTHR19325 (PANTHER); IPR000436 (PROSITE_PROFILES); IPR000436 (PROSITE_PROFILES); IPR000436 (PROSITE_PROFILES); IPR000436 (SUPERFAMILY); IPR000436 (SUPERFAMILY); IPR000436 (SUPERFAMILY)
1199	474897_length_1225_cvg_54.7_tip_1_1	591	histone 1o	408	2.21E-29	71.80%	0.119	IPR005819 (PRINTS); IPR005818 (SMART); IPR005818 (PFAM); IPR011991 (G3DSA:1.10.10.GENE3D); PTHR11467 (PANTHER); IPR005818 (PROSITE_PROFILES); SSF46785 (SUPERFAMILY)
1201	474959_length_1226_cvg_3.4_tip_1_4	18	dnaj homolog subfamily b member 9	409	1.42E-61	56.10%	0.186	Coil (COILS); IPR001623 (PRINTS); IPR001623 (SMART); IPR001623 (G3DSA:1.10.287.GENE3D); IPR001623 (PFAM); PTHR24077 (PANTHER); IPR018253 (PROSITE_PATTERNS); IPR001623 (PROSITE_PROFILES); IPR001623 (SUPERFAMILY)

1202	475091_length_1228_cvg_6.8_tip_0_4	39	coagulation factor xi	409	1.68E-64	61.90%	0.124	IPR001314 (PRINTS); IPR001254 (SMART); G3DSA:2.40.10.10 (GENE3D); G3DSA:2.40.10.10 (GENE3D); IPR001254 (PFAM); G3DSA:2.40.10.10 (GENE3D); PTHR24256 (PANTHER); IPR018114 (PROSITE_PATTERNS); IPR018114 (PROSITE_PATTERNS); IPR001254 (PROSITE_PROFILES); IPR009003 (SUPERFAMILY)
1203	475119_length_1229_cvg_18.4_tip_1_1	94	ankyrin repeat family a protein 2	410	8.32E-73	67.70%	0.204	IPR002110 (SMART); IPR020683 (PFAM); IPR020683 (G3DSA:1.25.40.GENE3D); IPR002110 (PFAM); PTHR24124 (PANTHER); IPR002110 (PROSITE_PROFILES); IPR002110 (PROSITE_PROFILES); IPR020683 (PROSITE_PROFILES); IPR002110 (PROSITE_PROFILES); IPR020683 (SUPERFAMILY)
1204	475165_length_1230_cvg_4.4_tip_1_2	32	fukutin-related partial	410	1.20E-26	72.00%	0.286	IPR007074 (PFAM); PTHR13627 (PANTHER); PTHR13627:SF25 (PANTHER); CYTOPLASMIC_DOMAIN (PHOBIUS); TRANSMEMBRANE (PHOBIUS); CYTOPLASMIC_DOMAIN (PHOBIUS); NON_CYTOPLASMIC_DOMAIN (PHOBIUS); TRANSMEMBRANE (PHOBIUS); NON_CYTOPLASMIC_DOMAIN (PHOBIUS); TRANSMEMBRANE (PHOBIUS); TMhelix (TMHMM); TMhelix (TMHMM); TMhelix (TMHMM)
1205	475171_length_1230_cvg_12.1_tip_1_5	66	zinc finger protein 271 (zinc finger protein 7) (zinc finger protein znfphex133) (epstein-barr virus-induced zinc finger protein) (znf-eb) (ct-zfp48) (zinc finger protein	410	1.02E-37	54.20%	0.131	IPR015880 (SMART); PF13894 (PFAM); IPR013087 (G3DSA:3.30.160.GENE3D); PF13465 (PFAM); IPR013087 (G3DSA:3.30.160.GENE3D); PTHR24375 (PANTHER); PTHR24375:SF106 (PANTHER); IPR007087 (PROSITE_PATTERNS); IPR007087 (PROSITE_PATTERNS); IPR007087 (PROSITE_PATTERNS); IPR007087 (PROSITE_PATTERNS); IPR007087 (PROSITE_PATTERNS); IPR007087 (PROSITE_PATTERNS); IPR007087 (PROSITE_PATTERNS); IPR007087 (PROSITE_PATTERNS); IPR007087 (PROSITE_PROFILES); IPR007087 (PROSITE_PROFILES); IPR007087 (PROSITE_PROFILES); IPR007087 (PROSITE_PROFILES); IPR007087 (PROSITE_PROFILES); IPR007087 (PROSITE_PROFILES); IPR007087 (PROSITE_PROFILES); IPR007087 (PROSITE_PROFILES); SSF57667 (SUPERFAMILY); SSF57667 (SUPERFAMILY); SSF57667 (SUPERFAMILY); SSF57667 (SUPERFAMILY); SSF57667 (SUPERFAMILY)
1206	475175_length_1230_cvg_65.9_tip_1_3	1197	hemagglutinin	410	6.36E-93	67.80%	0.114	IPR023612 (PRINTS); G3DSA:1.10.390.10 (GENE3D); IPR013856 (PFAM); IPR013856 (G3DSA:3.10.170.GENE3D); IPR001570 (PFAM); IPR007280 (PFAM); CYTOPLASMIC_DOMAIN (PHOBIUS); NON_CYTOPLASMIC_DOMAIN (PHOBIUS); TRANSMEMBRANE (PHOBIUS); TRANSMEMBRANE (PHOBIUS); NON_CYTOPLASMIC_DOMAIN (PHOBIUS); SSF55486 (SUPERFAMILY); TMhelix (TMHMM); TMhelix (TMHMM)
1222	475901_length_1245_cvg_19.6_tip_1_2	117	ankyrin repeat domain-containing protein 16-like	410	2.31E-112	63.60%	0.104	IPR002110 (PRINTS); IPR002110 (SMART); IPR020683 (G3DSA:1.25.40.GENE3D); IPR020683 (PFAM); PTHR24181:SF20 (PANTHER); PTHR24181 (PANTHER); IPR020683 (PROSITE_PROFILES); IPR002110 (PROSITE_PROFILES); IPR002110 (PROSITE_PROFILES); IPR002110 (PROSITE_PROFILES); IPR002110 (PROSITE_PROFILES); IPR002110 (PROSITE_PROFILES); IPR002110 (PROSITE_PROFILES); IPR020683 (SUPERFAMILY)
1209	475349_length_1233_cvg_35.6_tip_1_4	154	ligand of numb protein x 2	411	2.40E-55	69.10%	0.166	IPR001478 (SMART); IPR001478 (PFAM); IPR001478 (G3DSA:2.30.42.GENE3D); PTHR19964 (PANTHER); TRANSMEMBRANE (PHOBIUS); NON_CYTOPLASMIC_DOMAIN (PHOBIUS); TRANSMEMBRANE (PHOBIUS); CYTOPLASMIC_DOMAIN (PHOBIUS); NON_CYTOPLASMIC_DOMAIN (PHOBIUS); IPR001478 (PROSITE_PROFILES); IPR001478 (SUPERFAMILY); TMhelix (TMHMM); TMhelix (TMHMM); TMhelix (TMHMM)

1210	475375_length_1234_cvg_9.4_tip_1_5	48	gastrula zinc finger	411	1.20E-76	55.80%	0.115	IPR015880 (SMART); IPR013087 (G3DSA:3.30.160.GENE3D); PF13894 (PFAM); IPR013087 (G3DSA:3.30.160.GENE3D); PF13912 (PFAM); IPR013087 (G3DSA:3.30.160.GENE3D); IPR013087 (G3DSA:3.30.160.GENE3D); IPR013087 (G3DSA:3.30.160.GENE3D); PF13465 (PFAM); PTHR24409 (PANTHER); IPR007087 (PROSITE_PATTERNS); IPR007087 (PROSITE_PATTERNS); IPR007087 (PROSITE_PATTERNS); IPR007087 (PROSITE_PATTERNS); IPR007087 (PROSITE_PATTERNS); IPR007087 (PROSITE_PATTERNS); IPR007087 (PROSITE_PATTERNS); IPR007087 (PROSITE_PATTERNS); IPR007087 (PROSITE_PATTERNS); IPR007087 (PROSITE_PATTERNS); IPR007087 (PROSITE_PATTERNS); IPR007087 (PROSITE_PATTERNS); IPR007087 (PROSITE_PROFILES); IPR007087 (PROSITE_PROFILES); IPR007087 (PROSITE_PROFILES); IPR007087 (PROSITE_PROFILES); IPR007087 (PROSITE_PROFILES); IPR007087 (PROSITE_PROFILES); IPR007087 (PROSITE_PROFILES); IPR007087 (PROSITE_PROFILES); IPR007087 (PROSITE_PROFILES); IPR007087 (PROSITE_PROFILES); IPR007087 (PROSITE_PROFILES); IPR007087 (PROSITE_PROFILES); IPR007087 (PROSITE_PROFILES); SSF57667 (SUPERFAMILY); SSF57667 (SUPERFAMILY); SSF57667 (SUPERFAMILY); SSF57667 (SUPERFAMILY); SSF57667 (SUPERFAMILY); SSF57667 (SUPERFAMILY)
1212	475421_length_1235_cvg_4.2_tip_1_2	29	contactin associated protein 1	411	2.36E-51	50.90%	0.102	Coil (COILS); IPR000885 (PFAM); IPR014716 (G3DSA:3.90.215.GENE3D); PTHR10127 (PANTHER); PTHR10127:SF575 (PANTHER); IPR002181 (SUPERFAMILY)
1211	475405_length_1235_cvg_61.7_tip_1_3	773	protein them6	412	1.51E-35	58.40%	0.131	Coil (COILS); IPR029069 (G3DSA:3.10.129.GENE3D); PF13279 (PFAM); PTHR12475 (PANTHER); NON_CYTOPLASMIC_DOMAIN (PHOBIUS); TRANSMEMBRANE (PHOBIUS); NON_CYTOPLASMIC_DOMAIN (PHOBIUS); CYTOPLASMIC_DOMAIN (PHOBIUS); CYTOPLASMIC_DOMAIN (PHOBIUS); TRANSMEMBRANE (PHOBIUS); TRANSMEMBRANE (PHOBIUS); IPR029069 (SUPERFAMILY); TMhelix (TMHMM); TMhelix (TMHMM)
1213	475445_length_1235_cvg_3.0_tip_1_4	18	vitellogenin fused with superoxide dismutase	412	1.40E-39	47.50%	0.125	IPR001846 (SMART); IPR001846 (PFAM); PTHR23345 (PANTHER); PTHR23345:SF1 (PANTHER); IPR001846 (PROSITE_PROFILES)
1215	475483_length_1236_cvg_7.4_tip_1_5	44	endothelin-converting enzyme 1-like	412	2.59E-36	55.70%	0.246	IPR008753 (PFAM); G3DSA:1.10.1380.10 (GENE3D); IPR000718 (PANTHER); NON_CYTOPLASMIC_DOMAIN (PHOBIUS); TRANSMEMBRANE (PHOBIUS); CYTOPLASMIC_DOMAIN (PHOBIUS); SSF55486 (SUPERFAMILY); TMhelix (TMHMM)
1217	475583_length_1238_cvg_64.1_tip_1_5	901	beta-galactosidase-1-like protein 2	412	4.59E-105	68.50%	0.258	IPR001944 (PRINTS); IPR013781 (G3DSA:3.20.20.GENE3D); IPR001944 (PFAM); IPR001944 (PANTHER); PTHR23421:SF66 (PANTHER); IPR019801 (PROSITE_PATTERNS); IPR017853 (SUPERFAMILY)
1216	475509_length_1237_cvg_71.5_tip_1_3	1103	chymotrypsinogen a	413	1.38E-91	69.80%	0.097	IPR001314 (PRINTS); IPR001254 (SMART); G3DSA:2.40.10.10 (GENE3D); G3DSA:2.40.10.10 (GENE3D); G3DSA:2.40.10.10 (GENE3D); IPR001254 (PFAM); PTHR24260:SF47 (PANTHER); PTHR24260 (PANTHER); NON_CYTOPLASMIC_DOMAIN (PHOBIUS); TRANSMEMBRANE (PHOBIUS); TRANSMEMBRANE (PHOBIUS); NON_CYTOPLASMIC_DOMAIN (PHOBIUS); CYTOPLASMIC_DOMAIN (PHOBIUS); IPR001254 (PROSITE_PROFILES); IPR009003 (SUPERFAMILY); TMhelix (TMHMM); TMhelix (TMHMM)
1218	475597_length_1239_cvg_4.2_tip_1_0	29	serine protease easter-like	413	5.31E-65	59.40%	0.125	IPR001314 (PRINTS); IPR001254 (SMART); G3DSA:2.40.10.10 (GENE3D); G3DSA:2.40.10.10 (GENE3D); IPR001254 (PFAM); G3DSA:2.40.10.10 (GENE3D); PTHR24260:SF3 (PANTHER); PTHR24260 (PANTHER); IPR018114 (PROSITE_PATTERNS); IPR018114 (PROSITE_PATTERNS); TRANSMEMBRANE (PHOBIUS); CYTOPLASMIC_DOMAIN (PHOBIUS); NON_CYTOPLASMIC_DOMAIN (PHOBIUS); IPR001254 (PROSITE_PROFILES); IPR009003 (SUPERFAMILY)
1220	475667_length_1240_cvg_2.8_tip_1_4	23	angio-associated migratory cell	413	3.56E-71	65.30%	0.153	IPR001680 (SMART); IPR015943 (G3DSA:2.130.10.GENE3D); IPR001680 (PFAM); PTHR19857 (PANTHER); IPR001680 (PROSITE_PROFILES); IPR017986 (PROSITE_PROFILES); IPR001680 (PROSITE_PROFILES); IPR011047 (SUPERFAMILY)
1219	475645_length_1240_cvg_8.1_tip_1_3	58	internalin a	414	1.89E-125	68.60%	0.12	IPR003591 (SMART); SM00364 (SMART); IPR025875 (PFAM); G3DSA:3.80.10.10 (GENE3D); IPR001611 (PFAM); IPR001611 (PFAM); PTHR23155 (PANTHER); PTHR23155:SF408 (PANTHER); IPR001611 (PROSITE_PROFILES); IPR001611 (PROSITE_PROFILES); IPR001611 (PROSITE_PROFILES); IPR001611 (PROSITE_PROFILES); IPR001611 (PROSITE_PROFILES); IPR001611 (PROSITE_PROFILES); IPR001611 (PROSITE_PROFILES); IPR001611 (PROSITE_PROFILES); IPR001611 (PROSITE_PROFILES); SSF52058 (SUPERFAMILY)

1221	475687_length_1241_cvg_63.3_tip_1_4	523	phenoloxidase subunit a3-like	414	8.06E-108	62.70%	0.175	IPR000896 (PFAM); IPR005203 (G3DSA:2.60.40.GENE3D); IPR005203 (PFAM); IPR008922 (G3DSA:1.10.1280.GENE3D); IPR013788 (PANTHER); PTHR11511:SF24 (PANTHER); IPR013788 (PROSITE_PATTERNS); IPR002227 (PROSITE_PATTERNS); IPR008922 (SUPERFAMILY); IPR014756 (SUPERFAMILY)
1223	476091_length_1248_cvg_11.2_tip_1_3	62	leucine-rich repeats and immunoglobulin-like domains protein 2-like	416	3.48E-60	61.60%	0.122	PR00019 (PRINTS); IPR003591 (SMART); SM00365 (SMART); IPR001611 (PFAM); IPR025875 (PFAM); G3DSA:3.80.10.10 (GENE3D); PTHR24373 (PANTHER); NON_CYTOPLASMIC_DOMAIN (PHOBIUS); TRANSMEMBRANE (PHOBIUS); TRANSMEMBRANE (PHOBIUS); CYTOPLASMIC_DOMAIN (PHOBIUS); TRANSMEMBRANE (PHOBIUS); CYTOPLASMIC_DOMAIN (PHOBIUS); NON_CYTOPLASMIC_DOMAIN (PHOBIUS); IPR001611 (PROSITE_PROFILES); IPR001611 (PROSITE_PROFILES); IPR001611 (PROSITE_PROFILES); IPR001611 (PROSITE_PROFILES); IPR001611 (PROSITE_PROFILES); IPR001611 (PROSITE_PROFILES); IPR001611 (PROSITE_PROFILES); IPR001611 (PROSITE_PROFILES); SSF52058 (SUPERFAMILY); TMhelix (TMHMM); TMhelix (TMHMM)
1224	476121_length_1249_cvg_16.2_tip_1_5	94	mitochondrial	416	2.36E-40	76.60%	0.133	IPR012336 (G3DSA:3.40.30.GENE3D); IPR013766 (PFAM); IPR005746 (PANTHER); PTHR10438:SF254 (PANTHER); NON_CYTOPLASMIC_DOMAIN (PHOBIUS); NON_CYTOPLASMIC_DOMAIN (PHOBIUS); TRANSMEMBRANE (PHOBIUS); NON_CYTOPLASMIC_DOMAIN (PHOBIUS); TRANSMEMBRANE (PHOBIUS); TRANSMEMBRANE (PHOBIUS); NON_CYTOPLASMIC_DOMAIN (PHOBIUS); CYTOPLASMIC_DOMAIN (PHOBIUS); TRANSMEMBRANE (PHOBIUS); CYTOPLASMIC_DOMAIN (PHOBIUS); TRANSMEMBRANE (PHOBIUS); TRANSMEMBRANE (PHOBIUS); CYTOPLASMIC_DOMAIN (PHOBIUS); CYTOPLASMIC_DOMAIN (PHOBIUS); TRANSMEMBRANE (PHOBIUS); IPR012336 (PROSITE_PROFILES); IPR012336 (SUPERFAMILY); TMhelix (TMHMM); TMhelix (TMHMM); TMhelix (TMHMM); TMhelix (TMHMM); TMhelix (TMHMM); TMhelix (TMHMM)
1225	476207_length_1250_cvg_4.0_tip_1_4	31	nicotinamide phosphoribosyltransferase-like	417	4.19E-138	81.90%	0.114	G3DSA:3.20.140.10 (GENE3D); IPR007229 (PFAM); IPR007229 (PANTHER); IPR016471 (PTHR11098:PANTHER); TRANSMEMBRANE (PHOBIUS); NON_CYTOPLASMIC_DOMAIN (PHOBIUS); CYTOPLASMIC_DOMAIN (PHOBIUS); IPR002638 (SUPERFAMILY)
1226	476247_length_1251_cvg_48.4_tip_1_5	335	cytolysin src-1-like	417	3.30E-21	51.60%	0.124	IPR015926 (G3DSA:2.60.270.GENE3D); TRANSMEMBRANE (PHOBIUS); NON_CYTOPLASMIC_DOMAIN (PHOBIUS); CYTOPLASMIC_DOMAIN (PHOBIUS); IPR015926 (SUPERFAMILY); TMhelix (TMHMM); TMhelix (TMHMM)
1227	476255_length_1251_cvg_61.3_tip_1_3	995	26s protease regulatory subunit 10b	417	0	96.70%	0.097	Coil (COILS); IPR003593 (SMART); IPR005937 (TIGRFAM); G3DSA:1.10.8.60 (GENE3D); IPR003959 (PFAM); IPR027417 (G3DSA:3.40.50.GENE3D); PTHR23073 (PANTHER); PTHR23073:SF24 (PANTHER); IPR003960 (PROSITE_PATTERNS); IPR027417 (SUPERFAMILY)
1228	476383_length_1254_cvg_6.7_tip_1_5	37	acid ceramidase	418	3.08E-153	71.90%	0.618 Y	Coil (COILS); IPR029130 (PFAM); IPR029132 (PFAM); IPR016699 (PIRSF); NON_CYTOPLASMIC_DOMAIN (PHOBIUS); SIGNAL_PEPTIDE_H_REGION (PHOBIUS); SIGNAL_PEPTIDE (PHOBIUS); SIGNAL_PEPTIDE_N_REGION (PHOBIUS); SIGNAL_PEPTIDE_C_REGION (PHOBIUS); SignalP-noTM (SIGNALP_EUK)
1229	476425_length_1255_cvg_10.5_tip_1_1	65	kunitz-like protease inhibitor	418	1.03E-40	55.50%	0.142	IPR002223 (PRINTS); IPR002223 (SMART); IPR002223 (G3DSA:4.10.410.GENE3D); IPR002223 (PFAM); IPR002223 (G3DSA:4.10.410.GENE3D); IPR008037 (PFAM); PTHR10083 (PANTHER); IPR020901 (PROSITE_PATTERNS); IPR002223 (PROSITE_PROFILES); IPR020862 (PROSITE_PROFILES); IPR020862 (PROSITE_PROFILES); IPR002223 (PROSITE_PROFILES); IPR020862 (PROSITE_PROFILES); IPR008037 (SUPERFAMILY); IPR008037 (SUPERFAMILY); IPR002223 (SUPERFAMILY); IPR008037 (SUPERFAMILY); IPR002223 (SUPERFAMILY)
1230	476449_length_1256_cvg_3.4_tip_0_5	23	ankyrin repeat and kh domain-containing protein mask	418	0	88.00%	0.155	IPR002110 (PRINTS); IPR002110 (SMART); IPR020683 (G3DSA:1.25.40.GENE3D); IPR020683 (PFAM); IPR020683 (G3DSA:1.25.40.GENE3D); IPR002110 (PFAM); IPR020683 (G3DSA:1.25.40.GENE3D); IPR020683 (G3DSA:1.25.40.GENE3D); PTHR23206 (PANTHER); IPR002110 (PROSITE_PROFILES); IPR002110 (PROSITE_PROFILES); IPR002110 (PROSITE_PROFILES); IPR002110 (PROSITE_PROFILES); IPR020683 (PROSITE_PROFILES); IPR002110 (PROSITE_PROFILES); IPR002110 (PROSITE_PROFILES); IPR002110 (PROSITE_PROFILES); IPR002110 (PROSITE_PROFILES); IPR002110 (PROSITE_PROFILES); IPR002110 (PROSITE_PROFILES); IPR020683 (SUPERFAMILY)

1238	477460_length_1278_cvg_91.2_tip_1_1	6691	transmembrane protease serine 6 isoform x2	418	1.57E-40	53.40%	0.155		IPR001314 (PRINTS); IPR001254 (SMART); G3DSA:2.40.10.10 (GENE3D); G3DSA:2.40.10.10 (GENE3D); G3DSA:2.40.10.10 (GENE3D); IPR001254 (PFAM); PTHR24268 (PANTHER); IPR018114 (PROSITE_PATTERNS); IPR018114 (PROSITE_PATTERNS); NON_CYTOPLASMIC_DOMAIN (PHOBIUS); TRANSMEMBRANE (PHOBIUS); CYTOPLASMIC_DOMAIN (PHOBIUS); IPR001254 (PROSITE_PROFILES); IPR009003 (SUPERFAMILY)
1231	476467_length_1256_cvg_8.8_tip_1_0	40	hemocytin	419	3.55E-38	46.60%	0.102		IPR001846 (PFAM); IPR014853 (PFAM); IPR001846 (PROSITE_PROFILES); IPR002919 (SUPERFAMILY)
1232	476524_length_1258_cvg_6.5_tip_1_3	47	phosphatidylinositol -trisphosphate 3-phosphatase and dual-specificity protein phosphatase pten isoform x4	420	8.25E-123	70.90%	0.118		IPR003595 (SMART); G3DSA:2.60.40.1110 (GENE3D); IPR000340 (PFAM); IPR029021 (G3DSA:3.90.190.GENE3D); IPR014020 (PFAM); PTHR12305 (PANTHER); IPR016130 (PROSITE_PATTERNS); TRANSMEMBRANE (PHOBIUS); NON_CYTOPLASMIC_DOMAIN (PHOBIUS); CYTOPLASMIC_DOMAIN (PHOBIUS); IPR029023 (PROSITE_PROFILES); IPR014020 (PROSITE_PROFILES); IPR000008 (SUPERFAMILY); IPR029021 (SUPERFAMILY); TMhelix (TMHMM)
1233	476786_length_1263_cvg_16.3_tip_1_4	81	zinc finger protein 300-like isoform x1	421	1.65E-18	60.60%	0.13		IPR015880 (SMART); IPR013087 (G3DSA:3.30.160.GENE3D); PF13465 (PFAM); IPR013087 (G3DSA:3.30.160.GENE3D); IPR007087 (PFAM); PTHR24409 (PANTHER); PTHR24409:SF14 (PANTHER); IPR007087 (PROSITE_PATTERNS); IPR007087 (PROSITE_PATTERNS); IPR007087 (PROSITE_PATTERNS); IPR026590 (PROSITE_PROFILES); IPR007087 (PROSITE_PROFILES); IPR007087 (PROSITE_PROFILES); IPR007087 (PROSITE_PROFILES); SSF57667 (SUPERFAMILY); SSF57667 (SUPERFAMILY)
1242	477978_length_1290_cvg_12.6_tip_1_0	87	adenosine deaminase-like protein	422	1.49E-108	69.70%	0.097		G3DSA:3.20.20.140 (GENE3D); IPR001365 (PFAM); PTHR11409:SF21 (PANTHER); PTHR11409 (PANTHER); SSF51556 (SUPERFAMILY)
1234	477142_length_1271_cvg_12.3_tip_1_4	64	p38 mitogen-activated protein kinase	424	1.09E-127	90.00%	0.108		Coil (COILS); IPR008352 (PRINTS); IPR002290 (SMART); IPR000719 (PFAM); G3DSA:1.10.510.10 (GENE3D); PTHR24055:SF110 (PANTHER); PTHR24055 (PANTHER); NON_CYTOPLASMIC_DOMAIN (PHOBIUS); TRANSMEMBRANE (PHOBIUS); CYTOPLASMIC_DOMAIN (PHOBIUS); IPR000719 (PROSITE_PROFILES); IPR011009 (SUPERFAMILY)
1235	477200_length_1272_cvg_3.1_tip_1_1	23	prolyl 3-hydroxylase 2-like isoform x1	424	3.30E-105	68.90%	0.103		Coil (COILS); IPR006620 (SMART); IPR005123 (PFAM); PTHR14049 (PANTHER); PTHR14049:SF9 (PANTHER); IPR005123 (PROSITE_PROFILES)
1236	477370_length_1276_cvg_5.7_tip_1_3	24	agap000560-pa-like protein	426	0	74.50%	0.13		IPR003593 (SMART); IPR027417 (G3DSA:3.40.50.GENE3D); IPR003959 (PFAM); PTHR23076:SF47 (PANTHER); PTHR23076 (PANTHER); CYTOPLASMIC_DOMAIN (PHOBIUS); TRANSMEMBRANE (PHOBIUS); NON_CYTOPLASMIC_DOMAIN (PHOBIUS); CYTOPLASMIC_DOMAIN (PHOBIUS); TRANSMEMBRANE (PHOBIUS); IPR027417 (SUPERFAMILY); TMhelix (TMHMM)
1237	477392_length_1277_cvg_2.3_tip_1_4	18	-like protein subfamily c member 11	426	0	81.50%	0.102		IPR001623 (PRINTS); IPR001623 (SMART); IPR001623 (G3DSA:1.10.287.GENE3D); IPR001623 (PFAM); PTHR24078:SF159 (PANTHER); PTHR24078 (PANTHER); IPR001623 (PROSITE_PROFILES); IPR001623 (SUPERFAMILY)
1239	477520_length_1279_cvg_2.0_tip_1_2	13	zinc finger protein 271 (zinc finger protein 7) (zinc finger protein znfphex133) (epstein-barr virus-induced zinc finger protein) (znf-eb) (ct-zfp48) (zinc finger protein	426	3.97E-41	54.70%	0.101		IPR015880 (SMART); IPR013087 (G3DSA:3.30.160.GENE3D); IPR007087 (PFAM); IPR013087 (G3DSA:3.30.160.GENE3D); PF13465 (PFAM); PF13894 (PFAM); IPR013087 (G3DSA:3.30.160.GENE3D); PTHR24409:SF14 (PANTHER); PTHR24409 (PANTHER); IPR007087 (PROSITE_PATTERNS); IPR007087 (PROSITE_PATTERNS); IPR007087 (PROSITE_PATTERNS); IPR007087 (PROSITE_PATTERNS); IPR007087 (PROSITE_PATTERNS); IPR007087 (PROSITE_PATTERNS); IPR007087 (PROSITE_PATTERNS); IPR007087 (PROSITE_PATTERNS); CYTOPLASMIC_DOMAIN (PHOBIUS); TRANSMEMBRANE (PHOBIUS); NON_CYTOPLASMIC_DOMAIN (PHOBIUS); IPR007087 (PROSITE_PROFILES); IPR007087 (PROSITE_PROFILES); IPR007087 (PROSITE_PROFILES); IPR007087 (PROSITE_PROFILES); IPR007087 (PROSITE_PROFILES); IPR007087 (PROSITE_PROFILES); IPR007087 (PROSITE_PROFILES); IPR007087 (PROSITE_PROFILES); IPR007087 (PROSITE_PROFILES); SSF57667 (SUPERFAMILY); SSF57667 (SUPERFAMILY); SSF57667 (SUPERFAMILY); SSF57667 (SUPERFAMILY)
1240	477702_length_1284_cvg_4.6_tip_1_4	22	neuroglian-like isoform 2	428	0	80.00%	0.108		IPR003598 (SMART); IPR003599 (SMART); IPR003961 (SMART); IPR013783 (G3DSA:2.60.40.GENE3D); IPR003961 (PFAM); IPR013098 (PFAM); IPR013783 (G3DSA:2.60.40.GENE3D); IPR013783 (G3DSA:2.60.40.GENE3D); IPR013783 (G3DSA:2.60.40.GENE3D); PTHR10489:SF553 (PANTHER); PTHR10489 (PANTHER); IPR003961 (PROSITE_PROFILES); IPR003961 (PROSITE_PROFILES); IPR007110 (PROSITE_PROFILES); IPR007110 (PROSITE_PROFILES); IPR003961 (SUPERFAMILY); SSF48726 (SUPERFAMILY); SSF48726 (SUPERFAMILY)
1254	478894_length_1312_cvg_35.7_tip_1_0	196	gamma-glutamyl hydrolase	428	7.53E-100	65.80%	0.546	Y	IPR011697 (PFAM); IPR029062 (G3DSA:3.40.50.GENE3D); IPR015527 (PANTHER); IPR015527 (PROSITE_PROFILES); SignalP-noTM (SIGNALP_EUK); IPR029062 (SUPERFAMILY); TMhelix (TMHMM); TMhelix (TMHMM); TMhelix (TMHMM)

1241	477904_length_1288_cvg_10.4_tip_1_4	61	retinoid-inducible serine carboxypeptidase	429	3.30E-110	68.40%	0.132	IPR001563 (PRINTS); IPR001563 (PFAM); IPR029058 (G3DSA:3.40.50.GENE3D); PTHR11802:SF3 (PANTHER); IPR001563 (PANTHER); IPR018202 (PROSITE_PATTERNS); IPR029058 (SUPERFAMILY)
1250	478692_length_1307_cvg_4.0_tip_1_1	30	zinc finger protein 501-like	429	1.72E-34	50.70%	0.102	IPR015880 (SMART); PF13465 (PFAM); PF13894 (PFAM); IPR013087 (G3DSA:3.30.160.GENE3D); IPR007087 (PFAM); IPR013087 (G3DSA:3.30.160.GENE3D); PTHR24409:SF14 (PANTHER); PTHR24409 (PANTHER); IPR007087 (PROSITE_PATTERNS); IPR007087 (PROSITE_PATTERNS); IPR007087 (PROSITE_PATTERNS); IPR007087 (PROSITE_PATTERNS); IPR007087 (PROSITE_PATTERNS); IPR007087 (PROSITE_PATTERNS); IPR007087 (PROSITE_PATTERNS); IPR007087 (PROSITE_PATTERNS); IPR007087 (PROSITE_PROFILES); IPR007087 (PROSITE_PROFILES); IPR007087 (PROSITE_PROFILES); IPR007087 (PROSITE_PROFILES); IPR007087 (PROSITE_PROFILES); IPR007087 (PROSITE_PROFILES); IPR007087 (PROSITE_PROFILES); IPR007087 (PROSITE_PROFILES); IPR007087 (PROSITE_PROFILES); SSF57667 (SUPERFAMILY); SSF57667 (SUPERFAMILY); SSF57667 (SUPERFAMILY); SSF57667 (SUPERFAMILY)
1256	479060_length_1316_cvg_15.4_tip_1_2	101	xanthine dehydrogenase	431	4.10E-09	61.20%	0.194	IPR002888 (G3DSA:1.10.150.GENE3D); IPR002888 (PFAM); CYTOPLASMIC_DOMAIN (PHOBIUS); NON_CYTOPLASMIC_DOMAIN (PHOBIUS); TRANSMEMBRANE (PHOBIUS); CYTOPLASMIC_DOMAIN (PHOBIUS); TRANSMEMBRANE (PHOBIUS); TRANSMEMBRANE (PHOBIUS); NON_CYTOPLASMIC_DOMAIN (PHOBIUS); IPR002888 (SUPERFAMILY)
1243	478176_length_1295_cvg_52.3_tip_1_1	3868	serpin 3	432	1.31E-88	64.10%	0.105	IPR023796 (SMART); IPR023796 (PFAM); G3DSA:2.30.39.10 (GENE3D); G3DSA:3.30.497.10 (GENE3D); IPR000215 (PANTHER); IPR023796 (SUPERFAMILY)
1244	478218_length_1296_cvg_2.0_tip_1_2	15	endoglucanase	432	0	97.60%	0.13	IPR002037 (PRINTS); IPR002037 (PFAM); IPR018513 (PFAM); IPR012341 (G3DSA:1.50.10.GENE3D); IPR019834 (PROSITE_PATTERNS); NON_CYTOPLASMIC_DOMAIN (PHOBIUS); NON_CYTOPLASMIC_DOMAIN (PHOBIUS); TRANSMEMBRANE (PHOBIUS); CYTOPLASMIC_DOMAIN (PHOBIUS); TRANSMEMBRANE (PHOBIUS); IPR008928 (SUPERFAMILY); TMhelix (TMHMM); TMhelix (TMHMM)
1245	478252_length_1296_cvg_18.8_tip_1_0	115	PREDICTED: uncharacterized protein LOC101859571	432	3.74E-06	39.00%	0.138	IPR000998 (SMART); IPR000998 (PFAM); CYTOPLASMIC_DOMAIN (PHOBIUS); NON_CYTOPLASMIC_DOMAIN (PHOBIUS); TRANSMEMBRANE (PHOBIUS); NON_CYTOPLASMIC_DOMAIN (PHOBIUS); TRANSMEMBRANE (PHOBIUS); IPR000998 (PROSITE_PROFILES); IPR013320 (SUPERFAMILY); TMhelix (TMHMM)
1246	478524_length_1304_cvg_15.7_tip_1_1	118	leucine-rich repeat and calponin homology domain-containing protein 1-like	435	1.44E-119	65.60%	0.271	IPR003591 (SMART); SM00364 (SMART); IPR001611 (PFAM); IPR001611 (PFAM); G3DSA:3.80.10.10 (GENE3D); IPR001611 (PFAM); PTHR23155 (PANTHER); PTHR23155:SF423 (PANTHER); IPR001611 (PROSITE_PROFILES); IPR001611 (PROSITE_PROFILES); IPR001611 (PROSITE_PROFILES); IPR001611 (PROSITE_PROFILES); IPR001611 (PROSITE_PROFILES); IPR001611 (PROSITE_PROFILES); IPR001611 (PROSITE_PROFILES); SSF52058 (SUPERFAMILY)
1247	478552_length_1304_cvg_4.8_tip_1_4	33	protein partial	435	7.03E-48	48.50%	0.134	IPR000884 (SMART); IPR000884 (PFAM); G3DSA:2.20.100.10 (GENE3D); PTHR11348 (PANTHER); PTHR11348:SF17 (PANTHER); TRANSMEMBRANE (PHOBIUS); NON_CYTOPLASMIC_DOMAIN (PHOBIUS); CYTOPLASMIC_DOMAIN (PHOBIUS); IPR000884 (PROSITE_PROFILES); IPR000884 (SUPERFAMILY); TMhelix (TMHMM)
1248	478572_length_1305_cvg_15.1_tip_1_2	111	serine threonine-protein kinase sbk1	435	7.03E-148	71.40%	0.325	IPR002290 (SMART); G3DSA:1.10.510.10 (GENE3D); G3DSA:3.30.200.20 (GENE3D); IPR000719 (PFAM); PTHR24359 (PANTHER); IPR008271 (PROSITE_PATTERNS); SIGNAL_PEPTIDE_C_REGION (PHOBIUS); NON_CYTOPLASMIC_DOMAIN (PHOBIUS); SIGNAL_PEPTIDE_N_REGION (PHOBIUS); SIGNAL_PEPTIDE (PHOBIUS); SIGNAL_PEPTIDE_H_REGION (PHOBIUS); IPR000719 (PROSITE_PROFILES); SignalP-TM (SIGNALP_GRAM_POSITIVE); IPR011009 (SUPERFAMILY)
1249	478646_length_1306_cvg_25.9_tip_1_5	134	carboxypeptidase n subunit 2	435	7.27E-29	55.50%	0.173	IPR003591 (SMART); IPR001611 (PFAM); G3DSA:3.80.10.10 (GENE3D); IPR001611 (PFAM); PTHR24373 (PANTHER); PTHR24373:SF98 (PANTHER); SSF52058 (SUPERFAMILY)
1251	478746_length_1308_cvg_9.2_tip_1_2	65	follistatin-a isoform x2	436	7.06E-104	66.80%	0.13	IPR003645 (SMART); IPR002350 (SMART); G3DSA:3.30.60.30 (GENE3D); IPR002350 (PFAM); IPR002350 (PFAM); PTHR10913 (PANTHER); PTHR10913:SF39 (PANTHER); IPR002350 (PROSITE_PROFILES); IPR002350 (PROSITE_PROFILES); IPR002350 (PROSITE_PROFILES); SSF100895 (SUPERFAMILY); SSF100895 (SUPERFAMILY); SSF100895 (SUPERFAMILY)

1252	478752_length_1309_cvg_2.4_tip_1_2	19	cytochrome p450 cyp44	436	6.34E-145	65.30%	0.099	IPR002401 (PRINTS); IPR001128 (PRINTS); IPR001128 (G3DSA:1.10.630.GENE3D); IPR001128 (PFAM); PTHR24291:SF1 (PANTHER); PTHR24291 (PANTHER); IPR017972 (PROSITE_PATTERNS); IPR001128 (SUPERFAMILY)
1271	479790_length_1333_cvg_59.8_tip_1_3	588	huga_polan ame: full=hyaluronidase short=hya ame: full=hyaluronoglucosaminidase ame: allergen=pol a 2 flags: partial	436	8.44E-72	57.00%	0.262	IPR001329 (PRINTS); IPR018155 (PRINTS); IPR013785 (G3DSA:3.20.20.GENE3D); IPR018155 (PFAM); PTHR11769:SF8 (PANTHER); IPR018155 (PANTHER); SIGNAL_PEPTIDE (PHOBIUS); SIGNAL_PEPTIDE_C_REGION (PHOBIUS); SIGNAL_PEPTIDE_H_REGION (PHOBIUS); SIGNAL_PEPTIDE_N_REGION (PHOBIUS); NON_CYTOPLASMIC_DOMAIN (PHOBIUS); IPR017853 (SUPERFAMILY); TMhelix (TMHMM)
1253	478810_length_1310_cvg_2.9_tip_1_1	25	---NA---	437			0.173	NON_CYTOPLASMIC_DOMAIN (PHOBIUS); CYTOPLASMIC_DOMAIN (PHOBIUS); TRANSMEMBRANE (PHOBIUS); CYTOPLASMIC_DOMAIN (PHOBIUS); TRANSMEMBRANE (PHOBIUS); TMhelix (TMHMM)
1265	479538_length_1326_cvg_63.3_tip_1_4	5958	ferritin	437	2.13E-83	84.70%	0.275	Coil (COILS); IPR008331 (PFAM); IPR012347 (G3DSA:1.20.1260.GENE3D); IPR001519 (PANTHER); IPR014034 (PROSITE_PATTERNS); IPR014034 (PROSITE_PATTERNS); CYTOPLASMIC_DOMAIN (PHOBIUS); CYTOPLASMIC_DOMAIN (PHOBIUS); TRANSMEMBRANE (PHOBIUS); TRANSMEMBRANE (PHOBIUS); TRANSMEMBRANE (PHOBIUS); CYTOPLASMIC_DOMAIN (PHOBIUS); NON_CYTOPLASMIC_DOMAIN (PHOBIUS); NON_CYTOPLASMIC_DOMAIN (PHOBIUS); TRANSMEMBRANE (PHOBIUS); IPR009040 (PROSITE_PROFILES); IPR009078 (SUPERFAMILY); TMhelix (TMHMM); TMhelix (TMHMM); TMhelix (TMHMM)
1267	479624_length_1328_cvg_5.4_tip_1_4	39	zinc finger protein 571- partial	437	4.98E-30	52.30%	0.103	IPR015880 (SMART); IPR013087 (G3DSA:3.30.160.GENE3D); PF13912 (PFAM); IPR013087 (G3DSA:3.30.160.GENE3D); PF13465 (PFAM); PTHR24409 (PANTHER); PTHR24409:SF13 (PANTHER); IPR007087 (PROSITE_PATTERNS); IPR007087 (PROSITE_PATTERNS); IPR007087 (PROSITE_PATTERNS); IPR007087 (PROSITE_PATTERNS); IPR007087 (PROSITE_PATTERNS); IPR007087 (PROSITE_PROFILES); IPR007087 (PROSITE_PROFILES); IPR007087 (PROSITE_PROFILES); IPR007087 (PROSITE_PROFILES); IPR007087 (PROSITE_PROFILES); IPR007087 (PROSITE_PROFILES); SSF57667 (SUPERFAMILY); SSF57667 (SUPERFAMILY); SSF57667 (SUPERFAMILY); SSF57667 (SUPERFAMILY)
1276	480012_length_1338_cvg_5.6_tip_1_0	36	guanine nucleotide-binding protein subunit beta-5	438	2.20E-90	92.80%	0.127	IPR001680 (SMART); IPR001680 (PFAM); IPR015943 (G3DSA:2.130.10.GENE3D); IPR016346 (PANTHER); PTHR19850:SF12 (PANTHER); IPR019775 (PROSITE_PATTERNS); NON_CYTOPLASMIC_DOMAIN (PHOBIUS); TRANSMEMBRANE (PHOBIUS); CYTOPLASMIC_DOMAIN (PHOBIUS); IPR001680 (PROSITE_PROFILES); IPR001680 (PROSITE_PROFILES); IPR017986 (PROSITE_PROFILES); IPR001680 (PROSITE_PROFILES); IPR001680 (PROSITE_PROFILES); IPR017986 (SUPERFAMILY); IPR017986 (SUPERFAMILY); TMhelix (TMHMM)
1255	479036_length_1316_cvg_5.8_tip_1_1	47	prophenol oxidase activating enzyme precursor	439	3.37E-48	52.10%	0.156	IPR001314 (PRINTS); IPR001254 (SMART); G3DSA:2.40.10.10 (GENE3D); IPR001254 (PFAM); IPR022700 (PFAM); G3DSA:2.40.10.10 (GENE3D); G3DSA:2.40.10.10 (GENE3D); PTHR24268:SF54 (PANTHER); PTHR24268 (PANTHER); IPR018114 (PROSITE_PATTERNS); NON_CYTOPLASMIC_DOMAIN (PHOBIUS); CYTOPLASMIC_DOMAIN (PHOBIUS); TRANSMEMBRANE (PHOBIUS); IPR001254 (PROSITE_PROFILES); IPR009003 (SUPERFAMILY); TMhelix (TMHMM)
1257	479090_length_1317_cvg_6.3_tip_1_2	41	glucose dehydrogenase	439	1.01E-39	81.10%	0.127	G3DSA:3.30.560.10 (GENE3D); IPR007867 (PFAM); G3DSA:3.50.50.60 (GENE3D); PTHR11552:SF68 (PANTHER); PTHR11552 (PANTHER); NON_CYTOPLASMIC_DOMAIN (PHOBIUS); CYTOPLASMIC_DOMAIN (PHOBIUS); TRANSMEMBRANE (PHOBIUS); SSF51905 (SUPERFAMILY); SSF54373 (SUPERFAMILY)
1258	479118_length_1317_cvg_4.6_tip_1_5	28	cyclin-c	439	9.44E-166	90.90%	0.118	IPR013763 (SMART); IPR006671 (PFAM); IPR013763 (G3DSA:1.10.472.GENE3D); IPR015429 (PANTHER); IPR013763 (SUPERFAMILY); IPR013763 (SUPERFAMILY)
1259	479134_length_1317_cvg_59.0_tip_1_2	430	epoxide hydrolase 4	439	6.69E-104	62.00%	0.099	IPR000073 (PRINTS); IPR000639 (PRINTS); IPR000073 (PFAM); IPR029058 (G3DSA:3.40.50.GENE3D); PTHR10992:SF721 (PANTHER); PTHR10992 (PANTHER); NON_CYTOPLASMIC_DOMAIN (PHOBIUS); NON_CYTOPLASMIC_DOMAIN (PHOBIUS); TRANSMEMBRANE (PHOBIUS); TRANSMEMBRANE (PHOBIUS); CYTOPLASMIC_DOMAIN (PHOBIUS); IPR029058 (SUPERFAMILY); TMhelix (TMHMM)
1260	479224_length_1319_cvg_6.7_tip_1_5	26	otogelin-like protein	439	2.04E-15	45.30%	0.102	no IPS match

1261	479366_length_1322_cvg_10.2_tip_1_5	78	peptidyl-prolyl cis-trans isomerase nima-interacting 1	440	2.25E-70	85.00%	0.103	Coil (COILS); IPR001202 (SMART); G3DSA:3.10.50.40 (GENE3D); G3DSA:2.20.70.10 (GENE3D); IPR000297 (PFAM); IPR001202 (PFAM); PTHR10657 (PANTHER); IPR001202 (PROSITE_PATTERNS); IPR023058 (PROSITE_PATTERNS); TRANSMEMBRANE (PHOBIUS); TRANSMEMBRANE (PHOBIUS); NON_CYTOPLASMIC_DOMAIN (PHOBIUS); NON_CYTOPLASMIC_DOMAIN (PHOBIUS); TRANSMEMBRANE (PHOBIUS); CYTOPLASMIC_DOMAIN (PHOBIUS); TRANSMEMBRANE (PHOBIUS); NON_CYTOPLASMIC_DOMAIN (PHOBIUS); CYTOPLASMIC_DOMAIN (PHOBIUS); CYTOPLASMIC_DOMAIN (PHOBIUS); TRANSMEMBRANE (PHOBIUS); IPR000297 (PROSITE_PROFILES); IPR001202 (PROSITE_PROFILES); SSF54534 (SUPERFAMILY); IPR001202 (SUPERFAMILY); TMhelix (TMHMM)
1262	479388_length_1323_cvg_5.4_tip_1_2	24	zinc finger protein 699-like	441	8.82E-47	58.50%	0.122	IPR015880 (SMART); PF13894 (PFAM); IPR013087 (G3DSA:3.30.160.GENE3D); IPR013087 (G3DSA:3.30.160.GENE3D); IPR013087 (G3DSA:3.30.160.GENE3D); PF13465 (PFAM); IPR013087 (G3DSA:3.30.160.GENE3D); IPR013087 (G3DSA:3.30.160.GENE3D); IPR007087 (PFAM); PTHR24409 (PANTHER); PTHR24409:SF14 (PANTHER); IPR007087 (PROSITE_PATTERNS); IPR007087 (PROSITE_PATTERNS); IPR007087 (PROSITE_PATTERNS); IPR007087 (PROSITE_PATTERNS); IPR007087 (PROSITE_PATTERNS); IPR007087 (PROSITE_PATTERNS); IPR007087 (PROSITE_PATTERNS); IPR007087 (PROSITE_PATTERNS); IPR007087 (PROSITE_PATTERNS); IPR007087 (PROSITE_PROFILES); IPR007087 (PROSITE_PROFILES); IPR007087 (PROSITE_PROFILES); IPR007087 (PROSITE_PROFILES); IPR007087 (PROSITE_PROFILES); IPR007087 (PROSITE_PROFILES); IPR007087 (PROSITE_PROFILES); IPR007087 (PROSITE_PROFILES); IPR007087 (PROSITE_PROFILES); IPR007087 (PROSITE_PROFILES); SSF57667 (SUPERFAMILY); SSF57667 (SUPERFAMILY); SSF57667 (SUPERFAMILY); SSF57667 (SUPERFAMILY); SSF57667 (SUPERFAMILY)
1263	479420_length_1323_cvg_7.5_tip_1_2	45	carboxypeptidase d	441	2.13E-136	70.90%	0.112	IPR000834 (PRINTS); IPR000834 (SMART); G3DSA:3.40.630.10 (GENE3D); IPR014766 (G3DSA:2.60.40.GENE3D); PF13620 (PFAM); IPR000834 (PFAM); PTHR11532 (PANTHER); IPR000834 (PROSITE_PATTERNS); IPR000834 (PROSITE_PATTERNS); IPR008969 (SUPERFAMILY); SSF53187 (SUPERFAMILY)
1285	480538_length_1354_cvg_57.3_tip_1_2	394	ankyrin repeat domain-containing protein partial	441	1.43E-48	71.40%	0.378 Y	IPR002110 (SMART); IPR002110 (PFAM); IPR020683 (G3DSA:1.25.40.GENE3D); IPR020683 (PFAM); PTHR24149 (PANTHER); IPR002110 (PROSITE_PROFILES); IPR020683 (PROSITE_PROFILES); IPR002110 (PROSITE_PROFILES); IPR002110 (PROSITE_PROFILES); IPR020683 (SUPERFAMILY)
1264	479472_length_1325_cvg_8.6_tip_1_0	55	tyrosine-protein phosphatase	442	7.13E-33	84.60%	0.141	IPR000242 (PRINTS); IPR000242 (SMART); IPR003595 (SMART); IPR029021 (G3DSA:3.90.190.GENE3D); IPR000242 (PFAM); PTHR19134 (PANTHER); PTHR19134:SF274 (PANTHER); IPR016130 (PROSITE_PATTERNS); TRANSMEMBRANE (PHOBIUS); TRANSMEMBRANE (PHOBIUS); CYTOPLASMIC_DOMAIN (PHOBIUS); CYTOPLASMIC_DOMAIN (PHOBIUS); NON_CYTOPLASMIC_DOMAIN (PHOBIUS); NON_CYTOPLASMIC_DOMAIN (PHOBIUS); TRANSMEMBRANE (PHOBIUS); CYTOPLASMIC_DOMAIN (PHOBIUS); TRANSMEMBRANE (PHOBIUS); TRANSMEMBRANE (PHOBIUS); NON_CYTOPLASMIC_DOMAIN (PHOBIUS); TRANSMEMBRANE (PHOBIUS); CYTOPLASMIC_DOMAIN (PHOBIUS); IPR000387 (PROSITE_PROFILES); IPR000242 (PROSITE_PROFILES); IPR029021 (SUPERFAMILY); TMhelix (TMHMM); TMhelix (TMHMM); TMhelix (TMHMM); TMhelix (TMHMM)
1266	479618_length_1328_cvg_10.8_tip_1_2	39	low quality protein: twitchin-like	442	0	78.90%	0.124	IPR003599 (SMART); IPR003598 (SMART); IPR013098 (PFAM); IPR013783 (G3DSA:2.60.40.GENE3D); IPR013783 (G3DSA:2.60.40.GENE3D); PTHR19897:SF148 (PANTHER); PTHR19897 (PANTHER); IPR007110 (PROSITE_PROFILES); IPR007110 (PROSITE_PROFILES); SSF48726 (SUPERFAMILY); SSF48726 (SUPERFAMILY); SSF48726 (SUPERFAMILY); SSF48726 (SUPERFAMILY)
1268	479680_length_1330_cvg_27.3_tip_1_5	266	micos complex subunit mic27	443	2.35E-26	54.60%	0.128	IPR019166 (PFAM); PTHR14564:SF1 (PANTHER); PTHR14564 (PANTHER); NON_CYTOPLASMIC_DOMAIN (PHOBIUS); NON_CYTOPLASMIC_DOMAIN (PHOBIUS); CYTOPLASMIC_DOMAIN (PHOBIUS); TRANSMEMBRANE (PHOBIUS); TRANSMEMBRANE (PHOBIUS); TRANSMEMBRANE (PHOBIUS); CYTOPLASMIC_DOMAIN (PHOBIUS)

1284	480518_length_1353_cvg_5.1_tip_1_4	36	zinc finger protein	443	6.82E-33	54.50%	0.101	IPR015880 (SMART); IPR013087 (G3DSA:3.30.160.GENE3D); IPR013087 (G3DSA:3.30.160.GENE3D); PF13465 (PFAM); PF13894 (PFAM); PTHR24409:SF13 (PANTHER); PTHR24409 (PANTHER); IPR007087 (PROSITE_PATTERNS); IPR007087 (PROSITE_PATTERNS); IPR007087 (PROSITE_PATTERNS); IPR007087 (PROSITE_PATTERNS); IPR007087 (PROSITE_PATTERNS); IPR007087 (PROSITE_PATTERNS); IPR007087 (PROSITE_PROFILES); IPR007087 (PROSITE_PROFILES); IPR007087 (PROSITE_PROFILES); IPR007087 (PROSITE_PROFILES); IPR007087 (PROSITE_PROFILES); IPR007087 (PROSITE_PROFILES); SSF57667 (SUPERFAMILY); SSF57667 (SUPERFAMILY); SSF57667 (SUPERFAMILY)
1269	479730_length_1331_cvg_7.9_tip_1_4	40	ribosome biogenesis protein bop1 homolog	444	0	78.20%	0.106	IPR001680 (SMART); IPR015943 (G3DSA:2.130.10.GENE3D); IPR001680 (PFAM); IPR012953 (PFAM); PTHR17605 (PANTHER); IPR019775 (PROSITE_PATTERNS); IPR001680 (PROSITE_PROFILES); IPR017986 (PROSITE_PROFILES); IPR001680 (PROSITE_PROFILES); IPR017986 (PROSITE_PROFILES); IPR017986 (SUPERFAMILY)
1273	479852_length_1334_cvg_3.6_tip_1_2	22	agrin- partial	444	2.14E-25	79.60%	0.246	IPR001791 (PFAM); IPR013320 (G3DSA:2.60.120.GENE3D); PTHR10574 (PANTHER); NON_CYTOPLASMIC_DOMAIN (PHOBIUS); NON_CYTOPLASMIC_DOMAIN (PHOBIUS); TRANSMEMBRANE (PHOBIUS); TRANSMEMBRANE (PHOBIUS); CYTOPLASMIC_DOMAIN (PHOBIUS); TRANSMEMBRANE (PHOBIUS); CYTOPLASMIC_DOMAIN (PHOBIUS); IPR013320 (SUPERFAMILY)
1270	479780_length_1333_cvg_8.2_tip_1_0	68	probable trna n6-adenosine threonylcarbamoyltransferase	445	0	89.00%	0.546 Y	IPR017861 (PRINTS); G3DSA:3.30.420.40 (GENE3D); IPR000905 (PFAM); IPR017861 (TIGRFAM); PTHR11735 (PANTHER); PTHR11735:SF6 (PANTHER); IPR017860 (PROSITE_PATTERNS); SignalP-noTM (SIGNALP_EUK); SSF53067 (SUPERFAMILY)
1272	479822_length_1334_cvg_4.9_tip_1_3	29	dentin sialophosphoprotein isoform x1	445	2.92E-119	67.40%	0.097	IPR015880 (SMART); PF13465 (PFAM); IPR013087 (G3DSA:3.30.160.GENE3D); IPR013087 (G3DSA:3.30.160.GENE3D); IPR013087 (G3DSA:3.30.160.GENE3D); PTHR24409 (PANTHER); IPR007087 (PROSITE_PATTERNS); IPR007087 (PROSITE_PATTERNS); IPR007087 (PROSITE_PATTERNS); IPR007087 (PROSITE_PATTERNS); IPR007087 (PROSITE_PATTERNS); IPR007087 (PROSITE_PATTERNS); IPR007087 (PROSITE_PATTERNS); IPR007087 (PROSITE_PATTERNS); NON_CYTOPLASMIC_DOMAIN (PHOBIUS); TRANSMEMBRANE (PHOBIUS); CYTOPLASMIC_DOMAIN (PHOBIUS); IPR007087 (PROSITE_PROFILES); IPR007087 (PROSITE_PROFILES); IPR007087 (PROSITE_PROFILES); IPR007087 (PROSITE_PROFILES); IPR007087 (PROSITE_PROFILES); IPR007087 (PROSITE_PROFILES); IPR007087 (PROSITE_PROFILES); IPR007087 (PROSITE_PROFILES); IPR007087 (PROSITE_PROFILES); IPR007087 (PROSITE_PROFILES); SSF57667 (SUPERFAMILY); SSF57667 (SUPERFAMILY); SSF57667 (SUPERFAMILY); SSF57667 (SUPERFAMILY); SSF57667 (SUPERFAMILY); SSF57667 (SUPERFAMILY); TMhelix (TMHMM)
1274	479910_length_1336_cvg_67.7_tip_1_2	1593	beta-galactosidase-1-like protein 2	445	2.14E-108	61.80%	0.134	IPR001944 (PRINTS); IPR001944 (PFAM); IPR013781 (G3DSA:3.20.20.GENE3D); IPR008979 (G3DSA:2.60.120.GENE3D); PTHR23421:SF66 (PANTHER); IPR001944 (PANTHER); IPR008979 (SUPERFAMILY); IPR017853 (SUPERFAMILY)
1275	479960_length_1337_cvg_12.4_tip_1_5	82	phospholipase lecithinase hemolysin	445	1.27E-16	44.80%	0.185	TRANSMEMBRANE (PHOBIUS); NON_CYTOPLASMIC_DOMAIN (PHOBIUS); TRANSMEMBRANE (PHOBIUS); TRANSMEMBRANE (PHOBIUS); CYTOPLASMIC_DOMAIN (PHOBIUS); NON_CYTOPLASMIC_DOMAIN (PHOBIUS); TRANSMEMBRANE (PHOBIUS); NON_CYTOPLASMIC_DOMAIN (PHOBIUS); CYTOPLASMIC_DOMAIN (PHOBIUS); TMhelix (TMHMM); TMhelix (TMHMM)
1277	480096_length_1341_cvg_2.0_tip_1_2	12	d-erythrose-4-phosphate dehydrogenase	447	0	99.90%	0.123	IPR020831 (PRINTS); IPR020828 (SMART); IPR006422 (TIGRFAM); IPR020829 (PFAM); IPR020828 (PFAM); G3DSA:3.30.360.10 (GENE3D); IPR016040 (G3DSA:3.40.50.GENE3D); IPR020831 (PANTHER); PTHR10836:SF33 (PANTHER); IPR020830 (PROSITE_PATTERNS); IPR006422 (HAMAP); SSF55347 (SUPERFAMILY); SSF51735 (SUPERFAMILY)
1278	480200_length_1343_cvg_8.0_tip_1_1	40	guanylate kinase isoform x2	448	1.27E-89	83.40%	0.247	IPR008145 (SMART); IPR008145 (PFAM); G3DSA:3.30.63.10 (GENE3D); IPR017665 (TIGRFAM); IPR027417 (G3DSA:3.40.50.GENE3D); IPR027417 (G3DSA:3.40.50.GENE3D); PTHR23117 (PANTHER); PTHR23117:SF13 (PANTHER); NON_CYTOPLASMIC_DOMAIN (PHOBIUS); TRANSMEMBRANE (PHOBIUS); CYTOPLASMIC_DOMAIN (PHOBIUS); TRANSMEMBRANE (PHOBIUS); NON_CYTOPLASMIC_DOMAIN (PHOBIUS); IPR008144 (PROSITE_PROFILES); IPR027417 (SUPERFAMILY); TMhelix (TMHMM)

1279	480236_length_1344_cvg_3.9_tip_1_1	30	threonine synthase-like 2	448	3.96E-108	72.50%	0.141	G3DSA:3.40.50.1100 (GENE3D); IPR029144 (G3DSA:3.90.1380.GENE3D); IPR029144 (PFAM); PTHR10314 (PANTHER); IPR027457 (PTHR10314:PANTHER); IPR001926 (SUPERFAMILY)
1280	480238_length_1344_cvg_54.3_tip_1_4	452	superoxide dismutase	448	2.39E-43	66.30%	0.147	IPR001424 (PRINTS); IPR001424 (G3DSA:2.60.40.GENE3D); IPR001424 (PFAM); PTHR10003 (PANTHER); IPR018152 (PROSITE_PATTERNS); IPR018152 (PROSITE_PATTERNS); NON_CYTOPLASMIC_DOMAIN (PHOBIUS); TRANSMEMBRANE (PHOBIUS); CYTOPLASMIC_DOMAIN (PHOBIUS); IPR001424 (SUPERFAMILY)
1281	480276_length_1346_cvg_4.7_tip_1_5	38	gpi-anchor transamidase	448	1.88E-106	75.40%	0.106	IPR001096 (PRINTS); IPR001096 (PFAM); IPR001096 (PANTHER); IPR028361 (PTHR12000:PANTHER); NON_CYTOPLASMIC_DOMAIN (PHOBIUS); TRANSMEMBRANE (PHOBIUS); TRANSMEMBRANE (PHOBIUS); CYTOPLASMIC_DOMAIN (PHOBIUS); NON_CYTOPLASMIC_DOMAIN (PHOBIUS); TRANSMEMBRANE (PHOBIUS); CYTOPLASMIC_DOMAIN (PHOBIUS); TMhelix (TMHMM); TMhelix (TMHMM); TMhelix (TMHMM)
1282	480454_length_1352_cvg_5.4_tip_1_1	36	leukocyte elastase inhibitor a-like	451	5.64E-49	56.60%	0.128	IPR023796 (SMART); IPR023796 (PFAM); G3DSA:3.30.497.10 (GENE3D); IPR000215 (PANTHER); NON_CYTOPLASMIC_DOMAIN (PHOBIUS); NON_CYTOPLASMIC_DOMAIN (PHOBIUS); CYTOPLASMIC_DOMAIN (PHOBIUS); TRANSMEMBRANE (PHOBIUS); TRANSMEMBRANE (PHOBIUS); TRANSMEMBRANE (PHOBIUS); CYTOPLASMIC_DOMAIN (PHOBIUS); NON_CYTOPLASMIC_DOMAIN (PHOBIUS); TRANSMEMBRANE (PHOBIUS); TRANSMEMBRANE (PHOBIUS); CYTOPLASMIC_DOMAIN (PHOBIUS); IPR023796 (SUPERFAMILY); TMhelix (TMHMM); TMhelix (TMHMM); TMhelix (TMHMM)
1283	480460_length_1352_cvg_2.0_tip_1_1	16	early growth response	451	3.99E-114	57.00%	0.103	IPR015880 (SMART); PF13465 (PFAM); IPR013087 (G3DSA:3.30.160.GENE3D); IPR013087 (G3DSA:3.30.160.GENE3D); IPR013087 (G3DSA:3.30.160.GENE3D); PTHR10042 (PANTHER); PTHR10042:SF24 (PANTHER); IPR007087 (PROSITE_PATTERNS); IPR007087 (PROSITE_PATTERNS); IPR007087 (PROSITE_PATTERNS); IPR007087 (PROSITE_PROFILES); IPR007087 (PROSITE_PROFILES); IPR007087 (PROSITE_PROFILES); SSF57667 (SUPERFAMILY); SSF57667 (SUPERFAMILY)
1286	480662_length_1357_cvg_6.8_tip_1_4	52	cleavage stimulation factor subunit 1-like	452	0	85.70%	0.138	IPR001680 (SMART); IPR015943 (G3DSA:2.130.10.GENE3D); IPR001680 (PFAM); PTHR22840 (PANTHER); PTHR22840:SF8 (PANTHER); IPR019775 (PROSITE_PATTERNS); IPR001680 (PROSITE_PROFILES); IPR001680 (PROSITE_PROFILES); IPR001680 (PROSITE_PROFILES); IPR001680 (PROSITE_PROFILES); IPR017986 (PROSITE_PROFILES); IPR017986 (SUPERFAMILY)
1287	480764_length_1359_cvg_6.4_tip_1_1	46	activated cdc42 kinase 1 isoform x2	453	0	79.70%	0.229	IPR001245 (PRINTS); IPR020635 (SMART); G3DSA:1.10.510.10 (GENE3D); IPR001245 (PFAM); G3DSA:2.30.30.40 (GENE3D); G3DSA:3.30.200.20 (GENE3D); IPR030220 (PTHR24418:PANTHER); PTHR24418 (PANTHER); IPR017441 (PROSITE_PATTERNS); IPR008266 (PROSITE_PATTERNS); IPR000719 (PROSITE_PROFILES); IPR011009 (SUPERFAMILY)
1288	480844_length_1362_cvg_62.0_tip_1_2	395	cyclin-g-associated kinase-like	454	9.20E-155	78.50%	0.172	IPR002290 (SMART); G3DSA:3.30.200.20 (GENE3D); G3DSA:1.10.510.10 (GENE3D); IPR000719 (PFAM); PTHR23172:SF19 (PANTHER); PTHR23172 (PANTHER); IPR008271 (PROSITE_PATTERNS); IPR000719 (PROSITE_PROFILES); IPR011009 (SUPERFAMILY)
1290	480888_length_1363_cvg_55.7_tip_1_1	663	cathepsin l	454	0	80.90%	0.139	IPR000668 (PRINTS); IPR000668 (SMART); IPR013201 (SMART); IPR000668 (PFAM); IPR013201 (PFAM); G3DSA:3.90.70.10 (GENE3D); IPR013128 (PANTHER); PTHR12411:SF57 (PANTHER); IPR025660 (PROSITE_PATTERNS); IPR000169 (PROSITE_PATTERNS); IPR025661 (PROSITE_PATTERNS); CYTOPLASMIC_DOMAIN (PHOBIUS); TRANSMEMBRANE (PHOBIUS); TRANSMEMBRANE (PHOBIUS); NON_CYTOPLASMIC_DOMAIN (PHOBIUS); TRANSMEMBRANE (PHOBIUS); NON_CYTOPLASMIC_DOMAIN (PHOBIUS); CYTOPLASMIC_DOMAIN (PHOBIUS); SSF54001 (SUPERFAMILY); TMhelix (TMHMM)
1289	480874_length_1363_cvg_8.3_tip_1_0	66	lysyl-trna synthetase	455	0	85.80%	0.11	Coil (COILS); IPR018149 (PRINTS); IPR004364 (PFAM); IPR004365 (PFAM); IPR012340 (G3DSA:2.40.50.GENE3D); IPR002313 (TIGRFAM); G3DSA:3.30.930.10 (GENE3D); IPR018150 (PANTHER); PTHR22594:SF4 (PANTHER); IPR006195 (PROSITE_PROFILES); IPR012340 (SUPERFAMILY); SSF55681 (SUPERFAMILY)
1291	480944_length_1365_cvg_78.9_tip_1_2	134111	carboxypeptidase b-like	455	6.11E-127	62.40%	0.395 Y	IPR000834 (PRINTS); IPR000834 (SMART); IPR000834 (PFAM); IPR003146 (PFAM); G3DSA:3.40.630.10 (GENE3D); IPR003146 (G3DSA:3.30.70.GENE3D); PTHR11705 (PANTHER); IPR000834 (PROSITE_PATTERNS); SSF53187 (SUPERFAMILY); IPR009020 (SUPERFAMILY)

1292	480978_length_1366_cvg_5.6_tip_1_5	33	alk tyrosine kinase receptor isoform x1	455	9.06E-112	58.40%	0.124		IPR002172 (SMART); IPR000998 (SMART); IPR002172 (G3DSA:4.10.400.GENE3D); IPR000998 (PFAM); IPR002172 (PFAM); PTHR23282 (PANTHER); IPR023415 (PROSITE_PATTERNS); IPR002172 (PROSITE_PROFILES); IPR000998 (PROSITE_PROFILES); IPR000998 (PROSITE_PROFILES); IPR002172 (SUPERFAMILY); IPR013320 (SUPERFAMILY); IPR013320 (SUPERFAMILY)
1306	481774_length_1390_cvg_10.9_tip_1_0	63	gamma-interferon-inducible lysosomal thiol reductase	455	4.09E-48	57.80%	0.251		IPR004911 (PFAM); PTHR13234:SF8 (PANTHER); IPR004911 (PANTHER); TRANSMEMBRANE (PHOBIUS); CYTOPLASMIC_DOMAIN (PHOBIUS); NON_CYTOPLASMIC_DOMAIN (PHOBIUS); NON_CYTOPLASMIC_DOMAIN (PHOBIUS); CYTOPLASMIC_DOMAIN (PHOBIUS); TRANSMEMBRANE (PHOBIUS); TRANSMEMBRANE (PHOBIUS); TMhelix (TMHMM); TMhelix (TMHMM)
1307	481780_length_1391_cvg_63.6_tip_1_5	401	copper chaperone for superoxide dismutase	455	1.71E-111	77.00%	0.434	Y	IPR001424 (PRINTS); IPR006121 (PFAM); IPR001424 (PFAM); IPR001424 (G3DSA:2.60.40.GENE3D); G3DSA:3.30.70.100 (GENE3D); PTHR10003 (PANTHER); IPR018152 (PROSITE_PATTERNS); SIGNAL_PEPTIDE_N_REGION (PHOBIUS); SIGNAL_PEPTIDE_C_REGION (PHOBIUS); TRANSMEMBRANE (PHOBIUS); CYTOPLASMIC_DOMAIN (PHOBIUS); NON_CYTOPLASMIC_DOMAIN (PHOBIUS); SIGNAL_PEPTIDE (PHOBIUS); SIGNAL_PEPTIDE_H_REGION (PHOBIUS); IPR006121 (PROSITE_PROFILES); IPR001424 (SUPERFAMILY); IPR006121 (SUPERFAMILY)
1295	481114_length_1369_cvg_3.3_tip_1_1	31	mam and ldl-receptor class a domain-containing protein 1	456	8.76E-35	46.60%	0.104		IPR002172 (PRINTS); IPR002172 (SMART); G3DSA:2.10.25.10 (GENE3D); IPR002172 (G3DSA:4.10.400.GENE3D); IPR000998 (PFAM); IPR002172 (PFAM); IPR002172 (G3DSA:4.10.400.GENE3D); PTHR23282 (PANTHER); PTHR23282:SF67 (PANTHER); IPR023415 (PROSITE_PATTERNS); IPR023415 (PROSITE_PATTERNS); IPR023415 (PROSITE_PATTERNS); IPR013032 (PROSITE_PATTERNS); TRANSMEMBRANE (PHOBIUS); NON_CYTOPLASMIC_DOMAIN (PHOBIUS); CYTOPLASMIC_DOMAIN (PHOBIUS); TRANSMEMBRANE (PHOBIUS); NON_CYTOPLASMIC_DOMAIN (PHOBIUS); TRANSMEMBRANE (PHOBIUS); CYTOPLASMIC_DOMAIN (PHOBIUS); IPR002172 (PROSITE_PROFILES); IPR000742 (PROSITE_PROFILES); IPR000998 (PROSITE_PROFILES); IPR002172 (PROSITE_PROFILES); IPR002172 (PROSITE_PROFILES); IPR002172 (SUPERFAMILY); SSF57196 (SUPERFAMILY); IPR002172 (SUPERFAMILY); TMhelix (TMHMM)
1297	481134_length_1370_cvg_90.6_tip_1_2	176340	carboxypeptidase b-like	456	1.31E-126	62.00%	0.723	Y	IPR000834 (PRINTS); IPR000834 (SMART); G3DSA:3.40.630.10 (GENE3D); IPR003146 (G3DSA:3.30.70.GENE3D); IPR003146 (PFAM); IPR000834 (PFAM); PTHR11705 (PANTHER); IPR000834 (PROSITE_PATTERNS); SIGNAL_PEPTIDE_C_REGION (PHOBIUS); NON_CYTOPLASMIC_DOMAIN (PHOBIUS); SIGNAL_PEPTIDE_N_REGION (PHOBIUS); SIGNAL_PEPTIDE (PHOBIUS); SIGNAL_PEPTIDE_H_REGION (PHOBIUS); SignalP-noTM (SIGNALP_EUK); SSF53187 (SUPERFAMILY); IPR009020 (SUPERFAMILY)
1314	482070_length_1400_cvg_62.4_tip_1_2	3335	adp-ribosylation factor-like protein 8b-a	456	7.72E-124	97.70%	0.114		IPR006689 (PRINTS); IPR006687 (SMART); IPR003579 (SMART); IPR024156 (SMART); IPR006689 (PFAM); IPR005225 (TIGRFAM); IPR027417 (G3DSA:3.40.50.GENE3D); PTHR11711:SF122 (PANTHER); PTHR11711 (PANTHER); IPR024156 (PROSITE_PROFILES); IPR027417 (SUPERFAMILY)
1293	481090_length_1369_cvg_3.2_tip_1_3	27	upf0454 protein c12orf49 homolog	457	1.59E-106	76.00%	0.102		IPR019352 (PFAM); IPR019352 (PANTHER); CYTOPLASMIC_DOMAIN (PHOBIUS); NON_CYTOPLASMIC_DOMAIN (PHOBIUS); TRANSMEMBRANE (PHOBIUS); TMhelix (TMHMM)
1294	481112_length_1369_cvg_3.0_tip_1_0	22	zinc finger protein	457	1.45E-22	59.40%	0.166		IPR015880 (SMART); IPR013087 (G3DSA:3.30.160.GENE3D); PF13894 (PFAM); IPR013087 (G3DSA:3.30.160.GENE3D); IPR013087 (G3DSA:3.30.160.GENE3D); IPR007087 (PFAM); PTHR24409 (PANTHER); PTHR24409:SF14 (PANTHER); IPR007087 (PROSITE_PATTERNS); IPR007087 (PROSITE_PATTERNS); IPR007087 (PROSITE_PATTERNS); IPR007087 (PROSITE_PATTERNS); IPR007087 (PROSITE_PROFILES); IPR007087 (PROSITE_PROFILES); IPR007087 (PROSITE_PROFILES); IPR007087 (PROSITE_PROFILES); SSF57667 (SUPERFAMILY); SSF57667 (SUPERFAMILY)
1296	481132_length_1370_cvg_2.6_tip_1_1	23	endothelin-converting enzyme 1 isoform x2	457	2.66E-150	66.30%	0.191		IPR018497 (PRINTS); IPR024079 (G3DSA:3.40.390.GENE3D); IPR018497 (PFAM); IPR008753 (PFAM); IPR029733 (PTHR11733:PANTHER); IPR000718 (PANTHER); TRANSMEMBRANE (PHOBIUS); CYTOPLASMIC_DOMAIN (PHOBIUS); NON_CYTOPLASMIC_DOMAIN (PHOBIUS); SSF55486 (SUPERFAMILY); TMhelix (TMHMM)
1298	481206_length_1373_cvg_18.9_tip_1_5	132	15-hydroxyprostaglandin dehydrogenase	457	7.56E-64	63.10%	0.121		IPR002198 (PRINTS); IPR002347 (PRINTS); IPR002198 (PFAM); IPR016040 (G3DSA:3.40.50.GENE3D); PTHR24310 (PANTHER); NON_CYTOPLASMIC_DOMAIN (PHOBIUS); TRANSMEMBRANE (PHOBIUS); CYTOPLASMIC_DOMAIN (PHOBIUS); SSF51735 (SUPERFAMILY)

1299	481332_length_1377_cvg_4.9_tip_1_5	31	tight junction protein zo-3	459	2.89E-42	73.50%	0.1	Coil (COILS); IPR001478 (SMART); IPR001478 (PFAM); IPR001478 (G3DSA:2.30.42.GENE3D); PTHR19964:SF22 (PANTHER); PTHR19964 (PANTHER); IPR001478 (PROSITE_PROFILES); IPR001478 (SUPERFAMILY)
1300	481356_length_1378_cvg_12.3_tip_1_1	117	aspartate--trna cytoplasmic	459	0	92.60%	0.123	IPR002312 (PRINTS); IPR004523 (TIGRFAM); IPR004364 (PFAM); G3DSA:3.30.930.10 (GENE3D); IPR018150 (PANTHER); IPR004523 (PTHR22594:PANTHER); TRANSMEMBRANE (PHOBIUS); CYTOPLASMIC_DOMAIN (PHOBIUS); NON_CYTOPLASMIC_DOMAIN (PHOBIUS); NON_CYTOPLASMIC_DOMAIN (PHOBIUS); TRANSMEMBRANE (PHOBIUS); CYTOPLASMIC_DOMAIN (PHOBIUS); TRANSMEMBRANE (PHOBIUS); IPR006195 (PROSITE_PROFILES); SSF55681 (SUPERFAMILY)
1313	482018_length_1398_cvg_10.7_tip_1_3	80	collagen alpha type	459	6.77E-76	59.00%	0.112	IPR016186 (G3DSA:3.10.100.GENE3D); IPR010515 (PFAM); IPR026917 (PTHR24023:PANTHER); PTHR24023 (PANTHER); IPR016187 (SUPERFAMILY)
1301	481522_length_1382_cvg_4.4_tip_1_3	28	gastrula zinc finger	461	1.27E-43	56.30%	0.107	IPR015880 (SMART); PF13912 (PFAM); IPR013087 (G3DSA:3.30.160.GENE3D); PF13465 (PFAM); IPR013087 (G3DSA:3.30.160.GENE3D); IPR013087 (G3DSA:3.30.160.GENE3D); PF13894 (PFAM); PTHR24409:SF14 (PANTHER); PTHR24409 (PANTHER); IPR007087 (PROSITE_PATTERNS); IPR007087 (PROSITE_PATTERNS); IPR007087 (PROSITE_PATTERNS); IPR007087 (PROSITE_PATTERNS); IPR007087 (PROSITE_PATTERNS); IPR007087 (PROSITE_PATTERNS); IPR007087 (PROSITE_PATTERNS); IPR007087 (PROSITE_PATTERNS); IPR007087 (PROSITE_PATTERNS); IPR007087 (PROSITE_PROFILES); IPR007087 (PROSITE_PROFILES); IPR007087 (PROSITE_PROFILES); IPR007087 (PROSITE_PROFILES); IPR007087 (PROSITE_PROFILES); IPR007087 (PROSITE_PROFILES); IPR007087 (PROSITE_PROFILES); IPR007087 (PROSITE_PROFILES); IPR007087 (PROSITE_PROFILES); SSF57667 (SUPERFAMILY); SSF57667 (SUPERFAMILY); SSF57667 (SUPERFAMILY); SSF57667 (SUPERFAMILY)
1302	481522_length_1382_cvg_4.4_tip_1_4	28	zinc finger protein	461	2.52E-29	57.00%	0.119	IPR015880 (SMART); IPR013087 (G3DSA:3.30.160.GENE3D); PF13465 (PFAM); IPR013087 (G3DSA:3.30.160.GENE3D); PF13894 (PFAM); IPR013087 (G3DSA:3.30.160.GENE3D); IPR013087 (G3DSA:3.30.160.GENE3D); PTHR24409:SF14 (PANTHER); PTHR24409 (PANTHER); IPR007087 (PROSITE_PATTERNS); IPR007087 (PROSITE_PATTERNS); IPR007087 (PROSITE_PATTERNS); IPR007087 (PROSITE_PATTERNS); IPR007087 (PROSITE_PATTERNS); IPR007087 (PROSITE_PROFILES); IPR007087 (PROSITE_PROFILES); IPR007087 (PROSITE_PROFILES); IPR007087 (PROSITE_PROFILES); IPR007087 (PROSITE_PROFILES); SSF57667 (SUPERFAMILY); SSF57667 (SUPERFAMILY); SSF57667 (SUPERFAMILY)
1303	481550_length_1383_cvg_9.1_tip_1_2	58	leucine-rich repeat-containing protein 9-like	461	1.93E-24	56.50%	0.232	IPR003591 (SMART); G3DSA:3.80.10.10 (GENE3D); IPR001611 (PFAM); PTHR10588 (PANTHER); IPR001611 (PROSITE_PROFILES); IPR001611 (PROSITE_PROFILES); IPR001611 (PROSITE_PROFILES); IPR001611 (PROSITE_PROFILES); SSF52075 (SUPERFAMILY)
1304	481558_length_1383_cvg_18.1_tip_1_5	117	annexin a4	461	3.49E-95	67.70%	0.1	IPR001464 (PRINTS); IPR018502 (SMART); IPR018502 (G3DSA:1.10.220.GENE3D); IPR018502 (G3DSA:1.10.220.GENE3D); IPR018502 (G3DSA:1.10.220.GENE3D); IPR018502 (G3DSA:1.10.220.GENE3D); IPR018502 (PFAM); PTHR10502 (PANTHER); IPR018252 (PROSITE_PATTERNS); IPR018252 (PROSITE_PATTERNS); IPR018252 (PROSITE_PATTERNS); SSF47874 (SUPERFAMILY)
1326	482762_length_1420_cvg_33.9_tip_1_4	261	plasminogen-like isoform x2	461	4.60E-115	75.50%	0.107	IPR001314 (PRINTS); IPR001254 (SMART); G3DSA:2.40.10.10 (GENE3D); IPR001254 (PFAM); G3DSA:2.40.10.10 (GENE3D); PTHR24265 (PANTHER); PTHR24265:SF76 (PANTHER); IPR018114 (PROSITE_PATTERNS); IPR018114 (PROSITE_PATTERNS); NON_CYTOPLASMIC_DOMAIN (PHOBIUS); TRANSMEMBRANE (PHOBIUS); CYTOPLASMIC_DOMAIN (PHOBIUS); NON_CYTOPLASMIC_DOMAIN (PHOBIUS); TRANSMEMBRANE (PHOBIUS); CYTOPLASMIC_DOMAIN (PHOBIUS); TRANSMEMBRANE (PHOBIUS); IPR001254 (PROSITE_PROFILES); IPR009003 (SUPERFAMILY); TMhelix (TMHMM); TMhelix (TMHMM)

1305	481768_length_1390_cvg_19.6_tip_1_5	140	dnaj homolog subfamily c member 3-like	463	0	73.40%	0.144	Coil (COILS); IPR001623 (PRINTS); IPR019734 (SMART); IPR001623 (SMART); IPR001623 (PFAM); IPR011990 (G3DSA:1.25.40.GENE3D); IPR011990 (G3DSA:1.25.40.GENE3D); IPR001623 (G3DSA:1.10.287.GENE3D); PF13414 (PFAM); IPR013105 (PFAM); PTHR24078:SF156 (PANTHER); PTHR24078 (PANTHER); TRANSMEMBRANE (PHOBIUS); NON_CYTOPLASMIC_DOMAIN (PHOBIUS); CYTOPLASMIC_DOMAIN (PHOBIUS); IPR013026 (PROSITE_PROFILES); IPR019734 (PROSITE_PROFILES); IPR013026 (PROSITE_PROFILES); IPR001623 (PROSITE_PROFILES); IPR019734 (PROSITE_PROFILES); IPR019734 (PROSITE_PROFILES); IPR019734 (PROSITE_PROFILES); IPR019734 (PROSITE_PROFILES); IPR019734 (PROSITE_PROFILES); SSF48452 (SUPERFAMILY); IPR001623 (SUPERFAMILY); TMhelix (TMHMM)
1316	482098_length_1401_cvg_5.5_tip_1_4	34	protein creg1	463	1.39E-17	74.70%	0.12	PF13883 (PFAM); IPR012349 (G3DSA:2.30.110.GENE3D); PTHR13343:SF17 (PANTHER); PTHR13343 (PANTHER); TRANSMEMBRANE (PHOBIUS); NON_CYTOPLASMIC_DOMAIN (PHOBIUS); CYTOPLASMIC_DOMAIN (PHOBIUS); TRANSMEMBRANE (PHOBIUS); NON_CYTOPLASMIC_DOMAIN (PHOBIUS); CYTOPLASMIC_DOMAIN (PHOBIUS); TRANSMEMBRANE (PHOBIUS); IPR012349 (SUPERFAMILY); TMhelix (TMHMM); TMhelix (TMHMM); TMhelix (TMHMM)
1308	481814_length_1392_cvg_6.1_tip_1_0	68	gastrula zinc finger	464	2.05E-49	57.90%	0.104	IPR015880 (SMART); PF13465 (PFAM); IPR013087 (G3DSA:3.30.160.GENE3D); IPR013087 (G3DSA:3.30.160.GENE3D); IPR013087 (G3DSA:3.30.160.GENE3D); IPR013087 (G3DSA:3.30.160.GENE3D); PF13912 (PFAM); PTHR24409 (PANTHER); IPR007087 (PROSITE_PATTERNS); IPR007087 (PROSITE_PATTERNS); IPR007087 (PROSITE_PATTERNS); IPR007087 (PROSITE_PATTERNS); IPR007087 (PROSITE_PATTERNS); IPR007087 (PROSITE_PATTERNS); IPR007087 (PROSITE_PATTERNS); IPR007087 (PROSITE_PROFILES); IPR007087 (PROSITE_PROFILES); IPR007087 (PROSITE_PROFILES); IPR007087 (PROSITE_PROFILES); IPR007087 (PROSITE_PROFILES); IPR007087 (PROSITE_PROFILES); IPR007087 (PROSITE_PROFILES); IPR007087 (PROSITE_PROFILES); SSF57667 (SUPERFAMILY); SSF57667 (SUPERFAMILY); SSF57667 (SUPERFAMILY)
1309	481828_length_1392_cvg_5.9_tip_1_1	52	3-oxoacyl-acp partial	464	4.43E-138	94.20%	0.183	IPR002198 (PRINTS); IPR002347 (PRINTS); IPR014030 (PFAM); IPR009081 (G3DSA:1.10.1200.GENE3D); IPR009081 (PFAM); PF13561 (PFAM); IPR003231 (TIGRFAM); IPR016038 (G3DSA:3.40.47.GENE3D); IPR016040 (G3DSA:3.40.50.GENE3D); PTHR11712 (PANTHER); IPR006162 (PROSITE_PATTERNS); IPR020904 (PROSITE_PATTERNS); SIGNAL_PEPTIDE_H_REGION (PHOBIUS); CYTOPLASMIC_DOMAIN (PHOBIUS); SIGNAL_PEPTIDE_N_REGION (PHOBIUS); SIGNAL_PEPTIDE (PHOBIUS); TRANSMEMBRANE (PHOBIUS); NON_CYTOPLASMIC_DOMAIN (PHOBIUS); SIGNAL_PEPTIDE_C_REGION (PHOBIUS); IPR003231 (HAMAP); IPR009081 (PROSITE_PROFILES); IPR009081 (SUPERFAMILY); SSF51735 (SUPERFAMILY); IPR016039 (SUPERFAMILY)
1310	481872_length_1394_cvg_59.7_tip_1_1	995	peptidyl-prolyl cis-trans isomerase e	465	1.72E-168	84.30%	0.114	IPR002130 (PRINTS); IPR000504 (SMART); IPR000504 (PFAM); IPR029000 (G3DSA:2.40.100.GENE3D); IPR012677 (G3DSA:3.30.70.GENE3D); IPR002130 (PFAM); IPR024936 (PANTHER); PTHR11071:SF242 (PANTHER); IPR020892 (PROSITE_PATTERNS); IPR000504 (PROSITE_PROFILES); IPR002130 (PROSITE_PROFILES); SSF54928 (SUPERFAMILY); IPR029000 (SUPERFAMILY)
1311	481990_length_1398_cvg_4.7_tip_1_2	37	zinc finger protein 271-like	466	1.64E-63	66.80%	0.102	IPR015880 (SMART); IPR013087 (G3DSA:3.30.160.GENE3D); IPR007087 (PFAM); PF13465 (PFAM); IPR013087 (G3DSA:3.30.160.GENE3D); IPR013087 (G3DSA:3.30.160.GENE3D); PTHR24409 (PANTHER); IPR007087 (PROSITE_PATTERNS); IPR007087 (PROSITE_PATTERNS); IPR007087 (PROSITE_PATTERNS); IPR007087 (PROSITE_PATTERNS); IPR007087 (PROSITE_PATTERNS); IPR007087 (PROSITE_PATTERNS); IPR007087 (PROSITE_PATTERNS); IPR007087 (PROSITE_PATTERNS); TRANSMEMBRANE (PHOBIUS); TRANSMEMBRANE (PHOBIUS); NON_CYTOPLASMIC_DOMAIN (PHOBIUS); CYTOPLASMIC_DOMAIN (PHOBIUS); TRANSMEMBRANE (PHOBIUS); TRANSMEMBRANE (PHOBIUS); CYTOPLASMIC_DOMAIN (PHOBIUS); CYTOPLASMIC_DOMAIN (PHOBIUS); NON_CYTOPLASMIC_DOMAIN (PHOBIUS); IPR007087 (PROSITE_PROFILES); IPR007087 (PROSITE_PROFILES); IPR007087 (PROSITE_PROFILES); IPR007087 (PROSITE_PROFILES); IPR007087 (PROSITE_PROFILES); IPR007087 (PROSITE_PROFILES); IPR007087 (PROSITE_PROFILES); IPR007087 (PROSITE_PROFILES); SSF57667 (SUPERFAMILY); SSF57667 (SUPERFAMILY); SSF57667 (SUPERFAMILY); SSF57667 (SUPERFAMILY); TMhelix (TMHMM); TMhelix (TMHMM); TMhelix (TMHMM); TMhelix (TMHMM)

1312	482004_length_1398_cvg_10.5_tip_1_5	82	wd repeat-containing protein 3	466	2.37E-143	69.20%	0.357 Y	IPR020472 (PRINTS); IPR001680 (SMART); IPR015943 (G3DSA:2.130.10.GENE3D); IPR001680 (PFAM); PTHR19853:SF0 (PANTHER); PTHR19853 (PANTHER); IPR019775 (PROSITE_PATTERNS); IPR019775 (PROSITE_PATTERNS); IPR001680 (PROSITE_PROFILES); IPR001680 (PROSITE_PROFILES); IPR001680 (PROSITE_PROFILES); IPR001680 (PROSITE_PROFILES); IPR017986 (PROSITE_PROFILES); IPR017986 (SUPERFAMILY); TMhelix (TMHMM)
1315	482076_length_1400_cvg_3.1_tip_1_5	25	fat-like cadherin-related tumor suppressor homolog isoform x1	466	1.93E-143	76.20%	0.106	IPR002126 (PRINTS); IPR002126 (SMART); IPR002126 (G3DSA:2.60.40.GENE3D); IPR002126 (G3DSA:2.60.40.GENE3D); IPR002126 (PFAM); PTHR24026:SF39 (PANTHER); PTHR24026 (PANTHER); IPR020894 (PROSITE_PATTERNS); TRANSMEMBRANE (PHOBIUS); CYTOPLASMIC_DOMAIN (PHOBIUS); NON_CYTOPLASMIC_DOMAIN (PHOBIUS); IPR002126 (PROSITE_PROFILES); IPR002126 (PROSITE_PROFILES); IPR002126 (PROSITE_PROFILES); IPR015919 (SUPERFAMILY); IPR015919 (SUPERFAMILY); IPR015919 (SUPERFAMILY); TMhelix (TMHMM)
1329	482860_length_1423_cvg_106.2_tip_1_2	138989	transmembrane protease serine 4	466	4.08E-39	52.40%	0.106	IPR001314 (PRINTS); IPR001254 (SMART); G3DSA:2.40.10.10 (GENE3D); G3DSA:2.40.10.10 (GENE3D); G3DSA:2.40.10.10 (GENE3D); IPR001254 (PFAM); PTHR24268 (PANTHER); IPR018114 (PROSITE_PATTERNS); IPR018114 (PROSITE_PATTERNS); IPR001254 (PROSITE_PROFILES); IPR009003 (SUPERFAMILY)
1327	482802_length_1422_cvg_5.2_tip_1_0	32	interstitial collagenase	467	7.09E-24	61.10%	0.153	IPR018487 (SMART); IPR000585 (G3DSA:2.110.10.GENE3D); IPR018487 (PFAM); PTHR10201 (PANTHER); IPR018486 (PROSITE_PATTERNS); CYTOPLASMIC_DOMAIN (PHOBIUS); NON_CYTOPLASMIC_DOMAIN (PHOBIUS); TRANSMEMBRANE (PHOBIUS); IPR018487 (PROSITE_PROFILES); IPR018487 (PROSITE_PROFILES); IPR000585 (SUPERFAMILY)
1334	483152_length_1431_cvg_59.0_tip_1_5	431	cytolysin src-1-like isoform x2	467	7.84E-25	51.20%	0.111	IPR015926 (G3DSA:2.60.270.GENE3D); IPR009104 (PFAM); NON_CYTOPLASMIC_DOMAIN (PHOBIUS); TRANSMEMBRANE (PHOBIUS); CYTOPLASMIC_DOMAIN (PHOBIUS); IPR015926 (SUPERFAMILY); TMhelix (TMHMM)
1317	482188_length_1403_cvg_5.6_tip_1_1	33	nucleolar protein 58-like	468	0	83.60%	0.166	IPR012976 (SMART); IPR002687 (PFAM); IPR012976 (PFAM); IPR012974 (PFAM); PTHR10894 (PANTHER); IPR002687 (PROSITE_PROFILES); SSF89124 (SUPERFAMILY)
1318	482370_length_1409_cvg_42.8_tip_1_3	505	cyclin-h	470	7.64E-157	81.70%	0.121	IPR013763 (SMART); IPR013763 (G3DSA:1.10.472.GENE3D); IPR013763 (G3DSA:1.10.472.GENE3D); IPR006671 (PFAM); IPR027081 (TIGRFAM); IPR015429 (PANTHER); IPR027081 (PTHR10026:PANTHER); IPR013763 (SUPERFAMILY); IPR013763 (SUPERFAMILY)
1319	482376_length_1409_cvg_8.8_tip_1_4	55	scp-like extracellular domain containing protein 1	470	6.33E-37	49.50%	0.101	IPR002413 (PRINTS); IPR001283 (PRINTS); IPR014044 (SMART); IPR002172 (SMART); IPR002172 (G3DSA:4.10.400.GENE3D); IPR014044 (G3DSA:3.40.33.GENE3D); IPR002172 (PFAM); IPR002172 (G3DSA:4.10.400.GENE3D); IPR014044 (PFAM); IPR001283 (PANTHER); IPR018244 (PROSITE_PATTERNS); IPR023415 (PROSITE_PATTERNS); IPR023415 (PROSITE_PATTERNS); TRANSMEMBRANE (PHOBIUS); CYTOPLASMIC_DOMAIN (PHOBIUS); NON_CYTOPLASMIC_DOMAIN (PHOBIUS); IPR002172 (PROSITE_PROFILES); IPR002172 (PROSITE_PROFILES); IPR002172 (SUPERFAMILY); IPR014044 (SUPERFAMILY); IPR002172 (SUPERFAMILY); TMhelix (TMHMM)
1320	482402_length_1410_cvg_16.6_tip_1_4	112	zinc finger protein 431-like isoform x2	470	1.99E-37	53.40%	0.115	IPR015880 (SMART); IPR013087 (G3DSA:3.30.160.GENE3D); PF13912 (PFAM); PF13465 (PFAM); IPR013087 (G3DSA:3.30.160.GENE3D); PTHR24409:SF13 (PANTHER); PTHR24409 (PANTHER); IPR007087 (PROSITE_PATTERNS); IPR007087 (PROSITE_PATTERNS); IPR007087 (PROSITE_PATTERNS); IPR007087 (PROSITE_PATTERNS); IPR007087 (PROSITE_PROFILES); IPR007087 (PROSITE_PROFILES); IPR007087 (PROSITE_PROFILES); IPR007087 (PROSITE_PROFILES); SSF57667 (SUPERFAMILY); SSF57667 (SUPERFAMILY); SSF57667 (SUPERFAMILY)
1321	482456_length_1412_cvg_62.8_tip_1_3	14361	disulfide isomerase	471	0	81.70%	0.622 Y	PR00421 (PRINTS); IPR005788 (TIGRFAM); IPR013766 (PFAM); IPR012336 (G3DSA:3.40.30.GENE3D); PF13848 (PFAM); IPR012336 (G3DSA:3.40.30.GENE3D); IPR005792 (TIGRFAM); PTHR18929 (PANTHER); PTHR18929:SF48 (PANTHER); IPR017937 (PROSITE_PATTERNS); IPR017937 (PROSITE_PATTERNS); SIGNAL_PEPTIDE_H_REGION (PHOBIUS); SIGNAL_PEPTIDE_N_REGION (PHOBIUS); SIGNAL_PEPTIDE (PHOBIUS); SIGNAL_PEPTIDE_C_REGION (PHOBIUS); NON_CYTOPLASMIC_DOMAIN (PHOBIUS); IPR012336 (PROSITE_PROFILES); IPR012336 (PROSITE_PROFILES); SignalP-noTM (SIGNALP_EUK); IPR012336 (SUPERFAMILY); IPR012336 (SUPERFAMILY); IPR012336 (SUPERFAMILY); IPR012336 (SUPERFAMILY)

1322	482486_length_1413_cvg_17.5_tip_1_2	125	tho complex subunit 3	471	0	86.90%	0.104	IPR020472 (PRINTS); IPR001680 (SMART); IPR001680 (PFAM); IPR011659 (PFAM); IPR015943 (G3DSA:2.130.10.GENE3D); IPR015943 (G3DSA:2.130.10.GENE3D); PTHR22839 (PANTHER); IPR019775 (PROSITE_PATTERNS); TRANSMEMBRANE (PHOBIUS); NON_CYTOPLASMIC_DOMAIN (PHOBIUS); CYTOPLASMIC_DOMAIN (PHOBIUS); TRANSMEMBRANE (PHOBIUS); NON_CYTOPLASMIC_DOMAIN (PHOBIUS); IPR001680 (PROSITE_PROFILES); IPR001680 (PROSITE_PROFILES); IPR001680 (PROSITE_PROFILES); IPR017986 (PROSITE_PROFILES); IPR017986 (SUPERFAMILY); TMhelix (TMHMM); TMhelix (TMHMM)
1323	482516_length_1414_cvg_2.3_tip_1_2	22	kae1 qri7 required for threonylcarbamoyladenosine t a37 formation in trna	471	5.73E-35	99.60%	0.111	IPR000905 (PFAM); G3DSA:3.30.420.40 (GENE3D); PTHR11735 (PANTHER); SSF53067 (SUPERFAMILY)
1324	482552_length_1415_cvg_6.9_tip_1_1	66	beta-galactosidase and hygromycin phosphotransferase fusion protein	472	0	100.00%	0.124	IPR006101 (PRINTS); IPR006103 (PFAM); IPR013781 (G3DSA:3.20.20.GENE3D); IPR013812 (G3DSA:2.60.40.GENE3D); IPR008979 (G3DSA:2.60.120.GENE3D); IPR006102 (PFAM); IPR006104 (PFAM); PTHR10066:SF57 (PANTHER); PTHR10066 (PANTHER); IPR023232 (PROSITE_PATTERNS); IPR023230 (PROSITE_PATTERNS); IPR006102 (SUPERFAMILY); IPR008979 (SUPERFAMILY); IPR017853 (SUPERFAMILY)
1325	482658_length_1417_cvg_11.2_tip_1_2	80	mitochondrial cardiolipin hydrolase-like	472	1.28E-60	66.40%	0.151	G3DSA:3.30.870.10 (GENE3D); IPR025202 (PFAM); PTHR21248 (PANTHER); TRANSMEMBRANE (PHOBIUS); TRANSMEMBRANE (PHOBIUS); CYTOPLASMIC_DOMAIN (PHOBIUS); TRANSMEMBRANE (PHOBIUS); CYTOPLASMIC_DOMAIN (PHOBIUS); NON_CYTOPLASMIC_DOMAIN (PHOBIUS); NON_CYTOPLASMIC_DOMAIN (PHOBIUS); TRANSMEMBRANE (PHOBIUS); CYTOPLASMIC_DOMAIN (PHOBIUS); IPR001736 (PROSITE_PROFILES); SSF56024 (SUPERFAMILY); TMhelix (TMHMM); TMhelix (TMHMM); TMhelix (TMHMM)
1339	483386_length_1441_cvg_8.1_tip_1_3	56	arylsulfatase b-like	473	6.23E-143	66.20%	0.285	IPR017849 (G3DSA:3.40.720.GENE3D); G3DSA:3.30.1120.10 (GENE3D); IPR000917 (PFAM); PTHR10342:SF206 (PANTHER); PTHR10342 (PANTHER); IPR024607 (PROSITE_PATTERNS); SIGNAL_PEPTIDE_C_REGION (PHOBIUS); SIGNAL_PEPTIDE_N_REGION (PHOBIUS); SIGNAL_PEPTIDE_H_REGION (PHOBIUS); NON_CYTOPLASMIC_DOMAIN (PHOBIUS); SIGNAL_PEPTIDE (PHOBIUS); IPR017850 (SUPERFAMILY)
1328	482836_length_1422_cvg_63.1_tip_0_3	205020	suppressor of tumorigenicity 14 protein	474	9.87E-70	51.60%	0.103	IPR002172 (PRINTS); IPR001254 (SMART); IPR002172 (SMART); IPR001254 (PFAM); G3DSA:2.40.10.10 (GENE3D); IPR002172 (G3DSA:4.10.400.GENE3D); G3DSA:2.40.10.10 (GENE3D); G3DSA:4.10.1220.10 (GENE3D); IPR002172 (G3DSA:4.10.400.GENE3D); IPR002172 (PFAM); PTHR24256 (PANTHER); IPR018114 (PROSITE_PATTERNS); IPR023415 (PROSITE_PATTERNS); IPR023415 (PROSITE_PATTERNS); IPR018114 (PROSITE_PATTERNS); IPR023415 (PROSITE_PATTERNS); IPR023415 (PROSITE_PATTERNS); IPR002172 (PROSITE_PROFILES); IPR002172 (PROSITE_PROFILES); IPR002172 (PROSITE_PROFILES); IPR002172 (PROSITE_PROFILES); IPR001254 (PROSITE_PROFILES); IPR002172 (SUPERFAMILY); IPR002172 (SUPERFAMILY); IPR002172 (SUPERFAMILY); IPR002172 (SUPERFAMILY); IPR009003 (SUPERFAMILY)
1357	484108_length_1466_cvg_80.7_tip_1_0	170597	suppressor of tumorigenicity 14 protein homolog isoform x2	474	1.76E-52	45.80%	0.104	IPR002172 (PRINTS); IPR001254 (SMART); IPR002172 (SMART); G3DSA:2.40.10.10 (GENE3D); IPR002172 (PFAM); G3DSA:2.40.10.10 (GENE3D); IPR002172 (G3DSA:4.10.400.GENE3D); IPR002172 (G3DSA:4.10.400.GENE3D); G3DSA:2.40.10.10 (GENE3D); IPR001254 (PFAM); PTHR24256:SF106 (PANTHER); PTHR24256 (PANTHER); IPR023415 (PROSITE_PATTERNS); IPR023415 (PROSITE_PATTERNS); IPR023415 (PROSITE_PATTERNS); NON_CYTOPLASMIC_DOMAIN (PHOBIUS); TRANSMEMBRANE (PHOBIUS); CYTOPLASMIC_DOMAIN (PHOBIUS); IPR001254 (PROSITE_PROFILES); IPR002172 (PROSITE_PROFILES); IPR002172 (PROSITE_PROFILES); IPR002172 (PROSITE_PROFILES); IPR009003 (SUPERFAMILY); IPR002172 (SUPERFAMILY); IPR002172 (SUPERFAMILY); IPR002172 (SUPERFAMILY)
1330	482912_length_1424_cvg_12.8_tip_1_0	80	sparc-related modular calcium-binding protein 1-like isoform x2	475	5.65E-124	56.90%	0.103	IPR000716 (SMART); G3DSA:3.30.60.30 (GENE3D); IPR000716 (PFAM); IPR019577 (PFAM); IPR002350 (PFAM); IPR000716 (G3DSA:4.10.800.GENE3D); IPR011992 (G3DSA:1.10.238.GENE3D); PTHR12352 (PANTHER); PTHR12352:SF3 (PANTHER); IPR018247 (PROSITE_PATTERNS); IPR000716 (PROSITE_PATTERNS); IPR000716 (PROSITE_PATTERNS); IPR018247 (PROSITE_PATTERNS); IPR000716 (PROSITE_PROFILES); IPR002350 (PROSITE_PROFILES); IPR000716 (PROSITE_PROFILES); SSF100895 (SUPERFAMILY); IPR000716 (SUPERFAMILY); IPR000716 (SUPERFAMILY); SSF47473 (SUPERFAMILY); SSF47473 (SUPERFAMILY)

1331	482978_length_1427_cvg_55.6_tip_1_5	346	probable cytosolic iron-sulfur protein assembly protein ciao1	475	2.60E-167	80.30%	0.099	IPR020472 (PRINTS); IPR001680 (SMART); IPR015943 (G3DSA:2.130.10.GENE3D); IPR001680 (PFAM); PTHR19920:SF0 (PANTHER); PTHR19920 (PANTHER); IPR019775 (PROSITE_PATTERNS); IPR001680 (PROSITE_PROFILES); IPR001680 (PROSITE_PROFILES); IPR001680 (PROSITE_PROFILES); IPR001680 (PROSITE_PROFILES); IPR017986 (PROSITE_PROFILES); IPR001680 (PROSITE_PROFILES); IPR028608 (HAMAP); IPR001680 (PROSITE_PROFILES); IPR017986 (SUPERFAMILY)
1332	483076_length_1429_cvg_30.1_tip_1_5	165	cytochrome p450	476	4.55E-90	77.60%	0.107	IPR002401 (PRINTS); IPR001128 (PRINTS); IPR001128 (G3DSA:1.10.630.GENE3D); IPR001128 (PFAM); PTHR24290 (PANTHER); PTHR24290:SF0 (PANTHER); IPR017972 (PROSITE_PATTERNS); CYTOPLASMIC_DOMAIN (PHOBIUS); TRANSMEMBRANE (PHOBIUS); NON_CYTOPLASMIC_DOMAIN (PHOBIUS); IPR001128 (SUPERFAMILY)
1347	483674_length_1449_cvg_49.5_tip_1_3	336	hypothetical protein DAPPUDRAFT_196945	476	5.35E-89	64.80%	0.149	IPR019791 (PRINTS); IPR019791 (G3DSA:1.10.640.GENE3D); IPR019791 (PFAM); PTHR11475 (PANTHER); TRANSMEMBRANE (PHOBIUS); NON_CYTOPLASMIC_DOMAIN (PHOBIUS); CYTOPLASMIC_DOMAIN (PHOBIUS); IPR019791 (PROSITE_PROFILES); IPR010255 (SUPERFAMILY)
1333	483082_length_1429_cvg_2.1_tip_1_0	23	endoplasmic reticulum mannosyl-oligosaccharide - alpha-mannosidase	477	4.70E-135	81.30%	0.105	IPR001382 (PRINTS); IPR001382 (PFAM); IPR001382 (G3DSA:1.50.10.GENE3D); PTHR11742:SF7 (PANTHER); IPR001382 (PANTHER); IPR001382 (SUPERFAMILY)
1335	483156_length_1432_cvg_9.8_tip_1_4	71	lachesin	477	9.25E-88	66.30%	0.116	IPR003599 (SMART); IPR003598 (SMART); IPR013783 (G3DSA:2.60.40.GENE3D); IPR013098 (PFAM); IPR013783 (G3DSA:2.60.40.GENE3D); PTHR19831:SF43 (PANTHER); PTHR19831 (PANTHER); IPR007110 (PROSITE_PROFILES); IPR007110 (PROSITE_PROFILES); IPR007110 (PROSITE_PROFILES); SSF48726 (SUPERFAMILY); SSF48726 (SUPERFAMILY); SSF48726 (SUPERFAMILY)
1336	483158_length_1432_cvg_8.1_tip_1_1	41	syntaxin-12	477	4.29E-95	72.00%	0.196	IPR006011 (SMART); IPR000727 (SMART); PF14523 (PFAM); G3DSA:1.20.58.70 (GENE3D); IPR000727 (PFAM); PTHR19957 (PANTHER); PTHR19957:SF87 (PANTHER); IPR006012 (PROSITE_PATTERNS); TRANSMEMBRANE (PHOBIUS); TRANSMEMBRANE (PHOBIUS); NON_CYTOPLASMIC_DOMAIN (PHOBIUS); CYTOPLASMIC_DOMAIN (PHOBIUS); CYTOPLASMIC_DOMAIN (PHOBIUS); IPR000727 (PROSITE_PROFILES); IPR010989 (SUPERFAMILY); TMhelix (TMHMM); TMhelix (TMHMM)
1337	483218_length_1434_cvg_8.3_tip_1_2	53	limbic system-associated membrane protein	478	4.55E-150	69.70%	0.122	IPR003598 (SMART); IPR003599 (SMART); IPR013783 (G3DSA:2.60.40.GENE3D); IPR013098 (PFAM); IPR013783 (G3DSA:2.60.40.GENE3D); IPR003961 (PFAM); IPR013783 (G3DSA:2.60.40.GENE3D); PTHR19831 (PANTHER); PTHR19831:SF47 (PANTHER); IPR007110 (PROSITE_PROFILES); IPR007110 (PROSITE_PROFILES); IPR003961 (PROSITE_PROFILES); IPR007110 (PROSITE_PROFILES); SSF48726 (SUPERFAMILY); IPR003961 (SUPERFAMILY); SSF48726 (SUPERFAMILY); SSF48726 (SUPERFAMILY)
1338	483352_length_1439_cvg_61.2_tip_1_5	2986	muscle m-line assembly protein unc-89-like	479	5.04E-158	75.90%	0.103	Coil (COILS); IPR003599 (SMART); IPR003598 (SMART); IPR013098 (PFAM); IPR013783 (G3DSA:2.60.40.GENE3D); IPR013783 (G3DSA:2.60.40.GENE3D); IPR013783 (G3DSA:2.60.40.GENE3D); IPR013783 (G3DSA:2.60.40.GENE3D); PTHR19897 (PANTHER); PTHR19897:SF148 (PANTHER); IPR007110 (PROSITE_PROFILES); IPR007110 (PROSITE_PROFILES); IPR007110 (PROSITE_PROFILES); SSF48726 (SUPERFAMILY); SSF48726 (SUPERFAMILY); SSF48726 (SUPERFAMILY); SSF48726 (SUPERFAMILY)
1362	484396_length_1476_cvg_61.0_tip_1_4	641	hyaluronidase-like	480	2.93E-73	56.90%	0.105	IPR018155 (PRINTS); IPR013785 (G3DSA:3.20.20.GENE3D); IPR018155 (PFAM); PTHR11769:SF8 (PANTHER); IPR018155 (PANTHER); NON_CYTOPLASMIC_DOMAIN (PHOBIUS); CYTOPLASMIC_DOMAIN (PHOBIUS); TRANSMEMBRANE (PHOBIUS); IPR017853 (SUPERFAMILY)
1340	483392_length_1441_cvg_5.7_tip_1_0	40	apical endosomal glycoprotein	481	5.92E-15	46.20%	0.131	IPR000998 (PFAM); TRANSMEMBRANE (PHOBIUS); CYTOPLASMIC_DOMAIN (PHOBIUS); NON_CYTOPLASMIC_DOMAIN (PHOBIUS); TRANSMEMBRANE (PHOBIUS); NON_CYTOPLASMIC_DOMAIN (PHOBIUS); IPR000998 (PROSITE_PROFILES); IPR000998 (PROSITE_PROFILES); IPR013320 (SUPERFAMILY); TMhelix (TMHMM); TMhelix (TMHMM)

1341	483480_length_1444_cvg_63.5_tip_1_2	10717	st14a protein	481	5.16E-44	50.30%	0.852 Y	IPR001314 (PRINTS); IPR001254 (SMART); IPR002172 (SMART); G3DSA:2.40.10.10 (GENE3D); G3DSA:2.40.10.10 (GENE3D); IPR002172 (G3DSA:4.10.400.GENE3D); IPR001254 (PFAM); G3DSA:2.40.10.10 (GENE3D); IPR002172 (PFAM); PTHR24268 (PANTHER); IPR018114 (PROSITE_PATTERNS); IPR018114 (PROSITE_PATTERNS); IPR023415 (PROSITE_PATTERNS); SIGNAL_PEPTIDE_N_REGION (PHOBIUS); CYTOPLASMIC_DOMAIN (PHOBIUS); SIGNAL_PEPTIDE_C_REGION (PHOBIUS); SIGNAL_PEPTIDE (PHOBIUS); TRANSMEMBRANE (PHOBIUS); SIGNAL_PEPTIDE_H_REGION (PHOBIUS); NON_CYTOPLASMIC_DOMAIN (PHOBIUS); IPR001254 (PROSITE_PROFILES); IPR002172 (PROSITE_PROFILES); SignalP-noTM (SIGNALP_EUK); SignalP-TM (SIGNALP_GRAM_POSITIVE); IPR002172 (SUPERFAMILY); IPR009003 (SUPERFAMILY); TMhelix (TMHMM)
1359	484188_length_1468_cvg_25.3_tip_1_0	210	kielin chordin-like protein	481	1.10E-34	48.20%	0.316	IPR001791 (SMART); IPR013320 (SUPERFAMILY)
1342	483544_length_1446_cvg_72.0_tip_1_2	1785	26kda protease	482	4.36E-46	56.90%	0.306	IPR001314 (PRINTS); IPR002172 (SMART); IPR001254 (SMART); IPR002172 (PFAM); G3DSA:2.40.10.10 (GENE3D); IPR002172 (G3DSA:4.10.400.GENE3D); G3DSA:2.40.10.10 (GENE3D); IPR001254 (PFAM); G3DSA:2.40.10.10 (GENE3D); PTHR24268 (PANTHER); IPR018114 (PROSITE_PATTERNS); IPR023415 (PROSITE_PATTERNS); IPR018114 (PROSITE_PATTERNS); NON_CYTOPLASMIC_DOMAIN (PHOBIUS); TRANSMEMBRANE (PHOBIUS); CYTOPLASMIC_DOMAIN (PHOBIUS); IPR001254 (PROSITE_PROFILES); IPR002172 (PROSITE_PROFILES); IPR009003 (SUPERFAMILY); IPR002172 (SUPERFAMILY)
1343	483580_length_1447_cvg_9.2_tip_1_2	71	3-ketodihydrosphingosine reductase	482	1.95E-119	71.70%	0.117	IPR002347 (PRINTS); IPR002198 (PRINTS); IPR002198 (PFAM); IPR016040 (G3DSA:3.40.50.GENE3D); PTHR24320:SF1 (PANTHER); PTHR24320 (PANTHER); IPR020904 (PROSITE_PATTERNS); NON_CYTOPLASMIC_DOMAIN (PHOBIUS); TRANSMEMBRANE (PHOBIUS); NON_CYTOPLASMIC_DOMAIN (PHOBIUS); CYTOPLASMIC_DOMAIN (PHOBIUS); TRANSMEMBRANE (PHOBIUS); NON_CYTOPLASMIC_DOMAIN (PHOBIUS); TRANSMEMBRANE (PHOBIUS); CYTOPLASMIC_DOMAIN (PHOBIUS); TRANSMEMBRANE (PHOBIUS); SSF51735 (SUPERFAMILY); TMhelix (TMHMM); TMhelix (TMHMM); TMhelix (TMHMM); TMhelix (TMHMM)
1344	483614_length_1448_cvg_18.2_tip_1_1	127	pitslre serine threonine-protein kinase cdc2l1	483	0	88.10%	0.102	IPR002290 (SMART); G3DSA:3.30.200.20 (GENE3D); IPR000719 (PFAM); G3DSA:1.10.510.10 (GENE3D); PTHR24056:SF107 (PANTHER); PTHR24056 (PANTHER); IPR008271 (PROSITE_PATTERNS); IPR000719 (PROSITE_PROFILES); IPR011009 (SUPERFAMILY)
1345	483628_length_1448_cvg_9.8_tip_1_1	68	protein kinase domain-containing cytoplasmic	483	2.02E-104	58.60%	0.142	IPR022049 (PFAM); G3DSA:3.30.200.20 (GENE3D); G3DSA:1.10.510.10 (GENE3D); PTHR26392 (PANTHER); PTHR26392:SF71 (PANTHER); TRANSMEMBRANE (PHOBIUS); CYTOPLASMIC_DOMAIN (PHOBIUS); NON_CYTOPLASMIC_DOMAIN (PHOBIUS); IPR000719 (PROSITE_PROFILES); IPR011009 (SUPERFAMILY); TMhelix (TMHMM)
1346	483648_length_1449_cvg_9.0_tip_1_4	69	adiponectin receptor protein	483	0	80.70%	0.138	IPR004254 (PFAM); IPR004254 (PANTHER); PTHR20855:SF15 (PANTHER); CYTOPLASMIC_DOMAIN (PHOBIUS); CYTOPLASMIC_DOMAIN (PHOBIUS); NON_CYTOPLASMIC_DOMAIN (PHOBIUS); TRANSMEMBRANE (PHOBIUS); NON_CYTOPLASMIC_DOMAIN (PHOBIUS); TRANSMEMBRANE (PHOBIUS); CYTOPLASMIC_DOMAIN (PHOBIUS); CYTOPLASMIC_DOMAIN (PHOBIUS); NON_CYTOPLASMIC_DOMAIN (PHOBIUS); TRANSMEMBRANE (PHOBIUS); NON_CYTOPLASMIC_DOMAIN (PHOBIUS); TRANSMEMBRANE (PHOBIUS); TRANSMEMBRANE (PHOBIUS); TRANSMEMBRANE (PHOBIUS); CYTOPLASMIC_DOMAIN (PHOBIUS); TRANSMEMBRANE (PHOBIUS); TRANSMEMBRANE (PHOBIUS); TMhelix (TMHMM); TMhelix (TMHMM); TMhelix (TMHMM); TMhelix (TMHMM); TMhelix (TMHMM); TMhelix (TMHMM); TMhelix (TMHMM)
1348	483684_length_1450_cvg_13.3_tip_1_4	89	zinc finger protein 239- partial	483	1.05E-56	66.00%	0.102	IPR015880 (SMART); PF13465 (PFAM); IPR013087 (G3DSA:3.30.160.GENE3D); IPR013087 (G3DSA:3.30.160.GENE3D); IPR013087 (G3DSA:3.30.160.GENE3D); PTHR24409 (PANTHER); IPR007087 (PROSITE_PATTERNS); IPR007087 (PROSITE_PATTERNS); IPR007087 (PROSITE_PATTERNS); IPR007087 (PROSITE_PATTERNS); IPR007087 (PROSITE_PATTERNS); IPR007087 (PROSITE_PROFILES); IPR007087 (PROSITE_PROFILES); IPR007087 (PROSITE_PROFILES); IPR007087 (PROSITE_PROFILES); IPR007087 (PROSITE_PROFILES); IPR007087 (PROSITE_PROFILES); IPR007087 (PROSITE_PROFILES); IPR007087 (PROSITE_PROFILES); SSF57667 (SUPERFAMILY); SSF57667 (SUPERFAMILY); SSF57667 (SUPERFAMILY); SSF57667 (SUPERFAMILY)

1350	483712_length_1451_cvg_10.6_tip_1_5	83	retinol dehydrogenase 12	483	2.72E-95	61.10%	0.215		IPR002347 (PRINTS); IPR016040 (G3DSA:3.40.50.GENE3D); IPR002198 (PFAM); PTHR24320 (PANTHER); PTHR24320:SF55 (PANTHER); TRANSMEMBRANE (PHOBIUS); NON_CYTOPLASMIC_DOMAIN (PHOBIUS); NON_CYTOPLASMIC_DOMAIN (PHOBIUS); TRANSMEMBRANE (PHOBIUS); CYTOPLASMIC_DOMAIN (PHOBIUS); SSF51735 (SUPERFAMILY); TMhelix (TMHMM); TMhelix (TMHMM); TMhelix (TMHMM); TMhelix (TMHMM)
1349	483686_length_1450_cvg_23.0_tip_1_0	161	zinc finger protein 235-like	484	5.76E-40	49.10%	0.1		IPR015880 (SMART); IPR003656 (SMART); IPR007087 (PFAM); PF13894 (PFAM); IPR013087 (G3DSA:3.30.160.GENE3D); PF13465 (PFAM); IPR013087 (G3DSA:3.30.160.GENE3D); IPR013087 (G3DSA:3.30.160.GENE3D); PTHR24409:SF14 (PANTHER); PTHR24409 (PANTHER); IPR007087 (PROSITE_PATTERNS); IPR007087 (PROSITE_PATTERNS); IPR007087 (PROSITE_PATTERNS); IPR007087 (PROSITE_PATTERNS); IPR007087 (PROSITE_PATTERNS); IPR007087 (PROSITE_PATTERNS); IPR007087 (PROSITE_PATTERNS); IPR007087 (PROSITE_PROFILES); IPR007087 (PROSITE_PROFILES); IPR007087 (PROSITE_PROFILES); IPR007087 (PROSITE_PROFILES); IPR007087 (PROSITE_PROFILES); IPR007087 (PROSITE_PROFILES); IPR007087 (PROSITE_PROFILES); SSF57667 (SUPERFAMILY); SSF57667 (SUPERFAMILY); SSF57667 (SUPERFAMILY); SSF57667 (SUPERFAMILY)
1351	483730_length_1451_cvg_60.5_tip_1_1	1617	ribosome biogenesis protein wdr12 homolog	484	1.58E-180	74.50%	0.134		IPR020472 (PRINTS); IPR001680 (SMART); IPR015943 (G3DSA:2.130.10.GENE3D); IPR001680 (PFAM); IPR012972 (PFAM); IPR015943 (G3DSA:2.130.10.GENE3D); PTHR19855 (PANTHER); IPR019775 (PROSITE_PATTERNS); IPR001680 (PROSITE_PROFILES); IPR028599 (HAMAP); IPR001680 (PROSITE_PROFILES); IPR001680 (PROSITE_PROFILES); IPR001680 (PROSITE_PROFILES); IPR017986 (PROSITE_PROFILES); IPR017986 (SUPERFAMILY)
1352	483792_length_1454_cvg_10.2_tip_1_5	56	vanin-like protein 1	484	3.96E-38	52.20%	0.166		IPR003010 (PFAM); IPR003010 (G3DSA:3.60.110.GENE3D); IPR012101 (PANTHER); TRANSMEMBRANE (PHOBIUS); NON_CYTOPLASMIC_DOMAIN (PHOBIUS); CYTOPLASMIC_DOMAIN (PHOBIUS); TRANSMEMBRANE (PHOBIUS); CYTOPLASMIC_DOMAIN (PHOBIUS); IPR003010 (PROSITE_PROFILES); IPR003010 (SUPERFAMILY); TMhelix (TMHMM)
1363	484408_length_1477_cvg_66.4_tip_0_1	92717	transmembrane protease serine 6	484	5.90E-45	49.40%	0.834	Y	IPR001314 (PRINTS); IPR002172 (SMART); IPR001254 (SMART); IPR002172 (PFAM); IPR002172 (G3DSA:4.10.400.GENE3D); G3DSA:2.40.10.10 (GENE3D); IPR001254 (PFAM); G3DSA:2.40.10.10 (GENE3D); G3DSA:2.40.10.10 (GENE3D); PTHR24256 (PANTHER); IPR018114 (PROSITE_PATTERNS); IPR023415 (PROSITE_PATTERNS); SIGNAL_PEPTIDE_H_REGION (PHOBIUS); SIGNAL_PEPTIDE_C_REGION (PHOBIUS); SIGNAL_PEPTIDE_N_REGION (PHOBIUS); SIGNAL_PEPTIDE (PHOBIUS); NON_CYTOPLASMIC_DOMAIN (PHOBIUS); IPR001254 (PROSITE_PROFILES); IPR002172 (PROSITE_PROFILES); SignalP-noTM (SIGNALP_EUK); IPR002172 (SUPERFAMILY); IPR009003 (SUPERFAMILY)
1364	484492_length_1480_cvg_39.5_tip_1_4	224	trans-l-3-hydroxyproline dehydratase	484	1.05E-143	76.00%	0.301		G3DSA:3.10.310.10 (GENE3D); G3DSA:3.10.310.10 (GENE3D); IPR008794 (PFAM); CYTOPLASMIC_DOMAIN (PHOBIUS); TRANSMEMBRANE (PHOBIUS); TRANSMEMBRANE (PHOBIUS); NON_CYTOPLASMIC_DOMAIN (PHOBIUS); NON_CYTOPLASMIC_DOMAIN (PHOBIUS); CYTOPLASMIC_DOMAIN (PHOBIUS); TRANSMEMBRANE (PHOBIUS); SSF54506 (SUPERFAMILY); TMhelix (TMHMM); TMhelix (TMHMM); TMhelix (TMHMM)
1353	483806_length_1455_cvg_18.5_tip_1_1	152	leucine-rich repeat protein 1 isoform x2	485	2.22E-120	64.10%	0.246		PR00019 (PRINTS); IPR003591 (SMART); IPR001611 (PFAM); G3DSA:3.80.10.10 (GENE3D); PTHR23155 (PANTHER); IPR001611 (PROSITE_PROFILES); IPR001611 (PROSITE_PROFILES); IPR001611 (PROSITE_PROFILES); IPR001611 (PROSITE_PROFILES); IPR001611 (PROSITE_PROFILES); SSF52058 (SUPERFAMILY)

1354	483854_length_1457_cvg_8.3_tip_1_2	56	gastrula zinc finger	485	6.95E-73	55.10%	0.105		IPR015880 (SMART); IPR013087 (G3DSA:3.30.160.GENE3D); IPR007087 (PFAM); PF13894 (PFAM); IPR013087 (G3DSA:3.30.160.GENE3D); IPR013087 (G3DSA:3.30.160.GENE3D); IPR013087 (G3DSA:3.30.160.GENE3D); IPR013087 (G3DSA:3.30.160.GENE3D); PF13465 (PFAM); PTHR24409 (PANTHER); IPR007087 (PROSITE_PATTERNS); IPR007087 (PROSITE_PATTERNS); IPR007087 (PROSITE_PATTERNS); IPR007087 (PROSITE_PATTERNS); IPR007087 (PROSITE_PATTERNS); IPR007087 (PROSITE_PATTERNS); IPR007087 (PROSITE_PATTERNS); IPR007087 (PROSITE_PATTERNS); IPR007087 (PROSITE_PATTERNS); IPR007087 (PROSITE_PATTERNS); IPR007087 (PROSITE_PROFILES); IPR007087 (PROSITE_PROFILES); IPR007087 (PROSITE_PROFILES); IPR007087 (PROSITE_PROFILES); IPR007087 (PROSITE_PROFILES); IPR007087 (PROSITE_PROFILES); IPR007087 (PROSITE_PROFILES); IPR007087 (PROSITE_PROFILES); IPR007087 (PROSITE_PROFILES); IPR007087 (PROSITE_PROFILES); IPR007087 (PROSITE_PROFILES); SSF57667 (SUPERFAMILY); SSF57667 (SUPERFAMILY); SSF57667 (SUPERFAMILY); SSF57667 (SUPERFAMILY); SSF57667 (SUPERFAMILY); SSF57667 (SUPERFAMILY)
1361	484382_length_1476_cvg_45.3_tip_1_4	424	26s protease regulatory subunit 4	485	0	97.10%	0.106		IPR003593 (SMART); G3DSA:1.10.8.60 (GENE3D); IPR003959 (PFAM); IPR005937 (TIGRFAM); IPR027417 (G3DSA:3.40.50.GENE3D); PTHR23073 (PANTHER); PTHR23073:SF9 (PANTHER); IPR003960 (PROSITE_PATTERNS); IPR027417 (SUPERFAMILY)
1365	484506_length_1480_cvg_5.7_tip_1_0	56	zinc finger protein 492-like	485	4.55E-93	74.80%	0.103		IPR015880 (SMART); IPR013087 (G3DSA:3.30.160.GENE3D); PF13912 (PFAM); IPR013087 (G3DSA:3.30.160.GENE3D); IPR007087 (PFAM); PF13465 (PFAM); IPR013087 (G3DSA:3.30.160.GENE3D); PTHR24409 (PANTHER); IPR007087 (PROSITE_PATTERNS); IPR007087 (PROSITE_PATTERNS); IPR007087 (PROSITE_PATTERNS); IPR007087 (PROSITE_PATTERNS); IPR007087 (PROSITE_PATTERNS); IPR007087 (PROSITE_PATTERNS); IPR007087 (PROSITE_PATTERNS); NON_CYTOPLASMIC_DOMAIN (PHOBIUS); CYTOPLASMIC_DOMAIN (PHOBIUS); TRANSMEMBRANE (PHOBIUS); IPR007087 (PROSITE_PROFILES); IPR007087 (PROSITE_PROFILES); IPR007087 (PROSITE_PROFILES); IPR007087 (PROSITE_PROFILES); IPR007087 (PROSITE_PROFILES); IPR007087 (PROSITE_PROFILES); IPR007087 (PROSITE_PROFILES); SSF57667 (SUPERFAMILY); SSF57667 (SUPERFAMILY); SSF57667 (SUPERFAMILY); SSF57667 (SUPERFAMILY); TMhelix (TMHMM)
1355	483926_length_1460_cvg_14.4_tip_1_2	88	wd repeat-containing protein partial	486	1.20E-165	70.50%	0.241		IPR001680 (SMART); IPR007319 (PFAM); IPR015943 (G3DSA:2.130.10.GENE3D); IPR001680 (PFAM); PTHR13889:SF2 (PANTHER); PTHR13889 (PANTHER); IPR017986 (PROSITE_PROFILES); IPR001680 (PROSITE_PROFILES); IPR001680 (PROSITE_PROFILES); IPR011047 (SUPERFAMILY); TMhelix (TMHMM)
1367	484520_length_1481_cvg_60.6_tip_1_5	777	ectonucleotide pyrophosphatase phosphodiesterase family member 6	486	3.39E-120	55.50%	0.624	Y	IPR002591 (PFAM); IPR017849 (G3DSA:3.40.720.GENE3D); IPR024873 (PANTHER); NON_CYTOPLASMIC_DOMAIN (PHOBIUS); SIGNAL_PEPTIDE_H_REGION (PHOBIUS); CYTOPLASMIC_DOMAIN (PHOBIUS); SIGNAL_PEPTIDE_C_REGION (PHOBIUS); SIGNAL_PEPTIDE (PHOBIUS); TRANSMEMBRANE (PHOBIUS); SIGNAL_PEPTIDE_N_REGION (PHOBIUS); SignalP-noTM (SIGNALP_EUK); IPR017850 (SUPERFAMILY)
1368	484524_length_1481_cvg_22.4_tip_1_2	168	annexin b9-like isoform x3	486	3.94E-127	73.20%	0.11		IPR001464 (PRINTS); IPR002391 (PRINTS); IPR018502 (SMART); IPR018502 (PFAM); IPR018502 (G3DSA:1.10.220.GENE3D); IPR018502 (G3DSA:1.10.220.GENE3D); IPR018502 (G3DSA:1.10.220.GENE3D); IPR018502 (G3DSA:1.10.220.GENE3D); PTHR10502 (PANTHER); IPR018252 (PROSITE_PATTERNS); IPR018252 (PROSITE_PATTERNS); TRANSMEMBRANE (PHOBIUS); NON_CYTOPLASMIC_DOMAIN (PHOBIUS); NON_CYTOPLASMIC_DOMAIN (PHOBIUS); TRANSMEMBRANE (PHOBIUS); CYTOPLASMIC_DOMAIN (PHOBIUS); SSF47874 (SUPERFAMILY)
1370	484816_length_1491_cvg_67.3_tip_1_5	2316	limulus clotting factor partial	486	2.03E-68	51.90%	0.141		IPR001314 (PRINTS); IPR001254 (SMART); IPR001881 (SMART); IPR000742 (SMART); IPR000436 (SMART); PF14670 (PFAM); G3DSA:2.10.25.10 (GENE3D); G3DSA:2.40.10.10 (GENE3D); G3DSA:2.40.10.10 (GENE3D); G3DSA:2.10.70.10 (GENE3D); G3DSA:2.40.10.10 (GENE3D); IPR000436 (PFAM); IPR001254 (PFAM); PTHR24265 (PANTHER); IPR018114 (PROSITE_PATTERNS); IPR000152 (PROSITE_PATTERNS); IPR018097 (PROSITE_PATTERNS); IPR013032 (PROSITE_PATTERNS); IPR018114 (PROSITE_PATTERNS); IPR000436 (PROSITE_PROFILES); IPR001254 (PROSITE_PROFILES); IPR000436 (PROSITE_PROFILES); IPR000742 (PROSITE_PROFILES); IPR000436 (SUPERFAMILY); SSF57196 (SUPERFAMILY); IPR009003 (SUPERFAMILY)

1373	484904_length_1494_cvg_57.6_tip_1_4	500	platelet-activating factor acetylhydrolase	487	2.00E-102	57.20%	0.192	Coil (COILS); IPR005065 (PFAM); IPR029058 (G3DSA:3.40.50.GENE3D); IPR005065 (PANTHER); IPR029058 (SUPERFAMILY)
1356	484074_length_1465_cvg_27.4_tip_1_5	193	epidermal retinol dehydrogenase 2-like isoform x2	488	6.37E-112	71.70%	0.102	Coil (COILS); IPR002198 (PRINTS); IPR002347 (PRINTS); IPR016040 (G3DSA:3.40.50.GENE3D); IPR002198 (PFAM); PTHR24316 (PANTHER); PTHR24316:SF289 (PANTHER); TRANSMEMBRANE (PHOBIUS); NON_CYTOPLASMIC_DOMAIN (PHOBIUS); NON_CYTOPLASMIC_DOMAIN (PHOBIUS); TRANSMEMBRANE (PHOBIUS); CYTOPLASMIC_DOMAIN (PHOBIUS); CYTOPLASMIC_DOMAIN (PHOBIUS); TRANSMEMBRANE (PHOBIUS); TRANSMEMBRANE (PHOBIUS); NON_CYTOPLASMIC_DOMAIN (PHOBIUS); SSF51735 (SUPERFAMILY); TMhelix (TMHMM); TMhelix (TMHMM); TMhelix (TMHMM)
1358	484112_length_1466_cvg_22.0_tip_1_1	167	upf0378 protein kiaa0100	489	8.84E-62	54.20%	0.091	TRANSMEMBRANE (PHOBIUS); NON_CYTOPLASMIC_DOMAIN (PHOBIUS); CYTOPLASMIC_DOMAIN (PHOBIUS); NON_CYTOPLASMIC_DOMAIN (PHOBIUS); TRANSMEMBRANE (PHOBIUS); TMhelix (TMHMM)
1371	484844_length_1492_cvg_6.7_tip_1_2	44	zinc finger protein 84-like isoform x2	489	1.28E-62	55.10%	0.098	IPR015880 (SMART); PF13894 (PFAM); IPR013087 (G3DSA:3.30.160.GENE3D); IPR013087 (G3DSA:3.30.160.GENE3D); IPR013087 (G3DSA:3.30.160.GENE3D); IPR013087 (G3DSA:3.30.160.GENE3D); PF13465 (PFAM); IPR007087 (PFAM); PTHR24409 (PANTHER); IPR007087 (PROSITE_PATTERNS); IPR007087 (PROSITE_PATTERNS); IPR007087 (PROSITE_PATTERNS); IPR007087 (PROSITE_PATTERNS); IPR007087 (PROSITE_PATTERNS); IPR007087 (PROSITE_PATTERNS); IPR007087 (PROSITE_PATTERNS); IPR007087 (PROSITE_PATTERNS); IPR007087 (PROSITE_PATTERNS); CYTOPLASMIC_DOMAIN (PHOBIUS); NON_CYTOPLASMIC_DOMAIN (PHOBIUS); NON_CYTOPLASMIC_DOMAIN (PHOBIUS); CYTOPLASMIC_DOMAIN (PHOBIUS); TRANSMEMBRANE (PHOBIUS); TRANSMEMBRANE (PHOBIUS); TRANSMEMBRANE (PHOBIUS); IPR007087 (PROSITE_PROFILES); IPR007087 (PROSITE_PROFILES); IPR007087 (PROSITE_PROFILES); IPR007087 (PROSITE_PROFILES); IPR007087 (PROSITE_PROFILES); IPR007087 (PROSITE_PROFILES); IPR007087 (PROSITE_PROFILES); IPR007087 (PROSITE_PROFILES); IPR007087 (PROSITE_PROFILES); IPR007087 (PROSITE_PROFILES); SSF57667 (SUPERFAMILY); SSF57667 (SUPERFAMILY); SSF57667 (SUPERFAMILY); SSF57667 (SUPERFAMILY); SSF57667 (SUPERFAMILY); TMhelix (TMHMM); TMhelix (TMHMM)
1376	484986_length_1497_cvg_36.0_tip_1_3	250	n-acetylglucosamine-6- partial	490	4.38E-71	53.00%	0.117	IPR000917 (PFAM); IPR017849 (G3DSA:3.40.720.GENE3D); PTHR10342:SF208 (PANTHER); PTHR10342 (PANTHER); IPR017850 (SUPERFAMILY)
1360	484350_length_1474_cvg_8.0_tip_1_1	59	exocyst complex component 6	491	0	82.20%	0.242	Coil (COILS); Coil (COILS); IPR007225 (PFAM); IPR007225 (PANTHER); TRANSMEMBRANE (PHOBIUS); NON_CYTOPLASMIC_DOMAIN (PHOBIUS); NON_CYTOPLASMIC_DOMAIN (PHOBIUS); CYTOPLASMIC_DOMAIN (PHOBIUS); TRANSMEMBRANE (PHOBIUS); TMhelix (TMHMM); TMhelix (TMHMM)
1366	484512_length_1481_cvg_10.2_tip_1_4	89	apolipoprotein d	494	1.38E-07	45.60%	0.129	IPR012674 (G3DSA:2.40.128.GENE3D); CYTOPLASMIC_DOMAIN (PHOBIUS); NON_CYTOPLASMIC_DOMAIN (PHOBIUS); TRANSMEMBRANE (PHOBIUS); IPR011038 (SUPERFAMILY)
1369	484680_length_1486_cvg_5.7_tip_1_1	53	aldehyde mitochondrial	495	0	85.60%	0.105	IPR016162 (G3DSA:3.40.605.GENE3D); IPR016163 (G3DSA:3.40.309.GENE3D); IPR015590 (PFAM); PTHR11699:SF151 (PANTHER); PTHR11699 (PANTHER); IPR029510 (PROSITE_PATTERNS); IPR016160 (PROSITE_PATTERNS); IPR016161 (SUPERFAMILY)
1372	484878_length_1493_cvg_2.0_tip_1_2	14	maltase a2-like isoform x1	497	1.48E-83	53.20%	0.1	IPR006589 (SMART); IPR006047 (PFAM); IPR013781 (G3DSA:3.20.20.GENE3D); PTHR10357:SF133 (PANTHER); IPR015902 (PANTHER); NON_CYTOPLASMIC_DOMAIN (PHOBIUS); TRANSMEMBRANE (PHOBIUS); NON_CYTOPLASMIC_DOMAIN (PHOBIUS); CYTOPLASMIC_DOMAIN (PHOBIUS); TRANSMEMBRANE (PHOBIUS); CYTOPLASMIC_DOMAIN (PHOBIUS); TRANSMEMBRANE (PHOBIUS); IPR017853 (SUPERFAMILY)
1374	484924_length_1495_cvg_19.2_tip_1_3	137	d-aspartate oxidase	499	1.82E-98	63.70%	0.114	IPR006076 (PFAM); IPR016040 (G3DSA:3.40.50.GENE3D); IPR023209 (PANTHER); PTHR11530:SF0 (PANTHER); IPR006181 (PROSITE_PATTERNS); NON_CYTOPLASMIC_DOMAIN (PHOBIUS); TRANSMEMBRANE (PHOBIUS); CYTOPLASMIC_DOMAIN (PHOBIUS); TRANSMEMBRANE (PHOBIUS); NON_CYTOPLASMIC_DOMAIN (PHOBIUS); SSF51971 (SUPERFAMILY); SSF54373 (SUPERFAMILY); TMhelix (TMHMM); TMhelix (TMHMM)

1375	484970_length_1497_cvg_19.3_tip_1_3	142	zinc finger and scan domain-containing protein 2-like	499	1.18E-60	64.90%	0.211		IPR015880 (SMART); PF13465 (PFAM); IPR013087 (G3DSA:3.30.160.GENE3D); IPR013087 (G3DSA:3.30.160.GENE3D); IPR013087 (G3DSA:3.30.160.GENE3D); PTHR24409 (PANTHER); IPR007087 (PROSITE_PATTERNS); IPR007087 (PROSITE_PATTERNS); IPR007087 (PROSITE_PATTERNS); IPR007087 (PROSITE_PATTERNS); IPR007087 (PROSITE_PATTERNS); IPR007087 (PROSITE_PATTERNS); IPR007087 (PROSITE_PATTERNS); IPR007087 (PROSITE_PATTERNS); IPR007087 (PROSITE_PROFILES); IPR007087 (PROSITE_PROFILES); IPR007087 (PROSITE_PROFILES); IPR007087 (PROSITE_PROFILES); IPR007087 (PROSITE_PROFILES); IPR007087 (PROSITE_PROFILES); IPR007087 (PROSITE_PROFILES); SSF57667 (SUPERFAMILY); SSF57667 (SUPERFAMILY); SSF57667 (SUPERFAMILY); SSF57667 (SUPERFAMILY)
1377	485176_length_1505_cvg_3.5_tip_1_5	20	zinc c2h2 type domain-containing protein	501	2.46E-20	61.70%	0.106		IPR015880 (SMART); IPR007087 (PFAM); PF13894 (PFAM); IPR013087 (G3DSA:3.30.160.GENE3D); PTHR24409:SF14 (PANTHER); PTHR24409 (PANTHER); IPR007087 (PROSITE_PATTERNS); IPR007087 (PROSITE_PATTERNS); IPR007087 (PROSITE_PATTERNS); IPR007087 (PROSITE_PROFILES); IPR007087 (PROSITE_PROFILES); IPR007087 (PROSITE_PROFILES); SSF57667 (SUPERFAMILY); SSF57667 (SUPERFAMILY)
1378	485184_length_1506_cvg_57.1_tip_1_5	421	zinc finger protein ozf-like	502	7.80E-27	52.90%	0.518	Y	IPR000210 (SMART); IPR015880 (SMART); G3DSA:3.30.710.10 (GENE3D); IPR013087 (G3DSA:3.30.160.GENE3D); PF13465 (PFAM); IPR013087 (G3DSA:3.30.160.GENE3D); IPR013087 (G3DSA:3.30.160.GENE3D); IPR013069 (PFAM); PTHR24409 (PANTHER); PTHR24409:SF13 (PANTHER); IPR007087 (PROSITE_PATTERNS); IPR007087 (PROSITE_PATTERNS); IPR007087 (PROSITE_PATTERNS); SIGNAL_PEPTIDE (PHOBIUS); NON_CYTOPLASMIC_DOMAIN (PHOBIUS); SIGNAL_PEPTIDE_H_REGION (PHOBIUS); SIGNAL_PEPTIDE_N_REGION (PHOBIUS); SIGNAL_PEPTIDE_C_REGION (PHOBIUS); IPR007087 (PROSITE_PROFILES); IPR007087 (PROSITE_PROFILES); IPR007087 (PROSITE_PROFILES); IPR000210 (PROSITE_PROFILES); SignalP-noTM (SIGNALP_EUK); IPR011333 (SUPERFAMILY); SSF57667 (SUPERFAMILY); SSF57667 (SUPERFAMILY)
1379	485192_length_1506_cvg_8.1_tip_1_4	74	omega-amidase nit2-like	502	1.00E-143	82.80%	0.513	Y	IPR003010 (G3DSA:3.60.110.GENE3D); IPR003010 (PFAM); PTHR23088:SF20 (PANTHER); PTHR23088 (PANTHER); SIGNAL_PEPTIDE_H_REGION (PHOBIUS); TRANSMEMBRANE (PHOBIUS); TRANSMEMBRANE (PHOBIUS); CYTOPLASMIC_DOMAIN (PHOBIUS); SIGNAL_PEPTIDE (PHOBIUS); NON_CYTOPLASMIC_DOMAIN (PHOBIUS); SIGNAL_PEPTIDE_N_REGION (PHOBIUS); SIGNAL_PEPTIDE_C_REGION (PHOBIUS); NON_CYTOPLASMIC_DOMAIN (PHOBIUS); IPR003010 (PROSITE_PROFILES); SignalP-noTM (SIGNALP_EUK); IPR003010 (SUPERFAMILY); TMhelix (TMHMM); TMhelix (TMHMM)
1380	485248_length_1508_cvg_2.3_tip_1_5	19	zinc finger protein 271-like	502	1.16E-65	46.70%	0.098		IPR015880 (SMART); IPR013087 (G3DSA:3.30.160.GENE3D); IPR013087 (G3DSA:3.30.160.GENE3D); IPR013087 (G3DSA:3.30.160.GENE3D); PF13465 (PFAM); IPR007087 (PFAM); PF13894 (PFAM); IPR013087 (G3DSA:3.30.160.GENE3D); IPR013087 (G3DSA:3.30.160.GENE3D); PTHR24409 (PANTHER); PTHR24409:SF14 (PANTHER); IPR007087 (PROSITE_PATTERNS); IPR007087 (PROSITE_PATTERNS); IPR007087 (PROSITE_PATTERNS); IPR007087 (PROSITE_PATTERNS); IPR007087 (PROSITE_PATTERNS); IPR007087 (PROSITE_PATTERNS); IPR007087 (PROSITE_PATTERNS); IPR007087 (PROSITE_PATTERNS); IPR007087 (PROSITE_PATTERNS); IPR007087 (PROSITE_PATTERNS); IPR007087 (PROSITE_PROFILES); IPR007087 (PROSITE_PROFILES); IPR007087 (PROSITE_PROFILES); IPR007087 (PROSITE_PROFILES); IPR007087 (PROSITE_PROFILES); IPR007087 (PROSITE_PROFILES); IPR007087 (PROSITE_PROFILES); IPR007087 (PROSITE_PROFILES); IPR007087 (PROSITE_PROFILES); IPR007087 (PROSITE_PROFILES); SSF57667 (SUPERFAMILY); SSF57667 (SUPERFAMILY); SSF57667 (SUPERFAMILY); SSF57667 (SUPERFAMILY); SSF57667 (SUPERFAMILY); SSF57667 (SUPERFAMILY); SSF57667 (SUPERFAMILY)
1381	485388_length_1513_cvg_8.3_tip_1_4	58	maltase 2-like	504	0	66.40%	0.108		IPR006589 (SMART); IPR006047 (PFAM); G3DSA:3.90.400.10 (GENE3D); IPR013781 (G3DSA:3.20.20.GENE3D); IPR015902 (PANTHER); PTHR10357:SF76 (PANTHER); IPR017853 (SUPERFAMILY)

1382	485418_length_1514_cvg_24.0_tip_0_5	152	calcium calmodulin-dependent serine protein kinase	504	0	74.30%	0.104	IPR001478 (SMART); IPR001452 (SMART); IPR004172 (SMART); IPR008145 (SMART); G3DSA:3.30.63.10 (GENE3D); G3DSA:2.30.30.40 (GENE3D); IPR001478 (PFAM); IPR011511 (PFAM); IPR001478 (G3DSA:2.30.42.GENE3D); IPR014775 (PFAM); IPR027417 (G3DSA:3.40.50.GENE3D); IPR008145 (PFAM); PTHR23122 (PANTHER); IPR020590 (PROSITE_PATTERNS); IPR004172 (PROSITE_PROFILES); IPR008144 (PROSITE_PROFILES); IPR001478 (PROSITE_PROFILES); IPR001452 (PROSITE_PROFILES); SSF101288 (SUPERFAMILY); IPR027417 (SUPERFAMILY); IPR001452 (SUPERFAMILY); IPR001478 (SUPERFAMILY)
1383	485484_length_1517_cvg_40.3_tip_1_3	263	torsin-1b-like isoform x1	506	2.77E-35	53.30%	0.115	IPR027417 (G3DSA:3.40.50.GENE3D); IPR010448 (PFAM); IPR030553 (PTHR10760:PANTHER); IPR010448 (PANTHER); CYTOPLASMIC_DOMAIN (PHOBIUS); TRANSMEMBRANE (PHOBIUS); CYTOPLASMIC_DOMAIN (PHOBIUS); NON_CYTOPLASMIC_DOMAIN (PHOBIUS); TRANSMEMBRANE (PHOBIUS); IPR027417 (SUPERFAMILY); TMhelix (TMHMM); TMhelix (TMHMM)
1384	485534_length_1519_cvg_4.5_tip_1_5	49	purple acid phosphatase	506	0	74.30%	0.116	IPR004843 (PFAM); IPR015914 (G3DSA:2.60.40.GENE3D); IPR029052 (G3DSA:3.60.21.GENE3D); IPR025733 (PFAM); PTHR22953:SF9 (PANTHER); PTHR22953 (PANTHER); IPR008963 (SUPERFAMILY); IPR029052 (SUPERFAMILY)
1385	485540_length_1519_cvg_7.8_tip_1_4	55	microfibrillar-associated protein 1-like	506	9.68E-177	78.20%	0.107	Coil (COILS); Coil (COILS); Coil (COILS); Coil (COILS); IPR009730 (PFAM); PTHR15327:SF0 (PANTHER); IPR009730 (PANTHER)
1386	485696_length_1525_cvg_19.4_tip_1_0	85	methylmalonate-semialdehyde dehydrogenase	509	0	88.50%	0.105	IPR015590 (PFAM); IPR016162 (G3DSA:3.40.605.GENE3D); IPR010061 (TIGRFAM); IPR016163 (G3DSA:3.40.309.GENE3D); PTHR11699 (PANTHER); IPR010061 (PTHR11699:PANTHER); IPR016160 (PROSITE_PATTERNS); IPR016161 (SUPERFAMILY); TMhelix (TMHMM)
1387	485728_length_1527_cvg_8.7_tip_1_1	87	peroxisomal biogenesis factor 7	509	4.02E-123	73.70%	0.106	IPR020472 (PRINTS); IPR001680 (SMART); IPR001680 (PFAM); IPR015943 (G3DSA:2.130.10.GENE3D); PTHR22850 (PANTHER); PTHR22850:SF98 (PANTHER); IPR019775 (PROSITE_PATTERNS); IPR019775 (PROSITE_PATTERNS); IPR019775 (PROSITE_PATTERNS); TRANSMEMBRANE (PHOBIUS); NON_CYTOPLASMIC_DOMAIN (PHOBIUS); CYTOPLASMIC_DOMAIN (PHOBIUS); TRANSMEMBRANE (PHOBIUS); NON_CYTOPLASMIC_DOMAIN (PHOBIUS); IPR001680 (PROSITE_PROFILES); IPR001680 (PROSITE_PROFILES); IPR017986 (PROSITE_PROFILES); IPR001680 (PROSITE_PROFILES); IPR001680 (PROSITE_PROFILES); IPR017986 (SUPERFAMILY); TMhelix (TMHMM); TMhelix (TMHMM)
1388	485774_length_1529_cvg_51.8_tip_1_5	373	3-hydroxybutyrate dehydrogenase type 2	509	1.91E-118	81.00%	0.119	IPR002198 (PRINTS); IPR002347 (PRINTS); IPR002198 (PFAM); IPR016040 (G3DSA:3.40.50.GENE3D); PTHR24321 (PANTHER); PTHR24321:SF3 (PANTHER); IPR020904 (PROSITE_PATTERNS); CYTOPLASMIC_DOMAIN (PHOBIUS); TRANSMEMBRANE (PHOBIUS); NON_CYTOPLASMIC_DOMAIN (PHOBIUS); SSF51735 (SUPERFAMILY); TMhelix (TMHMM)
1389	485856_length_1533_cvg_4.5_tip_1_3	37	aldehyde dehydrogenase mitochondrial	511	0	81.10%	0.115	IPR016163 (G3DSA:3.40.309.GENE3D); IPR015590 (PFAM); IPR016162 (G3DSA:3.40.605.GENE3D); PTHR11699:SF145 (PANTHER); PTHR11699 (PANTHER); IPR029510 (PROSITE_PATTERNS); IPR016160 (PROSITE_PATTERNS); IPR016161 (SUPERFAMILY)
1406	486526_length_1559_cvg_10.0_tip_1_0	78	zinc finger protein 501-like	512	3.39E-47	56.20%	0.1	IPR015880 (SMART); IPR013087 (G3DSA:3.30.160.GENE3D); IPR013087 (G3DSA:3.30.160.GENE3D); IPR013087 (G3DSA:3.30.160.GENE3D); PF13465 (PFAM); IPR007087 (PFAM); IPR013087 (G3DSA:3.30.160.GENE3D); PF13894 (PFAM); PTHR24409:SF14 (PANTHER); PTHR24409 (PANTHER); IPR007087 (PROSITE_PATTERNS); IPR007087 (PROSITE_PATTERNS); IPR007087 (PROSITE_PATTERNS); IPR007087 (PROSITE_PATTERNS); IPR007087 (PROSITE_PATTERNS); IPR007087 (PROSITE_PATTERNS); IPR007087 (PROSITE_PATTERNS); IPR007087 (PROSITE_PATTERNS); IPR007087 (PROSITE_PATTERNS); IPR007087 (PROSITE_PATTERNS); IPR007087 (PROSITE_PROFILES); IPR007087 (PROSITE_PROFILES); IPR007087 (PROSITE_PROFILES); IPR007087 (PROSITE_PROFILES); IPR007087 (PROSITE_PROFILES); IPR007087 (PROSITE_PROFILES); IPR007087 (PROSITE_PROFILES); IPR007087 (PROSITE_PROFILES); IPR007087 (PROSITE_PROFILES); IPR007087 (PROSITE_PROFILES); SSF57667 (SUPERFAMILY); SSF57667 (SUPERFAMILY); SSF57667 (SUPERFAMILY); SSF57667 (SUPERFAMILY); SSF57667 (SUPERFAMILY)

1390	486004_length_1539_cvg_6.5_tip_1_0	48	acidic phospholipase a2 pa4 isoform x2	513	1.00E-42	56.90%	0.898	Y	IPR001211 (PFAM); IPR016090 (G3DSA:1.20.90.GENE3D); PTHR12253 (PANTHER); IPR013090 (PROSITE_PATTERNS); SIGNAL_PEPTIDE (PHOBIUS); NON_CYTOPLASMIC_DOMAIN (PHOBIUS); SIGNAL_PEPTIDE_C_REGION (PHOBIUS); SIGNAL_PEPTIDE_H_REGION (PHOBIUS); SIGNAL_PEPTIDE_N_REGION (PHOBIUS); SignalP-noTM (SIGNALP_GRAM_NEGATIVE); SignalP-TM (SIGNALP_GRAM_POSITIVE); SignalP-noTM (SIGNALP_EUK); IPR016090 (SUPERFAMILY)
1391	486082_length_1541_cvg_8.6_tip_1_2	73	bola-like protein ddb_g0274169	513	9.51E-47	82.00%	0.144		IPR002634 (G3DSA:3.30.300.GENE3D); IPR002634 (PFAM); PTHR12735 (PANTHER); CYTOPLASMIC_DOMAIN (PHOBIUS); TRANSMEMBRANE (PHOBIUS); NON_CYTOPLASMIC_DOMAIN (PHOBIUS); IPR002634 (SUPERFAMILY)
1398	486310_length_1551_cvg_22.0_tip_1_3	221	serine proteinase stubble-like	513	2.38E-69	56.40%	0.099		IPR001314 (PRINTS); IPR001254 (SMART); G3DSA:2.40.10.10 (GENE3D); G3DSA:2.40.10.10 (GENE3D); IPR001254 (PFAM); PTHR24268 (PANTHER); IPR001254 (PROSITE_PROFILES); IPR009003 (SUPERFAMILY)
1410	486648_length_1564_cvg_38.0_tip_1_1	248	zinc finger protein 569-like	513	1.85E-25	47.90%	0.181		IPR015880 (SMART); IPR013087 (G3DSA:3.30.160.GENE3D); PF13912 (PFAM); PF13894 (PFAM); PF13465 (PFAM); IPR013087 (G3DSA:3.30.160.GENE3D); IPR013087 (G3DSA:3.30.160.GENE3D); PTHR24409 (PANTHER); PTHR24409:SF13 (PANTHER); IPR007087 (PROSITE_PATTERNS); IPR007087 (PROSITE_PATTERNS); IPR007087 (PROSITE_PATTERNS); IPR007087 (PROSITE_PATTERNS); IPR007087 (PROSITE_PATTERNS); IPR007087 (PROSITE_PROFILES); IPR007087 (PROSITE_PROFILES); IPR007087 (PROSITE_PROFILES); IPR007087 (PROSITE_PROFILES); IPR007087 (PROSITE_PROFILES); SSF57667 (SUPERFAMILY); SSF57667 (SUPERFAMILY); SSF57667 (SUPERFAMILY)
1392	486106_length_1542_cvg_6.8_tip_1_4	59	folate receptor alpha-like	514	3.66E-80	68.10%	0.299		IPR018143 (PFAM); PTHR10517:SF3 (PANTHER); IPR004269 (PANTHER); NON_CYTOPLASMIC_DOMAIN (PHOBIUS); CYTOPLASMIC_DOMAIN (PHOBIUS); NON_CYTOPLASMIC_DOMAIN (PHOBIUS); CYTOPLASMIC_DOMAIN (PHOBIUS); TRANSMEMBRANE (PHOBIUS); TRANSMEMBRANE (PHOBIUS); TRANSMEMBRANE (PHOBIUS); NON_CYTOPLASMIC_DOMAIN (PHOBIUS); TRANSMEMBRANE (PHOBIUS); TMhelix (TMHMM); TMhelix (TMHMM); TMhelix (TMHMM); TMhelix (TMHMM)
1393	486138_length_1543_cvg_37.2_tip_1_2	302	proclotting enzyme	514	8.60E-128	65.80%	0.111		IPR001314 (PRINTS); IPR006604 (SMART); IPR001254 (SMART); G3DSA:2.40.10.10 (GENE3D); IPR001254 (PFAM); G3DSA:2.40.10.10 (GENE3D); PTHR24268:SF81 (PANTHER); PTHR24268 (PANTHER); IPR018114 (PROSITE_PATTERNS); IPR018114 (PROSITE_PATTERNS); TRANSMEMBRANE (PHOBIUS); NON_CYTOPLASMIC_DOMAIN (PHOBIUS); CYTOPLASMIC_DOMAIN (PHOBIUS); NON_CYTOPLASMIC_DOMAIN (PHOBIUS); TRANSMEMBRANE (PHOBIUS); IPR001254 (PROSITE_PROFILES); IPR009003 (SUPERFAMILY); TMhelix (TMHMM)
1394	486148_length_1543_cvg_5.2_tip_1_4	47	patj-like protein	514	0	77.20%	0.1		IPR001478 (SMART); IPR001478 (PFAM); IPR001478 (G3DSA:2.30.42.GENE3D); IPR001478 (G3DSA:2.30.42.GENE3D); IPR001478 (G3DSA:2.30.42.GENE3D); PTHR19964:SF34 (PANTHER); PTHR19964 (PANTHER); IPR001478 (PROSITE_PROFILES); IPR001478 (PROSITE_PROFILES); IPR001478 (PROSITE_PROFILES); IPR001478 (SUPERFAMILY); IPR001478 (SUPERFAMILY); IPR001478 (SUPERFAMILY)
1395	486166_length_1545_cvg_11.1_tip_1_2	60	26s protease regulatory subunit 8	515	0	97.10%	0.1		Coil (COILS); IPR003593 (SMART); IPR005937 (TIGRFAM); IPR003959 (PFAM); IPR027417 (G3DSA:3.40.50.GENE3D); G3DSA:1.10.8.60 (GENE3D); PTHR23073 (PANTHER); PTHR23073:SF12 (PANTHER); IPR003960 (PROSITE_PATTERNS); TRANSMEMBRANE (PHOBIUS); CYTOPLASMIC_DOMAIN (PHOBIUS); CYTOPLASMIC_DOMAIN (PHOBIUS); TRANSMEMBRANE (PHOBIUS); NON_CYTOPLASMIC_DOMAIN (PHOBIUS); IPR027417 (SUPERFAMILY)
1396	486286_length_1549_cvg_4.2_tip_1_2	32	nas-15 protein	516	1.21E-46	59.80%	0.101		IPR001506 (PRINTS); IPR006026 (SMART); IPR001506 (PFAM); IPR024079 (G3DSA:3.40.390.GENE3D); PTHR10127 (PANTHER); TRANSMEMBRANE (PHOBIUS); TRANSMEMBRANE (PHOBIUS); NON_CYTOPLASMIC_DOMAIN (PHOBIUS); CYTOPLASMIC_DOMAIN (PHOBIUS); CYTOPLASMIC_DOMAIN (PHOBIUS); SSF55486 (SUPERFAMILY); TMhelix (TMHMM)
1397	486288_length_1550_cvg_6.1_tip_1_5	42	allatostatin neuropeptide precursor	516	2.31E-44	53.60%	0.097		IPR010276 (PFAM); CYTOPLASMIC_DOMAIN (PHOBIUS); CYTOPLASMIC_DOMAIN (PHOBIUS); TRANSMEMBRANE (PHOBIUS); TRANSMEMBRANE (PHOBIUS); NON_CYTOPLASMIC_DOMAIN (PHOBIUS)

1399	486358_length_1553_cvg_2.4_tip_1_1	29	e3 ubiquitin-protein ligase mib2	518	0	70.00%	0.424	Y	Coil (COILS); IPR001841 (SMART); IPR002110 (SMART); IPR020683 (G3DSA:1.25.40.GENE3D); IPR020683 (PFAM); IPR013083 (G3DSA:3.30.40.GENE3D); PF13920 (PFAM); IPR020683 (G3DSA:1.25.40.GENE3D); IPR013083 (G3DSA:3.30.40.GENE3D); IPR002110 (PFAM); PTHR24202:SF4 (PANTHER); PTHR24202 (PANTHER); CYTOPLASMIC_DOMAIN (PHOBIUS); TRANSMEMBRANE (PHOBIUS); NON_CYTOPLASMIC_DOMAIN (PHOBIUS); IPR002110 (PROSITE_PROFILES); IPR001841 (PROSITE_PROFILES); IPR002110 (PROSITE_PROFILES); IPR002110 (PROSITE_PROFILES); IPR002110 (PROSITE_PROFILES); IPR020683 (PROSITE_PROFILES); IPR001841 (PROSITE_PROFILES); IPR020683 (SUPERFAMILY)
1400	486420_length_1555_cvg_2.0_tip_1_4	27	ly6 plaur domain-containing protein 6b-like isoform x1	518	2.64E-38	74.90%	0.112		G3DSA:2.10.60.10 (GENE3D); PTHR31171 (PANTHER); CYTOPLASMIC_DOMAIN (PHOBIUS); NON_CYTOPLASMIC_DOMAIN (PHOBIUS); TRANSMEMBRANE (PHOBIUS); SSF57302 (SUPERFAMILY)
1421	487016_length_1581_cvg_66.7_tip_1_2	1169	chitinase domain-containing protein 1 isoform x1	518	2.97E-149	70.30%	0.13		IPR011583 (SMART); IPR013781 (G3DSA:3.20.20.GENE3D); IPR001223 (PFAM); PTHR11177:SF135 (PANTHER); PTHR11177 (PANTHER); CYTOPLASMIC_DOMAIN (PHOBIUS); NON_CYTOPLASMIC_DOMAIN (PHOBIUS); TRANSMEMBRANE (PHOBIUS); IPR017853 (SUPERFAMILY); TMhelix (TMHMM)
1401	486424_length_1555_cvg_63.6_tip_1_3	1357	ferritin 1-like protein a	519	3.11E-45	64.60%	0.256		IPR008331 (PFAM); IPR012347 (G3DSA:1.20.1260.GENE3D); PTHR11431:SF29 (PANTHER); IPR001519 (PANTHER); TRANSMEMBRANE (PHOBIUS); NON_CYTOPLASMIC_DOMAIN (PHOBIUS); CYTOPLASMIC_DOMAIN (PHOBIUS); IPR009040 (PROSITE_PROFILES); IPR009078 (SUPERFAMILY)
1402	486466_length_1557_cvg_24.2_tip_1_1	175	polycomb protein eed	519	0	86.10%	0.104		IPR001680 (SMART); IPR015943 (G3DSA:2.130.10.GENE3D); IPR001680 (PFAM); PTHR10253 (PANTHER); IPR019775 (PROSITE_PATTERNS); IPR001680 (PROSITE_PROFILES); IPR001680 (PROSITE_PROFILES); IPR017986 (PROSITE_PROFILES); IPR017986 (SUPERFAMILY)
1403	486472_length_1557_cvg_11.1_tip_1_3	75	sh3 domain-containing ring finger protein 3 isoform x1	519	3.94E-68	52.50%	0.116		IPR001452 (SMART); G3DSA:2.30.30.40 (GENE3D); IPR001452 (PFAM); PTHR10661 (PANTHER); PTHR10661:SF7 (PANTHER); TRANSMEMBRANE (PHOBIUS); NON_CYTOPLASMIC_DOMAIN (PHOBIUS); CYTOPLASMIC_DOMAIN (PHOBIUS); IPR001452 (PROSITE_PROFILES); IPR001452 (SUPERFAMILY); TMhelix (TMHMM)
1404	486474_length_1557_cvg_27.4_tip_1_4	147	tropinone reductase 2-like	519	4.42E-134	83.50%	0.168		IPR002198 (PRINTS); IPR002347 (PRINTS); IPR002198 (PFAM); IPR016040 (G3DSA:3.40.50.GENE3D); PTHR24322:SF111 (PANTHER); PTHR24322 (PANTHER); IPR020904 (PROSITE_PATTERNS); TRANSMEMBRANE (PHOBIUS); TRANSMEMBRANE (PHOBIUS); CYTOPLASMIC_DOMAIN (PHOBIUS); NON_CYTOPLASMIC_DOMAIN (PHOBIUS); NON_CYTOPLASMIC_DOMAIN (PHOBIUS); TRANSMEMBRANE (PHOBIUS); CYTOPLASMIC_DOMAIN (PHOBIUS); TRANSMEMBRANE (PHOBIUS); TRANSMEMBRANE (PHOBIUS); TRANSMEMBRANE (PHOBIUS); CYTOPLASMIC_DOMAIN (PHOBIUS); CYTOPLASMIC_DOMAIN (PHOBIUS); NON_CYTOPLASMIC_DOMAIN (PHOBIUS); SSF51735 (SUPERFAMILY); TMhelix (TMHMM); TMhelix (TMHMM); TMhelix (TMHMM); TMhelix (TMHMM); TMhelix (TMHMM)
1405	486484_length_1557_cvg_2.9_tip_1_1	29	dipeptidyl peptidase 1 isoform x1	519	0	71.30%	0.684	Y	IPR000668 (PRINTS); IPR000668 (SMART); IPR014882 (PFAM); IPR000668 (PFAM); IPR014882 (G3DSA:2.40.128.GENE3D); G3DSA:3.90.70.10 (GENE3D); IPR013128 (PANTHER); PTHR12411:SF314 (PANTHER); IPR000169 (PROSITE_PATTERNS); IPR025660 (PROSITE_PATTERNS); IPR025661 (PROSITE_PATTERNS); SignalP-noTM (SIGNALP_EUK); IPR014882 (SUPERFAMILY); SSF54001 (SUPERFAMILY)
1416	486860_length_1575_cvg_49.2_tip_1_5	395	dnaj homolog subfamily c member 9	519	1.54E-85	75.60%	0.113		Coil (COILS); Coil (COILS); IPR001623 (PRINTS); IPR001623 (SMART); IPR001623 (G3DSA:1.10.287.GENE3D); IPR001623 (PFAM); PTHR24078:SF166 (PANTHER); PTHR24078 (PANTHER); IPR018253 (PROSITE_PATTERNS); CYTOPLASMIC_DOMAIN (PHOBIUS); TRANSMEMBRANE (PHOBIUS); CYTOPLASMIC_DOMAIN (PHOBIUS); NON_CYTOPLASMIC_DOMAIN (PHOBIUS); TRANSMEMBRANE (PHOBIUS); IPR001623 (PROSITE_PROFILES); IPR001623 (SUPERFAMILY); TMhelix (TMHMM); TMhelix (TMHMM); TMhelix (TMHMM)
1419	486986_length_1580_cvg_18.7_tip_1_2	159	---NA---	519			0.101		CYTOPLASMIC_DOMAIN (PHOBIUS); TRANSMEMBRANE (PHOBIUS); CYTOPLASMIC_DOMAIN (PHOBIUS); NON_CYTOPLASMIC_DOMAIN (PHOBIUS); TRANSMEMBRANE (PHOBIUS); NON_CYTOPLASMIC_DOMAIN (PHOBIUS); TRANSMEMBRANE (PHOBIUS)
1407	486542_length_1560_cvg_20.6_tip_1_3	143	tl5a_tactr ame: full=techylectin-5a flags: precursor	520	5.15E-54	60.80%	0.106		Coil (COILS); IPR002181 (SMART); IPR002181 (PFAM); IPR014716 (G3DSA:3.90.215.GENE3D); IPR014715 (G3DSA:4.10.530.GENE3D); PTHR19143 (PANTHER); IPR020837 (PROSITE_PATTERNS); IPR002181 (PROSITE_PROFILES); IPR002181 (SUPERFAMILY)

1408	486546_length_1560_cvg_16.8_tip_1_3	115	orcokinin peptides type a-like isoform x2	520	1.53E-53	62.70%	0.284	no IPS match
1424	487174_length_1590_cvg_21.7_tip_1_0	191	adp-dependent glucokinase	520	3.60E-171	73.70%	0.119	IPR007666 (PFAM); IPR029056 (G3DSA:3.40.1190.GENE3D); IPR007666 (PANTHER); PTHR21208:SF1 (PANTHER); CYTOPLASMIC_DOMAIN (PHOBIUS); TRANSMEMBRANE (PHOBIUS); TRANSMEMBRANE (PHOBIUS); CYTOPLASMIC_DOMAIN (PHOBIUS); NON_CYTOPLASMIC_DOMAIN (PHOBIUS); NON_CYTOPLASMIC_DOMAIN (PHOBIUS); TRANSMEMBRANE (PHOBIUS); IPR007666 (PROSITE_PROFILES); IPR029056 (SUPERFAMILY); TMhelix (TMHMM); TMhelix (TMHMM); TMhelix (TMHMM)
1409	486598_length_1562_cvg_15.7_tip_1_4	106	prophenoloxidase activating factor	521	1.15E-128	66.60%	0.109	IPR001314 (PRINTS); IPR001254 (SMART); IPR001254 (PFAM); G3DSA:2.40.10.10 (GENE3D); G3DSA:2.40.10.10 (GENE3D); PTHR24268 (PANTHER); PTHR24268:SF95 (PANTHER); IPR018114 (PROSITE_PATTERNS); IPR001254 (PROSITE_PROFILES); IPR009003 (SUPERFAMILY)
1425	487218_length_1592_cvg_59.4_tip_1_3	659	26s protease regulatory subunit 7	522	0	95.90%	0.153	IPR003593 (SMART); IPR005937 (TIGRFAM); G3DSA:1.10.8.60 (GENE3D); IPR027417 (G3DSA:3.40.50.GENE3D); IPR003959 (PFAM); PTHR23073:SF13 (PANTHER); PTHR23073 (PANTHER); IPR003960 (PROSITE_PATTERNS); CYTOPLASMIC_DOMAIN (PHOBIUS); NON_CYTOPLASMIC_DOMAIN (PHOBIUS); TRANSMEMBRANE (PHOBIUS); IPR027417 (SUPERFAMILY); TMhelix (TMHMM)
1411	486722_length_1568_cvg_3.6_tip_1_1	33	flavin-containing monooxygenase fmo gs-ox-like 4	523	6.99E-92	61.10%	0.103	IPR000960 (PRINTS); IPR020946 (PFAM); G3DSA:3.50.50.60 (GENE3D); G3DSA:3.50.50.60 (GENE3D); PTHR23023:SF4 (PANTHER); PTHR23023 (PANTHER); CYTOPLASMIC_DOMAIN (PHOBIUS); TRANSMEMBRANE (PHOBIUS); NON_CYTOPLASMIC_DOMAIN (PHOBIUS); SSF51905 (SUPERFAMILY); SSF51905 (SUPERFAMILY); TMhelix (TMHMM); TMhelix (TMHMM)
1412	486768_length_1570_cvg_7.4_tip_1_2	61	wd repeat-containing protein partial	523	3.79E-112	94.60%	0.226	IPR001680 (SMART); IPR015943 (G3DSA:2.130.10.GENE3D); IPR001680 (PFAM); PTHR19863 (PANTHER); TRANSMEMBRANE (PHOBIUS); CYTOPLASMIC_DOMAIN (PHOBIUS); NON_CYTOPLASMIC_DOMAIN (PHOBIUS); IPR001680 (PROSITE_PROFILES); IPR017986 (PROSITE_PROFILES); IPR001680 (PROSITE_PROFILES); IPR001680 (PROSITE_PROFILES); IPR017986 (SUPERFAMILY); TMhelix (TMHMM)
1413	486782_length_1571_cvg_30.4_tip_1_2	241	antigen 5 scp domain-containing	523	2.66E-32	51.30%	0.102	IPR002413 (PRINTS); IPR001283 (PRINTS); IPR014044 (SMART); IPR014044 (PFAM); IPR014044 (G3DSA:3.40.33.GENE3D); IPR001283 (PANTHER); NON_CYTOPLASMIC_DOMAIN (PHOBIUS); TRANSMEMBRANE (PHOBIUS); TRANSMEMBRANE (PHOBIUS); CYTOPLASMIC_DOMAIN (PHOBIUS); NON_CYTOPLASMIC_DOMAIN (PHOBIUS); TRANSMEMBRANE (PHOBIUS); CYTOPLASMIC_DOMAIN (PHOBIUS); IPR014044 (SUPERFAMILY); TMhelix (TMHMM); TMhelix (TMHMM); TMhelix (TMHMM)
1414	486790_length_1571_cvg_6.8_tip_1_1	67	ras suppressor protein 1	524	6.80E-156	87.60%	0.099	SM00364 (SMART); IPR003591 (SMART); G3DSA:3.80.10.10 (GENE3D); IPR025875 (PFAM); IPR001611 (PFAM); PTHR23155 (PANTHER); TRANSMEMBRANE (PHOBIUS); CYTOPLASMIC_DOMAIN (PHOBIUS); TRANSMEMBRANE (PHOBIUS); CYTOPLASMIC_DOMAIN (PHOBIUS); NON_CYTOPLASMIC_DOMAIN (PHOBIUS); IPR001611 (PROSITE_PROFILES); IPR001611 (PROSITE_PROFILES); IPR001611 (PROSITE_PROFILES); IPR001611 (PROSITE_PROFILES); IPR001611 (PROSITE_PROFILES); IPR001611 (PROSITE_PROFILES); SSF52058 (SUPERFAMILY); TMhelix (TMHMM); TMhelix (TMHMM)
1415	486830_length_1573_cvg_8.0_tip_1_0	60	zinc finger protein	525	9.51E-42	52.20%	0.1	Coil (COILS); IPR015880 (SMART); PF13465 (PFAM); IPR013087 (G3DSA:3.30.160.GENE3D); IPR013087 (G3DSA:3.30.160.GENE3D); IPR007087 (PFAM); PF13894 (PFAM); IPR013087 (G3DSA:3.30.160.GENE3D); PTHR24409 (PANTHER); PTHR24409:SF14 (PANTHER); IPR007087 (PROSITE_PATTERNS); IPR007087 (PROSITE_PATTERNS); IPR007087 (PROSITE_PATTERNS); IPR007087 (PROSITE_PATTERNS); IPR007087 (PROSITE_PATTERNS); IPR007087 (PROSITE_PATTERNS); IPR007087 (PROSITE_PATTERNS); IPR007087 (PROSITE_PATTERNS); IPR007087 (PROSITE_PROFILES); IPR007087 (PROSITE_PROFILES); IPR007087 (PROSITE_PROFILES); IPR007087 (PROSITE_PROFILES); IPR007087 (PROSITE_PROFILES); IPR007087 (PROSITE_PROFILES); IPR007087 (PROSITE_PROFILES); IPR007087 (PROSITE_PROFILES); SSF57667 (SUPERFAMILY); SSF57667 (SUPERFAMILY); SSF57667 (SUPERFAMILY); SSF57667 (SUPERFAMILY); SSF57667 (SUPERFAMILY)

1418	486924_length_1577_cvg_13.0_tip_1_5	91	serine protease mitochondrial	525	2.06E-130	73.50%	0.125	IPR001940 (PRINTS); IPR001478 (SMART); G3DSA:2.40.10.10 (GENE3D); PF13365 (PFAM); IPR001478 (PFAM); G3DSA:2.40.10.10 (GENE3D); IPR001478 (G3DSA:2.30.42.GENE3D); PTHR22939:SF69 (PANTHER); PTHR22939 (PANTHER); CYTOPLASMIC_DOMAIN (PHOBIUS); NON_CYTOPLASMIC_DOMAIN (PHOBIUS); TRANSMEMBRANE (PHOBIUS); IPR001478 (PROSITE_PROFILES); IPR009003 (SUPERFAMILY); IPR001478 (SUPERFAMILY)
1417	486912_length_1577_cvg_71.0_tip_1_0	659	transcription factor sp5-like	526	9.69E-74	53.80%	0.29	IPR015880 (SMART); IPR013087 (G3DSA:3.30.160.GENE3D); IPR013087 (G3DSA:3.30.160.GENE3D); PF13465 (PFAM); PTHR23223 (PANTHER); PTHR23223:SF15 (PANTHER); IPR007087 (PROSITE_PATTERNS); IPR007087 (PROSITE_PATTERNS); IPR007087 (PROSITE_PATTERNS); IPR007087 (PROSITE_PROFILES); IPR007087 (PROSITE_PROFILES); IPR007087 (PROSITE_PROFILES); SSF57667 (SUPERFAMILY); SSF57667 (SUPERFAMILY)
1420	486992_length_1580_cvg_3.7_tip_1_1	29	dehydrogenase reductase sdr family member 11-like	527	2.70E-102	74.50%	0.13	IPR002198 (PRINTS); IPR002347 (PRINTS); IPR002198 (PFAM); IPR016040 (G3DSA:3.40.50.GENE3D); PTHR24322 (PANTHER); PTHR24322:SF310 (PANTHER); CYTOPLASMIC_DOMAIN (PHOBIUS); TRANSMEMBRANE (PHOBIUS); NON_CYTOPLASMIC_DOMAIN (PHOBIUS); TRANSMEMBRANE (PHOBIUS); TRANSMEMBRANE (PHOBIUS); TRANSMEMBRANE (PHOBIUS); CYTOPLASMIC_DOMAIN (PHOBIUS); CYTOPLASMIC_DOMAIN (PHOBIUS); NON_CYTOPLASMIC_DOMAIN (PHOBIUS); SSF51735 (SUPERFAMILY); TMhelix (TMHMM); TMhelix (TMHMM); TMhelix (TMHMM); TMhelix (TMHMM)
1432	487456_length_1607_cvg_63.0_tip_1_2	855	serine protease snake	527	3.88E-75	54.10%	0.446 Y	IPR001314 (PRINTS); IPR001254 (SMART); IPR006604 (SMART); IPR001254 (PFAM); G3DSA:2.40.10.10 (GENE3D); G3DSA:2.40.10.10 (GENE3D); G3DSA:2.40.10.10 (GENE3D); PTHR24260:SF46 (PANTHER); PTHR24260 (PANTHER); IPR018114 (PROSITE_PATTERNS); IPR018114 (PROSITE_PATTERNS); IPR001254 (PROSITE_PROFILES); IPR009003 (SUPERFAMILY)
1422	487042_length_1583_cvg_20.6_tip_1_4	209	beta- -galactosyltransferase 7	528	1.03E-126	72.30%	0.526 Y	IPR003859 (PRINTS); IPR027791 (PFAM); IPR029044 (G3DSA:3.90.550.GENE3D); IPR027995 (PFAM); PTHR19300:SF1 (PANTHER); IPR003859 (PANTHER); TRANSMEMBRANE (PHOBIUS); CYTOPLASMIC_DOMAIN (PHOBIUS); TRANSMEMBRANE (PHOBIUS); TRANSMEMBRANE (PHOBIUS); CYTOPLASMIC_DOMAIN (PHOBIUS); TRANSMEMBRANE (PHOBIUS); NON_CYTOPLASMIC_DOMAIN (PHOBIUS); TRANSMEMBRANE (PHOBIUS); CYTOPLASMIC_DOMAIN (PHOBIUS); NON_CYTOPLASMIC_DOMAIN (PHOBIUS); NON_CYTOPLASMIC_DOMAIN (PHOBIUS); TRANSMEMBRANE (PHOBIUS); CYTOPLASMIC_DOMAIN (PHOBIUS); SignalP-noTM (SIGNALP_EUK); IPR029044 (SUPERFAMILY); TMhelix (TMHMM); TMhelix (TMHMM); TMhelix (TMHMM); TMhelix (TMHMM); TMhelix (TMHMM)
1423	487134_length_1588_cvg_8.5_tip_1_1	75	neuroparsin-like protein	529	2.13E-18	61.20%	0.104	IPR010850 (PFAM); IPR011390 (PANTHER); TRANSMEMBRANE (PHOBIUS); TRANSMEMBRANE (PHOBIUS); TRANSMEMBRANE (PHOBIUS); CYTOPLASMIC_DOMAIN (PHOBIUS); TRANSMEMBRANE (PHOBIUS); CYTOPLASMIC_DOMAIN (PHOBIUS); NON_CYTOPLASMIC_DOMAIN (PHOBIUS); NON_CYTOPLASMIC_DOMAIN (PHOBIUS); CYTOPLASMIC_DOMAIN (PHOBIUS); TRANSMEMBRANE (PHOBIUS); TRANSMEMBRANE (PHOBIUS); NON_CYTOPLASMIC_DOMAIN (PHOBIUS); CYTOPLASMIC_DOMAIN (PHOBIUS); TRANSMEMBRANE (PHOBIUS); TRANSMEMBRANE (PHOBIUS); NON_CYTOPLASMIC_DOMAIN (PHOBIUS); CYTOPLASMIC_DOMAIN (PHOBIUS); IPR010850 (PRODOM); IPR009030 (SUPERFAMILY); TMhelix (TMHMM); TMhelix (TMHMM); TMhelix (TMHMM); TMhelix (TMHMM); TMhelix (TMHMM); TMhelix (TMHMM)
1426	487238_length_1593_cvg_18.7_tip_1_5	154	arylsulfatase a-like	531	0	68.20%	0.440 Y	G3DSA:3.30.1120.10 (GENE3D); IPR017849 (G3DSA:3.40.720.GENE3D); IPR000917 (PFAM); PF14707 (PFAM); PTHR10342 (PANTHER); PTHR10342:SF22 (PANTHER); IPR024607 (PROSITE_PATTERNS); IPR024607 (PROSITE_PATTERNS); SIGNAL_PEPTIDE_C_REGION (PHOBIUS); SIGNAL_PEPTIDE_H_REGION (PHOBIUS); SIGNAL_PEPTIDE (PHOBIUS); SIGNAL_PEPTIDE_N_REGION (PHOBIUS); NON_CYTOPLASMIC_DOMAIN (PHOBIUS); IPR017850 (SUPERFAMILY); TMhelix (TMHMM)
1427	487240_length_1593_cvg_66.8_tip_1_3	2640	twitchin isoform x1	531	1.58E-137	70.40%	0.1	IPR003598 (SMART); IPR003599 (SMART); IPR013783 (G3DSA:2.60.40.GENE3D); IPR013783 (G3DSA:2.60.40.GENE3D); IPR013783 (G3DSA:2.60.40.GENE3D); IPR013098 (PFAM); PTHR19897 (PANTHER); CYTOPLASMIC_DOMAIN (PHOBIUS); TRANSMEMBRANE (PHOBIUS); NON_CYTOPLASMIC_DOMAIN (PHOBIUS); IPR007110 (PROSITE_PROFILES); IPR007110 (PROSITE_PROFILES); IPR007110 (PROSITE_PROFILES); SSF48726 (SUPERFAMILY); SSF48726 (SUPERFAMILY); SSF48726 (SUPERFAMILY)

1428	487328_length_1598_cvg_13.6_tip_1_4	133	pdz domain-containing protein 11	533	4.33E-50	76.90%	0.105		IPR001478 (SMART); IPR001478 (G3DSA:2.30.42.GENE3D); IPR001478 (PFAM); PTHR14063 (PANTHER); IPR001478 (PROSITE_PROFILES); IPR001478 (SUPERFAMILY)
1438	487638_length_1617_cvg_9.7_tip_1_4	72	serine proteinase inhibitor b3	533	5.43E-73	53.90%	0.186		IPR023796 (SMART); IPR023796 (PFAM); G3DSA:3.30.497.10 (GENE3D); G3DSA:2.30.39.10 (GENE3D); IPR000215 (PANTHER); IPR023795 (PROSITE_PATTERNS); TRANSMEMBRANE (PHOBIUS); NON_CYTOPLASMIC_DOMAIN (PHOBIUS); CYTOPLASMIC_DOMAIN (PHOBIUS); IPR023796 (SUPERFAMILY); TMhelix (TMHMM)
1429	487412_length_1604_cvg_15.0_tip_1_4	105	gamma-aminobutyric acid type b receptor subunit 1 isoform x1	535	0	75.80%	0.097		Coil (COILS); PR01176 (PRINTS); IPR002456 (PRINTS); IPR017978 (PFAM); PTHR10519:SF3 (PANTHER); IPR002455 (PANTHER); TRANSMEMBRANE (PHOBIUS); NON_CYTOPLASMIC_DOMAIN (PHOBIUS); CYTOPLASMIC_DOMAIN (PHOBIUS); NON_CYTOPLASMIC_DOMAIN (PHOBIUS); CYTOPLASMIC_DOMAIN (PHOBIUS); NON_CYTOPLASMIC_DOMAIN (PHOBIUS); NON_CYTOPLASMIC_DOMAIN (PHOBIUS); TRANSMEMBRANE (PHOBIUS); TRANSMEMBRANE (PHOBIUS); TRANSMEMBRANE (PHOBIUS); CYTOPLASMIC_DOMAIN (PHOBIUS); TRANSMEMBRANE (PHOBIUS); TRANSMEMBRANE (PHOBIUS); CYTOPLASMIC_DOMAIN (PHOBIUS); TRANSMEMBRANE (PHOBIUS); IPR017978 (PROSITE_PROFILES); TMhelix (TMHMM); TMhelix (TMHMM); TMhelix (TMHMM); TMhelix (TMHMM); TMhelix (TMHMM); TMhelix (TMHMM); TMhelix (TMHMM)
1430	487450_length_1606_cvg_48.3_tip_1_4	342	transmembrane protease serine 6	535	1.55E-49	53.00%	0.912	Y	IPR001314 (PRINTS); IPR001254 (SMART); IPR002172 (SMART); IPR001254 (PFAM); IPR002172 (PFAM); G3DSA:2.40.10.10 (GENE3D); G3DSA:2.40.10.10 (GENE3D); G3DSA:2.40.10.10 (GENE3D); IPR002172 (G3DSA:4.10.400.GENE3D); PTHR24256 (PANTHER); IPR018114 (PROSITE_PATTERNS); IPR018114 (PROSITE_PATTERNS); IPR023415 (PROSITE_PATTERNS); SIGNAL_PEPTIDE (PHOBIUS); SIGNAL_PEPTIDE_H_REGION (PHOBIUS); NON_CYTOPLASMIC_DOMAIN (PHOBIUS); SIGNAL_PEPTIDE_C_REGION (PHOBIUS); SIGNAL_PEPTIDE_N_REGION (PHOBIUS); CYTOPLASMIC_DOMAIN (PHOBIUS); TRANSMEMBRANE (PHOBIUS); IPR001254 (PROSITE_PROFILES); IPR002172 (PROSITE_PROFILES); SignalP-noTM (SIGNALP_EUK); IPR002172 (SUPERFAMILY); IPR009003 (SUPERFAMILY); TMhelix (TMHMM); TMhelix (TMHMM); TMhelix (TMHMM)
1454	487998_length_1634_cvg_11.8_tip_1_4	117	acetylcholine receptor subunit alpha-type acr-16	535	6.06E-112	59.90%	0.171		IPR006201 (PRINTS); IPR006202 (PFAM); IPR006029 (PFAM); IPR027361 (G3DSA:1.20.120.GENE3D); IPR006202 (G3DSA:2.70.170.GENE3D); PTHR18945:SF490 (PANTHER); IPR006201 (PANTHER); NON_CYTOPLASMIC_DOMAIN (PHOBIUS); TRANSMEMBRANE (PHOBIUS); TRANSMEMBRANE (PHOBIUS); TRANSMEMBRANE (PHOBIUS); TRANSMEMBRANE (PHOBIUS); NON_CYTOPLASMIC_DOMAIN (PHOBIUS); CYTOPLASMIC_DOMAIN (PHOBIUS); CYTOPLASMIC_DOMAIN (PHOBIUS); CYTOPLASMIC_DOMAIN (PHOBIUS); IPR006029 (SUPERFAMILY); IPR006202 (SUPERFAMILY); TMhelix (TMHMM); TMhelix (TMHMM); TMhelix (TMHMM); TMhelix (TMHMM)
1431	487454_length_1607_cvg_12.5_tip_1_3	97	wd40 repeat-containing protein smu1	536	0	93.90%	0.105		IPR020472 (PRINTS); IPR006594 (SMART); IPR006595 (SMART); IPR001680 (SMART); IPR015943 (G3DSA:2.130.10.GENE3D); IPR001680 (PFAM); PTHR22848 (PANTHER); IPR019775 (PROSITE_PATTERNS); IPR019775 (PROSITE_PATTERNS); IPR017986 (PROSITE_PROFILES); IPR006594 (PROSITE_PROFILES); IPR001680 (PROSITE_PROFILES); IPR001680 (PROSITE_PROFILES); IPR006595 (PROSITE_PROFILES); IPR001680 (PROSITE_PROFILES); IPR001680 (PROSITE_PROFILES); IPR001680 (PROSITE_PROFILES); IPR017986 (SUPERFAMILY)
1433	487470_length_1607_cvg_4.3_tip_1_4	42	serine threonine-protein kinase nek7	536	2.76E-178	91.00%	0.145		IPR002290 (SMART); IPR000719 (PFAM); G3DSA:1.10.510.10 (GENE3D); G3DSA:3.30.200.20 (GENE3D); PTHR24362 (PANTHER); PTHR24362:SF228 (PANTHER); IPR008271 (PROSITE_PATTERNS); IPR017441 (PROSITE_PATTERNS); NON_CYTOPLASMIC_DOMAIN (PHOBIUS); TRANSMEMBRANE (PHOBIUS); TRANSMEMBRANE (PHOBIUS); CYTOPLASMIC_DOMAIN (PHOBIUS); NON_CYTOPLASMIC_DOMAIN (PHOBIUS); IPR000719 (PROSITE_PROFILES); IPR011009 (SUPERFAMILY); TMhelix (TMHMM); TMhelix (TMHMM)
1434	487506_length_1610_cvg_8.7_tip_1_5	76	26s protease regulatory subunit 6b	536	0	96.20%	0.098		Coil (COILS); IPR003593 (SMART); IPR027417 (G3DSA:3.40.50.GENE3D); G3DSA:1.10.8.60 (GENE3D); IPR005937 (TIGRFAM); IPR003959 (PFAM); PTHR23073:SF8 (PANTHER); PTHR23073 (PANTHER); IPR003960 (PROSITE_PATTERNS); IPR027417 (SUPERFAMILY)

1440	487690_length_1619_cvg_35.6_tip_1_1	292	hydroxysteroid (17-beta)	536	1.15E-78	62.00%	0.107		IPR002198 (PRINTS); IPR002347 (PRINTS); IPR002198 (PFAM); IPR016040 (G3DSA:3.40.50.GENE3D); PTHR24316:SF68 (PANTHER); PTHR24316 (PANTHER); CYTOPLASMIC_DOMAIN (PHOBIUS); CYTOPLASMIC_DOMAIN (PHOBIUS); TRANSMEMBRANE (PHOBIUS); TRANSMEMBRANE (PHOBIUS); TRANSMEMBRANE (PHOBIUS); NON_CYTOPLASMIC_DOMAIN (PHOBIUS); NON_CYTOPLASMIC_DOMAIN (PHOBIUS); TRANSMEMBRANE (PHOBIUS); NON_CYTOPLASMIC_DOMAIN (PHOBIUS); SSF51735 (SUPERFAMILY); TMhelix (TMHMM); TMhelix (TMHMM); TMhelix (TMHMM); TMhelix (TMHMM)
1444	487764_length_1622_cvg_10.8_tip_1_0	90	neuroendocrine partial	536	1.88E-108	62.30%	0.13		IPR002884 (PFAM); IPR000209 (G3DSA:3.40.50.GENE3D); IPR008979 (G3DSA:2.60.120.GENE3D); IPR000209 (PFAM); PTHR10795:SF340 (PANTHER); IPR015500 (PANTHER); IPR023828 (PROSITE_PATTERNS); IPR000209 (SUPERFAMILY); IPR008979 (SUPERFAMILY)
1435	487590_length_1614_cvg_52.5_tip_1_5	357	cartilage intermediate layer protein 2-like	538	8.32E-18	43.90%	0.109		IPR025155 (PFAM); NON_CYTOPLASMIC_DOMAIN (PHOBIUS); CYTOPLASMIC_DOMAIN (PHOBIUS); TRANSMEMBRANE (PHOBIUS); TMhelix (TMHMM)
1437	487618_length_1616_cvg_5.0_tip_1_5	41	serine threonine-protein phosphatase pp1-beta catalytic subunit	538	0	97.30%	0.115		IPR006186 (PRINTS); IPR006186 (SMART); IPR004843 (PFAM); IPR029052 (G3DSA:3.60.21.GENE3D); PTHR11668:SF204 (PANTHER); PTHR11668 (PANTHER); IPR006186 (PROSITE_PATTERNS); TRANSMEMBRANE (PHOBIUS); NON_CYTOPLASMIC_DOMAIN (PHOBIUS); NON_CYTOPLASMIC_DOMAIN (PHOBIUS); CYTOPLASMIC_DOMAIN (PHOBIUS); TRANSMEMBRANE (PHOBIUS); IPR029052 (SUPERFAMILY)
1436	487608_length_1615_cvg_16.3_tip_1_3	115	flavin-containing monooxygenase fmo gs-ox4-like isoform x1	539	8.10E-134	62.90%	0.308		IPR000960 (PRINTS); IPR020946 (PFAM); G3DSA:3.50.50.60 (GENE3D); G3DSA:3.50.50.60 (GENE3D); PTHR23023:SF4 (PANTHER); PTHR23023 (PANTHER); SSF51905 (SUPERFAMILY); SSF51905 (SUPERFAMILY); TMhelix (TMHMM)
1439	487678_length_1619_cvg_73.4_tip_0_5	137565	venom serine carboxypeptidase-like	539	1.89E-59	58.30%	0.102		IPR001563 (PFAM); IPR029058 (G3DSA:3.40.50.GENE3D); IPR001563 (PANTHER); IPR018202 (PROSITE_PATTERNS); IPR029058 (SUPERFAMILY)
1441	487710_length_1620_cvg_11.6_tip_1_5	85	carbohydrate sulfotransferase 11-like	540	1.54E-66	55.10%	0.642	Y	IPR005331 (PFAM); IPR018011 (PANTHER); SIGNAL_PEPTIDE_H_REGION (PHOBIUS); SIGNAL_PEPTIDE_N_REGION (PHOBIUS); NON_CYTOPLASMIC_DOMAIN (PHOBIUS); SIGNAL_PEPTIDE_C_REGION (PHOBIUS); SIGNAL_PEPTIDE (PHOBIUS); SignalP-noTM (SIGNALP_EUK)
1442	487734_length_1621_cvg_5.2_tip_1_5	48	aldehyde dimeric nadp-preferring isoform x4	540	0	74.30%	0.108		IPR012394 (PIRSF); IPR015590 (PFAM); IPR016163 (G3DSA:3.40.309.GENE3D); IPR016162 (G3DSA:3.40.605.GENE3D); PTHR11699 (PANTHER); PTHR11699:SF15 (PANTHER); IPR029510 (PROSITE_PATTERNS); CYTOPLASMIC_DOMAIN (PHOBIUS); NON_CYTOPLASMIC_DOMAIN (PHOBIUS); TRANSMEMBRANE (PHOBIUS); IPR016161 (SUPERFAMILY); TMhelix (TMHMM)
1443	487762_length_1622_cvg_32.7_tip_1_2	265	notchless protein homolog 1	540	0	83.20%	0.115		IPR001632 (PRINTS); IPR020472 (PRINTS); IPR001680 (SMART); IPR015943 (G3DSA:2.130.10.GENE3D); IPR001680 (PFAM); IPR012972 (PFAM); PTHR19848:SF0 (PANTHER); PTHR19848 (PANTHER); IPR019775 (PROSITE_PATTERNS); IPR019775 (PROSITE_PATTERNS); IPR019775 (PROSITE_PATTERNS); IPR019775 (PROSITE_PATTERNS); IPR001680 (PROSITE_PROFILES); IPR017986 (PROSITE_PROFILES); IPR001680 (PROSITE_PROFILES); IPR001680 (PROSITE_PROFILES); IPR001680 (PROSITE_PROFILES); IPR001680 (PROSITE_PROFILES); IPR001680 (PROSITE_PROFILES); IPR001680 (PROSITE_PROFILES); IPR011047 (SUPERFAMILY)
1464	488288_length_1650_cvg_26.2_tip_1_1	267	dermatopontin 2	540	2.97E-37	58.70%	0.574	Y	PF14704 (PFAM); IPR026645 (PANTHER); SIGNAL_PEPTIDE_C_REGION (PHOBIUS); SIGNAL_PEPTIDE_N_REGION (PHOBIUS); SIGNAL_PEPTIDE (PHOBIUS); NON_CYTOPLASMIC_DOMAIN (PHOBIUS); SIGNAL_PEPTIDE_H_REGION (PHOBIUS); SignalP-TM (SIGNALP_GRAM_NEGATIVE); SignalP-TM (SIGNALP_EUK); SignalP-TM (SIGNALP_GRAM_POSITIVE); TMhelix (TMHMM)
1445	487780_length_1623_cvg_44.9_tip_1_5	293	ras-related protein rab-10	541	1.76E-116	92.90%	0.182		IPR001806 (PRINTS); IPR024156 (SMART); IPR003579 (SMART); IPR002041 (SMART); IPR020849 (SMART); IPR003578 (SMART); IPR027417 (G3DSA:3.40.50.GENE3D); IPR005225 (TIGRFAM); IPR001806 (PFAM); PTHR24073:SF456 (PANTHER); PTHR24073 (PANTHER); NON_CYTOPLASMIC_DOMAIN (PHOBIUS); TRANSMEMBRANE (PHOBIUS); CYTOPLASMIC_DOMAIN (PHOBIUS); PS51419 (PROSITE_PROFILES); IPR027417 (SUPERFAMILY)

1446	487788_length_1623_cvg_10.8_tip_1_2	84	gastrula zinc finger isoform x1	541	3.49E-52	52.80%	0.1		IPR015880 (SMART); IPR007087 (PFAM); IPR013087 (G3DSA:3.30.160.GENE3D); PF13894 (PFAM); IPR013087 (G3DSA:3.30.160.GENE3D); IPR013087 (G3DSA:3.30.160.GENE3D); IPR013087 (G3DSA:3.30.160.GENE3D); PF13465 (PFAM); PF13912 (PFAM); PTHR24409:SF14 (PANTHER); PTHR24409 (PANTHER); IPR007087 (PROSITE_PATTERNS); IPR007087 (PROSITE_PATTERNS); IPR007087 (PROSITE_PATTERNS); IPR007087 (PROSITE_PATTERNS); IPR007087 (PROSITE_PATTERNS); IPR007087 (PROSITE_PATTERNS); IPR007087 (PROSITE_PATTERNS); IPR007087 (PROSITE_PATTERNS); TRANSMEMBRANE (PHOBIUS); CYTOPLASMIC_DOMAIN (PHOBIUS); NON_CYTOPLASMIC_DOMAIN (PHOBIUS); CYTOPLASMIC_DOMAIN (PHOBIUS); TRANSMEMBRANE (PHOBIUS); TRANSMEMBRANE (PHOBIUS); NON_CYTOPLASMIC_DOMAIN (PHOBIUS); IPR007087 (PROSITE_PROFILES); IPR007087 (PROSITE_PROFILES); IPR007087 (PROSITE_PROFILES); IPR007087 (PROSITE_PROFILES); IPR007087 (PROSITE_PROFILES); IPR007087 (PROSITE_PROFILES); IPR007087 (PROSITE_PROFILES); IPR007087 (PROSITE_PROFILES); IPR007087 (PROSITE_PROFILES); SSF57667 (SUPERFAMILY); SSF57667 (SUPERFAMILY); SSF57667 (SUPERFAMILY); SSF57667 (SUPERFAMILY); SSF57667 (SUPERFAMILY); TMhelix (TMHMM)
1447	487808_length_1624_cvg_18.4_tip_1_5	117	adp-ribosylation factor-like protein 3	541	5.38E-86	65.20%	0.247		IPR006689 (PRINTS); IPR024156 (SMART); IPR006687 (SMART); IPR027417 (G3DSA:3.40.50.GENE3D); IPR006689 (PFAM); PTHR11711 (PANTHER); PTHR11711:SF15 (PANTHER); CYTOPLASMIC_DOMAIN (PHOBIUS); NON_CYTOPLASMIC_DOMAIN (PHOBIUS); TRANSMEMBRANE (PHOBIUS); TRANSMEMBRANE (PHOBIUS); TRANSMEMBRANE (PHOBIUS); NON_CYTOPLASMIC_DOMAIN (PHOBIUS); CYTOPLASMIC_DOMAIN (PHOBIUS); IPR024156 (PROSITE_PROFILES); IPR027417 (SUPERFAMILY); TMhelix (TMHMM); TMhelix (TMHMM); TMhelix (TMHMM); TMhelix (TMHMM)
1448	487848_length_1626_cvg_5.1_tip_1_4	44	hemicentin-2 isoform x3	542	6.02E-91	67.40%	0.263		IPR003599 (SMART); IPR003598 (SMART); IPR013098 (PFAM); IPR013783 (G3DSA:2.60.40.GENE3D); IPR013783 (G3DSA:2.60.40.GENE3D); PTHR23279 (PANTHER); TRANSMEMBRANE (PHOBIUS); NON_CYTOPLASMIC_DOMAIN (PHOBIUS); CYTOPLASMIC_DOMAIN (PHOBIUS); TRANSMEMBRANE (PHOBIUS); NON_CYTOPLASMIC_DOMAIN (PHOBIUS); TRANSMEMBRANE (PHOBIUS); CYTOPLASMIC_DOMAIN (PHOBIUS); CYTOPLASMIC_DOMAIN (PHOBIUS); TRANSMEMBRANE (PHOBIUS); TRANSMEMBRANE (PHOBIUS); NON_CYTOPLASMIC_DOMAIN (PHOBIUS); IPR007110 (PROSITE_PROFILES); IPR007110 (PROSITE_PROFILES); SSF48726 (SUPERFAMILY); SSF48726 (SUPERFAMILY); TMhelix (TMHMM); TMhelix (TMHMM); TMhelix (TMHMM); TMhelix (TMHMM)
1449	487850_length_1626_cvg_37.9_tip_1_1	301	scp-like extracellular domain containing protein 1	542	3.59E-43	53.20%	0.867	Y	IPR001283 (PRINTS); IPR002413 (PRINTS); IPR002172 (SMART); IPR014044 (SMART); IPR002172 (G3DSA:4.10.400.GENE3D); IPR002172 (PFAM); IPR014044 (G3DSA:3.40.33.GENE3D); IPR014044 (PFAM); IPR001283 (PANTHER); IPR023415 (PROSITE_PATTERNS); NON_CYTOPLASMIC_DOMAIN (PHOBIUS); SIGNAL_PEPTIDE_N_REGION (PHOBIUS); SIGNAL_PEPTIDE_H_REGION (PHOBIUS); SIGNAL_PEPTIDE_C_REGION (PHOBIUS); SIGNAL_PEPTIDE (PHOBIUS); IPR002172 (PROSITE_PROFILES); SignalP-TM (SIGNALP_GRAM_POSITIVE); SignalP-noTM (SIGNALP_EUK); IPR014044 (SUPERFAMILY); IPR002172 (SUPERFAMILY)
1451	487890_length_1628_cvg_18.2_tip_1_2	160	inactive dipeptidyl peptidase 10	542	2.68E-99	62.10%	0.101		IPR029058 (G3DSA:3.40.50.GENE3D); IPR002469 (PFAM); IPR002469 (G3DSA:2.140.10.GENE3D); IPR001375 (PFAM); PTHR11731 (PANTHER); PTHR11731:SF93 (PANTHER); NON_CYTOPLASMIC_DOMAIN (PHOBIUS); CYTOPLASMIC_DOMAIN (PHOBIUS); TRANSMEMBRANE (PHOBIUS); SSF82171 (SUPERFAMILY); IPR029058 (SUPERFAMILY)
1450	487888_length_1628_cvg_37.6_tip_1_0	408	ras-related protein rab-37 isoform x2	543	3.43E-111	87.10%	0.146		IPR001806 (PRINTS); IPR003578 (SMART); IPR024156 (SMART); IPR002041 (SMART); IPR003579 (SMART); IPR020849 (SMART); IPR027417 (G3DSA:3.40.50.GENE3D); IPR001806 (PFAM); IPR005225 (TIGRFAM); PTHR24073 (PANTHER); PTHR24073:SF342 (PANTHER); CYTOPLASMIC_DOMAIN (PHOBIUS); TRANSMEMBRANE (PHOBIUS); NON_CYTOPLASMIC_DOMAIN (PHOBIUS); PS51419 (PROSITE_PROFILES); IPR027417 (SUPERFAMILY); TMhelix (TMHMM)
1452	487914_length_1629_cvg_21.3_tip_1_1	237	dehydrogenase reductase sdr family member 4-like isoform x1	543	1.11E-104	74.60%	0.114		IPR002347 (PRINTS); IPR002198 (PRINTS); PF13561 (PFAM); IPR016040 (G3DSA:3.40.50.GENE3D); PTHR24322:SF239 (PANTHER); PTHR24322 (PANTHER); IPR020904 (PROSITE_PATTERNS); CYTOPLASMIC_DOMAIN (PHOBIUS); NON_CYTOPLASMIC_DOMAIN (PHOBIUS); TRANSMEMBRANE (PHOBIUS); TRANSMEMBRANE (PHOBIUS); NON_CYTOPLASMIC_DOMAIN (PHOBIUS); SSF51735 (SUPERFAMILY); TMhelix (TMHMM); TMhelix (TMHMM)

1453	487928_length_1630_cvg_3.4_tip_1_4	27	titin- partial	543	1.92E-22	52.00%	0.111		NON_CYTOPLASMIC_DOMAIN (PHOBIUS); TRANSMEMBRANE (PHOBIUS); CYTOPLASMIC_DOMAIN (PHOBIUS); NON_CYTOPLASMIC_DOMAIN (PHOBIUS); TRANSMEMBRANE (PHOBIUS); TMhelix (TMHMM); TMhelix (TMHMM); TMhelix (TMHMM)
1466	488386_length_1656_cvg_7.5_tip_0_1	78	transducin beta-like protein 2	544	1.69E-136	73.50%	0.105		Coil (COILS); IPR001680 (SMART); IPR015943 (G3DSA:2.130.10.GENE3D); IPR001680 (PFAM); PTHR22847:SF380 (PANTHER); PTHR22847 (PANTHER); IPR019775 (PROSITE_PATTERNS); CYTOPLASMIC_DOMAIN (PHOBIUS); NON_CYTOPLASMIC_DOMAIN (PHOBIUS); TRANSMEMBRANE (PHOBIUS); IPR001680 (PROSITE_PROFILES); IPR017986 (PROSITE_PROFILES); IPR001680 (PROSITE_PROFILES); IPR017986 (SUPERFAMILY)
1455	488042_length_1637_cvg_3.4_tip_1_2	28	aspartate--trna mitochondrial	545	0	72.50%	0.102		IPR002312 (PRINTS); G3DSA:3.30.930.10 (GENE3D); IPR004364 (PFAM); IPR004115 (G3DSA:3.30.1360.GENE3D); IPR004524 (TIGRFAM); IPR029351 (PFAM); IPR004524 (PTHR22594:PANTHER); IPR018150 (PANTHER); IPR006195 (PROSITE_PROFILES); SSF55681 (SUPERFAMILY); IPR004115 (SUPERFAMILY)
1465	488360_length_1655_cvg_57.1_tip_1_0	535	phosphoglycerate kinase	546	0	87.40%	0.142		IPR001576 (PRINTS); IPR015824 (G3DSA:3.40.50.GENE3D); IPR015901 (G3DSA:3.40.50.GENE3D); IPR001576 (PFAM); IPR001576 (PANTHER); PTHR11406:SF6 (PANTHER); IPR015911 (PROSITE_PATTERNS); NON_CYTOPLASMIC_DOMAIN (PHOBIUS); NON_CYTOPLASMIC_DOMAIN (PHOBIUS); TRANSMEMBRANE (PHOBIUS); CYTOPLASMIC_DOMAIN (PHOBIUS); TRANSMEMBRANE (PHOBIUS); IPR001576 (HAMAP); IPR001576 (SUPERFAMILY)
1456	488116_length_1641_cvg_12.1_tip_1_0	129	sulfhydryl oxidase 2-like	547	9.50E-63	60.60%	0.135		IPR017905 (G3DSA:1.20.120.GENE3D); IPR017905 (PFAM); PTHR22897 (PANTHER); PTHR22897:SF8 (PANTHER); IPR017905 (PROSITE_PROFILES); IPR017905 (SUPERFAMILY)
1457	488116_length_1641_cvg_12.1_tip_1_1	129	sulfhydryl oxidase 1-like	547	5.07E-34	64.60%	0.118		IPR012336 (G3DSA:3.40.30.GENE3D); PTHR22897 (PANTHER); NON_CYTOPLASMIC_DOMAIN (PHOBIUS); TRANSMEMBRANE (PHOBIUS); CYTOPLASMIC_DOMAIN (PHOBIUS)
1487	488918_length_1689_cvg_61.8_tip_1_2	795	er membrane protein complex subunit 10	547	9.38E-72	63.70%	0.18		IPR029615 (PTHR21397:PANTHER); PTHR21397 (PANTHER); CYTOPLASMIC_DOMAIN (PHOBIUS); NON_CYTOPLASMIC_DOMAIN (PHOBIUS); NON_CYTOPLASMIC_DOMAIN (PHOBIUS); CYTOPLASMIC_DOMAIN (PHOBIUS); TRANSMEMBRANE (PHOBIUS); TRANSMEMBRANE (PHOBIUS); TRANSMEMBRANE (PHOBIUS); TRANSMEMBRANE (PHOBIUS); NON_CYTOPLASMIC_DOMAIN (PHOBIUS); CYTOPLASMIC_DOMAIN (PHOBIUS); TRANSMEMBRANE (PHOBIUS); CYTOPLASMIC_DOMAIN (PHOBIUS); TRANSMEMBRANE (PHOBIUS); TMhelix (TMHMM); TMhelix (TMHMM); TMhelix (TMHMM); TMhelix (TMHMM); TMhelix (TMHMM); TMhelix (TMHMM); TMhelix (TMHMM)
1458	488172_length_1645_cvg_7.0_tip_1_5	59	ga-binding protein subunit beta-2	548	4.33E-101	57.40%	0.122		Coil (COILS); IPR002110 (PRINTS); IPR002110 (SMART); IPR020683 (G3DSA:1.25.40.GENE3D); IPR020683 (PFAM); PTHR24193:SF73 (PANTHER); PTHR24193 (PANTHER); IPR002110 (PROSITE_PROFILES); IPR020683 (PROSITE_PROFILES); IPR002110 (PROSITE_PROFILES); IPR002110 (PROSITE_PROFILES); IPR020683 (SUPERFAMILY)
1459	488196_length_1646_cvg_39.1_tip_1_3	367	polyubiquitin-c-like isoform 2	549	2.52E-155	99.00%	0.102		IPR019956 (PRINTS); IPR000626 (SMART); G3DSA:3.10.20.90 (GENE3D); IPR000626 (PFAM); PTHR10666 (PANTHER); IPR019954 (PROSITE_PATTERNS); IPR019954 (PROSITE_PATTERNS); IPR019954 (PROSITE_PATTERNS); NON_CYTOPLASMIC_DOMAIN (PHOBIUS); TRANSMEMBRANE (PHOBIUS); TRANSMEMBRANE (PHOBIUS); CYTOPLASMIC_DOMAIN (PHOBIUS); CYTOPLASMIC_DOMAIN (PHOBIUS); CYTOPLASMIC_DOMAIN (PHOBIUS); NON_CYTOPLASMIC_DOMAIN (PHOBIUS); NON_CYTOPLASMIC_DOMAIN (PHOBIUS); TRANSMEMBRANE (PHOBIUS); TRANSMEMBRANE (PHOBIUS); TRANSMEMBRANE (PHOBIUS); IPR000626 (PROSITE_PROFILES); IPR000626 (PROSITE_PROFILES); IPR000626 (PROSITE_PROFILES); IPR029071 (SUPERFAMILY); IPR029071 (SUPERFAMILY); IPR029071 (SUPERFAMILY); TMhelix (TMHMM); TMhelix (TMHMM)
1460	488230_length_1647_cvg_2.5_tip_1_3	24	isoform cra_b	549	9.92E-76	78.50%	0.136		IPR002290 (SMART); G3DSA:3.30.200.20 (GENE3D); IPR000719 (PFAM); G3DSA:1.10.510.10 (GENE3D); PTHR24348 (PANTHER); IPR008271 (PROSITE_PATTERNS); IPR017441 (PROSITE_PATTERNS); IPR000719 (PROSITE_PROFILES); IPR011009 (SUPERFAMILY)
1461	488230_length_1647_cvg_2.5_tip_1_5	24	serine threonine-protein kinase ulk3	549	5.59E-93	66.50%	0.417	Y	Coil (COILS); IPR007330 (SMART); IPR002290 (SMART); IPR007330 (G3DSA:1.20.58.GENE3D); IPR000719 (PFAM); IPR007330 (PFAM); IPR007330 (G3DSA:1.20.58.GENE3D); G3DSA:1.10.510.10 (GENE3D); PTHR24348 (PANTHER); IPR000719 (PROSITE_PROFILES); IPR007330 (SUPERFAMILY); IPR011009 (SUPERFAMILY); IPR007330 (SUPERFAMILY)

1462	488236_length_1648_cvg_21.3_tip_1_5	156	gamma-interferon-inducible lysosomal thiol reductase-like	549	1.00E-59	63.80%	0.136	IPR004911 (PFAM); IPR004911 (PANTHER); TRANSMEMBRANE (PHOBIUS); TRANSMEMBRANE (PHOBIUS); NON_CYTOPLASMIC_DOMAIN (PHOBIUS); CYTOPLASMIC_DOMAIN (PHOBIUS); CYTOPLASMIC_DOMAIN (PHOBIUS); NON_CYTOPLASMIC_DOMAIN (PHOBIUS); TRANSMEMBRANE (PHOBIUS); TMhelix (TMHMM); TMhelix (TMHMM)
1479	488656_length_1673_cvg_56.3_tip_1_0	514	hemagglutinin	549	5.53E-121	58.60%	0.108	IPR023612 (PRINTS); IPR013856 (G3DSA:3.10.170.GENE3D); IPR013856 (PFAM); IPR001570 (PFAM); G3DSA:1.10.390.10 (GENE3D); IPR007280 (PFAM); SSF55486 (SUPERFAMILY)
1463	488254_length_1649_cvg_12.0_tip_1_4	110	n-acetylglucosamine-1-phosphotransferase subunit gamma-like	550	5.63E-70	59.60%	0.332	Coil (COILS); PF13015 (PFAM); IPR009011 (G3DSA:2.70.130.GENE3D); PTHR12630 (PANTHER); PTHR12630:SF6 (PANTHER); IPR009011 (SUPERFAMILY)
1478	488634_length_1672_cvg_11.0_tip_1_0	95	zinc finger protein 425	550	1.76E-55	52.60%	0.1	IPR015880 (SMART); IPR013087 (G3DSA:3.30.160.GENE3D); IPR013087 (G3DSA:3.30.160.GENE3D); IPR007087 (PFAM); IPR013087 (G3DSA:3.30.160.GENE3D); PF13894 (PFAM); PF13465 (PFAM); PTHR24409 (PANTHER); PTHR24409:SF14 (PANTHER); IPR007087 (PROSITE_PATTERNS); IPR007087 (PROSITE_PATTERNS); IPR007087 (PROSITE_PATTERNS); IPR007087 (PROSITE_PATTERNS); IPR007087 (PROSITE_PATTERNS); IPR007087 (PROSITE_PATTERNS); IPR007087 (PROSITE_PATTERNS); IPR007087 (PROSITE_PATTERNS); IPR007087 (PROSITE_PATTERNS); IPR007087 (PROSITE_PATTERNS); IPR007087 (PROSITE_PATTERNS); IPR007087 (PROSITE_PATTERNS); IPR007087 (PROSITE_PROFILES); IPR007087 (PROSITE_PROFILES); IPR007087 (PROSITE_PROFILES); IPR007087 (PROSITE_PROFILES); IPR007087 (PROSITE_PROFILES); IPR007087 (PROSITE_PROFILES); IPR007087 (PROSITE_PROFILES); IPR007087 (PROSITE_PROFILES); IPR007087 (PROSITE_PROFILES); IPR007087 (PROSITE_PROFILES); IPR007087 (PROSITE_PROFILES); IPR007087 (PROSITE_PROFILES); SSF57667 (SUPERFAMILY); SSF57667 (SUPERFAMILY); SSF57667 (SUPERFAMILY); SSF57667 (SUPERFAMILY); SSF57667 (SUPERFAMILY); SSF57667 (SUPERFAMILY); SSF57667 (SUPERFAMILY)
1481	488730_length_1678_cvg_43.0_tip_1_1	438	protein sel-1 homolog 1	551	0	85.50%	0.1	IPR006597 (SMART); IPR011990 (G3DSA:1.25.40.GENE3D); IPR006597 (PFAM); IPR011990 (G3DSA:1.25.40.GENE3D); PTHR11102 (PANTHER); PTHR11102:SF55 (PANTHER); NON_CYTOPLASMIC_DOMAIN (PHOBIUS); CYTOPLASMIC_DOMAIN (PHOBIUS); TRANSMEMBRANE (PHOBIUS); NON_CYTOPLASMIC_DOMAIN (PHOBIUS); TRANSMEMBRANE (PHOBIUS); SSF81901 (SUPERFAMILY); SSF81901 (SUPERFAMILY); TMhelix (TMHMM); TMhelix (TMHMM)
1467	488400_length_1657_cvg_2.1_tip_1_3	23	acetylornithine deacetylase	553	0	99.00%	0.113	IPR010169 (TIGRFAM); IPR002933 (PFAM); G3DSA:3.40.630.10 (GENE3D); IPR011650 (PFAM); IPR011650 (G3DSA:3.30.70.GENE3D); PTHR11014 (PANTHER); PTHR11014:SF75 (PANTHER); IPR001261 (PROSITE_PATTERNS); IPR001261 (PROSITE_PATTERNS); IPR011650 (SUPERFAMILY); SSF53187 (SUPERFAMILY)
1468	488410_length_1659_cvg_7.2_tip_1_1	55	zinc finger protein 774-like	553	1.45E-21	59.90%	0.1	IPR015880 (SMART); IPR013087 (G3DSA:3.30.160.GENE3D); PF13465 (PFAM); IPR007087 (PFAM); IPR013087 (G3DSA:3.30.160.GENE3D); IPR013087 (G3DSA:3.30.160.GENE3D); IPR013087 (G3DSA:3.30.160.GENE3D); PTHR24409 (PANTHER); IPR007087 (PROSITE_PATTERNS); IPR007087 (PROSITE_PATTERNS); IPR007087 (PROSITE_PATTERNS); IPR007087 (PROSITE_PATTERNS); IPR007087 (PROSITE_PROFILES); IPR007087 (PROSITE_PROFILES); IPR007087 (PROSITE_PROFILES); IPR007087 (PROSITE_PROFILES); SSF57667 (SUPERFAMILY); SSF57667 (SUPERFAMILY)
1469	488436_length_1661_cvg_18.5_tip_1_0	163	venom carboxylesterase-6 isoform x2	554	2.91E-50	53.10%	0.169	IPR002018 (PFAM); IPR029058 (G3DSA:3.40.50.GENE3D); PTHR11559 (PANTHER); CYTOPLASMIC_DOMAIN (PHOBIUS); TRANSMEMBRANE (PHOBIUS); CYTOPLASMIC_DOMAIN (PHOBIUS); TRANSMEMBRANE (PHOBIUS); TRANSMEMBRANE (PHOBIUS); TRANSMEMBRANE (PHOBIUS); NON_CYTOPLASMIC_DOMAIN (PHOBIUS); CYTOPLASMIC_DOMAIN (PHOBIUS); NON_CYTOPLASMIC_DOMAIN (PHOBIUS); NON_CYTOPLASMIC_DOMAIN (PHOBIUS); TRANSMEMBRANE (PHOBIUS); IPR029058 (SUPERFAMILY); TMhelix (TMHMM); TMhelix (TMHMM); TMhelix (TMHMM); TMhelix (TMHMM); TMhelix (TMHMM)
1470	488456_length_1662_cvg_2.3_tip_1_5	23	hypothetical protein Y032_0075g928	554	2.08E-23	50.20%	0.112	PTHR23022 (PANTHER); PTHR23022:SF64 (PANTHER); TRANSMEMBRANE (PHOBIUS); NON_CYTOPLASMIC_DOMAIN (PHOBIUS); NON_CYTOPLASMIC_DOMAIN (PHOBIUS); CYTOPLASMIC_DOMAIN (PHOBIUS); TRANSMEMBRANE (PHOBIUS); TMhelix (TMHMM); TMhelix (TMHMM)

1471	488466_length_1662_cvg_50.2_tip_1_0	384	u3 small nucleolar rna-interacting protein 2-like isoform x1	554	0	72.50%	0.228		Coil (COILS); IPR020472 (PRINTS); IPR001680 (SMART); IPR015943 (G3DSA:2.130.10.GENE3D); IPR001680 (PFAM); PTHR19865 (PANTHER); IPR019775 (PROSITE_PATTERNS); TRANSMEMBRANE (PHOBIUS); CYTOPLASMIC_DOMAIN (PHOBIUS); NON_CYTOPLASMIC_DOMAIN (PHOBIUS); IPR001680 (PROSITE_PROFILES); IPR001680 (PROSITE_PROFILES); IPR001680 (PROSITE_PROFILES); IPR001680 (PROSITE_PROFILES); IPR001680 (PROSITE_PROFILES); IPR001680 (PROSITE_PROFILES); IPR017986 (PROSITE_PROFILES); IPR017986 (SUPERFAMILY); TMhelix (TMHMM)
1472	488486_length_1663_cvg_20.5_tip_1_1	188	gastrula zinc finger	554	3.16E-41	56.20%	0.256		IPR015880 (SMART); IPR013087 (G3DSA:3.30.160.GENE3D); IPR013087 (G3DSA:3.30.160.GENE3D); PF13465 (PFAM); IPR007087 (PFAM); PTHR24409 (PANTHER); PTHR24409:SF13 (PANTHER); IPR007087 (PROSITE_PATTERNS); IPR007087 (PROSITE_PATTERNS); IPR007087 (PROSITE_PATTERNS); IPR007087 (PROSITE_PATTERNS); IPR007087 (PROSITE_PATTERNS); IPR007087 (PROSITE_PATTERNS); IPR007087 (PROSITE_PATTERNS); IPR007087 (PROSITE_PATTERNS); TRANSMEMBRANE (PHOBIUS); CYTOPLASMIC_DOMAIN (PHOBIUS); NON_CYTOPLASMIC_DOMAIN (PHOBIUS); IPR007087 (PROSITE_PROFILES); IPR007087 (PROSITE_PROFILES); IPR007087 (PROSITE_PROFILES); IPR007087 (PROSITE_PROFILES); IPR007087 (PROSITE_PROFILES); IPR007087 (PROSITE_PROFILES); IPR007087 (PROSITE_PROFILES); IPR007087 (PROSITE_PROFILES); SSF57667 (SUPERFAMILY); SSF57667 (SUPERFAMILY); SSF57667 (SUPERFAMILY); SSF57667 (SUPERFAMILY)
1473	488490_length_1663_cvg_6.2_tip_1_0	42	cytochrome p450 6k1-like	555	2.42E-129	59.20%	0.131		IPR002401 (PRINTS); IPR001128 (PRINTS); IPR001128 (PFAM); IPR001128 (G3DSA:1.10.630.GENE3D); PTHR24292 (PANTHER); PTHR24292:SF24 (PANTHER); IPR017972 (PROSITE_PATTERNS); CYTOPLASMIC_DOMAIN (PHOBIUS); NON_CYTOPLASMIC_DOMAIN (PHOBIUS); TRANSMEMBRANE (PHOBIUS); IPR001128 (SUPERFAMILY); TMhelix (TMHMM)
1474	488538_length_1667_cvg_61.3_tip_1_4	774	retinal dehydrogenase 1-like	556	0	80.40%	0.507	Y	IPR016162 (G3DSA:3.40.605.GENE3D); IPR016163 (G3DSA:3.40.309.GENE3D); IPR015590 (PFAM); PTHR11699 (PANTHER); PTHR11699:SF145 (PANTHER); IPR029510 (PROSITE_PATTERNS); IPR016160 (PROSITE_PATTERNS); SignalP-noTM (SIGNALP_EUK); IPR016161 (SUPERFAMILY)
1475	488554_length_1668_cvg_64.5_tip_1_2	1113	serine proteinase stubble	556	4.45E-31	45.80%	0.221		PR01217 (PRINTS); IPR001254 (SMART); G3DSA:2.40.10.10 (GENE3D); G3DSA:2.40.10.10 (GENE3D); G3DSA:2.40.10.10 (GENE3D); IPR001254 (PFAM); PTHR24258:SF96 (PANTHER); PTHR24258 (PANTHER); TRANSMEMBRANE (PHOBIUS); NON_CYTOPLASMIC_DOMAIN (PHOBIUS); CYTOPLASMIC_DOMAIN (PHOBIUS); IPR001254 (PROSITE_PROFILES); IPR009003 (SUPERFAMILY); TMhelix (TMHMM)
1476	488582_length_1669_cvg_8.2_tip_1_3	66	zinc finger protein 287-like	557	7.42E-73	56.10%	0.104		IPR015880 (SMART); PF13912 (PFAM); IPR013087 (G3DSA:3.30.160.GENE3D); IPR013087 (G3DSA:3.30.160.GENE3D); IPR013087 (G3DSA:3.30.160.GENE3D); PF13465 (PFAM); PTHR24409 (PANTHER); IPR007087 (PROSITE_PATTERNS); IPR007087 (PROSITE_PATTERNS); IPR007087 (PROSITE_PATTERNS); IPR007087 (PROSITE_PATTERNS); IPR007087 (PROSITE_PATTERNS); IPR007087 (PROSITE_PATTERNS); IPR007087 (PROSITE_PATTERNS); IPR007087 (PROSITE_PATTERNS); IPR007087 (PROSITE_PATTERNS); IPR007087 (PROSITE_PROFILES); IPR007087 (PROSITE_PROFILES); IPR007087 (PROSITE_PROFILES); IPR007087 (PROSITE_PROFILES); IPR007087 (PROSITE_PROFILES); IPR007087 (PROSITE_PROFILES); IPR007087 (PROSITE_PROFILES); IPR007087 (PROSITE_PROFILES); IPR007087 (PROSITE_PROFILES); SSF57667 (SUPERFAMILY); SSF57667 (SUPERFAMILY); SSF57667 (SUPERFAMILY); SSF57667 (SUPERFAMILY); SSF57667 (SUPERFAMILY)
1477	488590_length_1670_cvg_10.5_tip_1_0	110	macro domain-containing protein 2	557	1.91E-83	69.50%	0.104		Coil (COILS); IPR002589 (SMART); IPR002589 (PFAM); G3DSA:3.40.220.10 (GENE3D); PTHR11106 (PANTHER); CYTOPLASMIC_DOMAIN (PHOBIUS); TRANSMEMBRANE (PHOBIUS); CYTOPLASMIC_DOMAIN (PHOBIUS); NON_CYTOPLASMIC_DOMAIN (PHOBIUS); TRANSMEMBRANE (PHOBIUS); IPR002589 (PROSITE_PROFILES); SSF52949 (SUPERFAMILY); TMhelix (TMHMM)

1480	488714_length_1677_cvg_25.3_tip_1_3	186	3-hydroxyisobutyrate mitochondrial	559	5.52E-151	81.30%	0.306	IPR013328 (G3DSA:1.10.1040.GENE3D); IPR011548 (TIGRFAM); IPR006115 (PFAM); IPR029154 (PFAM); IPR016040 (G3DSA:3.40.50.GENE3D); PTHR22981 (PANTHER); PTHR22981:SF51 (PANTHER); IPR002204 (PROSITE_PATTERNS); CYTOPLASMIC_DOMAIN (PHOBIUS); SIGNAL_PEPTIDE (PHOBIUS); SIGNAL_PEPTIDE_C_REGION (PHOBIUS); TRANSMEMBRANE (PHOBIUS); SIGNAL_PEPTIDE_H_REGION (PHOBIUS); SIGNAL_PEPTIDE_N_REGION (PHOBIUS); NON_CYTOPLASMIC_DOMAIN (PHOBIUS); SignalP-TM (SIGNALP_GRAM_POSITIVE); IPR008927 (SUPERFAMILY); SSF51735 (SUPERFAMILY)
1482	488762_length_1679_cvg_4.1_tip_1_2	37	basement membrane-specific heparan sulfate proteoglycan core isoform x2	559	4.72E-104	75.40%	0.145	IPR013106 (SMART); IPR003598 (SMART); IPR003599 (SMART); IPR013106 (PFAM); IPR013783 (G3DSA:2.60.40.GENE3D); PF13895 (PFAM); PTHR23279 (PANTHER); TRANSMEMBRANE (PHOBIUS); CYTOPLASMIC_DOMAIN (PHOBIUS); TRANSMEMBRANE (PHOBIUS); CYTOPLASMIC_DOMAIN (PHOBIUS); TRANSMEMBRANE (PHOBIUS); NON_CYTOPLASMIC_DOMAIN (PHOBIUS); NON_CYTOPLASMIC_DOMAIN (PHOBIUS); TRANSMEMBRANE (PHOBIUS); NON_CYTOPLASMIC_DOMAIN (PHOBIUS); IPR007110 (PROSITE_PROFILES); IPR007110 (PROSITE_PROFILES); SSF48726 (SUPERFAMILY); SSF48726 (SUPERFAMILY); TMhelix (TMHMM); TMhelix (TMHMM)
1483	488774_length_1680_cvg_35.5_tip_1_1	274	zinc finger protein	560	1.90E-15	61.00%	0.1	IPR015880 (SMART); PF13894 (PFAM); IPR013087 (G3DSA:3.30.160.GENE3D); PF13465 (PFAM); IPR013087 (G3DSA:3.30.160.GENE3D); PTHR24409 (PANTHER); PTHR24409:SF13 (PANTHER); IPR007087 (PROSITE_PATTERNS); IPR007087 (PROSITE_PATTERNS); IPR007087 (PROSITE_PATTERNS); CYTOPLASMIC_DOMAIN (PHOBIUS); NON_CYTOPLASMIC_DOMAIN (PHOBIUS); TRANSMEMBRANE (PHOBIUS); IPR007087 (PROSITE_PROFILES); IPR007087 (PROSITE_PROFILES); IPR007087 (PROSITE_PROFILES); SSF57667 (SUPERFAMILY); SSF57667 (SUPERFAMILY)
1495	489138_length_1706_cvg_28.1_tip_1_3	1128	adp-ribosylation factor-related protein 1	560	1.52E-123	94.40%	0.22	IPR001806 (PRINTS); IPR006687 (SMART); IPR003579 (SMART); IPR024156 (SMART); IPR027417 (G3DSA:3.40.50.GENE3D); IPR006689 (PFAM); IPR005225 (TIGRFAM); PTHR11711 (PANTHER); PTHR11711:SF19 (PANTHER); NON_CYTOPLASMIC_DOMAIN (PHOBIUS); CYTOPLASMIC_DOMAIN (PHOBIUS); TRANSMEMBRANE (PHOBIUS); CYTOPLASMIC_DOMAIN (PHOBIUS); NON_CYTOPLASMIC_DOMAIN (PHOBIUS); TRANSMEMBRANE (PHOBIUS); TRANSMEMBRANE (PHOBIUS); TRANSMEMBRANE (PHOBIUS); CYTOPLASMIC_DOMAIN (PHOBIUS); IPR024156 (PROSITE_PROFILES); IPR027417 (SUPERFAMILY); TMhelix (TMHMM); TMhelix (TMHMM)
1484	488816_length_1683_cvg_5.9_tip_1_1	76	cd109 antigen-like isoform x2	561	0	74.80%	0.168	IPR009048 (G3DSA:2.60.40.GENE3D); IPR011626 (PFAM); IPR008930 (G3DSA:1.50.10.GENE3D); IPR009048 (PFAM); PTHR11412:SF84 (PANTHER); PTHR11412 (PANTHER); NON_CYTOPLASMIC_DOMAIN (PHOBIUS); CYTOPLASMIC_DOMAIN (PHOBIUS); TRANSMEMBRANE (PHOBIUS); IPR008930 (SUPERFAMILY); IPR009048 (SUPERFAMILY); TMhelix (TMHMM)
1496	489166_length_1707_cvg_9.6_tip_1_0	103	aael005189- partial	561	3.87E-128	69.30%	0.129	IPR003112 (SMART); IPR003112 (PFAM); PTHR23192:SF33 (PANTHER); PTHR23192 (PANTHER); TRANSMEMBRANE (PHOBIUS); CYTOPLASMIC_DOMAIN (PHOBIUS); NON_CYTOPLASMIC_DOMAIN (PHOBIUS); IPR003112 (PROSITE_PROFILES); TMhelix (TMHMM); TMhelix (TMHMM)
1485	488842_length_1685_cvg_11.5_tip_1_0	165	hemocyte protein-glutamine gamma-glutamyltransferase-like	562	0	72.10%	0.145	IPR002931 (SMART); IPR002931 (PFAM); IPR002931 (G3DSA:3.90.260.GENE3D); IPR013783 (G3DSA:2.60.40.GENE3D); IPR008958 (PFAM); IPR023608 (PIRSF); IPR013783 (G3DSA:2.60.40.GENE3D); IPR023608 (PANTHER); PTHR11590:SF40 (PANTHER); IPR008958 (SUPERFAMILY); SSF54001 (SUPERFAMILY); IPR008958 (SUPERFAMILY)
1486	488890_length_1688_cvg_9.6_tip_1_5	70	sepiapterin reductase-like	562	1.75E-80	63.70%	0.114	IPR002347 (PRINTS); IPR006393 (TIGRFAM); IPR016040 (G3DSA:3.40.50.GENE3D); IPR002198 (PFAM); PTHR24318 (PANTHER); PTHR24318:SF1 (PANTHER); TRANSMEMBRANE (PHOBIUS); NON_CYTOPLASMIC_DOMAIN (PHOBIUS); CYTOPLASMIC_DOMAIN (PHOBIUS); SSF51735 (SUPERFAMILY)
1501	489258_length_1713_cvg_40.4_tip_1_1	357	46 kda fk506-binding nuclear protein	562	7.78E-47	73.40%	0.248	Coil (COILS); G3DSA:3.10.50.40 (GENE3D); IPR001179 (PFAM); IPR023566 (PANTHER); IPR001179 (PROSITE_PROFILES); IPR024057 (SUPERFAMILY); SSF54534 (SUPERFAMILY)

1488	488944_length_1691_cvg_61.4_tip_1_2	720	protein tumorous imaginal mitochondrial-like isoform x2	563	0	75.10%	0.199	IPR001623 (PRINTS); IPR001623 (SMART); IPR001305 (G3DSA:2.10.230.GENE3D); IPR001305 (PFAM); IPR001623 (PFAM); IPR002939 (PFAM); G3DSA:2.60.260.20 (GENE3D); IPR001623 (G3DSA:1.10.287.GENE3D); PTHR24076 (PANTHER); PTHR24076:SF79 (PANTHER); IPR018253 (PROSITE_PATTERNS); IPR001623 (PROSITE_PROFILES); IPR012724 (HAMAP); IPR001305 (PROSITE_PROFILES); IPR001305 (SUPERFAMILY); IPR001623 (SUPERFAMILY); IPR008971 (SUPERFAMILY); IPR008971 (SUPERFAMILY)
1504	489370_length_1722_cvg_7.2_tip_1_1	90	wd repeat-containing protein 3	565	0	78.20%	0.114	Coil (COILS); IPR020472 (PRINTS); IPR001680 (SMART); IPR001680 (PFAM); IPR015943 (G3DSA:2.130.10.GENE3D); IPR007148 (PFAM); PTHR19853 (PANTHER); PTHR19853:SF0 (PANTHER); IPR019775 (PROSITE_PATTERNS); IPR001680 (PROSITE_PROFILES); IPR001680 (PROSITE_PROFILES); IPR001680 (PROSITE_PROFILES); IPR017986 (PROSITE_PROFILES); IPR001680 (PROSITE_PROFILES); IPR017986 (SUPERFAMILY)
1489	489026_length_1698_cvg_4.7_tip_1_4	51	centromeric protein	566	1.74E-103	66.60%	0.276	Coil (COILS); Coil (COILS); Coil (COILS); Coil (COILS); Coil (COILS); IPR027881 (PFAM); PTHR15742 (PANTHER); PTHR15742:SF0 (PANTHER)
1490	489042_length_1699_cvg_6.2_tip_1_2	40	guanine nucleotide-binding protein g g g subunit beta-1 isoform x1	566	0	98.40%	0.159	IPR001632 (PRINTS); IPR020472 (PRINTS); IPR001680 (SMART); IPR001680 (PFAM); IPR015943 (G3DSA:2.130.10.GENE3D); IPR016346 (PANTHER); IPR019775 (PROSITE_PATTERNS); IPR019775 (PROSITE_PATTERNS); IPR019775 (PROSITE_PATTERNS); IPR019775 (PROSITE_PATTERNS); TRANSMEMBRANE (PHOBIUS); TRANSMEMBRANE (PHOBIUS); NON_CYTOPLASMIC_DOMAIN (PHOBIUS); NON_CYTOPLASMIC_DOMAIN (PHOBIUS); CYTOPLASMIC_DOMAIN (PHOBIUS); TRANSMEMBRANE (PHOBIUS); CYTOPLASMIC_DOMAIN (PHOBIUS); NON_CYTOPLASMIC_DOMAIN (PHOBIUS); TRANSMEMBRANE (PHOBIUS); IPR001680 (PROSITE_PROFILES); IPR001680 (PROSITE_PROFILES); IPR001680 (PROSITE_PROFILES); IPR001680 (PROSITE_PROFILES); IPR017986 (PROSITE_PROFILES); IPR001680 (PROSITE_PROFILES); IPR001680 (PROSITE_PROFILES); IPR017986 (SUPERFAMILY); TMhelix (TMHMM); TMhelix (TMHMM); TMhelix (TMHMM)
1515	489604_length_1739_cvg_68.2_tip_1_4	2297	bola-like protein 2-like	566	1.16E-33	83.10%	0.261	IPR002634 (G3DSA:3.30.300.GENE3D); IPR002634 (PFAM); PTHR12735:SF2 (PANTHER); PTHR12735 (PANTHER); CYTOPLASMIC_DOMAIN (PHOBIUS); NON_CYTOPLASMIC_DOMAIN (PHOBIUS); CYTOPLASMIC_DOMAIN (PHOBIUS); TRANSMEMBRANE (PHOBIUS); TRANSMEMBRANE (PHOBIUS); TRANSMEMBRANE (PHOBIUS); NON_CYTOPLASMIC_DOMAIN (PHOBIUS); IPR002634 (SUPERFAMILY)
1491	489048_length_1699_cvg_4.2_tip_1_3	40	wee1-like protein kinase	567	0	74.30%	0.257	IPR002290 (SMART); IPR017164 (PIRSF); G3DSA:3.30.200.20 (GENE3D); G3DSA:1.10.510.10 (GENE3D); IPR000719 (PFAM); PTHR11042:SF69 (PANTHER); PTHR11042 (PANTHER); IPR008271 (PROSITE_PATTERNS); IPR017441 (PROSITE_PATTERNS); IPR000719 (PROSITE_PROFILES); IPR011009 (SUPERFAMILY)
1492	489070_length_1700_cvg_74.4_tip_1_1	19098	tissue factor pathway inhibitor 2	567	3.91E-31	62.20%	0.136	Coil (COILS); IPR002223 (PRINTS); IPR002223 (SMART); IPR002223 (G3DSA:4.10.410.GENE3D); IPR002223 (G3DSA:4.10.410.GENE3D); IPR002223 (PFAM); PTHR10083 (PANTHER); IPR020901 (PROSITE_PATTERNS); IPR020901 (PROSITE_PATTERNS); NON_CYTOPLASMIC_DOMAIN (PHOBIUS); TRANSMEMBRANE (PHOBIUS); TRANSMEMBRANE (PHOBIUS); CYTOPLASMIC_DOMAIN (PHOBIUS); TRANSMEMBRANE (PHOBIUS); CYTOPLASMIC_DOMAIN (PHOBIUS); TRANSMEMBRANE (PHOBIUS); NON_CYTOPLASMIC_DOMAIN (PHOBIUS); CYTOPLASMIC_DOMAIN (PHOBIUS); IPR002223 (PROSITE_PROFILES); IPR002223 (PROSITE_PROFILES); IPR002223 (SUPERFAMILY); IPR002223 (SUPERFAMILY); TMhelix (TMHMM); TMhelix (TMHMM); TMhelix (TMHMM); TMhelix (TMHMM)
1493	489096_length_1702_cvg_13.3_tip_1_4	104	zinc finger protein 235-like	567	2.32E-28	43.50%	0.099	IPR015880 (SMART); PF13894 (PFAM); IPR013087 (G3DSA:3.30.160.GENE3D); IPR013087 (G3DSA:3.30.160.GENE3D); PF13465 (PFAM); IPR007087 (PFAM); PTHR24409 (PANTHER); PTHR24409:SF14 (PANTHER); IPR007087 (PROSITE_PATTERNS); IPR007087 (PROSITE_PATTERNS); IPR007087 (PROSITE_PATTERNS); IPR007087 (PROSITE_PATTERNS); IPR007087 (PROSITE_PATTERNS); IPR007087 (PROSITE_PROFILES); IPR007087 (PROSITE_PROFILES); IPR007087 (PROSITE_PROFILES); IPR007087 (PROSITE_PROFILES); IPR007087 (PROSITE_PROFILES); SSF57667 (SUPERFAMILY); SSF57667 (SUPERFAMILY); SSF57667 (SUPERFAMILY)
1494	489106_length_1703_cvg_8.6_tip_1_2	61	low quality protein: selenoprotein h	567	1.62E-18	63.70%	0.102	IPR020478 (PRINTS); IPR017956 (SMART); IPR011893 (PFAM); IPR011893 (TIGRFAM); IPR012336 (G3DSA:3.40.30.GENE3D); IPR017956 (PFAM); CYTOPLASMIC_DOMAIN (PHOBIUS); NON_CYTOPLASMIC_DOMAIN (PHOBIUS); TRANSMEMBRANE (PHOBIUS); IPR012336 (SUPERFAMILY); TMhelix (TMHMM)

1510	489494_length_1731_cvg_37.5_tip_1_4	318	aael014742- partial	567	5.69E-86	56.40%	0.107	IPR002110 (PRINTS); IPR002110 (SMART); IPR002110 (PFAM); IPR020683 (G3DSA:1.25.40.GENE3D); IPR020683 (G3DSA:1.25.40.GENE3D); IPR020683 (G3DSA:1.25.40.GENE3D); IPR020683 (PFAM); IPR020683 (G3DSA:1.25.40.GENE3D); PTHR24133 (PANTHER); PTHR24133:SF17 (PANTHER); IPR002110 (PROSITE_PROFILES); IPR002110 (PROSITE_PROFILES); IPR002110 (PROSITE_PROFILES); IPR002110 (PROSITE_PROFILES); IPR002110 (PROSITE_PROFILES); IPR002110 (PROSITE_PROFILES); IPR002110 (PROSITE_PROFILES); IPR002110 (PROSITE_PROFILES); IPR002110 (PROSITE_PROFILES); IPR020683 (PROSITE_PROFILES); IPR002110 (PROSITE_PROFILES); IPR002110 (PROSITE_PROFILES); IPR002110 (PROSITE_PROFILES); IPR020683 (SUPERFAMILY); IPR020683 (SUPERFAMILY)
1497	489180_length_1708_cvg_7.6_tip_1_5	58	spermine oxidase-like	569	1.22E-132	62.60%	0.122	IPR001613 (PRINTS); G3DSA:3.50.50.60 (GENE3D); IPR002937 (PFAM); PTHR10742:SF255 (PANTHER); PTHR10742 (PANTHER); TRANSMEMBRANE (PHOBIUS); CYTOPLASMIC_DOMAIN (PHOBIUS); NON_CYTOPLASMIC_DOMAIN (PHOBIUS); NON_CYTOPLASMIC_DOMAIN (PHOBIUS); TRANSMEMBRANE (PHOBIUS); SSF54373 (SUPERFAMILY); SSF51905 (SUPERFAMILY); TMhelix (TMHMM); TMhelix (TMHMM)
1503	489316_length_1717_cvg_6.1_tip_1_5	75	ves g 5 allergen	569	1.55E-41	51.50%	0.318	IPR001283 (PRINTS); IPR002413 (PRINTS); IPR002172 (SMART); IPR014044 (SMART); IPR014044 (G3DSA:3.40.33.GENE3D); IPR002172 (G3DSA:4.10.400.GENE3D); IPR002172 (G3DSA:4.10.400.GENE3D); IPR002172 (PFAM); IPR014044 (PFAM); IPR001283 (PANTHER); IPR018244 (PROSITE_PATTERNS); IPR018244 (PROSITE_PATTERNS); SIGNAL_PEPTIDE_C_REGION (PHOBIUS); SIGNAL_PEPTIDE (PHOBIUS); SIGNAL_PEPTIDE_H_REGION (PHOBIUS); NON_CYTOPLASMIC_DOMAIN (PHOBIUS); SIGNAL_PEPTIDE_N_REGION (PHOBIUS); IPR002172 (PROSITE_PROFILES); IPR002172 (PROSITE_PROFILES); IPR002172 (SUPERFAMILY); IPR014044 (SUPERFAMILY); IPR002172 (SUPERFAMILY)
1498	489196_length_1710_cvg_3.8_tip_1_5	41	chondroitin sulfate synthase 1-like	570	2.05E-86	53.10%	0.261	IPR008428 (PFAM); IPR029044 (G3DSA:3.90.550.GENE3D); PTHR12369:SF11 (PANTHER); PTHR12369 (PANTHER); NON_CYTOPLASMIC_DOMAIN (PHOBIUS); CYTOPLASMIC_DOMAIN (PHOBIUS); CYTOPLASMIC_DOMAIN (PHOBIUS); TRANSMEMBRANE (PHOBIUS); TRANSMEMBRANE (PHOBIUS); IPR029044 (SUPERFAMILY); TMhelix (TMHMM); TMhelix (TMHMM)
1499	489242_length_1712_cvg_3.8_tip_1_5	36	hemagglutinin proteinase	570	5.02E-102	54.70%	0.191	IPR023612 (PRINTS); IPR001570 (PFAM); IPR025711 (PFAM); IPR011096 (PFAM); G3DSA:1.10.390.10 (GENE3D); IPR013856 (G3DSA:3.10.170.GENE3D); IPR013856 (PFAM); TRANSMEMBRANE (PHOBIUS); CYTOPLASMIC_DOMAIN (PHOBIUS); NON_CYTOPLASMIC_DOMAIN (PHOBIUS); SSF55486 (SUPERFAMILY); TMhelix (TMHMM)
1500	489250_length_1712_cvg_61.5_tip_1_5	939	clotting factor b-like	570	2.33E-66	60.30%	0.226	IPR001314 (PRINTS); IPR001254 (SMART); IPR001254 (PFAM); G3DSA:2.40.10.10 (GENE3D); G3DSA:2.40.10.10 (GENE3D); G3DSA:2.40.10.10 (GENE3D); PTHR24256 (PANTHER); IPR018114 (PROSITE_PATTERNS); IPR018114 (PROSITE_PATTERNS); TRANSMEMBRANE (PHOBIUS); NON_CYTOPLASMIC_DOMAIN (PHOBIUS); CYTOPLASMIC_DOMAIN (PHOBIUS); IPR001254 (PROSITE_PROFILES); IPR009003 (SUPERFAMILY)
1512	489540_length_1734_cvg_66.7_tip_1_4	931	abhydrolase domain-containing protein 15-like	570	1.93E-80	57.80%	0.098	IPR029058 (G3DSA:3.40.50.GENE3D); IPR000073 (PFAM); PTHR10794:SF28 (PANTHER); PTHR10794 (PANTHER); TRANSMEMBRANE (PHOBIUS); CYTOPLASMIC_DOMAIN (PHOBIUS); CYTOPLASMIC_DOMAIN (PHOBIUS); NON_CYTOPLASMIC_DOMAIN (PHOBIUS); TRANSMEMBRANE (PHOBIUS); IPR029058 (SUPERFAMILY); TMhelix (TMHMM); TMhelix (TMHMM)
1514	489598_length_1739_cvg_60.3_tip_1_3	816	low quality protein: prohormone-4	571	1.95E-24	99.00%	0.12	CYTOPLASMIC_DOMAIN (PHOBIUS); TRANSMEMBRANE (PHOBIUS); NON_CYTOPLASMIC_DOMAIN (PHOBIUS); CYTOPLASMIC_DOMAIN (PHOBIUS); TRANSMEMBRANE (PHOBIUS); NON_CYTOPLASMIC_DOMAIN (PHOBIUS); TRANSMEMBRANE (PHOBIUS); TMhelix (TMHMM); TMhelix (TMHMM); TMhelix (TMHMM); TMhelix (TMHMM)
1502	489270_length_1714_cvg_10.8_tip_1_0	56	trehalase-like isoform x1	572	1.43E-100	69.40%	0.105	IPR001661 (PRINTS); IPR001661 (PFAM); IPR001661 (PANTHER); PTHR23403:SF1 (PANTHER); IPR018232 (PROSITE_PATTERNS); TRANSMEMBRANE (PHOBIUS); NON_CYTOPLASMIC_DOMAIN (PHOBIUS); TRANSMEMBRANE (PHOBIUS); NON_CYTOPLASMIC_DOMAIN (PHOBIUS); CYTOPLASMIC_DOMAIN (PHOBIUS); CYTOPLASMIC_DOMAIN (PHOBIUS); TRANSMEMBRANE (PHOBIUS); IPR008928 (SUPERFAMILY); TMhelix (TMHMM); TMhelix (TMHMM); TMhelix (TMHMM)

1506	489406_length_1724_cvg_64.9_tip_1_5	835	di-n-acetylchitobiase	574	5.24E-114	63.10%	0.469 Y	IPR011583 (SMART); IPR013781 (G3DSA:3.20.20.GENE3D); IPR001223 (PFAM); PTHR11177:SF146 (PANTHER); PTHR11177 (PANTHER); NON_CYTOPLASMIC_DOMAIN (PHOBIUS); SIGNAL_PEPTIDE_H_REGION (PHOBIUS); SIGNAL_PEPTIDE_N_REGION (PHOBIUS); SIGNAL_PEPTIDE_C_REGION (PHOBIUS); SIGNAL_PEPTIDE (PHOBIUS); IPR017853 (SUPERFAMILY); TMhelix (TMHMM)
1505	489386_length_1723_cvg_13.0_tip_1_0	82	apolipoprotein d-like	575	1.43E-57	62.20%	0.127	IPR003057 (PRINTS); IPR000566 (PFAM); IPR012674 (G3DSA:2.40.128.GENE3D); PTHR10612:SF7 (PANTHER); PTHR10612 (PANTHER); IPR022272 (PROSITE_PATTERNS); CYTOPLASMIC_DOMAIN (PHOBIUS); TRANSMEMBRANE (PHOBIUS); NON_CYTOPLASMIC_DOMAIN (PHOBIUS); IPR011038 (SUPERFAMILY); TMhelix (TMHMM)
1507	489412_length_1725_cvg_3.8_tip_1_0	51	fmrf_musdo ame: full=fmrfamide neuropeptides contains: ame: full= amide-2 contains: ame: full= amide-3 contains: ame: full= amide-4 contains: ame: full= amide-5 contains: ame: full= amide-6 contains: ame: full= amide-7 contains: ame: full= amide-8 ame: full= amide-10 contains: ame: full= amide-9 ame: full= amide-11 ame: full= amide-12 ame: full= amide-13 contains: ame: full= amide-14 contains: ame: full= amide-15 contains: ame: full= amide-17 contains: ame: full= amide-16 contains: ame: full= amide-18 flags: precursor	575	1.65E-19	58.10%	0.609 Y	IPR002544 (PFAM); PTHR20986 (PANTHER); PTHR20986:SF1 (PANTHER); TRANSMEMBRANE (PHOBIUS); SIGNAL_PEPTIDE_C_REGION (PHOBIUS); NON_CYTOPLASMIC_DOMAIN (PHOBIUS); SIGNAL_PEPTIDE (PHOBIUS); SIGNAL_PEPTIDE_N_REGION (PHOBIUS); SIGNAL_PEPTIDE_H_REGION (PHOBIUS); NON_CYTOPLASMIC_DOMAIN (PHOBIUS); CYTOPLASMIC_DOMAIN (PHOBIUS); TRANSMEMBRANE (PHOBIUS); SignalP-noTM (SIGNALP_EUK)
1508	489444_length_1727_cvg_8.3_tip_1_5	90	integrin-linked protein kinase	575	0	87.10%	0.236	IPR002110 (SMART); IPR020683 (G3DSA:1.25.40.GENE3D); IPR016253 (PIRSF); G3DSA:1.10.510.10 (GENE3D); IPR002110 (PFAM); IPR020683 (PFAM); IPR001245 (PFAM); G3DSA:3.30.200.20 (GENE3D); PTHR23257:SF30 (PANTHER); PTHR23257 (PANTHER); NON_CYTOPLASMIC_DOMAIN (PHOBIUS); TRANSMEMBRANE (PHOBIUS); CYTOPLASMIC_DOMAIN (PHOBIUS); IPR020683 (PROSITE_PROFILES); IPR002110 (PROSITE_PROFILES); IPR000719 (PROSITE_PROFILES); IPR002110 (PROSITE_PROFILES); IPR002110 (PROSITE_PROFILES); IPR020683 (SUPERFAMILY); IPR011009 (SUPERFAMILY); TMhelix (TMHMM)
1509	489474_length_1729_cvg_3.0_tip_1_5	24	von willebrand factor like 1	576	1.07E-20	39.50%	0.102	IPR001007 (PROSITE_PATTERNS); PD968187 (PRODOM); IPR001007 (PROSITE_PROFILES); IPR002919 (SUPERFAMILY)
1511	489504_length_1732_cvg_5.9_tip_1_2	54	---NA---	577			0.12	IPR002350 (SMART); IPR002350 (PFAM); G3DSA:3.30.60.30 (GENE3D); NON_CYTOPLASMIC_DOMAIN (PHOBIUS); TRANSMEMBRANE (PHOBIUS); CYTOPLASMIC_DOMAIN (PHOBIUS); TRANSMEMBRANE (PHOBIUS); NON_CYTOPLASMIC_DOMAIN (PHOBIUS); IPR002350 (PROSITE_PROFILES); SSF100895 (SUPERFAMILY); TMhelix (TMHMM); TMhelix (TMHMM)
1520	489852_length_1756_cvg_60.5_tip_1_5	1153	serine protease easter-like	577	1.62E-71	54.70%	0.217	IPR001314 (PRINTS); IPR006604 (SMART); IPR001254 (SMART); IPR022700 (PFAM); IPR001254 (PFAM); G3DSA:2.40.10.10 (GENE3D); G3DSA:2.40.10.10 (GENE3D); G3DSA:2.40.10.10 (GENE3D); PTHR24260 (PANTHER); PTHR24260:SF3 (PANTHER); IPR018114 (PROSITE_PATTERNS); IPR018114 (PROSITE_PATTERNS); NON_CYTOPLASMIC_DOMAIN (PHOBIUS); NON_CYTOPLASMIC_DOMAIN (PHOBIUS); CYTOPLASMIC_DOMAIN (PHOBIUS); TRANSMEMBRANE (PHOBIUS); CYTOPLASMIC_DOMAIN (PHOBIUS); TRANSMEMBRANE (PHOBIUS); TRANSMEMBRANE (PHOBIUS); IPR001254 (PROSITE_PROFILES); IPR009003 (SUPERFAMILY); TMhelix (TMHMM); TMhelix (TMHMM); TMhelix (TMHMM)
1539	490204_length_1781_cvg_68.8_tip_1_5	6700	cathepsin b	577	4.14E-164	78.80%	0.13	IPR000668 (PRINTS); IPR000668 (SMART); IPR012599 (PFAM); IPR000668 (PFAM); G3DSA:3.90.70.10 (GENE3D); IPR015643 (PTHR12411:PANTHER); IPR013128 (PANTHER); IPR025661 (PROSITE_PATTERNS); IPR025660 (PROSITE_PATTERNS); IPR000169 (PROSITE_PATTERNS); CYTOPLASMIC_DOMAIN (PHOBIUS); TRANSMEMBRANE (PHOBIUS); NON_CYTOPLASMIC_DOMAIN (PHOBIUS); CYTOPLASMIC_DOMAIN (PHOBIUS); TRANSMEMBRANE (PHOBIUS); SSF54001 (SUPERFAMILY); TMhelix (TMHMM); TMhelix (TMHMM)

1513	489590_length_1738_cvg_24.1_tip_1_0	208	zinc finger protein 227 isoform x1	580	2.22E-38	52.00%	0.109		IPR015880 (SMART); IPR013087 (G3DSA:3.30.160.GENE3D); IPR013087 (G3DSA:3.30.160.GENE3D); PF13894 (PFAM); IPR013087 (G3DSA:3.30.160.GENE3D); IPR007087 (PFAM); PF13465 (PFAM); PTHR24409:SF13 (PANTHER); PTHR24409 (PANTHER); IPR007087 (PROSITE_PATTERNS); IPR007087 (PROSITE_PATTERNS); IPR007087 (PROSITE_PATTERNS); IPR007087 (PROSITE_PATTERNS); IPR007087 (PROSITE_PATTERNS); IPR007087 (PROSITE_PATTERNS); IPR007087 (PROSITE_PATTERNS); IPR007087 (PROSITE_PROFILES); IPR007087 (PROSITE_PROFILES); IPR007087 (PROSITE_PROFILES); IPR007087 (PROSITE_PROFILES); IPR007087 (PROSITE_PROFILES); IPR007087 (PROSITE_PROFILES); IPR007087 (PROSITE_PROFILES); SSF57667 (SUPERFAMILY); SSF57667 (SUPERFAMILY); SSF57667 (SUPERFAMILY); SSF57667 (SUPERFAMILY)
1516	489612_length_1740_cvg_53.8_tip_1_5	526	ras-related protein rab-1a	580	1.99E-130	93.90%	0.155		IPR001806 (PRINTS); IPR003578 (SMART); IPR003579 (SMART); IPR002041 (SMART); IPR020849 (SMART); IPR024156 (SMART); IPR005225 (TIGRFAM); IPR027417 (G3DSA:3.40.50.GENE3D); IPR001806 (PFAM); PTHR24073:SF455 (PANTHER); PTHR24073 (PANTHER); NON_CYTOPLASMIC_DOMAIN (PHOBIUS); TRANSMEMBRANE (PHOBIUS); CYTOPLASMIC_DOMAIN (PHOBIUS); TRANSMEMBRANE (PHOBIUS); NON_CYTOPLASMIC_DOMAIN (PHOBIUS); PS51419 (PROSITE_PROFILES); IPR027417 (SUPERFAMILY); TMhelix (TMHMM); TMhelix (TMHMM)
1517	489634_length_1741_cvg_63.1_tip_1_1	1573	cytosolic non-specific dipeptidase	580	0	81.70%	0.127		IPR011650 (G3DSA:3.30.70.GENE3D); IPR011650 (PFAM); G3DSA:3.40.630.10 (GENE3D); IPR002933 (PFAM); PTHR11014:SF51 (PANTHER); PTHR11014 (PANTHER); IPR001261 (PROSITE_PATTERNS); CYTOPLASMIC_DOMAIN (PHOBIUS); NON_CYTOPLASMIC_DOMAIN (PHOBIUS); TRANSMEMBRANE (PHOBIUS); SSF53187 (SUPERFAMILY); TMhelix (TMHMM)
1542	490286_length_1787_cvg_51.5_tip_1_3	6584	phospholipid-hydroperoxide glutathione peroxidase	581	2.47E-81	80.70%	0.32		IPR000889 (PRINTS); IPR012336 (G3DSA:3.40.30.GENE3D); IPR000889 (PFAM); IPR000889 (PANTHER); IPR029760 (PROSITE_PATTERNS); CYTOPLASMIC_DOMAIN (PHOBIUS); SIGNAL_PEPTIDE_H_REGION (PHOBIUS); TRANSMEMBRANE (PHOBIUS); SIGNAL_PEPTIDE (PHOBIUS); CYTOPLASMIC_DOMAIN (PHOBIUS); NON_CYTOPLASMIC_DOMAIN (PHOBIUS); NON_CYTOPLASMIC_DOMAIN (PHOBIUS); TRANSMEMBRANE (PHOBIUS); SIGNAL_PEPTIDE_C_REGION (PHOBIUS); SIGNAL_PEPTIDE_N_REGION (PHOBIUS); TRANSMEMBRANE (PHOBIUS); IPR000889 (PROSITE_PROFILES); IPR012336 (SUPERFAMILY)
1518	489798_length_1753_cvg_16.4_tip_1_0	170	amine oxidase	585	6.89E-107	65.90%	0.121		IPR001613 (PRINTS); IPR002937 (PFAM); G3DSA:1.10.405.10 (GENE3D); G3DSA:3.90.660.10 (GENE3D); PTHR10742 (PANTHER); PTHR10742:SF253 (PANTHER); CYTOPLASMIC_DOMAIN (PHOBIUS); TRANSMEMBRANE (PHOBIUS); NON_CYTOPLASMIC_DOMAIN (PHOBIUS); SSF54373 (SUPERFAMILY); SSF51905 (SUPERFAMILY)
1519	489820_length_1754_cvg_5.3_tip_1_4	49	t-lymphocyte activation antigen cd86-like	585	8.96E-116	84.90%	0.258		IPR003599 (SMART); IPR013106 (SMART); IPR003598 (SMART); IPR013106 (PFAM); IPR013783 (G3DSA:2.60.40.GENE3D); PF13895 (PFAM); PTHR23279:SF1 (PANTHER); PTHR23279 (PANTHER); NON_CYTOPLASMIC_DOMAIN (PHOBIUS); TRANSMEMBRANE (PHOBIUS); CYTOPLASMIC_DOMAIN (PHOBIUS); IPR007110 (PROSITE_PROFILES); IPR007110 (PROSITE_PROFILES); SSF48726 (SUPERFAMILY); SSF48726 (SUPERFAMILY)
1521	489854_length_1756_cvg_8.1_tip_1_5	66	complement component q subcomponent-like protein	585	2.44E-35	60.80%	0.544	Y	IPR001073 (PRINTS); IPR001073 (SMART); IPR008983 (G3DSA:2.60.120.GENE3D); IPR001073 (PFAM); PTHR22923:SF51 (PANTHER); PTHR22923 (PANTHER); SIGNAL_PEPTIDE_N_REGION (PHOBIUS); SIGNAL_PEPTIDE_C_REGION (PHOBIUS); SIGNAL_PEPTIDE_H_REGION (PHOBIUS); NON_CYTOPLASMIC_DOMAIN (PHOBIUS); SIGNAL_PEPTIDE (PHOBIUS); IPR001073 (PROSITE_PROFILES); SignalP-noTM (SIGNALP_EUK); SignalP-TM (SIGNALP_GRAM_POSITIVE); IPR008983 (SUPERFAMILY); TMhelix (TMHMM); TMhelix (TMHMM); TMhelix (TMHMM)
1522	489882_length_1758_cvg_56.5_tip_1_5	803	nucleolar protein 56	586	0	87.50%	0.177		Coil (COILS); IPR012976 (SMART); IPR012974 (PFAM); IPR012976 (PFAM); IPR002687 (PFAM); PTHR10894:SF0 (PANTHER); PTHR10894 (PANTHER); IPR002687 (PROSITE_PROFILES); SSF89124 (SUPERFAMILY)

1523	489904_length_1760_cvg_25.2_tip_1_5	162	wd repeat-containing protein 61	586	1.16E-139	87.30%	0.103	IPR001680 (SMART); IPR001680 (PFAM); IPR015943 (G3DSA:2.130.10.GENE3D); PTHR22841 (PANTHER); CYTOPLASMIC_DOMAIN (PHOBIUS); TRANSMEMBRANE (PHOBIUS); NON_CYTOPLASMIC_DOMAIN (PHOBIUS); IPR001680 (PROSITE_PROFILES); IPR001680 (PROSITE_PROFILES); IPR017986 (PROSITE_PROFILES); IPR001680 (PROSITE_PROFILES); IPR001680 (PROSITE_PROFILES); IPR001680 (PROSITE_PROFILES); IPR017986 (SUPERFAMILY); TMhelix (TMHMM)
1528	490034_length_1768_cvg_5.4_tip_1_4	59	multiple inositol polyphosphate phosphatase 1	586	1.23E-71	59.50%	0.202	IPR029033 (G3DSA:3.40.50.GENE3D); IPR000560 (PFAM); PTHR20963 (PANTHER); PTHR20963:SF8 (PANTHER); TRANSMEMBRANE (PHOBIUS); TRANSMEMBRANE (PHOBIUS); CYTOPLASMIC_DOMAIN (PHOBIUS); NON_CYTOPLASMIC_DOMAIN (PHOBIUS); TRANSMEMBRANE (PHOBIUS); NON_CYTOPLASMIC_DOMAIN (PHOBIUS); CYTOPLASMIC_DOMAIN (PHOBIUS); IPR029033 (SUPERFAMILY); TMhelix (TMHMM); TMhelix (TMHMM)
1524	489922_length_1761_cvg_17.3_tip_1_2	154	von willebrand domain-containing protein	587	1.16E-120	54.40%	0.241	IPR002035 (G3DSA:3.40.50.GENE3D); PF13768 (PFAM); PTHR10338 (PANTHER); PTHR10338:SF95 (PANTHER); IPR002035 (PROSITE_PROFILES); IPR002035 (SUPERFAMILY)
1525	489930_length_1761_cvg_29.6_tip_1_0	231	factor d-like protein	587	2.01E-79	50.70%	0.108	IPR001314 (PRINTS); IPR001254 (SMART); IPR001254 (PFAM); G3DSA:2.40.10.10 (GENE3D); G3DSA:2.40.10.10 (GENE3D); G3DSA:2.40.10.10 (GENE3D); PTHR24268:SF95 (PANTHER); PTHR24268 (PANTHER); IPR018114 (PROSITE_PATTERNS); IPR001254 (PROSITE_PROFILES); IPR009003 (SUPERFAMILY)
1543	490318_length_1790_cvg_61.0_tip_1_2	713	atpase family aaa domain-containing protein 1-b	588	1.68E-164	77.60%	0.122	IPR003593 (SMART); G3DSA:1.10.8.60 (GENE3D); IPR027417 (G3DSA:3.40.50.GENE3D); IPR003959 (PFAM); PTHR23074 (PANTHER); PTHR23074:SF20 (PANTHER); IPR003960 (PROSITE_PATTERNS); CYTOPLASMIC_DOMAIN (PHOBIUS); NON_CYTOPLASMIC_DOMAIN (PHOBIUS); TRANSMEMBRANE (PHOBIUS); TRANSMEMBRANE (PHOBIUS); NON_CYTOPLASMIC_DOMAIN (PHOBIUS); TRANSMEMBRANE (PHOBIUS); CYTOPLASMIC_DOMAIN (PHOBIUS); IPR027417 (SUPERFAMILY)
1526	490004_length_1766_cvg_10.6_tip_1_0	78	von willebrand domain-containing protein	589	5.18E-90	61.20%	0.099	Coil (COILS); IPR013694 (SMART); IPR013694 (PFAM); PTHR10338 (PANTHER); PTHR10338:SF95 (PANTHER); NON_CYTOPLASMIC_DOMAIN (PHOBIUS); NON_CYTOPLASMIC_DOMAIN (PHOBIUS); CYTOPLASMIC_DOMAIN (PHOBIUS); TRANSMEMBRANE (PHOBIUS); TRANSMEMBRANE (PHOBIUS); CYTOPLASMIC_DOMAIN (PHOBIUS); CYTOPLASMIC_DOMAIN (PHOBIUS); TRANSMEMBRANE (PHOBIUS); TRANSMEMBRANE (PHOBIUS); IPR013694 (PROSITE_PROFILES); IPR002035 (SUPERFAMILY); TMhelix (TMHMM); TMhelix (TMHMM)
1527	490022_length_1767_cvg_56.8_tip_1_3	493	golgi-associated plant pathogenesis-related protein 1	589	2.39E-157	62.40%	0.103	IPR001283 (PRINTS); IPR014044 (SMART); IPR014044 (PFAM); IPR014044 (G3DSA:3.40.33.GENE3D); PTHR10334:SF189 (PANTHER); IPR001283 (PANTHER); IPR018244 (PROSITE_PATTERNS); IPR018244 (PROSITE_PATTERNS); IPR014044 (SUPERFAMILY); IPR014044 (SUPERFAMILY)
1545	490388_length_1795_cvg_9.4_tip_1_1	87	peptidyl-alpha-hydroxyglycine alpha-amidating lyase 2-like	590	1.18E-109	60.50%	0.208	IPR000720 (PRINTS); IPR011042 (G3DSA:2.120.10.GENE3D); IPR001258 (PFAM); PTHR10680 (PANTHER); IPR013017 (PROSITE_PROFILES); IPR013017 (PROSITE_PROFILES); IPR013017 (PROSITE_PROFILES); IPR013017 (PROSITE_PROFILES); SSF101898 (SUPERFAMILY)
1549	490454_length_1798_cvg_50.8_tip_1_3	517	prophenoloxidase activating factor	590	2.15E-131	65.60%	0.106	IPR001314 (PRINTS); IPR001254 (SMART); IPR001254 (PFAM); G3DSA:2.40.10.10 (GENE3D); G3DSA:2.40.10.10 (GENE3D); G3DSA:2.40.10.10 (GENE3D); PTHR24268 (PANTHER); PTHR24268:SF95 (PANTHER); IPR018114 (PROSITE_PATTERNS); TRANSMEMBRANE (PHOBIUS); CYTOPLASMIC_DOMAIN (PHOBIUS); TRANSMEMBRANE (PHOBIUS); NON_CYTOPLASMIC_DOMAIN (PHOBIUS); CYTOPLASMIC_DOMAIN (PHOBIUS); IPR001254 (PROSITE_PROFILES); IPR009003 (SUPERFAMILY); TMhelix (TMHMM); TMhelix (TMHMM)

1529	490066_length_1772_cvg_26.8_tip_1_0	217	zinc finger protein 850-like isoform x1	591	8.26E-49	55.10%	0.156	IPR015880 (SMART); PF13465 (PFAM); IPR013087 (G3DSA:3.30.160.GENE3D); IPR013087 (G3DSA:3.30.160.GENE3D); IPR007087 (PFAM); IPR013087 (G3DSA:3.30.160.GENE3D); IPR013087 (G3DSA:3.30.160.GENE3D); PF13894 (PFAM); PTHR24409:SF14 (PANTHER); PTHR24409 (PANTHER); IPR007087 (PROSITE_PATTERNS); IPR007087 (PROSITE_PATTERNS); IPR007087 (PROSITE_PATTERNS); IPR007087 (PROSITE_PATTERNS); IPR007087 (PROSITE_PATTERNS); IPR007087 (PROSITE_PATTERNS); IPR007087 (PROSITE_PATTERNS); IPR007087 (PROSITE_PATTERNS); IPR007087 (PROSITE_PATTERNS); IPR007087 (PROSITE_PROFILES); IPR007087 (PROSITE_PROFILES); IPR007087 (PROSITE_PROFILES); IPR007087 (PROSITE_PROFILES); IPR007087 (PROSITE_PROFILES); IPR007087 (PROSITE_PROFILES); IPR007087 (PROSITE_PROFILES); IPR007087 (PROSITE_PROFILES); IPR007087 (PROSITE_PROFILES); SSF57667 (SUPERFAMILY); SSF57667 (SUPERFAMILY); SSF57667 (SUPERFAMILY); SSF57667 (SUPERFAMILY); SSF57667 (SUPERFAMILY)
1530	490084_length_1773_cvg_9.9_tip_1_3	65	ikaros family zinc finger protein	591	3.28E-112	53.80%	0.13	IPR015880 (SMART); IPR013087 (G3DSA:3.30.160.GENE3D); PF13894 (PFAM); PF13465 (PFAM); IPR013087 (G3DSA:3.30.160.GENE3D); IPR013087 (G3DSA:3.30.160.GENE3D); PTHR24385 (PANTHER); PTHR24385:SF23 (PANTHER); IPR007087 (PROSITE_PATTERNS); IPR007087 (PROSITE_PATTERNS); IPR007087 (PROSITE_PATTERNS); IPR007087 (PROSITE_PATTERNS); IPR007087 (PROSITE_PROFILES); IPR007087 (PROSITE_PROFILES); IPR007087 (PROSITE_PROFILES); IPR007087 (PROSITE_PROFILES); SSF57667 (SUPERFAMILY); SSF57667 (SUPERFAMILY); SSF57667 (SUPERFAMILY)
1531	490098_length_1773_cvg_11.4_tip_1_1	85	insulin-like growth factor-binding protein complex acid labile subunit	591	9.08E-86	54.10%	0.224	SM00365 (SMART); IPR000483 (SMART); IPR003591 (SMART); IPR001611 (PFAM); G3DSA:3.80.10.10 (GENE3D); G3DSA:3.80.10.10 (GENE3D); IPR001611 (PFAM); PTHR24373 (PANTHER); TRANSMEMBRANE (PHOBIUS); CYTOPLASMIC_DOMAIN (PHOBIUS); NON_CYTOPLASMIC_DOMAIN (PHOBIUS); IPR001611 (PROSITE_PROFILES); IPR001611 (PROSITE_PROFILES); IPR001611 (PROSITE_PROFILES); IPR001611 (PROSITE_PROFILES); IPR001611 (PROSITE_PROFILES); IPR001611 (PROSITE_PROFILES); IPR001611 (PROSITE_PROFILES); IPR001611 (PROSITE_PROFILES); IPR001611 (PROSITE_PROFILES); IPR001611 (PROSITE_PROFILES); SSF52058 (SUPERFAMILY); TMhelix (TMHMM)
1532	490108_length_1774_cvg_76.9_tip_1_1	7794	peptidyl-prolyl cis-trans isomerase b	591	1.63E-106	86.10%	0.859 Y	IPR002130 (PRINTS); IPR029000 (G3DSA:2.40.100.GENE3D); IPR002130 (PFAM); PTHR11071:SF63 (PANTHER); IPR024936 (PANTHER); IPR020892 (PROSITE_PATTERNS); NON_CYTOPLASMIC_DOMAIN (PHOBIUS); CYTOPLASMIC_DOMAIN (PHOBIUS); NON_CYTOPLASMIC_DOMAIN (PHOBIUS); TRANSMEMBRANE (PHOBIUS); SIGNAL_PEPTIDE_N_REGION (PHOBIUS); SIGNAL_PEPTIDE (PHOBIUS); SIGNAL_PEPTIDE_C_REGION (PHOBIUS); TRANSMEMBRANE (PHOBIUS); SIGNAL_PEPTIDE_H_REGION (PHOBIUS); IPR002130 (PROSITE_PROFILES); SignalP-noTM (SIGNALP_EUK); IPR029000 (SUPERFAMILY)
1533	490112_length_1775_cvg_15.9_tip_1_2	152	aael003561- partial	591	6.84E-24	52.80%	0.159	IPR001211 (PRINTS); IPR016090 (SMART); IPR016090 (PFAM); IPR016090 (G3DSA:1.20.90.GENE3D); IPR001211 (PANTHER); PTHR11716:SF45 (PANTHER); IPR013090 (PROSITE_PATTERNS); IPR013090 (PROSITE_PATTERNS); NON_CYTOPLASMIC_DOMAIN (PHOBIUS); NON_CYTOPLASMIC_DOMAIN (PHOBIUS); CYTOPLASMIC_DOMAIN (PHOBIUS); TRANSMEMBRANE (PHOBIUS); CYTOPLASMIC_DOMAIN (PHOBIUS); TRANSMEMBRANE (PHOBIUS); TRANSMEMBRANE (PHOBIUS); IPR016090 (SUPERFAMILY); TMhelix (TMHMM)
1535	490114_length_1775_cvg_2.3_tip_1_2	20	zinc finger partial	591	2.93E-14	59.10%	0.114	IPR015880 (SMART); IPR013087 (G3DSA:3.30.160.GENE3D); PF13894 (PFAM); IPR013087 (G3DSA:3.30.160.GENE3D); IPR007087 (PFAM); PTHR24409 (PANTHER); PTHR24409:SF13 (PANTHER); IPR007087 (PROSITE_PATTERNS); IPR007087 (PROSITE_PATTERNS); IPR007087 (PROSITE_PATTERNS); NON_CYTOPLASMIC_DOMAIN (PHOBIUS); CYTOPLASMIC_DOMAIN (PHOBIUS); TRANSMEMBRANE (PHOBIUS); IPR007087 (PROSITE_PROFILES); IPR007087 (PROSITE_PROFILES); IPR007087 (PROSITE_PROFILES); SSF57667 (SUPERFAMILY); SSF57667 (SUPERFAMILY)
1550	490456_length_1798_cvg_37.0_tip_1_4	245	transitional endoplasmic reticulum atpase ter94	591	0	93.00%	0.114	IPR003593 (SMART); IPR027417 (G3DSA:3.40.50.GENE3D); G3DSA:1.10.8.60 (GENE3D); IPR003959 (PFAM); IPR027417 (G3DSA:3.40.50.GENE3D); IPR015415 (PFAM); PTHR23077 (PANTHER); PTHR23077:SF69 (PANTHER); IPR003960 (PROSITE_PATTERNS); IPR027417 (SUPERFAMILY)

1534	490114_length_1775_cvg_2.3_tip_1_1	20	zinc finger protein 37-like	592	6.09E-19	52.10%	0.101	IPR015880 (SMART); PF13894 (PFAM); IPR013087 (G3DSA:3.30.160.GENE3D); IPR013087 (G3DSA:3.30.160.GENE3D); IPR013087 (G3DSA:3.30.160.GENE3D); PF13465 (PFAM); PTHR24409 (PANTHER); IPR007087 (PROSITE_PATTERNS); IPR007087 (PROSITE_PATTERNS); IPR007087 (PROSITE_PATTERNS); IPR007087 (PROSITE_PATTERNS); IPR007087 (PROSITE_PROFILES); IPR007087 (PROSITE_PROFILES); IPR007087 (PROSITE_PROFILES); IPR007087 (PROSITE_PROFILES); SSF57667 (SUPERFAMILY); SSF57667 (SUPERFAMILY); SSF57667 (SUPERFAMILY)
1548	490430_length_1797_cvg_38.0_tip_1_3	394	superoxide dismutase	592	8.81E-62	68.70%	0.203	IPR001424 (PRINTS); IPR001424 (G3DSA:2.60.40.GENE3D); IPR001424 (G3DSA:2.60.40.GENE3D); IPR001424 (PFAM); PTHR10003 (PANTHER); IPR018152 (PROSITE_PATTERNS); IPR018152 (PROSITE_PATTERNS); NON_CYTOPLASMIC_DOMAIN (PHOBIUS); TRANSMEMBRANE (PHOBIUS); CYTOPLASMIC_DOMAIN (PHOBIUS); IPR001424 (SUPERFAMILY); IPR001424 (SUPERFAMILY); TMhelix (TMHMM)
1536	490156_length_1778_cvg_4.0_tip_1_1	39	fez family zinc finger protein 2	593	0	80.10%	0.103	Coil (COILS); IPR015880 (SMART); IPR007087 (PFAM); IPR013087 (G3DSA:3.30.160.GENE3D); PF13912 (PFAM); IPR013087 (G3DSA:3.30.160.GENE3D); PF13465 (PFAM); IPR013087 (G3DSA:3.30.160.GENE3D); PTHR24409 (PANTHER); IPR007087 (PROSITE_PATTERNS); IPR007087 (PROSITE_PATTERNS); IPR007087 (PROSITE_PATTERNS); IPR007087 (PROSITE_PATTERNS); IPR007087 (PROSITE_PATTERNS); IPR007087 (PROSITE_PATTERNS); IPR007087 (PROSITE_PATTERNS); IPR007087 (PROSITE_PROFILES); IPR007087 (PROSITE_PROFILES); IPR007087 (PROSITE_PROFILES); IPR007087 (PROSITE_PROFILES); IPR007087 (PROSITE_PROFILES); IPR007087 (PROSITE_PROFILES); SSF57667 (SUPERFAMILY); SSF57667 (SUPERFAMILY); SSF57667 (SUPERFAMILY)
1537	490162_length_1778_cvg_4.1_tip_1_4	28	cgmp-dependent protein isozyme 2 forms cd4 t1 t3a t3b isoform x2	593	0	90.20%	0.105	Coil (COILS); IPR002374 (PRINTS); IPR002290 (SMART); IPR000595 (SMART); G3DSA:1.10.510.10 (GENE3D); IPR000595 (PFAM); IPR000719 (PFAM); IPR014710 (G3DSA:2.60.120.GENE3D); IPR002374 (PIRSF); IPR014710 (G3DSA:2.60.120.GENE3D); G3DSA:3.30.200.20 (GENE3D); PTHR24353:SF65 (PANTHER); PTHR24353 (PANTHER); IPR017441 (PROSITE_PATTERNS); IPR008271 (PROSITE_PATTERNS); IPR018488 (PROSITE_PATTERNS); IPR018488 (PROSITE_PATTERNS); IPR018488 (PROSITE_PATTERNS); IPR018488 (PROSITE_PATTERNS); IPR000719 (PROSITE_PROFILES); IPR000595 (PROSITE_PROFILES); IPR000595 (PROSITE_PROFILES); IPR011009 (SUPERFAMILY); IPR018490 (SUPERFAMILY); IPR018490 (SUPERFAMILY)
1538	490178_length_1779_cvg_5.5_tip_1_4	46	cation channel sperm-associated protein 2	593	1.56E-72	54.60%	0.132	Coil (COILS); IPR005821 (PFAM); IPR027359 (G3DSA:1.20.120.GENE3D); PTHR10037 (PANTHER); IPR028747 (PTHR10037:PANTHER); NON_CYTOPLASMIC_DOMAIN (PHOBIUS); TRANSMEMBRANE (PHOBIUS); TRANSMEMBRANE (PHOBIUS); NON_CYTOPLASMIC_DOMAIN (PHOBIUS); CYTOPLASMIC_DOMAIN (PHOBIUS); CYTOPLASMIC_DOMAIN (PHOBIUS); TRANSMEMBRANE (PHOBIUS); TRANSMEMBRANE (PHOBIUS); TRANSMEMBRANE (PHOBIUS); CYTOPLASMIC_DOMAIN (PHOBIUS); TRANSMEMBRANE (PHOBIUS); NON_CYTOPLASMIC_DOMAIN (PHOBIUS); CYTOPLASMIC_DOMAIN (PHOBIUS); SSF81324 (SUPERFAMILY); TMhelix (TMHMM); TMhelix (TMHMM); TMhelix (TMHMM); TMhelix (TMHMM); TMhelix (TMHMM)
1553	490528_length_1803_cvg_19.4_tip_1_3	162	zinc finger protein 37 homolog isoform x1	593	2.31E-57	64.00%	0.132	IPR015880 (SMART); IPR000210 (SMART); IPR013087 (G3DSA:3.30.160.GENE3D); PF13465 (PFAM); IPR007087 (PFAM); IPR013087 (G3DSA:3.30.160.GENE3D); G3DSA:3.30.710.10 (GENE3D); IPR013087 (G3DSA:3.30.160.GENE3D); IPR013069 (PFAM); PTHR24409 (PANTHER); IPR007087 (PROSITE_PATTERNS); IPR007087 (PROSITE_PATTERNS); IPR007087 (PROSITE_PATTERNS); IPR007087 (PROSITE_PATTERNS); IPR007087 (PROSITE_PATTERNS); IPR007087 (PROSITE_PATTERNS); IPR007087 (PROSITE_PATTERNS); CYTOPLASMIC_DOMAIN (PHOBIUS); NON_CYTOPLASMIC_DOMAIN (PHOBIUS); TRANSMEMBRANE (PHOBIUS); IPR007087 (PROSITE_PROFILES); IPR007087 (PROSITE_PROFILES); IPR007087 (PROSITE_PROFILES); IPR007087 (PROSITE_PROFILES); IPR007087 (PROSITE_PROFILES); IPR007087 (PROSITE_PROFILES); IPR000210 (PROSITE_PROFILES); IPR007087 (PROSITE_PROFILES); SSF57667 (SUPERFAMILY); SSF57667 (SUPERFAMILY); SSF57667 (SUPERFAMILY); IPR011333 (SUPERFAMILY); SSF57667 (SUPERFAMILY); TMhelix (TMHMM)

1565	491000_length_1841_cvg_9.8_tip_1_4	126	puromycin-sensitive aminopeptidase	593	3.49E-101	80.00%	0.113	Coil (COILS); IPR024571 (PFAM); IPR001930 (PANTHER); PTHR11533:SF163 (PANTHER); NON_CYTOPLASMIC_DOMAIN (PHOBIUS); TRANSMEMBRANE (PHOBIUS); CYTOPLASMIC_DOMAIN (PHOBIUS); TRANSMEMBRANE (PHOBIUS); NON_CYTOPLASMIC_DOMAIN (PHOBIUS); TMhelix (TMHMM); TMhelix (TMHMM); TMhelix (TMHMM)
1540	490220_length_1783_cvg_6.1_tip_1_5	53	nitrilase and fragile histidine triad fusion protein hit	594	0	69.50%	0.112	Coil (COILS); IPR003010 (G3DSA:3.60.110.GENE3D); IPR001310 (PFAM); IPR011146 (G3DSA:3.30.428.GENE3D); IPR003010 (PFAM); PTHR23088:SF5 (PANTHER); PTHR23088 (PANTHER); IPR019808 (PROSITE_PATTERNS); IPR001110 (PROSITE_PATTERNS); IPR011146 (PROSITE_PROFILES); IPR003010 (PROSITE_PROFILES); IPR003010 (SUPERFAMILY); IPR011146 (SUPERFAMILY)
1541	490258_length_1785_cvg_21.6_tip_1_0	229	villin-1-like isoform x2	595	0	66.30%	0.1	IPR007122 (PRINTS); IPR003128 (SMART); IPR007122 (SMART); IPR029006 (G3DSA:3.40.20.GENE3D); IPR029006 (G3DSA:3.40.20.GENE3D); IPR007123 (PFAM); IPR029006 (G3DSA:3.40.20.GENE3D); IPR003128 (PFAM); IPR003128 (G3DSA:1.10.950.GENE3D); IPR029006 (G3DSA:3.40.20.GENE3D); IPR007122 (PANTHER); IPR003128 (PROSITE_PROFILES); SSF55753 (SUPERFAMILY); SSF55753 (SUPERFAMILY); SSF55753 (SUPERFAMILY); SSF82754 (SUPERFAMILY); IPR003128 (SUPERFAMILY)
1552	490520_length_1803_cvg_23.2_tip_1_2	206	lamin dm0-like isoform x1	596	1.17E-170	74.40%	0.148	Coil (COILS); Coil (COILS); Coil (COILS); Coil (COILS); G3DSA:1.20.5.170 (GENE3D); IPR001322 (G3DSA:2.60.40.GENE3D); IPR001322 (PFAM); IPR001664 (PFAM); IPR027696 (PTHR23239:PANTHER); IPR001664 (PANTHER); IPR018039 (PROSITE_PATTERNS); SSF64593 (SUPERFAMILY); IPR001322 (SUPERFAMILY)
1544	490344_length_1791_cvg_57.0_tip_1_5	877	n-acetylated-alpha-linked acidic dipeptidase	597	3.38E-143	59.60%	0.136	IPR007484 (PFAM); G3DSA:3.50.30.30 (GENE3D); IPR007365 (G3DSA:1.20.930.GENE3D); G3DSA:3.40.630.10 (GENE3D); IPR007365 (PFAM); PTHR10404 (PANTHER); TRANSMEMBRANE (PHOBIUS); CYTOPLASMIC_DOMAIN (PHOBIUS); NON_CYTOPLASMIC_DOMAIN (PHOBIUS); TRANSMEMBRANE (PHOBIUS); NON_CYTOPLASMIC_DOMAIN (PHOBIUS); SSF53187 (SUPERFAMILY); IPR007365 (SUPERFAMILY); SSF52025 (SUPERFAMILY)
1546	490422_length_1797_cvg_17.8_tip_1_2	180	acyl- delta desaturase	599	1.88E-128	72.50%	0.304	IPR015876 (PRINTS); IPR005804 (PFAM); PTHR11351 (PANTHER); PTHR11351:SF28 (PANTHER); TRANSMEMBRANE (PHOBIUS); CYTOPLASMIC_DOMAIN (PHOBIUS); TRANSMEMBRANE (PHOBIUS); TRANSMEMBRANE (PHOBIUS); TRANSMEMBRANE (PHOBIUS); TRANSMEMBRANE (PHOBIUS); TRANSMEMBRANE (PHOBIUS); TRANSMEMBRANE (PHOBIUS); NON_CYTOPLASMIC_DOMAIN (PHOBIUS); CYTOPLASMIC_DOMAIN (PHOBIUS); TRANSMEMBRANE (PHOBIUS); NON_CYTOPLASMIC_DOMAIN (PHOBIUS); CYTOPLASMIC_DOMAIN (PHOBIUS); CYTOPLASMIC_DOMAIN (PHOBIUS); NON_CYTOPLASMIC_DOMAIN (PHOBIUS); NON_CYTOPLASMIC_DOMAIN (PHOBIUS); CYTOPLASMIC_DOMAIN (PHOBIUS); TMhelix (TMHMM); TMhelix (TMHMM); TMhelix (TMHMM); TMhelix (TMHMM); TMhelix (TMHMM); TMhelix (TMHMM); TMhelix (TMHMM); TMhelix (TMHMM)
1547	490426_length_1797_cvg_14.2_tip_1_1	118	zinc finger protein	599	6.28E-32	53.10%	0.134	IPR015880 (SMART); IPR007087 (PFAM); PF13894 (PFAM); IPR013087 (G3DSA:3.30.160.GENE3D); IPR013087 (G3DSA:3.30.160.GENE3D); PF13465 (PFAM); PTHR24409:SF14 (PANTHER); PTHR24409 (PANTHER); IPR007087 (PROSITE_PATTERNS); IPR007087 (PROSITE_PATTERNS); IPR007087 (PROSITE_PATTERNS); IPR007087 (PROSITE_PATTERNS); IPR007087 (PROSITE_PATTERNS); IPR007087 (PROSITE_PATTERNS); TRANSMEMBRANE (PHOBIUS); CYTOPLASMIC_DOMAIN (PHOBIUS); NON_CYTOPLASMIC_DOMAIN (PHOBIUS); IPR007087 (PROSITE_PROFILES); IPR007087 (PROSITE_PROFILES); IPR007087 (PROSITE_PROFILES); IPR007087 (PROSITE_PROFILES); IPR007087 (PROSITE_PROFILES); IPR007087 (PROSITE_PROFILES); SSF57667 (SUPERFAMILY); SSF57667 (SUPERFAMILY); SSF57667 (SUPERFAMILY)
1559	490836_length_1826_cvg_28.3_tip_1_5	233	c protein immunoglobulin-a-binding beta antigen	599	8.34E-33	56.40%	0.298	G3DSA:2.160.10.10 (GENE3D); TRANSMEMBRANE (PHOBIUS); CYTOPLASMIC_DOMAIN (PHOBIUS); NON_CYTOPLASMIC_DOMAIN (PHOBIUS); TRANSMEMBRANE (PHOBIUS); NON_CYTOPLASMIC_DOMAIN (PHOBIUS)
1551	490470_length_1799_cvg_8.8_tip_1_1	96	plexin domain-containing protein 2-like	600	3.99E-117	70.40%	0.335	IPR016201 (SMART); G3DSA:3.30.1680.10 (GENE3D); PTHR13055 (PANTHER); PTHR13055:SF12 (PANTHER)
1554	490552_length_1805_cvg_2.2_tip_1_1	27	hypothetical protein BRAFLDRAFT_125057	602	3.29E-12	47.10%	0.107	no IPS match

1555	490576_length_1807_cvg_4.9_tip_1_4	50	tripartite motif-containing protein 2-like isoform x5	602	9.84E-155	85.60%	0.116	IPR001258 (PFAM); IPR011042 (G3DSA:2.120.10.GENE3D); PTHR24103 (PANTHER); PTHR24103:SF1 (PANTHER); TRANSMEMBRANE (PHOBIUS); NON_CYTOPLASMIC_DOMAIN (PHOBIUS); NON_CYTOPLASMIC_DOMAIN (PHOBIUS); TRANSMEMBRANE (PHOBIUS); CYTOPLASMIC_DOMAIN (PHOBIUS); TRANSMEMBRANE (PHOBIUS); NON_CYTOPLASMIC_DOMAIN (PHOBIUS); CYTOPLASMIC_DOMAIN (PHOBIUS); CYTOPLASMIC_DOMAIN (PHOBIUS); TRANSMEMBRANE (PHOBIUS); TRANSMEMBRANE (PHOBIUS); IPR013017 (PROSITE_PROFILES); IPR013017 (PROSITE_PROFILES); IPR013017 (PROSITE_PROFILES); IPR013017 (PROSITE_PROFILES); IPR013017 (PROSITE_PROFILES); IPR013017 (PROSITE_PROFILES); SSF101898 (SUPERFAMILY); TMhelix (TMHMM); TMhelix (TMHMM)
1563	490958_length_1837_cvg_6.6_tip_1_3	60	abl interactor 2-like	604	2.81E-24	89.00%	0.137	IPR001452 (PRINTS); IPR001452 (SMART); IPR001452 (PFAM); G3DSA:2.30.30.40 (GENE3D); IPR028457 (PANTHER); TRANSMEMBRANE (PHOBIUS); CYTOPLASMIC_DOMAIN (PHOBIUS); NON_CYTOPLASMIC_DOMAIN (PHOBIUS); TRANSMEMBRANE (PHOBIUS); TRANSMEMBRANE (PHOBIUS); NON_CYTOPLASMIC_DOMAIN (PHOBIUS); CYTOPLASMIC_DOMAIN (PHOBIUS); TRANSMEMBRANE (PHOBIUS); NON_CYTOPLASMIC_DOMAIN (PHOBIUS); TRANSMEMBRANE (PHOBIUS); CYTOPLASMIC_DOMAIN (PHOBIUS); IPR001452 (PROSITE_PROFILES); IPR001452 (SUPERFAMILY); TMhelix (TMHMM); TMhelix (TMHMM); TMhelix (TMHMM); TMhelix (TMHMM)
1556	490716_length_1818_cvg_4.1_tip_1_2	37	aael017480- partial	606	1.98E-44	55.20%	0.171	IPR002110 (PRINTS); IPR002110 (SMART); IPR020683 (G3DSA:1.25.40.GENE3D); IPR020683 (G3DSA:1.25.40.GENE3D); PF13637 (PFAM); IPR020683 (G3DSA:1.25.40.GENE3D); IPR020683 (PFAM); PTHR24198 (PANTHER); IPR002110 (PROSITE_PROFILES); IPR002110 (PROSITE_PROFILES); IPR020683 (PROSITE_PROFILES); IPR002110 (PROSITE_PROFILES); IPR002110 (PROSITE_PROFILES); IPR002110 (PROSITE_PROFILES); IPR002110 (PROSITE_PROFILES); IPR020683 (SUPERFAMILY)
1567	491014_length_1843_cvg_48.3_tip_1_0	486	reticulocalbin-2	607	5.53E-131	73.80%	0.165	IPR002048 (SMART); IPR011992 (PFAM); IPR011992 (G3DSA:1.10.238.GENE3D); PTHR10827:SF48 (PANTHER); PTHR10827 (PANTHER); IPR018247 (PROSITE_PATTERNS); IPR018247 (PROSITE_PATTERNS); IPR018247 (PROSITE_PATTERNS); IPR018247 (PROSITE_PATTERNS); IPR018247 (PROSITE_PATTERNS); IPR018247 (PROSITE_PATTERNS); NON_CYTOPLASMIC_DOMAIN (PHOBIUS); NON_CYTOPLASMIC_DOMAIN (PHOBIUS); TRANSMEMBRANE (PHOBIUS); TRANSMEMBRANE (PHOBIUS); CYTOPLASMIC_DOMAIN (PHOBIUS); TRANSMEMBRANE (PHOBIUS); CYTOPLASMIC_DOMAIN (PHOBIUS); IPR002048 (PROSITE_PROFILES); IPR002048 (PROSITE_PROFILES); IPR002048 (PROSITE_PROFILES); IPR002048 (PROSITE_PROFILES); IPR002048 (PROSITE_PROFILES); IPR002048 (PROSITE_PROFILES); SSF47473 (SUPERFAMILY); SSF47473 (SUPERFAMILY); TMhelix (TMHMM); TMhelix (TMHMM)
1557	490808_length_1824_cvg_11.0_tip_1_1	88	low quality protein: zinc finger protein 782	608	2.44E-55	50.50%	0.176	IPR015880 (SMART); PF13912 (PFAM); PF13465 (PFAM); IPR013087 (G3DSA:3.30.160.GENE3D); IPR007087 (PFAM); PF13894 (PFAM); IPR013087 (G3DSA:3.30.160.GENE3D); IPR013087 (G3DSA:3.30.160.GENE3D); PTHR24409 (PANTHER); PTHR24409:SF13 (PANTHER); IPR007087 (PROSITE_PATTERNS); IPR007087 (PROSITE_PATTERNS); IPR007087 (PROSITE_PATTERNS); IPR007087 (PROSITE_PATTERNS); IPR007087 (PROSITE_PATTERNS); IPR007087 (PROSITE_PATTERNS); IPR007087 (PROSITE_PATTERNS); IPR007087 (PROSITE_PATTERNS); IPR007087 (PROSITE_PATTERNS); CYTOPLASMIC_DOMAIN (PHOBIUS); NON_CYTOPLASMIC_DOMAIN (PHOBIUS); TRANSMEMBRANE (PHOBIUS); IPR007087 (PROSITE_PROFILES); IPR007087 (PROSITE_PROFILES); IPR007087 (PROSITE_PROFILES); IPR007087 (PROSITE_PROFILES); IPR007087 (PROSITE_PROFILES); IPR007087 (PROSITE_PROFILES); IPR007087 (PROSITE_PROFILES); IPR007087 (PROSITE_PROFILES); IPR007087 (PROSITE_PROFILES); SSF57667 (SUPERFAMILY); SSF57667 (SUPERFAMILY); SSF57667 (SUPERFAMILY); SSF57667 (SUPERFAMILY); SSF57667 (SUPERFAMILY)
1558	490816_length_1824_cvg_12.7_tip_1_4	119	laminin subunit alpha	608	1.93E-132	54.60%	0.12	IPR001791 (SMART); IPR013320 (G3DSA:2.60.120.GENE3D); IPR013320 (G3DSA:2.60.120.GENE3D); IPR001791 (PFAM); PTHR10574:SF39 (PANTHER); PTHR10574 (PANTHER); IPR001791 (PROSITE_PROFILES); IPR001791 (PROSITE_PROFILES); IPR001791 (PROSITE_PROFILES); IPR013320 (SUPERFAMILY); IPR013320 (SUPERFAMILY); IPR013320 (SUPERFAMILY)

1572	491170_length_1854_cvg_46.4_tip_1_3	519	upf0764 protein c16orf89 homolog	608	2.58E-46	47.50%	0.264	TRANSMEMBRANE (PHOBIUS); CYTOPLASMIC_DOMAIN (PHOBIUS); CYTOPLASMIC_DOMAIN (PHOBIUS); TRANSMEMBRANE (PHOBIUS); NON_CYTOPLASMIC_DOMAIN (PHOBIUS); TMhelix (TMHMM); TMhelix (TMHMM)
1560	490860_length_1828_cvg_12.2_tip_1_0	86	zinc finger protein 420-like isoform x1	610	1.09E-53	55.50%	0.244	IPR015880 (SMART); IPR013087 (G3DSA:3.30.160.GENE3D); PF13465 (PFAM); IPR007087 (PFAM); IPR013087 (G3DSA:3.30.160.GENE3D); PF13894 (PFAM); PTHR24409 (PANTHER); PTHR24409:SF14 (PANTHER); IPR007087 (PROSITE_PATTERNS); IPR007087 (PROSITE_PATTERNS); IPR007087 (PROSITE_PATTERNS); IPR007087 (PROSITE_PATTERNS); IPR007087 (PROSITE_PATTERNS); IPR007087 (PROSITE_PATTERNS); IPR007087 (PROSITE_PATTERNS); IPR007087 (PROSITE_PATTERNS); IPR007087 (PROSITE_PATTERNS); IPR007087 (PROSITE_PROFILES); IPR007087 (PROSITE_PROFILES); IPR007087 (PROSITE_PROFILES); IPR007087 (PROSITE_PROFILES); IPR007087 (PROSITE_PROFILES); IPR007087 (PROSITE_PROFILES); IPR007087 (PROSITE_PROFILES); IPR007087 (PROSITE_PROFILES); IPR007087 (PROSITE_PROFILES); SSF57667 (SUPERFAMILY); SSF57667 (SUPERFAMILY); SSF57667 (SUPERFAMILY); SSF57667 (SUPERFAMILY); SSF57667 (SUPERFAMILY)
1577	491216_length_1858_cvg_61.4_tip_1_5	4050	polyubiquitin-c	610	0	99.60%	0.104	IPR019956 (PRINTS); IPR000626 (SMART); IPR000626 (PFAM); G3DSA:3.10.20.90 (GENE3D); G3DSA:3.10.20.90 (GENE3D); PTHR10666 (PANTHER); PTHR10666:SF112 (PANTHER); IPR019954 (PROSITE_PATTERNS); IPR019954 (PROSITE_PATTERNS); IPR019954 (PROSITE_PATTERNS); IPR019954 (PROSITE_PATTERNS); IPR019954 (PROSITE_PATTERNS); TRANSMEMBRANE (PHOBIUS); NON_CYTOPLASMIC_DOMAIN (PHOBIUS); CYTOPLASMIC_DOMAIN (PHOBIUS); NON_CYTOPLASMIC_DOMAIN (PHOBIUS); CYTOPLASMIC_DOMAIN (PHOBIUS); TRANSMEMBRANE (PHOBIUS); TRANSMEMBRANE (PHOBIUS); IPR000626 (PROSITE_PROFILES); IPR000626 (PROSITE_PROFILES); IPR000626 (PROSITE_PROFILES); IPR000626 (PROSITE_PROFILES); IPR000626 (PROSITE_PROFILES); IPR000626 (PROSITE_PROFILES); IPR029071 (SUPERFAMILY); IPR029071 (SUPERFAMILY); IPR029071 (SUPERFAMILY); IPR029071 (SUPERFAMILY); IPR029071 (SUPERFAMILY); IPR029071 (SUPERFAMILY); TMhelix (TMHMM); TMhelix (TMHMM); TMhelix (TMHMM); TMhelix (TMHMM)
1561	490920_length_1833_cvg_10.8_tip_1_0	103	periodic tryptophan protein 2 homolog	611	0	79.00%	0.114	IPR001680 (SMART); IPR007148 (PFAM); IPR015943 (G3DSA:2.130.10.GENE3D); IPR015943 (G3DSA:2.130.10.GENE3D); IPR001680 (PFAM); IPR027145 (PANTHER); IPR019775 (PROSITE_PATTERNS); IPR019775 (PROSITE_PATTERNS); IPR017986 (PROSITE_PROFILES); IPR001680 (PROSITE_PROFILES); IPR001680 (PROSITE_PROFILES); IPR001680 (PROSITE_PROFILES); IPR011047 (SUPERFAMILY)
1578	491236_length_1860_cvg_62.1_tip_1_3	2733	adp-ribosylation factor 2	612	1.90E-103	95.70%	0.115	IPR006689 (PRINTS); IPR024156 (SMART); IPR006687 (SMART); IPR006689 (PFAM); IPR005225 (TIGRFAM); IPR027417 (G3DSA:3.40.50.GENE3D); PTHR11711:SF153 (PANTHER); PTHR11711 (PANTHER); NON_CYTOPLASMIC_DOMAIN (PHOBIUS); TRANSMEMBRANE (PHOBIUS); CYTOPLASMIC_DOMAIN (PHOBIUS); TRANSMEMBRANE (PHOBIUS); NON_CYTOPLASMIC_DOMAIN (PHOBIUS); CYTOPLASMIC_DOMAIN (PHOBIUS); CYTOPLASMIC_DOMAIN (PHOBIUS); NON_CYTOPLASMIC_DOMAIN (PHOBIUS); TRANSMEMBRANE (PHOBIUS); TRANSMEMBRANE (PHOBIUS); TRANSMEMBRANE (PHOBIUS); IPR024156 (PROSITE_PROFILES); IPR027417 (SUPERFAMILY); TMhelix (TMHMM); TMhelix (TMHMM); TMhelix (TMHMM); TMhelix (TMHMM)
1562	490950_length_1837_cvg_47.1_tip_1_3	332	low quality protein: otogelin	613	1.08E-15	47.40%	0.1	PD968187 (PRODOM)
1564	490994_length_1840_cvg_4.8_tip_1_1	49	protein partial	613	0	100.00%	0.103	IPR006530 (TIGRFAM); IPR006530 (PFAM); PTHR32305:SF6 (PANTHER); PTHR32305 (PANTHER)
1566	491010_length_1842_cvg_14.3_tip_1_5	107	probable serine carboxypeptidase cpvl	614	6.27E-179	71.80%	0.236	IPR001563 (PRINTS); IPR029058 (G3DSA:3.40.50.GENE3D); IPR001563 (PFAM); PTHR11802:SF30 (PANTHER); IPR001563 (PANTHER); IPR018202 (PROSITE_PATTERNS); IPR018202 (PROSITE_PATTERNS); NON_CYTOPLASMIC_DOMAIN (PHOBIUS); TRANSMEMBRANE (PHOBIUS); TRANSMEMBRANE (PHOBIUS); CYTOPLASMIC_DOMAIN (PHOBIUS); NON_CYTOPLASMIC_DOMAIN (PHOBIUS); IPR029058 (SUPERFAMILY); TMhelix (TMHMM)

1568	491080_length_1847_cvg_3.9_tip_1_5	38	chitinase 1 precursor	615	0	70.50%	0.128		IPR002557 (SMART); IPR011583 (SMART); IPR002557 (G3DSA:2.170.140.GENE3D); IPR029070 (G3DSA:3.10.50.GENE3D); IPR013781 (G3DSA:3.20.20.GENE3D); IPR001223 (PFAM); IPR002557 (PFAM); PTHR11177 (PANTHER); PTHR11177:SF144 (PANTHER); IPR002557 (PROSITE_PROFILES); IPR017853 (SUPERFAMILY); IPR029070 (SUPERFAMILY); IPR002557 (SUPERFAMILY)
1583	491356_length_1872_cvg_15.8_tip_1_1	174	a disintegrin and metalloproteinase with thrombospondin motifs 18	615	1.58E-60	55.10%	0.112		IPR006586 (SMART); IPR001590 (PFAM); IPR024079 (G3DSA:3.40.390.GENE3D); PTHR13723 (PANTHER); IPR001590 (PROSITE_PROFILES); SSF55486 (SUPERFAMILY)
1569	491124_length_1851_cvg_14.5_tip_1_2	135	rna-binding protein 5-like	617	4.61E-151	64.90%	0.106		Coil (COILS); IPR000467 (SMART); IPR000467 (PFAM); PTHR13948 (PANTHER); IPR000467 (PROSITE_PROFILES); IPR007087 (PROSITE_PROFILES)
1570	491130_length_1852_cvg_12.4_tip_1_4	101	secreted frizzled-related protein 3	617	1.01E-78	59.70%	0.254		Coil (COILS); IPR020067 (SMART); IPR018933 (SMART); G3DSA:2.40.50.120 (GENE3D); IPR020067 (PFAM); IPR020067 (G3DSA:1.10.2000.GENE3D); IPR018933 (PFAM); PTHR11309:SF83 (PANTHER); IPR015526 (PANTHER); SIGNAL_PEPTIDE_H_REGION (PHOBIUS); SIGNAL_PEPTIDE_N_REGION (PHOBIUS); SIGNAL_PEPTIDE_C_REGION (PHOBIUS); SIGNAL_PEPTIDE (PHOBIUS); NON_CYTOPLASMIC_DOMAIN (PHOBIUS); IPR020067 (PROSITE_PROFILES); IPR001134 (PROSITE_PROFILES); IPR008993 (SUPERFAMILY); IPR020067 (SUPERFAMILY)
1571	491144_length_1852_cvg_5.1_tip_1_4	58	flavin-containing monooxygenase fmo gs-ox3	617	2.52E-141	64.30%	0.273		IPR000960 (PRINTS); G3DSA:3.50.50.60 (GENE3D); G3DSA:3.50.50.60 (GENE3D); IPR020946 (PFAM); PTHR23023 (PANTHER); PTHR23023:SF4 (PANTHER); SSF51905 (SUPERFAMILY); SSF51905 (SUPERFAMILY)
1579	491274_length_1864_cvg_57.3_tip_1_3	812	tribbles homolog 2	617	1.53E-121	71.70%	0.11		IPR002290 (SMART); IPR000719 (PFAM); G3DSA:3.30.200.20 (GENE3D); G3DSA:1.10.510.10 (GENE3D); PTHR22961:SF4 (PANTHER); IPR024104 (PANTHER); CYTOPLASMIC_DOMAIN (PHOBIUS); NON_CYTOPLASMIC_DOMAIN (PHOBIUS); TRANSMEMBRANE (PHOBIUS); IPR000719 (PROSITE_PROFILES); IPR011009 (SUPERFAMILY)
1573	491178_length_1855_cvg_5.5_tip_1_3	47	dual specificity mitogen-activated protein kinase kinase 4-like	619	0	89.90%	0.117		IPR002290 (SMART); G3DSA:3.30.200.20 (GENE3D); IPR000719 (PFAM); G3DSA:1.10.510.10 (GENE3D); PTHR24360:SF46 (PANTHER); PTHR24360 (PANTHER); IPR008271 (PROSITE_PATTERNS); IPR017441 (PROSITE_PATTERNS); IPR000719 (PROSITE_PROFILES); IPR011009 (SUPERFAMILY)
1574	491208_length_1857_cvg_8.9_tip_1_4	104	scp-like extracellular domain containing protein 1	619	1.91E-69	60.30%	0.237		IPR002413 (PRINTS); IPR001283 (PRINTS); IPR014044 (SMART); IPR014044 (G3DSA:3.40.33.GENE3D); IPR014044 (PFAM); PTHR10334:SF174 (PANTHER); IPR001283 (PANTHER); IPR018244 (PROSITE_PATTERNS); IPR018244 (PROSITE_PATTERNS); NON_CYTOPLASMIC_DOMAIN (PHOBIUS); TRANSMEMBRANE (PHOBIUS); TRANSMEMBRANE (PHOBIUS); NON_CYTOPLASMIC_DOMAIN (PHOBIUS); CYTOPLASMIC_DOMAIN (PHOBIUS); IPR000998 (PROSITE_PROFILES); IPR013320 (SUPERFAMILY); IPR014044 (SUPERFAMILY); TMhelix (TMHMM)
1576	491210_length_1858_cvg_7.9_tip_1_2	79	#NAME?	619	5.14E-46	71.60%	0.215		IPR001830 (PFAM); G3DSA:3.40.50.2000 (GENE3D); PTHR10788:SF6 (PANTHER); PTHR10788 (PANTHER); TRANSMEMBRANE (PHOBIUS); NON_CYTOPLASMIC_DOMAIN (PHOBIUS); CYTOPLASMIC_DOMAIN (PHOBIUS); NON_CYTOPLASMIC_DOMAIN (PHOBIUS); TRANSMEMBRANE (PHOBIUS); TRANSMEMBRANE (PHOBIUS); CYTOPLASMIC_DOMAIN (PHOBIUS); CYTOPLASMIC_DOMAIN (PHOBIUS); TRANSMEMBRANE (PHOBIUS); SSF53756 (SUPERFAMILY); TMhelix (TMHMM); TMhelix (TMHMM); TMhelix (TMHMM)
1575	491210_length_1858_cvg_7.9_tip_1_0	79	trehalose 6-phosphate synthase 1	620	4.53E-28	68.90%	0.364	Y	IPR001830 (PFAM); G3DSA:3.40.50.2000 (GENE3D); G3DSA:3.40.50.2000 (GENE3D); PTHR10788:SF6 (PANTHER); PTHR10788 (PANTHER); SIGNAL_PEPTIDE_N_REGION (PHOBIUS); NON_CYTOPLASMIC_DOMAIN (PHOBIUS); SIGNAL_PEPTIDE_C_REGION (PHOBIUS); SIGNAL_PEPTIDE (PHOBIUS); SIGNAL_PEPTIDE_H_REGION (PHOBIUS); SSF53756 (SUPERFAMILY)
1593	491566_length_1895_cvg_10.1_tip_1_5	84	transposable element tcb1 partial	621	6.00E-25	60.70%	0.122		IPR002172 (PRINTS); IPR002172 (SMART); IPR002172 (G3DSA:4.10.400.GENE3D); IPR002172 (PFAM); IPR002172 (G3DSA:4.10.400.GENE3D); PTHR10529 (PANTHER); IPR023415 (PROSITE_PATTERNS); IPR023415 (PROSITE_PATTERNS); IPR002172 (PROSITE_PROFILES); IPR002172 (PROSITE_PROFILES); IPR002172 (SUPERFAMILY); IPR002172 (SUPERFAMILY)

1595	491592_length_1897_cvg_31.6_tip_1_2	271	low quality protein: hemocytin-like	621	4.77E-75	53.50%	0.114	IPR006207 (SMART); IPR006208 (PFAM); PTHR11339:SF229 (PANTHER); PTHR11339 (PANTHER); TRANSMEMBRANE (PHOBIUS); CYTOPLASMIC_DOMAIN (PHOBIUS); NON_CYTOPLASMIC_DOMAIN (PHOBIUS); NON_CYTOPLASMIC_DOMAIN (PHOBIUS); TRANSMEMBRANE (PHOBIUS); CYTOPLASMIC_DOMAIN (PHOBIUS); CYTOPLASMIC_DOMAIN (PHOBIUS); TRANSMEMBRANE (PHOBIUS); TRANSMEMBRANE (PHOBIUS); IPR006207 (PROSITE_PROFILES); SSF57603 (SUPERFAMILY); SSF57603 (SUPERFAMILY)
1580	491326_length_1869_cvg_6.1_tip_1_5	51	insulin-degrading enzyme	623	2.93E-178	72.80%	0.113	IPR007863 (PFAM); IPR011237 (G3DSA:3.30.830.GENE3D); IPR011237 (G3DSA:3.30.830.GENE3D); PTHR11851:SF85 (PANTHER); PTHR11851 (PANTHER); IPR011249 (SUPERFAMILY); IPR011249 (SUPERFAMILY)
1581	491342_length_1871_cvg_27.1_tip_1_4	453904	suppressor of tumorigenicity 14 protein	624	2.70E-51	52.30%	0.126	IPR001314 (PRINTS); IPR002172 (SMART); IPR001254 (SMART); IPR002172 (PFAM); G3DSA:2.40.10.10 (GENE3D); IPR001254 (PFAM); IPR002172 (G3DSA:4.10.400.GENE3D); G3DSA:2.40.10.10 (GENE3D); G3DSA:2.40.10.10 (GENE3D); PTHR24256 (PANTHER); IPR018114 (PROSITE_PATTERNS); IPR018114 (PROSITE_PATTERNS); IPR023415 (PROSITE_PATTERNS); TRANSMEMBRANE (PHOBIUS); NON_CYTOPLASMIC_DOMAIN (PHOBIUS); CYTOPLASMIC_DOMAIN (PHOBIUS); IPR001254 (PROSITE_PROFILES); IPR002172 (PROSITE_PROFILES); IPR009003 (SUPERFAMILY); IPR002172 (SUPERFAMILY)
1582	491352_length_1872_cvg_30.3_tip_1_1	311	angiotensin-converting enzyme	624	0	78.10%	0.133	IPR001548 (PRINTS); IPR001548 (PFAM); PTHR10514:SF17 (PANTHER); IPR001548 (PANTHER); TRANSMEMBRANE (PHOBIUS); CYTOPLASMIC_DOMAIN (PHOBIUS); NON_CYTOPLASMIC_DOMAIN (PHOBIUS); CYTOPLASMIC_DOMAIN (PHOBIUS); TRANSMEMBRANE (PHOBIUS); SSF55486 (SUPERFAMILY); TMhelix (TMHMM); TMhelix (TMHMM)
1596	491594_length_1897_cvg_41.0_tip_1_5	360	probable cytochrome p450 49a1	624	6.74E-137	60.50%	0.146	IPR002401 (PRINTS); IPR001128 (PRINTS); IPR001128 (G3DSA:1.10.630.GENE3D); IPR001128 (PFAM); PTHR24305 (PANTHER); PTHR24305:SF49 (PANTHER); IPR017972 (PROSITE_PATTERNS); NON_CYTOPLASMIC_DOMAIN (PHOBIUS); CYTOPLASMIC_DOMAIN (PHOBIUS); TRANSMEMBRANE (PHOBIUS); CYTOPLASMIC_DOMAIN (PHOBIUS); TRANSMEMBRANE (PHOBIUS); IPR001128 (SUPERFAMILY); TMhelix (TMHMM); TMhelix (TMHMM)
1584	491412_length_1879_cvg_4.8_tip_1_2	60	wd repeat-containing protein 37	626	0	77.10%	0.12	IPR020472 (PRINTS); IPR001680 (SMART); IPR015943 (G3DSA:2.130.10.GENE3D); IPR001680 (PFAM); IPR015943 (G3DSA:2.130.10.GENE3D); PTHR19855:SF12 (PANTHER); PTHR19855 (PANTHER); IPR019775 (PROSITE_PATTERNS); IPR001680 (PROSITE_PROFILES); IPR001680 (PROSITE_PROFILES); IPR001680 (PROSITE_PROFILES); IPR001680 (PROSITE_PROFILES); IPR017986 (PROSITE_PROFILES); IPR017986 (SUPERFAMILY)
1585	491418_length_1879_cvg_41.5_tip_1_4	361	serine threonine-protein kinase grp-like	626	0	74.50%	0.129	IPR002290 (SMART); IPR000719 (PFAM); G3DSA:3.30.200.20 (GENE3D); G3DSA:1.10.510.10 (GENE3D); PTHR24344 (PANTHER); IPR017441 (PROSITE_PATTERNS); IPR008271 (PROSITE_PATTERNS); TRANSMEMBRANE (PHOBIUS); NON_CYTOPLASMIC_DOMAIN (PHOBIUS); CYTOPLASMIC_DOMAIN (PHOBIUS); IPR000719 (PROSITE_PROFILES); IPR011009 (SUPERFAMILY); TMhelix (TMHMM)
1586	491458_length_1883_cvg_9.4_tip_1_1	78	serine threonine-protein kinase osr1 isoform x2	628	0	83.30%	0.111	IPR002290 (SMART); G3DSA:1.10.510.10 (GENE3D); G3DSA:3.10.20.90 (GENE3D); G3DSA:3.30.200.20 (GENE3D); IPR000719 (PFAM); IPR024678 (PFAM); PTHR24361:SF201 (PANTHER); PTHR24361 (PANTHER); IPR017441 (PROSITE_PATTERNS); IPR000719 (PROSITE_PROFILES); IPR011009 (SUPERFAMILY)
1587	491470_length_1884_cvg_5.4_tip_1_1	54	97 kda heat shock	628	0	78.90%	0.126	IPR013126 (PRINTS); G3DSA:3.30.420.40 (GENE3D); G3DSA:3.90.640.10 (GENE3D); IPR029047 (G3DSA:2.60.34.GENE3D); G3DSA:3.30.30.30 (GENE3D); IPR013126 (PFAM); G3DSA:3.30.420.40 (GENE3D); PTHR19375:SF78 (PANTHER); PTHR19375 (PANTHER); IPR018181 (PROSITE_PATTERNS); SSF53067 (SUPERFAMILY); SSF53067 (SUPERFAMILY); IPR029047 (SUPERFAMILY)
1588	491480_length_1885_cvg_31.5_tip_1_2	248	carbohydrate sulfotransferase 11	628	5.95E-30	49.40%	0.098	IPR005331 (PFAM); IPR018011 (PANTHER); TRANSMEMBRANE (PHOBIUS); NON_CYTOPLASMIC_DOMAIN (PHOBIUS); CYTOPLASMIC_DOMAIN (PHOBIUS); TMhelix (TMHMM)

1589	491492_length_1886_cvg_14.4_tip_1_0	113	zinc finger protein 850-like isoform x1	629	4.94E-43	58.90%	0.111	IPR015880 (SMART); PF13465 (PFAM); IPR013087 (G3DSA:3.30.160.GENE3D); IPR007087 (PFAM); IPR013087 (G3DSA:3.30.160.GENE3D); PF13894 (PFAM); PTHR24409 (PANTHER); PTHR24409:SF13 (PANTHER); IPR007087 (PROSITE_PATTERNS); IPR007087 (PROSITE_PATTERNS); IPR007087 (PROSITE_PATTERNS); IPR007087 (PROSITE_PATTERNS); IPR007087 (PROSITE_PATTERNS); IPR007087 (PROSITE_PATTERNS); IPR007087 (PROSITE_PATTERNS); IPR007087 (PROSITE_PATTERNS); TRANSMEMBRANE (PHOBIUS); TRANSMEMBRANE (PHOBIUS); CYTOPLASMIC_DOMAIN (PHOBIUS); CYTOPLASMIC_DOMAIN (PHOBIUS); NON_CYTOPLASMIC_DOMAIN (PHOBIUS); IPR007087 (PROSITE_PROFILES); IPR007087 (PROSITE_PROFILES); IPR007087 (PROSITE_PROFILES); IPR007087 (PROSITE_PROFILES); IPR007087 (PROSITE_PROFILES); IPR007087 (PROSITE_PROFILES); IPR007087 (PROSITE_PROFILES); IPR007087 (PROSITE_PROFILES); SSF57667 (SUPERFAMILY); SSF57667 (SUPERFAMILY); SSF57667 (SUPERFAMILY); SSF57667 (SUPERFAMILY); TMhelix (TMHMM); TMhelix (TMHMM)
1590	491500_length_1887_cvg_40.1_tip_1_4	293	ankyrin sam and basic leucine zipper domain-containing protein 1-like	629	1.35E-130	60.40%	0.155	IPR001660 (SMART); IPR002110 (SMART); IPR021129 (PFAM); IPR013761 (G3DSA:1.10.150.GENE3D); IPR020683 (G3DSA:1.25.40.GENE3D); IPR020683 (PFAM); PTHR24157 (PANTHER); IPR001660 (PROSITE_PROFILES); IPR002110 (PROSITE_PROFILES); IPR020683 (PROSITE_PROFILES); IPR002110 (PROSITE_PROFILES); IPR002110 (PROSITE_PROFILES); IPR013761 (SUPERFAMILY); IPR020683 (SUPERFAMILY)
1591	491502_length_1887_cvg_15.3_tip_1_0	134	4-trimethylaminobutyraldehyde partial	629	0	74.30%	0.22	IPR016163 (G3DSA:3.40.309.GENE3D); IPR015590 (PFAM); IPR016162 (G3DSA:3.40.605.GENE3D); PTHR11699:SF114 (PANTHER); PTHR11699 (PANTHER); IPR016160 (PROSITE_PATTERNS); IPR029510 (PROSITE_PATTERNS); IPR016161 (SUPERFAMILY)
1601	491684_length_1907_cvg_41.7_tip_1_4	326	low quality protein: zinc finger protein 345-like	629	1.31E-42	55.20%	0.101	IPR015880 (SMART); IPR013087 (G3DSA:3.30.160.GENE3D); PF13465 (PFAM); IPR013087 (G3DSA:3.30.160.GENE3D); IPR007087 (PFAM); PF13894 (PFAM); PTHR24409 (PANTHER); PTHR24409:SF13 (PANTHER); IPR007087 (PROSITE_PATTERNS); IPR007087 (PROSITE_PATTERNS); IPR007087 (PROSITE_PATTERNS); IPR007087 (PROSITE_PATTERNS); IPR007087 (PROSITE_PATTERNS); IPR007087 (PROSITE_PATTERNS); IPR007087 (PROSITE_PATTERNS); IPR007087 (PROSITE_PATTERNS); IPR007087 (PROSITE_PATTERNS); IPR007087 (PROSITE_PROFILES); IPR007087 (PROSITE_PROFILES); IPR007087 (PROSITE_PROFILES); IPR007087 (PROSITE_PROFILES); IPR007087 (PROSITE_PROFILES); IPR007087 (PROSITE_PROFILES); IPR007087 (PROSITE_PROFILES); IPR007087 (PROSITE_PROFILES); IPR007087 (PROSITE_PROFILES); SSF57667 (SUPERFAMILY); SSF57667 (SUPERFAMILY); SSF57667 (SUPERFAMILY); SSF57667 (SUPERFAMILY)
1617	492074_length_1950_cvg_10.7_tip_1_4	92	pleiotropic regulator 1	629	0	81.50%	0.167	IPR020472 (PRINTS); IPR001680 (SMART); IPR015943 (G3DSA:2.130.10.GENE3D); IPR001680 (PFAM); PTHR19923 (PANTHER); PTHR19923:SF0 (PANTHER); IPR019775 (PROSITE_PATTERNS); IPR019775 (PROSITE_PATTERNS); IPR001680 (PROSITE_PROFILES); IPR001680 (PROSITE_PROFILES); IPR001680 (PROSITE_PROFILES); IPR001680 (PROSITE_PROFILES); IPR001680 (PROSITE_PROFILES); IPR017986 (PROSITE_PROFILES); IPR017986 (SUPERFAMILY)
1592	491518_length_1889_cvg_50.9_tip_1_3	555	2-oxoglutarate and iron-dependent oxygenase domain-containing protein 3-like	630	1.26E-137	74.40%	0.106	IPR006620 (SMART); IPR005123 (PFAM); PTHR14650 (PANTHER); PTHR14650:SF1 (PANTHER); TRANSMEMBRANE (PHOBIUS); NON_CYTOPLASMIC_DOMAIN (PHOBIUS); CYTOPLASMIC_DOMAIN (PHOBIUS); TRANSMEMBRANE (PHOBIUS); CYTOPLASMIC_DOMAIN (PHOBIUS); TRANSMEMBRANE (PHOBIUS); NON_CYTOPLASMIC_DOMAIN (PHOBIUS); IPR005123 (PROSITE_PROFILES)
1602	491790_length_1919_cvg_36.2_tip_1_5	405	beta- -n-acetylgalactosaminyltransferase bre-4	631	8.65E-92	70.50%	0.133	IPR003859 (PRINTS); IPR029044 (G3DSA:3.90.550.GENE3D); IPR027995 (PFAM); IPR027791 (PFAM); IPR003859 (PANTHER); CYTOPLASMIC_DOMAIN (PHOBIUS); CYTOPLASMIC_DOMAIN (PHOBIUS); TRANSMEMBRANE (PHOBIUS); TRANSMEMBRANE (PHOBIUS); NON_CYTOPLASMIC_DOMAIN (PHOBIUS); IPR029044 (SUPERFAMILY); TMhelix (TMHMM); TMhelix (TMHMM); TMhelix (TMHMM)

1594	491580_length_1897_cvg_15.6_tip_1_1	107	arginine abc periplasmic arginine-binding protein	632	0	99.40%	0.126	IPR001320 (SMART); IPR001638 (SMART); PF00497 (PFAM); IPR005768 (TIGRFAM); G3DSA:3.40.190.10 (GENE3D); G3DSA:3.40.190.10 (GENE3D); PTHR18966:SF152 (PANTHER); PTHR18966 (PANTHER); IPR018313 (PROSITE_PATTERNS); NON_CYTOPLASMIC_DOMAIN (PHOBIUS); TRANSMEMBRANE (PHOBIUS); NON_CYTOPLASMIC_DOMAIN (PHOBIUS); TRANSMEMBRANE (PHOBIUS); CYTOPLASMIC_DOMAIN (PHOBIUS); SSF53850 (SUPERFAMILY)
1597	491630_length_1903_cvg_18.7_tip_1_5	192	btb poz domain-containing protein 17	634	0	75.30%	0.142	IPR000210 (SMART); IPR011705 (SMART); G3DSA:3.30.710.10 (GENE3D); IPR013069 (PFAM); IPR011705 (PFAM); PTHR24410:SF9 (PANTHER); PTHR24410 (PANTHER); TRANSMEMBRANE (PHOBIUS); CYTOPLASMIC_DOMAIN (PHOBIUS); NON_CYTOPLASMIC_DOMAIN (PHOBIUS); IPR000210 (PROSITE_PROFILES); IPR011333 (SUPERFAMILY); TMhelix (TMHMM)
1598	491634_length_1903_cvg_3.0_tip_1_2	31	zinc finger protein 436-like isoform x2	634	5.85E-42	59.70%	0.321	IPR000210 (SMART); IPR015880 (SMART); IPR013087 (G3DSA:3.30.160.GENE3D); IPR013087 (G3DSA:3.30.160.GENE3D); IPR013069 (PFAM); G3DSA:3.30.710.10 (GENE3D); PF13465 (PFAM); PTHR24409 (PANTHER); IPR007087 (PROSITE_PATTERNS); IPR007087 (PROSITE_PATTERNS); IPR007087 (PROSITE_PATTERNS); IPR007087 (PROSITE_PATTERNS); IPR007087 (PROSITE_PATTERNS); IPR007087 (PROSITE_PATTERNS); CYTOPLASMIC_DOMAIN (PHOBIUS); TRANSMEMBRANE (PHOBIUS); NON_CYTOPLASMIC_DOMAIN (PHOBIUS); IPR007087 (PROSITE_PROFILES); IPR007087 (PROSITE_PROFILES); IPR007087 (PROSITE_PROFILES); IPR007087 (PROSITE_PROFILES); IPR000210 (PROSITE_PROFILES); IPR007087 (PROSITE_PROFILES); IPR007087 (PROSITE_PROFILES); SSF57667 (SUPERFAMILY); IPR011333 (SUPERFAMILY); SSF57667 (SUPERFAMILY); SSF57667 (SUPERFAMILY); SSF57667 (SUPERFAMILY); TMhelix (TMHMM)
1599	491638_length_1903_cvg_30.6_tip_1_3	343	protein disulfide-isomerase a6	635	1.61E-140	81.70%	0.102	PR00421 (PRINTS); IPR013766 (PFAM); IPR012336 (G3DSA:3.40.30.GENE3D); IPR005788 (TIGRFAM); PTHR18929:SF38 (PANTHER); PTHR18929 (PANTHER); IPR017937 (PROSITE_PATTERNS); TRANSMEMBRANE (PHOBIUS); NON_CYTOPLASMIC_DOMAIN (PHOBIUS); CYTOPLASMIC_DOMAIN (PHOBIUS); IPR012336 (PROSITE_PROFILES); IPR012336 (SUPERFAMILY); IPR012336 (SUPERFAMILY); TMhelix (TMHMM)
1600	491674_length_1907_cvg_4.7_tip_1_3	48	sortilin-related l(dlr class) a repeats-containing-like isoform x2	636	0	69.90%	0.114	IPR002172 (PRINTS); IPR002172 (SMART); IPR000742 (SMART); IPR000033 (SMART); IPR002172 (G3DSA:4.10.400.GENE3D); IPR002172 (PFAM); G3DSA:2.10.25.10 (GENE3D); IPR000033 (PFAM); IPR011042 (G3DSA:2.120.10.GENE3D); IPR002172 (G3DSA:4.10.400.GENE3D); IPR002172 (G3DSA:4.10.400.GENE3D); PTHR12106:SF7 (PANTHER); PTHR12106 (PANTHER); IPR023415 (PROSITE_PATTERNS); IPR023415 (PROSITE_PATTERNS); IPR023415 (PROSITE_PATTERNS); IPR023415 (PROSITE_PATTERNS); IPR023415 (PROSITE_PATTERNS); IPR023415 (PROSITE_PATTERNS); IPR000033 (PROSITE_PROFILES); IPR002172 (PROSITE_PROFILES); IPR000033 (PROSITE_PROFILES); IPR002172 (PROSITE_PROFILES); IPR002172 (PROSITE_PROFILES); IPR000033 (PROSITE_PROFILES); IPR002172 (PROSITE_PROFILES); IPR002172 (PROSITE_PROFILES); IPR000033 (PROSITE_PROFILES); IPR002172 (PROSITE_PROFILES); IPR002172 (SUPERFAMILY); IPR002172 (SUPERFAMILY); IPR002172 (SUPERFAMILY); IPR002172 (SUPERFAMILY); IPR002172 (SUPERFAMILY); IPR002172 (SUPERFAMILY); SSF63825 (SUPERFAMILY)
1611	491958_length_1937_cvg_17.7_tip_1_0	152	adp-ribosylation factor-like protein 4a	638	7.82E-109	86.80%	0.114	IPR006689 (PRINTS); IPR024156 (SMART); IPR003579 (SMART); IPR006687 (SMART); IPR006689 (PFAM); IPR027417 (G3DSA:3.40.50.GENE3D); IPR005225 (TIGRFAM); PTHR11711 (PANTHER); PTHR11711:SF121 (PANTHER); NON_CYTOPLASMIC_DOMAIN (PHOBIUS); TRANSMEMBRANE (PHOBIUS); CYTOPLASMIC_DOMAIN (PHOBIUS); IPR024156 (PROSITE_PROFILES); IPR027417 (SUPERFAMILY)
1615	492050_length_1948_cvg_50.0_tip_1_2	652	peptidylglycine alpha-hydroxylating monooxygenase	639	1.50E-167	78.10%	0.157	IPR000720 (PRINTS); IPR000323 (G3DSA:2.60.120.GENE3D); IPR000323 (PFAM); PF03712 (PFAM); IPR014784 (G3DSA:2.60.120.GENE3D); PTHR10680 (PANTHER); IPR014783 (PROSITE_PATTERNS); TRANSMEMBRANE (PHOBIUS); TRANSMEMBRANE (PHOBIUS); TRANSMEMBRANE (PHOBIUS); NON_CYTOPLASMIC_DOMAIN (PHOBIUS); CYTOPLASMIC_DOMAIN (PHOBIUS); NON_CYTOPLASMIC_DOMAIN (PHOBIUS); NON_CYTOPLASMIC_DOMAIN (PHOBIUS); CYTOPLASMIC_DOMAIN (PHOBIUS); TRANSMEMBRANE (PHOBIUS); IPR008977 (SUPERFAMILY); IPR008977 (SUPERFAMILY); IPR008977 (SUPERFAMILY); TMhelix (TMHMM); TMhelix (TMHMM); TMhelix (TMHMM); TMhelix (TMHMM)

1603	491808_length_1920_cvg_5.8_tip_1_2	65	antigen partial	640	0	99.10%	0.184		IPR024973 (PFAM); IPR012332 (G3DSA:2.160.20.GENE3D); PTHR12338 (PANTHER); IPR011050 (SUPERFAMILY)
1604	491846_length_1924_cvg_60.1_tip_1_1	8613	retinal dehydrogenase 1-like	641	0	82.10%	0.207		IPR016162 (G3DSA:3.40.605.GENE3D); IPR015590 (PFAM); IPR016163 (G3DSA:3.40.309.GENE3D); PTHR11699 (PANTHER); PTHR11699:SF145 (PANTHER); IPR029510 (PROSITE_PATTERNS); IPR016160 (PROSITE_PATTERNS); NON_CYTOPLASMIC_DOMAIN (PHOBIUS); CYTOPLASMIC_DOMAIN (PHOBIUS); TRANSMEMBRANE (PHOBIUS); NON_CYTOPLASMIC_DOMAIN (PHOBIUS); TRANSMEMBRANE (PHOBIUS); IPR016161 (SUPERFAMILY); TMhelix (TMHMM)
1610	491948_length_1937_cvg_22.4_tip_1_3	207	gtp-binding protein sar1	641	5.09E-123	93.90%	0.249		IPR006689 (PRINTS); IPR006687 (SMART); IPR024156 (SMART); IPR006689 (PFAM); IPR005225 (TIGRFAM); IPR027417 (G3DSA:3.40.50.GENE3D); PTHR11711:SF133 (PANTHER); PTHR11711 (PANTHER); TRANSMEMBRANE (PHOBIUS); TRANSMEMBRANE (PHOBIUS); TRANSMEMBRANE (PHOBIUS); CYTOPLASMIC_DOMAIN (PHOBIUS); NON_CYTOPLASMIC_DOMAIN (PHOBIUS); CYTOPLASMIC_DOMAIN (PHOBIUS); CYTOPLASMIC_DOMAIN (PHOBIUS); TRANSMEMBRANE (PHOBIUS); NON_CYTOPLASMIC_DOMAIN (PHOBIUS); IPR006687 (PROSITE_PROFILES); IPR027417 (SUPERFAMILY); TMhelix (TMHMM); TMhelix (TMHMM); TMhelix (TMHMM)
1605	491878_length_1927_cvg_13.8_tip_1_2	152	serine threonine-protein phosphatase 6 regulatory ankyrin repeat subunit c-like	642	8.46E-96	55.00%	0.123		IPR002110 (SMART); IPR002110 (PFAM); IPR020683 (G3DSA:1.25.40.GENE3D); IPR020683 (G3DSA:1.25.40.GENE3D); IPR020683 (PFAM); IPR020683 (G3DSA:1.25.40.GENE3D); PTHR24198 (PANTHER); TRANSMEMBRANE (PHOBIUS); CYTOPLASMIC_DOMAIN (PHOBIUS); NON_CYTOPLASMIC_DOMAIN (PHOBIUS); IPR002110 (PROSITE_PROFILES); IPR002110 (PROSITE_PROFILES); IPR002110 (PROSITE_PROFILES); IPR002110 (PROSITE_PROFILES); IPR002110 (PROSITE_PROFILES); IPR002110 (PROSITE_PROFILES); IPR020683 (PROSITE_PROFILES); IPR020683 (SUPERFAMILY)
1616	492058_length_1949_cvg_61.8_tip_1_1	554	u8-agatoxin-ao1a-like isoform x2	642	3.78E-32	78.10%	0.366	Y	IPR004169 (PFAM); TRANSMEMBRANE (PHOBIUS); TRANSMEMBRANE (PHOBIUS); CYTOPLASMIC_DOMAIN (PHOBIUS); TRANSMEMBRANE (PHOBIUS); TRANSMEMBRANE (PHOBIUS); NON_CYTOPLASMIC_DOMAIN (PHOBIUS); NON_CYTOPLASMIC_DOMAIN (PHOBIUS); TRANSMEMBRANE (PHOBIUS); CYTOPLASMIC_DOMAIN (PHOBIUS); CYTOPLASMIC_DOMAIN (PHOBIUS); NON_CYTOPLASMIC_DOMAIN (PHOBIUS); SSF57059 (SUPERFAMILY); TMhelix (TMHMM); TMhelix (TMHMM); TMhelix (TMHMM); TMhelix (TMHMM)
1618	492108_length_1954_cvg_20.3_tip_1_1	268	mitochondrial 10-formyltetrahydrofolate dehydrogenase	643	0	91.80%	0.134		IPR016162 (G3DSA:3.40.605.GENE3D); IPR015590 (PFAM); IPR016163 (G3DSA:3.40.309.GENE3D); PTHR11699 (PANTHER); PTHR11699:SF118 (PANTHER); IPR029510 (PROSITE_PATTERNS); IPR016160 (PROSITE_PATTERNS); IPR016161 (SUPERFAMILY)
1606	491908_length_1932_cvg_24.5_tip_1_0	163	agap005714-pa-like protein	644	1.06E-156	50.20%	0.1		IPR000436 (SMART); IPR001881 (SMART); IPR000742 (SMART); IPR003410 (PFAM); G3DSA:2.10.25.10 (GENE3D); IPR001881 (PFAM); G3DSA:2.10.25.10 (GENE3D); G3DSA:2.10.70.10 (GENE3D); G3DSA:2.10.70.10 (GENE3D); IPR000436 (PFAM); PF14670 (PFAM); G3DSA:2.10.25.10 (GENE3D); G3DSA:2.10.25.10 (GENE3D); IPR023413 (G3DSA:2.40.155.GENE3D); PTHR24034 (PANTHER); IPR013032 (PROSITE_PATTERNS); IPR000152 (PROSITE_PATTERNS); IPR013032 (PROSITE_PATTERNS); IPR000152 (PROSITE_PATTERNS); IPR018097 (PROSITE_PATTERNS); IPR018097 (PROSITE_PATTERNS); IPR000152 (PROSITE_PATTERNS); IPR013032 (PROSITE_PATTERNS); IPR013032 (PROSITE_PATTERNS); IPR003410 (PROSITE_PROFILES); IPR000742 (PROSITE_PROFILES); IPR000742 (PROSITE_PROFILES); IPR000742 (PROSITE_PROFILES); IPR000742 (PROSITE_PROFILES); IPR000436 (PROSITE_PROFILES); IPR000436 (PROSITE_PROFILES); IPR000436 (SUPERFAMILY); SSF57196 (SUPERFAMILY); SSF57196 (SUPERFAMILY); SSF57196 (SUPERFAMILY); IPR009030 (SUPERFAMILY); IPR000436 (SUPERFAMILY)
1607	491922_length_1934_cvg_57.3_tip_1_3	629	wd repeat-containing protein 86-like	645	8.52E-128	65.60%	0.118		IPR020472 (PRINTS); IPR001680 (SMART); IPR015943 (G3DSA:2.130.10.GENE3D); IPR001680 (PFAM); IPR015943 (G3DSA:2.130.10.GENE3D); PTHR22844:SF166 (PANTHER); PTHR22844 (PANTHER); IPR019775 (PROSITE_PATTERNS); IPR019775 (PROSITE_PATTERNS); NON_CYTOPLASMIC_DOMAIN (PHOBIUS); TRANSMEMBRANE (PHOBIUS); CYTOPLASMIC_DOMAIN (PHOBIUS); IPR001680 (PROSITE_PROFILES); IPR001680 (PROSITE_PROFILES); IPR001680 (PROSITE_PROFILES); IPR001680 (PROSITE_PROFILES); IPR017986 (PROSITE_PROFILES); IPR017986 (SUPERFAMILY); IPR011047 (SUPERFAMILY); TMhelix (TMHMM)

1609	491946_length_1937_cvg_48.6_tip_1_5	571	zinc finger protein 239-like	645	3.55E-26	53.40%	0.138	IPR015880 (SMART); IPR013087 (G3DSA:3.30.160.GENE3D); IPR013087 (G3DSA:3.30.160.GENE3D); PF13465 (PFAM); IPR007087 (PFAM); PF13894 (PFAM); PTHR24409 (PANTHER); IPR007087 (PROSITE_PATTERNS); IPR007087 (PROSITE_PATTERNS); IPR007087 (PROSITE_PATTERNS); IPR007087 (PROSITE_PATTERNS); IPR007087 (PROSITE_PATTERNS); IPR007087 (PROSITE_PROFILES); IPR007087 (PROSITE_PROFILES); IPR007087 (PROSITE_PROFILES); IPR007087 (PROSITE_PROFILES); IPR007087 (PROSITE_PROFILES); IPR007087 (PROSITE_PROFILES); SSF57667 (SUPERFAMILY); SSF57667 (SUPERFAMILY); SSF57667 (SUPERFAMILY)
1622	492202_length_1966_cvg_63.6_tip_1_5	1552	hypothetical protein TcasGA2_TC013968	645	1.82E-10	46.29%	0.136	Coil (COILS); PTHR11008:SF13 (PANTHER); PTHR11008 (PANTHER); CYTOPLASMIC_DOMAIN (PHOBIUS); NON_CYTOPLASMIC_DOMAIN (PHOBIUS); TRANSMEMBRANE (PHOBIUS); TRANSMEMBRANE (PHOBIUS); TRANSMEMBRANE (PHOBIUS); TRANSMEMBRANE (PHOBIUS); CYTOPLASMIC_DOMAIN (PHOBIUS); NON_CYTOPLASMIC_DOMAIN (PHOBIUS); NON_CYTOPLASMIC_DOMAIN (PHOBIUS); IPR020234 (PRODOM); TMhelix (TMHMM); TMhelix (TMHMM); TMhelix (TMHMM); TMhelix (TMHMM); TMhelix (TMHMM)
1608	491932_length_1936_cvg_17.9_tip_1_0	182	cytochrome p450	646	0	69.90%	0.33	Coil (COILS); IPR002401 (PRINTS); IPR001128 (PRINTS); IPR001128 (PFAM); IPR001128 (G3DSA:1.10.630.GENE3D); PTHR24290:SF0 (PANTHER); PTHR24290 (PANTHER); IPR017972 (PROSITE_PATTERNS); TRANSMEMBRANE (PHOBIUS); NON_CYTOPLASMIC_DOMAIN (PHOBIUS); SIGNAL_PEPTIDE_H_REGION (PHOBIUS); SIGNAL_PEPTIDE_C_REGION (PHOBIUS); CYTOPLASMIC_DOMAIN (PHOBIUS); SIGNAL_PEPTIDE (PHOBIUS); SIGNAL_PEPTIDE_N_REGION (PHOBIUS); IPR001128 (SUPERFAMILY); TMhelix (TMHMM)
1612	491968_length_1939_cvg_57.0_tip_1_2	489	cell recognition protein caspr4 isoform 5	646	1.08E-66	54.10%	0.103	IPR001073 (SMART); IPR001073 (PFAM); IPR000885 (PFAM); IPR014716 (G3DSA:3.90.215.GENE3D); IPR008983 (G3DSA:2.60.120.GENE3D); PTHR10127:SF575 (PANTHER); PTHR10127 (PANTHER); TRANSMEMBRANE (PHOBIUS); NON_CYTOPLASMIC_DOMAIN (PHOBIUS); TRANSMEMBRANE (PHOBIUS); TRANSMEMBRANE (PHOBIUS); TRANSMEMBRANE (PHOBIUS); CYTOPLASMIC_DOMAIN (PHOBIUS); NON_CYTOPLASMIC_DOMAIN (PHOBIUS); NON_CYTOPLASMIC_DOMAIN (PHOBIUS); CYTOPLASMIC_DOMAIN (PHOBIUS); IPR002181 (PROSITE_PROFILES); IPR001073 (PROSITE_PROFILES); IPR008983 (SUPERFAMILY); IPR002181 (SUPERFAMILY); IPR002181 (SUPERFAMILY); TMhelix (TMHMM); TMhelix (TMHMM); TMhelix (TMHMM); TMhelix (TMHMM)
1625	492252_length_1972_cvg_13.9_tip_1_4	246	coatomer subunit alpha	647	0	81.70%	0.109	IPR010714 (PFAM); IPR006692 (PFAM); PTHR19876 (PANTHER)
1613	492018_length_1944_cvg_26.8_tip_1_3	265	ankyrin-1-like isoform x3	648	0	80.20%	0.108	IPR002110 (PRINTS); IPR001496 (SMART); IPR002110 (SMART); IPR001496 (SMART); IPR002110 (PFAM); IPR002110 (PFAM); IPR020683 (G3DSA:1.25.40.GENE3D); IPR020683 (G3DSA:1.25.40.GENE3D); IPR020683 (PFAM); IPR020683 (G3DSA:1.25.40.GENE3D); IPR001496 (PFAM); PTHR24133 (PANTHER); TRANSMEMBRANE (PHOBIUS); CYTOPLASMIC_DOMAIN (PHOBIUS); NON_CYTOPLASMIC_DOMAIN (PHOBIUS); IPR001496 (PROSITE_PROFILES); IPR002110 (PROSITE_PROFILES); IPR002110 (PROSITE_PROFILES); IPR020683 (PROSITE_PROFILES); IPR002110 (PROSITE_PROFILES); IPR002110 (PROSITE_PROFILES); IPR002110 (PROSITE_PROFILES); IPR002110 (PROSITE_PROFILES); IPR020683 (SUPERFAMILY); SSF158235 (SUPERFAMILY); TMhelix (TMHMM); TMhelix (TMHMM)
1614	492038_length_1946_cvg_59.0_tip_1_0	789	ddb1- and cul4-associated factor 13-like	649	0	80.80%	0.104	IPR001680 (SMART); IPR001680 (PFAM); IPR007287 (PFAM); IPR015943 (G3DSA:2.130.10.GENE3D); PTHR22851:SF0 (PANTHER); PTHR22851 (PANTHER); IPR019775 (PROSITE_PATTERNS); NON_CYTOPLASMIC_DOMAIN (PHOBIUS); TRANSMEMBRANE (PHOBIUS); CYTOPLASMIC_DOMAIN (PHOBIUS); IPR001680 (PROSITE_PROFILES); IPR001680 (PROSITE_PROFILES); IPR017986 (PROSITE_PROFILES); IPR001680 (PROSITE_PROFILES); IPR001680 (PROSITE_PROFILES); IPR017986 (SUPERFAMILY); TMhelix (TMHMM)

1623	492218_length_1968_cvg_44.2_tip_1_3	554	hypothetical protein L798_04491	649	4.36E-34	63.40%	0.107	IPR003961 (SMART); IPR003961 (PFAM); IPR013783 (G3DSA:2.60.40.GENE3D); PTHR21104 (PANTHER); TRANSMEMBRANE (PHOBIUS); CYTOPLASMIC_DOMAIN (PHOBIUS); NON_CYTOPLASMIC_DOMAIN (PHOBIUS); NON_CYTOPLASMIC_DOMAIN (PHOBIUS); NON_CYTOPLASMIC_DOMAIN (PHOBIUS); TRANSMEMBRANE (PHOBIUS); CYTOPLASMIC_DOMAIN (PHOBIUS); TRANSMEMBRANE (PHOBIUS); TRANSMEMBRANE (PHOBIUS); TRANSMEMBRANE (PHOBIUS); CYTOPLASMIC_DOMAIN (PHOBIUS); IPR003961 (PROSITE_PROFILES); IPR003961 (SUPERFAMILY); TMhelix (TMHMM); TMhelix (TMHMM); TMhelix (TMHMM)
1619	492124_length_1956_cvg_7.0_tip_1_3	97	esterase fe4	652	1.61E-65	70.80%	0.108	IPR029058 (G3DSA:3.40.50.GENE3D); IPR002018 (PFAM); PTHR11559 (PANTHER); IPR019826 (PROSITE_PATTERNS); IPR019819 (PROSITE_PATTERNS); IPR029058 (SUPERFAMILY)
1620	492124_length_1956_cvg_7.0_tip_1_4	97	venom carboxylesterase-6	652	1.09E-38	53.10%	0.137	IPR029058 (G3DSA:3.40.50.GENE3D); IPR002018 (PFAM); PTHR11559 (PANTHER); CYTOPLASMIC_DOMAIN (PHOBIUS); NON_CYTOPLASMIC_DOMAIN (PHOBIUS); TRANSMEMBRANE (PHOBIUS); IPR029058 (SUPERFAMILY); TMhelix (TMHMM)
1621	492184_length_1963_cvg_3.8_tip_1_1	52	iron zinc purple acid phosphatase-like protein	654	0	75.30%	0.297	IPR015914 (G3DSA:2.60.40.GENE3D); IPR004843 (PFAM); IPR029052 (G3DSA:3.60.21.GENE3D); IPR025733 (PFAM); PTHR22953:SF9 (PANTHER); PTHR22953 (PANTHER); TRANSMEMBRANE (PHOBIUS); NON_CYTOPLASMIC_DOMAIN (PHOBIUS); CYTOPLASMIC_DOMAIN (PHOBIUS); TRANSMEMBRANE (PHOBIUS); CYTOPLASMIC_DOMAIN (PHOBIUS); IPR029052 (SUPERFAMILY); IPR008963 (SUPERFAMILY); TMhelix (TMHMM); TMhelix (TMHMM); TMhelix (TMHMM)
1624	492240_length_1970_cvg_7.8_tip_1_5	75	calcium calmodulin-dependent protein kinase ii isoform a	656	0	93.60%	0.138	IPR002290 (SMART); IPR013543 (PFAM); G3DSA:1.10.510.10 (GENE3D); G3DSA:3.30.200.20 (GENE3D); IPR000719 (PFAM); G3DSA:3.10.450.50 (GENE3D); PTHR24347:SF108 (PANTHER); IPR020636 (PANTHER); IPR017441 (PROSITE_PATTERNS); IPR008271 (PROSITE_PATTERNS); CYTOPLASMIC_DOMAIN (PHOBIUS); NON_CYTOPLASMIC_DOMAIN (PHOBIUS); TRANSMEMBRANE (PHOBIUS); IPR000719 (PROSITE_PROFILES); SSF54427 (SUPERFAMILY); IPR011009 (SUPERFAMILY)
1633	492442_length_1999_cvg_58.9_tip_1_2	2859	hemagglutinin amebocyte aggregation factor-like	657	1.22E-39	61.10%	0.159	PF14704 (PFAM); IPR026645 (PANTHER); CYTOPLASMIC_DOMAIN (PHOBIUS); NON_CYTOPLASMIC_DOMAIN (PHOBIUS); TRANSMEMBRANE (PHOBIUS); TMhelix (TMHMM)
1631	492378_length_1989_cvg_61.9_tip_1_4	1087	lissencephaly-1 homolog	658	0	90.90%	0.171	Coil (COILS); IPR020472 (PRINTS); IPR001680 (SMART); IPR006594 (SMART); IPR015943 (G3DSA:2.130.10.GENE3D); IPR006594 (PFAM); IPR001680 (PFAM); PTHR22847 (PANTHER); IPR019775 (PROSITE_PATTERNS); IPR019775 (PROSITE_PATTERNS); IPR019775 (PROSITE_PATTERNS); IPR019775 (PROSITE_PATTERNS); IPR019775 (PROSITE_PATTERNS); IPR019775 (PROSITE_PATTERNS); NON_CYTOPLASMIC_DOMAIN (PHOBIUS); CYTOPLASMIC_DOMAIN (PHOBIUS); NON_CYTOPLASMIC_DOMAIN (PHOBIUS); TRANSMEMBRANE (PHOBIUS); TRANSMEMBRANE (PHOBIUS); IPR001680 (PROSITE_PROFILES); IPR017986 (PROSITE_PROFILES); IPR001680 (PROSITE_PROFILES); IPR001680 (PROSITE_PROFILES); IPR006594 (PROSITE_PROFILES); IPR001680 (PROSITE_PROFILES); IPR001680 (PROSITE_PROFILES); IPR001680 (PROSITE_PROFILES); IPR001680 (PROSITE_PROFILES); IPR017252 (HAMAP); SSF109925 (SUPERFAMILY); IPR017986 (SUPERFAMILY)
1626	492276_length_1975_cvg_2.7_tip_1_0	35	peroxisomal multifunctional enzyme type 2	659	0	71.90%	0.139	IPR002198 (PRINTS); IPR002347 (PRINTS); IPR013968 (SMART); PF13452 (PFAM); IPR003033 (G3DSA:3.30.1050.GENE3D); IPR002198 (PFAM); IPR016040 (G3DSA:3.40.50.GENE3D); IPR002539 (PFAM); IPR029069 (G3DSA:3.10.129.GENE3D); IPR029069 (G3DSA:3.10.129.GENE3D); PTHR24316:SF2 (PANTHER); PTHR24316 (PANTHER); IPR020904 (PROSITE_PATTERNS); IPR029069 (SUPERFAMILY); SSF51735 (SUPERFAMILY); IPR029069 (SUPERFAMILY)
1627	492346_length_1986_cvg_2.9_tip_1_4	42	transmembrane protease serine partial	662	1.07E-12	76.50%	0.101	IPR001254 (SMART); IPR001254 (PFAM); G3DSA:2.40.10.10 (GENE3D); PTHR24256 (PANTHER); TRANSMEMBRANE (PHOBIUS); NON_CYTOPLASMIC_DOMAIN (PHOBIUS); CYTOPLASMIC_DOMAIN (PHOBIUS); NON_CYTOPLASMIC_DOMAIN (PHOBIUS); TRANSMEMBRANE (PHOBIUS); CYTOPLASMIC_DOMAIN (PHOBIUS); TRANSMEMBRANE (PHOBIUS); IPR001254 (PROSITE_PROFILES); IPR009003 (SUPERFAMILY)

1628	492346_length_1986_cvg_2.9_tip_1_5	42	plasminogen activator spa	662	7.96E-10	67.40%	0.109		Coil (COILS); IPR001254 (PFAM); G3DSA:2.40.10.10 (GENE3D); PTHR24276 (PANTHER); NON_CYTOPLASMIC_DOMAIN (PHOBIUS); TRANSMEMBRANE (PHOBIUS); CYTOPLASMIC_DOMAIN (PHOBIUS); CYTOPLASMIC_DOMAIN (PHOBIUS); TRANSMEMBRANE (PHOBIUS); TRANSMEMBRANE (PHOBIUS); NON_CYTOPLASMIC_DOMAIN (PHOBIUS); IPR009003 (SUPERFAMILY); TMhelix (TMHMM)
1635	492480_length_2003_cvg_34.5_tip_1_2	394	retinoid-inducible serine carboxypeptidase-like	662	1.22E-25	65.30%	0.25		IPR001563 (PFAM); IPR029058 (G3DSA:3.40.50.GENE3D); IPR001563 (PANTHER); CYTOPLASMIC_DOMAIN (PHOBIUS); CYTOPLASMIC_DOMAIN (PHOBIUS); NON_CYTOPLASMIC_DOMAIN (PHOBIUS); NON_CYTOPLASMIC_DOMAIN (PHOBIUS); TRANSMEMBRANE (PHOBIUS); TRANSMEMBRANE (PHOBIUS); TRANSMEMBRANE (PHOBIUS); IPR029058 (SUPERFAMILY); TMhelix (TMHMM); TMhelix (TMHMM); TMhelix (TMHMM)
1629	492352_length_1987_cvg_9.1_tip_1_3	75	interleukin-1 receptor-associated kinase 4	663	1.26E-130	56.90%	0.102		IPR002290 (SMART); IPR011029 (G3DSA:1.10.533.GENE3D); IPR000719 (PFAM); G3DSA:3.30.200.20 (GENE3D); IPR029397 (PFAM); G3DSA:1.10.510.10 (GENE3D); PTHR24419 (PANTHER); PTHR24419:SF0 (PANTHER); IPR017441 (PROSITE_PATTERNS); IPR008271 (PROSITE_PATTERNS); CYTOPLASMIC_DOMAIN (PHOBIUS); NON_CYTOPLASMIC_DOMAIN (PHOBIUS); TRANSMEMBRANE (PHOBIUS); IPR000719 (PROSITE_PROFILES); IPR011009 (SUPERFAMILY); IPR011029 (SUPERFAMILY); TMhelix (TMHMM)
1630	492376_length_1989_cvg_28.6_tip_1_5	217	ww domain-containing oxidoreductase	663	1.36E-164	75.70%	0.101		Coil (COILS); IPR002347 (PRINTS); IPR001202 (SMART); IPR002198 (PFAM); IPR016040 (G3DSA:3.40.50.GENE3D); G3DSA:2.20.70.10 (GENE3D); PTHR24316:SF115 (PANTHER); PTHR24316 (PANTHER); IPR007087 (PROSITE_PATTERNS); IPR001202 (PROSITE_PROFILES); SSF51735 (SUPERFAMILY); IPR001202 (SUPERFAMILY)
1644	492690_length_2035_cvg_45.2_tip_1_2	1275	serine protease 56	664	5.77E-11	43.80%	0.1		IPR001254 (SMART); G3DSA:2.40.10.10 (GENE3D); NON_CYTOPLASMIC_DOMAIN (PHOBIUS); CYTOPLASMIC_DOMAIN (PHOBIUS); TRANSMEMBRANE (PHOBIUS); IPR001254 (PROSITE_PROFILES); IPR009003 (SUPERFAMILY); TMhelix (TMHMM)
1632	492426_length_1996_cvg_24.1_tip_1_5	281	probable cytochrome p450 49a1	665	1.58E-143	62.70%	0.163		IPR002401 (PRINTS); IPR001128 (PRINTS); IPR001128 (G3DSA:1.10.630.GENE3D); IPR001128 (PFAM); PTHR24305:SF49 (PANTHER); PTHR24305 (PANTHER); IPR017972 (PROSITE_PATTERNS); IPR001128 (SUPERFAMILY)
1637	492566_length_2019_cvg_24.4_tip_1_4	224	peptidase m4	665	1.48E-86	54.00%	0.440	Y	IPR023612 (PRINTS); G3DSA:1.10.390.10 (GENE3D); IPR013856 (G3DSA:3.10.170.GENE3D); IPR011096 (PFAM); IPR013856 (PFAM); IPR001570 (PFAM); NON_CYTOPLASMIC_DOMAIN (PHOBIUS); TRANSMEMBRANE (PHOBIUS); SIGNAL_PEPTIDE_C_REGION (PHOBIUS); TRANSMEMBRANE (PHOBIUS); TRANSMEMBRANE (PHOBIUS); NON_CYTOPLASMIC_DOMAIN (PHOBIUS); SIGNAL_PEPTIDE_N_REGION (PHOBIUS); CYTOPLASMIC_DOMAIN (PHOBIUS); CYTOPLASMIC_DOMAIN (PHOBIUS); SIGNAL_PEPTIDE_H_REGION (PHOBIUS); SIGNAL_PEPTIDE (PHOBIUS); TRANSMEMBRANE (PHOBIUS); NON_CYTOPLASMIC_DOMAIN (PHOBIUS); SSF55486 (SUPERFAMILY)
1634	492474_length_2003_cvg_19.7_tip_1_1	304	uncharacterized family 31 glucosidase kiaa1161 isoform x4	668	8.33E-90	69.30%	0.146		IPR000322 (PFAM); PTHR22762 (PANTHER); PTHR22762:SF73 (PANTHER); SSF51011 (SUPERFAMILY); IPR017853 (SUPERFAMILY)
1645	492692_length_2036_cvg_30.4_tip_1_2	320	zinc finger protein 850-like isoform x1	668	5.70E-44	57.90%	0.146		IPR015880 (SMART); IPR013087 (G3DSA:3.30.160.GENE3D); IPR013087 (G3DSA:3.30.160.GENE3D); IPR013087 (G3DSA:3.30.160.GENE3D); PF13465 (PFAM); IPR013087 (G3DSA:3.30.160.GENE3D); IPR007087 (PFAM); IPR013087 (G3DSA:3.30.160.GENE3D); PTHR24409:SF14 (PANTHER); PTHR24409 (PANTHER); IPR007087 (PROSITE_PATTERNS); IPR007087 (PROSITE_PATTERNS); IPR007087 (PROSITE_PATTERNS); IPR007087 (PROSITE_PATTERNS); IPR007087 (PROSITE_PATTERNS); IPR007087 (PROSITE_PATTERNS); IPR007087 (PROSITE_PATTERNS); IPR007087 (PROSITE_PROFILES); IPR007087 (PROSITE_PROFILES); IPR007087 (PROSITE_PROFILES); IPR007087 (PROSITE_PROFILES); IPR007087 (PROSITE_PROFILES); IPR007087 (PROSITE_PROFILES); IPR007087 (PROSITE_PROFILES); IPR007087 (PROSITE_PROFILES); SSF57667 (SUPERFAMILY); SSF57667 (SUPERFAMILY); SSF57667 (SUPERFAMILY); SSF57667 (SUPERFAMILY); SSF57667 (SUPERFAMILY)

1643	492678_length_2034_cvg_12.3_tip_1_1	145	calcium activated chlorine	669	2.51E-17	54.50%	0.108	IPR003961 (SMART); IPR013783 (G3DSA:2.60.40.GENE3D); PTHR10579 (PANTHER); CYTOPLASMIC_DOMAIN (PHOBIUS); NON_CYTOPLASMIC_DOMAIN (PHOBIUS); NON_CYTOPLASMIC_DOMAIN (PHOBIUS); TRANSMEMBRANE (PHOBIUS); CYTOPLASMIC_DOMAIN (PHOBIUS); NON_CYTOPLASMIC_DOMAIN (PHOBIUS); TRANSMEMBRANE (PHOBIUS); TRANSMEMBRANE (PHOBIUS); TRANSMEMBRANE (PHOBIUS); TRANSMEMBRANE (PHOBIUS); CYTOPLASMIC_DOMAIN (PHOBIUS); IPR003961 (SUPERFAMILY); TMhelix (TMHMM); TMhelix (TMHMM); TMhelix (TMHMM); TMhelix (TMHMM); TMhelix (TMHMM)
1636	492506_length_2010_cvg_62.3_tip_1_3	2857	methyltransferase-like protein 23	670	8.79E-94	75.50%	0.132	IPR000582 (PRINTS); IPR000504 (SMART); IPR002110 (SMART); IPR000582 (PFAM); IPR020683 (G3DSA:1.25.40.GENE3D); IPR012677 (G3DSA:3.30.70.GENE3D); IPR020683 (PFAM); IPR000504 (PFAM); IPR014352 (G3DSA:1.20.80.GENE3D); IPR019410 (PFAM); IPR029063 (G3DSA:3.40.50.GENE3D); PTHR24119 (PANTHER); PTHR24119:SF0 (PANTHER); IPR000582 (PROSITE_PROFILES); IPR000504 (PROSITE_PROFILES); IPR002110 (PROSITE_PROFILES); IPR002110 (PROSITE_PROFILES); IPR020683 (PROSITE_PROFILES); IPR029063 (SUPERFAMILY); IPR020683 (SUPERFAMILY); SSF54928 (SUPERFAMILY); IPR000582 (SUPERFAMILY)
1638	492576_length_2021_cvg_4.6_tip_1_5	66	hypothetical protein DAPPUDRAFT_316225	673	1.29E-57	50.40%	0.152	IPR001190 (PRINTS); IPR017448 (SMART); IPR002557 (SMART); IPR002557 (G3DSA:2.170.140.GENE3D); IPR016186 (G3DSA:3.10.100.GENE3D); IPR017448 (G3DSA:3.10.250.GENE3D); IPR001190 (PFAM); IPR002557 (PFAM); PTHR23301 (PANTHER); TRANSMEMBRANE (PHOBIUS); NON_CYTOPLASMIC_DOMAIN (PHOBIUS); TRANSMEMBRANE (PHOBIUS); CYTOPLASMIC_DOMAIN (PHOBIUS); NON_CYTOPLASMIC_DOMAIN (PHOBIUS); IPR001190 (PROSITE_PROFILES); IPR002557 (PROSITE_PROFILES); IPR002557 (PROSITE_PROFILES); IPR017448 (SUPERFAMILY); IPR002557 (SUPERFAMILY); IPR016187 (SUPERFAMILY); IPR002557 (SUPERFAMILY); TMhelix (TMHMM); TMhelix (TMHMM); TMhelix (TMHMM)
1647	492760_length_2045_cvg_39.2_tip_1_4	408	protein phosphatase 1 regulatory subunit 7	673	1.25E-117	77.00%	0.113	IPR003603 (SMART); IPR003591 (SMART); SM00365 (SMART); G3DSA:3.80.10.10 (GENE3D); IPR025875 (PFAM); G3DSA:3.80.10.10 (GENE3D); PF14580 (PFAM); PTHR10588 (PANTHER); IPR001611 (PROSITE_PROFILES); IPR001611 (PROSITE_PROFILES); IPR001611 (PROSITE_PROFILES); IPR001611 (PROSITE_PROFILES); IPR001611 (PROSITE_PROFILES); IPR001611 (PROSITE_PROFILES); IPR001611 (PROSITE_PROFILES); IPR001611 (PROSITE_PROFILES); IPR001611 (PROSITE_PROFILES); IPR001611 (PROSITE_PROFILES); IPR001611 (PROSITE_PROFILES); SSF52058 (SUPERFAMILY)
1639	492580_length_2021_cvg_2.2_tip_1_3	36	maguk p55 subfamily member 7 isoform x4	674	2.76E-179	87.30%	0.105	IPR008145 (SMART); IPR001452 (SMART); IPR008145 (PFAM); G3DSA:2.30.30.40 (GENE3D); IPR027417 (G3DSA:3.40.50.GENE3D); G3DSA:3.30.63.10 (GENE3D); IPR011511 (PFAM); PTHR23122 (PANTHER); PTHR23122:SF14 (PANTHER); IPR020590 (PROSITE_PATTERNS); IPR001452 (PROSITE_PROFILES); IPR008144 (PROSITE_PROFILES); IPR027417 (SUPERFAMILY); IPR001452 (SUPERFAMILY)
1648	492774_length_2050_cvg_61.1_tip_1_3	1486	cd166 antigen	675	5.06E-116	56.30%	0.137	IPR003599 (SMART); IPR003598 (SMART); IPR013783 (G3DSA:2.60.40.GENE3D); IPR013783 (G3DSA:2.60.40.GENE3D); PF13895 (PFAM); IPR013783 (G3DSA:2.60.40.GENE3D); IPR013162 (PFAM); IPR013783 (G3DSA:2.60.40.GENE3D); IPR013098 (PFAM); IPR013783 (G3DSA:2.60.40.GENE3D); PTHR11640 (PANTHER); PTHR11640:SF52 (PANTHER); CYTOPLASMIC_DOMAIN (PHOBIUS); TRANSMEMBRANE (PHOBIUS); TRANSMEMBRANE (PHOBIUS); CYTOPLASMIC_DOMAIN (PHOBIUS); NON_CYTOPLASMIC_DOMAIN (PHOBIUS); IPR007110 (PROSITE_PROFILES); IPR007110 (PROSITE_PROFILES); IPR007110 (PROSITE_PROFILES); IPR007110 (PROSITE_PROFILES); SSF48726 (SUPERFAMILY); SSF48726 (SUPERFAMILY); SSF48726 (SUPERFAMILY); SSF48726 (SUPERFAMILY); SSF48726 (SUPERFAMILY); TMhelix (TMHMM)
1640	492626_length_2027_cvg_27.7_tip_1_3	274	laccase 2	676	2.00E-143	60.20%	0.107	IPR011706 (PFAM); IPR008972 (G3DSA:2.60.40.GENE3D); IPR011707 (PFAM); IPR001117 (PFAM); IPR008972 (G3DSA:2.60.40.GENE3D); IPR008972 (G3DSA:2.60.40.GENE3D); PTHR11709:SF33 (PANTHER); PTHR11709 (PANTHER); IPR002355 (PROSITE_PATTERNS); CYTOPLASMIC_DOMAIN (PHOBIUS); TRANSMEMBRANE (PHOBIUS); TRANSMEMBRANE (PHOBIUS); NON_CYTOPLASMIC_DOMAIN (PHOBIUS); CYTOPLASMIC_DOMAIN (PHOBIUS); IPR008972 (SUPERFAMILY); IPR008972 (SUPERFAMILY); IPR008972 (SUPERFAMILY); TMhelix (TMHMM)

1641	492630_length_2028_cvg_20.5_tip_1_3	240	glucose-6-phosphate isomerase	676	0	87.70%	0.118	IPR001672 (PRINTS); IPR023096 (G3DSA:1.10.1390.GENE3D); IPR001672 (PFAM); G3DSA:3.40.50.10490 (GENE3D); IPR001672 (PANTHER); PTHR11469:SF4 (PANTHER); IPR018189 (PROSITE_PATTERNS); IPR018189 (PROSITE_PATTERNS); TRANSMEMBRANE (PHOBIUS); NON_CYTOPLASMIC_DOMAIN (PHOBIUS); CYTOPLASMIC_DOMAIN (PHOBIUS); IPR001672 (HAMAP); IPR001672 (PROSITE_PROFILES); SSF53697 (SUPERFAMILY)
1649	492794_length_2055_cvg_38.3_tip_1_4	415	carbonic anhydrase 1	677	1.63E-103	69.30%	0.109	IPR001148 (SMART); IPR001148 (G3DSA:3.10.200.GENE3D); IPR001148 (PFAM); IPR023561 (PANTHER); IPR018338 (PROSITE_PATTERNS); TRANSMEMBRANE (PHOBIUS); NON_CYTOPLASMIC_DOMAIN (PHOBIUS); CYTOPLASMIC_DOMAIN (PHOBIUS); IPR001148 (PROSITE_PROFILES); IPR001148 (SUPERFAMILY)
1642	492672_length_2033_cvg_10.7_tip_1_0	100	zinc finger protein 235-like	678	1.79E-49	51.90%	0.102	IPR015880 (SMART); PF13894 (PFAM); PF13465 (PFAM); IPR013087 (G3DSA:3.30.160.GENE3D); IPR007087 (PFAM); IPR013087 (G3DSA:3.30.160.GENE3D); IPR013087 (G3DSA:3.30.160.GENE3D); IPR013087 (G3DSA:3.30.160.GENE3D); PTHR24409 (PANTHER); IPR007087 (PROSITE_PATTERNS); IPR007087 (PROSITE_PATTERNS); IPR007087 (PROSITE_PATTERNS); IPR007087 (PROSITE_PATTERNS); IPR007087 (PROSITE_PATTERNS); IPR007087 (PROSITE_PATTERNS); IPR007087 (PROSITE_PATTERNS); IPR007087 (PROSITE_PATTERNS); IPR007087 (PROSITE_PATTERNS); IPR007087 (PROSITE_PATTERNS); IPR007087 (PROSITE_PATTERNS); IPR007087 (PROSITE_PROFILES); IPR007087 (PROSITE_PROFILES); IPR007087 (PROSITE_PROFILES); IPR007087 (PROSITE_PROFILES); IPR007087 (PROSITE_PROFILES); IPR007087 (PROSITE_PROFILES); IPR007087 (PROSITE_PROFILES); IPR007087 (PROSITE_PROFILES); IPR007087 (PROSITE_PROFILES); IPR007087 (PROSITE_PROFILES); SSF57667 (SUPERFAMILY); SSF57667 (SUPERFAMILY); SSF57667 (SUPERFAMILY); SSF57667 (SUPERFAMILY); SSF57667 (SUPERFAMILY)
1657	493042_length_2097_cvg_6.3_tip_1_2	56	zinc finger protein ozf-like	678	2.50E-28	52.80%	0.176	IPR000210 (SMART); IPR015880 (SMART); PF13465 (PFAM); IPR013069 (PFAM); IPR013087 (G3DSA:3.30.160.GENE3D); G3DSA:3.30.710.10 (GENE3D); PF13912 (PFAM); IPR013087 (G3DSA:3.30.160.GENE3D); IPR013087 (G3DSA:3.30.160.GENE3D); IPR007087 (PFAM); PTHR24409 (PANTHER); IPR007087 (PROSITE_PATTERNS); IPR007087 (PROSITE_PATTERNS); IPR007087 (PROSITE_PATTERNS); IPR007087 (PROSITE_PATTERNS); IPR007087 (PROSITE_PATTERNS); IPR007087 (PROSITE_PROFILES); IPR007087 (PROSITE_PROFILES); IPR007087 (PROSITE_PROFILES); IPR007087 (PROSITE_PROFILES); IPR000210 (PROSITE_PROFILES); IPR007087 (PROSITE_PROFILES); SSF57667 (SUPERFAMILY); SSF57667 (SUPERFAMILY); IPR011333 (SUPERFAMILY); SSF57667 (SUPERFAMILY)
1646	492722_length_2039_cvg_7.6_tip_1_3	120	von willebrand factor	680	4.22E-20	44.40%	0.103	PD968187 (PRODOM)
1650	492834_length_2059_cvg_54.1_tip_1_0	581	transforming growth factor-beta-induced protein ig-h3	683	0	57.60%	0.108	IPR000782 (SMART); IPR000782 (PFAM); IPR000782 (G3DSA:2.30.180.GENE3D); PTHR10900:SF73 (PANTHER); PTHR10900 (PANTHER); IPR000782 (PROSITE_PROFILES); IPR000782 (PROSITE_PROFILES); IPR000782 (PROSITE_PROFILES); IPR000782 (PROSITE_PROFILES); IPR000782 (PROSITE_PROFILES); IPR000782 (SUPERFAMILY); IPR000782 (SUPERFAMILY); IPR000782 (SUPERFAMILY); IPR000782 (SUPERFAMILY)
1651	492892_length_2072_cvg_7.1_tip_1_0	75	ccr4-not transcription complex subunit 6-like isoform x1	691	0	85.10%	0.113	IPR003591 (SMART); IPR025875 (PFAM); IPR005135 (G3DSA:3.60.10.GENE3D); G3DSA:3.80.10.10 (GENE3D); IPR005135 (PFAM); PTHR12121:SF34 (PANTHER); PTHR12121 (PANTHER); IPR001611 (PROSITE_PROFILES); IPR001611 (PROSITE_PROFILES); IPR001611 (PROSITE_PROFILES); IPR005135 (SUPERFAMILY); SSF52058 (SUPERFAMILY)

1652	492898_length_2073_cvg_6.1_tip_1_4	68	gastrula zinc finger	691	7.18E-64	58.50%	0.399 Y	Coil (COILS); IPR000210 (SMART); IPR015880 (SMART); IPR007087 (PFAM); IPR013069 (PFAM); IPR013087 (G3DSA:3.30.160.GENE3D); IPR013087 (G3DSA:3.30.160.GENE3D); PF13465 (PFAM); G3DSA:3.30.710.10 (GENE3D); IPR013087 (G3DSA:3.30.160.GENE3D); PF13894 (PFAM); IPR013087 (G3DSA:3.30.160.GENE3D); PTHR24409 (PANTHER); IPR007087 (PROSITE_PATTERNS); IPR007087 (PROSITE_PATTERNS); IPR007087 (PROSITE_PATTERNS); IPR007087 (PROSITE_PATTERNS); IPR007087 (PROSITE_PATTERNS); IPR007087 (PROSITE_PATTERNS); IPR007087 (PROSITE_PATTERNS); IPR007087 (PROSITE_PATTERNS); IPR007087 (PROSITE_PATTERNS); IPR007087 (PROSITE_PROFILES); IPR007087 (PROSITE_PROFILES); IPR007087 (PROSITE_PROFILES); IPR007087 (PROSITE_PROFILES); IPR000210 (PROSITE_PROFILES); IPR007087 (PROSITE_PROFILES); IPR007087 (PROSITE_PROFILES); IPR007087 (PROSITE_PROFILES); IPR007087 (PROSITE_PROFILES); IPR007087 (PROSITE_PROFILES); SSF57667 (SUPERFAMILY); SSF57667 (SUPERFAMILY); SSF57667 (SUPERFAMILY); SSF57667 (SUPERFAMILY); IPR011333 (SUPERFAMILY); SSF57667 (SUPERFAMILY)
1653	492972_length_2083_cvg_60.2_tip_1_0	1173	arylsulfatase b	695	4.30E-173	64.80%	0.289	IPR000917 (PFAM); G3DSA:3.30.1120.10 (GENE3D); IPR017849 (G3DSA:3.40.720.GENE3D); PTHR10342 (PANTHER); PTHR10342:SF206 (PANTHER); IPR024607 (PROSITE_PATTERNS); IPR024607 (PROSITE_PATTERNS); CYTOPLASMIC_DOMAIN (PHOBIUS); TRANSMEMBRANE (PHOBIUS); NON_CYTOPLASMIC_DOMAIN (PHOBIUS); CYTOPLASMIC_DOMAIN (PHOBIUS); TRANSMEMBRANE (PHOBIUS); IPR017850 (SUPERFAMILY); TMhelix (TMHMM)
1661	493116_length_2112_cvg_7.1_tip_1_5	95	counting factor associated protein d-like	695	0	76.80%	0.214	IPR000668 (PRINTS); IPR000668 (SMART); IPR013201 (SMART); G3DSA:3.90.70.10 (GENE3D); IPR013201 (PFAM); IPR000668 (PFAM); PTHR12411:SF284 (PANTHER); IPR013128 (PANTHER); IPR025660 (PROSITE_PATTERNS); IPR025661 (PROSITE_PATTERNS); IPR000169 (PROSITE_PATTERNS); NON_CYTOPLASMIC_DOMAIN (PHOBIUS); TRANSMEMBRANE (PHOBIUS); CYTOPLASMIC_DOMAIN (PHOBIUS); SSF54001 (SUPERFAMILY); TMhelix (TMHMM)
1654	492982_length_2087_cvg_5.6_tip_0_1	58	ankyrin isoform p	696	0	93.30%	0.122	IPR002110 (PRINTS); IPR002110 (SMART); IPR002110 (PFAM); IPR020683 (G3DSA:1.25.40.GENE3D); IPR020683 (G3DSA:1.25.40.GENE3D); IPR020683 (G3DSA:1.25.40.GENE3D); IPR020683 (G3DSA:1.25.40.GENE3D); IPR020683 (PFAM); PTHR24123 (PANTHER); PTHR24123:SF0 (PANTHER); IPR002110 (PROSITE_PROFILES); IPR002110 (PROSITE_PROFILES); IPR002110 (PROSITE_PROFILES); IPR002110 (PROSITE_PROFILES); IPR002110 (PROSITE_PROFILES); IPR002110 (PROSITE_PROFILES); IPR002110 (PROSITE_PROFILES); IPR002110 (PROSITE_PROFILES); IPR002110 (PROSITE_PROFILES); IPR002110 (PROSITE_PROFILES); IPR002110 (PROSITE_PROFILES); IPR020683 (PROSITE_PROFILES); IPR002110 (PROSITE_PROFILES); IPR002110 (PROSITE_PROFILES); IPR002110 (PROSITE_PROFILES); IPR020683 (SUPERFAMILY); IPR020683 (SUPERFAMILY); IPR020683 (SUPERFAMILY)
1655	493020_length_2093_cvg_6.2_tip_1_0	64	gastrula zinc finger isoform x1	698	6.63E-49	55.90%	0.114	IPR015880 (SMART); IPR013087 (G3DSA:3.30.160.GENE3D); IPR013087 (G3DSA:3.30.160.GENE3D); IPR007087 (PFAM); IPR013087 (G3DSA:3.30.160.GENE3D); PF13894 (PFAM); PTHR24409:SF14 (PANTHER); PTHR24409 (PANTHER); IPR007087 (PROSITE_PATTERNS); IPR007087 (PROSITE_PATTERNS); IPR007087 (PROSITE_PATTERNS); IPR007087 (PROSITE_PATTERNS); IPR007087 (PROSITE_PATTERNS); IPR007087 (PROSITE_PATTERNS); IPR007087 (PROSITE_PATTERNS); IPR007087 (PROSITE_PATTERNS); IPR007087 (PROSITE_PROFILES); IPR007087 (PROSITE_PROFILES); IPR007087 (PROSITE_PROFILES); IPR007087 (PROSITE_PROFILES); IPR007087 (PROSITE_PROFILES); IPR007087 (PROSITE_PROFILES); IPR007087 (PROSITE_PROFILES); IPR007087 (PROSITE_PROFILES); IPR007087 (PROSITE_PROFILES); SSF57667 (SUPERFAMILY); SSF57667 (SUPERFAMILY); SSF57667 (SUPERFAMILY); SSF57667 (SUPERFAMILY); SSF57667 (SUPERFAMILY); SSF57667 (SUPERFAMILY)

1656	493030_length_2095_cvg_6.5_tip_1_3	86	probable chitinase 3	699	3.08E-133	56.50%	0.266	IPR011583 (SMART); IPR002557 (SMART); IPR002557 (PFAM); IPR001223 (PFAM); IPR013781 (G3DSA:3.20.20.GENE3D); IPR029070 (G3DSA:3.10.50.GENE3D); IPR002557 (G3DSA:2.170.140.GENE3D); PTHR11177:SF112 (PANTHER); PTHR11177 (PANTHER); IPR001579 (PROSITE_PATTERNS); CYTOPLASMIC_DOMAIN (PHOBIUS); NON_CYTOPLASMIC_DOMAIN (PHOBIUS); TRANSMEMBRANE (PHOBIUS); NON_CYTOPLASMIC_DOMAIN (PHOBIUS); TRANSMEMBRANE (PHOBIUS); TRANSMEMBRANE (PHOBIUS); CYTOPLASMIC_DOMAIN (PHOBIUS); IPR002557 (PROSITE_PROFILES); IPR017853 (SUPERFAMILY); IPR002557 (SUPERFAMILY); IPR029070 (SUPERFAMILY); TMhelix (TMHMM); TMhelix (TMHMM)
1658	493048_length_2098_cvg_62.9_tip_1_4	5871	heat shock 70 kda protein cognate 3	699	0	95.00%	0.102	Coil (COILS); Coil (COILS); IPR013126 (PRINTS); IPR029047 (G3DSA:2.60.34.GENE3D); G3DSA:3.30.420.40 (GENE3D); G3DSA:3.90.640.10 (GENE3D); G3DSA:3.30.420.40 (GENE3D); IPR029048 (G3DSA:1.20.1270.GENE3D); IPR013126 (PFAM); PTHR19375:SF157 (PANTHER); PTHR19375 (PANTHER); IPR018181 (PROSITE_PATTERNS); IPR018181 (PROSITE_PATTERNS); NON_CYTOPLASMIC_DOMAIN (PHOBIUS); CYTOPLASMIC_DOMAIN (PHOBIUS); TRANSMEMBRANE (PHOBIUS); SSF53067 (SUPERFAMILY); SSF53067 (SUPERFAMILY); IPR029047 (SUPERFAMILY); IPR029048 (SUPERFAMILY); TMhelix (TMHMM); TMhelix (TMHMM)
1659	493056_length_2099_cvg_8.0_tip_1_2	86	mannosyl-oligosaccharide -alpha-mannosidase ia isoform x1	699	1.32E-53	87.40%	0.109	IPR001382 (PRINTS); IPR001382 (PFAM); IPR001382 (G3DSA:1.50.10.GENE3D); IPR001382 (PANTHER); PTHR11742:SF6 (PANTHER); NON_CYTOPLASMIC_DOMAIN (PHOBIUS); TRANSMEMBRANE (PHOBIUS); TRANSMEMBRANE (PHOBIUS); NON_CYTOPLASMIC_DOMAIN (PHOBIUS); NON_CYTOPLASMIC_DOMAIN (PHOBIUS); CYTOPLASMIC_DOMAIN (PHOBIUS); TRANSMEMBRANE (PHOBIUS); TRANSMEMBRANE (PHOBIUS); CYTOPLASMIC_DOMAIN (PHOBIUS); IPR001382 (SUPERFAMILY); TMhelix (TMHMM)
1663	493190_length_2123_cvg_3.0_tip_1_1	39	zinc finger protein 850-like isoform x1	699	2.15E-32	54.50%	0.103	IPR015880 (SMART); IPR013087 (G3DSA:3.30.160.GENE3D); PF13465 (PFAM); IPR013087 (G3DSA:3.30.160.GENE3D); IPR013087 (G3DSA:3.30.160.GENE3D); IPR007087 (PFAM); PTHR24409 (PANTHER); PTHR24409:SF14 (PANTHER); IPR007087 (PROSITE_PATTERNS); IPR007087 (PROSITE_PATTERNS); IPR007087 (PROSITE_PATTERNS); IPR007087 (PROSITE_PATTERNS); IPR007087 (PROSITE_PATTERNS); IPR007087 (PROSITE_PATTERNS); IPR007527 (PROSITE_PROFILES); IPR007087 (PROSITE_PROFILES); IPR007087 (PROSITE_PROFILES); IPR007087 (PROSITE_PROFILES); IPR007087 (PROSITE_PROFILES); IPR007087 (PROSITE_PROFILES); SSF57667 (SUPERFAMILY); SSF57667 (SUPERFAMILY); SSF57667 (SUPERFAMILY)
1665	493218_length_2132_cvg_4.2_tip_1_2	45	neural cell adhesion molecule 1-b	702	1.88E-71	57.20%	0.103	IPR003599 (SMART); IPR003961 (SMART); IPR003598 (SMART); IPR013098 (PFAM); IPR013783 (G3DSA:2.60.40.GENE3D); IPR003961 (PFAM); IPR013783 (G3DSA:2.60.40.GENE3D); PTHR19831 (PANTHER); PTHR19831:SF48 (PANTHER); NON_CYTOPLASMIC_DOMAIN (PHOBIUS); NON_CYTOPLASMIC_DOMAIN (PHOBIUS); TRANSMEMBRANE (PHOBIUS); TRANSMEMBRANE (PHOBIUS); CYTOPLASMIC_DOMAIN (PHOBIUS); TRANSMEMBRANE (PHOBIUS); CYTOPLASMIC_DOMAIN (PHOBIUS); TRANSMEMBRANE (PHOBIUS); NON_CYTOPLASMIC_DOMAIN (PHOBIUS); IPR007110 (PROSITE_PROFILES); IPR003961 (PROSITE_PROFILES); IPR007110 (PROSITE_PROFILES); IPR003961 (SUPERFAMILY); SSF48726 (SUPERFAMILY); SSF48726 (SUPERFAMILY); TMhelix (TMHMM); TMhelix (TMHMM); TMhelix (TMHMM)
1660	493102_length_2109_cvg_28.7_tip_1_0	306	dehydrogenase reductase sdr family member 11-like	703	3.58E-111	77.10%	0.165	IPR002347 (PRINTS); IPR002198 (PRINTS); IPR013968 (SMART); IPR002198 (PFAM); IPR016040 (G3DSA:3.40.50.GENE3D); PTHR24322 (PANTHER); PTHR24322:SF78 (PANTHER); IPR020904 (PROSITE_PATTERNS); TRANSMEMBRANE (PHOBIUS); CYTOPLASMIC_DOMAIN (PHOBIUS); NON_CYTOPLASMIC_DOMAIN (PHOBIUS); CYTOPLASMIC_DOMAIN (PHOBIUS); CYTOPLASMIC_DOMAIN (PHOBIUS); TRANSMEMBRANE (PHOBIUS); NON_CYTOPLASMIC_DOMAIN (PHOBIUS); TRANSMEMBRANE (PHOBIUS); CYTOPLASMIC_DOMAIN (PHOBIUS); TRANSMEMBRANE (PHOBIUS); NON_CYTOPLASMIC_DOMAIN (PHOBIUS); TRANSMEMBRANE (PHOBIUS); TRANSMEMBRANE (PHOBIUS); SSF51735 (SUPERFAMILY); TMhelix (TMHMM); TMhelix (TMHMM); TMhelix (TMHMM); TMhelix (TMHMM); TMhelix (TMHMM)
1662	493162_length_2121_cvg_2.0_tip_1_5	27	glutaminyl-peptide cyclotransferase	707	2.90E-39	68.60%	0.133	IPR007484 (PFAM); G3DSA:3.40.630.10 (GENE3D); PTHR12283 (PANTHER); SSF53187 (SUPERFAMILY)

1667	493264_length_2142_cvg_13.7_tip_1_3	121	katanin p60 atpase-containing subunit a-like 1	707	0	82.00%	0.528 Y	IPR003593 (SMART); IPR027417 (G3DSA:3.40.50.GENE3D); G3DSA:1.10.8.60 (GENE3D); IPR003959 (PFAM); IPR015415 (PFAM); PTHR23074:SF65 (PANTHER); PTHR23074 (PANTHER); IPR003960 (PROSITE_PATTERNS); CYTOPLASMIC_DOMAIN (PHOBIUS); TRANSMEMBRANE (PHOBIUS); TRANSMEMBRANE (PHOBIUS); SIGNAL_PEPTIDE_H_REGION (PHOBIUS); SIGNAL_PEPTIDE (PHOBIUS); NON_CYTOPLASMIC_DOMAIN (PHOBIUS); TRANSMEMBRANE (PHOBIUS); SIGNAL_PEPTIDE_N_REGION (PHOBIUS); CYTOPLASMIC_DOMAIN (PHOBIUS); NON_CYTOPLASMIC_DOMAIN (PHOBIUS); SIGNAL_PEPTIDE_C_REGION (PHOBIUS); IPR028596 (HAMAP); SignalP-noTM (SIGNALP_EUK); IPR027417 (SUPERFAMILY); TMhelix (TMHMM); TMhelix (TMHMM)
1664	493194_length_2124_cvg_11.2_tip_1_2	148	zinc finger protein 699-like	708	1.48E-55	56.90%	0.1	IPR015880 (SMART); PF13894 (PFAM); IPR013087 (G3DSA:3.30.160.GENE3D); PF13465 (PFAM); IPR013087 (G3DSA:3.30.160.GENE3D); IPR013087 (G3DSA:3.30.160.GENE3D); IPR007087 (PFAM); PTHR24375 (PANTHER); IPR007087 (PROSITE_PATTERNS); IPR007087 (PROSITE_PATTERNS); IPR007087 (PROSITE_PATTERNS); IPR007087 (PROSITE_PATTERNS); IPR007087 (PROSITE_PATTERNS); IPR007087 (PROSITE_PATTERNS); IPR007087 (PROSITE_PATTERNS); IPR007087 (PROSITE_PATTERNS); IPR007087 (PROSITE_PATTERNS); IPR007087 (PROSITE_PATTERNS); IPR007087 (PROSITE_PATTERNS); IPR007087 (PROSITE_PROFILES); IPR007087 (PROSITE_PROFILES); IPR007087 (PROSITE_PROFILES); IPR007087 (PROSITE_PROFILES); IPR007087 (PROSITE_PROFILES); IPR007087 (PROSITE_PROFILES); IPR007087 (PROSITE_PROFILES); IPR007087 (PROSITE_PROFILES); IPR007087 (PROSITE_PROFILES); IPR007087 (PROSITE_PROFILES); SSF57667 (SUPERFAMILY); SSF57667 (SUPERFAMILY); SSF57667 (SUPERFAMILY); SSF57667 (SUPERFAMILY); SSF57667 (SUPERFAMILY)
1666	493224_length_2132_cvg_7.0_tip_1_3	81	cyclin-dependent kinase 14 isoform x2	711	0	90.00%	0.1	IPR002290 (SMART); IPR000719 (PFAM); G3DSA:3.30.200.20 (GENE3D); G3DSA:1.10.510.10 (GENE3D); PTHR24056:SF155 (PANTHER); PTHR24056 (PANTHER); IPR008271 (PROSITE_PATTERNS); IPR017441 (PROSITE_PATTERNS); TRANSMEMBRANE (PHOBIUS); CYTOPLASMIC_DOMAIN (PHOBIUS); NON_CYTOPLASMIC_DOMAIN (PHOBIUS); NON_CYTOPLASMIC_DOMAIN (PHOBIUS); CYTOPLASMIC_DOMAIN (PHOBIUS); TRANSMEMBRANE (PHOBIUS); TRANSMEMBRANE (PHOBIUS); IPR000719 (PROSITE_PROFILES); IPR011009 (SUPERFAMILY); TMhelix (TMHMM); TMhelix (TMHMM); TMhelix (TMHMM)
1668	493286_length_2146_cvg_5.6_tip_1_2	62	n-acetylgalactosamine-6-sulfatase isoform x1	715	0	70.50%	0.097	IPR000917 (PFAM); PF14707 (PFAM); IPR017849 (G3DSA:3.40.720.GENE3D); IPR017849 (G3DSA:3.40.720.GENE3D); G3DSA:3.30.1120.10 (GENE3D); PTHR10342 (PANTHER); PTHR10342:SF27 (PANTHER); IPR024607 (PROSITE_PATTERNS); NON_CYTOPLASMIC_DOMAIN (PHOBIUS); CYTOPLASMIC_DOMAIN (PHOBIUS); NON_CYTOPLASMIC_DOMAIN (PHOBIUS); CYTOPLASMIC_DOMAIN (PHOBIUS); CYTOPLASMIC_DOMAIN (PHOBIUS); TRANSMEMBRANE (PHOBIUS); TRANSMEMBRANE (PHOBIUS); NON_CYTOPLASMIC_DOMAIN (PHOBIUS); TRANSMEMBRANE (PHOBIUS); TRANSMEMBRANE (PHOBIUS); TRANSMEMBRANE (PHOBIUS); IPR017850 (SUPERFAMILY)
1669	493320_length_2154_cvg_65.9_tip_1_2	3673	angiotensin-converting enzyme-like	718	0	71.20%	0.627 Y	IPR001548 (PRINTS); IPR001548 (PFAM); IPR001548 (PANTHER); NON_CYTOPLASMIC_DOMAIN (PHOBIUS); SIGNAL_PEPTIDE_C_REGION (PHOBIUS); SIGNAL_PEPTIDE (PHOBIUS); SIGNAL_PEPTIDE_H_REGION (PHOBIUS); SIGNAL_PEPTIDE_N_REGION (PHOBIUS); SignalP-noTM (SIGNALP_EUK); SSF55486 (SUPERFAMILY)
1676	493462_length_2185_cvg_17.3_tip_1_2	199	lethal giant larvae protein homolog 1-like isoform x5	720	0	75.60%	0.173	IPR000664 (PRINTS); IPR001680 (SMART); IPR015943 (G3DSA:2.130.10.GENE3D); IPR013577 (PFAM); PTHR10241 (PANTHER); PTHR10241:SF28 (PANTHER); IPR019775 (PROSITE_PATTERNS); IPR019775 (PROSITE_PATTERNS); CYTOPLASMIC_DOMAIN (PHOBIUS); NON_CYTOPLASMIC_DOMAIN (PHOBIUS); TRANSMEMBRANE (PHOBIUS); IPR017986 (PROSITE_PROFILES); IPR001680 (PROSITE_PROFILES); IPR017986 (SUPERFAMILY); IPR017986 (SUPERFAMILY); TMhelix (TMHMM)
1670	493358_length_2161_cvg_58.9_tip_1_3	645	muscle m-line assembly protein unc-89	721	3.33E-110	78.10%	0.097	IPR000719 (PFAM); G3DSA:3.30.200.20 (GENE3D); G3DSA:1.10.510.10 (GENE3D); IPR020675 (PANTHER); PTHR22964:SF45 (PANTHER); IPR000719 (PROSITE_PROFILES); IPR011009 (SUPERFAMILY)

1671	493376_length_2164_cvg_67.1_tip_1_4	879	serine-threonine kinase receptor-associated protein	721	0	85.50%	0.115	IPR001680 (SMART); IPR001680 (PFAM); IPR015943 (G3DSA:2.130.10.GENE3D); PTHR19877 (PANTHER); IPR019775 (PROSITE_PATTERNS); TRANSMEMBRANE (PHOBIUS); TRANSMEMBRANE (PHOBIUS); CYTOPLASMIC_DOMAIN (PHOBIUS); NON_CYTOPLASMIC_DOMAIN (PHOBIUS); NON_CYTOPLASMIC_DOMAIN (PHOBIUS); TRANSMEMBRANE (PHOBIUS); CYTOPLASMIC_DOMAIN (PHOBIUS); IPR001680 (PROSITE_PROFILES); IPR017986 (PROSITE_PROFILES); IPR001680 (PROSITE_PROFILES); IPR001680 (PROSITE_PROFILES); IPR001680 (PROSITE_PROFILES); IPR017986 (SUPERFAMILY); TMhelix (TMHMM); TMhelix (TMHMM)
1672	493384_length_2167_cvg_3.0_tip_1_2	39	serine proteinase stubble	722	5.05E-19	89.30%	0.125	IPR001254 (PFAM); G3DSA:2.40.10.10 (GENE3D); PTHR24256:SF1 (PANTHER); PTHR24256 (PANTHER); TRANSMEMBRANE (PHOBIUS); CYTOPLASMIC_DOMAIN (PHOBIUS); NON_CYTOPLASMIC_DOMAIN (PHOBIUS); TRANSMEMBRANE (PHOBIUS); CYTOPLASMIC_DOMAIN (PHOBIUS); TRANSMEMBRANE (PHOBIUS); NON_CYTOPLASMIC_DOMAIN (PHOBIUS); IPR001254 (PROSITE_PROFILES); IPR009003 (SUPERFAMILY); TMhelix (TMHMM); TMhelix (TMHMM); TMhelix (TMHMM); TMhelix (TMHMM)
1673	493388_length_2169_cvg_11.9_tip_1_5	109	glutamate-rich wd repeat-containing protein 1	723	0	72.40%	0.107	IPR020472 (PRINTS); IPR001680 (SMART); IPR015943 (G3DSA:2.130.10.GENE3D); IPR001680 (PFAM); IPR022052 (PFAM); PTHR22850 (PANTHER); PTHR22850:SF6 (PANTHER); IPR019775 (PROSITE_PATTERNS); TRANSMEMBRANE (PHOBIUS); CYTOPLASMIC_DOMAIN (PHOBIUS); CYTOPLASMIC_DOMAIN (PHOBIUS); TRANSMEMBRANE (PHOBIUS); NON_CYTOPLASMIC_DOMAIN (PHOBIUS); NON_CYTOPLASMIC_DOMAIN (PHOBIUS); TRANSMEMBRANE (PHOBIUS); IPR001680 (PROSITE_PROFILES); IPR001680 (PROSITE_PROFILES); IPR017986 (PROSITE_PROFILES); IPR001680 (PROSITE_PROFILES); IPR017986 (SUPERFAMILY)
1674	493404_length_2171_cvg_11.7_tip_1_0	148	cadherin-89d isoform x1	724	1.25E-13	44.80%	0.506 Y	IPR002126 (PRINTS); IPR002126 (SMART); IPR002126 (PFAM); IPR002126 (G3DSA:2.60.40.GENE3D); IPR002126 (G3DSA:2.60.40.GENE3D); PTHR24027 (PANTHER); CYTOPLASMIC_DOMAIN (PHOBIUS); TRANSMEMBRANE (PHOBIUS); NON_CYTOPLASMIC_DOMAIN (PHOBIUS); TRANSMEMBRANE (PHOBIUS); NON_CYTOPLASMIC_DOMAIN (PHOBIUS); CYTOPLASMIC_DOMAIN (PHOBIUS); TRANSMEMBRANE (PHOBIUS); IPR002126 (PROSITE_PROFILES); SignalP-noTM (SIGNALP_EUK); IPR015919 (SUPERFAMILY); IPR015919 (SUPERFAMILY); TMhelix (TMHMM); TMhelix (TMHMM); TMhelix (TMHMM)
1682	493534_length_2202_cvg_8.7_tip_1_0	106	abhydrolase domain-containing protein 16a-like	726	0	73.10%	0.117	IPR029059 (PFAM); IPR029058 (G3DSA:3.40.50.GENE3D); PTHR12277:SF44 (PANTHER); PTHR12277 (PANTHER); IPR029058 (SUPERFAMILY)
1684	493548_length_2205_cvg_6.4_tip_1_5	72	von willebrand factor a domain-containing protein partial	726	0	70.80%	0.11	Coil (COILS); IPR002035 (SMART); PF13519 (PFAM); PTHR21610:SF9 (PANTHER); PTHR21610 (PANTHER); IPR002035 (PROSITE_PROFILES); IPR002035 (SUPERFAMILY)
1675	493456_length_2184_cvg_4.1_tip_1_5	42	fidgetin-like protein 1	728	0	61.30%	0.115	IPR003593 (SMART); G3DSA:1.10.8.60 (GENE3D); IPR027417 (G3DSA:3.40.50.GENE3D); IPR003959 (PFAM); IPR015415 (PFAM); PTHR23074:SF75 (PANTHER); PTHR23074 (PANTHER); IPR003960 (PROSITE_PATTERNS); IPR027417 (SUPERFAMILY)
1677	493504_length_2196_cvg_14.2_tip_1_3	142	dna polymerase iii subunit gamma tau	732	2.68E-12	60.20%	0.115	NON_CYTOPLASMIC_DOMAIN (PHOBIUS); CYTOPLASMIC_DOMAIN (PHOBIUS); TRANSMEMBRANE (PHOBIUS)
1678	493504_length_2196_cvg_14.2_tip_1_5	142	gastrula zinc finger	732	3.76E-52	55.60%	0.131	IPR015880 (SMART); IPR013087 (G3DSA:3.30.160.GENE3D); IPR013087 (G3DSA:3.30.160.GENE3D); PF13894 (PFAM); PF13465 (PFAM); IPR007087 (PFAM); IPR013087 (G3DSA:3.30.160.GENE3D); IPR013087 (G3DSA:3.30.160.GENE3D); PTHR24409:SF14 (PANTHER); PTHR24409 (PANTHER); IPR007087 (PROSITE_PATTERNS); IPR007087 (PROSITE_PATTERNS); IPR007087 (PROSITE_PATTERNS); IPR007087 (PROSITE_PATTERNS); IPR007087 (PROSITE_PATTERNS); IPR007087 (PROSITE_PATTERNS); IPR007087 (PROSITE_PATTERNS); IPR007087 (PROSITE_PATTERNS); IPR007087 (PROSITE_PATTERNS); IPR007087 (PROSITE_PATTERNS); IPR007087 (PROSITE_PROFILES); IPR007087 (PROSITE_PROFILES); IPR007087 (PROSITE_PROFILES); IPR007087 (PROSITE_PROFILES); IPR007087 (PROSITE_PROFILES); IPR007087 (PROSITE_PROFILES); IPR007087 (PROSITE_PROFILES); IPR007087 (PROSITE_PROFILES); IPR007087 (PROSITE_PROFILES); IPR007087 (PROSITE_PROFILES); SSF57667 (SUPERFAMILY); SSF57667 (SUPERFAMILY); SSF57667 (SUPERFAMILY); SSF57667 (SUPERFAMILY); SSF57667 (SUPERFAMILY)

1679	493508_length_2197_cvg_10.8_tip_1_1	115	cyclin-dependent kinase 9-like	732	0	93.40%	0.109		IPR002290 (SMART); IPR000719 (PFAM); G3DSA:1.10.510.10 (GENE3D); G3DSA:3.30.200.20 (GENE3D); PTHR24056 (PANTHER); PTHR24056:SF158 (PANTHER); IPR008271 (PROSITE_PATTERNS); CYTOPLASMIC_DOMAIN (PHOBIUS); TRANSMEMBRANE (PHOBIUS); NON_CYTOPLASMIC_DOMAIN (PHOBIUS); TRANSMEMBRANE (PHOBIUS); NON_CYTOPLASMIC_DOMAIN (PHOBIUS); CYTOPLASMIC_DOMAIN (PHOBIUS); TRANSMEMBRANE (PHOBIUS); TRANSMEMBRANE (PHOBIUS); NON_CYTOPLASMIC_DOMAIN (PHOBIUS); IPR000719 (PROSITE_PROFILES); IPR011009 (SUPERFAMILY); TMhelix (TMHMM); TMhelix (TMHMM); TMhelix (TMHMM); TMhelix (TMHMM)
1688	493638_length_2224_cvg_6.3_tip_1_2	109	krueppel-like factor 3 isoform x1	732	1.26E-61	71.20%	0.139		IPR015880 (SMART); PF13465 (PFAM); IPR013087 (G3DSA:3.30.160.GENE3D); IPR013087 (G3DSA:3.30.160.GENE3D); PTHR23223 (PANTHER); PTHR23223:SF132 (PANTHER); IPR007087 (PROSITE_PATTERNS); IPR007087 (PROSITE_PATTERNS); IPR007087 (PROSITE_PATTERNS); TRANSMEMBRANE (PHOBIUS); CYTOPLASMIC_DOMAIN (PHOBIUS); NON_CYTOPLASMIC_DOMAIN (PHOBIUS); IPR007087 (PROSITE_PROFILES); IPR007087 (PROSITE_PROFILES); IPR007087 (PROSITE_PROFILES); SSF57667 (SUPERFAMILY); SSF57667 (SUPERFAMILY)
1692	493678_length_2235_cvg_58.9_tip_1_1	722	vegetative cell wall protein gp1-like	732	4.28E-14	48.90%	0.162		NON_CYTOPLASMIC_DOMAIN (PHOBIUS); TRANSMEMBRANE (PHOBIUS); NON_CYTOPLASMIC_DOMAIN (PHOBIUS); CYTOPLASMIC_DOMAIN (PHOBIUS); TRANSMEMBRANE (PHOBIUS); NON_CYTOPLASMIC_DOMAIN (PHOBIUS); TRANSMEMBRANE (PHOBIUS); CYTOPLASMIC_DOMAIN (PHOBIUS); CYTOPLASMIC_DOMAIN (PHOBIUS); CYTOPLASMIC_DOMAIN (PHOBIUS); CYTOPLASMIC_DOMAIN (PHOBIUS); TRANSMEMBRANE (PHOBIUS); TRANSMEMBRANE (PHOBIUS); TRANSMEMBRANE (PHOBIUS); TRANSMEMBRANE (PHOBIUS); TRANSMEMBRANE (PHOBIUS); NON_CYTOPLASMIC_DOMAIN (PHOBIUS); TMhelix (TMHMM); TMhelix (TMHMM); TMhelix (TMHMM)
1693	493678_length_2235_cvg_58.9_tip_1_3	722	muc19_human ame: full=mucin-19 short=muc-19 flags: partial	732	1.21E-33	50.70%	0.099		CYTOPLASMIC_DOMAIN (PHOBIUS); NON_CYTOPLASMIC_DOMAIN (PHOBIUS); TRANSMEMBRANE (PHOBIUS); TMhelix (TMHMM)
1680	493512_length_2198_cvg_13.8_tip_1_0	150	nicotinic acetylcholine receptor subunit alpha10	733	6.59E-105	59.10%	0.675	Y	IPR006201 (PRINTS); IPR006029 (PFAM); IPR006202 (PFAM); IPR027361 (G3DSA:1.20.120.GENE3D); IPR006202 (G3DSA:2.70.170.GENE3D); PTHR18945:SF490 (PANTHER); IPR006201 (PANTHER); CYTOPLASMIC_DOMAIN (PHOBIUS); SIGNAL_PEPTIDE_C_REGION (PHOBIUS); NON_CYTOPLASMIC_DOMAIN (PHOBIUS); TRANSMEMBRANE (PHOBIUS); TRANSMEMBRANE (PHOBIUS); CYTOPLASMIC_DOMAIN (PHOBIUS); TRANSMEMBRANE (PHOBIUS); TRANSMEMBRANE (PHOBIUS); SIGNAL_PEPTIDE_N_REGION (PHOBIUS); NON_CYTOPLASMIC_DOMAIN (PHOBIUS); TRANSMEMBRANE (PHOBIUS); CYTOPLASMIC_DOMAIN (PHOBIUS); TRANSMEMBRANE (PHOBIUS); CYTOPLASMIC_DOMAIN (PHOBIUS); TRANSMEMBRANE (PHOBIUS); SIGNAL_PEPTIDE (PHOBIUS); TRANSMEMBRANE (PHOBIUS); CYTOPLASMIC_DOMAIN (PHOBIUS); NON_CYTOPLASMIC_DOMAIN (PHOBIUS); TRANSMEMBRANE (PHOBIUS); SIGNAL_PEPTIDE_H_REGION (PHOBIUS); NON_CYTOPLASMIC_DOMAIN (PHOBIUS); NON_CYTOPLASMIC_DOMAIN (PHOBIUS); CYTOPLASMIC_DOMAIN (PHOBIUS); NON_CYTOPLASMIC_DOMAIN (PHOBIUS); TRANSMEMBRANE (PHOBIUS); TRANSMEMBRANE (PHOBIUS); SignalP-noTM (SIGNALP_EUK); IPR006202 (SUPERFAMILY); IPR006029 (SUPERFAMILY); TMhelix (TMHMM); TMhelix (TMHMM); TMhelix (TMHMM); TMhelix (TMHMM); TMhelix (TMHMM); TMhelix (TMHMM); TMhelix (TMHMM); TMhelix (TMHMM); TMhelix (TMHMM); TMhelix (TMHMM)
1689	493640_length_2225_cvg_28.7_tip_1_1	253	dnaj homolog subfamily c member 7	733	0	74.80%	0.109		IPR001623 (PRINTS); IPR019734 (SMART); IPR001623 (SMART); PF13414 (PFAM); IPR019734 (PFAM); IPR001623 (PFAM); IPR013105 (PFAM); IPR001623 (G3DSA:1.10.287.GENE3D); IPR011990 (G3DSA:1.25.40.GENE3D); IPR011990 (G3DSA:1.25.40.GENE3D); PTHR24078 (PANTHER); PTHR24078:SF140 (PANTHER); IPR018253 (PROSITE_PATTERNS); IPR019734 (PROSITE_PROFILES); IPR019734 (PROSITE_PROFILES); IPR013026 (PROSITE_PROFILES); IPR019734 (PROSITE_PROFILES); IPR019734 (PROSITE_PROFILES); IPR019734 (PROSITE_PROFILES); IPR019734 (PROSITE_PROFILES); IPR001623 (PROSITE_PROFILES); IPR019734 (PROSITE_PROFILES); IPR001623 (SUPERFAMILY); SSF48452 (SUPERFAMILY); SSF48452 (SUPERFAMILY)

1681	493522_length_2200_cvg_4.7_tip_1_0	63	zinc finger protein 658-like isoform x1	734	5.98E-34	55.10%	0.113	IPR000210 (SMART); IPR015880 (SMART); IPR013069 (PFAM); IPR013087 (G3DSA:3.30.160.GENE3D); G3DSA:3.30.710.10 (GENE3D); IPR013087 (G3DSA:3.30.160.GENE3D); IPR013087 (G3DSA:3.30.160.GENE3D); IPR007087 (PFAM); PF13465 (PFAM); PTHR24409:SF14 (PANTHER); PTHR24409 (PANTHER); IPR007087 (PROSITE_PATTERNS); IPR007087 (PROSITE_PATTERNS); IPR007087 (PROSITE_PATTERNS); IPR007087 (PROSITE_PATTERNS); IPR007087 (PROSITE_PATTERNS); IPR007087 (PROSITE_PATTERNS); IPR000210 (PROSITE_PROFILES); IPR007087 (PROSITE_PROFILES); IPR007087 (PROSITE_PROFILES); IPR007087 (PROSITE_PROFILES); IPR007087 (PROSITE_PROFILES); IPR007087 (PROSITE_PROFILES); IPR007087 (PROSITE_PROFILES); SSF57667 (SUPERFAMILY); IPR011333 (SUPERFAMILY); SSF57667 (SUPERFAMILY); SSF57667 (SUPERFAMILY)
1683	493538_length_2203_cvg_62.7_tip_1_2	1260	tyrosine--trna cytoplasmic	734	0	79.80%	0.133	IPR002307 (PRINTS); IPR012340 (G3DSA:2.40.50.GENE3D); IPR002307 (TIGRFAM); IPR002547 (PFAM); IPR002305 (PFAM); IPR014729 (G3DSA:3.40.50.GENE3D); G3DSA:1.10.240.10 (GENE3D); PTHR11946 (PANTHER); IPR002547 (PROSITE_PROFILES); IPR012340 (SUPERFAMILY); IPR010987 (SUPERFAMILY); SSF52374 (SUPERFAMILY)
1685	493568_length_2208_cvg_35.5_tip_1_2	299	alpha-mannosidase 2	736	1.40E-160	58.80%	0.1	IPR011682 (PFAM); IPR013780 (G3DSA:2.60.40.GENE3D); G3DSA:1.10.287.530 (GENE3D); G3DSA:2.70.98.30 (GENE3D); IPR015341 (PFAM); PTHR11607:SF4 (PANTHER); PTHR11607 (PANTHER); CYTOPLASMIC_DOMAIN (PHOBIUS); NON_CYTOPLASMIC_DOMAIN (PHOBIUS); TRANSMEMBRANE (PHOBIUS); IPR028995 (SUPERFAMILY); IPR011013 (SUPERFAMILY); TMhelix (TMHMM)
1702	493750_length_2257_cvg_6.7_tip_1_5	644	zinc finger protein 239-like isoform x2	738	2.65E-77	68.10%	0.141	IPR015880 (SMART); IPR013087 (G3DSA:3.30.160.GENE3D); IPR013087 (G3DSA:3.30.160.GENE3D); IPR013087 (G3DSA:3.30.160.GENE3D); IPR013087 (G3DSA:3.30.160.GENE3D); IPR007087 (PFAM); PF13465 (PFAM); PTHR24377 (PANTHER); IPR007087 (PROSITE_PATTERNS); IPR007087 (PROSITE_PATTERNS); IPR007087 (PROSITE_PATTERNS); IPR007087 (PROSITE_PATTERNS); IPR007087 (PROSITE_PATTERNS); IPR007087 (PROSITE_PATTERNS); IPR007087 (PROSITE_PATTERNS); IPR007087 (PROSITE_PATTERNS); CYTOPLASMIC_DOMAIN (PHOBIUS); NON_CYTOPLASMIC_DOMAIN (PHOBIUS); CYTOPLASMIC_DOMAIN (PHOBIUS); TRANSMEMBRANE (PHOBIUS); TRANSMEMBRANE (PHOBIUS); NON_CYTOPLASMIC_DOMAIN (PHOBIUS); TRANSMEMBRANE (PHOBIUS); NON_CYTOPLASMIC_DOMAIN (PHOBIUS); CYTOPLASMIC_DOMAIN (PHOBIUS); TRANSMEMBRANE (PHOBIUS); TRANSMEMBRANE (PHOBIUS); IPR007087 (PROSITE_PROFILES); IPR007087 (PROSITE_PROFILES); IPR007087 (PROSITE_PROFILES); IPR007087 (PROSITE_PROFILES); IPR007087 (PROSITE_PROFILES); IPR007087 (PROSITE_PROFILES); IPR007087 (PROSITE_PROFILES); IPR007087 (PROSITE_PROFILES); SSF57667 (SUPERFAMILY); SSF57667 (SUPERFAMILY); SSF57667 (SUPERFAMILY); SSF57667 (SUPERFAMILY); TMhelix (TMHMM); TMhelix (TMHMM); TMhelix (TMHMM)
1700	493742_length_2254_cvg_39.4_tip_1_2	425	serine threonine-protein phosphatase 2b catalytic subunit 2-like isoform x1	739	0	94.90%	0.106	IPR006186 (PRINTS); IPR006186 (SMART); IPR004843 (PFAM); IPR029052 (G3DSA:3.60.21.GENE3D); PTHR11668:SF186 (PANTHER); PTHR11668 (PANTHER); IPR006186 (PROSITE_PATTERNS); IPR029052 (SUPERFAMILY)
1686	493620_length_2221_cvg_16.8_tip_1_0	192	peptidylprolyl isomerase domain and wd repeat-containing protein 1	741	0	85.10%	0.109	IPR002130 (PRINTS); IPR001680 (SMART); IPR029000 (G3DSA:2.40.100.GENE3D); IPR001680 (PFAM); IPR015943 (G3DSA:2.130.10.GENE3D); IPR002130 (PFAM); PTHR11071:SF228 (PANTHER); IPR024936 (PANTHER); IPR002130 (PROSITE_PROFILES); IPR017986 (PROSITE_PROFILES); IPR029000 (SUPERFAMILY); IPR017986 (SUPERFAMILY)
1687	493632_length_2222_cvg_8.8_tip_1_0	92	pyrroline-5-carboxylate dehydrogenase	741	0	80.50%	0.314	IPR015590 (PFAM); IPR016163 (G3DSA:3.40.309.GENE3D); IPR016162 (G3DSA:3.40.605.GENE3D); IPR005931 (TIGRFAM); PTHR11699:SF18 (PANTHER); PTHR11699 (PANTHER); IPR016160 (PROSITE_PATTERNS); CYTOPLASMIC_DOMAIN (PHOBIUS); NON_CYTOPLASMIC_DOMAIN (PHOBIUS); TRANSMEMBRANE (PHOBIUS); TRANSMEMBRANE (PHOBIUS); NON_CYTOPLASMIC_DOMAIN (PHOBIUS); IPR016161 (SUPERFAMILY); TMhelix (TMHMM)

1690	493650_length_2230_cvg_52.7_tip_1_3	1148	adp-ribosylation factor 1	744	2.23E-126	98.70%	0.313		IPR006689 (PRINTS); IPR006687 (SMART); IPR024156 (SMART); IPR003579 (SMART); IPR006689 (PFAM); IPR027417 (G3DSA:3.40.50.GENE3D); IPR005225 (TIGRFAM); PTHR11711 (PANTHER); PTHR11711:SF118 (PANTHER); CYTOPLASMIC_DOMAIN (PHOBIUS); NON_CYTOPLASMIC_DOMAIN (PHOBIUS); TRANSMEMBRANE (PHOBIUS); NON_CYTOPLASMIC_DOMAIN (PHOBIUS); TRANSMEMBRANE (PHOBIUS); NON_CYTOPLASMIC_DOMAIN (PHOBIUS); TRANSMEMBRANE (PHOBIUS); NON_CYTOPLASMIC_DOMAIN (PHOBIUS); TRANSMEMBRANE (PHOBIUS); CYTOPLASMIC_DOMAIN (PHOBIUS); TRANSMEMBRANE (PHOBIUS); TRANSMEMBRANE (PHOBIUS); CYTOPLASMIC_DOMAIN (PHOBIUS); TRANSMEMBRANE (PHOBIUS); CYTOPLASMIC_DOMAIN (PHOBIUS); NON_CYTOPLASMIC_DOMAIN (PHOBIUS); TRANSMEMBRANE (PHOBIUS); IPR024156 (PROSITE_PROFILES); IPR027417 (SUPERFAMILY); TMhelix (TMHMM); TMhelix (TMHMM); TMhelix (TMHMM); TMhelix (TMHMM)
1691	493674_length_2234_cvg_3.6_tip_1_2	51	nephrin isoform x1	744	1.09E-158	65.40%	0.101		IPR003598 (SMART); IPR003599 (SMART); IPR013783 (G3DSA:2.60.40.GENE3D); IPR013783 (G3DSA:2.60.40.GENE3D); IPR013783 (G3DSA:2.60.40.GENE3D); IPR013162 (PFAM); IPR013098 (PFAM); PTHR11640:SF31 (PANTHER); PTHR11640 (PANTHER); IPR007110 (PROSITE_PROFILES); IPR007110 (PROSITE_PROFILES); IPR007110 (PROSITE_PROFILES); IPR007110 (PROSITE_PROFILES); IPR007110 (PROSITE_PROFILES); SSF48726 (SUPERFAMILY); SSF48726 (SUPERFAMILY); SSF48726 (SUPERFAMILY); SSF48726 (SUPERFAMILY); SSF48726 (SUPERFAMILY)
1699	493740_length_2254_cvg_26.6_tip_1_2	360	ectoderm-expressed 3	744	3.92E-154	57.30%	0.135		IPR001944 (PRINTS); IPR001944 (PFAM); IPR013781 (G3DSA:3.20.20.GENE3D); IPR008979 (G3DSA:2.60.120.GENE3D); IPR001944 (PANTHER); PTHR23421:SF13 (PANTHER); IPR019801 (PROSITE_PATTERNS); IPR017853 (SUPERFAMILY); IPR008979 (SUPERFAMILY)
1704	493768_length_2263_cvg_19.3_tip_1_5	224	coatomer subunit beta isoform x2	744	0	78.20%	0.129		IPR006692 (PFAM); PTHR19876 (PANTHER); PTHR19876:SF2 (PANTHER); IPR011044 (SUPERFAMILY)
1694	493680_length_2235_cvg_12.4_tip_1_2	122	zinc finger protein 77-like isoform x2	745	1.06E-29	56.30%	0.15		IPR015880 (SMART); IPR013087 (G3DSA:3.30.160.GENE3D); IPR013087 (G3DSA:3.30.160.GENE3D); IPR007087 (PFAM); PF13894 (PFAM); PTHR24409 (PANTHER); PTHR24409:SF14 (PANTHER); IPR007087 (PROSITE_PATTERNS); IPR007087 (PROSITE_PATTERNS); IPR007087 (PROSITE_PATTERNS); IPR007087 (PROSITE_PATTERNS); IPR007087 (PROSITE_PATTERNS); IPR007087 (PROSITE_PATTERNS); IPR007087 (PROSITE_PATTERNS); CYTOPLASMIC_DOMAIN (PHOBIUS); NON_CYTOPLASMIC_DOMAIN (PHOBIUS); TRANSMEMBRANE (PHOBIUS); TRANSMEMBRANE (PHOBIUS); CYTOPLASMIC_DOMAIN (PHOBIUS); TRANSMEMBRANE (PHOBIUS); NON_CYTOPLASMIC_DOMAIN (PHOBIUS); IPR007087 (PROSITE_PROFILES); IPR007087 (PROSITE_PROFILES); IPR007087 (PROSITE_PROFILES); IPR007087 (PROSITE_PROFILES); IPR007087 (PROSITE_PROFILES); IPR007087 (PROSITE_PROFILES); SSF57667 (SUPERFAMILY); SSF57667 (SUPERFAMILY); SSF57667 (SUPERFAMILY); SSF57667 (SUPERFAMILY)
1695	493686_length_2240_cvg_62.9_tip_1_4	6242	hemocyanin subunit type 1 precursor	747	0	75.50%	0.372	Y	IPR013788 (PRINTS); IPR005203 (G3DSA:2.60.40.GENE3D); IPR000896 (PFAM); IPR005204 (G3DSA:1.20.1370.GENE3D); IPR005204 (PFAM); IPR005203 (PFAM); IPR008922 (G3DSA:1.10.1280.GENE3D); IPR013788 (PANTHER); PTHR11511:SF5 (PANTHER); IPR013788 (PROSITE_PATTERNS); IPR013788 (PROSITE_PATTERNS); IPR002227 (PROSITE_PATTERNS); CYTOPLASMIC_DOMAIN (PHOBIUS); TRANSMEMBRANE (PHOBIUS); SIGNAL_PEPTIDE_N_REGION (PHOBIUS); SIGNAL_PEPTIDE (PHOBIUS); SIGNAL_PEPTIDE_H_REGION (PHOBIUS); SIGNAL_PEPTIDE_C_REGION (PHOBIUS); NON_CYTOPLASMIC_DOMAIN (PHOBIUS); IPR005204 (SUPERFAMILY); IPR008922 (SUPERFAMILY); IPR014756 (SUPERFAMILY)
1696	493706_length_2246_cvg_5.6_tip_1_1	66	lachesin-like isoform x1	749	2.91E-86	73.00%	0.32		IPR003598 (SMART); IPR003599 (SMART); IPR013783 (G3DSA:2.60.40.GENE3D); IPR013098 (PFAM); PTHR23279:SF12 (PANTHER); PTHR23279 (PANTHER); CYTOPLASMIC_DOMAIN (PHOBIUS); TRANSMEMBRANE (PHOBIUS); NON_CYTOPLASMIC_DOMAIN (PHOBIUS); IPR007110 (PROSITE_PROFILES); IPR007110 (PROSITE_PROFILES); SSF48726 (SUPERFAMILY); SSF48726 (SUPERFAMILY); TMhelix (TMHMM)

1697	493726_length_2251_cvg_10.8_tip_1_4	120	glycoside catalytic core	750	8.25E-94	54.00%	0.173		Coil (COILS); IPR001547 (PFAM); IPR013781 (G3DSA:3.20.20.GENE3D); PTHR31308 (PANTHER); CYTOPLASMIC_DOMAIN (PHOBIUS); NON_CYTOPLASMIC_DOMAIN (PHOBIUS); TRANSMEMBRANE (PHOBIUS); TRANSMEMBRANE (PHOBIUS); NON_CYTOPLASMIC_DOMAIN (PHOBIUS); TRANSMEMBRANE (PHOBIUS); CYTOPLASMIC_DOMAIN (PHOBIUS); IPR017853 (SUPERFAMILY)
1698	493738_length_2253_cvg_15.0_tip_1_5	155	venom carboxylesterase-6 isoform x2	751	2.24E-137	58.20%	0.909	Y	IPR029058 (G3DSA:3.40.50.GENE3D); IPR002018 (PFAM); PTHR11559 (PANTHER); IPR019826 (PROSITE_PATTERNS); IPR019819 (PROSITE_PATTERNS); SIGNAL_PEPTIDE_H_REGION (PHOBIUS); SIGNAL_PEPTIDE_C_REGION (PHOBIUS); CYTOPLASMIC_DOMAIN (PHOBIUS); NON_CYTOPLASMIC_DOMAIN (PHOBIUS); SIGNAL_PEPTIDE (PHOBIUS); SIGNAL_PEPTIDE_N_REGION (PHOBIUS); TRANSMEMBRANE (PHOBIUS); SignalP-noTM (SIGNALP_GRAM_NEGATIVE); SignalP-noTM (SIGNALP_EUK); SignalP-TM (SIGNALP_GRAM_POSITIVE); IPR029058 (SUPERFAMILY)
1701	493746_length_2255_cvg_4.0_tip_1_0	58	cytochrome p450 2j6-like	752	9.31E-105	56.00%	0.146		IPR001128 (PRINTS); IPR002401 (PRINTS); IPR001128 (PFAM); IPR001128 (G3DSA:1.10.630.GENE3D); PTHR24300 (PANTHER); IPR017972 (PROSITE_PATTERNS); TRANSMEMBRANE (PHOBIUS); CYTOPLASMIC_DOMAIN (PHOBIUS); CYTOPLASMIC_DOMAIN (PHOBIUS); NON_CYTOPLASMIC_DOMAIN (PHOBIUS); TRANSMEMBRANE (PHOBIUS); IPR001128 (SUPERFAMILY); TMhelix (TMHMM)
1703	493758_length_2261_cvg_19.4_tip_1_0	306	egf domain protein	754	1.65E-11	41.60%	0.467	Y	IPR002223 (PRINTS); IPR003645 (SMART); IPR002223 (SMART); IPR008197 (SMART); IPR002223 (PFAM); IPR008197 (G3DSA:4.10.75.GENE3D); IPR008197 (PFAM); IPR002223 (G3DSA:4.10.410.GENE3D); PTHR10083 (PANTHER); IPR020901 (PROSITE_PATTERNS); TRANSMEMBRANE (PHOBIUS); CYTOPLASMIC_DOMAIN (PHOBIUS); NON_CYTOPLASMIC_DOMAIN (PHOBIUS); CYTOPLASMIC_DOMAIN (PHOBIUS); TRANSMEMBRANE (PHOBIUS); IPR008197 (PROSITE_PROFILES); IPR008197 (PROSITE_PROFILES); IPR008197 (PROSITE_PROFILES); IPR008197 (PROSITE_PROFILES); IPR002223 (PROSITE_PROFILES); IPR008197 (PROSITE_PROFILES); IPR008197 (PROSITE_PROFILES); IPR002223 (SUPERFAMILY); IPR008197 (SUPERFAMILY); TMhelix (TMHMM); TMhelix (TMHMM)
1705	493778_length_2267_cvg_53.8_tip_1_0	567	utp--glucose-1-phosphate uridylyltransferase-like	756	0	84.40%	0.108		IPR002618 (PFAM); IPR029044 (G3DSA:3.90.550.GENE3D); IPR002618 (PANTHER); IPR016267 (PTHR11952:PANTHER); CYTOPLASMIC_DOMAIN (PHOBIUS); TRANSMEMBRANE (PHOBIUS); TRANSMEMBRANE (PHOBIUS); NON_CYTOPLASMIC_DOMAIN (PHOBIUS); TRANSMEMBRANE (PHOBIUS); CYTOPLASMIC_DOMAIN (PHOBIUS); NON_CYTOPLASMIC_DOMAIN (PHOBIUS); IPR029044 (SUPERFAMILY); TMhelix (TMHMM); TMhelix (TMHMM); TMhelix (TMHMM)
1706	493828_length_2280_cvg_32.5_tip_1_2	939	multiple inositol polyphosphate phosphatase 1	760	1.94E-142	61.20%	0.29		IPR029033 (G3DSA:3.40.50.GENE3D); IPR000560 (PFAM); PTHR20963 (PANTHER); PTHR20963:SF8 (PANTHER); IPR000560 (PROSITE_PATTERNS); NON_CYTOPLASMIC_DOMAIN (PHOBIUS); TRANSMEMBRANE (PHOBIUS); CYTOPLASMIC_DOMAIN (PHOBIUS); TRANSMEMBRANE (PHOBIUS); CYTOPLASMIC_DOMAIN (PHOBIUS); TRANSMEMBRANE (PHOBIUS); TRANSMEMBRANE (PHOBIUS); NON_CYTOPLASMIC_DOMAIN (PHOBIUS); NON_CYTOPLASMIC_DOMAIN (PHOBIUS); IPR029033 (SUPERFAMILY); TMhelix (TMHMM)
1707	493852_length_2287_cvg_3.7_tip_1_2	42	transforming growth factor beta superfamily signaling ligand	762	3.21E-87	54.20%	0.151		IPR001839 (SMART); IPR001111 (PFAM); IPR029034 (G3DSA:2.10.90.GENE3D); IPR001839 (PFAM); PTHR11848:SF131 (PANTHER); IPR015615 (PANTHER); IPR017948 (PROSITE_PATTERNS); TRANSMEMBRANE (PHOBIUS); NON_CYTOPLASMIC_DOMAIN (PHOBIUS); CYTOPLASMIC_DOMAIN (PHOBIUS); IPR001839 (PROSITE_PROFILES); IPR029034 (SUPERFAMILY); TMhelix (TMHMM); TMhelix (TMHMM)
1711	493940_length_2317_cvg_39.4_tip_1_5	443	myotrophin-like isoform 2	764	6.55E-53	84.20%	0.141		IPR002110 (SMART); IPR020683 (G3DSA:1.25.40.GENE3D); IPR020683 (PFAM); PTHR24152 (PANTHER); TRANSMEMBRANE (PHOBIUS); NON_CYTOPLASMIC_DOMAIN (PHOBIUS); CYTOPLASMIC_DOMAIN (PHOBIUS); TRANSMEMBRANE (PHOBIUS); NON_CYTOPLASMIC_DOMAIN (PHOBIUS); CYTOPLASMIC_DOMAIN (PHOBIUS); TRANSMEMBRANE (PHOBIUS); NON_CYTOPLASMIC_DOMAIN (PHOBIUS); TRANSMEMBRANE (PHOBIUS); TRANSMEMBRANE (PHOBIUS); CYTOPLASMIC_DOMAIN (PHOBIUS); NON_CYTOPLASMIC_DOMAIN (PHOBIUS); TRANSMEMBRANE (PHOBIUS); CYTOPLASMIC_DOMAIN (PHOBIUS); TRANSMEMBRANE (PHOBIUS); IPR002110 (PROSITE_PROFILES); IPR020683 (PROSITE_PROFILES); IPR020683 (SUPERFAMILY); TMhelix (TMHMM); TMhelix (TMHMM)

1713	493972_length_2326_cvg_48.4_tip_1_5	665	facilitated trehalose transporter tret1-like isoform x1	766	5.31E-111	58.80%	0.11	IPR003663 (PRINTS); G3DSA:1.20.1250.20 (GENE3D); IPR003663 (TIGRFAM); IPR005828 (PFAM); PTHR24063 (PANTHER); IPR005829 (PROSITE_PATTERNS); TRANSMEMBRANE (PHOBIUS); CYTOPLASMIC_DOMAIN (PHOBIUS); TRANSMEMBRANE (PHOBIUS); CYTOPLASMIC_DOMAIN (PHOBIUS); CYTOPLASMIC_DOMAIN (PHOBIUS); TRANSMEMBRANE (PHOBIUS); TRANSMEMBRANE (PHOBIUS); NON_CYTOPLASMIC_DOMAIN (PHOBIUS); TRANSMEMBRANE (PHOBIUS); CYTOPLASMIC_DOMAIN (PHOBIUS); NON_CYTOPLASMIC_DOMAIN (PHOBIUS); TRANSMEMBRANE (PHOBIUS); TRANSMEMBRANE (PHOBIUS); CYTOPLASMIC_DOMAIN (PHOBIUS); TRANSMEMBRANE (PHOBIUS); TRANSMEMBRANE (PHOBIUS); NON_CYTOPLASMIC_DOMAIN (PHOBIUS); NON_CYTOPLASMIC_DOMAIN (PHOBIUS); NON_CYTOPLASMIC_DOMAIN (PHOBIUS); TRANSMEMBRANE (PHOBIUS); TRANSMEMBRANE (PHOBIUS); CYTOPLASMIC_DOMAIN (PHOBIUS); NON_CYTOPLASMIC_DOMAIN (PHOBIUS); IPR020846 (PROSITE_PROFILES); SSF141571 (SUPERFAMILY); IPR020846 (SUPERFAMILY); TMhelix (TMHMM); TMhelix (TMHMM); TMhelix (TMHMM); TMhelix (TMHMM); TMhelix (TMHMM); TMhelix (TMHMM); TMhelix (TMHMM); TMhelix (TMHMM); TMhelix (TMHMM); TMhelix (TMHMM); TMhelix (TMHMM)
1708	493900_length_2307_cvg_50.2_tip_1_5	597	low quality protein: hemocytin	769	1.09E-79	47.10%	0.819 Y	IPR001846 (SMART); IPR001846 (PFAM); IPR014853 (PFAM); G3DSA:2.10.25.10 (GENE3D); PTHR11339 (PANTHER); PTHR11339:SF25 (PANTHER); IPR001846 (PROSITE_PROFILES); IPR001846 (PROSITE_PROFILES); SignalP-noTM (SIGNALP_EUK); IPR002919 (SUPERFAMILY)
1709	493908_length_2309_cvg_10.0_tip_1_2	141	neuropeptides capa receptor-like	769	1.38E-149	74.00%	0.327	Coil (COILS); IPR005390 (PRINTS); IPR000276 (PRINTS); IPR000276 (PFAM); G3DSA:1.20.1070.10 (GENE3D); PTHR24243 (PANTHER); PTHR24243:SF107 (PANTHER); IPR000276 (PROSITE_PATTERNS); TRANSMEMBRANE (PHOBIUS); TRANSMEMBRANE (PHOBIUS); TRANSMEMBRANE (PHOBIUS); CYTOPLASMIC_DOMAIN (PHOBIUS); TRANSMEMBRANE (PHOBIUS); NON_CYTOPLASMIC_DOMAIN (PHOBIUS); CYTOPLASMIC_DOMAIN (PHOBIUS); CYTOPLASMIC_DOMAIN (PHOBIUS); TRANSMEMBRANE (PHOBIUS); CYTOPLASMIC_DOMAIN (PHOBIUS); NON_CYTOPLASMIC_DOMAIN (PHOBIUS); NON_CYTOPLASMIC_DOMAIN (PHOBIUS); TRANSMEMBRANE (PHOBIUS); TRANSMEMBRANE (PHOBIUS); NON_CYTOPLASMIC_DOMAIN (PHOBIUS); IPR017452 (PROSITE_PROFILES); SSF81321 (SUPERFAMILY); TMhelix (TMHMM); TMhelix (TMHMM); TMhelix (TMHMM); TMhelix (TMHMM); TMhelix (TMHMM); TMhelix (TMHMM); TMhelix (TMHMM)
1710	493912_length_2309_cvg_3.2_tip_1_1	49	probable chitinase 3	770	4.44E-130	56.80%	0.106	IPR011583 (SMART); IPR002557 (SMART); IPR013781 (G3DSA:3.20.20.GENE3D); IPR002557 (PFAM); IPR029070 (G3DSA:3.10.50.GENE3D); IPR001223 (PFAM); IPR002557 (G3DSA:2.170.140.GENE3D); PTHR11177 (PANTHER); PTHR11177:SF112 (PANTHER); IPR001579 (PROSITE_PATTERNS); NON_CYTOPLASMIC_DOMAIN (PHOBIUS); TRANSMEMBRANE (PHOBIUS); CYTOPLASMIC_DOMAIN (PHOBIUS); CYTOPLASMIC_DOMAIN (PHOBIUS); TRANSMEMBRANE (PHOBIUS); TRANSMEMBRANE (PHOBIUS); TRANSMEMBRANE (PHOBIUS); NON_CYTOPLASMIC_DOMAIN (PHOBIUS); CYTOPLASMIC_DOMAIN (PHOBIUS); IPR002557 (PROSITE_PROFILES); IPR002557 (PROSITE_PROFILES); IPR002557 (SUPERFAMILY); IPR029070 (SUPERFAMILY); IPR017853 (SUPERFAMILY); IPR002557 (SUPERFAMILY); TMhelix (TMHMM); TMhelix (TMHMM)
1721	494088_length_2364_cvg_65.4_tip_1_5	46408	heat shock cognate protein 70	773	0	94.80%	0.179	Coil (COILS); IPR013126 (PRINTS); IPR029048 (G3DSA:1.20.1270.GENE3D); IPR029047 (G3DSA:2.60.34.GENE3D); G3DSA:3.30.420.40 (GENE3D); G3DSA:3.30.30.30 (GENE3D); G3DSA:3.30.420.40 (GENE3D); IPR013126 (PFAM); G3DSA:3.90.640.10 (GENE3D); PTHR19375:SF142 (PANTHER); PTHR19375 (PANTHER); IPR018181 (PROSITE_PATTERNS); IPR018181 (PROSITE_PATTERNS); IPR018181 (PROSITE_PATTERNS); TRANSMEMBRANE (PHOBIUS); TRANSMEMBRANE (PHOBIUS); CYTOPLASMIC_DOMAIN (PHOBIUS); NON_CYTOPLASMIC_DOMAIN (PHOBIUS); NON_CYTOPLASMIC_DOMAIN (PHOBIUS); IPR029047 (SUPERFAMILY); SSF53067 (SUPERFAMILY); SSF53067 (SUPERFAMILY); IPR029048 (SUPERFAMILY)

1712	493964_length_2323_cvg_13.9_tip_1_5	187	poc1 centriolar protein homolog a	774	0	73.40%	0.231		Coil (COILS); IPR001680 (SMART); IPR001680 (PFAM); IPR015943 (G3DSA:2.130.10.GENE3D); PTHR22847:SF319 (PANTHER); PTHR22847 (PANTHER); IPR019775 (PROSITE_PATTERNS); IPR001680 (PROSITE_PROFILES); IPR001680 (PROSITE_PROFILES); IPR001680 (PROSITE_PROFILES); IPR001680 (PROSITE_PROFILES); IPR001680 (PROSITE_PROFILES); IPR017986 (PROSITE_PROFILES); IPR001680 (PROSITE_PROFILES); IPR017986 (SUPERFAMILY)
1717	494042_length_2347_cvg_9.0_tip_1_1	95	zinc finger protein 501 isoform x2	774	6.80E-11	60.30%	0.172		IPR000210 (SMART); IPR015880 (SMART); IPR013087 (G3DSA:3.30.160.GENE3D); IPR013087 (G3DSA:3.30.160.GENE3D); IPR013069 (PFAM); G3DSA:3.30.710.10 (GENE3D); PTHR24409 (PANTHER); PTHR24409:SF14 (PANTHER); IPR007087 (PROSITE_PATTERNS); NON_CYTOPLASMIC_DOMAIN (PHOBIUS); CYTOPLASMIC_DOMAIN (PHOBIUS); TRANSMEMBRANE (PHOBIUS); IPR007087 (PROSITE_PROFILES); IPR000210 (PROSITE_PROFILES); IPR011333 (SUPERFAMILY); SSF57667 (SUPERFAMILY); TMhelix (TMHMM)
1714	493994_length_2334_cvg_5.1_tip_1_4	57	cadherin egf lag seven-pass g-type receptor 1-like isoform x6	778	2.63E-35	44.10%	0.151		IPR000832 (PFAM); PTHR12011:SF74 (PANTHER); PTHR12011 (PANTHER); TRANSMEMBRANE (PHOBIUS); TRANSMEMBRANE (PHOBIUS); TRANSMEMBRANE (PHOBIUS); NON_CYTOPLASMIC_DOMAIN (PHOBIUS); NON_CYTOPLASMIC_DOMAIN (PHOBIUS); CYTOPLASMIC_DOMAIN (PHOBIUS); TRANSMEMBRANE (PHOBIUS); CYTOPLASMIC_DOMAIN (PHOBIUS); NON_CYTOPLASMIC_DOMAIN (PHOBIUS); CYTOPLASMIC_DOMAIN (PHOBIUS); NON_CYTOPLASMIC_DOMAIN (PHOBIUS); NON_CYTOPLASMIC_DOMAIN (PHOBIUS); TRANSMEMBRANE (PHOBIUS); TRANSMEMBRANE (PHOBIUS); CYTOPLASMIC_DOMAIN (PHOBIUS); NON_CYTOPLASMIC_DOMAIN (PHOBIUS); NON_CYTOPLASMIC_DOMAIN (PHOBIUS); TRANSMEMBRANE (PHOBIUS); TRANSMEMBRANE (PHOBIUS); TRANSMEMBRANE (PHOBIUS); TRANSMEMBRANE (PHOBIUS); TRANSMEMBRANE (PHOBIUS); CYTOPLASMIC_DOMAIN (PHOBIUS); TRANSMEMBRANE (PHOBIUS); CYTOPLASMIC_DOMAIN (PHOBIUS); TRANSMEMBRANE (PHOBIUS); CYTOPLASMIC_DOMAIN (PHOBIUS); IPR017981 (PROSITE_PROFILES); TMhelix (TMHMM); TMhelix (TMHMM); TMhelix (TMHMM); TMhelix (TMHMM); TMhelix (TMHMM); TMhelix (TMHMM); TMhelix (TMHMM); TMhelix (TMHMM); TMhelix (TMHMM); TMhelix (TMHMM)
1715	494014_length_2341_cvg_45.5_tip_1_4	478	iduronate 2-sulfatase	780	0	67.60%	0.462	Y	G3DSA:3.30.1120.10 (GENE3D); IPR017849 (G3DSA:3.40.720.GENE3D); IPR000917 (PFAM); PTHR10342:SF185 (PANTHER); PTHR10342 (PANTHER); IPR024607 (PROSITE_PATTERNS); NON_CYTOPLASMIC_DOMAIN (PHOBIUS); NON_CYTOPLASMIC_DOMAIN (PHOBIUS); TRANSMEMBRANE (PHOBIUS); TRANSMEMBRANE (PHOBIUS); CYTOPLASMIC_DOMAIN (PHOBIUS); SignalP-noTM (SIGNALP_EUK); IPR017850 (SUPERFAMILY)
1716	494016_length_2341_cvg_17.7_tip_1_3	202	wd repeat-containing protein 82	781	0	94.80%	0.18		IPR001680 (SMART); IPR015943 (G3DSA:2.130.10.GENE3D); IPR001680 (PFAM); PTHR19861:SF2 (PANTHER); PTHR19861 (PANTHER); IPR019775 (PROSITE_PATTERNS); TRANSMEMBRANE (PHOBIUS); TRANSMEMBRANE (PHOBIUS); CYTOPLASMIC_DOMAIN (PHOBIUS); CYTOPLASMIC_DOMAIN (PHOBIUS); CYTOPLASMIC_DOMAIN (PHOBIUS); NON_CYTOPLASMIC_DOMAIN (PHOBIUS); TRANSMEMBRANE (PHOBIUS); TRANSMEMBRANE (PHOBIUS); NON_CYTOPLASMIC_DOMAIN (PHOBIUS); TRANSMEMBRANE (PHOBIUS); NON_CYTOPLASMIC_DOMAIN (PHOBIUS); IPR001680 (PROSITE_PROFILES); IPR017986 (PROSITE_PROFILES); IPR001680 (PROSITE_PROFILES); IPR001680 (PROSITE_PROFILES); IPR017986 (SUPERFAMILY); TMhelix (TMHMM); TMhelix (TMHMM); TMhelix (TMHMM)

1725	494110_length_2371_cvg_31.6_tip_1_2	502	monocarboxylate transporter 10 isoform x1	781	2.91E-147	75.30%	0.338	IPR011701 (PFAM); G3DSA:1.20.1250.20 (GENE3D); PTHR11360:SF90 (PANTHER); PTHR11360 (PANTHER); NON_CYTOPLASMIC_DOMAIN (PHOBIUS); TRANSMEMBRANE (PHOBIUS); TRANSMEMBRANE (PHOBIUS); TRANSMEMBRANE (PHOBIUS); TRANSMEMBRANE (PHOBIUS); NON_CYTOPLASMIC_DOMAIN (PHOBIUS); CYTOPLASMIC_DOMAIN (PHOBIUS); NON_CYTOPLASMIC_DOMAIN (PHOBIUS); NON_CYTOPLASMIC_DOMAIN (PHOBIUS); CYTOPLASMIC_DOMAIN (PHOBIUS); NON_CYTOPLASMIC_DOMAIN (PHOBIUS); CYTOPLASMIC_DOMAIN (PHOBIUS); TRANSMEMBRANE (PHOBIUS); TRANSMEMBRANE (PHOBIUS); CYTOPLASMIC_DOMAIN (PHOBIUS); TRANSMEMBRANE (PHOBIUS); TRANSMEMBRANE (PHOBIUS); CYTOPLASMIC_DOMAIN (PHOBIUS); TRANSMEMBRANE (PHOBIUS); TRANSMEMBRANE (PHOBIUS); NON_CYTOPLASMIC_DOMAIN (PHOBIUS); CYTOPLASMIC_DOMAIN (PHOBIUS); CYTOPLASMIC_DOMAIN (PHOBIUS); NON_CYTOPLASMIC_DOMAIN (PHOBIUS); TRANSMEMBRANE (PHOBIUS); TRANSMEMBRANE (PHOBIUS); TRANSMEMBRANE (PHOBIUS); TRANSMEMBRANE (PHOBIUS); NON_CYTOPLASMIC_DOMAIN (PHOBIUS); IPR020846 (SUPERFAMILY); TMhelix (TMHMM); TMhelix (TMHMM); TMhelix (TMHMM); TMhelix (TMHMM); TMhelix (TMHMM); TMhelix (TMHMM); TMhelix (TMHMM); TMhelix (TMHMM); TMhelix (TMHMM); TMhelix (TMHMM); TMhelix (TMHMM); TMhelix (TMHMM); TMhelix (TMHMM); TMhelix (TMHMM)
1718	494052_length_2350_cvg_7.4_tip_1_0	85	peptidoglycan-associated lipoprotein	784	2.91E-123	99.70%	0.153	IPR006664 (PRINTS); IPR014169 (TIGRFAM); IPR006665 (G3DSA:3.30.1330.GENE3D); IPR006665 (PFAM); PTHR30128 (PANTHER); IPR006690 (PROSITE_PATTERNS); TRANSMEMBRANE (PHOBIUS); CYTOPLASMIC_DOMAIN (PHOBIUS); CYTOPLASMIC_DOMAIN (PHOBIUS); NON_CYTOPLASMIC_DOMAIN (PHOBIUS); TRANSMEMBRANE (PHOBIUS); NON_CYTOPLASMIC_DOMAIN (PHOBIUS); NON_CYTOPLASMIC_DOMAIN (PHOBIUS); TRANSMEMBRANE (PHOBIUS); TRANSMEMBRANE (PHOBIUS); IPR006665 (PROSITE_PROFILES); IPR006665 (SUPERFAMILY); TMhelix (TMHMM); TMhelix (TMHMM); TMhelix (TMHMM); TMhelix (TMHMM)
1719	494060_length_2353_cvg_10.3_tip_1_2	113	27 kda hemolymph	784	1.31E-42	57.30%	0.104	IPR009832 (PFAM); PTHR20997 (PANTHER); TRANSMEMBRANE (PHOBIUS); NON_CYTOPLASMIC_DOMAIN (PHOBIUS); CYTOPLASMIC_DOMAIN (PHOBIUS); TRANSMEMBRANE (PHOBIUS); NON_CYTOPLASMIC_DOMAIN (PHOBIUS); NON_CYTOPLASMIC_DOMAIN (PHOBIUS); TRANSMEMBRANE (PHOBIUS); TRANSMEMBRANE (PHOBIUS); CYTOPLASMIC_DOMAIN (PHOBIUS); TMhelix (TMHMM); TMhelix (TMHMM); TMhelix (TMHMM)
1720	494072_length_2359_cvg_6.4_tip_1_2	107	krueppel-like factor 10	786	1.58E-65	63.20%	0.127	IPR015880 (SMART); IPR013087 (G3DSA:3.30.160.GENE3D); PF13465 (PFAM); IPR013087 (G3DSA:3.30.160.GENE3D); PTHR23223:SF21 (PANTHER); PTHR23223 (PANTHER); IPR007087 (PROSITE_PATTERNS); IPR007087 (PROSITE_PATTERNS); IPR007087 (PROSITE_PATTERNS); TRANSMEMBRANE (PHOBIUS); CYTOPLASMIC_DOMAIN (PHOBIUS); TRANSMEMBRANE (PHOBIUS); NON_CYTOPLASMIC_DOMAIN (PHOBIUS); TRANSMEMBRANE (PHOBIUS); TRANSMEMBRANE (PHOBIUS); NON_CYTOPLASMIC_DOMAIN (PHOBIUS); NON_CYTOPLASMIC_DOMAIN (PHOBIUS); TRANSMEMBRANE (PHOBIUS); NON_CYTOPLASMIC_DOMAIN (PHOBIUS); CYTOPLASMIC_DOMAIN (PHOBIUS); CYTOPLASMIC_DOMAIN (PHOBIUS); TRANSMEMBRANE (PHOBIUS); IPR007087 (PROSITE_PROFILES); IPR007087 (PROSITE_PROFILES); IPR007087 (PROSITE_PROFILES); SSF57667 (SUPERFAMILY); SSF57667 (SUPERFAMILY); TMhelix (TMHMM); TMhelix (TMHMM); TMhelix (TMHMM); TMhelix (TMHMM)
1735	494262_length_2441_cvg_64.7_tip_1_4	13301	y-box factor homolog isoform x2	787	7.25E-57	77.00%	0.102	IPR002059 (PRINTS); IPR011129 (SMART); IPR012340 (G3DSA:2.40.50.GENE3D); IPR002059 (PFAM); PTHR11544 (PANTHER); IPR019844 (PROSITE_PATTERNS); NON_CYTOPLASMIC_DOMAIN (PHOBIUS); CYTOPLASMIC_DOMAIN (PHOBIUS); TRANSMEMBRANE (PHOBIUS); NON_CYTOPLASMIC_DOMAIN (PHOBIUS); TRANSMEMBRANE (PHOBIUS); IPR012340 (SUPERFAMILY); TMhelix (TMHMM)

1722	494090_length_2365_cvg_22.5_tip_1_2	247	zinc finger protein 135-like	788	1.41E-38	51.40%	0.113		IPR015880 (SMART); IPR013087 (G3DSA:3.30.160.GENE3D); PF13465 (PFAM); IPR013087 (G3DSA:3.30.160.GENE3D); PTHR24409 (PANTHER); PTHR24409:SF13 (PANTHER); IPR007087 (PROSITE_PATTERNS); IPR007087 (PROSITE_PATTERNS); IPR007087 (PROSITE_PATTERNS); IPR007087 (PROSITE_PATTERNS); IPR007087 (PROSITE_PATTERNS); IPR007087 (PROSITE_PATTERNS); CYTOPLASMIC_DOMAIN (PHOBIUS); TRANSMEMBRANE (PHOBIUS); NON_CYTOPLASMIC_DOMAIN (PHOBIUS); IPR007087 (PROSITE_PROFILES); IPR007087 (PROSITE_PROFILES); IPR007087 (PROSITE_PROFILES); IPR007087 (PROSITE_PROFILES); IPR007087 (PROSITE_PROFILES); IPR007087 (PROSITE_PROFILES); IPR007087 (PROSITE_PROFILES); SSF57667 (SUPERFAMILY); SSF57667 (SUPERFAMILY); SSF57667 (SUPERFAMILY); SSF57667 (SUPERFAMILY); TMhelix (TMHMM)
1723	494098_length_2368_cvg_65.0_tip_1_1	1339	hemocyanin subunit type 1 precursor	789	0	70.10%	0.139		IPR013788 (PRINTS); IPR008922 (G3DSA:1.10.1280.GENE3D); IPR005203 (G3DSA:2.60.40.GENE3D); IPR005203 (PFAM); IPR005204 (G3DSA:1.20.1370.GENE3D); IPR000896 (PFAM); IPR005204 (PFAM); IPR013788 (PANTHER); IPR002227 (PROSITE_PATTERNS); IPR013788 (PROSITE_PATTERNS); IPR013788 (PROSITE_PATTERNS); IPR008922 (SUPERFAMILY); IPR005204 (SUPERFAMILY); IPR014756 (SUPERFAMILY)
1724	494102_length_2369_cvg_15.6_tip_1_0	162	atp-dependent zinc metalloprotease yme1 homolog isoform x2	790	0	76.30%	0.781	Y	IPR003593 (SMART); IPR027417 (G3DSA:3.40.50.GENE3D); IPR000642 (PFAM); IPR003959 (PFAM); IPR005936 (TIGRFAM); G3DSA:1.10.8.60 (GENE3D); PTHR23076 (PANTHER); PTHR23076:SF37 (PANTHER); IPR003960 (PROSITE_PATTERNS); NON_CYTOPLASMIC_DOMAIN (PHOBIUS); TRANSMEMBRANE (PHOBIUS); CYTOPLASMIC_DOMAIN (PHOBIUS); TRANSMEMBRANE (PHOBIUS); NON_CYTOPLASMIC_DOMAIN (PHOBIUS); IPR005936 (HAMAP); SignalP-noTM (SIGNALP_EUK); SignalP-TM (SIGNALP_GRAM_POSITIVE); IPR027417 (SUPERFAMILY); SSF140990 (SUPERFAMILY); TMhelix (TMHMM); TMhelix (TMHMM)
1726	494122_length_2374_cvg_6.5_tip_1_5	91	tyrosine-protein phosphatase non-receptor type 1 isoform x1	791	8.51E-157	66.40%	0.235		Coil (COILS); IPR000242 (PRINTS); IPR003595 (SMART); IPR000242 (SMART); IPR000242 (PFAM); IPR029021 (G3DSA:3.90.190.GENE3D); PTHR19134:SF200 (PANTHER); PTHR19134 (PANTHER); IPR016130 (PROSITE_PATTERNS); IPR000242 (PROSITE_PROFILES); IPR000387 (PROSITE_PROFILES); IPR029021 (SUPERFAMILY)
1730	494204_length_2418_cvg_13.8_tip_1_5	858	transcription factor iiia-like	791	2.37E-77	55.20%	0.115		Coil (COILS); IPR015880 (SMART); IPR013087 (G3DSA:3.30.160.GENE3D); IPR013087 (G3DSA:3.30.160.GENE3D); IPR013087 (G3DSA:3.30.160.GENE3D); IPR013087 (G3DSA:3.30.160.GENE3D); PF13894 (PFAM); IPR013087 (G3DSA:3.30.160.GENE3D); IPR007087 (PFAM); IPR013087 (G3DSA:3.30.160.GENE3D); PF13465 (PFAM); IPR013087 (G3DSA:3.30.160.GENE3D); PTHR24382:SF29 (PANTHER); PTHR24382 (PANTHER); IPR007087 (PROSITE_PATTERNS); IPR007087 (PROSITE_PATTERNS); IPR007087 (PROSITE_PATTERNS); IPR007087 (PROSITE_PATTERNS); IPR007087 (PROSITE_PATTERNS); IPR007087 (PROSITE_PATTERNS); IPR007087 (PROSITE_PATTERNS); IPR007087 (PROSITE_PATTERNS); IPR007087 (PROSITE_PATTERNS); IPR007087 (PROSITE_PROFILES); IPR007087 (PROSITE_PROFILES); IPR007087 (PROSITE_PROFILES); IPR007087 (PROSITE_PROFILES); IPR007087 (PROSITE_PROFILES); IPR007087 (PROSITE_PROFILES); IPR007087 (PROSITE_PROFILES); IPR007087 (PROSITE_PROFILES); IPR007087 (PROSITE_PROFILES); SSF57667 (SUPERFAMILY); SSF57667 (SUPERFAMILY); SSF57667 (SUPERFAMILY); SSF57667 (SUPERFAMILY); SSF57667 (SUPERFAMILY)
1729	494170_length_2399_cvg_78.7_tip_1_2	14353	hemocytin isoform x2	792	9.12E-19	40.90%	0.104		IPR006207 (SMART); NON_CYTOPLASMIC_DOMAIN (PHOBIUS); TRANSMEMBRANE (PHOBIUS); CYTOPLASMIC_DOMAIN (PHOBIUS); NON_CYTOPLASMIC_DOMAIN (PHOBIUS); TRANSMEMBRANE (PHOBIUS); IPR001007 (PROSITE_PROFILES); TMhelix (TMHMM); TMhelix (TMHMM)
1727	494150_length_2385_cvg_11.2_tip_1_2	120	retinol dehydrogenase 13-like	795	1.85E-87	66.60%	0.189		IPR002347 (PRINTS); IPR002198 (PRINTS); IPR002198 (PFAM); IPR016040 (G3DSA:3.40.50.GENE3D); PTHR24320 (PANTHER); IPR020904 (PROSITE_PATTERNS); CYTOPLASMIC_DOMAIN (PHOBIUS); TRANSMEMBRANE (PHOBIUS); TRANSMEMBRANE (PHOBIUS); NON_CYTOPLASMIC_DOMAIN (PHOBIUS); NON_CYTOPLASMIC_DOMAIN (PHOBIUS); SSF51735 (SUPERFAMILY); TMhelix (TMHMM); TMhelix (TMHMM)

1728	494156_length_2388_cvg_7.9_tip_1_4	110	membrane-associated guanylate ww and pdz domain-containing protein 1-like isoform x1	796	4.10E-35	81.40%	0.161	IPR001478 (SMART); IPR001478 (G3DSA:2.30.42.GENE3D); IPR001478 (PFAM); PTHR10316:SF40 (PANTHER); PTHR10316 (PANTHER); NON_CYTOPLASMIC_DOMAIN (PHOBIUS); TRANSMEMBRANE (PHOBIUS); CYTOPLASMIC_DOMAIN (PHOBIUS); IPR001478 (PROSITE_PROFILES); IPR001478 (SUPERFAMILY); TMhelix (TMHMM); TMhelix (TMHMM)
1736	494282_length_2452_cvg_51.4_tip_1_5	868	heat shock 70 kda protein cognate 5	800	0	89.00%	0.22	Coil (COILS); IPR013126 (PRINTS); G3DSA:3.30.30.30 (GENE3D); G3DSA:3.90.640.10 (GENE3D); IPR012725 (TIGRFAM); IPR029048 (G3DSA:1.20.1270.GENE3D); IPR013126 (PFAM); G3DSA:3.30.420.40 (GENE3D); G3DSA:3.30.420.40 (GENE3D); IPR029047 (G3DSA:2.60.34.GENE3D); PTHR19375:SF173 (PANTHER); PTHR19375 (PANTHER); IPR018181 (PROSITE_PATTERNS); IPR018181 (PROSITE_PATTERNS); IPR018181 (PROSITE_PATTERNS); IPR012725 (HAMAP); SSF53067 (SUPERFAMILY); IPR029047 (SUPERFAMILY); SSF53067 (SUPERFAMILY)
1731	494218_length_2425_cvg_7.6_tip_1_3	91	phospholipase a2	809	1.44E-26	48.90%	0.161	IPR016090 (SMART); IPR016090 (PFAM); IPR016090 (G3DSA:1.20.90.GENE3D); IPR001211 (PANTHER); PTHR11716:SF45 (PANTHER); CYTOPLASMIC_DOMAIN (PHOBIUS); TRANSMEMBRANE (PHOBIUS); NON_CYTOPLASMIC_DOMAIN (PHOBIUS); TRANSMEMBRANE (PHOBIUS); NON_CYTOPLASMIC_DOMAIN (PHOBIUS); NON_CYTOPLASMIC_DOMAIN (PHOBIUS); TRANSMEMBRANE (PHOBIUS); CYTOPLASMIC_DOMAIN (PHOBIUS); TRANSMEMBRANE (PHOBIUS); CYTOPLASMIC_DOMAIN (PHOBIUS); NON_CYTOPLASMIC_DOMAIN (PHOBIUS); TRANSMEMBRANE (PHOBIUS); TRANSMEMBRANE (PHOBIUS); IPR016090 (SUPERFAMILY); TMhelix (TMHMM); TMhelix (TMHMM); TMhelix (TMHMM); TMhelix (TMHMM); TMhelix (TMHMM); TMhelix (TMHMM)
1732	494230_length_2433_cvg_32.0_tip_1_0	1145	beta- -n-acetylgalactosaminyltransferase bre-4	811	1.40E-121	66.60%	0.323	IPR003859 (PRINTS); IPR027791 (PFAM); IPR029044 (G3DSA:3.90.550.GENE3D); IPR009053 (G3DSA:1.10.287.GENE3D); IPR027995 (PFAM); IPR004127 (PFAM); IPR003859 (PANTHER); PTHR19300:SF30 (PANTHER); TRANSMEMBRANE (PHOBIUS); TRANSMEMBRANE (PHOBIUS); NON_CYTOPLASMIC_DOMAIN (PHOBIUS); CYTOPLASMIC_DOMAIN (PHOBIUS); CYTOPLASMIC_DOMAIN (PHOBIUS); IPR009053 (SUPERFAMILY); IPR029044 (SUPERFAMILY); TMhelix (TMHMM)
1733	494232_length_2433_cvg_9.6_tip_1_1	137	carboxypeptidase e	811	0	73.90%	0.138	IPR000834 (PRINTS); IPR000834 (SMART); IPR014766 (G3DSA:2.60.40.GENE3D); PF13620 (PFAM); IPR000834 (PFAM); G3DSA:3.40.630.10 (GENE3D); PTHR11532:SF41 (PANTHER); PTHR11532 (PANTHER); IPR000834 (PROSITE_PATTERNS); IPR000834 (PROSITE_PATTERNS); TRANSMEMBRANE (PHOBIUS); CYTOPLASMIC_DOMAIN (PHOBIUS); CYTOPLASMIC_DOMAIN (PHOBIUS); TRANSMEMBRANE (PHOBIUS); NON_CYTOPLASMIC_DOMAIN (PHOBIUS); TRANSMEMBRANE (PHOBIUS); TRANSMEMBRANE (PHOBIUS); NON_CYTOPLASMIC_DOMAIN (PHOBIUS); NON_CYTOPLASMIC_DOMAIN (PHOBIUS); SSF53187 (SUPERFAMILY); IPR008969 (SUPERFAMILY); TMhelix (TMHMM); TMhelix (TMHMM)
1734	494234_length_2434_cvg_63.2_tip_1_3	1092	pdgf- and vegf-related factor 1-like precursor	812	7.84E-53	55.80%	0.159	IPR000072 (SMART); IPR000072 (PFAM); IPR029034 (G3DSA:2.10.90.GENE3D); PTHR11633 (PANTHER); PTHR11633:SF1 (PANTHER); CYTOPLASMIC_DOMAIN (PHOBIUS); TRANSMEMBRANE (PHOBIUS); TRANSMEMBRANE (PHOBIUS); CYTOPLASMIC_DOMAIN (PHOBIUS); NON_CYTOPLASMIC_DOMAIN (PHOBIUS); NON_CYTOPLASMIC_DOMAIN (PHOBIUS); NON_CYTOPLASMIC_DOMAIN (PHOBIUS); TRANSMEMBRANE (PHOBIUS); TRANSMEMBRANE (PHOBIUS); IPR000072 (PROSITE_PROFILES); IPR029034 (SUPERFAMILY); TMhelix (TMHMM); TMhelix (TMHMM); TMhelix (TMHMM); TMhelix (TMHMM)
1741	494322_length_2472_cvg_12.8_tip_1_2	170	mitogen-activated protein kinase 15-like	814	0	73.00%	0.151	G3DSA:1.10.510.10 (GENE3D); IPR000719 (PFAM); G3DSA:3.30.200.20 (GENE3D); PTHR24055:SF79 (PANTHER); PTHR24055 (PANTHER); IPR017441 (PROSITE_PATTERNS); IPR003527 (PROSITE_PATTERNS); TRANSMEMBRANE (PHOBIUS); NON_CYTOPLASMIC_DOMAIN (PHOBIUS); CYTOPLASMIC_DOMAIN (PHOBIUS); CYTOPLASMIC_DOMAIN (PHOBIUS); TRANSMEMBRANE (PHOBIUS); TRANSMEMBRANE (PHOBIUS); NON_CYTOPLASMIC_DOMAIN (PHOBIUS); IPR000719 (PROSITE_PROFILES); IPR011009 (SUPERFAMILY); TMhelix (TMHMM); TMhelix (TMHMM)
1746	494368_length_2489_cvg_89.6_tip_1_0	17714	membrane metallo-endopeptidase-like 1-like	816	0	67.50%	0.146	IPR018497 (PRINTS); IPR024079 (G3DSA:3.40.390.GENE3D); IPR008753 (PFAM); IPR018497 (PFAM); IPR000718 (PANTHER); PTHR11733:SF113 (PANTHER); NON_CYTOPLASMIC_DOMAIN (PHOBIUS); NON_CYTOPLASMIC_DOMAIN (PHOBIUS); TRANSMEMBRANE (PHOBIUS); TRANSMEMBRANE (PHOBIUS); CYTOPLASMIC_DOMAIN (PHOBIUS); SSF55486 (SUPERFAMILY); TMhelix (TMHMM); TMhelix (TMHMM)

1737	494290_length_2454_cvg_4.2_tip_1_1	97	ectonucleoside triphosphate diphosphohydrolase 2-like isoform x4	818	2.62E-105	54.30%	0.168	IPR000407 (PFAM); IPR000407 (PANTHER); IPR000407 (PROSITE_PATTERNS); CYTOPLASMIC_DOMAIN (PHOBIUS); TRANSMEMBRANE (PHOBIUS); NON_CYTOPLASMIC_DOMAIN (PHOBIUS); TRANSMEMBRANE (PHOBIUS); CYTOPLASMIC_DOMAIN (PHOBIUS); TRANSMEMBRANE (PHOBIUS); NON_CYTOPLASMIC_DOMAIN (PHOBIUS); TMhelix (TMHMM); TMhelix (TMHMM); TMhelix (TMHMM)
1743	494354_length_2483_cvg_12.6_tip_1_4	155	leucine-rich repeat-containing protein 47-like	819	8.81E-173	67.80%	0.121	IPR003591 (SMART); IPR005146 (SMART); SM00364 (SMART); IPR025875 (PFAM); IPR001611 (PFAM); G3DSA:3.80.10.10 (GENE3D); IPR005146 (PFAM); PTHR10947 (PANTHER); PTHR10947:SF2 (PANTHER); NON_CYTOPLASMIC_DOMAIN (PHOBIUS); CYTOPLASMIC_DOMAIN (PHOBIUS); CYTOPLASMIC_DOMAIN (PHOBIUS); TRANSMEMBRANE (PHOBIUS); TRANSMEMBRANE (PHOBIUS); TRANSMEMBRANE (PHOBIUS); NON_CYTOPLASMIC_DOMAIN (PHOBIUS); IPR001611 (PROSITE_PROFILES); IPR001611 (PROSITE_PROFILES); IPR001611 (PROSITE_PROFILES); IPR001611 (PROSITE_PROFILES); IPR001611 (PROSITE_PROFILES); IPR001611 (PROSITE_PROFILES); IPR001611 (PROSITE_PROFILES); SSF52058 (SUPERFAMILY)
1738	494300_length_2458_cvg_5.1_tip_1_0	77	eukaryotic translation initiation factor 2-alpha kinase 3	820	1.36E-112	52.50%	0.173	IPR002290 (SMART); IPR000719 (PFAM); G3DSA:1.10.510.10 (GENE3D); G3DSA:3.30.200.20 (GENE3D); PTHR11042 (PANTHER); PTHR11042:SF86 (PANTHER); IPR017441 (PROSITE_PATTERNS); IPR008271 (PROSITE_PATTERNS); NON_CYTOPLASMIC_DOMAIN (PHOBIUS); CYTOPLASMIC_DOMAIN (PHOBIUS); TRANSMEMBRANE (PHOBIUS); IPR000719 (PROSITE_PROFILES); IPR011009 (SUPERFAMILY); TMhelix (TMHMM)
1739	494302_length_2458_cvg_66.9_tip_1_3	22192	enolase	820	0	89.50%	0.196	IPR000941 (PRINTS); IPR029017 (G3DSA:3.30.390.GENE3D); IPR020811 (PFAM); IPR000941 (TIGRFAM); IPR029065 (G3DSA:3.20.20.GENE3D); IPR020810 (PFAM); IPR000941 (PANTHER); IPR020809 (PROSITE_PATTERNS); TRANSMEMBRANE (PHOBIUS); TRANSMEMBRANE (PHOBIUS); TRANSMEMBRANE (PHOBIUS); NON_CYTOPLASMIC_DOMAIN (PHOBIUS); NON_CYTOPLASMIC_DOMAIN (PHOBIUS); CYTOPLASMIC_DOMAIN (PHOBIUS); CYTOPLASMIC_DOMAIN (PHOBIUS); IPR000941 (HAMAP); IPR029017 (SUPERFAMILY); IPR029065 (SUPERFAMILY); TMhelix (TMHMM); TMhelix (TMHMM); TMhelix (TMHMM)
1744	494356_length_2483_cvg_48.3_tip_1_0	1135	extracellular matrix protein partial	820	5.62E-22	40.10%	0.106	CYTOPLASMIC_DOMAIN (PHOBIUS); TRANSMEMBRANE (PHOBIUS); NON_CYTOPLASMIC_DOMAIN (PHOBIUS); IPR001007 (PROSITE_PROFILES); IPR006207 (PROSITE_PROFILES); TMhelix (TMHMM)
1740	494320_length_2472_cvg_63.9_tip_1_1	837	zinc metalloproteinase nas-13-like isoform 1	824	7.99E-133	54.80%	0.291	IPR001506 (PRINTS); IPR003582 (SMART); IPR006026 (SMART); IPR001506 (PFAM); IPR024079 (G3DSA:3.40.390.GENE3D); IPR003582 (PFAM); PTHR10127 (PANTHER); PTHR10127:SF550 (PANTHER); TRANSMEMBRANE (PHOBIUS); TRANSMEMBRANE (PHOBIUS); CYTOPLASMIC_DOMAIN (PHOBIUS); TRANSMEMBRANE (PHOBIUS); CYTOPLASMIC_DOMAIN (PHOBIUS); CYTOPLASMIC_DOMAIN (PHOBIUS); NON_CYTOPLASMIC_DOMAIN (PHOBIUS); NON_CYTOPLASMIC_DOMAIN (PHOBIUS); NON_CYTOPLASMIC_DOMAIN (PHOBIUS); TRANSMEMBRANE (PHOBIUS); TRANSMEMBRANE (PHOBIUS); TRANSMEMBRANE (PHOBIUS); TRANSMEMBRANE (PHOBIUS); CYTOPLASMIC_DOMAIN (PHOBIUS); NON_CYTOPLASMIC_DOMAIN (PHOBIUS); IPR003582 (PROSITE_PROFILES); IPR003582 (PROSITE_PROFILES); IPR003582 (PROSITE_PROFILES); SSF55486 (SUPERFAMILY); TMhelix (TMHMM); TMhelix (TMHMM); TMhelix (TMHMM); TMhelix (TMHMM)
1754	494442_length_2523_cvg_56.4_tip_1_0	863	alpha beta hydrolase domain-containing protein 13	824	1.76E-137	75.10%	0.129	IPR029058 (G3DSA:3.40.50.GENE3D); IPR029059 (PFAM); PTHR12277 (PANTHER); CYTOPLASMIC_DOMAIN (PHOBIUS); NON_CYTOPLASMIC_DOMAIN (PHOBIUS); TRANSMEMBRANE (PHOBIUS); TRANSMEMBRANE (PHOBIUS); NON_CYTOPLASMIC_DOMAIN (PHOBIUS); CYTOPLASMIC_DOMAIN (PHOBIUS); CYTOPLASMIC_DOMAIN (PHOBIUS); TRANSMEMBRANE (PHOBIUS); TRANSMEMBRANE (PHOBIUS); NON_CYTOPLASMIC_DOMAIN (PHOBIUS); TRANSMEMBRANE (PHOBIUS); TRANSMEMBRANE (PHOBIUS); NON_CYTOPLASMIC_DOMAIN (PHOBIUS); CYTOPLASMIC_DOMAIN (PHOBIUS); CYTOPLASMIC_DOMAIN (PHOBIUS); TRANSMEMBRANE (PHOBIUS); TRANSMEMBRANE (PHOBIUS); IPR029058 (SUPERFAMILY); TMhelix (TMHMM); TMhelix (TMHMM); TMhelix (TMHMM)

1742	494342_length_2478_cvg_16.0_tip_1_0	193	n-sulphoglucosamine partial	826	0	78.30%	0.127		Coil (COILS); IPR000917 (PFAM); IPR017849 (G3DSA:3.40.720.GENE3D); G3DSA:3.30.1120.10 (GENE3D); PTHR10342 (PANTHER); PTHR10342:SF76 (PANTHER); IPR024607 (PROSITE_PATTERNS); TRANSMEMBRANE (PHOBIUS); TRANSMEMBRANE (PHOBIUS); NON_CYTOPLASMIC_DOMAIN (PHOBIUS); CYTOPLASMIC_DOMAIN (PHOBIUS); TRANSMEMBRANE (PHOBIUS); CYTOPLASMIC_DOMAIN (PHOBIUS); NON_CYTOPLASMIC_DOMAIN (PHOBIUS); CYTOPLASMIC_DOMAIN (PHOBIUS); NON_CYTOPLASMIC_DOMAIN (PHOBIUS); TRANSMEMBRANE (PHOBIUS); TRANSMEMBRANE (PHOBIUS); IPR017850 (SUPERFAMILY)
1750	494414_length_2513_cvg_14.3_tip_1_2	223	inversin-like isoform x3	827	1.96E-58	74.30%	0.219		IPR002110 (SMART); IPR000048 (SMART); IPR000048 (PFAM); IPR020683 (PFAM); IPR020683 (G3DSA:1.25.40.GENE3D); PTHR24178 (PANTHER); CYTOPLASMIC_DOMAIN (PHOBIUS); NON_CYTOPLASMIC_DOMAIN (PHOBIUS); TRANSMEMBRANE (PHOBIUS); IPR002110 (PROSITE_PROFILES); IPR002110 (PROSITE_PROFILES); IPR020683 (PROSITE_PROFILES); IPR002110 (PROSITE_PROFILES); IPR000048 (PROSITE_PROFILES); IPR020683 (SUPERFAMILY)
1745	494366_length_2486_cvg_10.5_tip_1_3	109	group 3 secretory phospholipase a2	829	1.07E-45	68.30%	0.137		IPR001211 (PFAM); IPR016090 (G3DSA:1.20.90.GENE3D); PTHR12253 (PANTHER); IPR016090 (SUPERFAMILY)
1752	494420_length_2515_cvg_60.2_tip_1_3	1991	hemocyanin subunit type 2 precursor	830	0	73.10%	0.881	Y	IPR013788 (PRINTS); IPR005203 (PFAM); IPR000896 (PFAM); IPR005204 (PFAM); IPR005204 (G3DSA:1.20.1370.GENE3D); IPR008922 (G3DSA:1.10.1280.GENE3D); IPR005203 (G3DSA:2.60.40.GENE3D); IPR013788 (PANTHER); IPR013788 (PROSITE_PATTERNS); IPR002227 (PROSITE_PATTERNS); IPR013788 (PROSITE_PATTERNS); SIGNAL_PEPTIDE_C_REGION (PHOBIUS); NON_CYTOPLASMIC_DOMAIN (PHOBIUS); SIGNAL_PEPTIDE_N_REGION (PHOBIUS); SIGNAL_PEPTIDE_H_REGION (PHOBIUS); CYTOPLASMIC_DOMAIN (PHOBIUS); TRANSMEMBRANE (PHOBIUS); SIGNAL_PEPTIDE (PHOBIUS); SignalP-noTM (SIGNALP_EUK); IPR008922 (SUPERFAMILY); IPR014756 (SUPERFAMILY); IPR005204 (SUPERFAMILY); TMhelix (TMHMM)
1747	494378_length_2498_cvg_32.0_tip_1_4	374	e3 ubiquitin-protein ligase hakai	833	6.69E-75	74.20%	0.326		PTHR13480:SF0 (PANTHER); PTHR13480 (PANTHER); IPR017907 (PROSITE_PATTERNS); CYTOPLASMIC_DOMAIN (PHOBIUS); NON_CYTOPLASMIC_DOMAIN (PHOBIUS); TRANSMEMBRANE (PHOBIUS); TRANSMEMBRANE (PHOBIUS); CYTOPLASMIC_DOMAIN (PHOBIUS); TRANSMEMBRANE (PHOBIUS); TRANSMEMBRANE (PHOBIUS); NON_CYTOPLASMIC_DOMAIN (PHOBIUS); NON_CYTOPLASMIC_DOMAIN (PHOBIUS); IPR001841 (PROSITE_PROFILES); SSF57850 (SUPERFAMILY); TMhelix (TMHMM); TMhelix (TMHMM); TMhelix (TMHMM); TMhelix (TMHMM)
1748	494396_length_2502_cvg_14.6_tip_1_3	177	glucosylceramidase-like	834	3.74E-180	66.20%	0.111		IPR001139 (PRINTS); IPR013781 (G3DSA:3.20.20.GENE3D); IPR001139 (PFAM); IPR013780 (G3DSA:2.60.40.GENE3D); PTHR11069:SF7 (PANTHER); IPR001139 (PANTHER); NON_CYTOPLASMIC_DOMAIN (PHOBIUS); TRANSMEMBRANE (PHOBIUS); CYTOPLASMIC_DOMAIN (PHOBIUS); TRANSMEMBRANE (PHOBIUS); TRANSMEMBRANE (PHOBIUS); CYTOPLASMIC_DOMAIN (PHOBIUS); NON_CYTOPLASMIC_DOMAIN (PHOBIUS); IPR017853 (SUPERFAMILY); SSF51011 (SUPERFAMILY)
1749	494412_length_2512_cvg_4.6_tip_1_2	72	telomerase protein component 1-like	837	2.62E-36	42.10%	0.101		IPR020472 (PRINTS); IPR001680 (SMART); IPR015943 (G3DSA:2.130.10.GENE3D); IPR001680 (PFAM); IPR015943 (G3DSA:2.130.10.GENE3D); PTHR22847 (PANTHER); IPR019775 (PROSITE_PATTERNS); IPR001680 (PROSITE_PROFILES); IPR001680 (PROSITE_PROFILES); IPR001680 (PROSITE_PROFILES); IPR001680 (PROSITE_PROFILES); IPR017986 (PROSITE_PROFILES); IPR001680 (PROSITE_PROFILES); IPR017986 (PROSITE_PROFILES); IPR001680 (PROSITE_PROFILES); IPR017986 (SUPERFAMILY); IPR017986 (SUPERFAMILY)

1751	494418_length_2515_cvg_6.5_tip_1_2	121	leucine-rich repeats and immunoglobulin-like domains protein 2-like	838	1.53E-148	69.60%	0.131	SM00365 (SMART); IPR003591 (SMART); G3DSA:3.80.10.10 (GENE3D); IPR001611 (PFAM); G3DSA:3.80.10.10 (GENE3D); IPR001611 (PFAM); PTHR24373:SF98 (PANTHER); PTHR24373 (PANTHER); TRANSMEMBRANE (PHOBIUS); CYTOPLASMIC_DOMAIN (PHOBIUS); CYTOPLASMIC_DOMAIN (PHOBIUS); NON_CYTOPLASMIC_DOMAIN (PHOBIUS); TRANSMEMBRANE (PHOBIUS); CYTOPLASMIC_DOMAIN (PHOBIUS); NON_CYTOPLASMIC_DOMAIN (PHOBIUS); TRANSMEMBRANE (PHOBIUS); TRANSMEMBRANE (PHOBIUS); TRANSMEMBRANE (PHOBIUS); CYTOPLASMIC_DOMAIN (PHOBIUS); TRANSMEMBRANE (PHOBIUS); NON_CYTOPLASMIC_DOMAIN (PHOBIUS); IPR001611 (PROSITE_PROFILES); IPR001611 (PROSITE_PROFILES); IPR001611 (PROSITE_PROFILES); IPR001611 (PROSITE_PROFILES); IPR001611 (PROSITE_PROFILES); IPR001611 (PROSITE_PROFILES); IPR001611 (PROSITE_PROFILES); IPR001611 (PROSITE_PROFILES); IPR001611 (PROSITE_PROFILES); IPR001611 (PROSITE_PROFILES); IPR001611 (PROSITE_PROFILES); IPR001611 (PROSITE_PROFILES); IPR001611 (PROSITE_PROFILES); SSF52058 (SUPERFAMILY); TMhelix (TMHMM); TMhelix (TMHMM); TMhelix (TMHMM); TMhelix (TMHMM); TMhelix (TMHMM)
1753	494424_length_2516_cvg_5.8_tip_1_5	75	translocation protein sec63 homolog	838	0	73.90%	0.164	IPR001623 (PRINTS); IPR001623 (SMART); IPR004179 (SMART); IPR004179 (SMART); IPR001623 (G3DSA:1.10.287.GENE3D); IPR000008 (G3DSA:2.60.40.GENE3D); IPR004179 (PFAM); IPR001623 (PFAM); G3DSA:1.10.3380.10 (GENE3D); G3DSA:1.10.150.20 (GENE3D); IPR027137 (PANTHER); PTHR24075:SF0 (PANTHER); TRANSMEMBRANE (PHOBIUS); NON_CYTOPLASMIC_DOMAIN (PHOBIUS); CYTOPLASMIC_DOMAIN (PHOBIUS); CYTOPLASMIC_DOMAIN (PHOBIUS); TRANSMEMBRANE (PHOBIUS); NON_CYTOPLASMIC_DOMAIN (PHOBIUS); TRANSMEMBRANE (PHOBIUS); TRANSMEMBRANE (PHOBIUS); NON_CYTOPLASMIC_DOMAIN (PHOBIUS); IPR001623 (PROSITE_PROFILES); SSF158702 (SUPERFAMILY); IPR001623 (SUPERFAMILY); IPR014756 (SUPERFAMILY); TMhelix (TMHMM); TMhelix (TMHMM); TMhelix (TMHMM); TMhelix (TMHMM)
1755	494450_length_2528_cvg_18.8_tip_1_4	148	gastrula zinc finger	843	1.10E-51	51.80%	0.172	IPR001713 (PRINTS); IPR000010 (SMART); IPR015880 (SMART); G3DSA:3.10.450.10 (GENE3D); IPR013087 (G3DSA:3.30.160.GENE3D); IPR000010 (PFAM); IPR013087 (G3DSA:3.30.160.GENE3D); PF13465 (PFAM); IPR007087 (PFAM); IPR013087 (G3DSA:3.30.160.GENE3D); PF13894 (PFAM); PTHR24409 (PANTHER); PTHR24409:SF14 (PANTHER); IPR018073 (PROSITE_PATTERNS); IPR007087 (PROSITE_PATTERNS); IPR007087 (PROSITE_PATTERNS); IPR007087 (PROSITE_PATTERNS); IPR007087 (PROSITE_PATTERNS); IPR007087 (PROSITE_PATTERNS); IPR007087 (PROSITE_PATTERNS); IPR007087 (PROSITE_PATTERNS); IPR007087 (PROSITE_PATTERNS); IPR007087 (PROSITE_PATTERNS); IPR007087 (PROSITE_PATTERNS); IPR007087 (PROSITE_PROFILES); IPR007087 (PROSITE_PROFILES); IPR007087 (PROSITE_PROFILES); IPR007087 (PROSITE_PROFILES); IPR007087 (PROSITE_PROFILES); IPR007087 (PROSITE_PROFILES); IPR007087 (PROSITE_PROFILES); IPR007087 (PROSITE_PROFILES); IPR007087 (PROSITE_PROFILES); IPR007087 (PROSITE_PROFILES); SSF57667 (SUPERFAMILY); SSF57667 (SUPERFAMILY); SSF57667 (SUPERFAMILY); SSF54403 (SUPERFAMILY); SSF57667 (SUPERFAMILY); SSF57667 (SUPERFAMILY); SSF57667 (SUPERFAMILY)
1759	494530_length_2592_cvg_7.2_tip_1_2	118	low-density lipoprotein receptor	854	1.38E-15	40.90%	0.224	IPR002172 (PRINTS); IPR002172 (SMART); IPR000998 (SMART); IPR000859 (SMART); IPR000859 (PFAM); IPR002172 (G3DSA:4.10.400.GENE3D); IPR000859 (G3DSA:2.60.120.GENE3D); IPR002172 (G3DSA:4.10.400.GENE3D); IPR002172 (PFAM); IPR000998 (PFAM); IPR002172 (G3DSA:4.10.400.GENE3D); PTHR23282:SF75 (PANTHER); PTHR23282 (PANTHER); TRANSMEMBRANE (PHOBIUS); CYTOPLASMIC_DOMAIN (PHOBIUS); TRANSMEMBRANE (PHOBIUS); NON_CYTOPLASMIC_DOMAIN (PHOBIUS); TRANSMEMBRANE (PHOBIUS); CYTOPLASMIC_DOMAIN (PHOBIUS); NON_CYTOPLASMIC_DOMAIN (PHOBIUS); IPR002172 (PROSITE_PROFILES); IPR002172 (PROSITE_PROFILES); IPR000998 (PROSITE_PROFILES); IPR000859 (PROSITE_PROFILES); IPR000998 (PROSITE_PROFILES); IPR002172 (SUPERFAMILY); IPR013320 (SUPERFAMILY); IPR000859 (SUPERFAMILY); IPR013320 (SUPERFAMILY); TMhelix (TMHMM); TMhelix (TMHMM)

1756	494494_length_2568_cvg_55.2_tip_1_5	17610	suppressor of tumorigenicity 14 partial	856	2.10E-67	53.40%	0.738	Y	IPR002172 (PRINTS); IPR001254 (SMART); IPR002172 (SMART); IPR002172 (PFAM); G3DSA:2.40.10.10 (GENE3D); IPR001254 (PFAM); G3DSA:2.40.10.10 (GENE3D); IPR002172 (G3DSA:4.10.400.GENE3D); IPR002172 (G3DSA:4.10.400.GENE3D); G3DSA:2.40.10.10 (GENE3D); PTHR24256 (PANTHER); IPR023415 (PROSITE_PATTERNS); IPR023415 (PROSITE_PATTERNS); IPR023415 (PROSITE_PATTERNS); IPR018114 (PROSITE_PATTERNS); IPR018114 (PROSITE_PATTERNS); SIGNAL_PEPTIDE (PHOBIUS); SIGNAL_PEPTIDE_C_REGION (PHOBIUS); SIGNAL_PEPTIDE_H_REGION (PHOBIUS); SIGNAL_PEPTIDE_N_REGION (PHOBIUS); NON_CYTOPLASMIC_DOMAIN (PHOBIUS); CYTOPLASMIC_DOMAIN (PHOBIUS); TRANSMEMBRANE (PHOBIUS); IPR002172 (PROSITE_PROFILES); IPR002172 (PROSITE_PROFILES); IPR001254 (PROSITE_PROFILES); IPR002172 (PROSITE_PROFILES); SignalP-noTM (SIGNALP_EUK); IPR002172 (SUPERFAMILY); IPR002172 (SUPERFAMILY); IPR002172 (SUPERFAMILY); IPR009003 (SUPERFAMILY); TMhelix (TMHMM)
1757	494510_length_2578_cvg_56.0_tip_1_3	709	cathepsin d	860	0	80.70%	0.655	Y	IPR001461 (PRINTS); IPR012848 (PFAM); IPR021109 (G3DSA:2.40.70.GENE3D); IPR021109 (G3DSA:2.40.70.GENE3D); IPR001461 (PFAM); PTHR13683:SF230 (PANTHER); IPR001461 (PANTHER); IPR001969 (PROSITE_PATTERNS); IPR001969 (PROSITE_PATTERNS); CYTOPLASMIC_DOMAIN (PHOBIUS); SIGNAL_PEPTIDE_C_REGION (PHOBIUS); NON_CYTOPLASMIC_DOMAIN (PHOBIUS); TRANSMEMBRANE (PHOBIUS); SIGNAL_PEPTIDE_N_REGION (PHOBIUS); TRANSMEMBRANE (PHOBIUS); SIGNAL_PEPTIDE_H_REGION (PHOBIUS); CYTOPLASMIC_DOMAIN (PHOBIUS); SIGNAL_PEPTIDE (PHOBIUS); TRANSMEMBRANE (PHOBIUS); NON_CYTOPLASMIC_DOMAIN (PHOBIUS); SignalP-noTM (SIGNALP_EUK); IPR021109 (SUPERFAMILY); TMhelix (TMHMM); TMhelix (TMHMM)
1758	494518_length_2581_cvg_39.6_tip_1_2	714	tyrosine-protein phosphatase non-receptor type 9 isoform x1	860	0	67.70%	0.287		IPR000242 (PRINTS); IPR003595 (SMART); IPR000242 (SMART); IPR001251 (G3DSA:3.40.525.GENE3D); IPR001251 (PFAM); IPR029021 (G3DSA:3.90.190.GENE3D); IPR000242 (PFAM); PTHR19134:SF232 (PANTHER); PTHR19134 (PANTHER); IPR016130 (PROSITE_PATTERNS); NON_CYTOPLASMIC_DOMAIN (PHOBIUS); NON_CYTOPLASMIC_DOMAIN (PHOBIUS); TRANSMEMBRANE (PHOBIUS); CYTOPLASMIC_DOMAIN (PHOBIUS); TRANSMEMBRANE (PHOBIUS); TRANSMEMBRANE (PHOBIUS); CYTOPLASMIC_DOMAIN (PHOBIUS); IPR000242 (PROSITE_PROFILES); IPR000387 (PROSITE_PROFILES); IPR001251 (PROSITE_PROFILES); IPR029021 (SUPERFAMILY); IPR001251 (SUPERFAMILY)
1761	494560_length_2635_cvg_10.4_tip_1_4	138	polypeptide n-acetylgalactosaminyltransferase 5 isoform x2	870	0	85.90%	0.101		IPR000772 (SMART); IPR000772 (PFAM); IPR001173 (PFAM); IPR029044 (G3DSA:3.90.550.GENE3D); G3DSA:2.80.10.50 (GENE3D); PTHR11675 (PANTHER); PTHR11675:SF39 (PANTHER); NON_CYTOPLASMIC_DOMAIN (PHOBIUS); CYTOPLASMIC_DOMAIN (PHOBIUS); TRANSMEMBRANE (PHOBIUS); IPR000772 (PROSITE_PROFILES); IPR029044 (SUPERFAMILY); IPR000772 (SUPERFAMILY); TMhelix (TMHMM); TMhelix (TMHMM)
1760	494540_length_2614_cvg_6.6_tip_1_2	83	aminopeptidase n	871	0	100.00%	0.375	Y	IPR014782 (PRINTS); IPR012779 (TIGRFAM); IPR024601 (G3DSA:1.25.50.GENE3D); G3DSA:1.10.390.10 (GENE3D); IPR024601 (PFAM); IPR014782 (PFAM); PTHR11533:SF152 (PANTHER); IPR001930 (PANTHER); SSF63737 (SUPERFAMILY); SSF55486 (SUPERFAMILY)
1763	494574_length_2650_cvg_15.9_tip_1_1	195	exocyst complex component 3	877	0	74.20%	0.135		Coil (COILS); IPR010326 (PFAM); IPR010326 (PANTHER); PTHR21292:SF1 (PANTHER)
1765	494582_length_2662_cvg_21.5_tip_1_0	266	vitellogenin 2	879	2.10E-66	43.80%	0.129		IPR001846 (SMART); IPR015255 (PFAM); IPR001846 (PFAM); PTHR23345:SF1 (PANTHER); PTHR23345 (PANTHER); NON_CYTOPLASMIC_DOMAIN (PHOBIUS); CYTOPLASMIC_DOMAIN (PHOBIUS); TRANSMEMBRANE (PHOBIUS); IPR001846 (PROSITE_PROFILES); IPR015819 (SUPERFAMILY)
1766	494590_length_2671_cvg_20.0_tip_1_3	260	zinc finger protein 239- partial	880	9.82E-52	60.10%	0.136		IPR015880 (SMART); IPR013087 (G3DSA:3.30.160.GENE3D); IPR013087 (G3DSA:3.30.160.GENE3D); PF13912 (PFAM); PF13465 (PFAM); IPR013087 (G3DSA:3.30.160.GENE3D); PTHR24409:SF13 (PANTHER); PTHR24409 (PANTHER); IPR007087 (PROSITE_PATTERNS); IPR007087 (PROSITE_PATTERNS); IPR007087 (PROSITE_PATTERNS); IPR007087 (PROSITE_PATTERNS); IPR007087 (PROSITE_PATTERNS); NON_CYTOPLASMIC_DOMAIN (PHOBIUS); CYTOPLASMIC_DOMAIN (PHOBIUS); TRANSMEMBRANE (PHOBIUS); IPR007087 (PROSITE_PROFILES); IPR007087 (PROSITE_PROFILES); IPR007087 (PROSITE_PROFILES); IPR007087 (PROSITE_PROFILES); IPR007087 (PROSITE_PROFILES); IPR007087 (PROSITE_PROFILES); SSF57667 (SUPERFAMILY); SSF57667 (SUPERFAMILY); SSF57667 (SUPERFAMILY)

1762	494566_length_2642_cvg_17.9_tip_1_3	294	serine threonine-protein phosphatase 6 catalytic subunit	881	0	93.80%	0.164		IPR006186 (PRINTS); IPR006186 (SMART); IPR029052 (G3DSA:3.60.21.GENE3D); IPR004843 (PFAM); PTHR11668 (PANTHER); PTHR11668:SF24 (PANTHER); IPR006186 (PROSITE_PATTERNS); TRANSMEMBRANE (PHOBIUS); CYTOPLASMIC_DOMAIN (PHOBIUS); NON_CYTOPLASMIC_DOMAIN (PHOBIUS); NON_CYTOPLASMIC_DOMAIN (PHOBIUS); TRANSMEMBRANE (PHOBIUS); IPR029052 (SUPERFAMILY); TMhelix (TMHMM)
1764	494576_length_2652_cvg_28.8_tip_1_3	563	hemagglutinin	884	6.30E-90	66.00%	0.113		IPR023612 (PRINTS); IPR007280 (PFAM); IPR013856 (G3DSA:3.10.170.GENE3D); G3DSA:1.10.390.10 (GENE3D); IPR013856 (PFAM); IPR001570 (PFAM); TRANSMEMBRANE (PHOBIUS); NON_CYTOPLASMIC_DOMAIN (PHOBIUS); TRANSMEMBRANE (PHOBIUS); NON_CYTOPLASMIC_DOMAIN (PHOBIUS); TRANSMEMBRANE (PHOBIUS); CYTOPLASMIC_DOMAIN (PHOBIUS); CYTOPLASMIC_DOMAIN (PHOBIUS); CYTOPLASMIC_DOMAIN (PHOBIUS); TRANSMEMBRANE (PHOBIUS); SSF55486 (SUPERFAMILY); TMhelix (TMHMM); TMhelix (TMHMM); TMhelix (TMHMM); TMhelix (TMHMM); TMhelix (TMHMM); TMhelix (TMHMM); TMhelix (TMHMM); TMhelix (TMHMM)
1767	494594_length_2687_cvg_54.2_tip_1_0	1848	cell surface protein	896	1.12E-13	42.70%	0.396	Y	NON_CYTOPLASMIC_DOMAIN (PHOBIUS); NON_CYTOPLASMIC_DOMAIN (PHOBIUS); CYTOPLASMIC_DOMAIN (PHOBIUS); TRANSMEMBRANE (PHOBIUS); TRANSMEMBRANE (PHOBIUS); TRANSMEMBRANE (PHOBIUS); TRANSMEMBRANE (PHOBIUS); CYTOPLASMIC_DOMAIN (PHOBIUS); NON_CYTOPLASMIC_DOMAIN (PHOBIUS); CYTOPLASMIC_DOMAIN (PHOBIUS); TRANSMEMBRANE (PHOBIUS); TMhelix (TMHMM); TMhelix (TMHMM); TMhelix (TMHMM)
1768	494602_length_2700_cvg_12.4_tip_1_2	138	#NAME?	900	1.50E-116	99.80%	0.17		IPR002180 (PFAM); IPR002180 (TIGRFAM); IPR000728 (PFAM); IPR016188 (G3DSA:3.30.1330.GENE3D); IPR002180 (G3DSA:3.40.50.GENE3D); IPR002180 (PANTHER); PTHR21058:SF0 (PANTHER); IPR002180 (HAMAP); IPR016188 (SUPERFAMILY); IPR002180 (SUPERFAMILY)
1770	494614_length_2713_cvg_32.5_tip_1_3	433	zinc finger protein	900	5.30E-30	51.80%	0.121		Coil (COILS); IPR015880 (SMART); IPR013087 (G3DSA:3.30.160.GENE3D); IPR013087 (G3DSA:3.30.160.GENE3D); PF13894 (PFAM); PF13465 (PFAM); PTHR24409:SF14 (PANTHER); PTHR24409 (PANTHER); IPR007087 (PROSITE_PATTERNS); IPR007087 (PROSITE_PATTERNS); IPR007087 (PROSITE_PATTERNS); IPR007087 (PROSITE_PATTERNS); IPR007087 (PROSITE_PATTERNS); IPR007087 (PROSITE_PROFILES); IPR007087 (PROSITE_PROFILES); IPR007087 (PROSITE_PROFILES); IPR007087 (PROSITE_PROFILES); IPR007087 (PROSITE_PROFILES); SSF57667 (SUPERFAMILY); SSF57667 (SUPERFAMILY)
1769	494604_length_2701_cvg_15.9_tip_1_3	231	dnaj homolog subfamily b member 11	901	0	84.50%	0.733	Y	IPR001623 (PRINTS); IPR001623 (SMART); IPR002939 (PFAM); G3DSA:2.60.260.20 (GENE3D); IPR001623 (G3DSA:1.10.287.GENE3D); G3DSA:2.60.260.20 (GENE3D); IPR001623 (PFAM); PTHR24077 (PANTHER); PTHR24077:SF210 (PANTHER); IPR018253 (PROSITE_PATTERNS); TRANSMEMBRANE (PHOBIUS); TRANSMEMBRANE (PHOBIUS); NON_CYTOPLASMIC_DOMAIN (PHOBIUS); TRANSMEMBRANE (PHOBIUS); NON_CYTOPLASMIC_DOMAIN (PHOBIUS); CYTOPLASMIC_DOMAIN (PHOBIUS); CYTOPLASMIC_DOMAIN (PHOBIUS); IPR001623 (PROSITE_PROFILES); SignalP-noTM (SIGNALP_EUK); IPR008971 (SUPERFAMILY); IPR008971 (SUPERFAMILY); IPR001623 (SUPERFAMILY)
1771	494624_length_2732_cvg_3.2_tip_1_0	52	cd109 antigen	911	0	64.00%	0.105		IPR008930 (G3DSA:1.50.10.GENE3D); IPR011626 (PFAM); PTHR11412 (PANTHER); PTHR11412:SF85 (PANTHER); IPR008930 (SUPERFAMILY)
1772	494632_length_2737_cvg_7.2_tip_1_0	122	kr domain protein	913	5.29E-148	99.00%	0.359	Y	IPR002347 (PRINTS); IPR002198 (PRINTS); IPR002198 (PFAM); IPR016040 (G3DSA:3.40.50.GENE3D); PTHR24322:SF265 (PANTHER); PTHR24322 (PANTHER); IPR020904 (PROSITE_PATTERNS); SSF51735 (SUPERFAMILY)
1773	494646_length_2769_cvg_9.7_tip_1_2	134	fimbrial family protein	923	3.44E-120	99.50%	0.734	Y	IPR000259 (G3DSA:2.60.40.GENE3D); IPR000259 (PFAM); NON_CYTOPLASMIC_DOMAIN (PHOBIUS); SIGNAL_PEPTIDE_C_REGION (PHOBIUS); SIGNAL_PEPTIDE_H_REGION (PHOBIUS); SIGNAL_PEPTIDE (PHOBIUS); SIGNAL_PEPTIDE_N_REGION (PHOBIUS); SignalP-noTM (SIGNALP_GRAM_NEGATIVE); SignalP-noTM (SIGNALP_EUK); IPR008966 (SUPERFAMILY)
1774	494648_length_2772_cvg_6.8_tip_1_0	156	angiotensin-converting enzyme-like	924	2.05E-162	61.70%	0.271		Coil (COILS); IPR001548 (PRINTS); IPR001548 (PFAM); IPR001548 (PANTHER); TRANSMEMBRANE (PHOBIUS); CYTOPLASMIC_DOMAIN (PHOBIUS); NON_CYTOPLASMIC_DOMAIN (PHOBIUS); SSF55486 (SUPERFAMILY); TMhelix (TMHMM)
1775	494654_length_2789_cvg_49.8_tip_1_1	9780	intestinal mucin-like	930	1.59E-24	40.90%	0.1		IPR006207 (SMART); IPR001007 (SMART); IPR001007 (PFAM); IPR001007 (PROSITE_PATTERNS); TRANSMEMBRANE (PHOBIUS); NON_CYTOPLASMIC_DOMAIN (PHOBIUS); CYTOPLASMIC_DOMAIN (PHOBIUS); IPR001007 (PROSITE_PROFILES); IPR006207 (PROSITE_PROFILES)

1776	494658_length_2792_cvg_37.7_tip_1_0	450	map kinase-interacting serine threonine-protein kinase 1	931	0	81.40%	0.12	IPR002290 (SMART); G3DSA:1.10.510.10 (GENE3D); IPR000719 (PFAM); G3DSA:3.30.200.20 (GENE3D); PTHR24349 (PANTHER); IPR008271 (PROSITE_PATTERNS); TRANSMEMBRANE (PHOBIUS); TRANSMEMBRANE (PHOBIUS); NON_CYTOPLASMIC_DOMAIN (PHOBIUS); NON_CYTOPLASMIC_DOMAIN (PHOBIUS); CYTOPLASMIC_DOMAIN (PHOBIUS); IPR000719 (PROSITE_PROFILES); IPR011009 (SUPERFAMILY)
1778	494688_length_2881_cvg_24.9_tip_1_2	642	hemocytin isoform x1	951	3.85E-26	41.20%	0.118	NON_CYTOPLASMIC_DOMAIN (PHOBIUS); NON_CYTOPLASMIC_DOMAIN (PHOBIUS); CYTOPLASMIC_DOMAIN (PHOBIUS); TRANSMEMBRANE (PHOBIUS); TRANSMEMBRANE (PHOBIUS); IPR006207 (PROSITE_PROFILES); TMhelix (TMHMM); TMhelix (TMHMM)
1777	494686_length_2881_cvg_32.9_tip_1_0	1056	gamma-glutamyltransferase 7	961	1.91E-108	54.80%	0.225	IPR000101 (PRINTS); IPR000101 (PFAM); PTHR11686:SF18 (PANTHER); IPR000101 (PANTHER); TRANSMEMBRANE (PHOBIUS); NON_CYTOPLASMIC_DOMAIN (PHOBIUS); TRANSMEMBRANE (PHOBIUS); CYTOPLASMIC_DOMAIN (PHOBIUS); CYTOPLASMIC_DOMAIN (PHOBIUS); IPR029055 (SUPERFAMILY); TMhelix (TMHMM)
1780	494714_length_2926_cvg_18.0_tip_1_4	327	vitellogenin receptor	967	3.98E-83	45.10%	0.106	IPR002172 (PRINTS); IPR002172 (SMART); IPR001881 (SMART); IPR000033 (SMART); IPR000742 (SMART); G3DSA:2.10.25.10 (GENE3D); IPR002172 (G3DSA:4.10.400.GENE3D); G3DSA:2.10.25.10 (GENE3D); IPR001881 (PFAM); IPR002172 (G3DSA:4.10.400.GENE3D); IPR002172 (G3DSA:4.10.400.GENE3D); IPR002172 (PFAM); IPR011042 (G3DSA:2.120.10.GENE3D); PTHR10529 (PANTHER); PTHR10529:SF204 (PANTHER); IPR023415 (PROSITE_PATTERNS); IPR018097 (PROSITE_PATTERNS); IPR023415 (PROSITE_PATTERNS); IPR000152 (PROSITE_PATTERNS); IPR013032 (PROSITE_PATTERNS); IPR013032 (PROSITE_PATTERNS); IPR023415 (PROSITE_PATTERNS); IPR023415 (PROSITE_PATTERNS); NON_CYTOPLASMIC_DOMAIN (PHOBIUS); CYTOPLASMIC_DOMAIN (PHOBIUS); TRANSMEMBRANE (PHOBIUS); IPR002172 (PROSITE_PROFILES); IPR000742 (PROSITE_PROFILES); IPR000033 (PROSITE_PROFILES); IPR002172 (PROSITE_PROFILES); IPR000033 (PROSITE_PROFILES); IPR000033 (PROSITE_PROFILES); IPR002172 (PROSITE_PROFILES); IPR002172 (PROSITE_PROFILES); SSF57196 (SUPERFAMILY); IPR002172 (SUPERFAMILY); IPR002172 (SUPERFAMILY); SSF63825 (SUPERFAMILY); IPR002172 (SUPERFAMILY); IPR002172 (SUPERFAMILY); TMhelix (TMHMM)
1779	494712_length_2921_cvg_36.2_tip_1_5	512	titin- partial	973	3.93E-148	72.50%	0.111	PR00014 (PRINTS); IPR003961 (SMART); IPR013783 (G3DSA:2.60.40.GENE3D); IPR013783 (G3DSA:2.60.40.GENE3D); IPR003961 (PFAM); PTHR19897 (PANTHER); PTHR19897:SF142 (PANTHER); NON_CYTOPLASMIC_DOMAIN (PHOBIUS); TRANSMEMBRANE (PHOBIUS); CYTOPLASMIC_DOMAIN (PHOBIUS); CYTOPLASMIC_DOMAIN (PHOBIUS); NON_CYTOPLASMIC_DOMAIN (PHOBIUS); TRANSMEMBRANE (PHOBIUS); TRANSMEMBRANE (PHOBIUS); IPR003961 (PROSITE_PROFILES); IPR003961 (PROSITE_PROFILES); IPR003961 (PROSITE_PROFILES); IPR003961 (PROSITE_PROFILES); IPR003961 (SUPERFAMILY); IPR003961 (SUPERFAMILY); TMhelix (TMHMM); TMhelix (TMHMM); TMhelix (TMHMM)
1781	494728_length_2965_cvg_14.9_tip_1_3	200	transducin beta-like protein 3	974	9.14E-175	69.00%	0.103	Coil (COILS); Coil (COILS); IPR020472 (PRINTS); IPR001680 (SMART); IPR013934 (PFAM); IPR015943 (G3DSA:2.130.10.GENE3D); IPR001680 (PFAM); PTHR19854 (PANTHER); PTHR19854:SF15 (PANTHER); CYTOPLASMIC_DOMAIN (PHOBIUS); NON_CYTOPLASMIC_DOMAIN (PHOBIUS); NON_CYTOPLASMIC_DOMAIN (PHOBIUS); TRANSMEMBRANE (PHOBIUS); TRANSMEMBRANE (PHOBIUS); TRANSMEMBRANE (PHOBIUS); CYTOPLASMIC_DOMAIN (PHOBIUS); IPR001680 (PROSITE_PROFILES); IPR001680 (PROSITE_PROFILES); IPR017986 (PROSITE_PROFILES); IPR001680 (PROSITE_PROFILES); IPR001680 (PROSITE_PROFILES); IPR001680 (PROSITE_PROFILES); IPR017986 (SUPERFAMILY)
1782	494732_length_2973_cvg_42.5_tip_1_0	2428	receptor-type tyrosine-protein phosphatase n2	991	0	87.30%	0.232	IPR000242 (PRINTS); IPR000242 (SMART); IPR003595 (SMART); IPR000242 (PFAM); IPR029021 (G3DSA:3.90.190.GENE3D); PTHR19134:SF2 (PANTHER); PTHR19134 (PANTHER); IPR016130 (PROSITE_PATTERNS); TRANSMEMBRANE (PHOBIUS); NON_CYTOPLASMIC_DOMAIN (PHOBIUS); CYTOPLASMIC_DOMAIN (PHOBIUS); NON_CYTOPLASMIC_DOMAIN (PHOBIUS); TRANSMEMBRANE (PHOBIUS); IPR000242 (PROSITE_PROFILES); IPR000387 (PROSITE_PROFILES); IPR029021 (SUPERFAMILY); TMhelix (TMHMM); TMhelix (TMHMM)

1784	494748_length_3081_cvg_12.5_tip_1_4	231	serine threonine-protein phosphatase 2a catalytic subunit alpha isoform	1018	0	97.10%	0.1	Coil (COILS); IPR006186 (PRINTS); IPR006186 (SMART); IPR004843 (PFAM); IPR029052 (G3DSA:3.60.21.GENE3D); PTHR11668 (PANTHER); PTHR11668:SF216 (PANTHER); IPR006186 (PROSITE_PATTERNS); NON_CYTOPLASMIC_DOMAIN (PHOBIUS); NON_CYTOPLASMIC_DOMAIN (PHOBIUS); NON_CYTOPLASMIC_DOMAIN (PHOBIUS); CYTOPLASMIC_DOMAIN (PHOBIUS); CYTOPLASMIC_DOMAIN (PHOBIUS); TRANSMEMBRANE (PHOBIUS); CYTOPLASMIC_DOMAIN (PHOBIUS); TRANSMEMBRANE (PHOBIUS); TRANSMEMBRANE (PHOBIUS); TRANSMEMBRANE (PHOBIUS); TRANSMEMBRANE (PHOBIUS); NON_CYTOPLASMIC_DOMAIN (PHOBIUS); TRANSMEMBRANE (PHOBIUS); IPR029052 (SUPERFAMILY); TMhelix (TMHMM); TMhelix (TMHMM); TMhelix (TMHMM); TMhelix (TMHMM); TMhelix (TMHMM); TMhelix (TMHMM); TMhelix (TMHMM)
1783	494746_length_3076_cvg_16.5_tip_1_5	183	neutral alpha-glucosidase ab-like	1025	0	73.80%	0.125	IPR025887 (PFAM); IPR000322 (PFAM); PTHR22762 (PANTHER); PTHR22762:SF54 (PANTHER); NON_CYTOPLASMIC_DOMAIN (PHOBIUS); CYTOPLASMIC_DOMAIN (PHOBIUS); TRANSMEMBRANE (PHOBIUS); SSF51011 (SUPERFAMILY); IPR017853 (SUPERFAMILY); IPR011013 (SUPERFAMILY)
1785	494762_length_3126_cvg_12.6_tip_1_3	165	atp-dependent rna helicase ddx1	1035	0	83.50%	0.14	IPR014001 (SMART); IPR001650 (SMART); IPR003877 (SMART); IPR027417 (G3DSA:3.40.50.GENE3D); IPR001650 (PFAM); IPR027417 (G3DSA:3.40.50.GENE3D); IPR011545 (PFAM); IPR003877 (PFAM); PTHR24031 (PANTHER); PTHR24031:SF211 (PANTHER); NON_CYTOPLASMIC_DOMAIN (PHOBIUS); TRANSMEMBRANE (PHOBIUS); CYTOPLASMIC_DOMAIN (PHOBIUS); IPR014014 (PROSITE_PROFILES); IPR001650 (PROSITE_PROFILES); IPR001870 (PROSITE_PROFILES); IPR014001 (PROSITE_PROFILES); IPR013320 (SUPERFAMILY); IPR027417 (SUPERFAMILY); IPR027417 (SUPERFAMILY); TMhelix (TMHMM)
1786	494766_length_3145_cvg_18.6_tip_1_1	382	basement membrane-specific heparan sulfate proteoglycan core protein	1048	6.20E-96	58.40%	0.102	IPR003599 (SMART); IPR003598 (SMART); IPR013783 (G3DSA:2.60.40.GENE3D); IPR013783 (G3DSA:2.60.40.GENE3D); IPR013151 (PFAM); IPR013783 (G3DSA:2.60.40.GENE3D); PF13895 (PFAM); IPR013098 (PFAM); IPR013783 (G3DSA:2.60.40.GENE3D); PTHR10489 (PANTHER); CYTOPLASMIC_DOMAIN (PHOBIUS); NON_CYTOPLASMIC_DOMAIN (PHOBIUS); NON_CYTOPLASMIC_DOMAIN (PHOBIUS); TRANSMEMBRANE (PHOBIUS); TRANSMEMBRANE (PHOBIUS); NON_CYTOPLASMIC_DOMAIN (PHOBIUS); NON_CYTOPLASMIC_DOMAIN (PHOBIUS); TRANSMEMBRANE (PHOBIUS); TRANSMEMBRANE (PHOBIUS); TRANSMEMBRANE (PHOBIUS); CYTOPLASMIC_DOMAIN (PHOBIUS); CYTOPLASMIC_DOMAIN (PHOBIUS); TRANSMEMBRANE (PHOBIUS); CYTOPLASMIC_DOMAIN (PHOBIUS); TRANSMEMBRANE (PHOBIUS); IPR007110 (PROSITE_PROFILES); IPR007110 (PROSITE_PROFILES); IPR007110 (PROSITE_PROFILES); IPR007110 (PROSITE_PROFILES); SSF48726 (SUPERFAMILY); SSF48726 (SUPERFAMILY); SSF48726 (SUPERFAMILY); SSF48726 (SUPERFAMILY); TMhelix (TMHMM); TMhelix (TMHMM); TMhelix (TMHMM); TMhelix (TMHMM); TMhelix (TMHMM)
1787	494770_length_3157_cvg_69.1_tip_1_1	12425	plasminogen activator spa	1052	8.89E-58	55.50%	0.231	IPR001314 (PRINTS); IPR001254 (SMART); G3DSA:2.40.10.10 (GENE3D); IPR001254 (PFAM); G3DSA:2.40.10.10 (GENE3D); G3DSA:2.40.10.10 (GENE3D); PTHR24276 (PANTHER); IPR018114 (PROSITE_PATTERNS); IPR018114 (PROSITE_PATTERNS); CYTOPLASMIC_DOMAIN (PHOBIUS); NON_CYTOPLASMIC_DOMAIN (PHOBIUS); TRANSMEMBRANE (PHOBIUS); TRANSMEMBRANE (PHOBIUS); TRANSMEMBRANE (PHOBIUS); CYTOPLASMIC_DOMAIN (PHOBIUS); NON_CYTOPLASMIC_DOMAIN (PHOBIUS); IPR001254 (PROSITE_PROFILES); IPR009003 (SUPERFAMILY); TMhelix (TMHMM)
1788	494776_length_3200_cvg_5.9_tip_1_1	91	zinc finger protein	1067	6.37E-37	59.40%	0.109	Coil (COILS); IPR015880 (SMART); IPR007087 (PFAM); PF13465 (PFAM); IPR013087 (G3DSA:3.30.160.GENE3D); IPR013087 (G3DSA:3.30.160.GENE3D); PF13894 (PFAM); IPR013087 (G3DSA:3.30.160.GENE3D); PTHR24409:SF14 (PANTHER); PTHR24409 (PANTHER); IPR007087 (PROSITE_PATTERNS); IPR007087 (PROSITE_PATTERNS); IPR007087 (PROSITE_PATTERNS); IPR007087 (PROSITE_PATTERNS); IPR007087 (PROSITE_PATTERNS); IPR007087 (PROSITE_PATTERNS); TRANSMEMBRANE (PHOBIUS); NON_CYTOPLASMIC_DOMAIN (PHOBIUS); CYTOPLASMIC_DOMAIN (PHOBIUS); IPR007087 (PROSITE_PROFILES); IPR007087 (PROSITE_PROFILES); IPR007087 (PROSITE_PROFILES); IPR007087 (PROSITE_PROFILES); IPR007087 (PROSITE_PROFILES); IPR007087 (PROSITE_PROFILES); IPR007087 (PROSITE_PROFILES); IPR007087 (PROSITE_PROFILES); SSF57667 (SUPERFAMILY); SSF57667 (SUPERFAMILY); SSF57667 (SUPERFAMILY); SSF57667 (SUPERFAMILY)

1789	494788_length_3281_cvg_14.9_tip_1_3	215	zinc finger protein 184-like	1074	0	59.00%	0.165		Coil (COILS); IPR015880 (SMART); IPR013087 (G3DSA:3.30.160.GENE3D); PF13912 (PFAM); IPR013087 (G3DSA:3.30.160.GENE3D); IPR013087 (G3DSA:3.30.160.GENE3D); IPR007087 (PFAM); PF13894 (PFAM); IPR013087 (G3DSA:3.30.160.GENE3D); IPR013087 (G3DSA:3.30.160.GENE3D); PF13465 (PFAM); PTHR24409 (PANTHER); IPR007087 (PROSITE_PATTERNS); IPR007087 (PROSITE_PATTERNS); IPR007087 (PROSITE_PATTERNS); IPR007087 (PROSITE_PATTERNS); IPR007087 (PROSITE_PATTERNS); IPR007087 (PROSITE_PATTERNS); IPR007087 (PROSITE_PATTERNS); IPR007087 (PROSITE_PATTERNS); IPR007087 (PROSITE_PATTERNS); IPR007087 (PROSITE_PATTERNS); IPR007087 (PROSITE_PATTERNS); IPR007087 (PROSITE_PATTERNS); IPR007087 (PROSITE_PROFILES); IPR007087 (PROSITE_PROFILES); IPR007087 (PROSITE_PROFILES); IPR007087 (PROSITE_PROFILES); IPR007087 (PROSITE_PROFILES); IPR007087 (PROSITE_PROFILES); IPR007087 (PROSITE_PROFILES); IPR007087 (PROSITE_PROFILES); IPR007087 (PROSITE_PROFILES); IPR007087 (PROSITE_PROFILES); IPR007087 (PROSITE_PROFILES); IPR007087 (PROSITE_PROFILES); SSF57667 (SUPERFAMILY); SSF57667 (SUPERFAMILY); SSF57667 (SUPERFAMILY); SSF57667 (SUPERFAMILY); SSF57667 (SUPERFAMILY); SSF57667 (SUPERFAMILY); SSF57667 (SUPERFAMILY)
1790	494794_length_3315_cvg_51.9_tip_1_0	1313	endoplasmic reticulum resident protein 29	1105	3.99E-88	71.00%	0.496	Y	Coil (COILS); IPR012336 (G3DSA:3.40.30.GENE3D); IPR011679 (G3DSA:1.20.1150.GENE3D); IPR012883 (PFAM); IPR011679 (PFAM); PTHR12211:SF0 (PANTHER); PTHR12211 (PANTHER); CYTOPLASMIC_DOMAIN (PHOBIUS); SIGNAL_PEPTIDE_N_REGION (PHOBIUS); SIGNAL_PEPTIDE (PHOBIUS); SIGNAL_PEPTIDE_H_REGION (PHOBIUS); NON_CYTOPLASMIC_DOMAIN (PHOBIUS); TRANSMEMBRANE (PHOBIUS); NON_CYTOPLASMIC_DOMAIN (PHOBIUS); TRANSMEMBRANE (PHOBIUS); CYTOPLASMIC_DOMAIN (PHOBIUS); SIGNAL_PEPTIDE_C_REGION (PHOBIUS); TRANSMEMBRANE (PHOBIUS); IPR012336 (SUPERFAMILY); IPR011679 (SUPERFAMILY); TMhelix (TMHMM); TMhelix (TMHMM); TMhelix (TMHMM); TMhelix (TMHMM)
1791	494814_length_3423_cvg_42.1_tip_1_0	893	chorion peroxidase-like	1141	4.40E-170	55.40%	0.363	Y	IPR019791 (PRINTS); IPR019791 (PFAM); IPR019791 (G3DSA:1.10.640.GENE3D); PTHR11475 (PANTHER); SIGNAL_PEPTIDE_C_REGION (PHOBIUS); NON_CYTOPLASMIC_DOMAIN (PHOBIUS); SIGNAL_PEPTIDE_H_REGION (PHOBIUS); SIGNAL_PEPTIDE (PHOBIUS); SIGNAL_PEPTIDE_N_REGION (PHOBIUS); IPR019791 (PROSITE_PROFILES); IPR010255 (SUPERFAMILY); TMhelix (TMHMM)
1792	494826_length_3564_cvg_31.2_tip_1_4	896	granulins- partial	1177	4.38E-50	52.10%	0.149		IPR000118 (SMART); IPR000118 (PFAM); PTHR12274:SF1 (PANTHER); PTHR12274 (PANTHER); IPR000118 (PROSITE_PATTERNS); IPR000118 (PROSITE_PATTERNS); NON_CYTOPLASMIC_DOMAIN (PHOBIUS); NON_CYTOPLASMIC_DOMAIN (PHOBIUS); CYTOPLASMIC_DOMAIN (PHOBIUS); TRANSMEMBRANE (PHOBIUS); CYTOPLASMIC_DOMAIN (PHOBIUS); TRANSMEMBRANE (PHOBIUS); TRANSMEMBRANE (PHOBIUS); NON_CYTOPLASMIC_DOMAIN (PHOBIUS); TRANSMEMBRANE (PHOBIUS); NON_CYTOPLASMIC_DOMAIN (PHOBIUS); TRANSMEMBRANE (PHOBIUS); NON_CYTOPLASMIC_DOMAIN (PHOBIUS); TRANSMEMBRANE (PHOBIUS); CYTOPLASMIC_DOMAIN (PHOBIUS); TRANSMEMBRANE (PHOBIUS); TRANSMEMBRANE (PHOBIUS); CYTOPLASMIC_DOMAIN (PHOBIUS); SSF57277 (SUPERFAMILY); SSF57277 (SUPERFAMILY); TMhelix (TMHMM); TMhelix (TMHMM); TMhelix (TMHMM); TMhelix (TMHMM)
1793	494838_length_3723_cvg_57.9_tip_1_0	1902	annu_scham ame: full=annulin ame: full=protein-glutamine gamma-glutamyltransferase ame: full=transglutaminase	1241	0	74.00%	0.115		IPR002931 (SMART); IPR008958 (PFAM); IPR001102 (PFAM); IPR013783 (G3DSA:2.60.40.GENE3D); IPR013783 (G3DSA:2.60.40.GENE3D); IPR013783 (G3DSA:2.60.40.GENE3D); IPR002931 (G3DSA:3.90.260.GENE3D); IPR002931 (PFAM); IPR023608 (PANTHER); PTHR11590:SF16 (PANTHER); CYTOPLASMIC_DOMAIN (PHOBIUS); TRANSMEMBRANE (PHOBIUS); TRANSMEMBRANE (PHOBIUS); NON_CYTOPLASMIC_DOMAIN (PHOBIUS); TRANSMEMBRANE (PHOBIUS); NON_CYTOPLASMIC_DOMAIN (PHOBIUS); TRANSMEMBRANE (PHOBIUS); NON_CYTOPLASMIC_DOMAIN (PHOBIUS); CYTOPLASMIC_DOMAIN (PHOBIUS); TRANSMEMBRANE (PHOBIUS); CYTOPLASMIC_DOMAIN (PHOBIUS); IPR008958 (SUPERFAMILY); IPR014756 (SUPERFAMILY); IPR008958 (SUPERFAMILY); SSF54001 (SUPERFAMILY); TMhelix (TMHMM)

1794	494844_length_3965_cvg_29.6_tip_1_1	1103	neutral and basic amino acid transport protein rbat	1322	3.57E-160	56.50%	0.189	IPR006589 (SMART); G3DSA:3.90.400.10 (GENE3D); IPR013781 (G3DSA:3.20.20.GENE3D); IPR006047 (PFAM); IPR013780 (G3DSA:2.60.40.GENE3D); PTHR10357:SF77 (PANTHER); IPR015902 (PANTHER); CYTOPLASMIC_DOMAIN (PHOBIUS); TRANSMEMBRANE (PHOBIUS); NON_CYTOPLASMIC_DOMAIN (PHOBIUS); CYTOPLASMIC_DOMAIN (PHOBIUS); TRANSMEMBRANE (PHOBIUS); TRANSMEMBRANE (PHOBIUS); TRANSMEMBRANE (PHOBIUS); NON_CYTOPLASMIC_DOMAIN (PHOBIUS); CYTOPLASMIC_DOMAIN (PHOBIUS); IPR017853 (SUPERFAMILY); TMhelix (TMHMM)
1795	494846_length_3995_cvg_22.9_tip_1_2	2782	long form-like	1322	0	86.30%	0.123	Coil (COILS); Coil (COILS); Coil (COILS); Coil (COILS); Coil (COILS); Coil (COILS); Coil (COILS); Coil (COILS); Coil (COILS); Coil (COILS); Coil (COILS); Coil (COILS); IPR002928 (PFAM); PTHR13140:SF164 (PANTHER); PTHR13140 (PANTHER); SSF90257 (SUPERFAMILY); SSF90257 (SUPERFAMILY); SSF90257 (SUPERFAMILY); SSF90257 (SUPERFAMILY)
1796	494848_length_4019_cvg_62.7_tip_1_2	179426	levansucrase	1339	0	99.30%	0.146	IPR023296 (G3DSA:2.115.10.GENE3D); IPR003469 (PFAM); TRANSMEMBRANE (PHOBIUS); TRANSMEMBRANE (PHOBIUS); TRANSMEMBRANE (PHOBIUS); NON_CYTOPLASMIC_DOMAIN (PHOBIUS); CYTOPLASMIC_DOMAIN (PHOBIUS); TRANSMEMBRANE (PHOBIUS); NON_CYTOPLASMIC_DOMAIN (PHOBIUS); CYTOPLASMIC_DOMAIN (PHOBIUS); NON_CYTOPLASMIC_DOMAIN (PHOBIUS); IPR023296 (SUPERFAMILY); TMhelix (TMHMM)
1797	494848_length_4019_cvg_62.7_tip_1_5	179426	hypothetical protein ECP02989421_5334	1339	6.03E-84	83.90%	0.119	Coil (COILS); TRANSMEMBRANE (PHOBIUS); TRANSMEMBRANE (PHOBIUS); TRANSMEMBRANE (PHOBIUS); TRANSMEMBRANE (PHOBIUS); CYTOPLASMIC_DOMAIN (PHOBIUS); NON_CYTOPLASMIC_DOMAIN (PHOBIUS); CYTOPLASMIC_DOMAIN (PHOBIUS); NON_CYTOPLASMIC_DOMAIN (PHOBIUS); CYTOPLASMIC_DOMAIN (PHOBIUS); TRANSMEMBRANE (PHOBIUS); TRANSMEMBRANE (PHOBIUS); CYTOPLASMIC_DOMAIN (PHOBIUS); NON_CYTOPLASMIC_DOMAIN (PHOBIUS); SignalP-TM (SIGNALP_GRAM_POSITIVE); TMhelix (TMHMM); TMhelix (TMHMM); TMhelix (TMHMM); TMhelix (TMHMM); TMhelix (TMHMM); TMhelix (TMHMM)
1798	494854_length_4495_cvg_43.9_tip_1_5	7406	myosin heavy chain isoform 2	1498	0	90.00%	0.107	Coil (COILS); Coil (COILS); Coil (COILS); Coil (COILS); Coil (COILS); Coil (COILS); Coil (COILS); Coil (COILS); Coil (COILS); Coil (COILS); Coil (COILS); IPR002928 (PFAM); G3DSA:1.20.5.340 (GENE3D); PTHR13140:SF364 (PANTHER); PTHR13140 (PANTHER); CYTOPLASMIC_DOMAIN (PHOBIUS); TRANSMEMBRANE (PHOBIUS); NON_CYTOPLASMIC_DOMAIN (PHOBIUS); SSF90257 (SUPERFAMILY); SSF90257 (SUPERFAMILY); SSF90257 (SUPERFAMILY); SSF90257 (SUPERFAMILY); SSF90257 (SUPERFAMILY); SSF90257 (SUPERFAMILY); TMhelix (TMHMM)

Supplementary Table 6: Whole body transcriptome (SOAP*denovo*-Tran contigs that match against secreted proteins [UniProtSL0243)]

Nr.	Contig name	Read Numbers	Blast result (evalue=10^{-5}, BlastP, Nr database)	Seq. length	min. evalue	mean similarity	SignalP	InterProScan
2290	55825_length_139_cvg_14.0_tip_0_5	0	phospholipase b-like 2	46	1.08E-15	86.70%	0.098	IPR007000 (PFAM); PTHR12370:SF3 (PANTHER); IPR007000 (PANTHER)
2291	55827_length_139_cvg_9.3_tip_0_5	0	phospholipase b-like 2	46	6.75E-13	80.90%	0.099	IPR007000 (PFAM)
2292	56659_length_141_cvg_63.0_tip_0_0	0	---NA---	47			0.115	PF14704 (PFAM)
2293	56661_length_141_cvg_63.0_tip_0_0	0	hemagglutinin amebocyte aggregation factor	47	2.38E-06	65.00%	0.114	PF14704 (PFAM)
2294	56779_length_141_cvg_8.3_tip_1_4	0	---NA---	47			0.11	IPR002223 (G3DSA:4.10.410.GENE3D); IPR002223 (PFAM); IPR002223 (SUPERFAMILY)
2296	57979_length_142_cvg_2.0_tip_1_1	1	very low-density lipoprotein receptor	47	9.80E-12	72.90%	0.104	IPR002172 (PFAM); G3DSA:4.10.1220.10 (GENE3D); IPR002172 (G3DSA:4.10.400.GENE3D); IPR002172 (SUPERFAMILY)
2297	58471_length_142_cvg_2.0_tip_1_5	2	notch 2	47	3.22E-07	67.00%	0.148	IPR011641 (PFAM)
2299	59727_length_142_cvg_4.6_tip_1_4	2	dipeptidyl peptidase 1	47	1.59E-16	84.20%	0.1	IPR000668 (PFAM); G3DSA:3.90.70.10 (GENE3D); PTHR12411:SF314 (PANTHER); IPR013128 (PANTHER); IPR000169 (PROSITE_PATTERNS); SSF54001 (SUPERFAMILY)
2300	59761_length_142_cvg_2.0_tip_1_4	1	reelin-like isoform x4	47	7.42E-08	77.89%	0.106	no IPS match
2301	60087_length_142_cvg_2.0_tip_1_2	2	tyrosine-protein kinase btk29a	47	4.31E-21	95.20%	0.26	IPR001245 (PFAM); G3DSA:1.10.510.10 (GENE3D); PTHR24418:SF162 (PANTHER); PTHR24418 (PANTHER); IPR000719 (PROSITE_PROFILES); IPR011009 (SUPERFAMILY)
2302	60507_length_142_cvg_2.0_tip_1_1	1	proclotting enzyme	47	1.10E-11	75.60%	0.138	IPR001254 (PFAM); G3DSA:2.40.10.10 (GENE3D); PTHR24268 (PANTHER); IPR009003 (SUPERFAMILY)
2303	61187_length_142_cvg_3.0_tip_1_1	1	tachykinins isoform x3	47	3.85E-10	76.60%	0.107	no IPS match
2305	61595_length_143_cvg_2.0_tip_1_2	2	polypeptide n-acetylgalactosaminyltransferase 5 isoform x2	47	8.62E-24	93.40%	0.183	PTHR11675 (PANTHER); PTHR11675:SF21 (PANTHER)
2308	62575_length_143_cvg_2.6_tip_1_2	2	er degradation-enhancing alpha-mannosidase-like protein 1	47	2.09E-19	92.80%	0.095	IPR001382 (PFAM); IPR001382 (G3DSA:1.50.10.GENE3D); IPR001382 (PANTHER); PTHR11742:SF25 (PANTHER); IPR001382 (SUPERFAMILY)
2311	63651_length_143_cvg_2.0_tip_1_2	2	xanthine dehydrogenase	47	8.20E-19	93.40%	0.109	IPR012675 (G3DSA:3.10.20.GENE3D); PTHR11908 (PANTHER); PTHR11908:SF32 (PANTHER); IPR001041 (SUPERFAMILY)
2312	63711_length_143_cvg_4.0_tip_1_2	0	retinol dehydrogenase 14-like	47	4.95E-12	78.00%	0.109	IPR016040 (G3DSA:3.40.50.GENE3D); PTHR24316:SF305 (PANTHER); PTHR24316 (PANTHER); SSF51735 (SUPERFAMILY)
2314	64527_length_143_cvg_2.0_tip_1_2	1	dentin matrix protein 4- partial	47	5.03E-21	94.80%	0.103	IPR029207 (PFAM); PTHR12450:SF8 (PANTHER); IPR024869 (PANTHER)
2295	57913_length_142_cvg_2.0_tip_1_3	1	von willebrand factor a domain-containing protein 8-partial	48	7.13E-19	92.20%	0.095	IPR011704 (PFAM); PTHR21610 (PANTHER); PTHR21610:SF9 (PANTHER)
2298	59651_length_142_cvg_2.0_tip_1_3	2	protein fam132a	48	3.28E-09	77.90%	0.2	no IPS match
2304	61511_length_143_cvg_2.0_tip_1_1	1	---NA---	48			0.156	no IPS match
2306	62125_length_143_cvg_2.9_tip_1_0	1	ubiquitin carboxyl-terminal hydrolase	48	9.26E-11	68.00%	0.104	no IPS match
2307	62573_length_143_cvg_3.0_tip_1_1	1	alpha beta hydrolase domain-containing protein 17c	48	1.04E-16	92.20%	0.477 Y	PTHR12277:SF52 (PANTHER); PTHR12277 (PANTHER); SIGNAL_PEPTIDE (PHOBIUS); SIGNAL_PEPTIDE_N_REGION (PHOBIUS); SIGNAL_PEPTIDE_H_REGION (PHOBIUS); NON_CYTOPLASMIC_DOMAIN (PHOBIUS); SIGNAL_PEPTIDE_C_REGION (PHOBIUS); SignalP-noTM (SIGNALP_EUK)
2309	63159_length_143_cvg_2.0_tip_1_3	2	matrix metalloproteinase-14-like	48	6.04E-20	87.80%	0.115	IPR000585 (G3DSA:2.110.10.GENE3D); IPR018487 (PFAM); PTHR10201:SF135 (PANTHER); PTHR10201 (PANTHER); IPR018487 (PROSITE_PROFILES); IPR000585 (SUPERFAMILY)
2310	63567_length_143_cvg_2.0_tip_1_0	1	epidermal growth factor receptor isoform x1	48	8.09E-21	90.40%	0.102	G3DSA:2.10.220.10 (GENE3D); PTHR24416:SF95 (PANTHER); PTHR24416 (PANTHER)
2313	64021_length_143_cvg_2.0_tip_1_4	1	fic domain-containing partial	48	6.07E-21	93.80%	0.106	IPR003812 (G3DSA:1.10.3290.GENE3D); IPR003812 (PFAM); PTHR13504:SF12 (PANTHER); PTHR13504 (PANTHER); IPR003812 (PROSITE_PROFILES); IPR003812 (SUPERFAMILY)
2315	64691_length_143_cvg_3.0_tip_1_0	3	ring finger and spry domain-containing protein 1-like	48	2.00E-20	92.40%	0.098	PTHR13363 (PANTHER)
2316	65057_length_144_cvg_2.0_tip_1_2	1	matrix metalloproteinase	48	1.50E-10	69.20%	0.144	IPR021190 (PRINTS); IPR001818 (PFAM); IPR024079 (G3DSA:3.40.390.GENE3D); PTHR10201 (PANTHER); SSF55486 (SUPERFAMILY)
2317	65739_length_144_cvg_3.0_tip_1_2	3	de-cadherin-like isoform x2	48	7.32E-09	73.70%	0.096	no IPS match
2318	65967_length_144_cvg_2.0_tip_1_5	2	adenylate cyclase type 8 isoform x2	48	6.93E-23	93.80%	0.124	IPR001054 (G3DSA:3.30.70.GENE3D); IPR001054 (PFAM); PTHR11920:SF236 (PANTHER); PTHR11920 (PANTHER); IPR018297 (PROSITE_PATTERNS); IPR001054 (PROSITE_PROFILES); IPR029787 (SUPERFAMILY)

2319	66459_length_144_cvg_2.0_tip_1_2	2	receptor-type tyrosine-protein phosphatase kappa-like	48	4.22E-16	85.10%	0.1	IPR000242 (PRINTS); IPR000242 (PFAM); IPR029021 (G3DSA:3.90.190.GENE3D); PTHR19134:SF227 (PANTHER); PTHR19134 (PANTHER); IPR016130 (PROSITE_PATTERNS); IPR000387 (PROSITE_PROFILES); IPR000242 (PROSITE_PROFILES); IPR029021 (SUPERFAMILY)
2320	67017_length_144_cvg_2.0_tip_1_0	2	beta- -galactosyltransferase 1-like	48	2.83E-12	71.60%	0.103	IPR027791 (PFAM); IPR029044 (G3DSA:3.90.550.GENE3D); IPR003859 (PANTHER); IPR029044 (SUPERFAMILY)
2321	67221_length_144_cvg_2.0_tip_1_1	1	tyrosine-protein kinase transmembrane receptor ror1-like	48	2.11E-19	86.90%	0.1	G3DSA:1.10.510.10 (GENE3D); IPR001245 (PFAM); PTHR24416 (PANTHER); PTHR24416:SF309 (PANTHER); IPR008266 (PROSITE_PATTERNS); IPR000719 (PROSITE_PROFILES); IPR011009 (SUPERFAMILY)
2322	67235_length_144_cvg_6.2_tip_1_4	0	---NA---	48			0.113	IPR002223 (PFAM); IPR002223 (G3DSA:4.10.410.GENE3D); IPR002223 (SUPERFAMILY)
2323	67577_length_144_cvg_2.0_tip_1_1	2	epidermal growth factor receptor isoform x2	48	1.17E-16	89.00%	0.097	IPR000494 (G3DSA:3.80.20.GENE3D); SSF52058 (SUPERFAMILY)
2324	68047_length_144_cvg_2.3_tip_1_5	0	ubiquitin carboxyl-terminal hydrolase	48	4.84E-11	69.38%	0.103	no IPS match
2326	69411_length_145_cvg_2.0_tip_1_1	2	partial	48	1.47E-23	99.40%	0.106	IPR011614 (G3DSA:2.40.180.GENE3D); IPR011614 (PFAM); IPR018028 (PANTHER); IPR020835 (SUPERFAMILY)
2325	68553_length_145_cvg_2.0_tip_1_3	1	zinc finger and scan domain-containing protein 2-like	49	2.24E-17	84.40%	0.147	IPR013087 (G3DSA:3.30.160.GENE3D); PF13465 (PFAM); PTHR24409 (PANTHER); IPR007087 (PROSITE_PATTERNS); IPR007087 (PROSITE_PROFILES); SSF57667 (SUPERFAMILY)
2327	69591_length_145_cvg_2.0_tip_1_3	2	cre-bli-4 protein	49	6.85E-10	75.60%	0.101	IPR000209 (G3DSA:3.40.50.GENE3D); IPR000209 (SUPERFAMILY)
2328	70853_length_145_cvg_2.0_tip_1_3	2	fibrillin-2-like	49	7.15E-19	91.20%	0.116	G3DSA:2.10.25.10 (GENE3D); IPR001881 (PFAM); PTHR24039:SF20 (PANTHER); IPR011398 (PANTHER); IPR000152 (PROSITE_PATTERNS); SSF57196 (SUPERFAMILY)
2329	71019_length_145_cvg_2.0_tip_1_0	1	moxd1 homolog 1-like	49	7.03E-08	67.70%	0.146	IPR000323 (G3DSA:2.60.120.GENE3D); IPR000323 (PFAM); IPR008977 (SUPERFAMILY)
2330	73573_length_146_cvg_2.0_tip_1_3	1	polypeptide n-acetylgalactosaminyltransferase 5 isoform x1	49	1.34E-22	93.90%	0.126	PTHR11675:SF21 (PANTHER); PTHR11675 (PANTHER); IPR029044 (SUPERFAMILY)
2331	73881_length_146_cvg_62.1_tip_1_3	21	leucine-rich repeat-containing protein 45- partial	49	2.14E-06	70.00%	0.248	CYTOPLASMIC_DOMAIN (PHOBIUS); TRANSMEMBRANE (PHOBIUS); NON_CYTOPLASMIC_DOMAIN (PHOBIUS); TMhelix (TMHMM)
2332	74393_length_146_cvg_2.0_tip_1_0	2	---NA---	49			0.106	IPR018155 (PFAM); IPR013785 (G3DSA:3.20.20.GENE3D); IPR017853 (SUPERFAMILY)
2333	74545_length_146_cvg_3.0_tip_1_0	1	furin-like protease isoforms 1 1-x 2-like	49	1.12E-18	88.80%	0.113	IPR000209 (G3DSA:3.40.50.GENE3D); IPR015500 (PANTHER); PTHR10795:SF71 (PANTHER); IPR000209 (SUPERFAMILY)
2334	74645_length_146_cvg_2.0_tip_1_0	2	14/03/2003	49	1.06E-13	88.70%	0.102	G3DSA:1.20.190.20 (GENE3D); IPR023410 (PFAM); IPR000308 (PANTHER); IPR023410 (SUPERFAMILY)
2335	75617_length_147_cvg_3.0_tip_1_5	1	protein 5nuc-like	49	2.64E-15	82.20%	0.096	IPR029052 (G3DSA:3.60.21.GENE3D); PTHR11575:SF21 (PANTHER); IPR006179 (PANTHER); IPR029052 (SUPERFAMILY)
2336	75849_length_147_cvg_3.0_tip_1_0	0	dopamine beta hydroxylase	49	4.12E-09	66.20%	0.101	IPR005018 (PFAM); IPR000945 (PANTHER); PTHR10157:SF23 (PANTHER); IPR005018 (PROSITE_PROFILES)
2337	76097_length_147_cvg_2.0_tip_1_3	1	serine threonine-protein kinase tao1 isoform x1	49	1.54E-24	95.50%	0.112	IPR000719 (PFAM); G3DSA:1.10.510.10 (GENE3D); PTHR24361:SF259 (PANTHER); PTHR24361 (PANTHER); IPR000719 (PROSITE_PROFILES); IPR011009 (SUPERFAMILY)
2338	77657_length_147_cvg_3.0_tip_1_5	3	endothelial lipase	49	5.92E-11	75.00%	0.206	IPR013818 (PFAM); IPR029058 (G3DSA:3.40.50.GENE3D); IPR000734 (PANTHER); IPR029058 (SUPERFAMILY)
2341	80069_length_148_cvg_5.0_tip_1_5	0	collagen type iv	49	6.43E-20	87.20%	0.137	IPR001442 (SMART); IPR001442 (G3DSA:2.170.240.GENE3D); IPR001442 (PFAM); PTHR24023:SF397 (PANTHER); PTHR24023 (PANTHER); IPR001442 (PROSITE_PROFILES); IPR016187 (SUPERFAMILY)
2342	80565_length_148_cvg_2.0_tip_1_5	0	vitellogenin partial	49	1.28E-14	85.40%	0.098	no IPS match
2344	81249_length_148_cvg_2.0_tip_1_2	2	bone morphogenetic protein 10	49	2.59E-20	82.80%	0.149	IPR001839 (SMART); IPR029034 (G3DSA:2.10.90.GENE3D); IPR001839 (PFAM); IPR015615 (PANTHER); PTHR11848:SF146 (PANTHER); IPR017948 (PROSITE_PATTERNS); IPR001839 (PROSITE_PROFILES); IPR029034 (SUPERFAMILY)
2345	82099_length_149_cvg_2.0_tip_1_2	2	epidermal growth factor receptor	49	2.85E-11	74.60%	0.126	IPR006212 (SMART); G3DSA:2.10.220.10 (GENE3D); PF14843 (PFAM); IPR009030 (SUPERFAMILY)
2346	82301_length_149_cvg_2.0_tip_1_5	1	lysosomal pro-x	49	2.71E-16	81.50%	0.101	IPR008758 (PFAM); PTHR11010:SF31 (PANTHER); IPR008758 (PANTHER)
2348	82517_length_149_cvg_4.0_tip_1_5	1	isoform a	49	2.85E-19	84.40%	0.11	IPR001223 (PFAM); IPR013781 (G3DSA:3.20.20.GENE3D); PTHR11177:SF144 (PANTHER); PTHR11177 (PANTHER); IPR001579 (PROSITE_PATTERNS); IPR017853 (SUPERFAMILY)
2349	82575_length_149_cvg_2.0_tip_1_2	1	carboxypeptidase s	49	6.53E-13	83.00%	0.104	G3DSA:3.40.630.10 (GENE3D); PTHR11014 (PANTHER); PTHR11014:SF66 (PANTHER); SSF53187 (SUPERFAMILY)
2339	78887_length_148_cvg_3.0_tip_1_3	0	lysosomal pro-x carboxypeptidase	50	5.75E-17	84.00%	0.098	IPR008758 (PFAM); PTHR11010:SF11 (PANTHER); IPR008758 (PANTHER)
2340	79833_length_148_cvg_2.0_tip_1_0	2	---NA---	50			0.101	IPR001304 (PFAM); IPR016186 (G3DSA:3.10.100.GENE3D); IPR001304 (PROSITE_PROFILES); IPR016187 (SUPERFAMILY)
2343	81157_length_148_cvg_2.0_tip_1_3	2	---NA---	50			0.096	no IPS match

2347	82389_length_149_cvg_2.0_tip_1_1	3	isoform b	50	5.36E-21	92.20%	0.097	G3DSA:3.30.200.20 (GENE3D); IPR001245 (PFAM); PTHR24418 (PANTHER); PTHR24418:SF0 (PANTHER); IPR000719 (PROSITE_PROFILES); IPR011009 (SUPERFAMILY)
2350	82673_length_149_cvg_6.0_tip_1_4	0	---NA---	50			0.103	PF14704 (PFAM)
2351	83441_length_149_cvg_2.0_tip_1_4	3	peptidyl-prolyl cis-trans isomerase a- partial	50	7.60E-20	87.20%	0.145	IPR002130 (PRINTS); IPR029000 (G3DSA:2.40.100.GENE3D); IPR002130 (PFAM); IPR024936 (PANTHER); IPR002130 (PROSITE_PROFILES); IPR029000 (SUPERFAMILY)
2352	83571_length_149_cvg_2.0_tip_1_0	2	zinc finger protein	50	2.22E-19	85.20%	0.096	IPR013087 (G3DSA:3.30.160.GENE3D); IPR013087 (G3DSA:3.30.160.GENE3D); PF13465 (PFAM); PTHR23228 (PANTHER); PTHR23228:SF14 (PANTHER); IPR007087 (PROSITE_PATTERNS); IPR007087 (PROSITE_PROFILES); IPR007087 (PROSITE_PROFILES); SSF57667 (SUPERFAMILY)
2353	83839_length_149_cvg_2.0_tip_1_1	3	serine protease nudel	50	2.42E-21	82.10%	0.12	IPR002172 (G3DSA:4.10.400.GENE3D); IPR002172 (SUPERFAMILY)
2354	87869_length_150_cvg_2.0_tip_1_4	2	laminin subunit alpha	50	1.55E-16	77.20%	0.117	G3DSA:2.10.25.10 (GENE3D); IPR002049 (PFAM); PTHR10574 (PANTHER); PTHR10574:SF39 (PANTHER)
2355	87911_length_150_cvg_3.0_tip_1_4	2	chondroitin sulfate synthase 1-like	50	1.34E-20	91.80%	0.1	IPR008428 (PFAM); PTHR12369 (PANTHER); PTHR12369:SF11 (PANTHER)
2356	88159_length_150_cvg_3.0_tip_1_4	1	aael007486- partial	50	2.38E-17	85.50%	0.117	IPR029058 (G3DSA:3.40.50.GENE3D); IPR002018 (PFAM); PTHR11559 (PANTHER); IPR019826 (PROSITE_PATTERNS); IPR029058 (SUPERFAMILY)
2357	88747_length_150_cvg_3.0_tip_1_0	2	calcium-activated chloride channel regulator 4-like	50	7.39E-09	64.80%	0.121	IPR013642 (PFAM)
2358	88941_length_150_cvg_2.0_tip_1_3	2	von willebrand factor type egf and pentraxin domain-containing protein 1	50	4.72E-13	80.00%	0.107	IPR001881 (SMART); IPR000742 (SMART); IPR000742 (PFAM); G3DSA:2.10.25.10 (GENE3D); PTHR24049:SF6 (PANTHER); PTHR24049 (PANTHER); IPR013032 (PROSITE_PATTERNS); IPR013032 (PROSITE_PATTERNS); IPR000742 (PROSITE_PROFILES); SSF57196 (SUPERFAMILY)
2359	89473_length_150_cvg_4.0_tip_1_1	3	basement membrane-specific heparan sulfate proteoglycan core protein	50	5.77E-12	75.70%	0.105	no IPS match
2360	89787_length_150_cvg_2.0_tip_1_4	1	dopamine beta-hydroxylase	50	1.79E-06	67.40%	0.112	IPR000945 (PANTHER)
2361	90033_length_150_cvg_4.0_tip_1_4	2	glyceraldehyde-3-phosphate partial	50	2.57E-20	91.20%	0.104	IPR020831 (PRINTS); IPR020829 (PFAM); IPR016040 (G3DSA:3.40.50.GENE3D); IPR020831 (PANTHER); IPR020830 (PROSITE_PATTERNS); SSF55347 (SUPERFAMILY)
2362	90143_length_150_cvg_2.0_tip_1_3	1	fibrillin-2-like	50	3.08E-17	83.10%	0.122	G3DSA:2.10.25.10 (GENE3D); IPR001881 (PFAM); PTHR24039:SF20 (PANTHER); IPR011398 (PANTHER); SSF57196 (SUPERFAMILY)
2363	92997_length_150_cvg_2.0_tip_1_3	1	sialate o-acetylesterase isoform x1	50	4.23E-10	78.90%	0.177	IPR005181 (PFAM)
2364	93721_length_150_cvg_2.0_tip_1_0	3	sushi von willebrand factor type a egf pentraxin domain-containing partial	50	9.78E-08	60.60%	0.111	IPR000436 (PFAM); G3DSA:2.10.70.10 (GENE3D); G3DSA:2.10.70.10 (GENE3D); IPR000436 (PROSITE_PROFILES); IPR000436 (PROSITE_PROFILES); IPR000436 (SUPERFAMILY)
2365	93927_length_150_cvg_4.0_tip_1_4	4	heat shock protein 70	50	1.58E-16	84.80%	0.103	IPR013126 (PFAM); IPR029047 (G3DSA:2.60.34.GENE3D); PTHR19375:SF156 (PANTHER); PTHR19375 (PANTHER); IPR029047 (SUPERFAMILY)
2366	94339_length_151_cvg_3.0_tip_1_5	5	xanthine dehydrogenase partial	50	2.62E-24	94.40%	0.098	IPR002888 (PFAM); IPR002888 (G3DSA:1.10.150.GENE3D); PTHR11908:SF32 (PANTHER); PTHR11908 (PANTHER); IPR002888 (SUPERFAMILY)
2367	95511_length_151_cvg_2.0_tip_1_2	4	syntaxin 1a	50	9.32E-19	83.10%	0.102	Coil (COILS); G3DSA:1.20.58.70 (GENE3D); IPR006011 (PFAM); IPR010989 (SUPERFAMILY)
2368	95759_length_152_cvg_2.0_tip_1_0	2	secreted protein with kunitz	51	2.22E-18	76.50%	0.114	IPR002223 (PRINTS); IPR002223 (SMART); IPR002223 (PFAM); IPR002223 (G3DSA:4.10.410.GENE3D); PTHR10083 (PANTHER); IPR020901 (PROSITE_PATTERNS); IPR002223 (PROSITE_PROFILES); IPR002223 (SUPERFAMILY)
2369	95975_length_152_cvg_102.6_tip_1_0	308	trypsin-like serine proteinase 1	51	1.20E-09	64.50%	0.32	G3DSA:2.40.10.10 (GENE3D); SIGNAL_PEPTIDE_C_REGION (PHOBIUS); SIGNAL_PEPTIDE_H_REGION (PHOBIUS); SIGNAL_PEPTIDE (PHOBIUS); SIGNAL_PEPTIDE_N_REGION (PHOBIUS); NON_CYTOPLASMIC_DOMAIN (PHOBIUS); IPR009003 (SUPERFAMILY)
2370	96301_length_152_cvg_5.2_tip_1_3	0	heat shock protein partial	51	7.87E-23	95.30%	0.11	G3DSA:3.30.420.40 (GENE3D); IPR013126 (PFAM); G3DSA:3.90.640.10 (GENE3D); PTHR19375:SF137 (PANTHER); PTHR19375 (PANTHER); SSF53067 (SUPERFAMILY)
2371	97170_length_153_cvg_3.0_tip_1_3	4	kielin chordin-like protein	51	9.36E-17	78.70%	0.14	IPR014853 (SMART); IPR014853 (PFAM); PTHR11339 (PANTHER)
2372	98334_length_154_cvg_2.0_tip_1_2	2	receptor-type tyrosine-protein phosphatase mu	51	1.80E-10	73.10%	0.105	IPR000242 (PFAM); IPR029021 (G3DSA:3.90.190.GENE3D); PTHR19134 (PANTHER); IPR000242 (PROSITE_PROFILES); IPR029021 (SUPERFAMILY)
2	102236_length_158_cvg_3.0_tip_1_2	3	protein cbg25156	52	5.14E-06	54.00%	0.102	IPR002172 (PFAM); IPR002172 (G3DSA:4.10.400.GENE3D); IPR002172 (PROSITE_PROFILES); IPR002172 (SUPERFAMILY)
2373	98804_length_155_cvg_2.0_tip_1_1	2	deoxyribonuclease-1-like 2-like	52	2.24E-10	81.10%	0.099	IPR005135 (G3DSA:3.60.10.GENE3D); IPR016202 (PANTHER); IPR005135 (SUPERFAMILY)
2374	99236_length_155_cvg_36.2_tip_0_0	28	egcse_cyano ame: full=endoglycoceramidase short=egcase ame: full=glycosphingolipid-specific enzyme short=gsl-specific enzyme flags: precursor	52	1.16E-11	80.33%	0.102	IPR013781 (G3DSA:3.20.20.GENE3D); IPR017853 (SUPERFAMILY)

2375	99238_length_155_cvg_63.0_tip_0_0	95	egcse_cyano ame: full=endoglycoceramidase short=egcase ame: full=glycosphingolipid-specific enzyme short=gsl-specific enzyme flags: precursor	52	1.16E-11	80.33%	0.102	IPR013781 (G3DSA:3.20.20.GENE3D); IPR017853 (SUPERFAMILY)
2376	99640_length_155_cvg_2.0_tip_1_1	2	Af1-cadherin	52	1.81E-06	67.50%	0.106	no IPS match
1	102208_length_158_cvg_2.0_tip_1_4	2	---NA---	53			0.104	IPR008160 (PFAM)
3	103152_length_159_cvg_2.0_tip_1_4	0	variable lymphocyte receptor a	53	1.94E-06	69.00%	0.128	PR00019 (PRINTS); IPR003591 (SMART); G3DSA:3.80.10.10 (GENE3D); IPR001611 (PFAM); IPR001611 (PROSITE_PROFILES); IPR001611 (PROSITE_PROFILES); SSF52058 (SUPERFAMILY)
4	103416_length_160_cvg_4.0_tip_1_1	4	proclotting enzyme	53	7.72E-10	65.40%	0.136	IPR001254 (PFAM); G3DSA:2.40.10.10 (GENE3D); PTHR24260:SF3 (PANTHER); PTHR24260 (PANTHER); IPR018114 (PROSITE_PATTERNS); IPR009003 (SUPERFAMILY)
6	104090_length_160_cvg_3.0_tip_1_4	4	secretory phospholipase a2 receptor	53	1.82E-06	65.33%	0.1	IPR001304 (PFAM); IPR016186 (G3DSA:3.10.100.GENE3D); IPR001304 (PROSITE_PROFILES); IPR016187 (SUPERFAMILY)
5	103454_length_160_cvg_63.0_tip_1_0	573	serine protease inhibitor dipetalogastin-like	54	3.24E-06	72.00%	0.099	IPR002350 (PFAM); G3DSA:3.30.60.30 (GENE3D); IPR002350 (PROSITE_PROFILES); SSF100895 (SUPERFAMILY)
7	104256_length_161_cvg_2.0_tip_1_3	0	transforming growth factor-beta-induced protein ig-h3	54	5.16E-20	72.70%	0.103	IPR000782 (G3DSA:2.30.180.GENE3D); IPR000782 (PFAM); PTHR10900 (PANTHER); PTHR10900:SF73 (PANTHER); IPR000782 (PROSITE_PROFILES); IPR000782 (SUPERFAMILY)
8	104358_length_161_cvg_62.0_tip_0_0	15	colostrum trypsin inhibitor-like	54	2.06E-13	74.70%	0.101	IPR002223 (PRINTS); IPR002223 (SMART); IPR002223 (PFAM); IPR002223 (G3DSA:4.10.410.GENE3D); PTHR10083 (PANTHER); IPR020901 (PROSITE_PATTERNS); IPR002223 (PROSITE_PROFILES); IPR002223 (SUPERFAMILY)
9	104644_length_161_cvg_63.0_tip_0_3	248	glyceraldehyde-3-phosphate dehydrogenase	54	2.54E-13	89.70%	0.123	PTHR10836:SF31 (PANTHER); IPR020831 (PANTHER)
10	104710_length_161_cvg_3.7_tip_1_3	2	xylosyltransferase 1-like	54	4.86E-12	63.60%	0.102	no IPS match
11	104890_length_161_cvg_2.0_tip_1_0	2	leucomyosuppressin precursor	54	8.56E-23	81.50%	0.247	NON_CYTOPLASMIC_DOMAIN (PHOBIUS); SIGNAL_PEPTIDE_H_REGION (PHOBIUS); SIGNAL_PEPTIDE_C_REGION (PHOBIUS); SIGNAL_PEPTIDE (PHOBIUS); SIGNAL_PEPTIDE_N_REGION (PHOBIUS)
12	105574_length_162_cvg_2.0_tip_1_5	1	epidermal growth factor receptor isoform x1	54	6.73E-29	100.00%	0.131	G3DSA:1.10.510.10 (GENE3D); IPR001245 (PFAM); PTHR24416 (PANTHER); PTHR24416:SF95 (PANTHER); IPR000719 (PROSITE_PROFILES); IPR011009 (SUPERFAMILY)
13	105650_length_162_cvg_63.0_tip_0_4	24	fibroleukin-like isoform x1	54	5.25E-07	68.00%	0.106	IPR002181 (PFAM); IPR014716 (G3DSA:3.90.215.GENE3D); IPR002181 (PROSITE_PROFILES); IPR002181 (SUPERFAMILY)
14	105984_length_163_cvg_4.0_tip_1_1	4	hyaluronidase- partial	54	9.85E-09	70.90%	0.102	IPR013785 (G3DSA:3.20.20.GENE3D); IPR018155 (PFAM); IPR018155 (PANTHER); PTHR11769:SF8 (PANTHER); IPR017853 (SUPERFAMILY)
15	106044_length_163_cvg_3.0_tip_1_5	3	---NA---	54			0.104	IPR013642 (PFAM)
16	106196_length_163_cvg_2.0_tip_1_4	1	down syndrome cell adhesion	54	5.55E-22	90.00%	0.114	IPR013783 (G3DSA:2.60.40.GENE3D); IPR013783 (G3DSA:2.60.40.GENE3D); PTHR10489 (PANTHER); PTHR10489:SF567 (PANTHER); IPR003961 (PROSITE_PROFILES); IPR003961 (PROSITE_PROFILES); IPR003961 (SUPERFAMILY)
17	106244_length_163_cvg_2.0_tip_1_2	1	peptidyl-alpha-hydroxyglycine alpha-amidating lyase 1	54	2.03E-10	70.30%	0.107	IPR001258 (PFAM)
18	106402_length_163_cvg_3.4_tip_1_4	1	peptidyl-prolyl cis-trans isomerase nima-interacting 1-like	54	6.32E-23	82.80%	0.11	G3DSA:3.10.50.40 (GENE3D); PTHR10657 (PANTHER); SSF54534 (SUPERFAMILY)
20	106972_length_164_cvg_2.0_tip_1_2	2	attractin-like protein 1	54	7.18E-23	94.80%	0.193	PTHR10574:SF3 (PANTHER); PTHR10574 (PANTHER); NON_CYTOPLASMIC_DOMAIN (PHOBIUS); CYTOPLASMIC_DOMAIN (PHOBIUS); TRANSMEMBRANE (PHOBIUS); TMhelix (TMHMM)
19	106958_length_164_cvg_2.0_tip_1_4	1	cub and sushi domain-containing protein 1	55	1.52E-07	53.80%	0.1	IPR000436 (SMART); G3DSA:2.10.70.10 (GENE3D); IPR000436 (PFAM); IPR000436 (PROSITE_PROFILES); IPR000436 (SUPERFAMILY)
21	106986_length_164_cvg_9.0_tip_1_1	4	adenosine deaminase cecr1-like	55	3.33E-19	84.10%	0.108	IPR001365 (PFAM); G3DSA:3.20.20.140 (GENE3D); PTHR11409 (PANTHER); PTHR11409:SF39 (PANTHER); SSF51556 (SUPERFAMILY)
22	108732_length_166_cvg_28.2_tip_0_5	4	techylectin- partial	55	9.16E-12	72.90%	0.127	IPR002181 (PFAM); IPR014716 (G3DSA:3.90.215.GENE3D); PTHR19143 (PANTHER); IPR002181 (PROSITE_PROFILES); IPR002181 (SUPERFAMILY)
23	108752_length_166_cvg_62.0_tip_0_4	13	hemagglutinin amebocyte aggregation factor-like	55	9.40E-10	66.50%	0.102	PF14704 (PFAM); IPR026645 (PANTHER)
24	108754_length_166_cvg_63.0_tip_0_4	44	hemagglutinin amebocyte aggregation factor-like	55	1.79E-08	63.40%	0.099	PF14704 (PFAM); PTHR15040:SF3 (PANTHER); IPR026645 (PANTHER)
25	109168_length_167_cvg_63.0_tip_0_0	31	---NA---	56			0.152	no IPS match
26	109720_length_168_cvg_2.0_tip_1_2	1	laminin a	56	1.32E-17	75.70%	0.1	G3DSA:2.10.25.10 (GENE3D); IPR002049 (PFAM); IPR013032 (PROSITE_PATTERNS); IPR002049 (PROSITE_PATTERNS); IPR002049 (PROSITE_PROFILES); SSF57196 (SUPERFAMILY)
27	110249_length_168_cvg_4.0_tip_1_2	6	er degradation-enhancing alpha-mannosidase-like protein 2	56	7.56E-27	92.20%	0.202	IPR001382 (G3DSA:1.50.10.GENE3D); IPR001382 (PFAM); IPR001382 (PANTHER); PTHR11742:SF37 (PANTHER); IPR001382 (SUPERFAMILY)
28	110419_length_168_cvg_2.0_tip_1_3	0	carboxypeptidase a2-like	56	1.43E-14	73.80%	0.13	G3DSA:3.40.630.10 (GENE3D); IPR000834 (PFAM); PTHR11705 (PANTHER); SSF53187 (SUPERFAMILY)
29	110659_length_169_cvg_29.0_tip_1_4	1	hemagglutinin amebocyte aggregation factor-like	56	1.73E-10	70.90%	0.105	PF14704 (PFAM); IPR026645 (PANTHER)

30	110727_length_169_cvg_2.2_tip_1_2	4	ankyrin domain-containing protein	56	5.83E-09	68.50%	0.138	IPR002110 (SMART); IPR020683 (G3DSA:1.25.40.GENE3D); PF13857 (PFAM); PTHR24193 (PANTHER); IPR002110 (PROSITE_PROFILES); IPR020683 (PROSITE_PROFILES); IPR020683 (SUPERFAMILY)
32	111137_length_169_cvg_2.0_tip_1_5	3	imaginal disc growth factor	56	1.95E-23	86.80%	0.134	IPR013781 (G3DSA:3.20.20.GENE3D); IPR001223 (PFAM); IPR017853 (SUPERFAMILY)
31	110923_length_169_cvg_122.7_tip_0_3	596	anti-lipopolysaccharide factor isoform 7	57	4.20E-15	73.60%	0.118	IPR024509 (PFAM); IPR024716 (PRODOM)
33	111991_length_171_cvg_2.0_tip_1_3	1	neurogenic locus protein partial	57	6.03E-16	70.50%	0.11	IPR000742 (SMART); IPR001881 (SMART); G3DSA:2.10.25.10 (GENE3D); G3DSA:2.10.25.10 (GENE3D); IPR000742 (PFAM); PTHR24033:SF0 (PANTHER); PTHR24033 (PANTHER); IPR013032 (PROSITE_PATTERNS); IPR013032 (PROSITE_PATTERNS); IPR000742 (PROSITE_PROFILES); IPR000742 (PROSITE_PROFILES); SSF57196 (SUPERFAMILY)
34	112135_length_171_cvg_63.0_tip_1_5	88	---NA---	57			0.097	G3DSA:3.30.60.30 (GENE3D); IPR002350 (PFAM); IPR002350 (PROSITE_PROFILES); SSF100895 (SUPERFAMILY)
35	112379_length_171_cvg_2.0_tip_1_2	2	low quality protein: von willebrand factor type egf and pentraxin domain-containing protein 1	57	9.43E-18	79.20%	0.112	PR00010 (PRINTS); IPR001881 (SMART); IPR000742 (SMART); G3DSA:2.10.25.10 (GENE3D); IPR000742 (PFAM); G3DSA:2.10.25.10 (GENE3D); PTHR24049 (PANTHER); PTHR24049:SF6 (PANTHER); IPR013032 (PROSITE_PATTERNS); IPR013032 (PROSITE_PATTERNS); IPR000152 (PROSITE_PATTERNS); IPR000742 (PROSITE_PROFILES); SSF57196 (SUPERFAMILY)
36	113019_length_172_cvg_2.0_tip_1_1	2	tyrosine-protein kinase src64b isoform x1	57	3.50E-21	86.20%	0.11	IPR001245 (PFAM); G3DSA:1.10.510.10 (GENE3D); PTHR24418 (PANTHER); IPR008266 (PROSITE_PATTERNS); IPR000719 (PROSITE_PROFILES); IPR011009 (SUPERFAMILY)
37	113913_length_174_cvg_63.0_tip_1_2	510	partial	58	1.37E-16	80.80%	0.117	G3DSA:2.40.10.10 (GENE3D); IPR001254 (PFAM); PTHR24264 (PANTHER); IPR009003 (SUPERFAMILY)
38	114249_length_174_cvg_63.0_tip_0_5	75	hemagglutinin amebocyte aggregation factor-like	58	1.37E-10	66.80%	0.119	PF14704 (PFAM); IPR026645 (PANTHER)
39	114309_length_174_cvg_4.0_tip_1_4	5	proprotein convertase subtilisin kexin type 5	58	2.66E-11	71.20%	0.108	G3DSA:3.30.70.850 (GENE3D)
40	114729_length_175_cvg_2.0_tip_1_4	2	acid sphingomyelinase-like phosphodiesterase 3a	58	1.88E-07	57.67%	0.794 Y	NON_CYTOPLASMIC_DOMAIN (PHOBIUS); SIGNAL_PEPTIDE_N_REGION (PHOBIUS); SIGNAL_PEPTIDE_H_REGION (PHOBIUS); SIGNAL_PEPTIDE (PHOBIUS); SIGNAL_PEPTIDE_C_REGION (PHOBIUS); SignalP-TM (SIGNALP_GRAM_POSITIVE); SignalP-noTM (SIGNALP_EUK); SignalP-noTM (SIGNALP_GRAM_NEGATIVE)
41	115223_length_176_cvg_11.0_tip_1_5	9	peptidase m4	58	1.58E-09	69.50%	0.099	IPR023612 (PRINTS); IPR013856 (PFAM); IPR013856 (G3DSA:3.10.170.GENE3D); SSF55486 (SUPERFAMILY)
42	115267_length_176_cvg_20.0_tip_1_2	15	hemagglutinin amebocyte aggregation factor-like	58	3.77E-10	64.60%	0.102	PF14704 (PFAM); IPR026645 (PANTHER)
43	115341_length_176_cvg_2.5_tip_1_0	2	alcohol dehydrogenase	59	1.51E-13	69.80%	0.127	IPR023210 (G3DSA:3.20.20.GENE3D); PTHR11732:SF183 (PANTHER); IPR001395 (PANTHER); IPR018170 (PROSITE_PATTERNS); IPR023210 (SUPERFAMILY)
44	115509_length_176_cvg_63.0_tip_0_0	265	chitinase partial	59	1.26E-15	70.00%	0.147	IPR013781 (G3DSA:3.20.20.GENE3D); IPR001223 (PFAM); IPR029070 (G3DSA:3.10.50.GENE3D); PTHR11177 (PANTHER); PTHR11177:SF112 (PANTHER); IPR029070 (SUPERFAMILY)
45	115511_length_176_cvg_63.0_tip_0_0	158	chitinase partial	59	6.65E-16	69.00%	0.146	IPR001223 (PFAM); IPR013781 (G3DSA:3.20.20.GENE3D); IPR029070 (G3DSA:3.10.50.GENE3D); PTHR11177 (PANTHER); PTHR11177:SF112 (PANTHER); IPR029070 (SUPERFAMILY)
46	115799_length_177_cvg_4.0_tip_1_4	4	cysteine-rich with egf-like domain protein 2	59	1.02E-12	79.60%	0.101	no IPS match
47	116507_length_178_cvg_2.0_tip_1_5	1	proprotein convertase subtilisin kexin type	59	1.39E-17	77.00%	0.11	G3DSA:3.30.70.850 (GENE3D); PTHR10795:SF325 (PANTHER); IPR015500 (PANTHER); IPR009020 (SUPERFAMILY)
48	116763_length_178_cvg_2.0_tip_1_1	1	hexamerin 1	59	1.23E-21	85.30%	0.1	IPR005203 (PFAM); IPR005203 (G3DSA:2.60.40.GENE3D); PTHR11511:SF5 (PANTHER); IPR013788 (PANTHER); IPR014756 (SUPERFAMILY)
49	117133_length_179_cvg_2.0_tip_1_3	2	---NA---	60			0.1	IPR018155 (PFAM); IPR013785 (G3DSA:3.20.20.GENE3D); IPR017853 (SUPERFAMILY)
50	117839_length_180_cvg_4.0_tip_1_2	4	glyceraldehyde-3-phosphate dehydrogenase	60	1.59E-23	88.30%	0.159	G3DSA:3.30.360.10 (GENE3D); IPR020829 (PFAM); PTHR10836:SF31 (PANTHER); IPR020831 (PANTHER); SSF55347 (SUPERFAMILY)
51	117991_length_180_cvg_3.0_tip_1_4	1	peripheral plasma membrane protein cask isoform x5	60	2.59E-32	96.00%	0.113	IPR011511 (PFAM); G3DSA:2.30.30.40 (GENE3D); PTHR23122 (PANTHER); PTHR23122:SF7 (PANTHER); IPR001452 (PROSITE_PROFILES); IPR001452 (SUPERFAMILY)
52	118069_length_180_cvg_12.1_tip_0_0	4	angiopoietin-1 isoform x1	60	2.60E-12	68.60%	0.108	IPR002181 (PFAM); IPR014716 (G3DSA:3.90.215.GENE3D); PTHR19143 (PANTHER); IPR002181 (PROSITE_PROFILES); IPR002181 (SUPERFAMILY)
53	118075_length_180_cvg_2.0_tip_1_5	2	zinc c2h2 type	60	8.11E-09	68.60%	0.099	IPR015880 (SMART); PF13465 (PFAM); IPR013087 (G3DSA:3.30.160.GENE3D); PTHR24409 (PANTHER); PTHR24409:SF14 (PANTHER); IPR007087 (PROSITE_PATTERNS); IPR007087 (PROSITE_PROFILES); SSF57667 (SUPERFAMILY)
54	118147_length_180_cvg_23.0_tip_0_0	4	microfibril-associated glycoprotein 4-like	60	4.71E-12	68.20%	0.118	IPR002181 (PFAM); IPR014716 (G3DSA:3.90.215.GENE3D); PTHR19143 (PANTHER); IPR002181 (PROSITE_PROFILES); IPR002181 (SUPERFAMILY)
55	118417_length_181_cvg_32.8_tip_1_5	10	glycosyl hydrolase family cellulase	60	2.73E-13	68.50%	0.098	IPR001547 (PFAM); IPR013781 (G3DSA:3.20.20.GENE3D); PTHR31308 (PANTHER); IPR017853 (SUPERFAMILY)

56	118419_length_181_cvg_63.0_tip_1_5	61	glycosyl hydrolase family cellulase	60	9.00E-14	68.00%	0.098	IPR013781 (G3DSA:3.20.20.GENE3D); IPR001547 (PFAM); PTHR31308 (PANTHER); IPR017853 (SUPERFAMILY)
58	119217_length_182_cvg_2.0_tip_1_5	3	peptidyl-alpha-hydroxyglycine alpha-amidating lyase 2-like	60	3.15E-12	68.60%	0.098	PTHR10680 (PANTHER)
57	118701_length_181_cvg_3.0_tip_1_3	2	endothelin-converting enzyme 1 isoform x2	61	3.91E-22	82.30%	0.311	IPR018497 (PFAM); IPR024079 (G3DSA:3.40.390.GENE3D); PTHR11733:SF124 (PANTHER); IPR000718 (PANTHER); SSF55486 (SUPERFAMILY)
59	119432_length_183_cvg_2.0_tip_1_4	1	probable serine carboxypeptidase cpvl	61	3.46E-21	85.00%	0.143	IPR029058 (G3DSA:3.40.50.GENE3D); IPR001563 (PFAM); PTHR11802:SF30 (PANTHER); IPR001563 (PANTHER); IPR029058 (SUPERFAMILY)
60	119548_length_183_cvg_10.8_tip_1_2	59	---NA---	61			0.222	TMhelix (TMHMM)
61	121708_length_187_cvg_2.0_tip_1_3	1	membrane metallo-endopeptidase-like 1 isoform x2	63	8.56E-21	85.80%	0.114	IPR024079 (G3DSA:3.40.390.GENE3D); IPR018497 (PFAM); PTHR11733:SF111 (PANTHER); IPR000718 (PANTHER); SSF55486 (SUPERFAMILY)
62	122478_length_188_cvg_3.0_tip_1_1	2	cade_drome ame: full=de-cadherin ame: full=protein shotgun flags: precursor	63	4.02E-09	59.90%	0.111	IPR013320 (G3DSA:2.60.120.GENE3D)
63	122908_length_189_cvg_2.0_tip_1_1	1	moxd1 homolog 2-like	63	3.72E-09	64.70%	0.141	PF03712 (PFAM); IPR014784 (G3DSA:2.60.120.GENE3D); IPR000945 (PANTHER); IPR014783 (PROSITE_PATTERNS); IPR008977 (SUPERFAMILY)
65	124034_length_191_cvg_2.0_tip_1_5	2	insulin-like growth factor-binding protein complex acid labile chain	63	4.88E-16	82.80%	0.113	IPR001611 (PFAM); G3DSA:3.80.10.10 (GENE3D); PTHR24365 (PANTHER); PTHR24365:SF83 (PANTHER); SSF52058 (SUPERFAMILY)
66	124064_length_191_cvg_12.5_tip_0_5	7	ankyrin domain-containing protein	63	2.26E-10	67.10%	0.134	IPR002110 (SMART); IPR020683 (G3DSA:1.25.40.GENE3D); IPR020683 (PFAM); PTHR24193 (PANTHER); IPR002110 (PROSITE_PROFILES); IPR020683 (PROSITE_PROFILES); IPR020683 (SUPERFAMILY)
64	123634_length_190_cvg_2.0_tip_1_3	3	aldehyde oxidase	64	1.41E-19	81.00%	0.11	IPR008274 (PFAM); IPR008274 (G3DSA:3.30.365.GENE3D); PTHR11908:SF65 (PANTHER); PTHR11908 (PANTHER); IPR008274 (SUPERFAMILY)
67	124910_length_192_cvg_2.0_tip_1_4	1	protein kinase related to protein kinase isoform e	64	3.14E-39	100.00%	0.098	IPR000719 (PFAM); G3DSA:1.10.510.10 (GENE3D); PTHR24357:SF80 (PANTHER); PTHR24357 (PANTHER); IPR000719 (PROSITE_PROFILES); IPR011009 (SUPERFAMILY)
68	125060_length_193_cvg_3.0_tip_1_0	1	zinc finger protein 235-like	65	1.07E-11	64.60%	0.098	IPR015880 (SMART); IPR013087 (G3DSA:3.30.160.GENE3D); PF13894 (PFAM); IPR013087 (G3DSA:3.30.160.GENE3D); IPR007087 (PFAM); PTHR24409 (PANTHER); IPR007087 (PROSITE_PATTERNS); IPR007087 (PROSITE_PATTERNS); IPR007087 (PROSITE_PROFILES); IPR007087 (PROSITE_PROFILES); SSF57667 (SUPERFAMILY)
69	125208_length_193_cvg_3.2_tip_1_3	6	predicted protein	65	3.20E-08	48.50%	0.115	no IPS match
70	126392_length_195_cvg_3.0_tip_1_3	2	heat shock protein partial	65	7.35E-37	99.80%	0.1	G3DSA:3.90.640.10 (GENE3D); IPR013126 (PFAM); PTHR19375 (PANTHER); PTHR19375:SF142 (PANTHER); SSF53067 (SUPERFAMILY)
71	126710_length_195_cvg_2.0_tip_1_2	3	latrophilin cirl isoform x1	65	7.59E-21	78.00%	0.117	IPR000922 (PFAM); PTHR12011 (PANTHER); PTHR12011:SF260 (PANTHER); IPR000922 (PROSITE_PROFILES)
72	127202_length_196_cvg_4.8_tip_1_4	2	gly-xaa carboxypeptidase	65	1.45E-13	72.90%	0.107	IPR011650 (G3DSA:3.30.70.GENE3D); IPR011650 (PFAM); PTHR11014 (PANTHER); PTHR11014:SF66 (PANTHER)
73	127602_length_197_cvg_2.0_tip_1_2	4	low quality protein: zinc finger protein 233	65	1.52E-15	68.10%	0.105	IPR015880 (SMART); PF13465 (PFAM); IPR013087 (G3DSA:3.30.160.GENE3D); IPR013087 (G3DSA:3.30.160.GENE3D); PTHR24409 (PANTHER); IPR007087 (PROSITE_PATTERNS); IPR007087 (PROSITE_PROFILES); SSF57667 (SUPERFAMILY)
74	128210_length_198_cvg_21.3_tip_0_0	17	stearoyl- desaturase partial	66	3.32E-28	88.10%	0.263	IPR015876 (PRINTS); PTHR11351 (PANTHER); PTHR11351:SF28 (PANTHER); CYTOPLASMIC_DOMAIN (PHOBIUS); NON_CYTOPLASMIC_DOMAIN (PHOBIUS); TRANSMEMBRANE (PHOBIUS); TMhelix (TMHMM)
75	128230_length_198_cvg_34.0_tip_0_3	18	fatty acid	66	2.45E-28	87.40%	0.262	IPR015876 (PRINTS); PTHR11351 (PANTHER); TRANSMEMBRANE (PHOBIUS); CYTOPLASMIC_DOMAIN (PHOBIUS); NON_CYTOPLASMIC_DOMAIN (PHOBIUS); TMhelix (TMHMM)
77	128768_length_199_cvg_2.0_tip_1_2	2	protein kinase c-binding protein nell1-like	66	4.08E-21	84.90%	0.148	PTHR24042:SF4 (PANTHER); PTHR24042 (PANTHER)
76	128360_length_199_cvg_2.9_tip_1_3	3	xanthine dehydrogenase oxidase	67	1.05E-26	82.80%	0.106	IPR008274 (PFAM); IPR008274 (G3DSA:3.30.365.GENE3D); PTHR11908:SF32 (PANTHER); PTHR11908 (PANTHER); IPR008274 (SUPERFAMILY)
78	128970_length_200_cvg_2.0_tip_1_3	3	zinc finger partial	67	3.74E-07	58.30%	0.102	PF13465 (PFAM); IPR013087 (G3DSA:3.30.160.GENE3D); IPR013087 (G3DSA:3.30.160.GENE3D); PTHR24409 (PANTHER); PTHR24409:SF20 (PANTHER); IPR007087 (PROSITE_PATTERNS); IPR007087 (PROSITE_PROFILES); SSF57667 (SUPERFAMILY)
79	129482_length_201_cvg_2.0_tip_1_0	0	angiotensin-converting enzyme	67	5.20E-30	85.80%	0.113	IPR001548 (PRINTS); IPR001548 (PFAM); IPR001548 (PANTHER); SSF55486 (SUPERFAMILY)
80	130160_length_202_cvg_3.6_tip_1_5	5	epididymal sperm-binding protein 1	67	3.97E-07	58.33%	0.113	IPR000562 (SMART); IPR000562 (G3DSA:2.10.10.GENE3D); IPR000562 (PFAM); IPR000562 (PROSITE_PROFILES); IPR013806 (SUPERFAMILY)
81	130698_length_203_cvg_3.0_tip_1_1	2	lysosomal alpha-glucosidase-like	68	6.55E-27	83.70%	0.108	IPR000322 (PFAM); PTHR22762:SF69 (PANTHER); PTHR22762 (PANTHER); IPR017853 (SUPERFAMILY)

82	132066_length_206_cvg_2.0_tip_1_5	2	ankyrin repeat domain-containing protein 49	68	4.98E-09	64.70%	0.555 Y	IPR002110 (SMART); IPR020683 (PFAM); IPR020683 (G3DSA:1.25.40.GENE3D); PTHR24188 (PANTHER); NON_CYTOPLASMIC_DOMAIN (PHOBIUS); SIGNAL_PEPTIDE (PHOBIUS); SIGNAL_PEPTIDE_H_REGION (PHOBIUS); SIGNAL_PEPTIDE_N_REGION (PHOBIUS); SIGNAL_PEPTIDE_C_REGION (PHOBIUS); IPR002110 (PROSITE_PROFILES); IPR020683 (PROSITE_PROFILES); SignalP-noTM (SIGNALP_EUK); IPR020683 (SUPERFAMILY)
83	133900_length_210_cvg_2.0_tip_1_5	3	gastrula zinc finger protein - partial	70	4.74E-16	69.10%	0.104	IPR015880 (SMART); IPR013087 (G3DSA:3.30.160.GENE3D); IPR013087 (G3DSA:3.30.160.GENE3D); PF13465 (PFAM); PTHR24409 (PANTHER); IPR007087 (PROSITE_PATTERNS); IPR007087 (PROSITE_PROFILES); IPR007087 (PROSITE_PROFILES); SSF57667 (SUPERFAMILY)
84	133914_length_210_cvg_11.8_tip_1_0	18	glucose dehydrogenase	70	1.41E-09	75.90%	0.24	IPR000172 (PFAM); G3DSA:3.50.50.60 (GENE3D); PTHR11552:SF77 (PANTHER); PTHR11552 (PANTHER); IPR000172 (PROSITE_PATTERNS); SSF51905 (SUPERFAMILY)
85	134808_length_212_cvg_84.8_tip_1_3	563	---NA---	71			0.316	IPR000716 (G3DSA:4.10.800.GENE3D); CYTOPLASMIC_DOMAIN (PHOBIUS); NON_CYTOPLASMIC_DOMAIN (PHOBIUS); TRANSMEMBRANE (PHOBIUS); IPR000716 (PROSITE_PROFILES); SignalP-TM (SIGNALP_GRAM_POSITIVE); IPR000716 (SUPERFAMILY); TMhelix (TMHMM)
86	134910_length_212_cvg_4.0_tip_1_0	0	ectonucleotide pyrophosphatase phosphodiesterase family member 3	71	7.18E-12	73.40%	0.156	IPR002591 (PFAM); IPR017849 (G3DSA:3.40.720.GENE3D); IPR024873 (PANTHER); IPR017850 (SUPERFAMILY)
87	134978_length_212_cvg_2.0_tip_1_0	2	aminopeptidase n	71	1.99E-14	64.30%	0.114	G3DSA:1.10.390.10 (GENE3D); PTHR11533:SF148 (PANTHER); IPR001930 (PANTHER); SSF55486 (SUPERFAMILY)
88	135286_length_212_cvg_2.0_tip_1_1	1	plexin- partial	71	2.10E-30	87.60%	0.099	IPR015943 (G3DSA:2.130.10.GENE3D); IPR001627 (PFAM); PTHR22625 (PANTHER); PTHR22625:SF16 (PANTHER); IPR001627 (PROSITE_PROFILES); IPR001627 (SUPERFAMILY); TMhelix (TMHMM)
89	135342_length_213_cvg_4.0_tip_1_2	7	allatostatin neuropeptide precursor	71	9.50E-12	69.40%	0.105	IPR010276 (PFAM)
90	135558_length_213_cvg_17.0_tip_1_2	17	serpin b10	71	1.28E-12	68.30%	0.137	IPR023796 (PFAM); G3DSA:3.30.497.10 (GENE3D); IPR000215 (PANTHER); IPR023796 (SUPERFAMILY)
91	135936_length_214_cvg_4.0_tip_1_5	6	neuroligin- x- partial	71	1.65E-08	62.40%	0.279	IPR029058 (G3DSA:3.40.50.GENE3D); IPR002018 (PFAM); PTHR11559 (PANTHER); IPR029058 (SUPERFAMILY)
93	136164_length_214_cvg_104.9_tip_0_1	593	unnamed protein product	71	1.62E-06	60.00%	0.182	IPR002350 (SMART); IPR002350 (PFAM); G3DSA:3.30.60.30 (GENE3D); IPR002350 (PROSITE_PROFILES); SSF100895 (SUPERFAMILY)
92	136140_length_214_cvg_2.0_tip_1_0	4	lactase-phlorizin hydrolase	72	1.90E-23	77.80%	0.109	IPR001360 (PRINTS); IPR001360 (PFAM); IPR013781 (G3DSA:3.20.20.GENE3D); IPR001360 (PANTHER); IPR017853 (SUPERFAMILY)
94	136796_length_216_cvg_2.0_tip_1_3	2	serine proteinase stubble-like	72	7.24E-21	73.00%	0.118	IPR001254 (PFAM); G3DSA:2.40.10.10 (GENE3D); PTHR24276 (PANTHER); IPR018114 (PROSITE_PATTERNS); IPR001254 (PROSITE_PROFILES); IPR009003 (SUPERFAMILY)
95	137262_length_216_cvg_2.0_tip_1_0	2	---NA---	72			0.13	no IPS match
96	137368_length_217_cvg_3.9_tip_1_5	28	glutamine-rich protein 2	72	3.69E-09	63.00%	0.102	PR01217 (PRINTS)
97	137538_length_217_cvg_2.0_tip_1_4	5	---NA---	72			0.103	IPR014020 (PFAM); G3DSA:2.60.40.1110 (GENE3D); IPR000008 (SUPERFAMILY)
98	137568_length_217_cvg_63.0_tip_0_5	504	probable endoglycoceramidase	72	2.77E-21	70.40%	0.119	IPR013781 (G3DSA:3.20.20.GENE3D); IPR001547 (PFAM); PTHR31308 (PANTHER); IPR017853 (SUPERFAMILY)
99	137624_length_217_cvg_3.0_tip_1_4	4	glyceraldehyde-3-phosphate dehydrogenase	72	9.07E-17	72.80%	0.102	IPR020828 (SMART); IPR020828 (PFAM); IPR016040 (G3DSA:3.40.50.GENE3D); IPR020831 (PANTHER); SSF51735 (SUPERFAMILY)
100	137784_length_218_cvg_25.7_tip_1_1	33	hemagglutinin amebocyte aggregation factor-like	73	2.41E-13	63.70%	0.108	PF14704 (PFAM); IPR026645 (PANTHER)
101	138088_length_218_cvg_2.0_tip_1_4	3	ankyrin repeat domain-containing protein 29-like	73	2.35E-12	63.50%	0.103	IPR002110 (SMART); IPR020683 (G3DSA:1.25.40.GENE3D); IPR020683 (PFAM); PTHR24188 (PANTHER); IPR020683 (PROSITE_PROFILES); IPR002110 (PROSITE_PROFILES); IPR002110 (PROSITE_PROFILES); IPR020683 (SUPERFAMILY)
102	138150_length_218_cvg_2.0_tip_1_0	2	transferrin	73	9.85E-16	70.60%	0.099	IPR001156 (PRINTS); IPR001156 (PFAM); G3DSA:3.40.190.10 (GENE3D); PTHR11485:SF18 (PANTHER); PTHR11485 (PANTHER); IPR001156 (PROSITE_PROFILES); SSF53850 (SUPERFAMILY)
103	138792_length_219_cvg_2.0_tip_1_3	3	fad-linked sulfhydryl oxidase partial	73	3.12E-32	81.00%	0.155	IPR017905 (PFAM); IPR017905 (G3DSA:1.20.120.GENE3D); PTHR12645 (PANTHER); IPR017905 (PROSITE_PROFILES); IPR017905 (SUPERFAMILY)
104	138936_length_220_cvg_2.0_tip_1_2	4	peptidyl-prolyl cis-trans cyclophilin-type family	73	1.49E-34	88.50%	0.122	IPR002130 (PRINTS); IPR029000 (G3DSA:2.40.100.GENE3D); IPR002130 (PFAM); IPR024936 (PANTHER); IPR002130 (PROSITE_PROFILES); IPR029000 (SUPERFAMILY)
105	139150_length_220_cvg_2.0_tip_1_3	4	gamma-glutamyl hydrolase-like	74	1.43E-15	75.30%	0.105	IPR029062 (G3DSA:3.40.50.GENE3D); IPR011697 (PFAM); PTHR11315:SF2 (PANTHER); IPR015527 (PANTHER)
106	139391_length_221_cvg_4.1_tip_1_3	6	---NA---	74			0.107	IPR001846 (PFAM); IPR001846 (PROSITE_PROFILES)
107	139997_length_222_cvg_3.0_tip_1_0	5	colostrum trypsin inhibitor-like	74	5.32E-16	71.50%	0.103	IPR002223 (PRINTS); IPR002223 (SMART); IPR002223 (PFAM); IPR002223 (G3DSA:4.10.410.GENE3D); PTHR10083 (PANTHER); IPR020901 (PROSITE_PATTERNS); IPR002223 (PROSITE_PROFILES); IPR002223 (SUPERFAMILY)

108	140211_length_222_cvg_9.5_tip_1_3	12	insulin-like receptor	74	2.66E-29	80.20%	0.107	G3DSA:1.10.510.10 (GENE3D); IPR001245 (PFAM); PTHR24416 (PANTHER); PTHR24416:SF254 (PANTHER); IPR011009 (SUPERFAMILY)
109	140481_length_223_cvg_2.0_tip_1_4	1	phosphodiesterase i	74	9.33E-15	62.90%	0.178	IPR017849 (G3DSA:3.40.720.GENE3D); IPR002591 (PFAM); IPR024873 (PANTHER); IPR017850 (SUPERFAMILY)
110	140609_length_223_cvg_2.0_tip_1_5	4	ankyrin repeat and sterile alpha motif domain-containing protein 1b	74	9.38E-27	93.20%	0.107	IPR002110 (SMART); IPR020683 (G3DSA:1.25.40.GENE3D); IPR020683 (PFAM); PTHR24174 (PANTHER); IPR020683 (PROSITE_PROFILES); IPR002110 (PROSITE_PROFILES); IPR002110 (PROSITE_PROFILES); IPR020683 (SUPERFAMILY)
111	140885_length_223_cvg_2.0_tip_1_2	4	endothelial zinc finger protein induced by tumor necrosis factor partial	74	7.77E-17	64.90%	0.112	IPR015880 (SMART); IPR013087 (G3DSA:3.30.160.GENE3D); PF13465 (PFAM); IPR013087 (G3DSA:3.30.160.GENE3D); IPR013087 (G3DSA:3.30.160.GENE3D); PTHR24409 (PANTHER); IPR007087 (PROSITE_PATTERNS); IPR007087 (PROSITE_PATTERNS); IPR007087 (PROSITE_PROFILES); IPR007087 (PROSITE_PROFILES); SSF57667 (SUPERFAMILY)
112	141043_length_224_cvg_3.0_tip_1_4	4	sparc-related modular calcium-binding protein partial	75	4.48E-21	73.90%	0.1	IPR019577 (PFAM); IPR011992 (G3DSA:1.10.238.GENE3D); PTHR12352 (PANTHER); IPR018247 (PROSITE_PATTERNS); SSF47473 (SUPERFAMILY)
113	141131_length_224_cvg_2.0_tip_1_3	4	low density lipoprotein receptor-related protein 5 isoform cra_a	75	4.36E-06	57.00%	0.103	IPR002172 (SMART); IPR002172 (G3DSA:4.10.400.GENE3D); IPR002172 (PFAM); IPR023415 (PROSITE_PATTERNS); IPR002172 (PROSITE_PROFILES); IPR002172 (SUPERFAMILY)
114	141465_length_225_cvg_15.8_tip_1_0	24	transcriptional regulator atrx homolog	75	3.24E-09	60.00%	0.1	no IPS match
115	141927_length_225_cvg_3.0_tip_1_0	4	esterase e4-like	75	3.48E-25	78.10%	0.201	IPR002018 (PFAM); IPR029058 (G3DSA:3.40.50.GENE3D); PTHR11559 (PANTHER); IPR029058 (SUPERFAMILY)
120	142911_length_227_cvg_37.8_tip_1_5	53	gram positive anchor	75	3.77E-11	55.00%	0.176	no IPS match
121	143127_length_227_cvg_2.0_tip_1_2	3	signal cub and egf-like domain-containing protein 1 isoform x1	75	1.23E-20	79.20%	0.123	PTHR24046 (PANTHER); PTHR24046:SF4 (PANTHER)
116	142017_length_226_cvg_2.0_tip_1_0	3	low quality protein: kinase d-interacting substrate of 220 kda-like	76	6.50E-32	85.30%	0.105	IPR002110 (SMART); IPR020683 (G3DSA:1.25.40.GENE3D); IPR020683 (PFAM); PTHR24139 (PANTHER); PTHR24139:SF10 (PANTHER); IPR002110 (PROSITE_PROFILES); IPR020683 (PROSITE_PROFILES); IPR020683 (SUPERFAMILY)
117	142265_length_226_cvg_115.4_tip_1_0	2726	anti-lipopolysaccharide factor isoform 7	76	1.47E-08	57.50%	0.909 Y	IPR024509 (PFAM); NON_CYTOPLASMIC_DOMAIN (PHOBIUS); SIGNAL_PEPTIDE_N_REGION (PHOBIUS); SIGNAL_PEPTIDE_C_REGION (PHOBIUS); SIGNAL_PEPTIDE_H_REGION (PHOBIUS); SIGNAL_PEPTIDE (PHOBIUS); IPR024716 (PRODOM); SignalP-noTM (SIGNALP_EUK); SignalP-TM (SIGNALP_GRAM_POSITIVE); TMhelix (TMHMM)
118	142449_length_226_cvg_3.0_tip_1_3	6	carboxypeptidase b-like	76	3.85E-25	78.80%	0.137	G3DSA:3.40.630.10 (GENE3D); IPR000834 (PFAM); PTHR11705:SF61 (PANTHER); PTHR11705 (PANTHER); SSF53187 (SUPERFAMILY)
119	142567_length_227_cvg_2.0_tip_1_1	3	er degradation-enhancing alpha-mannosidase-like 3	76	3.42E-31	90.80%	0.108	IPR001382 (PFAM); IPR001382 (G3DSA:1.50.10.GENE3D); IPR001382 (PANTHER); PTHR11742:SF38 (PANTHER); IPR001382 (SUPERFAMILY)
122	143285_length_228_cvg_2.0_tip_1_3	3	lutropin-choriogonadotropic hormone receptor	76	3.10E-06	59.50%	0.113	G3DSA:3.80.10.10 (GENE3D); IPR001611 (PFAM); PTHR24373 (PANTHER); IPR001611 (PROSITE_PROFILES); IPR001611 (PROSITE_PROFILES); IPR001611 (PROSITE_PROFILES); SSF52058 (SUPERFAMILY)
123	143623_length_228_cvg_2.0_tip_1_5	3	aael015439- partial	76	5.13E-30	79.20%	0.163	IPR001254 (PFAM); G3DSA:2.40.10.10 (GENE3D); G3DSA:2.40.10.10 (GENE3D); PTHR24256 (PANTHER); PTHR24256:SF103 (PANTHER); IPR009003 (SUPERFAMILY); TMhelix (TMHMM)
124	143747_length_228_cvg_3.0_tip_1_5	6	d chain coupling of remote alternating-access transport mechanisms for protons and substrates in the multidrug efflux pump acrb	76	3.13E-13	62.20%	0.144	IPR002110 (SMART); IPR020683 (PFAM); IPR020683 (G3DSA:1.25.40.GENE3D); PTHR24182 (PANTHER); IPR020683 (PROSITE_PROFILES); IPR002110 (PROSITE_PROFILES); IPR002110 (PROSITE_PROFILES); IPR020683 (SUPERFAMILY)
125	144217_length_229_cvg_9.2_tip_1_5	11	PREDICTED: hyaluronidase-1-like	76	2.47E-06	63.00%	0.105	IPR018155 (PFAM); IPR013785 (G3DSA:3.20.20.GENE3D); IPR017853 (SUPERFAMILY)
126	144573_length_230_cvg_2.0_tip_1_3	3	heat shock protein partial	77	2.36E-35	88.10%	0.104	IPR013126 (PRINTS); G3DSA:3.30.420.40 (GENE3D); G3DSA:3.30.30.30 (GENE3D); IPR013126 (PFAM); PTHR19375:SF137 (PANTHER); PTHR19375 (PANTHER); IPR018181 (PROSITE_PATTERNS); SSF53067 (SUPERFAMILY)
127	145295_length_231_cvg_2.0_tip_1_2	5	prolyl 4- beta subunit	77	7.18E-18	70.60%	0.113	IPR012336 (G3DSA:3.40.30.GENE3D); PTHR18929 (PANTHER); PTHR18929:SF74 (PANTHER); IPR012336 (SUPERFAMILY)
128	145327_length_231_cvg_2.0_tip_1_3	3	zinc finger protein 109	77	2.31E-14	60.20%	0.146	IPR015880 (SMART); PF13465 (PFAM); IPR013087 (G3DSA:3.30.160.GENE3D); IPR013087 (G3DSA:3.30.160.GENE3D); IPR007087 (PFAM); IPR013087 (G3DSA:3.30.160.GENE3D); PTHR24409 (PANTHER); IPR007087 (PROSITE_PATTERNS); IPR007087 (PROSITE_PATTERNS); IPR007087 (PROSITE_PROFILES); IPR007087 (PROSITE_PROFILES); SSF57667 (SUPERFAMILY); SSF57667 (SUPERFAMILY)
129	145649_length_231_cvg_3.0_tip_1_3	5	zinc c2h2 type	77	3.25E-11	62.50%	0.118	IPR015880 (SMART); IPR013087 (G3DSA:3.30.160.GENE3D); PF13465 (PFAM); IPR013087 (G3DSA:3.30.160.GENE3D); PTHR24409 (PANTHER); PTHR24409:SF13 (PANTHER); IPR007087 (PROSITE_PATTERNS); IPR007087 (PROSITE_PATTERNS); IPR007087 (PROSITE_PROFILES); IPR007087 (PROSITE_PROFILES); SSF57667 (SUPERFAMILY)

130	145901_length_232_cvg_2.0_tip_1_4	4	fat-like cadherin-related tumor suppressor homolog isoform x3	77	3.34E-13	63.80%	0.114	IPR002126 (G3DSA:2.60.40.GENE3D); IPR002126 (PFAM); PTHR24028 (PANTHER); IPR002126 (PROSITE_PROFILES); IPR015919 (SUPERFAMILY)
131	145965_length_232_cvg_2.0_tip_1_1	2	zinc finger protein 502-like	77	2.12E-14	67.00%	0.099	IPR015880 (SMART); IPR013087 (G3DSA:3.30.160.GENE3D); IPR013087 (G3DSA:3.30.160.GENE3D); PF13894 (PFAM); PTHR24409 (PANTHER); PTHR24409:SF14 (PANTHER); IPR007087 (PROSITE_PATTERNS); IPR007087 (PROSITE_PATTERNS); IPR007087 (PROSITE_PROFILES); IPR007087 (PROSITE_PROFILES); SSF57667 (SUPERFAMILY)
133	146185_length_232_cvg_2.0_tip_1_4	7	26s protease regulatory subunit 6a-b	77	1.32E-45	99.60%	0.102	IPR003959 (PFAM); IPR027417 (G3DSA:3.40.50.GENE3D); PTHR23073:SF7 (PANTHER); PTHR23073 (PANTHER); IPR027417 (SUPERFAMILY)
135	146945_length_233_cvg_2.4_tip_1_5	6	gamma-glutamyl hydrolase	77	1.78E-13	63.80%	0.116	IPR029062 (G3DSA:3.40.50.GENE3D); IPR015527 (PANTHER)
132	145969_length_232_cvg_2.0_tip_1_3	4	beta- -n-acetylgalactosaminyltransferase bre-4	78	4.90E-42	88.40%	0.128	IPR003859 (PRINTS); IPR029044 (G3DSA:3.90.550.GENE3D); IPR027791 (PFAM); PTHR19300:SF30 (PANTHER); IPR003859 (PANTHER); IPR029044 (SUPERFAMILY)
134	146727_length_233_cvg_2.0_tip_1_3	5	pancreatic triacylglycerol lipase-like	78	2.12E-28	78.60%	0.108	IPR013818 (PFAM); IPR029058 (G3DSA:3.40.50.GENE3D); IPR000734 (PANTHER); IPR029058 (SUPERFAMILY)
136	147193_length_234_cvg_2.0_tip_1_1	1	carboxypeptidase b-like	78	3.05E-21	80.70%	0.148	IPR000834 (PRINTS); IPR000834 (PFAM); G3DSA:3.40.630.10 (GENE3D); PTHR11705 (PANTHER); IPR000834 (PROSITE_PATTERNS); SSF53187 (SUPERFAMILY)
137	147511_length_234_cvg_2.0_tip_1_1	5	phospholipase a2 isozyme partial	78	1.90E-09	67.10%	0.098	IPR001211 (PFAM); IPR016090 (G3DSA:1.20.90.GENE3D); IPR016090 (SUPERFAMILY)
138	147779_length_234_cvg_2.0_tip_1_1	4	syntaxin partial	78	9.56E-18	78.90%	0.1	Coil (COILS); IPR000727 (SMART); IPR000727 (PFAM); G3DSA:1.20.5.110 (GENE3D); IPR028669 (PTHR19957:PANTHER); PTHR19957 (PANTHER); IPR006012 (PROSITE_PATTERNS); IPR000727 (PROSITE_PROFILES); IPR010989 (SUPERFAMILY)
139	147899_length_234_cvg_2.0_tip_1_0	3	vacuolar-sorting protein snf8-like	78	3.01E-18	92.20%	0.19	Coil (COILS); IPR007286 (PFAM); PTHR12806:SF0 (PANTHER); IPR007286 (PANTHER)
140	148541_length_235_cvg_12.0_tip_1_1	2	vktti_najna ame: full=kunitz-type serine protease inhibitor ame: full=venom trypsin inhibitor	78	1.60E-14	72.40%	0.122	IPR002223 (PRINTS); IPR002223 (SMART); IPR002223 (G3DSA:4.10.410.GENE3D); IPR002223 (PFAM); PTHR10083 (PANTHER); IPR020901 (PROSITE_PATTERNS); IPR002223 (PROSITE_PROFILES); IPR002223 (SUPERFAMILY)
141	149653_length_236_cvg_2.0_tip_1_1	4	zinc c2h2 type domain-containing protein	79	4.73E-18	62.80%	0.108	IPR015880 (SMART); PF13465 (PFAM); IPR013087 (G3DSA:3.30.160.GENE3D); PTHR24409 (PANTHER); IPR007087 (PROSITE_PATTERNS); IPR007087 (PROSITE_PATTERNS); IPR007087 (PROSITE_PROFILES); IPR007087 (PROSITE_PROFILES); SSF57667 (SUPERFAMILY); SSF57667 (SUPERFAMILY)
142	149803_length_237_cvg_2.0_tip_1_5	5	plexin- partial	79	1.62E-40	96.80%	0.404 Y	IPR013548 (PFAM); PTHR22625 (PANTHER); IPR008936 (SUPERFAMILY)
143	150497_length_237_cvg_12.2_tip_1_4	20	adp-ribosylation factor	79	8.89E-10	88.80%	0.249	IPR027417 (G3DSA:3.40.50.GENE3D); IPR006689 (PFAM); PTHR11711:SF142 (PANTHER); PTHR11711 (PANTHER); IPR027417 (SUPERFAMILY)
144	150837_length_238_cvg_2.0_tip_1_1	3	zinc finger protein 543	79	1.13E-12	57.30%	0.111	IPR015880 (SMART); IPR013087 (G3DSA:3.30.160.GENE3D); IPR013087 (G3DSA:3.30.160.GENE3D); PF13894 (PFAM); PF13465 (PFAM); PTHR24409:SF14 (PANTHER); PTHR24409 (PANTHER); IPR007087 (PROSITE_PATTERNS); IPR007087 (PROSITE_PATTERNS); IPR007087 (PROSITE_PROFILES); IPR007087 (PROSITE_PROFILES); SSF57667 (SUPERFAMILY)
145	150891_length_238_cvg_2.7_tip_1_2	4	cd4-specific ankyrin repeat protein	79	3.34E-10	61.10%	0.118	IPR002110 (PRINTS); IPR002110 (SMART); IPR020683 (G3DSA:1.25.40.GENE3D); IPR020683 (PFAM); PTHR24198 (PANTHER); IPR002110 (PROSITE_PROFILES); IPR020683 (PROSITE_PROFILES); IPR002110 (PROSITE_PROFILES); IPR020683 (SUPERFAMILY)
146	151231_length_238_cvg_2.0_tip_1_5	3	endothelin-converting enzyme 1 isoform x3	79	1.48E-24	79.20%	0.394 Y	IPR008753 (PFAM); G3DSA:1.10.1380.10 (GENE3D); IPR029733 (PTHR11733:PANTHER); IPR000718 (PANTHER); SSF55486 (SUPERFAMILY)
147	151369_length_238_cvg_2.0_tip_1_2	3	mam and ldl-receptor class a domain-containing protein 1-like	79	3.15E-10	58.38%	0.118	IPR000998 (PFAM); IPR000998 (PROSITE_PROFILES); IPR013320 (SUPERFAMILY)
148	151459_length_238_cvg_3.0_tip_1_4	4	protein creg1	79	8.82E-20	74.90%	0.12	IPR012349 (G3DSA:2.30.110.GENE3D); PF13883 (PFAM); PTHR13343:SF17 (PANTHER); PTHR13343 (PANTHER); IPR012349 (SUPERFAMILY)
149	151739_length_239_cvg_45.0_tip_1_3	62	14-3-3 protein zeta isoform x1	80	1.06E-43	91.60%	0.108	IPR000308 (PRINTS); G3DSA:1.20.190.20 (GENE3D); IPR023410 (PFAM); IPR000308 (PANTHER); IPR023410 (SUPERFAMILY)
150	152047_length_239_cvg_2.9_tip_1_4	8	n-sulphoglucosamine sulphohydrolase	80	2.21E-22	86.20%	0.115	IPR000917 (PFAM); IPR017849 (G3DSA:3.40.720.GENE3D); PTHR10342 (PANTHER); PTHR10342:SF76 (PANTHER); IPR024607 (PROSITE_PATTERNS); IPR017850 (SUPERFAMILY)
151	152999_length_240_cvg_2.0_tip_1_0	5	low-density lipoprotein receptor-related protein 2	80	9.38E-38	85.80%	0.107	IPR002172 (PRINTS); IPR002172 (SMART); IPR002172 (PFAM); IPR002172 (G3DSA:4.10.400.GENE3D); PTHR10529 (PANTHER); IPR002172 (PROSITE_PROFILES); IPR002172 (SUPERFAMILY)
152	153083_length_240_cvg_2.0_tip_1_5	5	basement membrane-specific heparan sulfate proteoglycan core protein	80	2.05E-25	65.90%	0.108	IPR013783 (G3DSA:2.60.40.GENE3D)
153	153595_length_241_cvg_63.0_tip_0_2	3159	probable chitinase 3	80	1.66E-24	72.50%	0.099	IPR001223 (PFAM); IPR013781 (G3DSA:3.20.20.GENE3D); PTHR11177 (PANTHER); PTHR11177:SF109 (PANTHER); IPR017853 (SUPERFAMILY)

154	153649_length_241_cvg_3.0_tip_1_5	7	---NA---	80			0.099	IPR002413 (PRINTS); IPR014044 (G3DSA:3.40.33.GENE3D)
155	153699_length_241_cvg_2.0_tip_1_1	1	serine threonine-protein kinase 10 isoform x3	80	5.31E-45	95.20%	0.108	G3DSA:1.10.510.10 (GENE3D); IPR000719 (PFAM); PTHR24361:SF249 (PANTHER); PTHR24361 (PANTHER); IPR008271 (PROSITE_PATTERNS); IPR000719 (PROSITE_PROFILES); IPR011009 (SUPERFAMILY)
156	153789_length_241_cvg_2.4_tip_1_5	5	chemosensory protein 1 partial	80	1.13E-31	80.30%	0.107	IPR005055 (PFAM); IPR005055 (G3DSA:1.10.2080.GENE3D); IPR005055 (PANTHER); PTHR11257:SF4 (PANTHER); IPR005055 (SUPERFAMILY)
157	154119_length_241_cvg_2.0_tip_1_2	4	fibrillin-2-like isoform x2	80	2.88E-32	81.90%	0.141	IPR017878 (PFAM); IPR017878 (G3DSA:3.90.290.GENE3D); IPR011398 (PANTHER); IPR017878 (PROSITE_PROFILES); IPR017878 (SUPERFAMILY)
158	154173_length_241_cvg_2.0_tip_1_2	4	zinc finger and btb domain-containing protein	80	5.33E-14	62.70%	0.106	IPR015880 (SMART); IPR007087 (PFAM); PF13894 (PFAM); IPR013087 (G3DSA:3.30.160.GENE3D); IPR013087 (G3DSA:3.30.160.GENE3D); PTHR24409:SF13 (PANTHER); PTHR24409 (PANTHER); IPR007087 (PROSITE_PATTERNS); IPR007087 (PROSITE_PATTERNS); IPR007087 (PROSITE_PROFILES); IPR007087 (PROSITE_PROFILES); SSF57667 (SUPERFAMILY)
159	154475_length_241_cvg_2.0_tip_1_3	5	ankyrin repeat-containing protein	81	1.63E-07	65.00%	0.105	IPR002110 (SMART); IPR020683 (G3DSA:1.25.40.GENE3D); IPR020683 (PFAM); IPR002110 (PROSITE_PROFILES); IPR020683 (PROSITE_PROFILES); IPR002110 (PROSITE_PROFILES); IPR020683 (SUPERFAMILY)
160	154631_length_242_cvg_3.0_tip_1_0	5	af296336_1collagen-like surface protein	81	8.84E-06	56.00%	0.1	IPR008160 (PFAM)
161	154955_length_242_cvg_2.0_tip_1_0	3	prenylcysteine oxidase	81	2.84E-13	62.60%	0.097	IPR010795 (PFAM); IPR017046 (PANTHER)
162	155341_length_242_cvg_2.0_tip_1_3	3	d chain in silico and in vitro co-evolution of a high affinity complementary protein-protein interface	81	5.59E-10	62.80%	0.112	IPR002110 (SMART); IPR020683 (PFAM); IPR020683 (G3DSA:1.25.40.GENE3D); PTHR24193 (PANTHER); IPR002110 (PROSITE_PROFILES); IPR020683 (PROSITE_PROFILES); IPR002110 (PROSITE_PROFILES); IPR020683 (SUPERFAMILY)
163	155465_length_242_cvg_2.0_tip_1_4	7	neurogenic locus notch homolog protein 3-like	81	2.41E-32	76.80%	0.133	IPR004094 (G3DSA:2.10.22.GENE3D); IPR004094 (PFAM); PTHR19441 (PANTHER); PTHR19441:SF10 (PANTHER); IPR004094 (PROSITE_PROFILES); IPR011061 (SUPERFAMILY)
164	155839_length_243_cvg_3.0_tip_1_3	7	gastrula zinc finger	81	1.72E-23	73.50%	0.099	IPR015880 (SMART); PF13465 (PFAM); IPR013087 (G3DSA:3.30.160.GENE3D); IPR013087 (G3DSA:3.30.160.GENE3D); PTHR24409 (PANTHER); IPR007087 (PROSITE_PATTERNS); IPR007087 (PROSITE_PATTERNS); IPR007087 (PROSITE_PATTERNS); IPR007087 (PROSITE_PROFILES); IPR007087 (PROSITE_PROFILES); IPR007087 (PROSITE_PROFILES); SSF57667 (SUPERFAMILY); SSF57667 (SUPERFAMILY)
165	155869_length_243_cvg_2.0_tip_1_0	4	low quality protein: cadherin-related tumor suppressor-like	81	1.37E-08	62.70%	0.126	IPR000742 (SMART); G3DSA:2.10.25.10 (GENE3D); IPR000742 (PFAM); IPR013032 (PROSITE_PATTERNS); IPR000742 (PROSITE_PROFILES); SSF57196 (SUPERFAMILY)
166	156013_length_243_cvg_2.0_tip_1_2	6	chemosensory protein-14	81	2.58E-39	89.00%	0.105	IPR005055 (PFAM); IPR005055 (G3DSA:1.10.2080.GENE3D); PTHR11257:SF4 (PANTHER); IPR005055 (PANTHER); IPR005055 (SUPERFAMILY)
167	156111_length_243_cvg_2.0_tip_1_2	7	xanthine dehydrogenase oxidase	81	9.87E-28	77.60%	0.111	IPR008274 (G3DSA:3.30.365.GENE3D); IPR008274 (PFAM); PTHR11908 (PANTHER); PTHR11908:SF32 (PANTHER); IPR008274 (SUPERFAMILY)
168	156173_length_243_cvg_2.0_tip_1_2	4	papain family cysteine protease	81	1.77E-29	78.30%	0.145	IPR000668 (PFAM); G3DSA:3.90.70.10 (GENE3D); PTHR12411:SF15 (PANTHER); IPR013128 (PANTHER); SSF54001 (SUPERFAMILY)
169	156369_length_243_cvg_2.0_tip_1_5	5	down syndrome cell adhesion molecule-like protein dscam2	81	1.37E-25	74.90%	0.163	IPR003961 (PFAM); IPR013783 (G3DSA:2.60.40.GENE3D); PTHR10489 (PANTHER); PTHR10489:SF580 (PANTHER); IPR003961 (PROSITE_PROFILES); IPR003961 (SUPERFAMILY)
170	156399_length_243_cvg_2.0_tip_1_2	5	trehalase isoform x3	81	1.92E-15	66.70%	0.105	PTHR23403:SF1 (PANTHER); IPR001661 (PANTHER); IPR008928 (SUPERFAMILY)
171	156445_length_243_cvg_4.0_tip_1_3	7	5 -nucleotidase-like	81	4.02E-20	71.10%	0.122	IPR029052 (G3DSA:3.60.21.GENE3D); IPR006179 (PANTHER); PTHR11575:SF21 (PANTHER); IPR006146 (PROSITE_PATTERNS); IPR029052 (SUPERFAMILY)
172	156507_length_243_cvg_13.0_tip_1_0	22	plasma alpha-l-fucosidase-like	81	1.05E-28	78.80%	0.581 Y	IPR013781 (G3DSA:3.20.20.GENE3D); IPR000933 (PFAM); PTHR10030:SF26 (PANTHER); IPR000933 (PANTHER); TRANSMEMBRANE (PHOBIUS); SIGNAL_PEPTIDE_H_REGION (PHOBIUS); SIGNAL_PEPTIDE (PHOBIUS); NON_CYTOPLASMIC_DOMAIN (PHOBIUS); CYTOPLASMIC_DOMAIN (PHOBIUS); SIGNAL_PEPTIDE_C_REGION (PHOBIUS); SIGNAL_PEPTIDE_N_REGION (PHOBIUS); SignalP-TM (SIGNALP_EUK); IPR017853 (SUPERFAMILY); TMhelix (TMHMM); TMhelix (TMHMM)
173	156993_length_243_cvg_2.7_tip_1_4	5	von willebrand factor type egf and pentraxin domain-containing protein 1-like	81	1.35E-19	73.00%	0.106	IPR000436 (SMART); IPR000436 (PFAM); G3DSA:2.10.70.10 (GENE3D); IPR000436 (PROSITE_PROFILES); IPR000436 (SUPERFAMILY)
174	157621_length_244_cvg_2.6_tip_1_1	4	dnaj homolog subfamily c member 3-like	81	1.17E-33	85.80%	0.106	IPR019734 (SMART); PF13414 (PFAM); IPR011990 (G3DSA:1.25.40.GENE3D); IPR013026 (PROSITE_PROFILES); IPR019734 (PROSITE_PROFILES); IPR019734 (PROSITE_PROFILES); SSF48452 (SUPERFAMILY)
175	157681_length_244_cvg_2.5_tip_1_4	4	membrane metallo-endopeptidase-like 1 isoform x4	81	1.22E-12	62.40%	0.102	G3DSA:1.10.1380.10 (GENE3D); IPR008753 (PFAM); PTHR11733:SF111 (PANTHER); IPR000718 (PANTHER); SSF55486 (SUPERFAMILY)

176	157713_length_244_cvg_2.0_tip_1_2	4	tyrosine-protein kinase src42a	81	1.36E-11	59.60%	0.205	IPR003591 (SMART); IPR001611 (PFAM); G3DSA:3.80.10.10 (GENE3D); PTHR24373 (PANTHER); IPR001611 (PROSITE_PROFILES); IPR001611 (PROSITE_PROFILES); IPR001611 (PROSITE_PROFILES); SSF52058 (SUPERFAMILY)
177	157865_length_244_cvg_2.9_tip_1_4	4	endothelin-converting enzyme, putative	81	6.34E-06	62.00%	0.104	IPR008753 (PFAM); G3DSA:1.10.1380.10 (GENE3D); SSF55486 (SUPERFAMILY)
183	159332_length_245_cvg_2.0_tip_1_2	6	phospholipase b-like 2	81	7.94E-31	84.20%	0.123	IPR007000 (PFAM); PTHR12370:SF3 (PANTHER); IPR007000 (PANTHER)
178	157985_length_244_cvg_119.9_tip_0_0	5053	kazal-type protease inhibitor	82	3.46E-12	56.00%	0.112	IPR002350 (SMART); G3DSA:3.30.60.30 (GENE3D); G3DSA:3.30.60.30 (GENE3D); IPR002350 (PFAM); IPR002350 (PFAM); IPR002350 (PROSITE_PROFILES); IPR002350 (PROSITE_PROFILES); SSF100895 (SUPERFAMILY); SSF100895 (SUPERFAMILY)
179	158298_length_244_cvg_2.0_tip_1_0	5	sortilin-related receptor-like	82	2.15E-28	81.60%	0.132	G3DSA:2.130.10.140 (GENE3D); PTHR12106:SF7 (PANTHER); PTHR12106 (PANTHER); SSF110296 (SUPERFAMILY)
180	158366_length_245_cvg_2.0_tip_1_3	4	c-type lectin ctl- partial	82	7.62E-07	55.70%	0.894 Y	IPR016186 (G3DSA:3.10.100.GENE3D); NON_CYTOPLASMIC_DOMAIN (PHOBIUS); SIGNAL_PEPTIDE_N_REGION (PHOBIUS); SIGNAL_PEPTIDE (PHOBIUS); SIGNAL_PEPTIDE_H_REGION (PHOBIUS); SIGNAL_PEPTIDE_C_REGION (PHOBIUS); IPR001304 (PROSITE_PROFILES); SignalP-noTM (SIGNALP_EUK); IPR016187 (SUPERFAMILY)
181	158552_length_245_cvg_3.0_tip_1_1	7	parkinson disease 7 domain-containing protein 1	82	2.14E-17	82.80%	0.101	IPR029062 (G3DSA:3.40.50.GENE3D); IPR029062 (SUPERFAMILY)
182	159262_length_245_cvg_2.0_tip_1_4	4	core histone macro-	82	1.55E-34	86.20%	0.107	IPR002119 (PRINTS); IPR002119 (SMART); IPR009072 (G3DSA:1.10.20.GENE3D); IPR007125 (PFAM); PTHR23430 (PANTHER); PTHR23430:SF33 (PANTHER); IPR009072 (SUPERFAMILY)
184	159406_length_245_cvg_2.0_tip_1_4	5	thrombospondin type-1 domain-containing protein 4	82	3.18E-28	56.40%	0.108	IPR000884 (SMART); G3DSA:2.20.100.10 (GENE3D); IPR000884 (PFAM); PTHR13723 (PANTHER); IPR000884 (PROSITE_PROFILES); IPR000884 (SUPERFAMILY); IPR000884 (SUPERFAMILY)
185	159576_length_245_cvg_2.0_tip_1_3	5	trehalose 6-phosphate synthase 1a isoform	82	7.54E-36	91.30%	0.097	G3DSA:3.40.50.2000 (GENE3D); IPR001830 (PFAM); PTHR10788:SF6 (PANTHER); PTHR10788 (PANTHER); SSF53756 (SUPERFAMILY)
186	159872_length_246_cvg_2.0_tip_1_3	7	vitellogenin partial	82	1.08E-17	58.50%	0.101	no IPS match
189	161756_length_247_cvg_3.0_tip_1_1	7	tyrosine-protein phosphatase	82	6.19E-36	84.80%	0.132	IPR000242 (PFAM); IPR029021 (G3DSA:3.90.190.GENE3D); PTHR19134 (PANTHER); IPR000242 (PROSITE_PROFILES); IPR029021 (SUPERFAMILY)
190	162168_length_247_cvg_63.0_tip_0_4	4625	trypsin 3	82	1.52E-29	77.80%	0.114	IPR001254 (PFAM); G3DSA:2.40.10.10 (GENE3D); PTHR24256 (PANTHER); IPR018114 (PROSITE_PATTERNS); IPR001254 (PROSITE_PROFILES); IPR009003 (SUPERFAMILY)
193	163066_length_248_cvg_4.0_tip_1_5	9	probable carboxypeptidase pm20d1	82	4.56E-20	66.70%	0.138	IPR011650 (G3DSA:3.30.70.GENE3D); IPR011650 (PFAM); PTHR11014 (PANTHER); PTHR11014:SF66 (PANTHER); TMhelix (TMHMM)
194	163226_length_248_cvg_2.0_tip_1_5	3	agap003656-pa-like protein	82	6.32E-30	81.80%	0.111	IPR002172 (PRINTS); IPR002172 (SMART); IPR002172 (G3DSA:4.10.400.GENE3D); IPR002172 (G3DSA:4.10.400.GENE3D); IPR002172 (PFAM); PTHR10529 (PANTHER); IPR023415 (PROSITE_PATTERNS); IPR023415 (PROSITE_PATTERNS); IPR002172 (PROSITE_PROFILES); IPR002172 (PROSITE_PROFILES); IPR002172 (SUPERFAMILY); IPR002172 (SUPERFAMILY)
187	161218_length_247_cvg_2.0_tip_1_3	5	achain 3ank: a designed ankyrin repeat protein with three identical consensus repeats	83	6.61E-13	65.60%	0.112	IPR002110 (SMART); IPR020683 (G3DSA:1.25.40.GENE3D); IPR020683 (PFAM); PTHR24193 (PANTHER); IPR002110 (PROSITE_PROFILES); IPR002110 (PROSITE_PROFILES); IPR020683 (PROSITE_PROFILES); IPR020683 (SUPERFAMILY)
188	161490_length_247_cvg_2.0_tip_1_3	4	peptidyl-prolyl cis-trans isomerase h	83	6.37E-45	95.00%	0.105	IPR002130 (PRINTS); IPR029000 (G3DSA:2.40.100.GENE3D); IPR002130 (PFAM); IPR024936 (PANTHER); PTHR11071:SF58 (PANTHER); IPR002130 (PROSITE_PROFILES); IPR029000 (SUPERFAMILY)
191	162352_length_248_cvg_2.0_tip_1_0	5	hypothetical protein Y032_0312g2164	83	6.13E-11	63.10%	0.106	no IPS match
192	162710_length_248_cvg_2.0_tip_1_0	4	adp-dependent glucokinase-like	83	6.39E-24	73.30%	0.107	IPR007666 (PFAM); IPR029056 (G3DSA:3.40.1190.GENE3D); IPR007666 (PANTHER); PTHR21208:SF1 (PANTHER); IPR007666 (PROSITE_PROFILES); IPR029056 (SUPERFAMILY)
195	163295_length_248_cvg_2.4_tip_1_1	5	insulin-like growth factor-binding protein complex acid labile chain	83	3.96E-11	61.50%	0.139	PR00019 (PRINTS); IPR003591 (SMART); G3DSA:3.80.10.10 (GENE3D); IPR001611 (PFAM); PTHR24365 (PANTHER); IPR001611 (PROSITE_PROFILES); IPR001611 (PROSITE_PROFILES); IPR001611 (PROSITE_PROFILES); SSF52058 (SUPERFAMILY)
196	163613_length_249_cvg_2.0_tip_1_1	6	carboxypeptidase d	83	4.14E-18	68.30%	0.108	PF13620 (PFAM); IPR014766 (G3DSA:2.60.40.GENE3D); PTHR11532 (PANTHER); IPR027062 (PTHR11532:PANTHER); IPR008969 (SUPERFAMILY)
197	163879_length_249_cvg_2.0_tip_1_1	4	brevican core protein	83	1.15E-11	52.00%	0.125	IPR016186 (G3DSA:3.10.100.GENE3D); IPR001304 (PFAM); PTHR22802 (PANTHER); IPR001304 (PROSITE_PROFILES); IPR016187 (SUPERFAMILY)
198	164509_length_249_cvg_2.0_tip_1_1	6	zinc finger protein 420-like	83	3.28E-14	64.60%	0.1	IPR015880 (SMART); PF13465 (PFAM); IPR013087 (G3DSA:3.30.160.GENE3D); IPR013087 (G3DSA:3.30.160.GENE3D); PTHR24409 (PANTHER); PTHR24409:SF14 (PANTHER); IPR007087 (PROSITE_PATTERNS); IPR007087 (PROSITE_PATTERNS); IPR007087 (PROSITE_PROFILES); IPR007087 (PROSITE_PROFILES); SSF57667 (SUPERFAMILY)

199	164761_length_249_cvg_2.0_tip_1_0	2	follistatin-related protein 5	83	1.14E-40	87.80%	0.098	IPR011992 (G3DSA:1.10.238.GENE3D); PTHR10913:SF14 (PANTHER); PTHR10913 (PANTHER); IPR018247 (PROSITE_PATTERNS); SSF47473 (SUPERFAMILY)
200	165005_length_250_cvg_4.5_tip_1_2	6	glucose dehydrogenase	83	2.13E-16	65.70%	0.101	IPR007867 (PFAM); G3DSA:3.30.560.10 (GENE3D); PTHR11552 (PANTHER); PTHR11552:SF77 (PANTHER); SSF54373 (SUPERFAMILY)
201	165059_length_250_cvg_2.0_tip_1_4	4	nucleolar protein partial	83	6.52E-31	80.30%	0.113	PTHR10894 (PANTHER)
202	165317_length_250_cvg_3.0_tip_1_5	6	leucine-rich repeats containing	83	9.13E-19	62.70%	0.112	IPR001611 (PFAM); IPR001611 (PFAM); G3DSA:3.80.10.10 (GENE3D); PTHR24365 (PANTHER); IPR001611 (PROSITE_PROFILES); IPR001611 (PROSITE_PROFILES); SSF52058 (SUPERFAMILY)
203	165351_length_250_cvg_2.0_tip_1_5	7	neutral endopeptidase	83	1.26E-24	72.50%	0.113	IPR018497 (PRINTS); IPR024079 (G3DSA:3.40.390.GENE3D); IPR018497 (PFAM); PTHR11733:SF113 (PANTHER); IPR000718 (PANTHER); NON_CYTOPLASMIC_DOMAIN (PHOBIUS); CYTOPLASMIC_DOMAIN (PHOBIUS); TRANSMEMBRANE (PHOBIUS); SSF55486 (SUPERFAMILY); TMhelix (TMHMM)
205	166061_length_250_cvg_3.0_tip_1_4	7	isoform cra_c	83	1.91E-10	66.80%	0.136	IPR001254 (PFAM); G3DSA:2.40.10.10 (GENE3D); PTHR24264 (PANTHER); PTHR24264:SF9 (PANTHER); IPR009003 (SUPERFAMILY)
206	166185_length_250_cvg_2.0_tip_1_2	7	low-density lipoprotein	83	1.21E-34	84.60%	0.13	IPR002172 (PRINTS); IPR002172 (SMART); IPR002172 (PFAM); IPR002172 (G3DSA:4.10.400.GENE3D); PTHR10529 (PANTHER); PTHR10529:SF204 (PANTHER); IPR023415 (PROSITE_PATTERNS); IPR002172 (PROSITE_PROFILES); IPR002172 (PROSITE_PROFILES); IPR002172 (SUPERFAMILY); IPR002172 (SUPERFAMILY)
204	166041_length_250_cvg_2.7_tip_1_0	7	heat shock cognate 71 kda	84	5.72E-30	84.10%	0.178	IPR013126 (PRINTS); IPR013126 (PFAM); G3DSA:3.30.30.30 (GENE3D); G3DSA:3.30.420.40 (GENE3D); PTHR19375 (PANTHER); IPR018181 (PROSITE_PATTERNS); SSF53067 (SUPERFAMILY)
207	166767_length_251_cvg_2.0_tip_1_1	5	protein lap1-like	84	9.95E-25	78.30%	0.097	IPR001611 (PFAM); G3DSA:3.80.10.10 (GENE3D); IPR025875 (PFAM); PTHR23155 (PANTHER); PTHR23155:SF466 (PANTHER); IPR001611 (PROSITE_PROFILES); IPR001611 (PROSITE_PROFILES); IPR001611 (PROSITE_PROFILES); IPR001611 (PROSITE_PROFILES); SSF52075 (SUPERFAMILY)
208	166999_length_251_cvg_2.0_tip_1_4	5	agrn partial	84	5.06E-13	62.70%	0.113	IPR001791 (PFAM); IPR013320 (G3DSA:2.60.120.GENE3D); IPR001791 (PROSITE_PROFILES); IPR013320 (SUPERFAMILY)
209	167047_length_251_cvg_3.0_tip_1_0	6	#NAME?	84	2.59E-23	83.30%	0.418 Y	IPR001623 (PRINTS); IPR001623 (SMART); IPR001623 (PFAM); IPR001623 (G3DSA:1.10.287.GENE3D); PTHR24077 (PANTHER); IPR001623 (PROSITE_PROFILES); IPR001623 (SUPERFAMILY)
210	167061_length_251_cvg_3.0_tip_1_4	8	mam and ldl-receptor class a domain-containing protein 1	84	1.97E-13	57.20%	0.102	IPR002172 (G3DSA:4.10.400.GENE3D); IPR000998 (PFAM); IPR000998 (PROSITE_PROFILES); IPR013320 (SUPERFAMILY); IPR002172 (SUPERFAMILY)
211	167177_length_251_cvg_3.0_tip_1_4	7	zinc finger protein rotund isoform x1	84	2.75E-48	98.00%	0.106	IPR015880 (SMART); IPR013087 (G3DSA:3.30.160.GENE3D); IPR013087 (G3DSA:3.30.160.GENE3D); IPR013087 (G3DSA:3.30.160.GENE3D); PF13465 (PFAM); IPR016608 (PTHR11389:PANTHER); PTHR11389 (PANTHER); IPR007087 (PROSITE_PATTERNS); IPR007087 (PROSITE_PATTERNS); IPR007087 (PROSITE_PATTERNS); IPR007087 (PROSITE_PROFILES); IPR007087 (PROSITE_PROFILES); IPR007087 (PROSITE_PROFILES); SSF57667 (SUPERFAMILY); SSF57667 (SUPERFAMILY)
212	167645_length_251_cvg_2.0_tip_1_4	7	mam and ldl-receptor class a domain-containing protein 1	84	2.54E-13	62.70%	0.405 Y	IPR000998 (PFAM); IPR002172 (PFAM); IPR002172 (G3DSA:4.10.400.GENE3D); PTHR23282 (PANTHER); PTHR23282:SF75 (PANTHER); IPR000998 (PROSITE_PROFILES); IPR002172 (PROSITE_PROFILES); IPR013320 (SUPERFAMILY); IPR002172 (SUPERFAMILY)
213	167831_length_252_cvg_2.0_tip_1_5	4	laminin subunit alpha	84	1.19E-35	88.60%	0.098	IPR008211 (SMART); IPR008211 (PFAM); PTHR10574:SF39 (PANTHER); PTHR10574 (PANTHER); IPR008211 (PROSITE_PROFILES)
214	167865_length_252_cvg_2.9_tip_1_5	7	iron zinc purple acid phosphatase-like protein	84	7.51E-39	85.80%	0.143	IPR029052 (G3DSA:3.60.21.GENE3D); IPR004843 (PFAM); PTHR22953:SF9 (PANTHER); PTHR22953 (PANTHER); IPR029052 (SUPERFAMILY)
215	168045_length_252_cvg_3.0_tip_1_0	6	protein disulfide-isomerase partial	84	5.09E-27	72.60%	0.107	IPR013766 (PFAM); IPR012336 (G3DSA:3.40.30.GENE3D); PTHR18929 (PANTHER); PTHR18929:SF56 (PANTHER); IPR012336 (PROSITE_PROFILES); IPR012336 (SUPERFAMILY)
216	168115_length_252_cvg_3.0_tip_1_5	8	zinc finger protein 429-like isoform x2	84	1.05E-39	90.10%	0.114	IPR015880 (SMART); PF13465 (PFAM); PF13894 (PFAM); IPR013087 (G3DSA:3.30.160.GENE3D); IPR013087 (G3DSA:3.30.160.GENE3D); PTHR24409 (PANTHER); IPR007087 (PROSITE_PATTERNS); IPR007087 (PROSITE_PROFILES); IPR007087 (PROSITE_PROFILES); SSF57667 (SUPERFAMILY)
217	168207_length_252_cvg_2.0_tip_1_0	5	serine protease like protein	84	2.25E-22	67.40%	0.259	G3DSA:2.40.10.10 (GENE3D); IPR001254 (PFAM); PTHR24268:SF94 (PANTHER); PTHR24268 (PANTHER); IPR018114 (PROSITE_PATTERNS); IPR001254 (PROSITE_PROFILES); IPR009003 (SUPERFAMILY)

218	168229_length_252_cvg_3.0_tip_1_4	5	muscle ankyrin repeat protein 3	84	1.89E-09	57.80%	0.117	IPR002110 (PRINTS); IPR002110 (SMART); IPR020683 (PFAM); IPR020683 (G3DSA:1.25.40.GENE3D); PTHR24198 (PANTHER); IPR002110 (PROSITE_PROFILES); IPR002110 (PROSITE_PROFILES); IPR020683 (PROSITE_PROFILES); IPR020683 (SUPERFAMILY)
219	168643_length_252_cvg_2.4_tip_1_3	3	phosphoglycerate kinase	84	8.23E-17	60.60%	0.103	IPR001576 (PFAM); IPR015901 (G3DSA:3.40.50.GENE3D); IPR001576 (PANTHER); PTHR11406:SF6 (PANTHER); IPR001576 (SUPERFAMILY)
220	168973_length_252_cvg_2.0_tip_1_2	6	zinc c2h2 type domain-containing protein	84	6.40E-15	58.40%	0.101	IPR015880 (SMART); IPR013087 (G3DSA:3.30.160.GENE3D); IPR013087 (G3DSA:3.30.160.GENE3D); PF13465 (PFAM); PTHR24409 (PANTHER); PTHR24409:SF14 (PANTHER); IPR007087 (PROSITE_PATTERNS); IPR007087 (PROSITE_PATTERNS); IPR007087 (PROSITE_PROFILES); IPR007087 (PROSITE_PROFILES); SSF57667 (SUPERFAMILY); SSF57667 (SUPERFAMILY)
222	169329_length_253_cvg_5.0_tip_1_4	6	laminin subunit gamma-1	84	8.37E-13	69.30%	0.104	no IPS match
224	169869_length_253_cvg_2.0_tip_1_2	5	a disintegrin and metalloproteinase with thrombospondin motifs 12 isoform x1	84	2.16E-09	64.60%	0.1	IPR002870 (PFAM); PTHR11905 (PANTHER)
227	171233_length_254_cvg_2.0_tip_1_5	4	zinc finger protein	84	2.98E-13	60.40%	0.107	IPR015880 (SMART); IPR007087 (PFAM); IPR013087 (G3DSA:3.30.160.GENE3D); IPR013087 (G3DSA:3.30.160.GENE3D); PF13894 (PFAM); PTHR24409:SF14 (PANTHER); PTHR24409 (PANTHER); IPR007087 (PROSITE_PATTERNS); IPR007087 (PROSITE_PATTERNS); IPR007087 (PROSITE_PROFILES); IPR007087 (PROSITE_PROFILES); SSF57667 (SUPERFAMILY); SSF57667 (SUPERFAMILY)
229	171623_length_254_cvg_3.3_tip_1_2	10	achain 3ank: a designed ankyrin repeat protein with three identical consensus repeats	84	1.55E-14	62.30%	0.132	IPR002110 (SMART); IPR020683 (G3DSA:1.25.40.GENE3D); IPR020683 (PFAM); PTHR24193 (PANTHER); IPR020683 (PROSITE_PROFILES); IPR002110 (PROSITE_PROFILES); IPR002110 (PROSITE_PROFILES); IPR020683 (SUPERFAMILY)
221	169169_length_253_cvg_2.0_tip_1_0	6	attractin-like protein 1 isoform x1	85	1.82E-25	74.80%	0.12	IPR015915 (G3DSA:2.120.10.GENE3D); PF13418 (PFAM); PTHR10574 (PANTHER); PTHR10574:SF3 (PANTHER); SSF117281 (SUPERFAMILY)
223	169741_length_253_cvg_3.0_tip_1_0	9	collagen alpha-1 chain-like	85	4.49E-10	62.30%	0.104	IPR008160 (PFAM); PTHR24023 (PANTHER); PTHR24023:SF372 (PANTHER)
225	170415_length_253_cvg_2.0_tip_1_0	6	aldehyde mitochondrial precursor	85	2.82E-37	85.10%	0.113	IPR016163 (G3DSA:3.40.309.GENE3D); IPR015590 (PFAM); PTHR11699 (PANTHER); IPR015657 (PTHR11699:PANTHER); IPR016161 (SUPERFAMILY)
226	170917_length_254_cvg_2.0_tip_1_3	2	retinol dehydrogenase 14 isoform x2	85	4.01E-27	80.70%	0.106	IPR002198 (PFAM); IPR016040 (G3DSA:3.40.50.GENE3D); PTHR24320 (PANTHER); CYTOPLASMIC_DOMAIN (PHOBIUS); TRANSMEMBRANE (PHOBIUS); NON_CYTOPLASMIC_DOMAIN (PHOBIUS); SSF51735 (SUPERFAMILY)
228	171329_length_254_cvg_2.0_tip_1_3	3	mucin-19 isoform x1	85	2.66E-08	59.80%	0.169	IPR001846 (PFAM); IPR001846 (PROSITE_PROFILES)
230	171683_length_254_cvg_3.9_tip_1_0	10	c-type mannose receptor 2	85	1.04E-06	62.57%	0.111	IPR001304 (PFAM); IPR016186 (G3DSA:3.10.100.GENE3D); IPR001304 (PROSITE_PROFILES); IPR016187 (SUPERFAMILY)
231	171863_length_255_cvg_2.0_tip_1_0	5	signal cub and egf-like domain-containing protein partial	85	1.30E-18	67.60%	0.113	IPR001881 (SMART); IPR000742 (SMART); PF14670 (PFAM); G3DSA:2.10.25.10 (GENE3D); G3DSA:2.10.25.10 (GENE3D); IPR026823 (PFAM); PTHR24034 (PANTHER); IPR013032 (PROSITE_PATTERNS); IPR018097 (PROSITE_PATTERNS); IPR000152 (PROSITE_PATTERNS); SSF57196 (SUPERFAMILY); SSF57196 (SUPERFAMILY)
232	171907_length_255_cvg_2.0_tip_1_4	6	group xv phospholipase a2	85	3.53E-40	84.70%	0.107	IPR003386 (PFAM); PTHR11440 (PANTHER); PTHR11440:SF47 (PANTHER)
233	171921_length_255_cvg_2.9_tip_1_1	9	thyroglobulin type-1 repeat-containing domain protein	85	3.69E-19	65.70%	0.108	IPR000716 (SMART); IPR000716 (G3DSA:4.10.800.GENE3D); IPR000716 (PFAM); PTHR12352 (PANTHER); IPR000716 (PROSITE_PATTERNS); IPR000716 (PROSITE_PROFILES); IPR000716 (SUPERFAMILY)
234	171959_length_255_cvg_4.0_tip_1_5	14	---NA---	85			0.114	IPR002172 (SMART); IPR002172 (PFAM); IPR002172 (G3DSA:4.10.400.GENE3D); IPR023415 (PROSITE_PATTERNS); IPR002172 (PROSITE_PROFILES); IPR002172 (SUPERFAMILY)
235	172043_length_255_cvg_2.0_tip_1_1	6	glucose dehydrogenase	85	2.45E-30	77.20%	0.166	G3DSA:3.50.50.60 (GENE3D); IPR000172 (PFAM); PTHR11552:SF77 (PANTHER); PTHR11552 (PANTHER); IPR000172 (PROSITE_PATTERNS); SSF51905 (SUPERFAMILY)
236	172289_length_255_cvg_17.0_tip_1_1	23	14-3-3 partial	85	3.61E-48	94.10%	0.106	IPR000308 (PRINTS); IPR023410 (SMART); G3DSA:1.20.190.20 (GENE3D); IPR023410 (PFAM); IPR000308 (PANTHER); IPR023410 (SUPERFAMILY)
237	172529_length_255_cvg_3.0_tip_1_2	9	collagen alpha-1 chain isoform x1	85	2.71E-07	63.70%	0.751 Y	IPR008160 (PFAM); PTHR24023 (PANTHER); PTHR24023:SF372 (PANTHER); NON_CYTOPLASMIC_DOMAIN (PHOBIUS); SIGNAL_PEPTIDE_C_REGION (PHOBIUS); SIGNAL_PEPTIDE_H_REGION (PHOBIUS); SIGNAL_PEPTIDE_N_REGION (PHOBIUS); SIGNAL_PEPTIDE (PHOBIUS); SignalP-TM (SIGNALP_GRAM_POSITIVE); SignalP-noTM (SIGNALP_EUK)
238	172529_length_255_cvg_3.0_tip_1_5	9	hypothetical protein	85	2.12E-06	51.00%	0.103	no IPS match
239	172807_length_255_cvg_3.0_tip_1_1	7	ecotropic virus integration site 1 protein	85	5.33E-13	62.30%	0.104	IPR015880 (SMART); IPR013087 (G3DSA:3.30.160.GENE3D); PF13465 (PFAM); IPR013087 (G3DSA:3.30.160.GENE3D); IPR013087 (G3DSA:3.30.160.GENE3D); PTHR24409 (PANTHER); IPR007087 (PROSITE_PATTERNS); IPR007087 (PROSITE_PATTERNS); IPR007087 (PROSITE_PROFILES); IPR007087 (PROSITE_PROFILES); SSF57667 (SUPERFAMILY)

240	172905_length_255_cvg_2.0_tip_1_4	7	laminin subunit gamma- partial	85	1.10E-39	83.30%	0.1	IPR008211 (SMART); IPR008979 (G3DSA:2.60.120.GENE3D); IPR008211 (PFAM); PTHR10574:SF228 (PANTHER); PTHR10574 (PANTHER); IPR008211 (PROSITE_PROFILES)
241	173453_length_256_cvg_2.0_tip_1_4	4	receptor-type tyrosine-protein phosphatase kappa	85	7.99E-39	82.60%	0.113	IPR000242 (PFAM); IPR029021 (G3DSA:3.90.190.GENE3D); PTHR19134 (PANTHER); PTHR19134:SF227 (PANTHER); IPR000242 (PROSITE_PROFILES); IPR029021 (SUPERFAMILY)
242	174007_length_256_cvg_2.0_tip_1_1	8	molting fluid carboxypeptidase a precursor	85	4.16E-29	74.20%	0.11	IPR000834 (PFAM); G3DSA:3.40.630.10 (GENE3D); PTHR11705 (PANTHER); SSF53187 (SUPERFAMILY)
249	175291_length_257_cvg_2.0_tip_1_2	6	notch-regulated ankyrin repeat-containing protein	85	1.75E-32	74.40%	0.112	IPR002110 (SMART); IPR020683 (G3DSA:1.25.40.GENE3D); PF13857 (PFAM); PTHR24203 (PANTHER); IPR002110 (PROSITE_PROFILES); IPR020683 (PROSITE_PROFILES); IPR020683 (SUPERFAMILY)
243	174331_length_256_cvg_4.6_tip_1_3	9	protein sda1 homolog	86	4.88E-33	86.50%	0.161	IPR012977 (PFAM); IPR027312 (PTHR12730:PANTHER); PTHR12730 (PANTHER)
244	174991_length_257_cvg_3.0_tip_1_1	7	oocyte zinc finger protein 6	86	4.26E-17	59.70%	0.103	IPR015880 (SMART); PF13465 (PFAM); PF13894 (PFAM); IPR013087 (G3DSA:3.30.160.GENE3D); IPR013087 (G3DSA:3.30.160.GENE3D); PTHR24409:SF14 (PANTHER); PTHR24409 (PANTHER); IPR007087 (PROSITE_PATTERNS); IPR007087 (PROSITE_PATTERNS); IPR007087 (PROSITE_PATTERNS); IPR007087 (PROSITE_PROFILES); IPR007087 (PROSITE_PROFILES); IPR007087 (PROSITE_PROFILES); IPR007087 (PROSITE_PROFILES); SSF57667 (SUPERFAMILY); SSF57667 (SUPERFAMILY)
245	175015_length_257_cvg_2.0_tip_1_3	7	zinc finger protein	86	8.16E-13	60.30%	0.107	IPR015880 (SMART); PF12874 (PFAM); PF13465 (PFAM); IPR013087 (G3DSA:3.30.160.GENE3D); IPR013087 (G3DSA:3.30.160.GENE3D); PTHR24409:SF14 (PANTHER); PTHR24409 (PANTHER); IPR007087 (PROSITE_PATTERNS); IPR007087 (PROSITE_PATTERNS); IPR007087 (PROSITE_PROFILES); IPR007087 (PROSITE_PROFILES); SSF57667 (SUPERFAMILY); SSF57667 (SUPERFAMILY)
246	175203_length_257_cvg_2.0_tip_1_4	3	neural-cadherin- partial	86	1.86E-38	90.10%	0.103	IPR000233 (PFAM); IPR027397 (G3DSA:4.10.900.GENE3D); PTHR24027:SF251 (PANTHER); PTHR24027 (PANTHER)
247	175219_length_257_cvg_3.0_tip_1_3	7	enolase	86	1.32E-34	86.80%	0.118	IPR000941 (PRINTS); IPR020810 (PFAM); IPR029065 (G3DSA:3.20.20.GENE3D); IPR000941 (PANTHER); IPR029065 (SUPERFAMILY)
248	175233_length_257_cvg_2.0_tip_1_4	5	zinc finger protein partial	86	5.46E-18	61.70%	0.1	IPR015880 (SMART); IPR013087 (G3DSA:3.30.160.GENE3D); IPR007087 (PFAM); IPR013087 (G3DSA:3.30.160.GENE3D); PF13465 (PFAM); IPR013087 (G3DSA:3.30.160.GENE3D); PTHR24409 (PANTHER); IPR007087 (PROSITE_PATTERNS); IPR007087 (PROSITE_PATTERNS); IPR007087 (PROSITE_PROFILES); IPR007087 (PROSITE_PROFILES); IPR007087 (PROSITE_PROFILES); SSF57667 (SUPERFAMILY); SSF57667 (SUPERFAMILY)
250	175461_length_257_cvg_12.5_tip_1_0	19	adp-ribosylation factor 2	86	1.61E-13	99.70%	0.133	IPR006689 (PFAM); IPR027417 (G3DSA:3.40.50.GENE3D); PTHR11711:SF145 (PANTHER); PTHR11711 (PANTHER); TRANSMEMBRANE (PHOBIUS); NON_CYTOPLASMIC_DOMAIN (PHOBIUS); CYTOPLASMIC_DOMAIN (PHOBIUS); IPR027417 (SUPERFAMILY); TMhelix (TMHMM)
251	175837_length_258_cvg_6.0_tip_1_4	15	plexin domain-containing protein 2	86	4.77E-30	81.00%	0.118	PTHR13055 (PANTHER); PTHR13055:SF12 (PANTHER)
252	175879_length_258_cvg_2.0_tip_1_4	3	pro-epidermal growth factor	86	1.29E-28	72.30%	0.101	IPR000742 (SMART); IPR001881 (SMART); IPR001881 (PFAM); G3DSA:2.10.25.10 (GENE3D); G3DSA:2.10.25.10 (GENE3D); PF14670 (PFAM); PTHR24034 (PANTHER); IPR000152 (PROSITE_PATTERNS); IPR000152 (PROSITE_PATTERNS); IPR018097 (PROSITE_PATTERNS); IPR018097 (PROSITE_PATTERNS); IPR013032 (PROSITE_PATTERNS); SSF57196 (SUPERFAMILY); SSF57196 (SUPERFAMILY)
253	176117_length_258_cvg_2.0_tip_1_5	4	afadin isoform x3	86	3.03E-43	93.10%	0.108	IPR001478 (SMART); IPR001478 (PFAM); IPR001478 (G3DSA:2.30.42.GENE3D); PTHR10398 (PANTHER); IPR001478 (PROSITE_PROFILES); IPR001478 (SUPERFAMILY)
254	176407_length_258_cvg_3.0_tip_1_4	7	ldlr2-a protein	86	1.07E-11	65.40%	0.121	IPR002172 (PRINTS); IPR002172 (SMART); IPR002172 (G3DSA:4.10.400.GENE3D); IPR002172 (PFAM); IPR002172 (G3DSA:4.10.400.GENE3D); PTHR10529 (PANTHER); IPR023415 (PROSITE_PATTERNS); IPR002172 (PROSITE_PROFILES); IPR002172 (SUPERFAMILY)
255	176943_length_258_cvg_2.0_tip_1_3	4	apical endosomal glyco	86	4.84E-21	66.60%	0.113	IPR000998 (PFAM); PTHR23282 (PANTHER); PTHR23282:SF75 (PANTHER); IPR000998 (PROSITE_PROFILES); IPR013320 (SUPERFAMILY)
256	177043_length_259_cvg_2.9_tip_1_2	8	c-terminal tandem repeated domain in type 4 procollagen	86	2.06E-13	67.90%	0.109	IPR008160 (PFAM); PTHR24023 (PANTHER); PTHR24023:SF379 (PANTHER)
259	177133_length_259_cvg_8.0_tip_1_4	13	serpin b12	86	4.36E-12	74.10%	0.108	IPR023796 (PFAM); G3DSA:2.30.39.10 (GENE3D); PTHR11461:SF52 (PANTHER); IPR000215 (PANTHER); IPR023795 (PROSITE_PATTERNS); IPR023796 (SUPERFAMILY)
261	177457_length_259_cvg_4.0_tip_1_1	9	retinol dehydrogenase 11-like	86	4.24E-17	61.80%	0.501 Y	PTHR24320 (PANTHER); PTHR24320:SF59 (PANTHER); SignalP-noTM (SIGNALP_EUK)

262	177821_length_259_cvg_6.0_tip_1_5	16	zinc finger partial	86	9.23E-15	62.70%	0.104	IPR015880 (SMART); IPR013087 (G3DSA:3.30.160.GENE3D); PF13465 (PFAM); IPR013087 (G3DSA:3.30.160.GENE3D); PTHR24409 (PANTHER); PTHR24409:SF13 (PANTHER); IPR007087 (PROSITE_PATTERNS); IPR007087 (PROSITE_PATTERNS); IPR007087 (PROSITE_PROFILES); IPR007087 (PROSITE_PROFILES); SSF57667 (SUPERFAMILY); SSF57667 (SUPERFAMILY)
263	178049_length_259_cvg_2.0_tip_1_4	3	sialate o- partial	86	3.65E-19	67.80%	0.12	IPR005181 (PFAM); PTHR22901 (PANTHER); IPR013830 (SUPERFAMILY)
265	179285_length_260_cvg_3.0_tip_1_2	6	tyrosine-protein kinase wsck	86	5.04E-24	62.20%	0.157	IPR001245 (PFAM); G3DSA:1.10.510.10 (GENE3D); PTHR24416 (PANTHER); PTHR24416:SF95 (PANTHER); IPR011009 (SUPERFAMILY)
257	177043_length_259_cvg_2.9_tip_1_3	8	cell surface	87	3.29E-12	56.60%	0.124	no IPS match
258	177107_length_259_cvg_2.1_tip_1_0	5	heat shock protein 70	87	1.64E-23	66.70%	0.104	Coil (COILS); IPR013126 (PFAM); IPR029047 (G3DSA:2.60.34.GENE3D); PTHR19375 (PANTHER); PTHR19375:SF156 (PANTHER); IPR029047 (SUPERFAMILY)
260	177377_length_259_cvg_9.3_tip_1_3	20	hemagglutinin amebocyte aggregation factor-like	87	4.08E-16	67.60%	0.177	PF14704 (PFAM); IPR026645 (PANTHER)
264	179139_length_260_cvg_3.0_tip_1_4	8	macrophage mannose receptor 1	87	2.55E-13	58.00%	0.112	IPR001304 (PFAM); IPR016186 (G3DSA:3.10.100.GENE3D); IPR016186 (G3DSA:3.10.100.GENE3D); PTHR22803 (PANTHER); IPR018378 (PROSITE_PATTERNS); IPR001304 (PROSITE_PROFILES); IPR001304 (PROSITE_PROFILES); IPR016187 (SUPERFAMILY); IPR016187 (SUPERFAMILY)
266	179607_length_261_cvg_62.2_tip_0_4	275	angiopoietin-4 isoform x2	87	2.10E-19	70.30%	0.103	IPR002181 (SMART); IPR014716 (G3DSA:3.90.215.GENE3D); IPR002181 (PFAM); PTHR19143 (PANTHER); IPR002181 (PROSITE_PROFILES); IPR002181 (SUPERFAMILY)
267	179781_length_261_cvg_52.0_tip_1_3	107	---NA---	87			0.914 Y	IPR014044 (G3DSA:3.40.33.GENE3D); SIGNAL_PEPTIDE_C_REGION (PHOBIUS); SIGNAL_PEPTIDE_N_REGION (PHOBIUS); SIGNAL_PEPTIDE (PHOBIUS); SIGNAL_PEPTIDE_H_REGION (PHOBIUS); NON_CYTOPLASMIC_DOMAIN (PHOBIUS); SignalP-TM (SIGNALP_GRAM_POSITIVE); SignalP-noTM (SIGNALP_GRAM_NEGATIVE); SignalP-noTM (SIGNALP_EUK); TMhelix (TMHMM)
268	180769_length_262_cvg_2.0_tip_1_4	5	PREDICTED: uncharacterized protein C4orf29 homolog isoform X5	87	6.29E-34	81.10%	0.116	IPR019149 (PFAM); PTHR13617 (PANTHER)
269	180967_length_262_cvg_3.0_tip_1_2	7	aquaporin	87	1.20E-27	71.90%	0.211	IPR000425 (PRINTS); IPR000425 (PFAM); IPR023271 (G3DSA:1.20.1080.GENE3D); IPR000425 (PANTHER); TRANSMEMBRANE (PHOBIUS); CYTOPLASMIC_DOMAIN (PHOBIUS); CYTOPLASMIC_DOMAIN (PHOBIUS); TRANSMEMBRANE (PHOBIUS); NON_CYTOPLASMIC_DOMAIN (PHOBIUS); IPR023271 (SUPERFAMILY); TMhelix (TMHMM); TMhelix (TMHMM)
270	181025_length_262_cvg_2.0_tip_1_5	4	hormone-sensitive lipase	87	7.78E-33	75.60%	0.207	IPR013094 (PFAM); IPR029058 (G3DSA:3.40.50.GENE3D); PTHR23025 (PANTHER); PTHR23025:SF3 (PANTHER); IPR029058 (SUPERFAMILY)
271	181171_length_262_cvg_2.0_tip_1_5	6	zinc finger protein 271 (zinc finger protein 7) (zinc finger protein znfphex133) (epstein-barr virus-induced zinc finger protein) (znf-eb) (ct-zfp48) (zinc finger protein	87	1.86E-22	69.70%	0.1	IPR015880 (SMART); IPR013087 (G3DSA:3.30.160.GENE3D); IPR013087 (G3DSA:3.30.160.GENE3D); IPR007087 (PFAM); IPR013087 (G3DSA:3.30.160.GENE3D); PF13465 (PFAM); PTHR24409 (PANTHER); IPR007087 (PROSITE_PATTERNS); IPR007087 (PROSITE_PATTERNS); IPR007087 (PROSITE_PROFILES); IPR007087 (PROSITE_PROFILES); IPR007087 (PROSITE_PROFILES); SSF57667 (SUPERFAMILY); SSF57667 (SUPERFAMILY)
272	181231_length_262_cvg_2.0_tip_1_5	4	basement membrane-specific heparan sulfate proteoglycan core protein	87	2.02E-28	73.30%	0.1	G3DSA:2.10.25.10 (GENE3D); PTHR10574 (PANTHER); PTHR10574:SF252 (PANTHER)
274	181721_length_262_cvg_2.0_tip_1_1	6	extracellular serine threonine protein kinase fam20c isoform x1	87	9.00E-18	72.10%	0.152	PTHR12450:SF8 (PANTHER); IPR024869 (PANTHER)
275	181775_length_263_cvg_4.0_tip_1_5	10	millepora cytotoxin-1-like	87	1.92E-07	65.14%	0.102	PF14704 (PFAM); IPR026645 (PANTHER)
276	182183_length_263_cvg_2.0_tip_1_2	6	krueppel-like factor 13	87	1.70E-13	62.30%	0.102	IPR015880 (SMART); IPR013087 (G3DSA:3.30.160.GENE3D); PF13894 (PFAM); IPR013087 (G3DSA:3.30.160.GENE3D); IPR007087 (PFAM); PTHR24409:SF14 (PANTHER); PTHR24409 (PANTHER); IPR007087 (PROSITE_PATTERNS); IPR007087 (PROSITE_PATTERNS); IPR007087 (PROSITE_PROFILES); IPR007087 (PROSITE_PROFILES); SSF57667 (SUPERFAMILY)
277	182245_length_263_cvg_2.0_tip_1_2	4	zinc finger protein 254-like	87	6.90E-20	68.70%	0.104	IPR015880 (SMART); IPR013087 (G3DSA:3.30.160.GENE3D); PF13465 (PFAM); PTHR24409 (PANTHER); IPR007087 (PROSITE_PATTERNS); IPR007087 (PROSITE_PATTERNS); IPR007087 (PROSITE_PROFILES); IPR007087 (PROSITE_PROFILES); SSF57667 (SUPERFAMILY)
278	182273_length_263_cvg_2.0_tip_1_2	5	zinc c2h2 type domain-containing protein	87	1.08E-15	59.50%	0.1	IPR015880 (SMART); IPR007087 (PFAM); PF13465 (PFAM); IPR013087 (G3DSA:3.30.160.GENE3D); PTHR24409 (PANTHER); PTHR24409:SF14 (PANTHER); IPR007087 (PROSITE_PATTERNS); IPR007087 (PROSITE_PATTERNS); IPR007087 (PROSITE_PROFILES); IPR007087 (PROSITE_PROFILES); SSF57667 (SUPERFAMILY)
279	182317_length_263_cvg_2.0_tip_1_2	3	mam and ldl-receptor class a domain-containing protein 2-like	87	9.52E-24	72.70%	0.103	IPR000998 (PFAM); PTHR23282 (PANTHER); IPR000998 (PROSITE_PROFILES); IPR013320 (SUPERFAMILY); TMhelix (TMHMM)

273	181569_length_262_cvg_3.0_tip_1_0	7	transferrin	88	4.55E-19	64.30%	0.099		IPR001156 (PRINTS); G3DSA:3.40.190.10 (GENE3D); IPR001156 (PFAM); PTHR11485 (PANTHER); IPR001156 (PROSITE_PROFILES); SSF53850 (SUPERFAMILY)
280	182529_length_263_cvg_4.0_tip_1_3	8	fras1-related extracellular matrix protein 1 isoform x1	88	5.24E-12	56.50%	0.167		IPR001304 (PFAM); IPR016186 (G3DSA:3.10.100.GENE3D); IPR001304 (PROSITE_PROFILES); IPR016187 (SUPERFAMILY)
281	182805_length_264_cvg_25.4_tip_1_0	55	aldose reductase	88	6.70E-27	85.40%	0.364	Y	IPR023210 (PFAM); IPR023210 (G3DSA:3.20.20.GENE3D); PTHR11732:SF173 (PANTHER); IPR001395 (PANTHER); IPR018170 (PROSITE_PATTERNS); IPR023210 (SUPERFAMILY)
282	182807_length_264_cvg_3.9_tip_1_3	7	endo-beta- -glucanase	88	1.96E-14	80.00%	0.22		IPR012341 (G3DSA:1.50.10.GENE3D); IPR001701 (PFAM); PTHR22298 (PANTHER); SignalP-TM (SIGNALP_GRAM_POSITIVE); IPR008928 (SUPERFAMILY)
283	182895_length_264_cvg_2.0_tip_1_4	7	serine protease nudel	88	1.07E-43	89.10%	0.113		G3DSA:2.40.10.10 (GENE3D); IPR001254 (PFAM); PTHR24265 (PANTHER); IPR001254 (PROSITE_PROFILES); IPR009003 (SUPERFAMILY)
284	182909_length_264_cvg_2.0_tip_1_0	5	hemicentin-1 precursor	88	6.59E-29	73.80%	0.142		PR01705 (PRINTS); IPR000884 (SMART); G3DSA:2.20.100.10 (GENE3D); IPR000884 (PFAM); PTHR19897 (PANTHER); IPR000884 (PROSITE_PROFILES); IPR000884 (PROSITE_PROFILES); IPR000884 (SUPERFAMILY); IPR000884 (SUPERFAMILY)
285	183009_length_264_cvg_2.7_tip_1_5	6	wilms tumor partial	88	1.63E-26	69.80%	0.125		IPR015880 (SMART); IPR013087 (G3DSA:3.30.160.GENE3D); IPR007087 (PFAM); IPR013087 (G3DSA:3.30.160.GENE3D); PF13465 (PFAM); IPR013087 (G3DSA:3.30.160.GENE3D); PTHR24409 (PANTHER); IPR007087 (PROSITE_PATTERNS); IPR007087 (PROSITE_PATTERNS); IPR007087 (PROSITE_PROFILES); IPR007087 (PROSITE_PROFILES); SSF57667 (SUPERFAMILY); SSF57667 (SUPERFAMILY)
286	183023_length_264_cvg_2.0_tip_1_0	7	trehalase-like isoform x1	88	6.01E-26	70.30%	0.116		IPR001661 (PFAM); IPR001661 (PANTHER); PTHR23403:SF1 (PANTHER); IPR018232 (PROSITE_PATTERNS); IPR008928 (SUPERFAMILY)
287	183287_length_264_cvg_2.8_tip_1_4	6	zinc finger protein 431 isoform x2	88	6.86E-18	68.50%	0.146		IPR015880 (SMART); IPR013087 (G3DSA:3.30.160.GENE3D); PF13465 (PFAM); PTHR24409:SF13 (PANTHER); PTHR24409 (PANTHER); IPR007087 (PROSITE_PATTERNS); IPR007087 (PROSITE_PATTERNS); IPR007087 (PROSITE_PROFILES); IPR007087 (PROSITE_PROFILES); IPR007087 (PROSITE_PROFILES); SSF57667 (SUPERFAMILY); SSF57667 (SUPERFAMILY)
288	183293_length_264_cvg_4.0_tip_1_5	12	thioredoxin domain-containing protein 11	88	4.06E-22	63.10%	0.111		IPR012336 (G3DSA:3.40.30.GENE3D); IPR013766 (PFAM); PTHR18929 (PANTHER); PTHR18929:SF61 (PANTHER); IPR012336 (SUPERFAMILY); TMhelix (TMHMM)
289	183395_length_264_cvg_9.8_tip_1_0	21	alpha-esterase 47 isoform l	88	2.91E-11	72.50%	0.496	Y	IPR002018 (PFAM); IPR029058 (G3DSA:3.40.50.GENE3D); PTHR11559 (PANTHER); SIGNAL_PEPTIDE_H_REGION (PHOBIUS); SIGNAL_PEPTIDE_C_REGION (PHOBIUS); NON_CYTOPLASMIC_DOMAIN (PHOBIUS); SIGNAL_PEPTIDE_N_REGION (PHOBIUS); SIGNAL_PEPTIDE (PHOBIUS); SignalP-TM (SIGNALP_GRAM_NEGATIVE); SignalP-TM (SIGNALP_GRAM_POSITIVE); IPR029058 (SUPERFAMILY); TMhelix (TMHMM)
292	184509_length_265_cvg_12.0_tip_1_2	33	melanoma-associated antigen e1- partial	88	6.88E-10	67.10%	0.428	Y	SignalP-TM (SIGNALP_GRAM_POSITIVE)
293	184667_length_265_cvg_4.0_tip_1_5	11	ribonuclease oy-like	88	2.72E-18	61.10%	0.112		IPR001568 (PFAM); IPR001568 (G3DSA:3.90.730.GENE3D); IPR001568 (PANTHER); IPR001568 (SUPERFAMILY)
295	185267_length_266_cvg_3.0_tip_1_5	4	ring finger and spry domain-containing protein 1-like	88	4.92E-35	79.80%	0.148		IPR003877 (PFAM); PTHR13363 (PANTHER); IPR001870 (PROSITE_PROFILES); IPR013320 (SUPERFAMILY)
290	184341_length_265_cvg_2.0_tip_1_0	6	hypothetical protein Phum_PHUM426340	89	4.69E-20	67.10%	0.133		IPR003410 (PFAM); PTHR24273:SF29 (PANTHER); PTHR24273 (PANTHER); IPR003410 (PROSITE_PROFILES)
291	184497_length_265_cvg_3.8_tip_1_3	15	urokinase-type plasminogen activator	89	3.23E-06	82.30%	0.148		IPR001254 (PFAM); G3DSA:2.40.10.10 (GENE3D); IPR009003 (SUPERFAMILY)
294	184747_length_265_cvg_5.0_tip_1_0	10	nuclear transport	89	2.79E-21	86.80%	0.121		IPR002075 (PFAM); G3DSA:3.10.450.50 (GENE3D); PTHR12612 (PANTHER); IPR018222 (PROSITE_PROFILES); SSF54427 (SUPERFAMILY)
296	185457_length_266_cvg_2.0_tip_1_4	4	nad h-hydrate epimerase-like	89	2.80E-38	78.90%	0.106		IPR004443 (PFAM); IPR004443 (G3DSA:3.40.50.GENE3D); PTHR13232 (PANTHER); IPR004443 (PROSITE_PROFILES); IPR004443 (SUPERFAMILY)
297	185923_length_266_cvg_6.0_tip_1_3	12	cd109 antigen-like isoform x1	89	1.08E-35	77.70%	0.108		IPR002890 (PFAM); PTHR11412:SF84 (PANTHER); PTHR11412 (PANTHER)
298	185997_length_267_cvg_3.0_tip_1_2	9	heat shock protein	89	7.68E-42	91.50%	0.112		IPR029048 (G3DSA:1.20.1270.GENE3D); IPR029047 (G3DSA:2.60.34.GENE3D); IPR013126 (PFAM); PTHR19375 (PANTHER); IPR029047 (SUPERFAMILY)
299	186277_length_267_cvg_2.0_tip_1_0	6	slit-like partial	89	4.88E-19	65.50%	0.247		IPR003591 (SMART); G3DSA:3.80.10.10 (GENE3D); IPR001611 (PFAM); IPR001611 (PROSITE_PROFILES); IPR001611 (PROSITE_PROFILES); SSF52058 (SUPERFAMILY)
300	186389_length_267_cvg_2.0_tip_1_2	5	receptor tyrosine phosphatase type r2a	89	3.37E-30	79.90%	0.134		IPR003598 (SMART); IPR003599 (SMART); IPR013783 (G3DSA:2.60.40.GENE3D); IPR013098 (PFAM); PTHR19134 (PANTHER); PTHR19134:SF199 (PANTHER); IPR007110 (PROSITE_PROFILES); SSF48726 (SUPERFAMILY)
301	186531_length_267_cvg_2.0_tip_1_3	6	transmembrane protease serine 12	89	8.34E-19	75.60%	0.148		IPR001254 (PFAM); G3DSA:2.40.10.10 (GENE3D); PTHR24264:SF9 (PANTHER); PTHR24264 (PANTHER); IPR018114 (PROSITE_PATTERNS); IPR001254 (PROSITE_PROFILES); IPR009003 (SUPERFAMILY); TMhelix (TMHMM)

302	186723_length_267_cvg_66.1_tip_1_3	225	plasma alpha-l-fucosidase-like	89	2.29E-26	77.60%	0.731 Y	IPR000933 (PFAM); IPR013781 (G3DSA:3.20.20.GENE3D); IPR000933 (PANTHER); PTHR10030:SF26 (PANTHER); SIGNAL_PEPTIDE_C_REGION (PHOBIUS); SIGNAL_PEPTIDE (PHOBIUS); SIGNAL_PEPTIDE_H_REGION (PHOBIUS); TRANSMEMBRANE (PHOBIUS); NON_CYTOPLASMIC_DOMAIN (PHOBIUS); SIGNAL_PEPTIDE_N_REGION (PHOBIUS); CYTOPLASMIC_DOMAIN (PHOBIUS); SignalP-noTM (SIGNALP_EUK); IPR017853 (SUPERFAMILY); TMhelix (TMHMM); TMhelix (TMHMM)
303	186757_length_267_cvg_2.0_tip_1_1	5	short-chain dehydrogenase	89	1.76E-22	71.50%	0.440 Y	IPR016040 (G3DSA:3.40.50.GENE3D); IPR002198 (PFAM); PTHR24322:SF308 (PANTHER); PTHR24322 (PANTHER); SSF51735 (SUPERFAMILY)
304	186821_length_267_cvg_2.0_tip_1_5	4	zinc finger protein	89	7.77E-17	59.00%	0.102	IPR015880 (SMART); PF13465 (PFAM); IPR013087 (G3DSA:3.30.160.GENE3D); IPR013087 (G3DSA:3.30.160.GENE3D); PTHR24409 (PANTHER); PTHR24409:SF14 (PANTHER); IPR007087 (PROSITE_PATTERNS); IPR007087 (PROSITE_PATTERNS); IPR007087 (PROSITE_PROFILES); IPR007087 (PROSITE_PROFILES); SSF57667 (SUPERFAMILY); SSF57667 (SUPERFAMILY)
305	187119_length_268_cvg_2.0_tip_1_1	6	spermatogenesis-associated protein 5	89	3.98E-48	92.80%	0.105	IPR003959 (PFAM); IPR027417 (G3DSA:3.40.50.GENE3D); PTHR23077 (PANTHER); PTHR23077:SF27 (PANTHER); IPR027417 (SUPERFAMILY)
306	187171_length_268_cvg_2.0_tip_1_5	5	zinc finger protein	89	2.80E-15	58.20%	0.109	IPR015880 (SMART); IPR013087 (G3DSA:3.30.160.GENE3D); IPR013087 (G3DSA:3.30.160.GENE3D); PF13465 (PFAM); PTHR24409 (PANTHER); PTHR24409:SF14 (PANTHER); IPR007087 (PROSITE_PATTERNS); IPR007087 (PROSITE_PATTERNS); IPR007087 (PROSITE_PROFILES); IPR007087 (PROSITE_PROFILES); SSF57667 (SUPERFAMILY)
308	187339_length_268_cvg_3.0_tip_1_2	7	c-terminal tandem repeated domain in type 4 procollagen	89	8.48E-10	63.20%	0.098	IPR008160 (PFAM); PTHR24023:SF379 (PANTHER); PTHR24023 (PANTHER)
310	187415_length_268_cvg_2.0_tip_1_4	6	wd repeat-containing protein 37-like	89	3.26E-47	92.80%	0.104	IPR001680 (SMART); IPR015943 (G3DSA:2.130.10.GENE3D); IPR001680 (PFAM); PTHR19855 (PANTHER); PTHR19855:SF12 (PANTHER); IPR019775 (PROSITE_PATTERNS); IPR001680 (PROSITE_PROFILES); IPR001680 (PROSITE_PROFILES); IPR017986 (PROSITE_PROFILES); IPR017986 (SUPERFAMILY)
312	187625_length_268_cvg_2.0_tip_1_4	6	---NA---	89			0.107	no IPS match
313	187661_length_268_cvg_2.0_tip_1_5	3	glucose dehydrogenase	89	3.27E-23	71.10%	0.143	G3DSA:3.50.50.60 (GENE3D); IPR000172 (PFAM); PTHR11552:SF77 (PANTHER); PTHR11552 (PANTHER); SSF51905 (SUPERFAMILY)
314	187993_length_269_cvg_3.2_tip_1_5	7	zinc finger protein 235	89	5.31E-15	61.20%	0.099	IPR015880 (SMART); PF13894 (PFAM); PF13465 (PFAM); IPR013087 (G3DSA:3.30.160.GENE3D); IPR013087 (G3DSA:3.30.160.GENE3D); PTHR24409 (PANTHER); PTHR24409:SF14 (PANTHER); IPR007087 (PROSITE_PATTERNS); IPR007087 (PROSITE_PATTERNS); IPR007087 (PROSITE_PROFILES); IPR007087 (PROSITE_PROFILES); SSF57667 (SUPERFAMILY)
316	188235_length_269_cvg_8.4_tip_1_2	23	prolow-density lipoprotein receptor-related protein 1-like	89	4.64E-08	64.00%	0.359 Y	IPR002172 (SMART); IPR002172 (PFAM); IPR002172 (G3DSA:4.10.400.GENE3D); IPR023415 (PROSITE_PATTERNS); NON_CYTOPLASMIC_DOMAIN (PHOBIUS); CYTOPLASMIC_DOMAIN (PHOBIUS); TRANSMEMBRANE (PHOBIUS); IPR002172 (PROSITE_PROFILES); IPR002172 (SUPERFAMILY)
319	188575_length_269_cvg_2.0_tip_1_5	4	dnaj homolog subfamily c member 30-like	89	4.76E-21	73.30%	0.113	IPR001623 (PRINTS); IPR001623 (SMART); IPR001623 (PFAM); IPR001623 (G3DSA:1.10.287.GENE3D); PTHR24078 (PANTHER); PTHR24078:SF171 (PANTHER); IPR018253 (PROSITE_PATTERNS); IPR001623 (PROSITE_PROFILES); IPR001623 (SUPERFAMILY)
307	187283_length_268_cvg_2.0_tip_1_0	6	er degradation-enhancing alpha-mannosidase-like 1	90	7.88E-40	84.40%	0.105	IPR001382 (PFAM); IPR001382 (G3DSA:1.50.10.GENE3D); IPR001382 (PANTHER); PTHR11742:SF25 (PANTHER); IPR001382 (SUPERFAMILY)
309	187339_length_268_cvg_3.0_tip_1_3	7	hypothetical protein, partial	90	1.75E-09	48.80%	0.119	no IPS match
311	187461_length_268_cvg_2.0_tip_1_3	6	leucine-rich repeat-containing protein 58	90	5.52E-10	60.40%	0.103	G3DSA:3.80.10.10 (GENE3D); IPR001611 (PFAM); IPR001611 (PROSITE_PROFILES); IPR001611 (PROSITE_PROFILES); IPR001611 (PROSITE_PROFILES); SSF52075 (SUPERFAMILY)
315	188197_length_269_cvg_4.0_tip_1_1	12	proprotein convertase subtilisin kexin type	90	5.34E-30	79.00%	0.106	IPR002884 (PFAM); IPR008979 (G3DSA:2.60.120.GENE3D); PTHR10795:SF71 (PANTHER); IPR015500 (PANTHER); IPR008979 (SUPERFAMILY)
317	188275_length_269_cvg_2.0_tip_1_4	7	convoluted-like protein	90	3.52E-15	66.30%	0.101	G3DSA:3.80.10.10 (GENE3D); IPR001611 (PFAM); PTHR24365 (PANTHER); PTHR24365:SF83 (PANTHER); SSF52058 (SUPERFAMILY)
318	188403_length_269_cvg_4.3_tip_1_3	9	immunoglobulin i-set domain-containing protein	90	1.14E-20	65.90%	0.113	IPR003598 (SMART); IPR013783 (G3DSA:2.60.40.GENE3D); IPR013098 (PFAM); PTHR10489 (PANTHER); IPR007110 (PROSITE_PROFILES); SSF48726 (SUPERFAMILY)
320	188879_length_269_cvg_3.0_tip_1_3	14	coagulation factor v	90	2.97E-09	64.90%	0.113	no IPS match
321	189091_length_270_cvg_3.0_tip_1_5	10	dappu_318553-like protein	90	1.55E-22	68.00%	0.127	IPR019791 (PRINTS); IPR019791 (PFAM); IPR019791 (G3DSA:1.10.640.GENE3D); PTHR11475 (PANTHER); IPR019791 (PROSITE_PROFILES); IPR010255 (SUPERFAMILY)
322	189153_length_270_cvg_4.5_tip_1_2	14	collagen alpha-1 chain	90	2.56E-10	57.80%	0.099	IPR008160 (PFAM); PTHR24023 (PANTHER); PTHR24023:SF379 (PANTHER)

323	189199_length_270_cvg_5.0_tip_1_5	13	krueppel-like factor 13	90	1.65E-45	85.70%	0.099	IPR015880 (SMART); IPR013087 (G3DSA:3.30.160.GENE3D); PF13465 (PFAM); IPR013087 (G3DSA:3.30.160.GENE3D); PTHR23223 (PANTHER); IPR007087 (PROSITE_PATTERNS); IPR007087 (PROSITE_PATTERNS); IPR007087 (PROSITE_PROFILES); IPR007087 (PROSITE_PROFILES); IPR007087 (PROSITE_PROFILES); SSF57667 (SUPERFAMILY)
324	189217_length_270_cvg_2.0_tip_1_2	6	abl interactor 2 isoform x1	90	5.75E-40	85.60%	0.119	IPR001452 (PRINTS); IPR001452 (SMART); G3DSA:2.30.30.40 (GENE3D); IPR001452 (PFAM); IPR028457 (PANTHER); IPR001452 (PROSITE_PROFILES); IPR001452 (SUPERFAMILY)
325	189585_length_270_cvg_4.0_tip_1_1	14	transforming growth factor-beta-induced protein ig-h3-like	90	5.57E-30	68.20%	0.102	IPR000782 (PFAM); IPR000782 (G3DSA:2.30.180.GENE3D); PTHR10900:SF73 (PANTHER); PTHR10900 (PANTHER); IPR000782 (PROSITE_PROFILES); IPR000782 (SUPERFAMILY)
326	189631_length_270_cvg_3.0_tip_1_5	6	cyclin-dependent kinase 2-like	90	5.04E-54	95.90%	0.201	IPR002290 (SMART); IPR000719 (PFAM); G3DSA:1.10.510.10 (GENE3D); PTHR24056 (PANTHER); PTHR24056:SF168 (PANTHER); IPR008271 (PROSITE_PATTERNS); IPR000719 (PROSITE_PROFILES); IPR011009 (SUPERFAMILY)
327	189721_length_270_cvg_4.0_tip_1_2	12	alpha-2-macroglobulin-like isoform x1	90	2.61E-14	60.20%	0.105	IPR015880 (SMART); IPR013087 (G3DSA:3.30.160.GENE3D); IPR013087 (G3DSA:3.30.160.GENE3D); PF13465 (PFAM); IPR013087 (G3DSA:3.30.160.GENE3D); IPR007087 (PFAM); PTHR24409 (PANTHER); IPR007087 (PROSITE_PATTERNS); IPR007087 (PROSITE_PATTERNS); IPR007087 (PROSITE_PROFILES); IPR007087 (PROSITE_PROFILES); IPR007087 (PROSITE_PROFILES); SSF57667 (SUPERFAMILY); SSF57667 (SUPERFAMILY)
328	189949_length_271_cvg_4.0_tip_1_2	12	titin isoform x1	90	2.23E-32	77.50%	0.106	IPR003599 (SMART); IPR013098 (PFAM); IPR013783 (G3DSA:2.60.40.GENE3D); PTHR19897:SF149 (PANTHER); PTHR19897 (PANTHER); IPR007110 (PROSITE_PROFILES); SSF48726 (SUPERFAMILY)
331	190273_length_271_cvg_2.0_tip_1_5	5	aldehyde dehydrogenase family 16 member a1-like	90	2.25E-42	93.20%	0.099	IPR016163 (G3DSA:3.40.309.GENE3D); IPR015590 (PFAM); IPR016162 (G3DSA:3.40.605.GENE3D); PTHR11699:SF98 (PANTHER); PTHR11699 (PANTHER); IPR029510 (PROSITE_PATTERNS); IPR016161 (SUPERFAMILY)
332	190347_length_271_cvg_2.0_tip_1_1	8	clipd6 protein	90	8.41E-12	58.00%	0.129	G3DSA:2.40.10.10 (GENE3D); IPR001254 (PFAM); PTHR24265:SF76 (PANTHER); PTHR24265 (PANTHER); IPR001254 (PROSITE_PROFILES); IPR009003 (SUPERFAMILY)
333	190471_length_271_cvg_2.0_tip_1_2	3	keratin-associated protein 5-9-like	90	2.23E-07	56.43%	0.322	no IPS match
334	190477_length_271_cvg_4.0_tip_1_2	9	heat shock protein 70	90	1.52E-19	67.60%	0.103	G3DSA:3.30.420.40 (GENE3D); IPR013126 (PFAM); PTHR19375 (PANTHER); SSF53067 (SUPERFAMILY)
335	190497_length_271_cvg_6.0_tip_1_2	7	striatin- partial	90	5.48E-54	97.30%	0.114	IPR001680 (SMART); IPR001680 (PFAM); IPR015943 (G3DSA:2.130.10.GENE3D); PTHR15653 (PANTHER); IPR019775 (PROSITE_PATTERNS); IPR017986 (PROSITE_PROFILES); IPR001680 (PROSITE_PROFILES); IPR017986 (SUPERFAMILY)
338	191023_length_272_cvg_2.0_tip_1_2	6	zinc finger protein 235	90	3.10E-13	57.20%	0.1	IPR015880 (SMART); PF13465 (PFAM); IPR013087 (G3DSA:3.30.160.GENE3D); IPR013087 (G3DSA:3.30.160.GENE3D); PTHR24409 (PANTHER); PTHR24409:SF14 (PANTHER); IPR007087 (PROSITE_PATTERNS); IPR007087 (PROSITE_PROFILES); IPR007087 (PROSITE_PROFILES); IPR002219 (PROSITE_PROFILES); IPR007087 (PROSITE_PROFILES); SSF57667 (SUPERFAMILY); SSF57667 (SUPERFAMILY)
341	191333_length_272_cvg_4.0_tip_1_2	9	titin- partial	90	5.46E-43	88.00%	0.098	IPR003598 (SMART); IPR003599 (SMART); IPR013098 (PFAM); IPR013783 (G3DSA:2.60.40.GENE3D); PTHR19897 (PANTHER); PTHR19897:SF149 (PANTHER); IPR007110 (PROSITE_PROFILES); SSF48726 (SUPERFAMILY)
329	189955_length_271_cvg_3.0_tip_1_3	8	glyoxal reductase	91	2.29E-27	72.30%	0.107	IPR020471 (PRINTS); IPR023210 (PFAM); IPR023210 (G3DSA:3.20.20.GENE3D); PTHR11732:SF133 (PANTHER); IPR001395 (PANTHER); IPR023210 (SUPERFAMILY)
330	190181_length_271_cvg_2.0_tip_1_3	4	aldehyde oxidase	91	6.95E-23	71.20%	0.126	IPR008274 (PFAM); IPR008274 (G3DSA:3.30.365.GENE3D); PTHR11908 (PANTHER); PTHR11908:SF65 (PANTHER); IPR008274 (SUPERFAMILY)
336	190535_length_271_cvg_9.2_tip_1_0	21	zinc finger protein 235-like	91	5.69E-22	67.20%	0.101	IPR015880 (SMART); IPR013087 (G3DSA:3.30.160.GENE3D); IPR013087 (G3DSA:3.30.160.GENE3D); IPR013087 (G3DSA:3.30.160.GENE3D); PF13465 (PFAM); IPR007087 (PFAM); PTHR24409 (PANTHER); IPR007087 (PROSITE_PATTERNS); IPR007087 (PROSITE_PATTERNS); IPR007087 (PROSITE_PROFILES); IPR007087 (PROSITE_PROFILES); IPR007087 (PROSITE_PROFILES); SSF57667 (SUPERFAMILY)
337	190807_length_272_cvg_2.0_tip_1_0	10	polypeptide n-acetylgalactosaminyltransferase 2	91	6.35E-21	60.60%	0.104	PTHR11675 (PANTHER); PTHR11675:SF24 (PANTHER)
339	191089_length_272_cvg_2.0_tip_1_3	6	protein flightless-1	91	3.09E-40	86.90%	0.165	IPR007123 (PFAM); IPR029006 (G3DSA:3.40.20.GENE3D); IPR029919 (PTHR11977:PANTHER); IPR007122 (PANTHER); SSF55753 (SUPERFAMILY)
340	191129_length_272_cvg_2.3_tip_1_3	8	heat shock 70 kda protein cognate 4	91	4.77E-21	88.70%	0.104	IPR013126 (PRINTS); IPR013126 (PFAM); G3DSA:3.30.420.40 (GENE3D); PTHR19375 (PANTHER); PTHR19375:SF155 (PANTHER); IPR018181 (PROSITE_PATTERNS); SSF53067 (SUPERFAMILY)

342	191811_length_273_cvg_19.9_tip_0_0	36	annexin partial	91	1.50E-28	76.80%	0.11	Coil (COILS); IPR001464 (PRINTS); IPR018502 (SMART); IPR018502 (G3DSA:1.10.220.GENE3D); IPR018502 (PFAM); PTHR10502 (PANTHER); IPR018252 (PROSITE_PATTERNS); SSF47874 (SUPERFAMILY)
343	191813_length_273_cvg_34.0_tip_0_0	60	annexin partial	91	6.08E-29	78.60%	0.103	IPR001464 (PRINTS); IPR018502 (SMART); IPR018502 (G3DSA:1.10.220.GENE3D); IPR018502 (PFAM); PTHR10502 (PANTHER); IPR018252 (PROSITE_PATTERNS); SSF47874 (SUPERFAMILY)
344	191951_length_273_cvg_68.5_tip_1_3	217	isoform cra_b	91	1.38E-56	100.00%	0.128	IPR019956 (PRINTS); IPR000626 (SMART); IPR000626 (PFAM); G3DSA:3.10.20.90 (GENE3D); G3DSA:3.10.20.90 (GENE3D); PTHR10666 (PANTHER); IPR019954 (PROSITE_PATTERNS); IPR000626 (PROSITE_PROFILES); IPR029071 (SUPERFAMILY)
345	192287_length_274_cvg_2.0_tip_1_4	5	hemicentin- partial	91	4.42E-34	81.40%	0.148	IPR023413 (G3DSA:2.40.155.GENE3D); IPR006605 (PFAM); PTHR19897 (PANTHER); PTHR19897:SF158 (PANTHER); IPR006605 (PROSITE_PROFILES); IPR009017 (SUPERFAMILY)
346	192597_length_274_cvg_2.0_tip_1_1	7	roundabout homolog 1-like isoform x3	91	3.43E-06	52.40%	0.102	IPR013098 (PFAM); IPR013783 (G3DSA:2.60.40.GENE3D); IPR007110 (PROSITE_PROFILES); SSF48726 (SUPERFAMILY)
349	193401_length_275_cvg_2.0_tip_1_2	7	perlucin 8	91	3.26E-06	61.00%	0.114	IPR016186 (G3DSA:3.10.100.GENE3D); IPR016187 (SUPERFAMILY)
352	193489_length_275_cvg_2.0_tip_1_2	7	protein pbmucl2- partial	91	4.38E-12	44.10%	0.156	no IPS match
354	193603_length_275_cvg_3.2_tip_1_5	11	ubiquitin family partial	91	1.74E-55	97.90%	0.168	IPR019956 (PRINTS); IPR000626 (SMART); IPR000626 (PFAM); G3DSA:3.10.20.90 (GENE3D); G3DSA:3.10.20.90 (GENE3D); PTHR10666 (PANTHER); IPR019954 (PROSITE_PATTERNS); IPR000626 (PROSITE_PROFILES); IPR029071 (SUPERFAMILY)
347	193227_length_275_cvg_2.0_tip_1_3	6	---NA---	92			0.576 Y	IPR012674 (G3DSA:2.40.128.GENE3D); SIGNAL_PEPTIDE (PHOBIUS); SIGNAL_PEPTIDE_C_REGION (PHOBIUS); SIGNAL_PEPTIDE_N_REGION (PHOBIUS); SIGNAL_PEPTIDE_H_REGION (PHOBIUS); NON_CYTOPLASMIC_DOMAIN (PHOBIUS); SignalP-TM (SIGNALP_EUK); IPR011038 (SUPERFAMILY); TMhelix (TMHMM)
348	193317_length_275_cvg_3.0_tip_1_0	8	heat shock cognate 71 kda protein	92	1.38E-21	96.00%	0.114	IPR013126 (PRINTS); G3DSA:3.30.420.40 (GENE3D); IPR013126 (PFAM); PTHR19375 (PANTHER); PTHR19375:SF155 (PANTHER); IPR018181 (PROSITE_PATTERNS); SSF53067 (SUPERFAMILY)
350	193453_length_275_cvg_2.4_tip_1_3	9	deoxyribonuclease-2-alpha isoform x3	92	7.09E-15	55.60%	0.133	IPR004947 (PFAM); IPR004947 (PANTHER)
351	193467_length_275_cvg_2.2_tip_1_1	8	phospholipase b-like lamina ancestor	92	1.61E-26	68.20%	0.172	IPR007000 (PFAM); IPR007000 (PANTHER); PTHR12370:SF3 (PANTHER)
353	193569_length_275_cvg_2.0_tip_1_3	8	hemocytin	92	1.22E-21	67.90%	0.104	G3DSA:2.10.25.10 (GENE3D)
355	193645_length_275_cvg_2.0_tip_1_4	2	zinc finger protein partial	92	3.51E-37	81.40%	0.118	IPR015880 (SMART); PF13465 (PFAM); IPR013087 (G3DSA:3.30.160.GENE3D); IPR013087 (G3DSA:3.30.160.GENE3D); IPR013087 (G3DSA:3.30.160.GENE3D); PTHR19818 (PANTHER); PTHR19818:SF68 (PANTHER); IPR007087 (PROSITE_PATTERNS); IPR007087 (PROSITE_PATTERNS); IPR007087 (PROSITE_PROFILES); IPR007087 (PROSITE_PROFILES); SSF57667 (SUPERFAMILY); SSF57667 (SUPERFAMILY)
356	193663_length_275_cvg_2.0_tip_1_0	7	zinc finger protein gfi-1b isoform x2	92	1.31E-15	61.90%	0.108	IPR015880 (SMART); IPR007087 (PFAM); PF13465 (PFAM); IPR013087 (G3DSA:3.30.160.GENE3D); IPR013087 (G3DSA:3.30.160.GENE3D); PTHR24409 (PANTHER); IPR007087 (PROSITE_PATTERNS); IPR007087 (PROSITE_PATTERNS); IPR007087 (PROSITE_PROFILES); IPR007087 (PROSITE_PROFILES); IPR007087 (PROSITE_PROFILES); SSF57667 (SUPERFAMILY)
357	193683_length_275_cvg_2.0_tip_1_1	6	protein creg1	92	8.85E-20	74.00%	0.113	IPR012349 (G3DSA:2.30.110.GENE3D); PF13883 (PFAM); PTHR13343 (PANTHER); PTHR13343:SF17 (PANTHER); IPR012349 (SUPERFAMILY)
358	193949_length_276_cvg_7.8_tip_1_5	26	krueppel-like factor 15	92	1.40E-19	87.00%	0.122	IPR015880 (SMART); PF13465 (PFAM); IPR013087 (G3DSA:3.30.160.GENE3D); IPR013087 (G3DSA:3.30.160.GENE3D); PTHR23223 (PANTHER); PTHR23223:SF146 (PANTHER); IPR007087 (PROSITE_PATTERNS); IPR007087 (PROSITE_PROFILES); SSF57667 (SUPERFAMILY)
359	194175_length_276_cvg_2.0_tip_1_0	3	fat-like cadherin-related tumor suppressor homolog	92	5.82E-28	70.70%	0.101	IPR002126 (PRINTS); IPR002126 (G3DSA:2.60.40.GENE3D); IPR002126 (PFAM); PTHR24026 (PANTHER); PTHR24026:SF39 (PANTHER); IPR020894 (PROSITE_PATTERNS); IPR002126 (PROSITE_PROFILES); IPR015919 (SUPERFAMILY); IPR015919 (SUPERFAMILY)
360	194203_length_276_cvg_2.3_tip_1_1	5	lysosomal pro-x partial	92	1.67E-37	82.30%	0.113	IPR008758 (PFAM); IPR029058 (G3DSA:3.40.50.GENE3D); PTHR11010:SF11 (PANTHER); IPR008758 (PANTHER); IPR029058 (SUPERFAMILY)
361	194363_length_276_cvg_2.0_tip_1_3	8	isoform c	92	6.49E-36	82.20%	0.121	IPR000716 (SMART); IPR000716 (G3DSA:4.10.800.GENE3D); IPR000716 (PFAM); PTHR12036 (PANTHER); IPR000716 (PROSITE_PATTERNS); IPR000716 (PROSITE_PROFILES); IPR000716 (SUPERFAMILY)
362	194735_length_277_cvg_3.0_tip_1_2	7	carboxypeptidase pm20d1	92	1.33E-29	72.90%	0.098	PTHR11014:SF66 (PANTHER); PTHR11014 (PANTHER)
370	195593_length_278_cvg_4.9_tip_1_5	13	vanin-like protein 1	92	1.63E-26	69.40%	0.137	IPR003010 (G3DSA:3.60.110.GENE3D); IPR012101 (PANTHER); IPR003010 (SUPERFAMILY)

363	195085_length_278_cvg_4.0_tip_1_4	10	neprilysin-2 isoform x3	93	2.51E-33	76.90%	0.255	IPR018497 (PRINTS); IPR018497 (PFAM); IPR024079 (G3DSA:3.40.390.GENE3D); IPR000718 (PANTHER); PTHR11733:SF113 (PANTHER); SSF55486 (SUPERFAMILY)
364	195145_length_278_cvg_6.0_tip_1_4	21	ankyrin repeat domain-containing protein 16	93	4.11E-16	63.90%	0.241	IPR002110 (SMART); IPR020683 (PFAM); IPR020683 (G3DSA:1.25.40.GENE3D); PTHR24198 (PANTHER); IPR020683 (PROSITE_PROFILES); IPR002110 (PROSITE_PROFILES); IPR020683 (SUPERFAMILY)
365	195201_length_278_cvg_3.0_tip_1_1	7	fat-like cadherin-related tumor suppressor homolog	93	6.22E-42	89.20%	0.108	IPR002126 (PRINTS); IPR002126 (SMART); IPR002126 (G3DSA:2.60.40.GENE3D); IPR002126 (PFAM); PTHR24026 (PANTHER); PTHR24026:SF39 (PANTHER); IPR020894 (PROSITE_PATTERNS); IPR002126 (PROSITE_PROFILES); IPR015919 (SUPERFAMILY)
366	195327_length_278_cvg_5.3_tip_1_1	17	low-density lipoprotein receptor-related protein 2	93	8.02E-42	86.60%	0.133	IPR000033 (SMART); IPR011042 (G3DSA:2.120.10.GENE3D); IPR000033 (PFAM); PTHR10529:SF210 (PANTHER); PTHR10529 (PANTHER); IPR000033 (PROSITE_PROFILES); IPR000033 (PROSITE_PROFILES); IPR000033 (PROSITE_PROFILES); SSF63825 (SUPERFAMILY)
367	195353_length_278_cvg_3.0_tip_1_4	8	serine threonine-protein kinase ulk3 isoform x1	93	3.48E-38	81.40%	0.111	G3DSA:1.10.510.10 (GENE3D); IPR000719 (PFAM); PTHR24348 (PANTHER); IPR000719 (PROSITE_PROFILES); IPR011009 (SUPERFAMILY)
368	195399_length_278_cvg_54.7_tip_1_1	179	---NA---	93			0.105	no IPS match
369	195575_length_278_cvg_2.0_tip_1_3	7	low-density lipoprotein receptor-related protein partial	93	9.44E-15	60.10%	0.118	IPR002172 (SMART); IPR002172 (PFAM); IPR002172 (G3DSA:4.10.400.GENE3D); PTHR10529 (PANTHER); IPR023415 (PROSITE_PATTERNS); IPR002172 (PROSITE_PROFILES); IPR002172 (SUPERFAMILY)
371	195843_length_279_cvg_12.0_tip_1_0	30	---NA---	93			0.099	no IPS match
372	196005_length_279_cvg_3.0_tip_1_0	10	matrix metalloproteinase 1 isoform 1	93	1.72E-34	77.80%	0.113	IPR018487 (SMART); IPR018487 (PFAM); IPR000585 (G3DSA:2.110.10.GENE3D); PTHR10201 (PANTHER); PTHR10201:SF131 (PANTHER); IPR018487 (PROSITE_PROFILES); IPR018487 (PROSITE_PROFILES); IPR000585 (SUPERFAMILY)
373	196093_length_279_cvg_2.0_tip_1_4	4	zinc finger protein 628	93	1.14E-29	76.40%	0.109	IPR015880 (SMART); IPR013087 (G3DSA:3.30.160.GENE3D); PF13465 (PFAM); IPR013087 (G3DSA:3.30.160.GENE3D); IPR013087 (G3DSA:3.30.160.GENE3D); PTHR24409 (PANTHER); IPR007087 (PROSITE_PATTERNS); IPR007087 (PROSITE_PATTERNS); IPR007087 (PROSITE_PROFILES); IPR007087 (PROSITE_PROFILES); IPR007087 (PROSITE_PROFILES); SSF57667 (SUPERFAMILY); SSF57667 (SUPERFAMILY)
374	196169_length_279_cvg_3.0_tip_1_0	11	metabotropic gaba-b receptor subtype isoform b	93	4.15E-39	86.10%	0.16	IPR002456 (PRINTS); G3DSA:3.40.50.2300 (GENE3D); IPR001828 (PFAM); PTHR10519:SF3 (PANTHER); IPR002455 (PANTHER); IPR028082 (SUPERFAMILY)
375	196549_length_280_cvg_2.0_tip_1_2	5	cysteine peptidase c	93	2.39E-26	79.00%	0.119	IPR000668 (PFAM); G3DSA:3.90.70.10 (GENE3D); PTHR12411:SF286 (PANTHER); IPR013128 (PANTHER); SSF54001 (SUPERFAMILY)
376	196601_length_280_cvg_3.0_tip_1_4	9	peroxisome biogenesis factor 1-like	93	2.23E-52	93.30%	0.24	IPR027417 (G3DSA:3.40.50.GENE3D); IPR003959 (PFAM); IPR025653 (PTHR23077:PANTHER); PTHR23077 (PANTHER); IPR027417 (SUPERFAMILY)
377	196671_length_280_cvg_2.0_tip_1_5	5	glypican- partial	93	1.13E-08	80.57%	0.125	IPR001863 (PFAM)
378	196755_length_280_cvg_93.4_tip_0_2	194	muc19_human ame: full=mucin-19 short=muc-19 flags: partial	93	9.48E-07	49.00%	0.119	no IPS match
380	197279_length_281_cvg_12.6_tip_1_2	15	bola-like protein 3	93	5.66E-23	76.40%	0.107	G3DSA:3.30.70.1500 (GENE3D); IPR002634 (PFAM); PTHR12735 (PANTHER); PTHR12735:SF4 (PANTHER); IPR002634 (SUPERFAMILY)
379	196973_length_281_cvg_2.0_tip_1_0	6	elastase	94	2.13E-20	64.20%	0.097	IPR023612 (PRINTS); IPR013856 (PFAM); IPR013856 (G3DSA:3.10.170.GENE3D); SSF55486 (SUPERFAMILY)
381	197575_length_282_cvg_2.0_tip_1_1	7	st14a protein	94	1.74E-09	55.90%	0.141	IPR001254 (PFAM); G3DSA:2.40.10.10 (GENE3D); IPR009003 (SUPERFAMILY)
382	197833_length_282_cvg_3.0_tip_1_4	8	serine threonine-protein kinase kin4	94	1.16E-41	81.20%	0.104	IPR000719 (PFAM); G3DSA:1.10.510.10 (GENE3D); PTHR24359 (PANTHER); PTHR24359:SF18 (PANTHER); IPR008271 (PROSITE_PATTERNS); IPR000719 (PROSITE_PROFILES); IPR011009 (SUPERFAMILY)
383	197843_length_282_cvg_2.0_tip_1_0	8	zinc finger protein 492-like	94	7.27E-28	76.00%	0.13	IPR015880 (SMART); IPR013087 (G3DSA:3.30.160.GENE3D); IPR013087 (G3DSA:3.30.160.GENE3D); IPR013087 (G3DSA:3.30.160.GENE3D); PF13465 (PFAM); PTHR24409 (PANTHER); PTHR24409:SF13 (PANTHER); IPR007087 (PROSITE_PATTERNS); IPR007087 (PROSITE_PATTERNS); IPR007087 (PROSITE_PROFILES); IPR007087 (PROSITE_PROFILES); SSF57667 (SUPERFAMILY)
384	198269_length_283_cvg_4.0_tip_1_1	9	leucine-rich repeat-containing protein 58-like	94	1.58E-31	79.70%	0.108	SM00364 (SMART); IPR003591 (SMART); IPR025875 (PFAM); G3DSA:3.80.10.10 (GENE3D); PTHR23155 (PANTHER); IPR001611 (PROSITE_PROFILES); IPR001611 (PROSITE_PROFILES); IPR001611 (PROSITE_PROFILES); IPR001611 (PROSITE_PROFILES); SSF52075 (SUPERFAMILY)
385	198529_length_283_cvg_18.2_tip_0_5	41	golgin subfamily a member 6-like protein 22	94	8.21E-09	52.20%	0.101	no IPS match

386	198611_length_283_cvg_3.0_tip_1_5	8	u5 small nuclear ribonucleoprotein 40 kda	94	9.69E-47	85.40%	0.108		IPR001680 (SMART); IPR015943 (G3DSA:2.130.10.GENE3D); IPR001680 (PFAM); PTHR22847 (PANTHER); IPR019775 (PROSITE_PATTERNS); IPR019775 (PROSITE_PATTERNS); IPR001680 (PROSITE_PROFILES); IPR001680 (PROSITE_PROFILES); IPR017986 (PROSITE_PROFILES); IPR017986 (SUPERFAMILY)
387	198777_length_284_cvg_3.0_tip_1_5	7	phospholipase b-like 2	94	6.80E-30	73.90%	0.101		IPR007000 (PFAM); IPR007000 (PANTHER)
389	199253_length_284_cvg_3.0_tip_1_5	9	c-type lectin domain family 4 member m-like	94	3.26E-19	60.00%	0.146		IPR001304 (SMART); IPR001304 (PFAM); IPR016186 (G3DSA:3.10.100.GENE3D); PTHR22801 (PANTHER); IPR001304 (PROSITE_PROFILES); IPR016187 (SUPERFAMILY)
390	199263_length_284_cvg_3.0_tip_1_5	10	krueppel-like factor partial	94	1.50E-37	86.80%	0.195		IPR015880 (SMART); IPR013087 (G3DSA:3.30.160.GENE3D); IPR013087 (G3DSA:3.30.160.GENE3D); PF13465 (PFAM); PTHR23223 (PANTHER); IPR007087 (PROSITE_PATTERNS); IPR007087 (PROSITE_PATTERNS); IPR007087 (PROSITE_PROFILES); IPR007087 (PROSITE_PROFILES); SSF57667 (SUPERFAMILY)
388	199067_length_284_cvg_2.0_tip_1_1	7	carboxypeptidase e	95	6.68E-28	65.90%	0.114		PF13620 (PFAM); IPR014766 (G3DSA:2.60.40.GENE3D); PTHR11532:SF41 (PANTHER); PTHR11532 (PANTHER); IPR008969 (SUPERFAMILY)
391	199589_length_285_cvg_2.0_tip_1_0	6	ankyrin repeat	95	1.04E-18	59.50%	0.127		IPR002110 (PRINTS); IPR002110 (SMART); IPR020683 (G3DSA:1.25.40.GENE3D); IPR020683 (PFAM); PTHR24188 (PANTHER); IPR020683 (PROSITE_PROFILES); IPR002110 (PROSITE_PROFILES); IPR002110 (PROSITE_PROFILES); IPR002110 (PROSITE_PROFILES); IPR020683 (SUPERFAMILY)
392	199851_length_285_cvg_4.0_tip_1_2	10	xanthine dehydrogenase-like	95	5.34E-31	79.00%	0.105		IPR000674 (PFAM); IPR000674 (G3DSA:3.90.1170.GENE3D); IPR008274 (G3DSA:3.30.365.GENE3D); PTHR11908:SF32 (PANTHER); PTHR11908 (PANTHER); IPR000674 (SUPERFAMILY)
395	200073_length_286_cvg_4.8_tip_1_5	13	basement membrane-specific heparan sulfate proteoglycan core protein	95	4.82E-24	66.70%	0.104		IPR000034 (PFAM); IPR000034 (PROSITE_PROFILES)
397	200207_length_286_cvg_3.4_tip_1_5	4	zinc finger protein 850-like	95	2.51E-15	63.90%	0.102		IPR015880 (SMART); IPR013087 (G3DSA:3.30.160.GENE3D); PF13465 (PFAM); IPR013087 (G3DSA:3.30.160.GENE3D); PTHR24409 (PANTHER); PTHR24409:SF14 (PANTHER); IPR007087 (PROSITE_PATTERNS); IPR007087 (PROSITE_PATTERNS); IPR007087 (PROSITE_PROFILES); IPR007087 (PROSITE_PROFILES); SSF57667 (SUPERFAMILY)
393	199867_length_286_cvg_2.0_tip_1_0	4	acid sphingomyelinase-like phosphodiesterase 3b	96	2.26E-20	56.20%	0.241		IPR029052 (G3DSA:3.60.21.GENE3D); PTHR10340 (PANTHER); IPR029052 (SUPERFAMILY)
394	199935_length_286_cvg_2.0_tip_1_3	4	von willebrand factor type egf and pentraxin domain-containing protein 1 isoform x2	96	2.59E-30	76.80%	0.102		IPR000436 (SMART); G3DSA:2.10.70.10 (GENE3D); IPR000436 (PFAM); PTHR19325 (PANTHER); IPR000436 (PROSITE_PROFILES); IPR000436 (PROSITE_PROFILES); IPR000436 (SUPERFAMILY); IPR000436 (SUPERFAMILY)
396	200095_length_286_cvg_2.0_tip_1_3	5	macrophage mannose receptor 1-like	96	6.01E-07	48.14%	0.104		IPR016186 (G3DSA:3.10.100.GENE3D); IPR016186 (G3DSA:3.10.100.GENE3D); PTHR22803:SF16 (PANTHER); PTHR22803 (PANTHER); IPR001304 (PROSITE_PROFILES); IPR016187 (SUPERFAMILY)
398	200413_length_287_cvg_6.0_tip_1_3	15	dnaj homolog subfamily c member 21-like	96	7.17E-36	87.30%	0.129		IPR001623 (PRINTS); IPR001623 (SMART); IPR001623 (G3DSA:1.10.287.GENE3D); IPR001623 (PFAM); PTHR24078 (PANTHER); PTHR24078:SF150 (PANTHER); IPR018253 (PROSITE_PATTERNS); IPR001623 (PROSITE_PROFILES); IPR001623 (SUPERFAMILY)
399	200589_length_287_cvg_2.0_tip_1_0	5	homeotic protein spalt-major-like	96	6.32E-59	97.00%	0.108		IPR015880 (SMART); IPR013087 (G3DSA:3.30.160.GENE3D); IPR013087 (G3DSA:3.30.160.GENE3D); PF13465 (PFAM); PTHR23233 (PANTHER); PTHR23233:SF49 (PANTHER); IPR007087 (PROSITE_PATTERNS); IPR007087 (PROSITE_PATTERNS); IPR007087 (PROSITE_PROFILES); IPR007087 (PROSITE_PROFILES); SSF57667 (SUPERFAMILY)
400	200803_length_287_cvg_3.0_tip_1_4	9	venom serine carboxypeptidase-like	96	4.64E-15	61.70%	0.195		IPR001563 (PFAM); IPR029058 (G3DSA:3.40.50.GENE3D); IPR001563 (PANTHER); IPR029058 (SUPERFAMILY)
401	201041_length_288_cvg_2.9_tip_1_2	11	zinc c2h2 type domain-containing protein	96	8.75E-15	58.30%	0.103		IPR015880 (SMART); IPR013087 (G3DSA:3.30.160.GENE3D); IPR013087 (G3DSA:3.30.160.GENE3D); PF13465 (PFAM); PTHR24409 (PANTHER); PTHR24409:SF14 (PANTHER); IPR007087 (PROSITE_PATTERNS); IPR007087 (PROSITE_PATTERNS); IPR007087 (PROSITE_PATTERNS); IPR007087 (PROSITE_PROFILES); IPR007087 (PROSITE_PROFILES); IPR007087 (PROSITE_PROFILES); SSF57667 (SUPERFAMILY); SSF57667 (SUPERFAMILY)
402	201145_length_288_cvg_2.0_tip_1_4	6	serine threonine-protein kinase osr1 isoform x1	96	2.02E-53	94.50%	0.485	Y	G3DSA:3.30.200.20 (GENE3D); IPR000719 (PFAM); G3DSA:1.10.510.10 (GENE3D); PTHR24361 (PANTHER); PTHR24361:SF201 (PANTHER); IPR000719 (PROSITE_PROFILES); SignalP-noTM (SIGNALP_EUK); IPR011009 (SUPERFAMILY)
403	201345_length_288_cvg_2.0_tip_1_4	4	transport and golgi organization protein 1	96	1.83E-17	65.00%	0.413	Y	G3DSA:2.30.30.40 (GENE3D); IPR001452 (SUPERFAMILY)

404	201455_length_289_cvg_2.0_tip_1_1	3	basement membrane-specific heparan sulfate proteoglycan core protein	96	4.75E-11	59.30%	0.105	IPR002172 (SMART); IPR002172 (G3DSA:4.10.400.GENE3D); IPR002172 (PFAM); PTHR10529:SF3 (PANTHER); PTHR10529 (PANTHER); IPR023415 (PROSITE_PATTERNS); IPR002172 (PROSITE_PROFILES); IPR002172 (SUPERFAMILY)
405	201465_length_289_cvg_2.0_tip_1_4	5	fat-like cadherin-related tumor suppressor-like protein	96	7.97E-38	87.80%	0.1	IPR002126 (PRINTS); IPR002126 (SMART); IPR002126 (G3DSA:2.60.40.GENE3D); IPR002126 (PFAM); PTHR24026:SF39 (PANTHER); PTHR24026 (PANTHER); IPR020894 (PROSITE_PATTERNS); IPR002126 (PROSITE_PROFILES); IPR015919 (SUPERFAMILY)
406	201801_length_289_cvg_30.0_tip_1_4	81	plasma alpha-l-fucosidase-like	96	3.21E-26	79.50%	0.606 Y	IPR013781 (G3DSA:3.20.20.GENE3D); IPR000933 (PFAM); IPR000933 (PANTHER); PTHR10030:SF26 (PANTHER); TRANSMEMBRANE (PHOBIUS); CYTOPLASMIC_DOMAIN (PHOBIUS); SIGNAL_PEPTIDE_H_REGION (PHOBIUS); SIGNAL_PEPTIDE (PHOBIUS); SIGNAL_PEPTIDE_C_REGION (PHOBIUS); SIGNAL_PEPTIDE_N_REGION (PHOBIUS); NON_CYTOPLASMIC_DOMAIN (PHOBIUS); SignalP-noTM (SIGNALP_EUK); IPR017853 (SUPERFAMILY); TMhelix (TMHMM); TMhelix (TMHMM)
408	202485_length_290_cvg_3.7_tip_1_5	10	ankyrin repeat	96	2.77E-19	60.50%	0.127	IPR002110 (PRINTS); IPR002110 (SMART); IPR020683 (G3DSA:1.25.40.GENE3D); IPR020683 (PFAM); PTHR24198 (PANTHER); IPR002110 (PROSITE_PROFILES); IPR002110 (PROSITE_PROFILES); IPR020683 (PROSITE_PROFILES); IPR002110 (PROSITE_PROFILES); IPR020683 (SUPERFAMILY)
407	201849_length_289_cvg_7.8_tip_1_3	37	leucine-rich repeats and immunoglobulin-like domains protein 3 isoform x2	97	3.47E-10	54.00%	0.112	no IPS match
409	202487_length_290_cvg_3.9_tip_1_1	14	glucose dehydrogenase	97	7.00E-23	74.80%	0.1	G3DSA:3.50.50.60 (GENE3D); IPR000172 (PFAM); PTHR11552 (PANTHER); PTHR11552:SF77 (PANTHER); IPR000172 (PROSITE_PATTERNS); SSF51905 (SUPERFAMILY)
410	202663_length_291_cvg_2.0_tip_1_4	5	zinc finger protein	97	1.59E-13	52.90%	0.101	IPR015880 (SMART); PF13894 (PFAM); IPR013087 (G3DSA:3.30.160.GENE3D); PTHR24375:SF106 (PANTHER); PTHR24375 (PANTHER); IPR007087 (PROSITE_PATTERNS); IPR007087 (PROSITE_PATTERNS); IPR007087 (PROSITE_PROFILES); IPR007087 (PROSITE_PROFILES); SSF57667 (SUPERFAMILY); SSF57667 (SUPERFAMILY)
411	202865_length_291_cvg_5.5_tip_1_3	21	achain structural determinants for improved thermal stability of designed ankyrin repeat proteins with a redesigned c- capping	97	2.86E-14	63.10%	0.141	IPR002110 (SMART); IPR020683 (PFAM); IPR020683 (G3DSA:1.25.40.GENE3D); PTHR24188 (PANTHER); IPR002110 (PROSITE_PROFILES); IPR020683 (PROSITE_PROFILES); IPR002110 (PROSITE_PROFILES); IPR020683 (SUPERFAMILY)
412	202903_length_291_cvg_4.0_tip_1_0	11	low-density lipoprotein receptor-related protein 2	97	7.71E-34	77.20%	0.107	IPR002172 (PRINTS); IPR002172 (SMART); IPR002172 (G3DSA:4.10.400.GENE3D); G3DSA:4.10.1220.10 (GENE3D); IPR002172 (PFAM); IPR002172 (G3DSA:4.10.400.GENE3D); PTHR10529 (PANTHER); IPR002172 (PROSITE_PROFILES); IPR002172 (PROSITE_PROFILES); IPR002172 (SUPERFAMILY); IPR002172 (SUPERFAMILY)
413	203111_length_292_cvg_2.0_tip_1_2	6	aael012493- partial	97	2.84E-36	80.20%	0.114	IPR006652 (SMART); IPR015915 (G3DSA:2.120.10.GENE3D); IPR006652 (PFAM); PTHR10574 (PANTHER); PTHR10574:SF3 (PANTHER); SSF117281 (SUPERFAMILY)
416	203233_length_292_cvg_4.0_tip_1_1	10	adp-ribosylation factor-like protein 4c	97	1.95E-54	92.20%	0.156	IPR006689 (PRINTS); IPR006689 (PFAM); IPR027417 (G3DSA:3.40.50.GENE3D); IPR005225 (TIGRFAM); PTHR11711:SF121 (PANTHER); PTHR11711 (PANTHER); IPR024156 (PROSITE_PROFILES); IPR027417 (SUPERFAMILY)
418	203411_length_292_cvg_2.5_tip_1_4	10	low affinity immunoglobulin epsilon fc receptor	97	7.88E-15	55.30%	0.183	IPR001304 (SMART); IPR016186 (G3DSA:3.10.100.GENE3D); IPR001304 (PFAM); PTHR22802 (PANTHER); IPR001304 (PROSITE_PROFILES); IPR016187 (SUPERFAMILY)
414	203131_length_292_cvg_4.7_tip_1_0	11	alcohol dehydrogenase	98	5.41E-13	69.90%	0.13	IPR023210 (G3DSA:3.20.20.GENE3D); IPR001395 (PANTHER); PTHR11732:SF144 (PANTHER); IPR018170 (PROSITE_PATTERNS); IPR023210 (SUPERFAMILY)
415	203181_length_292_cvg_3.9_tip_1_0	9	coagulation factor x- partial	98	6.74E-14	59.10%	0.103	IPR001254 (PFAM); G3DSA:2.40.10.10 (GENE3D); G3DSA:2.40.10.10 (GENE3D); PTHR24265 (PANTHER); IPR018114 (PROSITE_PATTERNS); IPR001254 (PROSITE_PROFILES); IPR009003 (SUPERFAMILY)
417	203409_length_292_cvg_3.0_tip_1_0	9	---NA---	98			0.103	Coil (COILS); IPR016186 (G3DSA:3.10.100.GENE3D); IPR016187 (SUPERFAMILY)
419	203787_length_293_cvg_3.0_tip_1_1	9	zinc finger and scan domain-containing protein 2-like isoform x1	98	1.87E-49	86.40%	0.103	IPR015880 (SMART); IPR013087 (G3DSA:3.30.160.GENE3D); IPR013087 (G3DSA:3.30.160.GENE3D); IPR013087 (G3DSA:3.30.160.GENE3D); PF13465 (PFAM); PTHR24409 (PANTHER); IPR007087 (PROSITE_PATTERNS); IPR007087 (PROSITE_PATTERNS); IPR007087 (PROSITE_PROFILES); IPR007087 (PROSITE_PROFILES); IPR007087 (PROSITE_PROFILES); SSF57667 (SUPERFAMILY); SSF57667 (SUPERFAMILY)
420	204119_length_294_cvg_2.0_tip_1_2	5	cbl-interacting protein kinase 16-like isoform x1	98	3.43E-45	86.10%	0.122	IPR000719 (PFAM); G3DSA:1.10.510.10 (GENE3D); PTHR24342 (PANTHER); IPR000719 (PROSITE_PROFILES); IPR011009 (SUPERFAMILY)

421	204223_length_294_cvg_2.0_tip_1_3	5	sucrase- intestinal-like	98	1.86E-18	60.30%	0.1		PTHR22762:SF69 (PANTHER); PTHR22762 (PANTHER); IPR011013 (SUPERFAMILY)
422	204269_length_294_cvg_2.0_tip_1_0	7	receptor-type tyrosine-protein phosphatase kappa	98	1.06E-45	84.80%	0.117		IPR000242 (PFAM); IPR029021 (G3DSA:3.90.190.GENE3D); PTHR19134:SF227 (PANTHER); PTHR19134 (PANTHER); IPR000242 (PROSITE_PROFILES); IPR029021 (SUPERFAMILY)
423	204527_length_294_cvg_2.0_tip_1_5	7	low-density lipoprotein	98	5.33E-28	70.40%	0.103		IPR000742 (SMART); IPR001881 (SMART); IPR001881 (PFAM); G3DSA:2.10.25.10 (GENE3D); G3DSA:2.10.25.10 (GENE3D); PTHR10529:SF204 (PANTHER); PTHR10529 (PANTHER); IPR018097 (PROSITE_PATTERNS); IPR018097 (PROSITE_PATTERNS); IPR000152 (PROSITE_PATTERNS); IPR000152 (PROSITE_PATTERNS); SSF57196 (SUPERFAMILY); SSF57196 (SUPERFAMILY)
425	205001_length_296_cvg_3.9_tip_1_2	17	heat shock cognate 71 kda	98	9.06E-50	98.00%	0.186		IPR013126 (PRINTS); G3DSA:3.30.30.30 (GENE3D); IPR013126 (PFAM); G3DSA:3.30.420.40 (GENE3D); PTHR19375 (PANTHER); PTHR19375:SF142 (PANTHER); IPR018181 (PROSITE_PATTERNS); SSF53067 (SUPERFAMILY)
427	205261_length_296_cvg_4.0_tip_1_5	11	tyrosine-protein phosphatase lar isoform x1	98	1.56E-35	80.00%	0.102		PR00014 (PRINTS); IPR003961 (SMART); IPR013783 (G3DSA:2.60.40.GENE3D); IPR003961 (PFAM); PTHR19134:SF204 (PANTHER); PTHR19134 (PANTHER); IPR003961 (PROSITE_PROFILES); IPR003961 (SUPERFAMILY)
424	204979_length_296_cvg_8.0_tip_1_4	21	pacifastin-related peptide precursor	99	5.92E-10	58.22%	0.626	Y	IPR008037 (PFAM); SIGNAL_PEPTIDE_H_REGION (PHOBIUS); SIGNAL_PEPTIDE_C_REGION (PHOBIUS); SIGNAL_PEPTIDE_N_REGION (PHOBIUS); SIGNAL_PEPTIDE (PHOBIUS); NON_CYTOPLASMIC_DOMAIN (PHOBIUS); IPR020862 (PROSITE_PROFILES); IPR020862 (PROSITE_PROFILES); SignalP-noTM (SIGNALP_EUK); IPR008037 (SUPERFAMILY); IPR008037 (SUPERFAMILY)
426	205107_length_296_cvg_2.8_tip_1_0	9	gamma-glutamyltranspeptidase 1	99	7.43E-35	75.80%	0.098		IPR000101 (PRINTS); IPR000101 (PFAM); IPR000101 (PANTHER); PTHR11686:SF15 (PANTHER); IPR029055 (SUPERFAMILY)
428	205423_length_296_cvg_2.8_tip_1_4	11	peripheral plasma membrane protein partial	99	4.19E-39	81.30%	0.598	Y	G3DSA:1.10.510.10 (GENE3D); IPR000719 (PFAM); PTHR24347:SF136 (PANTHER); IPR020636 (PANTHER); SIGNAL_PEPTIDE (PHOBIUS); NON_CYTOPLASMIC_DOMAIN (PHOBIUS); SIGNAL_PEPTIDE_N_REGION (PHOBIUS); SIGNAL_PEPTIDE_C_REGION (PHOBIUS); SIGNAL_PEPTIDE_H_REGION (PHOBIUS); IPR000719 (PROSITE_PROFILES); IPR011009 (SUPERFAMILY)
429	205491_length_297_cvg_3.0_tip_1_3	8	macrophage mannose receptor 1-like	99	9.94E-11	55.30%	0.108		IPR001304 (PFAM); IPR016186 (G3DSA:3.10.100.GENE3D); IPR016186 (G3DSA:3.10.100.GENE3D); PTHR22803 (PANTHER); PTHR22803:SF16 (PANTHER); IPR018378 (PROSITE_PATTERNS); IPR016187 (SUPERFAMILY)
430	206035_length_298_cvg_5.0_tip_1_2	16	peptidyl-prolyl cis-trans isomerase d	99	4.09E-21	67.90%	0.121		Coil (COILS); IPR019734 (SMART); IPR011990 (G3DSA:1.25.40.GENE3D); PF13414 (PFAM); IPR023566 (PANTHER); IPR019734 (PROSITE_PROFILES); IPR019734 (PROSITE_PROFILES); IPR013026 (PROSITE_PROFILES); SSF48452 (SUPERFAMILY)
431	206209_length_298_cvg_3.8_tip_1_2	10	peptidyl-prolyl cis-trans isomerase nima-interacting 1-like	99	4.75E-39	91.90%	0.101		IPR000297 (PFAM); G3DSA:3.10.50.40 (GENE3D); PTHR10657 (PANTHER); IPR023058 (PROSITE_PATTERNS); IPR000297 (PROSITE_PROFILES); SSF54534 (SUPERFAMILY)
432	206401_length_298_cvg_3.0_tip_1_5	12	zinc finger protein 525-like	99	8.32E-14	57.60%	0.121		IPR015880 (SMART); PF13894 (PFAM); PF13465 (PFAM); IPR013087 (G3DSA:3.30.160.GENE3D); PTHR24409 (PANTHER); PTHR24409:SF14 (PANTHER); IPR007087 (PROSITE_PATTERNS); IPR007087 (PROSITE_PATTERNS); IPR007087 (PROSITE_PROFILES); IPR007087 (PROSITE_PROFILES); SSF57667 (SUPERFAMILY); SSF57667 (SUPERFAMILY)
433	206523_length_299_cvg_2.0_tip_1_5	6	ring finger and spry domain-containing protein 1-like	99	1.00E-38	80.50%	0.098		PTHR13363 (PANTHER)
434	206945_length_299_cvg_2.0_tip_1_5	5	low-density lipoprotein	99	3.95E-27	65.70%	0.133		IPR002172 (PRINTS); IPR002172 (SMART); IPR002172 (G3DSA:4.10.400.GENE3D); IPR002172 (PFAM); PTHR10529 (PANTHER); IPR023415 (PROSITE_PATTERNS); IPR023415 (PROSITE_PATTERNS); IPR002172 (PROSITE_PROFILES); IPR002172 (PROSITE_PROFILES); IPR002172 (SUPERFAMILY); IPR002172 (SUPERFAMILY)
435	207021_length_300_cvg_3.9_tip_1_2	12	zinc finger protein 629 isoform x1	100	9.63E-25	78.20%	0.102		IPR015880 (SMART); PF13465 (PFAM); IPR013087 (G3DSA:3.30.160.GENE3D); IPR013087 (G3DSA:3.30.160.GENE3D); PTHR23223 (PANTHER); PTHR23223:SF130 (PANTHER); IPR007087 (PROSITE_PATTERNS); IPR007087 (PROSITE_PATTERNS); IPR007087 (PROSITE_PROFILES); IPR007087 (PROSITE_PROFILES); SSF57667 (SUPERFAMILY)
436	207225_length_300_cvg_3.0_tip_1_3	10	melanoma-associated antigen e1- partial	100	2.66E-15	65.60%	0.142		SignalP-TM (SIGNALP_GRAM_POSITIVE)
437	207615_length_301_cvg_4.0_tip_1_2	13	xanthine dehydrogenase isoform x1	100	2.17E-26	74.30%	0.099		IPR000674 (PFAM); IPR000674 (G3DSA:3.90.1170.GENE3D); PTHR11908 (PANTHER); PTHR11908:SF32 (PANTHER); IPR000674 (SUPERFAMILY)
438	208039_length_302_cvg_5.0_tip_1_5	18	collagen alpha-5 chain-like	100	2.36E-06	69.00%	0.131		CYTOPLASMIC_DOMAIN (PHOBIUS); NON_CYTOPLASMIC_DOMAIN (PHOBIUS); TRANSMEMBRANE (PHOBIUS); TMhelix (TMHMM)
439	208073_length_302_cvg_2.0_tip_1_2	5	lysosomal alpha-glucosidase-like	100	3.34E-55	89.40%	0.147		IPR000322 (PFAM); PTHR22762:SF7 (PANTHER); PTHR22762 (PANTHER); IPR030459 (PROSITE_PATTERNS); IPR017853 (SUPERFAMILY)
441	208365_length_302_cvg_3.0_tip_1_5	8	fukutin-related protein	100	3.27E-09	62.40%	0.098		no IPS match

440	208273_length_302_cvg_9.0_tip_1_0	26	group iii secreted phospholipase	101	1.96E-21	60.10%	0.137	IPR001211 (PFAM); IPR016090 (G3DSA:1.20.90.GENE3D); PTHR12253 (PANTHER); IPR016090 (SUPERFAMILY)
442	208379_length_302_cvg_3.0_tip_1_0	9	serine protease	101	1.29E-18	60.60%	0.101	IPR001254 (PFAM); G3DSA:2.40.10.10 (GENE3D); PTHR24268 (PANTHER); IPR001254 (PROSITE_PROFILES); IPR009003 (SUPERFAMILY)
443	208419_length_303_cvg_38.8_tip_1_0	112	actin-depolymerizing factor	101	5.92E-37	75.10%	0.102	IPR007122 (PRINTS); IPR007122 (SMART); IPR029006 (G3DSA:3.40.20.GENE3D); IPR007123 (PFAM); IPR007122 (PANTHER); SSF55753 (SUPERFAMILY)
444	208669_length_303_cvg_3.0_tip_1_2	9	zinc finger protein	101	1.39E-59	95.30%	0.105	IPR015880 (SMART); IPR013087 (G3DSA:3.30.160.GENE3D); IPR013087 (G3DSA:3.30.160.GENE3D); IPR007087 (PFAM); PF13465 (PFAM); IPR013087 (G3DSA:3.30.160.GENE3D); PTHR23228:SF26 (PANTHER); PTHR23228 (PANTHER); IPR007087 (PROSITE_PATTERNS); IPR007087 (PROSITE_PATTERNS); IPR007087 (PROSITE_PATTERNS); IPR007087 (PROSITE_PROFILES); IPR007087 (PROSITE_PROFILES); IPR007087 (PROSITE_PROFILES); IPR007087 (PROSITE_PROFILES); SSF57667 (SUPERFAMILY); SSF57667 (SUPERFAMILY)
445	208755_length_303_cvg_2.0_tip_1_0	6	cd4-specific ankyrin repeat protein	101	1.53E-15	54.30%	0.103	IPR002110 (SMART); IPR020683 (G3DSA:1.25.40.GENE3D); IPR020683 (PFAM); PTHR24198 (PANTHER); IPR002110 (PROSITE_PROFILES); IPR002110 (PROSITE_PROFILES); IPR002110 (PROSITE_PROFILES); IPR020683 (PROSITE_PROFILES); IPR020683 (SUPERFAMILY)
446	208879_length_304_cvg_88.6_tip_0_3	1828	kazal-type serine proteinase inhibitor 4	102	8.63E-10	53.30%	0.108	IPR002350 (SMART); IPR002350 (PFAM); G3DSA:3.30.60.30 (GENE3D); IPR002350 (PROSITE_PROFILES); IPR002350 (PROSITE_PROFILES); SSF100895 (SUPERFAMILY); SSF100895 (SUPERFAMILY)
447	209385_length_305_cvg_2.0_tip_1_0	7	angiogenic factor with g patch and fha domains 1-like	102	4.46E-14	63.00%	0.118	no IPS match
448	209417_length_305_cvg_2.0_tip_1_1	3	zinc finger protein 239- partial	102	2.99E-19	60.20%	0.107	IPR015880 (SMART); IPR013087 (G3DSA:3.30.160.GENE3D); IPR013087 (G3DSA:3.30.160.GENE3D); PF13465 (PFAM); IPR013087 (G3DSA:3.30.160.GENE3D); IPR013087 (G3DSA:3.30.160.GENE3D); PTHR24409 (PANTHER); IPR007087 (PROSITE_PATTERNS); IPR007087 (PROSITE_PATTERNS); IPR007087 (PROSITE_PROFILES); IPR007087 (PROSITE_PROFILES); IPR007087 (PROSITE_PROFILES); SSF57667 (SUPERFAMILY); SSF57667 (SUPERFAMILY)
449	210005_length_306_cvg_5.0_tip_1_3	18	peptidyl-prolyl cis-trans isomerase-like	102	3.98E-39	86.50%	0.612 Y	IPR002130 (PRINTS); IPR029000 (G3DSA:2.40.100.GENE3D); IPR002130 (PFAM); IPR024936 (PANTHER); IPR020892 (PROSITE_PATTERNS); IPR002130 (PROSITE_PROFILES); SignalP-noTM (SIGNALP_EUK); IPR029000 (SUPERFAMILY)
450	210057_length_306_cvg_4.0_tip_1_2	12	carboxypeptidase b-like	102	2.53E-12	54.70%	0.1	IPR003146 (PFAM); IPR003146 (G3DSA:3.30.70.GENE3D); PTHR11705 (PANTHER); IPR009020 (SUPERFAMILY)
451	210063_length_306_cvg_7.0_tip_1_1	27	very low-density lipoprotein receptor-like	102	1.11E-09	53.90%	0.114	IPR002172 (SMART); IPR002172 (PFAM); G3DSA:4.10.1220.10 (GENE3D); IPR002172 (G3DSA:4.10.400.GENE3D); IPR002172 (PROSITE_PROFILES); IPR002172 (SUPERFAMILY)
452	210285_length_307_cvg_2.0_tip_1_1	4	carboxypeptidase d-like	102	8.26E-16	71.50%	0.143	IPR000834 (PFAM); G3DSA:3.40.630.10 (GENE3D); SSF53187 (SUPERFAMILY)
453	210557_length_307_cvg_2.0_tip_1_4	6	polypeptide n-acetylgalactosaminyltransferase 5 isoform x2	102	1.87E-45	82.40%	0.1	IPR000772 (SMART); G3DSA:2.80.10.50 (GENE3D); IPR000772 (PFAM); PTHR11675:SF39 (PANTHER); PTHR11675 (PANTHER); IPR000772 (PROSITE_PROFILES); IPR000772 (SUPERFAMILY)
455	210767_length_308_cvg_3.7_tip_1_2	13	vesicle-fusing atpase 1-like	102	8.38E-32	76.40%	0.122	IPR003338 (SMART); G3DSA:2.40.40.20 (GENE3D); IPR003338 (PFAM); PTHR23078 (PANTHER); SignalP-TM (SIGNALP_GRAM_POSITIVE); IPR009010 (SUPERFAMILY)
456	210841_length_308_cvg_3.0_tip_1_2	10	aqp_aedae ame: full=aquaporin	102	4.97E-20	70.00%	0.242	IPR000425 (PRINTS); IPR023271 (G3DSA:1.20.1080.GENE3D); IPR000425 (PFAM); IPR000425 (PANTHER); PTHR19139:SF45 (PANTHER); IPR022357 (PROSITE_PATTERNS); CYTOPLASMIC_DOMAIN (PHOBIUS); NON_CYTOPLASMIC_DOMAIN (PHOBIUS); TRANSMEMBRANE (PHOBIUS); NON_CYTOPLASMIC_DOMAIN (PHOBIUS); TRANSMEMBRANE (PHOBIUS); IPR023271 (SUPERFAMILY); TMhelix (TMHMM); TMhelix (TMHMM)
454	210689_length_307_cvg_7.0_tip_1_3	14	heat shock protein partial	103	7.78E-55	90.10%	0.101	G3DSA:3.30.30.30 (GENE3D); IPR013126 (PFAM); G3DSA:3.30.420.40 (GENE3D); PTHR19375 (PANTHER); PTHR19375:SF142 (PANTHER); SSF53067 (SUPERFAMILY)
457	210867_length_308_cvg_15.7_tip_1_3	59	huga_polan ame: full=hyaluronidase short=hya ame: full=hyaluronoglucosaminidase ame: allergen=pol a 2 flags: partial	103	1.14E-09	61.10%	0.366 Y	IPR013785 (G3DSA:3.20.20.GENE3D); IPR018155 (PFAM); TRANSMEMBRANE (PHOBIUS); CYTOPLASMIC_DOMAIN (PHOBIUS); NON_CYTOPLASMIC_DOMAIN (PHOBIUS); IPR017853 (SUPERFAMILY); TMhelix (TMHMM)
458	210911_length_308_cvg_8.6_tip_1_4	27	follistatin-related protein 5-like	103	7.23E-50	83.80%	0.107	PTHR10913 (PANTHER); PTHR10913:SF14 (PANTHER)
459	211137_length_308_cvg_3.6_tip_1_0	9	reticulocalbin-2-like isoform x1	103	1.85E-32	75.30%	0.104	IPR002048 (SMART); IPR011992 (G3DSA:1.10.238.GENE3D); IPR011992 (PFAM); PTHR10827:SF48 (PANTHER); PTHR10827 (PANTHER); IPR018247 (PROSITE_PATTERNS); IPR018247 (PROSITE_PATTERNS); IPR002048 (PROSITE_PROFILES); IPR002048 (PROSITE_PROFILES); SSF47473 (SUPERFAMILY)

460	211175_length_308_cvg_5.1_tip_1_4	16	vitellogenic carboxypeptidase	103	6.49E-33	73.60%	0.108	IPR001563 (PRINTS); IPR029058 (G3DSA:3.40.50.GENE3D); IPR001563 (PFAM); IPR001563 (PANTHER); IPR029058 (SUPERFAMILY)
461	211267_length_309_cvg_2.0_tip_1_1	6	dna polymerase alpha catalytic subunit	103	8.26E-28	71.00%	0.119	IPR000101 (PRINTS); IPR000101 (PFAM); IPR000101 (PANTHER); PTHR11686:SF18 (PANTHER); IPR029055 (SUPERFAMILY); TMhelix (TMHMM)
462	211443_length_309_cvg_11.0_tip_1_2	30	cd109 antigen	103	3.47E-32	74.20%	0.137	IPR011625 (PFAM); PTHR11412 (PANTHER); PTHR11412:SF84 (PANTHER)
463	211605_length_309_cvg_3.0_tip_1_1	10	neuroligin- y-linked isoform x1	103	6.47E-46	84.60%	0.142	IPR029058 (G3DSA:3.40.50.GENE3D); IPR002018 (PFAM); PTHR11559 (PANTHER); PTHR11559:SF157 (PANTHER); TRANSMEMBRANE (PHOBIUS); NON_CYTOPLASMIC_DOMAIN (PHOBIUS); CYTOPLASMIC_DOMAIN (PHOBIUS); IPR029058 (SUPERFAMILY)
464	211607_length_309_cvg_2.0_tip_1_0	6	lactose-binding lectin l-2-like	103	5.08E-10	58.60%	0.111	IPR001304 (PFAM); IPR016186 (G3DSA:3.10.100.GENE3D); IPR001304 (PROSITE_PROFILES); IPR016187 (SUPERFAMILY)
465	211609_length_309_cvg_2.4_tip_1_5	12	mitochondrial cardiolipin hydrolase	103	5.35E-37	70.80%	0.122	G3DSA:3.30.870.10 (GENE3D); IPR025202 (PFAM); PTHR21248 (PANTHER); IPR001736 (PROSITE_PROFILES); SSF56024 (SUPERFAMILY)
466	212195_length_311_cvg_2.0_tip_1_2	6	fez family zinc finger protein 2	103	6.26E-61	95.80%	0.121	IPR015880 (SMART); IPR007087 (PFAM); PF13465 (PFAM); IPR013087 (G3DSA:3.30.160.GENE3D); PF13912 (PFAM); IPR013087 (G3DSA:3.30.160.GENE3D); PTHR24409 (PANTHER); IPR007087 (PROSITE_PATTERNS); IPR007087 (PROSITE_PATTERNS); IPR007087 (PROSITE_PATTERNS); IPR007087 (PROSITE_PROFILES); IPR007087 (PROSITE_PROFILES); IPR007087 (PROSITE_PROFILES); IPR007087 (PROSITE_PROFILES); SSF57667 (SUPERFAMILY); SSF57667 (SUPERFAMILY)
467	212245_length_311_cvg_2.0_tip_1_1	4	hindsight transcription factor-like protein	104	5.24E-50	86.20%	0.11	IPR015880 (SMART); PF13894 (PFAM); IPR013087 (G3DSA:3.30.160.GENE3D); PF13465 (PFAM); IPR013087 (G3DSA:3.30.160.GENE3D); PTHR23233:SF42 (PANTHER); PTHR23233 (PANTHER); IPR007087 (PROSITE_PATTERNS); IPR007087 (PROSITE_PATTERNS); IPR007087 (PROSITE_PROFILES); IPR007087 (PROSITE_PROFILES); IPR007087 (PROSITE_PROFILES); SSF57667 (SUPERFAMILY); SSF57667 (SUPERFAMILY)
468	212339_length_311_cvg_2.1_tip_1_4	6	macrophage mannose receptor 1-like	104	1.28E-13	51.90%	0.159	IPR016186 (G3DSA:3.10.100.GENE3D); IPR001304 (PFAM); PTHR22803:SF16 (PANTHER); PTHR22803 (PANTHER); IPR001304 (PROSITE_PROFILES); IPR016187 (SUPERFAMILY)
469	212991_length_312_cvg_2.0_tip_1_1	6	low quality protein: zinc finger protein 665	104	3.61E-23	67.40%	0.101	IPR015880 (SMART); PF13465 (PFAM); IPR007087 (PFAM); IPR013087 (G3DSA:3.30.160.GENE3D); IPR013087 (G3DSA:3.30.160.GENE3D); PTHR24409:SF14 (PANTHER); PTHR24409 (PANTHER); IPR007087 (PROSITE_PATTERNS); IPR007087 (PROSITE_PATTERNS); IPR007087 (PROSITE_PROFILES); IPR007087 (PROSITE_PROFILES); IPR007087 (PROSITE_PROFILES); SSF57667 (SUPERFAMILY); SSF57667 (SUPERFAMILY)
470	213125_length_313_cvg_2.0_tip_1_4	9	partial	104	1.12E-11	52.10%	0.155	IPR000372 (SMART); G3DSA:3.80.10.10 (GENE3D); IPR000372 (PFAM)
471	213149_length_313_cvg_2.0_tip_1_4	6	hypothetical protein	104	7.98E-13	61.20%	0.101	G3DSA:1.20.120.20 (GENE3D); SSF47162 (SUPERFAMILY)
472	213357_length_313_cvg_2.1_tip_1_1	12	cubilin	104	1.01E-19	60.00%	0.155	IPR000859 (G3DSA:2.60.120.GENE3D); IPR000859 (PFAM); PTHR10127 (PANTHER); IPR000859 (PROSITE_PROFILES); IPR000859 (PROSITE_PROFILES); IPR000859 (SUPERFAMILY); IPR000859 (SUPERFAMILY)
473	213497_length_314_cvg_4.0_tip_1_2	14	adiponectin receptor protein	104	6.58E-41	90.20%	0.307	IPR004254 (PFAM); IPR004254 (PANTHER); PTHR20855:SF15 (PANTHER); CYTOPLASMIC_DOMAIN (PHOBIUS); NON_CYTOPLASMIC_DOMAIN (PHOBIUS); TRANSMEMBRANE (PHOBIUS); TRANSMEMBRANE (PHOBIUS); TRANSMEMBRANE (PHOBIUS); NON_CYTOPLASMIC_DOMAIN (PHOBIUS); CYTOPLASMIC_DOMAIN (PHOBIUS); TMhelix (TMHMM); TMhelix (TMHMM); TMhelix (TMHMM)
474	213513_length_314_cvg_4.7_tip_1_1	6	achain crystal structure of engineered northeast structural genomics consortium target	105	7.53E-16	60.60%	0.099	IPR002110 (SMART); IPR020683 (PFAM); IPR020683 (G3DSA:1.25.40.GENE3D); PTHR24193 (PANTHER); IPR002110 (PROSITE_PROFILES); IPR020683 (PROSITE_PROFILES); IPR002110 (PROSITE_PROFILES); IPR020683 (SUPERFAMILY)
475	213531_length_314_cvg_2.0_tip_1_0	7	epidermal growth factor receptor	105	3.10E-39	77.80%	0.101	IPR000494 (G3DSA:3.80.20.GENE3D); IPR000494 (PFAM); PTHR24416 (PANTHER); PTHR24416:SF88 (PANTHER); SSF52058 (SUPERFAMILY)
476	213597_length_314_cvg_2.3_tip_1_1	8	beta- -galactosyltransferase 3-like	105	3.72E-38	72.10%	0.126	IPR003859 (PRINTS); IPR027995 (PFAM); IPR029044 (G3DSA:3.90.550.GENE3D); IPR003859 (PANTHER); IPR029044 (SUPERFAMILY)
477	213665_length_314_cvg_3.0_tip_1_4	8	inversin protein	105	7.73E-42	80.00%	0.141	IPR002110 (SMART); IPR002110 (PFAM); IPR020683 (G3DSA:1.25.40.GENE3D); IPR020683 (PFAM); PTHR24178 (PANTHER); IPR020683 (PROSITE_PROFILES); IPR002110 (PROSITE_PROFILES); IPR002110 (PROSITE_PROFILES); IPR020683 (SUPERFAMILY)

478	213687_length_314_cvg_3.0_tip_1_1	10	zinc finger protein 316-like isoform x2	105	1.28E-15	61.40%	0.101	IPR015880 (SMART); IPR013087 (G3DSA:3.30.160.GENE3D); PF13465 (PFAM); PF13894 (PFAM); IPR013087 (G3DSA:3.30.160.GENE3D); PTHR24409 (PANTHER); IPR007087 (PROSITE_PATTERNS); IPR007087 (PROSITE_PATTERNS); IPR007087 (PROSITE_PROFILES); IPR007087 (PROSITE_PROFILES); IPR007087 (PROSITE_PROFILES); SSF57667 (SUPERFAMILY); SSF57667 (SUPERFAMILY)
479	213955_length_315_cvg_3.0_tip_1_2	9	lipophorin receptor	105	1.63E-54	88.40%	0.261	IPR000033 (SMART); IPR000033 (PFAM); IPR011042 (G3DSA:2.120.10.GENE3D); PTHR10529:SF208 (PANTHER); PTHR10529 (PANTHER); IPR000033 (PROSITE_PROFILES); IPR000033 (PROSITE_PROFILES); IPR000033 (PROSITE_PROFILES); SSF63825 (SUPERFAMILY)
480	214523_length_316_cvg_2.9_tip_1_2	13	tryptase-like isoform x1	105	1.21E-31	75.20%	0.193	G3DSA:2.40.10.10 (GENE3D); IPR001254 (PFAM); PTHR24268 (PANTHER); PTHR24268:SF95 (PANTHER); IPR001254 (PROSITE_PROFILES); IPR009003 (SUPERFAMILY)
481	214607_length_316_cvg_2.0_tip_1_1	7	protein flightless-1	105	1.64E-46	83.10%	0.123	IPR029006 (G3DSA:3.40.20.GENE3D); IPR007123 (PFAM); IPR029006 (G3DSA:3.40.20.GENE3D); IPR007122 (PANTHER); IPR029919 (PTHR11977:PANTHER); SSF55753 (SUPERFAMILY); SSF55753 (SUPERFAMILY)
482	214615_length_316_cvg_2.0_tip_1_4	6	c-type lectin domain family 4 member e-like	105	9.66E-12	54.30%	0.106	IPR001304 (PFAM); IPR016186 (G3DSA:3.10.100.GENE3D); PTHR22802:SF216 (PANTHER); PTHR22802 (PANTHER); IPR001304 (PROSITE_PROFILES); IPR016187 (SUPERFAMILY)
483	214669_length_316_cvg_2.0_tip_1_2	8	zinc finger protein 271 (zinc finger protein 7) (zinc finger protein znfphex133) (epstein-barr virus-induced zinc finger protein) (znf-eb) (ct-zfp48) (zinc finger protein	105	4.29E-20	64.60%	0.1	IPR015880 (SMART); IPR013087 (G3DSA:3.30.160.GENE3D); IPR013087 (G3DSA:3.30.160.GENE3D); IPR013087 (G3DSA:3.30.160.GENE3D); PF13894 (PFAM); PF13465 (PFAM); PTHR24409 (PANTHER); IPR007087 (PROSITE_PATTERNS); IPR007087 (PROSITE_PATTERNS); IPR007087 (PROSITE_PATTERNS); IPR007087 (PROSITE_PROFILES); IPR007087 (PROSITE_PROFILES); IPR007087 (PROSITE_PROFILES); SSF57667 (SUPERFAMILY); SSF57667 (SUPERFAMILY)
484	214687_length_316_cvg_2.0_tip_1_2	7	mam domain-containing glycosylphosphatidylinositol anchor protein 1	105	2.49E-14	58.20%	0.102	IPR000998 (PRINTS); IPR000998 (PFAM); PTHR23282 (PANTHER); IPR000998 (PROSITE_PROFILES); IPR013320 (SUPERFAMILY)
485	214699_length_316_cvg_4.0_tip_1_4	11	lachesin-like isoform x1	105	3.18E-49	83.90%	0.099	IPR013783 (G3DSA:2.60.40.GENE3D); IPR013106 (PFAM); IPR013098 (PFAM); PTHR19831:SF43 (PANTHER); PTHR19831 (PANTHER); IPR007110 (PROSITE_PROFILES); SSF48726 (SUPERFAMILY); SSF48726 (SUPERFAMILY)
486	214833_length_317_cvg_48.0_tip_1_2	200	serine protease	105	3.89E-15	72.40%	0.121	G3DSA:2.40.10.10 (GENE3D); IPR001254 (PFAM); PTHR24256 (PANTHER); IPR001254 (PROSITE_PROFILES); IPR009003 (SUPERFAMILY)
487	214877_length_317_cvg_2.0_tip_1_5	6	tyrosine-protein phosphatase non-receptor type 9-like	105	1.54E-47	84.30%	0.099	IPR000242 (PRINTS); IPR029021 (G3DSA:3.90.190.GENE3D); IPR000242 (PFAM); PTHR19134 (PANTHER); PTHR19134:SF232 (PANTHER); IPR000242 (PROSITE_PROFILES); IPR029021 (SUPERFAMILY)
488	215163_length_317_cvg_2.0_tip_1_4	8	zinc finger protein	106	7.15E-16	55.70%	0.099	IPR015880 (SMART); IPR013087 (G3DSA:3.30.160.GENE3D); PF13465 (PFAM); IPR013087 (G3DSA:3.30.160.GENE3D); IPR013087 (G3DSA:3.30.160.GENE3D); PTHR24409 (PANTHER); PTHR24409:SF14 (PANTHER); IPR007087 (PROSITE_PATTERNS); IPR007087 (PROSITE_PATTERNS); IPR007087 (PROSITE_PATTERNS); IPR007087 (PROSITE_PROFILES); IPR007087 (PROSITE_PROFILES); IPR007087 (PROSITE_PROFILES); IPR007087 (PROSITE_PROFILES); SSF57667 (SUPERFAMILY); SSF57667 (SUPERFAMILY)
489	215167_length_317_cvg_4.2_tip_1_0	16	cysteine proteinase cg12163	106	2.13E-59	91.00%	0.107	G3DSA:3.90.70.10 (GENE3D); IPR000668 (PFAM); PTHR12411:SF316 (PANTHER); IPR013128 (PANTHER); IPR025660 (PROSITE_PATTERNS); SSF54001 (SUPERFAMILY)
490	215261_length_317_cvg_3.0_tip_1_1	9	glycoside hydrolase family 1	106	1.04E-30	80.20%	0.109	IPR013781 (G3DSA:3.20.20.GENE3D); IPR001360 (PFAM); IPR001360 (PANTHER); IPR018120 (PROSITE_PATTERNS); IPR017853 (SUPERFAMILY)
491	215343_length_318_cvg_2.0_tip_1_3	9	insulin-like growth factor-binding protein complex acid labile subunit	106	2.28E-18	62.60%	0.116	IPR003591 (SMART); G3DSA:3.80.10.10 (GENE3D); IPR001611 (PFAM); IPR001611 (PFAM); PTHR24365 (PANTHER); SSF52058 (SUPERFAMILY)
492	215355_length_318_cvg_2.0_tip_1_3	7	group xv phospholipase a2-like	106	7.02E-34	69.90%	0.126	PTHR11440:SF47 (PANTHER); PTHR11440 (PANTHER)
493	215401_length_318_cvg_2.6_tip_1_1	12	zinc finger protein 358	106	1.21E-21	60.10%	0.127	IPR015880 (SMART); IPR013087 (G3DSA:3.30.160.GENE3D); IPR013087 (G3DSA:3.30.160.GENE3D); PF13465 (PFAM); IPR013087 (G3DSA:3.30.160.GENE3D); IPR013087 (G3DSA:3.30.160.GENE3D); PTHR24409 (PANTHER); IPR007087 (PROSITE_PATTERNS); IPR007087 (PROSITE_PATTERNS); IPR007087 (PROSITE_PROFILES); IPR007087 (PROSITE_PROFILES); IPR007087 (PROSITE_PROFILES); IPR007087 (PROSITE_PROFILES); SSF57667 (SUPERFAMILY); SSF57667 (SUPERFAMILY)
494	215779_length_319_cvg_87.0_tip_1_4	3019	protein npc2 homolog	106	3.72E-09	67.00%	0.109	IPR003172 (PFAM); IPR003172 (G3DSA:2.60.40.GENE3D); PTHR11306 (PANTHER); PTHR11306:SF3 (PANTHER); IPR014756 (SUPERFAMILY)
495	215921_length_319_cvg_4.0_tip_1_2	12	leucine-rich repeat-containing protein 70 isoform x1	106	6.55E-13	57.90%	0.104	IPR003591 (SMART); IPR001611 (PFAM); IPR001611 (PFAM); G3DSA:3.80.10.10 (GENE3D); PTHR24365 (PANTHER); SSF52058 (SUPERFAMILY)
496	216109_length_319_cvg_2.5_tip_1_1	7	extensin-like protein:near transporter	106	1.43E-17	72.90%	0.125	no IPS match

499	216281_length_320_cvg_3.0_tip_1_5	9	ecdysteroid-regulated 16 kda	106	1.18E-25	73.80%	0.132	IPR003172 (G3DSA:2.60.40.GENE3D); IPR003172 (PFAM); PTHR11306:SF0 (PANTHER); PTHR11306 (PANTHER); IPR014756 (SUPERFAMILY)
497	216143_length_319_cvg_4.0_tip_1_3	13	neuroendocrine convertase 2	107	6.37E-65	97.50%	0.102	IPR000209 (PFAM); IPR000209 (G3DSA:3.40.50.GENE3D); PTHR10795:SF11 (PANTHER); IPR015500 (PANTHER); IPR022398 (PROSITE_PATTERNS); IPR000209 (SUPERFAMILY)
498	216157_length_320_cvg_2.0_tip_1_1	8	zinc finger protein 665-like isoform x1	107	3.72E-16	62.10%	0.116	IPR015880 (SMART); IPR013087 (G3DSA:3.30.160.GENE3D); PF13465 (PFAM); IPR013087 (G3DSA:3.30.160.GENE3D); PTHR24409 (PANTHER); IPR007087 (PROSITE_PATTERNS); IPR007087 (PROSITE_PATTERNS); IPR007087 (PROSITE_PROFILES); IPR007087 (PROSITE_PROFILES); SSF57667 (SUPERFAMILY)
500	216465_length_320_cvg_3.0_tip_1_0	8	neurogenic locus notch homolog protein 1 isoform x1	107	6.77E-29	78.50%	0.099	IPR008197 (PRINTS); IPR008197 (SMART); IPR008197 (G3DSA:4.10.75.GENE3D); IPR008197 (PFAM); PTHR19441 (PANTHER); PTHR19441:SF10 (PANTHER); IPR008197 (PROSITE_PROFILES); IPR008197 (SUPERFAMILY)
501	216589_length_320_cvg_2.0_tip_1_0	8	transmembrane protease serine partial	107	1.44E-19	69.10%	0.228	IPR001254 (PFAM); G3DSA:2.40.10.10 (GENE3D); PTHR24268 (PANTHER); PTHR24268:SF81 (PANTHER); IPR018114 (PROSITE_PATTERNS); IPR001254 (PROSITE_PROFILES); IPR009003 (SUPERFAMILY)
502	216869_length_321_cvg_2.0_tip_1_0	9	mlrp2_acrmi ame: full=mam and ldl-receptor class a domain-containing protein 2 ame: full=skeletal organic matrix mam and ldl-receptor 2 short=som mam and ldl-receptor partial	107	7.00E-22	61.80%	0.111	IPR002172 (SMART); IPR002172 (G3DSA:4.10.400.GENE3D); IPR000998 (PFAM); IPR002172 (PFAM); PTHR23282:SF75 (PANTHER); PTHR23282 (PANTHER); IPR023415 (PROSITE_PATTERNS); IPR000998 (PROSITE_PROFILES); IPR002172 (PROSITE_PROFILES); IPR002172 (SUPERFAMILY); IPR013320 (SUPERFAMILY)
503	216967_length_321_cvg_2.0_tip_1_1	9	discoidin domain-containing receptor partial	107	4.02E-43	79.40%	0.107	IPR020635 (SMART); IPR001245 (PFAM); G3DSA:1.10.510.10 (GENE3D); PTHR24416 (PANTHER); PTHR24416:SF210 (PANTHER); IPR000719 (PROSITE_PROFILES); IPR011009 (SUPERFAMILY)
504	217059_length_322_cvg_7.0_tip_1_4	23	esterase fe4	107	5.57E-32	67.70%	0.101	IPR002018 (PFAM); IPR029058 (G3DSA:3.40.50.GENE3D); PTHR11559 (PANTHER); IPR019819 (PROSITE_PATTERNS); IPR029058 (SUPERFAMILY)
505	217111_length_322_cvg_3.0_tip_1_4	12	papilin isoform x2	107	4.78E-44	82.50%	0.112	IPR002223 (PRINTS); IPR002223 (SMART); IPR002223 (G3DSA:4.10.410.GENE3D); IPR002223 (G3DSA:4.10.410.GENE3D); IPR002223 (PFAM); PTHR10083 (PANTHER); IPR020901 (PROSITE_PATTERNS); IPR002223 (PROSITE_PROFILES); IPR002223 (SUPERFAMILY); IPR002223 (SUPERFAMILY)
506	217381_length_322_cvg_2.0_tip_1_5	9	zinc finger protein 282	107	1.21E-18	57.70%	0.128	IPR015880 (SMART); PF13465 (PFAM); IPR013087 (G3DSA:3.30.160.GENE3D); IPR013087 (G3DSA:3.30.160.GENE3D); PTHR24409 (PANTHER); IPR007087 (PROSITE_PATTERNS); IPR007087 (PROSITE_PATTERNS); IPR007087 (PROSITE_PROFILES); IPR007087 (PROSITE_PROFILES); IPR007087 (PROSITE_PROFILES); SSF57667 (SUPERFAMILY); SSF57667 (SUPERFAMILY)
507	217667_length_323_cvg_2.0_tip_1_2	8	platelet glycoprotein v	107	3.31E-16	57.00%	0.132	IPR003591 (SMART); G3DSA:3.80.10.10 (GENE3D); IPR001611 (PFAM); PTHR24373:SF12 (PANTHER); PTHR24373 (PANTHER); IPR001611 (PROSITE_PROFILES); IPR001611 (PROSITE_PROFILES); SSF52058 (SUPERFAMILY)
508	217683_length_323_cvg_5.0_tip_1_3	16	plexin- partial	108	2.79E-52	85.60%	0.098	IPR016201 (SMART); IPR015943 (G3DSA:2.130.10.GENE3D); IPR002165 (PFAM); G3DSA:3.30.1680.10 (GENE3D); PTHR22625:SF16 (PANTHER); PTHR22625 (PANTHER); IPR016201 (SUPERFAMILY); IPR001627 (SUPERFAMILY)
509	217733_length_323_cvg_23.0_tip_1_1	71	---NA---	108			0.125	IPR008197 (SMART); IPR008197 (PFAM); IPR008197 (G3DSA:4.10.75.GENE3D); IPR008197 (PROSITE_PROFILES); IPR008197 (SUPERFAMILY)
510	217857_length_323_cvg_2.0_tip_1_4	6	dorsal-ventral patterning protein partial	108	1.33E-27	62.70%	0.122	IPR001007 (SMART); IPR001007 (PFAM); PTHR11339:SF223 (PANTHER); PTHR11339 (PANTHER); SSF57603 (SUPERFAMILY)
511	217871_length_324_cvg_63.0_tip_1_1	2038	---NA---	108			0.134	no IPS match
512	218203_length_324_cvg_5.0_tip_1_4	15	cd4-specific ankyrin repeat protein	108	1.38E-10	69.70%	0.122	IPR002110 (SMART); IPR020683 (G3DSA:1.25.40.GENE3D); IPR020683 (PFAM); PTHR24182 (PANTHER); IPR002110 (PROSITE_PROFILES); IPR020683 (PROSITE_PROFILES); IPR002110 (PROSITE_PROFILES); IPR020683 (SUPERFAMILY)
514	218323_length_325_cvg_18.7_tip_0_5	50	wnt inhibitory factor 1 precursor	108	9.96E-61	93.40%	0.123	IPR000742 (SMART); IPR000742 (PFAM); IPR013111 (PFAM); IPR013032 (PFAM); G3DSA:2.10.25.10 (GENE3D); PTHR24838:SF276 (PANTHER); PTHR24838 (PANTHER); IPR013032 (PROSITE_PATTERNS); IPR013032 (PROSITE_PATTERNS); IPR013032 (PROSITE_PATTERNS); IPR013032 (PROSITE_PATTERNS); IPR013032 (PROSITE_PATTERNS); IPR013032 (PROSITE_PATTERNS); IPR000742 (PROSITE_PROFILES); IPR000742 (PROSITE_PROFILES); SSF57196 (SUPERFAMILY)
515	218469_length_325_cvg_2.0_tip_1_5	4	protein kinase brain isozyme	108	7.76E-69	99.50%	0.134	IPR002290 (SMART); IPR000719 (PFAM); G3DSA:1.10.510.10 (GENE3D); G3DSA:3.30.200.20 (GENE3D); PTHR24357:SF75 (PANTHER); PTHR24357 (PANTHER); IPR008271 (PROSITE_PATTERNS); IPR000719 (PROSITE_PROFILES); IPR011009 (SUPERFAMILY)

516	218515_length_325_cvg_4.0_tip_1_5	11	reelin- partial	108	4.43E-26	70.70%	0.126		PTHR11841 (PANTHER)
517	218571_length_325_cvg_3.0_tip_1_4	8	low quality protein: carboxypeptidase n subunit 2	108	4.91E-08	54.70%	0.122		IPR003591 (SMART); PF13516 (PFAM); G3DSA:3.80.10.10 (GENE3D); IPR001611 (PFAM); PTHR24373:SF100 (PANTHER); PTHR24373 (PANTHER); IPR001611 (PROSITE_PROFILES); IPR001611 (PROSITE_PROFILES); SSF52058 (SUPERFAMILY)
519	218729_length_326_cvg_2.0_tip_1_2	11	endonuclease mitochondrial	108	3.35E-32	67.80%	0.107		IPR001604 (PFAM); IPR020821 (G3DSA:3.40.570.GENE3D); PTHR13966 (PANTHER); SSF54060 (SUPERFAMILY)
521	218787_length_326_cvg_2.0_tip_1_5	7	leucine-rich transmembrane	108	1.50E-09	59.60%	0.099		PR00019 (PRINTS); IPR003591 (SMART); IPR001611 (PFAM); G3DSA:3.80.10.10 (GENE3D); IPR001611 (PFAM); PTHR24365 (PANTHER); IPR001611 (PROSITE_PROFILES); IPR001611 (PROSITE_PROFILES); IPR001611 (PROSITE_PROFILES); SSF52058 (SUPERFAMILY)
522	218873_length_326_cvg_8.7_tip_1_5	27	14-3-3 protein	108	2.19E-51	84.60%	0.107		IPR000308 (PRINTS); IPR023410 (SMART); G3DSA:1.20.190.20 (GENE3D); IPR023410 (PFAM); IPR000308 (PANTHER); IPR023410 (SUPERFAMILY)
523	218943_length_326_cvg_3.0_tip_1_5	10	---NA---	108			0.157		IPR000436 (SMART); IPR000436 (PFAM); G3DSA:2.10.70.10 (GENE3D); IPR000436 (PROSITE_PROFILES); SignalP-TM (SIGNALP_GRAM_POSITIVE); IPR000436 (SUPERFAMILY)
513	218307_length_325_cvg_76.8_tip_0_0	1229	venom allergen 3-like	109	6.02E-15	50.30%	0.122		IPR014044 (PFAM); IPR014044 (G3DSA:3.40.33.GENE3D); IPR001283 (PANTHER); IPR014044 (SUPERFAMILY)
518	218661_length_325_cvg_2.0_tip_1_0	7	low quality protein: titin-like	109	6.35E-45	78.20%	0.103		IPR003598 (SMART); IPR013783 (G3DSA:2.60.40.GENE3D); IPR013098 (PFAM); PTHR19897 (PANTHER); PTHR19897:SF149 (PANTHER); IPR007110 (PROSITE_PROFILES); SSF48726 (SUPERFAMILY)
520	218749_length_326_cvg_5.0_tip_1_3	15	beta- -galactosyltransferase 5	109	1.19E-17	67.20%	0.099		Coil (COILS); IPR029044 (G3DSA:3.90.550.GENE3D); IPR027995 (PFAM); IPR003859 (PANTHER); IPR029044 (SUPERFAMILY)
524	219005_length_326_cvg_3.0_tip_1_3	9	vanin-like protein 1 isoform x1	109	8.10E-10	53.60%	0.106		IPR012101 (PANTHER); PTHR10609:SF14 (PANTHER)
525	219139_length_327_cvg_3.0_tip_1_3	9	gastrula zinc finger protein	109	5.52E-21	61.10%	0.103		IPR015880 (SMART); IPR008598 (PFAM); IPR013087 (G3DSA:3.30.160.GENE3D); IPR013087 (G3DSA:3.30.160.GENE3D); PF13465 (PFAM); PTHR24409 (PANTHER); PTHR24409:SF14 (PANTHER); IPR007087 (PROSITE_PATTERNS); IPR007087 (PROSITE_PATTERNS); IPR007087 (PROSITE_PATTERNS); IPR007087 (PROSITE_PROFILES); IPR007087 (PROSITE_PROFILES); IPR007087 (PROSITE_PROFILES); SSF57667 (SUPERFAMILY); SSF57667 (SUPERFAMILY)
526	219635_length_328_cvg_2.0_tip_1_3	8	vascular endothelial growth factor receptor 1-like	110	2.06E-53	91.90%	0.152		IPR020635 (SMART); G3DSA:1.10.510.10 (GENE3D); IPR001245 (PFAM); PTHR24416:SF298 (PANTHER); PTHR24416 (PANTHER); IPR008266 (PROSITE_PATTERNS); IPR000719 (PROSITE_PROFILES); IPR011009 (SUPERFAMILY)
527	219875_length_329_cvg_3.1_tip_1_4	9	peptidyl-prolyl cis-trans isomerase g-like	110	7.53E-31	70.30%	0.098		IPR000072 (PFAM); IPR029034 (G3DSA:2.10.90.GENE3D); PTHR21719:SF1 (PANTHER); PTHR21719 (PANTHER); IPR000072 (PROSITE_PROFILES); IPR029034 (SUPERFAMILY)
528	220621_length_330_cvg_2.0_tip_1_0	5	leucine-rich repeat-containing protein 47-like	110	1.35E-25	67.80%	0.11		IPR003591 (SMART); SM00364 (SMART); IPR001611 (PFAM); G3DSA:3.80.10.10 (GENE3D); PTHR10947:SF2 (PANTHER); PTHR10947 (PANTHER); IPR001611 (PROSITE_PROFILES); IPR001611 (PROSITE_PROFILES); IPR001611 (PROSITE_PROFILES); SSF52058 (SUPERFAMILY)
529	220687_length_331_cvg_3.0_tip_1_2	10	mam and ldl-receptor class a domain-containing protein 2-like	110	3.00E-10	50.60%	0.117		IPR000998 (PFAM); PTHR23282:SF75 (PANTHER); PTHR23282 (PANTHER); CYTOPLASMIC_DOMAIN (PHOBIUS); NON_CYTOPLASMIC_DOMAIN (PHOBIUS); TRANSMEMBRANE (PHOBIUS); IPR000998 (PROSITE_PROFILES); IPR013320 (SUPERFAMILY)
530	220711_length_331_cvg_6.0_tip_1_2	24	chemosensory protein	110	4.25E-42	81.30%	0.834	Y	IPR005055 (G3DSA:1.10.2080.GENE3D); IPR005055 (PFAM); IPR005055 (PANTHER); PTHR11257:SF4 (PANTHER); SIGNAL_PEPTIDE (PHOBIUS); SIGNAL_PEPTIDE_C_REGION (PHOBIUS); SIGNAL_PEPTIDE_H_REGION (PHOBIUS); NON_CYTOPLASMIC_DOMAIN (PHOBIUS); SIGNAL_PEPTIDE_N_REGION (PHOBIUS); SignalP-noTM (SIGNALP_EUK); IPR005055 (SUPERFAMILY)
531	221085_length_332_cvg_4.0_tip_1_3	11	attractin-like protein 1-like	111	6.44E-31	68.10%	0.111		IPR016201 (SMART); IPR002165 (PFAM); PTHR10574:SF3 (PANTHER); PTHR10574 (PANTHER)
532	221173_length_332_cvg_3.7_tip_1_0	10	alcohol dehydrogenase	111	2.40E-29	68.40%	0.13		IPR020471 (PRINTS); IPR023210 (PFAM); IPR023210 (G3DSA:3.20.20.GENE3D); IPR001395 (PANTHER); PTHR11732:SF144 (PANTHER); IPR023210 (SUPERFAMILY)
533	221235_length_332_cvg_2.0_tip_1_4	6	chondroitin sulfate	111	7.24E-53	85.80%	0.12		IPR003378 (PFAM); PTHR12369 (PANTHER); PTHR12369:SF11 (PANTHER)
534	221721_length_333_cvg_3.0_tip_1_1	9	reelin	111	2.77E-27	66.00%	0.105		G3DSA:2.10.25.10 (GENE3D); IPR013111 (PFAM); IPR006149 (PFAM); PTHR11841 (PANTHER); IPR013032 (PROSITE_PATTERNS); IPR013032 (PROSITE_PATTERNS); SSF57196 (SUPERFAMILY)
536	221925_length_334_cvg_3.0_tip_1_1	12	achain kringle 2 domain of human plasminogen: nmr solution structure of trans-4-aminomethylcyclohexane-1-carboxylic acid complex	111	8.26E-25	67.20%	0.1		PR00018 (PRINTS); IPR000001 (SMART); IPR000001 (PFAM); G3DSA:2.40.20.10 (GENE3D); PTHR24259 (PANTHER); IPR018056 (PROSITE_PATTERNS); IPR000001 (PROSITE_PROFILES); IPR013806 (SUPERFAMILY)
537	222013_length_334_cvg_2.0_tip_1_1	8	growth differentiation factor 8	111	9.42E-30	69.20%	0.105		IPR001111 (PFAM); IPR015615 (PANTHER); IPR015616 (PTHR11848:PANTHER)

535	221893_length_334_cvg_2.0_tip_1_3	7	tyrosine kinase receptor cad96ca	112	1.38E-53	89.60%	0.281		IPR001245 (PRINTS); IPR020635 (SMART); G3DSA:1.10.510.10 (GENE3D); IPR001245 (PFAM); PTHR24416:SF268 (PANTHER); PTHR24416 (PANTHER); IPR000719 (PROSITE_PROFILES); IPR011009 (SUPERFAMILY)
538	222423_length_335_cvg_4.0_tip_1_4	13	protein will die slowly	112	9.92E-73	98.20%	0.105		IPR001680 (SMART); IPR001680 (PFAM); IPR015943 (G3DSA:2.130.10.GENE3D); PTHR22847:SF308 (PANTHER); PTHR22847 (PANTHER); IPR019775 (PROSITE_PATTERNS); IPR001680 (PROSITE_PROFILES); IPR001680 (PROSITE_PROFILES); IPR017986 (PROSITE_PROFILES); IPR017986 (SUPERFAMILY)
539	222889_length_336_cvg_2.0_tip_1_2	9	low quality protein: sco-spondin	112	6.71E-12	54.10%	0.695	Y	IPR002172 (PRINTS); IPR002172 (SMART); IPR002172 (PFAM); IPR002172 (G3DSA:4.10.400.GENE3D); IPR002172 (G3DSA:4.10.400.GENE3D); PTHR10529:SF204 (PANTHER); PTHR10529 (PANTHER); SIGNAL_PEPTIDE_N_REGION (PHOBIUS); SIGNAL_PEPTIDE_C_REGION (PHOBIUS); NON_CYTOPLASMIC_DOMAIN (PHOBIUS); SIGNAL_PEPTIDE (PHOBIUS); SIGNAL_PEPTIDE_H_REGION (PHOBIUS); IPR002172 (PROSITE_PROFILES); IPR002172 (PROSITE_PROFILES); SignalP-noTM (SIGNALP_EUK); IPR002172 (SUPERFAMILY); IPR002172 (SUPERFAMILY); TMhelix (TMHMM)
540	222983_length_337_cvg_2.0_tip_1_4	8	scavenger partial	112	1.11E-47	82.70%	0.115		IPR001695 (PFAM); PTHR19331 (PANTHER); PTHR19331:SF77 (PANTHER); IPR001190 (PROSITE_PROFILES)
541	223121_length_337_cvg_2.0_tip_1_5	6	fibrillin-2-like	112	1.07E-47	84.10%	0.117		IPR000742 (SMART); IPR001881 (SMART); IPR001881 (PFAM); G3DSA:2.10.25.10 (GENE3D); G3DSA:2.10.25.10 (GENE3D); G3DSA:2.10.25.10 (GENE3D); IPR011398 (PANTHER); PTHR24039:SF20 (PANTHER); IPR013032 (PROSITE_PATTERNS); IPR000152 (PROSITE_PATTERNS); IPR000152 (PROSITE_PATTERNS); IPR000152 (PROSITE_PATTERNS); IPR018097 (PROSITE_PATTERNS); IPR013032 (PROSITE_PATTERNS); IPR018097 (PROSITE_PATTERNS); IPR000742 (PROSITE_PROFILES); IPR000742 (PROSITE_PROFILES); IPR009030 (SUPERFAMILY)
542	223239_length_337_cvg_7.2_tip_1_4	22	serine protease p69	112	2.11E-21	59.80%	0.105		IPR001254 (PFAM); G3DSA:2.40.10.10 (GENE3D); PTHR24260 (PANTHER); PTHR24260:SF45 (PANTHER); IPR018114 (PROSITE_PATTERNS); IPR001254 (PROSITE_PROFILES); IPR009003 (SUPERFAMILY)
543	223547_length_338_cvg_2.0_tip_1_0	7	laminin subunit alpha	113	2.70E-43	80.60%	0.109		IPR002049 (SMART); G3DSA:2.10.25.10 (GENE3D); G3DSA:2.10.25.10 (GENE3D); IPR002049 (PFAM); PTHR10574 (PANTHER); PTHR10574:SF39 (PANTHER); IPR002049 (PROSITE_PATTERNS); SSF57196 (SUPERFAMILY); SSF57196 (SUPERFAMILY)
544	223559_length_338_cvg_2.0_tip_1_1	7	calcium activated chlorine	113	3.38E-15	53.80%	0.102		IPR015394 (PFAM); PTHR10579 (PANTHER); PTHR10579:SF43 (PANTHER)
545	223841_length_339_cvg_4.0_tip_1_4	16	growth differentiation factor 8-like	113	1.32E-65	84.20%	0.101		IPR001839 (SMART); IPR029034 (G3DSA:2.10.90.GENE3D); IPR001839 (PFAM); IPR015616 (PTHR11848:PANTHER); IPR015615 (PANTHER); IPR017948 (PROSITE_PATTERNS); IPR001839 (PROSITE_PROFILES); IPR029034 (SUPERFAMILY)
546	223995_length_339_cvg_3.7_tip_1_5	13	lysosomal pro-x carboxypeptidase-like	113	4.32E-27	75.90%	0.198		IPR008758 (PFAM); IPR008758 (PANTHER); PTHR11010:SF11 (PANTHER); CYTOPLASMIC_DOMAIN (PHOBIUS); NON_CYTOPLASMIC_DOMAIN (PHOBIUS); TRANSMEMBRANE (PHOBIUS); TMhelix (TMHMM)
547	224017_length_339_cvg_3.9_tip_1_1	14	xanthine dehydrogenase oxidase	113	4.32E-51	84.00%	0.105		IPR008274 (PFAM); IPR008274 (G3DSA:3.30.365.GENE3D); PTHR11908:SF32 (PANTHER); PTHR11908 (PANTHER); IPR008274 (SUPERFAMILY)
548	224037_length_339_cvg_7.0_tip_1_2	25	cda2 isoform a	113	5.86E-50	83.70%	0.246		IPR002172 (SMART); IPR002557 (PFAM); IPR002172 (PFAM); IPR002172 (G3DSA:4.10.400.GENE3D); IPR002557 (G3DSA:2.170.140.GENE3D); PTHR11177:SF136 (PANTHER); PTHR11177 (PANTHER); IPR023415 (PROSITE_PATTERNS); IPR002172 (PROSITE_PROFILES); IPR002172 (SUPERFAMILY); IPR002557 (SUPERFAMILY)
549	224075_length_339_cvg_16.0_tip_1_5	31	acyl- delta desaturase	113	3.23E-55	83.10%	0.507	Y	IPR015876 (PRINTS); IPR005804 (PFAM); PTHR11351 (PANTHER); PTHR11351:SF28 (PANTHER); TRANSMEMBRANE (PHOBIUS); CYTOPLASMIC_DOMAIN (PHOBIUS); NON_CYTOPLASMIC_DOMAIN (PHOBIUS); SignalP-noTM (SIGNALP_EUK); TMhelix (TMHMM)
553	224635_length_341_cvg_22.7_tip_0_2	39	cytochrome p450 3a11-like	113	4.99E-36	73.60%	0.125		IPR002401 (PRINTS); IPR001128 (PFAM); IPR001128 (G3DSA:1.10.630.GENE3D); PTHR24292 (PANTHER); PTHR24292:SF24 (PANTHER); IPR017972 (PROSITE_PATTERNS); IPR001128 (SUPERFAMILY)
555	224743_length_341_cvg_5.2_tip_1_2	18	gastrula zinc finger	113	7.84E-20	62.10%	0.099		IPR015880 (SMART); IPR013087 (G3DSA:3.30.160.GENE3D); PF13465 (PFAM); IPR013087 (G3DSA:3.30.160.GENE3D); PTHR24409 (PANTHER); IPR007087 (PROSITE_PATTERNS); IPR007087 (PROSITE_PATTERNS); IPR007087 (PROSITE_PATTERNS); IPR007087 (PROSITE_PROFILES); IPR007087 (PROSITE_PROFILES); IPR007087 (PROSITE_PROFILES); SSF57667 (SUPERFAMILY); SSF57667 (SUPERFAMILY)
550	224163_length_340_cvg_8.0_tip_1_0	16	basement membrane-specific heparan sulfate proteoglycan core protein	114	1.95E-25	56.20%	0.101		IPR003599 (SMART); IPR003598 (SMART); IPR002172 (G3DSA:4.10.400.GENE3D); PF13895 (PFAM); IPR013783 (G3DSA:2.60.40.GENE3D); IPR007110 (PROSITE_PROFILES); SSF48726 (SUPERFAMILY); IPR002172 (SUPERFAMILY)

551	224375_length_340_cvg_7.0_tip_1_3	25	von willebrand factor a domain-containing protein 8-like	114	1.33E-54	85.00%	0.121	PTHR21610 (PANTHER); PTHR21610:SF9 (PANTHER); IPR002035 (SUPERFAMILY)
552	224557_length_341_cvg_14.3_tip_0_3	35	egf domain protein	114	5.25E-12	48.10%	0.101	IPR002223 (G3DSA:4.10.410.GENE3D); IPR023413 (G3DSA:2.40.155.GENE3D); IPR002223 (PFAM); IPR002223 (G3DSA:4.10.410.GENE3D); PTHR10083 (PANTHER); IPR013032 (PROSITE_PATTERNS); IPR020901 (PROSITE_PATTERNS); IPR002223 (PROSITE_PROFILES); IPR000742 (PROSITE_PROFILES); IPR002223 (SUPERFAMILY); SSF57196 (SUPERFAMILY); IPR002223 (SUPERFAMILY)
554	224699_length_341_cvg_2.0_tip_1_3	6	voltage-gated sodium partial	114	2.31E-57	91.60%	0.276	IPR005821 (PFAM); IPR027359 (G3DSA:1.20.120.GENE3D); PTHR10037 (PANTHER); IPR028814 (PTHR10037:PANTHER); CYTOPLASMIC_DOMAIN (PHOBIUS); TRANSMEMBRANE (PHOBIUS); NON_CYTOPLASMIC_DOMAIN (PHOBIUS); TRANSMEMBRANE (PHOBIUS); NON_CYTOPLASMIC_DOMAIN (PHOBIUS); SSF81324 (SUPERFAMILY); TMhelix (TMHMM); TMhelix (TMHMM)
556	224827_length_342_cvg_2.0_tip_1_3	7	low quality protein: titin-like	114	6.36E-49	86.00%	0.103	IPR013783 (G3DSA:2.60.40.GENE3D); IPR013098 (PFAM); PTHR19897 (PANTHER); PTHR19897:SF149 (PANTHER); IPR007110 (PROSITE_PROFILES); SSF48726 (SUPERFAMILY)
557	224915_length_342_cvg_7.0_tip_1_4	23	er degradation-enhancing alpha-mannosidase-like protein 2-like	114	1.56E-57	89.50%	0.1	IPR001382 (PRINTS); IPR001382 (G3DSA:1.50.10.GENE3D); IPR001382 (PFAM); PTHR11742:SF37 (PANTHER); IPR001382 (PANTHER); IPR001382 (SUPERFAMILY)
558	224987_length_342_cvg_2.0_tip_1_0	9	secreted frizzled-related protein 3	114	1.94E-31	71.10%	0.27	IPR020067 (SMART); IPR020067 (G3DSA:1.10.2000.GENE3D); IPR020067 (PFAM); IPR015526 (PANTHER); PTHR11309:SF83 (PANTHER); IPR020067 (PROSITE_PROFILES); IPR020067 (SUPERFAMILY)
559	225111_length_342_cvg_4.0_tip_1_1	16	maguk p55 subfamily member 7 isoform x4	114	1.81E-54	91.40%	0.1	IPR008145 (SMART); IPR008145 (PFAM); G3DSA:3.30.63.10 (GENE3D); IPR027417 (G3DSA:3.40.50.GENE3D); PTHR23122 (PANTHER); PTHR23122:SF14 (PANTHER); IPR020590 (PROSITE_PATTERNS); IPR008144 (PROSITE_PROFILES); IPR027417 (SUPERFAMILY)
561	225377_length_343_cvg_4.0_tip_1_2	15	maguk p55 subfamily member 7 isoform x1	114	6.25E-60	90.20%	0.113	IPR001452 (PRINTS); IPR001452 (SMART); IPR011511 (PFAM); G3DSA:2.30.30.40 (GENE3D); PTHR23122:SF14 (PANTHER); PTHR23122 (PANTHER); IPR001452 (PROSITE_PROFILES); IPR001452 (SUPERFAMILY)
560	225305_length_343_cvg_2.0_tip_1_0	6	gastrula zinc finger	115	1.26E-20	63.40%	0.102	IPR015880 (SMART); PF13894 (PFAM); IPR013087 (G3DSA:3.30.160.GENE3D); IPR007087 (PFAM); PF13465 (PFAM); IPR013087 (G3DSA:3.30.160.GENE3D); PTHR24409 (PANTHER); PTHR24409:SF13 (PANTHER); IPR007087 (PROSITE_PATTERNS); IPR007087 (PROSITE_PATTERNS); IPR007087 (PROSITE_PATTERNS); IPR007087 (PROSITE_PROFILES); IPR007087 (PROSITE_PROFILES); IPR007087 (PROSITE_PROFILES); SSF57667 (SUPERFAMILY); SSF57667 (SUPERFAMILY)
562	225475_length_343_cvg_4.0_tip_1_0	15	neuroendocrine convertase 2	115	1.45E-59	85.70%	0.1	IPR002884 (PFAM); IPR008979 (G3DSA:2.60.120.GENE3D); IPR015500 (PANTHER); PTHR10795:SF11 (PANTHER); IPR008979 (SUPERFAMILY)
563	225633_length_344_cvg_2.0_tip_1_1	11	gamma-glutamyltranspeptidase 1-like	115	1.48E-45	78.90%	0.103	IPR000101 (PRINTS); IPR000101 (PFAM); PTHR11686:SF15 (PANTHER); IPR000101 (PANTHER); IPR029055 (SUPERFAMILY)
564	225965_length_345_cvg_2.0_tip_1_4	10	protein yellow-like	115	8.80E-20	60.70%	0.112	IPR017996 (PFAM); IPR011042 (G3DSA:2.120.10.GENE3D)
565	225989_length_345_cvg_2.0_tip_1_1	11	spermatogenesis-associated protein 5-like	115	4.86E-45	80.60%	0.143	Coil (COILS); G3DSA:1.10.8.60 (GENE3D); IPR027417 (G3DSA:3.40.50.GENE3D); IPR003959 (PFAM); PTHR23077:SF27 (PANTHER); PTHR23077 (PANTHER); IPR003960 (PROSITE_PATTERNS); IPR027417 (SUPERFAMILY)
566	226157_length_345_cvg_11.0_tip_1_2	42	collagen alpha-2 partial	115	3.96E-66	89.20%	0.132	IPR001442 (SMART); IPR001442 (PFAM); IPR001442 (G3DSA:2.170.240.GENE3D); PTHR24023:SF397 (PANTHER); PTHR24023 (PANTHER); IPR001442 (PROSITE_PROFILES); IPR016187 (SUPERFAMILY)
567	226203_length_345_cvg_3.8_tip_1_0	13	zinc c2h2 type	115	9.90E-16	57.70%	0.099	IPR015880 (SMART); IPR013087 (G3DSA:3.30.160.GENE3D); PF13465 (PFAM); IPR013087 (G3DSA:3.30.160.GENE3D); IPR013087 (G3DSA:3.30.160.GENE3D); IPR007087 (PFAM); PTHR24409:SF14 (PANTHER); PTHR24409 (PANTHER); IPR007087 (PROSITE_PATTERNS); IPR007087 (PROSITE_PATTERNS); IPR007087 (PROSITE_PATTERNS); IPR007087 (PROSITE_PROFILES); IPR007087 (PROSITE_PROFILES); IPR007087 (PROSITE_PROFILES); SSF57667 (SUPERFAMILY); SSF57667 (SUPERFAMILY)
568	226251_length_345_cvg_2.2_tip_1_2	8	pr domain zinc finger protein 1 isoform x2	115	7.06E-21	65.00%	0.099	IPR015880 (SMART); IPR013087 (G3DSA:3.30.160.GENE3D); IPR013087 (G3DSA:3.30.160.GENE3D); PF13465 (PFAM); IPR013087 (G3DSA:3.30.160.GENE3D); PTHR24409 (PANTHER); PTHR24409:SF14 (PANTHER); IPR007087 (PROSITE_PATTERNS); IPR007087 (PROSITE_PATTERNS); IPR007087 (PROSITE_PATTERNS); IPR007087 (PROSITE_PROFILES); IPR007087 (PROSITE_PROFILES); IPR007087 (PROSITE_PROFILES); SSF57667 (SUPERFAMILY); SSF57667 (SUPERFAMILY)

569	226415_length_346_cvg_2.8_tip_1_5	8	zinc finger protein 891	115	2.01E-21	61.10%	0.1		IPR015880 (SMART); IPR013087 (G3DSA:3.30.160.GENE3D); PF13465 (PFAM); IPR013087 (G3DSA:3.30.160.GENE3D); PTHR24409:SF13 (PANTHER); PTHR24409 (PANTHER); IPR007087 (PROSITE_PATTERNS); IPR007087 (PROSITE_PATTERNS); IPR007087 (PROSITE_PATTERNS); IPR007087 (PROSITE_PATTERNS); IPR007087 (PROSITE_PROFILES); IPR007087 (PROSITE_PROFILES); IPR007087 (PROSITE_PROFILES); IPR007087 (PROSITE_PROFILES); SSF57667 (SUPERFAMILY); SSF57667 (SUPERFAMILY)
571	226789_length_347_cvg_2.0_tip_1_5	8	zinc finger protein 45-like	115	1.07E-24	59.10%	0.111		IPR015880 (SMART); PF13912 (PFAM); IPR013087 (G3DSA:3.30.160.GENE3D); PF13894 (PFAM); IPR013087 (G3DSA:3.30.160.GENE3D); IPR013087 (G3DSA:3.30.160.GENE3D); PF13465 (PFAM); PTHR24409 (PANTHER); IPR007087 (PROSITE_PATTERNS); IPR007087 (PROSITE_PATTERNS); IPR007087 (PROSITE_PATTERNS); IPR007087 (PROSITE_PROFILES); IPR007087 (PROSITE_PROFILES); IPR007087 (PROSITE_PROFILES); SSF57667 (SUPERFAMILY); SSF57667 (SUPERFAMILY)
573	226903_length_347_cvg_7.0_tip_1_2	27	26s protease regulatory subunit 6b	115	3.77E-75	99.90%	0.106		IPR027417 (G3DSA:3.40.50.GENE3D); IPR003959 (PFAM); PTHR23073 (PANTHER); IPR027417 (SUPERFAMILY)
575	226927_length_347_cvg_2.0_tip_1_2	9	matrix metalloproteinase	115	7.60E-22	79.10%	0.102		IPR018487 (SMART); IPR018487 (PFAM); IPR000585 (G3DSA:2.110.10.GENE3D); PTHR10201 (PANTHER); PTHR10201:SF131 (PANTHER); IPR018487 (PROSITE_PROFILES); IPR000585 (SUPERFAMILY)
576	226973_length_347_cvg_5.4_tip_1_2	21	low-density lipoprotein receptor-related protein 2	115	7.80E-60	86.30%	0.172		IPR002172 (PRINTS); IPR002172 (SMART); IPR002172 (G3DSA:4.10.400.GENE3D); IPR002172 (PFAM); IPR002172 (G3DSA:4.10.400.GENE3D); PTHR10529 (PANTHER); IPR023415 (PROSITE_PATTERNS); IPR023415 (PROSITE_PATTERNS); IPR002172 (PROSITE_PROFILES); IPR002172 (PROSITE_PROFILES); IPR002172 (PROSITE_PROFILES); IPR002172 (SUPERFAMILY); IPR002172 (SUPERFAMILY); IPR002172 (SUPERFAMILY)
570	226665_length_347_cvg_2.0_tip_1_3	6	otopetrin-2 isoform x1	116	1.38E-22	63.50%	0.122		IPR004878 (PFAM); PTHR21522:SF37 (PANTHER); IPR004878 (PANTHER); CYTOPLASMIC_DOMAIN (PHOBIUS); NON_CYTOPLASMIC_DOMAIN (PHOBIUS); TRANSMEMBRANE (PHOBIUS); TMhelix (TMHMM)
572	226857_length_347_cvg_2.0_tip_1_3	8	zinc finger protein 777- partial	116	6.09E-14	72.70%	0.122		IPR015880 (SMART); PF13465 (PFAM); IPR013087 (G3DSA:3.30.160.GENE3D); IPR013087 (G3DSA:3.30.160.GENE3D); PTHR24409 (PANTHER); IPR007087 (PROSITE_PATTERNS); IPR007087 (PROSITE_PATTERNS); IPR007087 (PROSITE_PROFILES); IPR007087 (PROSITE_PROFILES); SSF57667 (SUPERFAMILY)
574	226905_length_347_cvg_2.0_tip_1_0	6	endothelin-converting enzyme	116	1.94E-63	89.40%	0.118		IPR018497 (PRINTS); IPR024079 (G3DSA:3.40.390.GENE3D); IPR018497 (PFAM); IPR000718 (PANTHER); IPR029733 (PTHR11733:PANTHER); SSF55486 (SUPERFAMILY)
577	227059_length_348_cvg_3.0_tip_1_3	13	gastrula zinc finger	116	3.03E-23	61.30%	0.1		IPR015880 (SMART); PF13465 (PFAM); IPR013087 (G3DSA:3.30.160.GENE3D); IPR013087 (G3DSA:3.30.160.GENE3D); IPR013087 (G3DSA:3.30.160.GENE3D); PTHR24409 (PANTHER); IPR007087 (PROSITE_PATTERNS); IPR007087 (PROSITE_PATTERNS); IPR007087 (PROSITE_PATTERNS); IPR007087 (PROSITE_PROFILES); IPR007087 (PROSITE_PROFILES); IPR007087 (PROSITE_PROFILES); SSF57667 (SUPERFAMILY); SSF57667 (SUPERFAMILY)
578	227095_length_348_cvg_2.0_tip_1_3	6	---NA---	116			0.109		no IPS match
579	227153_length_348_cvg_2.8_tip_1_0	10	matrix metalloproteinase-9	116	8.65E-11	47.50%	0.161		IPR000562 (SMART); IPR000562 (PFAM); IPR000562 (G3DSA:2.10.10.GENE3D); IPR000562 (PROSITE_PROFILES); IPR000562 (PROSITE_PROFILES); IPR013806 (SUPERFAMILY); IPR013806 (SUPERFAMILY)
580	227213_length_348_cvg_3.0_tip_1_5	9	sortilin-related receptor-like isoform x2	116	7.26E-32	65.90%	0.108		G3DSA:2.130.10.140 (GENE3D); PTHR12106:SF7 (PANTHER); PTHR12106 (PANTHER); SSF110296 (SUPERFAMILY)
581	227261_length_348_cvg_3.4_tip_1_5	12	kielin chordin-like protein	116	4.18E-11	43.00%	0.103		G3DSA:2.10.70.10 (GENE3D); SSF57603 (SUPERFAMILY); SSF57603 (SUPERFAMILY); SSF57603 (SUPERFAMILY)
582	227341_length_348_cvg_2.0_tip_1_0	4	titin isoform x1	116	1.46E-34	68.30%	0.099		IPR003599 (SMART); IPR013098 (PFAM); IPR013783 (G3DSA:2.60.40.GENE3D); IPR020675 (PANTHER); IPR007110 (PROSITE_PROFILES); SSF48726 (SUPERFAMILY)
583	227533_length_349_cvg_2.0_tip_1_2	5	doublecortin-like protein kinase	116	3.73E-43	76.30%	0.592	Y	G3DSA:1.10.510.10 (GENE3D); IPR000719 (PFAM); PTHR24347:SF49 (PANTHER); IPR020636 (PANTHER); IPR000719 (PROSITE_PROFILES); SignalP-noTM (SIGNALP_EUK); IPR011009 (SUPERFAMILY)
585	227571_length_349_cvg_3.0_tip_1_2	9	laccase-1-like isoform x1	116	2.47E-47	79.00%	0.124		IPR011706 (PFAM); IPR008972 (G3DSA:2.60.40.GENE3D); PTHR11709:SF33 (PANTHER); PTHR11709 (PANTHER); IPR002355 (PROSITE_PATTERNS); IPR002355 (PROSITE_PATTERNS); IPR008972 (SUPERFAMILY)
588	227687_length_349_cvg_2.0_tip_1_4	7	de-cadherin-like isoform x1	116	6.28E-19	57.90%	0.11		IPR002126 (G3DSA:2.60.40.GENE3D); IPR015919 (SUPERFAMILY)
591	228041_length_350_cvg_6.1_tip_1_2	13	upf0764 protein c16orf89 homolog	116	6.29E-10	49.00%	0.176		no IPS match
584	227559_length_349_cvg_3.5_tip_1_3	14	down syndrome cell adhesion molecule-like protein dscam2 isoform x29	117	1.64E-59	90.00%	0.11		IPR003961 (PFAM); IPR013783 (G3DSA:2.60.40.GENE3D); PTHR10489 (PANTHER); PTHR10489:SF567 (PANTHER); IPR003961 (PROSITE_PROFILES); IPR003961 (PROSITE_PROFILES); IPR003961 (SUPERFAMILY)

586	227641_length_349_cvg_5.2_tip_1_3	12	serine (or cysteine) proteinase clade b member	117	1.09E-12	71.10%	0.113	G3DSA:2.30.39.10 (GENE3D); IPR023796 (PFAM); PTHR11461:SF52 (PANTHER); IPR000215 (PANTHER); IPR023795 (PROSITE_PATTERNS); IPR023796 (SUPERFAMILY)
587	227665_length_349_cvg_2.0_tip_1_0	7	fasciclin-2 isoform x4	117	1.65E-15	54.70%	0.13	IPR003598 (SMART); IPR003599 (SMART); IPR013783 (G3DSA:2.60.40.GENE3D); IPR013098 (PFAM); PTHR10489 (PANTHER); IPR007110 (PROSITE_PROFILES); SSF48726 (SUPERFAMILY)
589	227881_length_350_cvg_2.0_tip_1_3	12	cubilin, partial	117	8.92E-27	59.00%	0.118	IPR000859 (SMART); IPR000859 (G3DSA:2.60.120.GENE3D); IPR000859 (PFAM); PTHR10127 (PANTHER); IPR000859 (PROSITE_PROFILES); IPR000859 (SUPERFAMILY)
590	227923_length_350_cvg_2.0_tip_1_3	7	aael005189- partial	117	1.42E-33	68.50%	0.31	IPR003598 (SMART); IPR013783 (G3DSA:2.60.40.GENE3D); IPR013098 (PFAM); PTHR19831:SF50 (PANTHER); PTHR19831 (PANTHER); IPR007110 (PROSITE_PROFILES); SSF48726 (SUPERFAMILY)
592	228081_length_351_cvg_3.0_tip_1_3	13	phospholipase a2-like	117	2.95E-18	65.90%	0.101	IPR016090 (G3DSA:1.20.90.GENE3D); IPR016090 (PFAM); IPR013090 (PROSITE_PATTERNS); IPR016090 (SUPERFAMILY)
593	228127_length_351_cvg_3.0_tip_1_5	9	plexin-a4	117	2.19E-68	97.30%	0.109	IPR013548 (PFAM); PTHR22625 (PANTHER)
594	228175_length_351_cvg_3.0_tip_1_3	10	gastrula zinc finger protein - partial	117	2.44E-24	61.10%	0.105	IPR015880 (SMART); IPR013087 (G3DSA:3.30.160.GENE3D); PF13465 (PFAM); IPR013087 (G3DSA:3.30.160.GENE3D); IPR007087 (PFAM); PTHR24409 (PANTHER); PTHR24409:SF14 (PANTHER); IPR007087 (PROSITE_PATTERNS); IPR007087 (PROSITE_PATTERNS); IPR007087 (PROSITE_PATTERNS); IPR007087 (PROSITE_PATTERNS); IPR007087 (PROSITE_PROFILES); IPR007087 (PROSITE_PROFILES); IPR007087 (PROSITE_PROFILES); IPR007087 (PROSITE_PROFILES); SSF57667 (SUPERFAMILY); SSF57667 (SUPERFAMILY)
595	228499_length_352_cvg_2.0_tip_1_3	4	dlp hdl-bgbp precursor	118	5.73E-37	75.40%	0.1	IPR015255 (PFAM); IPR015817 (G3DSA:2.20.50.GENE3D); PTHR23345 (PANTHER); PTHR23345:SF11 (PANTHER); IPR015819 (SUPERFAMILY)
596	228609_length_352_cvg_3.0_tip_1_3	11	growth differentiation factor 8-like	118	1.23E-08	67.00%	0.107	IPR001839 (SMART); IPR029034 (G3DSA:2.10.90.GENE3D); IPR001839 (PFAM); IPR015615 (PANTHER); PTHR11848:SF126 (PANTHER); IPR001839 (PROSITE_PROFILES); IPR029034 (SUPERFAMILY)
597	228679_length_353_cvg_3.0_tip_1_0	9	bone morphogenetic protein receptor type-2	118	1.58E-48	79.80%	0.139	IPR000719 (PFAM); G3DSA:1.10.510.10 (GENE3D); PTHR23255:SF52 (PANTHER); IPR000333 (PANTHER); IPR000719 (PROSITE_PROFILES); IPR011009 (SUPERFAMILY)
598	228775_length_353_cvg_2.0_tip_1_1	10	achain crystal structure of anti-il4 darpin 44c12v5	118	1.19E-14	60.40%	0.108	IPR002110 (PRINTS); IPR002110 (SMART); IPR020683 (PFAM); IPR020683 (G3DSA:1.25.40.GENE3D); PTHR24198 (PANTHER); IPR020683 (PROSITE_PROFILES); IPR002110 (PROSITE_PROFILES); IPR002110 (PROSITE_PROFILES); IPR002110 (PROSITE_PROFILES); IPR020683 (SUPERFAMILY)
599	228811_length_353_cvg_2.0_tip_1_4	8	low-density lipoprotein	118	2.88E-40	68.00%	0.104	IPR002172 (PRINTS); IPR002172 (SMART); IPR002172 (PFAM); IPR002172 (G3DSA:4.10.400.GENE3D); IPR002172 (G3DSA:4.10.400.GENE3D); IPR002172 (G3DSA:4.10.400.GENE3D); PTHR10529 (PANTHER); IPR023415 (PROSITE_PATTERNS); IPR023415 (PROSITE_PATTERNS); IPR023415 (PROSITE_PATTERNS); IPR002172 (PROSITE_PROFILES); IPR002172 (PROSITE_PROFILES); IPR002172 (SUPERFAMILY); IPR002172 (SUPERFAMILY); IPR002172 (SUPERFAMILY)
600	228893_length_353_cvg_3.0_tip_1_0	5	hypothetical protein, partial	118	1.06E-11	49.40%	0.154	no IPS match
601	228893_length_353_cvg_3.0_tip_1_3	5	collagen alpha-1 chain	118	1.15E-34	76.50%	0.112	IPR008160 (PFAM); PTHR24023 (PANTHER); PTHR24023:SF164 (PANTHER)
602	229133_length_354_cvg_2.2_tip_1_2	9	protein rtoa- partial	118	6.98E-12	57.80%	0.121	SignalP-TM (SIGNALP_GRAM_POSITIVE)
603	229263_length_354_cvg_3.6_tip_1_0	13	zinc c2h2 type domain-containing protein	118	7.72E-16	58.30%	0.099	IPR015880 (SMART); IPR013087 (G3DSA:3.30.160.GENE3D); IPR013087 (G3DSA:3.30.160.GENE3D); PF13465 (PFAM); PTHR24409:SF14 (PANTHER); PTHR24409 (PANTHER); IPR007087 (PROSITE_PATTERNS); IPR007087 (PROSITE_PATTERNS); IPR007087 (PROSITE_PROFILES); IPR007087 (PROSITE_PROFILES); SSF57667 (SUPERFAMILY)
609	229860_length_356_cvg_2.6_tip_1_2	8	zinc finger protein 699-like isoform x2	118	3.25E-22	61.80%	0.106	IPR015880 (SMART); IPR013087 (G3DSA:3.30.160.GENE3D); PF13465 (PFAM); IPR013087 (G3DSA:3.30.160.GENE3D); PTHR24409:SF13 (PANTHER); PTHR24409 (PANTHER); IPR007087 (PROSITE_PATTERNS); IPR007087 (PROSITE_PATTERNS); IPR007087 (PROSITE_PATTERNS); IPR007087 (PROSITE_PATTERNS); IPR007087 (PROSITE_PATTERNS); IPR007087 (PROSITE_PROFILES); IPR007087 (PROSITE_PROFILES); IPR007087 (PROSITE_PROFILES); IPR007087 (PROSITE_PROFILES); IPR007087 (PROSITE_PROFILES); SSF57667 (SUPERFAMILY); SSF57667 (SUPERFAMILY); SSF57667 (SUPERFAMILY)
610	229898_length_356_cvg_2.0_tip_1_2	10	moxd1 homolog 1	118	2.92E-31	67.30%	0.097	IPR014784 (G3DSA:2.60.120.GENE3D); PF03712 (PFAM); IPR000945 (PANTHER); IPR008977 (SUPERFAMILY)
604	229396_length_355_cvg_3.8_tip_1_3	16	tyrosine-protein kinase src64b	119	2.61E-61	90.20%	0.118	IPR001245 (PRINTS); IPR020635 (SMART); IPR001245 (PFAM); G3DSA:1.10.510.10 (GENE3D); PTHR24418 (PANTHER); IPR008266 (PROSITE_PATTERNS); IPR000719 (PROSITE_PROFILES); IPR011009 (SUPERFAMILY)

605	229518_length_355_cvg_2.0_tip_1_0	9	limbic system-associated membrane protein	119	3.71E-40	74.40%	0.108	IPR003599 (SMART); IPR003598 (SMART); IPR013098 (PFAM); IPR013783 (G3DSA:2.60.40.GENE3D); PTHR19831 (PANTHER); PTHR19831:SF45 (PANTHER); IPR007110 (PROSITE_PROFILES); SSF48726 (SUPERFAMILY)
606	229700_length_356_cvg_3.0_tip_1_1	10	phosphatidylinositol -trisphosphate 3-phosphatase and dual-specificity protein phosphatase pten	119	2.69E-46	86.70%	0.214	IPR029021 (G3DSA:3.90.190.GENE3D); PTHR12305 (PANTHER); IPR029023 (PROSITE_PROFILES); IPR029021 (SUPERFAMILY)
607	229786_length_356_cvg_7.0_tip_1_1	29	frizzled-4	119	3.60E-49	87.10%	0.145	IPR020067 (SMART); IPR020067 (G3DSA:1.10.2000.GENE3D); IPR020067 (PFAM); IPR015526 (PANTHER); IPR026551 (PTHR11309:PANTHER); IPR020067 (PROSITE_PROFILES); IPR020067 (SUPERFAMILY)
608	229800_length_356_cvg_3.0_tip_1_1	13	zinc finger protein 271 (zinc finger protein 7) (zinc finger protein znfphex133) (epstein-barr virus-induced zinc finger protein) (znf-eb) (ct-zfp48) (zinc finger protein	119	1.43E-14	51.50%	0.136	IPR015880 (SMART); IPR013087 (G3DSA:3.30.160.GENE3D); IPR013087 (G3DSA:3.30.160.GENE3D); PF13894 (PFAM); PF13465 (PFAM); PTHR24409 (PANTHER); PTHR24409:SF14 (PANTHER); IPR007087 (PROSITE_PATTERNS); IPR007087 (PROSITE_PATTERNS); IPR007087 (PROSITE_PATTERNS); IPR007087 (PROSITE_PROFILES); IPR007087 (PROSITE_PROFILES); SSF57667 (SUPERFAMILY); SSF57667 (SUPERFAMILY)
611	229988_length_357_cvg_2.0_tip_1_4	7	zinc finger	119	1.48E-62	85.90%	0.109	IPR015880 (SMART); IPR013087 (G3DSA:3.30.160.GENE3D); IPR007087 (PFAM); IPR013087 (G3DSA:3.30.160.GENE3D); PTHR23228 (PANTHER); PTHR23228:SF78 (PANTHER); IPR007087 (PROSITE_PATTERNS); IPR007087 (PROSITE_PATTERNS); IPR007087 (PROSITE_PROFILES); IPR007087 (PROSITE_PROFILES); IPR007087 (PROSITE_PROFILES); SSF57667 (SUPERFAMILY); SSF57667 (SUPERFAMILY)
612	230108_length_357_cvg_3.0_tip_1_2	10	thrombospondin type-1 domain-containing protein 4-like	119	3.43E-41	77.10%	0.106	IPR010294 (PFAM); PTHR13723 (PANTHER)
613	230162_length_357_cvg_2.0_tip_1_1	9	zinc finger protein 519- partial	119	3.21E-23	60.00%	0.108	IPR015880 (SMART); IPR013087 (G3DSA:3.30.160.GENE3D); IPR013087 (G3DSA:3.30.160.GENE3D); IPR013087 (G3DSA:3.30.160.GENE3D); PF13894 (PFAM); IPR013087 (G3DSA:3.30.160.GENE3D); IPR007087 (PFAM); PTHR24409 (PANTHER); PTHR24409:SF14 (PANTHER); IPR007087 (PROSITE_PATTERNS); IPR007087 (PROSITE_PATTERNS); IPR007087 (PROSITE_PATTERNS); IPR007087 (PROSITE_PATTERNS); IPR007087 (PROSITE_PROFILES); IPR007087 (PROSITE_PROFILES); IPR007087 (PROSITE_PROFILES); IPR007087 (PROSITE_PROFILES); SSF57667 (SUPERFAMILY); SSF57667 (SUPERFAMILY)
614	230196_length_357_cvg_6.4_tip_1_2	22	hypothetical protein JL09_g3666	119	1.23E-07	54.00%	0.102	no IPS match
615	230340_length_357_cvg_3.5_tip_1_2	12	maguk p55 subfamily member 5	119	5.48E-44	79.50%	0.108	G3DSA:2.30.30.40 (GENE3D); PTHR23122 (PANTHER); PTHR23122:SF32 (PANTHER)
617	230394_length_358_cvg_2.0_tip_1_1	9	xanthine dehydrogenase oxidase isoform x1	119	2.71E-54	82.90%	0.164	IPR008274 (PFAM); IPR008274 (G3DSA:3.30.365.GENE3D); PTHR11908:SF32 (PANTHER); PTHR11908 (PANTHER); IPR022407 (PROSITE_PATTERNS); IPR008274 (SUPERFAMILY)
618	230436_length_358_cvg_3.0_tip_1_5	13	transient receptor potential channel pyrexia-like	119	2.19E-51	88.20%	0.129	IPR002110 (SMART); IPR020683 (G3DSA:1.25.40.GENE3D); IPR020683 (PFAM); PTHR24133 (PANTHER); IPR002110 (PROSITE_PROFILES); IPR020683 (PROSITE_PROFILES); IPR002110 (PROSITE_PROFILES); IPR020683 (SUPERFAMILY)
620	230594_length_358_cvg_2.0_tip_1_2	8	subfamily member 17	119	3.63E-45	82.30%	0.105	Coil (COILS); IPR001623 (PRINTS); IPR001623 (SMART); IPR001623 (PFAM); IPR001623 (G3DSA:1.10.287.GENE3D); PTHR24078 (PANTHER); PTHR24078:SF153 (PANTHER); IPR001623 (PROSITE_PROFILES); IPR001623 (SUPERFAMILY)
616	230384_length_358_cvg_3.0_tip_1_3	13	Le-cadherin	120	4.56E-28	61.00%	0.109	no IPS match
619	230496_length_358_cvg_2.4_tip_1_3	8	chorion peroxidase	120	2.17E-48	72.00%	0.112	IPR019791 (PFAM); IPR019791 (G3DSA:1.10.640.GENE3D); PTHR11475 (PANTHER); IPR019791 (PROSITE_PROFILES); IPR010255 (SUPERFAMILY)
621	230728_length_359_cvg_2.0_tip_1_3	10	protein ndnf-like	120	2.30E-17	49.30%	0.12	IPR013783 (G3DSA:2.60.40.GENE3D)
622	230872_length_359_cvg_21.7_tip_0_3	63	nidogen-2 isoform x2	120	3.18E-15	59.70%	0.101	IPR002223 (G3DSA:4.10.410.GENE3D); IPR002223 (PFAM); IPR023413 (G3DSA:2.40.155.GENE3D); IPR024731 (PFAM); IPR020901 (PROSITE_PATTERNS); IPR013032 (PROSITE_PATTERNS); IPR013032 (PROSITE_PATTERNS); IPR002223 (PROSITE_PROFILES); IPR000742 (PROSITE_PROFILES); IPR002223 (SUPERFAMILY)
623	231046_length_360_cvg_2.0_tip_1_1	9	beta- -n-acetylgalactosaminyltransferase bre-4-like	120	4.79E-43	69.20%	0.123	IPR027791 (PFAM); IPR029044 (G3DSA:3.90.550.GENE3D); IPR003859 (PANTHER); IPR029044 (SUPERFAMILY)
624	231272_length_360_cvg_3.0_tip_1_1	11	gastrula zinc finger protein - partial	120	5.78E-42	68.70%	0.106	IPR015880 (SMART); IPR013087 (G3DSA:3.30.160.GENE3D); IPR013087 (G3DSA:3.30.160.GENE3D); PF13912 (PFAM); IPR013087 (G3DSA:3.30.160.GENE3D); PF13465 (PFAM); PTHR24409 (PANTHER); IPR007087 (PROSITE_PATTERNS); IPR007087 (PROSITE_PATTERNS); IPR007087 (PROSITE_PATTERNS); IPR007087 (PROSITE_PATTERNS); IPR007087 (PROSITE_PROFILES); IPR007087 (PROSITE_PROFILES); IPR007087 (PROSITE_PROFILES); IPR007087 (PROSITE_PROFILES); SSF57667 (SUPERFAMILY); SSF57667 (SUPERFAMILY); SSF57667 (SUPERFAMILY)

625	231286_length_361_cvg_3.0_tip_1_5	11	lysosomal pro-x carboxypeptidase-like	120	2.03E-21	75.00%	0.134	IPR008758 (PFAM); PTHR11010:SF11 (PANTHER); IPR008758 (PANTHER); CYTOPLASMIC_DOMAIN (PHOBIUS); TRANSMEMBRANE (PHOBIUS); NON_CYTOPLASMIC_DOMAIN (PHOBIUS); TMhelix (TMHMM)
626	231358_length_361_cvg_2.0_tip_1_1	9	zinc finger protein 37-like	120	3.07E-16	60.00%	0.104	IPR015880 (SMART); IPR013087 (G3DSA:3.30.160.GENE3D); PF13465 (PFAM); IPR007087 (PFAM); IPR013087 (G3DSA:3.30.160.GENE3D); IPR013087 (G3DSA:3.30.160.GENE3D); PTHR24409:SF14 (PANTHER); PTHR24409 (PANTHER); IPR007087 (PROSITE_PATTERNS); IPR007087 (PROSITE_PATTERNS); IPR007087 (PROSITE_PATTERNS); IPR007087 (PROSITE_PROFILES); IPR007087 (PROSITE_PROFILES); IPR007087 (PROSITE_PROFILES); SSF57667 (SUPERFAMILY); SSF57667 (SUPERFAMILY)
627	231392_length_361_cvg_7.1_tip_1_4	24	lysosomal alpha-glucosidase-like	120	4.72E-51	78.00%	0.109	IPR000322 (PFAM); PTHR22762:SF7 (PANTHER); PTHR22762 (PANTHER); IPR017853 (SUPERFAMILY)
628	231532_length_361_cvg_2.5_tip_1_4	10	limbic system-associated membrane protein	120	3.14E-34	67.50%	0.098	IPR013783 (G3DSA:2.60.40.GENE3D); IPR013098 (PFAM); IPR013783 (G3DSA:2.60.40.GENE3D); PTHR19831:SF47 (PANTHER); PTHR19831 (PANTHER); IPR007110 (PROSITE_PROFILES); SSF48726 (SUPERFAMILY); SSF48726 (SUPERFAMILY)
631	231628_length_362_cvg_4.0_tip_1_5	17	nardilysin-like isoform x1	120	5.02E-34	72.10%	0.125	IPR011237 (G3DSA:3.30.830.GENE3D); IPR007863 (PFAM); PTHR11851 (PANTHER); PTHR11851:SF108 (PANTHER); IPR011249 (SUPERFAMILY)
629	231560_length_361_cvg_4.7_tip_1_0	20	c4b-binding protein beta	121	8.43E-58	83.70%	0.181	IPR013783 (G3DSA:2.60.40.GENE3D); SSF48726 (SUPERFAMILY)
630	231590_length_361_cvg_2.0_tip_1_0	5	proprotein convertase subtilisin kexin type 7 isoform x2	121	7.64E-55	81.50%	0.106	IPR000209 (PFAM); IPR000209 (G3DSA:3.40.50.GENE3D); IPR015500 (PANTHER); PTHR10795:SF9 (PANTHER); IPR000209 (SUPERFAMILY)
632	231698_length_362_cvg_60.5_tip_0_3	467	blastula protease 10-like	121	4.46E-37	69.10%	0.108	IPR001506 (PRINTS); IPR006026 (SMART); IPR024079 (G3DSA:3.40.390.GENE3D); IPR001506 (PFAM); PTHR10127 (PANTHER); SSF55486 (SUPERFAMILY)
633	231738_length_362_cvg_2.0_tip_1_4	7	ring finger and spry domain-containing protein 1-like	121	4.95E-15	81.10%	0.12	IPR001841 (SMART); IPR013083 (G3DSA:3.30.40.GENE3D); PF13920 (PFAM); IPR001841 (PROSITE_PROFILES); SSF57850 (SUPERFAMILY)
634	231760_length_362_cvg_2.0_tip_1_3	8	cartilage oligomeric matrix protein isoform x1	121	3.72E-26	58.90%	0.1	Coil (COILS); IPR024665 (PFAM)
635	231876_length_362_cvg_2.0_tip_1_4	10	carbohydrate sulfotransferase 11 isoform x1	121	4.59E-19	64.80%	0.128	IPR005331 (PFAM); PTHR12137:SF29 (PANTHER); IPR018011 (PANTHER)
636	232106_length_363_cvg_7.4_tip_1_3	25	peptidase inhibitor 16	121	2.30E-14	54.70%	0.151	IPR001283 (PRINTS); IPR014044 (G3DSA:3.40.33.GENE3D); IPR014044 (PFAM); IPR001283 (PANTHER); IPR018244 (PROSITE_PATTERNS); IPR014044 (SUPERFAMILY)
637	232128_length_363_cvg_2.0_tip_1_1	9	nedd4 family-interacting protein 1-like	121	5.46E-43	68.30%	0.269	IPR019325 (PFAM); PTHR13396 (PANTHER); TRANSMEMBRANE (PHOBIUS); NON_CYTOPLASMIC_DOMAIN (PHOBIUS); TRANSMEMBRANE (PHOBIUS); NON_CYTOPLASMIC_DOMAIN (PHOBIUS); CYTOPLASMIC_DOMAIN (PHOBIUS); TMhelix (TMHMM)
638	232190_length_363_cvg_3.0_tip_1_5	7	heat shock protein 70	121	3.14E-44	80.10%	0.097	Coil (COILS); IPR029048 (G3DSA:1.20.1270.GENE3D); IPR029047 (G3DSA:2.60.34.GENE3D); IPR013126 (PFAM); PTHR19375 (PANTHER); IPR029048 (SUPERFAMILY); IPR029047 (SUPERFAMILY)
639	232216_length_363_cvg_6.0_tip_1_1	23	trafficking protein particle complex subunit 1	121	1.70E-63	86.80%	0.167	IPR007233 (PFAM); G3DSA:3.30.450.70 (GENE3D); PTHR23249:SF16 (PANTHER); IPR007233 (PANTHER); IPR011012 (SUPERFAMILY)
641	232338_length_364_cvg_3.7_tip_1_2	14	trafficking protein particle complex subunit 1-like isoform x3	121	2.34E-08	71.00%	0.112	IPR007233 (PFAM)
643	232506_length_364_cvg_3.6_tip_1_2	14	mam and ldl-receptor class a domain-containing protein c10orf112-like	121	2.84E-06	52.50%	0.103	IPR000998 (PFAM); IPR000998 (PROSITE_PROFILES); IPR013320 (SUPERFAMILY)
640	232244_length_364_cvg_2.0_tip_1_0	8	low-density lipoprotein receptor-related protein 4-like	122	6.83E-06	63.17%	0.119	IPR002172 (SMART); IPR002172 (G3DSA:4.10.400.GENE3D); IPR002172 (PFAM); CYTOPLASMIC_DOMAIN (PHOBIUS); NON_CYTOPLASMIC_DOMAIN (PHOBIUS); TRANSMEMBRANE (PHOBIUS); IPR002172 (PROSITE_PROFILES); IPR002172 (SUPERFAMILY)
642	232426_length_364_cvg_2.0_tip_1_0	7	h aca ribonucleoprotein complex non-core subunit naf1	122	3.70E-48	79.30%	0.379 Y	G3DSA:2.40.10.230 (GENE3D); IPR007504 (PFAM); PTHR31991 (PANTHER); IPR009000 (SUPERFAMILY)
644	232852_length_366_cvg_2.0_tip_1_3	10	inversin- partial	122	3.09E-40	73.40%	0.143	IPR002110 (SMART); IPR020683 (PFAM); PF13637 (PFAM); IPR020683 (G3DSA:1.25.40.GENE3D); IPR020683 (G3DSA:1.25.40.GENE3D); PTHR24178 (PANTHER); IPR002110 (PROSITE_PROFILES); IPR002110 (PROSITE_PROFILES); IPR002110 (PROSITE_PROFILES); IPR020683 (PROSITE_PROFILES); IPR002110 (PROSITE_PROFILES); IPR020683 (SUPERFAMILY)
645	232870_length_366_cvg_3.0_tip_1_2	13	secreted frizzled-related protein 3	122	5.59E-16	59.00%	0.137	Coil (COILS); IPR018933 (SMART); G3DSA:2.40.50.120 (GENE3D); IPR018933 (PFAM); PTHR11309:SF83 (PANTHER); IPR015526 (PANTHER); IPR001134 (PROSITE_PROFILES); IPR008993 (SUPERFAMILY)
646	232906_length_366_cvg_3.0_tip_1_3	13	dehydrogenase reductase sdr family member on chromosome x	122	3.17E-33	68.60%	0.114	IPR016040 (G3DSA:3.40.50.GENE3D); IPR002198 (PFAM); PTHR24320 (PANTHER); SSF51735 (SUPERFAMILY)
647	232944_length_366_cvg_4.2_tip_1_4	16	laccase 1 isoform x1	122	1.85E-42	73.70%	0.13	IPR008972 (G3DSA:2.60.40.GENE3D); IPR001117 (PFAM); PTHR11709:SF33 (PANTHER); PTHR11709 (PANTHER); IPR008972 (SUPERFAMILY)
648	233048_length_366_cvg_3.0_tip_1_0	10	exocyst complex component 7	122	6.07E-37	77.70%	0.105	PTHR12542:SF7 (PANTHER); IPR004140 (PANTHER)

649	233188_length_367_cvg_3.0_tip_1_4	12	gamma-glutamyltranspeptidase 1-like	122	2.08E-23	57.10%	0.238		IPR000101 (PRINTS); IPR000101 (PFAM); IPR000101 (PANTHER); PTHR11686:SF18 (PANTHER); IPR029055 (SUPERFAMILY)
650	233386_length_368_cvg_2.0_tip_1_2	8	rega-1 protein	122	2.59E-08	52.20%	0.129		IPR003599 (SMART); IPR003598 (SMART); IPR013783 (G3DSA:2.60.40.GENE3D); IPR013098 (PFAM); PTHR19831 (PANTHER); IPR007110 (PROSITE_PROFILES); SSF48726 (SUPERFAMILY)
652	233436_length_368_cvg_2.0_tip_1_2	7	deoxyribonuclease-2- partial	122	2.97E-17	67.60%	0.115		IPR004947 (PFAM); IPR004947 (PANTHER)
651	233418_length_368_cvg_5.0_tip_1_0	16	metalloprotease i	123	6.43E-16	57.10%	0.118		IPR013856 (PFAM); IPR013856 (G3DSA:3.10.170.GENE3D)
653	233498_length_368_cvg_3.0_tip_1_4	12	zinc finger protein 628	123	1.22E-10	71.60%	0.114		IPR015880 (SMART); IPR013087 (G3DSA:3.30.160.GENE3D); PF13465 (PFAM); IPR013087 (G3DSA:3.30.160.GENE3D); PTHR24409 (PANTHER); IPR007087 (PROSITE_PATTERNS); IPR007087 (PROSITE_PROFILES); SSF57667 (SUPERFAMILY)
654	233610_length_369_cvg_3.0_tip_1_1	13	dnaj homolog subfamily c member 11	123	2.60E-55	90.00%	0.103		IPR001623 (PRINTS); IPR001623 (SMART); IPR001623 (PFAM); IPR001623 (G3DSA:1.10.287.GENE3D); PTHR24078 (PANTHER); PTHR24078:SF159 (PANTHER); IPR001623 (PROSITE_PROFILES); IPR001623 (SUPERFAMILY)
655	233618_length_369_cvg_2.0_tip_1_0	8	zinc finger partial	123	4.06E-23	58.00%	0.098		IPR015880 (SMART); PF13894 (PFAM); PF13465 (PFAM); IPR013087 (G3DSA:3.30.160.GENE3D); IPR013087 (G3DSA:3.30.160.GENE3D); IPR013087 (G3DSA:3.30.160.GENE3D); PTHR24409:SF14 (PANTHER); PTHR24409 (PANTHER); IPR007087 (PROSITE_PATTERNS); IPR007087 (PROSITE_PATTERNS); IPR007087 (PROSITE_PATTERNS); IPR007087 (PROSITE_PATTERNS); IPR007087 (PROSITE_PROFILES); IPR007087 (PROSITE_PROFILES); IPR007087 (PROSITE_PROFILES); IPR007087 (PROSITE_PROFILES); SSF57667 (SUPERFAMILY); SSF57667 (SUPERFAMILY); SSF57667 (SUPERFAMILY)
656	233818_length_369_cvg_2.0_tip_1_0	8	vascular endothelial growth factor receptor 1	123	4.07E-15	47.30%	0.141		PF13895 (PFAM); IPR013783 (G3DSA:2.60.40.GENE3D); SSF48726 (SUPERFAMILY)
657	233826_length_369_cvg_2.0_tip_1_0	10	cardioactive peptide	123	2.67E-11	60.50%	0.106		IPR024276 (PFAM); TRANSMEMBRANE (PHOBIUS); NON_CYTOPLASMIC_DOMAIN (PHOBIUS); CYTOPLASMIC_DOMAIN (PHOBIUS); IPR024276 (PRODOM); TMhelix (TMHMM)
658	233848_length_369_cvg_2.0_tip_1_1	9	membrane metallo-endopeptidase-like 1-like	123	3.16E-33	69.80%	0.108		G3DSA:1.10.1380.10 (GENE3D); IPR008753 (PFAM); IPR000718 (PANTHER); PTHR11733:SF113 (PANTHER); SSF55486 (SUPERFAMILY)
661	234068_length_370_cvg_2.9_tip_1_4	11	endochitinase isoform x1	123	4.71E-45	86.20%	0.104		IPR013781 (G3DSA:3.20.20.GENE3D); IPR001223 (PFAM); PTHR11177 (PANTHER); PTHR11177:SF144 (PANTHER); IPR017853 (SUPERFAMILY)
659	233910_length_370_cvg_43.9_tip_1_3	155	thioredoxin 1	124	8.45E-38	85.50%	0.126		IPR013766 (PFAM); IPR012336 (G3DSA:3.40.30.GENE3D); IPR005746 (PANTHER); IPR012336 (PROSITE_PROFILES); IPR012336 (SUPERFAMILY)
660	234056_length_370_cvg_3.0_tip_1_3	10	protein fam3c-like	124	1.48E-31	65.30%	0.113		PTHR14592:SF7 (PANTHER); IPR026101 (PANTHER)
662	234082_length_370_cvg_4.5_tip_0_0	16	protein creg1	124	9.68E-50	74.40%	0.117		PF13883 (PFAM); IPR012349 (G3DSA:2.30.110.GENE3D); PTHR13343 (PANTHER); PTHR13343:SF12 (PANTHER); IPR012349 (SUPERFAMILY)
663	234256_length_371_cvg_2.0_tip_1_1	7	n-alpha-acetyltransferase auxiliary subunit	124	3.12E-35	64.70%	0.176		IPR019183 (PFAM); PTHR22767 (PANTHER); PTHR22767:SF3 (PANTHER)
664	234290_length_371_cvg_2.0_tip_1_1	6	irregular chiasm c-roughest protein isoform x1	124	1.58E-42	71.60%	0.135		IPR003598 (SMART); IPR003599 (SMART); IPR013098 (PFAM); IPR013783 (G3DSA:2.60.40.GENE3D); PTHR11640 (PANTHER); PTHR11640:SF54 (PANTHER); IPR007110 (PROSITE_PROFILES); SSF48726 (SUPERFAMILY)
665	234380_length_371_cvg_2.0_tip_1_1	10	glucose dehydrogenase	124	1.31E-37	68.70%	0.108		IPR000172 (PFAM); G3DSA:3.30.560.10 (GENE3D); G3DSA:3.50.50.60 (GENE3D); PTHR11552 (PANTHER); PTHR11552:SF77 (PANTHER); SSF51905 (SUPERFAMILY)
666	234434_length_371_cvg_21.3_tip_0_1	93	acyl- delta desaturase	124	3.98E-65	87.00%	0.478	Y	IPR015876 (PRINTS); IPR005804 (PFAM); PTHR11351:SF28 (PANTHER); PTHR11351 (PANTHER); CYTOPLASMIC_DOMAIN (PHOBIUS); NON_CYTOPLASMIC_DOMAIN (PHOBIUS); TRANSMEMBRANE (PHOBIUS); SignalP-noTM (SIGNALP_EUK); TMhelix (TMHMM)
667	234488_length_372_cvg_5.0_tip_1_2	16	chitinase 1a	124	2.48E-58	82.50%	0.137		IPR013781 (G3DSA:3.20.20.GENE3D); IPR001223 (PFAM); IPR029070 (G3DSA:3.10.50.GENE3D); PTHR11177:SF144 (PANTHER); PTHR11177 (PANTHER); IPR017853 (SUPERFAMILY)
668	234628_length_372_cvg_2.0_tip_1_1	12	97 kda heat shock protein isoform x2	124	7.30E-42	87.80%	0.133		G3DSA:3.30.420.40 (GENE3D); IPR013126 (PFAM); G3DSA:3.30.30.30 (GENE3D); PTHR19375:SF78 (PANTHER); PTHR19375 (PANTHER); SSF53067 (SUPERFAMILY)
669	234878_length_373_cvg_2.0_tip_1_4	8	basement membrane-specific heparan sulfate proteoglycan core protein	124	1.68E-31	65.10%	0.103		IPR018031 (SMART); IPR000034 (PFAM); PTHR10574:SF39 (PANTHER); PTHR10574 (PANTHER); IPR000034 (PROSITE_PROFILES)
670	234888_length_373_cvg_9.0_tip_1_4	28	dipeptidyl peptidase 9	124	1.41E-66	91.40%	0.186		IPR029058 (G3DSA:3.40.50.GENE3D); IPR001375 (PFAM); PTHR11731:SF110 (PANTHER); PTHR11731 (PANTHER); IPR029058 (SUPERFAMILY)
671	234934_length_373_cvg_5.6_tip_1_1	23	calbindin-32 isoform x2	124	2.67E-75	98.00%	0.106		IPR002048 (SMART); IPR011992 (PFAM); IPR011992 (G3DSA:1.10.238.GENE3D); IPR002048 (PFAM); IPR011992 (G3DSA:1.10.238.GENE3D); PTHR19972 (PANTHER); IPR018247 (PROSITE_PATTERNS); IPR018247 (PROSITE_PATTERNS); IPR018247 (PROSITE_PATTERNS); IPR002048 (PROSITE_PROFILES); IPR002048 (PROSITE_PROFILES); IPR002048 (PROSITE_PROFILES); SSF47473 (SUPERFAMILY)

672	235024_length_373_cvg_2.0_tip_1_2	7	zinc finger protein	124	1.16E-24	56.00%	0.13	IPR015880 (SMART); IPR013087 (G3DSA:3.30.160.GENE3D); IPR013087 (G3DSA:3.30.160.GENE3D); IPR007087 (PFAM); PF13894 (PFAM); IPR013087 (G3DSA:3.30.160.GENE3D); PTHR24409:SF14 (PANTHER); PTHR24409 (PANTHER); IPR007087 (PROSITE_PATTERNS); IPR007087 (PROSITE_PATTERNS); IPR007087 (PROSITE_PATTERNS); IPR007087 (PROSITE_PATTERNS); IPR007087 (PROSITE_PROFILES); IPR007087 (PROSITE_PROFILES); IPR007087 (PROSITE_PROFILES); IPR007087 (PROSITE_PROFILES); SSF57667 (SUPERFAMILY); SSF57667 (SUPERFAMILY)
675	235188_length_374_cvg_3.0_tip_1_2	9	hemicentin-2	124	1.26E-23	63.00%	0.109	IPR000742 (SMART); IPR001881 (SMART); G3DSA:2.10.25.10 (GENE3D); G3DSA:2.10.25.10 (GENE3D); IPR026823 (PFAM); PTHR19897:SF158 (PANTHER); PTHR19897 (PANTHER); IPR000152 (PROSITE_PATTERNS); IPR018097 (PROSITE_PATTERNS); IPR013032 (PROSITE_PATTERNS); IPR000742 (PROSITE_PROFILES); SSF57196 (SUPERFAMILY)
673	235044_length_374_cvg_3.0_tip_1_0	11	epidermal growth factor receptor-like	125	1.18E-37	66.20%	0.104	IPR006212 (SMART); G3DSA:2.10.220.10 (GENE3D); IPR009030 (SUPERFAMILY)
674	235134_length_374_cvg_4.0_tip_1_4	14	reelin-like isoform x4	125	8.08E-42	63.40%	0.105	PTHR11841 (PANTHER)
676	235232_length_374_cvg_2.7_tip_1_3	11	chondroitin sulfate	125	1.33E-43	74.30%	0.165	IPR029044 (G3DSA:3.90.550.GENE3D); IPR008428 (PFAM); PTHR12369:SF11 (PANTHER); PTHR12369 (PANTHER); IPR029044 (SUPERFAMILY)
677	235398_length_375_cvg_8.0_tip_1_3	36	zinc finger protein 3-like isoform x1	125	3.43E-17	68.80%	0.117	IPR015880 (SMART); IPR013087 (G3DSA:3.30.160.GENE3D); IPR013087 (G3DSA:3.30.160.GENE3D); PF13465 (PFAM); IPR013087 (G3DSA:3.30.160.GENE3D); PTHR24409 (PANTHER); IPR007087 (PROSITE_PATTERNS); IPR007087 (PROSITE_PATTERNS); IPR007087 (PROSITE_PROFILES); IPR007087 (PROSITE_PROFILES); SSF57667 (SUPERFAMILY)
678	235448_length_375_cvg_2.0_tip_1_4	10	dual specificity testis-specific protein kinase 2	125	3.36E-57	83.70%	0.15	IPR002290 (SMART); G3DSA:1.10.510.10 (GENE3D); IPR001245 (PFAM); PTHR23257 (PANTHER); PTHR23257:SF324 (PANTHER); IPR008266 (PROSITE_PATTERNS); IPR000719 (PROSITE_PROFILES); IPR011009 (SUPERFAMILY)
679	235462_length_375_cvg_5.7_tip_1_2	35	va52_dolma ame: full=venom allergen ame: full=allergen dol m v-a ame: full=antigen 5 form 2 short=ag5-2 ame: full=cysteine-rich venom protein short=crvp ame: allergen=dol m flags: precursor	125	1.26E-06	56.40%	0.31	IPR014044 (G3DSA:3.40.33.GENE3D)
680	235512_length_375_cvg_2.7_tip_1_1	10	zinc finger protein	125	2.37E-22	58.50%	0.1	IPR015880 (SMART); IPR013087 (G3DSA:3.30.160.GENE3D); PF13894 (PFAM); IPR013087 (G3DSA:3.30.160.GENE3D); PF13465 (PFAM); IPR007087 (PFAM); PTHR24409:SF14 (PANTHER); PTHR24409 (PANTHER); IPR007087 (PROSITE_PATTERNS); IPR007087 (PROSITE_PATTERNS); IPR007087 (PROSITE_PATTERNS); IPR007087 (PROSITE_PROFILES); IPR007087 (PROSITE_PROFILES); IPR007087 (PROSITE_PROFILES); IPR007087 (PROSITE_PROFILES); SSF57667 (SUPERFAMILY); SSF57667 (SUPERFAMILY)
681	235564_length_376_cvg_5.4_tip_1_1	21	zinc finger protein zpr1	125	3.25E-48	77.10%	0.1	IPR004457 (SMART); IPR004457 (TIGRFAM); IPR004457 (PFAM); PTHR10876 (PANTHER); PTHR10876:SF0 (PANTHER)
682	235900_length_377_cvg_3.5_tip_1_2	19	trypsin-1-like isoform x2	125	6.54E-27	69.70%	0.33	IPR001254 (SMART); IPR001254 (PFAM); G3DSA:2.40.10.10 (GENE3D); PTHR24256 (PANTHER); IPR018114 (PROSITE_PATTERNS); IPR001254 (PROSITE_PROFILES); IPR009003 (SUPERFAMILY)
683	236088_length_377_cvg_3.0_tip_1_2	12	limbic system-associated membrane protein	125	4.88E-42	69.30%	0.119	IPR003598 (SMART); IPR013783 (G3DSA:2.60.40.GENE3D); PF13895 (PFAM); IPR013783 (G3DSA:2.60.40.GENE3D); PTHR19831 (PANTHER); PTHR19831:SF47 (PANTHER); IPR007110 (PROSITE_PROFILES); SSF48726 (SUPERFAMILY); SSF48726 (SUPERFAMILY)
684	236220_length_378_cvg_3.0_tip_1_4	13	wd sam and u-box domain-containing protein 1-like isoform x1	126	1.28E-27	61.80%	0.133	IPR001680 (SMART); IPR001680 (PFAM); IPR015943 (G3DSA:2.130.10.GENE3D); PTHR22847 (PANTHER); IPR019775 (PROSITE_PATTERNS); IPR017986 (PROSITE_PROFILES); IPR001680 (PROSITE_PROFILES); IPR017986 (SUPERFAMILY)
686	236576_length_379_cvg_2.0_tip_1_5	5	integrin alpha-ps1 isoform x2	126	1.43E-50	79.90%	0.119	IPR000413 (PRINTS); IPR013519 (SMART); G3DSA:2.130.10.130 (GENE3D); IPR013517 (PFAM); PTHR23220:SF77 (PANTHER); PTHR23220 (PANTHER); IPR013519 (PROSITE_PROFILES); IPR013519 (PROSITE_PROFILES); SSF69318 (SUPERFAMILY)
685	236356_length_379_cvg_3.0_tip_1_3	12	complement c1q tumor necrosis factor-related protein 4	127	1.46E-11	51.00%	0.129	IPR001073 (SMART); IPR001073 (PFAM); IPR008983 (G3DSA:2.60.120.GENE3D); PTHR22923 (PANTHER); IPR001073 (PROSITE_PROFILES); IPR008983 (SUPERFAMILY)
687	236904_length_381_cvg_5.0_tip_1_3	19	heat shock protein partial	127	9.54E-78	96.10%	0.113	G3DSA:3.30.420.40 (GENE3D); IPR013126 (PFAM); G3DSA:3.90.640.10 (GENE3D); G3DSA:3.30.420.40 (GENE3D); PTHR19375:SF142 (PANTHER); PTHR19375 (PANTHER); IPR018181 (PROSITE_PATTERNS); SSF53067 (SUPERFAMILY)

688	237056_length_381_cvg_5.0_tip_1_3	26	tyrosine-protein phosphatase 69d	127	4.04E-49	80.40%	0.138	IPR003595 (SMART); IPR029021 (G3DSA:3.90.190.GENE3D); IPR000242 (PFAM); IPR029021 (G3DSA:3.90.190.GENE3D); PTHR19134:SF277 (PANTHER); PTHR19134 (PANTHER); IPR000387 (PROSITE_PROFILES); IPR000242 (PROSITE_PROFILES); IPR029021 (SUPERFAMILY)
689	237070_length_381_cvg_2.0_tip_1_2	9	thromboxane a synthase-like protein	127	2.11E-39	69.30%	0.171	IPR002401 (PRINTS); IPR001128 (PFAM); IPR001128 (G3DSA:1.10.630.GENE3D); PTHR24292 (PANTHER); PTHR24292:SF24 (PANTHER); CYTOPLASMIC_DOMAIN (PHOBIUS); TRANSMEMBRANE (PHOBIUS); NON_CYTOPLASMIC_DOMAIN (PHOBIUS); IPR001128 (SUPERFAMILY); TMhelix (TMHMM)
690	237278_length_382_cvg_3.0_tip_1_2	13	polypeptide n-acetylgalactosaminyltransferase 5 isoform x1	127	3.47E-40	75.30%	0.101	PTHR11675:SF21 (PANTHER); PTHR11675 (PANTHER)
691	237386_length_383_cvg_8.6_tip_1_5	32	dnaj protein homolog 1-like	127	2.39E-35	96.10%	0.147	IPR001623 (PRINTS); IPR001623 (SMART); IPR001623 (G3DSA:1.10.287.GENE3D); IPR001623 (PFAM); PTHR24077 (PANTHER); IPR018253 (PROSITE_PATTERNS); IPR001623 (PROSITE_PROFILES); IPR001623 (SUPERFAMILY)
692	237414_length_383_cvg_3.0_tip_1_0	13	retinoid-inducible serine carboxypeptidase	128	4.84E-32	68.20%	0.114	IPR029058 (G3DSA:3.40.50.GENE3D); IPR001563 (PFAM); PTHR11802:SF30 (PANTHER); IPR001563 (PANTHER); IPR029058 (SUPERFAMILY)
693	237622_length_383_cvg_4.0_tip_1_3	18	mam and ldl-receptor class a domain-containing protein 1-like	128	1.27E-35	63.30%	0.114	IPR000998 (PFAM); PTHR23282 (PANTHER); IPR000998 (PROSITE_PROFILES); IPR013320 (SUPERFAMILY)
694	237654_length_384_cvg_4.0_tip_1_2	16	epidermal growth factor receptor	128	4.06E-73	94.00%	0.117	IPR020635 (SMART); G3DSA:1.10.510.10 (GENE3D); IPR001245 (PFAM); G3DSA:3.30.200.20 (GENE3D); PTHR24416:SF95 (PANTHER); PTHR24416 (PANTHER); IPR017441 (PROSITE_PATTERNS); NON_CYTOPLASMIC_DOMAIN (PHOBIUS); CYTOPLASMIC_DOMAIN (PHOBIUS); TRANSMEMBRANE (PHOBIUS); IPR000719 (PROSITE_PROFILES); IPR011009 (SUPERFAMILY)
695	237700_length_384_cvg_2.0_tip_1_4	9	zinc finger protein 665-like	128	8.24E-48	72.90%	0.1	IPR015880 (SMART); PF13465 (PFAM); IPR013087 (G3DSA:3.30.160.GENE3D); IPR013087 (G3DSA:3.30.160.GENE3D); IPR013087 (G3DSA:3.30.160.GENE3D); PTHR24409 (PANTHER); IPR007087 (PROSITE_PATTERNS); IPR007087 (PROSITE_PATTERNS); IPR007087 (PROSITE_PATTERNS); IPR007087 (PROSITE_PATTERNS); IPR007087 (PROSITE_PROFILES); IPR007087 (PROSITE_PROFILES); IPR007087 (PROSITE_PROFILES); IPR007087 (PROSITE_PROFILES); SSF57667 (SUPERFAMILY); SSF57667 (SUPERFAMILY)
696	237708_length_384_cvg_77.3_tip_1_0	768	hypothetical protein BRAFLDRAFT_77846	128	8.30E-22	62.90%	0.426 Y	IPR013781 (G3DSA:3.20.20.GENE3D); PTHR31308 (PANTHER); NON_CYTOPLASMIC_DOMAIN (PHOBIUS); TRANSMEMBRANE (PHOBIUS); CYTOPLASMIC_DOMAIN (PHOBIUS); SignalP-TM (SIGNALP_GRAM_POSITIVE); IPR017853 (SUPERFAMILY); TMhelix (TMHMM)
697	238136_length_386_cvg_5.6_tip_1_5	24	protein disulfide-isomerase a6	128	6.37E-57	80.90%	0.117	IPR012336 (G3DSA:3.40.30.GENE3D); IPR013766 (PFAM); PTHR18929:SF38 (PANTHER); PTHR18929 (PANTHER); IPR012336 (SUPERFAMILY)
698	238286_length_386_cvg_3.1_tip_1_2	11	collagen alpha-1 chain	128	2.09E-18	61.80%	0.1	IPR008160 (PFAM); PTHR24023 (PANTHER); PTHR24023:SF379 (PANTHER)
699	238286_length_386_cvg_3.1_tip_1_4	11	collagen-like protein	129	3.74E-12	47.80%	0.165	no IPS match
700	238448_length_387_cvg_2.0_tip_1_2	6	low-density lipoprotein receptor-related protein 2	129	6.98E-38	71.80%	0.103	IPR001881 (SMART); G3DSA:2.10.25.10 (GENE3D); IPR011042 (G3DSA:2.120.10.GENE3D); IPR001881 (PFAM); PTHR10529 (PANTHER); IPR018097 (PROSITE_PATTERNS); SSF57196 (SUPERFAMILY)
703	239032_length_389_cvg_10.8_tip_1_2	35	hemagglutinin proteinase	129	1.99E-15	50.30%	0.108	IPR013856 (G3DSA:3.10.170.GENE3D); IPR013856 (PFAM)
704	239156_length_389_cvg_4.0_tip_1_5	19	senseless- isoform a	129	1.16E-19	53.10%	0.101	IPR015880 (SMART); PF13912 (PFAM); IPR013087 (G3DSA:3.30.160.GENE3D); IPR013087 (G3DSA:3.30.160.GENE3D); PF13465 (PFAM); PTHR24409 (PANTHER); IPR007087 (PROSITE_PATTERNS); IPR007087 (PROSITE_PATTERNS); IPR007087 (PROSITE_PATTERNS); IPR007087 (PROSITE_PATTERNS); IPR007087 (PROSITE_PROFILES); IPR007087 (PROSITE_PROFILES); IPR007087 (PROSITE_PROFILES); SSF57667 (SUPERFAMILY); SSF57667 (SUPERFAMILY)
701	238796_length_388_cvg_2.2_tip_1_0	11	vascular non-inflammatory molecule 3-like	130	3.68E-13	54.00%	0.105	IPR003010 (G3DSA:3.60.110.GENE3D); IPR003010 (PFAM); IPR012101 (PANTHER); IPR003010 (PROSITE_PROFILES); IPR003010 (SUPERFAMILY)
702	239030_length_389_cvg_2.0_tip_1_0	9	trypsin- partial	130	5.65E-40	65.50%	0.158	IPR001254 (SMART); G3DSA:2.40.10.10 (GENE3D); IPR001254 (PFAM); G3DSA:2.40.10.10 (GENE3D); PTHR24258 (PANTHER); PTHR24258:SF4 (PANTHER); IPR001254 (PROSITE_PROFILES); IPR009003 (SUPERFAMILY)
705	239264_length_390_cvg_5.5_tip_1_2	21	lipase 3-like	130	5.95E-35	69.10%	0.094	IPR029058 (G3DSA:3.40.50.GENE3D); PTHR11005:SF6 (PANTHER); PTHR11005 (PANTHER); IPR029058 (SUPERFAMILY)
706	239272_length_390_cvg_2.0_tip_1_1	10	very low-density lipoprotein partial	130	6.12E-12	48.70%	0.108	IPR002172 (PRINTS); IPR002172 (SMART); IPR002172 (G3DSA:4.10.400.GENE3D); IPR002172 (G3DSA:4.10.400.GENE3D); IPR002172 (G3DSA:4.10.400.GENE3D); PTHR10529 (PANTHER); IPR002172 (PROSITE_PROFILES); IPR002172 (PROSITE_PROFILES); IPR002172 (SUPERFAMILY); IPR002172 (SUPERFAMILY)

707	239390_length_390_cvg_2.0_tip_1_2	7	tyrosine-protein phosphatase 69d-like	130	1.73E-46	76.50%	0.218	IPR029021 (G3DSA:3.90.190.GENE3D); IPR000242 (PFAM); PTHR19134 (PANTHER); PTHR19134:SF261 (PANTHER); IPR000387 (PROSITE_PROFILES); IPR000242 (PROSITE_PROFILES); IPR029021 (SUPERFAMILY)
708	239480_length_390_cvg_2.8_tip_1_3	11	granzyme c	130	1.24E-23	61.20%	0.104	IPR001254 (SMART); IPR001254 (PFAM); G3DSA:2.40.10.10 (GENE3D); PTHR24268 (PANTHER); IPR018114 (PROSITE_PATTERNS); IPR001254 (PROSITE_PROFILES); IPR009003 (SUPERFAMILY)
711	239736_length_391_cvg_3.0_tip_1_5	11	apolipoprotein d-like	130	1.64E-08	61.60%	0.141	IPR012674 (G3DSA:2.40.128.GENE3D); PTHR10612 (PANTHER); NON_CYTOPLASMIC_DOMAIN (PHOBIUS); CYTOPLASMIC_DOMAIN (PHOBIUS); TRANSMEMBRANE (PHOBIUS); IPR011038 (SUPERFAMILY); TMhelix (TMHMM)
713	239962_length_392_cvg_2.0_tip_1_2	7	uncharacterized protein ZBAI_02902	130	3.61E-11	61.90%	0.097	IPR015880 (SMART); IPR013087 (G3DSA:3.30.160.GENE3D); IPR013087 (G3DSA:3.30.160.GENE3D); PF13465 (PFAM); PTHR24409 (PANTHER); PTHR24409:SF14 (PANTHER); IPR007087 (PROSITE_PATTERNS); IPR007087 (PROSITE_PROFILES); IPR007087 (PROSITE_PROFILES); SSF57667 (SUPERFAMILY)
709	239524_length_391_cvg_2.2_tip_1_0	13	prolyl 3-hydroxylase 2	131	1.21E-23	75.00%	0.107	Coil (COILS); PTHR14049:SF9 (PANTHER); PTHR14049 (PANTHER)
710	239566_length_391_cvg_4.0_tip_1_0	17	maguk p55 subfamily member 5	131	4.98E-72	92.00%	0.107	Coil (COILS); Coil (COILS); IPR008145 (SMART); IPR027417 (G3DSA:3.40.50.GENE3D); IPR008145 (PFAM); PTHR23122 (PANTHER); PTHR23122:SF32 (PANTHER); IPR008144 (PROSITE_PROFILES); IPR027417 (SUPERFAMILY)
712	239872_length_392_cvg_2.0_tip_1_4	10	lipoprotein receptor	131	3.35E-23	68.30%	0.325	IPR000832 (PFAM); PTHR12011:SF260 (PANTHER); PTHR12011 (PANTHER); TRANSMEMBRANE (PHOBIUS); TRANSMEMBRANE (PHOBIUS); CYTOPLASMIC_DOMAIN (PHOBIUS); CYTOPLASMIC_DOMAIN (PHOBIUS); NON_CYTOPLASMIC_DOMAIN (PHOBIUS); TMhelix (TMHMM); TMhelix (TMHMM)
714	240232_length_393_cvg_10.0_tip_1_0	44	upf0669 protein c6orf120 homolog	131	3.73E-38	66.40%	0.106	PTHR31703 (PANTHER)
715	240286_length_393_cvg_2.0_tip_1_5	11	heat shock 70 kda protein 14	131	6.68E-50	73.40%	0.225	G3DSA:3.30.420.40 (GENE3D); IPR013126 (PFAM); G3DSA:3.90.640.10 (GENE3D); PTHR19375:SF158 (PANTHER); PTHR19375 (PANTHER); SSF53067 (SUPERFAMILY); TMhelix (TMHMM)
716	240302_length_394_cvg_3.0_tip_1_5	14	achain nmr solution dynamics and binding properties of the kringle iv type 8 module of apolipoprotein	131	7.52E-31	64.30%	0.709 Y	PR00018 (PRINTS); IPR000001 (SMART); G3DSA:2.40.20.10 (GENE3D); IPR000001 (PFAM); PTHR24259 (PANTHER); IPR018056 (PROSITE_PATTERNS); SIGNAL_PEPTIDE_C_REGION (PHOBIUS); SIGNAL_PEPTIDE_N_REGION (PHOBIUS); SIGNAL_PEPTIDE_H_REGION (PHOBIUS); SIGNAL_PEPTIDE (PHOBIUS); NON_CYTOPLASMIC_DOMAIN (PHOBIUS); IPR000001 (PROSITE_PROFILES); SignalP-noTM (SIGNALP_EUK); IPR013806 (SUPERFAMILY)
717	240326_length_394_cvg_2.0_tip_1_1	8	transforming growth factor-beta-induced protein ig-h3-like isoform x6	131	6.59E-43	68.10%	0.193	IPR000782 (SMART); IPR000782 (PFAM); IPR000782 (G3DSA:2.30.180.GENE3D); PTHR10900 (PANTHER); IPR000782 (PROSITE_PROFILES); IPR000782 (SUPERFAMILY)
719	240442_length_394_cvg_2.3_tip_1_1	8	polypeptide n-acetylgalactosaminyltransferase partial	131	5.58E-68	83.90%	0.104	G3DSA:2.80.10.50 (GENE3D); IPR000772 (PFAM); PTHR11675 (PANTHER); PTHR11675:SF24 (PANTHER); IPR000772 (PROSITE_PROFILES); IPR000772 (SUPERFAMILY); IPR029044 (SUPERFAMILY)
722	240666_length_395_cvg_2.0_tip_1_5	10	calcium-activated chloride channel regulator 2	131	1.07E-17	52.20%	0.101	IPR013783 (G3DSA:2.60.40.GENE3D); PTHR10579 (PANTHER); PTHR10579:SF43 (PANTHER)
718	240360_length_394_cvg_2.0_tip_1_0	8	attractin-like protein 1 isoform x1	132	1.28E-41	73.70%	0.149	G3DSA:2.10.25.10 (GENE3D); G3DSA:2.10.25.10 (GENE3D); IPR013111 (PFAM); IPR000859 (G3DSA:2.60.120.GENE3D); PTHR10574 (PANTHER); PTHR10574:SF3 (PANTHER); IPR013032 (PROSITE_PATTERNS); IPR013032 (PROSITE_PATTERNS); IPR000742 (PROSITE_PROFILES)
720	240582_length_395_cvg_11.9_tip_1_3	31	low quality protein: mucin-2-like	132	5.77E-07	44.29%	0.119	PR01217 (PRINTS); IPR001846 (PFAM); IPR001846 (PROSITE_PROFILES)
721	240612_length_395_cvg_3.0_tip_1_1	12	mlrp1_acrmi ame: full=mam and ldl-receptor class a domain-containing protein 1 ame: full=skeletal organic matrix mam and ldl-receptor 1 short=som mam and ldl-receptor partial	132	1.74E-16	64.40%	0.322	IPR000998 (PFAM); PTHR23282 (PANTHER); PTHR23282:SF75 (PANTHER); CYTOPLASMIC_DOMAIN (PHOBIUS); TRANSMEMBRANE (PHOBIUS); NON_CYTOPLASMIC_DOMAIN (PHOBIUS); IPR000998 (PROSITE_PROFILES); IPR000998 (PROSITE_PROFILES); IPR013320 (SUPERFAMILY); TMhelix (TMHMM)
723	240740_length_395_cvg_7.3_tip_1_3	35	ectonucleoside triphosphate diphosphohydrolase 5 isoform x2	132	5.93E-22	69.50%	0.14	IPR000407 (PFAM); IPR000407 (PANTHER); PTHR11782:SF30 (PANTHER); CYTOPLASMIC_DOMAIN (PHOBIUS); TRANSMEMBRANE (PHOBIUS); NON_CYTOPLASMIC_DOMAIN (PHOBIUS); TMhelix (TMHMM)
724	240796_length_396_cvg_2.0_tip_1_5	7	neural cell adhesion molecule 1 isoform 2	132	1.67E-12	49.20%	0.143	IPR013783 (G3DSA:2.60.40.GENE3D); PF13895 (PFAM); IPR013098 (PFAM); IPR013783 (G3DSA:2.60.40.GENE3D); PTHR10489 (PANTHER); IPR007110 (PROSITE_PROFILES); IPR007110 (PROSITE_PROFILES); SSF48726 (SUPERFAMILY); SSF48726 (SUPERFAMILY)
725	240826_length_396_cvg_27.0_tip_1_5	98	twitchin isoform x5	132	9.73E-44	76.00%	0.098	IPR013783 (G3DSA:2.60.40.GENE3D); IPR013783 (G3DSA:2.60.40.GENE3D); IPR013098 (PFAM); PTHR19897:SF155 (PANTHER); PTHR19897 (PANTHER); IPR007110 (PROSITE_PROFILES); SSF48726 (SUPERFAMILY); SSF48726 (SUPERFAMILY)

726	240874_length_396_cvg_3.0_tip_1_1	15	cytokine receptor	132	4.13E-23	59.50%	0.142	IPR013783 (G3DSA:2.60.40.GENE3D); IPR003961 (PFAM); PTHR10489 (PANTHER); IPR003961 (PROSITE_PROFILES); IPR003961 (PROSITE_PROFILES); IPR003961 (SUPERFAMILY)
728	241066_length_397_cvg_2.0_tip_1_2	10	wnt-receptor frizzled-like protein variant 2	132	4.31E-75	88.60%	0.111	IPR020067 (SMART); IPR020067 (G3DSA:1.10.2000.GENE3D); IPR020067 (PFAM); IPR026561 (PTHR11309:PANTHER); IPR015526 (PANTHER); IPR020067 (PROSITE_PROFILES); IPR020067 (SUPERFAMILY)
730	241142_length_397_cvg_111.6_tip_1_2	6720	dermatopontin 2	132	1.93E-16	60.80%	0.297	PF14704 (PFAM); IPR026645 (PANTHER); CYTOPLASMIC_DOMAIN (PHOBIUS); TRANSMEMBRANE (PHOBIUS); NON_CYTOPLASMIC_DOMAIN (PHOBIUS); TMhelix (TMHMM)
731	241170_length_397_cvg_3.0_tip_1_5	12	meprin a	132	1.43E-24	53.30%	0.104	G3DSA:2.170.300.10 (GENE3D); IPR024079 (G3DSA:3.40.390.GENE3D); IPR001506 (PFAM); IPR000859 (G3DSA:2.60.120.GENE3D); PTHR10127 (PANTHER); PTHR10127:SF580 (PANTHER); IPR000859 (PROSITE_PROFILES)
732	241208_length_397_cvg_2.5_tip_1_1	15	sco-spondin isoform x1	132	4.03E-17	52.80%	0.101	IPR002172 (PRINTS); IPR002172 (SMART); IPR002172 (G3DSA:4.10.400.GENE3D); IPR002172 (G3DSA:4.10.400.GENE3D); IPR002172 (PFAM); G3DSA:4.10.1220.10 (GENE3D); PTHR10529 (PANTHER); PTHR10529:SF208 (PANTHER); IPR002172 (PROSITE_PROFILES); IPR002172 (PROSITE_PROFILES); IPR002172 (SUPERFAMILY); IPR002172 (SUPERFAMILY); IPR002172 (SUPERFAMILY)
733	241226_length_397_cvg_2.0_tip_1_1	12	ectonucleoside triphosphate diphosphohydrolase 7	132	8.38E-23	72.60%	0.314	IPR000407 (PFAM); IPR000407 (PANTHER); PTHR11782:SF37 (PANTHER); SIGNAL_PEPTIDE_N_REGION (PHOBIUS); SIGNAL_PEPTIDE (PHOBIUS); SIGNAL_PEPTIDE_H_REGION (PHOBIUS); SIGNAL_PEPTIDE_C_REGION (PHOBIUS); NON_CYTOPLASMIC_DOMAIN (PHOBIUS); TMhelix (TMHMM)
727	241028_length_397_cvg_2.0_tip_1_0	6	down syndrome cell adhesion molecule-like protein dscam2	133	2.01E-62	88.00%	0.106	IPR003598 (SMART); IPR003599 (SMART); IPR013783 (G3DSA:2.60.40.GENE3D); IPR013098 (PFAM); PTHR10489:SF567 (PANTHER); PTHR10489 (PANTHER); IPR007110 (PROSITE_PROFILES); SSF48726 (SUPERFAMILY)
729	241076_length_397_cvg_9.1_tip_1_0	50	kallikrein-14- partial	133	2.26E-26	68.70%	0.102	IPR001254 (SMART); G3DSA:2.40.10.10 (GENE3D); IPR001254 (PFAM); PTHR24256 (PANTHER); IPR018114 (PROSITE_PATTERNS); CYTOPLASMIC_DOMAIN (PHOBIUS); TRANSMEMBRANE (PHOBIUS); NON_CYTOPLASMIC_DOMAIN (PHOBIUS); IPR001254 (PROSITE_PROFILES); IPR009003 (SUPERFAMILY); TMhelix (TMHMM)
734	241466_length_398_cvg_3.0_tip_1_1	12	15-hydroxyprostaglandin dehydrogenase	133	4.00E-64	78.10%	0.112	IPR002198 (PRINTS); IPR002424 (PRINTS); IPR016040 (G3DSA:3.40.50.GENE3D); IPR002198 (PFAM); PTHR24310 (PANTHER); SSF51735 (SUPERFAMILY)
735	241486_length_398_cvg_3.3_tip_1_4	15	peptidyl-prolyl cis-trans isomerase cwc27 homolog	133	2.84E-76	90.00%	0.16	IPR002130 (PRINTS); IPR002130 (PFAM); IPR029000 (G3DSA:2.40.100.GENE3D); IPR024936 (PIRSF); IPR024936 (PANTHER); PTHR11071:SF152 (PANTHER); IPR020892 (PROSITE_PATTERNS); IPR002130 (PROSITE_PROFILES); IPR029000 (SUPERFAMILY)
736	241638_length_399_cvg_3.5_tip_1_2	13	ubiquitin thioesterase partial	133	7.40E-19	55.10%	0.288	no IPS match
737	241706_length_399_cvg_5.8_tip_1_0	27	glucose dehydrogenase	133	2.09E-17	92.20%	0.122	G3DSA:3.50.50.60 (GENE3D); IPR007867 (PFAM); PTHR11552 (PANTHER); PTHR11552:SF90 (PANTHER); SSF51905 (SUPERFAMILY)
739	241854_length_400_cvg_4.0_tip_1_4	17	latrophilin cirl-like isoform x1	133	3.19E-12	54.00%	0.126	IPR000203 (PFAM); PTHR12011 (PANTHER); IPR000203 (PROSITE_PROFILES)
738	241836_length_400_cvg_2.0_tip_1_3	7	neurotrypsin isoform x1	134	2.99E-45	70.50%	0.109	IPR001190 (PRINTS); IPR017448 (SMART); IPR001190 (PFAM); IPR017448 (G3DSA:3.10.250.GENE3D); IPR002172 (G3DSA:4.10.400.GENE3D); PTHR19331 (PANTHER); IPR001190 (PROSITE_PROFILES); IPR017448 (SUPERFAMILY)
740	242326_length_402_cvg_3.0_tip_1_3	13	gastrula zinc finger protein - partial	134	3.39E-39	66.90%	0.106	IPR015880 (SMART); IPR013087 (G3DSA:3.30.160.GENE3D); IPR013087 (G3DSA:3.30.160.GENE3D); IPR013087 (G3DSA:3.30.160.GENE3D); IPR013087 (G3DSA:3.30.160.GENE3D); PF13465 (PFAM); PTHR24409 (PANTHER); IPR007087 (PROSITE_PATTERNS); IPR007087 (PROSITE_PATTERNS); IPR007087 (PROSITE_PATTERNS); IPR007087 (PROSITE_PATTERNS); IPR007087 (PROSITE_PROFILES); IPR007087 (PROSITE_PROFILES); IPR007087 (PROSITE_PROFILES); IPR007087 (PROSITE_PROFILES); SSF57667 (SUPERFAMILY); SSF57667 (SUPERFAMILY)
741	242444_length_402_cvg_2.0_tip_1_5	9	gastrula zinc finger	134	4.15E-40	64.10%	0.13	IPR015880 (SMART); IPR013087 (G3DSA:3.30.160.GENE3D); IPR013087 (G3DSA:3.30.160.GENE3D); PF13465 (PFAM); IPR013087 (G3DSA:3.30.160.GENE3D); PTHR24409 (PANTHER); IPR007087 (PROSITE_PATTERNS); IPR007087 (PROSITE_PATTERNS); IPR007087 (PROSITE_PATTERNS); IPR007087 (PROSITE_PATTERNS); IPR007087 (PROSITE_PROFILES); IPR007087 (PROSITE_PROFILES); IPR007087 (PROSITE_PROFILES); IPR007087 (PROSITE_PROFILES); SSF57667 (SUPERFAMILY); SSF57667 (SUPERFAMILY); SSF57667 (SUPERFAMILY)

742	242546_length_403_cvg_2.0_tip_1_5	12	eph receptor tyrosine	134	1.84E-84	92.30%	0.273	IPR001245 (PRINTS); IPR020635 (SMART); IPR013761 (G3DSA:1.10.150.GENE3D); G3DSA:1.10.510.10 (GENE3D); IPR001245 (PFAM); PTHR24416 (PANTHER); PTHR24416:SF263 (PANTHER); IPR001660 (PROSITE_PROFILES); IPR000719 (PROSITE_PROFILES); IPR011009 (SUPERFAMILY)
744	242696_length_403_cvg_3.0_tip_1_4	15	translocation protein sec63 homolog	134	6.44E-58	77.90%	0.125	IPR001623 (PRINTS); IPR001623 (SMART); IPR001623 (G3DSA:1.10.287.GENE3D); IPR001623 (PFAM); IPR027137 (PANTHER); PTHR24075:SF0 (PANTHER); CYTOPLASMIC_DOMAIN (PHOBIUS); TRANSMEMBRANE (PHOBIUS); NON_CYTOPLASMIC_DOMAIN (PHOBIUS); IPR001623 (PROSITE_PROFILES); IPR001623 (SUPERFAMILY); TMhelix (TMHMM)
745	242768_length_404_cvg_4.0_tip_1_2	18	calbindin isoform a	134	7.69E-85	93.50%	0.104	IPR002048 (SMART); IPR011992 (G3DSA:1.10.238.GENE3D); IPR011992 (G3DSA:1.10.238.GENE3D); IPR011992 (PFAM); PTHR19972 (PANTHER); IPR018247 (PROSITE_PATTERNS); IPR018247 (PROSITE_PATTERNS); IPR002048 (PROSITE_PROFILES); IPR002048 (PROSITE_PROFILES); IPR002048 (PROSITE_PROFILES); SSF47473 (SUPERFAMILY)
743	242676_length_403_cvg_3.0_tip_1_0	13	retinol dehydrogenase 13	135	1.51E-38	74.40%	0.166	IPR002347 (PRINTS); IPR002198 (PFAM); IPR016040 (G3DSA:3.40.50.GENE3D); PTHR24316:SF305 (PANTHER); PTHR24316 (PANTHER); TRANSMEMBRANE (PHOBIUS); CYTOPLASMIC_DOMAIN (PHOBIUS); NON_CYTOPLASMIC_DOMAIN (PHOBIUS); SSF51735 (SUPERFAMILY); TMhelix (TMHMM)
746	242790_length_404_cvg_4.0_tip_1_3	21	peptidyl-prolyl cis-trans isomerase h-like	135	2.42E-56	96.00%	0.1	IPR002130 (PRINTS); IPR029000 (G3DSA:2.40.100.GENE3D); IPR002130 (PFAM); PTHR11071:SF58 (PANTHER); IPR024936 (PANTHER); IPR002130 (PROSITE_PROFILES); IPR029000 (SUPERFAMILY)
747	242856_length_404_cvg_2.0_tip_1_0	10	gastrula zinc finger protein	135	2.77E-17	51.80%	0.102	IPR015880 (SMART); IPR013087 (G3DSA:3.30.160.GENE3D); PF13465 (PFAM); IPR013087 (G3DSA:3.30.160.GENE3D); PF13894 (PFAM); PTHR24409 (PANTHER); IPR007087 (PROSITE_PATTERNS); IPR007087 (PROSITE_PATTERNS); IPR007087 (PROSITE_PROFILES); IPR007087 (PROSITE_PROFILES); SSF57667 (SUPERFAMILY); SSF57667 (SUPERFAMILY)
748	242940_length_405_cvg_76.9_tip_1_2	614	epidermal retinol dehydrogenase partial	135	9.51E-22	71.10%	0.117	IPR016040 (G3DSA:3.40.50.GENE3D); PTHR24316:SF246 (PANTHER); PTHR24316 (PANTHER); NON_CYTOPLASMIC_DOMAIN (PHOBIUS); TRANSMEMBRANE (PHOBIUS); CYTOPLASMIC_DOMAIN (PHOBIUS); SSF51735 (SUPERFAMILY); TMhelix (TMHMM); TMhelix (TMHMM)
749	242952_length_405_cvg_28.0_tip_1_3	120	poly -specific endoribonuclease homolog	135	4.19E-20	62.50%	0.325	IPR018998 (PFAM); TRANSMEMBRANE (PHOBIUS); CYTOPLASMIC_DOMAIN (PHOBIUS); NON_CYTOPLASMIC_DOMAIN (PHOBIUS); SSF142877 (SUPERFAMILY); TMhelix (TMHMM)
750	243024_length_405_cvg_4.0_tip_1_3	20	dnaj homolog dnj-5	135	7.72E-67	85.30%	0.11	IPR001623 (PRINTS); IPR001623 (SMART); IPR001623 (PFAM); IPR001623 (G3DSA:1.10.287.GENE3D); PTHR24078 (PANTHER); PTHR24078:SF141 (PANTHER); IPR001623 (PROSITE_PROFILES); IPR001623 (SUPERFAMILY)
753	243376_length_406_cvg_5.0_tip_1_2	24	chemosensory protein	135	4.77E-49	77.70%	0.499 Y	IPR005055 (PFAM); IPR005055 (G3DSA:1.10.2080.GENE3D); PTHR11257:SF2 (PANTHER); IPR005055 (PANTHER); SIGNAL_PEPTIDE_C_REGION (PHOBIUS); NON_CYTOPLASMIC_DOMAIN (PHOBIUS); SIGNAL_PEPTIDE (PHOBIUS); SIGNAL_PEPTIDE_N_REGION (PHOBIUS); SIGNAL_PEPTIDE_H_REGION (PHOBIUS); IPR005055 (SUPERFAMILY)
754	243422_length_407_cvg_5.0_tip_1_2	24	carboxypeptidase n catalytic chain	135	4.46E-23	57.60%	0.269	G3DSA:3.40.630.10 (GENE3D); IPR014766 (G3DSA:2.60.40.GENE3D); PF13620 (PFAM); PTHR11532 (PANTHER); PTHR11532:SF50 (PANTHER); SSF53187 (SUPERFAMILY); IPR008969 (SUPERFAMILY)
751	243206_length_406_cvg_2.0_tip_1_0	7	---NA---	136			0.127	IPR008979 (G3DSA:2.60.120.GENE3D); IPR000421 (PFAM); IPR000421 (PROSITE_PATTERNS); IPR000421 (PROSITE_PROFILES); IPR008979 (SUPERFAMILY)
752	243346_length_406_cvg_3.0_tip_1_0	14	tyrosine-protein kinase hopscotch	136	5.86E-42	70.50%	0.117	IPR001245 (PRINTS); IPR020635 (SMART); IPR001245 (PFAM); G3DSA:1.10.510.10 (GENE3D); PTHR24418:SF216 (PANTHER); PTHR24418 (PANTHER); IPR000719 (PROSITE_PROFILES); IPR011009 (SUPERFAMILY)
755	243480_length_407_cvg_2.0_tip_1_1	8	isoform a	136	1.01E-45	67.30%	0.101	IPR005331 (PFAM); IPR027417 (G3DSA:3.40.50.GENE3D); IPR018011 (PANTHER); PTHR12137:SF30 (PANTHER)
756	243512_length_407_cvg_7.0_tip_1_1	28	cuticular protein	136	2.78E-30	75.40%	0.104	TMhelix (TMHMM)
757	243630_length_408_cvg_2.0_tip_1_1	11	laminin subunit gamma-1	136	2.36E-53	76.40%	0.109	IPR018031 (SMART); IPR000034 (PFAM); PTHR10574 (PANTHER); PTHR10574:SF231 (PANTHER); IPR000034 (PROSITE_PROFILES)
758	243634_length_408_cvg_2.2_tip_1_3	11	multicopper oxidase	136	9.66E-41	67.20%	0.103	IPR008972 (G3DSA:2.60.40.GENE3D); IPR008972 (G3DSA:2.60.40.GENE3D); IPR011707 (PFAM); PTHR11709:SF33 (PANTHER); PTHR11709 (PANTHER); IPR008972 (SUPERFAMILY)

759	243692_length_408_cvg_3.8_tip_1_4	16	down syndrome cell adhesion molecule-like partial	136	4.44E-55	79.10%	0.105	IPR003598 (SMART); IPR003599 (SMART); IPR013783 (G3DSA:2.60.40.GENE3D); IPR003961 (PFAM); IPR013098 (PFAM); IPR013783 (G3DSA:2.60.40.GENE3D); PTHR10489 (PANTHER); PTHR10489:SF567 (PANTHER); IPR007110 (PROSITE_PROFILES); IPR003961 (PROSITE_PROFILES); IPR003961 (SUPERFAMILY); SSF48726 (SUPERFAMILY)
760	243802_length_408_cvg_3.0_tip_1_5	17	receptor-type tyrosine-protein phosphatase kappa-like	136	2.29E-35	65.90%	0.108	IPR000242 (PRINTS); IPR000242 (SMART); IPR000242 (PFAM); IPR029021 (G3DSA:3.90.190.GENE3D); PTHR19134 (PANTHER); IPR000242 (PROSITE_PROFILES); IPR029021 (SUPERFAMILY)
761	244404_length_411_cvg_4.3_tip_1_0	22	matrix metalloproteinase-14 isoform x1	137	2.70E-18	57.00%	0.105	IPR018487 (SMART); IPR000585 (G3DSA:2.110.10.GENE3D); IPR018487 (PFAM); PTHR10201:SF131 (PANTHER); PTHR10201 (PANTHER); IPR018487 (PROSITE_PROFILES); IPR000585 (SUPERFAMILY)
762	244486_length_412_cvg_3.5_tip_1_2	16	protein phosphatase 1 regulatory subunit 16a isoform x3	137	2.62E-70	89.00%	0.12	IPR002110 (SMART); PF13857 (PFAM); IPR002110 (PFAM); IPR020683 (G3DSA:1.25.40.GENE3D); PTHR24186 (PANTHER); IPR002110 (PROSITE_PROFILES); IPR020683 (PROSITE_PROFILES); IPR002110 (PROSITE_PROFILES); IPR020683 (SUPERFAMILY)
765	244870_length_413_cvg_2.0_tip_1_5	9	death-associated protein kinase partial	137	6.79E-47	68.20%	0.196	IPR002110 (SMART); IPR020683 (PFAM); IPR020683 (G3DSA:1.25.40.GENE3D); IPR002110 (PFAM); IPR020683 (G3DSA:1.25.40.GENE3D); PTHR22964:SF46 (PANTHER); IPR020675 (PANTHER); IPR002110 (PROSITE_PROFILES); IPR002110 (PROSITE_PROFILES); IPR002110 (PROSITE_PROFILES); IPR002110 (PROSITE_PROFILES); IPR020683 (PROSITE_PROFILES); IPR020683 (SUPERFAMILY)
767	244928_length_413_cvg_2.0_tip_1_2	8	isoform c	137	7.35E-16	66.20%	0.102	IPR019149 (PFAM); PTHR13617 (PANTHER)
763	244754_length_413_cvg_17.9_tip_1_1	70	nidogen- partial	138	1.92E-15	51.00%	0.112	IPR000716 (SMART); IPR000716 (PFAM); IPR000716 (G3DSA:4.10.800.GENE3D); PTHR12352 (PANTHER); IPR000716 (PROSITE_PATTERNS); IPR000716 (PROSITE_PROFILES); IPR000716 (PROSITE_PROFILES); IPR000716 (SUPERFAMILY); IPR000716 (SUPERFAMILY)
764	244820_length_413_cvg_4.0_tip_1_0	16	adp-ribosylation factor-like protein 2	138	4.49E-26	89.20%	0.12	IPR006689 (PFAM); IPR027417 (G3DSA:3.40.50.GENE3D); PTHR11711:SF26 (PANTHER); PTHR11711 (PANTHER); IPR027417 (SUPERFAMILY)
766	244898_length_413_cvg_27.6_tip_1_0	159	inter-alpha-trypsin inhibitor heavy chain h3-like	138	3.81E-30	67.00%	0.105	PTHR10338:SF24 (PANTHER); PTHR10338 (PANTHER)
768	245048_length_414_cvg_3.3_tip_1_0	17	conserved hypothetical protein	138	8.11E-35	71.60%	0.107	IPR011030 (G3DSA:1.25.10.GENE3D); IPR015255 (PFAM); IPR001747 (PFAM); IPR015817 (G3DSA:2.20.50.GENE3D); PTHR23345 (PANTHER); PTHR23345:SF11 (PANTHER); IPR011030 (SUPERFAMILY); IPR015819 (SUPERFAMILY)
769	245114_length_414_cvg_2.0_tip_1_5	8	protein twisted gastrulation	138	3.82E-16	76.40%	0.169	IPR006761 (PFAM); PTHR12312:SF15 (PANTHER); IPR006761 (PANTHER)
772	245214_length_415_cvg_38.0_tip_1_2	189	cathepsin l	138	1.11E-37	89.20%	0.102	IPR000668 (PFAM); G3DSA:3.90.70.10 (GENE3D); PTHR12411:SF57 (PANTHER); IPR013128 (PANTHER); IPR025660 (PROSITE_PATTERNS); IPR025661 (PROSITE_PATTERNS); TRANSMEMBRANE (PHOBIUS); NON_CYTOPLASMIC_DOMAIN (PHOBIUS); TRANSMEMBRANE (PHOBIUS); CYTOPLASMIC_DOMAIN (PHOBIUS); CYTOPLASMIC_DOMAIN (PHOBIUS); SSF54001 (SUPERFAMILY); TMhelix (TMHMM); TMhelix (TMHMM)
773	245290_length_415_cvg_6.0_tip_1_2	23	laminin subunit gamma- partial	138	4.56E-28	62.70%	0.104	Coil (COILS); Coil (COILS)
775	245358_length_416_cvg_2.8_tip_1_5	14	plexin- partial	138	1.80E-66	84.50%	0.1	IPR015943 (G3DSA:2.130.10.GENE3D); IPR001627 (PFAM); PTHR22625:SF16 (PANTHER); PTHR22625 (PANTHER); IPR001627 (PROSITE_PROFILES); IPR001627 (SUPERFAMILY)
776	245384_length_416_cvg_2.0_tip_1_5	10	zinc finger protein 239- partial	138	5.33E-36	60.10%	0.105	IPR015880 (SMART); IPR013087 (G3DSA:3.30.160.GENE3D); IPR013087 (G3DSA:3.30.160.GENE3D); PF13465 (PFAM); IPR013087 (G3DSA:3.30.160.GENE3D); PTHR24409 (PANTHER); IPR007087 (PROSITE_PATTERNS); IPR007087 (PROSITE_PATTERNS); IPR007087 (PROSITE_PATTERNS); IPR007087 (PROSITE_PATTERNS); IPR007087 (PROSITE_PROFILES); IPR007087 (PROSITE_PROFILES); IPR007087 (PROSITE_PROFILES); IPR007087 (PROSITE_PROFILES); SSF57667 (SUPERFAMILY); SSF57667 (SUPERFAMILY); SSF57667 (SUPERFAMILY)
777	245440_length_416_cvg_2.0_tip_1_5	8	peptidyl-glycine alpha-amidating monooxygenase partial	138	8.46E-45	65.70%	0.183	IPR000720 (PRINTS); IPR014784 (G3DSA:2.60.120.GENE3D); PF03712 (PFAM); PTHR10680:SF13 (PANTHER); PTHR10680 (PANTHER); IPR014783 (PROSITE_PATTERNS); IPR008977 (SUPERFAMILY)
778	245454_length_416_cvg_2.0_tip_1_2	7	cytokine receptor	138	4.61E-38	72.70%	0.125	IPR013783 (G3DSA:2.60.40.GENE3D); IPR013783 (G3DSA:2.60.40.GENE3D); IPR003961 (PFAM); PTHR10489 (PANTHER); IPR003961 (PROSITE_PROFILES); IPR003961 (PROSITE_PROFILES); IPR003961 (SUPERFAMILY)

770	245158_length_415_cvg_2.0_tip_1_0	8	big brain	139	5.81E-45	73.00%	0.424 Y	IPR000425 (PRINTS); IPR000425 (PFAM); IPR023271 (G3DSA:1.20.1080.GENE3D); IPR000425 (PANTHER); PTHR19139:SF85 (PANTHER); IPR022357 (PROSITE_PATTERNS); NON_CYTOPLASMIC_DOMAIN (PHOBIUS); CYTOPLASMIC_DOMAIN (PHOBIUS); CYTOPLASMIC_DOMAIN (PHOBIUS); TRANSMEMBRANE (PHOBIUS); TRANSMEMBRANE (PHOBIUS); IPR023271 (SUPERFAMILY); TMhelix (TMHMM); TMhelix (TMHMM); TMhelix (TMHMM); TMhelix (TMHMM)
771	245160_length_415_cvg_3.0_tip_1_3	12	venom allergen 3-like	139	7.59E-24	51.80%	0.141	IPR001283 (PRINTS); IPR014044 (SMART); IPR014044 (PFAM); IPR014044 (G3DSA:3.40.33.GENE3D); IPR001283 (PANTHER); IPR014044 (SUPERFAMILY)
774	245294_length_415_cvg_73.7_tip_0_3	2392	otogelin- partial	139	3.86E-06	47.00%	0.117	no IPS match
779	245708_length_417_cvg_63.0_tip_0_0	594	kunitz bovine pancreatic trypsin inhibitor domain protein	139	1.29E-36	62.70%	0.102	IPR002223 (PRINTS); IPR002223 (SMART); IPR002223 (G3DSA:4.10.410.GENE3D); IPR002223 (G3DSA:4.10.410.GENE3D); IPR002223 (G3DSA:4.10.410.GENE3D); IPR002223 (PFAM); PTHR10083 (PANTHER); IPR020901 (PROSITE_PATTERNS); IPR020901 (PROSITE_PATTERNS); IPR002223 (PROSITE_PROFILES); IPR002223 (PROSITE_PROFILES); IPR002223 (SUPERFAMILY); IPR002223 (SUPERFAMILY)
780	245764_length_417_cvg_2.0_tip_1_5	10	zinc finger protein 45-like	139	2.69E-16	62.50%	0.15	IPR015880 (SMART); IPR007087 (PFAM); IPR013087 (G3DSA:3.30.160.GENE3D); PF13465 (PFAM); IPR013087 (G3DSA:3.30.160.GENE3D); IPR013087 (G3DSA:3.30.160.GENE3D); PTHR24409 (PANTHER); IPR007087 (PROSITE_PATTERNS); IPR007087 (PROSITE_PATTERNS); IPR007087 (PROSITE_PATTERNS); IPR007087 (PROSITE_PROFILES); IPR007087 (PROSITE_PROFILES); IPR007087 (PROSITE_PROFILES); SSF57667 (SUPERFAMILY); SSF57667 (SUPERFAMILY)
781	245782_length_417_cvg_2.0_tip_1_0	10	u5 small nuclear ribonucleoprotein 40 kda protein	139	7.74E-90	98.80%	0.101	IPR020472 (PRINTS); IPR001680 (SMART); IPR015943 (G3DSA:2.130.10.GENE3D); IPR001680 (PFAM); PTHR22847 (PANTHER); IPR019775 (PROSITE_PATTERNS); IPR019775 (PROSITE_PATTERNS); IPR001680 (PROSITE_PROFILES); IPR001680 (PROSITE_PROFILES); IPR017986 (PROSITE_PROFILES); IPR001680 (PROSITE_PROFILES); IPR017986 (SUPERFAMILY)
783	246012_length_418_cvg_3.8_tip_1_2	20	tyrosine-protein phosphatase lar isoform x4	139	1.45E-69	86.40%	0.109	PR00014 (PRINTS); IPR003961 (SMART); IPR013783 (G3DSA:2.60.40.GENE3D); IPR013783 (G3DSA:2.60.40.GENE3D); IPR003961 (PFAM); PTHR19134 (PANTHER); PTHR19134:SF204 (PANTHER); IPR003961 (PROSITE_PROFILES); IPR003961 (PROSITE_PROFILES); IPR003961 (SUPERFAMILY)
782	245848_length_418_cvg_5.9_tip_1_0	26	phospholipase b-like partial	140	9.17E-67	80.60%	0.1	IPR007000 (PFAM); PTHR12370:SF3 (PANTHER); IPR007000 (PANTHER)
784	246118_length_419_cvg_4.3_tip_1_4	21	integrin beta-ps	140	8.93E-36	64.70%	0.105	G3DSA:2.10.25.10 (GENE3D); G3DSA:2.10.25.10 (GENE3D); IPR013111 (PFAM); IPR015812 (PANTHER); PTHR10082:SF16 (PANTHER); IPR015812 (PROSITE_PATTERNS); IPR015812 (PROSITE_PATTERNS); SSF57196 (SUPERFAMILY); IPR012896 (SUPERFAMILY)
785	246120_length_419_cvg_2.9_tip_1_0	15	tripartite motif-containing protein 2-like isoform x5	140	1.58E-68	85.40%	0.097	IPR011042 (G3DSA:2.120.10.GENE3D); IPR001258 (PFAM); PTHR24103 (PANTHER); PTHR24103:SF1 (PANTHER); IPR013017 (PROSITE_PROFILES); IPR013017 (PROSITE_PROFILES); IPR013017 (PROSITE_PROFILES); IPR013017 (PROSITE_PROFILES); SSF101898 (SUPERFAMILY)
786	246292_length_420_cvg_3.2_tip_1_2	15	venom serine carboxypeptidase-like	140	1.72E-27	67.30%	0.107	IPR029058 (G3DSA:3.40.50.GENE3D); IPR001563 (PFAM); IPR001563 (PANTHER); IPR018202 (PROSITE_PATTERNS); IPR029058 (SUPERFAMILY)
787	246312_length_420_cvg_4.0_tip_1_5	19	zinc finger protein 358	140	4.48E-29	58.70%	0.101	IPR015880 (SMART); PF13465 (PFAM); IPR013087 (G3DSA:3.30.160.GENE3D); IPR013087 (G3DSA:3.30.160.GENE3D); PF13894 (PFAM); PTHR24409:SF14 (PANTHER); PTHR24409 (PANTHER); IPR007087 (PROSITE_PATTERNS); IPR007087 (PROSITE_PATTERNS); IPR007087 (PROSITE_PATTERNS); IPR007087 (PROSITE_PATTERNS); IPR007087 (PROSITE_PROFILES); IPR007087 (PROSITE_PROFILES); IPR007087 (PROSITE_PROFILES); IPR007087 (PROSITE_PROFILES); SSF57667 (SUPERFAMILY); SSF57667 (SUPERFAMILY)
788	246364_length_420_cvg_3.0_tip_1_1	15	basement membrane-specific heparan sulfate proteoglycan core protein	140	8.09E-17	55.50%	0.143	IPR002172 (PRINTS); IPR002172 (SMART); IPR002172 (G3DSA:4.10.400.GENE3D); IPR002172 (G3DSA:4.10.400.GENE3D); IPR002172 (PFAM); PTHR10529 (PANTHER); IPR023415 (PROSITE_PATTERNS); IPR023415 (PROSITE_PATTERNS); IPR002172 (PROSITE_PROFILES); IPR002172 (PROSITE_PROFILES); IPR002172 (SUPERFAMILY); IPR002172 (SUPERFAMILY)
789	246500_length_421_cvg_7.6_tip_1_4	43	carbonic anhydrase 1	140	1.05E-50	70.50%	0.108	IPR001148 (SMART); IPR001148 (PFAM); IPR001148 (G3DSA:3.10.200.GENE3D); IPR023561 (PANTHER); IPR001148 (PROSITE_PROFILES); IPR001148 (SUPERFAMILY)

790	246830_length_422_cvg_3.0_tip_1_2	13	basement membrane-specific heparan sulfate proteoglycan core protein	140	7.32E-47	69.90%	0.121	IPR002049 (SMART); G3DSA:2.10.25.10 (GENE3D); G3DSA:2.10.25.10 (GENE3D); G3DSA:2.10.25.10 (GENE3D); IPR002049 (PFAM); PTHR10574 (PANTHER); PTHR10574:SF148 (PANTHER); IPR013032 (PROSITE_PATTERNS); IPR002049 (PROSITE_PATTERNS); IPR013032 (PROSITE_PATTERNS); IPR002049 (PROSITE_PATTERNS); IPR002049 (PROSITE_PROFILES); IPR002049 (PROSITE_PROFILES); IPR002049 (PROSITE_PROFILES); SSF57196 (SUPERFAMILY); SSF57196 (SUPERFAMILY)
791	246870_length_422_cvg_2.7_tip_1_2	13	glutamyl aminopeptidase	140	4.04E-14	53.30%	0.114	IPR024571 (PFAM); IPR001930 (PANTHER); PTHR11533:SF165 (PANTHER)
792	246894_length_423_cvg_13.6_tip_1_4	47	ubiquitin family protein	141	1.44E-72	94.30%	0.139	IPR019956 (PRINTS); IPR000626 (SMART); G3DSA:3.10.20.90 (GENE3D); IPR000626 (PFAM); G3DSA:3.10.20.90 (GENE3D); PTHR10666 (PANTHER); IPR019954 (PROSITE_PATTERNS); IPR000626 (PROSITE_PROFILES); IPR000626 (PROSITE_PROFILES); IPR029071 (SUPERFAMILY); IPR029071 (SUPERFAMILY)
795	247390_length_425_cvg_3.5_tip_1_2	21	zinc finger protein 26-like	141	4.37E-22	55.80%	0.12	IPR015880 (SMART); IPR013087 (G3DSA:3.30.160.GENE3D); IPR013087 (G3DSA:3.30.160.GENE3D); PF13912 (PFAM); IPR013087 (G3DSA:3.30.160.GENE3D); PF13465 (PFAM); PTHR24409 (PANTHER); PTHR24409:SF13 (PANTHER); IPR007087 (PROSITE_PATTERNS); IPR007087 (PROSITE_PATTERNS); IPR007087 (PROSITE_PATTERNS); IPR007087 (PROSITE_PATTERNS); IPR007087 (PROSITE_PROFILES); IPR007087 (PROSITE_PROFILES); IPR007087 (PROSITE_PROFILES); IPR007087 (PROSITE_PROFILES); SSF57667 (SUPERFAMILY); SSF57667 (SUPERFAMILY)
793	247138_length_424_cvg_2.0_tip_1_0	8	e3 ubiquitin-protein ligase partial	142	2.53E-80	94.40%	0.135	IPR002110 (SMART); IPR020683 (G3DSA:1.25.40.GENE3D); IPR020683 (PFAM); IPR020683 (G3DSA:1.25.40.GENE3D); PTHR24202 (PANTHER); PTHR24202:SF52 (PANTHER); IPR002110 (PROSITE_PROFILES); IPR020683 (PROSITE_PROFILES); IPR002110 (PROSITE_PROFILES); IPR002110 (PROSITE_PROFILES); IPR020683 (SUPERFAMILY)
794	247334_length_425_cvg_4.0_tip_1_1	20	very low-density lipoprotein receptor-like	142	3.54E-21	64.20%	0.103	IPR002172 (PRINTS); IPR002172 (SMART); IPR002172 (PFAM); IPR002172 (G3DSA:4.10.400.GENE3D); IPR002172 (G3DSA:4.10.400.GENE3D); PTHR10529 (PANTHER); IPR023415 (PROSITE_PATTERNS); IPR023415 (PROSITE_PATTERNS); IPR002172 (PROSITE_PROFILES); IPR002172 (PROSITE_PROFILES); IPR002172 (SUPERFAMILY); IPR002172 (SUPERFAMILY)
796	247430_length_425_cvg_2.0_tip_1_3	14	complement component q subcomponent-like protein	142	5.91E-28	62.30%	0.102	IPR001073 (PRINTS); IPR001073 (SMART); IPR008983 (G3DSA:2.60.120.GENE3D); IPR001073 (PFAM); PTHR22923:SF51 (PANTHER); PTHR22923 (PANTHER); IPR001073 (PROSITE_PROFILES); IPR008983 (SUPERFAMILY)
797	247494_length_425_cvg_91.9_tip_0_3	1593	c-binding protein	142	7.49E-09	46.80%	0.135	IPR014853 (PFAM); G3DSA:2.10.25.10 (GENE3D); IPR002919 (SUPERFAMILY)
798	247510_length_426_cvg_8.8_tip_1_3	36	multiple epidermal growth factor-like domains protein 10 isoform x1	142	3.90E-42	70.50%	0.107	PR00011 (PRINTS); IPR002049 (SMART); IPR013032 (PFAM); G3DSA:2.170.300.10 (GENE3D); IPR002049 (PFAM); PTHR24035 (PANTHER); IPR013032 (PROSITE_PATTERNS); IPR013032 (PROSITE_PATTERNS); IPR013032 (PROSITE_PATTERNS); IPR000742 (PROSITE_PROFILES); IPR000742 (PROSITE_PROFILES)
799	247606_length_426_cvg_3.0_tip_1_4	17	syntaxin 1a	142	1.39E-96	99.30%	0.101	Coil (COILS); IPR006011 (SMART); G3DSA:1.20.58.70 (GENE3D); IPR006011 (PFAM); IPR028669 (PTHR19957:PANTHER); PTHR19957 (PANTHER); IPR010989 (SUPERFAMILY)
800	247666_length_426_cvg_4.0_tip_1_1	18	hemicentin- partial	142	1.70E-55	65.50%	0.106	G3DSA:2.10.25.10 (GENE3D); PTHR19897:SF158 (PANTHER); PTHR19897 (PANTHER)
801	247838_length_427_cvg_2.0_tip_1_2	13	repetin	142	2.47E-14	48.20%	0.1	no IPS match
802	247886_length_427_cvg_3.9_tip_1_5	21	macrophage mannose receptor 1	142	5.82E-31	60.40%	0.123	IPR016186 (G3DSA:3.10.100.GENE3D); IPR016186 (G3DSA:3.10.100.GENE3D); IPR001304 (PFAM); PTHR22803:SF16 (PANTHER); PTHR22803 (PANTHER); IPR018378 (PROSITE_PATTERNS); IPR001304 (PROSITE_PROFILES); IPR001304 (PROSITE_PROFILES); IPR016187 (SUPERFAMILY); IPR016187 (SUPERFAMILY)
803	247934_length_428_cvg_2.0_tip_1_4	9	cadherin-related tumor suppressor	143	1.51E-45	71.80%	0.101	IPR002126 (SMART); IPR002126 (G3DSA:2.60.40.GENE3D); IPR002126 (PFAM); PTHR24026:SF41 (PANTHER); PTHR24026 (PANTHER); IPR002126 (PROSITE_PROFILES); IPR015919 (SUPERFAMILY)
804	247950_length_428_cvg_6.1_tip_1_3	27	cd4-specific ankyrin repeat protein	143	3.15E-10	69.30%	0.112	IPR002110 (SMART); IPR020683 (PFAM); IPR020683 (G3DSA:1.25.40.GENE3D); PTHR24193 (PANTHER); IPR002110 (PROSITE_PROFILES); IPR002110 (PROSITE_PROFILES); IPR020683 (PROSITE_PROFILES); IPR020683 (SUPERFAMILY)
805	247964_length_428_cvg_3.0_tip_1_4	15	lysosomal alpha-glucosidase-like isoform x1	143	9.82E-67	81.40%	0.116	IPR025887 (PFAM); IPR000322 (PFAM); PTHR22762 (PANTHER); PTHR22762:SF57 (PANTHER); IPR011013 (SUPERFAMILY)

806	247994_length_428_cvg_2.0_tip_1_0	9	receptor-type tyrosine-protein phosphatase partial	143	6.07E-27	62.90%	0.107	IPR000242 (PRINTS); IPR003595 (SMART); IPR000242 (PFAM); IPR029021 (G3DSA:3.90.190.GENE3D); PTHR19134 (PANTHER); IPR016130 (PROSITE_PATTERNS); IPR000242 (PROSITE_PROFILES); IPR000387 (PROSITE_PROFILES); IPR029021 (SUPERFAMILY)
807	248020_length_428_cvg_4.9_tip_1_4	18	n-acetylglucosamine-6-sulfatase-like isoform x3	143	2.34E-65	76.00%	0.106	IPR000917 (PFAM); IPR017849 (G3DSA:3.40.720.GENE3D); PTHR10342 (PANTHER); PTHR10342:SF208 (PANTHER); IPR024607 (PROSITE_PATTERNS); IPR017850 (SUPERFAMILY)
808	248256_length_430_cvg_2.0_tip_1_2	11	5 - partial	143	2.22E-29	69.90%	0.106	IPR004843 (PFAM); IPR029052 (G3DSA:3.60.21.GENE3D); PTHR11575:SF21 (PANTHER); IPR006179 (PANTHER); IPR006146 (PROSITE_PATTERNS); IPR029052 (SUPERFAMILY)
809	248522_length_431_cvg_3.0_tip_1_1	12	transcriptional repressor ctcfl-like isoform x1	144	6.64E-83	93.50%	0.103	IPR015880 (SMART); IPR013087 (G3DSA:3.30.160.GENE3D); PF13465 (PFAM); IPR013087 (G3DSA:3.30.160.GENE3D); IPR013087 (G3DSA:3.30.160.GENE3D); IPR013087 (G3DSA:3.30.160.GENE3D); PTHR24375 (PANTHER); PTHR24375:SF1 (PANTHER); IPR007087 (PROSITE_PATTERNS); IPR007087 (PROSITE_PATTERNS); IPR007087 (PROSITE_PROFILES); IPR007087 (PROSITE_PROFILES); IPR007087 (PROSITE_PROFILES); IPR007087 (PROSITE_PROFILES); SSF57667 (SUPERFAMILY); SSF57667 (SUPERFAMILY); SSF57667 (SUPERFAMILY)
810	248626_length_431_cvg_18.0_tip_1_0	103	glycosyl-phosphatidylinositol-linked carbonic anhydrase	144	5.69E-15	54.20%	0.34	IPR001148 (PFAM); IPR001148 (G3DSA:3.10.200.GENE3D); IPR023561 (PANTHER); SIGNAL_PEPTIDE_H_REGION (PHOBIUS); NON_CYTOPLASMIC_DOMAIN (PHOBIUS); SIGNAL_PEPTIDE_N_REGION (PHOBIUS); SIGNAL_PEPTIDE (PHOBIUS); SIGNAL_PEPTIDE_C_REGION (PHOBIUS); IPR001148 (PROSITE_PROFILES); IPR001148 (SUPERFAMILY)
811	248652_length_431_cvg_3.0_tip_1_3	14	hemagglutinin amebocyte aggregation factor-like	144	2.02E-39	61.00%	0.14	PF14704 (PFAM); IPR026645 (PANTHER)
812	248662_length_431_cvg_3.4_tip_1_1	16	adam 17-like protease	144	1.34E-46	70.80%	0.106	IPR002870 (PFAM); PTHR11905:SF121 (PANTHER); PTHR11905 (PANTHER)
813	248728_length_432_cvg_3.6_tip_1_4	16	prostaglandin f synthase-like	144	9.46E-39	73.30%	0.291	IPR020471 (PRINTS); IPR023210 (PFAM); IPR023210 (G3DSA:3.20.20.GENE3D); PTHR11732:SF133 (PANTHER); IPR001395 (PANTHER); IPR023210 (SUPERFAMILY)
814	248748_length_432_cvg_5.0_tip_1_5	27	serine protease	144	6.64E-09	65.40%	0.129	G3DSA:2.40.10.10 (GENE3D); IPR001254 (PFAM); PTHR24268 (PANTHER); IPR009003 (SUPERFAMILY)
815	248864_length_432_cvg_55.0_tip_1_0	459	kallikrein-11 isoform x2	144	2.64E-21	65.20%	0.101	G3DSA:2.40.10.10 (GENE3D); IPR001254 (PFAM); PTHR24275 (PANTHER); IPR018114 (PROSITE_PATTERNS); IPR001254 (PROSITE_PROFILES); IPR009003 (SUPERFAMILY)
816	248936_length_433_cvg_7.7_tip_1_2	34	latrophilin-2-like isoform x3	144	1.41E-21	51.70%	0.498 Y	IPR000832 (PFAM); PTHR12011 (PANTHER); TRANSMEMBRANE (PHOBIUS); TRANSMEMBRANE (PHOBIUS); CYTOPLASMIC_DOMAIN (PHOBIUS); NON_CYTOPLASMIC_DOMAIN (PHOBIUS); CYTOPLASMIC_DOMAIN (PHOBIUS); TRANSMEMBRANE (PHOBIUS); NON_CYTOPLASMIC_DOMAIN (PHOBIUS); IPR017981 (PROSITE_PROFILES); TMhelix (TMHMM); TMhelix (TMHMM); TMhelix (TMHMM)
818	248982_length_433_cvg_2.0_tip_1_5	10	papilin isoform x1	144	1.68E-45	66.00%	0.101	IPR002223 (PRINTS); IPR002223 (SMART); IPR002223 (G3DSA:4.10.410.GENE3D); IPR002223 (PFAM); IPR002223 (G3DSA:4.10.410.GENE3D); IPR002223 (G3DSA:4.10.410.GENE3D); PTHR10083 (PANTHER); IPR020901 (PROSITE_PATTERNS); IPR020901 (PROSITE_PATTERNS); IPR002223 (PROSITE_PROFILES); IPR002223 (PROSITE_PROFILES); IPR002223 (PROSITE_PROFILES); IPR002223 (SUPERFAMILY); IPR002223 (SUPERFAMILY); IPR002223 (SUPERFAMILY)
819	249056_length_433_cvg_2.8_tip_1_2	14	dnaj homolog subfamily c member 3	144	3.27E-51	72.00%	0.103	Coil (COILS); IPR019734 (SMART); PF13414 (PFAM); IPR011990 (G3DSA:1.25.40.GENE3D); PTHR24078:SF132 (PANTHER); PTHR24078 (PANTHER); IPR019734 (PROSITE_PROFILES); IPR013026 (PROSITE_PROFILES); SSF48452 (SUPERFAMILY)
820	249062_length_433_cvg_3.6_tip_1_5	23	meprin a subunit beta-like	144	6.10E-29	61.30%	0.268	IPR001506 (PRINTS); IPR001506 (PFAM); IPR024079 (G3DSA:3.40.390.GENE3D); PTHR10127 (PANTHER); SSF55486 (SUPERFAMILY)
821	249102_length_434_cvg_5.0_tip_1_5	24	calcium calmodulin-dependent serine protein kinase	144	1.26E-46	73.60%	0.099	IPR001478 (SMART); IPR001478 (PFAM); G3DSA:2.30.30.40 (GENE3D); IPR001478 (G3DSA:2.30.42.GENE3D); PTHR23122 (PANTHER); IPR001478 (PROSITE_PROFILES); IPR001478 (SUPERFAMILY)
823	249306_length_434_cvg_3.4_tip_1_5	26	peptidoglycan linked protein	144	5.07E-08	49.63%	0.164	no IPS match
817	248976_length_433_cvg_2.0_tip_1_0	11	sh3 domain protein	145	7.14E-08	66.00%	0.102	no IPS match
822	249250_length_434_cvg_2.0_tip_1_4	7	adam 17-like protease	145	2.36E-73	86.80%	0.1	PTHR11905:SF121 (PANTHER); PTHR11905 (PANTHER); TRANSMEMBRANE (PHOBIUS); NON_CYTOPLASMIC_DOMAIN (PHOBIUS); CYTOPLASMIC_DOMAIN (PHOBIUS); TMhelix (TMHMM)
825	249530_length_436_cvg_3.0_tip_1_1	16	deoxyribonuclease- partial	145	1.44E-31	64.70%	0.114	IPR016202 (PRINTS); IPR016202 (SMART); IPR005135 (G3DSA:3.60.10.GENE3D); IPR016202 (PANTHER); IPR005135 (SUPERFAMILY)

826	249672_length_436_cvg_8.5_tip_1_1	24	heterochromatin-associated protein ment- partial	145	1.11E-10	77.40%	0.107	IPR023796 (PFAM); G3DSA:2.30.39.10 (GENE3D); PTHR11461:SF52 (PANTHER); IPR000215 (PANTHER); IPR023795 (PROSITE_PATTERNS); CYTOPLASMIC_DOMAIN (PHOBIUS); TRANSMEMBRANE (PHOBIUS); NON_CYTOPLASMIC_DOMAIN (PHOBIUS); IPR023796 (SUPERFAMILY)
828	249838_length_437_cvg_53.4_tip_1_5	2778	ribosomal protein s26	145	1.78E-59	89.80%	0.107	IPR000892 (PFAM); IPR000892 (PANTHER); IPR000892 (PROSITE_PATTERNS)
830	249868_length_437_cvg_2.2_tip_1_5	8	leucine-rich repeat neuronal protein 2	145	7.77E-19	53.70%	0.101	G3DSA:3.80.10.10 (GENE3D); IPR001611 (PFAM); PTHR24373:SF111 (PANTHER); PTHR24373 (PANTHER); SSF52058 (SUPERFAMILY)
824	249526_length_436_cvg_2.0_tip_1_0	15	vascular endothelial growth factor receptor partial	146	7.62E-17	49.00%	0.137	IPR003598 (SMART); IPR003599 (SMART); IPR013106 (PFAM); IPR013783 (G3DSA:2.60.40.GENE3D); IPR007110 (PROSITE_PROFILES); SSF48726 (SUPERFAMILY)
827	249676_length_436_cvg_5.0_tip_1_0	26	low-density lipoprotein receptor-related protein 1b	146	8.20E-14	57.50%	0.106	IPR002172 (SMART); IPR002172 (PFAM); G3DSA:4.10.1220.10 (GENE3D); IPR002172 (G3DSA:4.10.400.GENE3D); PTHR10529 (PANTHER); IPR002172 (PROSITE_PROFILES); IPR002172 (SUPERFAMILY); IPR002172 (SUPERFAMILY)
829	249844_length_437_cvg_4.0_tip_1_4	16	myosin light chain smooth muscle	146	2.88E-67	86.20%	0.1	Coil (COILS); IPR013783 (G3DSA:2.60.40.GENE3D); IPR013098 (PFAM); IPR013783 (G3DSA:2.60.40.GENE3D); PTHR19897:SF150 (PANTHER); PTHR19897 (PANTHER); IPR007110 (PROSITE_PROFILES); IPR007110 (PROSITE_PROFILES); SSF48726 (SUPERFAMILY); SSF48726 (SUPERFAMILY)
831	249900_length_437_cvg_5.8_tip_1_1	23	disintegrin and metalloproteinase domain-containing protein 10 isoform x1	146	5.90E-39	69.90%	0.111	IPR001762 (SMART); IPR001762 (G3DSA:4.10.70.GENE3D); IPR001762 (PFAM); PTHR11905:SF4 (PANTHER); PTHR11905 (PANTHER); IPR001762 (PROSITE_PROFILES); IPR001762 (SUPERFAMILY)
832	250054_length_438_cvg_4.8_tip_1_2	21	gamma-glutamyl hydrolase a-like isoform x3	146	7.41E-23	61.40%	0.114	IPR029062 (G3DSA:3.40.50.GENE3D); IPR015527 (PANTHER); IPR015527 (PROSITE_PROFILES); IPR029062 (SUPERFAMILY)
834	250112_length_439_cvg_6.6_tip_1_4	36	heat shock protein	146	7.82E-64	83.70%	0.101	IPR013126 (PRINTS); G3DSA:3.30.420.40 (GENE3D); IPR013126 (PFAM); IPR029047 (G3DSA:2.60.34.GENE3D); PTHR19375:SF142 (PANTHER); PTHR19375 (PANTHER); IPR029047 (SUPERFAMILY); SSF53067 (SUPERFAMILY)
835	250228_length_440_cvg_2.0_tip_1_5	8	calcium-activated chloride channel regulator 1-like	146	2.88E-28	61.00%	0.136	IPR002035 (G3DSA:3.40.50.GENE3D); PF13519 (PFAM); PTHR10579:SF8 (PANTHER); PTHR10579 (PANTHER); IPR002035 (PROSITE_PROFILES); IPR002035 (SUPERFAMILY)
833	250100_length_439_cvg_2.9_tip_1_0	15	cationic trypsin- partial	147	1.53E-30	56.30%	0.105	IPR001254 (SMART); IPR001254 (PFAM); G3DSA:2.40.10.10 (GENE3D); G3DSA:2.40.10.10 (GENE3D); PTHR24265 (PANTHER); IPR001254 (PROSITE_PROFILES); IPR009003 (SUPERFAMILY)
836	250340_length_440_cvg_38.6_tip_1_3	189	hemagglutinin amebocyte aggregation factor-like	147	1.23E-27	65.70%	0.113	PF14704 (PFAM); IPR026645 (PANTHER); TMhelix (TMHMM)
837	250380_length_441_cvg_2.8_tip_1_0	12	zinc finger protein 420-like	147	3.61E-33	62.80%	0.098	IPR015880 (SMART); IPR013087 (G3DSA:3.30.160.GENE3D); PF13465 (PFAM); IPR013087 (G3DSA:3.30.160.GENE3D); PTHR24409 (PANTHER); PTHR24409:SF13 (PANTHER); IPR007087 (PROSITE_PATTERNS); IPR007087 (PROSITE_PATTERNS); IPR007087 (PROSITE_PATTERNS); IPR007087 (PROSITE_PATTERNS); IPR007087 (PROSITE_PROFILES); IPR007087 (PROSITE_PROFILES); IPR007087 (PROSITE_PROFILES); IPR007087 (PROSITE_PROFILES); IPR007087 (PROSITE_PROFILES); SSF57667 (SUPERFAMILY); SSF57667 (SUPERFAMILY); SSF57667 (SUPERFAMILY)
838	250440_length_441_cvg_8.0_tip_1_5	43	alpha-2-macroglobulin-like protein 1 isoform x2	147	2.17E-21	54.10%	0.191	IPR011625 (PFAM); PTHR11412 (PANTHER); PTHR11412:SF80 (PANTHER)
839	250466_length_441_cvg_4.0_tip_1_3	13	trypsinogen 2	147	5.53E-28	72.40%	0.102	IPR001254 (PFAM); G3DSA:2.40.10.10 (GENE3D); PTHR24268 (PANTHER); IPR018114 (PROSITE_PATTERNS); TRANSMEMBRANE (PHOBIUS); CYTOPLASMIC_DOMAIN (PHOBIUS); NON_CYTOPLASMIC_DOMAIN (PHOBIUS); IPR001254 (PROSITE_PROFILES); IPR009003 (SUPERFAMILY)
840	250554_length_442_cvg_4.0_tip_1_1	22	disintegrin and metalloproteinase domain-containing protein 10 isoform x3	147	2.38E-45	67.90%	0.162	PTHR11905:SF4 (PANTHER); PTHR11905 (PANTHER); TRANSMEMBRANE (PHOBIUS); CYTOPLASMIC_DOMAIN (PHOBIUS); NON_CYTOPLASMIC_DOMAIN (PHOBIUS); TMhelix (TMHMM)
841	250628_length_442_cvg_13.7_tip_1_1	46	leukocyte elastase inhibitor	147	7.38E-26	55.70%	0.101	G3DSA:3.30.497.10 (GENE3D); IPR023796 (PFAM); G3DSA:2.30.39.10 (GENE3D); IPR000215 (PANTHER); PTHR11461:SF141 (PANTHER); IPR023796 (SUPERFAMILY)
842	250648_length_442_cvg_3.0_tip_1_1	13	polypeptide n-acetylgalactosaminyltransferase 2	147	1.62E-12	55.50%	0.103	IPR000772 (PFAM); G3DSA:2.80.10.50 (GENE3D); PTHR11675 (PANTHER); PTHR11675:SF24 (PANTHER); IPR000772 (PROSITE_PROFILES); IPR000772 (SUPERFAMILY)
843	250772_length_443_cvg_4.6_tip_1_5	23	interstitial collagenase	147	2.13E-58	74.30%	0.105	IPR021190 (PRINTS); IPR006026 (SMART); IPR001818 (PFAM); IPR024079 (G3DSA:3.40.390.GENE3D); PTHR10201 (PANTHER); SSF55486 (SUPERFAMILY)
844	251008_length_444_cvg_4.0_tip_1_0	21	heat shock 70 kda protein cognate 4- partial	148	1.17E-25	79.70%	0.121	Coil (COILS); G3DSA:3.30.420.40 (GENE3D); IPR013126 (PFAM); PTHR19375 (PANTHER); SSF53067 (SUPERFAMILY)
845	251064_length_444_cvg_7.0_tip_1_3	35	cyclin-g-associated kinase	148	6.63E-54	77.40%	0.106	IPR029021 (G3DSA:3.90.190.GENE3D); IPR000340 (PFAM); PTHR23172 (PANTHER); PTHR23172:SF19 (PANTHER); IPR029023 (PROSITE_PROFILES); IPR029021 (SUPERFAMILY)

846	251098_length_444_cvg_4.0_tip_1_3	19	c-type mannose receptor 2	148	1.59E-11	48.20%	0.108	IPR016186 (G3DSA:3.10.100.GENE3D); IPR016186 (G3DSA:3.10.100.GENE3D); IPR001304 (PFAM); PTHR22803 (PANTHER); IPR001304 (PROSITE_PROFILES); IPR016187 (SUPERFAMILY); IPR016187 (SUPERFAMILY)
848	251174_length_445_cvg_3.0_tip_1_2	18	histone-lysine n-methyltransferase prdm9	148	6.85E-39	65.60%	0.097	IPR015880 (SMART); IPR013087 (G3DSA:3.30.160.GENE3D); IPR013087 (G3DSA:3.30.160.GENE3D); IPR013087 (G3DSA:3.30.160.GENE3D); PF13465 (PFAM); IPR007087 (PFAM); IPR013087 (G3DSA:3.30.160.GENE3D); PTHR24409 (PANTHER); IPR007087 (PROSITE_PATTERNS); IPR007087 (PROSITE_PATTERNS); IPR007087 (PROSITE_PATTERNS); IPR007087 (PROSITE_PROFILES); IPR007087 (PROSITE_PROFILES); IPR007087 (PROSITE_PROFILES); IPR007087 (PROSITE_PROFILES); SSF57667 (SUPERFAMILY); SSF57667 (SUPERFAMILY)
849	251450_length_446_cvg_5.0_tip_1_5	22	calcium calmodulin-dependent protein kinase	148	7.64E-88	92.30%	0.128	IPR008145 (SMART); G3DSA:3.30.63.10 (GENE3D); IPR027417 (G3DSA:3.40.50.GENE3D); IPR008145 (PFAM); PTHR23122 (PANTHER); PTHR23122:SF7 (PANTHER); IPR020590 (PROSITE_PATTERNS); IPR008144 (PROSITE_PROFILES); IPR027417 (SUPERFAMILY)
847	251166_length_445_cvg_3.9_tip_1_3	15	beta-ketoacyl partial	149	1.14E-12	54.20%	0.1	SignalP-TM (SIGNALP_GRAM_POSITIVE)
850	251728_length_447_cvg_3.4_tip_1_4	16	g protein-coupled receptor kinase 2	149	1.78E-91	94.00%	0.103	Coil (COILS); IPR002290 (SMART); IPR000719 (PFAM); G3DSA:1.10.510.10 (GENE3D); PTHR24355:SF24 (PANTHER); PTHR24355 (PANTHER); IPR000719 (PROSITE_PROFILES); IPR011009 (SUPERFAMILY)
852	251978_length_449_cvg_4.0_tip_1_5	17	serine threonine-protein kinase genghis khan isoform x1	149	5.57E-81	98.50%	0.118	IPR002290 (SMART); G3DSA:1.10.510.10 (GENE3D); IPR000719 (PFAM); PTHR22988 (PANTHER); PTHR22988:SF2 (PANTHER); IPR008271 (PROSITE_PATTERNS); IPR000719 (PROSITE_PROFILES); IPR011009 (SUPERFAMILY)
851	251738_length_448_cvg_2.0_tip_1_3	13	matrix metalloproteinase-17 isoform x2	150	2.08E-29	52.40%	0.104	IPR000585 (G3DSA:2.110.10.GENE3D); IPR018487 (PFAM); IPR001818 (PFAM); IPR024079 (G3DSA:3.40.390.GENE3D); PTHR10201:SF135 (PANTHER); PTHR10201 (PANTHER); IPR018487 (PROSITE_PROFILES); IPR000585 (SUPERFAMILY); SSF55486 (SUPERFAMILY)
853	252028_length_449_cvg_4.2_tip_1_3	18	von willebrand factor type egf and pentraxin domain-containing protein 1-like isoform x2	150	1.41E-34	62.20%	0.112	IPR000742 (SMART); G3DSA:2.10.25.10 (GENE3D); IPR013032 (PFAM); G3DSA:2.10.25.10 (GENE3D); PTHR19325 (PANTHER); PTHR19325:SF43 (PANTHER); IPR013032 (PROSITE_PATTERNS); IPR013032 (PROSITE_PATTERNS); IPR013032 (PROSITE_PATTERNS); IPR013032 (PROSITE_PATTERNS); IPR000742 (PROSITE_PROFILES); IPR000742 (PROSITE_PROFILES); IPR000436 (PROSITE_PROFILES); IPR000742 (PROSITE_PROFILES); SSF57196 (SUPERFAMILY); IPR000436 (SUPERFAMILY)
854	252070_length_450_cvg_8.6_tip_1_0	35	locomotion-related protein hikaru genki	150	1.84E-23	48.60%	0.12	IPR000436 (SMART); IPR000436 (PFAM); G3DSA:2.10.70.10 (GENE3D); G3DSA:2.10.70.10 (GENE3D); G3DSA:2.10.70.10 (GENE3D); PTHR19325 (PANTHER); IPR000436 (PROSITE_PROFILES); IPR000436 (PROSITE_PROFILES); IPR000436 (SUPERFAMILY); IPR000436 (SUPERFAMILY)
855	252158_length_450_cvg_3.0_tip_1_1	21	low-density lipoprotein receptor-related protein 2	150	6.76E-07	64.75%	0.147	IPR002172 (SMART); IPR002172 (G3DSA:4.10.400.GENE3D); IPR002172 (PFAM); IPR002172 (PROSITE_PROFILES); IPR002172 (SUPERFAMILY)
856	252202_length_450_cvg_3.0_tip_1_0	11	slei family protein	150	7.06E-17	52.60%	0.096	TRANSMEMBRANE (PHOBIUS); NON_CYTOPLASMIC_DOMAIN (PHOBIUS); CYTOPLASMIC_DOMAIN (PHOBIUS); TMhelix (TMHMM)
857	252272_length_451_cvg_3.2_tip_1_0	18	cysteine proteinase	151	1.36E-58	76.20%	0.112	IPR000668 (SMART); IPR013201 (PFAM); G3DSA:3.90.70.10 (GENE3D); IPR000668 (PFAM); PTHR12411:SF316 (PANTHER); IPR013128 (PANTHER); IPR000169 (PROSITE_PATTERNS); SSF54001 (SUPERFAMILY)
858	252600_length_452_cvg_11.0_tip_1_3	70	dermatopontin 2	151	2.34E-07	76.50%	0.166	PF14704 (PFAM); IPR026645 (PANTHER)
859	252744_length_453_cvg_81.7_tip_0_1	741	fibrinogen-related protein	151	4.74E-44	66.90%	0.104	IPR002181 (SMART); IPR014716 (G3DSA:3.90.215.GENE3D); IPR002181 (PFAM); IPR014715 (G3DSA:4.10.530.GENE3D); PTHR19143 (PANTHER); IPR020837 (PROSITE_PATTERNS); IPR002181 (PROSITE_PROFILES); IPR002181 (SUPERFAMILY)
860	252914_length_454_cvg_3.5_tip_1_3	15	lysosomal pro-x carboxypeptidase	152	3.80E-10	79.00%	0.117	PTHR11010:SF11 (PANTHER); IPR008758 (PANTHER); CYTOPLASMIC_DOMAIN (PHOBIUS); TRANSMEMBRANE (PHOBIUS); NON_CYTOPLASMIC_DOMAIN (PHOBIUS)
861	253336_length_457_cvg_4.0_tip_1_4	20	dnak protein	152	1.39E-77	88.30%	0.119	IPR013126 (PRINTS); G3DSA:3.30.420.40 (GENE3D); IPR013126 (PFAM); G3DSA:3.30.420.40 (GENE3D); PTHR19375:SF142 (PANTHER); PTHR19375 (PANTHER); IPR018181 (PROSITE_PATTERNS); SSF53067 (SUPERFAMILY)
863	253506_length_457_cvg_4.1_tip_1_1	23	heat shock 70 kda protein 14	152	2.63E-28	52.90%	0.148	IPR029047 (G3DSA:2.60.34.GENE3D); IPR013126 (PFAM); PTHR19375 (PANTHER); PTHR19375:SF158 (PANTHER); IPR029047 (SUPERFAMILY)

865	253680_length_458_cvg_3.0_tip_1_5	18	pro-phenol oxidase subunit 2	152	3.78E-41	62.70%	0.11		IPR008922 (G3DSA:1.10.1280.GENE3D); IPR005203 (G3DSA:2.60.40.GENE3D); IPR000896 (PFAM); IPR005203 (PFAM); PTHR11511:SF24 (PANTHER); IPR013788 (PANTHER); IPR013788 (PROSITE_PATTERNS); IPR002227 (PROSITE_PATTERNS); IPR014756 (SUPERFAMILY); IPR008922 (SUPERFAMILY)
862	253386_length_457_cvg_2.0_tip_1_3	11	leucine-rich repeat-containing protein 15-like	153	7.65E-37	65.70%	0.101		IPR003591 (SMART); SM00365 (SMART); IPR001611 (PFAM); G3DSA:3.80.10.10 (GENE3D); IPR001611 (PFAM); IPR001611 (PFAM); PTHR24365 (PANTHER); IPR001611 (PROSITE_PROFILES); IPR001611 (PROSITE_PROFILES); IPR001611 (PROSITE_PROFILES); IPR001611 (PROSITE_PROFILES); SSF52058 (SUPERFAMILY)
864	253582_length_458_cvg_4.7_tip_1_0	25	collagen alpha-1 chain isoform x5	153	1.50E-10	50.10%	0.136		IPR010515 (PFAM)
866	253902_length_460_cvg_3.0_tip_1_5	13	gastrula zinc finger protein	153	9.58E-15	59.20%	0.104		IPR015880 (SMART); IPR013087 (G3DSA:3.30.160.GENE3D); PF13465 (PFAM); IPR007087 (PROSITE_PATTERNS); IPR007087 (PROSITE_PROFILES); IPR007087 (PROSITE_PROFILES); SSF57667 (SUPERFAMILY)
867	253922_length_460_cvg_2.0_tip_1_4	6	von willebrand factor type egf and pentraxin domain-containing protein 1	153	4.39E-28	54.50%	0.1		IPR000436 (SMART); G3DSA:2.10.70.10 (GENE3D); IPR000436 (PFAM); G3DSA:2.10.70.10 (GENE3D); PTHR19325 (PANTHER); IPR000436 (PROSITE_PROFILES); IPR000436 (PROSITE_PROFILES); IPR000436 (PROSITE_PROFILES); IPR000436 (SUPERFAMILY); IPR000436 (SUPERFAMILY); IPR000436 (SUPERFAMILY)
868	254136_length_461_cvg_110.7_tip_0_1	52567	hc subunit partial	154	8.99E-100	77.00%	0.1		IPR000896 (PFAM); IPR008922 (G3DSA:1.10.1280.GENE3D); IPR005203 (G3DSA:2.60.40.GENE3D); IPR005203 (PFAM); PTHR11511:SF5 (PANTHER); IPR013788 (PANTHER); IPR013788 (PROSITE_PATTERNS); IPR002227 (PROSITE_PATTERNS); IPR008922 (SUPERFAMILY); IPR014756 (SUPERFAMILY)
869	254348_length_463_cvg_2.7_tip_1_5	15	ww domain-containing oxidoreductase-like	154	5.14E-48	80.80%	0.124		IPR001202 (SMART); IPR001202 (PFAM); IPR016040 (G3DSA:3.40.50.GENE3D); G3DSA:2.20.70.10 (GENE3D); PTHR24310:SF30 (PANTHER); PTHR24310 (PANTHER); IPR001202 (PROSITE_PROFILES); SSF51735 (SUPERFAMILY); IPR001202 (SUPERFAMILY)
870	254370_length_463_cvg_7.0_tip_1_2	36	von willebrand factor a domain-containing protein 8	154	4.46E-44	84.40%	0.107		PTHR21610:SF9 (PANTHER); PTHR21610 (PANTHER)
871	254442_length_463_cvg_3.2_tip_1_2	17	protein flightless-1-like	154	2.73E-49	94.50%	0.102		IPR007122 (PRINTS); IPR007122 (SMART); IPR029006 (G3DSA:3.40.20.GENE3D); IPR007123 (PFAM); IPR029919 (PTHR11977:PANTHER); IPR007122 (PANTHER); SSF55753 (SUPERFAMILY)
872	254482_length_463_cvg_2.0_tip_1_5	13	adp-ribosylation factor-like protein 2	154	1.10E-71	95.90%	0.285		IPR006689 (PRINTS); IPR024156 (SMART); IPR027417 (G3DSA:3.40.50.GENE3D); IPR006689 (PFAM); IPR005225 (TIGRFAM); PTHR11711 (PANTHER); PTHR11711:SF26 (PANTHER); CYTOPLASMIC_DOMAIN (PHOBIUS); TRANSMEMBRANE (PHOBIUS); NON_CYTOPLASMIC_DOMAIN (PHOBIUS); IPR024156 (PROSITE_PROFILES); IPR027417 (SUPERFAMILY); TMhelix (TMHMM)
874	254574_length_464_cvg_2.0_tip_1_5	9	zinc finger protein partial	154	1.09E-69	81.20%	0.101		IPR015880 (SMART); PF13912 (PFAM); IPR013087 (G3DSA:3.30.160.GENE3D); IPR013087 (G3DSA:3.30.160.GENE3D); PF13465 (PFAM); IPR013087 (G3DSA:3.30.160.GENE3D); PTHR24409 (PANTHER); IPR007087 (PROSITE_PATTERNS); IPR007087 (PROSITE_PATTERNS); IPR007087 (PROSITE_PATTERNS); IPR007087 (PROSITE_PATTERNS); IPR007087 (PROSITE_PROFILES); IPR007087 (PROSITE_PROFILES); IPR007087 (PROSITE_PROFILES); IPR007087 (PROSITE_PROFILES); IPR007087 (PROSITE_PROFILES); IPR007087 (PROSITE_PROFILES); IPR007087 (PROSITE_PROFILES); SSF57667 (SUPERFAMILY); SSF57667 (SUPERFAMILY); SSF57667 (SUPERFAMILY)
873	254526_length_464_cvg_2.7_tip_1_0	11	fibrillin-2-like	155	2.37E-61	77.50%	0.101		IPR000742 (SMART); IPR001881 (SMART); G3DSA:2.10.25.10 (GENE3D); G3DSA:2.10.25.10 (GENE3D); IPR001881 (PFAM); PTHR24039:SF20 (PANTHER); IPR011398 (PANTHER); IPR000152 (PROSITE_PATTERNS); IPR000152 (PROSITE_PATTERNS); IPR018097 (PROSITE_PATTERNS); IPR018097 (PROSITE_PATTERNS); IPR013032 (PROSITE_PATTERNS); IPR018097 (PROSITE_PATTERNS); IPR000152 (PROSITE_PATTERNS); IPR013032 (PROSITE_PATTERNS); IPR013032 (PROSITE_PATTERNS); IPR000742 (PROSITE_PROFILES); IPR000742 (PROSITE_PROFILES); IPR000742 (PROSITE_PROFILES); IPR009030 (SUPERFAMILY)
875	254678_length_465_cvg_9.1_tip_1_1	43	spindolin-related protein	155	2.96E-40	57.80%	0.102		G3DSA:2.70.50.50 (GENE3D); IPR004302 (PFAM); IPR014756 (SUPERFAMILY)
876	254710_length_465_cvg_27.0_tip_1_0	100	atrial natriuretic peptide-converting enzyme	155	5.47E-08	53.70%	0.519	Y	IPR002172 (PRINTS); IPR002172 (G3DSA:4.10.400.GENE3D); IPR002172 (G3DSA:4.10.400.GENE3D); SIGNAL_PEPTIDE_C_REGION (PHOBIUS); SIGNAL_PEPTIDE (PHOBIUS); NON_CYTOPLASMIC_DOMAIN (PHOBIUS); SIGNAL_PEPTIDE_H_REGION (PHOBIUS); SIGNAL_PEPTIDE_N_REGION (PHOBIUS); SignalP-TM (SIGNALP_EUK); SignalP-TM (SIGNALP_GRAM_POSITIVE); IPR002172 (SUPERFAMILY); IPR002172 (SUPERFAMILY); TMhelix (TMHMM)
877	254736_length_465_cvg_3.0_tip_1_4	15	laminin subunit alpha	155	1.71E-64	74.70%	0.096		no IPS match

882	255068_length_467_cvg_49.0_tip_1_5	328	projectin short variant	155	1.08E-44	87.10%	0.32	IPR003598 (SMART); IPR013098 (PFAM); IPR013783 (G3DSA:2.60.40.GENE3D); PTHR19897:SF155 (PANTHER); PTHR19897 (PANTHER); IPR007110 (PROSITE_PROFILES); SSF48726 (SUPERFAMILY)
878	254914_length_466_cvg_4.9_tip_1_3	28	ankyrin repeat and socs box protein 3-like isoform x1	156	2.53E-28	56.10%	0.131	IPR002110 (PRINTS); IPR002110 (SMART); IPR020683 (G3DSA:1.25.40.GENE3D); IPR020683 (PFAM); PTHR24198 (PANTHER); IPR002110 (PROSITE_PROFILES); IPR002110 (PROSITE_PROFILES); IPR002110 (PROSITE_PROFILES); IPR002110 (PROSITE_PROFILES); IPR020683 (PROSITE_PROFILES); IPR020683 (SUPERFAMILY)
879	254916_length_466_cvg_3.4_tip_1_3	23	cognate 70 kda heat shock partial	156	9.15E-101	99.10%	0.134	IPR013126 (PRINTS); IPR029047 (G3DSA:2.60.34.GENE3D); IPR013126 (PFAM); PTHR19375:SF142 (PANTHER); PTHR19375 (PANTHER); IPR029047 (SUPERFAMILY)
880	255002_length_467_cvg_3.0_tip_1_0	14	multicopper oxidase- isoform a	156	1.59E-65	76.10%	0.116	IPR008972 (G3DSA:2.60.40.GENE3D); IPR011707 (PFAM); PTHR11709:SF33 (PANTHER); PTHR11709 (PANTHER); IPR008972 (SUPERFAMILY)
881	255040_length_467_cvg_2.5_tip_1_1	21	calcium calmodulin-dependent serine protein kinase	156	9.61E-16	66.50%	0.116	IPR014775 (PFAM); IPR004172 (PROSITE_PROFILES); SSF101288 (SUPERFAMILY)
883	255154_length_468_cvg_5.7_tip_1_1	36	tissue factor pathway inhibitor 2 precursor	156	3.47E-13	64.80%	0.126	IPR002223 (PRINTS); IPR002223 (SMART); IPR008037 (PFAM); IPR002223 (PFAM); IPR002223 (G3DSA:4.10.410.GENE3D); PTHR10083 (PANTHER); IPR020901 (PROSITE_PATTERNS); TRANSMEMBRANE (PHOBIUS); NON_CYTOPLASMIC_DOMAIN (PHOBIUS); CYTOPLASMIC_DOMAIN (PHOBIUS); IPR020862 (PROSITE_PROFILES); IPR002223 (PROSITE_PROFILES); IPR008037 (SUPERFAMILY); IPR002223 (SUPERFAMILY); TMhelix (TMHMM)
884	255184_length_468_cvg_2.0_tip_1_4	9	low-density lipoprotein receptor-related protein partial	156	1.02E-28	55.60%	0.121	IPR000033 (SMART); IPR011042 (G3DSA:2.120.10.GENE3D); IPR000033 (PFAM); PTHR10529 (PANTHER); IPR000033 (PROSITE_PROFILES); IPR000033 (PROSITE_PROFILES); SSF63825 (SUPERFAMILY)
885	255306_length_468_cvg_2.6_tip_1_3	24	zinc finger protein 311	156	8.16E-28	58.80%	0.11	IPR015880 (SMART); PF13465 (PFAM); IPR013087 (G3DSA:3.30.160.GENE3D); IPR013087 (G3DSA:3.30.160.GENE3D); PTHR24409:SF14 (PANTHER); PTHR24409 (PANTHER); IPR007087 (PROSITE_PATTERNS); IPR007087 (PROSITE_PATTERNS); IPR007087 (PROSITE_PATTERNS); IPR007087 (PROSITE_PATTERNS); IPR007087 (PROSITE_PROFILES); IPR007087 (PROSITE_PROFILES); IPR007087 (PROSITE_PROFILES); IPR007087 (PROSITE_PROFILES); SSF57667 (SUPERFAMILY); SSF57667 (SUPERFAMILY); SSF57667 (SUPERFAMILY)
886	255310_length_468_cvg_3.2_tip_1_2	17	calcium calmodulin-dependent protein kinase	156	2.68E-24	90.40%	0.12	IPR027417 (G3DSA:3.40.50.GENE3D); PTHR23122:SF7 (PANTHER); PTHR23122 (PANTHER); TRANSMEMBRANE (PHOBIUS); NON_CYTOPLASMIC_DOMAIN (PHOBIUS); CYTOPLASMIC_DOMAIN (PHOBIUS); TRANSMEMBRANE (PHOBIUS); CYTOPLASMIC_DOMAIN (PHOBIUS); IPR027417 (SUPERFAMILY); TMhelix (TMHMM); TMhelix (TMHMM)
887	255322_length_468_cvg_71.7_tip_1_5	1907	epididymal secretory protein e1	156	7.58E-12	56.70%	0.112	IPR003172 (PFAM); IPR003172 (G3DSA:2.60.40.GENE3D); PTHR11306 (PANTHER); IPR014756 (SUPERFAMILY)
888	255450_length_469_cvg_12.0_tip_1_2	58	hemagglutinin	156	2.26E-44	68.60%	0.103	G3DSA:1.10.390.10 (GENE3D); IPR001570 (PFAM); SSF55486 (SUPERFAMILY)
890	255652_length_470_cvg_2.1_tip_1_5	13	hypothetical protein LOTGIDRAFT_122202, partial	156	9.18E-08	56.75%	0.105	IPR013182 (PFAM)
889	255652_length_470_cvg_2.1_tip_1_3	13	dna-directed rna polymerase	157	6.62E-14	66.80%	0.1	no IPS match
891	255752_length_471_cvg_3.0_tip_1_0	17	collagen alpha-1 chain	157	1.05E-30	65.10%	0.106	IPR008160 (PFAM); PTHR24023 (PANTHER); PTHR24023:SF379 (PANTHER)
892	255752_length_471_cvg_3.0_tip_1_4	17	threonine-rich gpi-anchored partial	157	7.93E-24	46.80%	0.107	PTHR24023:SF405 (PANTHER); PTHR24023 (PANTHER)
893	255788_length_471_cvg_25.5_tip_1_0	127	type iv secretion protein	157	3.77E-16	45.50%	0.235	SIGNAL_PEPTIDE (PHOBIUS); SIGNAL_PEPTIDE_C_REGION (PHOBIUS); TRANSMEMBRANE (PHOBIUS); SIGNAL_PEPTIDE_H_REGION (PHOBIUS); CYTOPLASMIC_DOMAIN (PHOBIUS); NON_CYTOPLASMIC_DOMAIN (PHOBIUS); SIGNAL_PEPTIDE_N_REGION (PHOBIUS); SignalP-TM (SIGNALP_GRAM_POSITIVE)
894	255862_length_472_cvg_4.0_tip_1_1	22	upf0764 protein c16orf89 homolog	157	3.45E-11	51.30%	0.108	no IPS match
895	255908_length_472_cvg_3.0_tip_1_4	16	plexin partial	157	1.30E-92	94.80%	0.1	Coil (COILS); IPR013548 (PFAM); PTHR22625 (PANTHER); PTHR22625:SF16 (PANTHER)
896	255934_length_472_cvg_2.0_tip_1_4	10	dnak protein	157	2.60E-83	91.20%	0.117	IPR013126 (PRINTS); G3DSA:3.30.420.40 (GENE3D); G3DSA:3.30.420.40 (GENE3D); IPR013126 (PFAM); G3DSA:3.90.640.10 (GENE3D); PTHR19375:SF142 (PANTHER); PTHR19375 (PANTHER); IPR018181 (PROSITE_PATTERNS); SSF53067 (SUPERFAMILY); SSF53067 (SUPERFAMILY)

897	255982_length_472_cvg_2.0_tip_1_1	11	zinc finger protein 558	157	9.54E-18	62.10%	0.102	IPR015880 (SMART); IPR013087 (G3DSA:3.30.160.GENE3D); IPR007087 (PFAM); IPR013087 (G3DSA:3.30.160.GENE3D); IPR013087 (G3DSA:3.30.160.GENE3D); PF13465 (PFAM); PTHR24409:SF14 (PANTHER); PTHR24409 (PANTHER); IPR007087 (PROSITE_PATTERNS); IPR007087 (PROSITE_PATTERNS); IPR007087 (PROSITE_PATTERNS); IPR007087 (PROSITE_PROFILES); IPR007087 (PROSITE_PROFILES); IPR007087 (PROSITE_PROFILES); SSF57667 (SUPERFAMILY); SSF57667 (SUPERFAMILY)
898	256082_length_473_cvg_2.9_tip_1_0	24	iron zinc purple acid phosphatase-like isoform x1	158	3.69E-76	81.40%	0.117	IPR029052 (G3DSA:3.60.21.GENE3D); IPR004843 (PFAM); IPR015914 (G3DSA:2.60.40.GENE3D); PTHR22953:SF9 (PANTHER); PTHR22953 (PANTHER); IPR008963 (SUPERFAMILY); IPR029052 (SUPERFAMILY)
899	256254_length_474_cvg_2.0_tip_1_1	15	heat shock partial	158	1.07E-66	82.30%	0.104	Coil (COILS); Coil (COILS); IPR001623 (PRINTS); IPR001623 (SMART); IPR001623 (G3DSA:1.10.287.GENE3D); IPR001623 (PFAM); PTHR24078 (PANTHER); PTHR24078:SF125 (PANTHER); IPR018253 (PROSITE_PATTERNS); NON_CYTOPLASMIC_DOMAIN (PHOBIUS); TRANSMEMBRANE (PHOBIUS); CYTOPLASMIC_DOMAIN (PHOBIUS); IPR001623 (PROSITE_PROFILES); IPR001623 (SUPERFAMILY); TMhelix (TMHMM)
900	256320_length_474_cvg_3.0_tip_1_3	18	serine beta-lactamase-like protein mitochondrial isoform x2	158	1.08E-53	75.00%	0.12	Coil (COILS); IPR012338 (G3DSA:3.40.710.GENE3D); IPR001466 (PFAM); PTHR22935:SF64 (PANTHER); PTHR22935 (PANTHER); IPR012338 (SUPERFAMILY)
902	256498_length_475_cvg_3.4_tip_1_5	16	zinc finger protein	158	1.23E-25	55.50%	0.099	IPR015880 (SMART); PF13465 (PFAM); IPR007087 (PFAM); IPR013087 (G3DSA:3.30.160.GENE3D); PF13894 (PFAM); IPR013087 (G3DSA:3.30.160.GENE3D); PTHR24409 (PANTHER); PTHR24409:SF14 (PANTHER); IPR007087 (PROSITE_PATTERNS); IPR007087 (PROSITE_PATTERNS); IPR007087 (PROSITE_PATTERNS); IPR007087 (PROSITE_PATTERNS); IPR007087 (PROSITE_PROFILES); IPR007087 (PROSITE_PROFILES); IPR007087 (PROSITE_PROFILES); IPR007087 (PROSITE_PROFILES); IPR007087 (PROSITE_PROFILES); SSF57667 (SUPERFAMILY); SSF57667 (SUPERFAMILY); SSF57667 (SUPERFAMILY)
901	256356_length_475_cvg_4.0_tip_1_3	21	alpha- partial	159	5.46E-39	65.90%	0.117	IPR001599 (PFAM); PTHR11412:SF80 (PANTHER); PTHR11412 (PANTHER); IPR014756 (SUPERFAMILY)
904	257126_length_479_cvg_2.0_tip_1_2	12	ccr4-not transcription complex subunit 6-like isoform x7	159	6.27E-62	80.90%	0.121	IPR003591 (SMART); IPR025875 (PFAM); G3DSA:3.80.10.10 (GENE3D); PTHR12121:SF35 (PANTHER); PTHR12121 (PANTHER); IPR001611 (PROSITE_PROFILES); IPR001611 (PROSITE_PROFILES); IPR001611 (PROSITE_PROFILES); SSF52058 (SUPERFAMILY)
903	257014_length_478_cvg_4.0_tip_1_0	24	zinc finger protein 64 isoform x2	160	3.08E-59	77.80%	0.101	IPR015880 (SMART); IPR013087 (G3DSA:3.30.160.GENE3D); IPR013087 (G3DSA:3.30.160.GENE3D); PF13465 (PFAM); IPR013087 (G3DSA:3.30.160.GENE3D); PTHR24409 (PANTHER); IPR007087 (PROSITE_PATTERNS); IPR007087 (PROSITE_PROFILES); IPR007087 (PROSITE_PROFILES); IPR007087 (PROSITE_PROFILES); IPR007087 (PROSITE_PROFILES); SSF57667 (SUPERFAMILY); SSF57667 (SUPERFAMILY)
905	257166_length_479_cvg_4.0_tip_1_0	22	dehydrogenase reductase sdr family member 11	160	7.92E-59	77.30%	0.191	IPR002347 (PRINTS); IPR002198 (PFAM); IPR016040 (G3DSA:3.40.50.GENE3D); PTHR24322 (PANTHER); PTHR24322:SF310 (PANTHER); SSF51735 (SUPERFAMILY)
906	257470_length_481_cvg_4.6_tip_1_2	24	allatostatin precursor protein	160	4.96E-25	57.70%	0.099	IPR010276 (PFAM)
908	257508_length_482_cvg_6.9_tip_1_2	40	zinc finger protein 502	160	4.14E-37	60.00%	0.1	IPR015880 (SMART); IPR013087 (G3DSA:3.30.160.GENE3D); PF13912 (PFAM); IPR013087 (G3DSA:3.30.160.GENE3D); PF13465 (PFAM); IPR007087 (PFAM); IPR013087 (G3DSA:3.30.160.GENE3D); PTHR24409:SF13 (PANTHER); PTHR24409 (PANTHER); IPR007087 (PROSITE_PATTERNS); IPR007087 (PROSITE_PATTERNS); IPR007087 (PROSITE_PATTERNS); IPR007087 (PROSITE_PATTERNS); IPR007087 (PROSITE_PATTERNS); IPR007087 (PROSITE_PROFILES); IPR007087 (PROSITE_PROFILES); IPR007087 (PROSITE_PROFILES); IPR007087 (PROSITE_PROFILES); IPR007087 (PROSITE_PROFILES); SSF57667 (SUPERFAMILY); SSF57667 (SUPERFAMILY); SSF57667 (SUPERFAMILY)
909	257518_length_482_cvg_3.0_tip_1_5	18	tyrosine-protein phosphatase non-receptor	160	1.88E-43	69.90%	0.19	IPR000242 (PRINTS); IPR003595 (SMART); IPR000242 (PFAM); IPR029021 (G3DSA:3.90.190.GENE3D); PTHR19134 (PANTHER); IPR016130 (PROSITE_PATTERNS); IPR000242 (PROSITE_PROFILES); IPR000387 (PROSITE_PROFILES); IPR029021 (SUPERFAMILY)
907	257490_length_482_cvg_2.0_tip_1_3	11	macrophage mannose receptor 1-like isoform 2	161	5.55E-25	51.90%	0.119	IPR001304 (SMART); IPR016186 (G3DSA:3.10.100.GENE3D); IPR001304 (PFAM); PTHR22803 (PANTHER); PTHR22803:SF16 (PANTHER); IPR001304 (PROSITE_PROFILES); IPR016187 (SUPERFAMILY)
910	257626_length_483_cvg_2.7_tip_1_5	14	alpha-mannosidase 2x	161	6.04E-47	75.20%	0.102	IPR027291 (G3DSA:3.20.110.GENE3D); IPR000602 (PFAM); PTHR11607:SF4 (PANTHER); PTHR11607 (PANTHER); IPR011330 (SUPERFAMILY)

911	258184_length_486_cvg_3.4_tip_1_0	27	low-density lipoprotein receptor-related protein 1b-like	162	5.66E-09	74.10%	0.102	IPR002172 (PRINTS); IPR002172 (SMART); IPR002172 (G3DSA:4.10.400.GENE3D); IPR002172 (PFAM); IPR023415 (PROSITE_PATTERNS); IPR002172 (PROSITE_PROFILES); IPR002172 (SUPERFAMILY)
912	258288_length_487_cvg_3.0_tip_1_2	16	ankyrin repeat and sam domain-containing protein 6-like	162	7.07E-35	62.20%	0.609 Y	IPR002110 (PRINTS); IPR002110 (SMART); IPR020683 (PFAM); IPR020683 (G3DSA:1.25.40.GENE3D); IPR002110 (PFAM); PTHR10627 (PANTHER); PTHR10627:SF2 (PANTHER); IPR020683 (PROSITE_PROFILES); IPR002110 (PROSITE_PROFILES); IPR002110 (PROSITE_PROFILES); IPR002110 (PROSITE_PROFILES); SignalP-noTM (SIGNALP_EUK); IPR020683 (SUPERFAMILY)
913	258484_length_488_cvg_2.0_tip_1_2	8	zinc finger protein	162	6.95E-26	55.80%	0.1	IPR015880 (SMART); IPR013087 (G3DSA:3.30.160.GENE3D); IPR013087 (G3DSA:3.30.160.GENE3D); IPR007087 (PFAM); PTHR24409 (PANTHER); PTHR24409:SF14 (PANTHER); IPR007087 (PROSITE_PATTERNS); IPR007087 (PROSITE_PATTERNS); IPR007087 (PROSITE_PATTERNS); IPR007087 (PROSITE_PATTERNS); IPR007087 (PROSITE_PROFILES); IPR007087 (PROSITE_PROFILES); IPR007087 (PROSITE_PROFILES); IPR007087 (PROSITE_PROFILES); SSF57667 (SUPERFAMILY); SSF57667 (SUPERFAMILY); SSF57667 (SUPERFAMILY)
914	258486_length_488_cvg_4.0_tip_1_5	23	low quality protein: basement membrane-specific heparan sulfate proteoglycan core	162	4.03E-57	72.10%	0.096	IPR018031 (SMART); IPR000034 (PFAM); PTHR10574 (PANTHER); PTHR10574:SF252 (PANTHER); IPR000034 (PROSITE_PROFILES)
915	258490_length_488_cvg_57.0_tip_1_3	973	protein	163	2.00E-23	72.60%	0.115	IPR002181 (SMART); IPR014715 (G3DSA:4.10.530.GENE3D); IPR002181 (PFAM); PTHR19143 (PANTHER); IPR020837 (PROSITE_PATTERNS); IPR002181 (PROSITE_PROFILES); IPR002181 (SUPERFAMILY)
916	258510_length_488_cvg_4.0_tip_1_0	28	sucrase- intestinal-like	163	8.87E-31	57.10%	0.104	IPR000322 (PFAM); PTHR22762 (PANTHER); PTHR22762:SF7 (PANTHER); IPR017853 (SUPERFAMILY)
917	258606_length_489_cvg_2.6_tip_1_1	12	sialate o-acetylesterase-like	163	1.66E-15	57.10%	0.192	PTHR22901 (PANTHER); CYTOPLASMIC_DOMAIN (PHOBIUS); NON_CYTOPLASMIC_DOMAIN (PHOBIUS); TRANSMEMBRANE (PHOBIUS); TMhelix (TMHMM); TMhelix (TMHMM)
918	258638_length_489_cvg_2.5_tip_1_3	16	endo-b- -glucanase	163	6.88E-56	76.80%	0.232	IPR012341 (G3DSA:1.50.10.GENE3D); IPR001701 (PFAM); PTHR22298 (PANTHER); PTHR22298:SF17 (PANTHER); IPR018221 (PROSITE_PATTERNS); IPR008928 (SUPERFAMILY)
919	258676_length_490_cvg_42.0_tip_1_5	188	cell wall anchor partial	163	3.37E-17	40.00%	0.244	SSF141571 (SUPERFAMILY)
921	258788_length_491_cvg_2.7_tip_1_2	19	integrin-linked protein kinase	163	2.05E-50	90.40%	0.148	IPR001245 (PFAM); G3DSA:1.10.510.10 (GENE3D); PTHR23257:SF30 (PANTHER); PTHR23257 (PANTHER); IPR000719 (PROSITE_PROFILES); IPR011009 (SUPERFAMILY)
920	258772_length_490_cvg_12.2_tip_1_0	64	anti-lipopolysaccharide partial	164	4.44E-20	68.30%	0.11	IPR024509 (PFAM); IPR024716 (PRODOM)
922	259082_length_493_cvg_3.0_tip_1_5	22	5 -nucleotidase-like	164	9.80E-45	62.70%	0.112	IPR006179 (PRINTS); IPR008334 (PFAM); IPR008334 (G3DSA:3.90.780.GENE3D); IPR006179 (PANTHER); PTHR11575:SF21 (PANTHER); IPR008334 (SUPERFAMILY)
924	259344_length_494_cvg_3.0_tip_1_5	18	lachesin	164	4.48E-76	81.40%	0.129	IPR003599 (SMART); PF13895 (PFAM); IPR013783 (G3DSA:2.60.40.GENE3D); IPR013098 (PFAM); IPR013783 (G3DSA:2.60.40.GENE3D); PTHR19831 (PANTHER); PTHR19831:SF43 (PANTHER); IPR007110 (PROSITE_PROFILES); IPR007110 (PROSITE_PROFILES); SSF48726 (SUPERFAMILY); SSF48726 (SUPERFAMILY)
923	259312_length_494_cvg_63.0_tip_1_3	605	polyubiquitin-b isoform x1	165	3.14E-111	100.00%	0.102	IPR019956 (PRINTS); IPR000626 (SMART); G3DSA:3.10.20.90 (GENE3D); G3DSA:3.10.20.90 (GENE3D); IPR000626 (PFAM); PTHR10666 (PANTHER); IPR019954 (PROSITE_PATTERNS); IPR019954 (PROSITE_PATTERNS); IPR000626 (PROSITE_PROFILES); IPR000626 (PROSITE_PROFILES); IPR000626 (PROSITE_PROFILES); IPR029071 (SUPERFAMILY); IPR029071 (SUPERFAMILY); IPR029071 (SUPERFAMILY)
925	259428_length_495_cvg_46.7_tip_1_3	320	low affinity immunoglobulin epsilon fc receptor	165	6.18E-19	51.60%	0.113	IPR001304 (SMART); IPR001304 (PFAM); IPR016186 (G3DSA:3.10.100.GENE3D); PTHR22802 (PANTHER); IPR018378 (PROSITE_PATTERNS); IPR001304 (PROSITE_PROFILES); IPR016187 (SUPERFAMILY)
927	259944_length_499_cvg_2.0_tip_1_4	13	xanthine dehydrogenase	166	5.33E-45	64.80%	0.101	IPR016167 (G3DSA:3.30.43.GENE3D); IPR002888 (G3DSA:1.10.150.GENE3D); IPR002346 (PFAM); IPR002888 (PFAM); PTHR11908:SF32 (PANTHER); PTHR11908 (PANTHER); IPR016166 (SUPERFAMILY); IPR002888 (SUPERFAMILY)

926	259912_length_499_cvg_5.4_tip_1_3	24	zinc finger protein	167	2.16E-30	56.30%	0.105	IPR015880 (SMART); PF13894 (PFAM); IPR013087 (G3DSA:3.30.160.GENE3D); IPR007087 (PFAM); IPR013087 (G3DSA:3.30.160.GENE3D); PF13465 (PFAM); PTHR24409 (PANTHER); PTHR24409:SF14 (PANTHER); IPR007087 (PROSITE_PATTERNS); IPR007087 (PROSITE_PATTERNS); IPR007087 (PROSITE_PATTERNS); IPR007087 (PROSITE_PATTERNS); IPR007087 (PROSITE_PATTERNS); IPR007087 (PROSITE_PROFILES); IPR007087 (PROSITE_PROFILES); IPR007087 (PROSITE_PROFILES); IPR007087 (PROSITE_PROFILES); IPR007087 (PROSITE_PROFILES); SSF57667 (SUPERFAMILY); SSF57667 (SUPERFAMILY); SSF57667 (SUPERFAMILY)
928	260112_length_500_cvg_3.9_tip_1_1	23	acid sphingomyelinase-like phosphodiesterase 3b	167	1.13E-37	61.90%	0.218	IPR029052 (G3DSA:3.60.21.GENE3D); IPR004843 (PFAM); PTHR10340 (PANTHER); PTHR10340:SF14 (PANTHER); TRANSMEMBRANE (PHOBIUS); NON_CYTOPLASMIC_DOMAIN (PHOBIUS); CYTOPLASMIC_DOMAIN (PHOBIUS); IPR029052 (SUPERFAMILY)
929	260114_length_500_cvg_5.6_tip_1_1	26	low-density lipoprotein receptor-related protein 2	167	8.33E-67	79.00%	0.102	IPR002172 (PRINTS); IPR002172 (SMART); IPR002172 (G3DSA:4.10.400.GENE3D); G3DSA:4.10.1220.10 (GENE3D); IPR002172 (PFAM); IPR002172 (G3DSA:4.10.400.GENE3D); PTHR10529 (PANTHER); IPR023415 (PROSITE_PATTERNS); IPR023415 (PROSITE_PATTERNS); IPR023415 (PROSITE_PATTERNS); IPR002172 (PROSITE_PROFILES); IPR002172 (PROSITE_PROFILES); IPR002172 (PROSITE_PROFILES); IPR002172 (PROSITE_PROFILES); IPR002172 (SUPERFAMILY); IPR002172 (SUPERFAMILY); IPR002172 (SUPERFAMILY); IPR002172 (SUPERFAMILY)
930	260158_length_500_cvg_2.6_tip_1_3	20	clotting factor partial	167	7.42E-31	63.70%	0.136	IPR001254 (SMART); G3DSA:2.40.10.10 (GENE3D); IPR001254 (PFAM); PTHR24265 (PANTHER); IPR001254 (PROSITE_PROFILES); IPR009003 (SUPERFAMILY)
931	260210_length_501_cvg_2.0_tip_1_2	13	tyrosine-protein kinase abl	167	4.41E-55	71.00%	0.133	G3DSA:1.10.510.10 (GENE3D); IPR001245 (PFAM); PTHR24418 (PANTHER); PTHR24418:SF87 (PANTHER); IPR011009 (SUPERFAMILY)
932	260252_length_501_cvg_5.4_tip_1_2	29	multiple epidermal growth factor-like domains partial	167	3.34E-61	69.00%	0.119	PR00011 (PRINTS); IPR000742 (SMART); G3DSA:2.170.300.10 (GENE3D); IPR002049 (PFAM); PTHR24035 (PANTHER); PTHR24035:SF4 (PANTHER); IPR013032 (PROSITE_PATTERNS); IPR013032 (PROSITE_PATTERNS); IPR013032 (PROSITE_PATTERNS); IPR011489 (PROSITE_PROFILES); IPR000742 (PROSITE_PROFILES); IPR000742 (PROSITE_PROFILES); IPR000742 (PROSITE_PROFILES)
933	260262_length_501_cvg_2.9_tip_1_5	19	ankyrin repeat protein partial	167	3.37E-23	54.60%	0.16	IPR002110 (SMART); IPR020683 (PFAM); IPR002110 (PFAM); IPR020683 (G3DSA:1.25.40.GENE3D); PTHR24198 (PANTHER); IPR002110 (PROSITE_PROFILES); IPR002110 (PROSITE_PROFILES); IPR002110 (PROSITE_PROFILES); IPR002110 (PROSITE_PROFILES); IPR002110 (PROSITE_PROFILES); IPR020683 (PROSITE_PROFILES); IPR020683 (SUPERFAMILY)
934	260440_length_502_cvg_2.0_tip_1_4	17	ga-binding protein subunit beta-2	167	4.11E-68	81.90%	0.148	IPR002110 (PRINTS); IPR002110 (SMART); IPR020683 (PFAM); IPR020683 (G3DSA:1.25.40.GENE3D); IPR020683 (G3DSA:1.25.40.GENE3D); IPR002110 (PFAM); PTHR24193 (PANTHER); PTHR24193:SF72 (PANTHER); IPR002110 (PROSITE_PROFILES); IPR002110 (PROSITE_PROFILES); IPR020683 (PROSITE_PROFILES); IPR002110 (PROSITE_PROFILES); IPR020683 (SUPERFAMILY)
935	260520_length_503_cvg_3.0_tip_1_5	15	leucine-rich repeat protein soc-2 homolog	167	8.95E-98	93.70%	0.111	IPR003591 (SMART); IPR001611 (PFAM); G3DSA:3.80.10.10 (GENE3D); IPR001611 (PFAM); PTHR23155 (PANTHER); IPR027036 (PTHR23155:PANTHER); IPR001611 (PROSITE_PROFILES); IPR001611 (PROSITE_PROFILES); IPR001611 (PROSITE_PROFILES); IPR001611 (PROSITE_PROFILES); SSF52058 (SUPERFAMILY)
936	260748_length_505_cvg_4.0_tip_1_1	23	pancreatic triacylglycerol lipase	168	1.89E-24	58.90%	0.585 Y	IPR029058 (G3DSA:3.40.50.GENE3D); IPR013818 (PFAM); IPR000734 (PANTHER); NON_CYTOPLASMIC_DOMAIN (PHOBIUS); SIGNAL_PEPTIDE (PHOBIUS); SIGNAL_PEPTIDE_N_REGION (PHOBIUS); SIGNAL_PEPTIDE_C_REGION (PHOBIUS); SIGNAL_PEPTIDE_H_REGION (PHOBIUS); SignalP-noTM (SIGNALP_EUK); IPR029058 (SUPERFAMILY)
937	260948_length_506_cvg_6.0_tip_1_1	34	von willebrand factor type egf and pentraxin domain-containing protein 1	169	3.33E-10	42.70%	0.101	IPR000436 (SMART); G3DSA:2.10.70.10 (GENE3D); G3DSA:2.10.70.10 (GENE3D); IPR000436 (PFAM); PTHR19325 (PANTHER); IPR000436 (PROSITE_PROFILES); IPR000436 (PROSITE_PROFILES); IPR000436 (SUPERFAMILY); IPR000436 (SUPERFAMILY)
942	261358_length_509_cvg_10.0_tip_1_2	43	low quality protein: tenascin	169	1.04E-25	65.00%	0.106	IPR002181 (SMART); IPR014715 (G3DSA:4.10.530.GENE3D); IPR014716 (G3DSA:3.90.215.GENE3D); IPR002181 (PFAM); PTHR19143 (PANTHER); IPR020837 (PROSITE_PATTERNS); IPR002181 (PROSITE_PROFILES); IPR002181 (SUPERFAMILY)
938	261150_length_508_cvg_3.5_tip_1_3	18	---NA---	170			0.122	IPR001073 (PRINTS); IPR001073 (PFAM); IPR008983 (G3DSA:2.60.120.GENE3D); IPR001073 (PROSITE_PROFILES); IPR008983 (SUPERFAMILY); IPR008983 (SUPERFAMILY)

939	261190_length_508_cvg_3.7_tip_1_0	22	rac protein kinase drac-	170	3.02E-93	87.60%	0.366 Y	IPR000961 (SMART); G3DSA:1.10.510.10 (GENE3D); IPR000719 (PFAM); IPR017892 (PFAM); PTHR24351 (PANTHER); PTHR24351:SF64 (PANTHER); IPR000719 (PROSITE_PROFILES); IPR000961 (PROSITE_PROFILES); IPR011009 (SUPERFAMILY)
940	261192_length_508_cvg_6.0_tip_1_0	31	mannosyl-oligosaccharide -alpha-mannosidase ib	170	4.08E-94	89.00%	0.2	IPR001382 (PRINTS); IPR001382 (G3DSA:1.50.10.GENE3D); IPR001382 (PFAM); IPR001382 (PANTHER); PTHR11742:SF37 (PANTHER); IPR001382 (SUPERFAMILY)
941	261272_length_509_cvg_4.0_tip_1_0	23	von willebrand factor a domain-containing protein partial	170	1.50E-28	55.60%	0.103	PTHR21610:SF9 (PANTHER); PTHR21610 (PANTHER)
943	261400_length_509_cvg_2.8_tip_1_4	13	u4 u6 small nuclear ribonucleoprotein prp4	170	4.56E-76	88.50%	0.238	IPR020472 (PRINTS); IPR001680 (SMART); IPR015943 (G3DSA:2.130.10.GENE3D); IPR001680 (PFAM); IPR027106 (PANTHER); IPR019775 (PROSITE_PATTERNS); IPR001680 (PROSITE_PROFILES); IPR001680 (PROSITE_PROFILES); IPR017986 (PROSITE_PROFILES); IPR001680 (PROSITE_PROFILES); IPR017986 (SUPERFAMILY)
944	261410_length_509_cvg_6.0_tip_1_1	33	phospholipase b-like partial	170	1.65E-76	77.70%	0.136	IPR007000 (PFAM); IPR007000 (PANTHER); PTHR12370:SF3 (PANTHER)
945	261436_length_510_cvg_4.3_tip_1_3	39	parkinson disease 7 domain-containing	170	2.24E-73	72.70%	0.256	IPR002818 (PFAM); IPR029062 (G3DSA:3.40.50.GENE3D); IPR029062 (SUPERFAMILY)
946	261456_length_510_cvg_2.9_tip_1_5	16	vitellogenin receptor	170	7.09E-18	47.90%	0.127	IPR002172 (PRINTS); IPR002172 (SMART); IPR002172 (G3DSA:4.10.400.GENE3D); IPR002172 (PFAM); IPR002172 (G3DSA:4.10.400.GENE3D); IPR002172 (G3DSA:4.10.400.GENE3D); PTHR10529 (PANTHER); IPR023415 (PROSITE_PATTERNS); IPR023415 (PROSITE_PATTERNS); IPR023415 (PROSITE_PATTERNS); IPR002172 (PROSITE_PROFILES); IPR002172 (PROSITE_PROFILES); IPR002172 (PROSITE_PROFILES); IPR002172 (SUPERFAMILY); IPR002172 (SUPERFAMILY); IPR002172 (SUPERFAMILY); IPR002172 (SUPERFAMILY)
948	261652_length_511_cvg_2.0_tip_1_2	16	peptidyl-prolyl cis-trans isomerase-like 4	170	1.62E-77	80.80%	0.103	IPR002130 (PRINTS); IPR002130 (PFAM); IPR029000 (G3DSA:2.40.100.GENE3D); PTHR11071:SF156 (PANTHER); IPR024936 (PANTHER); IPR002130 (PROSITE_PROFILES); IPR029000 (SUPERFAMILY)
950	261702_length_511_cvg_4.0_tip_1_5	27	low quality protein: cubilin- partial	170	3.50E-43	58.80%	0.231	IPR000859 (SMART); IPR000859 (PFAM); IPR000859 (G3DSA:2.60.120.GENE3D); PTHR10127 (PANTHER); IPR000859 (PROSITE_PROFILES); IPR000859 (PROSITE_PROFILES); IPR000859 (SUPERFAMILY); IPR000859 (SUPERFAMILY)
951	261730_length_512_cvg_4.0_tip_1_5	22	arylsulfatase b	170	2.16E-35	57.30%	0.102	IPR017849 (G3DSA:3.40.720.GENE3D); IPR000917 (PFAM); G3DSA:3.30.1120.10 (GENE3D); PTHR10342:SF206 (PANTHER); PTHR10342 (PANTHER); IPR017850 (SUPERFAMILY)
952	261800_length_512_cvg_86.0_tip_1_2	4982	cathepsin l2	170	1.19E-77	80.90%	0.103	IPR000668 (SMART); G3DSA:3.90.70.10 (GENE3D); IPR000668 (PFAM); IPR013128 (PANTHER); PTHR12411:SF265 (PANTHER); IPR025661 (PROSITE_PATTERNS); IPR025660 (PROSITE_PATTERNS); SSF54001 (SUPERFAMILY)
947	261570_length_511_cvg_12.8_tip_1_0	71	signal cub and egf-like domain-containing protein 2	171	4.56E-09	50.70%	0.113	IPR000742 (SMART); IPR001881 (SMART); G3DSA:2.10.25.10 (GENE3D); IPR024731 (PFAM); IPR013032 (PROSITE_PATTERNS); IPR000152 (PROSITE_PATTERNS); IPR013032 (PROSITE_PATTERNS); IPR000742 (PROSITE_PROFILES); SSF57196 (SUPERFAMILY); IPR009030 (SUPERFAMILY)
949	261688_length_511_cvg_9.0_tip_1_0	45	zinc finger protein 233	171	3.06E-32	57.00%	0.113	IPR015880 (SMART); IPR013087 (G3DSA:3.30.160.GENE3D); PF13465 (PFAM); IPR013087 (G3DSA:3.30.160.GENE3D); PTHR24409:SF13 (PANTHER); PTHR24409 (PANTHER); IPR007087 (PROSITE_PATTERNS); IPR007087 (PROSITE_PATTERNS); IPR007087 (PROSITE_PROFILES); IPR007087 (PROSITE_PROFILES); IPR007087 (PROSITE_PROFILES); SSF57667 (SUPERFAMILY); SSF57667 (SUPERFAMILY); SSF57667 (SUPERFAMILY)
953	261878_length_513_cvg_6.2_tip_1_2	39	cubilin isoform x2	171	1.78E-31	56.70%	0.107	IPR000859 (SMART); IPR000859 (PFAM); IPR000859 (G3DSA:2.60.120.GENE3D); PTHR10127 (PANTHER); IPR000859 (PROSITE_PROFILES); IPR000859 (SUPERFAMILY)
954	261944_length_513_cvg_3.0_tip_1_2	15	aael017480- partial	171	2.77E-27	56.10%	0.145	IPR002110 (PRINTS); IPR002110 (SMART); IPR020683 (PFAM); IPR020683 (G3DSA:1.25.40.GENE3D); IPR002110 (PFAM); PTHR24198 (PANTHER); IPR002110 (PROSITE_PROFILES); IPR002110 (PROSITE_PROFILES); IPR002110 (PROSITE_PROFILES); IPR002110 (PROSITE_PROFILES); IPR020683 (PROSITE_PROFILES); IPR020683 (SUPERFAMILY)
955	262022_length_514_cvg_6.1_tip_1_3	37	wd repeat-containing protein 61	172	1.22E-89	90.80%	0.109	IPR020472 (PRINTS); IPR001680 (SMART); IPR015943 (G3DSA:2.130.10.GENE3D); IPR001680 (PFAM); PTHR22841 (PANTHER); IPR001680 (PROSITE_PROFILES); IPR001680 (PROSITE_PROFILES); IPR001680 (PROSITE_PROFILES); IPR017986 (PROSITE_PROFILES); IPR001680 (PROSITE_PROFILES); IPR017986 (SUPERFAMILY)
956	262080_length_514_cvg_38.0_tip_1_0	165	low-density lipoprotein receptor-related protein partial	172	3.72E-07	64.50%	0.12	IPR002172 (SMART); IPR002172 (PFAM); IPR002172 (G3DSA:4.10.400.GENE3D); IPR023415 (PROSITE_PATTERNS); IPR002172 (PROSITE_PROFILES); IPR002172 (SUPERFAMILY)

957	262116_length_515_cvg_4.6_tip_1_0	26	von willebrand factor a domain-containing protein 8	172	2.37E-20	58.40%	0.114	PTHR21610 (PANTHER); PTHR21610:SF9 (PANTHER)
958	262156_length_515_cvg_3.2_tip_1_0	25	diuretic hormone receptor-like isoform x1	172	3.76E-65	77.10%	0.141	IPR002001 (PRINTS); IPR000832 (PRINTS); IPR000832 (PFAM); G3DSA:1.20.1070.10 (GENE3D); PTHR12011 (PANTHER); PTHR12011:SF249 (PANTHER); NON_CYTOPLASMIC_DOMAIN (PHOBIUS); TRANSMEMBRANE (PHOBIUS); CYTOPLASMIC_DOMAIN (PHOBIUS); CYTOPLASMIC_DOMAIN (PHOBIUS); NON_CYTOPLASMIC_DOMAIN (PHOBIUS); TRANSMEMBRANE (PHOBIUS); TRANSMEMBRANE (PHOBIUS); IPR017981 (PROSITE_PROFILES); SSF81321 (SUPERFAMILY); TMhelix (TMHMM); TMhelix (TMHMM); TMhelix (TMHMM)
959	262328_length_516_cvg_33.6_tip_1_2	192	hemagglutinin	172	8.87E-44	70.80%	0.098	G3DSA:1.10.390.10 (GENE3D); IPR001570 (PFAM); SSF55486 (SUPERFAMILY)
960	262426_length_517_cvg_15.7_tip_1_0	93	md-2-related lipid-recognition	173	2.45E-24	53.50%	0.785 Y	IPR003172 (SMART); IPR003172 (PFAM); IPR003172 (G3DSA:2.60.40.GENE3D); PTHR11306 (PANTHER); PTHR11306:SF4 (PANTHER); SIGNAL_PEPTIDE (PHOBIUS); SIGNAL_PEPTIDE_C_REGION (PHOBIUS); SIGNAL_PEPTIDE_H_REGION (PHOBIUS); NON_CYTOPLASMIC_DOMAIN (PHOBIUS); SIGNAL_PEPTIDE_N_REGION (PHOBIUS); SignalP-noTM (SIGNALP_GRAM_NEGATIVE); SignalP-noTM (SIGNALP_EUK); SignalP-TM (SIGNALP_GRAM_POSITIVE); IPR014756 (SUPERFAMILY); TMhelix (TMHMM)
961	262740_length_519_cvg_2.9_tip_1_5	17	centaurin-gamma 1a	173	2.02E-74	72.10%	0.164	IPR002110 (SMART); IPR020683 (PFAM); IPR020683 (G3DSA:1.25.40.GENE3D); IPR001164 (PFAM); PTHR23180 (PANTHER); PTHR23180:SF229 (PANTHER); IPR020683 (PROSITE_PROFILES); IPR002110 (PROSITE_PROFILES); SSF57863 (SUPERFAMILY); IPR020683 (SUPERFAMILY)
962	262746_length_519_cvg_7.0_tip_1_5	46	alpha-2-macroglobulin-like protein 1	173	1.37E-53	71.10%	0.105	IPR019565 (PFAM); IPR008930 (G3DSA:1.50.10.GENE3D); PTHR11412 (PANTHER); IPR019742 (PROSITE_PATTERNS); IPR008930 (SUPERFAMILY)
963	262750_length_519_cvg_3.9_tip_1_0	29	glypican-5 isoform x5	173	1.06E-33	57.10%	0.192	IPR001863 (PFAM); IPR001863 (PANTHER)
964	262808_length_520_cvg_6.0_tip_1_2	34	von willebrand factor a domain-containing protein 8-like	173	3.31E-85	85.80%	0.125	PTHR21610 (PANTHER); PTHR21610:SF9 (PANTHER)
965	262902_length_520_cvg_3.0_tip_1_4	18	plexin- partial	173	7.77E-99	93.70%	0.144	IPR013548 (PFAM); PTHR22625 (PANTHER); IPR008936 (SUPERFAMILY)
966	263080_length_522_cvg_6.0_tip_1_2	34	ankyrin repeat domain-containing protein 49-like	174	1.41E-56	71.10%	0.134	IPR002110 (PRINTS); IPR002110 (SMART); IPR002110 (PFAM); IPR020683 (G3DSA:1.25.40.GENE3D); IPR020683 (PFAM); PTHR24144 (PANTHER); IPR002110 (PROSITE_PROFILES); IPR002110 (PROSITE_PROFILES); IPR020683 (PROSITE_PROFILES); IPR002110 (PROSITE_PROFILES); IPR020683 (SUPERFAMILY)
967	263084_length_522_cvg_14.0_tip_1_0	83	wd repeat-containing protein 86-like	174	4.95E-43	72.50%	0.16	IPR020472 (PRINTS); IPR001680 (SMART); IPR015943 (G3DSA:2.130.10.GENE3D); IPR001680 (PFAM); PTHR22844 (PANTHER); PTHR22844:SF166 (PANTHER); IPR001680 (PROSITE_PROFILES); IPR017986 (PROSITE_PROFILES); IPR001680 (PROSITE_PROFILES); IPR017986 (SUPERFAMILY)
968	263164_length_522_cvg_8.9_tip_1_5	50	cytochrome p450 partial	174	4.43E-52	66.60%	0.1	IPR001128 (PRINTS); IPR002401 (PRINTS); IPR001128 (PFAM); IPR001128 (G3DSA:1.10.630.GENE3D); PTHR24291 (PANTHER); IPR017972 (PROSITE_PATTERNS); IPR001128 (SUPERFAMILY)
969	263188_length_522_cvg_3.2_tip_1_5	24	enoyl- delta isomerase mitochondrial	174	6.37E-45	67.20%	0.179	Coil (COILS); IPR000582 (PRINTS); IPR029045 (G3DSA:3.90.226.GENE3D); IPR000582 (PFAM); IPR014352 (G3DSA:1.20.80.GENE3D); IPR001753 (PFAM); PTHR23310 (PANTHER); PTHR23310:SF55 (PANTHER); IPR022408 (PROSITE_PATTERNS); IPR000582 (PROSITE_PROFILES); IPR029045 (SUPERFAMILY); IPR000582 (SUPERFAMILY)
971	263214_length_523_cvg_5.2_tip_1_4	39	betaine aldehyde dehydrogenase	174	6.74E-36	80.20%	0.128	IPR016163 (G3DSA:3.40.309.GENE3D); IPR015590 (PFAM); PTHR11699 (PANTHER); PTHR11699:SF114 (PANTHER); IPR016161 (SUPERFAMILY)
972	263216_length_523_cvg_3.0_tip_1_4	16	low quality protein: low-density lipoprotein receptor-related protein 1- partial	174	3.22E-23	56.10%	0.354 Y	IPR002172 (PRINTS); IPR002172 (SMART); IPR002172 (G3DSA:4.10.400.GENE3D); IPR002172 (G3DSA:4.10.400.GENE3D); IPR002172 (PFAM); PTHR10529 (PANTHER); IPR023415 (PROSITE_PATTERNS); IPR023415 (PROSITE_PATTERNS); IPR023415 (PROSITE_PATTERNS); CYTOPLASMIC_DOMAIN (PHOBIUS); TRANSMEMBRANE (PHOBIUS); NON_CYTOPLASMIC_DOMAIN (PHOBIUS); IPR002172 (PROSITE_PROFILES); IPR002172 (PROSITE_PROFILES); IPR002172 (PROSITE_PROFILES); IPR002172 (SUPERFAMILY); IPR002172 (SUPERFAMILY); IPR002172 (SUPERFAMILY); TMhelix (TMHMM)
970	263212_length_523_cvg_2.0_tip_1_3	14	bifunctional protein ncoat isoform x2	175	1.03E-81	92.20%	0.201	IPR011496 (PFAM); PTHR13170 (PANTHER); PTHR13170:SF17 (PANTHER); IPR017853 (SUPERFAMILY)
973	263374_length_524_cvg_42.1_tip_1_3	228	hemagglutinin amebocyte aggregation factor-like	175	1.37E-23	57.40%	0.194	PF14704 (PFAM); IPR026645 (PANTHER); TRANSMEMBRANE (PHOBIUS); CYTOPLASMIC_DOMAIN (PHOBIUS); NON_CYTOPLASMIC_DOMAIN (PHOBIUS); TMhelix (TMHMM)

974	263484_length_524_cvg_2.1_tip_1_1	20	adenosine deaminase cecr1-like	175	1.07E-34	63.50%	0.367 Y	IPR013659 (PFAM); PTHR11409:SF39 (PANTHER); PTHR11409 (PANTHER); NON_CYTOPLASMIC_DOMAIN (PHOBIUS); SIGNAL_PEPTIDE_C_REGION (PHOBIUS); SIGNAL_PEPTIDE_H_REGION (PHOBIUS); SIGNAL_PEPTIDE (PHOBIUS); SIGNAL_PEPTIDE_N_REGION (PHOBIUS); SSF51556 (SUPERFAMILY); TMhelix (TMHMM)
975	263584_length_525_cvg_2.8_tip_1_0	23	map kinase-activated protein kinase 2	175	2.39E-102	88.90%	0.113	IPR002290 (SMART); G3DSA:1.10.510.10 (GENE3D); G3DSA:3.30.200.20 (GENE3D); IPR000719 (PFAM); PTHR24349:SF64 (PANTHER); PTHR24349 (PANTHER); IPR008271 (PROSITE_PATTERNS); IPR017441 (PROSITE_PATTERNS); IPR000719 (PROSITE_PROFILES); IPR011009 (SUPERFAMILY)
976	263706_length_526_cvg_5.6_tip_1_1	41	protein disulfide-isomerase a4-like	175	1.03E-57	67.40%	0.916 Y	PR00421 (PRINTS); IPR013766 (PFAM); IPR005788 (TIGRFAM); IPR012336 (G3DSA:3.40.30.GENE3D); PTHR18929:SF56 (PANTHER); PTHR18929 (PANTHER); IPR017937 (PROSITE_PATTERNS); SIGNAL_PEPTIDE_H_REGION (PHOBIUS); SIGNAL_PEPTIDE (PHOBIUS); SIGNAL_PEPTIDE_N_REGION (PHOBIUS); SIGNAL_PEPTIDE_C_REGION (PHOBIUS); NON_CYTOPLASMIC_DOMAIN (PHOBIUS); IPR012336 (PROSITE_PROFILES); SignalP-noTM (SIGNALP_EUK); SignalP-noTM (SIGNALP_GRAM_NEGATIVE); IPR012336 (SUPERFAMILY)
977	263732_length_526_cvg_54.5_tip_1_0	1385	ubiquitin-60s ribosomal protein l40	176	1.59E-85	99.20%	0.16	IPR019956 (PRINTS); IPR000626 (SMART); G3DSA:3.10.20.90 (GENE3D); IPR000626 (PFAM); IPR001975 (PFAM); PTHR10666 (PANTHER); IPR019954 (PROSITE_PATTERNS); IPR000626 (PROSITE_PROFILES); IPR029071 (SUPERFAMILY); IPR011332 (SUPERFAMILY)
978	263748_length_527_cvg_3.8_tip_1_1	22	low quality protein: reelin-like	176	2.57E-29	54.50%	0.219	PTHR11841 (PANTHER)
979	263918_length_528_cvg_5.4_tip_1_3	33	ga-binding protein subunit beta-2	176	2.51E-10	68.30%	0.117	IPR002110 (SMART); IPR020683 (PFAM); IPR020683 (G3DSA:1.25.40.GENE3D); PTHR24182 (PANTHER); IPR020683 (PROSITE_PROFILES); IPR002110 (PROSITE_PROFILES); IPR020683 (SUPERFAMILY)
984	264200_length_530_cvg_19.5_tip_1_5	141	hypothetical protein	176	7.03E-13	50.40%	0.383 Y	CYTOPLASMIC_DOMAIN (PHOBIUS); TRANSMEMBRANE (PHOBIUS); NON_CYTOPLASMIC_DOMAIN (PHOBIUS); TMhelix (TMHMM)
980	264012_length_529_cvg_15.4_tip_1_0	79	lysosomal alpha-mannosidase-like	177	7.20E-31	67.60%	0.137	IPR011682 (PFAM); G3DSA:2.60.40.1360 (GENE3D); PTHR11607:SF3 (PANTHER); PTHR11607 (PANTHER); IPR011013 (SUPERFAMILY)
981	264042_length_529_cvg_22.6_tip_1_0	166	bone sialoprotein-binding partial	177	1.76E-08	49.00%	0.106	no IPS match
982	264096_length_529_cvg_2.9_tip_1_3	16	achain crystal structure of engineered northeast structural genomics consortium target	177	1.12E-11	64.60%	0.123	IPR002110 (SMART); IPR020683 (G3DSA:1.25.40.GENE3D); IPR020683 (PFAM); PTHR24182 (PANTHER); IPR020683 (PROSITE_PROFILES); IPR002110 (PROSITE_PROFILES); IPR002110 (PROSITE_PROFILES); IPR020683 (SUPERFAMILY)
983	264134_length_530_cvg_2.0_tip_1_0	18	beta-glucuronidase isoform x2	177	1.00E-74	75.60%	0.127	IPR006101 (PRINTS); IPR006103 (PFAM); IPR013781 (G3DSA:3.20.20.GENE3D); PTHR10066:SF51 (PANTHER); PTHR10066 (PANTHER); IPR023230 (PROSITE_PATTERNS); IPR023232 (PROSITE_PATTERNS); IPR017853 (SUPERFAMILY)
985	264300_length_531_cvg_6.1_tip_1_1	39	sortilin-related receptor	177	1.73E-10	41.60%	0.1	IPR002172 (PRINTS); IPR002172 (SMART); IPR002172 (PFAM); IPR002172 (G3DSA:4.10.400.GENE3D); PTHR10529 (PANTHER); IPR023415 (PROSITE_PATTERNS); IPR023415 (PROSITE_PATTERNS); IPR002172 (PROSITE_PROFILES); IPR002172 (PROSITE_PROFILES); IPR002172 (SUPERFAMILY); IPR002172 (SUPERFAMILY)
986	264324_length_531_cvg_3.9_tip_1_0	30	zinc finger protein	177	4.77E-18	55.90%	0.103	IPR015880 (SMART); PF13465 (PFAM); IPR013087 (G3DSA:3.30.160.GENE3D); IPR003656 (PFAM); IPR013087 (G3DSA:3.30.160.GENE3D); PTHR24409:SF14 (PANTHER); PTHR24409 (PANTHER); IPR007087 (PROSITE_PATTERNS); IPR007087 (PROSITE_PATTERNS); IPR007087 (PROSITE_PATTERNS); IPR007087 (PROSITE_PROFILES); IPR007087 (PROSITE_PROFILES); IPR007087 (PROSITE_PROFILES); SSF57667 (SUPERFAMILY); SSF57667 (SUPERFAMILY)
987	264366_length_532_cvg_3.0_tip_1_1	21	short neuropeptide f	177	1.51E-22	68.60%	0.211	no IPS match
990	264526_length_533_cvg_3.9_tip_1_5	25	integrin alpha-ps2-like	177	1.62E-39	61.30%	0.141	IPR000413 (PRINTS); IPR013519 (SMART); G3DSA:2.130.10.130 (GENE3D); IPR013517 (PFAM); PTHR23220 (PANTHER); IPR013519 (PROSITE_PROFILES); IPR013519 (PROSITE_PROFILES); SSF69318 (SUPERFAMILY)
988	264492_length_533_cvg_32.3_tip_0_4	206	neurotransmitter gated ion channel	178	4.54E-53	66.30%	0.139	IPR006202 (PFAM); IPR006202 (G3DSA:2.70.170.GENE3D); IPR027361 (G3DSA:1.20.120.GENE3D); PTHR18945:SF490 (PANTHER); IPR006201 (PANTHER); CYTOPLASMIC_DOMAIN (PHOBIUS); TRANSMEMBRANE (PHOBIUS); NON_CYTOPLASMIC_DOMAIN (PHOBIUS); IPR006202 (SUPERFAMILY); IPR006029 (SUPERFAMILY); TMhelix (TMHMM)

989	264508_length_533_cvg_3.7_tip_1_1	22	serine threonine-protein phosphatase 6 regulatory ankyrin repeat subunit c-like	178	9.02E-28	54.70%	0.136	IPR002110 (PRINTS); IPR002110 (SMART); IPR020683 (PFAM); IPR020683 (G3DSA:1.25.40.GENE3D); PTHR24198 (PANTHER); IPR002110 (PROSITE_PROFILES); IPR020683 (PROSITE_PROFILES); IPR002110 (PROSITE_PROFILES); IPR002110 (PROSITE_PROFILES); IPR002110 (PROSITE_PROFILES); IPR020683 (SUPERFAMILY)
991	264620_length_534_cvg_2.1_tip_1_5	16	dentin sialophosphoprotein isoform x1	178	1.32E-59	71.20%	0.107	IPR015880 (SMART); PF13465 (PFAM); IPR013087 (G3DSA:3.30.160.GENE3D); IPR013087 (G3DSA:3.30.160.GENE3D); IPR013087 (G3DSA:3.30.160.GENE3D); PTHR24409 (PANTHER); IPR007087 (PROSITE_PATTERNS); IPR007087 (PROSITE_PATTERNS); IPR007087 (PROSITE_PATTERNS); IPR007087 (PROSITE_PATTERNS); IPR007087 (PROSITE_PROFILES); IPR007087 (PROSITE_PROFILES); IPR007087 (PROSITE_PROFILES); IPR007087 (PROSITE_PROFILES); IPR007087 (PROSITE_PROFILES); IPR007087 (PROSITE_PROFILES); SSF57667 (SUPERFAMILY); SSF57667 (SUPERFAMILY); SSF57667 (SUPERFAMILY)
992	264668_length_534_cvg_3.0_tip_1_4	18	aldehyde dehydrogenase	178	5.94E-66	69.00%	0.183	IPR016163 (G3DSA:3.40.309.GENE3D); IPR015590 (PFAM); PTHR11699 (PANTHER); IPR015657 (PTHR11699:PANTHER); IPR016161 (SUPERFAMILY); TMhelix (TMHMM)
995	264776_length_535_cvg_2.7_tip_1_4	16	zinc finger protein 239-like	178	2.05E-24	60.00%	0.106	IPR015880 (SMART); PF13465 (PFAM); IPR013087 (G3DSA:3.30.160.GENE3D); IPR013087 (G3DSA:3.30.160.GENE3D); IPR013087 (G3DSA:3.30.160.GENE3D); PF13912 (PFAM); PTHR24409 (PANTHER); IPR007087 (PROSITE_PATTERNS); IPR007087 (PROSITE_PATTERNS); IPR007087 (PROSITE_PROFILES); IPR007087 (PROSITE_PROFILES); IPR007087 (PROSITE_PROFILES); SSF57667 (SUPERFAMILY); SSF57667 (SUPERFAMILY)
997	264798_length_536_cvg_17.6_tip_1_2	105	membrane glycoprotein lig-	178	2.46E-23	52.60%	0.106	IPR003591 (SMART); IPR001611 (PFAM); G3DSA:3.80.10.10 (GENE3D); IPR026906 (PFAM); G3DSA:3.80.10.10 (GENE3D); PTHR24365 (PANTHER); IPR001611 (PROSITE_PROFILES); IPR001611 (PROSITE_PROFILES); IPR001611 (PROSITE_PROFILES); IPR001611 (PROSITE_PROFILES); IPR001611 (PROSITE_PROFILES); IPR001611 (PROSITE_PROFILES); SSF52058 (SUPERFAMILY)
993	264700_length_535_cvg_17.3_tip_1_0	93	apolipoprotein d-like	179	8.20E-34	61.20%	0.363 Y	IPR003057 (PRINTS); IPR012674 (G3DSA:2.40.128.GENE3D); PTHR10612:SF7 (PANTHER); PTHR10612 (PANTHER); TRANSMEMBRANE (PHOBIUS); CYTOPLASMIC_DOMAIN (PHOBIUS); NON_CYTOPLASMIC_DOMAIN (PHOBIUS); IPR011038 (SUPERFAMILY); TMhelix (TMHMM)
994	264740_length_535_cvg_2.0_tip_1_3	15	caseinolytic peptidase b protein homolog	179	7.79E-53	75.70%	0.099	IPR002110 (SMART); IPR002110 (PFAM); IPR020683 (G3DSA:1.25.40.GENE3D); IPR020683 (PFAM); PTHR24193 (PANTHER); IPR002110 (PROSITE_PROFILES); IPR002110 (PROSITE_PROFILES); IPR020683 (PROSITE_PROFILES); IPR020683 (SUPERFAMILY)
996	264778_length_535_cvg_11.0_tip_1_0	64	ribosome biogenesis protein wdr12 homolog	179	5.03E-83	80.40%	0.157	IPR020472 (PRINTS); IPR001680 (SMART); IPR015943 (G3DSA:2.130.10.GENE3D); IPR001680 (PFAM); PTHR19855 (PANTHER); IPR019775 (PROSITE_PATTERNS); IPR001680 (PROSITE_PROFILES); IPR017986 (PROSITE_PROFILES); IPR001680 (PROSITE_PROFILES); IPR001680 (PROSITE_PROFILES); IPR017986 (SUPERFAMILY)
998	264824_length_536_cvg_5.9_tip_1_4	34	low affinity immunoglobulin epsilon fc receptor-like	179	3.28E-17	46.40%	0.132	IPR001304 (SMART); IPR001304 (PFAM); IPR016186 (G3DSA:3.10.100.GENE3D); PTHR22802 (PANTHER); IPR001304 (PROSITE_PROFILES); IPR016187 (SUPERFAMILY)
999	265016_length_537_cvg_2.0_tip_1_3	15	chorion peroxidase	179	2.84E-25	67.80%	0.12	IPR019791 (PFAM); IPR019791 (G3DSA:1.10.640.GENE3D); PTHR11475 (PANTHER); IPR019791 (PROSITE_PROFILES); IPR010255 (SUPERFAMILY)
1001	265202_length_539_cvg_19.7_tip_1_2	109	peptidyl-prolyl cis-trans isomerase b	179	3.66E-43	54.60%	0.109	IPR024936 (PIRSF); IPR029000 (G3DSA:2.40.100.GENE3D); IPR002130 (PFAM); PTHR11071:SF78 (PANTHER); IPR024936 (PANTHER); IPR002130 (PROSITE_PROFILES); IPR029000 (SUPERFAMILY)
1002	265266_length_539_cvg_3.9_tip_1_2	26	aael017046- partial	179	2.08E-59	74.30%	0.920 Y	IPR021190 (PRINTS); IPR002477 (PFAM); IPR002477 (G3DSA:1.10.101.GENE3D); IPR001818 (PFAM); IPR024079 (G3DSA:3.40.390.GENE3D); PTHR10201:SF131 (PANTHER); PTHR10201 (PANTHER); SIGNAL_PEPTIDE_H_REGION (PHOBIUS); SIGNAL_PEPTIDE_C_REGION (PHOBIUS); NON_CYTOPLASMIC_DOMAIN (PHOBIUS); SIGNAL_PEPTIDE (PHOBIUS); SIGNAL_PEPTIDE_N_REGION (PHOBIUS); SignalP-noTM (SIGNALP_EUK); IPR002477 (SUPERFAMILY); SSF55486 (SUPERFAMILY); TMhelix (TMHMM)
1000	265166_length_539_cvg_4.6_tip_1_4	29	low quality protein: neurogenic locus notch homolog protein 3-like	180	8.45E-52	65.50%	0.101	IPR000742 (SMART); IPR013032 (PFAM); G3DSA:2.10.25.10 (GENE3D)
1003	265272_length_539_cvg_4.0_tip_1_3	26	alcohol dehydrogenase	180	1.32E-51	71.80%	0.182	IPR020471 (PRINTS); IPR023210 (PFAM); IPR023210 (G3DSA:3.20.20.GENE3D); IPR001395 (PANTHER); PTHR11732:SF183 (PANTHER); IPR018170 (PROSITE_PATTERNS); IPR023210 (SUPERFAMILY)
1004	265302_length_540_cvg_4.2_tip_1_0	26	attractin-like protein 1	180	3.81E-81	79.80%	0.1	G3DSA:2.10.25.10 (GENE3D); PTHR10574:SF3 (PANTHER); PTHR10574 (PANTHER)

1005	265314_length_540_cvg_73.1_tip_1_4	1003	carboxypeptidase b-like	180	1.69E-64	71.80%	0.118	IPR000834 (SMART); IPR000834 (PFAM); G3DSA:3.40.630.10 (GENE3D); PTHR11705 (PANTHER); SSF53187 (SUPERFAMILY)
1006	265338_length_540_cvg_2.0_tip_1_3	16	peroxisomal biogenesis factor 7	180	2.14E-81	76.10%	0.102	IPR020472 (PRINTS); IPR001680 (SMART); IPR015943 (G3DSA:2.130.10.GENE3D); IPR001680 (PFAM); PTHR22850 (PANTHER); PTHR22850:SF98 (PANTHER); IPR019775 (PROSITE_PATTERNS); IPR019775 (PROSITE_PATTERNS); IPR001680 (PROSITE_PROFILES); IPR001680 (PROSITE_PROFILES); IPR017986 (PROSITE_PROFILES); IPR001680 (PROSITE_PROFILES); IPR001680 (PROSITE_PROFILES); IPR017986 (SUPERFAMILY)
1007	265358_length_540_cvg_5.3_tip_1_2	40	alpha-2-macroglobulin-like protein 1	180	1.37E-21	47.80%	0.22	IPR011625 (PFAM); PTHR11412 (PANTHER)
1008	265556_length_542_cvg_61.6_tip_1_0	18917	vitellogenin 2	181	1.31E-22	53.40%	0.540 Y	IPR015816 (G3DSA:2.30.230.GENE3D); IPR001747 (PFAM); PTHR23345 (PANTHER); TRANSMEMBRANE (PHOBIUS); CYTOPLASMIC_DOMAIN (PHOBIUS); NON_CYTOPLASMIC_DOMAIN (PHOBIUS); SignalP-TM (SIGNALP_EUK); IPR015819 (SUPERFAMILY); TMhelix (TMHMM)
1009	265608_length_542_cvg_5.3_tip_1_3	29	c4b-binding protein beta	181	6.74E-59	66.40%	0.108	IPR000436 (SMART); G3DSA:2.10.70.10 (GENE3D); IPR000436 (PFAM); G3DSA:2.10.70.10 (GENE3D); PTHR19325 (PANTHER); PTHR19325:SF312 (PANTHER); IPR000436 (PROSITE_PROFILES); IPR000436 (PROSITE_PROFILES); IPR000436 (SUPERFAMILY); IPR000436 (SUPERFAMILY)
1010	265698_length_543_cvg_3.4_tip_1_5	22	gastrula zinc finger	181	9.02E-23	63.50%	0.097	IPR015880 (SMART); IPR013087 (G3DSA:3.30.160.GENE3D); PF13465 (PFAM); IPR013087 (G3DSA:3.30.160.GENE3D); IPR013087 (G3DSA:3.30.160.GENE3D); PTHR24409 (PANTHER); IPR007087 (PROSITE_PATTERNS); IPR007087 (PROSITE_PATTERNS); IPR007087 (PROSITE_PATTERNS); IPR007087 (PROSITE_PROFILES); IPR007087 (PROSITE_PROFILES); IPR007087 (PROSITE_PROFILES); SSF57667 (SUPERFAMILY); SSF57667 (SUPERFAMILY)
1011	265786_length_544_cvg_4.0_tip_1_2	28	peroxidasin homolog	181	1.69E-16	46.40%	0.112	IPR019791 (G3DSA:1.10.640.GENE3D); IPR019791 (G3DSA:1.10.640.GENE3D); IPR019791 (PFAM); PTHR11475 (PANTHER); IPR019791 (PROSITE_PROFILES); IPR010255 (SUPERFAMILY)
1012	265804_length_544_cvg_2.0_tip_1_5	13	von willebrand factor d and egf domain-containing	181	7.04E-47	58.10%	0.118	IPR000742 (SMART); IPR000742 (PFAM); G3DSA:2.170.300.10 (GENE3D); IPR013032 (PFAM); PTHR24838 (PANTHER); IPR013032 (PROSITE_PATTERNS); IPR013032 (PROSITE_PATTERNS); IPR013032 (PROSITE_PATTERNS); IPR013032 (PROSITE_PATTERNS); IPR013032 (PROSITE_PATTERNS); IPR013032 (PROSITE_PATTERNS); IPR013032 (PROSITE_PATTERNS); IPR013032 (PROSITE_PATTERNS); IPR000742 (PROSITE_PROFILES); IPR000742 (PROSITE_PROFILES); IPR000742 (PROSITE_PROFILES); SSF57196 (SUPERFAMILY); SSF57196 (SUPERFAMILY)
1013	265840_length_544_cvg_2.2_tip_1_1	22	group xiia secretory phospholipase a2	181	1.80E-52	63.10%	0.659 Y	IPR016090 (G3DSA:1.20.90.GENE3D); IPR010711 (PFAM); PTHR12824:SF8 (PANTHER); IPR010711 (PANTHER); IPR013090 (PROSITE_PATTERNS); SIGNAL_PEPTIDE_C_REGION (PHOBIUS); SIGNAL_PEPTIDE_H_REGION (PHOBIUS); SIGNAL_PEPTIDE (PHOBIUS); NON_CYTOPLASMIC_DOMAIN (PHOBIUS); SIGNAL_PEPTIDE_N_REGION (PHOBIUS); SignalP-noTM (SIGNALP_EUK); IPR016090 (SUPERFAMILY); TMhelix (TMHMM)
1014	265852_length_544_cvg_2.0_tip_1_1	11	rna polymerase ii largest subunit	181	7.19E-47	84.90%	0.097	PR01217 (PRINTS); IPR000684 (PFAM); IPR000684 (PROSITE_PATTERNS); IPR000684 (PROSITE_PATTERNS); IPR000684 (PROSITE_PATTERNS); IPR000684 (PROSITE_PATTERNS); IPR000684 (PROSITE_PATTERNS); IPR000684 (PROSITE_PATTERNS); IPR000684 (PROSITE_PATTERNS); IPR000684 (PROSITE_PATTERNS); IPR000684 (PROSITE_PATTERNS); IPR000684 (PROSITE_PATTERNS); IPR000684 (PROSITE_PATTERNS); IPR000684 (PROSITE_PATTERNS); IPR000684 (PROSITE_PATTERNS)
1015	265928_length_545_cvg_3.4_tip_1_1	25	dehydrogenase reductase sdr family protein 7-like	182	8.87E-87	84.10%	0.104	IPR002347 (PRINTS); IPR016040 (G3DSA:3.40.50.GENE3D); IPR002198 (PFAM); PTHR24322:SF325 (PANTHER); PTHR24322 (PANTHER); IPR020904 (PROSITE_PATTERNS); NON_CYTOPLASMIC_DOMAIN (PHOBIUS); CYTOPLASMIC_DOMAIN (PHOBIUS); TRANSMEMBRANE (PHOBIUS); SSF51735 (SUPERFAMILY)
1016	266006_length_546_cvg_11.4_tip_1_3	62	serpin b3	182	1.86E-29	67.10%	0.123	G3DSA:2.30.39.10 (GENE3D); IPR023796 (PFAM); G3DSA:3.30.497.10 (GENE3D); IPR000215 (PANTHER); IPR023795 (PROSITE_PATTERNS); IPR023796 (SUPERFAMILY)
1017	266082_length_546_cvg_3.5_tip_1_1	22	mam and ldl-receptor class a domain-containing protein 1-like	182	4.22E-15	48.00%	0.119	IPR000998 (SMART); IPR000998 (PFAM); IPR000998 (PROSITE_PROFILES); IPR013320 (SUPERFAMILY)
1021	266184_length_547_cvg_25.1_tip_1_2	88	gamma-glutamyl hydrolase-like	182	3.57E-24	69.70%	0.203	IPR011697 (PFAM); IPR029062 (G3DSA:3.40.50.GENE3D); PTHR11315:SF2 (PANTHER); IPR015527 (PANTHER); IPR015527 (PROSITE_PROFILES)
1023	266228_length_548_cvg_12.2_tip_1_5	57	hypothetical protein	182	2.31E-08	74.17%	0.117	IPR010790 (PFAM)

1018	266112_length_547_cvg_3.0_tip_1_3	22	isoform b	183	8.95E-31	65.60%	0.518	Y	IPR002347 (PRINTS); IPR002198 (PFAM); IPR016040 (G3DSA:3.40.50.GENE3D); PTHR24320:SF38 (PANTHER); PTHR24320 (PANTHER); SIGNAL_PEPTIDE_N_REGION (PHOBIUS); SIGNAL_PEPTIDE_H_REGION (PHOBIUS); NON_CYTOPLASMIC_DOMAIN (PHOBIUS); SIGNAL_PEPTIDE_C_REGION (PHOBIUS); SIGNAL_PEPTIDE (PHOBIUS); SSF51735 (SUPERFAMILY); TMhelix (TMHMM)
1019	266146_length_547_cvg_6.3_tip_1_0	44	muscle m-line assembly protein unc-89	183	1.80E-47	66.90%	0.103		IPR003598 (SMART); IPR003599 (SMART); IPR013783 (G3DSA:2.60.40.GENE3D); IPR013783 (G3DSA:2.60.40.GENE3D); IPR013098 (PFAM); PTHR19897 (PANTHER); IPR007110 (PROSITE_PROFILES); IPR007110 (PROSITE_PROFILES); SSF48726 (SUPERFAMILY); SSF48726 (SUPERFAMILY)
1020	266150_length_547_cvg_27.9_tip_1_3	174	translocase of inner mitochondrial membrane 8 homolog b	183	2.05E-33	78.30%	0.124		IPR004217 (G3DSA:1.10.287.GENE3D); IPR004217 (PFAM); PTHR21535 (PANTHER); PTHR21535:SF25 (PANTHER); CYTOPLASMIC_DOMAIN (PHOBIUS); NON_CYTOPLASMIC_DOMAIN (PHOBIUS); TRANSMEMBRANE (PHOBIUS); IPR004217 (SUPERFAMILY); TMhelix (TMHMM)
1022	266212_length_547_cvg_3.0_tip_1_3	18	insulin receptor	183	7.00E-102	88.00%	0.103		IPR001245 (PRINTS); IPR020635 (SMART); IPR001245 (PFAM); G3DSA:1.10.510.10 (GENE3D); G3DSA:3.30.200.20 (GENE3D); PTHR24416:SF254 (PANTHER); PTHR24416 (PANTHER); IPR002011 (PROSITE_PATTERNS); IPR008266 (PROSITE_PATTERNS); IPR000719 (PROSITE_PROFILES); IPR011009 (SUPERFAMILY)
1024	266258_length_548_cvg_3.0_tip_1_3	18	venom protease-like	183	2.11E-43	59.00%	0.267		IPR001314 (PRINTS); IPR001254 (SMART); G3DSA:2.40.10.10 (GENE3D); G3DSA:2.40.10.10 (GENE3D); G3DSA:2.40.10.10 (GENE3D); IPR001254 (PFAM); PTHR24268:SF81 (PANTHER); PTHR24268 (PANTHER); IPR018114 (PROSITE_PATTERNS); IPR001254 (PROSITE_PROFILES); IPR009003 (SUPERFAMILY)
1025	266274_length_548_cvg_2.0_tip_1_4	16	antigen 5 scp domain-containing	183	9.84E-12	56.50%	0.311		IPR014044 (G3DSA:3.40.33.GENE3D); IPR014044 (PFAM); IPR001283 (PANTHER); IPR014044 (SUPERFAMILY)
1026	266284_length_548_cvg_60.0_tip_1_0	514	---NA---	183			0.187		Coil (COILS); IPR014716 (G3DSA:3.90.215.GENE3D); IPR002181 (PFAM); TRANSMEMBRANE (PHOBIUS); CYTOPLASMIC_DOMAIN (PHOBIUS); NON_CYTOPLASMIC_DOMAIN (PHOBIUS); IPR002181 (PROSITE_PROFILES); IPR002181 (SUPERFAMILY); TMhelix (TMHMM)
1027	266370_length_549_cvg_3.3_tip_1_4	20	third variable lymphocyte receptor	183	2.55E-11	51.00%	0.18		G3DSA:3.80.10.10 (GENE3D); IPR001611 (PFAM); PTHR24365 (PANTHER); SSF52058 (SUPERFAMILY)
1028	266408_length_549_cvg_4.0_tip_1_4	28	low quality protein: titin-like	183	2.83E-47	62.10%	0.101		IPR013098 (PFAM); IPR013783 (G3DSA:2.60.40.GENE3D); IPR013783 (G3DSA:2.60.40.GENE3D); PTHR19897 (PANTHER); IPR007110 (PROSITE_PROFILES); SSF48726 (SUPERFAMILY); SSF48726 (SUPERFAMILY)
1029	266458_length_550_cvg_9.6_tip_1_4	66	integrin-linked protein kinase	183	5.15E-42	91.20%	0.227		IPR002110 (SMART); IPR020683 (G3DSA:1.25.40.GENE3D); IPR020683 (PFAM); PTHR23257:SF30 (PANTHER); PTHR23257 (PANTHER); IPR020683 (PROSITE_PROFILES); IPR002110 (PROSITE_PROFILES); IPR020683 (SUPERFAMILY); TMhelix (TMHMM)
1032	266642_length_551_cvg_13.8_tip_1_2	74	beta- -n-acetylgalactosaminyltransferase bre-4-like	183	1.75E-49	58.90%	0.145		IPR003859 (PRINTS); IPR027791 (PFAM); IPR027995 (PFAM); IPR029044 (G3DSA:3.90.550.GENE3D); IPR003859 (PANTHER); PTHR19300:SF30 (PANTHER); IPR029044 (SUPERFAMILY)
1030	266478_length_550_cvg_3.9_tip_1_3	23	transmembrane protease serine 9	184	2.84E-16	53.20%	0.106		IPR002172 (SMART); IPR001254 (PFAM); IPR002172 (G3DSA:4.10.400.GENE3D); IPR002172 (PFAM); G3DSA:2.40.10.10 (GENE3D); PTHR24265 (PANTHER); IPR018114 (PROSITE_PATTERNS); IPR002172 (PROSITE_PROFILES); IPR002172 (SUPERFAMILY); IPR009003 (SUPERFAMILY)
1031	266550_length_551_cvg_3.0_tip_1_3	16	von willebrand factor	184	1.07E-10	47.20%	0.12		IPR001846 (PFAM); PTHR11339 (PANTHER); CYTOPLASMIC_DOMAIN (PHOBIUS); TRANSMEMBRANE (PHOBIUS); CYTOPLASMIC_DOMAIN (PHOBIUS); NON_CYTOPLASMIC_DOMAIN (PHOBIUS); TRANSMEMBRANE (PHOBIUS); IPR001846 (PROSITE_PROFILES); TMhelix (TMHMM); TMhelix (TMHMM); TMhelix (TMHMM)
1033	266666_length_552_cvg_80.8_tip_1_3	4434	glyceraldehyde 3-phosphate dehydrogenase	184	3.14E-12	90.80%	0.128		IPR020831 (PANTHER); CYTOPLASMIC_DOMAIN (PHOBIUS); NON_CYTOPLASMIC_DOMAIN (PHOBIUS); TRANSMEMBRANE (PHOBIUS)
1034	266750_length_552_cvg_3.2_tip_1_4	17	cytochrome p450 2u1-like	184	2.24E-58	67.40%	0.666	Y	IPR001128 (PRINTS); IPR002401 (PRINTS); IPR001128 (PFAM); IPR001128 (G3DSA:1.10.630.GENE3D); PTHR24300 (PANTHER); SignalP-noTM (SIGNALP_EUK); IPR001128 (SUPERFAMILY)
1035	266754_length_552_cvg_3.8_tip_1_5	28	fibronectin type 3 and ankyrin repeat domains protein 1	184	2.95E-42	69.50%	0.455	Y	IPR002110 (PRINTS); IPR002110 (SMART); IPR020683 (PFAM); IPR020683 (G3DSA:1.25.40.GENE3D); IPR002110 (PFAM); PTHR24183:SF1 (PANTHER); PTHR24183 (PANTHER); SIGNAL_PEPTIDE_N_REGION (PHOBIUS); SIGNAL_PEPTIDE_H_REGION (PHOBIUS); SIGNAL_PEPTIDE_C_REGION (PHOBIUS); NON_CYTOPLASMIC_DOMAIN (PHOBIUS); SIGNAL_PEPTIDE (PHOBIUS); IPR020683 (PROSITE_PROFILES); IPR002110 (PROSITE_PROFILES); IPR002110 (PROSITE_PROFILES); IPR002110 (PROSITE_PROFILES); IPR002110 (PROSITE_PROFILES); SignalP-noTM (SIGNALP_EUK); IPR020683 (SUPERFAMILY)

1036	266804_length_553_cvg_17.0_tip_1_3	120	fatty acid binding protein	185	2.55E-54	75.10%	0.157	IPR000463 (PRINTS); IPR012674 (G3DSA:2.40.128.GENE3D); IPR000566 (PFAM); PTHR11955 (PANTHER); IPR000463 (PROSITE_PATTERNS); IPR011038 (SUPERFAMILY)
1037	267028_length_555_cvg_4.0_tip_1_4	25	isoform b	185	1.17E-11	63.20%	0.116	IPR001254 (PFAM); G3DSA:2.40.10.10 (GENE3D); PTHR24268 (PANTHER); PTHR24268:SF94 (PANTHER); IPR018114 (PROSITE_PATTERNS); IPR009003 (SUPERFAMILY)
1038	267034_length_555_cvg_2.0_tip_1_2	13	deleted in autism protein 1 homolog	185	1.74E-34	54.40%	0.114	IPR022049 (PFAM); PTHR32073 (PANTHER); PTHR32073:SF7 (PANTHER); IPR020519 (PRODOM)
1039	267106_length_556_cvg_2.0_tip_1_2	12	CG4553	185	2.82E-24	61.70%	0.142	Coil (COILS); Coil (COILS); IPR010487 (PFAM); PTHR21133 (PANTHER); PTHR21133:SF0 (PANTHER)
1040	267130_length_556_cvg_2.5_tip_1_5	14	#NAME?	185	3.12E-84	79.80%	0.124	G3DSA:3.40.50.2000 (GENE3D); IPR023214 (G3DSA:3.40.50.GENE3D); IPR001830 (PFAM); PTHR10788:SF6 (PANTHER); PTHR10788 (PANTHER); SSF53756 (SUPERFAMILY)
1041	267146_length_556_cvg_5.0_tip_1_2	34	sco- partial	185	1.40E-33	61.20%	0.148	IPR000421 (SMART); IPR000421 (PFAM); IPR008979 (G3DSA:2.60.120.GENE3D); PTHR24543 (PANTHER); IPR000421 (PROSITE_PATTERNS); IPR000421 (PROSITE_PROFILES); IPR008979 (SUPERFAMILY)
1042	267160_length_556_cvg_2.0_tip_1_5	14	zinc finger protein 184-like	185	1.97E-43	64.80%	0.115	IPR015880 (SMART); IPR013087 (G3DSA:3.30.160.GENE3D); IPR013087 (G3DSA:3.30.160.GENE3D); PF13465 (PFAM); PTHR24409 (PANTHER); IPR007087 (PROSITE_PATTERNS); IPR007087 (PROSITE_PATTERNS); IPR007087 (PROSITE_PROFILES); IPR007087 (PROSITE_PROFILES); SSF57667 (SUPERFAMILY)
1044	267644_length_560_cvg_2.0_tip_1_2	15	intersectin-2 isoform x1	186	3.34E-66	75.00%	0.107	IPR001452 (PRINTS); PR00499 (PRINTS); IPR001452 (SMART); IPR001452 (PFAM); G3DSA:2.30.30.40 (GENE3D); G3DSA:2.30.30.40 (GENE3D); PTHR11216:SF59 (PANTHER); PTHR11216 (PANTHER); IPR001452 (PROSITE_PROFILES); IPR001452 (PROSITE_PROFILES); IPR001452 (SUPERFAMILY); IPR001452 (SUPERFAMILY)
1045	267646_length_560_cvg_3.1_tip_1_5	21	ectonucleotide pyrophosphatase phosphodiesterase family member 6	186	5.03E-43	58.10%	0.1	IPR017849 (G3DSA:3.40.720.GENE3D); IPR002591 (PFAM); IPR024873 (PANTHER); IPR017850 (SUPERFAMILY)
1043	267468_length_559_cvg_2.0_tip_1_3	12	protein cbfa2t1-like isoform x5	187	2.77E-69	78.30%	0.125	Coil (COILS); Coil (COILS); IPR013289 (PRINTS); IPR002893 (PFAM); PTHR10379 (PANTHER); IPR002893 (PROSITE_PATTERNS); IPR002893 (PROSITE_PROFILES); SSF144232 (SUPERFAMILY)
1046	267704_length_561_cvg_8.2_tip_1_4	43	reticulocalbin-2	187	1.36E-45	69.90%	0.412 Y	IPR011992 (PFAM); IPR011992 (G3DSA:1.10.238.GENE3D); PTHR10827:SF48 (PANTHER); PTHR10827 (PANTHER); IPR018247 (PROSITE_PATTERNS); IPR002048 (PROSITE_PROFILES); IPR002048 (PROSITE_PROFILES); SSF47473 (SUPERFAMILY)
1047	267760_length_561_cvg_3.2_tip_1_0	21	zinc finger protein 628 isoform x1	187	9.99E-16	57.20%	0.137	Coil (COILS); IPR015880 (SMART); IPR013087 (G3DSA:3.30.160.GENE3D); PF13465 (PFAM); IPR013087 (G3DSA:3.30.160.GENE3D); PTHR24409:SF14 (PANTHER); PTHR24409 (PANTHER); IPR007087 (PROSITE_PATTERNS); IPR007087 (PROSITE_PATTERNS); IPR007087 (PROSITE_PROFILES); IPR007087 (PROSITE_PROFILES); IPR007087 (PROSITE_PROFILES); SSF57667 (SUPERFAMILY); SSF57667 (SUPERFAMILY)
1048	267766_length_561_cvg_2.9_tip_1_4	23	hornerin isoform x2	187	4.62E-18	46.80%	0.141	G3DSA:2.40.10.10 (GENE3D); IPR001254 (PFAM); G3DSA:2.40.10.10 (GENE3D); PTHR24268 (PANTHER); IPR001254 (PROSITE_PROFILES); IPR009003 (SUPERFAMILY)
1049	267780_length_561_cvg_2.9_tip_1_1	18	elastase 2b	187	7.99E-22	55.40%	0.099	G3DSA:2.40.10.10 (GENE3D); G3DSA:2.40.10.10 (GENE3D); IPR001254 (PFAM); IPR002172 (G3DSA:4.10.400.GENE3D); PTHR24265 (PANTHER); IPR018114 (PROSITE_PATTERNS); IPR001254 (PROSITE_PROFILES); IPR002172 (PROSITE_PROFILES); IPR002172 (SUPERFAMILY); IPR009003 (SUPERFAMILY)
1051	267848_length_562_cvg_50.9_tip_1_4	742	hemagglutinin amebocyte aggregation factor-like	187	2.70E-10	72.20%	0.108	PF14704 (PFAM); IPR026645 (PANTHER); CYTOPLASMIC_DOMAIN (PHOBIUS); CYTOPLASMIC_DOMAIN (PHOBIUS); NON_CYTOPLASMIC_DOMAIN (PHOBIUS); TRANSMEMBRANE (PHOBIUS); TRANSMEMBRANE (PHOBIUS); TMhelix (TMHMM); TMhelix (TMHMM)
1052	267910_length_562_cvg_2.0_tip_1_5	16	upf0764 protein c16orf89 homolog	187	2.71E-21	52.20%	0.183	no IPS match
1053	267914_length_562_cvg_4.2_tip_1_5	31	dentin sialophosphoprotein isoform x1	187	5.39E-40	59.30%	0.098	IPR015880 (SMART); IPR013087 (G3DSA:3.30.160.GENE3D); IPR013087 (G3DSA:3.30.160.GENE3D); PF13465 (PFAM); PTHR24409:SF14 (PANTHER); PTHR24409 (PANTHER); IPR007087 (PROSITE_PATTERNS); IPR007087 (PROSITE_PATTERNS); IPR007087 (PROSITE_PATTERNS); IPR007087 (PROSITE_PATTERNS); IPR007087 (PROSITE_PATTERNS); IPR007087 (PROSITE_PROFILES); IPR007087 (PROSITE_PROFILES); IPR007087 (PROSITE_PROFILES); IPR007087 (PROSITE_PROFILES); IPR007087 (PROSITE_PROFILES); SSF57667 (SUPERFAMILY); SSF57667 (SUPERFAMILY); SSF57667 (SUPERFAMILY)

1050	267828_length_562_cvg_2.0_tip_1_0	15	zinc finger protein 77-like	188	9.68E-15	65.90%	0.118		IPR015880 (SMART); IPR013087 (G3DSA:3.30.160.GENE3D); IPR013087 (G3DSA:3.30.160.GENE3D); PF13465 (PFAM); PTHR24409:SF13 (PANTHER); PTHR24409 (PANTHER); IPR007087 (PROSITE_PATTERNS); IPR007087 (PROSITE_PATTERNS); IPR007087 (PROSITE_PROFILES); IPR007087 (PROSITE_PROFILES); IPR007087 (PROSITE_PROFILES); SSF57667 (SUPERFAMILY)
1054	268168_length_565_cvg_2.0_tip_1_4	14	17-beta-hydroxysteroid dehydrogenase type partial	188	2.18E-80	72.20%	0.106		IPR002198 (PRINTS); IPR002347 (PRINTS); IPR016040 (G3DSA:3.40.50.GENE3D); IPR002198 (PFAM); PTHR24316 (PANTHER); PTHR24316:SF215 (PANTHER); IPR020904 (PROSITE_PATTERNS); SSF51735 (SUPERFAMILY)
1055	268356_length_567_cvg_2.0_tip_1_1	11	adp-ribosylation factor-like protein 3-like	189	2.15E-72	67.00%	0.329		IPR006689 (PRINTS); IPR024156 (SMART); IPR005225 (TIGRFAM); IPR027417 (G3DSA:3.40.50.GENE3D); IPR006689 (PFAM); PTHR11711:SF15 (PANTHER); PTHR11711 (PANTHER); TRANSMEMBRANE (PHOBIUS); NON_CYTOPLASMIC_DOMAIN (PHOBIUS); CYTOPLASMIC_DOMAIN (PHOBIUS); IPR024156 (PROSITE_PROFILES); IPR027417 (SUPERFAMILY); TMhelix (TMHMM)
1056	268400_length_567_cvg_4.0_tip_1_3	28	carboxypeptidase a2-like	189	3.15E-35	59.00%	0.642	Y	G3DSA:3.40.630.10 (GENE3D); IPR003146 (G3DSA:3.30.70.GENE3D); IPR000834 (PFAM); IPR003146 (PFAM); PTHR11705 (PANTHER); SIGNAL_PEPTIDE_N_REGION (PHOBIUS); SIGNAL_PEPTIDE_C_REGION (PHOBIUS); SIGNAL_PEPTIDE_H_REGION (PHOBIUS); SIGNAL_PEPTIDE (PHOBIUS); NON_CYTOPLASMIC_DOMAIN (PHOBIUS); SignalP-noTM (SIGNALP_EUK); IPR009020 (SUPERFAMILY); SSF53187 (SUPERFAMILY)
1057	268548_length_568_cvg_3.0_tip_1_1	21	protein toll	189	6.89E-26	49.90%	0.909	Y	IPR003591 (SMART); G3DSA:3.80.10.10 (GENE3D); IPR001611 (PFAM); NON_CYTOPLASMIC_DOMAIN (PHOBIUS); SIGNAL_PEPTIDE_C_REGION (PHOBIUS); SIGNAL_PEPTIDE_N_REGION (PHOBIUS); SIGNAL_PEPTIDE_H_REGION (PHOBIUS); SIGNAL_PEPTIDE (PHOBIUS); SignalP-TM (SIGNALP_GRAM_POSITIVE); SignalP-noTM (SIGNALP_GRAM_NEGATIVE); SignalP-noTM (SIGNALP_EUK); SSF52058 (SUPERFAMILY)
1060	268704_length_569_cvg_56.5_tip_1_5	2643	ubiquitin-40s ribosomal protein s27a-like	189	3.41E-94	96.10%	0.244		IPR019956 (PRINTS); IPR000626 (SMART); G3DSA:3.10.20.90 (GENE3D); IPR002906 (PFAM); IPR000626 (PFAM); PTHR10666:SF99 (PANTHER); PTHR10666 (PANTHER); IPR019954 (PROSITE_PATTERNS); IPR000626 (PROSITE_PROFILES); IPR029071 (SUPERFAMILY); IPR011332 (SUPERFAMILY)
1058	268584_length_568_cvg_6.9_tip_1_3	49	contactin-associated protein like 5-1	190	2.81E-69	73.70%	0.103		G3DSA:2.10.25.10 (GENE3D); PTHR10127:SF346 (PANTHER); PTHR10127 (PANTHER); IPR000742 (PROSITE_PROFILES); IPR002181 (SUPERFAMILY)
1059	268676_length_569_cvg_8.6_tip_1_0	52	epithelial chloride channel partial	190	6.47E-34	63.10%	0.138		IPR013642 (PFAM); PTHR10579:SF8 (PANTHER); PTHR10579 (PANTHER); TRANSMEMBRANE (PHOBIUS); CYTOPLASMIC_DOMAIN (PHOBIUS); NON_CYTOPLASMIC_DOMAIN (PHOBIUS); TMhelix (TMHMM)
1061	268728_length_570_cvg_6.4_tip_1_1	40	dna-directed rna polymerase ii subunit rpb1	190	4.36E-07	53.00%	0.104		PR01217 (PRINTS)
1062	268848_length_571_cvg_5.5_tip_1_1	30	adenosine deaminase cecr1	190	1.83E-57	62.90%	0.157		G3DSA:3.20.20.140 (GENE3D); IPR001365 (PFAM); PTHR11409:SF39 (PANTHER); PTHR11409 (PANTHER); SSF51556 (SUPERFAMILY)
1063	268930_length_571_cvg_3.0_tip_1_3	21	hemocyte protein-glutamine gamma-glutamyltransferase-like	191	3.27E-79	76.50%	0.105		IPR013783 (G3DSA:2.60.40.GENE3D); IPR002931 (PFAM); IPR002931 (G3DSA:3.90.260.GENE3D); IPR023608 (PANTHER); PTHR11590:SF40 (PANTHER); SSF54001 (SUPERFAMILY)
1064	268934_length_572_cvg_2.0_tip_1_0	19	transforming growth factor-beta-induced protein ig-h3-like	191	7.07E-82	66.00%	0.102		IPR000782 (SMART); IPR000782 (PFAM); IPR000782 (G3DSA:2.30.180.GENE3D); PTHR10900:SF73 (PANTHER); PTHR10900 (PANTHER); IPR011489 (PROSITE_PROFILES); IPR000782 (PROSITE_PROFILES); IPR000782 (SUPERFAMILY)
1065	269042_length_572_cvg_4.3_tip_1_3	28	hemagglutinin	191	4.34E-27	54.60%	0.175		G3DSA:2.60.120.380 (GENE3D); G3DSA:1.10.390.10 (GENE3D); IPR001570 (PFAM); IPR007280 (PFAM); SSF55486 (SUPERFAMILY)
1066	269072_length_573_cvg_2.9_tip_1_5	20	sucrase- intestinal	191	2.23E-33	57.80%	0.103		PTHR22762 (PANTHER); PTHR22762:SF7 (PANTHER); IPR011013 (SUPERFAMILY)
1067	269194_length_574_cvg_2.0_tip_1_1	15	collagen-like protein	191	3.82E-19	46.40%	0.12		no IPS match
1068	269194_length_574_cvg_2.0_tip_1_4	15	collagen alpha-1 chain	191	1.56E-27	57.60%	0.128		IPR008160 (PFAM); PTHR24023 (PANTHER)
1069	269224_length_574_cvg_14.0_tip_1_3	88	serine protease nudel-like	192	6.28E-07	52.00%	0.102		IPR002172 (SMART); IPR003014 (PFAM); IPR002172 (PFAM); IPR002172 (G3DSA:4.10.400.GENE3D); G3DSA:3.50.4.10 (GENE3D); IPR002172 (PROSITE_PROFILES); IPR002172 (SUPERFAMILY)
1070	269402_length_576_cvg_3.0_tip_1_1	24	myosin heavy chain variant c	192	5.05E-113	98.50%	0.112		Coil (COILS); Coil (COILS); IPR002928 (PFAM); PTHR13140 (PANTHER); PTHR13140:SF364 (PANTHER); SSF57997 (SUPERFAMILY)
1071	269534_length_577_cvg_4.0_tip_1_2	33	hypothetical protein KGM_21983	192	1.03E-41	57.70%	0.114		IPR000859 (SMART); IPR000859 (PFAM); IPR000859 (G3DSA:2.60.120.GENE3D); IPR000859 (PROSITE_PROFILES); IPR000859 (SUPERFAMILY)

1072	269592_length_577_cvg_2.0_tip_1_5	13	zinc finger protein ozf	192	3.86E-79	59.30%	0.103	IPR015880 (SMART); IPR007087 (PFAM); IPR013087 (G3DSA:3.30.160.GENE3D); IPR013087 (G3DSA:3.30.160.GENE3D); IPR013087 (G3DSA:3.30.160.GENE3D); PF13465 (PFAM); IPR013087 (G3DSA:3.30.160.GENE3D); PTHR24409 (PANTHER); IPR007087 (PROSITE_PATTERNS); IPR007087 (PROSITE_PATTERNS); IPR007087 (PROSITE_PATTERNS); IPR007087 (PROSITE_PATTERNS); IPR007087 (PROSITE_PROFILES); IPR007087 (PROSITE_PROFILES); IPR007087 (PROSITE_PROFILES); IPR007087 (PROSITE_PROFILES); IPR007087 (PROSITE_PROFILES); IPR007087 (PROSITE_PROFILES); SSF57667 (SUPERFAMILY); SSF57667 (SUPERFAMILY); SSF57667 (SUPERFAMILY)
1074	269632_length_578_cvg_3.0_tip_1_5	23	protein kinase c epsilon partial	192	1.06E-127	95.50%	0.096	IPR002290 (SMART); G3DSA:1.10.510.10 (GENE3D); G3DSA:3.30.200.20 (GENE3D); IPR000719 (PFAM); PTHR24357:SF73 (PANTHER); PTHR24357 (PANTHER); IPR008271 (PROSITE_PATTERNS); IPR000719 (PROSITE_PROFILES); IPR011009 (SUPERFAMILY)
1075	269690_length_578_cvg_47.9_tip_1_2	228	---NA---	192			0.182	TMhelix (TMHMM); TMhelix (TMHMM)
1073	269610_length_578_cvg_5.1_tip_1_4	30	cell recognition protein caspr4 isoform 5	193	1.88E-39	55.60%	0.13	PTHR10127 (PANTHER); PTHR10127:SF575 (PANTHER)
1076	269710_length_579_cvg_5.1_tip_1_4	41	isoform b	193	6.99E-89	76.30%	0.681 Y	IPR002172 (SMART); IPR002557 (SMART); IPR002557 (G3DSA:2.170.140.GENE3D); IPR002172 (PFAM); IPR002172 (G3DSA:4.10.400.GENE3D); IPR002557 (PFAM); PTHR11177 (PANTHER); PTHR11177:SF136 (PANTHER); IPR023415 (PROSITE_PATTERNS); NON_CYTOPLASMIC_DOMAIN (PHOBIUS); SIGNAL_PEPTIDE (PHOBIUS); SIGNAL_PEPTIDE_N_REGION (PHOBIUS); SIGNAL_PEPTIDE_C_REGION (PHOBIUS); SIGNAL_PEPTIDE_H_REGION (PHOBIUS); IPR002172 (PROSITE_PROFILES); IPR002557 (PROSITE_PROFILES); SignalP-noTM (SIGNALP_EUK); IPR002557 (SUPERFAMILY); IPR002172 (SUPERFAMILY)
1077	269718_length_579_cvg_2.0_tip_1_2	15	endothelin-converting enzyme 1-like	193	3.31E-44	53.20%	0.106	IPR018497 (PFAM); IPR024079 (G3DSA:3.40.390.GENE3D); PTHR11733:SF124 (PANTHER); IPR000718 (PANTHER); SSF55486 (SUPERFAMILY)
1078	269724_length_579_cvg_9.0_tip_1_3	63	perlucin-like protein	193	7.30E-22	54.70%	0.137	IPR001304 (SMART); IPR001304 (PFAM); IPR016186 (G3DSA:3.10.100.GENE3D); PTHR22802 (PANTHER); IPR018378 (PROSITE_PATTERNS); IPR001304 (PROSITE_PROFILES); IPR016187 (SUPERFAMILY)
1079	269776_length_579_cvg_3.0_tip_1_3	26	spermatogenesis-associated protein 20	193	4.65E-58	80.90%	0.134	IPR012336 (G3DSA:3.40.30.GENE3D); IPR004879 (PFAM); PTHR12145 (PANTHER); PTHR12145:SF11 (PANTHER); IPR012336 (SUPERFAMILY)
1080	269816_length_580_cvg_3.7_tip_1_2	26	lysosomal pro-x carboxypeptidase	193	2.83E-80	71.10%	0.244	IPR008758 (PFAM); IPR008758 (PANTHER); PTHR11010:SF11 (PANTHER)
1081	269822_length_580_cvg_65.2_tip_1_2	6630	cathepsin l1-like	193	2.65E-76	80.70%	0.101	IPR000668 (SMART); IPR000668 (PFAM); G3DSA:3.90.70.10 (GENE3D); PTHR12411:SF265 (PANTHER); IPR013128 (PANTHER); IPR025661 (PROSITE_PATTERNS); IPR025660 (PROSITE_PATTERNS); SSF54001 (SUPERFAMILY)
1083	269944_length_581_cvg_34.0_tip_1_2	296	histone h2a-like	193	1.03E-79	97.30%	0.316	IPR002119 (PRINTS); IPR002119 (SMART); IPR007125 (PFAM); IPR009072 (G3DSA:1.10.20.GENE3D); PTHR23430:SF45 (PANTHER); PTHR23430 (PANTHER); IPR002119 (PROSITE_PATTERNS); IPR009072 (SUPERFAMILY)
1082	269942_length_581_cvg_5.5_tip_1_3	44	39s ribosomal protein mitochondrial	194	5.45E-83	74.20%	0.102	IPR008914 (G3DSA:3.90.280.GENE3D); IPR008914 (PFAM); PTHR11362 (PANTHER); PTHR11362:SF16 (PANTHER); IPR008914 (SUPERFAMILY)
1084	270030_length_582_cvg_7.0_tip_1_4	53	ankyrin repeat partial	194	6.68E-37	60.40%	0.115	IPR002110 (SMART); PF13637 (PFAM); PF13857 (PFAM); IPR020683 (G3DSA:1.25.40.GENE3D); PTHR24198 (PANTHER); IPR002110 (PROSITE_PROFILES); IPR020683 (PROSITE_PROFILES); IPR002110 (PROSITE_PROFILES); IPR020683 (SUPERFAMILY)
1085	270168_length_583_cvg_2.0_tip_1_4	16	cysteine-rich with egf-like domain protein 2	194	1.75E-73	69.60%	0.109	G3DSA:2.170.300.10 (GENE3D); IPR001881 (PFAM); PTHR24838:SF13 (PANTHER); PTHR24838 (PANTHER); IPR018097 (PROSITE_PATTERNS); IPR013032 (PROSITE_PATTERNS); IPR002049 (PROSITE_PATTERNS); IPR000742 (PROSITE_PROFILES); IPR009030 (SUPERFAMILY)
1086	270176_length_584_cvg_4.0_tip_1_1	27	adrenodoxin-like mitochondrial	195	1.44E-77	85.00%	0.191	IPR001055 (PRINTS); IPR001041 (PFAM); IPR012675 (G3DSA:3.10.20.GENE3D); PTHR23426 (PANTHER); PTHR23426:SF1 (PANTHER); IPR018298 (PROSITE_PATTERNS); IPR001041 (PROSITE_PROFILES); IPR001041 (SUPERFAMILY)
1087	270184_length_584_cvg_2.0_tip_1_0	20	ankyrin repeat domain-containing protein 39	195	5.24E-54	67.50%	0.221	IPR002110 (SMART); PF13637 (PFAM); IPR020683 (G3DSA:1.25.40.GENE3D); PF13857 (PFAM); PTHR24148 (PANTHER); IPR002110 (PROSITE_PROFILES); IPR020683 (PROSITE_PROFILES); IPR002110 (PROSITE_PROFILES); IPR002110 (PROSITE_PROFILES); IPR020683 (SUPERFAMILY)

1088	270266_length_585_cvg_19.7_tip_1_2	146	adiponectin receptor protein	195	8.19E-56	83.30%	0.204	IPR004254 (PFAM); PTHR20855:SF15 (PANTHER); IPR004254 (PANTHER); NON_CYTOPLASMIC_DOMAIN (PHOBIUS); TRANSMEMBRANE (PHOBIUS); TRANSMEMBRANE (PHOBIUS); NON_CYTOPLASMIC_DOMAIN (PHOBIUS); CYTOPLASMIC_DOMAIN (PHOBIUS); CYTOPLASMIC_DOMAIN (PHOBIUS); TRANSMEMBRANE (PHOBIUS); TRANSMEMBRANE (PHOBIUS); CYTOPLASMIC_DOMAIN (PHOBIUS); TMhelix (TMHMM); TMhelix (TMHMM); TMhelix (TMHMM); TMhelix (TMHMM)
1089	270294_length_585_cvg_2.2_tip_1_1	15	guanine nucleotide-binding protein subunit beta-5	195	3.14E-128	93.20%	0.107	IPR020472 (PRINTS); IPR001632 (PRINTS); IPR001680 (SMART); IPR001680 (PFAM); IPR015943 (G3DSA:2.130.10.GENE3D); IPR016346 (PANTHER); PTHR19850:SF12 (PANTHER); IPR019775 (PROSITE_PATTERNS); IPR019775 (PROSITE_PATTERNS); IPR001680 (PROSITE_PROFILES); IPR001680 (PROSITE_PROFILES); IPR017986 (PROSITE_PROFILES); IPR001680 (PROSITE_PROFILES); IPR017986 (SUPERFAMILY)
1090	270364_length_585_cvg_14.5_tip_1_4	104	iron zinc purple acid phosphatase-like partial	195	3.79E-62	71.20%	0.27	IPR003961 (PFAM); IPR015914 (G3DSA:2.60.40.GENE3D); IPR029052 (G3DSA:3.60.21.GENE3D); IPR018946 (PFAM); PTHR22953 (PANTHER); PTHR22953:SF9 (PANTHER); IPR008963 (SUPERFAMILY); TMhelix (TMHMM)
1091	270476_length_587_cvg_7.2_tip_1_2	48	alpha-mannosidase 2x	195	6.13E-98	81.50%	0.102	IPR000602 (PFAM); IPR027291 (G3DSA:3.20.110.GENE3D); PTHR11607 (PANTHER); PTHR11607:SF4 (PANTHER); IPR011330 (SUPERFAMILY)
1092	270540_length_587_cvg_2.3_tip_1_4	23	zinc finger protein 1 homolog	196	1.28E-16	54.10%	0.106	IPR015880 (SMART); IPR013087 (G3DSA:3.30.160.GENE3D); PF13465 (PFAM); IPR013087 (G3DSA:3.30.160.GENE3D); PTHR24409 (PANTHER); IPR007087 (PROSITE_PATTERNS); IPR007087 (PROSITE_PROFILES); IPR007087 (PROSITE_PROFILES); SSF57667 (SUPERFAMILY)
1093	270670_length_589_cvg_4.0_tip_1_2	31	tkl protein kinase	196	3.95E-44	54.30%	0.098	IPR003591 (SMART); IPR001611 (PFAM); G3DSA:3.80.10.10 (GENE3D); PTHR24365 (PANTHER); IPR001611 (PROSITE_PROFILES); IPR001611 (PROSITE_PROFILES); IPR001611 (PROSITE_PROFILES); IPR001611 (PROSITE_PROFILES); IPR001611 (PROSITE_PROFILES); IPR001611 (PROSITE_PROFILES); IPR001611 (PROSITE_PROFILES); SSF52058 (SUPERFAMILY)
1094	270678_length_589_cvg_3.9_tip_1_1	23	adenylate cyclase type 9	196	2.06E-52	84.20%	0.254	IPR001054 (PFAM); IPR001054 (G3DSA:3.30.70.GENE3D); PTHR11920:SF272 (PANTHER); PTHR11920 (PANTHER); IPR018297 (PROSITE_PATTERNS); NON_CYTOPLASMIC_DOMAIN (PHOBIUS); TRANSMEMBRANE (PHOBIUS); CYTOPLASMIC_DOMAIN (PHOBIUS); IPR001054 (PROSITE_PROFILES); IPR029787 (SUPERFAMILY); TMhelix (TMHMM)
1095	270730_length_589_cvg_4.2_tip_1_1	26	gastrula zinc finger isoform x1	196	6.55E-63	68.50%	0.103	IPR015880 (SMART); IPR013087 (G3DSA:3.30.160.GENE3D); PF13465 (PFAM); IPR013087 (G3DSA:3.30.160.GENE3D); IPR013087 (G3DSA:3.30.160.GENE3D); IPR013087 (G3DSA:3.30.160.GENE3D); PTHR24409 (PANTHER); IPR007087 (PROSITE_PATTERNS); IPR007087 (PROSITE_PATTERNS); IPR007087 (PROSITE_PATTERNS); IPR007087 (PROSITE_PATTERNS); IPR007087 (PROSITE_PATTERNS); IPR007087 (PROSITE_PROFILES); IPR007087 (PROSITE_PROFILES); IPR007087 (PROSITE_PROFILES); IPR007087 (PROSITE_PROFILES); IPR007087 (PROSITE_PROFILES); IPR007087 (PROSITE_PROFILES); IPR007087 (PROSITE_PROFILES); SSF57667 (SUPERFAMILY); SSF57667 (SUPERFAMILY); SSF57667 (SUPERFAMILY); SSF57667 (SUPERFAMILY)
1098	270758_length_590_cvg_2.0_tip_1_5	12	collagen alpha-1 chain	196	6.42E-45	59.30%	0.103	IPR008160 (PFAM); PTHR24023 (PANTHER)
1100	270820_length_590_cvg_3.4_tip_1_5	23	low-density lipoprotein receptor-related protein 2	196	2.28E-94	83.90%	0.102	IPR000742 (SMART); IPR001881 (SMART); IPR002172 (SMART); IPR026823 (PFAM); IPR002172 (PFAM); IPR011042 (G3DSA:2.120.10.GENE3D); IPR002172 (G3DSA:4.10.400.GENE3D); G3DSA:2.10.25.10 (GENE3D); G3DSA:2.10.25.10 (GENE3D); PTHR10529 (PANTHER); IPR000152 (PROSITE_PATTERNS); IPR000152 (PROSITE_PATTERNS); IPR013032 (PROSITE_PATTERNS); IPR018097 (PROSITE_PATTERNS); IPR023415 (PROSITE_PATTERNS); IPR018097 (PROSITE_PATTERNS); IPR000742 (PROSITE_PROFILES); IPR002172 (PROSITE_PROFILES); IPR000742 (PROSITE_PROFILES); SSF57196 (SUPERFAMILY); IPR002172 (SUPERFAMILY); SSF63825 (SUPERFAMILY); SSF57196 (SUPERFAMILY)
1096	270744_length_589_cvg_5.7_tip_1_0	33	disks large 1 tumor suppressor protein	197	1.15E-86	86.60%	0.113	IPR001478 (SMART); IPR001478 (G3DSA:2.30.42.GENE3D); IPR001478 (G3DSA:2.30.42.GENE3D); IPR001478 (PFAM); IPR016313 (PANTHER); IPR001478 (PROSITE_PROFILES); IPR001478 (PROSITE_PROFILES); IPR001478 (SUPERFAMILY); IPR001478 (SUPERFAMILY)
1097	270758_length_590_cvg_2.0_tip_1_1	12	c protein immunoglobulin-a-binding beta antigen	197	4.79E-27	43.80%	0.138	PTHR24023 (PANTHER); PTHR24023:SF405 (PANTHER)
1099	270790_length_590_cvg_48.9_tip_0_3	299	amine oxidase	197	1.03E-67	70.70%	0.112	IPR001613 (PRINTS); IPR002937 (PFAM); G3DSA:3.90.660.10 (GENE3D); PTHR10742 (PANTHER); PTHR10742:SF253 (PANTHER); SSF54373 (SUPERFAMILY); SSF51905 (SUPERFAMILY)

1101	270864_length_591_cvg_9.1_tip_1_3	44	PREDICTED: uncharacterized protein LOC105270491 isoform X2	197	3.11E-10	52.30%	0.101	IPR002172 (SMART); IPR003014 (PFAM); IPR002172 (PFAM); G3DSA:3.50.4.10 (GENE3D); IPR002172 (G3DSA:4.10.400.GENE3D); IPR023415 (PROSITE_PATTERNS); IPR003609 (PROSITE_PROFILES); IPR002172 (PROSITE_PROFILES); IPR002172 (SUPERFAMILY)
1102	271030_length_592_cvg_5.3_tip_1_1	33	gastrula zinc finger	197	7.66E-46	62.40%	0.103	IPR015880 (SMART); IPR013087 (G3DSA:3.30.160.GENE3D); IPR013087 (G3DSA:3.30.160.GENE3D); PF13465 (PFAM); IPR007087 (PFAM); IPR013087 (G3DSA:3.30.160.GENE3D); PTHR24409 (PANTHER); IPR007087 (PROSITE_PATTERNS); IPR007087 (PROSITE_PATTERNS); IPR007087 (PROSITE_PATTERNS); IPR007087 (PROSITE_PATTERNS); IPR007087 (PROSITE_PATTERNS); IPR007087 (PROSITE_PATTERNS); IPR007087 (PROSITE_PROFILES); IPR007087 (PROSITE_PROFILES); IPR007087 (PROSITE_PROFILES); IPR007087 (PROSITE_PROFILES); IPR007087 (PROSITE_PROFILES); IPR007087 (PROSITE_PROFILES); IPR007087 (PROSITE_PROFILES); SSF57667 (SUPERFAMILY); SSF57667 (SUPERFAMILY); SSF57667 (SUPERFAMILY); SSF57667 (SUPERFAMILY)
1103	271194_length_594_cvg_3.0_tip_1_3	29	kielin chordin-like protein isoform x2	198	1.40E-35	57.70%	0.105	IPR002223 (G3DSA:4.10.410.GENE3D); G3DSA:2.10.25.10 (GENE3D); IPR013032 (PROSITE_PATTERNS); IPR000742 (PROSITE_PROFILES); IPR000436 (PROSITE_PROFILES); SSF57196 (SUPERFAMILY); IPR002223 (SUPERFAMILY)
1106	271382_length_596_cvg_4.3_tip_1_2	30	zinc finger protein 271 (zinc finger protein 7) (zinc finger protein znfphex133) (epstein-barr virus-induced zinc finger protein) (znf-eb) (ct-zfp48) (zinc finger protein	198	1.89E-28	59.40%	0.099	IPR015880 (SMART); IPR013087 (G3DSA:3.30.160.GENE3D); IPR013087 (G3DSA:3.30.160.GENE3D); PF13465 (PFAM); IPR007087 (PFAM); IPR013087 (G3DSA:3.30.160.GENE3D); PTHR24409:SF14 (PANTHER); PTHR24409 (PANTHER); IPR007087 (PROSITE_PATTERNS); IPR007087 (PROSITE_PATTERNS); IPR007087 (PROSITE_PATTERNS); IPR007087 (PROSITE_PATTERNS); IPR007087 (PROSITE_PATTERNS); IPR007087 (PROSITE_PATTERNS); IPR007087 (PROSITE_PROFILES); IPR007087 (PROSITE_PROFILES); IPR007087 (PROSITE_PROFILES); IPR007087 (PROSITE_PROFILES); IPR007087 (PROSITE_PROFILES); IPR007087 (PROSITE_PROFILES); IPR007087 (PROSITE_PROFILES); SSF57667 (SUPERFAMILY); SSF57667 (SUPERFAMILY); SSF57667 (SUPERFAMILY)
1104	271250_length_595_cvg_3.0_tip_1_0	24	agap011650-pa-like protein	199	1.23E-11	72.50%	0.2	IPR000242 (PRINTS); IPR000242 (PFAM); IPR029021 (G3DSA:3.90.190.GENE3D); PTHR19134 (PANTHER); IPR000387 (PROSITE_PROFILES); IPR000242 (PROSITE_PROFILES); IPR029021 (SUPERFAMILY)
1105	271342_length_596_cvg_2.0_tip_1_3	16	contactin- partial	199	1.02E-46	62.30%	0.105	PR00014 (PRINTS); IPR003961 (SMART); IPR013783 (G3DSA:2.60.40.GENE3D); IPR003961 (PFAM); PTHR10489 (PANTHER); PTHR10489:SF525 (PANTHER); IPR003961 (PROSITE_PROFILES); IPR003961 (PROSITE_PROFILES); IPR003961 (PROSITE_PROFILES); IPR003961 (SUPERFAMILY)
1107	271384_length_596_cvg_2.7_tip_1_1	23	lysosomal alpha- partial	199	6.01E-86	75.90%	0.192	IPR015341 (PFAM); IPR015341 (G3DSA:1.20.1270.GENE3D); IPR000602 (PFAM); IPR027291 (G3DSA:3.20.110.GENE3D); PTHR11607 (PANTHER); PTHR11607:SF3 (PANTHER); IPR028995 (SUPERFAMILY); IPR011330 (SUPERFAMILY)
1108	271416_length_597_cvg_2.0_tip_1_1	17	low quality protein: de-cadherin	199	1.85E-20	52.80%	0.108	IPR000233 (PFAM); IPR027397 (G3DSA:4.10.900.GENE3D); PTHR24027 (PANTHER); PTHR24027:SF276 (PANTHER); CYTOPLASMIC_DOMAIN (PHOBIUS); TRANSMEMBRANE (PHOBIUS); NON_CYTOPLASMIC_DOMAIN (PHOBIUS); TMhelix (TMHMM)
1109	271432_length_597_cvg_4.4_tip_1_4	25	zinc finger protein 271 (zinc finger protein 7) (zinc finger protein znfphex133) (epstein-barr virus-induced zinc finger protein) (znf-eb) (ct-zfp48) (zinc finger protein	199	9.99E-32	58.00%	0.099	IPR015880 (SMART); IPR013087 (G3DSA:3.30.160.GENE3D); IPR013087 (G3DSA:3.30.160.GENE3D); PF13894 (PFAM); IPR013087 (G3DSA:3.30.160.GENE3D); PF13465 (PFAM); PTHR24409:SF14 (PANTHER); PTHR24409 (PANTHER); IPR007087 (PROSITE_PATTERNS); IPR007087 (PROSITE_PATTERNS); IPR007087 (PROSITE_PATTERNS); IPR007087 (PROSITE_PATTERNS); IPR007087 (PROSITE_PATTERNS); IPR007087 (PROSITE_PROFILES); IPR007087 (PROSITE_PROFILES); IPR007087 (PROSITE_PROFILES); IPR007087 (PROSITE_PROFILES); IPR007087 (PROSITE_PROFILES); SSF57667 (SUPERFAMILY); SSF57667 (SUPERFAMILY)
1110	271542_length_598_cvg_4.0_tip_1_1	29	zinc finger protein partial	199	2.02E-14	57.10%	0.101	IPR015880 (SMART); IPR013087 (G3DSA:3.30.160.GENE3D); IPR007087 (PFAM); PF13465 (PFAM); IPR013087 (G3DSA:3.30.160.GENE3D); IPR013087 (G3DSA:3.30.160.GENE3D); PTHR24409 (PANTHER); IPR007087 (PROSITE_PATTERNS); IPR007087 (PROSITE_PATTERNS); IPR007087 (PROSITE_PATTERNS); IPR007087 (PROSITE_PROFILES); IPR007087 (PROSITE_PROFILES); IPR007087 (PROSITE_PROFILES); IPR007087 (PROSITE_PROFILES); SSF57667 (SUPERFAMILY); SSF57667 (SUPERFAMILY)
1111	271570_length_598_cvg_4.0_tip_1_1	29	retinol dehydrogenase 13-like	199	1.59E-82	76.50%	0.236	IPR002347 (PRINTS); IPR016040 (G3DSA:3.40.50.GENE3D); PTHR24320:SF55 (PANTHER); PTHR24320 (PANTHER); SSF51735 (SUPERFAMILY)
1112	271598_length_599_cvg_62.0_tip_1_5	11082	translationally controlled tumor protein	199	3.58E-72	79.60%	0.269	IPR018105 (PRINTS); IPR011323 (G3DSA:2.170.150.GENE3D); IPR018105 (PFAM); IPR018105 (PANTHER); IPR011057 (SUPERFAMILY)

1113	271624_length_599_cvg_4.5_tip_1_0	39	aael003561- partial	200	2.18E-26	51.80%	0.263		IPR001211 (PRINTS); IPR016090 (SMART); IPR016090 (PFAM); IPR016090 (G3DSA:1.20.90.GENE3D); PTHR11716:SF45 (PANTHER); IPR001211 (PANTHER); IPR013090 (PROSITE_PATTERNS); IPR013090 (PROSITE_PATTERNS); CYTOPLASMIC_DOMAIN (PHOBIUS); NON_CYTOPLASMIC_DOMAIN (PHOBIUS); TRANSMEMBRANE (PHOBIUS); IPR016090 (SUPERFAMILY); TMhelix (TMHMM)
1114	271688_length_600_cvg_5.0_tip_1_0	43	zinc finger protein ozf-like isoform x2	200	5.58E-40	60.00%	0.103		IPR015880 (SMART); IPR013087 (G3DSA:3.30.160.GENE3D); PF13465 (PFAM); IPR013087 (G3DSA:3.30.160.GENE3D); IPR013087 (G3DSA:3.30.160.GENE3D); IPR007087 (PFAM); PF13894 (PFAM); PTHR24409 (PANTHER); PTHR24409:SF13 (PANTHER); IPR007087 (PROSITE_PATTERNS); IPR007087 (PROSITE_PATTERNS); IPR007087 (PROSITE_PATTERNS); IPR007087 (PROSITE_PATTERNS); IPR007087 (PROSITE_PATTERNS); IPR007087 (PROSITE_PATTERNS); IPR007087 (PROSITE_PROFILES); IPR007087 (PROSITE_PROFILES); IPR007087 (PROSITE_PROFILES); IPR007087 (PROSITE_PROFILES); IPR007087 (PROSITE_PROFILES); IPR007087 (PROSITE_PROFILES); SSF57667 (SUPERFAMILY); SSF57667 (SUPERFAMILY); SSF57667 (SUPERFAMILY)
1115	271696_length_600_cvg_3.8_tip_1_4	28	contactin-associated 2 precursor	200	3.85E-29	53.20%	0.104		IPR008983 (G3DSA:2.60.120.GENE3D); IPR014716 (G3DSA:3.90.215.GENE3D); IPR001073 (PFAM); PTHR10127:SF575 (PANTHER); PTHR10127 (PANTHER); IPR001073 (PROSITE_PROFILES); IPR008983 (SUPERFAMILY); IPR002181 (SUPERFAMILY)
1116	271758_length_600_cvg_59.4_tip_1_4	718	plasminogen activator spa	200	1.63E-36	56.40%	0.444	Y	IPR001314 (PRINTS); IPR001254 (SMART); G3DSA:2.40.10.10 (GENE3D); G3DSA:2.40.10.10 (GENE3D); G3DSA:2.40.10.10 (GENE3D); IPR001254 (PFAM); PTHR24276 (PANTHER); IPR018114 (PROSITE_PATTERNS); NON_CYTOPLASMIC_DOMAIN (PHOBIUS); TRANSMEMBRANE (PHOBIUS); CYTOPLASMIC_DOMAIN (PHOBIUS); IPR001254 (PROSITE_PROFILES); IPR009003 (SUPERFAMILY); TMhelix (TMHMM)
1117	271804_length_601_cvg_3.5_tip_1_2	21	heat shock protein 70	200	1.86E-22	74.80%	0.1		IPR029048 (G3DSA:1.20.1270.GENE3D); PTHR19375 (PANTHER); PTHR19375:SF155 (PANTHER); IPR029048 (SUPERFAMILY)
1118	271810_length_601_cvg_14.8_tip_1_5	110	plasminogen activator spa	200	5.36E-28	55.50%	0.366	Y	IPR001314 (PRINTS); IPR001254 (SMART); G3DSA:2.40.10.10 (GENE3D); G3DSA:2.40.10.10 (GENE3D); G3DSA:2.40.10.10 (GENE3D); IPR001254 (PFAM); PTHR24276 (PANTHER); IPR018114 (PROSITE_PATTERNS); SIGNAL_PEPTIDE_N_REGION (PHOBIUS); SIGNAL_PEPTIDE_H_REGION (PHOBIUS); NON_CYTOPLASMIC_DOMAIN (PHOBIUS); SIGNAL_PEPTIDE_C_REGION (PHOBIUS); SIGNAL_PEPTIDE (PHOBIUS); IPR001254 (PROSITE_PROFILES); IPR009003 (SUPERFAMILY); TMhelix (TMHMM)
1119	271856_length_602_cvg_73.4_tip_1_3	5433	cathepsin l2 cysteine protease	201	9.07E-62	68.60%	0.348	Y	IPR000668 (SMART); IPR013201 (SMART); IPR000668 (PFAM); G3DSA:3.90.70.10 (GENE3D); IPR013201 (PFAM); PTHR12411:SF57 (PANTHER); IPR013128 (PANTHER); IPR000169 (PROSITE_PATTERNS); SIGNAL_PEPTIDE (PHOBIUS); SIGNAL_PEPTIDE_C_REGION (PHOBIUS); SIGNAL_PEPTIDE_H_REGION (PHOBIUS); SIGNAL_PEPTIDE_N_REGION (PHOBIUS); NON_CYTOPLASMIC_DOMAIN (PHOBIUS); SSF54001 (SUPERFAMILY)
1120	271870_length_602_cvg_3.9_tip_1_3	33	sucrase- intestinal-like	201	1.31E-43	70.40%	0.298		IPR000322 (PFAM); PTHR22762 (PANTHER); PTHR22762:SF7 (PANTHER); IPR017853 (SUPERFAMILY)
1121	271922_length_602_cvg_2.0_tip_1_3	14	tolloid-like protein 2 isoform x1	201	9.01E-73	68.50%	0.132		IPR002172 (SMART); IPR000859 (SMART); IPR000859 (PFAM); IPR000859 (G3DSA:2.60.120.GENE3D); IPR002172 (G3DSA:4.10.400.GENE3D); PTHR10127:SF557 (PANTHER); PTHR10127 (PANTHER); CYTOPLASMIC_DOMAIN (PHOBIUS); TRANSMEMBRANE (PHOBIUS); NON_CYTOPLASMIC_DOMAIN (PHOBIUS); IPR000859 (PROSITE_PROFILES); IPR000859 (SUPERFAMILY); IPR002172 (SUPERFAMILY); TMhelix (TMHMM)
1122	272074_length_604_cvg_4.0_tip_1_4	38	sortilin-related receptor-like	201	2.84E-66	66.20%	0.131		PTHR12106 (PANTHER); PTHR12106:SF7 (PANTHER)
1123	272226_length_606_cvg_7.5_tip_1_3	53	nf-kappa-b inhibitor	202	8.37E-63	66.20%	0.143		IPR002110 (SMART); IPR020683 (PFAM); IPR020683 (G3DSA:1.25.40.GENE3D); PTHR24139 (PANTHER); IPR002110 (PROSITE_PROFILES); IPR002110 (PROSITE_PROFILES); IPR002110 (PROSITE_PROFILES); IPR020683 (PROSITE_PROFILES); IPR020683 (SUPERFAMILY)
1125	272328_length_607_cvg_5.6_tip_1_2	44	mitochondrial import inner membrane translocase subunit tim13	202	9.92E-44	86.30%	0.171		IPR004217 (PFAM); IPR004217 (G3DSA:1.10.287.GENE3D); PTHR19338 (PANTHER); IPR004217 (SUPERFAMILY)
1126	272382_length_607_cvg_17.8_tip_1_2	138	myotrophin-like isoform 2	202	1.51E-55	84.20%	0.14		IPR002110 (SMART); IPR020683 (PFAM); IPR020683 (G3DSA:1.25.40.GENE3D); PTHR24152 (PANTHER); IPR020683 (PROSITE_PROFILES); IPR002110 (PROSITE_PROFILES); IPR020683 (SUPERFAMILY)

1124	272316_length_607_cvg_4.0_tip_1_3	31	zinc finger protein partial	203	1.26E-32	61.40%	0.106		IPR015880 (SMART); IPR007087 (PFAM); IPR013087 (G3DSA:3.30.160.GENE3D); PF13465 (PFAM); IPR013087 (G3DSA:3.30.160.GENE3D); IPR013087 (G3DSA:3.30.160.GENE3D); IPR013087 (G3DSA:3.30.160.GENE3D); PTHR24409 (PANTHER); IPR007087 (PROSITE_PATTERNS); IPR007087 (PROSITE_PATTERNS); IPR007087 (PROSITE_PATTERNS); IPR007087 (PROSITE_PATTERNS); IPR007087 (PROSITE_PROFILES); IPR007087 (PROSITE_PROFILES); IPR007087 (PROSITE_PROFILES); IPR007087 (PROSITE_PROFILES); SSF57667 (SUPERFAMILY); SSF57667 (SUPERFAMILY); SSF57667 (SUPERFAMILY)
1127	272508_length_609_cvg_3.5_tip_1_2	26	fat-like cadherin-related tumor suppressor homolog isoform x1	203	6.36E-90	80.00%	0.098		IPR000742 (SMART); G3DSA:2.10.25.10 (GENE3D); IPR013320 (G3DSA:2.60.120.GENE3D); IPR001791 (PFAM); G3DSA:2.10.25.10 (GENE3D); PTHR24026:SF39 (PANTHER); PTHR24026 (PANTHER); IPR013032 (PROSITE_PATTERNS); IPR013032 (PROSITE_PATTERNS); IPR013032 (PROSITE_PATTERNS); IPR013032 (PROSITE_PATTERNS); IPR000742 (PROSITE_PROFILES); IPR001791 (PROSITE_PROFILES); IPR013320 (SUPERFAMILY)
1128	272584_length_609_cvg_67.9_tip_0_3	2316	mite allergen lep d partial	203	1.45E-17	49.80%	0.124		PTHR11008 (PANTHER); PTHR11008:SF13 (PANTHER); IPR020234 (PRODOM)
1129	272632_length_610_cvg_4.0_tip_1_4	29	reelin- partial	203	1.71E-42	58.90%	0.273		PTHR11841 (PANTHER); IPR000421 (PROSITE_PROFILES)
1130	272640_length_610_cvg_2.7_tip_1_3	25	laminin a	204	1.04E-78	72.70%	0.115		Coil (COILS); Coil (COILS); PR00011 (PRINTS); IPR002049 (SMART); G3DSA:2.10.25.10 (GENE3D); IPR002049 (PFAM); PTHR10574 (PANTHER); PTHR10574:SF231 (PANTHER); IPR002049 (PROSITE_PATTERNS); IPR002049 (PROSITE_PATTERNS); IPR002049 (PROSITE_PROFILES); IPR002049 (PROSITE_PROFILES); SSF57196 (SUPERFAMILY); SSF57196 (SUPERFAMILY)
1131	272684_length_611_cvg_52.3_tip_0_3	336	heat shock 70 kda protein cognate 4	204	3.41E-40	78.70%	0.1		IPR013126 (PFAM); IPR029048 (G3DSA:1.20.1270.GENE3D); PTHR19375:SF142 (PANTHER); PTHR19375 (PANTHER); IPR029048 (SUPERFAMILY)
1132	272730_length_611_cvg_4.5_tip_1_3	34	furin-like protease isoform 1-crr isoform x2	204	2.41E-15	53.60%	0.115		IPR006212 (SMART); G3DSA:2.10.220.10 (GENE3D); NON_CYTOPLASMIC_DOMAIN (PHOBIUS); TRANSMEMBRANE (PHOBIUS); CYTOPLASMIC_DOMAIN (PHOBIUS); IPR009030 (SUPERFAMILY); TMhelix (TMHMM)
1133	272770_length_612_cvg_6.9_tip_1_1	52	fasciclin-2 isoform x2	204	6.30E-11	47.50%	0.441	Y	IPR003598 (SMART); IPR003599 (SMART); IPR013783 (G3DSA:2.60.40.GENE3D); IPR013783 (G3DSA:2.60.40.GENE3D); IPR013098 (PFAM); PTHR10489 (PANTHER); NON_CYTOPLASMIC_DOMAIN (PHOBIUS); SIGNAL_PEPTIDE (PHOBIUS); SIGNAL_PEPTIDE_N_REGION (PHOBIUS); SIGNAL_PEPTIDE_C_REGION (PHOBIUS); SIGNAL_PEPTIDE_H_REGION (PHOBIUS); IPR007110 (PROSITE_PROFILES); SSF48726 (SUPERFAMILY)
1134	272796_length_612_cvg_2.0_tip_1_0	16	zinc finger protein 26	204	1.04E-52	62.60%	0.1		IPR015880 (SMART); IPR013087 (G3DSA:3.30.160.GENE3D); IPR013087 (G3DSA:3.30.160.GENE3D); IPR007087 (PFAM); IPR013087 (G3DSA:3.30.160.GENE3D); PF13465 (PFAM); IPR013087 (G3DSA:3.30.160.GENE3D); PTHR24409 (PANTHER); IPR007087 (PROSITE_PATTERNS); IPR007087 (PROSITE_PATTERNS); IPR007087 (PROSITE_PATTERNS); IPR007087 (PROSITE_PATTERNS); IPR007087 (PROSITE_PATTERNS); IPR007087 (PROSITE_PATTERNS); IPR007087 (PROSITE_PATTERNS); IPR007087 (PROSITE_PROFILES); IPR007087 (PROSITE_PROFILES); IPR007087 (PROSITE_PROFILES); IPR007087 (PROSITE_PROFILES); IPR007087 (PROSITE_PROFILES); IPR007087 (PROSITE_PROFILES); IPR007087 (PROSITE_PROFILES); SSF57667 (SUPERFAMILY); SSF57667 (SUPERFAMILY); SSF57667 (SUPERFAMILY); SSF57667 (SUPERFAMILY)
1135	272892_length_613_cvg_2.7_tip_1_4	23	zinc finger protein 235	204	6.53E-11	48.90%	0.099		IPR015880 (SMART); PF13894 (PFAM); IPR007087 (PFAM); IPR013087 (G3DSA:3.30.160.GENE3D); PTHR24409:SF14 (PANTHER); PTHR24409 (PANTHER); IPR007087 (PROSITE_PATTERNS); IPR007087 (PROSITE_PATTERNS); IPR007087 (PROSITE_PROFILES); IPR007087 (PROSITE_PROFILES); SSF57667 (SUPERFAMILY)
1136	272938_length_614_cvg_62.2_tip_0_3	6350	inactive pancreatic lipase-related protein 1	205	9.02E-57	63.60%	0.126		IPR002331 (PRINTS); IPR000734 (PRINTS); IPR029058 (G3DSA:3.40.50.GENE3D); IPR013818 (PFAM); IPR000734 (PANTHER); IPR029058 (SUPERFAMILY)
1137	273012_length_615_cvg_4.0_tip_1_3	27	zinc finger protein 676-like	205	2.85E-36	66.40%	0.101		IPR015880 (SMART); PF13465 (PFAM); IPR013087 (G3DSA:3.30.160.GENE3D); IPR013087 (G3DSA:3.30.160.GENE3D); PTHR24409 (PANTHER); IPR007087 (PROSITE_PATTERNS); IPR007087 (PROSITE_PATTERNS); IPR007087 (PROSITE_PROFILES); IPR007087 (PROSITE_PROFILES); SSF57667 (SUPERFAMILY)

1138	273030_length_615_cvg_20.8_tip_1_0	107	protein shifted	205	6.42E-19	68.90%	0.105	IPR000742 (SMART); G3DSA:2.170.300.10 (GENE3D); IPR013032 (PFAM); PTHR24838 (PANTHER); PTHR24838:SF276 (PANTHER); IPR013032 (PROSITE_PATTERNS); IPR013032 (PROSITE_PATTERNS); IPR013032 (PROSITE_PATTERNS); IPR013032 (PROSITE_PATTERNS); IPR000742 (PROSITE_PROFILES); IPR000742 (PROSITE_PROFILES)
1139	273038_length_615_cvg_2.0_tip_1_3	21	c-type lectin domain family 4 member g-like isoform x2	205	1.56E-09	45.80%	0.112	IPR001304 (SMART); IPR001304 (PFAM); IPR016186 (G3DSA:3.10.100.GENE3D); PTHR22802 (PANTHER); IPR001304 (PROSITE_PROFILES); IPR016187 (SUPERFAMILY)
1140	273040_length_615_cvg_4.9_tip_1_3	42	glucose dehydrogenase	205	5.11E-55	68.70%	0.156	IPR000172 (PFAM); G3DSA:3.50.50.60 (GENE3D); PTHR11552 (PANTHER); PTHR11552:SF77 (PANTHER); IPR000172 (PROSITE_PATTERNS); CYTOPLASMIC_DOMAIN (PHOBIUS); NON_CYTOPLASMIC_DOMAIN (PHOBIUS); TRANSMEMBRANE (PHOBIUS); SSF51905 (SUPERFAMILY); TMhelix (TMHMM)
1141	273046_length_615_cvg_2.0_tip_1_3	13	zinc finger protein 271 (zinc finger protein 7) (zinc finger protein znfphex133) (epstein-barr virus-induced zinc finger protein) (znf-eb) (ct-zfp48) (zinc finger protein	205	2.16E-92	68.80%	0.113	IPR015880 (SMART); IPR007087 (PFAM); IPR013087 (G3DSA:3.30.160.GENE3D); IPR013087 (G3DSA:3.30.160.GENE3D); IPR013087 (G3DSA:3.30.160.GENE3D); IPR013087 (G3DSA:3.30.160.GENE3D); PF13465 (PFAM); PTHR24409 (PANTHER); IPR007087 (PROSITE_PATTERNS); IPR007087 (PROSITE_PATTERNS); IPR007087 (PROSITE_PATTERNS); IPR007087 (PROSITE_PATTERNS); IPR007087 (PROSITE_PATTERNS); IPR007087 (PROSITE_PROFILES); IPR007087 (PROSITE_PROFILES); IPR007087 (PROSITE_PROFILES); IPR007087 (PROSITE_PROFILES); IPR007087 (PROSITE_PROFILES); IPR007087 (PROSITE_PROFILES); IPR007087 (PROSITE_PROFILES); SSF57667 (SUPERFAMILY); SSF57667 (SUPERFAMILY); SSF57667 (SUPERFAMILY); SSF57667 (SUPERFAMILY)
1142	273062_length_615_cvg_2.0_tip_1_2	19	leukotriene a-4 hydrolase	205	1.28E-71	73.10%	0.135	IPR014782 (PRINTS); IPR014782 (PFAM); PTHR11533:SF4 (PANTHER); IPR001930 (PANTHER); SSF63737 (SUPERFAMILY)
1143	273112_length_616_cvg_9.4_tip_1_1	81	---NA---	205			0.118	no IPS match
1144	273200_length_617_cvg_3.0_tip_1_5	22	serine protease snake	205	3.67E-14	54.50%	0.143	IPR001254 (PFAM); G3DSA:2.40.10.10 (GENE3D); G3DSA:2.40.10.10 (GENE3D); G3DSA:2.40.10.10 (GENE3D); PTHR24268:SF81 (PANTHER); PTHR24268 (PANTHER); IPR001254 (PROSITE_PROFILES); IPR009003 (SUPERFAMILY); IPR009003 (SUPERFAMILY)
1145	273282_length_618_cvg_60.2_tip_1_0	468	thioredoxin peroxidase 2	206	5.01E-10	85.60%	0.101	IPR012336 (G3DSA:3.40.30.GENE3D); IPR019479 (PFAM); PTHR10681 (PANTHER); NON_CYTOPLASMIC_DOMAIN (PHOBIUS); CYTOPLASMIC_DOMAIN (PHOBIUS); TRANSMEMBRANE (PHOBIUS); IPR012336 (SUPERFAMILY)
1146	273362_length_618_cvg_36.4_tip_1_3	223	type iv secretion protein	206	3.40E-23	45.50%	0.206	no IPS match
1147	273366_length_618_cvg_3.0_tip_1_5	25	epithelial chloride channel partial	206	3.63E-15	48.40%	0.099	IPR002035 (G3DSA:3.40.50.GENE3D); PTHR10579:SF8 (PANTHER); PTHR10579 (PANTHER); IPR002035 (SUPERFAMILY)
1148	273368_length_619_cvg_5.9_tip_1_5	48	trypsinogen 1	206	1.73E-39	58.60%	0.215	IPR001314 (PRINTS); IPR001254 (SMART); IPR001254 (PFAM); G3DSA:2.40.10.10 (GENE3D); G3DSA:2.40.10.10 (GENE3D); G3DSA:2.40.10.10 (GENE3D); PTHR24265 (PANTHER); IPR018114 (PROSITE_PATTERNS); TRANSMEMBRANE (PHOBIUS); NON_CYTOPLASMIC_DOMAIN (PHOBIUS); CYTOPLASMIC_DOMAIN (PHOBIUS); IPR001254 (PROSITE_PROFILES); IPR009003 (SUPERFAMILY); TMhelix (TMHMM)
1149	273384_length_619_cvg_3.0_tip_1_0	23	map kinase-activated protein kinase 2	207	2.99E-102	87.70%	0.126	IPR002290 (SMART); IPR027442 (G3DSA:4.10.1170.GENE3D); G3DSA:1.10.510.10 (GENE3D); IPR000719 (PFAM); PTHR24349 (PANTHER); PTHR24349:SF63 (PANTHER); TRANSMEMBRANE (PHOBIUS); NON_CYTOPLASMIC_DOMAIN (PHOBIUS); CYTOPLASMIC_DOMAIN (PHOBIUS); IPR000719 (PROSITE_PROFILES); IPR011009 (SUPERFAMILY); TMhelix (TMHMM)
1150	273412_length_619_cvg_3.0_tip_1_0	23	alpha-mannosidase 2	207	7.43E-117	84.10%	0.104	IPR000602 (PFAM); IPR027291 (G3DSA:3.20.110.GENE3D); PTHR11607 (PANTHER); PTHR11607:SF22 (PANTHER); IPR011330 (SUPERFAMILY)
1151	273486_length_620_cvg_4.2_tip_1_0	38	sortilin-related l(dlr class) a repeats-containing-like isoform x2	207	5.88E-72	66.40%	0.101	PTHR12106 (PANTHER); PTHR12106:SF7 (PANTHER)
1152	273576_length_621_cvg_3.0_tip_1_3	19	receptor-type tyrosine-protein phosphatase partial	207	2.10E-17	51.50%	0.2	IPR000242 (PFAM); IPR029021 (G3DSA:3.90.190.GENE3D); PTHR19134 (PANTHER); TRANSMEMBRANE (PHOBIUS); NON_CYTOPLASMIC_DOMAIN (PHOBIUS); TRANSMEMBRANE (PHOBIUS); CYTOPLASMIC_DOMAIN (PHOBIUS); CYTOPLASMIC_DOMAIN (PHOBIUS); IPR000242 (PROSITE_PROFILES); IPR029021 (SUPERFAMILY); TMhelix (TMHMM); TMhelix (TMHMM)
1153	273602_length_621_cvg_8.1_tip_1_4	62	leucine-rich repeat-containing protein 57	207	4.16E-72	77.70%	0.099	IPR003591 (SMART); IPR025875 (PFAM); G3DSA:3.80.10.10 (GENE3D); IPR001611 (PFAM); PTHR23155 (PANTHER); IPR001611 (PROSITE_PROFILES); IPR001611 (PROSITE_PROFILES); IPR001611 (PROSITE_PROFILES); IPR001611 (PROSITE_PROFILES); SSF52058 (SUPERFAMILY)

1154	273632_length_622_cvg_3.2_tip_1_4	24	lysosomal pro-x carboxypeptidase	207	2.86E-82	72.00%	0.105	IPR008758 (PFAM); IPR008758 (PANTHER); PTHR11010:SF11 (PANTHER); IPR029058 (SUPERFAMILY)
1155	273726_length_623_cvg_3.4_tip_1_5	26	limulus clotting factor partial	207	4.36E-28	50.70%	0.122	G3DSA:2.40.10.10 (GENE3D); G3DSA:2.10.25.10 (GENE3D); IPR001254 (PFAM); G3DSA:2.40.10.10 (GENE3D); IPR026823 (PFAM); PTHR24265 (PANTHER); IPR018114 (PROSITE_PATTERNS); IPR013032 (PROSITE_PATTERNS); IPR001254 (PROSITE_PROFILES); IPR000436 (PROSITE_PROFILES); IPR009003 (SUPERFAMILY)
1156	273824_length_624_cvg_2.0_tip_1_4	18	ryanodine receptor 44f	208	1.61E-96	83.10%	0.113	IPR003032 (PFAM); IPR003877 (PFAM); IPR015925 (PANTHER); PTHR13715:SF74 (PANTHER); IPR001870 (PROSITE_PROFILES); IPR013320 (SUPERFAMILY)
1157	273856_length_625_cvg_2.0_tip_1_1	18	receptor-type tyrosine-protein phosphatase f	208	4.51E-81	78.80%	0.1	PR00014 (PRINTS); IPR003961 (SMART); IPR013783 (G3DSA:2.60.40.GENE3D); IPR003961 (PFAM); IPR013783 (G3DSA:2.60.40.GENE3D); PTHR10489 (PANTHER); IPR003961 (PROSITE_PROFILES); IPR003961 (PROSITE_PROFILES); IPR003961 (PROSITE_PROFILES); IPR003961 (SUPERFAMILY)
1158	274000_length_627_cvg_2.0_tip_1_0	13	tyrosine-protein kinase btk29a isoform x3	209	5.10E-126	92.10%	0.112	IPR001245 (PRINTS); IPR020635 (SMART); G3DSA:1.10.510.10 (GENE3D); IPR001245 (PFAM); G3DSA:3.30.200.20 (GENE3D); PTHR24418 (PANTHER); PTHR24418:SF162 (PANTHER); IPR008266 (PROSITE_PATTERNS); IPR017441 (PROSITE_PATTERNS); IPR000719 (PROSITE_PROFILES); IPR011009 (SUPERFAMILY)
1159	274060_length_627_cvg_7.5_tip_1_3	53	venom carboxylesterase-6-like	209	2.91E-50	52.30%	0.104	IPR029058 (G3DSA:3.40.50.GENE3D); IPR002018 (PFAM); PTHR11559:SF198 (PANTHER); PTHR11559 (PANTHER); IPR029058 (SUPERFAMILY)
1182	275450_length_645_cvg_8.7_tip_1_5	56	protoporphyrin ix magnesium chelatase	209	2.64E-17	60.20%	0.12	CYTOPLASMIC_DOMAIN (PHOBIUS); TRANSMEMBRANE (PHOBIUS); CYTOPLASMIC_DOMAIN (PHOBIUS); NON_CYTOPLASMIC_DOMAIN (PHOBIUS); TRANSMEMBRANE (PHOBIUS); TMhelix (TMHMM); TMhelix (TMHMM)
1160	274216_length_629_cvg_3.0_tip_1_3	27	latrophilin-3- partial	210	1.85E-18	60.70%	0.162	IPR000832 (PFAM); PTHR12011:SF222 (PANTHER); PTHR12011 (PANTHER); TRANSMEMBRANE (PHOBIUS); TRANSMEMBRANE (PHOBIUS); CYTOPLASMIC_DOMAIN (PHOBIUS); NON_CYTOPLASMIC_DOMAIN (PHOBIUS); CYTOPLASMIC_DOMAIN (PHOBIUS); TMhelix (TMHMM); TMhelix (TMHMM)
1161	274290_length_630_cvg_7.8_tip_1_0	61	spondin-1-like	210	1.54E-79	68.50%	0.233	IPR000884 (SMART); IPR000884 (PFAM); IPR009465 (PFAM); G3DSA:2.20.100.10 (GENE3D); PTHR11311 (PANTHER); IPR009465 (PROSITE_PROFILES); IPR000884 (PROSITE_PROFILES); IPR000884 (SUPERFAMILY)
1162	274328_length_630_cvg_6.3_tip_1_5	56	peptidyl-prolyl cis-trans isomerase-like 1	210	2.55E-79	92.50%	0.103	IPR002130 (PRINTS); IPR002130 (PFAM); IPR029000 (G3DSA:2.40.100.GENE3D); IPR024936 (PANTHER); IPR020892 (PROSITE_PATTERNS); IPR002130 (PROSITE_PROFILES); IPR029000 (SUPERFAMILY)
1163	274382_length_631_cvg_3.0_tip_1_4	29	serine threonine-protein kinase sgk1 isoform x1	210	1.55E-122	89.00%	0.097	IPR002290 (SMART); G3DSA:1.10.510.10 (GENE3D); IPR000719 (PFAM); PTHR24356 (PANTHER); PTHR24356:SF121 (PANTHER); IPR008271 (PROSITE_PATTERNS); IPR000719 (PROSITE_PROFILES); IPR000961 (PROSITE_PROFILES); IPR011009 (SUPERFAMILY)
1164	274388_length_631_cvg_22.1_tip_1_2	146	cytochrome p450 partial	210	2.37E-54	67.70%	0.1	IPR002401 (PRINTS); IPR001128 (PRINTS); IPR001128 (PFAM); IPR001128 (G3DSA:1.10.630.GENE3D); PTHR24305 (PANTHER); PTHR24305:SF49 (PANTHER); IPR017972 (PROSITE_PATTERNS); CYTOPLASMIC_DOMAIN (PHOBIUS); NON_CYTOPLASMIC_DOMAIN (PHOBIUS); TRANSMEMBRANE (PHOBIUS); IPR001128 (SUPERFAMILY); TMhelix (TMHMM)
1165	274394_length_631_cvg_5.1_tip_1_2	40	xanthine dehydrogenase	210	4.81E-81	83.40%	0.178	IPR008274 (PFAM); IPR008274 (G3DSA:3.30.365.GENE3D); PTHR11908:SF32 (PANTHER); PTHR11908 (PANTHER); IPR008274 (SUPERFAMILY)
1166	274404_length_631_cvg_5.3_tip_1_4	27	---NA---	210			0.118	Coil (COILS)
1167	274430_length_632_cvg_37.4_tip_1_2	330	poly -specific endoribonuclease homolog	210	1.38E-57	65.90%	0.116	IPR018998 (PFAM); PTHR12439 (PANTHER); SSF142877 (SUPERFAMILY)
1168	274538_length_633_cvg_8.0_tip_1_5	59	von willebrand factor a domain-containing protein 8-like	211	9.92E-48	80.70%	0.116	IPR011704 (PFAM); PTHR21610:SF9 (PANTHER); PTHR21610 (PANTHER)
1170	274602_length_634_cvg_3.9_tip_1_5	37	aael005189- partial	211	3.12E-80	69.20%	0.115	IPR003112 (SMART); IPR003112 (PFAM); PTHR23192 (PANTHER); PTHR23192:SF33 (PANTHER); IPR003112 (PROSITE_PROFILES); SSF50993 (SUPERFAMILY)
1171	274662_length_634_cvg_4.1_tip_1_5	33	pdz domain-containing protein 11	211	2.10E-52	76.90%	0.106	IPR001478 (SMART); IPR001478 (PFAM); IPR001478 (G3DSA:2.30.42.GENE3D); PTHR14063 (PANTHER); IPR001478 (PROSITE_PROFILES); IPR001478 (SUPERFAMILY)
1169	274598_length_634_cvg_23.2_tip_1_0	125	alpha-n-acetylgalactosaminidase isoform x4	212	4.66E-92	85.50%	0.15	IPR002241 (PRINTS); IPR000111 (PFAM); IPR013785 (G3DSA:3.20.20.GENE3D); PTHR11452 (PANTHER); PTHR11452:SF23 (PANTHER); IPR000111 (PROSITE_PATTERNS); TRANSMEMBRANE (PHOBIUS); CYTOPLASMIC_DOMAIN (PHOBIUS); NON_CYTOPLASMIC_DOMAIN (PHOBIUS); IPR017853 (SUPERFAMILY)

1172	274828_length_637_cvg_12.8_tip_1_2	84	trypsin-like serine proteinase	212	1.81E-54	60.50%	0.285		IPR001314 (PRINTS); IPR001254 (SMART); IPR001254 (PFAM); G3DSA:2.40.10.10 (GENE3D); G3DSA:2.40.10.10 (GENE3D); PTHR24256 (PANTHER); IPR018114 (PROSITE_PATTERNS); IPR018114 (PROSITE_PATTERNS); IPR001254 (PROSITE_PROFILES); IPR009003 (SUPERFAMILY)
1173	274938_length_638_cvg_8.5_tip_1_2	67	coatomer subunit beta partial	212	5.30E-143	95.20%	0.11		IPR001680 (SMART); IPR015943 (G3DSA:2.130.10.GENE3D); IPR001680 (PFAM); PTHR19876 (PANTHER); IPR001680 (PROSITE_PROFILES); IPR017986 (PROSITE_PROFILES); IPR001680 (PROSITE_PROFILES); IPR001680 (PROSITE_PROFILES); IPR017986 (SUPERFAMILY)
1175	275080_length_640_cvg_50.8_tip_1_2	314	muscle-specific protein 20	213	2.06E-84	86.10%	0.464	Y	IPR003096 (PRINTS); IPR001997 (PRINTS); IPR001715 (SMART); IPR000557 (PFAM); IPR001715 (PFAM); IPR001715 (G3DSA:1.10.418.GENE3D); PTHR18959 (PANTHER); PTHR18959:SF51 (PANTHER); IPR001715 (PROSITE_PROFILES); IPR000557 (PROSITE_PROFILES); SignalP-noTM (SIGNALP_EUK); IPR001715 (SUPERFAMILY)
1179	275192_length_641_cvg_86.6_tip_1_2	9375	chitinase partial	213	5.06E-80	75.60%	0.307		IPR011583 (SMART); IPR013781 (G3DSA:3.20.20.GENE3D); IPR001223 (PFAM); PTHR11177:SF112 (PANTHER); PTHR11177 (PANTHER); NON_CYTOPLASMIC_DOMAIN (PHOBIUS); TRANSMEMBRANE (PHOBIUS); CYTOPLASMIC_DOMAIN (PHOBIUS); IPR017853 (SUPERFAMILY); TMhelix (TMHMM)
1174	275060_length_640_cvg_10.5_tip_1_3	69	---NA---	214			0.145		no IPS match
1176	275116_length_641_cvg_3.7_tip_1_0	40	serine protease snake	214	5.86E-08	58.70%	0.103		IPR001254 (PFAM); G3DSA:2.40.10.10 (GENE3D); PTHR24260 (PANTHER); IPR018114 (PROSITE_PATTERNS); IPR009003 (SUPERFAMILY)
1177	275146_length_641_cvg_2.5_tip_1_4	23	cysteine protease inhibitor	214	2.67E-21	50.60%	0.098		IPR000010 (SMART); IPR000010 (PFAM); G3DSA:3.10.450.10 (GENE3D); G3DSA:3.10.450.10 (GENE3D); PTHR11413:SF26 (PANTHER); IPR027214 (PANTHER); IPR018073 (PROSITE_PATTERNS); SSF54403 (SUPERFAMILY); SSF54403 (SUPERFAMILY)
1178	275170_length_641_cvg_2.0_tip_1_4	14	fibronectin type-iii domain-containing protein 3a-like isoform x5	214	1.52E-73	74.10%	0.143		PR00014 (PRINTS); IPR003961 (SMART); IPR013783 (G3DSA:2.60.40.GENE3D); IPR003961 (PFAM); IPR013783 (G3DSA:2.60.40.GENE3D); IPR013783 (G3DSA:2.60.40.GENE3D); PTHR19900 (PANTHER); PTHR19900:SF50 (PANTHER); IPR003961 (PROSITE_PROFILES); IPR003961 (PROSITE_PROFILES); IPR003961 (PROSITE_PROFILES); IPR003961 (SUPERFAMILY); IPR003961 (SUPERFAMILY)
1180	275410_length_644_cvg_2.9_tip_1_4	26	eukaryotic translation initiation factor 2-alpha kinase partial	215	2.48E-79	72.00%	0.1		IPR002290 (SMART); G3DSA:1.10.510.10 (GENE3D); IPR000719 (PFAM); PTHR11042:SF86 (PANTHER); PTHR11042 (PANTHER); IPR008271 (PROSITE_PATTERNS); IPR000719 (PROSITE_PROFILES); IPR011009 (SUPERFAMILY)
1181	275424_length_644_cvg_8.2_tip_1_4	54	tyrosine-protein kinase dnt-like	215	6.34E-114	83.10%	0.107		IPR001245 (PRINTS); IPR020635 (SMART); G3DSA:1.10.510.10 (GENE3D); G3DSA:3.30.200.20 (GENE3D); IPR001245 (PFAM); PTHR24416 (PANTHER); PTHR24416:SF256 (PANTHER); IPR008266 (PROSITE_PATTERNS); IPR000719 (PROSITE_PROFILES); IPR011009 (SUPERFAMILY)
1183	275488_length_646_cvg_35.0_tip_1_4	268	peptidase s8	215	3.24E-25	69.80%	0.378	Y	IPR007280 (PFAM); SIGNAL_PEPTIDE (PHOBIUS); SIGNAL_PEPTIDE_N_REGION (PHOBIUS); SIGNAL_PEPTIDE_H_REGION (PHOBIUS); NON_CYTOPLASMIC_DOMAIN (PHOBIUS); SIGNAL_PEPTIDE_C_REGION (PHOBIUS); TMhelix (TMHMM)
1184	275542_length_646_cvg_3.0_tip_1_2	23	thrombospondin type-1 domain-containing protein partial	215	8.02E-36	64.00%	0.125		IPR000884 (SMART); IPR010909 (PFAM); IPR000884 (PFAM); G3DSA:2.20.100.10 (GENE3D); PTHR13723 (PANTHER); IPR010909 (PROSITE_PROFILES); IPR000884 (PROSITE_PROFILES); IPR000884 (SUPERFAMILY)
1185	275630_length_648_cvg_2.4_tip_1_4	27	zinc finger protein 226-like	216	2.91E-30	58.00%	0.1		IPR015880 (SMART); IPR013087 (G3DSA:3.30.160.GENE3D); IPR013087 (G3DSA:3.30.160.GENE3D); IPR007087 (PFAM); PF13465 (PFAM); IPR013087 (G3DSA:3.30.160.GENE3D); PF13912 (PFAM); PTHR24409:SF13 (PANTHER); PTHR24409 (PANTHER); IPR007087 (PROSITE_PATTERNS); IPR007087 (PROSITE_PATTERNS); IPR007087 (PROSITE_PATTERNS); IPR007087 (PROSITE_PATTERNS); IPR007087 (PROSITE_PATTERNS); IPR007087 (PROSITE_PROFILES); IPR007087 (PROSITE_PROFILES); IPR007087 (PROSITE_PROFILES); IPR007087 (PROSITE_PROFILES); IPR007087 (PROSITE_PROFILES); IPR007087 (PROSITE_PROFILES); SSF57667 (SUPERFAMILY); SSF57667 (SUPERFAMILY); SSF57667 (SUPERFAMILY)
1186	275650_length_648_cvg_4.0_tip_1_2	32	lactase-phlorizin hydrolase-like	216	2.02E-71	67.50%	0.112		IPR013781 (G3DSA:3.20.20.GENE3D); IPR001360 (PFAM); IPR001360 (PANTHER); IPR017853 (SUPERFAMILY)
1188	275706_length_649_cvg_5.0_tip_1_2	37	protein cbg06875	216	6.09E-17	62.10%	0.144		PTHR11660:SF46 (PANTHER); PTHR11660 (PANTHER)

1189	275756_length_649_cvg_64.9_tip_1_5	6486	cathepsin l2 cysteine protease	216	7.14E-64	69.10%	0.172	IPR000668 (SMART); IPR013201 (SMART); G3DSA:3.90.70.10 (GENE3D); IPR013201 (PFAM); IPR000668 (PFAM); IPR013128 (PANTHER); PTHR12411:SF57 (PANTHER); IPR000169 (PROSITE_PATTERNS); TRANSMEMBRANE (PHOBIUS); NON_CYTOPLASMIC_DOMAIN (PHOBIUS); CYTOPLASMIC_DOMAIN (PHOBIUS); SSF54001 (SUPERFAMILY)
1187	275700_length_649_cvg_3.6_tip_1_0	26	c3orf33 protein	217	6.65E-23	45.30%	0.15	IPR016071 (G3DSA:2.40.50.GENE3D); IPR016071 (SUPERFAMILY)
1190	275852_length_650_cvg_63.0_tip_0_0	869	kunitz bovine pancreatic trypsin inhibitor domain protein	217	2.58E-51	58.80%	0.11	IPR002223 (PRINTS); IPR002223 (SMART); IPR002223 (PFAM); IPR002223 (G3DSA:4.10.410.GENE3D); IPR002223 (G3DSA:4.10.410.GENE3D); PTHR10083 (PANTHER); IPR020901 (PROSITE_PATTERNS); IPR020901 (PROSITE_PATTERNS); IPR020901 (PROSITE_PATTERNS); IPR002223 (PROSITE_PROFILES); IPR002223 (PROSITE_PROFILES); IPR002223 (PROSITE_PROFILES); IPR002223 (SUPERFAMILY); IPR002223 (SUPERFAMILY); IPR002223 (SUPERFAMILY)
1191	275932_length_651_cvg_7.5_tip_1_2	59	apolipoprotein d-like	217	3.65E-34	56.70%	0.213	IPR000566 (PFAM); IPR012674 (G3DSA:2.40.128.GENE3D); PTHR10612 (PANTHER); PTHR10612:SF7 (PANTHER); TRANSMEMBRANE (PHOBIUS); CYTOPLASMIC_DOMAIN (PHOBIUS); NON_CYTOPLASMIC_DOMAIN (PHOBIUS); IPR011038 (SUPERFAMILY)
1192	275936_length_651_cvg_3.0_tip_1_3	22	low quality protein: maltase- intestinal-like	217	2.74E-54	59.90%	0.154	IPR000322 (PFAM); PTHR22762 (PANTHER); PTHR22762:SF76 (PANTHER); IPR017853 (SUPERFAMILY); SSF51011 (SUPERFAMILY)
1193	276038_length_653_cvg_5.6_tip_1_5	36	camp-dependent protein kinase catalytic subunit	217	3.28E-148	97.10%	0.109	IPR002290 (SMART); IPR000719 (PFAM); G3DSA:3.30.200.20 (GENE3D); G3DSA:1.10.510.10 (GENE3D); PTHR24353 (PANTHER); IPR008271 (PROSITE_PATTERNS); IPR017441 (PROSITE_PATTERNS); IPR000719 (PROSITE_PROFILES); IPR011009 (SUPERFAMILY)
1194	276048_length_653_cvg_7.9_tip_1_0	68	2-oxoglutarate and iron-dependent oxygenase domain-containing protein 3-like	218	2.94E-98	82.20%	0.147	IPR006620 (SMART); IPR005123 (PFAM); PTHR14650:SF1 (PANTHER); PTHR14650 (PANTHER); IPR005123 (PROSITE_PROFILES)
1195	276136_length_654_cvg_7.2_tip_1_0	56	chitinase 5 isoform x1	218	2.48E-57	57.30%	0.104	IPR002557 (SMART); IPR002557 (PFAM); IPR013781 (G3DSA:3.20.20.GENE3D); IPR029070 (G3DSA:3.10.50.GENE3D); IPR001223 (PFAM); IPR002557 (G3DSA:2.170.140.GENE3D); PTHR11177 (PANTHER); PTHR11177:SF144 (PANTHER); IPR002557 (PROSITE_PROFILES); IPR002557 (SUPERFAMILY); IPR029070 (SUPERFAMILY); IPR017853 (SUPERFAMILY)
1196	276140_length_654_cvg_21.0_tip_1_1	169	epithelial chloride channel partial	218	3.29E-45	55.30%	0.131	IPR013642 (PFAM); PTHR10579:SF8 (PANTHER); PTHR10579 (PANTHER)
1197	276150_length_654_cvg_3.5_tip_1_1	26	gastrula zinc finger	218	2.51E-37	54.60%	0.097	IPR015880 (SMART); IPR013087 (G3DSA:3.30.160.GENE3D); PF13912 (PFAM); PF13465 (PFAM); IPR013087 (G3DSA:3.30.160.GENE3D); IPR013087 (G3DSA:3.30.160.GENE3D); PF13894 (PFAM); IPR007087 (PFAM); PTHR24409 (PANTHER); IPR007087 (PROSITE_PATTERNS); IPR007087 (PROSITE_PATTERNS); IPR007087 (PROSITE_PATTERNS); IPR007087 (PROSITE_PROFILES); IPR007087 (PROSITE_PROFILES); IPR007087 (PROSITE_PROFILES); IPR007087 (PROSITE_PROFILES); SSF57667 (SUPERFAMILY); SSF57667 (SUPERFAMILY)
1198	276156_length_654_cvg_4.6_tip_1_4	34	basement membrane-specific heparan sulfate proteoglycan core partial	218	3.30E-24	66.40%	0.103	IPR003599 (SMART); IPR003598 (SMART); PF13895 (PFAM); IPR013783 (G3DSA:2.60.40.GENE3D); PTHR10489 (PANTHER); IPR007110 (PROSITE_PROFILES); SSF48726 (SUPERFAMILY)
1199	276170_length_655_cvg_69.3_tip_1_5	2551	vp302_lycmc ame: full=venom protein 302 flags: precursor	218	2.68E-14	55.50%	0.779 Y	IPR011390 (PANTHER); PTHR14186:SF8 (PANTHER); SIGNAL_PEPTIDE_H_REGION (PHOBIUS); SIGNAL_PEPTIDE_N_REGION (PHOBIUS); NON_CYTOPLASMIC_DOMAIN (PHOBIUS); SIGNAL_PEPTIDE (PHOBIUS); SIGNAL_PEPTIDE_C_REGION (PHOBIUS); IPR000867 (PROSITE_PROFILES); SignalP-TM (SIGNALP_GRAM_POSITIVE); SignalP-noTM (SIGNALP_GRAM_NEGATIVE); SignalP-noTM (SIGNALP_EUK); IPR009030 (SUPERFAMILY)
1200	276270_length_656_cvg_7.0_tip_1_3	53	fibrillin-2-like	219	7.63E-32	52.60%	0.1	PTHR24039:SF20 (PANTHER); IPR011398 (PANTHER)
1201	276334_length_657_cvg_12.8_tip_1_5	89	low-density lipoprotein receptor	219	1.29E-19	60.20%	0.132	IPR002172 (PRINTS); IPR002172 (SMART); G3DSA:4.10.1220.10 (GENE3D); IPR002172 (PFAM); IPR002172 (G3DSA:4.10.400.GENE3D); IPR002172 (G3DSA:4.10.400.GENE3D); PTHR10529 (PANTHER); IPR023415 (PROSITE_PATTERNS); IPR023415 (PROSITE_PATTERNS); IPR023415 (PROSITE_PATTERNS); IPR002172 (PROSITE_PROFILES); IPR002172 (PROSITE_PROFILES); IPR002172 (PROSITE_PROFILES); IPR002172 (SUPERFAMILY); IPR002172 (SUPERFAMILY); IPR002172 (SUPERFAMILY)
1202	276384_length_658_cvg_2.6_tip_1_2	19	neuroligin-2-like isoform x1	219	1.60E-88	76.30%	0.11	IPR002018 (PFAM); IPR029058 (G3DSA:3.40.50.GENE3D); PTHR11559 (PANTHER); PTHR11559:SF157 (PANTHER); IPR029058 (SUPERFAMILY)
1203	276554_length_660_cvg_47.0_tip_1_5	372	hemagglutinin amebocyte aggregation factor-like	220	2.76E-30	57.60%	0.446 Y	PF14704 (PFAM); IPR026645 (PANTHER); CYTOPLASMIC_DOMAIN (PHOBIUS); NON_CYTOPLASMIC_DOMAIN (PHOBIUS); NON_CYTOPLASMIC_DOMAIN (PHOBIUS); TRANSMEMBRANE (PHOBIUS); CYTOPLASMIC_DOMAIN (PHOBIUS); TRANSMEMBRANE (PHOBIUS); TRANSMEMBRANE (PHOBIUS)

1204	276570_length_660_cvg_3.5_tip_1_1	29	low quality protein: cubilin	220	8.05E-31	52.00%	0.122		IPR000859 (SMART); IPR000859 (PFAM); IPR000859 (G3DSA:2.60.120.GENE3D); IPR000859 (G3DSA:2.60.120.GENE3D); PTHR10127 (PANTHER); IPR000859 (PROSITE_PROFILES); IPR000859 (PROSITE_PROFILES); IPR000859 (SUPERFAMILY); IPR000859 (SUPERFAMILY)
1205	276728_length_663_cvg_4.0_tip_1_5	34	neuropilin and tolloid-like protein 2	221	1.63E-74	65.50%	0.106		IPR000859 (SMART); IPR000859 (PFAM); IPR000859 (G3DSA:2.60.120.GENE3D); IPR000859 (G3DSA:2.60.120.GENE3D); PTHR10127:SF572 (PANTHER); PTHR10127 (PANTHER); IPR000859 (PROSITE_PROFILES); IPR000859 (PROSITE_PROFILES); IPR000859 (SUPERFAMILY); IPR000859 (SUPERFAMILY)
1206	276758_length_663_cvg_54.6_tip_1_0	2120	chymotrypsin-like elastase family member 2a-like	221	1.37E-25	62.10%	0.176		IPR001314 (PRINTS); IPR001254 (SMART); G3DSA:2.40.10.10 (GENE3D); IPR001254 (PFAM); G3DSA:2.40.10.10 (GENE3D); PTHR24276 (PANTHER); IPR018114 (PROSITE_PATTERNS); NON_CYTOPLASMIC_DOMAIN (PHOBIUS); TRANSMEMBRANE (PHOBIUS); CYTOPLASMIC_DOMAIN (PHOBIUS); IPR001254 (PROSITE_PROFILES); IPR009003 (SUPERFAMILY); TMhelix (TMHMM)
1207	276804_length_664_cvg_74.3_tip_1_1	10802	trypsin-like serine proteinase 2	221	3.95E-31	60.40%	0.154		IPR001254 (SMART); G3DSA:2.40.10.10 (GENE3D); IPR001254 (PFAM); PTHR24276 (PANTHER); IPR018114 (PROSITE_PATTERNS); NON_CYTOPLASMIC_DOMAIN (PHOBIUS); CYTOPLASMIC_DOMAIN (PHOBIUS); TRANSMEMBRANE (PHOBIUS); IPR001254 (PROSITE_PROFILES); IPR009003 (SUPERFAMILY); TMhelix (TMHMM)
1228	277930_length_680_cvg_60.7_tip_1_3	1293	clotting factor b-like	221	3.75E-48	64.00%	0.105		IPR001314 (PRINTS); IPR001254 (SMART); G3DSA:2.40.10.10 (GENE3D); IPR001254 (PFAM); G3DSA:2.40.10.10 (GENE3D); PTHR24256 (PANTHER); IPR018114 (PROSITE_PATTERNS); IPR001254 (PROSITE_PROFILES); IPR009003 (SUPERFAMILY)
1208	276928_length_666_cvg_3.0_tip_1_2	27	cysteine proteinase	222	1.78E-30	54.60%	0.157		IPR000010 (SMART); G3DSA:3.10.450.10 (GENE3D); IPR000010 (PFAM); IPR027214 (PANTHER); IPR018073 (PROSITE_PATTERNS); IPR020381 (PRODOM); SSF54403 (SUPERFAMILY); SSF54403 (SUPERFAMILY)
1209	276934_length_666_cvg_6.8_tip_1_3	50	hyaluronidase-like	222	1.15E-49	57.00%	0.104		IPR018155 (PRINTS); IPR018155 (PFAM); IPR013785 (G3DSA:3.20.20.GENE3D); IPR018155 (PANTHER); PTHR11769:SF8 (PANTHER); IPR017853 (SUPERFAMILY)
1210	276950_length_666_cvg_3.3_tip_1_4	30	isoamyl acetate-hydrolyzing esterase 1 homolog	222	1.33E-70	66.90%	0.25		IPR001087 (PFAM); IPR013830 (G3DSA:3.40.50.GENE3D); PTHR14209 (PANTHER); IPR013830 (SUPERFAMILY)
1211	277028_length_667_cvg_22.9_tip_1_2	174	micos complex subunit mic27	222	7.69E-28	55.60%	0.173		IPR019166 (PFAM); PTHR14564:SF1 (PANTHER); PTHR14564 (PANTHER); TRANSMEMBRANE (PHOBIUS); CYTOPLASMIC_DOMAIN (PHOBIUS); NON_CYTOPLASMIC_DOMAIN (PHOBIUS); CYTOPLASMIC_DOMAIN (PHOBIUS); TRANSMEMBRANE (PHOBIUS); TMhelix (TMHMM); TMhelix (TMHMM)
1213	277066_length_667_cvg_26.0_tip_1_1	81	46 kda fk506-binding nuclear protein	222	1.21E-48	72.80%	0.106		G3DSA:3.10.50.40 (GENE3D); IPR001179 (PFAM); IPR023566 (PANTHER); IPR001179 (PROSITE_PROFILES); SSF54534 (SUPERFAMILY)
1216	277102_length_668_cvg_8.0_tip_1_5	72	hemocyte protein-glutamine gamma-glutamyltransferase-like	222	6.63E-46	70.20%	0.11		IPR008958 (PFAM); IPR013783 (G3DSA:2.60.40.GENE3D); IPR013783 (G3DSA:2.60.40.GENE3D); PTHR11590:SF40 (PANTHER); IPR023608 (PANTHER); IPR008958 (SUPERFAMILY); IPR008958 (SUPERFAMILY)
1212	277046_length_667_cvg_5.0_tip_1_0	40	uv radiation resistance-associated gene protein	223	5.43E-21	62.00%	0.123		Coil (COILS); PTHR15157 (PANTHER)
1214	277086_length_668_cvg_51.6_tip_1_1	527	epididymal secretory protein e1	223	8.15E-31	57.80%	0.423	Y	IPR003172 (SMART); IPR003172 (PFAM); IPR003172 (G3DSA:2.60.40.GENE3D); PTHR11306 (PANTHER); TRANSMEMBRANE (PHOBIUS); NON_CYTOPLASMIC_DOMAIN (PHOBIUS); CYTOPLASMIC_DOMAIN (PHOBIUS); IPR014756 (SUPERFAMILY); TMhelix (TMHMM)
1215	277092_length_668_cvg_5.0_tip_1_0	41	beta- -galactosyltransferase 7	223	1.48E-102	81.60%	0.171		IPR003859 (PRINTS); IPR027995 (PFAM); IPR029044 (G3DSA:3.90.550.GENE3D); IPR027791 (PFAM); IPR003859 (PANTHER); PTHR19300:SF1 (PANTHER); IPR029044 (SUPERFAMILY)
1217	277232_length_670_cvg_2.0_tip_1_2	22	irregular chiasm c-roughest protein isoform x1	223	2.14E-92	76.00%	0.099		IPR003598 (SMART); IPR013162 (PFAM); IPR013783 (G3DSA:2.60.40.GENE3D); PF13895 (PFAM); IPR013783 (G3DSA:2.60.40.GENE3D); IPR013783 (G3DSA:2.60.40.GENE3D); PTHR11640:SF54 (PANTHER); PTHR11640 (PANTHER); IPR007110 (PROSITE_PROFILES); IPR007110 (PROSITE_PROFILES); SSF48726 (SUPERFAMILY); SSF48726 (SUPERFAMILY); SSF48726 (SUPERFAMILY)
1218	277238_length_670_cvg_16.1_tip_1_5	152	thermolysin catalytic domain partial	223	1.66E-12	48.10%	0.367	Y	IPR025711 (PFAM); IPR011096 (PFAM); NON_CYTOPLASMIC_DOMAIN (PHOBIUS); CYTOPLASMIC_DOMAIN (PHOBIUS); TRANSMEMBRANE (PHOBIUS)
1219	277258_length_670_cvg_4.1_tip_1_1	42	n-acetyllactosaminide 3-alpha-galactosyltransferase	223	1.49E-21	63.90%	0.441	Y	IPR029044 (G3DSA:3.90.550.GENE3D); IPR027995 (PFAM); IPR003859 (PANTHER); IPR029044 (SUPERFAMILY)

1220	277324_length_671_cvg_2.1_tip_1_0	17	von willebrand factor type egf and pentraxin domain-containing protein partial	224	6.63E-53	53.40%	0.116	IPR000436 (SMART); IPR000436 (PFAM); G3DSA:2.10.70.10 (GENE3D); G3DSA:2.10.70.10 (GENE3D); PTHR19325 (PANTHER); IPR000436 (PROSITE_PROFILES); IPR000436 (PROSITE_PROFILES); IPR000436 (PROSITE_PROFILES); IPR000436 (PROSITE_PROFILES); IPR000436 (SUPERFAMILY); IPR000436 (SUPERFAMILY); IPR000436 (SUPERFAMILY); IPR000436 (SUPERFAMILY)
1221	277442_length_674_cvg_3.0_tip_1_3	27	abnormal spindle-like microcephaly-associated protein homolog	225	3.54E-67	65.10%	0.112	IPR003599 (SMART); IPR013783 (G3DSA:2.60.40.GENE3D); IPR003961 (PFAM); IPR013783 (G3DSA:2.60.40.GENE3D); IPR013783 (G3DSA:2.60.40.GENE3D); IPR013098 (PFAM); PTHR19900 (PANTHER); PTHR19900:SF44 (PANTHER); IPR007110 (PROSITE_PROFILES); IPR003961 (PROSITE_PROFILES); SSF48726 (SUPERFAMILY); IPR003961 (SUPERFAMILY); SSF48726 (SUPERFAMILY)
1222	277456_length_674_cvg_2.2_tip_1_4	23	fizzy-related protein homolog	225	2.58E-142	95.40%	0.1	IPR001680 (SMART); IPR015943 (G3DSA:2.130.10.GENE3D); IPR001680 (PFAM); PTHR19918:SF6 (PANTHER); PTHR19918 (PANTHER); IPR019775 (PROSITE_PATTERNS); IPR019775 (PROSITE_PATTERNS); IPR019775 (PROSITE_PATTERNS); IPR017986 (PROSITE_PROFILES); IPR001680 (PROSITE_PROFILES); IPR001680 (PROSITE_PROFILES); IPR011047 (SUPERFAMILY)
1223	277470_length_674_cvg_62.7_tip_1_3	19099	nidogen-2 isoform x5	225	6.59E-23	54.70%	0.653 Y	IPR000716 (SMART); IPR000716 (G3DSA:4.10.800.GENE3D); IPR000716 (PFAM); IPR022339 (PANTHER); IPR000716 (PROSITE_PATTERNS); IPR000716 (PROSITE_PATTERNS); SIGNAL_PEPTIDE_C_REGION (PHOBIUS); NON_CYTOPLASMIC_DOMAIN (PHOBIUS); SIGNAL_PEPTIDE_H_REGION (PHOBIUS); SIGNAL_PEPTIDE_N_REGION (PHOBIUS); SIGNAL_PEPTIDE (PHOBIUS); IPR000716 (PROSITE_PROFILES); IPR000716 (PROSITE_PROFILES); SignalP-noTM (SIGNALP_GRAM_NEGATIVE); SignalP-noTM (SIGNALP_EUK); IPR000716 (SUPERFAMILY); IPR000716 (SUPERFAMILY)
1224	277728_length_677_cvg_5.0_tip_1_2	40	ecto-nox disulfide-thiol exchanger 2	225	5.74E-39	69.60%	0.103	PTHR16001 (PANTHER)
1225	277738_length_678_cvg_5.7_tip_1_2	51	all-trans-retinol -reductase	226	1.15E-76	70.70%	0.311	PF13450 (PFAM); IPR016040 (G3DSA:3.40.50.GENE3D); PTHR10668:SF61 (PANTHER); PTHR10668 (PANTHER); NON_CYTOPLASMIC_DOMAIN (PHOBIUS); SIGNAL_PEPTIDE_C_REGION (PHOBIUS); SIGNAL_PEPTIDE (PHOBIUS); SIGNAL_PEPTIDE_N_REGION (PHOBIUS); SIGNAL_PEPTIDE_H_REGION (PHOBIUS); SignalP-TM (SIGNALP_GRAM_POSITIVE); SSF51905 (SUPERFAMILY); TMhelix (TMHMM)
1226	277760_length_678_cvg_40.4_tip_1_1	601	mucin-5ac- partial	226	1.67E-10	49.40%	0.28	IPR025155 (PFAM); CYTOPLASMIC_DOMAIN (PHOBIUS); NON_CYTOPLASMIC_DOMAIN (PHOBIUS); TRANSMEMBRANE (PHOBIUS); TMhelix (TMHMM)
1227	277768_length_678_cvg_63.4_tip_1_4	781	thioredoxin peroxidase	226	4.64E-112	90.60%	0.116	IPR012336 (G3DSA:3.40.30.GENE3D); IPR000866 (PFAM); IPR019479 (PFAM); PTHR10681 (PANTHER); PTHR10681:SF101 (PANTHER); IPR012336 (PROSITE_PROFILES); IPR012336 (SUPERFAMILY)
1229	277944_length_681_cvg_61.3_tip_1_2	2140	hypothetical protein DAPPUDRAFT_312473	227	1.89E-07	65.00%	0.101	IPR014044 (G3DSA:3.40.33.GENE3D); IPR001283 (PANTHER); PTHR10334:SF153 (PANTHER); CYTOPLASMIC_DOMAIN (PHOBIUS); NON_CYTOPLASMIC_DOMAIN (PHOBIUS); TRANSMEMBRANE (PHOBIUS); NON_CYTOPLASMIC_DOMAIN (PHOBIUS); TRANSMEMBRANE (PHOBIUS); TRANSMEMBRANE (PHOBIUS); CYTOPLASMIC_DOMAIN (PHOBIUS); IPR014044 (SUPERFAMILY); TMhelix (TMHMM); TMhelix (TMHMM)
1230	277960_length_681_cvg_3.1_tip_1_4	25	probable serine carboxypeptidase cpvl	227	2.60E-50	59.00%	0.116	IPR001563 (PRINTS); IPR029058 (G3DSA:3.40.50.GENE3D); IPR001563 (PFAM); IPR001563 (PANTHER); PTHR11802:SF30 (PANTHER); IPR018202 (PROSITE_PATTERNS); IPR029058 (SUPERFAMILY)
1231	277968_length_681_cvg_8.9_tip_1_2	64	cytochrome p450 partial	227	1.34E-66	67.10%	0.146	IPR001128 (PRINTS); IPR002401 (PRINTS); IPR001128 (G3DSA:1.10.630.GENE3D); IPR001128 (PFAM); PTHR24305:SF49 (PANTHER); PTHR24305 (PANTHER); IPR017972 (PROSITE_PATTERNS); CYTOPLASMIC_DOMAIN (PHOBIUS); NON_CYTOPLASMIC_DOMAIN (PHOBIUS); TRANSMEMBRANE (PHOBIUS); IPR001128 (SUPERFAMILY)
1232	277970_length_681_cvg_2.9_tip_1_5	27	cub and sushi domain-containing protein 3-like	227	9.30E-09	47.20%	0.278	IPR000859 (SMART); IPR000859 (G3DSA:2.60.120.GENE3D); IPR000859 (PFAM); PTHR22991:SF23 (PANTHER); PTHR22991 (PANTHER); IPR000859 (PROSITE_PROFILES); IPR000859 (SUPERFAMILY)
1233	277972_length_681_cvg_3.0_tip_1_1	28	ectonucleotide pyrophosphatase phosphodiesterase family member 7-like	227	1.77E-62	57.50%	0.098	IPR002591 (PFAM); IPR017849 (G3DSA:3.40.720.GENE3D); IPR024873 (PANTHER); IPR017850 (SUPERFAMILY)
1234	277984_length_681_cvg_3.4_tip_1_1	29	rna polymerase ii-associated protein 3	227	3.45E-28	52.90%	0.124	Coil (COILS); IPR019734 (SMART); PF13414 (PFAM); IPR011990 (G3DSA:1.25.40.GENE3D); PTHR22904 (PANTHER); PTHR22904:SF292 (PANTHER); IPR013026 (PROSITE_PROFILES); IPR019734 (PROSITE_PROFILES); IPR019734 (PROSITE_PROFILES); SSF48452 (SUPERFAMILY)

1235	278004_length_681_cvg_5.0_tip_1_4	45	low-density lipoprotein receptor-related protein 2	227	2.23E-38	57.20%	0.101	IPR002172 (PRINTS); IPR002172 (SMART); IPR002172 (PFAM); G3DSA:4.10.1220.10 (GENE3D); IPR002172 (G3DSA:4.10.400.GENE3D); IPR002172 (G3DSA:4.10.400.GENE3D); PTHR10529 (PANTHER); IPR023415 (PROSITE_PATTERNS); IPR023415 (PROSITE_PATTERNS); IPR023415 (PROSITE_PATTERNS); IPR023415 (PROSITE_PATTERNS); IPR002172 (PROSITE_PROFILES); IPR002172 (PROSITE_PROFILES); IPR002172 (PROSITE_PROFILES); IPR002172 (PROSITE_PROFILES); IPR002172 (SUPERFAMILY); IPR002172 (SUPERFAMILY); IPR002172 (SUPERFAMILY); IPR002172 (SUPERFAMILY)
1236	278014_length_681_cvg_5.0_tip_1_0	41	phenoloxidase subunit a3-like	227	3.51E-47	62.50%	0.098	IPR005203 (PFAM); IPR005203 (G3DSA:2.60.40.GENE3D); IPR013788 (PANTHER); PTHR11511:SF24 (PANTHER); IPR014756 (SUPERFAMILY)
1237	278134_length_683_cvg_5.9_tip_1_1	40	camp-dependent protein kinase catalytic	228	3.25E-124	86.40%	0.106	IPR000961 (SMART); IPR002290 (SMART); G3DSA:1.10.510.10 (GENE3D); IPR017892 (PFAM); IPR000719 (PFAM); PTHR24357:SF70 (PANTHER); PTHR24357 (PANTHER); IPR000961 (PROSITE_PROFILES); IPR000719 (PROSITE_PROFILES); IPR011009 (SUPERFAMILY)
1238	278136_length_683_cvg_3.8_tip_1_3	29	low quality protein: abc transporter f family member 4-like	228	2.42E-08	55.00%	0.112	Coil (COILS); Coil (COILS)
1239	278138_length_683_cvg_45.1_tip_1_1	471	macrophage migration inhibitory factor	228	2.90E-39	69.80%	0.114	G3DSA:3.30.429.10 (GENE3D); IPR001398 (PFAM); PTHR11954:SF6 (PANTHER); IPR001398 (PANTHER); IPR001398 (PRODOM); IPR014347 (SUPERFAMILY)
1240	278320_length_686_cvg_4.5_tip_1_5	36	serine protease mitochondrial	228	8.85E-98	78.00%	0.11	IPR001940 (PRINTS); G3DSA:2.40.10.10 (GENE3D); PF13365 (PFAM); G3DSA:2.40.10.10 (GENE3D); PTHR22939 (PANTHER); IPR009003 (SUPERFAMILY)
1241	278328_length_686_cvg_21.9_tip_1_0	169	guanine nucleotide-binding protein g g g subunit beta-1	229	3.32E-122	97.80%	0.125	Coil (COILS); IPR001632 (PRINTS); IPR001680 (SMART); IPR001680 (PFAM); IPR015943 (G3DSA:2.130.10.GENE3D); IPR016346 (PANTHER); IPR019775 (PROSITE_PATTERNS); IPR019775 (PROSITE_PATTERNS); IPR001680 (PROSITE_PROFILES); IPR017986 (PROSITE_PROFILES); IPR001680 (PROSITE_PROFILES); IPR017986 (SUPERFAMILY)
1242	278344_length_686_cvg_3.0_tip_1_3	26	protein gdap2 homolog	229	2.99E-83	73.20%	0.106	IPR002589 (SMART); G3DSA:3.40.220.10 (GENE3D); IPR002589 (PFAM); PTHR11106 (PANTHER); PTHR11106:SF59 (PANTHER); IPR002589 (PROSITE_PROFILES); SSF52949 (SUPERFAMILY)
1243	278366_length_687_cvg_6.4_tip_1_0	51	keratin-associated protein 5-9-like	229	2.20E-12	67.30%	0.095	no IPS match
1244	278438_length_688_cvg_6.7_tip_1_4	52	otopetrin-2 isoform x1	229	1.29E-84	77.70%	0.142	IPR004878 (PFAM); IPR004878 (PANTHER); PTHR21522:SF37 (PANTHER); CYTOPLASMIC_DOMAIN (PHOBIUS); CYTOPLASMIC_DOMAIN (PHOBIUS); TRANSMEMBRANE (PHOBIUS); TRANSMEMBRANE (PHOBIUS); NON_CYTOPLASMIC_DOMAIN (PHOBIUS); TRANSMEMBRANE (PHOBIUS); CYTOPLASMIC_DOMAIN (PHOBIUS); TRANSMEMBRANE (PHOBIUS); NON_CYTOPLASMIC_DOMAIN (PHOBIUS); TMhelix (TMHMM); TMhelix (TMHMM)
1245	278582_length_690_cvg_3.0_tip_1_1	28	chondroitin sulfate	230	3.87E-72	69.60%	0.101	IPR008428 (PFAM); PTHR12369:SF11 (PANTHER); PTHR12369 (PANTHER)
1246	278628_length_690_cvg_79.7_tip_1_0	4134	dermatopontin 2	230	3.28E-38	58.90%	0.25	PF14704 (PFAM); IPR026645 (PANTHER); NON_CYTOPLASMIC_DOMAIN (PHOBIUS); SIGNAL_PEPTIDE_C_REGION (PHOBIUS); SIGNAL_PEPTIDE_N_REGION (PHOBIUS); SIGNAL_PEPTIDE_H_REGION (PHOBIUS); SIGNAL_PEPTIDE (PHOBIUS); TMhelix (TMHMM)
1247	278632_length_690_cvg_21.0_tip_1_5	182	diuretic hormone class 2-like	230	1.39E-29	73.30%	0.15	NON_CYTOPLASMIC_DOMAIN (PHOBIUS); TRANSMEMBRANE (PHOBIUS); CYTOPLASMIC_DOMAIN (PHOBIUS); TMhelix (TMHMM)
1248	278780_length_693_cvg_2.9_tip_1_3	28	very low-density lipoprotein receptor-like isoform x1	231	2.34E-19	43.60%	0.101	IPR002172 (PRINTS); IPR002172 (SMART); IPR002172 (PFAM); IPR002172 (G3DSA:4.10.400.GENE3D); IPR002172 (G3DSA:4.10.400.GENE3D); IPR002172 (G3DSA:4.10.400.GENE3D); PTHR10529 (PANTHER); IPR002172 (PROSITE_PROFILES); IPR002172 (PROSITE_PROFILES); IPR002172 (PROSITE_PROFILES); IPR002172 (SUPERFAMILY); IPR002172 (SUPERFAMILY); IPR002172 (SUPERFAMILY)
1249	278786_length_693_cvg_39.0_tip_1_3	311	cytosolic non-specific dipeptidase	231	1.40E-109	84.00%	0.133	G3DSA:3.40.630.10 (GENE3D); IPR002933 (PFAM); PTHR11014:SF51 (PANTHER); PTHR11014 (PANTHER); IPR001261 (PROSITE_PATTERNS); SSF53187 (SUPERFAMILY)
1250	278906_length_694_cvg_5.9_tip_1_0	58	hemocyte protein-glutamine gamma-glutamyltransferase-like	232	6.44E-100	78.90%	0.146	IPR013783 (G3DSA:2.60.40.GENE3D); IPR002931 (G3DSA:3.90.260.GENE3D); IPR008958 (PFAM); IPR023608 (PANTHER); PTHR11590:SF40 (PANTHER); SSF54001 (SUPERFAMILY); IPR008958 (SUPERFAMILY)
1251	279026_length_697_cvg_4.0_tip_1_5	36	protein phosphatase 1 regulatory subunit 27	232	2.72E-61	70.90%	0.133	IPR002110 (SMART); IPR020683 (G3DSA:1.25.40.GENE3D); IPR020683 (PFAM); PTHR24179 (PANTHER); PTHR24179:SF23 (PANTHER); IPR002110 (PROSITE_PROFILES); IPR002110 (PROSITE_PROFILES); IPR020683 (PROSITE_PROFILES); IPR020683 (SUPERFAMILY)

1252	279050_length_697_cvg_4.0_tip_1_1	38	mitogen-activated protein kinase kinase kinase 4	232	9.90E-99	79.40%	0.11	IPR002290 (SMART); IPR000719 (PFAM); IPR016253 (PIRSF); G3DSA:1.10.510.10 (GENE3D); G3DSA:3.30.200.20 (GENE3D); PTHR24360:SF47 (PANTHER); PTHR24360 (PANTHER); IPR008271 (PROSITE_PATTERNS); IPR000719 (PROSITE_PROFILES); IPR011009 (SUPERFAMILY)
1253	279084_length_697_cvg_3.8_tip_1_3	32	fk506-binding protein 2	233	2.19E-67	84.70%	0.28	IPR001179 (PFAM); G3DSA:3.10.50.40 (GENE3D); IPR023566 (PANTHER); PTHR10516:SF134 (PANTHER); CYTOPLASMIC_DOMAIN (PHOBIUS); TRANSMEMBRANE (PHOBIUS); NON_CYTOPLASMIC_DOMAIN (PHOBIUS); IPR001179 (PROSITE_PROFILES); SSF54534 (SUPERFAMILY); TMhelix (TMHMM)
1254	279092_length_698_cvg_3.4_tip_1_4	31	serine threonine-protein phosphatase 6 regulatory ankyrin repeat subunit c-like	233	1.81E-46	55.60%	0.112	IPR002110 (PRINTS); IPR002110 (SMART); IPR020683 (G3DSA:1.25.40.GENE3D); IPR020683 (G3DSA:1.25.40.GENE3D); IPR002110 (PFAM); IPR020683 (G3DSA:1.25.40.GENE3D); IPR020683 (PFAM); PTHR24198 (PANTHER); IPR020683 (PROSITE_PROFILES); IPR002110 (PROSITE_PROFILES); IPR002110 (PROSITE_PROFILES); IPR002110 (PROSITE_PROFILES); IPR002110 (PROSITE_PROFILES); IPR002110 (PROSITE_PROFILES); IPR020683 (SUPERFAMILY)
1255	279194_length_699_cvg_6.0_tip_1_3	49	succinate-semialdehyde mitochondrial	233	6.56E-105	79.10%	0.103	IPR015590 (PFAM); IPR016162 (G3DSA:3.40.605.GENE3D); IPR016163 (G3DSA:3.40.309.GENE3D); PTHR11699:SF49 (PANTHER); PTHR11699 (PANTHER); IPR029510 (PROSITE_PATTERNS); IPR016161 (SUPERFAMILY)
1257	279274_length_700_cvg_18.9_tip_1_1	106	protein	233	4.08E-29	83.10%	0.146	TRANSMEMBRANE (PHOBIUS); CYTOPLASMIC_DOMAIN (PHOBIUS); NON_CYTOPLASMIC_DOMAIN (PHOBIUS)
1256	279266_length_700_cvg_82.0_tip_1_3	1240	nidogen-related basement membrane protein	234	5.72E-07	52.00%	0.103	IPR000716 (SMART); IPR000716 (G3DSA:4.10.800.GENE3D); IPR000716 (PFAM); IPR000716 (PROSITE_PATTERNS); IPR000716 (PROSITE_PROFILES); IPR000716 (SUPERFAMILY)
1258	279308_length_701_cvg_24.9_tip_1_0	171	protein spaetzle	234	8.59E-17	51.50%	0.099	CYTOPLASMIC_DOMAIN (PHOBIUS); NON_CYTOPLASMIC_DOMAIN (PHOBIUS); TRANSMEMBRANE (PHOBIUS); IPR029034 (SUPERFAMILY); TMhelix (TMHMM)
1259	279366_length_702_cvg_3.0_tip_1_5	20	kelch-like protein diablo	234	3.29E-136	95.00%	0.108	IPR011705 (SMART); IPR000210 (SMART); G3DSA:3.30.710.10 (GENE3D); IPR011705 (PFAM); IPR013069 (PFAM); PTHR24412 (PANTHER); IPR000210 (PROSITE_PROFILES); IPR011333 (SUPERFAMILY)
1260	279376_length_703_cvg_3.2_tip_1_1	33	follistatin-related protein 5- partial	234	2.36E-136	88.70%	0.098	PTHR10913 (PANTHER); PTHR10913:SF14 (PANTHER); SSF63825 (SUPERFAMILY)
1262	279402_length_703_cvg_5.6_tip_1_4	61	carcinolectin 5b partial	234	2.15E-43	65.20%	0.109	IPR002181 (SMART); IPR002181 (PFAM); IPR014715 (G3DSA:4.10.530.GENE3D); IPR014716 (G3DSA:3.90.215.GENE3D); PTHR19143 (PANTHER); IPR020837 (PROSITE_PATTERNS); IPR002181 (PROSITE_PROFILES); IPR002181 (SUPERFAMILY)
1261	279380_length_703_cvg_2.0_tip_1_3	18	zinc finger x-linked protein zxdb	235	6.17E-90	72.80%	0.101	IPR015880 (SMART); IPR007087 (PFAM); IPR013087 (G3DSA:3.30.160.GENE3D); IPR013087 (G3DSA:3.30.160.GENE3D); IPR013087 (G3DSA:3.30.160.GENE3D); PTHR19818 (PANTHER); PTHR19818:SF68 (PANTHER); IPR007087 (PROSITE_PATTERNS); IPR007087 (PROSITE_PATTERNS); IPR007087 (PROSITE_PATTERNS); IPR007087 (PROSITE_PATTERNS); IPR007087 (PROSITE_PATTERNS); IPR007087 (PROSITE_PATTERNS); IPR007087 (PROSITE_PROFILES); IPR007087 (PROSITE_PROFILES); IPR007087 (PROSITE_PROFILES); IPR007087 (PROSITE_PROFILES); IPR007087 (PROSITE_PROFILES); IPR007087 (PROSITE_PROFILES); IPR007087 (PROSITE_PROFILES); SSF57667 (SUPERFAMILY); SSF57667 (SUPERFAMILY); SSF57667 (SUPERFAMILY)
1264	279606_length_706_cvg_11.6_tip_1_5	88	membrane glycoprotein lig-	235	1.72E-16	47.20%	0.1	G3DSA:3.80.10.10 (GENE3D); PTHR24373 (PANTHER); IPR001611 (PROSITE_PROFILES); IPR001611 (PROSITE_PROFILES); SSF52058 (SUPERFAMILY)
1265	279630_length_706_cvg_3.0_tip_1_4	27	carboxypeptidase e-like	235	2.29E-118	81.50%	0.133	IPR000834 (SMART); G3DSA:3.40.630.10 (GENE3D); IPR000834 (PFAM); PTHR11532:SF41 (PANTHER); PTHR11532 (PANTHER); IPR000834 (PROSITE_PATTERNS); SSF53187 (SUPERFAMILY)
1263	279578_length_706_cvg_6.2_tip_1_0	45	protein bowel	236	1.90E-105	82.30%	0.115	IPR015880 (SMART); PF13894 (PFAM); IPR013087 (G3DSA:3.30.160.GENE3D); IPR007087 (PFAM); IPR013087 (G3DSA:3.30.160.GENE3D); IPR013087 (G3DSA:3.30.160.GENE3D); PF13465 (PFAM); PTHR14196:SF0 (PANTHER); PTHR14196 (PANTHER); IPR007087 (PROSITE_PATTERNS); IPR007087 (PROSITE_PATTERNS); IPR007087 (PROSITE_PATTERNS); IPR007087 (PROSITE_PATTERNS); IPR007087 (PROSITE_PATTERNS); IPR007087 (PROSITE_PATTERNS); IPR007087 (PROSITE_PROFILES); IPR007087 (PROSITE_PROFILES); IPR007087 (PROSITE_PROFILES); IPR007087 (PROSITE_PROFILES); IPR007087 (PROSITE_PROFILES); IPR007087 (PROSITE_PROFILES); IPR007087 (PROSITE_PROFILES); SSF57667 (SUPERFAMILY); SSF57667 (SUPERFAMILY); SSF57667 (SUPERFAMILY)

1266	279660_length_707_cvg_3.0_tip_1_1	26	zinc finger protein 26-like	236	3.25E-39	55.70%	0.1		IPR015880 (SMART); IPR013087 (G3DSA:3.30.160.GENE3D); IPR013087 (G3DSA:3.30.160.GENE3D); PF13894 (PFAM); IPR007087 (PFAM); PF13465 (PFAM); IPR013087 (G3DSA:3.30.160.GENE3D); PTHR24409 (PANTHER); IPR007087 (PROSITE_PATTERNS); IPR007087 (PROSITE_PATTERNS); IPR007087 (PROSITE_PATTERNS); IPR007087 (PROSITE_PATTERNS); IPR007087 (PROSITE_PATTERNS); IPR007087 (PROSITE_PROFILES); IPR007087 (PROSITE_PROFILES); IPR007087 (PROSITE_PROFILES); IPR007087 (PROSITE_PROFILES); IPR007087 (PROSITE_PROFILES); SSF57667 (SUPERFAMILY); SSF57667 (SUPERFAMILY); SSF57667 (SUPERFAMILY)
1267	279692_length_707_cvg_36.0_tip_1_4	297	26s proteasome non-atpase regulatory subunit 10	236	2.84E-81	71.60%	0.142		IPR002110 (PRINTS); IPR002110 (SMART); IPR020683 (G3DSA:1.25.40.GENE3D); IPR020683 (PFAM); IPR020683 (G3DSA:1.25.40.GENE3D); PTHR24199 (PANTHER); IPR002110 (PROSITE_PROFILES); IPR002110 (PROSITE_PROFILES); IPR002110 (PROSITE_PROFILES); IPR002110 (PROSITE_PROFILES); IPR020683 (PROSITE_PROFILES); IPR002110 (PROSITE_PROFILES); IPR020683 (SUPERFAMILY)
1268	279732_length_708_cvg_10.8_tip_1_2	68	uncharacterized oxidoreductase	236	1.29E-62	69.10%	0.099		IPR002198 (PRINTS); IPR002347 (PRINTS); IPR002198 (PFAM); IPR016040 (G3DSA:3.40.50.GENE3D); PTHR24322:SF237 (PANTHER); PTHR24322 (PANTHER); SSF51735 (SUPERFAMILY)
1269	279790_length_709_cvg_8.7_tip_1_2	70	biotinidase	236	4.20E-29	54.40%	0.214		IPR003010 (G3DSA:3.60.110.GENE3D); IPR003010 (PFAM); IPR012101 (PANTHER); PTHR10609:SF14 (PANTHER); IPR003010 (PROSITE_PROFILES); IPR003010 (SUPERFAMILY)
1270	279854_length_710_cvg_27.8_tip_1_0	182	induced during granule regeneration 1	237	1.10E-14	50.14%	0.103		no IPS match
1271	279856_length_710_cvg_62.8_tip_1_4	1397	pacifastin light chain	237	6.30E-17	50.40%	0.846	Y	IPR008037 (PFAM); SIGNAL_PEPTIDE_C_REGION (PHOBIUS); SIGNAL_PEPTIDE_N_REGION (PHOBIUS); NON_CYTOPLASMIC_DOMAIN (PHOBIUS); SIGNAL_PEPTIDE_H_REGION (PHOBIUS); SIGNAL_PEPTIDE (PHOBIUS); IPR020862 (PROSITE_PROFILES); IPR020862 (PROSITE_PROFILES); IPR020862 (PROSITE_PROFILES); SignalP-TM (SIGNALP_GRAM_POSITIVE); SignalP-noTM (SIGNALP_EUK); IPR008037 (SUPERFAMILY); IPR008037 (SUPERFAMILY); IPR008037 (SUPERFAMILY); TMhelix (TMHMM)
1272	279926_length_711_cvg_66.3_tip_1_3	732	hemagglutinin amebocyte aggregation factor-like	237	1.50E-40	62.60%	0.677	Y	PF14704 (PFAM); IPR026645 (PANTHER); SIGNAL_PEPTIDE_H_REGION (PHOBIUS); NON_CYTOPLASMIC_DOMAIN (PHOBIUS); SIGNAL_PEPTIDE_C_REGION (PHOBIUS); CYTOPLASMIC_DOMAIN (PHOBIUS); SIGNAL_PEPTIDE_N_REGION (PHOBIUS); SIGNAL_PEPTIDE (PHOBIUS); TRANSMEMBRANE (PHOBIUS); SignalP-noTM (SIGNALP_EUK)
1274	280040_length_713_cvg_9.0_tip_1_5	76	peptidoglycan recognition protein 3 short class	237	6.63E-59	64.70%	0.878	Y	IPR002502 (SMART); IPR006619 (SMART); IPR002502 (G3DSA:3.40.80.GENE3D); IPR002502 (PFAM); IPR015510 (PANTHER); SIGNAL_PEPTIDE_N_REGION (PHOBIUS); SIGNAL_PEPTIDE (PHOBIUS); NON_CYTOPLASMIC_DOMAIN (PHOBIUS); SIGNAL_PEPTIDE_H_REGION (PHOBIUS); SIGNAL_PEPTIDE_C_REGION (PHOBIUS); SignalP-noTM (SIGNALP_EUK); IPR002502 (SUPERFAMILY); TMhelix (TMHMM)
1273	280038_length_713_cvg_4.0_tip_1_3	49	vitellogenin- partial	238	2.77E-14	44.70%	0.109		PTHR23345 (PANTHER); PTHR23345:SF1 (PANTHER); IPR001846 (PROSITE_PROFILES)
1275	280314_length_717_cvg_3.0_tip_1_4	39	plexin domain-containing protein 2-like	239	1.08E-37	46.20%	0.183		IPR016201 (SMART); G3DSA:3.30.1680.10 (GENE3D); PTHR13055 (PANTHER); PTHR13055:SF12 (PANTHER); CYTOPLASMIC_DOMAIN (PHOBIUS); TRANSMEMBRANE (PHOBIUS); NON_CYTOPLASMIC_DOMAIN (PHOBIUS); IPR016201 (SUPERFAMILY); TMhelix (TMHMM)
1276	280328_length_718_cvg_10.9_tip_0_5	68	marginal zone b- and b1-cell-specific protein	239	1.76E-43	59.10%	0.105		PTHR15881 (PANTHER); PTHR15881:SF1 (PANTHER)
1277	280358_length_718_cvg_3.1_tip_1_4	27	prenylcysteine oxidase-like	239	6.22E-52	61.70%	0.128		IPR010795 (PFAM); IPR017046 (PANTHER)
1278	280388_length_719_cvg_7.0_tip_1_3	61	receptor-type tyrosine-protein phosphatase n2	240	9.18E-157	92.50%	0.11		IPR000242 (PRINTS); IPR003595 (SMART); IPR000242 (SMART); IPR029021 (G3DSA:3.90.190.GENE3D); IPR000242 (PFAM); PTHR19134 (PANTHER); PTHR19134:SF2 (PANTHER); IPR016130 (PROSITE_PATTERNS); IPR000242 (PROSITE_PROFILES); IPR000387 (PROSITE_PROFILES); IPR029021 (SUPERFAMILY)
1279	280396_length_719_cvg_11.8_tip_1_1	85	protein them6	240	7.53E-25	55.30%	0.137		PF13279 (PFAM); IPR029069 (G3DSA:3.10.129.GENE3D); PTHR12475 (PANTHER); IPR029069 (SUPERFAMILY)
1280	280500_length_720_cvg_4.0_tip_1_3	33	protein tag-53	240	6.40E-66	58.50%	0.113		IPR016201 (SMART); IPR015915 (G3DSA:2.120.10.GENE3D); IPR002165 (PFAM); PF13854 (PFAM); PTHR10574 (PANTHER); PTHR10574:SF3 (PANTHER); SSF117281 (SUPERFAMILY)
1281	280550_length_721_cvg_3.0_tip_1_5	34	probable gpi-anchored adhesin-like protein pga55	240	3.07E-57	75.00%	0.144		IPR001478 (SMART); IPR001478 (PFAM); IPR001478 (G3DSA:2.30.42.GENE3D); PTHR11324 (PANTHER); IPR001478 (PROSITE_PROFILES); IPR001478 (SUPERFAMILY)

1282	280568_length_722_cvg_8.6_tip_1_1	84	receptor-type tyrosine-protein phosphatase f-like isoform x1	241	5.97E-48	70.70%	0.138	IPR000242 (PRINTS); IPR000242 (SMART); IPR029021 (G3DSA:3.90.190.GENE3D); IPR000242 (PFAM); PTHR19134 (PANTHER); CYTOPLASMIC_DOMAIN (PHOBIUS); NON_CYTOPLASMIC_DOMAIN (PHOBIUS); TRANSMEMBRANE (PHOBIUS); IPR000242 (PROSITE_PROFILES); IPR029021 (SUPERFAMILY); TMhelix (TMHMM)
1283	280648_length_723_cvg_3.2_tip_1_4	34	glucose dehydrogenase	241	4.63E-54	65.10%	0.161	IPR007867 (PFAM); IPR000172 (PFAM); G3DSA:3.30.560.10 (GENE3D); G3DSA:3.50.50.60 (GENE3D); PTHR11552 (PANTHER); PTHR11552:SF77 (PANTHER); SSF54373 (SUPERFAMILY); SSF51905 (SUPERFAMILY)
1284	280670_length_723_cvg_4.2_tip_1_3	35	interleukin-1 receptor-associated kinase 4	241	1.19E-86	69.50%	0.106	IPR002290 (SMART); G3DSA:3.30.200.20 (GENE3D); G3DSA:1.10.510.10 (GENE3D); IPR000719 (PFAM); PTHR24420 (PANTHER); IPR017441 (PROSITE_PATTERNS); IPR008271 (PROSITE_PATTERNS); IPR000719 (PROSITE_PROFILES); IPR011009 (SUPERFAMILY)
1285	280696_length_724_cvg_10.6_tip_1_4	87	coiled-coil domain-containing protein partial	241	3.12E-15	50.20%	0.113	SignalP-TM (SIGNALP_GRAM_POSITIVE)
1286	280706_length_724_cvg_6.0_tip_1_4	53	protein disulfide-isomerase a6	241	2.16E-83	74.30%	0.251	PR00421 (PRINTS); IPR012336 (G3DSA:3.40.30.GENE3D); IPR005788 (TIGRFAM); IPR013766 (PFAM); PTHR18929:SF38 (PANTHER); PTHR18929 (PANTHER); IPR017937 (PROSITE_PATTERNS); IPR017937 (PROSITE_PATTERNS); CYTOPLASMIC_DOMAIN (PHOBIUS); TRANSMEMBRANE (PHOBIUS); NON_CYTOPLASMIC_DOMAIN (PHOBIUS); IPR012336 (PROSITE_PROFILES); IPR012336 (SUPERFAMILY); IPR012336 (SUPERFAMILY)
1287	280712_length_724_cvg_4.3_tip_1_4	38	serine protease easter	241	2.71E-37	60.30%	0.103	IPR001314 (PRINTS); IPR001254 (SMART); IPR001254 (PFAM); G3DSA:2.40.10.10 (GENE3D); PTHR24268 (PANTHER); IPR018114 (PROSITE_PATTERNS); IPR001254 (PROSITE_PROFILES); IPR009003 (SUPERFAMILY)
1289	280726_length_724_cvg_4.0_tip_1_4	40	carboxypeptidase b	241	1.03E-97	68.20%	0.15	IPR000834 (SMART); IPR000834 (PFAM); G3DSA:3.40.630.10 (GENE3D); PTHR11705 (PANTHER); SSF53187 (SUPERFAMILY)
1290	280800_length_725_cvg_4.0_tip_1_2	35	tyrosine-protein kinase hopscotch	241	2.43E-79	67.20%	0.108	IPR001245 (PRINTS); IPR020635 (SMART); IPR016253 (PIRSF); IPR001245 (PFAM); G3DSA:3.30.200.20 (GENE3D); G3DSA:1.10.510.10 (GENE3D); PTHR24418 (PANTHER); IPR008266 (PROSITE_PATTERNS); IPR000719 (PROSITE_PROFILES); IPR011009 (SUPERFAMILY)
1288	280716_length_724_cvg_33.4_tip_1_0	265	adp-ribosylation factor 2	242	3.69E-105	95.80%	0.115	IPR006689 (PRINTS); IPR024156 (SMART); IPR006687 (SMART); IPR005225 (TIGRFAM); IPR006689 (PFAM); IPR027417 (G3DSA:3.40.50.GENE3D); PTHR11711:SF153 (PANTHER); PTHR11711 (PANTHER); IPR024156 (PROSITE_PROFILES); IPR027417 (SUPERFAMILY)
1291	280838_length_726_cvg_11.0_tip_1_5	80	hypoxia up-regulated protein 1 isoform x1	242	1.08E-95	80.30%	0.303	IPR013126 (PRINTS); G3DSA:3.30.30.30 (GENE3D); G3DSA:3.30.420.40 (GENE3D); IPR013126 (PFAM); PTHR19375 (PANTHER); PTHR19375:SF90 (PANTHER); SSF53067 (SUPERFAMILY)
1292	280880_length_727_cvg_59.2_tip_0_3	632	low quality protein: mucin-19	243	6.57E-16	43.20%	0.133	SignalP-TM (SIGNALP_GRAM_POSITIVE)
1293	280924_length_727_cvg_12.1_tip_1_0	128	membrane metallo-endopeptidase-like 1-like	243	2.35E-27	54.10%	0.151	IPR008753 (PFAM); G3DSA:1.10.1380.10 (GENE3D); IPR000718 (PANTHER); NON_CYTOPLASMIC_DOMAIN (PHOBIUS); CYTOPLASMIC_DOMAIN (PHOBIUS); TRANSMEMBRANE (PHOBIUS); SSF55486 (SUPERFAMILY); TMhelix (TMHMM)
1294	280974_length_728_cvg_7.2_tip_1_3	81	ankyrin repeat and mynd domain-containing protein 2	243	1.90E-91	71.60%	0.099	IPR002110 (SMART); IPR020683 (G3DSA:1.25.40.GENE3D); IPR020683 (PFAM); PTHR24150 (PANTHER); IPR002110 (PROSITE_PROFILES); IPR002110 (PROSITE_PROFILES); IPR020683 (PROSITE_PROFILES); IPR020683 (SUPERFAMILY)
1295	281142_length_731_cvg_57.0_tip_1_3	693	granulins- partial	244	4.22E-36	53.10%	0.552 Y	IPR000118 (SMART); IPR000118 (PFAM); PTHR12274 (PANTHER); PTHR12274:SF1 (PANTHER); IPR000118 (PROSITE_PATTERNS); IPR000118 (PROSITE_PATTERNS); TRANSMEMBRANE (PHOBIUS); NON_CYTOPLASMIC_DOMAIN (PHOBIUS); CYTOPLASMIC_DOMAIN (PHOBIUS); SignalP-noTM (SIGNALP_EUK); SSF57277 (SUPERFAMILY); SSF57277 (SUPERFAMILY); TMhelix (TMHMM)
1296	281144_length_731_cvg_31.0_tip_1_3	286	protein twin sister of ft	244	1.93E-24	59.50%	0.164	IPR008914 (PFAM); IPR008914 (G3DSA:3.90.280.GENE3D); PTHR11362 (PANTHER); IPR001858 (PROSITE_PATTERNS); NON_CYTOPLASMIC_DOMAIN (PHOBIUS); CYTOPLASMIC_DOMAIN (PHOBIUS); TRANSMEMBRANE (PHOBIUS); IPR008914 (SUPERFAMILY)
1297	281254_length_733_cvg_6.2_tip_1_1	59	fat-like cadherin-related tumor suppressor homolog isoform x3	244	6.25E-88	70.90%	0.101	IPR001881 (SMART); IPR000742 (SMART); G3DSA:2.10.25.10 (GENE3D); IPR001881 (PFAM); G3DSA:2.10.25.10 (GENE3D); PTHR24026 (PANTHER); PTHR24026:SF39 (PANTHER); IPR000152 (PROSITE_PATTERNS); IPR018097 (PROSITE_PATTERNS); IPR013032 (PROSITE_PATTERNS); NON_CYTOPLASMIC_DOMAIN (PHOBIUS); TRANSMEMBRANE (PHOBIUS); CYTOPLASMIC_DOMAIN (PHOBIUS); IPR000742 (PROSITE_PROFILES); IPR000742 (PROSITE_PROFILES); IPR000742 (PROSITE_PROFILES); SSF57196 (SUPERFAMILY); SSF57196 (SUPERFAMILY); TMhelix (TMHMM)

1298	281370_length_736_cvg_10.7_tip_1_2	97	aael007448- partial	245	1.77E-57	72.00%	0.105		IPR029058 (G3DSA:3.40.50.GENE3D); IPR001375 (PFAM); PTHR11731 (PANTHER); PTHR11731:SF103 (PANTHER); IPR029058 (SUPERFAMILY)
1299	281374_length_736_cvg_3.1_tip_1_2	38	cadherin-related family member partial	245	7.28E-18	50.70%	0.101		IPR002126 (PRINTS); IPR002126 (SMART); IPR002126 (PFAM); IPR002126 (G3DSA:2.60.40.GENE3D); IPR002126 (G3DSA:2.60.40.GENE3D); PTHR24027 (PANTHER); IPR002126 (PROSITE_PROFILES); IPR002126 (PROSITE_PROFILES); IPR015919 (SUPERFAMILY); IPR015919 (SUPERFAMILY)
1301	281410_length_736_cvg_2.8_tip_1_1	31	lysosomal alpha-glucosidase-like	245	8.69E-41	60.90%	0.118		PTHR22762 (PANTHER); PTHR22762:SF7 (PANTHER); TRANSMEMBRANE (PHOBIUS); TRANSMEMBRANE (PHOBIUS); CYTOPLASMIC_DOMAIN (PHOBIUS); NON_CYTOPLASMIC_DOMAIN (PHOBIUS); CYTOPLASMIC_DOMAIN (PHOBIUS); TMhelix (TMHMM); TMhelix (TMHMM)
1300	281388_length_736_cvg_4.4_tip_1_0	42	multiple pdz domain	246	1.35E-56	61.20%	0.148		IPR001478 (SMART); IPR001478 (PFAM); IPR001478 (G3DSA:2.30.42.GENE3D); IPR001478 (G3DSA:2.30.42.GENE3D); PTHR19964 (PANTHER); PTHR19964:SF34 (PANTHER); IPR001478 (PROSITE_PROFILES); IPR001478 (PROSITE_PROFILES); IPR001478 (SUPERFAMILY); IPR001478 (SUPERFAMILY)
1302	281486_length_738_cvg_64.1_tip_1_1	1935	14-3-3 protein zeta isoform x1	246	8.18E-139	96.00%	0.122		Coil (COILS); IPR000308 (PRINTS); IPR023410 (SMART); IPR023410 (PFAM); G3DSA:1.20.190.20 (GENE3D); IPR000308 (PIRSF); IPR000308 (PANTHER); IPR023409 (PROSITE_PATTERNS); IPR023410 (SUPERFAMILY)
1303	281500_length_738_cvg_3.0_tip_1_0	25	sh3 and multiple ankyrin repeat domains protein partial	246	2.65E-125	87.80%	0.142		IPR002110 (SMART); IPR020683 (PFAM); IPR020683 (G3DSA:1.25.40.GENE3D); PTHR24135 (PANTHER); IPR002110 (PROSITE_PROFILES); IPR002110 (PROSITE_PROFILES); IPR020683 (PROSITE_PROFILES); IPR020683 (SUPERFAMILY)
1304	281580_length_739_cvg_5.0_tip_1_1	47	dehydrogenase reductase sdr family member 11-like	246	1.78E-12	72.00%	0.106		PTHR24322 (PANTHER); PTHR24322:SF310 (PANTHER)
1306	281614_length_740_cvg_6.9_tip_1_5	74	3-hydroxybutyrate dehydrogenase type 2	246	1.18E-87	84.30%	0.114		IPR002347 (PRINTS); IPR002198 (PRINTS); PF13561 (PFAM); IPR016040 (G3DSA:3.40.50.GENE3D); PTHR24321 (PANTHER); PTHR24321:SF3 (PANTHER); IPR020904 (PROSITE_PATTERNS); TRANSMEMBRANE (PHOBIUS); CYTOPLASMIC_DOMAIN (PHOBIUS); NON_CYTOPLASMIC_DOMAIN (PHOBIUS); SSF51735 (SUPERFAMILY); TMhelix (TMHMM)
1308	281634_length_740_cvg_5.4_tip_1_2	44	zinc finger protein 726	246	3.04E-37	66.30%	0.105		IPR015880 (SMART); IPR013087 (G3DSA:3.30.160.GENE3D); PF13465 (PFAM); PF13912 (PFAM); IPR013087 (G3DSA:3.30.160.GENE3D); PTHR24409 (PANTHER); IPR007087 (PROSITE_PATTERNS); IPR007087 (PROSITE_PATTERNS); IPR007087 (PROSITE_PATTERNS); IPR007087 (PROSITE_PATTERNS); IPR007087 (PROSITE_PROFILES); IPR007087 (PROSITE_PROFILES); IPR007087 (PROSITE_PROFILES); IPR007087 (PROSITE_PROFILES); SSF57667 (SUPERFAMILY); SSF57667 (SUPERFAMILY)
1305	281586_length_739_cvg_59.2_tip_1_3	903	pacifastin light chain	247	1.04E-16	51.00%	0.425	Y	IPR008037 (PFAM); TRANSMEMBRANE (PHOBIUS); NON_CYTOPLASMIC_DOMAIN (PHOBIUS); CYTOPLASMIC_DOMAIN (PHOBIUS); IPR020862 (PROSITE_PROFILES); IPR020862 (PROSITE_PROFILES); IPR020862 (PROSITE_PROFILES); IPR008037 (SUPERFAMILY); IPR008037 (SUPERFAMILY); IPR008037 (SUPERFAMILY); TMhelix (TMHMM)
1307	281624_length_740_cvg_55.0_tip_1_0	687	ras-related protein rab-1a	247	3.18E-134	94.30%	0.178		IPR001806 (PRINTS); IPR003579 (SMART); IPR003578 (SMART); IPR020849 (SMART); IPR002041 (SMART); IPR024156 (SMART); IPR027417 (G3DSA:3.40.50.GENE3D); IPR005225 (TIGRFAM); IPR001806 (PFAM); PTHR24073 (PANTHER); PTHR24073:SF455 (PANTHER); PS51419 (PROSITE_PROFILES); IPR027417 (SUPERFAMILY)
1309	281782_length_743_cvg_3.0_tip_1_4	29	acyl- -binding domain-containing protein 5 isoform x3	248	2.43E-39	65.90%	0.128		IPR000582 (PRINTS); IPR000582 (PFAM); IPR014352 (G3DSA:1.20.80.GENE3D); PTHR23310:SF14 (PANTHER); PTHR23310 (PANTHER); IPR000582 (PROSITE_PROFILES); IPR000582 (SUPERFAMILY)
1310	281810_length_744_cvg_4.0_tip_1_3	37	cyclin-g-associated kinase-like	248	4.89E-101	79.90%	0.159		IPR002290 (SMART); IPR000719 (PFAM); G3DSA:3.30.200.20 (GENE3D); G3DSA:1.10.510.10 (GENE3D); PTHR23172 (PANTHER); PTHR23172:SF19 (PANTHER); IPR008271 (PROSITE_PATTERNS); IPR000719 (PROSITE_PROFILES); IPR011009 (SUPERFAMILY)
1311	281824_length_744_cvg_95.4_tip_0_3	2633	blastula protease 10-like	248	9.34E-33	50.40%	0.158		IPR000859 (SMART); IPR000859 (G3DSA:2.60.120.GENE3D); IPR001506 (PFAM); IPR000859 (PFAM); IPR024079 (G3DSA:3.40.390.GENE3D); PTHR10127 (PANTHER); PTHR10127:SF580 (PANTHER); IPR000859 (PROSITE_PROFILES); IPR000859 (SUPERFAMILY)

1312	281826_length_744_cvg_2.0_tip_1_3	22	gastrula zinc finger protein - partial	248	2.16E-48	54.00%	0.145	IPR015880 (SMART); PF13912 (PFAM); IPR013087 (G3DSA:3.30.160.GENE3D); PF13894 (PFAM); IPR013087 (G3DSA:3.30.160.GENE3D); IPR013087 (G3DSA:3.30.160.GENE3D); IPR013087 (G3DSA:3.30.160.GENE3D); IPR007087 (PFAM); PF13465 (PFAM); PTHR24409 (PANTHER); IPR007087 (PROSITE_PATTERNS); IPR007087 (PROSITE_PATTERNS); IPR007087 (PROSITE_PATTERNS); IPR007087 (PROSITE_PATTERNS); IPR007087 (PROSITE_PATTERNS); IPR007087 (PROSITE_PATTERNS); IPR007087 (PROSITE_PROFILES); IPR007087 (PROSITE_PROFILES); IPR007087 (PROSITE_PROFILES); IPR007087 (PROSITE_PROFILES); IPR007087 (PROSITE_PROFILES); IPR007087 (PROSITE_PROFILES); SSF57667 (SUPERFAMILY); SSF57667 (SUPERFAMILY); SSF57667 (SUPERFAMILY)
1313	281858_length_745_cvg_4.4_tip_1_0	50	---NA---	249			0.105	IPR002172 (SMART); IPR002172 (PFAM); IPR002172 (G3DSA:4.10.400.GENE3D); IPR002172 (PROSITE_PROFILES); IPR003609 (PROSITE_PROFILES); IPR003609 (PROSITE_PROFILES); IPR002172 (SUPERFAMILY)
1314	281878_length_746_cvg_2.7_tip_1_4	36	zinc finger	249	9.19E-29	58.10%	0.133	IPR015880 (SMART); IPR013087 (G3DSA:3.30.160.GENE3D); IPR013087 (G3DSA:3.30.160.GENE3D); PF13912 (PFAM); PF13465 (PFAM); IPR007087 (PFAM); PTHR24409 (PANTHER); PTHR24409:SF14 (PANTHER); IPR007087 (PROSITE_PATTERNS); IPR007087 (PROSITE_PATTERNS); IPR007087 (PROSITE_PATTERNS); IPR007087 (PROSITE_PATTERNS); IPR007087 (PROSITE_PATTERNS); IPR007087 (PROSITE_PATTERNS); IPR007087 (PROSITE_PROFILES); IPR007087 (PROSITE_PROFILES); IPR007087 (PROSITE_PROFILES); IPR007087 (PROSITE_PROFILES); IPR007087 (PROSITE_PROFILES); IPR007087 (PROSITE_PROFILES); IPR007087 (PROSITE_PROFILES); SSF57667 (SUPERFAMILY); SSF57667 (SUPERFAMILY); SSF57667 (SUPERFAMILY)
1315	281924_length_746_cvg_6.8_tip_1_3	60	galectin partial	249	1.12E-60	69.70%	0.098	IPR001079 (SMART); IPR001079 (SMART); IPR001079 (PFAM); IPR013320 (G3DSA:2.60.120.GENE3D); PTHR11346 (PANTHER); IPR001079 (PROSITE_PROFILES); IPR013320 (SUPERFAMILY)
1316	281972_length_748_cvg_2.9_tip_1_1	29	hypothetical protein	249	6.15E-11	45.00%	0.125	no IPS match
1317	281972_length_748_cvg_2.9_tip_1_4	29	tachykinins isoform x1	249	2.14E-41	54.20%	0.118	no IPS match
1318	282054_length_749_cvg_72.8_tip_0_3	3764	hypothetical protein BRAFLDRAFT_77844	250	7.20E-47	55.60%	0.149	IPR001547 (PFAM); IPR013781 (G3DSA:3.20.20.GENE3D); PTHR31308 (PANTHER); IPR017853 (SUPERFAMILY)
1319	282090_length_750_cvg_67.4_tip_1_4	1503	hemagglutinin amebocyte aggregation factor-like	250	2.58E-39	61.40%	0.116	PF14704 (PFAM); IPR026645 (PANTHER); NON_CYTOPLASMIC_DOMAIN (PHOBIUS); TRANSMEMBRANE (PHOBIUS); CYTOPLASMIC_DOMAIN (PHOBIUS); TMhelix (TMHMM)
1320	282098_length_750_cvg_2.0_tip_1_2	20	variable lymphocyte receptor a	250	2.85E-10	51.10%	0.116	IPR003591 (SMART); SM00364 (SMART); G3DSA:3.80.10.10 (GENE3D); IPR001611 (PFAM); IPR001611 (PFAM); PTHR24365 (PANTHER); IPR001611 (PROSITE_PROFILES); IPR001611 (PROSITE_PROFILES); IPR001611 (PROSITE_PROFILES); IPR001611 (PROSITE_PROFILES); IPR001611 (PROSITE_PROFILES); IPR001611 (PROSITE_PROFILES); IPR001611 (PROSITE_PROFILES); SSF52058 (SUPERFAMILY)
1321	282162_length_751_cvg_15.9_tip_1_4	154	ribosome biogenesis protein wdr12 homolog	250	2.77E-89	71.50%	0.103	IPR001680 (SMART); IPR012972 (PFAM); IPR001680 (PFAM); IPR015943 (G3DSA:2.130.10.GENE3D); PTHR19855 (PANTHER); IPR017986 (PROSITE_PROFILES); IPR001680 (PROSITE_PROFILES); IPR017986 (SUPERFAMILY)
1322	282216_length_753_cvg_62.0_tip_1_3	1433	kazal-type serine proteinase inhibitor 3	251	3.29E-24	56.90%	0.572 Y	IPR002350 (SMART); G3DSA:3.30.60.30 (GENE3D); IPR002350 (PFAM); G3DSA:3.30.60.30 (GENE3D); IPR002350 (PFAM); PTHR10913:SF46 (PANTHER); PTHR10913 (PANTHER); TRANSMEMBRANE (PHOBIUS); CYTOPLASMIC_DOMAIN (PHOBIUS); NON_CYTOPLASMIC_DOMAIN (PHOBIUS); NON_CYTOPLASMIC_DOMAIN (PHOBIUS); CYTOPLASMIC_DOMAIN (PHOBIUS); TRANSMEMBRANE (PHOBIUS); TRANSMEMBRANE (PHOBIUS); IPR002350 (PROSITE_PROFILES); IPR002350 (PROSITE_PROFILES); SignalP-TM (SIGNALP_EUK); SSF100895 (SUPERFAMILY); SSF100895 (SUPERFAMILY); TMhelix (TMHMM); TMhelix (TMHMM); TMhelix (TMHMM)
1323	282228_length_753_cvg_5.7_tip_1_2	50	myosin light chain	251	1.60E-100	76.00%	0.108	IPR003599 (SMART); IPR013783 (G3DSA:2.60.40.GENE3D); IPR013783 (G3DSA:2.60.40.GENE3D); IPR013783 (G3DSA:2.60.40.GENE3D); PF13895 (PFAM); IPR013098 (PFAM); PTHR19897 (PANTHER); IPR007110 (PROSITE_PROFILES); IPR007110 (PROSITE_PROFILES); IPR007110 (PROSITE_PROFILES); SSF48726 (SUPERFAMILY); SSF48726 (SUPERFAMILY); SSF48726 (SUPERFAMILY)
1324	282234_length_753_cvg_52.2_tip_1_1	454	annexin b10-like	251	8.53E-84	69.70%	0.103	IPR001464 (PRINTS); IPR018502 (SMART); IPR018502 (PFAM); IPR018502 (G3DSA:1.10.220.GENE3D); IPR018502 (G3DSA:1.10.220.GENE3D); IPR018502 (G3DSA:1.10.220.GENE3D); PTHR10502 (PANTHER); IPR018252 (PROSITE_PATTERNS); SSF47874 (SUPERFAMILY)

1326	282324_length_754_cvg_4.8_tip_1_5	47	cuticlin- partial	251	3.31E-133	81.80%	0.109	IPR001507 (SMART); IPR001507 (PFAM); PTHR22907:SF1 (PANTHER); PTHR22907 (PANTHER); IPR001507 (PROSITE_PROFILES)
1325	282276_length_754_cvg_2.1_tip_1_3	24	gamma-aminobutyric acid type b receptor subunit 1	252	8.53E-134	86.60%	0.223	PR01176 (PRINTS); IPR002456 (PRINTS); IPR017978 (PFAM); IPR002455 (PANTHER); PTHR10519:SF3 (PANTHER); TRANSMEMBRANE (PHOBIUS); NON_CYTOPLASMIC_DOMAIN (PHOBIUS); TRANSMEMBRANE (PHOBIUS); NON_CYTOPLASMIC_DOMAIN (PHOBIUS); TRANSMEMBRANE (PHOBIUS); TRANSMEMBRANE (PHOBIUS); CYTOPLASMIC_DOMAIN (PHOBIUS); NON_CYTOPLASMIC_DOMAIN (PHOBIUS); CYTOPLASMIC_DOMAIN (PHOBIUS); TRANSMEMBRANE (PHOBIUS); CYTOPLASMIC_DOMAIN (PHOBIUS); TRANSMEMBRANE (PHOBIUS); CYTOPLASMIC_DOMAIN (PHOBIUS); IPR017978 (PROSITE_PROFILES); TMhelix (TMHMM); TMhelix (TMHMM); TMhelix (TMHMM); TMhelix (TMHMM); TMhelix (TMHMM); TMhelix (TMHMM)
1327	282388_length_756_cvg_55.3_tip_1_1	1503	apolipoprotein d-like	252	5.13E-14	46.10%	0.438 Y	IPR000566 (PFAM); IPR012674 (G3DSA:2.40.128.GENE3D); PTHR10612 (PANTHER); PTHR10612:SF10 (PANTHER); IPR011038 (SUPERFAMILY)
1328	282460_length_757_cvg_4.0_tip_1_2	38	receptor-type tyrosine-protein phosphatase kappa	252	1.87E-94	74.40%	0.208	IPR000242 (PRINTS); IPR000242 (SMART); IPR000242 (PFAM); IPR029021 (G3DSA:3.90.190.GENE3D); PTHR19134:SF252 (PANTHER); PTHR19134 (PANTHER); CYTOPLASMIC_DOMAIN (PHOBIUS); TRANSMEMBRANE (PHOBIUS); NON_CYTOPLASMIC_DOMAIN (PHOBIUS); IPR000242 (PROSITE_PROFILES); IPR029021 (SUPERFAMILY); TMhelix (TMHMM)
1329	282484_length_758_cvg_6.0_tip_1_0	58	l-xylulose reductase	253	2.67E-92	78.50%	0.112	IPR002198 (PRINTS); IPR002347 (PRINTS); IPR016040 (G3DSA:3.40.50.GENE3D); PF13561 (PFAM); PTHR24311:SF6 (PANTHER); PTHR24311 (PANTHER); IPR020904 (PROSITE_PATTERNS); SSF51735 (SUPERFAMILY)
1330	282492_length_758_cvg_66.5_tip_1_3	1104	af425264_1lysozyme precursor	253	2.80E-46	63.90%	0.132	IPR001916 (PRINTS); IPR000974 (PRINTS); IPR001916 (SMART); G3DSA:1.10.530.10 (GENE3D); IPR001916 (PFAM); PTHR11407 (PANTHER); IPR019799 (PROSITE_PATTERNS); NON_CYTOPLASMIC_DOMAIN (PHOBIUS); CYTOPLASMIC_DOMAIN (PHOBIUS); TRANSMEMBRANE (PHOBIUS); IPR001916 (PROSITE_PROFILES); IPR023346 (SUPERFAMILY)
1331	282494_length_758_cvg_3.0_tip_1_3	32	3-hydroxyisobutyrate dehydrogenase	253	1.10E-113	85.30%	0.134	IPR029154 (PFAM); IPR006115 (PFAM); IPR013328 (G3DSA:1.10.1040.GENE3D); IPR011548 (TIGRFAM); IPR015815 (PIRSF); IPR016040 (G3DSA:3.40.50.GENE3D); PTHR22981:SF51 (PANTHER); PTHR22981 (PANTHER); IPR008927 (SUPERFAMILY); SSF51735 (SUPERFAMILY)
1334	282648_length_761_cvg_75.0_tip_1_2	15917	trypsinogen 2	253	8.60E-99	72.90%	0.816 Y	IPR001314 (PRINTS); IPR001254 (SMART); G3DSA:2.40.10.10 (GENE3D); G3DSA:2.40.10.10 (GENE3D); IPR001254 (PFAM); G3DSA:2.40.10.10 (GENE3D); PTHR24276 (PANTHER); IPR018114 (PROSITE_PATTERNS); IPR018114 (PROSITE_PATTERNS); NON_CYTOPLASMIC_DOMAIN (PHOBIUS); SIGNAL_PEPTIDE (PHOBIUS); SIGNAL_PEPTIDE_C_REGION (PHOBIUS); SIGNAL_PEPTIDE_N_REGION (PHOBIUS); SIGNAL_PEPTIDE_H_REGION (PHOBIUS); IPR001254 (PROSITE_PROFILES); SignalP-TM (SIGNALP_GRAM_POSITIVE); SignalP-noTM (SIGNALP_GRAM_NEGATIVE); SignalP-noTM (SIGNALP_EUK); IPR009003 (SUPERFAMILY); TMhelix (TMHMM)
1336	282660_length_761_cvg_13.3_tip_1_5	150	slit homolog 1	253	1.61E-51	67.10%	0.271	IPR003591 (SMART); IPR001611 (PFAM); IPR001611 (PFAM); G3DSA:3.80.10.10 (GENE3D); PTHR24373 (PANTHER); PTHR24373:SF98 (PANTHER); SSF52058 (SUPERFAMILY)
1332	282606_length_760_cvg_10.1_tip_1_3	95	peptidyl-prolyl cis-trans isomerase-like 3	254	1.69E-97	91.00%	0.145	IPR002130 (PRINTS); IPR002130 (PFAM); IPR029000 (G3DSA:2.40.100.GENE3D); IPR024936 (PANTHER); IPR020892 (PROSITE_PATTERNS); IPR002130 (PROSITE_PROFILES); IPR029000 (SUPERFAMILY)
1333	282608_length_760_cvg_8.8_tip_1_3	96	hemocytin isoform x2	254	1.85E-08	43.20%	0.109	IPR014853 (PFAM); IPR001846 (PROSITE_PROFILES)
1335	282656_length_761_cvg_3.0_tip_1_0	33	sialate o-acetylesterase isoform x1	254	2.89E-70	60.10%	0.338	IPR005181 (PFAM); PTHR22901 (PANTHER); IPR013830 (SUPERFAMILY)
1337	282764_length_763_cvg_2.3_tip_1_4	28	papilin isoform x2	254	3.21E-75	66.00%	0.124	IPR002223 (PRINTS); IPR002223 (SMART); IPR002223 (PFAM); IPR002223 (G3DSA:4.10.410.GENE3D); IPR002223 (G3DSA:4.10.410.GENE3D); PTHR10083 (PANTHER); IPR020901 (PROSITE_PATTERNS); IPR020901 (PROSITE_PATTERNS); IPR020901 (PROSITE_PATTERNS); IPR002223 (PROSITE_PROFILES); IPR002223 (PROSITE_PROFILES); IPR002223 (PROSITE_PROFILES); IPR002223 (SUPERFAMILY); IPR002223 (SUPERFAMILY); IPR002223 (SUPERFAMILY)
1338	282834_length_764_cvg_17.8_tip_1_5	164	epoxide hydrolase 4-like	254	8.34E-58	67.60%	0.104	IPR000639 (PRINTS); IPR000073 (PRINTS); IPR000073 (PFAM); IPR029058 (G3DSA:3.40.50.GENE3D); PTHR10992:SF721 (PANTHER); PTHR10992 (PANTHER); NON_CYTOPLASMIC_DOMAIN (PHOBIUS); CYTOPLASMIC_DOMAIN (PHOBIUS); TRANSMEMBRANE (PHOBIUS); IPR029058 (SUPERFAMILY); TMhelix (TMHMM)

1339	282860_length_764_cvg_2.0_tip_1_4	24	mitogen-activated protein kinase erk-	255	3.00E-145	87.10%	0.107		IPR002290 (SMART); G3DSA:3.30.200.20 (GENE3D); G3DSA:1.10.510.10 (GENE3D); IPR000719 (PFAM); PTHR24360 (PANTHER); PTHR24360:SF15 (PANTHER); IPR017441 (PROSITE_PATTERNS); IPR008271 (PROSITE_PATTERNS); IPR000719 (PROSITE_PROFILES); IPR011009 (SUPERFAMILY)
1340	282982_length_767_cvg_15.4_tip_1_4	195	na(+) h(+) exchange regulatory cofactor nhe-rf2	256	2.30E-56	73.60%	0.161		IPR001478 (SMART); IPR001478 (PFAM); IPR001478 (G3DSA:2.30.42.GENE3D); PTHR14191 (PANTHER); IPR001478 (PROSITE_PROFILES); IPR001478 (SUPERFAMILY)
1341	283054_length_768_cvg_11.0_tip_1_0	102	serpin b3-like	256	8.79E-46	53.30%	0.171		IPR023796 (SMART); G3DSA:2.30.39.10 (GENE3D); G3DSA:3.30.497.10 (GENE3D); IPR023796 (PFAM); IPR000215 (PANTHER); IPR023796 (SUPERFAMILY)
1343	283096_length_769_cvg_65.2_tip_1_2	1112	ferritin	256	7.28E-86	84.70%	0.726	Y	Coil (COILS); IPR008331 (PFAM); IPR012347 (G3DSA:1.20.1260.GENE3D); IPR001519 (PANTHER); IPR014034 (PROSITE_PATTERNS); IPR014034 (PROSITE_PATTERNS); SIGNAL_PEPTIDE_H_REGION (PHOBIUS); SIGNAL_PEPTIDE_C_REGION (PHOBIUS); NON_CYTOPLASMIC_DOMAIN (PHOBIUS); SIGNAL_PEPTIDE_N_REGION (PHOBIUS); SIGNAL_PEPTIDE (PHOBIUS); IPR009040 (PROSITE_PROFILES); SignalP-noTM (SIGNALP_EUK); IPR009078 (SUPERFAMILY)
1342	283084_length_769_cvg_22.7_tip_1_0	193	ectonucleoside triphosphate diphosphohydrolase 5-partial	257	1.81E-19	81.90%	0.156		IPR000407 (PFAM); IPR000407 (PANTHER)
1344	283142_length_770_cvg_10.5_tip_1_3	129	pdz domain-containing protein gipc3	257	1.22E-141	85.80%	0.11		IPR001478 (SMART); IPR001478 (G3DSA:2.30.42.GENE3D); IPR001478 (PFAM); PTHR12259 (PANTHER); IPR001478 (PROSITE_PROFILES); IPR001478 (SUPERFAMILY)
1345	283236_length_772_cvg_4.7_tip_1_2	45	zinc finger protein 431-like isoform x2	257	8.18E-34	55.90%	0.1		IPR015880 (SMART); PF13912 (PFAM); PF13465 (PFAM); IPR013087 (G3DSA:3.30.160.GENE3D); IPR013087 (G3DSA:3.30.160.GENE3D); IPR013087 (G3DSA:3.30.160.GENE3D); PTHR24409 (PANTHER); IPR007087 (PROSITE_PATTERNS); IPR007087 (PROSITE_PATTERNS); IPR007087 (PROSITE_PATTERNS); IPR007087 (PROSITE_PROFILES); IPR007087 (PROSITE_PROFILES); IPR007087 (PROSITE_PROFILES); IPR007087 (PROSITE_PROFILES); SSF57667 (SUPERFAMILY); SSF57667 (SUPERFAMILY)
1346	283428_length_776_cvg_14.7_tip_1_1	119	collagen alpha type	259	5.31E-54	70.60%	0.111		IPR010515 (PFAM); IPR016186 (G3DSA:3.10.100.GENE3D); PTHR24023:SF461 (PANTHER); PTHR24023 (PANTHER); IPR016187 (SUPERFAMILY)
1347	283462_length_777_cvg_3.8_tip_1_0	31	zinc finger protein 271-like	259	2.78E-71	66.80%	0.099		IPR015880 (SMART); PF13912 (PFAM); IPR013087 (G3DSA:3.30.160.GENE3D); IPR013087 (G3DSA:3.30.160.GENE3D); IPR013087 (G3DSA:3.30.160.GENE3D); PF13465 (PFAM); IPR013087 (G3DSA:3.30.160.GENE3D); IPR013087 (G3DSA:3.30.160.GENE3D); IPR007087 (PFAM); PTHR24409 (PANTHER); IPR007087 (PROSITE_PATTERNS); IPR007087 (PROSITE_PATTERNS); IPR007087 (PROSITE_PATTERNS); IPR007087 (PROSITE_PATTERNS); IPR007087 (PROSITE_PATTERNS); IPR007087 (PROSITE_PATTERNS); IPR007087 (PROSITE_PATTERNS); IPR007087 (PROSITE_PROFILES); IPR007087 (PROSITE_PROFILES); IPR007087 (PROSITE_PROFILES); IPR007087 (PROSITE_PROFILES); IPR007087 (PROSITE_PROFILES); IPR007087 (PROSITE_PROFILES); IPR007087 (PROSITE_PROFILES); SSF57667 (SUPERFAMILY); SSF57667 (SUPERFAMILY); SSF57667 (SUPERFAMILY); SSF57667 (SUPERFAMILY)
1348	283470_length_777_cvg_2.6_tip_1_3	26	heat-shock protein 105	259	8.36E-127	81.30%	0.123		IPR013126 (PRINTS); IPR013126 (PFAM); G3DSA:3.30.420.40 (GENE3D); G3DSA:3.30.420.40 (GENE3D); G3DSA:3.90.640.10 (GENE3D); PTHR19375 (PANTHER); PTHR19375:SF78 (PANTHER); IPR018181 (PROSITE_PATTERNS); SSF53067 (SUPERFAMILY); SSF53067 (SUPERFAMILY)
1384	284792_length_807_cvg_6.4_tip_1_1	75	virulence metalloprotease	259	1.10E-18	54.40%	0.286		IPR011096 (PFAM); IPR025711 (PFAM); SIGNAL_PEPTIDE_C_REGION (PHOBIUS); SIGNAL_PEPTIDE (PHOBIUS); CYTOPLASMIC_DOMAIN (PHOBIUS); TRANSMEMBRANE (PHOBIUS); SIGNAL_PEPTIDE_N_REGION (PHOBIUS); SIGNAL_PEPTIDE_H_REGION (PHOBIUS); NON_CYTOPLASMIC_DOMAIN (PHOBIUS); TMhelix (TMHMM)
1349	283644_length_780_cvg_10.8_tip_1_3	122	glutathione peroxidase 7	260	7.44E-76	71.80%	0.12		IPR000889 (PRINTS); IPR000889 (PFAM); IPR012336 (G3DSA:3.40.30.GENE3D); IPR000889 (PANTHER); PTHR11592:SF10 (PANTHER); IPR029759 (PROSITE_PATTERNS); IPR029760 (PROSITE_PATTERNS); IPR000889 (PROSITE_PROFILES); IPR012336 (SUPERFAMILY); TMhelix (TMHMM)
1350	283702_length_782_cvg_2.0_tip_1_2	25	glutamate synthase	260	2.96E-143	88.70%	0.102		PR00419 (PRINTS); PF13450 (PFAM); IPR028261 (PFAM); G3DSA:1.10.1060.10 (GENE3D); G3DSA:3.50.50.60 (GENE3D); PTHR11938 (PANTHER); PTHR11938:SF61 (PANTHER); IPR009051 (SUPERFAMILY); SSF51971 (SUPERFAMILY)

1351	283704_length_782_cvg_3.7_tip_1_3	38	serine protease p153	261	1.07E-09	50.10%	0.115	IPR002172 (SMART); IPR002172 (PFAM); IPR003014 (PFAM); IPR002172 (G3DSA:4.10.400.GENE3D); G3DSA:3.50.4.10 (GENE3D); IPR002172 (PROSITE_PROFILES); IPR003609 (PROSITE_PROFILES); IPR003609 (PROSITE_PROFILES); IPR002172 (SUPERFAMILY)
1352	283712_length_782_cvg_4.7_tip_1_1	41	multiple epidermal growth factor-like domains protein 10 isoform x1	261	1.86E-94	67.90%	0.201	PR00011 (PRINTS); IPR002049 (SMART); IPR000742 (SMART); G3DSA:2.170.300.10 (GENE3D); IPR013032 (PFAM); G3DSA:2.10.25.10 (GENE3D); IPR002049 (PFAM); PTHR24035:SF4 (PANTHER); PTHR24035 (PANTHER); IPR013032 (PROSITE_PATTERNS); IPR013032 (PROSITE_PATTERNS); IPR013032 (PROSITE_PATTERNS); IPR013032 (PROSITE_PATTERNS); IPR013032 (PROSITE_PATTERNS); IPR013032 (PROSITE_PATTERNS); IPR013032 (PROSITE_PATTERNS); IPR013032 (PROSITE_PATTERNS); IPR013032 (PROSITE_PATTERNS); IPR000742 (PROSITE_PROFILES); IPR000742 (PROSITE_PROFILES); IPR000742 (PROSITE_PROFILES); IPR000742 (PROSITE_PROFILES); IPR000742 (PROSITE_PROFILES)
1353	283720_length_782_cvg_7.3_tip_1_3	75	spermatogenesis-associated protein 20- partial	261	2.49E-86	70.70%	0.118	PTHR12145:SF11 (PANTHER); PTHR12145 (PANTHER); IPR008928 (SUPERFAMILY)
1354	283734_length_782_cvg_5.0_tip_1_3	43	low-density lipoprotein	261	5.13E-80	63.90%	0.146	IPR000033 (SMART); IPR000742 (SMART); IPR000033 (PFAM); G3DSA:2.10.25.10 (GENE3D); G3DSA:2.10.25.10 (GENE3D); IPR011042 (G3DSA:2.120.10.GENE3D); G3DSA:2.10.25.10 (GENE3D); IPR013032 (PFAM); PTHR10529 (PANTHER); PTHR10529:SF204 (PANTHER); IPR013032 (PROSITE_PATTERNS); IPR000742 (PROSITE_PROFILES); IPR000033 (PROSITE_PROFILES); IPR000742 (PROSITE_PROFILES); IPR000033 (PROSITE_PROFILES); SSF63825 (SUPERFAMILY)
1355	283848_length_785_cvg_10.9_tip_1_4	98	mam and ldl-receptor class a domain-containing protein c10orf112-like	262	2.05E-06	50.33%	0.099	IPR000998 (SMART); IPR000998 (PFAM); PTHR23282:SF75 (PANTHER); PTHR23282 (PANTHER); IPR000998 (PROSITE_PROFILES); IPR013320 (SUPERFAMILY)
1356	283868_length_785_cvg_3.9_tip_1_1	39	p60 family protein	262	2.23E-23	47.90%	0.102	no IPS match
1357	283930_length_787_cvg_8.7_tip_1_4	77	protein tanc2 isoform x2	262	2.23E-91	77.20%	0.252	IPR002110 (SMART); IPR020683 (G3DSA:1.25.40.GENE3D); IPR020683 (G3DSA:1.25.40.GENE3D); IPR020683 (PFAM); IPR002110 (PFAM); PTHR24166 (PANTHER); PTHR24166:SF8 (PANTHER); IPR002110 (PROSITE_PROFILES); IPR002110 (PROSITE_PROFILES); IPR002110 (PROSITE_PROFILES); IPR020683 (PROSITE_PROFILES); IPR020683 (SUPERFAMILY)
1358	283938_length_787_cvg_4.5_tip_1_2	44	acid sphingomyelinase-like phosphodiesterase 3b	262	7.84E-17	55.20%	0.112	PTHR10340 (PANTHER)
1359	283944_length_787_cvg_61.0_tip_1_2	1226	clotting factor b-like	262	4.37E-12	54.10%	0.299	G3DSA:2.40.10.10 (GENE3D); IPR001254 (PFAM); PTHR24276 (PANTHER); IPR018114 (PROSITE_PATTERNS); CYTOPLASMIC_DOMAIN (PHOBIUS); NON_CYTOPLASMIC_DOMAIN (PHOBIUS); TRANSMEMBRANE (PHOBIUS); IPR009003 (SUPERFAMILY)
1361	283976_length_788_cvg_10.0_tip_1_5	122	coagulation factor x	262	2.85E-23	58.50%	0.128	IPR001314 (PRINTS); IPR001254 (SMART); G3DSA:2.40.10.10 (GENE3D); IPR001254 (PFAM); G3DSA:2.40.10.10 (GENE3D); PTHR24265:SF76 (PANTHER); PTHR24265 (PANTHER); IPR018114 (PROSITE_PATTERNS); IPR001254 (PROSITE_PROFILES); IPR009003 (SUPERFAMILY)
1362	283984_length_788_cvg_2.4_tip_1_2	41	endothelin-converting enzyme 1 isoform x2	262	2.70E-94	67.10%	0.145	IPR018497 (PRINTS); IPR024079 (G3DSA:3.40.390.GENE3D); IPR018497 (PFAM); IPR008753 (PFAM); IPR029733 (PTHR11733:PANTHER); IPR000718 (PANTHER); SSF55486 (SUPERFAMILY)
1360	283966_length_787_cvg_2.0_tip_1_0	27	zinc finger protein 420-like isoform x1	263	2.02E-47	57.40%	0.121	IPR015880 (SMART); IPR007087 (PFAM); PF13894 (PFAM); IPR013087 (G3DSA:3.30.160.GENE3D); IPR013087 (G3DSA:3.30.160.GENE3D); IPR013087 (G3DSA:3.30.160.GENE3D); PF13465 (PFAM); PTHR24409:SF14 (PANTHER); PTHR24409 (PANTHER); IPR007087 (PROSITE_PATTERNS); IPR007087 (PROSITE_PATTERNS); IPR007087 (PROSITE_PATTERNS); IPR007087 (PROSITE_PATTERNS); IPR007087 (PROSITE_PATTERNS); IPR007087 (PROSITE_PATTERNS); IPR007087 (PROSITE_PATTERNS); IPR007087 (PROSITE_PROFILES); IPR007087 (PROSITE_PROFILES); IPR007087 (PROSITE_PROFILES); IPR007087 (PROSITE_PROFILES); IPR007087 (PROSITE_PROFILES); IPR007087 (PROSITE_PROFILES); IPR007087 (PROSITE_PROFILES); SSF57667 (SUPERFAMILY); SSF57667 (SUPERFAMILY); SSF57667 (SUPERFAMILY); SSF57667 (SUPERFAMILY); SSF57667 (SUPERFAMILY)

1363	284020_length_788_cvg_2.1_tip_1_3	22	asparagine-rich zinc finger protein azf1-like isoform x2	263	5.63E-65	69.00%	0.107		IPR015880 (SMART); IPR013087 (G3DSA:3.30.160.GENE3D); PF13465 (PFAM); IPR013087 (G3DSA:3.30.160.GENE3D); IPR007087 (PFAM); IPR013087 (G3DSA:3.30.160.GENE3D); PTHR24409 (PANTHER); IPR007087 (PROSITE_PATTERNS); IPR007087 (PROSITE_PATTERNS); IPR007087 (PROSITE_PATTERNS); IPR007087 (PROSITE_PATTERNS); IPR007087 (PROSITE_PATTERNS); IPR007087 (PROSITE_PROFILES); IPR007087 (PROSITE_PROFILES); IPR007087 (PROSITE_PROFILES); IPR007087 (PROSITE_PROFILES); IPR007087 (PROSITE_PROFILES); IPR007087 (PROSITE_PROFILES); SSF57667 (SUPERFAMILY); SSF57667 (SUPERFAMILY); SSF57667 (SUPERFAMILY)
1364	284028_length_789_cvg_6.4_tip_1_1	62	insulin-like growth factor binding protein 7-like protein	263	2.79E-08	51.80%	0.636	Y	IPR000867 (SMART); IPR000867 (PFAM); IPR011390 (PANTHER); PTHR14186:SF8 (PANTHER); NON_CYTOPLASMIC_DOMAIN (PHOBIUS); SIGNAL_PEPTIDE (PHOBIUS); SIGNAL_PEPTIDE_C_REGION (PHOBIUS); SIGNAL_PEPTIDE_N_REGION (PHOBIUS); SIGNAL_PEPTIDE_H_REGION (PHOBIUS); IPR000867 (PROSITE_PROFILES); SignalP-noTM (SIGNALP_EUK); IPR009030 (SUPERFAMILY); TMhelix (TMHMM)
1365	284038_length_789_cvg_60.9_tip_1_1	80801	soma ferritin-like	263	2.07E-74	80.50%	0.113		IPR012347 (G3DSA:1.20.1260.GENE3D); IPR008331 (PFAM); IPR001519 (PANTHER); IPR009040 (PROSITE_PROFILES); IPR009078 (SUPERFAMILY)
1366	284116_length_790_cvg_2.9_tip_1_5	30	periostin- partial	263	2.47E-23	50.10%	0.363	Y	IPR000782 (SMART); IPR000782 (G3DSA:2.30.180.GENE3D); IPR000782 (PFAM); PTHR10900 (PANTHER); PTHR10900:SF72 (PANTHER); TRANSMEMBRANE (PHOBIUS); CYTOPLASMIC_DOMAIN (PHOBIUS); NON_CYTOPLASMIC_DOMAIN (PHOBIUS); IPR000782 (PROSITE_PROFILES); IPR000782 (SUPERFAMILY)
1367	284208_length_792_cvg_42.2_tip_1_0	303	probable nuclear transport factor 2 isoform x1	264	3.98E-47	82.00%	0.097		IPR002075 (PFAM); G3DSA:3.10.450.50 (GENE3D); PTHR12612 (PANTHER); IPR018222 (PROSITE_PROFILES); SSF54427 (SUPERFAMILY)
1368	284316_length_794_cvg_12.4_tip_1_4	92	tunicate retinoic acid-inducible modular protease	265	7.10E-13	48.40%	0.133		IPR002172 (PRINTS); IPR002172 (SMART); IPR002172 (G3DSA:4.10.400.GENE3D); IPR002172 (G3DSA:4.10.400.GENE3D); IPR003014 (PFAM); IPR002172 (PFAM); IPR023415 (PROSITE_PATTERNS); IPR003609 (PROSITE_PROFILES); IPR002172 (PROSITE_PROFILES); IPR003609 (PROSITE_PROFILES); IPR002172 (PROSITE_PROFILES); IPR002172 (SUPERFAMILY); SSF57414 (SUPERFAMILY); IPR002172 (SUPERFAMILY)
1369	284342_length_795_cvg_59.0_tip_1_1	1132	pacifastin light chain	265	6.92E-16	51.90%	0.474	Y	IPR008197 (PRINTS); IPR008197 (SMART); IPR008197 (PFAM); IPR008197 (G3DSA:4.10.75.GENE3D); IPR008037 (PFAM); IPR020862 (PROSITE_PROFILES); IPR020862 (PROSITE_PROFILES); IPR020862 (PROSITE_PROFILES); IPR008197 (PROSITE_PROFILES); IPR008037 (SUPERFAMILY); IPR008037 (SUPERFAMILY); IPR008037 (SUPERFAMILY); IPR008197 (SUPERFAMILY); TMhelix (TMHMM)
1370	284350_length_795_cvg_59.9_tip_1_5	9361	unnamed protein product	265	1.58E-09	73.00%	0.148		no IPS match
1371	284378_length_796_cvg_3.5_tip_1_0	40	adp-ribosylation factor-like protein 1	266	5.11E-90	95.50%	0.104		IPR006689 (PRINTS); IPR006687 (SMART); IPR024156 (SMART); IPR027417 (G3DSA:3.40.50.GENE3D); IPR005225 (TIGRFAM); IPR006689 (PFAM); PTHR11711:SF41 (PANTHER); PTHR11711 (PANTHER); IPR024156 (PROSITE_PROFILES); IPR027417 (SUPERFAMILY)
1372	284434_length_798_cvg_10.9_tip_1_4	133	beta-galactosidase-1-like protein 2	266	1.21E-77	69.50%	0.148		IPR001944 (PRINTS); IPR013781 (G3DSA:3.20.20.GENE3D); IPR001944 (PFAM); PTHR23421:SF66 (PANTHER); IPR001944 (PANTHER); IPR019801 (PROSITE_PATTERNS); IPR017853 (SUPERFAMILY)
1373	284462_length_798_cvg_3.3_tip_1_5	33	e3 ubiquitin-protein ligase sh3rf1	266	3.43E-29	56.70%	0.106		IPR001452 (PRINTS); IPR001452 (SMART); G3DSA:2.30.30.40 (GENE3D); IPR001452 (PFAM); PTHR10661 (PANTHER); PTHR10661:SF66 (PANTHER); IPR001452 (PROSITE_PROFILES); SignalP-TM (SIGNALP_GRAM_POSITIVE); IPR001452 (SUPERFAMILY)
1376	284530_length_800_cvg_4.5_tip_1_5	43	ves g 5 allergen	266	4.88E-44	51.40%	0.467	Y	IPR001283 (PRINTS); IPR002413 (PRINTS); IPR014044 (SMART); IPR014044 (G3DSA:3.40.33.GENE3D); IPR014044 (PFAM); IPR001283 (PANTHER); NON_CYTOPLASMIC_DOMAIN (PHOBIUS); TRANSMEMBRANE (PHOBIUS); CYTOPLASMIC_DOMAIN (PHOBIUS); SignalP-TM (SIGNALP_GRAM_POSITIVE); IPR014044 (SUPERFAMILY); TMhelix (TMHMM)
1374	284508_length_800_cvg_4.0_tip_1_4	41	probable chitinase	267	1.07E-20	50.40%	0.138		IPR011583 (SMART); IPR013781 (G3DSA:3.20.20.GENE3D); IPR001223 (PFAM); PTHR11177 (PANTHER); IPR017853 (SUPERFAMILY)
1375	284526_length_800_cvg_4.6_tip_1_3	54	estradiol 17-beta-dehydrogenase 12	267	1.45E-40	67.00%	0.12		IPR002198 (PRINTS); IPR002347 (PRINTS); IPR016040 (G3DSA:3.40.50.GENE3D); IPR002198 (PFAM); PTHR24316:SF68 (PANTHER); PTHR24316 (PANTHER); NON_CYTOPLASMIC_DOMAIN (PHOBIUS); CYTOPLASMIC_DOMAIN (PHOBIUS); TRANSMEMBRANE (PHOBIUS); CYTOPLASMIC_DOMAIN (PHOBIUS); TRANSMEMBRANE (PHOBIUS); SSF51735 (SUPERFAMILY); TMhelix (TMHMM); TMhelix (TMHMM)
1377	284536_length_801_cvg_4.8_tip_1_5	58	calcium-activated chloride channel regulator 1-like	267	5.11E-25	53.80%	0.106		PTHR10579 (PANTHER); PTHR10579:SF8 (PANTHER); NON_CYTOPLASMIC_DOMAIN (PHOBIUS); TRANSMEMBRANE (PHOBIUS); CYTOPLASMIC_DOMAIN (PHOBIUS); TMhelix (TMHMM)

1378	284652_length_803_cvg_10.3_tip_0_0	83	d-aspartate oxidase	268	1.60E-114	73.90%	0.1	G3DSA:3.30.9.10 (GENE3D); IPR006076 (PFAM); PTHR11530:SF2 (PANTHER); IPR023209 (PANTHER); SSF54373 (SUPERFAMILY); SSF51971 (SUPERFAMILY)
1379	284664_length_803_cvg_3.6_tip_1_0	32	pleiotropic regulator 1	268	6.70E-110	72.30%	0.109	IPR020472 (PRINTS); IPR001680 (SMART); IPR015943 (G3DSA:2.130.10.GENE3D); IPR001680 (PFAM); PTHR19923:SF0 (PANTHER); PTHR19923 (PANTHER); IPR019775 (PROSITE_PATTERNS); IPR001680 (PROSITE_PROFILES); IPR001680 (PROSITE_PROFILES); IPR001680 (PROSITE_PROFILES); IPR017986 (PROSITE_PROFILES); IPR017986 (SUPERFAMILY)
1380	284672_length_804_cvg_43.9_tip_1_3	712	peptidase	268	6.45E-10	48.20%	0.141	IPR025711 (PFAM); CYTOPLASMIC_DOMAIN (PHOBIUS); NON_CYTOPLASMIC_DOMAIN (PHOBIUS); TRANSMEMBRANE (PHOBIUS); TMhelix (TMHMM)
1381	284680_length_804_cvg_3.0_tip_1_3	35	cgmp-dependent protein isozyme 2 forms cd4 t1 t3a t3b-like isoform x3	268	4.57E-143	90.10%	0.11	IPR002290 (SMART); IPR000961 (SMART); G3DSA:1.10.510.10 (GENE3D); IPR000719 (PFAM); PTHR24353 (PANTHER); PTHR24353:SF65 (PANTHER); IPR008271 (PROSITE_PATTERNS); IPR000961 (PROSITE_PROFILES); IPR000719 (PROSITE_PROFILES); IPR011009 (SUPERFAMILY)
1382	284740_length_806_cvg_3.7_tip_1_4	32	peptidyl-prolyl cis-trans isomerase d	269	5.13E-120	75.20%	0.096	IPR002130 (PRINTS); IPR029000 (G3DSA:2.40.100.GENE3D); IPR023114 (G3DSA:1.10.150.GENE3D); IPR002130 (PFAM); PTHR11071:SF78 (PANTHER); IPR024936 (PANTHER); IPR020892 (PROSITE_PATTERNS); IPR002130 (PROSITE_PROFILES); IPR029000 (SUPERFAMILY)
1383	284780_length_806_cvg_2.0_tip_1_0	22	microtubule-associated protein futsch isoform x1	269	7.86E-118	95.00%	0.151	IPR002290 (SMART); G3DSA:1.10.510.10 (GENE3D); IPR000719 (PFAM); G3DSA:3.30.200.20 (GENE3D); PTHR24343:SF26 (PANTHER); PTHR24343 (PANTHER); IPR017441 (PROSITE_PATTERNS); IPR008271 (PROSITE_PATTERNS); IPR000719 (PROSITE_PROFILES); IPR011009 (SUPERFAMILY)
1385	284806_length_807_cvg_42.5_tip_1_3	703	ecdysteroid-regulated 16 kda protein	269	1.02E-17	54.20%	0.563 Y	IPR003172 (SMART); IPR003172 (PFAM); IPR003172 (G3DSA:2.60.40.GENE3D); PTHR11306 (PANTHER); SIGNAL_PEPTIDE_H_REGION (PHOBIUS); SIGNAL_PEPTIDE_C_REGION (PHOBIUS); NON_CYTOPLASMIC_DOMAIN (PHOBIUS); SIGNAL_PEPTIDE_N_REGION (PHOBIUS); SIGNAL_PEPTIDE (PHOBIUS); SignalP-noTM (SIGNALP_EUK); IPR014756 (SUPERFAMILY)
1386	284826_length_807_cvg_3.0_tip_1_5	32	sortilin-related receptor-like	269	1.49E-106	76.30%	0.141	IPR000033 (SMART); IPR000033 (PFAM); IPR011042 (G3DSA:2.120.10.GENE3D); PTHR12106:SF7 (PANTHER); PTHR12106 (PANTHER); IPR000033 (PROSITE_PROFILES); IPR000033 (PROSITE_PROFILES); IPR000033 (PROSITE_PROFILES); IPR000033 (PROSITE_PROFILES); SSF63825 (SUPERFAMILY)
1388	284850_length_808_cvg_3.8_tip_1_4	34	zinc finger protein 271 (zinc finger protein 7) (zinc finger protein znfphex133) (epstein-barr virus-induced zinc finger protein) (znf-eb) (ct-zfp48) (zinc finger protein	269	9.87E-41	54.30%	0.108	IPR015880 (SMART); IPR013087 (G3DSA:3.30.160.GENE3D); PF13909 (PFAM); IPR007087 (PFAM); IPR013087 (G3DSA:3.30.160.GENE3D); PF13894 (PFAM); IPR013087 (G3DSA:3.30.160.GENE3D); PF13465 (PFAM); PTHR24409 (PANTHER); PTHR24409:SF14 (PANTHER); IPR007087 (PROSITE_PATTERNS); IPR007087 (PROSITE_PATTERNS); IPR007087 (PROSITE_PATTERNS); IPR007087 (PROSITE_PATTERNS); IPR007087 (PROSITE_PATTERNS); IPR007087 (PROSITE_PATTERNS); IPR007087 (PROSITE_PATTERNS); IPR007087 (PROSITE_PROFILES); IPR007087 (PROSITE_PROFILES); IPR007087 (PROSITE_PROFILES); IPR007087 (PROSITE_PROFILES); IPR007087 (PROSITE_PROFILES); IPR007087 (PROSITE_PROFILES); IPR007087 (PROSITE_PROFILES); IPR007087 (PROSITE_PROFILES); IPR007087 (PROSITE_PROFILES); SSF57667 (SUPERFAMILY); SSF57667 (SUPERFAMILY); SSF57667 (SUPERFAMILY); SSF57667 (SUPERFAMILY); SSF57667 (SUPERFAMILY)
1414	285300_length_820_cvg_2.0_tip_1_1	23	cd109 partial	269	1.10E-42	63.80%	0.284	PTHR11412:SF85 (PANTHER); PTHR11412 (PANTHER); NON_CYTOPLASMIC_DOMAIN (PHOBIUS); TRANSMEMBRANE (PHOBIUS); CYTOPLASMIC_DOMAIN (PHOBIUS)
1387	284846_length_808_cvg_5.0_tip_1_3	60	maltase partial	270	2.85E-60	63.60%	0.145	IPR006589 (SMART); IPR006047 (PFAM); IPR013781 (G3DSA:3.20.20.GENE3D); IPR015902 (PANTHER); PTHR10357:SF77 (PANTHER); TRANSMEMBRANE (PHOBIUS); CYTOPLASMIC_DOMAIN (PHOBIUS); NON_CYTOPLASMIC_DOMAIN (PHOBIUS); IPR017853 (SUPERFAMILY); TMhelix (TMHMM)
1389	284920_length_810_cvg_3.7_tip_1_1	41	zinc finger protein partial	270	1.54E-91	74.10%	0.121	IPR015880 (SMART); IPR007087 (PFAM); IPR013087 (G3DSA:3.30.160.GENE3D); PF13465 (PFAM); IPR013087 (G3DSA:3.30.160.GENE3D); IPR013087 (G3DSA:3.30.160.GENE3D); PF13894 (PFAM); PTHR24409 (PANTHER); IPR007087 (PROSITE_PATTERNS); IPR007087 (PROSITE_PATTERNS); IPR007087 (PROSITE_PATTERNS); IPR007087 (PROSITE_PROFILES); IPR007087 (PROSITE_PROFILES); IPR007087 (PROSITE_PROFILES); IPR007087 (PROSITE_PROFILES); IPR007087 (PROSITE_PROFILES); SSF57667 (SUPERFAMILY); SSF57667 (SUPERFAMILY); SSF57667 (SUPERFAMILY)

1392	284976_length_811_cvg_19.9_tip_1_2	188	pneumococcal surface protein c	270	2.91E-13	44.90%	0.455	Y	SIGNAL_PEPTIDE_N_REGION (PHOBIUS); NON_CYTOPLASMIC_DOMAIN (PHOBIUS); SIGNAL_PEPTIDE_C_REGION (PHOBIUS); SIGNAL_PEPTIDE_H_REGION (PHOBIUS); SIGNAL_PEPTIDE (PHOBIUS); SignalP-noTM (SIGNALP_EUK)
1395	284992_length_812_cvg_4.9_tip_1_5	58	neurexin-4 isoform x1	270	4.25E-31	48.70%	0.491	Y	Coil (COILS); IPR002223 (PRINTS); IPR002223 (SMART); IPR002223 (PFAM); IPR002223 (G3DSA:4.10.410.GENE3D); IPR013320 (G3DSA:2.60.120.GENE3D); PTHR10083 (PANTHER); IPR029858 (PTHR10083:PANTHER); IPR020901 (PROSITE_PATTERNS); IPR002223 (PROSITE_PROFILES); SignalP-noTM (SIGNALP_EUK); IPR002223 (SUPERFAMILY)
1390	284942_length_811_cvg_5.0_tip_1_0	46	laminin subunit gamma-1	271	2.36E-62	66.90%	0.105		Coil (COILS); Coil (COILS); Coil (COILS); PTHR10574:SF228 (PANTHER); PTHR10574 (PANTHER)
1391	284970_length_811_cvg_11.8_tip_1_3	95	dipeptidyl peptidase 1 isoform x1	271	4.43E-80	83.00%	0.102		IPR000668 (SMART); IPR000668 (PFAM); G3DSA:3.90.70.10 (GENE3D); G3DSA:2.40.50.170 (GENE3D); PTHR12411:SF314 (PANTHER); IPR013128 (PANTHER); IPR025660 (PROSITE_PATTERNS); IPR025661 (PROSITE_PATTERNS); SSF54001 (SUPERFAMILY)
1393	284984_length_812_cvg_12.6_tip_1_3	116	peptidyl-prolyl cis-trans isomerase cyp11 isoform x1	271	2.84E-106	88.20%	0.133		IPR002130 (PRINTS); IPR002130 (PFAM); IPR029000 (G3DSA:2.40.100.GENE3D); PTHR11071:SF229 (PANTHER); IPR024936 (PANTHER); IPR020892 (PROSITE_PATTERNS); IPR002130 (PROSITE_PROFILES); IPR029000 (SUPERFAMILY)
1394	284990_length_812_cvg_2.0_tip_1_4	28	thrombospondin type-1 domain-containing protein 4-like	271	1.44E-67	63.60%	0.122		IPR000884 (SMART); IPR000884 (PFAM); G3DSA:2.20.100.10 (GENE3D); PTHR13723 (PANTHER); IPR000884 (PROSITE_PROFILES); IPR000884 (PROSITE_PROFILES); IPR000884 (PROSITE_PROFILES); IPR000884 (SUPERFAMILY); IPR000884 (SUPERFAMILY); IPR000884 (SUPERFAMILY)
1396	285024_length_813_cvg_10.7_tip_1_4	82	peptidase inhibitor 16	271	8.28E-06	52.00%	0.106		IPR014044 (G3DSA:3.40.33.GENE3D); IPR018244 (PROSITE_PATTERNS); CYTOPLASMIC_DOMAIN (PHOBIUS); TRANSMEMBRANE (PHOBIUS); CYTOPLASMIC_DOMAIN (PHOBIUS); NON_CYTOPLASMIC_DOMAIN (PHOBIUS); TRANSMEMBRANE (PHOBIUS); TRANSMEMBRANE (PHOBIUS); NON_CYTOPLASMIC_DOMAIN (PHOBIUS); TMhelix (TMHMM); TMhelix (TMHMM)
1397	285030_length_813_cvg_3.4_tip_1_1	37	calponin homology domain containing	271	9.47E-67	58.60%	0.136		Coil (COILS); IPR001611 (PFAM); G3DSA:3.80.10.10 (GENE3D); PTHR23155 (PANTHER); PTHR23155:SF423 (PANTHER); IPR001611 (PROSITE_PROFILES); IPR001611 (PROSITE_PROFILES); SSF52075 (SUPERFAMILY)
1398	285036_length_813_cvg_5.0_tip_1_3	50	protein twisted gastrulation	271	7.73E-83	78.40%	0.142		IPR006761 (PFAM); PTHR12312:SF15 (PANTHER); IPR006761 (PANTHER); TRANSMEMBRANE (PHOBIUS); CYTOPLASMIC_DOMAIN (PHOBIUS); NON_CYTOPLASMIC_DOMAIN (PHOBIUS)
1399	285054_length_814_cvg_14.7_tip_1_0	112	abhydrolase domain-containing protein partial	272	4.73E-121	75.70%	0.137		IPR029059 (PFAM); IPR029058 (G3DSA:3.40.50.GENE3D); PTHR12277:SF44 (PANTHER); PTHR12277 (PANTHER); IPR029058 (SUPERFAMILY)
1400	285078_length_815_cvg_4.3_tip_1_0	44	apolipoprotein partial	272	2.31E-53	55.30%	0.103		PR00018 (PRINTS); IPR000001 (SMART); G3DSA:2.40.20.10 (GENE3D); IPR000001 (PFAM); PTHR24259 (PANTHER); IPR018056 (PROSITE_PATTERNS); IPR018056 (PROSITE_PATTERNS); IPR000001 (PROSITE_PROFILES); IPR000001 (PROSITE_PROFILES); IPR013806 (SUPERFAMILY); IPR013806 (SUPERFAMILY); IPR013806 (SUPERFAMILY)
1401	285114_length_815_cvg_9.4_tip_1_1	89	cysteine string protein	272	3.60E-100	75.80%	0.233		IPR001623 (PRINTS); IPR001623 (SMART); IPR001623 (G3DSA:1.10.287.GENE3D); IPR001623 (PFAM); PTHR24078:SF5 (PANTHER); PTHR24078 (PANTHER); IPR018253 (PROSITE_PATTERNS); IPR001623 (PROSITE_PROFILES); IPR001623 (SUPERFAMILY)
1402	285124_length_816_cvg_50.3_tip_1_5	873	Adrenodoxin, mitochondrial	272	2.53E-52	76.90%	0.123		IPR001055 (PRINTS); IPR001041 (PFAM); IPR012675 (G3DSA:3.10.20.GENE3D); PTHR23426 (PANTHER); PTHR23426:SF21 (PANTHER); IPR018298 (PROSITE_PATTERNS); IPR001041 (PROSITE_PROFILES); IPR001041 (SUPERFAMILY)
1403	285134_length_816_cvg_4.7_tip_1_4	53	carboxypeptidase m isoform x2	272	1.57E-41	69.50%	0.219		PF13620 (PFAM); IPR014766 (G3DSA:2.60.40.GENE3D); IPR027062 (PTHR11532:PANTHER); PTHR11532 (PANTHER); IPR008969 (SUPERFAMILY)
1404	285168_length_817_cvg_3.0_tip_1_4	39	zinc finger protein 350-like isoform x1	272	9.55E-46	79.80%	0.1		IPR015880 (SMART); PF13465 (PFAM); IPR007087 (PFAM); IPR013087 (G3DSA:3.30.160.GENE3D); IPR013087 (G3DSA:3.30.160.GENE3D); IPR013087 (G3DSA:3.30.160.GENE3D); IPR013087 (G3DSA:3.30.160.GENE3D); PTHR24409 (PANTHER); IPR007087 (PROSITE_PATTERNS); IPR007087 (PROSITE_PATTERNS); IPR007087 (PROSITE_PATTERNS); IPR007087 (PROSITE_PATTERNS); IPR007087 (PROSITE_PATTERNS); IPR007087 (PROSITE_PROFILES); IPR007087 (PROSITE_PROFILES); IPR007087 (PROSITE_PROFILES); IPR007087 (PROSITE_PROFILES); IPR007087 (PROSITE_PROFILES); IPR007087 (PROSITE_PROFILES); IPR007087 (PROSITE_PROFILES); SSF57667 (SUPERFAMILY); SSF57667 (SUPERFAMILY); SSF57667 (SUPERFAMILY); SSF57667 (SUPERFAMILY)
1405	285190_length_817_cvg_6.4_tip_1_4	88	gamma-interferon-inducible lysosomal thiol reductase	272	7.12E-44	51.30%	0.322		IPR004911 (PFAM); IPR004911 (PANTHER); PTHR13234:SF8 (PANTHER)

1406	285202_length_818_cvg_5.9_tip_1_2	52	lipophorin receptor	272	2.51E-74	63.30%	0.121		IPR000742 (SMART); IPR000033 (SMART); IPR001881 (SMART); IPR002172 (SMART); G3DSA:2.10.25.10 (GENE3D); IPR011042 (G3DSA:2.120.10.GENE3D); IPR002172 (PFAM); IPR000033 (PFAM); G3DSA:2.10.25.10 (GENE3D); IPR001881 (PFAM); IPR002172 (G3DSA:4.10.400.GENE3D); PTHR10529 (PANTHER); IPR023415 (PROSITE_PATTERNS); IPR018097 (PROSITE_PATTERNS); IPR000152 (PROSITE_PATTERNS); IPR000033 (PROSITE_PROFILES); IPR002172 (PROSITE_PROFILES); IPR000033 (PROSITE_PROFILES); IPR000033 (PROSITE_PROFILES); IPR002172 (SUPERFAMILY); SSF63825 (SUPERFAMILY); IPR009030 (SUPERFAMILY)
1407	285226_length_818_cvg_3.6_tip_1_4	53	zona pellucida-binding protein 2-like isoform x2	273	1.09E-07	49.00%	0.119		G3DSA:2.10.50.10 (GENE3D); IPR011641 (PFAM)
1408	285240_length_819_cvg_6.0_tip_1_3	66	insulin-like growth factor-binding protein 7 precursor	273	4.55E-74	58.90%	0.544	Y	IPR002350 (SMART); IPR003598 (SMART); IPR000867 (SMART); IPR000867 (PFAM); G3DSA:3.30.60.30 (GENE3D); IPR013783 (G3DSA:2.60.40.GENE3D); IPR002350 (PFAM); IPR013098 (PFAM); IPR011390 (PANTHER); PTHR14186:SF9 (PANTHER); SIGNAL_PEPTIDE_H_REGION (PHOBIUS); SIGNAL_PEPTIDE_N_REGION (PHOBIUS); SIGNAL_PEPTIDE (PHOBIUS); SIGNAL_PEPTIDE_C_REGION (PHOBIUS); NON_CYTOPLASMIC_DOMAIN (PHOBIUS); IPR000867 (PROSITE_PROFILES); IPR002350 (PROSITE_PROFILES); IPR007110 (PROSITE_PROFILES); SignalP-TM (SIGNALP_EUK); SSF48726 (SUPERFAMILY); IPR009030 (SUPERFAMILY); SSF100895 (SUPERFAMILY)
1409	285248_length_819_cvg_8.7_tip_1_4	99	trypsinogen 2	273	2.07E-93	67.20%	0.520	Y	IPR001314 (PRINTS); IPR001254 (SMART); G3DSA:2.40.10.10 (GENE3D); IPR001254 (PFAM); G3DSA:2.40.10.10 (GENE3D); G3DSA:2.40.10.10 (GENE3D); PTHR24265 (PANTHER); IPR018114 (PROSITE_PATTERNS); SIGNAL_PEPTIDE (PHOBIUS); NON_CYTOPLASMIC_DOMAIN (PHOBIUS); SIGNAL_PEPTIDE_C_REGION (PHOBIUS); SIGNAL_PEPTIDE_H_REGION (PHOBIUS); SIGNAL_PEPTIDE_N_REGION (PHOBIUS); IPR001254 (PROSITE_PROFILES); SignalP-noTM (SIGNALP_EUK); IPR009003 (SUPERFAMILY)
1410	285254_length_819_cvg_42.8_tip_1_3	373	kielin chordin-like protein	273	2.67E-18	40.00%	0.784	Y	IPR001846 (PFAM); G3DSA:2.10.70.10 (GENE3D); PTHR11339 (PANTHER); NON_CYTOPLASMIC_DOMAIN (PHOBIUS); SIGNAL_PEPTIDE_H_REGION (PHOBIUS); SIGNAL_PEPTIDE (PHOBIUS); SIGNAL_PEPTIDE_C_REGION (PHOBIUS); SIGNAL_PEPTIDE_N_REGION (PHOBIUS); IPR001846 (PROSITE_PROFILES); SignalP-noTM (SIGNALP_EUK); SignalP-noTM (SIGNALP_GRAM_NEGATIVE); SignalP-TM (SIGNALP_GRAM_POSITIVE); SSF57603 (SUPERFAMILY); TMhelix (TMHMM)
1411	285268_length_819_cvg_3.8_tip_1_3	51	multiple pdz domain	273	1.13E-89	67.70%	0.096		IPR001478 (SMART); IPR001478 (G3DSA:2.30.42.GENE3D); IPR001478 (G3DSA:2.30.42.GENE3D); IPR001478 (PFAM); PTHR19964 (PANTHER); TRANSMEMBRANE (PHOBIUS); CYTOPLASMIC_DOMAIN (PHOBIUS); NON_CYTOPLASMIC_DOMAIN (PHOBIUS); NON_CYTOPLASMIC_DOMAIN (PHOBIUS); TRANSMEMBRANE (PHOBIUS); IPR001478 (PROSITE_PROFILES); IPR001478 (PROSITE_PROFILES); IPR001478 (SUPERFAMILY); IPR001478 (SUPERFAMILY)
1412	285270_length_819_cvg_3.2_tip_1_4	33	carbonyl reductase	273	6.10E-93	74.50%	0.125		IPR002198 (PRINTS); IPR002347 (PRINTS); IPR016040 (G3DSA:3.40.50.GENE3D); IPR002198 (PFAM); PTHR24322:SF58 (PANTHER); PTHR24322 (PANTHER); SSF51735 (SUPERFAMILY)
1413	285286_length_820_cvg_16.7_tip_1_3	141	reelin- partial	274	5.10E-52	69.20%	0.107		PTHR11841 (PANTHER)
1415	285328_length_821_cvg_56.9_tip_1_0	1286	carboxypeptidase	274	2.39E-52	59.20%	0.25		IPR000834 (PRINTS); IPR000834 (SMART); IPR000834 (PFAM); G3DSA:3.40.630.10 (GENE3D); IPR003146 (PFAM); IPR003146 (G3DSA:3.30.70.GENE3D); PTHR11705 (PANTHER); IPR000834 (PROSITE_PATTERNS); SSF53187 (SUPERFAMILY); IPR009020 (SUPERFAMILY)
1416	285354_length_821_cvg_49.6_tip_1_0	444	phosphatidylinositol 4-phosphate 3-kinase c2 domain-containing subunit beta- partial	274	7.60E-17	43.00%	0.107		G3DSA:1.20.120.20 (GENE3D); SSF47162 (SUPERFAMILY)
1417	285402_length_822_cvg_6.0_tip_1_3	59	e3 ubiquitin-protein ligase mib2	274	1.75E-138	79.80%	0.102		IPR002110 (PRINTS); IPR002110 (SMART); IPR020683 (PFAM); IPR020683 (G3DSA:1.25.40.GENE3D); IPR020683 (G3DSA:1.25.40.GENE3D); PTHR24202 (PANTHER); PTHR24202:SF4 (PANTHER); IPR002110 (PROSITE_PROFILES); IPR002110 (PROSITE_PROFILES); IPR020683 (PROSITE_PROFILES); IPR002110 (PROSITE_PROFILES); IPR002110 (PROSITE_PROFILES); IPR020683 (SUPERFAMILY)

1418	285494_length_824_cvg_6.0_tip_1_1	62	probable cytosolic iron-sulfur protein assembly protein ciao1 isoform x1	275	3.51E-134	82.90%	0.101	IPR020472 (PRINTS); IPR001680 (SMART); IPR001680 (PFAM); IPR015943 (G3DSA:2.130.10.GENE3D); PTHR19920:SF0 (PANTHER); PTHR19920 (PANTHER); IPR019775 (PROSITE_PATTERNS); IPR001680 (PROSITE_PROFILES); IPR001680 (PROSITE_PROFILES); IPR001680 (PROSITE_PROFILES); IPR017986 (PROSITE_PROFILES); IPR001680 (PROSITE_PROFILES); IPR001680 (PROSITE_PROFILES); IPR017986 (SUPERFAMILY)
1419	285512_length_825_cvg_5.7_tip_1_4	58	alpha-mannosidase partial	275	3.15E-111	74.30%	0.101	G3DSA:2.70.98.30 (GENE3D); IPR011682 (PFAM); PTHR11607:SF22 (PANTHER); PTHR11607 (PANTHER); IPR011013 (SUPERFAMILY)
1420	285562_length_826_cvg_3.0_tip_1_1	37	aldehyde dehydrogenase family 9 member a1-b-like	275	5.35E-124	77.90%	0.122	IPR015590 (PFAM); IPR016163 (G3DSA:3.40.309.GENE3D); IPR016162 (G3DSA:3.40.605.GENE3D); PTHR11699:SF114 (PANTHER); PTHR11699 (PANTHER); IPR029510 (PROSITE_PATTERNS); IPR016160 (PROSITE_PATTERNS); IPR016161 (SUPERFAMILY)
1421	285566_length_826_cvg_4.5_tip_1_5	52	hook dna-binding motif-containing protein 1	275	5.79E-07	52.00%	0.098	no IPS match
1422	285582_length_827_cvg_3.2_tip_1_5	40	anti-lipopolysaccharide factor like protein	275	6.84E-15	57.50%	0.161	IPR024509 (PFAM); NON_CYTOPLASMIC_DOMAIN (PHOBIUS); CYTOPLASMIC_DOMAIN (PHOBIUS); TRANSMEMBRANE (PHOBIUS); NON_CYTOPLASMIC_DOMAIN (PHOBIUS); TRANSMEMBRANE (PHOBIUS); IPR024716 (PRODOM); TMhelix (TMHMM); TMhelix (TMHMM)
1423	285602_length_828_cvg_2.1_tip_1_3	27	adam 10	276	2.06E-145	83.60%	0.141	IPR024079 (G3DSA:3.40.390.GENE3D); PF13688 (PFAM); PTHR11905 (PANTHER); PTHR11905:SF4 (PANTHER); IPR001590 (PROSITE_PROFILES); SSF55486 (SUPERFAMILY)
1424	285638_length_828_cvg_3.7_tip_1_0	47	iduronate 2-sulfatase-like	276	2.72E-83	59.70%	0.118	G3DSA:3.30.1120.10 (GENE3D); IPR000917 (PFAM); IPR017849 (G3DSA:3.40.720.GENE3D); PTHR10342:SF185 (PANTHER); PTHR10342 (PANTHER); IPR017850 (SUPERFAMILY)
1425	285766_length_832_cvg_2.0_tip_1_5	36	iduronate 2- partial	277	1.70E-114	77.60%	0.630 Y	IPR017849 (G3DSA:3.40.720.GENE3D); IPR000917 (PFAM); PTHR10342:SF185 (PANTHER); PTHR10342 (PANTHER); IPR024607 (PROSITE_PATTERNS); SIGNAL_PEPTIDE_C_REGION (PHOBIUS); SIGNAL_PEPTIDE_N_REGION (PHOBIUS); SIGNAL_PEPTIDE (PHOBIUS); NON_CYTOPLASMIC_DOMAIN (PHOBIUS); SIGNAL_PEPTIDE_H_REGION (PHOBIUS); SignalP-noTM (SIGNALP_EUK); IPR017850 (SUPERFAMILY)
1426	285832_length_834_cvg_7.0_tip_1_1	62	maltase- intestinal-like	278	9.63E-61	58.30%	0.15	IPR000322 (PFAM); PTHR22762 (PANTHER); PTHR22762:SF7 (PANTHER); SSF51011 (SUPERFAMILY); IPR017853 (SUPERFAMILY)
1427	285844_length_834_cvg_10.5_tip_1_1	106	dnaj homolog subfamily c member 9	278	2.53E-89	75.10%	0.116	Coil (COILS); Coil (COILS); IPR001623 (PRINTS); IPR001623 (SMART); IPR001623 (PFAM); IPR001623 (G3DSA:1.10.287.GENE3D); PTHR24078 (PANTHER); PTHR24078:SF166 (PANTHER); IPR018253 (PROSITE_PATTERNS); IPR001623 (PROSITE_PROFILES); IPR001623 (SUPERFAMILY)
1428	285904_length_836_cvg_7.5_tip_1_0	92	cytolysin src-1-like isoform x2	279	2.05E-25	51.00%	0.114	IPR009104 (PFAM); IPR015926 (G3DSA:2.60.270.GENE3D); IPR015926 (SUPERFAMILY)
1429	285924_length_836_cvg_4.7_tip_1_0	43	alpha-mannosidase 2	279	9.21E-139	78.50%	0.102	IPR015341 (SMART); IPR000602 (PFAM); IPR015341 (PFAM); IPR015341 (G3DSA:1.20.1270.GENE3D); IPR027291 (G3DSA:3.20.110.GENE3D); PTHR11607 (PANTHER); PTHR11607:SF22 (PANTHER); IPR028995 (SUPERFAMILY); IPR011330 (SUPERFAMILY)
1430	285934_length_836_cvg_2.8_tip_1_1	28	lpxtg motif protein	279	1.68E-09	54.00%	0.174	Coil (COILS)
1431	285992_length_838_cvg_4.0_tip_1_1	45	low quality protein: mucin-19	279	7.12E-07	53.00%	0.142	CYTOPLASMIC_DOMAIN (PHOBIUS); CYTOPLASMIC_DOMAIN (PHOBIUS); TRANSMEMBRANE (PHOBIUS); TRANSMEMBRANE (PHOBIUS); NON_CYTOPLASMIC_DOMAIN (PHOBIUS); NON_CYTOPLASMIC_DOMAIN (PHOBIUS); TRANSMEMBRANE (PHOBIUS)
1432	285994_length_838_cvg_5.5_tip_1_4	51	ectonucleoside triphosphate diphosphohydrolase 5 isoform x2	279	5.84E-101	67.00%	0.168	IPR000407 (PFAM); PTHR11782:SF35 (PANTHER); IPR000407 (PANTHER)
1433	286034_length_839_cvg_3.0_tip_1_5	38	arylsulfatase a-like	279	7.51E-114	71.30%	0.686 Y	IPR017849 (G3DSA:3.40.720.GENE3D); IPR000917 (PFAM); PTHR10342 (PANTHER); PTHR10342:SF22 (PANTHER); IPR024607 (PROSITE_PATTERNS); SIGNAL_PEPTIDE_C_REGION (PHOBIUS); SIGNAL_PEPTIDE_H_REGION (PHOBIUS); NON_CYTOPLASMIC_DOMAIN (PHOBIUS); SIGNAL_PEPTIDE (PHOBIUS); SIGNAL_PEPTIDE_N_REGION (PHOBIUS); SignalP-noTM (SIGNALP_EUK); IPR017850 (SUPERFAMILY)
1434	286050_length_840_cvg_7.9_tip_1_0	103	macrophage mannose receptor 1-like	280	2.07E-26	48.90%	0.117	IPR001304 (SMART); IPR016186 (G3DSA:3.10.100.GENE3D); IPR001304 (PFAM); PTHR22803 (PANTHER); PTHR22803:SF16 (PANTHER); IPR018378 (PROSITE_PATTERNS); TRANSMEMBRANE (PHOBIUS); NON_CYTOPLASMIC_DOMAIN (PHOBIUS); CYTOPLASMIC_DOMAIN (PHOBIUS); IPR001304 (PROSITE_PROFILES); IPR016187 (SUPERFAMILY); TMhelix (TMHMM)
1435	286074_length_840_cvg_8.0_tip_1_5	67	flavin-containing monooxygenase fmo gs-ox-like 4	280	4.25E-102	66.20%	0.103	IPR000960 (PRINTS); G3DSA:3.50.50.60 (GENE3D); G3DSA:3.50.50.60 (GENE3D); IPR020946 (PFAM); PTHR23023:SF4 (PANTHER); PTHR23023 (PANTHER); SSF51905 (SUPERFAMILY); SSF51905 (SUPERFAMILY)

1436	286100_length_841_cvg_3.0_tip_1_1	35	zinc finger protein 665-like isoform x4	280	2.04E-47	54.20%	0.111		IPR015880 (SMART); IPR007087 (PFAM); IPR013087 (G3DSA:3.30.160.GENE3D); PF13912 (PFAM); IPR013087 (G3DSA:3.30.160.GENE3D); PF13894 (PFAM); PF13465 (PFAM); IPR013087 (G3DSA:3.30.160.GENE3D); PTHR24409 (PANTHER); IPR007087 (PROSITE_PATTERNS); IPR007087 (PROSITE_PATTERNS); IPR007087 (PROSITE_PATTERNS); IPR007087 (PROSITE_PATTERNS); IPR007087 (PROSITE_PATTERNS); IPR007087 (PROSITE_PATTERNS); IPR007087 (PROSITE_PATTERNS); IPR007087 (PROSITE_PROFILES); IPR007087 (PROSITE_PROFILES); IPR007087 (PROSITE_PROFILES); IPR007087 (PROSITE_PROFILES); IPR007087 (PROSITE_PROFILES); IPR007087 (PROSITE_PROFILES); IPR007087 (PROSITE_PROFILES); IPR007087 (PROSITE_PROFILES); SSF57667 (SUPERFAMILY); SSF57667 (SUPERFAMILY); SSF57667 (SUPERFAMILY); SSF57667 (SUPERFAMILY); SSF57667 (SUPERFAMILY)
1439	286152_length_842_cvg_8.0_tip_1_2	85	serine protease	280	8.10E-99	70.90%	0.171		IPR029058 (G3DSA:3.40.50.GENE3D); IPR008758 (PFAM); PTHR11010:SF9 (PANTHER); IPR008758 (PANTHER); CYTOPLASMIC_DOMAIN (PHOBIUS); NON_CYTOPLASMIC_DOMAIN (PHOBIUS); TRANSMEMBRANE (PHOBIUS); IPR029058 (SUPERFAMILY); TMhelix (TMHMM)
1437	286114_length_841_cvg_2.0_tip_1_0	20	zinc finger protein 570-like	281	1.27E-27	57.30%	0.132		IPR015880 (SMART); IPR013087 (G3DSA:3.30.160.GENE3D); IPR013087 (G3DSA:3.30.160.GENE3D); IPR013087 (G3DSA:3.30.160.GENE3D); IPR007087 (PFAM); PF13894 (PFAM); PF13465 (PFAM); PTHR24409:SF14 (PANTHER); PTHR24409 (PANTHER); IPR007087 (PROSITE_PATTERNS); IPR007087 (PROSITE_PATTERNS); IPR007087 (PROSITE_PATTERNS); IPR007087 (PROSITE_PATTERNS); IPR007087 (PROSITE_PROFILES); IPR007087 (PROSITE_PROFILES); IPR007087 (PROSITE_PROFILES); IPR007087 (PROSITE_PROFILES); IPR007087 (PROSITE_PROFILES); SSF57667 (SUPERFAMILY); SSF57667 (SUPERFAMILY); SSF57667 (SUPERFAMILY)
1438	286118_length_841_cvg_4.0_tip_1_0	49	neuroendocrine protein 7b2	281	4.16E-85	66.20%	0.32		Coil (COILS); IPR007945 (PFAM); IPR007945 (PANTHER); PTHR12738:SF0 (PANTHER); NON_CYTOPLASMIC_DOMAIN (PHOBIUS); CYTOPLASMIC_DOMAIN (PHOBIUS); TRANSMEMBRANE (PHOBIUS)
1440	286174_length_843_cvg_3.5_tip_1_2	45	calcium calmodulin-dependent protein kinase ii isoform a	281	0	96.60%	0.106		IPR002290 (SMART); G3DSA:1.10.510.10 (GENE3D); IPR016253 (PIRSF); G3DSA:3.30.200.20 (GENE3D); IPR000719 (PFAM); IPR020636 (PANTHER); PTHR24347:SF108 (PANTHER); IPR008271 (PROSITE_PATTERNS); IPR017441 (PROSITE_PATTERNS); TRANSMEMBRANE (PHOBIUS); CYTOPLASMIC_DOMAIN (PHOBIUS); NON_CYTOPLASMIC_DOMAIN (PHOBIUS); IPR000719 (PROSITE_PROFILES); IPR011009 (SUPERFAMILY)
1441	286230_length_844_cvg_8.0_tip_1_1	76	integrin-linked protein kinase	281	3.51E-171	88.00%	0.134		IPR020635 (SMART); IPR002110 (SMART); IPR020683 (G3DSA:1.25.40.GENE3D); G3DSA:3.30.200.20 (GENE3D); IPR016253 (PIRSF); IPR001245 (PFAM); G3DSA:1.10.510.10 (GENE3D); IPR020683 (PFAM); PTHR23257 (PANTHER); PTHR23257:SF30 (PANTHER); IPR000719 (PROSITE_PROFILES); IPR002110 (PROSITE_PROFILES); IPR020683 (PROSITE_PROFILES); IPR002110 (PROSITE_PROFILES); IPR020683 (SUPERFAMILY); IPR011009 (SUPERFAMILY)
1442	286266_length_845_cvg_56.6_tip_1_4	738	aael003561- partial	282	5.04E-26	49.90%	0.809	Y	IPR001211 (PRINTS); IPR016090 (SMART); IPR016090 (G3DSA:1.20.90.GENE3D); IPR016090 (PFAM); IPR001211 (PANTHER); IPR013090 (PROSITE_PATTERNS); SIGNAL_PEPTIDE_N_REGION (PHOBIUS); NON_CYTOPLASMIC_DOMAIN (PHOBIUS); SIGNAL_PEPTIDE_H_REGION (PHOBIUS); SIGNAL_PEPTIDE_C_REGION (PHOBIUS); SIGNAL_PEPTIDE (PHOBIUS); SignalP-noTM (SIGNALP_EUK); IPR016090 (SUPERFAMILY)
1443	286286_length_845_cvg_5.8_tip_1_1	57	protein o-linked-mannose beta- -n-acetylglucosaminyltransferase 1	282	5.22E-120	77.40%	0.11		IPR004139 (PANTHER); PTHR10468:SF4 (PANTHER); CYTOPLASMIC_DOMAIN (PHOBIUS); TRANSMEMBRANE (PHOBIUS); NON_CYTOPLASMIC_DOMAIN (PHOBIUS); TMhelix (TMHMM)
1444	286306_length_845_cvg_46.7_tip_1_3	528	phospholipase a2 isozymes pa3a pa3b pa5	282	2.09E-50	46.80%	0.463	Y	IPR016090 (G3DSA:1.20.90.GENE3D); IPR001211 (PFAM); PTHR12253 (PANTHER); PTHR12253:SF8 (PANTHER); SignalP-noTM (SIGNALP_EUK); IPR016090 (SUPERFAMILY)
1445	286312_length_846_cvg_9.9_tip_1_3	91	arylsulfatase b	282	4.60E-106	78.20%	0.199		IPR017849 (G3DSA:3.40.720.GENE3D); IPR000917 (PFAM); PTHR10342:SF189 (PANTHER); PTHR10342 (PANTHER); IPR024607 (PROSITE_PATTERNS); IPR024607 (PROSITE_PATTERNS); TRANSMEMBRANE (PHOBIUS); NON_CYTOPLASMIC_DOMAIN (PHOBIUS); TRANSMEMBRANE (PHOBIUS); CYTOPLASMIC_DOMAIN (PHOBIUS); NON_CYTOPLASMIC_DOMAIN (PHOBIUS); IPR017850 (SUPERFAMILY)
1446	286326_length_846_cvg_3.0_tip_1_2	43	tropinone reductase 2-like	282	2.07E-137	83.60%	0.169		IPR002347 (PRINTS); IPR002198 (PRINTS); IPR016040 (G3DSA:3.40.50.GENE3D); IPR002198 (PFAM); PTHR24322 (PANTHER); PTHR24322:SF111 (PANTHER); IPR020904 (PROSITE_PATTERNS); SSF51735 (SUPERFAMILY); TMhelix (TMHMM)

1447	286356_length_847_cvg_2.4_tip_1_2	34	pancreatic lipase-related protein	282	4.73E-29	57.40%	0.114	IPR013818 (PFAM); IPR029058 (G3DSA:3.40.50.GENE3D); IPR000734 (PANTHER); IPR029058 (SUPERFAMILY)
1448	286362_length_847_cvg_61.9_tip_1_5	4885	hemagglutinin amebocyte aggregation factor-like	282	8.79E-38	57.70%	0.152	PF14704 (PFAM); IPR026645 (PANTHER); TRANSMEMBRANE (PHOBIUS); NON_CYTOPLASMIC_DOMAIN (PHOBIUS); CYTOPLASMIC_DOMAIN (PHOBIUS); TMhelix (TMHMM)
1449	286658_length_855_cvg_4.0_tip_1_5	47	kielin130 protein	285	4.73E-13	46.00%	0.12	IPR001846 (PFAM); IPR001846 (PROSITE_PROFILES)
1451	286706_length_856_cvg_3.0_tip_1_1	41	carbonic anhydrase 1	285	3.31E-33	68.40%	0.129	IPR001148 (SMART); IPR001148 (G3DSA:3.10.200.GENE3D); IPR001148 (PFAM); IPR023561 (PANTHER); IPR001148 (PROSITE_PROFILES); IPR001148 (SUPERFAMILY)
1450	286686_length_856_cvg_3.2_tip_1_0	32	proteinase inhibitor i4 serpin	286	5.00E-11	53.30%	0.114	IPR023796 (PFAM); G3DSA:3.30.497.10 (GENE3D); IPR000215 (PANTHER); IPR023796 (SUPERFAMILY)
1452	286758_length_857_cvg_62.8_tip_1_4	834	cre-nas-7 protein	286	7.86E-34	53.90%	0.903 Y	IPR001506 (PRINTS); IPR006026 (SMART); IPR001506 (PFAM); IPR024079 (G3DSA:3.40.390.GENE3D); PTHR10127 (PANTHER); CYTOPLASMIC_DOMAIN (PHOBIUS); SIGNAL_PEPTIDE_C_REGION (PHOBIUS); SIGNAL_PEPTIDE_N_REGION (PHOBIUS); NON_CYTOPLASMIC_DOMAIN (PHOBIUS); TRANSMEMBRANE (PHOBIUS); SIGNAL_PEPTIDE_H_REGION (PHOBIUS); SIGNAL_PEPTIDE (PHOBIUS); SignalP-noTM (SIGNALP_EUK); SSF55486 (SUPERFAMILY); TMhelix (TMHMM)
1453	286908_length_861_cvg_4.0_tip_1_4	45	carboxypeptidase d-like	287	1.31E-101	69.40%	0.443 Y	Coil (COILS); IPR000834 (PRINTS); IPR000834 (SMART); G3DSA:3.40.630.10 (GENE3D); IPR000834 (PFAM); IPR015567 (PTHR11532:PANTHER); PTHR11532 (PANTHER); IPR000834 (PROSITE_PATTERNS); IPR000834 (PROSITE_PATTERNS); SIGNAL_PEPTIDE_C_REGION (PHOBIUS); NON_CYTOPLASMIC_DOMAIN (PHOBIUS); SIGNAL_PEPTIDE_H_REGION (PHOBIUS); SIGNAL_PEPTIDE_N_REGION (PHOBIUS); SIGNAL_PEPTIDE (PHOBIUS); SignalP-noTM (SIGNALP_GRAM_NEGATIVE); SSF53187 (SUPERFAMILY)
1455	286970_length_863_cvg_57.7_tip_1_5	1108	blastula protease 10-like	287	1.46E-30	56.20%	0.546 Y	IPR001506 (PRINTS); IPR006026 (SMART); IPR001506 (PFAM); IPR024079 (G3DSA:3.40.390.GENE3D); PTHR10127:SF580 (PANTHER); PTHR10127 (PANTHER); SIGNAL_PEPTIDE_N_REGION (PHOBIUS); SIGNAL_PEPTIDE_C_REGION (PHOBIUS); NON_CYTOPLASMIC_DOMAIN (PHOBIUS); SIGNAL_PEPTIDE (PHOBIUS); SIGNAL_PEPTIDE_H_REGION (PHOBIUS); SignalP-noTM (SIGNALP_EUK); SSF55486 (SUPERFAMILY); TMhelix (TMHMM)
1454	286934_length_862_cvg_4.3_tip_1_3	40	aael005189- partial	288	9.88E-32	74.00%	0.108	IPR003112 (PFAM); PTHR23192 (PANTHER); PTHR23192:SF33 (PANTHER); IPR003112 (PROSITE_PROFILES)
1456	286996_length_864_cvg_6.0_tip_1_2	57	collagen alpha 1 chain	288	4.51E-50	69.30%	0.114	IPR010515 (PFAM); IPR016186 (G3DSA:3.10.100.GENE3D); IPR026917 (PTHR24023:PANTHER); PTHR24023 (PANTHER); CYTOPLASMIC_DOMAIN (PHOBIUS); TRANSMEMBRANE (PHOBIUS); NON_CYTOPLASMIC_DOMAIN (PHOBIUS); IPR016187 (SUPERFAMILY)
1485	288156_length_900_cvg_7.8_tip_1_1	75	peptidyl-alpha-hydroxyglycine alpha-amidating lyase 2-like	288	5.44E-31	60.60%	0.139	IPR001258 (PFAM); IPR011042 (G3DSA:2.120.10.GENE3D); PTHR10680 (PANTHER); SSF63825 (SUPERFAMILY)
1457	287060_length_866_cvg_54.2_tip_1_1	619	trypsinogen 2	289	3.18E-70	59.00%	0.882 Y	IPR001314 (PRINTS); IPR001254 (SMART); G3DSA:2.40.10.10 (GENE3D); G3DSA:2.40.10.10 (GENE3D); G3DSA:2.40.10.10 (GENE3D); IPR001254 (PFAM); PTHR24265 (PANTHER); IPR018114 (PROSITE_PATTERNS); IPR018114 (PROSITE_PATTERNS); SIGNAL_PEPTIDE_N_REGION (PHOBIUS); NON_CYTOPLASMIC_DOMAIN (PHOBIUS); SIGNAL_PEPTIDE_C_REGION (PHOBIUS); SIGNAL_PEPTIDE_H_REGION (PHOBIUS); SIGNAL_PEPTIDE (PHOBIUS); IPR001254 (PROSITE_PROFILES); SignalP-noTM (SIGNALP_EUK); SignalP-noTM (SIGNALP_GRAM_NEGATIVE); SignalP-TM (SIGNALP_GRAM_POSITIVE); IPR009003 (SUPERFAMILY)
1458	287066_length_866_cvg_3.0_tip_1_1	31	omega-amidase nit2-like	289	7.19E-142	83.20%	0.101	IPR003010 (PFAM); IPR003010 (G3DSA:3.60.110.GENE3D); PTHR23088:SF20 (PANTHER); PTHR23088 (PANTHER); IPR003010 (PROSITE_PROFILES); IPR003010 (SUPERFAMILY)
1459	287076_length_867_cvg_32.0_tip_1_1	334	sparc	289	7.39E-112	78.70%	0.109	Coil (COILS); Coil (COILS); IPR003645 (SMART); IPR011992 (G3DSA:1.10.238.GENE3D); G3DSA:3.30.60.30 (GENE3D); IPR019577 (PFAM); PTHR13866:SF14 (PANTHER); PTHR13866 (PANTHER); IPR001999 (PROSITE_PATTERNS); IPR018247 (PROSITE_PATTERNS); SSF47473 (SUPERFAMILY); SSF100895 (SUPERFAMILY)
1460	287102_length_867_cvg_12.9_tip_1_4	142	u8-agatoxin-ao1a-like isoform x2	289	3.62E-35	73.00%	0.702 Y	IPR004169 (PFAM); SIGNAL_PEPTIDE_N_REGION (PHOBIUS); SIGNAL_PEPTIDE_H_REGION (PHOBIUS); CYTOPLASMIC_DOMAIN (PHOBIUS); NON_CYTOPLASMIC_DOMAIN (PHOBIUS); TRANSMEMBRANE (PHOBIUS); TRANSMEMBRANE (PHOBIUS); SIGNAL_PEPTIDE (PHOBIUS); NON_CYTOPLASMIC_DOMAIN (PHOBIUS); SIGNAL_PEPTIDE_C_REGION (PHOBIUS); SignalP-TM (SIGNALP_EUK); SignalP-TM (SIGNALP_GRAM_NEGATIVE); SSF57059 (SUPERFAMILY); TMhelix (TMHMM); TMhelix (TMHMM)

1461	287158_length_869_cvg_67.3_tip_1_4	7534	zinc metalloproteinase nas-13	290	2.73E-63	58.30%	0.797 Y	IPR001506 (PRINTS); IPR006026 (SMART); IPR001506 (PFAM); IPR024079 (G3DSA:3.40.390.GENE3D); PTHR10127 (PANTHER); NON_CYTOPLASMIC_DOMAIN (PHOBIUS); SIGNAL_PEPTIDE_N_REGION (PHOBIUS); SIGNAL_PEPTIDE_H_REGION (PHOBIUS); SIGNAL_PEPTIDE_C_REGION (PHOBIUS); SIGNAL_PEPTIDE (PHOBIUS); SignalP-noTM (SIGNALP_EUK); SSF55486 (SUPERFAMILY)
1462	287202_length_870_cvg_5.8_tip_1_1	68	coagulation factor x-like	290	8.91E-28	57.20%	0.138	IPR001314 (PRINTS); IPR001254 (SMART); IPR002172 (SMART); IPR001254 (PFAM); G3DSA:2.40.10.10 (GENE3D); G3DSA:2.40.10.10 (GENE3D); IPR002172 (PFAM); IPR002172 (G3DSA:4.10.400.GENE3D); IPR002172 (G3DSA:4.10.400.GENE3D); PTHR24265 (PANTHER); IPR002172 (PROSITE_PROFILES); IPR001254 (PROSITE_PROFILES); IPR003609 (PROSITE_PROFILES); IPR009003 (SUPERFAMILY); IPR002172 (SUPERFAMILY)
1463	287228_length_871_cvg_3.0_tip_1_0	35	chorion peroxidase-like	291	4.52E-120	67.60%	0.119	IPR019791 (PRINTS); IPR019791 (PFAM); IPR019791 (G3DSA:1.10.640.GENE3D); PTHR11475 (PANTHER); IPR019791 (PROSITE_PROFILES); IPR010255 (SUPERFAMILY)
1464	287280_length_872_cvg_9.8_tip_1_4	88	secreted partial	291	1.76E-45	75.60%	0.107	IPR003585 (SMART); IPR027789 (PFAM); PTHR10915:SF1 (PANTHER); IPR001050 (PANTHER); IPR030479 (PROSITE_PATTERNS); CYTOPLASMIC_DOMAIN (PHOBIUS); TRANSMEMBRANE (PHOBIUS); NON_CYTOPLASMIC_DOMAIN (PHOBIUS); TMhelix (TMHMM)
1465	287308_length_873_cvg_36.8_tip_1_4	488	histone -like	291	3.26E-81	99.20%	0.105	IPR002119 (PRINTS); IPR002119 (SMART); IPR009072 (G3DSA:1.10.20.GENE3D); IPR007125 (PFAM); PTHR23430 (PANTHER); PTHR23430:SF34 (PANTHER); IPR002119 (PROSITE_PATTERNS); IPR009072 (SUPERFAMILY)
1466	287358_length_875_cvg_5.3_tip_1_0	52	probable isocitrate dehydrogenase	292	8.62E-161	87.40%	0.104	IPR024084 (G3DSA:3.40.718.GENE3D); IPR024084 (PFAM); PTHR11835:SF39 (PANTHER); IPR001804 (PANTHER); IPR019818 (PROSITE_PATTERNS); SSF53659 (SUPERFAMILY)
1467	287400_length_876_cvg_6.2_tip_1_4	59	copper chaperone for superoxide dismutase	292	3.46E-80	73.20%	0.169	IPR001424 (PRINTS); IPR001424 (PFAM); IPR001424 (G3DSA:2.60.40.GENE3D); IPR006121 (PFAM); G3DSA:3.30.70.100 (GENE3D); PTHR10003 (PANTHER); IPR006121 (PROSITE_PROFILES); IPR001424 (SUPERFAMILY); IPR006121 (SUPERFAMILY)
1468	287422_length_877_cvg_6.3_tip_1_4	67	macrophage mannose receptor partial	292	8.91E-27	47.50%	0.14	IPR001304 (SMART); IPR016186 (G3DSA:3.10.100.GENE3D); IPR001304 (PFAM); IPR016186 (G3DSA:3.10.100.GENE3D); PTHR22803 (PANTHER); PTHR22803:SF16 (PANTHER); IPR001304 (PROSITE_PROFILES); IPR001304 (PROSITE_PROFILES); IPR001304 (PROSITE_PROFILES); IPR016187 (SUPERFAMILY); IPR016187 (SUPERFAMILY); IPR016187 (SUPERFAMILY)
1469	287454_length_878_cvg_54.2_tip_1_5	1064	cathepsin l	292	1.44E-130	79.00%	0.15	IPR000668 (SMART); IPR013201 (SMART); IPR000668 (PFAM); G3DSA:3.90.70.10 (GENE3D); IPR013201 (PFAM); IPR013128 (PANTHER); PTHR12411:SF57 (PANTHER); IPR000169 (PROSITE_PATTERNS); CYTOPLASMIC_DOMAIN (PHOBIUS); TRANSMEMBRANE (PHOBIUS); NON_CYTOPLASMIC_DOMAIN (PHOBIUS); CYTOPLASMIC_DOMAIN (PHOBIUS); TRANSMEMBRANE (PHOBIUS); SSF54001 (SUPERFAMILY); TMhelix (TMHMM)
1470	287522_length_879_cvg_11.9_tip_1_0	126	protein shifted isoform x1	293	1.11E-80	87.70%	0.143	IPR013309 (PRINTS); IPR003306 (SMART); IPR003306 (PFAM); PTHR24838:SF276 (PANTHER); PTHR24838 (PANTHER); CYTOPLASMIC_DOMAIN (PHOBIUS); NON_CYTOPLASMIC_DOMAIN (PHOBIUS); TRANSMEMBRANE (PHOBIUS); NON_CYTOPLASMIC_DOMAIN (PHOBIUS); TRANSMEMBRANE (PHOBIUS); IPR003306 (PRODOM); IPR003306 (PROSITE_PROFILES); TMhelix (TMHMM)
1471	287570_length_881_cvg_13.4_tip_1_1	152	lipophorin receptor isoform e	294	4.71E-13	53.10%	0.786 Y	IPR002172 (PRINTS); IPR002172 (SMART); IPR002172 (G3DSA:4.10.400.GENE3D); IPR002172 (PFAM); IPR002172 (G3DSA:4.10.400.GENE3D); PTHR10529:SF208 (PANTHER); PTHR10529 (PANTHER); IPR023415 (PROSITE_PATTERNS); SIGNAL_PEPTIDE_C_REGION (PHOBIUS); SIGNAL_PEPTIDE (PHOBIUS); SIGNAL_PEPTIDE_H_REGION (PHOBIUS); NON_CYTOPLASMIC_DOMAIN (PHOBIUS); SIGNAL_PEPTIDE_N_REGION (PHOBIUS); IPR002172 (PROSITE_PROFILES); IPR002172 (PROSITE_PROFILES); SignalP-noTM (SIGNALP_EUK); IPR002172 (SUPERFAMILY)
1472	287586_length_881_cvg_27.1_tip_1_3	351	polyubiquitin-c-like isoform 2	294	1.82E-107	98.90%	0.181	IPR019956 (PRINTS); IPR000626 (SMART); G3DSA:3.10.20.90 (GENE3D); IPR000626 (PFAM); PTHR10666 (PANTHER); IPR019954 (PROSITE_PATTERNS); IPR019954 (PROSITE_PATTERNS); CYTOPLASMIC_DOMAIN (PHOBIUS); NON_CYTOPLASMIC_DOMAIN (PHOBIUS); TRANSMEMBRANE (PHOBIUS); IPR000626 (PROSITE_PROFILES); IPR000626 (PROSITE_PROFILES); IPR029071 (SUPERFAMILY); IPR029071 (SUPERFAMILY)

1473	287626_length_882_cvg_7.2_tip_1_4	90	low-density lipoprotein receptor-related protein 2-like	294	1.05E-24	52.60%	0.11	IPR002172 (PRINTS); IPR002172 (SMART); IPR002172 (G3DSA:4.10.400.GENE3D); IPR002172 (G3DSA:4.10.400.GENE3D); IPR002172 (G3DSA:4.10.400.GENE3D); IPR002172 (PFAM); PTHR10529 (PANTHER); PTHR10529:SF204 (PANTHER); IPR023415 (PROSITE_PATTERNS); IPR023415 (PROSITE_PATTERNS); IPR023415 (PROSITE_PATTERNS); IPR002172 (PROSITE_PROFILES); IPR002172 (PROSITE_PROFILES); IPR002172 (PROSITE_PROFILES); IPR002172 (PROSITE_PROFILES); IPR002172 (SUPERFAMILY); IPR002172 (SUPERFAMILY); IPR002172 (SUPERFAMILY); IPR002172 (SUPERFAMILY)
1474	287636_length_882_cvg_4.4_tip_1_4	49	zinc finger protein 239-like isoform x2	294	4.45E-80	75.30%	0.108	IPR015880 (SMART); PF13465 (PFAM); IPR013087 (G3DSA:3.30.160.GENE3D); IPR013087 (G3DSA:3.30.160.GENE3D); IPR013087 (G3DSA:3.30.160.GENE3D); PTHR24377 (PANTHER); IPR007087 (PROSITE_PATTERNS); IPR007087 (PROSITE_PATTERNS); IPR007087 (PROSITE_PATTERNS); IPR007087 (PROSITE_PATTERNS); IPR007087 (PROSITE_PATTERNS); IPR007087 (PROSITE_PATTERNS); IPR007087 (PROSITE_PATTERNS); NON_CYTOPLASMIC_DOMAIN (PHOBIUS); NON_CYTOPLASMIC_DOMAIN (PHOBIUS); TRANSMEMBRANE (PHOBIUS); TRANSMEMBRANE (PHOBIUS); CYTOPLASMIC_DOMAIN (PHOBIUS); IPR007087 (PROSITE_PROFILES); IPR007087 (PROSITE_PROFILES); IPR007087 (PROSITE_PROFILES); IPR007087 (PROSITE_PROFILES); IPR007087 (PROSITE_PROFILES); IPR007087 (PROSITE_PROFILES); IPR007087 (PROSITE_PROFILES); SSF57667 (SUPERFAMILY); SSF57667 (SUPERFAMILY); SSF57667 (SUPERFAMILY); SSF57667 (SUPERFAMILY)
1475	287642_length_883_cvg_7.4_tip_1_1	66	protein disulfide-isomerase a6	294	2.25E-41	88.20%	0.101	PTHR18929 (PANTHER); PTHR18929:SF38 (PANTHER)
1476	287786_length_887_cvg_22.7_tip_1_4	216	slit homolog 2 protein isoform x1	296	7.79E-08	44.00%	0.450 Y	IPR000742 (SMART); G3DSA:2.10.25.10 (GENE3D); IPR000742 (PFAM); G3DSA:2.10.25.10 (GENE3D); G3DSA:2.10.25.10 (GENE3D); IPR000742 (PROSITE_PROFILES); IPR000742 (PROSITE_PROFILES)
1477	287862_length_890_cvg_58.5_tip_1_2	2142	anti-lipopolysaccharide factor	296	7.40E-33	68.50%	0.832 Y	IPR024509 (PFAM); SIGNAL_PEPTIDE_N_REGION (PHOBIUS); TRANSMEMBRANE (PHOBIUS); CYTOPLASMIC_DOMAIN (PHOBIUS); SIGNAL_PEPTIDE_C_REGION (PHOBIUS); NON_CYTOPLASMIC_DOMAIN (PHOBIUS); SIGNAL_PEPTIDE_H_REGION (PHOBIUS); SIGNAL_PEPTIDE (PHOBIUS); IPR024716 (PRODOM); SignalP-noTM (SIGNALP_EUK); SignalP-noTM (SIGNALP_GRAM_NEGATIVE); TMhelix (TMHMM); TMhelix (TMHMM)
1478	287884_length_891_cvg_6.5_tip_1_4	71	exocyst complex component 7	297	2.78E-139	74.70%	0.109	IPR004140 (PFAM); IPR004140 (PANTHER); PTHR12542:SF7 (PANTHER); IPR016159 (SUPERFAMILY)
1479	287898_length_891_cvg_7.1_tip_1_1	83	von willebrand factor type egf and pentraxin domain-containing protein 1	297	1.07E-40	45.00%	0.153	IPR000436 (SMART); IPR000436 (PFAM); G3DSA:2.10.70.10 (GENE3D); PTHR19325:SF330 (PANTHER); PTHR19325 (PANTHER); CYTOPLASMIC_DOMAIN (PHOBIUS); TRANSMEMBRANE (PHOBIUS); NON_CYTOPLASMIC_DOMAIN (PHOBIUS); IPR000436 (PROSITE_PROFILES); IPR000436 (PROSITE_PROFILES); IPR000436 (SUPERFAMILY); TMhelix (TMHMM)
1480	287920_length_892_cvg_32.8_tip_1_5	324	neuferricin -like protein	297	4.46E-74	66.90%	0.211	IPR001199 (PFAM); IPR001199 (G3DSA:3.10.120.GENE3D); PTHR10281:SF4 (PANTHER); PTHR10281 (PANTHER); CYTOPLASMIC_DOMAIN (PHOBIUS); TRANSMEMBRANE (PHOBIUS); NON_CYTOPLASMIC_DOMAIN (PHOBIUS); IPR001199 (SUPERFAMILY); TMhelix (TMHMM)
1481	287934_length_892_cvg_5.9_tip_1_1	56	serine proteinase	297	1.30E-36	51.80%	0.219	IPR001314 (PRINTS); IPR001254 (SMART); IPR001254 (PFAM); G3DSA:2.40.10.10 (GENE3D); G3DSA:2.40.10.10 (GENE3D); PTHR24260 (PANTHER); IPR018114 (PROSITE_PATTERNS); TRANSMEMBRANE (PHOBIUS); NON_CYTOPLASMIC_DOMAIN (PHOBIUS); NON_CYTOPLASMIC_DOMAIN (PHOBIUS); CYTOPLASMIC_DOMAIN (PHOBIUS); TRANSMEMBRANE (PHOBIUS); IPR001254 (PROSITE_PROFILES); IPR009003 (SUPERFAMILY)
1482	288010_length_895_cvg_2.7_tip_1_5	38	carboxylesterase 4a	298	6.57E-58	63.40%	0.213	IPR029058 (G3DSA:3.40.50.GENE3D); IPR002018 (PFAM); PTHR11559 (PANTHER); IPR019819 (PROSITE_PATTERNS); TRANSMEMBRANE (PHOBIUS); CYTOPLASMIC_DOMAIN (PHOBIUS); TRANSMEMBRANE (PHOBIUS); NON_CYTOPLASMIC_DOMAIN (PHOBIUS); CYTOPLASMIC_DOMAIN (PHOBIUS); IPR029058 (SUPERFAMILY)
1483	288054_length_897_cvg_5.5_tip_1_2	68	venom carboxylesterase-6-like	299	3.78E-41	53.20%	0.102	IPR002018 (PFAM); IPR029058 (G3DSA:3.40.50.GENE3D); PTHR11559 (PANTHER); IPR029058 (SUPERFAMILY)
1484	288068_length_897_cvg_5.9_tip_1_1	58	syntaxin-12	299	4.31E-55	72.50%	0.104	IPR000727 (SMART); PF14523 (PFAM); IPR000727 (PFAM); G3DSA:1.20.5.110 (GENE3D); PTHR19957:SF87 (PANTHER); PTHR19957 (PANTHER); IPR006012 (PROSITE_PATTERNS); TRANSMEMBRANE (PHOBIUS); CYTOPLASMIC_DOMAIN (PHOBIUS); NON_CYTOPLASMIC_DOMAIN (PHOBIUS); CYTOPLASMIC_DOMAIN (PHOBIUS); TRANSMEMBRANE (PHOBIUS); IPR000727 (PROSITE_PROFILES); IPR010989 (SUPERFAMILY); TMhelix (TMHMM); TMhelix (TMHMM); TMhelix (TMHMM)

1486	288312_length_906_cvg_5.2_tip_1_5	58	leucine-rich repeat-containing protein 15-like isoform x2	302	1.82E-56	59.80%	0.104		Coil (COILS); IPR003591 (SMART); G3DSA:3.80.10.10 (GENE3D); IPR001611 (PFAM); PTHR24373 (PANTHER); IPR001611 (PROSITE_PROFILES); IPR001611 (PROSITE_PROFILES); IPR001611 (PROSITE_PROFILES); SSF52058 (SUPERFAMILY)
1487	288352_length_907_cvg_8.8_tip_1_4	109	---NA---	302			0.123		CYTOPLASMIC_DOMAIN (PHOBIUS); TRANSMEMBRANE (PHOBIUS); NON_CYTOPLASMIC_DOMAIN (PHOBIUS)
1488	288372_length_907_cvg_6.2_tip_1_1	63	collagen alpha-1 chain	302	1.73E-44	55.10%	0.099		IPR008160 (PFAM); PTHR24023:SF397 (PANTHER); PTHR24023 (PANTHER)
1489	288372_length_907_cvg_6.2_tip_1_4	63	mucin-2-like isoform x2	302	2.20E-38	44.40%	0.137		PTHR24023:SF405 (PANTHER); PTHR24023 (PANTHER)
1490	288390_length_908_cvg_79.6_tip_0_4	3230	muscle m-line assembly protein unc-89-like	303	1.20E-80	75.70%	0.101		Coil (COILS); IPR003599 (SMART); IPR013783 (G3DSA:2.60.40.GENE3D); IPR013783 (G3DSA:2.60.40.GENE3D); IPR013783 (G3DSA:2.60.40.GENE3D); IPR013098 (PFAM); PTHR19897:SF148 (PANTHER); PTHR19897 (PANTHER); IPR007110 (PROSITE_PROFILES); SSF48726 (SUPERFAMILY); SSF48726 (SUPERFAMILY); SSF48726 (SUPERFAMILY)
1491	288392_length_908_cvg_31.4_tip_1_1	336	cell recognition protein caspr4 isoform 5	303	2.92E-45	52.10%	0.139		IPR001073 (PFAM); IPR008983 (G3DSA:2.60.120.GENE3D); PTHR10127 (PANTHER); PTHR10127:SF575 (PANTHER); IPR001073 (PROSITE_PROFILES); IPR002181 (SUPERFAMILY); IPR008983 (SUPERFAMILY)
1492	288400_length_908_cvg_12.5_tip_1_3	142	glycosyl-phosphatidylinositol-linked carbonic anhydrase	303	4.63E-54	67.90%	0.11		IPR001148 (SMART); IPR001148 (PFAM); IPR001148 (G3DSA:3.10.200.GENE3D); IPR023561 (PANTHER); IPR018338 (PROSITE_PATTERNS); IPR001148 (PROSITE_PROFILES); IPR001148 (SUPERFAMILY)
1493	288416_length_908_cvg_12.8_tip_1_4	149	striatin- partial	303	3.52E-153	82.20%	0.104		IPR020472 (PRINTS); IPR001680 (SMART); IPR001680 (PFAM); IPR015943 (G3DSA:2.130.10.GENE3D); PTHR15653 (PANTHER); IPR019775 (PROSITE_PATTERNS); IPR017986 (PROSITE_PROFILES); IPR001680 (PROSITE_PROFILES); IPR001680 (PROSITE_PROFILES); IPR001680 (PROSITE_PROFILES); IPR017986 (SUPERFAMILY)
1494	288488_length_912_cvg_13.0_tip_1_5	139	cytochrome c oxidase assembly factor 7 homolog	304	3.49E-72	65.80%	0.097		Coil (COILS); IPR006597 (SMART); IPR006597 (PFAM); IPR011990 (G3DSA:1.25.40.GENE3D); PTHR13891:SF1 (PANTHER); PTHR13891 (PANTHER); SSF81901 (SUPERFAMILY)
1495	288508_length_912_cvg_74.6_tip_1_1	9441	trypsinogen 2	304	2.00E-72	62.30%	0.339		IPR001314 (PRINTS); IPR001254 (SMART); G3DSA:2.40.10.10 (GENE3D); G3DSA:2.40.10.10 (GENE3D); IPR001254 (PFAM); G3DSA:2.40.10.10 (GENE3D); PTHR24265 (PANTHER); IPR001254 (PROSITE_PROFILES); SignalP-TM (SIGNALP_GRAM_POSITIVE); IPR009003 (SUPERFAMILY)
1496	288512_length_912_cvg_4.6_tip_1_0	54	isoform cra_a	304	3.65E-14	52.90%	0.203		no IPS match
1497	288512_length_912_cvg_4.6_tip_1_3	54	coagulation factor v	304	1.57E-17	49.00%	0.145		TRANSMEMBRANE (PHOBIUS); TRANSMEMBRANE (PHOBIUS); TRANSMEMBRANE (PHOBIUS); TRANSMEMBRANE (PHOBIUS); NON_CYTOPLASMIC_DOMAIN (PHOBIUS); TRANSMEMBRANE (PHOBIUS); CYTOPLASMIC_DOMAIN (PHOBIUS); NON_CYTOPLASMIC_DOMAIN (PHOBIUS); CYTOPLASMIC_DOMAIN (PHOBIUS); NON_CYTOPLASMIC_DOMAIN (PHOBIUS); NON_CYTOPLASMIC_DOMAIN (PHOBIUS); TRANSMEMBRANE (PHOBIUS); CYTOPLASMIC_DOMAIN (PHOBIUS); TMhelix (TMHMM); TMhelix (TMHMM); TMhelix (TMHMM); TMhelix (TMHMM); TMhelix (TMHMM)
1498	288512_length_912_cvg_4.6_tip_1_4	54	coagulation factor v	304	7.65E-19	44.10%	0.167		NON_CYTOPLASMIC_DOMAIN (PHOBIUS); CYTOPLASMIC_DOMAIN (PHOBIUS); TRANSMEMBRANE (PHOBIUS); NON_CYTOPLASMIC_DOMAIN (PHOBIUS); TRANSMEMBRANE (PHOBIUS); NON_CYTOPLASMIC_DOMAIN (PHOBIUS); TRANSMEMBRANE (PHOBIUS); CYTOPLASMIC_DOMAIN (PHOBIUS); TRANSMEMBRANE (PHOBIUS); TRANSMEMBRANE (PHOBIUS); CYTOPLASMIC_DOMAIN (PHOBIUS); TMhelix (TMHMM); TMhelix (TMHMM); TMhelix (TMHMM)
1499	288512_length_912_cvg_4.6_tip_1_5	54	coagulation factor v	304	2.51E-10	45.70%	0.506	Y	CYTOPLASMIC_DOMAIN (PHOBIUS); TRANSMEMBRANE (PHOBIUS); NON_CYTOPLASMIC_DOMAIN (PHOBIUS); TRANSMEMBRANE (PHOBIUS); SIGNAL_PEPTIDE (PHOBIUS); NON_CYTOPLASMIC_DOMAIN (PHOBIUS); SIGNAL_PEPTIDE_H_REGION (PHOBIUS); SIGNAL_PEPTIDE_N_REGION (PHOBIUS); SIGNAL_PEPTIDE_C_REGION (PHOBIUS); CYTOPLASMIC_DOMAIN (PHOBIUS); TRANSMEMBRANE (PHOBIUS); SignalP-TM (SIGNALP_EUK)
1500	288520_length_913_cvg_27.3_tip_1_5	249	aldo-keto reductase family 1 member b10	304	2.54E-127	76.80%	0.099		IPR020471 (PRINTS); IPR023210 (G3DSA:3.20.20.GENE3D); IPR023210 (PFAM); IPR020471 (PIRSF); PTHR11732:SF202 (PANTHER); IPR001395 (PANTHER); IPR018170 (PROSITE_PATTERNS); IPR018170 (PROSITE_PATTERNS); IPR023210 (SUPERFAMILY)

1501	288536_length_913_cvg_2.7_tip_1_3	38	very low-density lipoprotein receptor	305	6.24E-18	42.00%	0.114	IPR002172 (PRINTS); IPR002172 (SMART); IPR002172 (G3DSA:4.10.400.GENE3D); IPR002172 (PFAM); IPR002172 (G3DSA:4.10.400.GENE3D); PTHR10529 (PANTHER); IPR023415 (PROSITE_PATTERNS); IPR023415 (PROSITE_PATTERNS); IPR023415 (PROSITE_PATTERNS); IPR002172 (PROSITE_PROFILES); IPR002172 (PROSITE_PROFILES); IPR002172 (PROSITE_PROFILES); IPR002172 (SUPERFAMILY); IPR002172 (SUPERFAMILY); IPR002172 (SUPERFAMILY)
1502	288570_length_914_cvg_10.9_tip_1_3	94	adp-ribosylation factor 6	305	7.15E-124	99.60%	0.535 Y	IPR006689 (PRINTS); IPR003579 (SMART); IPR006687 (SMART); IPR024156 (SMART); IPR005225 (TIGRFAM); IPR027417 (G3DSA:3.40.50.GENE3D); IPR006689 (PFAM); PTHR11711:SF106 (PANTHER); PTHR11711 (PANTHER); IPR024156 (PROSITE_PROFILES); SignalP-noTM (SIGNALP_EUK); IPR027417 (SUPERFAMILY)
1503	288726_length_920_cvg_6.8_tip_1_2	76	fasciclin-2 isoform x6	306	1.34E-14	55.80%	0.113	IPR003599 (SMART); IPR003598 (SMART); IPR013783 (G3DSA:2.60.40.GENE3D); IPR013098 (PFAM); PF13895 (PFAM); PTHR19831 (PANTHER); NON_CYTOPLASMIC_DOMAIN (PHOBIUS); CYTOPLASMIC_DOMAIN (PHOBIUS); TRANSMEMBRANE (PHOBIUS); IPR007110 (PROSITE_PROFILES); IPR007110 (PROSITE_PROFILES); SSF48726 (SUPERFAMILY); SSF48726 (SUPERFAMILY); TMhelix (TMHMM)
1504	288742_length_920_cvg_12.8_tip_1_2	460	hemagglutinin amebocyte aggregation factor-like	306	1.63E-08	69.20%	0.105	Coil (COILS); PF14704 (PFAM); IPR026645 (PANTHER); CYTOPLASMIC_DOMAIN (PHOBIUS); TRANSMEMBRANE (PHOBIUS); TRANSMEMBRANE (PHOBIUS); NON_CYTOPLASMIC_DOMAIN (PHOBIUS); TRANSMEMBRANE (PHOBIUS); TRANSMEMBRANE (PHOBIUS); NON_CYTOPLASMIC_DOMAIN (PHOBIUS); TRANSMEMBRANE (PHOBIUS); CYTOPLASMIC_DOMAIN (PHOBIUS); CYTOPLASMIC_DOMAIN (PHOBIUS); NON_CYTOPLASMIC_DOMAIN (PHOBIUS); TMhelix (TMHMM); TMhelix (TMHMM); TMhelix (TMHMM)
1505	288746_length_921_cvg_8.4_tip_1_0	87	ion transport peptide-like	307	1.72E-26	69.70%	0.157	IPR001166 (PRINTS); IPR001166 (G3DSA:1.10.2010.GENE3D); IPR001166 (PFAM); CYTOPLASMIC_DOMAIN (PHOBIUS); NON_CYTOPLASMIC_DOMAIN (PHOBIUS); TRANSMEMBRANE (PHOBIUS); IPR001166 (SUPERFAMILY); TMhelix (TMHMM)
1506	288746_length_921_cvg_8.4_tip_1_2	87	ion transport peptide isoform x2	307	6.99E-06	86.00%	0.357 Y	IPR001166 (PFAM); IPR001166 (G3DSA:1.10.2010.GENE3D); IPR001166 (SUPERFAMILY)
1507	288792_length_921_cvg_6.5_tip_1_5	67	allatostatin precursor	307	1.65E-06	48.00%	0.104	TRANSMEMBRANE (PHOBIUS); NON_CYTOPLASMIC_DOMAIN (PHOBIUS); TRANSMEMBRANE (PHOBIUS); CYTOPLASMIC_DOMAIN (PHOBIUS); CYTOPLASMIC_DOMAIN (PHOBIUS)
1508	288800_length_921_cvg_26.4_tip_1_1	299	gamma-interferon-inducible lysosomal thiol reductase	307	3.42E-50	57.10%	0.282	IPR004911 (PFAM); PTHR13234:SF8 (PANTHER); IPR004911 (PANTHER); SIGNAL_PEPTIDE_C_REGION (PHOBIUS); SIGNAL_PEPTIDE_H_REGION (PHOBIUS); SIGNAL_PEPTIDE_N_REGION (PHOBIUS); NON_CYTOPLASMIC_DOMAIN (PHOBIUS); SIGNAL_PEPTIDE (PHOBIUS)
1509	288828_length_923_cvg_13.7_tip_1_4	132	serine protease	308	8.64E-106	73.80%	0.114	IPR008758 (PFAM); PTHR11010:SF9 (PANTHER); IPR008758 (PANTHER); TRANSMEMBRANE (PHOBIUS); CYTOPLASMIC_DOMAIN (PHOBIUS); NON_CYTOPLASMIC_DOMAIN (PHOBIUS)
1510	288842_length_923_cvg_3.6_tip_1_1	47	n-alpha-acetyltransferase auxiliary subunit	308	5.56E-116	75.40%	0.112	IPR011990 (G3DSA:1.25.40.GENE3D); PTHR22767 (PANTHER); PTHR22767:SF3 (PANTHER); IPR013026 (PROSITE_PROFILES); SSF48452 (SUPERFAMILY)
1511	288876_length_925_cvg_9.2_tip_1_4	103	steroid membrane receptor 25-	308	4.43E-64	81.50%	0.119	IPR001199 (G3DSA:3.10.120.GENE3D); IPR001199 (PFAM); PTHR10281 (PANTHER); PTHR10281:SF25 (PANTHER); IPR001199 (SUPERFAMILY)
1512	288904_length_925_cvg_88.5_tip_1_1	134475	hemocyanin subunit type 1 precursor	308	7.50E-124	69.30%	0.115	IPR005203 (PFAM); IPR005203 (G3DSA:2.60.40.GENE3D); IPR013788 (PANTHER); IPR014756 (SUPERFAMILY)
1513	288958_length_927_cvg_4.0_tip_1_1	52	zinc finger protein 275	309	1.05E-20	57.50%	0.113	IPR015880 (SMART); IPR013087 (G3DSA:3.30.160.GENE3D); PF13894 (PFAM); PF13913 (PFAM); IPR007087 (PFAM); IPR013087 (G3DSA:3.30.160.GENE3D); PTHR24409 (PANTHER); IPR007087 (PROSITE_PATTERNS); IPR007087 (PROSITE_PATTERNS); IPR007087 (PROSITE_PATTERNS); IPR007087 (PROSITE_PATTERNS); IPR007087 (PROSITE_PROFILES); IPR007087 (PROSITE_PROFILES); IPR007087 (PROSITE_PROFILES); IPR007087 (PROSITE_PROFILES); SSF57667 (SUPERFAMILY); SSF57667 (SUPERFAMILY)
1514	288990_length_929_cvg_12.6_tip_1_3	115	prolyl 3-hydroxylase 1-like isoform x2	310	3.27E-115	68.70%	0.102	IPR011990 (G3DSA:1.25.40.GENE3D); PTHR13986:SF8 (PANTHER); PTHR13986 (PANTHER)
1515	289056_length_930_cvg_6.1_tip_1_2	51	dipeptidyl aminopeptidase-like protein 6 isoform x1	310	2.00E-83	62.70%	0.104	IPR002469 (G3DSA:2.140.10.GENE3D); IPR002469 (PFAM); PTHR11731 (PANTHER); PTHR11731:SF22 (PANTHER); TRANSMEMBRANE (PHOBIUS); NON_CYTOPLASMIC_DOMAIN (PHOBIUS); CYTOPLASMIC_DOMAIN (PHOBIUS); SSF82171 (SUPERFAMILY); TMhelix (TMHMM)

1517	289080_length_931_cvg_30.1_tip_1_4	359	urokinase-type plasminogen activator	310	3.72E-64	55.00%	0.1	IPR001314 (PRINTS); IPR001254 (SMART); G3DSA:2.40.10.10 (GENE3D); G3DSA:2.40.10.10 (GENE3D); G3DSA:2.40.10.10 (GENE3D); IPR001254 (PFAM); PTHR24265 (PANTHER); IPR018114 (PROSITE_PATTERNS); IPR018114 (PROSITE_PATTERNS); IPR001254 (PROSITE_PROFILES); IPR009003 (SUPERFAMILY)
1518	289108_length_932_cvg_3.1_tip_1_2	37	alcohol dehydrogenase	310	9.82E-109	66.20%	0.119	IPR020471 (PRINTS); IPR023210 (G3DSA:3.20.20.GENE3D); IPR020471 (PIRSF); IPR023210 (PFAM); IPR001395 (PANTHER); PTHR11732:SF183 (PANTHER); IPR018170 (PROSITE_PATTERNS); IPR018170 (PROSITE_PATTERNS); IPR018170 (PROSITE_PATTERNS); IPR023210 (SUPERFAMILY)
1516	289070_length_931_cvg_58.7_tip_1_0	923	coagulation factor v	311	3.09E-15	43.60%	0.283	TRANSMEMBRANE (PHOBIUS); NON_CYTOPLASMIC_DOMAIN (PHOBIUS); TRANSMEMBRANE (PHOBIUS); TRANSMEMBRANE (PHOBIUS); TRANSMEMBRANE (PHOBIUS); NON_CYTOPLASMIC_DOMAIN (PHOBIUS); TRANSMEMBRANE (PHOBIUS); CYTOPLASMIC_DOMAIN (PHOBIUS); CYTOPLASMIC_DOMAIN (PHOBIUS); TRANSMEMBRANE (PHOBIUS); CYTOPLASMIC_DOMAIN (PHOBIUS); NON_CYTOPLASMIC_DOMAIN (PHOBIUS); CYTOPLASMIC_DOMAIN (PHOBIUS); TRANSMEMBRANE (PHOBIUS); NON_CYTOPLASMIC_DOMAIN (PHOBIUS); NON_CYTOPLASMIC_DOMAIN (PHOBIUS); TRANSMEMBRANE (PHOBIUS); TMhelix (TMHMM); TMhelix (TMHMM); TMhelix (TMHMM); TMhelix (TMHMM); TMhelix (TMHMM); TMhelix (TMHMM); TMhelix (TMHMM); TMhelix (TMHMM); TMhelix (TMHMM)
1519	289154_length_934_cvg_3.5_tip_1_3	51	zinc finger protein 676-like	312	1.65E-103	65.70%	0.115	IPR015880 (SMART); IPR013087 (G3DSA:3.30.160.GENE3D); PF13465 (PFAM); IPR013087 (G3DSA:3.30.160.GENE3D); IPR013087 (G3DSA:3.30.160.GENE3D); IPR013087 (G3DSA:3.30.160.GENE3D); PTHR24409 (PANTHER); IPR007087 (PROSITE_PATTERNS); IPR007087 (PROSITE_PATTERNS); IPR007087 (PROSITE_PATTERNS); IPR007087 (PROSITE_PATTERNS); IPR007087 (PROSITE_PATTERNS); IPR007087 (PROSITE_PATTERNS); IPR007087 (PROSITE_PATTERNS); IPR007087 (PROSITE_PATTERNS); IPR007087 (PROSITE_PATTERNS); IPR007087 (PROSITE_PROFILES); IPR007087 (PROSITE_PROFILES); IPR007087 (PROSITE_PROFILES); IPR007087 (PROSITE_PROFILES); IPR007087 (PROSITE_PROFILES); IPR007087 (PROSITE_PROFILES); IPR007087 (PROSITE_PROFILES); IPR007087 (PROSITE_PROFILES); IPR007087 (PROSITE_PROFILES); SSF57667 (SUPERFAMILY); SSF57667 (SUPERFAMILY); SSF57667 (SUPERFAMILY); SSF57667 (SUPERFAMILY); SSF57667 (SUPERFAMILY)
1520	289198_length_936_cvg_3.1_tip_1_3	51	probable g-protein coupled receptor 133	312	1.13E-55	52.50%	0.2	IPR000832 (PFAM); PTHR12011 (PANTHER); PTHR12011:SF222 (PANTHER); NON_CYTOPLASMIC_DOMAIN (PHOBIUS); TRANSMEMBRANE (PHOBIUS); TRANSMEMBRANE (PHOBIUS); CYTOPLASMIC_DOMAIN (PHOBIUS); TRANSMEMBRANE (PHOBIUS); NON_CYTOPLASMIC_DOMAIN (PHOBIUS); CYTOPLASMIC_DOMAIN (PHOBIUS); NON_CYTOPLASMIC_DOMAIN (PHOBIUS); TRANSMEMBRANE (PHOBIUS); NON_CYTOPLASMIC_DOMAIN (PHOBIUS); TRANSMEMBRANE (PHOBIUS); TRANSMEMBRANE (PHOBIUS); CYTOPLASMIC_DOMAIN (PHOBIUS); IPR017981 (PROSITE_PROFILES); TMhelix (TMHMM); TMhelix (TMHMM); TMhelix (TMHMM); TMhelix (TMHMM)
1522	289254_length_938_cvg_4.6_tip_1_2	47	alkaline tissue-nonspecific isozyme-like	312	1.09E-94	64.50%	0.502 Y	IPR001952 (PRINTS); IPR001952 (SMART); IPR017849 (G3DSA:3.40.720.GENE3D); IPR001952 (PFAM); PTHR11596 (PANTHER); IPR018299 (PROSITE_PATTERNS); NON_CYTOPLASMIC_DOMAIN (PHOBIUS); SIGNAL_PEPTIDE_N_REGION (PHOBIUS); SIGNAL_PEPTIDE (PHOBIUS); SIGNAL_PEPTIDE_H_REGION (PHOBIUS); SIGNAL_PEPTIDE_C_REGION (PHOBIUS); SignalP-noTM (SIGNALP_EUK); IPR017850 (SUPERFAMILY); TMhelix (TMHMM)
1521	289244_length_938_cvg_6.6_tip_1_3	105	lipophorin receptor	313	8.58E-38	59.90%	0.137	IPR000033 (SMART); PF14670 (PFAM); G3DSA:2.10.25.10 (GENE3D); IPR000033 (PFAM); IPR011042 (G3DSA:2.120.10.GENE3D); PTHR10529 (PANTHER); IPR000033 (PROSITE_PROFILES); IPR000033 (PROSITE_PROFILES); SSF57196 (SUPERFAMILY); SSF63825 (SUPERFAMILY)
1523	289290_length_940_cvg_4.5_tip_1_1	58	adp-ribosylation factor-related protein 1	313	1.24E-125	94.40%	0.112	IPR001806 (PRINTS); IPR024156 (SMART); IPR003579 (SMART); IPR006687 (SMART); IPR006689 (PFAM); IPR005225 (TIGRFAM); IPR027417 (G3DSA:3.40.50.GENE3D); PTHR11711:SF19 (PANTHER); PTHR11711 (PANTHER); IPR024156 (PROSITE_PROFILES); IPR027417 (SUPERFAMILY)
1526	289324_length_941_cvg_3.5_tip_1_5	55	endothelin-converting enzyme 1 isoform x2	313	6.49E-89	64.20%	0.113	G3DSA:1.10.1380.10 (GENE3D); IPR008753 (PFAM); IPR000718 (PANTHER); SSF55486 (SUPERFAMILY)

1524	289314_length_941_cvg_9.3_tip_1_0	120	macrophage mannose receptor 1	314	9.39E-53	51.60%	0.12		IPR002352 (PRINTS); IPR001304 (SMART); IPR016186 (G3DSA:3.10.100.GENE3D); IPR016186 (G3DSA:3.10.100.GENE3D); IPR001304 (PFAM); IPR016186 (G3DSA:3.10.100.GENE3D); PTHR22803:SF16 (PANTHER); PTHR22803 (PANTHER); IPR018378 (PROSITE_PATTERNS); IPR001304 (PROSITE_PROFILES); IPR001304 (PROSITE_PROFILES); IPR016187 (SUPERFAMILY); IPR016187 (SUPERFAMILY)
1525	289320_length_941_cvg_3.9_tip_1_3	44	epidermal growth factor receptor isoform x1	314	5.60E-93	65.00%	0.137		IPR001245 (PFAM); G3DSA:1.10.510.10 (GENE3D); PTHR24416:SF90 (PANTHER); PTHR24416 (PANTHER); IPR000719 (PROSITE_PROFILES); IPR011009 (SUPERFAMILY)
1527	289336_length_942_cvg_25.6_tip_1_4	261	sulfakinin-like peptide	314	1.02E-15	65.70%	0.198		IPR013152 (PROSITE_PATTERNS); IPR013152 (PROSITE_PATTERNS); CYTOPLASMIC_DOMAIN (PHOBIUS); TRANSMEMBRANE (PHOBIUS); NON_CYTOPLASMIC_DOMAIN (PHOBIUS); TMhelix (TMHMM)
1528	289494_length_947_cvg_57.0_tip_1_2	922	mitochondrial manganese superoxide dismutase	315	1.01E-121	86.10%	0.122		Coil (COILS); IPR001189 (PRINTS); G3DSA:1.10.287.990 (GENE3D); IPR019832 (PFAM); IPR019831 (PFAM); PTHR11404:SF6 (PANTHER); IPR001189 (PANTHER); IPR019833 (PROSITE_PATTERNS); TRANSMEMBRANE (PHOBIUS); CYTOPLASMIC_DOMAIN (PHOBIUS); NON_CYTOPLASMIC_DOMAIN (PHOBIUS); IPR019831 (SUPERFAMILY); IPR019832 (SUPERFAMILY); TMhelix (TMHMM)
1529	289548_length_948_cvg_61.0_tip_1_3	843	coagulation factor v	316	2.61E-09	45.00%	0.510	Y	CYTOPLASMIC_DOMAIN (PHOBIUS); NON_CYTOPLASMIC_DOMAIN (PHOBIUS); TRANSMEMBRANE (PHOBIUS); NON_CYTOPLASMIC_DOMAIN (PHOBIUS); SIGNAL_PEPTIDE (PHOBIUS); SIGNAL_PEPTIDE_C_REGION (PHOBIUS); SIGNAL_PEPTIDE_H_REGION (PHOBIUS); CYTOPLASMIC_DOMAIN (PHOBIUS); TRANSMEMBRANE (PHOBIUS); TRANSMEMBRANE (PHOBIUS); NON_CYTOPLASMIC_DOMAIN (PHOBIUS); TRANSMEMBRANE (PHOBIUS); NON_CYTOPLASMIC_DOMAIN (PHOBIUS); SIGNAL_PEPTIDE_N_REGION (PHOBIUS); CYTOPLASMIC_DOMAIN (PHOBIUS); TRANSMEMBRANE (PHOBIUS); CYTOPLASMIC_DOMAIN (PHOBIUS); NON_CYTOPLASMIC_DOMAIN (PHOBIUS); TRANSMEMBRANE (PHOBIUS); CYTOPLASMIC_DOMAIN (PHOBIUS); TRANSMEMBRANE (PHOBIUS); TRANSMEMBRANE (PHOBIUS); TRANSMEMBRANE (PHOBIUS); SignalP-TM (SIGNALP_EUK); TMhelix (TMHMM); TMhelix (TMHMM); TMhelix (TMHMM); TMhelix (TMHMM); TMhelix (TMHMM); TMhelix (TMHMM); TMhelix (TMHMM); TMhelix (TMHMM); TMhelix (TMHMM)
1533	289596_length_950_cvg_4.8_tip_1_2	68	membrane glycoprotein lig-	316	1.84E-17	46.60%	0.725	Y	IPR003591 (SMART); IPR001611 (PFAM); G3DSA:3.80.10.10 (GENE3D); PTHR24365 (PANTHER); NON_CYTOPLASMIC_DOMAIN (PHOBIUS); SIGNAL_PEPTIDE_N_REGION (PHOBIUS); SIGNAL_PEPTIDE_H_REGION (PHOBIUS); SIGNAL_PEPTIDE (PHOBIUS); SIGNAL_PEPTIDE_C_REGION (PHOBIUS); IPR001611 (PROSITE_PROFILES); IPR001611 (PROSITE_PROFILES); IPR001611 (PROSITE_PROFILES); IPR001611 (PROSITE_PROFILES); IPR001611 (PROSITE_PROFILES); SignalP-noTM (SIGNALP_EUK); SSF52058 (SUPERFAMILY)
1530	289576_length_950_cvg_31.1_tip_1_3	428	aael000271- partial	317	2.81E-71	67.70%	0.111		IPR029062 (G3DSA:3.40.50.GENE3D); IPR011697 (PFAM); IPR015527 (PANTHER); IPR015527 (PROSITE_PROFILES); IPR029062 (SUPERFAMILY)
1531	289586_length_950_cvg_4.0_tip_1_4	43	plexin- partial	317	7.98E-100	78.40%	0.116		IPR002909 (SMART); IPR013783 (G3DSA:2.60.40.GENE3D); IPR002909 (PFAM); IPR013783 (G3DSA:2.60.40.GENE3D); PTHR22625 (PANTHER); PTHR22625:SF16 (PANTHER); IPR014756 (SUPERFAMILY); IPR014756 (SUPERFAMILY)
1532	289590_length_950_cvg_10.0_tip_1_4	134	prohormone-1-like	317	6.87E-30	83.90%	0.115		no IPS match
1534	289612_length_951_cvg_8.5_tip_1_3	92	low-density lipoprotein receptor-related protein 2	317	2.19E-23	58.80%	0.102		IPR002172 (PRINTS); IPR002172 (SMART); IPR002172 (G3DSA:4.10.400.GENE3D); IPR002172 (G3DSA:4.10.400.GENE3D); IPR002172 (PFAM); PTHR10529 (PANTHER); IPR023415 (PROSITE_PATTERNS); NON_CYTOPLASMIC_DOMAIN (PHOBIUS); CYTOPLASMIC_DOMAIN (PHOBIUS); TRANSMEMBRANE (PHOBIUS); IPR002172 (PROSITE_PROFILES); IPR002172 (PROSITE_PROFILES); IPR002172 (PROSITE_PROFILES); SignalP-TM (SIGNALP_GRAM_POSITIVE); IPR002172 (SUPERFAMILY); IPR002172 (SUPERFAMILY); IPR002172 (SUPERFAMILY); TMhelix (TMHMM)

1535	289624_length_951_cvg_5.5_tip_1_3	80	transcription factor partial	317	2.36E-58	54.20%	0.106	IPR015880 (SMART); PF13894 (PFAM); IPR013087 (G3DSA:3.30.160.GENE3D); IPR007087 (PFAM); IPR013087 (G3DSA:3.30.160.GENE3D); IPR013087 (G3DSA:3.30.160.GENE3D); IPR013087 (G3DSA:3.30.160.GENE3D); IPR013087 (G3DSA:3.30.160.GENE3D); PTHR24382 (PANTHER); PTHR24382:SF29 (PANTHER); IPR007087 (PROSITE_PATTERNS); IPR007087 (PROSITE_PATTERNS); IPR007087 (PROSITE_PATTERNS); IPR007087 (PROSITE_PATTERNS); IPR007087 (PROSITE_PATTERNS); IPR007087 (PROSITE_PATTERNS); IPR007087 (PROSITE_PATTERNS); IPR007087 (PROSITE_PROFILES); IPR007087 (PROSITE_PROFILES); IPR007087 (PROSITE_PROFILES); IPR007087 (PROSITE_PROFILES); IPR007087 (PROSITE_PROFILES); IPR007087 (PROSITE_PROFILES); IPR007087 (PROSITE_PROFILES); SSF57667 (SUPERFAMILY); SSF57667 (SUPERFAMILY); SSF57667 (SUPERFAMILY); SSF57667 (SUPERFAMILY)
1536	289626_length_951_cvg_69.6_tip_0_4	8101	hemocytin isoform x1	317	1.60E-31	43.40%	0.111	G3DSA:2.10.25.10 (GENE3D); IPR001846 (PFAM); PTHR11339 (PANTHER); PTHR11339:SF25 (PANTHER); IPR001846 (PROSITE_PROFILES); IPR002919 (SUPERFAMILY)
1537	289642_length_952_cvg_37.1_tip_1_5	456	plasminogen activator spa	317	1.62E-59	60.80%	0.162	IPR001314 (PRINTS); IPR001254 (SMART); G3DSA:2.40.10.10 (GENE3D); G3DSA:2.40.10.10 (GENE3D); IPR001254 (PFAM); PTHR24265 (PANTHER); IPR018114 (PROSITE_PATTERNS); IPR018114 (PROSITE_PATTERNS); NON_CYTOPLASMIC_DOMAIN (PHOBIUS); CYTOPLASMIC_DOMAIN (PHOBIUS); TRANSMEMBRANE (PHOBIUS); IPR001254 (PROSITE_PROFILES); IPR009003 (SUPERFAMILY)
1538	289672_length_953_cvg_7.0_tip_1_5	82	complement c1q tumor necrosis factor-related protein 6 precursor	317	4.90E-20	52.20%	0.334	Coil (COILS); IPR001073 (PRINTS); IPR001073 (SMART); IPR008983 (G3DSA:2.60.120.GENE3D); IPR001073 (PFAM); PTHR22923 (PANTHER); PTHR22923:SF51 (PANTHER); NON_CYTOPLASMIC_DOMAIN (PHOBIUS); CYTOPLASMIC_DOMAIN (PHOBIUS); TRANSMEMBRANE (PHOBIUS); IPR001073 (PROSITE_PROFILES); IPR008983 (SUPERFAMILY); TMhelix (TMHMM)
1539	289742_length_957_cvg_3.9_tip_1_5	50	---NA---	319			0.115	IPR000998 (SMART); IPR000998 (PFAM); TRANSMEMBRANE (PHOBIUS); NON_CYTOPLASMIC_DOMAIN (PHOBIUS); CYTOPLASMIC_DOMAIN (PHOBIUS); IPR000998 (PROSITE_PROFILES); IPR013320 (SUPERFAMILY); TMhelix (TMHMM)
1540	289844_length_961_cvg_6.2_tip_1_1	82	balbiani ring protein 3	320	3.40E-18	49.10%	0.154	IPR000072 (SMART); IPR029034 (G3DSA:2.10.90.GENE3D); IPR004153 (PFAM); IPR000072 (PFAM); PTHR11633:SF1 (PANTHER); PTHR11633 (PANTHER); TRANSMEMBRANE (PHOBIUS); NON_CYTOPLASMIC_DOMAIN (PHOBIUS); CYTOPLASMIC_DOMAIN (PHOBIUS); TRANSMEMBRANE (PHOBIUS); CYTOPLASMIC_DOMAIN (PHOBIUS); IPR000072 (PROSITE_PROFILES); IPR029034 (SUPERFAMILY); TMhelix (TMHMM)
1541	289942_length_965_cvg_42.0_tip_1_0	472	peroxiredoxin partial	322	3.23E-116	83.50%	0.13	IPR000866 (PFAM); IPR019479 (PFAM); IPR012336 (G3DSA:3.40.30.GENE3D); G3DSA:3.30.1020.10 (GENE3D); PTHR10681:SF77 (PANTHER); PTHR10681 (PANTHER); CYTOPLASMIC_DOMAIN (PHOBIUS); TRANSMEMBRANE (PHOBIUS); NON_CYTOPLASMIC_DOMAIN (PHOBIUS); IPR012336 (PROSITE_PROFILES); IPR012336 (SUPERFAMILY); TMhelix (TMHMM)
1542	289958_length_966_cvg_2.0_tip_1_0	22	retinol dehydrogenase 13-like	322	2.29E-29	65.00%	0.107	IPR016040 (G3DSA:3.40.50.GENE3D); PTHR24320:SF55 (PANTHER); PTHR24320 (PANTHER); SSF51735 (SUPERFAMILY)
1543	289992_length_967_cvg_7.1_tip_1_4	83	tho complex subunit 3	322	3.64E-164	89.10%	0.107	IPR001680 (SMART); IPR015943 (G3DSA:2.130.10.GENE3D); IPR001680 (PFAM); IPR015943 (G3DSA:2.130.10.GENE3D); IPR011659 (PFAM); PTHR22839 (PANTHER); IPR019775 (PROSITE_PATTERNS); TRANSMEMBRANE (PHOBIUS); NON_CYTOPLASMIC_DOMAIN (PHOBIUS); CYTOPLASMIC_DOMAIN (PHOBIUS); IPR001680 (PROSITE_PROFILES); IPR001680 (PROSITE_PROFILES); IPR017986 (PROSITE_PROFILES); IPR017986 (SUPERFAMILY); TMhelix (TMHMM)
1544	289994_length_967_cvg_33.9_tip_0_0	492	contactin associated protein 1	323	3.92E-52	52.70%	0.103	IPR001073 (PFAM); IPR008983 (G3DSA:2.60.120.GENE3D); PTHR10127:SF575 (PANTHER); PTHR10127 (PANTHER); IPR001073 (PROSITE_PROFILES); IPR002181 (SUPERFAMILY); IPR008983 (SUPERFAMILY)
1545	290002_length_967_cvg_4.5_tip_1_3	60	achain tachylectin 5a from tachypleus tridentatus (japanese horseshoe crab)	323	9.76E-55	60.20%	0.15	IPR002181 (SMART); IPR014716 (G3DSA:3.90.215.GENE3D); IPR014715 (G3DSA:4.10.530.GENE3D); IPR002181 (PFAM); PTHR19143 (PANTHER); IPR020837 (PROSITE_PATTERNS); IPR002181 (PROSITE_PROFILES); IPR002181 (SUPERFAMILY)
1546	290028_length_968_cvg_7.1_tip_1_3	88	neurogenic locus notch partial	323	4.75E-143	81.00%	0.103	IPR001507 (SMART); IPR001507 (PFAM); PTHR22907 (PANTHER); TRANSMEMBRANE (PHOBIUS); CYTOPLASMIC_DOMAIN (PHOBIUS); NON_CYTOPLASMIC_DOMAIN (PHOBIUS); IPR001507 (PROSITE_PROFILES); TMhelix (TMHMM)
1547	290030_length_968_cvg_4.8_tip_1_3	49	isoform i	323	2.47E-71	57.30%	0.1	IPR001791 (SMART); IPR013320 (G3DSA:2.60.120.GENE3D); IPR001791 (PFAM); PTHR15036 (PANTHER); IPR001791 (PROSITE_PROFILES); IPR013320 (SUPERFAMILY)

1548	290036_length_969_cvg_4.0_tip_1_1	44	peptidylprolyl isomerase domain and wd repeat-containing protein 1	323	0	88.60%	0.132	IPR002130 (PRINTS); IPR002130 (PFAM); IPR015943 (G3DSA:2.130.10.GENE3D); IPR029000 (G3DSA:2.40.100.GENE3D); PTHR11071:SF228 (PANTHER); IPR024936 (PANTHER); IPR002130 (PROSITE_PROFILES); IPR029000 (SUPERFAMILY); SSF69322 (SUPERFAMILY)
1549	290064_length_970_cvg_3.6_tip_1_1	46	slit homolog 3 protein	323	1.26E-08	47.40%	0.122	IPR001611 (PFAM); G3DSA:3.80.10.10 (GENE3D); PTHR24365 (PANTHER); PTHR24365:SF265 (PANTHER); CYTOPLASMIC_DOMAIN (PHOBIUS); NON_CYTOPLASMIC_DOMAIN (PHOBIUS); TRANSMEMBRANE (PHOBIUS); SSF52058 (SUPERFAMILY); TMhelix (TMHMM); TMhelix (TMHMM)
1550	290110_length_972_cvg_21.6_tip_1_5	260	plcl_mytga ame: full=perlucin-like protein flags: precursor	324	9.99E-13	44.50%	0.296	IPR001304 (SMART); IPR001304 (PFAM); IPR016186 (G3DSA:3.10.100.GENE3D); PTHR22802 (PANTHER); IPR001304 (PROSITE_PROFILES); IPR016187 (SUPERFAMILY)
1551	290130_length_973_cvg_2.7_tip_1_5	42	trypsin-1- partial	324	1.20E-46	54.20%	0.318	IPR001314 (PRINTS); IPR001254 (SMART); G3DSA:2.40.10.10 (GENE3D); G3DSA:2.40.10.10 (GENE3D); G3DSA:2.40.10.10 (GENE3D); IPR001254 (PFAM); PTHR24265 (PANTHER); IPR018114 (PROSITE_PATTERNS); TRANSMEMBRANE (PHOBIUS); CYTOPLASMIC_DOMAIN (PHOBIUS); CYTOPLASMIC_DOMAIN (PHOBIUS); TRANSMEMBRANE (PHOBIUS); NON_CYTOPLASMIC_DOMAIN (PHOBIUS); IPR001254 (PROSITE_PROFILES); IPR009003 (SUPERFAMILY); TMhelix (TMHMM)
1556	290198_length_974_cvg_3.4_tip_1_2	46	cub domain protein	324	7.82E-11	47.80%	0.102	IPR000859 (SMART); IPR000859 (G3DSA:2.60.120.GENE3D); IPR000859 (PFAM); PTHR10127 (PANTHER); IPR000859 (PROSITE_PROFILES); IPR000859 (SUPERFAMILY)
1552	290152_length_973_cvg_4.9_tip_1_3	63	sulfhydryl oxidase 1-like	325	3.25E-65	64.40%	0.135	IPR013766 (PFAM); IPR012336 (G3DSA:3.40.30.GENE3D); IPR012336 (G3DSA:3.40.30.GENE3D); PTHR22897 (PANTHER); IPR012336 (PROSITE_PROFILES); IPR012336 (SUPERFAMILY)
1553	290168_length_974_cvg_5.5_tip_1_1	62	zinc finger protein zpr1	325	1.28E-128	73.50%	0.113	IPR004457 (SMART); IPR004457 (TIGRFAM); IPR004457 (PFAM); PTHR10876 (PANTHER); PTHR10876:SF0 (PANTHER)
1554	290176_length_974_cvg_70.9_tip_1_1	3207	trypsinogen h2_1g	325	1.15E-58	57.10%	0.934 Y	IPR001314 (PRINTS); IPR001254 (SMART); G3DSA:2.40.10.10 (GENE3D); IPR001254 (PFAM); G3DSA:2.40.10.10 (GENE3D); PTHR24265 (PANTHER); TRANSMEMBRANE (PHOBIUS); CYTOPLASMIC_DOMAIN (PHOBIUS); NON_CYTOPLASMIC_DOMAIN (PHOBIUS); IPR001254 (PROSITE_PROFILES); SignalP-noTM (SIGNALP_EUK); IPR009003 (SUPERFAMILY); TMhelix (TMHMM)
1555	290196_length_974_cvg_6.5_tip_1_3	75	serine protease inhibitor	325	8.01E-09	68.00%	0.121	G3DSA:2.30.39.10 (GENE3D); IPR023796 (PFAM); IPR000215 (PANTHER); PTHR11461:SF52 (PANTHER); IPR023795 (PROSITE_PATTERNS); IPR023796 (SUPERFAMILY)
1557	290236_length_976_cvg_32.7_tip_1_0	415	protein them6	326	2.40E-36	58.40%	0.129	Coil (COILS); IPR029069 (G3DSA:3.10.129.GENE3D); PF13279 (PFAM); PTHR12475 (PANTHER); NON_CYTOPLASMIC_DOMAIN (PHOBIUS); CYTOPLASMIC_DOMAIN (PHOBIUS); TRANSMEMBRANE (PHOBIUS); IPR029069 (SUPERFAMILY); TMhelix (TMHMM)
1558	290312_length_978_cvg_36.7_tip_1_0	465	cytochrome p450 2j2-like	326	9.16E-47	64.90%	0.099	IPR002401 (PRINTS); IPR001128 (PRINTS); IPR001128 (G3DSA:1.10.630.GENE3D); IPR001128 (PFAM); PTHR24300 (PANTHER); IPR017972 (PROSITE_PATTERNS); IPR001128 (SUPERFAMILY)
1559	290322_length_978_cvg_30.3_tip_1_4	368	thioredoxin-dependent peroxide reductase	326	1.76E-126	84.70%	0.172	IPR000866 (PFAM); IPR019479 (PFAM); IPR012336 (G3DSA:3.40.30.GENE3D); PTHR10681 (PANTHER); PTHR10681:SF101 (PANTHER); IPR012336 (PROSITE_PROFILES); IPR012336 (SUPERFAMILY)
1560	290430_length_982_cvg_5.2_tip_1_0	69	protein phosphatase 1 regulatory subunit 16a	328	1.03E-62	63.20%	0.223	IPR002110 (SMART); IPR020683 (G3DSA:1.25.40.GENE3D); IPR020683 (PFAM); PTHR24186 (PANTHER); IPR002110 (PROSITE_PROFILES); IPR002110 (PROSITE_PROFILES); IPR020683 (PROSITE_PROFILES); IPR020683 (SUPERFAMILY)
1561	290470_length_985_cvg_2.6_tip_1_4	44	vascular endothelial growth factor receptor 1 isoform x1	328	2.32E-18	49.80%	0.287	IPR003599 (SMART); IPR013783 (G3DSA:2.60.40.GENE3D); IPR013783 (G3DSA:2.60.40.GENE3D); PTHR15360 (PANTHER); IPR007110 (PROSITE_PROFILES); SSF48726 (SUPERFAMILY); SSF48726 (SUPERFAMILY)
1562	290490_length_986_cvg_28.2_tip_1_3	336	mesencephalic astrocyte-derived neurotrophic factor homolog	329	2.64E-77	85.10%	0.794 Y	IPR019345 (PFAM); IPR011001 (G3DSA:1.10.225.GENE3D); PTHR12990 (PANTHER); PTHR12990:SF5 (PANTHER); NON_CYTOPLASMIC_DOMAIN (PHOBIUS); SIGNAL_PEPTIDE_N_REGION (PHOBIUS); SIGNAL_PEPTIDE_C_REGION (PHOBIUS); SIGNAL_PEPTIDE_H_REGION (PHOBIUS); SIGNAL_PEPTIDE (PHOBIUS); SignalP-noTM (SIGNALP_EUK); SSF68906 (SUPERFAMILY)
1563	290518_length_987_cvg_24.0_tip_1_0	296	venom allergen 3-like	329	1.16E-48	55.00%	0.792 Y	IPR001283 (PRINTS); IPR002413 (PRINTS); IPR014044 (SMART); IPR014044 (G3DSA:3.40.33.GENE3D); IPR014044 (PFAM); IPR001283 (PANTHER); IPR018244 (PROSITE_PATTERNS); IPR018244 (PROSITE_PATTERNS); SIGNAL_PEPTIDE (PHOBIUS); NON_CYTOPLASMIC_DOMAIN (PHOBIUS); SIGNAL_PEPTIDE_C_REGION (PHOBIUS); SIGNAL_PEPTIDE_H_REGION (PHOBIUS); SIGNAL_PEPTIDE_N_REGION (PHOBIUS); SignalP-noTM (SIGNALP_GRAM_NEGATIVE); SignalP-noTM (SIGNALP_EUK); SignalP-TM (SIGNALP_GRAM_POSITIVE); IPR014044 (SUPERFAMILY)

1564	290568_length_989_cvg_52.5_tip_0_3	2172	14-3-3 partial	330	1.43E-27	100.00%	0.113	G3DSA:1.20.190.20 (GENE3D); IPR023410 (PFAM); IPR000308 (PANTHER); PTHR18860:SF21 (PANTHER); IPR023409 (PROSITE_PATTERNS); IPR023410 (SUPERFAMILY)
1565	290586_length_990_cvg_55.1_tip_1_3	28480	chitinase partial	330	6.46E-51	59.90%	0.104	IPR002557 (SMART); IPR002557 (G3DSA:2.170.140.GENE3D); IPR029070 (G3DSA:3.10.50.GENE3D); IPR002557 (PFAM); IPR001223 (PFAM); IPR013781 (G3DSA:3.20.20.GENE3D); PTHR11177 (PANTHER); PTHR11177:SF112 (PANTHER); IPR002557 (PROSITE_PROFILES); IPR017853 (SUPERFAMILY); IPR002557 (SUPERFAMILY); IPR029070 (SUPERFAMILY)
1566	290588_length_990_cvg_5.0_tip_1_0	52	coiled-coil domain-containing protein 134-like	330	1.02E-53	65.10%	0.148	IPR026321 (PFAM); IPR026321 (PANTHER); CYTOPLASMIC_DOMAIN (PHOBIUS); TRANSMEMBRANE (PHOBIUS); NON_CYTOPLASMIC_DOMAIN (PHOBIUS)
1567	290598_length_990_cvg_14.6_tip_0_5	149	nidogen- partial	330	8.40E-109	62.50%	0.104	IPR000742 (SMART); IPR000033 (SMART); IPR024731 (PFAM); IPR011042 (G3DSA:2.120.10.GENE3D); IPR023413 (G3DSA:2.40.155.GENE3D); PTHR24044 (PANTHER); PTHR24044:SF253 (PANTHER); IPR013032 (PROSITE_PATTERNS); IPR013032 (PROSITE_PATTERNS); IPR013032 (PROSITE_PATTERNS); IPR013032 (PROSITE_PATTERNS); IPR013032 (PROSITE_PATTERNS); IPR013032 (PROSITE_PATTERNS); IPR000742 (PROSITE_PROFILES); IPR000742 (PROSITE_PROFILES); IPR000742 (PROSITE_PROFILES); IPR000742 (PROSITE_PROFILES); IPR000742 (PROSITE_PROFILES); IPR000742 (PROSITE_PROFILES); IPR000033 (PROSITE_PROFILES); IPR009030 (SUPERFAMILY)
1568	290606_length_990_cvg_10.8_tip_1_1	139	collagen alpha-1 chain	330	1.59E-14	58.10%	0.244	IPR002172 (SMART); IPR002172 (PFAM); IPR002172 (G3DSA:4.10.400.GENE3D); IPR008160 (PFAM); PTHR24023:SF372 (PANTHER); PTHR24023 (PANTHER); IPR023415 (PROSITE_PATTERNS); IPR002172 (PROSITE_PROFILES); IPR002172 (SUPERFAMILY)
1569	290606_length_990_cvg_10.8_tip_1_3	139	serine-aspartate repeat protein f	330	2.33E-21	46.50%	0.102	no IPS match
1570	290650_length_992_cvg_58.0_tip_1_1	3075	peptidyl-prolyl cis-trans isomerase	331	1.63E-101	92.50%	0.32	IPR002130 (PRINTS); IPR029000 (G3DSA:2.40.100.GENE3D); IPR002130 (PFAM); PTHR11071:SF116 (PANTHER); IPR024936 (PANTHER); IPR020892 (PROSITE_PATTERNS); IPR002130 (PROSITE_PROFILES); IPR029000 (SUPERFAMILY)
1571	290664_length_992_cvg_3.1_tip_1_3	47	peroxidasin extracellular matrix-associated peroxidase	331	6.24E-08	62.70%	0.102	IPR019791 (PFAM); IPR019791 (G3DSA:1.10.640.GENE3D); PTHR11475 (PANTHER); IPR019791 (PROSITE_PROFILES); IPR010255 (SUPERFAMILY)
1572	290744_length_996_cvg_8.3_tip_1_0	123	5 -nucleotidase	332	5.96E-120	70.80%	0.11	IPR008334 (G3DSA:3.90.780.GENE3D); IPR008334 (PFAM); IPR006179 (PANTHER); PTHR11575:SF7 (PANTHER); IPR008334 (SUPERFAMILY)
1573	290762_length_996_cvg_4.6_tip_1_4	66	zinc finger protein	332	1.94E-24	52.20%	0.102	IPR015880 (SMART); IPR013087 (G3DSA:3.30.160.GENE3D); PF13894 (PFAM); IPR007087 (PFAM); IPR013087 (G3DSA:3.30.160.GENE3D); PF13465 (PFAM); IPR013087 (G3DSA:3.30.160.GENE3D); PTHR24409:SF14 (PANTHER); PTHR24409 (PANTHER); IPR007087 (PROSITE_PATTERNS); IPR007087 (PROSITE_PATTERNS); IPR007087 (PROSITE_PATTERNS); IPR007087 (PROSITE_PATTERNS); IPR007087 (PROSITE_PROFILES); IPR007087 (PROSITE_PROFILES); IPR007087 (PROSITE_PROFILES); IPR007087 (PROSITE_PROFILES); IPR007087 (PROSITE_PROFILES); SSF57667 (SUPERFAMILY); SSF57667 (SUPERFAMILY); SSF57667 (SUPERFAMILY)
1574	290862_length_1000_cvg_3.1_tip_1_4	48	atrial natriuretic peptide-converting enzyme-like	333	4.19E-46	61.70%	0.1	Coil (COILS); IPR002172 (SMART); IPR020067 (SMART); IPR020067 (PFAM); IPR020067 (G3DSA:1.10.2000.GENE3D); IPR002172 (G3DSA:4.10.400.GENE3D); IPR002172 (PFAM); IPR023415 (PROSITE_PATTERNS); IPR020067 (PROSITE_PROFILES); IPR002172 (PROSITE_PROFILES); IPR020067 (SUPERFAMILY); IPR002172 (SUPERFAMILY)
1575	290870_length_1001_cvg_81.1_tip_1_2	18311	der p 3 allergen	333	4.30E-60	61.00%	0.886 Y	IPR001314 (PRINTS); IPR001254 (SMART); G3DSA:2.40.10.10 (GENE3D); G3DSA:2.40.10.10 (GENE3D); IPR001254 (PFAM); PTHR24276 (PANTHER); IPR018114 (PROSITE_PATTERNS); IPR018114 (PROSITE_PATTERNS); SIGNAL_PEPTIDE_C_REGION (PHOBIUS); SIGNAL_PEPTIDE_N_REGION (PHOBIUS); SIGNAL_PEPTIDE (PHOBIUS); NON_CYTOPLASMIC_DOMAIN (PHOBIUS); SIGNAL_PEPTIDE_H_REGION (PHOBIUS); IPR001254 (PROSITE_PROFILES); SignalP-noTM (SIGNALP_EUK); IPR009003 (SUPERFAMILY)
1576	290874_length_1001_cvg_52.9_tip_1_3	936	scp-like extracellular domain containing protein 1	334	5.99E-45	52.60%	0.854 Y	IPR002413 (PRINTS); IPR001283 (PRINTS); IPR014044 (SMART); IPR014044 (PFAM); IPR014044 (G3DSA:3.40.33.GENE3D); IPR001283 (PANTHER); SIGNAL_PEPTIDE_H_REGION (PHOBIUS); SIGNAL_PEPTIDE (PHOBIUS); NON_CYTOPLASMIC_DOMAIN (PHOBIUS); SIGNAL_PEPTIDE_C_REGION (PHOBIUS); SIGNAL_PEPTIDE_N_REGION (PHOBIUS); SignalP-noTM (SIGNALP_EUK); SignalP-TM (SIGNALP_GRAM_POSITIVE); SignalP-noTM (SIGNALP_GRAM_NEGATIVE); IPR014044 (SUPERFAMILY); TMhelix (TMHMM)

1577	290896_length_1002_cvg_9.5_tip_1_4	130	spondin-1 isoform x3	334	2.10E-110	72.80%	0.132	IPR002861 (PFAM); IPR009465 (PFAM); PTHR11311 (PANTHER); NON_CYTOPLASMIC_DOMAIN (PHOBIUS); TRANSMEMBRANE (PHOBIUS); CYTOPLASMIC_DOMAIN (PHOBIUS); IPR002861 (PROSITE_PROFILES); IPR009465 (PROSITE_PROFILES); TMhelix (TMHMM)
1579	290912_length_1003_cvg_11.8_tip_1_5	125	peptidyl-prolyl cis-trans isomerase a2	334	2.24E-35	54.30%	0.116	IPR002130 (PFAM); IPR029000 (G3DSA:2.40.100.GENE3D); IPR024936 (PANTHER); PTHR11071:SF202 (PANTHER); NON_CYTOPLASMIC_DOMAIN (PHOBIUS); CYTOPLASMIC_DOMAIN (PHOBIUS); TRANSMEMBRANE (PHOBIUS); NON_CYTOPLASMIC_DOMAIN (PHOBIUS); TRANSMEMBRANE (PHOBIUS); IPR002130 (PROSITE_PROFILES); IPR029000 (SUPERFAMILY); TMhelix (TMHMM); TMhelix (TMHMM); TMhelix (TMHMM); TMhelix (TMHMM)
1578	290910_length_1003_cvg_6.4_tip_1_3	107	biotinidase isoform x2	335	1.41E-10	45.80%	0.1	PTHR10609:SF14 (PANTHER); IPR012101 (PANTHER); CYTOPLASMIC_DOMAIN (PHOBIUS); TRANSMEMBRANE (PHOBIUS); NON_CYTOPLASMIC_DOMAIN (PHOBIUS); TMhelix (TMHMM)
1580	290914_length_1003_cvg_3.7_tip_1_0	59	vascular endothelial growth factor receptor 2	335	8.11E-52	55.90%	0.27	G3DSA:1.10.510.10 (GENE3D); IPR001245 (PFAM); PTHR24416:SF298 (PANTHER); PTHR24416 (PANTHER); IPR000719 (PROSITE_PROFILES); IPR011009 (SUPERFAMILY)
1581	290962_length_1005_cvg_3.0_tip_1_0	45	ankyrin repeat domain-containing protein 17	335	0	95.80%	0.12	IPR002110 (PRINTS); IPR002110 (SMART); IPR020683 (G3DSA:1.25.40.GENE3D); IPR020683 (G3DSA:1.25.40.GENE3D); IPR020683 (G3DSA:1.25.40.GENE3D); IPR020683 (G3DSA:1.25.40.GENE3D); PF13637 (PFAM); IPR020683 (PFAM); IPR002110 (PFAM); PTHR23206 (PANTHER); IPR002110 (PROSITE_PROFILES); IPR002110 (PROSITE_PROFILES); IPR002110 (PROSITE_PROFILES); IPR020683 (PROSITE_PROFILES); IPR002110 (PROSITE_PROFILES); IPR002110 (PROSITE_PROFILES); IPR002110 (PROSITE_PROFILES); IPR002110 (PROSITE_PROFILES); IPR002110 (PROSITE_PROFILES); IPR002110 (PROSITE_PROFILES); IPR002110 (PROSITE_PROFILES); IPR020683 (SUPERFAMILY)
1582	290968_length_1005_cvg_12.9_tip_1_3	191	d-dopachrome decarboxylase-a-like	335	2.36E-24	62.60%	0.112	IPR001398 (PFAM); G3DSA:3.30.429.10 (GENE3D); PTHR11954:SF22 (PANTHER); IPR001398 (PANTHER); TRANSMEMBRANE (PHOBIUS); TRANSMEMBRANE (PHOBIUS); CYTOPLASMIC_DOMAIN (PHOBIUS); NON_CYTOPLASMIC_DOMAIN (PHOBIUS); NON_CYTOPLASMIC_DOMAIN (PHOBIUS); IPR001398 (PRODOM); IPR014347 (SUPERFAMILY); TMhelix (TMHMM)
1583	290994_length_1006_cvg_19.2_tip_1_4	217	---NA---	335			0.106	no IPS match
1584	291008_length_1006_cvg_16.6_tip_1_4	183	endoplasmic reticulum resident protein 29	335	1.04E-95	70.80%	0.767 Y	Coil (COILS); IPR011679 (PFAM); IPR012336 (G3DSA:3.40.30.GENE3D); IPR011679 (G3DSA:1.20.1150.GENE3D); IPR012883 (PFAM); PTHR12211 (PANTHER); PTHR12211:SF0 (PANTHER); SIGNAL_PEPTIDE_H_REGION (PHOBIUS); SIGNAL_PEPTIDE_N_REGION (PHOBIUS); SIGNAL_PEPTIDE_C_REGION (PHOBIUS); NON_CYTOPLASMIC_DOMAIN (PHOBIUS); SIGNAL_PEPTIDE (PHOBIUS); SignalP-noTM (SIGNALP_EUK); SignalP-TM (SIGNALP_GRAM_POSITIVE); SignalP-noTM (SIGNALP_GRAM_NEGATIVE); IPR012336 (SUPERFAMILY); IPR011679 (SUPERFAMILY)
1585	291052_length_1008_cvg_59.2_tip_1_5	5006	hemagglutinin amebocyte aggregation factor-like	336	2.24E-41	61.00%	0.252	PF14704 (PFAM); IPR026645 (PANTHER); TRANSMEMBRANE (PHOBIUS); TRANSMEMBRANE (PHOBIUS); CYTOPLASMIC_DOMAIN (PHOBIUS); NON_CYTOPLASMIC_DOMAIN (PHOBIUS); NON_CYTOPLASMIC_DOMAIN (PHOBIUS); TMhelix (TMHMM); TMhelix (TMHMM)
1586	291054_length_1008_cvg_8.9_tip_1_1	118	carbonyl reductase	336	2.71E-128	74.90%	0.144	IPR002347 (PRINTS); IPR002198 (PRINTS); IPR016040 (G3DSA:3.40.50.GENE3D); IPR002198 (PFAM); PTHR24322:SF58 (PANTHER); PTHR24322 (PANTHER); SSF51735 (SUPERFAMILY)
1587	291056_length_1008_cvg_2.0_tip_1_3	32	insulin-like receptor	336	4.88E-105	65.20%	0.116	IPR006212 (SMART); G3DSA:2.10.220.10 (GENE3D); IPR013783 (G3DSA:2.60.40.GENE3D); IPR000494 (PFAM); IPR006211 (PFAM); IPR000494 (G3DSA:3.80.20.GENE3D); IPR000494 (G3DSA:3.80.20.GENE3D); PTHR24416:SF95 (PANTHER); PTHR24416 (PANTHER); IPR009030 (SUPERFAMILY); SSF52058 (SUPERFAMILY)
1609	291966_length_1052_cvg_8.7_tip_1_0	139	aminoacyl trna synthase complex-interacting multifunctional protein 1	338	4.43E-100	68.30%	0.117	Coil (COILS); IPR002547 (PFAM); IPR012340 (G3DSA:2.40.50.GENE3D); PTHR11586 (PANTHER); PTHR11586:SF1 (PANTHER); IPR002547 (PROSITE_PROFILES); IPR012340 (SUPERFAMILY)
1588	291226_length_1016_cvg_64.0_tip_1_4	11353	snaclec stejaggregin-a subunit beta-1-like isoform x2	339	5.60E-17	48.40%	0.305	IPR001304 (SMART); IPR016186 (G3DSA:3.10.100.GENE3D); IPR001304 (PFAM); PTHR22802 (PANTHER); SIGNAL_PEPTIDE (PHOBIUS); SIGNAL_PEPTIDE_H_REGION (PHOBIUS); SIGNAL_PEPTIDE_N_REGION (PHOBIUS); CYTOPLASMIC_DOMAIN (PHOBIUS); TRANSMEMBRANE (PHOBIUS); NON_CYTOPLASMIC_DOMAIN (PHOBIUS); SIGNAL_PEPTIDE_C_REGION (PHOBIUS); IPR001304 (PROSITE_PROFILES); IPR016187 (SUPERFAMILY); TMhelix (TMHMM)
1589	291242_length_1017_cvg_29.2_tip_1_2	429	chorion peroxidase-like	339	5.25E-41	68.70%	0.116	IPR019791 (G3DSA:1.10.640.GENE3D); IPR019791 (PFAM); PTHR11475 (PANTHER); IPR019791 (PROSITE_PROFILES); IPR010255 (SUPERFAMILY)

1590	291274_length_1018_cvg_46.1_tip_1_1	1427	low quality protein: glutathione peroxidase 1-like	339	5.44E-87	79.30%	0.148	IPR000889 (PRINTS); IPR012336 (G3DSA:3.40.30.GENE3D); IPR000889 (PFAM); PTHR11592:SF21 (PANTHER); IPR000889 (PANTHER); IPR000889 (PROSITE_PROFILES); IPR012336 (SUPERFAMILY)
1591	291302_length_1020_cvg_3.3_tip_1_0	41	ww domain-containing oxidoreductase	340	1.05E-103	72.30%	0.128	IPR002198 (PFAM); IPR016040 (G3DSA:3.40.50.GENE3D); PTHR24316 (PANTHER); PTHR24316:SF115 (PANTHER); NON_CYTOPLASMIC_DOMAIN (PHOBIUS); TRANSMEMBRANE (PHOBIUS); CYTOPLASMIC_DOMAIN (PHOBIUS); TRANSMEMBRANE (PHOBIUS); CYTOPLASMIC_DOMAIN (PHOBIUS); SSF51735 (SUPERFAMILY); TMhelix (TMHMM)
1592	291358_length_1023_cvg_6.9_tip_1_2	83	collagen triple helix repeat family protein	341	5.96E-13	53.88%	0.095	TRANSMEMBRANE (PHOBIUS); NON_CYTOPLASMIC_DOMAIN (PHOBIUS); CYTOPLASMIC_DOMAIN (PHOBIUS); TMhelix (TMHMM)
1593	291358_length_1023_cvg_6.9_tip_1_5	83	hypothetical protein	341	4.56E-16	54.50%	0.352 Y	no IPS match
1594	291398_length_1024_cvg_64.1_tip_1_5	20409	glyceraldehyde-3-phosphate dehydrogenase	341	0	90.30%	0.107	IPR020831 (PRINTS); IPR020828 (SMART); IPR016040 (G3DSA:3.40.50.GENE3D); IPR020828 (PFAM); IPR020831 (PIRSF); G3DSA:3.30.360.10 (GENE3D); IPR006424 (TIGRFAM); IPR020829 (PFAM); IPR020831 (PANTHER); IPR020830 (PROSITE_PATTERNS); SSF51735 (SUPERFAMILY); SSF55347 (SUPERFAMILY)
1595	291534_length_1030_cvg_2.1_tip_1_0	29	prion-like-(q n-rich) domain-bearing protein 25 isoform x2	344	1.72E-14	42.00%	0.104	no IPS match
1596	291608_length_1033_cvg_3.0_tip_1_1	46	hemocytin	344	3.15E-38	45.40%	0.114	G3DSA:2.10.25.10 (GENE3D); IPR014853 (PFAM); IPR001846 (PROSITE_PROFILES); IPR001846 (PROSITE_PROFILES); IPR002919 (SUPERFAMILY)
1597	291614_length_1034_cvg_5.5_tip_1_2	74	von willebrand factor a domain-containing protein 8-like	344	7.95E-130	71.40%	0.103	IPR011042 (G3DSA:2.120.10.GENE3D); PTHR21610:SF9 (PANTHER); PTHR21610 (PANTHER)
1598	291628_length_1034_cvg_62.5_tip_1_4	1449	epidermal retinol dehydrogenase 2-like	345	3.57E-94	72.60%	0.109	Coil (COILS); IPR002198 (PRINTS); IPR002347 (PRINTS); IPR016040 (G3DSA:3.40.50.GENE3D); IPR002198 (PFAM); PTHR24316 (PANTHER); PTHR24316:SF289 (PANTHER); CYTOPLASMIC_DOMAIN (PHOBIUS); NON_CYTOPLASMIC_DOMAIN (PHOBIUS); TRANSMEMBRANE (PHOBIUS); SSF51735 (SUPERFAMILY); TMhelix (TMHMM)
1599	291636_length_1034_cvg_9.1_tip_1_0	94	tissue plasminogen activator	345	5.64E-21	70.60%	0.099	G3DSA:2.40.10.10 (GENE3D); IPR001254 (PFAM); PTHR24256 (PANTHER); IPR018114 (PROSITE_PATTERNS); TRANSMEMBRANE (PHOBIUS); NON_CYTOPLASMIC_DOMAIN (PHOBIUS); CYTOPLASMIC_DOMAIN (PHOBIUS); IPR001254 (PROSITE_PROFILES); IPR009003 (SUPERFAMILY); TMhelix (TMHMM); TMhelix (TMHMM)
1600	291638_length_1035_cvg_11.2_tip_1_1	140	i-type lysozyme-like protein 2	345	1.71E-40	66.40%	0.102	IPR008597 (PFAM); PTHR11195:SF13 (PANTHER); IPR008597 (PANTHER); IPR018247 (PROSITE_PATTERNS)
1601	291640_length_1035_cvg_14.2_tip_1_5	219	trypsin 3	345	1.54E-57	60.80%	0.806 Y	IPR001314 (PRINTS); IPR001254 (SMART); IPR001254 (PFAM); G3DSA:2.40.10.10 (GENE3D); G3DSA:2.40.10.10 (GENE3D); G3DSA:2.40.10.10 (GENE3D); PTHR24265 (PANTHER); IPR018114 (PROSITE_PATTERNS); CYTOPLASMIC_DOMAIN (PHOBIUS); SIGNAL_PEPTIDE_C_REGION (PHOBIUS); NON_CYTOPLASMIC_DOMAIN (PHOBIUS); TRANSMEMBRANE (PHOBIUS); SIGNAL_PEPTIDE_H_REGION (PHOBIUS); SIGNAL_PEPTIDE_N_REGION (PHOBIUS); SIGNAL_PEPTIDE (PHOBIUS); IPR001254 (PROSITE_PROFILES); SignalP-TM (SIGNALP_GRAM_POSITIVE); SignalP-noTM (SIGNALP_EUK); SignalP-noTM (SIGNALP_GRAM_NEGATIVE); IPR009003 (SUPERFAMILY)
1602	291790_length_1042_cvg_16.0_tip_1_1	203	brain peptide idl-like protein	347	5.10E-109	87.90%	0.107	IPR002172 (SMART); IPR002172 (PFAM); IPR002172 (G3DSA:4.10.400.GENE3D); PTHR20967 (PANTHER); IPR023415 (PROSITE_PATTERNS); CYTOPLASMIC_DOMAIN (PHOBIUS); TRANSMEMBRANE (PHOBIUS); NON_CYTOPLASMIC_DOMAIN (PHOBIUS); NON_CYTOPLASMIC_DOMAIN (PHOBIUS); TRANSMEMBRANE (PHOBIUS); IPR002172 (PROSITE_PROFILES); IPR002172 (SUPERFAMILY); TMhelix (TMHMM); TMhelix (TMHMM)
1603	291812_length_1043_cvg_47.6_tip_1_2	637	27 kda hemolymph	347	1.58E-30	49.50%	0.191	IPR009832 (PFAM); PTHR20997 (PANTHER); TRANSMEMBRANE (PHOBIUS); CYTOPLASMIC_DOMAIN (PHOBIUS); NON_CYTOPLASMIC_DOMAIN (PHOBIUS)
1604	291856_length_1045_cvg_51.2_tip_1_1	851	zinc metalloproteinase nas-4-like	348	5.31E-43	55.30%	0.907 Y	IPR001506 (PRINTS); IPR006026 (SMART); IPR001506 (PFAM); IPR024079 (G3DSA:3.40.390.GENE3D); PTHR10127 (PANTHER); SIGNAL_PEPTIDE_H_REGION (PHOBIUS); SIGNAL_PEPTIDE (PHOBIUS); SIGNAL_PEPTIDE_C_REGION (PHOBIUS); SIGNAL_PEPTIDE_N_REGION (PHOBIUS); NON_CYTOPLASMIC_DOMAIN (PHOBIUS); SignalP-noTM (SIGNALP_EUK); SignalP-TM (SIGNALP_GRAM_NEGATIVE); SSF55486 (SUPERFAMILY)

1618	292074_length_1058_cvg_4.0_tip_1_4	54	serine-threonine kinase receptor-associated protein	348	0	85.90%	0.13	IPR020472 (PRINTS); IPR001680 (SMART); IPR001680 (PFAM); IPR015943 (G3DSA:2.130.10.GENE3D); PTHR19877 (PANTHER); IPR019775 (PROSITE_PATTERNS); IPR001680 (PROSITE_PROFILES); IPR017986 (PROSITE_PROFILES); IPR001680 (PROSITE_PROFILES); IPR001680 (PROSITE_PROFILES); IPR001680 (PROSITE_PROFILES); IPR017986 (SUPERFAMILY)
1606	291906_length_1048_cvg_39.8_tip_1_1	595	adiponectin receptor protein	349	3.06E-111	84.20%	0.282	IPR004254 (PFAM); IPR004254 (PANTHER); PTHR20855:SF15 (PANTHER); NON_CYTOPLASMIC_DOMAIN (PHOBIUS); TRANSMEMBRANE (PHOBIUS); TRANSMEMBRANE (PHOBIUS); CYTOPLASMIC_DOMAIN (PHOBIUS); CYTOPLASMIC_DOMAIN (PHOBIUS); NON_CYTOPLASMIC_DOMAIN (PHOBIUS); NON_CYTOPLASMIC_DOMAIN (PHOBIUS); TRANSMEMBRANE (PHOBIUS); CYTOPLASMIC_DOMAIN (PHOBIUS); TRANSMEMBRANE (PHOBIUS); TRANSMEMBRANE (PHOBIUS); TMhelix (TMHMM); TMhelix (TMHMM); TMhelix (TMHMM); TMhelix (TMHMM)
1608	291914_length_1049_cvg_8.0_tip_1_5	88	macrophage mannose receptor 1-like	349	5.03E-46	49.50%	0.103	IPR001304 (SMART); IPR016186 (G3DSA:3.10.100.GENE3D); IPR016186 (G3DSA:3.10.100.GENE3D); IPR001304 (PFAM); PTHR22803:SF16 (PANTHER); PTHR22803 (PANTHER); IPR018378 (PROSITE_PATTERNS); IPR001304 (PROSITE_PROFILES); IPR001304 (PROSITE_PROFILES); IPR016187 (SUPERFAMILY); IPR016187 (SUPERFAMILY)
1605	291890_length_1048_cvg_6.5_tip_1_0	75	dehydrogenase reductase sdr family member 11-like	350	1.62E-97	74.70%	0.165	IPR002198 (PRINTS); IPR002347 (PRINTS); IPR002198 (PFAM); IPR005639 (G3DSA:1.20.190.GENE3D); IPR016040 (G3DSA:3.40.50.GENE3D); PTHR24322:SF310 (PANTHER); PTHR24322 (PANTHER); SSF51735 (SUPERFAMILY)
1607	291912_length_1049_cvg_8.2_tip_1_1	109	c-type mannose receptor 2	350	4.69E-16	47.30%	0.178	IPR001304 (SMART); IPR001304 (PFAM); IPR016186 (G3DSA:3.10.100.GENE3D); PTHR22802:SF149 (PANTHER); PTHR22802 (PANTHER); IPR001304 (PROSITE_PROFILES); IPR016187 (SUPERFAMILY); TMhelix (TMHMM)
1610	291988_length_1053_cvg_4.9_tip_1_0	64	low quality protein: hemocytin	351	4.90E-34	48.40%	0.107	IPR025155 (PFAM); SSF57603 (SUPERFAMILY)
1611	291996_length_1053_cvg_23.6_tip_1_4	214	inactive hydroxysteroid dehydrogenase-like protein 1 isoform x1	351	2.85E-97	71.90%	0.104	Coil (COILS); IPR002347 (PRINTS); IPR002198 (PRINTS); IPR002198 (PFAM); IPR016040 (G3DSA:3.40.50.GENE3D); PTHR24316:SF317 (PANTHER); PTHR24316 (PANTHER); IPR020904 (PROSITE_PATTERNS); SSF51735 (SUPERFAMILY)
1612	292014_length_1054_cvg_8.0_tip_1_0	102	serine threonine-protein kinase pim-3	352	8.31E-140	77.70%	0.174	IPR002290 (SMART); IPR000719 (PFAM); G3DSA:1.10.510.10 (GENE3D); G3DSA:3.30.200.20 (GENE3D); IPR017348 (PIRSF); PTHR22984 (PANTHER); PTHR22984:SF1 (PANTHER); IPR008271 (PROSITE_PATTERNS); IPR017441 (PROSITE_PATTERNS); IPR000719 (PROSITE_PROFILES); IPR011009 (SUPERFAMILY)
1613	292018_length_1054_cvg_3.7_tip_1_0	63	probable serine carboxypeptidase cpvl isoform x1	352	8.48E-93	65.30%	0.334	IPR001563 (PRINTS); IPR001563 (PFAM); IPR029058 (G3DSA:3.40.50.GENE3D); IPR001563 (PANTHER); PTHR11802:SF30 (PANTHER); IPR029058 (SUPERFAMILY)
1614	292032_length_1056_cvg_4.8_tip_1_4	62	laminin a	352	1.93E-53	65.80%	0.097	IPR001791 (SMART); IPR001791 (PFAM); IPR013320 (G3DSA:2.60.120.GENE3D); PTHR10574:SF39 (PANTHER); PTHR10574 (PANTHER); IPR001791 (PROSITE_PROFILES); IPR013320 (SUPERFAMILY)
1615	292034_length_1056_cvg_3.1_tip_1_2	49	serine protease p153	352	3.67E-08	49.80%	0.102	IPR002172 (PRINTS); IPR003609 (SMART); IPR002172 (SMART); IPR002172 (G3DSA:4.10.400.GENE3D); G3DSA:3.50.4.10 (GENE3D); IPR003014 (PFAM); IPR002172 (PFAM); IPR023415 (PROSITE_PATTERNS); IPR003609 (PROSITE_PROFILES); IPR002172 (PROSITE_PROFILES); IPR003609 (PROSITE_PROFILES); SSF57414 (SUPERFAMILY); SSF57414 (SUPERFAMILY); IPR002172 (SUPERFAMILY)
1616	292038_length_1056_cvg_22.4_tip_1_2	338	ovalbumin-related protein x-like isoform x1	352	1.23E-72	61.10%	0.198	IPR023796 (SMART); G3DSA:3.30.497.10 (GENE3D); IPR023796 (PFAM); G3DSA:2.30.39.10 (GENE3D); PTHR11461:SF124 (PANTHER); IPR000215 (PANTHER); TRANSMEMBRANE (PHOBIUS); NON_CYTOPLASMIC_DOMAIN (PHOBIUS); NON_CYTOPLASMIC_DOMAIN (PHOBIUS); CYTOPLASMIC_DOMAIN (PHOBIUS); CYTOPLASMIC_DOMAIN (PHOBIUS); TRANSMEMBRANE (PHOBIUS); TRANSMEMBRANE (PHOBIUS); IPR023796 (SUPERFAMILY)
1617	292048_length_1056_cvg_7.8_tip_1_1	104	pc3-like endoprotease variant b-like	352	1.72E-30	57.20%	0.187	IPR015500 (PRINTS); IPR000209 (G3DSA:3.40.50.GENE3D); G3DSA:3.30.70.850 (GENE3D); IPR000209 (PFAM); PTHR10795:SF310 (PANTHER); IPR015500 (PANTHER); IPR023827 (PROSITE_PATTERNS); CYTOPLASMIC_DOMAIN (PHOBIUS); TRANSMEMBRANE (PHOBIUS); NON_CYTOPLASMIC_DOMAIN (PHOBIUS); IPR009020 (SUPERFAMILY); IPR000209 (SUPERFAMILY)
1619	292084_length_1059_cvg_5.2_tip_1_4	73	5 -nucleotidase	353	1.21E-148	80.40%	0.363 Y	IPR004843 (PFAM); IPR029052 (G3DSA:3.60.21.GENE3D); PTHR11575:SF7 (PANTHER); IPR006179 (PANTHER); IPR029052 (SUPERFAMILY)

1620	292148_length_1062_cvg_67.9_tip_1_4	1015	membrane glycoprotein lig-	354	2.97E-19	46.00%	0.670	Y	IPR003591 (SMART); IPR001611 (PFAM); G3DSA:3.80.10.10 (GENE3D); PTHR24365 (PANTHER); NON_CYTOPLASMIC_DOMAIN (PHOBIUS); TRANSMEMBRANE (PHOBIUS); CYTOPLASMIC_DOMAIN (PHOBIUS); IPR001611 (PROSITE_PROFILES); IPR001611 (PROSITE_PROFILES); IPR001611 (PROSITE_PROFILES); IPR001611 (PROSITE_PROFILES); IPR001611 (PROSITE_PROFILES); SignalP-TM (SIGNALP_GRAM_POSITIVE); SignalP-noTM (SIGNALP_GRAM_NEGATIVE); SignalP-noTM (SIGNALP_EUK); SSF52058 (SUPERFAMILY)
1622	292208_length_1064_cvg_2.2_tip_1_5	35	leucine-rich repeat-containing protein 15	354	7.93E-23	46.80%	0.327		IPR003591 (SMART); IPR001611 (PFAM); G3DSA:3.80.10.10 (GENE3D); PTHR24373 (PANTHER); PTHR24373:SF112 (PANTHER); NON_CYTOPLASMIC_DOMAIN (PHOBIUS); CYTOPLASMIC_DOMAIN (PHOBIUS); TRANSMEMBRANE (PHOBIUS); SSF52058 (SUPERFAMILY)
1621	292186_length_1063_cvg_4.9_tip_1_3	78	laccase 1	355	2.30E-76	63.10%	0.1		IPR008972 (G3DSA:2.60.40.GENE3D); IPR011706 (PFAM); PTHR11709:SF33 (PANTHER); PTHR11709 (PANTHER); IPR002355 (PROSITE_PATTERNS); IPR002355 (PROSITE_PATTERNS); IPR008972 (SUPERFAMILY)
1624	292246_length_1067_cvg_4.8_tip_1_5	63	chymotrypsinogen b isoform x2	355	4.06E-111	63.50%	0.103		IPR001314 (PRINTS); IPR001254 (SMART); G3DSA:2.40.10.10 (GENE3D); G3DSA:2.20.100.10 (GENE3D); IPR001254 (PFAM); G3DSA:2.40.10.10 (GENE3D); PTHR24265:SF76 (PANTHER); PTHR24265 (PANTHER); IPR018114 (PROSITE_PATTERNS); IPR018114 (PROSITE_PATTERNS); IPR000884 (PROSITE_PROFILES); IPR001254 (PROSITE_PROFILES); IPR009003 (SUPERFAMILY)
1625	292254_length_1067_cvg_8.6_tip_1_2	100	kielin chordin-like	355	1.62E-12	39.10%	0.105		IPR001846 (PFAM); PTHR11339 (PANTHER); IPR001846 (PROSITE_PROFILES)
1623	292236_length_1066_cvg_40.9_tip_1_0	562	apolipoprotein d	356	6.38E-09	44.60%	0.880	Y	IPR000566 (PFAM); IPR012674 (G3DSA:2.40.128.GENE3D); PTHR10612 (PANTHER); PTHR10612:SF7 (PANTHER); IPR022272 (PROSITE_PATTERNS); SIGNAL_PEPTIDE_H_REGION (PHOBIUS); TRANSMEMBRANE (PHOBIUS); SIGNAL_PEPTIDE (PHOBIUS); SIGNAL_PEPTIDE_C_REGION (PHOBIUS); SIGNAL_PEPTIDE_N_REGION (PHOBIUS); NON_CYTOPLASMIC_DOMAIN (PHOBIUS); CYTOPLASMIC_DOMAIN (PHOBIUS); SignalP-TM (SIGNALP_GRAM_POSITIVE); SignalP-noTM (SIGNALP_EUK); SignalP-noTM (SIGNALP_GRAM_NEGATIVE); IPR011038 (SUPERFAMILY); TMhelix (TMHMM)
1626	292258_length_1067_cvg_66.9_tip_1_1	234983	hemocyanin subunit type 2 precursor	356	0	72.40%	0.282		IPR013788 (PRINTS); IPR005204 (G3DSA:1.20.1370.GENE3D); IPR005204 (PFAM); IPR000896 (PFAM); IPR008922 (G3DSA:1.10.1280.GENE3D); PTHR11511:SF5 (PANTHER); IPR013788 (PANTHER); IPR013788 (PROSITE_PATTERNS); CYTOPLASMIC_DOMAIN (PHOBIUS); TRANSMEMBRANE (PHOBIUS); NON_CYTOPLASMIC_DOMAIN (PHOBIUS); IPR008922 (SUPERFAMILY); IPR005204 (SUPERFAMILY)
1627	292306_length_1069_cvg_2.0_tip_1_1	33	isoform cra_a	356	1.90E-51	57.70%	0.115		IPR015880 (SMART); IPR013087 (G3DSA:3.30.160.GENE3D); IPR013087 (G3DSA:3.30.160.GENE3D); IPR013087 (G3DSA:3.30.160.GENE3D); PF13465 (PFAM); PF13912 (PFAM); IPR013087 (G3DSA:3.30.160.GENE3D); PTHR24409 (PANTHER); IPR007087 (PROSITE_PATTERNS); IPR007087 (PROSITE_PATTERNS); IPR007087 (PROSITE_PATTERNS); IPR007087 (PROSITE_PATTERNS); IPR007087 (PROSITE_PATTERNS); IPR007087 (PROSITE_PATTERNS); IPR007087 (PROSITE_PATTERNS); IPR007087 (PROSITE_PATTERNS); IPR007087 (PROSITE_PROFILES); IPR007087 (PROSITE_PROFILES); IPR007087 (PROSITE_PROFILES); IPR007087 (PROSITE_PROFILES); IPR007087 (PROSITE_PROFILES); IPR007087 (PROSITE_PROFILES); IPR007087 (PROSITE_PROFILES); IPR007087 (PROSITE_PROFILES); SSF57667 (SUPERFAMILY); SSF57667 (SUPERFAMILY); SSF57667 (SUPERFAMILY); SSF57667 (SUPERFAMILY)
1628	292314_length_1070_cvg_2.0_tip_1_3	34	cyclin-dependent kinase 9-like	357	0	93.40%	0.183		IPR002290 (SMART); G3DSA:1.10.510.10 (GENE3D); IPR000719 (PFAM); G3DSA:3.30.200.20 (GENE3D); PTHR24056:SF158 (PANTHER); PTHR24056 (PANTHER); IPR008271 (PROSITE_PATTERNS); TRANSMEMBRANE (PHOBIUS); CYTOPLASMIC_DOMAIN (PHOBIUS); NON_CYTOPLASMIC_DOMAIN (PHOBIUS); IPR000719 (PROSITE_PROFILES); IPR011009 (SUPERFAMILY)
1629	292412_length_1074_cvg_5.5_tip_1_3	63	aael008062- partial	358	4.43E-117	90.70%	0.1		IPR013320 (G3DSA:2.60.120.GENE3D); IPR008859 (PFAM); PTHR10199 (PANTHER); IPR008859 (PROSITE_PROFILES); IPR013320 (SUPERFAMILY)
1630	292456_length_1076_cvg_7.3_tip_1_4	72	i-type lysozyme	359	1.34E-35	65.20%	0.343	Y	IPR008597 (PFAM); IPR008597 (PANTHER); IPR018247 (PROSITE_PATTERNS); NON_CYTOPLASMIC_DOMAIN (PHOBIUS); TRANSMEMBRANE (PHOBIUS); CYTOPLASMIC_DOMAIN (PHOBIUS); CYTOPLASMIC_DOMAIN (PHOBIUS); TRANSMEMBRANE (PHOBIUS); TMhelix (TMHMM)

1631	292500_length_1078_cvg_35.9_tip_1_0	495	steroid dehydrogenase	360	1.03E-116	68.80%	0.247	Coil (COILS); IPR002347 (PRINTS); IPR002198 (PRINTS); IPR002198 (PFAM); IPR016040 (G3DSA:3.40.50.GENE3D); IPR002198 (PIRSF); PTHR24316:SF68 (PANTHER); PTHR24316 (PANTHER); NON_CYTOPLASMIC_DOMAIN (PHOBIUS); CYTOPLASMIC_DOMAIN (PHOBIUS); TRANSMEMBRANE (PHOBIUS); CYTOPLASMIC_DOMAIN (PHOBIUS); NON_CYTOPLASMIC_DOMAIN (PHOBIUS); TRANSMEMBRANE (PHOBIUS); TRANSMEMBRANE (PHOBIUS); SSF51735 (SUPERFAMILY); TMhelix (TMHMM); TMhelix (TMHMM); TMhelix (TMHMM)
1632	292524_length_1080_cvg_54.0_tip_1_0	110785	hemocyanin subunit type 1 precursor	360	0	72.30%	0.202	IPR013788 (PRINTS); IPR000896 (PFAM); IPR008922 (G3DSA:1.10.1280.GENE3D); IPR005204 (PFAM); IPR005204 (G3DSA:1.20.1370.GENE3D); IPR013788 (PANTHER); PTHR11511:SF5 (PANTHER); IPR013788 (PROSITE_PATTERNS); NON_CYTOPLASMIC_DOMAIN (PHOBIUS); TRANSMEMBRANE (PHOBIUS); CYTOPLASMIC_DOMAIN (PHOBIUS); IPR005204 (SUPERFAMILY); IPR008922 (SUPERFAMILY)
1633	292538_length_1081_cvg_18.5_tip_1_1	231	serine protease snake	360	1.85E-69	61.20%	0.141	IPR001314 (PRINTS); IPR001254 (SMART); G3DSA:2.40.10.10 (GENE3D); IPR001254 (PFAM); G3DSA:2.40.10.10 (GENE3D); G3DSA:2.40.10.10 (GENE3D); PTHR24260:SF46 (PANTHER); PTHR24260 (PANTHER); IPR018114 (PROSITE_PATTERNS); IPR018114 (PROSITE_PATTERNS); IPR001254 (PROSITE_PROFILES); IPR009003 (SUPERFAMILY)
1635	292548_length_1081_cvg_16.6_tip_1_5	215	spondin-1	360	2.22E-82	60.30%	0.141	IPR002223 (PRINTS); IPR002223 (SMART); IPR000884 (SMART); IPR002223 (PFAM); G3DSA:2.20.100.10 (GENE3D); IPR000884 (PFAM); G3DSA:2.20.100.10 (GENE3D); IPR002223 (G3DSA:4.10.410.GENE3D); PTHR11311:SF11 (PANTHER); PTHR11311 (PANTHER); IPR020901 (PROSITE_PATTERNS); IPR002223 (PROSITE_PROFILES); IPR000884 (PROSITE_PROFILES); IPR000884 (PROSITE_PROFILES); IPR000884 (PROSITE_PROFILES); IPR000884 (PROSITE_PROFILES); IPR000884 (SUPERFAMILY); IPR000884 (SUPERFAMILY); IPR000884 (SUPERFAMILY); IPR002223 (SUPERFAMILY); IPR000884 (SUPERFAMILY)
1634	292546_length_1081_cvg_16.3_tip_1_0	218	superoxide dismutase	361	1.04E-63	68.70%	0.28	IPR001424 (PRINTS); IPR001424 (PFAM); IPR001424 (G3DSA:2.60.40.GENE3D); PTHR10003 (PANTHER); IPR018152 (PROSITE_PATTERNS); IPR018152 (PROSITE_PATTERNS); SIGNAL_PEPTIDE_H_REGION (PHOBIUS); SIGNAL_PEPTIDE_C_REGION (PHOBIUS); NON_CYTOPLASMIC_DOMAIN (PHOBIUS); SIGNAL_PEPTIDE (PHOBIUS); SIGNAL_PEPTIDE_N_REGION (PHOBIUS); SignalP-TM (SIGNALP_GRAM_POSITIVE); IPR001424 (SUPERFAMILY); IPR001424 (SUPERFAMILY); TMhelix (TMHMM)
1636	292716_length_1090_cvg_51.0_tip_1_1	732	eukaryotic translation initiation factor 3 subunit i-like	363	0	84.20%	0.121	IPR001680 (SMART); IPR001680 (PFAM); IPR015943 (G3DSA:2.130.10.GENE3D); PTHR19877 (PANTHER); IPR019775 (PROSITE_PATTERNS); IPR017986 (PROSITE_PROFILES); IPR001680 (PROSITE_PROFILES); IPR027525 (HAMAP); IPR001680 (PROSITE_PROFILES); IPR001680 (PROSITE_PROFILES); IPR001680 (PROSITE_PROFILES); IPR017986 (PROSITE_PROFILES); IPR017986 (SUPERFAMILY)
1637	292772_length_1093_cvg_12.8_tip_1_5	167	peroxisomal trans-2-enoyl- reductase-like	364	1.17E-126	74.10%	0.156	IPR002347 (PRINTS); PF13561 (PFAM); IPR016040 (G3DSA:3.40.50.GENE3D); PTHR24317 (PANTHER); SSF51735 (SUPERFAMILY)
1638	292808_length_1095_cvg_10.2_tip_1_5	182	acrosin- partial	365	5.64E-55	53.90%	0.395 Y	IPR001314 (PRINTS); IPR001254 (SMART); G3DSA:2.40.10.10 (GENE3D); IPR001254 (PFAM); G3DSA:2.40.10.10 (GENE3D); PTHR24265 (PANTHER); IPR018114 (PROSITE_PATTERNS); IPR018114 (PROSITE_PATTERNS); NON_CYTOPLASMIC_DOMAIN (PHOBIUS); SIGNAL_PEPTIDE_C_REGION (PHOBIUS); SIGNAL_PEPTIDE_H_REGION (PHOBIUS); SIGNAL_PEPTIDE_N_REGION (PHOBIUS); SIGNAL_PEPTIDE (PHOBIUS); IPR001254 (PROSITE_PROFILES); IPR009003 (SUPERFAMILY)
1639	292814_length_1095_cvg_9.2_tip_1_4	132	serine threonine-protein phosphatase 6 catalytic subunit	365	0	93.80%	0.102	IPR006186 (PRINTS); IPR006186 (SMART); IPR004843 (PFAM); IPR029052 (G3DSA:3.60.21.GENE3D); PTHR11668:SF24 (PANTHER); PTHR11668 (PANTHER); IPR006186 (PROSITE_PATTERNS); IPR029052 (SUPERFAMILY)
1640	292830_length_1095_cvg_75.6_tip_1_3	2404	glycosyl-phosphatidylinositol-linked carbonic anhydrase	365	2.87E-85	63.40%	0.127	IPR001148 (SMART); IPR001148 (G3DSA:3.10.200.GENE3D); IPR001148 (PFAM); IPR023561 (PANTHER); IPR018338 (PROSITE_PATTERNS); IPR001148 (PROSITE_PROFILES); IPR001148 (SUPERFAMILY)
1641	292832_length_1095_cvg_4.0_tip_1_2	51	sco- partial	365	2.71E-41	54.00%	0.107	IPR002919 (PFAM); IPR025155 (PFAM); G3DSA:2.10.25.10 (GENE3D)
1642	292836_length_1096_cvg_62.7_tip_1_2	1505	cytosolic non-specific dipeptidase	365	5.74E-140	82.00%	0.291	IPR011650 (PFAM); IPR011650 (G3DSA:3.30.70.GENE3D); G3DSA:3.40.630.10 (GENE3D); IPR002933 (PFAM); PTHR11014 (PANTHER); PTHR11014:SF51 (PANTHER); TRANSMEMBRANE (PHOBIUS); CYTOPLASMIC_DOMAIN (PHOBIUS); NON_CYTOPLASMIC_DOMAIN (PHOBIUS); SSF53187 (SUPERFAMILY); TMhelix (TMHMM)

1643	292872_length_1097_cvg_3.0_tip_1_0	49	u4 u6 small nuclear ribonucleoprotein prp31	366	0	89.50%	0.099	IPR012976 (SMART); IPR002687 (PFAM); IPR019175 (PFAM); IPR012976 (PFAM); IPR027105 (PANTHER); IPR002687 (PROSITE_PROFILES); SSF89124 (SUPERFAMILY)
1644	292882_length_1098_cvg_7.6_tip_1_3	99	beta-n-acetylglucosaminidase-like partial	366	1.28E-95	60.60%	0.827 Y	IPR025705 (PRINTS); IPR029019 (PFAM); IPR015883 (PFAM); IPR013781 (G3DSA:3.20.20.GENE3D); IPR029018 (G3DSA:3.30.379.GENE3D); PTHR22600 (PANTHER); PTHR22600:SF4 (PANTHER); SIGNAL_PEPTIDE_N_REGION (PHOBIUS); SIGNAL_PEPTIDE_C_REGION (PHOBIUS); SIGNAL_PEPTIDE_H_REGION (PHOBIUS); SIGNAL_PEPTIDE (PHOBIUS); NON_CYTOPLASMIC_DOMAIN (PHOBIUS); SignalP-TM (SIGNALP_GRAM_POSITIVE); SignalP-noTM (SIGNALP_EUK); IPR029018 (SUPERFAMILY); IPR017853 (SUPERFAMILY); TMhelix (TMHMM)
1646	292922_length_1100_cvg_66.2_tip_1_5	3789	nuclease-sensitive element-binding protein 1 isoform x4	366	1.30E-59	75.70%	0.112	IPR002059 (PRINTS); IPR011129 (SMART); IPR012340 (G3DSA:2.40.50.GENE3D); IPR002059 (PFAM); PTHR11544 (PANTHER); IPR019844 (PROSITE_PATTERNS); IPR012340 (SUPERFAMILY)
1645	292902_length_1099_cvg_14.8_tip_1_3	184	wd g-beta repeat protein	367	7.35E-111	97.80%	0.133	IPR020472 (PRINTS); IPR001680 (SMART); IPR001680 (PFAM); IPR015943 (G3DSA:2.130.10.GENE3D); IPR016346 (PANTHER); IPR019775 (PROSITE_PATTERNS); IPR019775 (PROSITE_PATTERNS); NON_CYTOPLASMIC_DOMAIN (PHOBIUS); TRANSMEMBRANE (PHOBIUS); NON_CYTOPLASMIC_DOMAIN (PHOBIUS); CYTOPLASMIC_DOMAIN (PHOBIUS); CYTOPLASMIC_DOMAIN (PHOBIUS); TRANSMEMBRANE (PHOBIUS); TRANSMEMBRANE (PHOBIUS); IPR001680 (PROSITE_PROFILES); IPR017986 (PROSITE_PROFILES); IPR001680 (PROSITE_PROFILES); IPR001680 (PROSITE_PROFILES); IPR001680 (PROSITE_PROFILES); IPR017986 (SUPERFAMILY); TMhelix (TMHMM); TMhelix (TMHMM)
1647	292936_length_1100_cvg_4.0_tip_1_0	57	---NA---	367			0.097	Coil (COILS); Coil (COILS); Coil (COILS); Coil (COILS); Coil (COILS); Coil (COILS); Coil (COILS)
1648	292938_length_1100_cvg_27.0_tip_1_4	342	3-hydroxyisobutyrate dehydrogenase	367	1.23E-64	54.90%	0.126	IPR029154 (PFAM); IPR016040 (G3DSA:3.40.50.GENE3D); IPR013328 (G3DSA:1.10.1040.GENE3D); IPR006115 (PFAM); PTHR22981 (PANTHER); PTHR22981:SF7 (PANTHER); IPR029752 (PROSITE_PATTERNS); SSF51735 (SUPERFAMILY); IPR008927 (SUPERFAMILY)
1649	292942_length_1101_cvg_31.8_tip_1_0	462	phospholipase a2-like	367	2.63E-39	61.90%	0.149	IPR016090 (G3DSA:1.20.90.GENE3D); IPR001211 (PFAM); PTHR12253 (PANTHER); IPR013090 (PROSITE_PATTERNS); IPR016090 (SUPERFAMILY)
1650	292946_length_1101_cvg_3.0_tip_1_2	44	vitellogenin fused with superoxide dismutase	367	2.31E-40	47.40%	0.153	IPR011030 (G3DSA:1.25.10.GENE3D); IPR001747 (PFAM); PTHR23345:SF1 (PANTHER); PTHR23345 (PANTHER); IPR001747 (PROSITE_PROFILES); IPR011030 (SUPERFAMILY)
1651	292976_length_1102_cvg_41.7_tip_1_3	683	serine protease1 2	368	1.62E-55	56.90%	0.783 Y	IPR001314 (PRINTS); IPR001254 (SMART); IPR001254 (PFAM); G3DSA:2.40.10.10 (GENE3D); G3DSA:2.40.10.10 (GENE3D); PTHR24260 (PANTHER); IPR018114 (PROSITE_PATTERNS); IPR018114 (PROSITE_PATTERNS); IPR001254 (PROSITE_PROFILES); SignalP-noTM (SIGNALP_EUK); IPR009003 (SUPERFAMILY)
1652	293034_length_1105_cvg_3.0_tip_1_2	46	isoform b	368	1.15E-95	63.40%	0.103	IPR015812 (PRINTS); G3DSA:2.10.25.10 (GENE3D); IPR014836 (G3DSA:1.20.5.GENE3D); G3DSA:2.10.25.10 (GENE3D); IPR012896 (PFAM); IPR014836 (PFAM); G3DSA:1.20.5.100 (GENE3D); IPR015812 (PANTHER); PTHR10082:SF16 (PANTHER); IPR015812 (PROSITE_PATTERNS); IPR015812 (PROSITE_PATTERNS); IPR015812 (PROSITE_PATTERNS); NON_CYTOPLASMIC_DOMAIN (PHOBIUS); TRANSMEMBRANE (PHOBIUS); CYTOPLASMIC_DOMAIN (PHOBIUS); SSF57196 (SUPERFAMILY); IPR012896 (SUPERFAMILY); TMhelix (TMHMM)
1653	293056_length_1105_cvg_8.2_tip_1_5	111	annexin b9-like isoform x3	368	5.56E-128	73.40%	0.107	IPR002391 (PRINTS); IPR001464 (PRINTS); IPR018502 (SMART); IPR018502 (PFAM); IPR018502 (G3DSA:1.10.220.GENE3D); IPR018502 (G3DSA:1.10.220.GENE3D); IPR018502 (G3DSA:1.10.220.GENE3D); IPR018502 (G3DSA:1.10.220.GENE3D); PTHR10502 (PANTHER); IPR018252 (PROSITE_PATTERNS); IPR018252 (PROSITE_PATTERNS); SSF47874 (SUPERFAMILY)
1654	293070_length_1106_cvg_6.5_tip_1_5	63	cytoglobin-2-like isoform 1	368	1.81E-45	61.90%	0.137	IPR002336 (PRINTS); IPR000971 (PFAM); IPR012292 (G3DSA:1.10.490.GENE3D); PTHR11442 (PANTHER); PTHR11442:SF36 (PANTHER); CYTOPLASMIC_DOMAIN (PHOBIUS); TRANSMEMBRANE (PHOBIUS); TRANSMEMBRANE (PHOBIUS); NON_CYTOPLASMIC_DOMAIN (PHOBIUS); CYTOPLASMIC_DOMAIN (PHOBIUS); TRANSMEMBRANE (PHOBIUS); NON_CYTOPLASMIC_DOMAIN (PHOBIUS); IPR000971 (PROSITE_PROFILES); IPR009050 (SUPERFAMILY); TMhelix (TMHMM); TMhelix (TMHMM); TMhelix (TMHMM)

1655	293090_length_1107_cvg_11.0_tip_1_5	140	acetylcholine receptor subunit alpha-type acr-16	369	1.30E-100	57.90%	0.133		IPR006201 (PRINTS); IPR006029 (PFAM); IPR006202 (G3DSA:2.70.170.GENE3D); IPR027361 (G3DSA:1.20.120.GENE3D); IPR006202 (PFAM); IPR006201 (PANTHER); PTHR18945:SF490 (PANTHER); TRANSMEMBRANE (PHOBIUS); NON_CYTOPLASMIC_DOMAIN (PHOBIUS); TRANSMEMBRANE (PHOBIUS); CYTOPLASMIC_DOMAIN (PHOBIUS); CYTOPLASMIC_DOMAIN (PHOBIUS); TRANSMEMBRANE (PHOBIUS); NON_CYTOPLASMIC_DOMAIN (PHOBIUS); TRANSMEMBRANE (PHOBIUS); CYTOPLASMIC_DOMAIN (PHOBIUS); IPR006029 (SUPERFAMILY); IPR006202 (SUPERFAMILY); TMhelix (TMHMM); TMhelix (TMHMM); TMhelix (TMHMM); TMhelix (TMHMM)
1656	293134_length_1110_cvg_9.9_tip_1_5	195	pacifastin light chain	370	5.44E-42	41.70%	0.401	Y	IPR006552 (SMART); IPR008037 (PFAM); IPR020862 (PROSITE_PROFILES); IPR001190 (PROSITE_PROFILES); IPR020862 (PROSITE_PROFILES); IPR020862 (PROSITE_PROFILES); IPR020862 (PROSITE_PROFILES); IPR020862 (PROSITE_PROFILES); IPR020862 (PROSITE_PROFILES); IPR020862 (PROSITE_PROFILES); IPR008037 (SUPERFAMILY); IPR008037 (SUPERFAMILY); IPR008037 (SUPERFAMILY); IPR008037 (SUPERFAMILY); IPR008037 (SUPERFAMILY); IPR008037 (SUPERFAMILY); IPR008037 (SUPERFAMILY); IPR008037 (SUPERFAMILY)
1657	293138_length_1110_cvg_3.0_tip_1_0	47	aael008062- partial	370	7.58E-156	76.10%	0.114		IPR003367 (PFAM); IPR028974 (G3DSA:4.10.1080.GENE3D); IPR013320 (G3DSA:2.60.120.GENE3D); IPR008859 (PFAM); PTHR10199 (PANTHER); PTHR10199:SF76 (PANTHER); IPR013032 (PROSITE_PATTERNS); IPR000742 (PROSITE_PROFILES); IPR017897 (PROSITE_PROFILES); IPR017897 (PROSITE_PROFILES); IPR017897 (PROSITE_PROFILES); IPR017897 (PROSITE_PROFILES); IPR017897 (PROSITE_PROFILES); IPR017897 (PROSITE_PROFILES); IPR017897 (PROSITE_PROFILES); IPR017897 (PROSITE_PROFILES); IPR008859 (PROSITE_PROFILES); IPR028974 (SUPERFAMILY); IPR013320 (SUPERFAMILY); IPR028974 (SUPERFAMILY); IPR028974 (SUPERFAMILY)
1658	293146_length_1110_cvg_3.0_tip_1_4	59	perlucin-like protein	370	4.37E-19	53.20%	0.313		IPR001304 (SMART); IPR001304 (PFAM); IPR016186 (G3DSA:3.10.100.GENE3D); PTHR22802 (PANTHER); IPR018378 (PROSITE_PATTERNS); CYTOPLASMIC_DOMAIN (PHOBIUS); CYTOPLASMIC_DOMAIN (PHOBIUS); TRANSMEMBRANE (PHOBIUS); NON_CYTOPLASMIC_DOMAIN (PHOBIUS); TRANSMEMBRANE (PHOBIUS); IPR001304 (PROSITE_PROFILES); IPR016187 (SUPERFAMILY); TMhelix (TMHMM); TMhelix (TMHMM)
1659	293168_length_1112_cvg_43.5_tip_1_5	955	zonadhesin-like isoform x1	370	4.99E-12	45.50%	0.118		IPR001846 (PFAM); NON_CYTOPLASMIC_DOMAIN (PHOBIUS); NON_CYTOPLASMIC_DOMAIN (PHOBIUS); CYTOPLASMIC_DOMAIN (PHOBIUS); TRANSMEMBRANE (PHOBIUS); TRANSMEMBRANE (PHOBIUS); IPR001846 (PROSITE_PROFILES); TMhelix (TMHMM)
1660	293190_length_1113_cvg_8.9_tip_1_3	134	inactive dipeptidyl peptidase 10	371	3.76E-99	58.80%	0.104		IPR002469 (PFAM); IPR029058 (G3DSA:3.40.50.GENE3D); IPR002469 (G3DSA:2.140.10.GENE3D); PTHR11731 (PANTHER); PTHR11731:SF93 (PANTHER); SSF82171 (SUPERFAMILY)
1661	293208_length_1113_cvg_6.1_tip_1_1	102	titin- partial	371	6.62E-20	57.80%	0.104		IPR003598 (SMART); IPR003599 (SMART); IPR013783 (G3DSA:2.60.40.GENE3D); IPR013098 (PFAM); PTHR19897 (PANTHER); IPR007110 (PROSITE_PROFILES); SSF48726 (SUPERFAMILY)
1662	293218_length_1114_cvg_7.4_tip_1_4	109	26s protease regulatory subunit 8	371	0	97.10%	0.1		Coil (COILS); IPR003593 (SMART); IPR005937 (TIGRFAM); G3DSA:1.10.8.60 (GENE3D); IPR003959 (PFAM); IPR027417 (G3DSA:3.40.50.GENE3D); PTHR23073:SF12 (PANTHER); PTHR23073 (PANTHER); IPR003960 (PROSITE_PATTERNS); IPR027417 (SUPERFAMILY)
1663	293264_length_1116_cvg_50.3_tip_1_4	814	alpha-n-acetylgalactosaminidase-like isoform x1	372	2.37E-146	87.00%	0.102		IPR002241 (PRINTS); IPR013785 (G3DSA:3.20.20.GENE3D); IPR013780 (G3DSA:2.60.40.GENE3D); PTHR11452:SF23 (PANTHER); PTHR11452 (PANTHER); IPR017853 (SUPERFAMILY); SSF51011 (SUPERFAMILY)
1664	293284_length_1117_cvg_65.3_tip_1_2	1841	epoxide hydrolase 4	372	1.76E-104	62.20%	0.091		IPR000639 (PRINTS); IPR000073 (PRINTS); IPR000073 (PFAM); IPR029058 (G3DSA:3.40.50.GENE3D); PTHR10992 (PANTHER); PTHR10992:SF721 (PANTHER); NON_CYTOPLASMIC_DOMAIN (PHOBIUS); CYTOPLASMIC_DOMAIN (PHOBIUS); TRANSMEMBRANE (PHOBIUS); IPR029058 (SUPERFAMILY); TMhelix (TMHMM)
1665	293326_length_1119_cvg_9.0_tip_1_1	124	acyl- delta desaturase	373	1.73E-159	76.60%	0.204		IPR015876 (PRINTS); IPR005804 (PFAM); PTHR11351 (PANTHER); PTHR11351:SF28 (PANTHER); CYTOPLASMIC_DOMAIN (PHOBIUS); CYTOPLASMIC_DOMAIN (PHOBIUS); CYTOPLASMIC_DOMAIN (PHOBIUS); TRANSMEMBRANE (PHOBIUS); TRANSMEMBRANE (PHOBIUS); NON_CYTOPLASMIC_DOMAIN (PHOBIUS); TRANSMEMBRANE (PHOBIUS); NON_CYTOPLASMIC_DOMAIN (PHOBIUS); TRANSMEMBRANE (PHOBIUS); TMhelix (TMHMM); TMhelix (TMHMM); TMhelix (TMHMM); TMhelix (TMHMM)

1666	293364_length_1121_cvg_27.9_tip_1_2	402	serine protease	373	2.54E-39	52.60%	0.31	IPR001314 (PRINTS); IPR001254 (SMART); G3DSA:2.40.10.10 (GENE3D); G3DSA:2.40.10.10 (GENE3D); IPR001254 (PFAM); PTHR24256 (PANTHER); IPR001254 (PROSITE_PROFILES); IPR009003 (SUPERFAMILY)
1667	293454_length_1126_cvg_4.2_tip_1_4	73	lipophorin precursor	375	2.29E-68	52.10%	0.209	IPR009454 (PFAM); IPR015255 (PFAM); IPR015817 (G3DSA:2.20.50.GENE3D); IPR015818 (G3DSA:2.20.80.GENE3D); PTHR23345 (PANTHER); IPR015819 (SUPERFAMILY)
1668	293476_length_1127_cvg_5.1_tip_1_3	76	low quality protein: leucine-rich repeat-containing protein ddb_g0290503	376	4.59E-60	59.10%	0.096	Coil (COILS); Coil (COILS); Coil (COILS); Coil (COILS); Coil (COILS); Coil (COILS)
1669	293482_length_1128_cvg_6.1_tip_1_5	86	transcription initiation factor tfiid subunit 5	376	0	84.50%	0.13	IPR020472 (PRINTS); IPR001680 (SMART); IPR015943 (G3DSA:2.130.10.GENE3D); IPR001680 (PFAM); PTHR19879 (PANTHER); PTHR19879:SF1 (PANTHER); IPR019775 (PROSITE_PATTERNS); IPR019775 (PROSITE_PATTERNS); IPR001680 (PROSITE_PROFILES); IPR017986 (PROSITE_PROFILES); IPR001680 (PROSITE_PROFILES); IPR001680 (PROSITE_PROFILES); IPR001680 (PROSITE_PROFILES); IPR001680 (PROSITE_PROFILES); IPR017986 (SUPERFAMILY)
1671	293522_length_1129_cvg_11.9_tip_1_4	211	low quality protein: selenoprotein h	376	5.14E-20	65.40%	0.107	IPR020478 (PRINTS); IPR017956 (SMART); IPR012336 (G3DSA:3.40.30.GENE3D); IPR011893 (TIGRFAM); IPR011893 (PFAM); IPR017956 (PFAM); IPR012336 (SUPERFAMILY)
1670	293514_length_1129_cvg_5.2_tip_1_0	66	zinc finger protein 467	377	5.94E-17	42.80%	0.096	IPR015880 (SMART); PF13912 (PFAM); IPR013087 (G3DSA:3.30.160.GENE3D); IPR013087 (G3DSA:3.30.160.GENE3D); PF13894 (PFAM); PTHR24409 (PANTHER); IPR007087 (PROSITE_PATTERNS); IPR007087 (PROSITE_PATTERNS); IPR007087 (PROSITE_PATTERNS); IPR007087 (PROSITE_PATTERNS); IPR007087 (PROSITE_PROFILES); IPR007087 (PROSITE_PROFILES); IPR007087 (PROSITE_PROFILES); IPR007087 (PROSITE_PROFILES); SSF57802 (SUPERFAMILY); SSF57667 (SUPERFAMILY)
1672	293538_length_1130_cvg_8.0_tip_1_4	106	neuroparsin-like protein	377	1.26E-18	61.30%	0.121	IPR010850 (PFAM); IPR011390 (PANTHER); NON_CYTOPLASMIC_DOMAIN (PHOBIUS); CYTOPLASMIC_DOMAIN (PHOBIUS); CYTOPLASMIC_DOMAIN (PHOBIUS); TRANSMEMBRANE (PHOBIUS); TRANSMEMBRANE (PHOBIUS); NON_CYTOPLASMIC_DOMAIN (PHOBIUS); TRANSMEMBRANE (PHOBIUS); IPR010850 (PRODOM); IPR009030 (SUPERFAMILY); TMhelix (TMHMM); TMhelix (TMHMM)
1673	293556_length_1132_cvg_29.4_tip_1_0	367	dehydrogenase reductase sdr family member 7	378	3.75E-109	67.50%	0.922 Y	IPR002198 (PRINTS); IPR002347 (PRINTS); IPR002198 (PFAM); IPR016040 (G3DSA:3.40.50.GENE3D); PTHR24322 (PANTHER); PTHR24322:SF31 (PANTHER); CYTOPLASMIC_DOMAIN (PHOBIUS); SIGNAL_PEPTIDE (PHOBIUS); SIGNAL_PEPTIDE_C_REGION (PHOBIUS); NON_CYTOPLASMIC_DOMAIN (PHOBIUS); SIGNAL_PEPTIDE_N_REGION (PHOBIUS); SIGNAL_PEPTIDE_H_REGION (PHOBIUS); TRANSMEMBRANE (PHOBIUS); SignalP-noTM (SIGNALP_EUK); SSF51735 (SUPERFAMILY); TMhelix (TMHMM); TMhelix (TMHMM)
1674	293566_length_1132_cvg_48.3_tip_1_0	590	low quality protein: galectin-4	378	1.06E-51	50.50%	0.162	IPR001079 (SMART); IPR001079 (SMART); IPR013320 (G3DSA:2.60.120.GENE3D); IPR013320 (G3DSA:2.60.120.GENE3D); IPR001079 (PFAM); PTHR11346 (PANTHER); CYTOPLASMIC_DOMAIN (PHOBIUS); TRANSMEMBRANE (PHOBIUS); NON_CYTOPLASMIC_DOMAIN (PHOBIUS); IPR001079 (PROSITE_PROFILES); IPR001079 (PROSITE_PROFILES); IPR013320 (SUPERFAMILY); IPR013320 (SUPERFAMILY); TMhelix (TMHMM)
1675	293572_length_1133_cvg_2.4_tip_1_3	44	ankyrin repeat protein	378	1.34E-18	49.20%	0.107	IPR002110 (SMART); IPR020683 (G3DSA:1.25.40.GENE3D); IPR020683 (G3DSA:1.25.40.GENE3D); PF13637 (PFAM); IPR020683 (PFAM); PTHR24198 (PANTHER); IPR002110 (PROSITE_PROFILES); IPR020683 (PROSITE_PROFILES); IPR020683 (SUPERFAMILY)
1676	293602_length_1134_cvg_28.4_tip_1_1	375	late trypsin	378	5.25E-49	55.70%	0.897 Y	IPR001314 (PRINTS); IPR001254 (SMART); G3DSA:2.40.10.10 (GENE3D); G3DSA:2.40.10.10 (GENE3D); IPR001254 (PFAM); G3DSA:2.40.10.10 (GENE3D); PTHR24268 (PANTHER); IPR018114 (PROSITE_PATTERNS); IPR018114 (PROSITE_PATTERNS); SIGNAL_PEPTIDE_H_REGION (PHOBIUS); SIGNAL_PEPTIDE (PHOBIUS); CYTOPLASMIC_DOMAIN (PHOBIUS); SIGNAL_PEPTIDE_N_REGION (PHOBIUS); TRANSMEMBRANE (PHOBIUS); SIGNAL_PEPTIDE_C_REGION (PHOBIUS); NON_CYTOPLASMIC_DOMAIN (PHOBIUS); IPR001254 (PROSITE_PROFILES); SignalP-TM (SIGNALP_GRAM_POSITIVE); SignalP-noTM (SIGNALP_EUK); SignalP-noTM (SIGNALP_GRAM_NEGATIVE); IPR009003 (SUPERFAMILY)
1678	293632_length_1135_cvg_2.6_tip_1_4	38	cyclin-h	378	7.46E-158	81.70%	0.103	IPR013763 (SMART); IPR013763 (G3DSA:1.10.472.GENE3D); IPR006671 (PFAM); IPR027081 (TIGRFAM); IPR013763 (G3DSA:1.10.472.GENE3D); IPR027081 (PTHR10026:PANTHER); IPR015429 (PANTHER); IPR013763 (SUPERFAMILY); IPR013763 (SUPERFAMILY)

1677	293622_length_1135_cvg_3.8_tip_1_0	62	ste20-related kinase adapter protein alpha	379	1.99E-142	66.20%	0.1	G3DSA:1.10.510.10 (GENE3D); G3DSA:3.30.200.20 (GENE3D); IPR000719 (PFAM); PTHR24361:SF273 (PANTHER); PTHR24361 (PANTHER); IPR000719 (PROSITE_PROFILES); IPR011009 (SUPERFAMILY)
1679	293646_length_1136_cvg_3.9_tip_1_4	53	protein takeout	379	5.16E-69	57.10%	0.12	IPR010562 (SMART); IPR010562 (PFAM); PTHR11008:SF9 (PANTHER); PTHR11008 (PANTHER)
1680	293660_length_1138_cvg_54.0_tip_1_5	1731	granulins precursor	379	7.73E-45	56.00%	0.152	IPR000118 (SMART); IPR000118 (PFAM); PTHR12274:SF1 (PANTHER); PTHR12274 (PANTHER); IPR000118 (PROSITE_PATTERNS); IPR000118 (PROSITE_PATTERNS); SSF57277 (SUPERFAMILY); SSF57277 (SUPERFAMILY)
1681	293692_length_1140_cvg_15.2_tip_1_2	224	lysosomal acid phosphatase	380	7.10E-118	66.50%	0.585 Y	IPR029033 (G3DSA:3.40.50.GENE3D); IPR000560 (PFAM); PTHR11567 (PANTHER); PTHR11567:SF26 (PANTHER); IPR000560 (PROSITE_PATTERNS); IPR000560 (PROSITE_PATTERNS); NON_CYTOPLASMIC_DOMAIN (PHOBIUS); SIGNAL_PEPTIDE_C_REGION (PHOBIUS); SIGNAL_PEPTIDE (PHOBIUS); SIGNAL_PEPTIDE_H_REGION (PHOBIUS); SIGNAL_PEPTIDE_N_REGION (PHOBIUS); SignalP-noTM (SIGNALP_GRAM_NEGATIVE); SignalP-noTM (SIGNALP_EUK); IPR029033 (SUPERFAMILY)
1682	293714_length_1141_cvg_2.3_tip_1_3	42	s-antigen partial	381	1.27E-34	62.00%	0.111	Coil (COILS); Coil (COILS); Coil (COILS)
1683	293792_length_1147_cvg_62.0_tip_1_4	4870	transmembrane protease serine 13	382	8.97E-11	47.90%	0.133	IPR001254 (SMART); IPR001254 (PFAM); G3DSA:2.40.10.10 (GENE3D); TRANSMEMBRANE (PHOBIUS); NON_CYTOPLASMIC_DOMAIN (PHOBIUS); CYTOPLASMIC_DOMAIN (PHOBIUS); IPR001254 (PROSITE_PROFILES); IPR009003 (SUPERFAMILY); TMhelix (TMHMM)
1684	293818_length_1149_cvg_5.0_tip_1_2	87	mam and ldl-receptor class a domain-containing protein c10orf112-like	383	1.93E-07	51.00%	0.111	IPR000998 (SMART); IPR000998 (PFAM); PTHR23282 (PANTHER); TRANSMEMBRANE (PHOBIUS); CYTOPLASMIC_DOMAIN (PHOBIUS); NON_CYTOPLASMIC_DOMAIN (PHOBIUS); IPR000998 (PROSITE_PROFILES); IPR013320 (SUPERFAMILY); TMhelix (TMHMM)
1685	293848_length_1151_cvg_71.6_tip_1_5	1886	zinc metalloproteinase nas-4-like	383	7.18E-50	58.70%	0.21	IPR001506 (PRINTS); IPR002557 (SMART); IPR006026 (SMART); IPR002557 (PFAM); IPR024079 (G3DSA:3.40.390.GENE3D); IPR001506 (PFAM); IPR002557 (G3DSA:2.170.140.GENE3D); PTHR10127 (PANTHER); NON_CYTOPLASMIC_DOMAIN (PHOBIUS); TRANSMEMBRANE (PHOBIUS); CYTOPLASMIC_DOMAIN (PHOBIUS); NON_CYTOPLASMIC_DOMAIN (PHOBIUS); TRANSMEMBRANE (PHOBIUS); IPR002557 (PROSITE_PROFILES); IPR002557 (SUPERFAMILY); SSF55486 (SUPERFAMILY); TMhelix (TMHMM)
1686	293910_length_1154_cvg_6.7_tip_1_2	95	tyrosine-protein kinase src64b	384	2.92E-141	73.70%	0.099	IPR000980 (PRINTS); IPR001452 (PRINTS); IPR020635 (SMART); IPR001452 (SMART); IPR000980 (SMART); IPR001452 (PFAM); G3DSA:3.30.200.20 (GENE3D); IPR000980 (G3DSA:3.30.505.GENE3D); IPR000980 (PFAM); G3DSA:2.30.30.40 (GENE3D); IPR001245 (PFAM); PTHR24418:SF35 (PANTHER); PTHR24418 (PANTHER); IPR017441 (PROSITE_PATTERNS); IPR000980 (PROSITE_PROFILES); IPR001452 (PROSITE_PROFILES); IPR000719 (PROSITE_PROFILES); IPR011009 (SUPERFAMILY); IPR000980 (SUPERFAMILY); IPR001452 (SUPERFAMILY)
1687	293930_length_1155_cvg_18.5_tip_1_1	257	multicopper oxidase	385	1.49E-55	63.80%	0.101	IPR008972 (G3DSA:2.60.40.GENE3D); IPR011706 (PFAM); PTHR11709 (PANTHER); PTHR11709:SF33 (PANTHER); IPR002355 (PROSITE_PATTERNS); TRANSMEMBRANE (PHOBIUS); TRANSMEMBRANE (PHOBIUS); CYTOPLASMIC_DOMAIN (PHOBIUS); CYTOPLASMIC_DOMAIN (PHOBIUS); NON_CYTOPLASMIC_DOMAIN (PHOBIUS); IPR008972 (SUPERFAMILY); TMhelix (TMHMM); TMhelix (TMHMM)
1688	293960_length_1157_cvg_12.7_tip_1_5	167	thioredoxin-like protein 1	385	5.73E-154	81.30%	0.132	PR00421 (PRINTS); IPR012336 (G3DSA:3.40.30.GENE3D); IPR010400 (G3DSA:2.60.120.GENE3D); IPR013766 (PFAM); IPR010400 (PFAM); IPR005746 (PANTHER); IPR017937 (PROSITE_PATTERNS); IPR010400 (PROSITE_PROFILES); IPR012336 (PROSITE_PROFILES); IPR008979 (SUPERFAMILY); IPR012336 (SUPERFAMILY)
1689	293970_length_1158_cvg_7.8_tip_1_3	105	ankyrin repeat protein	386	3.81E-17	44.40%	0.129	IPR002110 (PRINTS); IPR002110 (SMART); IPR020683 (G3DSA:1.25.40.GENE3D); IPR002110 (PFAM); IPR020683 (PFAM); PTHR24198 (PANTHER); IPR002110 (PROSITE_PROFILES); IPR002110 (PROSITE_PROFILES); IPR020683 (PROSITE_PROFILES); IPR002110 (PROSITE_PROFILES); IPR002110 (PROSITE_PROFILES); IPR020683 (SUPERFAMILY)
1690	293992_length_1159_cvg_36.3_tip_1_0	552	mite allergen lep d partial	387	4.50E-11	46.50%	0.514 Y	PTHR11008 (PANTHER); PTHR11008:SF13 (PANTHER); NON_CYTOPLASMIC_DOMAIN (PHOBIUS); TRANSMEMBRANE (PHOBIUS); CYTOPLASMIC_DOMAIN (PHOBIUS); SignalP-noTM (SIGNALP_EUK); TMhelix (TMHMM)
1691	294052_length_1163_cvg_5.2_tip_1_1	78	dual specificity mitogen-activated protein kinase kinase 4-like	388	1.41E-154	91.20%	0.114	IPR002290 (SMART); IPR000719 (PFAM); G3DSA:3.30.200.20 (GENE3D); G3DSA:1.10.510.10 (GENE3D); PTHR24360:SF46 (PANTHER); PTHR24360 (PANTHER); IPR008271 (PROSITE_PATTERNS); IPR000719 (PROSITE_PROFILES); IPR011009 (SUPERFAMILY)

1692	294068_length_1164_cvg_5.9_tip_1_4	91	spermatogenesis-associated protein 20	388	7.60E-173	72.20%	0.099	IPR004879 (PFAM); IPR012341 (G3DSA:1.50.10.GENE3D); PTHR12145:SF11 (PANTHER); PTHR12145 (PANTHER); IPR008928 (SUPERFAMILY)
1693	294072_length_1164_cvg_12.4_tip_1_0	149	apolipoprotein d-like	388	6.95E-31	65.20%	0.144	IPR012674 (G3DSA:2.40.128.GENE3D); IPR000566 (PFAM); PTHR10612 (PANTHER); CYTOPLASMIC_DOMAIN (PHOBIUS); NON_CYTOPLASMIC_DOMAIN (PHOBIUS); TRANSMEMBRANE (PHOBIUS); IPR011038 (SUPERFAMILY); TMhelix (TMHMM); TMhelix (TMHMM); TMhelix (TMHMM)
1694	294104_length_1165_cvg_47.2_tip_1_3	745	prophenoloxidase activating factor	389	2.88E-130	67.70%	0.12	IPR001314 (PRINTS); IPR001254 (SMART); IPR001254 (PFAM); G3DSA:2.40.10.10 (GENE3D); G3DSA:2.40.10.10 (GENE3D); G3DSA:2.40.10.10 (GENE3D); PTHR24268:SF95 (PANTHER); PTHR24268 (PANTHER); IPR018114 (PROSITE_PATTERNS); IPR001254 (PROSITE_PROFILES); IPR009003 (SUPERFAMILY)
1695	294114_length_1166_cvg_3.6_tip_1_0	87	er membrane protein complex subunit 10	389	2.28E-73	63.10%	0.672 Y	IPR029615 (PTHR21397:PANTHER); PTHR21397 (PANTHER); TRANSMEMBRANE (PHOBIUS); TRANSMEMBRANE (PHOBIUS); CYTOPLASMIC_DOMAIN (PHOBIUS); TRANSMEMBRANE (PHOBIUS); CYTOPLASMIC_DOMAIN (PHOBIUS); NON_CYTOPLASMIC_DOMAIN (PHOBIUS); CYTOPLASMIC_DOMAIN (PHOBIUS); NON_CYTOPLASMIC_DOMAIN (PHOBIUS); TRANSMEMBRANE (PHOBIUS); SignalP-noTM (SIGNALP_EUK); TMhelix (TMHMM); TMhelix (TMHMM); TMhelix (TMHMM); TMhelix (TMHMM)
1696	294130_length_1167_cvg_22.8_tip_1_2	307	dnaj homolog subfamily b member 6-like	389	5.36E-77	67.80%	0.214	Coil (COILS); IPR001623 (PRINTS); IPR001623 (SMART); IPR001623 (PFAM); IPR001623 (G3DSA:1.10.287.GENE3D); PTHR24077:SF225 (PANTHER); PTHR24077 (PANTHER); IPR018253 (PROSITE_PATTERNS); IPR001623 (PROSITE_PROFILES); IPR001623 (SUPERFAMILY)
1698	294154_length_1168_cvg_70.1_tip_0_1	3947	twitchin isoform x2	389	4.22E-139	76.40%	0.101	IPR003598 (SMART); IPR003599 (SMART); IPR013783 (G3DSA:2.60.40.GENE3D); IPR013783 (G3DSA:2.60.40.GENE3D); IPR013098 (PFAM); IPR013783 (G3DSA:2.60.40.GENE3D); PTHR19897 (PANTHER); IPR007110 (PROSITE_PROFILES); IPR007110 (PROSITE_PROFILES); IPR007110 (PROSITE_PROFILES); SSF48726 (SUPERFAMILY); SSF48726 (SUPERFAMILY); SSF48726 (SUPERFAMILY)
1697	294150_length_1168_cvg_5.0_tip_1_3	75	subtilisin-like protease spc6a	390	1.83E-64	59.60%	0.378 Y	IPR002884 (PFAM); IPR000209 (PFAM); IPR000209 (G3DSA:3.40.50.GENE3D); IPR008979 (G3DSA:2.60.120.GENE3D); IPR015500 (PANTHER); PTHR10795:SF340 (PANTHER); IPR023828 (PROSITE_PATTERNS); IPR000209 (SUPERFAMILY); IPR008979 (SUPERFAMILY)
1699	294174_length_1170_cvg_44.4_tip_1_3	650	meprin a subunit beta	390	6.47E-51	58.20%	0.199	IPR001506 (PRINTS); IPR006026 (SMART); IPR001506 (PFAM); IPR024079 (G3DSA:3.40.390.GENE3D); PTHR10127 (PANTHER); SSF55486 (SUPERFAMILY)
1700	294306_length_1179_cvg_4.9_tip_1_1	60	low quality protein: titin-like	393	2.38E-180	81.00%	0.099	IPR003599 (SMART); IPR003598 (SMART); IPR010939 (PFAM); IPR013783 (G3DSA:2.60.40.GENE3D); IPR013098 (PFAM); IPR013783 (G3DSA:2.60.40.GENE3D); PTHR19897 (PANTHER); PTHR19897:SF149 (PANTHER); IPR007110 (PROSITE_PROFILES); IPR007110 (PROSITE_PROFILES); IPR007110 (PROSITE_PROFILES); SSF48726 (SUPERFAMILY); SSF48726 (SUPERFAMILY); SSF48726 (SUPERFAMILY)
1701	294342_length_1182_cvg_14.8_tip_1_4	207	gpi-anchor transamidase	394	0	85.60%	0.781 Y	IPR001096 (PRINTS); IPR001096 (PIRSF); IPR028361 (PIRSF); IPR001096 (PFAM); IPR001096 (PANTHER); IPR028361 (PTHR12000:PANTHER); SIGNAL_PEPTIDE_N_REGION (PHOBIUS); NON_CYTOPLASMIC_DOMAIN (PHOBIUS); SIGNAL_PEPTIDE (PHOBIUS); SIGNAL_PEPTIDE_C_REGION (PHOBIUS); SIGNAL_PEPTIDE_H_REGION (PHOBIUS); SignalP-noTM (SIGNALP_GRAM_NEGATIVE); SignalP-TM (SIGNALP_GRAM_POSITIVE); SignalP-noTM (SIGNALP_EUK)
1702	294350_length_1182_cvg_68.3_tip_1_0	1245	kazal-type serine protease inhibitor 1 serpin	394	4.64E-10	61.00%	0.868 Y	IPR002350 (SMART); G3DSA:3.30.60.30 (GENE3D); IPR002350 (PFAM); IPR002350 (PROSITE_PATTERNS); SIGNAL_PEPTIDE_N_REGION (PHOBIUS); CYTOPLASMIC_DOMAIN (PHOBIUS); CYTOPLASMIC_DOMAIN (PHOBIUS); TRANSMEMBRANE (PHOBIUS); SIGNAL_PEPTIDE_C_REGION (PHOBIUS); NON_CYTOPLASMIC_DOMAIN (PHOBIUS); TRANSMEMBRANE (PHOBIUS); SIGNAL_PEPTIDE (PHOBIUS); SIGNAL_PEPTIDE_H_REGION (PHOBIUS); NON_CYTOPLASMIC_DOMAIN (PHOBIUS); TRANSMEMBRANE (PHOBIUS); IPR002350 (PROSITE_PROFILES); SignalP-noTM (SIGNALP_EUK); SSF100895 (SUPERFAMILY); TMhelix (TMHMM); TMhelix (TMHMM); TMhelix (TMHMM); TMhelix (TMHMM); TMhelix (TMHMM)

1703	294368_length_1183_cvg_37.9_tip_1_3	592	md-2-related lipid-recognition	395	9.15E-17	50.50%	0.401 Y	IPR003172 (SMART); IPR003172 (PFAM); IPR003172 (G3DSA:2.60.40.GENE3D); PTHR11306 (PANTHER); TRANSMEMBRANE (PHOBIUS); CYTOPLASMIC_DOMAIN (PHOBIUS); NON_CYTOPLASMIC_DOMAIN (PHOBIUS); TRANSMEMBRANE (PHOBIUS); NON_CYTOPLASMIC_DOMAIN (PHOBIUS); TRANSMEMBRANE (PHOBIUS); TRANSMEMBRANE (PHOBIUS); CYTOPLASMIC_DOMAIN (PHOBIUS); CYTOPLASMIC_DOMAIN (PHOBIUS); IPR014756 (SUPERFAMILY)
1704	294406_length_1187_cvg_3.7_tip_1_1	62	huga_polan ame: full=hyaluronidase short=hya ame: full=hyaluronoglucosaminidase ame: allergen=pol a 2 flags: partial	396	1.73E-72	57.00%	0.407 Y	IPR001329 (PRINTS); IPR018155 (PRINTS); IPR018155 (PFAM); IPR013785 (G3DSA:3.20.20.GENE3D); IPR018155 (PANTHER); PTHR11769:SF8 (PANTHER); SIGNAL_PEPTIDE (PHOBIUS); SIGNAL_PEPTIDE_C_REGION (PHOBIUS); SIGNAL_PEPTIDE_H_REGION (PHOBIUS); SIGNAL_PEPTIDE_N_REGION (PHOBIUS); NON_CYTOPLASMIC_DOMAIN (PHOBIUS); IPR017853 (SUPERFAMILY)
1705	294414_length_1188_cvg_5.0_tip_1_3	116	hyaluronidase precursor	396	4.70E-80	60.40%	0.287	IPR001329 (PRINTS); IPR018155 (PRINTS); IPR018155 (PFAM); IPR013785 (G3DSA:3.20.20.GENE3D); PTHR11769:SF8 (PANTHER); IPR018155 (PANTHER); NON_CYTOPLASMIC_DOMAIN (PHOBIUS); CYTOPLASMIC_DOMAIN (PHOBIUS); TRANSMEMBRANE (PHOBIUS); IPR017853 (SUPERFAMILY); TMhelix (TMHMM)
1706	294424_length_1188_cvg_12.0_tip_1_2	185	titin isoform x1	396	9.38E-36	45.30%	0.103	Coil (COILS); Coil (COILS); Coil (COILS); IPR003599 (SMART); IPR003598 (SMART); IPR013783 (G3DSA:2.60.40.GENE3D); IPR013098 (PFAM); PTHR19897 (PANTHER); IPR007110 (PROSITE_PROFILES); SSF48726 (SUPERFAMILY)
1707	294510_length_1194_cvg_27.0_tip_1_1	406	26s protease regulatory subunit 10b	398	0	96.70%	0.097	Coil (COILS); IPR003593 (SMART); IPR027417 (G3DSA:3.40.50.GENE3D); IPR003959 (PFAM); G3DSA:1.10.8.60 (GENE3D); IPR005937 (TIGRFAM); PTHR23073 (PANTHER); PTHR23073:SF24 (PANTHER); IPR003960 (PROSITE_PATTERNS); IPR027417 (SUPERFAMILY)
1708	294540_length_1196_cvg_33.5_tip_1_5	599	hemagglutinin proteinase	398	1.25E-43	48.80%	0.128	IPR023612 (PRINTS); IPR013856 (G3DSA:3.10.170.GENE3D); IPR011096 (PFAM); IPR025711 (PFAM); IPR013856 (PFAM); TRANSMEMBRANE (PHOBIUS); NON_CYTOPLASMIC_DOMAIN (PHOBIUS); CYTOPLASMIC_DOMAIN (PHOBIUS); SSF55486 (SUPERFAMILY)
1709	294542_length_1196_cvg_30.3_tip_1_1	467	agap005608-pa-like protein	399	1.72E-147	76.40%	0.131	Coil (COILS); Coil (COILS); IPR011992 (PFAM); IPR011992 (G3DSA:1.10.238.GENE3D); PTHR19237 (PANTHER); PTHR19237:SF20 (PANTHER); IPR018247 (PROSITE_PATTERNS); CYTOPLASMIC_DOMAIN (PHOBIUS); NON_CYTOPLASMIC_DOMAIN (PHOBIUS); TRANSMEMBRANE (PHOBIUS); IPR002048 (PROSITE_PROFILES); IPR002048 (PROSITE_PROFILES); SSF47473 (SUPERFAMILY); TMhelix (TMHMM)
1710	294602_length_1201_cvg_7.5_tip_1_5	123	3-hydroxyisobutyrate dehydrogenase	400	8.41E-78	57.20%	0.394 Y	IPR013328 (G3DSA:1.10.1040.GENE3D); IPR006115 (PFAM); IPR029154 (PFAM); IPR016040 (G3DSA:3.40.50.GENE3D); PTHR22981:SF7 (PANTHER); PTHR22981 (PANTHER); IPR029752 (PROSITE_PATTERNS); IPR008927 (SUPERFAMILY); SSF51735 (SUPERFAMILY)
1711	294622_length_1202_cvg_61.5_tip_1_2	2513	guanine nucleotide-binding protein subunit beta-like protein	400	0	93.10%	0.136	IPR020472 (PRINTS); IPR001680 (SMART); IPR015943 (G3DSA:2.130.10.GENE3D); IPR001680 (PFAM); IPR015943 (G3DSA:2.130.10.GENE3D); PTHR19868:SF0 (PANTHER); PTHR19868 (PANTHER); IPR019775 (PROSITE_PATTERNS); IPR019775 (PROSITE_PATTERNS); IPR019775 (PROSITE_PATTERNS); IPR019775 (PROSITE_PATTERNS); IPR001680 (PROSITE_PROFILES); IPR001680 (PROSITE_PROFILES); IPR001680 (PROSITE_PROFILES); IPR001680 (PROSITE_PROFILES); IPR001680 (PROSITE_PROFILES); IPR001680 (PROSITE_PROFILES); IPR017986 (PROSITE_PROFILES); IPR017986 (SUPERFAMILY)
1712	294624_length_1202_cvg_41.8_tip_1_5	653	mitochondrial matrix protein p33	400	3.87E-86	67.40%	0.14	IPR003428 (G3DSA:3.10.280.GENE3D); IPR003428 (PFAM); PTHR10826 (PANTHER); PTHR10826:SF1 (PANTHER); IPR003428 (SUPERFAMILY)
1713	294656_length_1204_cvg_2.9_tip_1_1	50	neural cell adhesion molecule 1 isoform x1	401	4.14E-50	52.60%	0.111	IPR003598 (SMART); IPR003599 (SMART); IPR013098 (PFAM); IPR013783 (G3DSA:2.60.40.GENE3D); IPR013783 (G3DSA:2.60.40.GENE3D); PTHR19831 (PANTHER); PTHR19831:SF40 (PANTHER); CYTOPLASMIC_DOMAIN (PHOBIUS); TRANSMEMBRANE (PHOBIUS); NON_CYTOPLASMIC_DOMAIN (PHOBIUS); IPR003961 (PROSITE_PROFILES); IPR007110 (PROSITE_PROFILES); IPR007110 (PROSITE_PROFILES); IPR003961 (SUPERFAMILY); SSF48726 (SUPERFAMILY); SSF48726 (SUPERFAMILY); TMhelix (TMHMM)
1721	294834_length_1216_cvg_27.1_tip_1_1	382	serine threonine-protein phosphatase 2a catalytic subunit alpha isoform	401	0	97.60%	0.176	Coil (COILS); IPR006186 (PRINTS); IPR006186 (SMART); IPR004843 (PFAM); IPR029052 (G3DSA:3.60.21.GENE3D); PTHR11668:SF216 (PANTHER); PTHR11668 (PANTHER); IPR006186 (PROSITE_PATTERNS); IPR029052 (SUPERFAMILY)
1714	294680_length_1206_cvg_62.6_tip_1_3	1475	hypothetical protein DAPPUDRAFT_250423	402	3.06E-79	49.90%	0.112	IPR005320 (PFAM); IPR029062 (G3DSA:3.40.50.GENE3D); IPR029062 (SUPERFAMILY)

1715	294690_length_1206_cvg_2.0_tip_1_5	45	low-density lipoprotein receptor class a domain-containing protein 3	402	1.41E-62	53.50%	0.107		IPR002172 (SMART); IPR002172 (G3DSA:4.10.400.GENE3D); IPR002172 (PFAM); IPR023415 (PROSITE_PATTERNS); CYTOPLASMIC_DOMAIN (PHOBIUS); NON_CYTOPLASMIC_DOMAIN (PHOBIUS); TRANSMEMBRANE (PHOBIUS); IPR002172 (PROSITE_PROFILES); IPR002172 (SUPERFAMILY); TMhelix (TMHMM)
1716	294712_length_1209_cvg_6.4_tip_1_0	118	arylsulfatase b	403	4.29E-99	68.10%	0.165		G3DSA:3.30.1120.10 (GENE3D); IPR000917 (PFAM); IPR017849 (G3DSA:3.40.720.GENE3D); PTHR10342:SF189 (PANTHER); PTHR10342 (PANTHER); IPR017850 (SUPERFAMILY)
1717	294716_length_1209_cvg_5.0_tip_1_3	74	ras-responsive element-binding protein 1	403	1.28E-103	64.50%	0.199		IPR015880 (SMART); IPR013087 (G3DSA:3.30.160.GENE3D); PF13465 (PFAM); IPR007087 (PFAM); IPR013087 (G3DSA:3.30.160.GENE3D); IPR013087 (G3DSA:3.30.160.GENE3D); IPR013087 (G3DSA:3.30.160.GENE3D); PF13894 (PFAM); PTHR23233:SF42 (PANTHER); PTHR23233 (PANTHER); IPR007087 (PROSITE_PATTERNS); IPR007087 (PROSITE_PATTERNS); IPR007087 (PROSITE_PATTERNS); IPR007087 (PROSITE_PATTERNS); IPR007087 (PROSITE_PATTERNS); IPR007087 (PROSITE_PATTERNS); IPR007087 (PROSITE_PROFILES); IPR007087 (PROSITE_PROFILES); IPR007087 (PROSITE_PROFILES); IPR007087 (PROSITE_PROFILES); IPR007087 (PROSITE_PROFILES); SSF57667 (SUPERFAMILY); SSF57667 (SUPERFAMILY); SSF57667 (SUPERFAMILY); SSF57667 (SUPERFAMILY)
1718	294804_length_1214_cvg_3.7_tip_1_1	64	laminin subunit alpha	405	1.03E-89	57.20%	0.101		Coil (COILS); IPR013320 (G3DSA:2.60.120.GENE3D); IPR010307 (PFAM); PTHR10574:SF39 (PANTHER); PTHR10574 (PANTHER); IPR013320 (SUPERFAMILY); SSF58100 (SUPERFAMILY)
1719	294810_length_1214_cvg_10.1_tip_1_1	157	angiotensin-converting enzyme-like	405	1.48E-158	73.20%	0.847	Y	IPR001548 (PRINTS); IPR001548 (PFAM); IPR001548 (PANTHER); SIGNAL_PEPTIDE_C_REGION (PHOBIUS); NON_CYTOPLASMIC_DOMAIN (PHOBIUS); SIGNAL_PEPTIDE_H_REGION (PHOBIUS); SIGNAL_PEPTIDE_N_REGION (PHOBIUS); SIGNAL_PEPTIDE (PHOBIUS); SignalP-noTM (SIGNALP_EUK); SSF55486 (SUPERFAMILY)
1720	294822_length_1215_cvg_11.7_tip_1_3	191	tropinone reductase 2-like	405	6.49E-122	76.50%	0.227		IPR002198 (PRINTS); IPR002347 (PRINTS); IPR013968 (SMART); IPR016040 (G3DSA:3.40.50.GENE3D); IPR002198 (PFAM); PTHR24322 (PANTHER); PTHR24322:SF111 (PANTHER); IPR020904 (PROSITE_PATTERNS); NON_CYTOPLASMIC_DOMAIN (PHOBIUS); CYTOPLASMIC_DOMAIN (PHOBIUS); TRANSMEMBRANE (PHOBIUS); SSF51735 (SUPERFAMILY)
1722	294840_length_1217_cvg_14.5_tip_1_5	229	isocitrate dehydrogenase	405	0	82.60%	0.113		Coil (COILS); IPR024084 (PFAM); IPR004434 (TIGRFAM); IPR024084 (G3DSA:3.40.718.GENE3D); IPR001804 (PANTHER); PTHR11835:SF42 (PANTHER); SSF53659 (SUPERFAMILY)
1723	294876_length_1219_cvg_9.3_tip_1_3	111	deoxyribonuclease-1	407	3.91E-83	61.90%	0.142		IPR016202 (PRINTS); IPR016202 (SMART); IPR005135 (G3DSA:3.60.10.GENE3D); IPR005135 (PFAM); IPR016202 (PANTHER); IPR005135 (SUPERFAMILY)
1724	294902_length_1220_cvg_6.3_tip_1_4	134	serine threonine-protein kinase mark2-like isoform x10	407	0	96.00%	0.184		IPR002290 (SMART); IPR000719 (PFAM); G3DSA:1.10.510.10 (GENE3D); G3DSA:3.30.200.20 (GENE3D); PTHR24346 (PANTHER); PTHR24346:SF23 (PANTHER); IPR017441 (PROSITE_PATTERNS); IPR008271 (PROSITE_PATTERNS); IPR000719 (PROSITE_PROFILES); IPR011009 (SUPERFAMILY)
1725	294926_length_1224_cvg_9.4_tip_1_3	149	chorion peroxidase-like	408	3.28E-62	56.40%	0.16		IPR019791 (PFAM); IPR019791 (G3DSA:1.10.640.GENE3D); IPR029590 (PTHR11475:PANTHER); PTHR11475 (PANTHER); TRANSMEMBRANE (PHOBIUS); NON_CYTOPLASMIC_DOMAIN (PHOBIUS); CYTOPLASMIC_DOMAIN (PHOBIUS); IPR019791 (PROSITE_PROFILES); IPR010255 (SUPERFAMILY); TMhelix (TMHMM)
1726	294956_length_1226_cvg_22.8_tip_1_3	407	glutaredoxin 3	409	5.31E-145	76.80%	0.117		IPR002109 (PFAM); IPR012336 (G3DSA:3.40.30.GENE3D); IPR012336 (G3DSA:3.40.30.GENE3D); IPR013766 (PFAM); IPR004480 (TIGRFAM); PTHR10293:SF40 (PANTHER); IPR004480 (PANTHER); CYTOPLASMIC_DOMAIN (PHOBIUS); NON_CYTOPLASMIC_DOMAIN (PHOBIUS); TRANSMEMBRANE (PHOBIUS); IPR002109 (PROSITE_PROFILES); IPR002109 (PROSITE_PROFILES); IPR012336 (PROSITE_PROFILES); IPR012336 (SUPERFAMILY); IPR012336 (SUPERFAMILY); IPR012336 (SUPERFAMILY)
1734	295142_length_1239_cvg_10.0_tip_1_2	145	glutaminyl-peptide cyclotransferase-like	409	4.28E-116	68.90%	0.174		G3DSA:3.40.630.10 (GENE3D); IPR007484 (PFAM); PTHR12283 (PANTHER); NON_CYTOPLASMIC_DOMAIN (PHOBIUS); CYTOPLASMIC_DOMAIN (PHOBIUS); TRANSMEMBRANE (PHOBIUS); SSF53187 (SUPERFAMILY)
1727	295016_length_1229_cvg_6.9_tip_1_3	103	cd109 antigen	410	0	74.70%	0.132		IPR002890 (PFAM); IPR011625 (PFAM); PTHR11412 (PANTHER); PTHR11412:SF84 (PANTHER)
1728	295050_length_1233_cvg_27.9_tip_0_1	377	cytochrome p450	411	1.02E-99	76.80%	0.295		IPR002401 (PRINTS); IPR001128 (PRINTS); IPR001128 (PFAM); IPR001128 (G3DSA:1.10.630.GENE3D); PTHR24290:SF0 (PANTHER); PTHR24290 (PANTHER); IPR017972 (PROSITE_PATTERNS); IPR001128 (SUPERFAMILY)
1729	295064_length_1233_cvg_2.8_tip_1_1	41	upf0364 protein c6orf211 homolog	411	1.64E-86	60.10%	0.179		IPR002791 (PFAM); PTHR12260 (PANTHER); PTHR12260:SF1 (PANTHER); IPR002791 (SUPERFAMILY)

1731	295094_length_1235_cvg_4.3_tip_1_5	58	#NAME?	411	1.06E-172	81.30%	0.227		G3DSA:3.40.50.2000 (GENE3D); G3DSA:3.40.50.2000 (GENE3D); IPR001830 (PFAM); PTHR10788:SF6 (PANTHER); PTHR10788 (PANTHER); SSF53756 (SUPERFAMILY)
1730	295080_length_1235_cvg_6.1_tip_1_3	104	torsin-1b-like isoform x1	412	1.68E-25	52.90%	0.122		IPR010448 (PFAM); IPR027417 (G3DSA:3.40.50.GENE3D); IPR030553 (PTHR10760:PANTHER); IPR010448 (PANTHER); NON_CYTOPLASMIC_DOMAIN (PHOBIUS); TRANSMEMBRANE (PHOBIUS); CYTOPLASMIC_DOMAIN (PHOBIUS); IPR027417 (SUPERFAMILY); TMhelix (TMHMM)
1732	295098_length_1235_cvg_4.2_tip_1_1	70	thioredoxin domain-containing protein 5	412	9.52E-168	71.80%	0.339		PR00421 (PRINTS); IPR012336 (G3DSA:3.40.30.GENE3D); IPR012336 (G3DSA:3.40.30.GENE3D); IPR005788 (TIGRFAM); IPR013766 (PFAM); PTHR18929:SF52 (PANTHER); PTHR18929 (PANTHER); IPR017937 (PROSITE_PATTERNS); IPR017937 (PROSITE_PATTERNS); IPR017937 (PROSITE_PATTERNS); IPR012336 (PROSITE_PROFILES); IPR012336 (PROSITE_PROFILES); IPR012336 (PROSITE_PROFILES); IPR012336 (SUPERFAMILY); IPR012336 (SUPERFAMILY); IPR012336 (SUPERFAMILY)
1733	295128_length_1238_cvg_52.6_tip_1_0	2232	histone 1o	413	2.63E-29	71.80%	0.126		IPR005819 (PRINTS); IPR005818 (SMART); IPR005818 (PFAM); IPR011991 (G3DSA:1.10.10.GENE3D); PTHR11467 (PANTHER); TRANSMEMBRANE (PHOBIUS); NON_CYTOPLASMIC_DOMAIN (PHOBIUS); CYTOPLASMIC_DOMAIN (PHOBIUS); IPR005818 (PROSITE_PROFILES); SSF46785 (SUPERFAMILY); TMhelix (TMHMM)
1735	295168_length_1242_cvg_4.9_tip_1_5	85	glypican- partial	414	3.97E-68	57.50%	0.151		IPR001863 (PFAM); IPR001863 (PANTHER); IPR019803 (PROSITE_PATTERNS); TRANSMEMBRANE (PHOBIUS); NON_CYTOPLASMIC_DOMAIN (PHOBIUS); CYTOPLASMIC_DOMAIN (PHOBIUS)
1736	295172_length_1242_cvg_4.0_tip_1_0	62	epithelial chloride channel	414	6.33E-34	45.30%	0.102		IPR015394 (PFAM); PTHR10579 (PANTHER); PTHR10579:SF43 (PANTHER)
1737	295182_length_1243_cvg_65.6_tip_1_0	6034	lipoxygenase-like protein	415	1.40E-54	58.10%	0.11		IPR013818 (PFAM); IPR001024 (G3DSA:2.60.60.GENE3D); IPR029058 (G3DSA:3.40.50.GENE3D); PTHR11610:SF8 (PANTHER); IPR000734 (PANTHER); IPR008976 (SUPERFAMILY); IPR029058 (SUPERFAMILY)
1738	295190_length_1244_cvg_36.0_tip_1_0	967	contactin associated protein	415	1.75E-19	49.00%	0.314		Coil (COILS); IPR014716 (G3DSA:3.90.215.GENE3D); IPR008983 (G3DSA:2.60.120.GENE3D); IPR002181 (PFAM); IPR001073 (PFAM); PTHR22923 (PANTHER); PTHR22923:SF49 (PANTHER); SIGNAL_PEPTIDE (PHOBIUS); CYTOPLASMIC_DOMAIN (PHOBIUS); TRANSMEMBRANE (PHOBIUS); SIGNAL_PEPTIDE_N_REGION (PHOBIUS); NON_CYTOPLASMIC_DOMAIN (PHOBIUS); SIGNAL_PEPTIDE_C_REGION (PHOBIUS); SIGNAL_PEPTIDE_H_REGION (PHOBIUS); IPR001073 (PROSITE_PROFILES); IPR002181 (SUPERFAMILY); IPR008983 (SUPERFAMILY); TMhelix (TMHMM)
1739	295222_length_1246_cvg_7.1_tip_1_2	96	multiple epidermal growth factor-like domains protein 10 isoform x2	415	2.69E-94	58.90%	0.112		PR00011 (PRINTS); IPR000742 (SMART); IPR002049 (SMART); IPR013032 (PFAM); G3DSA:2.170.300.10 (GENE3D); IPR002049 (PFAM); PTHR24035 (PANTHER); IPR013032 (PROSITE_PATTERNS); IPR013032 (PROSITE_PATTERNS); IPR013032 (PROSITE_PATTERNS); IPR013032 (PROSITE_PATTERNS); IPR013032 (PROSITE_PATTERNS); IPR013032 (PROSITE_PATTERNS); TRANSMEMBRANE (PHOBIUS); CYTOPLASMIC_DOMAIN (PHOBIUS); NON_CYTOPLASMIC_DOMAIN (PHOBIUS); IPR000742 (PROSITE_PROFILES); IPR000742 (PROSITE_PROFILES); IPR009030 (SUPERFAMILY); TMhelix (TMHMM)
1740	295240_length_1247_cvg_42.2_tip_1_5	511	dehydrogenase reductase sdr family member 4-like isoform x1	415	1.54E-106	74.40%	0.373	Y	IPR002198 (PRINTS); IPR002347 (PRINTS); IPR016040 (G3DSA:3.40.50.GENE3D); PF13561 (PFAM); PTHR24322:SF239 (PANTHER); PTHR24322 (PANTHER); IPR020904 (PROSITE_PATTERNS); SSF51735 (SUPERFAMILY)
1741	295278_length_1249_cvg_3.8_tip_1_1	69	leucine-rich repeat-containing protein 70 isoform x1	416	5.56E-15	50.30%	0.111		IPR003591 (SMART); IPR001611 (PFAM); IPR001611 (PFAM); G3DSA:3.80.10.10 (GENE3D); PTHR24369 (PANTHER); TRANSMEMBRANE (PHOBIUS); TRANSMEMBRANE (PHOBIUS); TRANSMEMBRANE (PHOBIUS); CYTOPLASMIC_DOMAIN (PHOBIUS); NON_CYTOPLASMIC_DOMAIN (PHOBIUS); CYTOPLASMIC_DOMAIN (PHOBIUS); NON_CYTOPLASMIC_DOMAIN (PHOBIUS); SSF52058 (SUPERFAMILY); TMhelix (TMHMM); TMhelix (TMHMM); TMhelix (TMHMM)
1742	295280_length_1249_cvg_13.9_tip_1_2	198	follistatin-related protein 1	416	1.29E-86	57.10%	0.856	Y	IPR002350 (SMART); IPR011992 (G3DSA:1.10.238.GENE3D); G3DSA:3.30.60.30 (GENE3D); IPR002350 (PFAM); IPR011992 (PFAM); PTHR10913:SF38 (PANTHER); PTHR10913 (PANTHER); IPR018247 (PROSITE_PATTERNS); SIGNAL_PEPTIDE_H_REGION (PHOBIUS); SIGNAL_PEPTIDE_C_REGION (PHOBIUS); SIGNAL_PEPTIDE (PHOBIUS); NON_CYTOPLASMIC_DOMAIN (PHOBIUS); SIGNAL_PEPTIDE_N_REGION (PHOBIUS); IPR002350 (PROSITE_PROFILES); IPR002048 (PROSITE_PROFILES); IPR002048 (PROSITE_PROFILES); SignalP-TM (SIGNALP_GRAM_POSITIVE); SignalP-noTM (SIGNALP_EUK); SSF47473 (SUPERFAMILY); SSF100895 (SUPERFAMILY); SSF57603 (SUPERFAMILY)

1744	295324_length_1253_cvg_9.2_tip_1_5	132	atp-dependent rna helicase vasa	417	4.99E-115	78.20%	0.121	IPR001650 (SMART); IPR011545 (PFAM); IPR027417 (G3DSA:3.40.50.GENE3D); IPR027417 (G3DSA:3.40.50.GENE3D); IPR001650 (PFAM); PTHR24031:SF168 (PANTHER); PTHR24031 (PANTHER); NON_CYTOPLASMIC_DOMAIN (PHOBIUS); TRANSMEMBRANE (PHOBIUS); CYTOPLASMIC_DOMAIN (PHOBIUS); CYTOPLASMIC_DOMAIN (PHOBIUS); NON_CYTOPLASMIC_DOMAIN (PHOBIUS); TRANSMEMBRANE (PHOBIUS); TRANSMEMBRANE (PHOBIUS); IPR014001 (PROSITE_PROFILES); IPR001650 (PROSITE_PROFILES); IPR027417 (SUPERFAMILY)
1743	295322_length_1253_cvg_24.0_tip_1_0	489	estradiol 17-beta-dehydrogenase 8	418	1.11E-88	72.40%	0.273	IPR002198 (PRINTS); IPR002347 (PRINTS); IPR013968 (SMART); IPR016040 (G3DSA:3.40.50.GENE3D); IPR002198 (PFAM); PTHR24316 (PANTHER); PTHR24316:SF254 (PANTHER); IPR020904 (PROSITE_PATTERNS); SSF51735 (SUPERFAMILY)
1745	295374_length_1256_cvg_52.8_tip_1_0	867	isoform b	419	5.96E-20	41.90%	0.101	CYTOPLASMIC_DOMAIN (PHOBIUS); TRANSMEMBRANE (PHOBIUS); NON_CYTOPLASMIC_DOMAIN (PHOBIUS); IPR006207 (PROSITE_PROFILES); IPR001007 (PROSITE_PROFILES); TMhelix (TMHMM)
1746	295408_length_1259_cvg_20.7_tip_1_5	291	achain structure and binding determinants of the recombinant kringle-2 domain of human plasminogen to an internal peptide from a group a streptococcal surface protein	419	1.15E-28	63.00%	0.101	PR00018 (PRINTS); IPR000001 (SMART); G3DSA:2.40.20.10 (GENE3D); IPR000001 (PFAM); IPR022041 (PFAM); PTHR24259 (PANTHER); IPR018056 (PROSITE_PATTERNS); CYTOPLASMIC_DOMAIN (PHOBIUS); NON_CYTOPLASMIC_DOMAIN (PHOBIUS); TRANSMEMBRANE (PHOBIUS); IPR000001 (PROSITE_PROFILES); IPR013806 (SUPERFAMILY); TMhelix (TMHMM)
1747	295410_length_1259_cvg_45.2_tip_1_1	799	ves g 5 allergen	420	5.42E-47	52.50%	0.860 Y	IPR001283 (PRINTS); IPR002413 (PRINTS); IPR014044 (SMART); IPR002172 (SMART); IPR002172 (PFAM); IPR014044 (G3DSA:3.40.33.GENE3D); IPR014044 (PFAM); IPR002172 (G3DSA:4.10.400.GENE3D); IPR001283 (PANTHER); IPR018244 (PROSITE_PATTERNS); IPR023415 (PROSITE_PATTERNS); IPR018244 (PROSITE_PATTERNS); NON_CYTOPLASMIC_DOMAIN (PHOBIUS); CYTOPLASMIC_DOMAIN (PHOBIUS); SIGNAL_PEPTIDE_H_REGION (PHOBIUS); SIGNAL_PEPTIDE_C_REGION (PHOBIUS); TRANSMEMBRANE (PHOBIUS); SIGNAL_PEPTIDE_N_REGION (PHOBIUS); SIGNAL_PEPTIDE (PHOBIUS); IPR002172 (PROSITE_PROFILES); SignalP-noTM (SIGNALP_EUK); SignalP-TM (SIGNALP_GRAM_POSITIVE); IPR014044 (SUPERFAMILY); IPR002172 (SUPERFAMILY); TMhelix (TMHMM)
1748	295466_length_1264_cvg_2.0_tip_1_2	42	zinc finger and btb domain-containing protein 24	421	7.19E-69	57.30%	0.113	IPR015880 (SMART); IPR013087 (G3DSA:3.30.160.GENE3D); IPR013087 (G3DSA:3.30.160.GENE3D); PF13894 (PFAM); PF13465 (PFAM); PTHR24409:SF14 (PANTHER); PTHR24409 (PANTHER); IPR007087 (PROSITE_PATTERNS); IPR007087 (PROSITE_PATTERNS); IPR007087 (PROSITE_PATTERNS); IPR007087 (PROSITE_PROFILES); IPR007087 (PROSITE_PROFILES); IPR007087 (PROSITE_PROFILES); IPR007087 (PROSITE_PROFILES); SSF57667 (SUPERFAMILY); SSF57667 (SUPERFAMILY)
1749	295474_length_1264_cvg_14.3_tip_1_3	245	venom protease-like	422	6.33E-114	58.80%	0.146	IPR001314 (PRINTS); IPR001254 (SMART); IPR006604 (SMART); G3DSA:2.40.10.10 (GENE3D); IPR001254 (PFAM); IPR022700 (PFAM); G3DSA:2.40.10.10 (GENE3D); PTHR24268:SF97 (PANTHER); PTHR24268 (PANTHER); IPR018114 (PROSITE_PATTERNS); IPR018114 (PROSITE_PATTERNS); IPR001254 (PROSITE_PROFILES); IPR009003 (SUPERFAMILY)
1750	295506_length_1267_cvg_68.2_tip_1_0	2203	serine proteinase stubble	423	9.01E-66	73.30%	0.155	Coil (COILS); IPR001314 (PRINTS); IPR001254 (SMART); G3DSA:2.40.10.10 (GENE3D); IPR001254 (PFAM); PTHR24268:SF95 (PANTHER); PTHR24268 (PANTHER); CYTOPLASMIC_DOMAIN (PHOBIUS); CYTOPLASMIC_DOMAIN (PHOBIUS); TRANSMEMBRANE (PHOBIUS); TRANSMEMBRANE (PHOBIUS); NON_CYTOPLASMIC_DOMAIN (PHOBIUS); NON_CYTOPLASMIC_DOMAIN (PHOBIUS); TRANSMEMBRANE (PHOBIUS); IPR001254 (PROSITE_PROFILES); IPR009003 (SUPERFAMILY); TMhelix (TMHMM); TMhelix (TMHMM)
1751	295510_length_1268_cvg_17.4_tip_1_3	255	low affinity immunoglobulin epsilon fc receptor	423	1.44E-08	51.40%	0.105	IPR001304 (PFAM); IPR016186 (G3DSA:3.10.100.GENE3D); PTHR22803 (PANTHER); IPR001304 (PROSITE_PROFILES); IPR016187 (SUPERFAMILY)
1752	295550_length_1270_cvg_68.1_tip_0_5	2242	low quality protein: twitchin-like	423	4.69E-178	78.90%	0.124	IPR003598 (SMART); IPR003599 (SMART); IPR013783 (G3DSA:2.60.40.GENE3D); IPR013098 (PFAM); IPR013783 (G3DSA:2.60.40.GENE3D); PTHR19897:SF148 (PANTHER); PTHR19897 (PANTHER); IPR007110 (PROSITE_PROFILES); SSF48726 (SUPERFAMILY); SSF48726 (SUPERFAMILY); SSF48726 (SUPERFAMILY); SSF48726 (SUPERFAMILY)
1753	295600_length_1274_cvg_33.3_tip_1_2	570	acid ceramidase	424	1.93E-157	71.60%	0.680 Y	Coil (COILS); IPR016699 (PIRSF); IPR029130 (PFAM); IPR029132 (PFAM); SignalP-noTM (SIGNALP_EUK)
1754	295622_length_1274_cvg_16.8_tip_1_0	344	adp-ribosylation factor-like protein 8b-a	425	3.71E-124	97.70%	0.118	IPR006689 (PRINTS); IPR024156 (SMART); IPR003579 (SMART); IPR006687 (SMART); IPR006689 (PFAM); IPR027417 (G3DSA:3.40.50.GENE3D); IPR005225 (TIGRFAM); PTHR11711:SF122 (PANTHER); PTHR11711 (PANTHER); IPR024156 (PROSITE_PROFILES); IPR027417 (SUPERFAMILY)

1755	295644_length_1275_cvg_6.2_tip_1_1	158	leukotriene a-4 hydrolase	425	0	76.00%	0.112	IPR014782 (PRINTS); G3DSA:1.10.390.10 (GENE3D); IPR014782 (PFAM); IPR015211 (PFAM); IPR001930 (PANTHER); PTHR11533:SF4 (PANTHER); SSF55486 (SUPERFAMILY); IPR016024 (SUPERFAMILY)
1756	295708_length_1280_cvg_6.6_tip_1_5	108	cytosolic phospholipase a2-like isoform x5	426	7.94E-148	69.80%	0.128	Coil (COILS); IPR000008 (SMART); IPR002642 (SMART); G3DSA:3.40.1090.10 (GENE3D); IPR002642 (PFAM); IPR000008 (PFAM); IPR000008 (G3DSA:2.60.40.GENE3D); PTHR10728 (PANTHER); PTHR10728:SF13 (PANTHER); IPR000008 (PROSITE_PROFILES); IPR002642 (PROSITE_PROFILES); IPR016035 (SUPERFAMILY); IPR000008 (SUPERFAMILY)
1759	295722_length_1282_cvg_7.8_tip_1_2	120	hypothetical protein MDA_GLEAN10014894	427	4.70E-41	40.00%	0.279	no IPS match
1757	295720_length_1282_cvg_13.8_tip_1_3	212	protein spaetzle	428	2.01E-28	54.30%	0.27	PTHR23199 (PANTHER); IPR029034 (SUPERFAMILY)
1758	295722_length_1282_cvg_7.8_tip_1_0	120	cell wall protein dan4-like	428	4.18E-45	47.10%	0.128	no IPS match
1760	295742_length_1284_cvg_3.0_tip_1_2	57	serine threonine-protein kinase 16	428	5.19E-144	76.90%	0.111	IPR002290 (SMART); IPR000719 (PFAM); G3DSA:3.30.200.20 (GENE3D); G3DSA:1.10.510.10 (GENE3D); PTHR22967 (PANTHER); IPR008271 (PROSITE_PATTERNS); IPR000719 (PROSITE_PROFILES); IPR011009 (SUPERFAMILY)
1761	295748_length_1284_cvg_61.2_tip_0_5	2519	muscle m-line assembly protein unc-89-like	428	0	83.80%	0.112	IPR003598 (SMART); IPR003599 (SMART); IPR013098 (PFAM); IPR013783 (G3DSA:2.60.40.GENE3D); IPR013783 (G3DSA:2.60.40.GENE3D); PTHR19897:SF155 (PANTHER); PTHR19897 (PANTHER); IPR007110 (PROSITE_PROFILES); IPR007110 (PROSITE_PROFILES); IPR007110 (PROSITE_PROFILES); IPR007110 (PROSITE_PROFILES); SSF48726 (SUPERFAMILY); SSF48726 (SUPERFAMILY); SSF48726 (SUPERFAMILY); SSF48726 (SUPERFAMILY)
1762	295806_length_1288_cvg_17.1_tip_1_3	245	mitochondrial acyl carrier protein 1 isoform b	430	2.42E-29	96.60%	0.104	IPR009081 (PFAM); IPR009081 (G3DSA:1.10.1200.GENE3D); PTHR20863:SF5 (PANTHER); PTHR20863 (PANTHER); TRANSMEMBRANE (PHOBIUS); NON_CYTOPLASMIC_DOMAIN (PHOBIUS); TRANSMEMBRANE (PHOBIUS); CYTOPLASMIC_DOMAIN (PHOBIUS); NON_CYTOPLASMIC_DOMAIN (PHOBIUS); CYTOPLASMIC_DOMAIN (PHOBIUS); TRANSMEMBRANE (PHOBIUS); IPR003231 (PRODOM); IPR009081 (PROSITE_PROFILES); IPR009081 (SUPERFAMILY); TMhelix (TMHMM)
1763	295842_length_1290_cvg_4.4_tip_1_4	86	multiple inositol polyphosphate phosphatase 1	430	2.08E-117	59.60%	0.870 Y	IPR029033 (G3DSA:3.40.50.GENE3D); IPR016274 (PIRSF); IPR000560 (PFAM); PTHR20963 (PANTHER); PTHR20963:SF8 (PANTHER); SIGNAL_PEPTIDE_H_REGION (PHOBIUS); SIGNAL_PEPTIDE (PHOBIUS); NON_CYTOPLASMIC_DOMAIN (PHOBIUS); SIGNAL_PEPTIDE_C_REGION (PHOBIUS); SIGNAL_PEPTIDE_N_REGION (PHOBIUS); SignalP-noTM (SIGNALP_EUK); IPR029033 (SUPERFAMILY)
1764	295868_length_1292_cvg_45.3_tip_1_4	707	peptidyl-prolyl cis-trans isomerase e	431	3.98E-169	84.30%	0.101	IPR002130 (PRINTS); IPR000504 (SMART); IPR012677 (G3DSA:3.30.70.GENE3D); IPR029000 (G3DSA:2.40.100.GENE3D); IPR002130 (PFAM); IPR000504 (PFAM); IPR024936 (PANTHER); PTHR11071:SF242 (PANTHER); IPR020892 (PROSITE_PATTERNS); IPR002130 (PROSITE_PROFILES); IPR000504 (PROSITE_PROFILES); IPR029000 (SUPERFAMILY); SSF54928 (SUPERFAMILY)
1765	295900_length_1296_cvg_6.0_tip_1_5	88	leucine rich repeat protein	432	5.24E-33	55.20%	0.551 Y	PR00019 (PRINTS); IPR003591 (SMART); SM00365 (SMART); SM00364 (SMART); G3DSA:3.80.10.10 (GENE3D); G3DSA:3.80.10.10 (GENE3D); IPR001611 (PFAM); IPR001611 (PFAM); G3DSA:3.80.10.10 (GENE3D); PTHR24365 (PANTHER); IPR001611 (PROSITE_PROFILES); IPR001611 (PROSITE_PROFILES); IPR001611 (PROSITE_PROFILES); IPR001611 (PROSITE_PROFILES); IPR001611 (PROSITE_PROFILES); IPR001611 (PROSITE_PROFILES); IPR001611 (PROSITE_PROFILES); IPR001611 (PROSITE_PROFILES); IPR001611 (PROSITE_PROFILES); IPR001611 (PROSITE_PROFILES); IPR001611 (PROSITE_PROFILES); IPR001611 (PROSITE_PROFILES); IPR001611 (PROSITE_PROFILES); IPR001611 (PROSITE_PROFILES); SignalP-noTM (SIGNALP_EUK); SSF52058 (SUPERFAMILY)
1766	295928_length_1298_cvg_35.7_tip_1_2	769	projectin short variant	432	2.85E-40	49.60%	0.1	IPR003599 (SMART); IPR003598 (SMART); IPR013783 (G3DSA:2.60.40.GENE3D); IPR013783 (G3DSA:2.60.40.GENE3D); IPR013098 (PFAM); IPR013783 (G3DSA:2.60.40.GENE3D); PTHR19897 (PANTHER); IPR007110 (PROSITE_PROFILES); IPR007110 (PROSITE_PROFILES); SSF48726 (SUPERFAMILY); SSF48726 (SUPERFAMILY); SSF48726 (SUPERFAMILY)

1767	295932_length_1298_cvg_57.5_tip_1_1	1543	phospholipid-hydroperoxide glutathione peroxidase	433	3.15E-80	80.50%	0.143		IPR000889 (PRINTS); IPR012336 (G3DSA:3.40.30.GENE3D); IPR000889 (PFAM); IPR000889 (PANTHER); IPR029760 (PROSITE_PATTERNS); CYTOPLASMIC_DOMAIN (PHOBIUS); TRANSMEMBRANE (PHOBIUS); TRANSMEMBRANE (PHOBIUS); CYTOPLASMIC_DOMAIN (PHOBIUS); NON_CYTOPLASMIC_DOMAIN (PHOBIUS); NON_CYTOPLASMIC_DOMAIN (PHOBIUS); TRANSMEMBRANE (PHOBIUS); NON_CYTOPLASMIC_DOMAIN (PHOBIUS); TRANSMEMBRANE (PHOBIUS); IPR000889 (PROSITE_PROFILES); IPR012336 (SUPERFAMILY)
1768	295984_length_1304_cvg_6.4_tip_1_3	90	27 kda hemolymph	435	4.38E-45	56.10%	0.148		IPR009832 (PFAM); PTHR20997 (PANTHER); CYTOPLASMIC_DOMAIN (PHOBIUS); TRANSMEMBRANE (PHOBIUS); CYTOPLASMIC_DOMAIN (PHOBIUS); NON_CYTOPLASMIC_DOMAIN (PHOBIUS); TRANSMEMBRANE (PHOBIUS)
1769	296022_length_1306_cvg_8.6_tip_1_2	154	receptor-type tyrosine-protein phosphatase epsilon isoform x1	435	6.82E-87	52.60%	0.175		Coil (COILS); IPR000242 (PRINTS); IPR003595 (SMART); IPR000242 (SMART); IPR000242 (PFAM); IPR029021 (G3DSA:3.90.190.GENE3D); IPR029021 (G3DSA:3.90.190.GENE3D); PTHR19134 (PANTHER); IPR016130 (PROSITE_PATTERNS); IPR000242 (PROSITE_PROFILES); IPR000387 (PROSITE_PROFILES); IPR000242 (PROSITE_PROFILES); IPR000387 (PROSITE_PROFILES); IPR029021 (SUPERFAMILY); IPR029021 (SUPERFAMILY)
1770	296032_length_1307_cvg_6.1_tip_1_3	139	variant surface antigen e	436	5.28E-20	44.50%	0.108		no IPS match
1771	296142_length_1319_cvg_16.8_tip_1_5	333	epidermal retinol dehydrogenase 2	439	1.38E-105	71.30%	0.153		IPR002347 (PRINTS); IPR002198 (PRINTS); IPR002198 (PFAM); IPR016040 (G3DSA:3.40.50.GENE3D); PTHR24316:SF289 (PANTHER); PTHR24316 (PANTHER); NON_CYTOPLASMIC_DOMAIN (PHOBIUS); CYTOPLASMIC_DOMAIN (PHOBIUS); TRANSMEMBRANE (PHOBIUS); SSF51735 (SUPERFAMILY); TMhelix (TMHMM)
1773	296202_length_1322_cvg_11.0_tip_1_5	151	n-acetylglucosamine-1-phosphotransferase subunit gamma-like	440	4.57E-71	59.60%	0.349	Y	Coil (COILS); IPR009011 (G3DSA:2.70.130.GENE3D); PF13015 (PFAM); PTHR12630 (PANTHER); PTHR12630:SF6 (PANTHER); IPR009011 (SUPERFAMILY)
1772	296184_length_1321_cvg_66.3_tip_1_3	3356	apolipoprotein d	441	5.36E-73	62.40%	0.142		IPR003057 (PRINTS); IPR012674 (G3DSA:2.40.128.GENE3D); IPR000566 (PFAM); PTHR10612 (PANTHER); PTHR10612:SF7 (PANTHER); IPR022272 (PROSITE_PATTERNS); NON_CYTOPLASMIC_DOMAIN (PHOBIUS); CYTOPLASMIC_DOMAIN (PHOBIUS); NON_CYTOPLASMIC_DOMAIN (PHOBIUS); TRANSMEMBRANE (PHOBIUS); TRANSMEMBRANE (PHOBIUS); IPR011038 (SUPERFAMILY); TMhelix (TMHMM); TMhelix (TMHMM)
1774	296220_length_1325_cvg_14.1_tip_1_3	295	gtp-binding protein sar1b	442	1.02E-124	93.20%	0.138		IPR006689 (PRINTS); IPR006687 (SMART); IPR024156 (SMART); IPR005225 (TIGRFAM); IPR006689 (PFAM); IPR027417 (G3DSA:3.40.50.GENE3D); PTHR11711 (PANTHER); PTHR11711:SF133 (PANTHER); NON_CYTOPLASMIC_DOMAIN (PHOBIUS); TRANSMEMBRANE (PHOBIUS); CYTOPLASMIC_DOMAIN (PHOBIUS); IPR006687 (PROSITE_PROFILES); IPR027417 (SUPERFAMILY)
1775	296266_length_1328_cvg_5.9_tip_1_0	99	maltase a5	443	1.01E-168	69.40%	0.164		IPR006589 (SMART); IPR006047 (PFAM); IPR013781 (G3DSA:3.20.20.GENE3D); G3DSA:3.90.400.10 (GENE3D); IPR015902 (PANTHER); PTHR10357:SF76 (PANTHER); IPR017853 (SUPERFAMILY)
1776	296268_length_1328_cvg_58.4_tip_1_4	3867	low quality protein: glutathione peroxidase 3	443	3.87E-59	63.90%	0.21		IPR012336 (G3DSA:3.40.30.GENE3D); IPR000889 (PFAM); IPR000889 (PANTHER); IPR029760 (PROSITE_PATTERNS); CYTOPLASMIC_DOMAIN (PHOBIUS); TRANSMEMBRANE (PHOBIUS); NON_CYTOPLASMIC_DOMAIN (PHOBIUS); IPR000889 (PROSITE_PROFILES); IPR012336 (SUPERFAMILY); TMhelix (TMHMM)
1777	296276_length_1329_cvg_55.1_tip_0_0	1151	diphenol oxidase	443	4.38E-20	65.20%	0.1		IPR000436 (SMART); G3DSA:2.10.70.10 (GENE3D); IPR000436 (PFAM); G3DSA:2.10.70.10 (GENE3D); G3DSA:2.10.70.10 (GENE3D); PTHR19325 (PANTHER); IPR000436 (PROSITE_PROFILES); IPR000436 (PROSITE_PROFILES); IPR000436 (PROSITE_PROFILES); IPR000436 (SUPERFAMILY); IPR000436 (SUPERFAMILY); IPR000436 (SUPERFAMILY)
1778	296288_length_1330_cvg_11.1_tip_1_1	219	atpase family aaa domain-containing protein 1-b	443	4.49E-166	78.10%	0.121		IPR003593 (SMART); G3DSA:1.10.8.60 (GENE3D); IPR003959 (PFAM); IPR027417 (G3DSA:3.40.50.GENE3D); PTHR23074:SF20 (PANTHER); PTHR23074 (PANTHER); IPR003960 (PROSITE_PATTERNS); IPR027417 (SUPERFAMILY)
1779	296310_length_1332_cvg_65.4_tip_1_4	3284	scp-like extracellular domain containing protein 1	444	4.21E-70	62.10%	0.295		IPR001283 (PRINTS); IPR002413 (PRINTS); IPR014044 (SMART); IPR014044 (G3DSA:3.40.33.GENE3D); IPR014044 (PFAM); PTHR10334:SF174 (PANTHER); IPR001283 (PANTHER); IPR018244 (PROSITE_PATTERNS); IPR018244 (PROSITE_PROFILES); TRANSMEMBRANE (PHOBIUS); NON_CYTOPLASMIC_DOMAIN (PHOBIUS); CYTOPLASMIC_DOMAIN (PHOBIUS); CYTOPLASMIC_DOMAIN (PHOBIUS); TRANSMEMBRANE (PHOBIUS); NON_CYTOPLASMIC_DOMAIN (PHOBIUS); TRANSMEMBRANE (PHOBIUS); IPR014044 (SUPERFAMILY); TMhelix (TMHMM); TMhelix (TMHMM)

1780	296312_length_1332_cvg_4.8_tip_1_5	94	glutamate-rich wd repeat-containing protein 1	444	1.60E-145	74.50%	0.108	IPR020472 (PRINTS); IPR001680 (SMART); IPR001680 (PFAM); IPR015943 (G3DSA:2.130.10.GENE3D); PTHR22850 (PANTHER); IPR019775 (PROSITE_PATTERNS); TRANSMEMBRANE (PHOBIUS); CYTOPLASMIC_DOMAIN (PHOBIUS); NON_CYTOPLASMIC_DOMAIN (PHOBIUS); IPR017986 (PROSITE_PROFILES); IPR001680 (PROSITE_PROFILES); IPR001680 (PROSITE_PROFILES); IPR001680 (PROSITE_PROFILES); IPR017986 (SUPERFAMILY)
1781	296376_length_1337_cvg_27.6_tip_1_0	456	chymotrypsinogen a	446	2.97E-95	59.30%	0.831 Y	IPR001314 (PRINTS); IPR001254 (SMART); G3DSA:2.40.10.10 (GENE3D); G3DSA:2.40.10.10 (GENE3D); G3DSA:2.40.10.10 (GENE3D); IPR001254 (PFAM); PTHR24260:SF47 (PANTHER); PTHR24260 (PANTHER); SIGNAL_PEPTIDE_C_REGION (PHOBIUS); NON_CYTOPLASMIC_DOMAIN (PHOBIUS); SIGNAL_PEPTIDE_N_REGION (PHOBIUS); SIGNAL_PEPTIDE (PHOBIUS); SIGNAL_PEPTIDE_H_REGION (PHOBIUS); IPR001254 (PROSITE_PROFILES); SignalP-noTM (SIGNALP_EUK); IPR009003 (SUPERFAMILY)
1782	296382_length_1338_cvg_84.0_tip_1_4	2771	membrane glycoprotein lig-	446	3.02E-20	47.60%	0.434 Y	IPR003591 (SMART); IPR001611 (PFAM); IPR001611 (PFAM); G3DSA:3.80.10.10 (GENE3D); PTHR24365 (PANTHER); SIGNAL_PEPTIDE_C_REGION (PHOBIUS); SIGNAL_PEPTIDE (PHOBIUS); SIGNAL_PEPTIDE_H_REGION (PHOBIUS); SIGNAL_PEPTIDE_N_REGION (PHOBIUS); NON_CYTOPLASMIC_DOMAIN (PHOBIUS); SignalP-TM (SIGNALP_GRAM_POSITIVE); SSF52058 (SUPERFAMILY)
1783	296386_length_1338_cvg_15.4_tip_1_3	308	platelet-activating factor acetylhydrolase	446	4.89E-103	57.20%	0.135	Coil (COILS); IPR016715 (PIRSF); IPR005065 (PFAM); IPR029058 (G3DSA:3.40.50.GENE3D); IPR005065 (PANTHER); IPR029058 (SUPERFAMILY)
1784	296400_length_1340_cvg_12.1_tip_1_0	204	alpha beta hydrolase domain-containing protein 13	447	2.04E-142	75.00%	0.105	IPR029059 (PFAM); IPR029058 (G3DSA:3.40.50.GENE3D); PTHR12277 (PANTHER); TRANSMEMBRANE (PHOBIUS); CYTOPLASMIC_DOMAIN (PHOBIUS); TRANSMEMBRANE (PHOBIUS); NON_CYTOPLASMIC_DOMAIN (PHOBIUS); CYTOPLASMIC_DOMAIN (PHOBIUS); CYTOPLASMIC_DOMAIN (PHOBIUS); TRANSMEMBRANE (PHOBIUS); TRANSMEMBRANE (PHOBIUS); NON_CYTOPLASMIC_DOMAIN (PHOBIUS); IPR029058 (SUPERFAMILY); TMhelix (TMHMM); TMhelix (TMHMM)
1785	296424_length_1342_cvg_17.6_tip_1_4	305	3-hydroxyisobutyrate dehydrogenase	447	1.35E-65	55.40%	0.16	IPR006115 (PFAM); IPR013328 (G3DSA:1.10.1040.GENE3D); IPR029154 (PFAM); IPR016040 (G3DSA:3.40.50.GENE3D); PTHR22981:SF7 (PANTHER); PTHR22981 (PANTHER); IPR029752 (PROSITE_PATTERNS); SSF51735 (SUPERFAMILY); IPR008927 (SUPERFAMILY)
1786	296444_length_1344_cvg_3.4_tip_1_2	73	zinc finger protein glis2-like	448	7.41E-77	75.90%	0.102	IPR015880 (SMART); PF13465 (PFAM); IPR013087 (G3DSA:3.30.160.GENE3D); IPR013087 (G3DSA:3.30.160.GENE3D); IPR013087 (G3DSA:3.30.160.GENE3D); IPR007087 (PFAM); IPR013087 (G3DSA:3.30.160.GENE3D); IPR013087 (G3DSA:3.30.160.GENE3D); PTHR19818 (PANTHER); IPR007087 (PROSITE_PATTERNS); IPR007087 (PROSITE_PATTERNS); IPR007087 (PROSITE_PATTERNS); IPR007087 (PROSITE_PATTERNS); IPR007087 (PROSITE_PROFILES); IPR007087 (PROSITE_PROFILES); IPR007087 (PROSITE_PROFILES); IPR007087 (PROSITE_PROFILES); SSF57667 (SUPERFAMILY); SSF57667 (SUPERFAMILY); SSF57667 (SUPERFAMILY)
1787	296492_length_1349_cvg_9.8_tip_1_4	192	g-protein coupled receptor mth2-like isoform x2	450	1.97E-90	54.50%	0.127	IPR000832 (PFAM); IPR010596 (PFAM); IPR023311 (G3DSA:2.170.180.GENE3D); PTHR12011:SF164 (PANTHER); PTHR12011 (PANTHER); CYTOPLASMIC_DOMAIN (PHOBIUS); NON_CYTOPLASMIC_DOMAIN (PHOBIUS); TRANSMEMBRANE (PHOBIUS); TRANSMEMBRANE (PHOBIUS); NON_CYTOPLASMIC_DOMAIN (PHOBIUS); TRANSMEMBRANE (PHOBIUS); CYTOPLASMIC_DOMAIN (PHOBIUS); NON_CYTOPLASMIC_DOMAIN (PHOBIUS); TRANSMEMBRANE (PHOBIUS); NON_CYTOPLASMIC_DOMAIN (PHOBIUS); TRANSMEMBRANE (PHOBIUS); CYTOPLASMIC_DOMAIN (PHOBIUS); TRANSMEMBRANE (PHOBIUS); TRANSMEMBRANE (PHOBIUS); CYTOPLASMIC_DOMAIN (PHOBIUS); IPR017981 (PROSITE_PROFILES); SSF63877 (SUPERFAMILY); TMhelix (TMHMM); TMhelix (TMHMM); TMhelix (TMHMM); TMhelix (TMHMM); TMhelix (TMHMM); TMhelix (TMHMM); TMhelix (TMHMM)
1788	296506_length_1351_cvg_5.6_tip_1_5	115	mam and ldl-receptor class a domain-containing protein 1-like	450	9.23E-16	40.00%	0.605 Y	IPR002172 (SMART); IPR000998 (SMART); IPR002172 (PFAM); IPR000998 (PFAM); IPR002172 (G3DSA:4.10.400.GENE3D); PTHR23282 (PANTHER); PTHR23282:SF75 (PANTHER); IPR002172 (PROSITE_PROFILES); IPR000998 (PROSITE_PROFILES); IPR000998 (PROSITE_PROFILES); SignalP-noTM (SIGNALP_EUK); IPR013320 (SUPERFAMILY); IPR013320 (SUPERFAMILY); IPR002172 (SUPERFAMILY)
1789	296512_length_1353_cvg_26.6_tip_1_0	473	annexin a5-like	451	2.40E-18	62.30%	0.102	CYTOPLASMIC_DOMAIN (PHOBIUS); TRANSMEMBRANE (PHOBIUS); NON_CYTOPLASMIC_DOMAIN (PHOBIUS); TMhelix (TMHMM)

1790	296548_length_1356_cvg_4.1_tip_1_4	95	latrophilin-3 isoform x10	452	2.57E-41	46.30%	0.109	IPR000832 (PRINTS); IPR000203 (SMART); IPR022624 (PFAM); IPR000203 (PFAM); IPR000832 (PFAM); PTHR12011 (PANTHER); TRANSMEMBRANE (PHOBIUS); TRANSMEMBRANE (PHOBIUS); NON_CYTOPLASMIC_DOMAIN (PHOBIUS); CYTOPLASMIC_DOMAIN (PHOBIUS); TRANSMEMBRANE (PHOBIUS); NON_CYTOPLASMIC_DOMAIN (PHOBIUS); CYTOPLASMIC_DOMAIN (PHOBIUS); NON_CYTOPLASMIC_DOMAIN (PHOBIUS); TRANSMEMBRANE (PHOBIUS); IPR000203 (PROSITE_PROFILES); IPR017981 (PROSITE_PROFILES); TMhelix (TMHMM); TMhelix (TMHMM); TMhelix (TMHMM); TMhelix (TMHMM)
1791	296556_length_1356_cvg_23.1_tip_1_5	300	mam and ldl-receptor class a domain-containing protein 1	452	5.89E-35	46.40%	0.107	IPR002172 (PRINTS); IPR002172 (SMART); IPR002172 (G3DSA:4.10.400.GENE3D); IPR000998 (PFAM); IPR002172 (PFAM); IPR002172 (G3DSA:4.10.400.GENE3D); G3DSA:2.10.25.10 (GENE3D); PTHR23282 (PANTHER); PTHR23282:SF67 (PANTHER); IPR023415 (PROSITE_PATTERNS); IPR023415 (PROSITE_PATTERNS); IPR023415 (PROSITE_PATTERNS); IPR013032 (PROSITE_PATTERNS); NON_CYTOPLASMIC_DOMAIN (PHOBIUS); CYTOPLASMIC_DOMAIN (PHOBIUS); TRANSMEMBRANE (PHOBIUS); IPR000742 (PROSITE_PROFILES); IPR002172 (PROSITE_PROFILES); IPR002172 (PROSITE_PROFILES); IPR000998 (PROSITE_PROFILES); IPR002172 (PROSITE_PROFILES); SSF57196 (SUPERFAMILY); IPR002172 (SUPERFAMILY); IPR002172 (SUPERFAMILY); TMhelix (TMHMM)
1792	296572_length_1359_cvg_13.3_tip_1_0	224	protein spaetzle	453	7.29E-31	53.20%	0.256	PTHR23199 (PANTHER); CYTOPLASMIC_DOMAIN (PHOBIUS); NON_CYTOPLASMIC_DOMAIN (PHOBIUS); TRANSMEMBRANE (PHOBIUS); IPR029034 (SUPERFAMILY); TMhelix (TMHMM)
1793	296590_length_1360_cvg_49.9_tip_1_5	3900	astakine variant 1	453	3.09E-20	62.00%	0.186	G3DSA:2.10.80.10 (GENE3D); CYTOPLASMIC_DOMAIN (PHOBIUS); NON_CYTOPLASMIC_DOMAIN (PHOBIUS); TRANSMEMBRANE (PHOBIUS); NON_CYTOPLASMIC_DOMAIN (PHOBIUS); TRANSMEMBRANE (PHOBIUS)
1794	296592_length_1360_cvg_7.8_tip_1_0	115	alpha-2-macroglobulin-like protein 1 isoform x2	454	5.32E-119	59.20%	0.649 Y	IPR002890 (PFAM); PTHR11412 (PANTHER); SIGNAL_PEPTIDE (PHOBIUS); NON_CYTOPLASMIC_DOMAIN (PHOBIUS); SIGNAL_PEPTIDE_C_REGION (PHOBIUS); SIGNAL_PEPTIDE_H_REGION (PHOBIUS); SIGNAL_PEPTIDE_N_REGION (PHOBIUS); SignalP-noTM (SIGNALP_EUK)
1795	296596_length_1361_cvg_21.6_tip_1_0	393	f5 8 type c domain protein	454	7.57E-31	38.40%	0.148	no IPS match
1796	296596_length_1361_cvg_21.6_tip_1_3	393	collagen alpha-1 chain	454	4.66E-36	44.10%	0.101	IPR008160 (PFAM); PTHR24023 (PANTHER)
1797	296612_length_1361_cvg_6.5_tip_1_4	154	cyclin-c	454	9.78E-157	91.20%	0.124	IPR013763 (SMART); IPR013763 (G3DSA:1.10.472.GENE3D); IPR006671 (PFAM); IPR015429 (PANTHER); TRANSMEMBRANE (PHOBIUS); CYTOPLASMIC_DOMAIN (PHOBIUS); NON_CYTOPLASMIC_DOMAIN (PHOBIUS); TRANSMEMBRANE (PHOBIUS); CYTOPLASMIC_DOMAIN (PHOBIUS); TRANSMEMBRANE (PHOBIUS); NON_CYTOPLASMIC_DOMAIN (PHOBIUS); IPR013763 (SUPERFAMILY); IPR013763 (SUPERFAMILY); TMhelix (TMHMM); TMhelix (TMHMM); TMhelix (TMHMM)
1798	296614_length_1362_cvg_59.2_tip_1_5	1535	group 3 secretory phospholipase partial	454	1.52E-87	55.90%	0.14	Coil (COILS); IPR001211 (PFAM); IPR016090 (G3DSA:1.20.90.GENE3D); PTHR12253 (PANTHER); IPR016090 (SUPERFAMILY)
1799	296634_length_1364_cvg_14.3_tip_1_5	237	cytochrome p450 3a4-like	454	3.24E-70	59.70%	0.164	IPR002401 (PRINTS); IPR001128 (PRINTS); IPR001128 (PFAM); IPR001128 (G3DSA:1.10.630.GENE3D); PTHR24292 (PANTHER); PTHR24292:SF24 (PANTHER); IPR017972 (PROSITE_PATTERNS); NON_CYTOPLASMIC_DOMAIN (PHOBIUS); CYTOPLASMIC_DOMAIN (PHOBIUS); TRANSMEMBRANE (PHOBIUS); IPR001128 (SUPERFAMILY)
1800	296638_length_1365_cvg_7.9_tip_1_0	148	uncharacterized oxidoreductase yrbe-like	455	3.52E-142	68.50%	0.106	IPR004104 (PFAM); IPR000683 (PFAM); IPR016040 (G3DSA:3.40.50.GENE3D); G3DSA:3.30.360.10 (GENE3D); PTHR22604:SF92 (PANTHER); PTHR22604 (PANTHER); SSF51735 (SUPERFAMILY); SSF55347 (SUPERFAMILY)
1801	296644_length_1365_cvg_6.0_tip_1_0	156	secreted frizzled-related protein 5	455	1.27E-117	63.80%	0.294	Coil (COILS); IPR020067 (SMART); IPR020067 (G3DSA:1.10.2000.GENE3D); IPR020067 (PFAM); IPR018933 (PFAM); PTHR11309:SF80 (PANTHER); IPR015526 (PANTHER); TRANSMEMBRANE (PHOBIUS); CYTOPLASMIC_DOMAIN (PHOBIUS); NON_CYTOPLASMIC_DOMAIN (PHOBIUS); IPR020067 (PROSITE_PROFILES); IPR001134 (PROSITE_PROFILES); IPR008993 (SUPERFAMILY); IPR020067 (SUPERFAMILY); TMhelix (TMHMM)
1803	296664_length_1367_cvg_15.8_tip_1_2	282	wd repeat-containing protein 86-like	455	1.31E-78	68.80%	0.11	IPR020472 (PRINTS); IPR001680 (SMART); IPR001680 (PFAM); IPR015943 (G3DSA:2.130.10.GENE3D); PTHR22844 (PANTHER); PTHR22844:SF166 (PANTHER); IPR019775 (PROSITE_PATTERNS); IPR019775 (PROSITE_PATTERNS); TRANSMEMBRANE (PHOBIUS); CYTOPLASMIC_DOMAIN (PHOBIUS); NON_CYTOPLASMIC_DOMAIN (PHOBIUS); CYTOPLASMIC_DOMAIN (PHOBIUS); TRANSMEMBRANE (PHOBIUS); IPR017986 (PROSITE_PROFILES); IPR001680 (PROSITE_PROFILES); IPR001680 (PROSITE_PROFILES); IPR017986 (SUPERFAMILY); TMhelix (TMHMM)

1802	296660_length_1366_cvg_30.2_tip_1_3	546	na+ h+ exchange regulatory cofactor nhe-rf2	456	1.22E-08	68.83%	0.109	no IPS match
1804	296690_length_1370_cvg_7.9_tip_1_3	144	dnaj homolog subfamily a member 1	457	0	85.90%	0.156	IPR001623 (PRINTS); IPR001623 (SMART); IPR001305 (G3DSA:2.10.230.GENE3D); IPR001305 (PFAM); G3DSA:2.60.260.20 (GENE3D); IPR001623 (G3DSA:1.10.287.GENE3D); IPR002939 (PFAM); IPR001623 (PFAM); G3DSA:2.60.260.20 (GENE3D); PTHR24076:SF75 (PANTHER); PTHR24076 (PANTHER); IPR018253 (PROSITE_PATTERNS); IPR012724 (HAMAP); IPR001623 (PROSITE_PROFILES); IPR001305 (PROSITE_PROFILES); IPR001305 (SUPERFAMILY); IPR008971 (SUPERFAMILY); IPR001623 (SUPERFAMILY); IPR008971 (SUPERFAMILY)
1805	296702_length_1370_cvg_6.9_tip_1_1	119	---NA---	457			0.106	Coil (COILS); Coil (COILS); Coil (COILS)
1806	296710_length_1370_cvg_12.6_tip_1_0	252	lethal giant larvae partial	457	0	76.70%	0.219	IPR000664 (PRINTS); IPR001680 (SMART); IPR015943 (G3DSA:2.130.10.GENE3D); IPR013577 (PFAM); PTHR10241 (PANTHER); PTHR10241:SF28 (PANTHER); IPR019775 (PROSITE_PATTERNS); IPR017986 (PROSITE_PROFILES); IPR017986 (SUPERFAMILY)
1807	296716_length_1371_cvg_10.9_tip_1_4	159	matrix metalloproteinase-9-like	457	1.27E-09	48.70%	0.149	IPR000562 (SMART); IPR000562 (G3DSA:2.10.10.GENE3D); IPR000562 (PFAM); IPR000562 (PROSITE_PROFILES); IPR000562 (PROSITE_PROFILES); IPR013806 (SUPERFAMILY); IPR013806 (SUPERFAMILY)
1808	296754_length_1375_cvg_30.1_tip_1_0	547	regulatory particle triple-a atpase isoform a	459	0	97.00%	0.105	IPR003593 (SMART); IPR005937 (TIGRFAM); IPR027417 (G3DSA:3.40.50.GENE3D); G3DSA:1.10.8.60 (GENE3D); IPR003959 (PFAM); PTHR23073 (PANTHER); PTHR23073:SF9 (PANTHER); IPR003960 (PROSITE_PATTERNS); IPR027417 (SUPERFAMILY)
1809	296760_length_1375_cvg_5.5_tip_1_0	127	insulin-like growth factor-binding protein complex acid labile subunit	459	1.40E-65	55.90%	0.372 Y	SM00365 (SMART); IPR003591 (SMART); G3DSA:3.80.10.10 (GENE3D); IPR001611 (PFAM); PTHR24365 (PANTHER); SIGNAL_PEPTIDE (PHOBIUS); SIGNAL_PEPTIDE_N_REGION (PHOBIUS); NON_CYTOPLASMIC_DOMAIN (PHOBIUS); SIGNAL_PEPTIDE_H_REGION (PHOBIUS); SIGNAL_PEPTIDE_C_REGION (PHOBIUS); IPR001611 (PROSITE_PROFILES); IPR001611 (PROSITE_PROFILES); IPR001611 (PROSITE_PROFILES); IPR001611 (PROSITE_PROFILES); IPR001611 (PROSITE_PROFILES); IPR001611 (PROSITE_PROFILES); IPR001611 (PROSITE_PROFILES); IPR001611 (PROSITE_PROFILES); IPR001611 (PROSITE_PROFILES); IPR001611 (PROSITE_PROFILES); SSF52058 (SUPERFAMILY)
1810	296834_length_1384_cvg_45.5_tip_1_5	976	hypothetical protein DAPPUDRAFT_308200	461	8.66E-35	49.70%	0.121	IPR010562 (SMART); IPR010562 (PFAM); PTHR11008:SF9 (PANTHER); PTHR11008 (PANTHER); IPR020234 (PRODOM)
1811	296848_length_1385_cvg_16.4_tip_1_4	297	protein tumorous imaginal mitochondrial-like isoform x2	462	0	79.80%	0.269	IPR001623 (PRINTS); IPR001623 (SMART); IPR001305 (G3DSA:2.10.230.GENE3D); IPR001623 (G3DSA:1.10.287.GENE3D); G3DSA:2.60.260.20 (GENE3D); IPR001623 (PFAM); IPR002939 (PFAM); IPR001305 (PFAM); PTHR24076:SF79 (PANTHER); PTHR24076 (PANTHER); IPR018253 (PROSITE_PATTERNS); IPR012724 (HAMAP); IPR001305 (PROSITE_PROFILES); IPR001623 (PROSITE_PROFILES); IPR001305 (SUPERFAMILY); IPR001623 (SUPERFAMILY); IPR008971 (SUPERFAMILY); IPR008971 (SUPERFAMILY)
1812	296912_length_1391_cvg_8.6_tip_1_4	170	carboxypeptidase n subunit 2	464	2.62E-18	41.80%	0.286	IPR001611 (PFAM); G3DSA:3.80.10.10 (GENE3D); IPR001611 (PFAM); PTHR24365 (PANTHER); NON_CYTOPLASMIC_DOMAIN (PHOBIUS); TRANSMEMBRANE (PHOBIUS); CYTOPLASMIC_DOMAIN (PHOBIUS); IPR001611 (PROSITE_PROFILES); IPR001611 (PROSITE_PROFILES); IPR001611 (PROSITE_PROFILES); IPR001611 (PROSITE_PROFILES); IPR001611 (PROSITE_PROFILES); IPR001611 (PROSITE_PROFILES); IPR001611 (PROSITE_PROFILES); SSF52058 (SUPERFAMILY); TMhelix (TMHMM)
1814	296940_length_1394_cvg_7.8_tip_1_2	162	thromboxane a synthase-like protein	464	1.08E-112	58.60%	0.271	IPR002401 (PRINTS); IPR001128 (G3DSA:1.10.630.GENE3D); IPR001128 (PFAM); PTHR24292:SF24 (PANTHER); PTHR24292 (PANTHER); NON_CYTOPLASMIC_DOMAIN (PHOBIUS); TRANSMEMBRANE (PHOBIUS); CYTOPLASMIC_DOMAIN (PHOBIUS); NON_CYTOPLASMIC_DOMAIN (PHOBIUS); TRANSMEMBRANE (PHOBIUS); TRANSMEMBRANE (PHOBIUS); CYTOPLASMIC_DOMAIN (PHOBIUS); IPR001128 (SUPERFAMILY); TMhelix (TMHMM)
1813	296928_length_1393_cvg_26.2_tip_1_3	397	laccase-5- partial	465	1.68E-131	63.80%	0.253	IPR008972 (G3DSA:2.60.40.GENE3D); IPR011707 (PFAM); IPR008972 (G3DSA:2.60.40.GENE3D); IPR001117 (PFAM); PTHR11709 (PANTHER); PTHR11709:SF33 (PANTHER); NON_CYTOPLASMIC_DOMAIN (PHOBIUS); CYTOPLASMIC_DOMAIN (PHOBIUS); TRANSMEMBRANE (PHOBIUS); SignalP-TM (SIGNALP_GRAM_POSITIVE); IPR008972 (SUPERFAMILY); IPR008972 (SUPERFAMILY); TMhelix (TMHMM)

1815	296966_length_1397_cvg_9.3_tip_1_0	125	g-protein coupled receptor mth2 isoform x1	466	5.22E-43	64.90%	0.298	IPR000832 (PFAM); PTHR12011:SF164 (PANTHER); PTHR12011 (PANTHER); CYTOPLASMIC_DOMAIN (PHOBIUS); TRANSMEMBRANE (PHOBIUS); TRANSMEMBRANE (PHOBIUS); NON_CYTOPLASMIC_DOMAIN (PHOBIUS); TRANSMEMBRANE (PHOBIUS); NON_CYTOPLASMIC_DOMAIN (PHOBIUS); CYTOPLASMIC_DOMAIN (PHOBIUS); TRANSMEMBRANE (PHOBIUS); CYTOPLASMIC_DOMAIN (PHOBIUS); IPR017981 (PROSITE_PROFILES); TMhelix (TMHMM); TMhelix (TMHMM); TMhelix (TMHMM); TMhelix (TMHMM)
1816	297004_length_1401_cvg_4.8_tip_1_3	92	sortilin-related receptor-like	467	8.71E-154	64.90%	0.162	IPR002172 (PRINTS); IPR002172 (SMART); IPR002172 (G3DSA:4.10.400.GENE3D); IPR002172 (G3DSA:4.10.400.GENE3D); IPR002172 (PFAM); IPR002172 (G3DSA:4.10.400.GENE3D); IPR002172 (G3DSA:4.10.400.GENE3D); IPR002172 (G3DSA:4.10.400.GENE3D); IPR002172 (G3DSA:4.10.400.GENE3D); G3DSA:2.10.25.10 (GENE3D); PTHR10529 (PANTHER); IPR023415 (PROSITE_PATTERNS); IPR023415 (PROSITE_PATTERNS); IPR023415 (PROSITE_PATTERNS); IPR023415 (PROSITE_PATTERNS); IPR023415 (PROSITE_PATTERNS); IPR023415 (PROSITE_PATTERNS); IPR023415 (PROSITE_PATTERNS); IPR023415 (PROSITE_PATTERNS); IPR002172 (PROSITE_PROFILES); IPR002172 (PROSITE_PROFILES); IPR002172 (PROSITE_PROFILES); IPR002172 (PROSITE_PROFILES); IPR002172 (PROSITE_PROFILES); IPR002172 (PROSITE_PROFILES); IPR002172 (PROSITE_PROFILES); IPR002172 (PROSITE_PROFILES); IPR002172 (PROSITE_PROFILES); IPR002172 (PROSITE_PROFILES); IPR002172 (SUPERFAMILY); IPR002172 (SUPERFAMILY); IPR002172 (SUPERFAMILY); IPR002172 (SUPERFAMILY); IPR002172 (SUPERFAMILY); IPR002172 (SUPERFAMILY); IPR002172 (SUPERFAMILY); IPR002172 (SUPERFAMILY); IPR002172 (SUPERFAMILY)
1817	297012_length_1401_cvg_4.0_tip_1_3	75	membrane-associated guanylate ww and pdz domain-containing protein 1-like isoform x2	467	8.24E-130	81.40%	0.109	IPR001478 (SMART); IPR001478 (G3DSA:2.30.42.GENE3D); IPR001478 (G3DSA:2.30.42.GENE3D); IPR001478 (PFAM); IPR001478 (G3DSA:2.30.42.GENE3D); PTHR10316:SF40 (PANTHER); PTHR10316 (PANTHER); IPR001478 (PROSITE_PROFILES); IPR001478 (PROSITE_PROFILES); IPR001478 (PROSITE_PROFILES); IPR001478 (SUPERFAMILY); IPR001478 (SUPERFAMILY); IPR001478 (SUPERFAMILY)
1819	297044_length_1406_cvg_3.8_tip_1_2	70	u3 small nucleolar rna-interacting protein 2-like isoform x2	468	6.12E-171	71.40%	0.465 Y	Coil (COILS); IPR020472 (PRINTS); IPR001680 (SMART); IPR001680 (PFAM); IPR015943 (G3DSA:2.130.10.GENE3D); PTHR19865 (PANTHER); IPR019775 (PROSITE_PATTERNS); IPR001680 (PROSITE_PROFILES); IPR001680 (PROSITE_PROFILES); IPR001680 (PROSITE_PROFILES); IPR001680 (PROSITE_PROFILES); IPR001680 (PROSITE_PROFILES); IPR001680 (PROSITE_PROFILES); IPR017986 (PROSITE_PROFILES); SignalP-noTM (SIGNALP_EUK); IPR017986 (SUPERFAMILY)
1818	297042_length_1406_cvg_4.0_tip_1_0	83	transducin beta-like protein 3	469	2.95E-101	75.40%	0.147	Coil (COILS); Coil (COILS); IPR001680 (SMART); IPR013934 (PFAM); IPR001680 (PFAM); IPR015943 (G3DSA:2.130.10.GENE3D); PTHR19854 (PANTHER); PTHR19854:SF15 (PANTHER); IPR017986 (PROSITE_PROFILES); IPR001680 (PROSITE_PROFILES); IPR001680 (PROSITE_PROFILES); IPR001680 (PROSITE_PROFILES); IPR017986 (SUPERFAMILY)
1820	297054_length_1407_cvg_62.0_tip_1_1	6271	disulfide isomerase	469	0	81.70%	0.668 Y	PR00421 (PRINTS); IPR005788 (TIGRFAM); IPR013766 (PFAM); IPR012336 (G3DSA:3.40.30.GENE3D); IPR012336 (G3DSA:3.40.30.GENE3D); PF13848 (PFAM); IPR005792 (TIGRFAM); PTHR18929:SF48 (PANTHER); PTHR18929 (PANTHER); IPR017937 (PROSITE_PATTERNS); IPR017937 (PROSITE_PATTERNS); NON_CYTOPLASMIC_DOMAIN (PHOBIUS); SIGNAL_PEPTIDE_H_REGION (PHOBIUS); SIGNAL_PEPTIDE_C_REGION (PHOBIUS); SIGNAL_PEPTIDE (PHOBIUS); SIGNAL_PEPTIDE_N_REGION (PHOBIUS); IPR012336 (PROSITE_PROFILES); IPR012336 (PROSITE_PROFILES); SignalP-noTM (SIGNALP_EUK); IPR012336 (SUPERFAMILY); IPR012336 (SUPERFAMILY); IPR012336 (SUPERFAMILY); IPR012336 (SUPERFAMILY)
1821	297066_length_1410_cvg_54.0_tip_1_2	1268	superoxide dismutase	470	2.04E-80	85.20%	0.208	IPR001424 (PRINTS); IPR001424 (G3DSA:2.60.40.GENE3D); IPR001424 (PFAM); PTHR10003:SF33 (PANTHER); PTHR10003 (PANTHER); IPR018152 (PROSITE_PATTERNS); IPR018152 (PROSITE_PATTERNS); CYTOPLASMIC_DOMAIN (PHOBIUS); TRANSMEMBRANE (PHOBIUS); NON_CYTOPLASMIC_DOMAIN (PHOBIUS); IPR001424 (SUPERFAMILY)
1822	297072_length_1410_cvg_4.0_tip_1_5	92	low-density lipoprotein receptor-related protein 2	470	4.27E-13	54.60%	0.106	IPR002172 (PRINTS); IPR002172 (SMART); IPR000998 (SMART); IPR002172 (G3DSA:4.10.400.GENE3D); IPR002172 (G3DSA:4.10.400.GENE3D); IPR002172 (PFAM); IPR002172 (G3DSA:4.10.400.GENE3D); IPR000998 (PFAM); PTHR10529 (PANTHER); IPR002172 (PROSITE_PROFILES); IPR000998 (PROSITE_PROFILES); IPR002172 (SUPERFAMILY); IPR013320 (SUPERFAMILY)

1823	297086_length_1412_cvg_3.1_tip_1_5	79	protein turtle	470	5.56E-69	49.90%	0.104		IPR003598 (SMART); IPR003599 (SMART); IPR003961 (SMART); IPR013783 (G3DSA:2.60.40.GENE3D); PF13895 (PFAM); IPR013783 (G3DSA:2.60.40.GENE3D); IPR003961 (PFAM); IPR013783 (G3DSA:2.60.40.GENE3D); IPR013098 (PFAM); IPR013783 (G3DSA:2.60.40.GENE3D); PTHR10489 (PANTHER); PTHR10489:SF510 (PANTHER); CYTOPLASMIC_DOMAIN (PHOBIUS); TRANSMEMBRANE (PHOBIUS); NON_CYTOPLASMIC_DOMAIN (PHOBIUS); IPR007110 (PROSITE_PROFILES); IPR007110 (PROSITE_PROFILES); IPR003961 (PROSITE_PROFILES); IPR003961 (PROSITE_PROFILES); SSF48726 (SUPERFAMILY); SSF48726 (SUPERFAMILY); IPR003961 (SUPERFAMILY); TMhelix (TMHMM)
1824	297104_length_1414_cvg_14.4_tip_1_4	235	acyl- delta desaturase	471	1.40E-129	71.50%	0.341	Y	IPR015876 (PRINTS); IPR005804 (PFAM); PTHR11351:SF28 (PANTHER); PTHR11351 (PANTHER); CYTOPLASMIC_DOMAIN (PHOBIUS); CYTOPLASMIC_DOMAIN (PHOBIUS); TRANSMEMBRANE (PHOBIUS); TRANSMEMBRANE (PHOBIUS); TRANSMEMBRANE (PHOBIUS); NON_CYTOPLASMIC_DOMAIN (PHOBIUS); TRANSMEMBRANE (PHOBIUS); CYTOPLASMIC_DOMAIN (PHOBIUS); NON_CYTOPLASMIC_DOMAIN (PHOBIUS); TMhelix (TMHMM); TMhelix (TMHMM); TMhelix (TMHMM); TMhelix (TMHMM); TMhelix (TMHMM)
1825	297114_length_1414_cvg_7.0_tip_1_2	121	sparc-related modular calcium-binding protein 2	471	4.10E-67	66.00%	0.101		IPR000716 (SMART); IPR000716 (PFAM); IPR000716 (G3DSA:4.10.800.GENE3D); IPR011992 (G3DSA:1.10.238.GENE3D); IPR019577 (PFAM); PTHR12352:SF3 (PANTHER); PTHR12352 (PANTHER); IPR000716 (PROSITE_PATTERNS); IPR018247 (PROSITE_PATTERNS); TRANSMEMBRANE (PHOBIUS); CYTOPLASMIC_DOMAIN (PHOBIUS); NON_CYTOPLASMIC_DOMAIN (PHOBIUS); NON_CYTOPLASMIC_DOMAIN (PHOBIUS); TRANSMEMBRANE (PHOBIUS); IPR000716 (PROSITE_PROFILES); SSF47473 (SUPERFAMILY); IPR000716 (SUPERFAMILY)
1827	297122_length_1415_cvg_8.2_tip_1_5	132	casein kinase ii subunit alpha	471	0	94.30%	0.254		IPR002290 (SMART); IPR000719 (PFAM); G3DSA:1.10.510.10 (GENE3D); G3DSA:3.30.200.20 (GENE3D); PTHR24054 (PANTHER); PTHR24054:SF28 (PANTHER); IPR008271 (PROSITE_PATTERNS); IPR017441 (PROSITE_PATTERNS); TRANSMEMBRANE (PHOBIUS); CYTOPLASMIC_DOMAIN (PHOBIUS); NON_CYTOPLASMIC_DOMAIN (PHOBIUS); IPR000719 (PROSITE_PROFILES); IPR011009 (SUPERFAMILY); TMhelix (TMHMM)
1826	297118_length_1415_cvg_16.3_tip_1_4	271	#NAME?	472	1.06E-138	78.30%	0.139		IPR002347 (PRINTS); PF13561 (PFAM); IPR016040 (G3DSA:3.40.50.GENE3D); PTHR24315:SF2 (PANTHER); PTHR24315 (PANTHER); SSF51735 (SUPERFAMILY)
1828	297132_length_1416_cvg_5.0_tip_1_1	90	protein sda1 homolog isoform x2	472	3.35E-165	72.50%	0.154		IPR007949 (PFAM); IPR027312 (PTHR12730:PANTHER); PTHR12730 (PANTHER); SIGNAL_PEPTIDE_N_REGION (PHOBIUS); SIGNAL_PEPTIDE_H_REGION (PHOBIUS); NON_CYTOPLASMIC_DOMAIN (PHOBIUS); SIGNAL_PEPTIDE_C_REGION (PHOBIUS); SIGNAL_PEPTIDE (PHOBIUS); IPR016024 (SUPERFAMILY)
1829	297186_length_1422_cvg_72.4_tip_1_0	59683	cathepsin partial	474	2.58E-144	75.70%	0.108		IPR000668 (PRINTS); IPR000668 (SMART); IPR013201 (SMART); G3DSA:3.90.70.10 (GENE3D); IPR013201 (PFAM); IPR000668 (PFAM); IPR013128 (PANTHER); PTHR12411:SF57 (PANTHER); IPR025661 (PROSITE_PATTERNS); IPR025660 (PROSITE_PATTERNS); IPR000169 (PROSITE_PATTERNS); SSF54001 (SUPERFAMILY)
1830	297194_length_1422_cvg_15.8_tip_1_1	244	hydroxyacyl dehydrogenase	474	4.04E-128	83.50%	0.251		IPR002198 (PRINTS); IPR002347 (PRINTS); IPR002198 (PFAM); IPR016040 (G3DSA:3.40.50.GENE3D); PTHR24316 (PANTHER); PTHR24316:SF251 (PANTHER); IPR020904 (PROSITE_PATTERNS); SIGNAL_PEPTIDE_C_REGION (PHOBIUS); SIGNAL_PEPTIDE_N_REGION (PHOBIUS); NON_CYTOPLASMIC_DOMAIN (PHOBIUS); SIGNAL_PEPTIDE_H_REGION (PHOBIUS); SIGNAL_PEPTIDE (PHOBIUS); SSF51735 (SUPERFAMILY)
1831	297200_length_1423_cvg_12.7_tip_1_5	203	er degradation-enhancing alpha-mannosidase-like protein 2	474	3.39E-83	74.00%	0.106		IPR001382 (PRINTS); IPR001382 (PFAM); IPR001382 (G3DSA:1.50.10.GENE3D); IPR001382 (PANTHER); PTHR11742:SF37 (PANTHER); IPR001382 (SUPERFAMILY)
1832	297202_length_1423_cvg_7.0_tip_1_3	125	vitellogenin fused with superoxide dismutase	475	1.69E-30	48.90%	0.109		IPR001846 (SMART); IPR001846 (PFAM); PTHR23345 (PANTHER); PTHR23345:SF1 (PANTHER); IPR001846 (PROSITE_PROFILES)
1833	297210_length_1425_cvg_7.2_tip_1_5	140	serpin 3	475	1.32E-105	64.90%	0.835	Y	IPR023796 (SMART); IPR023796 (PFAM); G3DSA:2.30.39.10 (GENE3D); G3DSA:3.30.497.10 (GENE3D); IPR000215 (PANTHER); IPR023795 (PROSITE_PATTERNS); SignalP-noTM (SIGNALP_EUK); IPR023796 (SUPERFAMILY)

1834	297222_length_1427_cvg_22.4_tip_1_3	470	prophenoloxidase activating factor	476	2.49E-133	65.90%	0.177	IPR001314 (PRINTS); IPR001254 (SMART); G3DSA:2.40.10.10 (GENE3D); G3DSA:2.40.10.10 (GENE3D); G3DSA:2.40.10.10 (GENE3D); IPR001254 (PFAM); PTHR24268:SF95 (PANTHER); PTHR24268 (PANTHER); IPR018114 (PROSITE_PATTERNS); CYTOPLASMIC_DOMAIN (PHOBIUS); TRANSMEMBRANE (PHOBIUS); NON_CYTOPLASMIC_DOMAIN (PHOBIUS); IPR001254 (PROSITE_PROFILES); IPR009003 (SUPERFAMILY); TMhelix (TMHMM)
1835	297234_length_1429_cvg_5.5_tip_1_4	118	peroxisomal sarcosine oxidase-like	476	7.15E-103	60.40%	0.244	G3DSA:3.30.9.10 (GENE3D); IPR006076 (PFAM); G3DSA:3.50.50.60 (GENE3D); PTHR10961:SF7 (PANTHER); PTHR10961 (PANTHER); SSF51905 (SUPERFAMILY); SSF54373 (SUPERFAMILY)
1837	297274_length_1435_cvg_33.5_tip_1_2	678	serine threonine-protein phosphatase alpha-2 isoform	478	0	97.40%	0.163	IPR006186 (PRINTS); IPR006186 (SMART); IPR029052 (G3DSA:3.60.21.GENE3D); IPR004843 (PFAM); PTHR11668:SF213 (PANTHER); PTHR11668 (PANTHER); IPR006186 (PROSITE_PATTERNS); IPR029052 (SUPERFAMILY)
1836	297272_length_1435_cvg_43.4_tip_1_3	1011	lipase 3	479	4.42E-141	67.70%	0.343 Y	IPR000073 (PFAM); IPR029058 (G3DSA:3.40.50.GENE3D); IPR006693 (PFAM); PTHR11005 (PANTHER); PTHR11005:SF6 (PANTHER); IPR029058 (SUPERFAMILY)
1838	297308_length_1440_cvg_10.0_tip_1_0	186	ves g 5 allergen	480	4.61E-44	53.20%	0.188	IPR002172 (PRINTS); IPR014044 (SMART); IPR002172 (SMART); IPR002172 (PFAM); IPR002172 (G3DSA:4.10.400.GENE3D); IPR014044 (PFAM); IPR002172 (G3DSA:4.10.400.GENE3D); IPR014044 (G3DSA:3.40.33.GENE3D); IPR001283 (PANTHER); IPR023415 (PROSITE_PATTERNS); IPR023415 (PROSITE_PATTERNS); IPR023415 (PROSITE_PATTERNS); TRANSMEMBRANE (PHOBIUS); TRANSMEMBRANE (PHOBIUS); NON_CYTOPLASMIC_DOMAIN (PHOBIUS); NON_CYTOPLASMIC_DOMAIN (PHOBIUS); CYTOPLASMIC_DOMAIN (PHOBIUS); IPR002172 (PROSITE_PROFILES); IPR002172 (PROSITE_PROFILES); IPR002172 (PROSITE_PROFILES); IPR002172 (SUPERFAMILY); IPR014044 (SUPERFAMILY); IPR002172 (SUPERFAMILY); IPR002172 (SUPERFAMILY); TMhelix (TMHMM)
1839	297312_length_1440_cvg_59.0_tip_1_1	1601	von willebrand factor like 1	480	3.79E-19	41.60%	0.107	IPR001007 (PFAM); IPR001007 (PROSITE_PATTERNS); IPR001007 (PROSITE_PROFILES); IPR006207 (PROSITE_PROFILES); SSF57603 (SUPERFAMILY)
1840	297326_length_1442_cvg_17.0_tip_1_1	330	apolipoprotein d	481	9.62E-08	45.60%	0.135	IPR012674 (G3DSA:2.40.128.GENE3D); CYTOPLASMIC_DOMAIN (PHOBIUS); TRANSMEMBRANE (PHOBIUS); NON_CYTOPLASMIC_DOMAIN (PHOBIUS); IPR011038 (SUPERFAMILY)
1841	297348_length_1443_cvg_17.4_tip_1_0	279	26s protease regulatory subunit 7	481	0	95.90%	0.186	IPR003593 (SMART); IPR005937 (TIGRFAM); G3DSA:1.10.8.60 (GENE3D); IPR027417 (G3DSA:3.40.50.GENE3D); IPR003959 (PFAM); PTHR23073 (PANTHER); PTHR23073:SF13 (PANTHER); IPR003960 (PROSITE_PATTERNS); CYTOPLASMIC_DOMAIN (PHOBIUS); NON_CYTOPLASMIC_DOMAIN (PHOBIUS); TRANSMEMBRANE (PHOBIUS); IPR027417 (SUPERFAMILY); TMhelix (TMHMM)
1842	297358_length_1444_cvg_7.6_tip_1_2	130	tyrosine-protein phosphatase lar	481	0	82.20%	0.124	IPR003961 (SMART); IPR029021 (G3DSA:3.90.190.GENE3D); IPR003961 (PFAM); IPR013783 (G3DSA:2.60.40.GENE3D); IPR013783 (G3DSA:2.60.40.GENE3D); PTHR19134:SF199 (PANTHER); PTHR19134 (PANTHER); NON_CYTOPLASMIC_DOMAIN (PHOBIUS); TRANSMEMBRANE (PHOBIUS); CYTOPLASMIC_DOMAIN (PHOBIUS); IPR003961 (PROSITE_PROFILES); IPR003961 (PROSITE_PROFILES); IPR003961 (SUPERFAMILY); IPR003961 (SUPERFAMILY); TMhelix (TMHMM)
1843	297368_length_1446_cvg_58.0_tip_1_4	1956	aminoacylase-1 isoform x1	482	1.77E-154	69.10%	0.264	IPR002933 (PFAM); IPR011650 (PFAM); IPR010159 (PIRSF); G3DSA:3.40.630.10 (GENE3D); IPR010159 (TIGRFAM); IPR011650 (G3DSA:3.30.70.GENE3D); PTHR11014 (PANTHER); PTHR11014:SF8 (PANTHER); IPR001261 (PROSITE_PATTERNS); IPR001261 (PROSITE_PATTERNS); NON_CYTOPLASMIC_DOMAIN (PHOBIUS); TRANSMEMBRANE (PHOBIUS); CYTOPLASMIC_DOMAIN (PHOBIUS); SSF53187 (SUPERFAMILY); IPR011650 (SUPERFAMILY)
1844	297380_length_1447_cvg_6.2_tip_1_1	142	ankyrin repeat and kh domain-containing protein 1-like	482	5.86E-40	51.20%	0.112	Coil (COILS); IPR002110 (SMART); IPR020683 (G3DSA:1.25.40.GENE3D); IPR020683 (PFAM); PTHR23206 (PANTHER); IPR002110 (PROSITE_PROFILES); IPR020683 (PROSITE_PROFILES); IPR020683 (SUPERFAMILY)
1845	297392_length_1448_cvg_17.4_tip_1_3	340	venom carboxylesterase-6	483	2.26E-92	58.40%	0.212	IPR029058 (G3DSA:3.40.50.GENE3D); IPR002018 (PFAM); PTHR11559 (PANTHER); IPR019826 (PROSITE_PATTERNS); TRANSMEMBRANE (PHOBIUS); CYTOPLASMIC_DOMAIN (PHOBIUS); NON_CYTOPLASMIC_DOMAIN (PHOBIUS); IPR029058 (SUPERFAMILY)

1846	297434_length_1453_cvg_60.9_tip_1_2	2756	lipase 3	484	8.35E-142	67.30%	0.171		IPR006693 (PFAM); IPR029058 (G3DSA:3.40.50.GENE3D); IPR000073 (PFAM); PTHR11005 (PANTHER); PTHR11005:SF6 (PANTHER); NON_CYTOPLASMIC_DOMAIN (PHOBIUS); CYTOPLASMIC_DOMAIN (PHOBIUS); TRANSMEMBRANE (PHOBIUS); IPR029058 (SUPERFAMILY); TMhelix (TMHMM)
1847	297456_length_1454_cvg_24.3_tip_1_3	302	adiponectin receptor protein	485	3.15E-151	70.70%	0.156		IPR004254 (PFAM); PTHR20855:SF15 (PANTHER); IPR004254 (PANTHER); NON_CYTOPLASMIC_DOMAIN (PHOBIUS); NON_CYTOPLASMIC_DOMAIN (PHOBIUS); TRANSMEMBRANE (PHOBIUS); TRANSMEMBRANE (PHOBIUS); TRANSMEMBRANE (PHOBIUS); TRANSMEMBRANE (PHOBIUS); NON_CYTOPLASMIC_DOMAIN (PHOBIUS); CYTOPLASMIC_DOMAIN (PHOBIUS); CYTOPLASMIC_DOMAIN (PHOBIUS); TRANSMEMBRANE (PHOBIUS); CYTOPLASMIC_DOMAIN (PHOBIUS); TRANSMEMBRANE (PHOBIUS); TRANSMEMBRANE (PHOBIUS); NON_CYTOPLASMIC_DOMAIN (PHOBIUS); CYTOPLASMIC_DOMAIN (PHOBIUS); TMhelix (TMHMM); TMhelix (TMHMM); TMhelix (TMHMM); TMhelix (TMHMM); TMhelix (TMHMM); TMhelix (TMHMM); TMhelix (TMHMM)
1848	297474_length_1456_cvg_82.0_tip_1_2	4246	ferritin 2 light chain homologue	485	1.33E-34	53.70%	0.843	Y	IPR012347 (G3DSA:1.20.1260.GENE3D); IPR008331 (PFAM); IPR001519 (PANTHER); PTHR11431:SF28 (PANTHER); NON_CYTOPLASMIC_DOMAIN (PHOBIUS); SIGNAL_PEPTIDE_N_REGION (PHOBIUS); SIGNAL_PEPTIDE (PHOBIUS); SIGNAL_PEPTIDE_C_REGION (PHOBIUS); SIGNAL_PEPTIDE_H_REGION (PHOBIUS); IPR009040 (PROSITE_PROFILES); SignalP-noTM (SIGNALP_EUK); SignalP-noTM (SIGNALP_GRAM_NEGATIVE); SignalP-TM (SIGNALP_GRAM_POSITIVE); IPR009078 (SUPERFAMILY)
1849	297562_length_1469_cvg_34.0_tip_1_3	4421	universal minicircle sequence binding protein	490	2.94E-27	53.00%	0.113		Coil (COILS); Coil (COILS); IPR001878 (SMART); IPR001878 (G3DSA:4.10.60.GENE3D); IPR001878 (G3DSA:4.10.60.GENE3D); IPR001878 (PFAM); PTHR23002 (PANTHER); NON_CYTOPLASMIC_DOMAIN (PHOBIUS); CYTOPLASMIC_DOMAIN (PHOBIUS); TRANSMEMBRANE (PHOBIUS); IPR001878 (PROSITE_PROFILES); IPR001878 (PROSITE_PROFILES); IPR001878 (PROSITE_PROFILES); IPR001878 (PROSITE_PROFILES); IPR001878 (PROSITE_PROFILES); IPR001878 (PROSITE_PROFILES); IPR001878 (SUPERFAMILY); IPR001878 (SUPERFAMILY); IPR001878 (SUPERFAMILY); IPR001878 (SUPERFAMILY)
1850	297580_length_1471_cvg_28.1_tip_1_1	770	perlucin-like protein	490	2.47E-19	53.70%	0.864	Y	Coil (COILS); IPR001304 (SMART); IPR001304 (PFAM); IPR016186 (G3DSA:3.10.100.GENE3D); PTHR22802 (PANTHER); IPR018378 (PROSITE_PATTERNS); NON_CYTOPLASMIC_DOMAIN (PHOBIUS); TRANSMEMBRANE (PHOBIUS); TRANSMEMBRANE (PHOBIUS); NON_CYTOPLASMIC_DOMAIN (PHOBIUS); CYTOPLASMIC_DOMAIN (PHOBIUS); IPR001304 (PROSITE_PROFILES); SignalP-noTM (SIGNALP_EUK); IPR016187 (SUPERFAMILY); TMhelix (TMHMM)
1851	297622_length_1475_cvg_8.5_tip_1_4	179	12 kda fk506-binding protein	492	1.23E-61	90.10%	0.166		IPR001179 (PFAM); G3DSA:3.10.50.40 (GENE3D); PTHR10516:SF272 (PANTHER); IPR023566 (PANTHER); CYTOPLASMIC_DOMAIN (PHOBIUS); TRANSMEMBRANE (PHOBIUS); NON_CYTOPLASMIC_DOMAIN (PHOBIUS); TRANSMEMBRANE (PHOBIUS); CYTOPLASMIC_DOMAIN (PHOBIUS); IPR001179 (PROSITE_PROFILES); SSF54534 (SUPERFAMILY)
1874	297934_length_1523_cvg_56.4_tip_1_1	279613	hemocyanin subunit type 2 precursor	492	0	74.50%	0.101		IPR005203 (G3DSA:2.60.40.GENE3D); IPR005203 (PFAM); IPR000896 (PFAM); IPR008922 (G3DSA:1.10.1280.GENE3D); IPR013788 (PANTHER); IPR002227 (PROSITE_PATTERNS); IPR013788 (PROSITE_PATTERNS); CYTOPLASMIC_DOMAIN (PHOBIUS); TRANSMEMBRANE (PHOBIUS); NON_CYTOPLASMIC_DOMAIN (PHOBIUS); IPR008922 (SUPERFAMILY); IPR014756 (SUPERFAMILY)
1852	297638_length_1478_cvg_3.0_tip_1_3	67	endoplasmic reticulum resident protein 44 isoform x3	493	0	79.10%	0.252		IPR012336 (G3DSA:3.40.30.GENE3D); IPR013766 (PFAM); IPR012336 (G3DSA:3.40.30.GENE3D); PF13848 (PFAM); PTHR18929 (PANTHER); PTHR18929:SF49 (PANTHER); IPR012336 (PROSITE_PROFILES); IPR012336 (SUPERFAMILY); IPR012336 (SUPERFAMILY); IPR012336 (SUPERFAMILY)
1853	297648_length_1478_cvg_8.6_tip_1_4	161	lysosomal alpha-mannosidase	493	5.13E-156	65.70%	0.147		IPR015341 (SMART); G3DSA:1.10.287.530 (GENE3D); IPR015341 (PFAM); IPR011682 (PFAM); IPR013780 (G3DSA:2.60.40.GENE3D); G3DSA:2.70.98.30 (GENE3D); PTHR11607 (PANTHER); PTHR11607:SF3 (PANTHER); IPR011013 (SUPERFAMILY); IPR028995 (SUPERFAMILY)
1854	297650_length_1479_cvg_37.3_tip_1_3	736	aminoacylase-1	493	0	75.60%	0.101		G3DSA:3.40.630.10 (GENE3D); IPR011650 (G3DSA:3.30.70.GENE3D); IPR002933 (PFAM); IPR011650 (PFAM); IPR010159 (PIRSF); IPR010159 (TIGRFAM); PTHR11014:SF8 (PANTHER); PTHR11014 (PANTHER); IPR001261 (PROSITE_PATTERNS); IPR001261 (PROSITE_PATTERNS); CYTOPLASMIC_DOMAIN (PHOBIUS); TRANSMEMBRANE (PHOBIUS); NON_CYTOPLASMIC_DOMAIN (PHOBIUS); NON_CYTOPLASMIC_DOMAIN (PHOBIUS); TRANSMEMBRANE (PHOBIUS); IPR011650 (SUPERFAMILY); SSF53187 (SUPERFAMILY); TMhelix (TMHMM); TMhelix (TMHMM)

1855	297680_length_1483_cvg_56.5_tip_0_2	1204	coagulation factor v	494	3.53E-10	43.00%	0.348 Y	TRANSMEMBRANE (PHOBIUS); CYTOPLASMIC_DOMAIN (PHOBIUS); TRANSMEMBRANE (PHOBIUS); NON_CYTOPLASMIC_DOMAIN (PHOBIUS); TRANSMEMBRANE (PHOBIUS); NON_CYTOPLASMIC_DOMAIN (PHOBIUS); CYTOPLASMIC_DOMAIN (PHOBIUS); NON_CYTOPLASMIC_DOMAIN (PHOBIUS); TRANSMEMBRANE (PHOBIUS); CYTOPLASMIC_DOMAIN (PHOBIUS); SIGNAL_PEPTIDE (PHOBIUS); TRANSMEMBRANE (PHOBIUS); CYTOPLASMIC_DOMAIN (PHOBIUS); NON_CYTOPLASMIC_DOMAIN (PHOBIUS); CYTOPLASMIC_DOMAIN (PHOBIUS); CYTOPLASMIC_DOMAIN (PHOBIUS); TRANSMEMBRANE (PHOBIUS); NON_CYTOPLASMIC_DOMAIN (PHOBIUS); SIGNAL_PEPTIDE_N_REGION (PHOBIUS); TRANSMEMBRANE (PHOBIUS); TRANSMEMBRANE (PHOBIUS); TRANSMEMBRANE (PHOBIUS); NON_CYTOPLASMIC_DOMAIN (PHOBIUS); CYTOPLASMIC_DOMAIN (PHOBIUS); SIGNAL_PEPTIDE_C_REGION (PHOBIUS); TRANSMEMBRANE (PHOBIUS); TRANSMEMBRANE (PHOBIUS); NON_CYTOPLASMIC_DOMAIN (PHOBIUS); TRANSMEMBRANE (PHOBIUS); TRANSMEMBRANE (PHOBIUS); TRANSMEMBRANE (PHOBIUS); TRANSMEMBRANE (PHOBIUS); CYTOPLASMIC_DOMAIN (PHOBIUS); SIGNAL_PEPTIDE_H_REGION (PHOBIUS); NON_CYTOPLASMIC_DOMAIN (PHOBIUS); TMhelix (TMHMM); TMhelix (TMHMM); TMhelix (TMHMM); TMhelix (TMHMM); TMhelix (TMHMM); TMhelix (TMHMM); TMhelix (TMHMM); TMhelix (TMHMM); TMhelix (TMHMM); TMhelix (TMHMM); TMhelix (TMHMM); TMhelix (TMHMM); TMhelix (TMHMM)
1856	297706_length_1486_cvg_2.0_tip_1_4	58	macrophage mannose receptor 1-like	495	3.26E-88	51.70%	0.11	IPR001304 (SMART); IPR016186 (G3DSA:3.10.100.GENE3D); IPR001304 (PFAM); IPR016186 (G3DSA:3.10.100.GENE3D); IPR016186 (G3DSA:3.10.100.GENE3D); IPR016186 (G3DSA:3.10.100.GENE3D); PTHR22803 (PANTHER); PTHR22803:SF16 (PANTHER); IPR018378 (PROSITE_PATTERNS); IPR018378 (PROSITE_PATTERNS); IPR001304 (PROSITE_PROFILES); IPR001304 (PROSITE_PROFILES); IPR001304 (PROSITE_PROFILES); IPR001304 (PROSITE_PROFILES); IPR016187 (SUPERFAMILY); IPR016187 (SUPERFAMILY); IPR016187 (SUPERFAMILY); IPR016187 (SUPERFAMILY)
1857	297730_length_1491_cvg_7.1_tip_1_2	147	flavin-containing monooxygenase fmo gs-ox4-like isoform x1	497	2.37E-134	62.90%	0.15	IPR000960 (PRINTS); IPR020946 (PFAM); G3DSA:3.50.50.60 (GENE3D); G3DSA:3.50.50.60 (GENE3D); PTHR23023 (PANTHER); PTHR23023:SF4 (PANTHER); SSF51905 (SUPERFAMILY); SSF51905 (SUPERFAMILY)
1858	297738_length_1492_cvg_7.4_tip_1_3	135	otopetrin-2-like isoform x2	498	4.54E-78	85.70%	0.128	IPR004878 (PFAM); PTHR21522:SF41 (PANTHER); IPR004878 (PANTHER); TRANSMEMBRANE (PHOBIUS); TRANSMEMBRANE (PHOBIUS); TRANSMEMBRANE (PHOBIUS); CYTOPLASMIC_DOMAIN (PHOBIUS); NON_CYTOPLASMIC_DOMAIN (PHOBIUS); CYTOPLASMIC_DOMAIN (PHOBIUS); NON_CYTOPLASMIC_DOMAIN (PHOBIUS); TRANSMEMBRANE (PHOBIUS); NON_CYTOPLASMIC_DOMAIN (PHOBIUS); TMhelix (TMHMM); TMhelix (TMHMM); TMhelix (TMHMM); TMhelix (TMHMM)
1859	297744_length_1493_cvg_5.7_tip_1_3	118	zinc finger protein	498	2.80E-31	53.40%	0.099	IPR015880 (SMART); IPR013087 (G3DSA:3.30.160.GENE3D); PF13894 (PFAM); IPR013087 (G3DSA:3.30.160.GENE3D); PF13465 (PFAM); PTHR24409:SF14 (PANTHER); PTHR24409 (PANTHER); IPR007087 (PROSITE_PATTERNS); IPR007087 (PROSITE_PATTERNS); IPR007087 (PROSITE_PATTERNS); IPR007087 (PROSITE_PATTERNS); IPR007087 (PROSITE_PATTERNS); IPR007087 (PROSITE_PROFILES); IPR007087 (PROSITE_PROFILES); IPR007087 (PROSITE_PROFILES); IPR007087 (PROSITE_PROFILES); IPR007087 (PROSITE_PROFILES); SSF57667 (SUPERFAMILY); SSF57667 (SUPERFAMILY); SSF57667 (SUPERFAMILY)
1860	297750_length_1493_cvg_5.6_tip_1_0	115	nicotinic acetylcholine receptor subunit alpha10	498	1.63E-84	56.30%	0.117	Coil (COILS); IPR006029 (PFAM); IPR006202 (PFAM); IPR027361 (G3DSA:1.20.120.GENE3D); IPR006202 (G3DSA:2.70.170.GENE3D); IPR006201 (PANTHER); PTHR18945:SF481 (PANTHER); TRANSMEMBRANE (PHOBIUS); TRANSMEMBRANE (PHOBIUS); NON_CYTOPLASMIC_DOMAIN (PHOBIUS); NON_CYTOPLASMIC_DOMAIN (PHOBIUS); CYTOPLASMIC_DOMAIN (PHOBIUS); TRANSMEMBRANE (PHOBIUS); CYTOPLASMIC_DOMAIN (PHOBIUS); NON_CYTOPLASMIC_DOMAIN (PHOBIUS); TRANSMEMBRANE (PHOBIUS); IPR006029 (SUPERFAMILY); IPR006202 (SUPERFAMILY); TMhelix (TMHMM); TMhelix (TMHMM); TMhelix (TMHMM); TMhelix (TMHMM)
1861	297754_length_1494_cvg_3.6_tip_1_1	78	discs large 1 tumor suppressor	498	0	89.80%	0.107	Coil (COILS); IPR008145 (SMART); IPR001478 (SMART); IPR001452 (SMART); IPR027417 (G3DSA:3.40.50.GENE3D); IPR011511 (PFAM); IPR001478 (PFAM); G3DSA:3.30.63.10 (GENE3D); IPR001478 (G3DSA:2.30.42.GENE3D); G3DSA:2.30.30.40 (GENE3D); IPR008145 (PFAM); IPR016313 (PANTHER); IPR008144 (PROSITE_PROFILES); IPR001478 (PROSITE_PROFILES); IPR001452 (PROSITE_PROFILES); IPR027417 (SUPERFAMILY); IPR001478 (SUPERFAMILY); IPR001452 (SUPERFAMILY)

1862	297756_length_1494_cvg_5.8_tip_1_4	105	sulfhydryl oxidase 2-like	498	5.70E-80	54.20%	0.108	IPR017905 (PFAM); IPR017905 (G3DSA:1.20.120.GENE3D); PTHR22897:SF8 (PANTHER); PTHR22897 (PANTHER); NON_CYTOPLASMIC_DOMAIN (PHOBIUS); CYTOPLASMIC_DOMAIN (PHOBIUS); TRANSMEMBRANE (PHOBIUS); IPR017905 (PROSITE_PROFILES); IPR017905 (SUPERFAMILY); TMhelix (TMHMM)
1863	297760_length_1496_cvg_5.7_tip_1_2	117	low-density lipoprotein receptor-related protein 2	498	8.47E-32	49.50%	0.108	IPR002172 (PRINTS); IPR002172 (SMART); IPR002172 (G3DSA:4.10.400.GENE3D); IPR002172 (PFAM); IPR002172 (G3DSA:4.10.400.GENE3D); IPR002172 (G3DSA:4.10.400.GENE3D); PTHR10529 (PANTHER); IPR023415 (PROSITE_PATTERNS); IPR023415 (PROSITE_PATTERNS); IPR023415 (PROSITE_PATTERNS); IPR002172 (PROSITE_PROFILES); IPR002172 (PROSITE_PROFILES); IPR002172 (PROSITE_PROFILES); IPR002172 (PROSITE_PROFILES); IPR002172 (PROSITE_PROFILES); IPR002172 (PROSITE_PROFILES); SignalP-TM (SIGNALP_GRAM_POSITIVE); IPR002172 (SUPERFAMILY); IPR002172 (SUPERFAMILY); IPR002172 (SUPERFAMILY); IPR002172 (SUPERFAMILY)
1864	297776_length_1498_cvg_55.8_tip_1_0	1417	fk506-binding protein 2 isoform x2	500	2.48E-99	79.00%	0.234	IPR011992 (PFAM); IPR011992 (G3DSA:1.10.238.GENE3D); G3DSA:3.10.50.40 (GENE3D); IPR001179 (PFAM); IPR023566 (PANTHER); PTHR10516:SF252 (PANTHER); IPR018247 (PROSITE_PATTERNS); IPR018247 (PROSITE_PATTERNS); SIGNAL_PEPTIDE_N_REGION (PHOBIUS); CYTOPLASMIC_DOMAIN (PHOBIUS); TRANSMEMBRANE (PHOBIUS); CYTOPLASMIC_DOMAIN (PHOBIUS); NON_CYTOPLASMIC_DOMAIN (PHOBIUS); NON_CYTOPLASMIC_DOMAIN (PHOBIUS); SIGNAL_PEPTIDE_C_REGION (PHOBIUS); SIGNAL_PEPTIDE_H_REGION (PHOBIUS); TRANSMEMBRANE (PHOBIUS); SIGNAL_PEPTIDE (PHOBIUS); TRANSMEMBRANE (PHOBIUS); IPR002048 (PROSITE_PROFILES); IPR001179 (PROSITE_PROFILES); IPR002048 (PROSITE_PROFILES); SSF47473 (SUPERFAMILY); SSF54534 (SUPERFAMILY); TMhelix (TMHMM)
1889	298156_length_1550_cvg_13.0_tip_1_3	264	lissencephaly-1 homolog isoform x1	500	0	91.90%	0.206	IPR020472 (PRINTS); IPR001680 (SMART); IPR015943 (G3DSA:2.130.10.GENE3D); IPR001680 (PFAM); PTHR22847 (PANTHER); IPR019775 (PROSITE_PATTERNS); IPR019775 (PROSITE_PATTERNS); IPR019775 (PROSITE_PATTERNS); IPR019775 (PROSITE_PATTERNS); IPR019775 (PROSITE_PATTERNS); IPR019775 (PROSITE_PATTERNS); TRANSMEMBRANE (PHOBIUS); CYTOPLASMIC_DOMAIN (PHOBIUS); NON_CYTOPLASMIC_DOMAIN (PHOBIUS); TRANSMEMBRANE (PHOBIUS); NON_CYTOPLASMIC_DOMAIN (PHOBIUS); IPR001680 (PROSITE_PROFILES); IPR001680 (PROSITE_PROFILES); IPR001680 (PROSITE_PROFILES); IPR017986 (PROSITE_PROFILES); IPR001680 (PROSITE_PROFILES); IPR001680 (PROSITE_PROFILES); IPR001680 (PROSITE_PROFILES); IPR001680 (PROSITE_PROFILES); IPR017986 (SUPERFAMILY)
1865	297800_length_1501_cvg_17.9_tip_1_0	333	neurofilament triplet h protein	501	1.43E-28	51.70%	0.108	TRANSMEMBRANE (PHOBIUS); CYTOPLASMIC_DOMAIN (PHOBIUS); TRANSMEMBRANE (PHOBIUS); NON_CYTOPLASMIC_DOMAIN (PHOBIUS); TRANSMEMBRANE (PHOBIUS); CYTOPLASMIC_DOMAIN (PHOBIUS); NON_CYTOPLASMIC_DOMAIN (PHOBIUS); TMhelix (TMHMM); TMhelix (TMHMM); TMhelix (TMHMM)
1866	297812_length_1503_cvg_8.6_tip_1_5	174	collagen alpha-1 chain-like	501	8.70E-121	81.50%	0.154	IPR001442 (SMART); IPR001442 (G3DSA:2.170.240.GENE3D); IPR001442 (PFAM); PTHR24023:SF381 (PANTHER); PTHR24023 (PANTHER); NON_CYTOPLASMIC_DOMAIN (PHOBIUS); CYTOPLASMIC_DOMAIN (PHOBIUS); TRANSMEMBRANE (PHOBIUS); IPR001442 (PROSITE_PROFILES); IPR001442 (PROSITE_PROFILES); IPR016187 (SUPERFAMILY); IPR016187 (SUPERFAMILY)
1867	297838_length_1506_cvg_32.4_tip_1_1	736	torsin- partial	502	1.39E-109	66.80%	0.441 Y	Coil (COILS); IPR027417 (G3DSA:3.40.50.GENE3D); IPR010448 (PFAM); IPR010448 (PANTHER); TRANSMEMBRANE (PHOBIUS); SIGNAL_PEPTIDE_H_REGION (PHOBIUS); CYTOPLASMIC_DOMAIN (PHOBIUS); SIGNAL_PEPTIDE (PHOBIUS); SIGNAL_PEPTIDE_C_REGION (PHOBIUS); NON_CYTOPLASMIC_DOMAIN (PHOBIUS); TRANSMEMBRANE (PHOBIUS); NON_CYTOPLASMIC_DOMAIN (PHOBIUS); SIGNAL_PEPTIDE_N_REGION (PHOBIUS); SignalP-TM (SIGNALP_GRAM_POSITIVE); IPR027417 (SUPERFAMILY); TMhelix (TMHMM)
1868	297866_length_1511_cvg_6.2_tip_1_2	135	scaffolding protein	503	1.01E-19	64.50%	0.099	Coil (COILS); NON_CYTOPLASMIC_DOMAIN (PHOBIUS); TRANSMEMBRANE (PHOBIUS); CYTOPLASMIC_DOMAIN (PHOBIUS)
1869	297878_length_1514_cvg_3.0_tip_1_0	73	hemicentin-1 isoform x1	505	6.59E-79	58.50%	0.101	IPR003598 (SMART); IPR013783 (G3DSA:2.60.40.GENE3D); PF13927 (PFAM); IPR013783 (G3DSA:2.60.40.GENE3D); PTHR11640 (PANTHER); PTHR11640:SF6 (PANTHER); NON_CYTOPLASMIC_DOMAIN (PHOBIUS); TRANSMEMBRANE (PHOBIUS); CYTOPLASMIC_DOMAIN (PHOBIUS); IPR007110 (PROSITE_PROFILES); IPR007110 (PROSITE_PROFILES); SSF48726 (SUPERFAMILY); SSF48726 (SUPERFAMILY); TMhelix (TMHMM)

1870	297896_length_1516_cvg_60.5_tip_1_5	27199	myosin heavy chain	505	0	88.80%	0.099		Coil (COILS); Coil (COILS); Coil (COILS); IPR000048 (SMART); IPR002928 (PFAM); G3DSA:1.20.5.340 (GENE3D); IPR027401 (G3DSA:4.10.270.GENE3D); PTHR13140 (PANTHER); PTHR13140:SF364 (PANTHER); IPR000048 (PROSITE_PROFILES); IPR001609 (PROSITE_PROFILES); SSF90257 (SUPERFAMILY); SSF90257 (SUPERFAMILY); IPR027417 (SUPERFAMILY); SSF90257 (SUPERFAMILY)
1871	297900_length_1517_cvg_52.1_tip_1_2	1715	masquerade-like protein	505	1.29E-63	49.40%	0.122		IPR001314 (PRINTS); IPR001254 (SMART); G3DSA:2.40.10.10 (GENE3D); G3DSA:2.40.10.10 (GENE3D); G3DSA:2.40.10.10 (GENE3D); IPR001254 (PFAM); PTHR24268 (PANTHER); PTHR24268:SF95 (PANTHER); IPR018114 (PROSITE_PATTERNS); IPR001254 (PROSITE_PROFILES); IPR009003 (SUPERFAMILY)
1872	297906_length_1517_cvg_21.1_tip_1_2	423	tribbles homolog 2	505	2.99E-123	74.40%	0.134		IPR002290 (SMART); IPR000719 (PFAM); G3DSA:3.30.200.20 (GENE3D); G3DSA:1.10.510.10 (GENE3D); IPR024104 (PANTHER); PTHR22961:SF4 (PANTHER); IPR000719 (PROSITE_PROFILES); IPR011009 (SUPERFAMILY)
1873	297922_length_1522_cvg_60.4_tip_1_3	6590	nidogen-2 isoform x5	508	5.80E-20	52.40%	0.735	Y	IPR000716 (SMART); IPR000716 (G3DSA:4.10.800.GENE3D); IPR000716 (PFAM); IPR022339 (PANTHER); IPR000716 (PROSITE_PATTERNS); IPR000716 (PROSITE_PATTERNS); SIGNAL_PEPTIDE (PHOBIUS); SIGNAL_PEPTIDE_N_REGION (PHOBIUS); NON_CYTOPLASMIC_DOMAIN (PHOBIUS); TRANSMEMBRANE (PHOBIUS); CYTOPLASMIC_DOMAIN (PHOBIUS); SIGNAL_PEPTIDE_C_REGION (PHOBIUS); SIGNAL_PEPTIDE_H_REGION (PHOBIUS); IPR000716 (PROSITE_PROFILES); IPR000716 (PROSITE_PROFILES); SignalP-noTM (SIGNALP_EUK); IPR000716 (SUPERFAMILY); IPR000716 (SUPERFAMILY)
1875	297942_length_1524_cvg_25.0_tip_1_2	557	agap007053-pa-like protein	508	9.14E-75	49.90%	0.162		Coil (COILS); IPR000716 (SMART); IPR000716 (PFAM); IPR000716 (G3DSA:4.10.800.GENE3D); PTHR12352 (PANTHER); PTHR12352:SF9 (PANTHER); NON_CYTOPLASMIC_DOMAIN (PHOBIUS); CYTOPLASMIC_DOMAIN (PHOBIUS); TRANSMEMBRANE (PHOBIUS); CYTOPLASMIC_DOMAIN (PHOBIUS); TRANSMEMBRANE (PHOBIUS); IPR000716 (PROSITE_PROFILES); IPR000716 (PROSITE_PROFILES); IPR000716 (PROSITE_PROFILES); IPR000716 (SUPERFAMILY); IPR000716 (SUPERFAMILY); IPR000716 (SUPERFAMILY); TMhelix (TMHMM)
1876	297982_length_1528_cvg_16.7_tip_1_3	265	cytochrome p450 monooxygenase	510	1.83E-125	58.70%	0.189		Coil (COILS); IPR001128 (PRINTS); IPR002401 (PRINTS); IPR001128 (PFAM); IPR001128 (G3DSA:1.10.630.GENE3D); PTHR24300 (PANTHER); IPR017972 (PROSITE_PATTERNS); TRANSMEMBRANE (PHOBIUS); CYTOPLASMIC_DOMAIN (PHOBIUS); NON_CYTOPLASMIC_DOMAIN (PHOBIUS); IPR001128 (SUPERFAMILY); TMhelix (TMHMM); TMhelix (TMHMM)
1877	297990_length_1529_cvg_9.0_tip_1_0	159	dnaj homolog subfamily b member 11	510	0	84.20%	0.860	Y	IPR001623 (PRINTS); IPR001623 (SMART); G3DSA:2.60.260.20 (GENE3D); G3DSA:2.60.260.20 (GENE3D); IPR002939 (PFAM); IPR001623 (PFAM); IPR001623 (G3DSA:1.10.287.GENE3D); PTHR24077 (PANTHER); PTHR24077:SF210 (PANTHER); IPR018253 (PROSITE_PATTERNS); CYTOPLASMIC_DOMAIN (PHOBIUS); NON_CYTOPLASMIC_DOMAIN (PHOBIUS); SIGNAL_PEPTIDE_C_REGION (PHOBIUS); TRANSMEMBRANE (PHOBIUS); SIGNAL_PEPTIDE_H_REGION (PHOBIUS); SIGNAL_PEPTIDE_N_REGION (PHOBIUS); SIGNAL_PEPTIDE (PHOBIUS); IPR001623 (PROSITE_PROFILES); SignalP-noTM (SIGNALP_EUK); IPR008971 (SUPERFAMILY); IPR001623 (SUPERFAMILY); IPR008971 (SUPERFAMILY); TMhelix (TMHMM)
1878	297996_length_1529_cvg_15.0_tip_1_1	232	chitinase domain-containing protein 1 isoform x1	510	1.74E-149	70.30%	0.165		IPR011583 (SMART); IPR001223 (PFAM); IPR013781 (G3DSA:3.20.20.GENE3D); PTHR11177:SF135 (PANTHER); PTHR11177 (PANTHER); TRANSMEMBRANE (PHOBIUS); CYTOPLASMIC_DOMAIN (PHOBIUS); NON_CYTOPLASMIC_DOMAIN (PHOBIUS); IPR017853 (SUPERFAMILY); TMhelix (TMHMM)
1879	297998_length_1529_cvg_6.0_tip_1_0	121	serine threonine-protein kinase warts	510	0	90.20%	0.111		IPR002290 (SMART); IPR000961 (SMART); G3DSA:3.30.200.20 (GENE3D); IPR000719 (PFAM); G3DSA:1.10.510.10 (GENE3D); IPR028741 (PTHR24356:PANTHER); PTHR24356 (PANTHER); IPR008271 (PROSITE_PATTERNS); NON_CYTOPLASMIC_DOMAIN (PHOBIUS); CYTOPLASMIC_DOMAIN (PHOBIUS); TRANSMEMBRANE (PHOBIUS); IPR000719 (PROSITE_PROFILES); IPR000961 (PROSITE_PROFILES); IPR011009 (SUPERFAMILY)
1880	298018_length_1534_cvg_32.6_tip_1_4	723	nidogen- partial	511	1.03E-106	73.40%	0.101		IPR000033 (SMART); IPR000033 (PFAM); IPR011042 (G3DSA:2.120.10.GENE3D); PTHR10529 (PANTHER); NON_CYTOPLASMIC_DOMAIN (PHOBIUS); CYTOPLASMIC_DOMAIN (PHOBIUS); TRANSMEMBRANE (PHOBIUS); IPR000033 (PROSITE_PROFILES); IPR000033 (PROSITE_PROFILES); IPR000033 (PROSITE_PROFILES); IPR000033 (PROSITE_PROFILES); IPR000033 (PROSITE_PROFILES); SSF63825 (SUPERFAMILY); TMhelix (TMHMM)

1882	298022_length_1534_cvg_4.3_tip_1_5	97	collagen and calcium-binding egf domain-containing protein 1	511	4.46E-100	52.80%	0.307	Coil (COILS); IPR000742 (SMART); IPR001881 (SMART); G3DSA:2.10.25.10 (GENE3D); IPR008160 (PFAM); PF14670 (PFAM); G3DSA:2.10.25.10 (GENE3D); PTHR24023 (PANTHER); IPR013032 (PROSITE_PATTERNS); NON_CYTOPLASMIC_DOMAIN (PHOBIUS); SIGNAL_PEPTIDE_H_REGION (PHOBIUS); SIGNAL_PEPTIDE_N_REGION (PHOBIUS); SIGNAL_PEPTIDE_C_REGION (PHOBIUS); SIGNAL_PEPTIDE (PHOBIUS); IPR000742 (PROSITE_PROFILES); SSF57196 (SUPERFAMILY)
1881	298022_length_1534_cvg_4.3_tip_1_0	97	hypothetical protein CRE_06355	512	7.35E-07	44.00%	0.137	no IPS match
1883	298032_length_1536_cvg_12.9_tip_1_3	236	von willebrand factor like 1	512	1.27E-07	38.75%	0.114	IPR001007 (PROSITE_PATTERNS); PD968187 (PRODOM); IPR001007 (PROSITE_PROFILES)
1884	298056_length_1538_cvg_35.0_tip_1_5	643	scp-like extracellular domain containing protein 1	512	7.00E-37	49.40%	0.417 Y	IPR002413 (PRINTS); IPR001283 (PRINTS); IPR002172 (SMART); IPR014044 (SMART); IPR014044 (PFAM); IPR002172 (G3DSA:4.10.400.GENE3D); IPR014044 (G3DSA:3.40.33.GENE3D); IPR002172 (G3DSA:4.10.400.GENE3D); IPR002172 (PFAM); IPR001283 (PANTHER); IPR023415 (PROSITE_PATTERNS); IPR023415 (PROSITE_PATTERNS); IPR018244 (PROSITE_PATTERNS); TRANSMEMBRANE (PHOBIUS); CYTOPLASMIC_DOMAIN (PHOBIUS); NON_CYTOPLASMIC_DOMAIN (PHOBIUS); IPR002172 (PROSITE_PROFILES); IPR002172 (PROSITE_PROFILES); IPR002172 (SUPERFAMILY); IPR002172 (SUPERFAMILY); IPR014044 (SUPERFAMILY); TMhelix (TMHMM)
1885	298088_length_1541_cvg_5.2_tip_1_0	97	carbohydrate sulfotransferase 11-like	514	9.54E-67	55.00%	0.176	IPR005331 (PFAM); IPR018011 (PANTHER); CYTOPLASMIC_DOMAIN (PHOBIUS); TRANSMEMBRANE (PHOBIUS); NON_CYTOPLASMIC_DOMAIN (PHOBIUS); TMhelix (TMHMM)
1886	298092_length_1543_cvg_3.9_tip_1_1	74	dnaj homolog subfamily b member 12	514	1.19E-135	81.60%	0.1	IPR001623 (PRINTS); IPR001623 (SMART); IPR001623 (G3DSA:1.10.287.GENE3D); IPR015399 (PFAM); IPR001623 (PFAM); PTHR24077:SF239 (PANTHER); PTHR24077 (PANTHER); IPR018253 (PROSITE_PATTERNS); TRANSMEMBRANE (PHOBIUS); CYTOPLASMIC_DOMAIN (PHOBIUS); NON_CYTOPLASMIC_DOMAIN (PHOBIUS); IPR001623 (PROSITE_PROFILES); IPR001623 (SUPERFAMILY); TMhelix (TMHMM)
1887	298120_length_1546_cvg_20.8_tip_1_5	436	acidic phospholipase a2 pa4 isoform x2	515	9.19E-43	56.90%	0.846 Y	IPR001211 (PFAM); IPR016090 (G3DSA:1.20.90.GENE3D); PTHR12253 (PANTHER); IPR013090 (PROSITE_PATTERNS); NON_CYTOPLASMIC_DOMAIN (PHOBIUS); SIGNAL_PEPTIDE_N_REGION (PHOBIUS); SIGNAL_PEPTIDE_C_REGION (PHOBIUS); SIGNAL_PEPTIDE_H_REGION (PHOBIUS); SIGNAL_PEPTIDE (PHOBIUS); SignalP-noTM (SIGNALP_GRAM_NEGATIVE); SignalP-noTM (SIGNALP_EUK); IPR016090 (SUPERFAMILY); TMhelix (TMHMM)
1888	298132_length_1547_cvg_9.3_tip_1_3	156	microfibrillar-associated protein 1-like	516	5.18E-177	78.00%	0.1	Coil (COILS); Coil (COILS); Coil (COILS); Coil (COILS); IPR009730 (PFAM); IPR009730 (PANTHER); PTHR15327:SF0 (PANTHER)
1890	298158_length_1550_cvg_6.8_tip_1_0	153	dentin sialophospho	517	5.74E-23	43.10%	0.113	no IPS match
1891	298196_length_1555_cvg_23.1_tip_1_3	360	iron zinc purple acid phosphatase-like protein	519	1.39E-129	78.40%	0.101	IPR029052 (G3DSA:3.60.21.GENE3D); IPR025733 (PFAM); IPR004843 (PFAM); PTHR22953 (PANTHER); PTHR22953:SF9 (PANTHER); TRANSMEMBRANE (PHOBIUS); CYTOPLASMIC_DOMAIN (PHOBIUS); NON_CYTOPLASMIC_DOMAIN (PHOBIUS); IPR029052 (SUPERFAMILY); TMhelix (TMHMM); TMhelix (TMHMM)
1892	298224_length_1557_cvg_6.2_tip_1_5	135	f-box wd repeat-containing protein 7	519	0	87.90%	0.104	IPR020472 (PRINTS); IPR001810 (SMART); IPR001680 (SMART); G3DSA:1.20.1280.50 (GENE3D); IPR001810 (PFAM); IPR015943 (G3DSA:2.130.10.GENE3D); IPR001680 (PFAM); PTHR22844 (PANTHER); PTHR22844:SF116 (PANTHER); IPR019775 (PROSITE_PATTERNS); IPR019775 (PROSITE_PATTERNS); IPR019775 (PROSITE_PATTERNS); IPR019775 (PROSITE_PATTERNS); IPR019775 (PROSITE_PATTERNS); IPR001680 (PROSITE_PROFILES); IPR001810 (PROSITE_PROFILES); IPR001680 (PROSITE_PROFILES); IPR001680 (PROSITE_PROFILES); IPR001680 (PROSITE_PROFILES); IPR017986 (PROSITE_PROFILES); IPR001680 (PROSITE_PROFILES); IPR001680 (PROSITE_PROFILES); IPR001680 (PROSITE_PROFILES); IPR001680 (PROSITE_PROFILES); IPR017986 (SUPERFAMILY); IPR001810 (SUPERFAMILY)
1893	298250_length_1561_cvg_55.2_tip_1_3	2208	beta-hexosaminidase subunit beta isoform x1	521	0	68.80%	0.289	IPR025705 (PRINTS); IPR025705 (PIRSF); IPR029019 (PFAM); IPR015883 (PFAM); IPR029018 (G3DSA:3.30.379.GENE3D); IPR013781 (G3DSA:3.20.20.GENE3D); PTHR22600:SF7 (PANTHER); PTHR22600 (PANTHER); IPR017853 (SUPERFAMILY); IPR029018 (SUPERFAMILY)
1894	298278_length_1565_cvg_60.3_tip_1_4	3928	ferritin 1-like protein a	522	3.93E-45	65.00%	0.499 Y	IPR012347 (G3DSA:1.20.1260.GENE3D); IPR008331 (PFAM); IPR001519 (PANTHER); PTHR11431:SF29 (PANTHER); TRANSMEMBRANE (PHOBIUS); NON_CYTOPLASMIC_DOMAIN (PHOBIUS); CYTOPLASMIC_DOMAIN (PHOBIUS); IPR009040 (PROSITE_PROFILES); SignalP-noTM (SIGNALP_EUK); IPR009078 (SUPERFAMILY)

1895	298280_length_1566_cvg_11.8_tip_1_1	311	protocadherin fat 4	522	1.17E-68	48.20%	0.112	IPR002126 (PRINTS); IPR002126 (SMART); IPR002126 (G3DSA:2.60.40.GENE3D); IPR002126 (G3DSA:2.60.40.GENE3D); IPR002126 (PFAM); PTHR24027 (PANTHER); IPR020894 (PROSITE_PATTERNS); IPR002126 (PROSITE_PROFILES); IPR002126 (PROSITE_PROFILES); IPR002126 (PROSITE_PROFILES); IPR015919 (SUPERFAMILY); IPR015919 (SUPERFAMILY); IPR015919 (SUPERFAMILY); IPR015919 (SUPERFAMILY)
1896	298282_length_1566_cvg_20.9_tip_1_4	334	---NA---	522			0.109	Coil (COILS); Coil (COILS); TRANSMEMBRANE (PHOBIUS); TRANSMEMBRANE (PHOBIUS); CYTOPLASMIC_DOMAIN (PHOBIUS); CYTOPLASMIC_DOMAIN (PHOBIUS); TRANSMEMBRANE (PHOBIUS); NON_CYTOPLASMIC_DOMAIN (PHOBIUS); NON_CYTOPLASMIC_DOMAIN (PHOBIUS)
1897	298332_length_1573_cvg_19.2_tip_1_1	409	ddb1- and cul4-associated factor 13-like	524	0	80.80%	0.101	IPR001680 (SMART); IPR015943 (G3DSA:2.130.10.GENE3D); IPR007287 (PFAM); IPR001680 (PFAM); PTHR22851:SF0 (PANTHER); PTHR22851 (PANTHER); IPR019775 (PROSITE_PATTERNS); IPR017986 (PROSITE_PROFILES); IPR001680 (PROSITE_PROFILES); IPR001680 (PROSITE_PROFILES); IPR001680 (PROSITE_PROFILES); IPR001680 (PROSITE_PROFILES); IPR017986 (SUPERFAMILY)
1898	298336_length_1573_cvg_34.2_tip_1_4	664	cathepsin l	524	4.40E-167	77.20%	0.116	IPR000668 (PRINTS); IPR013201 (SMART); IPR000668 (SMART); IPR000668 (PFAM); G3DSA:3.90.70.10 (GENE3D); IPR013201 (PFAM); PTHR12411:SF57 (PANTHER); IPR013128 (PANTHER); IPR025661 (PROSITE_PATTERNS); IPR025660 (PROSITE_PATTERNS); IPR000169 (PROSITE_PATTERNS); NON_CYTOPLASMIC_DOMAIN (PHOBIUS); CYTOPLASMIC_DOMAIN (PHOBIUS); TRANSMEMBRANE (PHOBIUS); SSF54001 (SUPERFAMILY); TMhelix (TMHMM)
1899	298338_length_1573_cvg_22.4_tip_1_2	508	plasminogen activator spa	524	6.97E-84	64.60%	0.486 Y	IPR001314 (PRINTS); IPR001254 (SMART); G3DSA:2.40.10.10 (GENE3D); IPR001254 (PFAM); G3DSA:2.40.10.10 (GENE3D); PTHR24256 (PANTHER); IPR018114 (PROSITE_PATTERNS); IPR018114 (PROSITE_PATTERNS); SIGNAL_PEPTIDE_C_REGION (PHOBIUS); SIGNAL_PEPTIDE (PHOBIUS); SIGNAL_PEPTIDE_H_REGION (PHOBIUS); SIGNAL_PEPTIDE_N_REGION (PHOBIUS); NON_CYTOPLASMIC_DOMAIN (PHOBIUS); IPR001254 (PROSITE_PROFILES); IPR009003 (SUPERFAMILY)
1900	298346_length_1575_cvg_8.0_tip_1_4	189	otopetrin-2-like isoform x3	525	2.40E-123	64.70%	0.102	IPR004878 (PFAM); IPR004878 (PANTHER); PTHR21522:SF41 (PANTHER); CYTOPLASMIC_DOMAIN (PHOBIUS); TRANSMEMBRANE (PHOBIUS); TRANSMEMBRANE (PHOBIUS); NON_CYTOPLASMIC_DOMAIN (PHOBIUS); NON_CYTOPLASMIC_DOMAIN (PHOBIUS); TRANSMEMBRANE (PHOBIUS); NON_CYTOPLASMIC_DOMAIN (PHOBIUS); CYTOPLASMIC_DOMAIN (PHOBIUS); TRANSMEMBRANE (PHOBIUS); TRANSMEMBRANE (PHOBIUS); NON_CYTOPLASMIC_DOMAIN (PHOBIUS); TRANSMEMBRANE (PHOBIUS); CYTOPLASMIC_DOMAIN (PHOBIUS); TRANSMEMBRANE (PHOBIUS); CYTOPLASMIC_DOMAIN (PHOBIUS); TRANSMEMBRANE (PHOBIUS); CYTOPLASMIC_DOMAIN (PHOBIUS); TMhelix (TMHMM); TMhelix (TMHMM); TMhelix (TMHMM); TMhelix (TMHMM); TMhelix (TMHMM); TMhelix (TMHMM); TMhelix (TMHMM); TMhelix (TMHMM)
1903	298356_length_1577_cvg_16.3_tip_1_2	380	hypothetical protein L798_00519	525	5.58E-71	50.80%	0.392 Y	IPR008930 (G3DSA:1.50.10.GENE3D); IPR002157 (PFAM); IPR002157 (PANTHER); PTHR10559:SF12 (PANTHER); SIGNAL_PEPTIDE_H_REGION (PHOBIUS); SIGNAL_PEPTIDE_N_REGION (PHOBIUS); SIGNAL_PEPTIDE_C_REGION (PHOBIUS); NON_CYTOPLASMIC_DOMAIN (PHOBIUS); SIGNAL_PEPTIDE (PHOBIUS); IPR008930 (SUPERFAMILY); TMhelix (TMHMM)
1901	298350_length_1576_cvg_7.0_tip_1_0	136	sortilin-related receptor-like	526	4.74E-11	44.00%	0.121	IPR002172 (PRINTS); IPR002172 (SMART); IPR002172 (G3DSA:4.10.400.GENE3D); IPR002172 (G3DSA:4.10.400.GENE3D); IPR000998 (PFAM); IPR002172 (PFAM); PTHR10529 (PANTHER); PTHR10529:SF222 (PANTHER); IPR002172 (PROSITE_PROFILES); IPR002172 (PROSITE_PROFILES); IPR000998 (PROSITE_PROFILES); IPR002172 (SUPERFAMILY); IPR002172 (SUPERFAMILY); IPR013320 (SUPERFAMILY)
1902	298354_length_1577_cvg_64.6_tip_0_0	2631	prophenoloxidase activating factor	526	2.85E-26	53.80%	0.604 Y	IPR001254 (PFAM); G3DSA:2.40.10.10 (GENE3D); G3DSA:2.40.10.10 (GENE3D); PTHR24268:SF95 (PANTHER); PTHR24268 (PANTHER); IPR018114 (PROSITE_PATTERNS); SIGNAL_PEPTIDE_H_REGION (PHOBIUS); NON_CYTOPLASMIC_DOMAIN (PHOBIUS); SIGNAL_PEPTIDE_C_REGION (PHOBIUS); SIGNAL_PEPTIDE (PHOBIUS); SIGNAL_PEPTIDE_N_REGION (PHOBIUS); SignalP-noTM (SIGNALP_EUK); IPR009003 (SUPERFAMILY); TMhelix (TMHMM)

1904	298362_length_1577_cvg_13.7_tip_1_3	293	aael014742- partial	526	1.98E-86	56.70%	0.138	IPR002110 (PRINTS); IPR002110 (SMART); IPR020683 (G3DSA:1.25.40.GENE3D); IPR020683 (G3DSA:1.25.40.GENE3D); IPR020683 (G3DSA:1.25.40.GENE3D); IPR020683 (PFAM); IPR020683 (G3DSA:1.25.40.GENE3D); IPR002110 (PFAM); IPR020683 (G3DSA:1.25.40.GENE3D); PTHR24133 (PANTHER); PTHR24133:SF17 (PANTHER); IPR002110 (PROSITE_PROFILES); IPR002110 (PROSITE_PROFILES); IPR002110 (PROSITE_PROFILES); IPR002110 (PROSITE_PROFILES); IPR002110 (PROSITE_PROFILES); IPR002110 (PROSITE_PROFILES); IPR002110 (PROSITE_PROFILES); IPR020683 (PROSITE_PROFILES); IPR002110 (PROSITE_PROFILES); IPR002110 (PROSITE_PROFILES); IPR002110 (PROSITE_PROFILES); IPR002110 (PROSITE_PROFILES); IPR002110 (PROSITE_PROFILES); IPR020683 (SUPERFAMILY); IPR020683 (SUPERFAMILY)
1905	298446_length_1589_cvg_18.9_tip_1_0	419	low quality protein: microtubule-associated protein 1a	530	3.19E-08	54.00%	0.106	no IPS match
1906	298450_length_1590_cvg_59.0_tip_1_3	5642	low affinity immunoglobulin epsilon fc receptor	530	1.07E-18	53.80%	0.113	IPR001304 (SMART); IPR001304 (PFAM); IPR016186 (G3DSA:3.10.100.GENE3D); PTHR22802 (PANTHER); IPR018378 (PROSITE_PATTERNS); NON_CYTOPLASMIC_DOMAIN (PHOBIUS); TRANSMEMBRANE (PHOBIUS); CYTOPLASMIC_DOMAIN (PHOBIUS); IPR001304 (PROSITE_PROFILES); IPR016187 (SUPERFAMILY); TMhelix (TMHMM)
1907	298452_length_1590_cvg_13.4_tip_1_5	309	flavin-containing monooxygenase fmo gs-ox3	530	2.14E-144	64.80%	0.177	IPR000960 (PRINTS); G3DSA:3.50.50.60 (GENE3D); G3DSA:3.50.50.60 (GENE3D); IPR020946 (PFAM); PTHR23023:SF4 (PANTHER); PTHR23023 (PANTHER); SSF51905 (SUPERFAMILY); SSF51905 (SUPERFAMILY)
1908	298468_length_1593_cvg_9.6_tip_1_4	219	alkaline tissue-nonspecific isozyme-like	531	1.80E-147	63.80%	0.141	IPR001952 (PRINTS); IPR001952 (SMART); IPR001952 (PFAM); IPR017849 (G3DSA:3.40.720.GENE3D); PTHR11596 (PANTHER); IPR018299 (PROSITE_PATTERNS); IPR017850 (SUPERFAMILY)
1909	298482_length_1594_cvg_5.2_tip_1_2	100	mam and ldl-receptor class a domain-containing protein c10orf112-like	531	4.66E-85	49.80%	0.097	IPR002172 (PRINTS); IPR002172 (SMART); IPR000998 (SMART); IPR002172 (G3DSA:4.10.400.GENE3D); IPR000998 (PFAM); IPR002172 (PFAM); IPR002172 (G3DSA:4.10.400.GENE3D); PTHR23282:SF75 (PANTHER); PTHR23282 (PANTHER); IPR023415 (PROSITE_PATTERNS); IPR002172 (PROSITE_PROFILES); IPR000998 (PROSITE_PROFILES); IPR000998 (PROSITE_PROFILES); IPR002172 (PROSITE_PROFILES); IPR000998 (PROSITE_PROFILES); IPR002172 (SUPERFAMILY); IPR013320 (SUPERFAMILY); IPR013320 (SUPERFAMILY); IPR002172 (SUPERFAMILY); IPR013320 (SUPERFAMILY)
1910	298520_length_1600_cvg_23.2_tip_1_5	491	aael014742- partial	533	2.40E-83	55.80%	0.127	IPR002110 (PRINTS); IPR002110 (SMART); IPR002110 (PFAM); IPR020683 (G3DSA:1.25.40.GENE3D); IPR020683 (G3DSA:1.25.40.GENE3D); IPR020683 (PFAM); PTHR24133:SF17 (PANTHER); PTHR24133 (PANTHER); IPR002110 (PROSITE_PROFILES); IPR002110 (PROSITE_PROFILES); IPR002110 (PROSITE_PROFILES); IPR002110 (PROSITE_PROFILES); IPR002110 (PROSITE_PROFILES); IPR002110 (PROSITE_PROFILES); IPR002110 (PROSITE_PROFILES); IPR002110 (PROSITE_PROFILES); IPR002110 (PROSITE_PROFILES); IPR002110 (PROSITE_PROFILES); IPR020683 (PROSITE_PROFILES); IPR002110 (PROSITE_PROFILES); IPR002110 (PROSITE_PROFILES); IPR020683 (SUPERFAMILY); IPR020683 (SUPERFAMILY)
1911	298522_length_1600_cvg_2.3_tip_1_1	67	endoplasmic reticulum mannosyl-oligosaccharide -alpha-mannosidase	533	0	80.70%	0.107	IPR001382 (PRINTS); IPR001382 (G3DSA:1.50.10.GENE3D); IPR001382 (PFAM); PTHR11742:SF7 (PANTHER); IPR001382 (PANTHER); IPR001382 (SUPERFAMILY)
1912	298540_length_1602_cvg_62.4_tip_1_4	3235	phosphoglycerate kinase	534	0	87.40%	0.258	IPR001576 (PRINTS); IPR015824 (G3DSA:3.40.50.GENE3D); IPR015901 (G3DSA:3.40.50.GENE3D); IPR001576 (PFAM); IPR001576 (PANTHER); PTHR11406:SF6 (PANTHER); IPR015911 (PROSITE_PATTERNS); IPR001576 (HAMAP); IPR001576 (SUPERFAMILY)
1913	298546_length_1603_cvg_5.8_tip_1_5	148	15-hydroxyprostaglandin dehydrogenase	534	1.14E-50	60.10%	0.201	IPR002347 (PRINTS); IPR002198 (PRINTS); IPR016040 (G3DSA:3.40.50.GENE3D); IPR002198 (PFAM); PTHR24310 (PANTHER); IPR020904 (PROSITE_PATTERNS); CYTOPLASMIC_DOMAIN (PHOBIUS); TRANSMEMBRANE (PHOBIUS); NON_CYTOPLASMIC_DOMAIN (PHOBIUS); TRANSMEMBRANE (PHOBIUS); NON_CYTOPLASMIC_DOMAIN (PHOBIUS); SSF51735 (SUPERFAMILY)

1914	298548_length_1604_cvg_41.9_tip_1_2	1238	secreted salivary gland	534	2.03E-31	49.50%	0.470 Y	IPR004991 (PFAM); IPR023307 (G3DSA:2.170.15.GENE3D); TRANSMEMBRANE (PHOBIUS); SIGNAL_PEPTIDE_H_REGION (PHOBIUS); SIGNAL_PEPTIDE (PHOBIUS); SIGNAL_PEPTIDE_N_REGION (PHOBIUS); TRANSMEMBRANE (PHOBIUS); NON_CYTOPLASMIC_DOMAIN (PHOBIUS); NON_CYTOPLASMIC_DOMAIN (PHOBIUS); CYTOPLASMIC_DOMAIN (PHOBIUS); SIGNAL_PEPTIDE_C_REGION (PHOBIUS); SignalP-noTM (SIGNALP_EUK); SSF56973 (SUPERFAMILY); TMhelix (TMHMM); TMhelix (TMHMM)
1915	298576_length_1610_cvg_10.5_tip_1_3	239	venom metalloproteinase 3 isoform x3	537	5.27E-97	61.80%	0.114	IPR024079 (G3DSA:3.40.390.GENE3D); PTHR13723 (PANTHER); PTHR13723:SF148 (PANTHER); SSF55486 (SUPERFAMILY)
1916	298578_length_1610_cvg_30.9_tip_1_1	560	myosin heavy chain	537	2.03E-12	47.33%	0.118	Coil (COILS); Coil (COILS)
1917	298584_length_1611_cvg_4.0_tip_1_4	95	urokinase-type plasminogen activator	537	1.90E-64	56.20%	0.129	IPR001314 (PRINTS); IPR002172 (SMART); IPR001254 (SMART); G3DSA:2.40.10.10 (GENE3D); IPR002172 (G3DSA:4.10.400.GENE3D); IPR002172 (PFAM); G3DSA:2.40.10.10 (GENE3D); IPR001254 (PFAM); G3DSA:2.40.10.10 (GENE3D); PTHR24265 (PANTHER); IPR018114 (PROSITE_PATTERNS); IPR018114 (PROSITE_PATTERNS); IPR001254 (PROSITE_PROFILES); IPR002172 (PROSITE_PROFILES); IPR009003 (SUPERFAMILY); IPR002172 (SUPERFAMILY)
1918	298608_length_1613_cvg_9.5_tip_1_1	192	cgmp-dependent protein kinase 1-like	538	0	83.10%	0.161	IPR002374 (PRINTS); IPR000595 (SMART); IPR002290 (SMART); IPR000961 (SMART); IPR000595 (PFAM); IPR000719 (PFAM); IPR014710 (G3DSA:2.60.120.GENE3D); G3DSA:3.30.200.20 (GENE3D); IPR014710 (G3DSA:2.60.120.GENE3D); G3DSA:1.10.510.10 (GENE3D); PTHR24353 (PANTHER); PTHR24353:SF33 (PANTHER); IPR017441 (PROSITE_PATTERNS); IPR018488 (PROSITE_PATTERNS); IPR008271 (PROSITE_PATTERNS); IPR018488 (PROSITE_PATTERNS); IPR000595 (PROSITE_PROFILES); IPR000595 (PROSITE_PROFILES); IPR000961 (PROSITE_PROFILES); IPR000719 (PROSITE_PROFILES); IPR018490 (SUPERFAMILY); IPR011009 (SUPERFAMILY); IPR018490 (SUPERFAMILY)
1919	298618_length_1615_cvg_7.4_tip_1_5	150	thioredoxin-related transmembrane protein 1-like	538	9.90E-98	71.30%	0.684 Y	IPR013766 (PFAM); IPR012336 (G3DSA:3.40.30.GENE3D); PTHR18929:SF81 (PANTHER); PTHR18929 (PANTHER); IPR017937 (PROSITE_PATTERNS); TRANSMEMBRANE (PHOBIUS); NON_CYTOPLASMIC_DOMAIN (PHOBIUS); SIGNAL_PEPTIDE (PHOBIUS); SIGNAL_PEPTIDE_N_REGION (PHOBIUS); SIGNAL_PEPTIDE_C_REGION (PHOBIUS); CYTOPLASMIC_DOMAIN (PHOBIUS); SIGNAL_PEPTIDE_H_REGION (PHOBIUS); IPR012336 (PROSITE_PROFILES); SignalP-noTM (SIGNALP_EUK); IPR012336 (SUPERFAMILY); TMhelix (TMHMM)
1920	298634_length_1616_cvg_5.3_tip_1_0	103	papilin isoform x2	539	7.98E-106	63.20%	0.143	IPR003599 (SMART); IPR003598 (SMART); IPR013783 (G3DSA:2.60.40.GENE3D); IPR013098 (PFAM); IPR013783 (G3DSA:2.60.40.GENE3D); IPR010909 (PFAM); PTHR10489 (PANTHER); CYTOPLASMIC_DOMAIN (PHOBIUS); CYTOPLASMIC_DOMAIN (PHOBIUS); TRANSMEMBRANE (PHOBIUS); NON_CYTOPLASMIC_DOMAIN (PHOBIUS); TRANSMEMBRANE (PHOBIUS); NON_CYTOPLASMIC_DOMAIN (PHOBIUS); TRANSMEMBRANE (PHOBIUS); IPR007110 (PROSITE_PROFILES); IPR010909 (PROSITE_PROFILES); IPR007110 (PROSITE_PROFILES); SSF48726 (SUPERFAMILY); SSF48726 (SUPERFAMILY); TMhelix (TMHMM); TMhelix (TMHMM)
1921	298636_length_1616_cvg_47.0_tip_1_1	1586	d-aspartate oxidase	539	1.91E-98	63.60%	0.13	IPR016040 (G3DSA:3.40.50.GENE3D); IPR006076 (PFAM); IPR023209 (PANTHER); PTHR11530:SF0 (PANTHER); IPR006181 (PROSITE_PATTERNS); SSF51971 (SUPERFAMILY); SSF54373 (SUPERFAMILY)
1922	298642_length_1618_cvg_38.1_tip_1_2	940	ras-related protein rab-10	539	1.71E-116	92.90%	0.207	IPR001806 (PRINTS); IPR024156 (SMART); IPR003579 (SMART); IPR002041 (SMART); IPR020849 (SMART); IPR003578 (SMART); IPR005225 (TIGRFAM); IPR001806 (PFAM); IPR027417 (G3DSA:3.40.50.GENE3D); PTHR24073 (PANTHER); PTHR24073:SF456 (PANTHER); NON_CYTOPLASMIC_DOMAIN (PHOBIUS); CYTOPLASMIC_DOMAIN (PHOBIUS); TRANSMEMBRANE (PHOBIUS); PS51419 (PROSITE_PROFILES); IPR027417 (SUPERFAMILY)
1923	298658_length_1619_cvg_13.6_tip_1_4	251	n-acetylglucosamine-6- partial	540	9.75E-96	55.90%	0.105	IPR017849 (G3DSA:3.40.720.GENE3D); IPR000917 (PFAM); PTHR10342:SF208 (PANTHER); PTHR10342 (PANTHER); IPR017850 (SUPERFAMILY)
1924	298666_length_1620_cvg_7.6_tip_1_0	152	periodic tryptophan protein 1 homolog	540	1.81E-151	64.20%	0.123	IPR020472 (PRINTS); IPR001680 (SMART); IPR001680 (PFAM); IPR015943 (G3DSA:2.130.10.GENE3D); PTHR14091 (PANTHER); IPR019775 (PROSITE_PATTERNS); IPR019775 (PROSITE_PATTERNS); IPR001680 (PROSITE_PROFILES); IPR001680 (PROSITE_PROFILES); IPR017986 (PROSITE_PROFILES); IPR001680 (PROSITE_PROFILES); IPR017986 (SUPERFAMILY)

1925	298680_length_1623_cvg_21.1_tip_1_5	516	fasciclin-3 isoform x2	541	9.74E-41	48.00%	0.107	IPR003599 (SMART); IPR013783 (G3DSA:2.60.40.GENE3D); IPR013783 (G3DSA:2.60.40.GENE3D); IPR013162 (PFAM); PF13895 (PFAM); PTHR11640 (PANTHER); PTHR11640:SF52 (PANTHER); CYTOPLASMIC_DOMAIN (PHOBIUS); NON_CYTOPLASMIC_DOMAIN (PHOBIUS); TRANSMEMBRANE (PHOBIUS); IPR007110 (PROSITE_PROFILES); IPR007110 (PROSITE_PROFILES); SSF48726 (SUPERFAMILY); SSF48726 (SUPERFAMILY); TMhelix (TMHMM)
1926	298690_length_1624_cvg_63.0_tip_1_2	5539	spore wall protein 2-like	541	1.36E-50	41.70%	0.104	no IPS match
1927	298706_length_1625_cvg_12.0_tip_1_5	286	plasminogen-like isoform 2	541	3.23E-100	52.90%	0.179	PR00018 (PRINTS); IPR000001 (SMART); G3DSA:2.40.20.10 (GENE3D); IPR000001 (PFAM); PTHR24259 (PANTHER); IPR018056 (PROSITE_PATTERNS); IPR018056 (PROSITE_PATTERNS); IPR018056 (PROSITE_PATTERNS); IPR018056 (PROSITE_PATTERNS); IPR000001 (PROSITE_PROFILES); IPR000001 (PROSITE_PROFILES); IPR000001 (PROSITE_PROFILES); IPR000001 (PROSITE_PROFILES); IPR013806 (SUPERFAMILY); IPR013806 (SUPERFAMILY); IPR013806 (SUPERFAMILY); IPR013806 (SUPERFAMILY)
1928	298710_length_1627_cvg_4.8_tip_1_1	103	pre-mrna-processing factor 19	542	0	91.00%	0.103	IPR020472 (PRINTS); IPR001680 (SMART); IPR003613 (SMART); IPR013915 (PFAM); IPR013083 (G3DSA:3.30.40.GENE3D); IPR001680 (PFAM); IPR015943 (G3DSA:2.130.10.GENE3D); IPR003613 (PFAM); PTHR13889 (PANTHER); IPR019775 (PROSITE_PATTERNS); IPR019775 (PROSITE_PATTERNS); IPR001680 (PROSITE_PROFILES); IPR001680 (PROSITE_PROFILES); IPR003613 (PROSITE_PROFILES); IPR001680 (PROSITE_PROFILES); IPR017986 (PROSITE_PROFILES); SSF57850 (SUPERFAMILY); IPR017986 (SUPERFAMILY)
1929	298712_length_1627_cvg_6.6_tip_1_0	223	di-n-acetylchitobiase	543	2.41E-114	63.10%	0.881 Y	IPR011583 (SMART); IPR001223 (PFAM); IPR013781 (G3DSA:3.20.20.GENE3D); PTHR11177 (PANTHER); PTHR11177:SF146 (PANTHER); SIGNAL_PEPTIDE (PHOBIUS); SIGNAL_PEPTIDE_C_REGION (PHOBIUS); NON_CYTOPLASMIC_DOMAIN (PHOBIUS); SIGNAL_PEPTIDE_N_REGION (PHOBIUS); SIGNAL_PEPTIDE_H_REGION (PHOBIUS); SignalP-noTM (SIGNALP_EUK); IPR017853 (SUPERFAMILY); TMhelix (TMHMM)
1930	298726_length_1629_cvg_10.2_tip_1_5	244	hypothetical protein LOTGIDRAFT_228404	543	2.51E-07	50.50%	0.267	IPR000998 (SMART); IPR000998 (PFAM); PTHR23282:SF75 (PANTHER); PTHR23282 (PANTHER); IPR000998 (PROSITE_PROFILES); IPR013320 (SUPERFAMILY)
1931	298740_length_1631_cvg_6.4_tip_1_4	167	aldehyde dimeric nadp-preferring isoform x2	544	0	74.50%	0.103	IPR016163 (G3DSA:3.40.309.GENE3D); IPR016162 (G3DSA:3.40.605.GENE3D); IPR015590 (PFAM); IPR012394 (PIRSF); PTHR11699 (PANTHER); PTHR11699:SF15 (PANTHER); IPR029510 (PROSITE_PATTERNS); NON_CYTOPLASMIC_DOMAIN (PHOBIUS); CYTOPLASMIC_DOMAIN (PHOBIUS); TRANSMEMBRANE (PHOBIUS); IPR016161 (SUPERFAMILY); TMhelix (TMHMM)
1932	298760_length_1633_cvg_36.1_tip_1_0	784	beta-galactosidase-1-like protein 2	545	6.16E-124	57.90%	0.144	IPR001944 (PRINTS); IPR013781 (G3DSA:3.20.20.GENE3D); IPR001944 (PFAM); IPR008979 (G3DSA:2.60.120.GENE3D); IPR001944 (PANTHER); PTHR23421:SF66 (PANTHER); IPR017853 (SUPERFAMILY); IPR008979 (SUPERFAMILY)
1933	298786_length_1638_cvg_55.6_tip_1_3	1277	---NA---	546			0.121	no IPS match
1934	298794_length_1640_cvg_5.9_tip_1_0	141	zinc finger protein 287-like	547	6.76E-73	56.10%	0.104	IPR015880 (SMART); PF13912 (PFAM); IPR013087 (G3DSA:3.30.160.GENE3D); IPR013087 (G3DSA:3.30.160.GENE3D); PF13465 (PFAM); IPR013087 (G3DSA:3.30.160.GENE3D); PTHR24409 (PANTHER); IPR007087 (PROSITE_PATTERNS); IPR007087 (PROSITE_PATTERNS); IPR007087 (PROSITE_PATTERNS); IPR007087 (PROSITE_PATTERNS); IPR007087 (PROSITE_PATTERNS); IPR007087 (PROSITE_PATTERNS); IPR007087 (PROSITE_PATTERNS); IPR007087 (PROSITE_PATTERNS); IPR007087 (PROSITE_PATTERNS); IPR007087 (PROSITE_PROFILES); IPR007087 (PROSITE_PROFILES); IPR007087 (PROSITE_PROFILES); IPR007087 (PROSITE_PROFILES); IPR007087 (PROSITE_PROFILES); IPR007087 (PROSITE_PROFILES); IPR007087 (PROSITE_PROFILES); IPR007087 (PROSITE_PROFILES); IPR007087 (PROSITE_PROFILES); SSF57667 (SUPERFAMILY); SSF57667 (SUPERFAMILY); SSF57667 (SUPERFAMILY); SSF57667 (SUPERFAMILY); SSF57667 (SUPERFAMILY)
1935	298834_length_1648_cvg_17.0_tip_1_2	411	purple acid phosphatase	549	0	73.70%	0.543 Y	IPR025733 (PFAM); IPR004843 (PFAM); IPR029052 (G3DSA:3.60.21.GENE3D); IPR015914 (G3DSA:2.60.40.GENE3D); PTHR22953:SF9 (PANTHER); PTHR22953 (PANTHER); SignalP-TM (SIGNALP_EUK); IPR008963 (SUPERFAMILY); IPR029052 (SUPERFAMILY)

1936	298838_length_1648_cvg_24.4_tip_1_2	918	macro domain-containing protein 2	549	2.25E-83	69.40%	0.102	Coil (COILS); IPR002589 (SMART); IPR002589 (PFAM); G3DSA:3.40.220.10 (GENE3D); PTHR11106 (PANTHER); CYTOPLASMIC_DOMAIN (PHOBIUS); TRANSMEMBRANE (PHOBIUS); TRANSMEMBRANE (PHOBIUS); NON_CYTOPLASMIC_DOMAIN (PHOBIUS); CYTOPLASMIC_DOMAIN (PHOBIUS); IPR002589 (PROSITE_PROFILES); SSF52949 (SUPERFAMILY); TMhelix (TMHMM)
1937	298840_length_1649_cvg_7.9_tip_1_0	196	bone morphogenetic protein 2-like	550	7.89E-18	51.90%	0.137	IPR001839 (SMART); IPR029034 (G3DSA:2.10.90.GENE3D); IPR001839 (PFAM); PTHR11848:SF126 (PANTHER); IPR015615 (PANTHER); IPR017948 (PROSITE_PATTERNS); IPR001839 (PROSITE_PROFILES); IPR029034 (SUPERFAMILY)
1938	298846_length_1650_cvg_42.2_tip_1_0	871	cadherin-89d isoform x1	550	3.37E-14	44.90%	0.136	IPR002126 (PRINTS); IPR002126 (SMART); IPR002126 (G3DSA:2.60.40.GENE3D); IPR002126 (PFAM); IPR002126 (G3DSA:2.60.40.GENE3D); PTHR24027 (PANTHER); CYTOPLASMIC_DOMAIN (PHOBIUS); TRANSMEMBRANE (PHOBIUS); TRANSMEMBRANE (PHOBIUS); TRANSMEMBRANE (PHOBIUS); NON_CYTOPLASMIC_DOMAIN (PHOBIUS); NON_CYTOPLASMIC_DOMAIN (PHOBIUS); CYTOPLASMIC_DOMAIN (PHOBIUS); IPR002126 (PROSITE_PROFILES); IPR015919 (SUPERFAMILY); IPR015919 (SUPERFAMILY); TMhelix (TMHMM); TMhelix (TMHMM); TMhelix (TMHMM)
1939	298864_length_1653_cvg_7.6_tip_1_1	186	vitellogenin receptor	551	1.24E-52	47.50%	0.107	IPR002172 (PRINTS); IPR000033 (SMART); IPR002172 (SMART); G3DSA:2.10.25.10 (GENE3D); IPR011042 (G3DSA:2.120.10.GENE3D); PF14670 (PFAM); IPR002172 (G3DSA:4.10.400.GENE3D); IPR002172 (PFAM); PTHR10529 (PANTHER); PTHR10529:SF204 (PANTHER); IPR023415 (PROSITE_PATTERNS); IPR023415 (PROSITE_PATTERNS); CYTOPLASMIC_DOMAIN (PHOBIUS); TRANSMEMBRANE (PHOBIUS); NON_CYTOPLASMIC_DOMAIN (PHOBIUS); IPR002172 (PROSITE_PROFILES); IPR002172 (PROSITE_PROFILES); IPR000033 (PROSITE_PROFILES); IPR000033 (PROSITE_PROFILES); IPR000033 (PROSITE_PROFILES); IPR000033 (PROSITE_PROFILES); IPR002172 (PROSITE_PROFILES); SSF57196 (SUPERFAMILY); IPR002172 (SUPERFAMILY); SSF63825 (SUPERFAMILY); IPR002172 (SUPERFAMILY); IPR002172 (SUPERFAMILY); TMhelix (TMHMM)
1940	298888_length_1659_cvg_7.3_tip_1_4	156	macrophage mannose receptor 1	553	1.17E-20	36.00%	0.107	IPR001304 (SMART); IPR001304 (PFAM); IPR016186 (G3DSA:3.10.100.GENE3D); IPR016186 (G3DSA:3.10.100.GENE3D); PTHR22803:SF55 (PANTHER); PTHR22803 (PANTHER); IPR001304 (PROSITE_PROFILES); IPR001304 (PROSITE_PROFILES); IPR001304 (PROSITE_PROFILES); IPR016187 (SUPERFAMILY); IPR016187 (SUPERFAMILY); IPR016187 (SUPERFAMILY)
1941	298890_length_1659_cvg_55.9_tip_1_4	2753	retinal dehydrogenase 1-like	553	0	80.40%	0.442 Y	IPR015590 (PFAM); IPR016163 (G3DSA:3.40.309.GENE3D); IPR016162 (G3DSA:3.40.605.GENE3D); PTHR11699:SF145 (PANTHER); PTHR11699 (PANTHER); IPR029510 (PROSITE_PATTERNS); IPR016160 (PROSITE_PATTERNS); IPR016161 (SUPERFAMILY)
1942	298910_length_1663_cvg_9.8_tip_1_2	213	leucine-rich repeat and immunoglobulin-like domain-containing nogo receptor-interacting protein 3 isoform x2	554	3.07E-141	64.60%	0.127	Coil (COILS); IPR003599 (SMART); IPR003598 (SMART); IPR013783 (G3DSA:2.60.40.GENE3D); IPR013098 (PFAM); G3DSA:3.80.10.10 (GENE3D); PTHR24373:SF38 (PANTHER); PTHR24373 (PANTHER); CYTOPLASMIC_DOMAIN (PHOBIUS); TRANSMEMBRANE (PHOBIUS); NON_CYTOPLASMIC_DOMAIN (PHOBIUS); IPR007110 (PROSITE_PROFILES); SSF52058 (SUPERFAMILY); SSF48726 (SUPERFAMILY); TMhelix (TMHMM)
1943	298912_length_1665_cvg_5.6_tip_1_0	163	cysteine-rich motor neuron 1 protein isoform x1	555	2.90E-59	51.70%	0.13	IPR002223 (PRINTS); IPR002223 (SMART); IPR000716 (SMART); IPR002223 (G3DSA:4.10.410.GENE3D); IPR002223 (PFAM); IPR000716 (PFAM); IPR000716 (G3DSA:4.10.800.GENE3D); PTHR10083 (PANTHER); IPR020901 (PROSITE_PATTERNS); IPR000716 (PROSITE_PATTERNS); CYTOPLASMIC_DOMAIN (PHOBIUS); NON_CYTOPLASMIC_DOMAIN (PHOBIUS); TRANSMEMBRANE (PHOBIUS); IPR000716 (PROSITE_PROFILES); IPR002223 (PROSITE_PROFILES); IPR000716 (SUPERFAMILY); IPR002223 (SUPERFAMILY); TMhelix (TMHMM)

1944	298914_length_1665_cvg_54.3_tip_1_3	1896	leucine rich repeat protein	555	3.31E-29	50.70%	0.196	IPR003591 (SMART); SM00364 (SMART); G3DSA:3.80.10.10 (GENE3D); G3DSA:3.80.10.10 (GENE3D); G3DSA:3.80.10.10 (GENE3D); IPR001611 (PFAM); IPR001611 (PFAM); PTHR24365 (PANTHER); CYTOPLASMIC_DOMAIN (PHOBIUS); NON_CYTOPLASMIC_DOMAIN (PHOBIUS); TRANSMEMBRANE (PHOBIUS); IPR001611 (PROSITE_PROFILES); IPR001611 (PROSITE_PROFILES); IPR001611 (PROSITE_PROFILES); IPR001611 (PROSITE_PROFILES); IPR001611 (PROSITE_PROFILES); IPR001611 (PROSITE_PROFILES); IPR001611 (PROSITE_PROFILES); IPR001611 (PROSITE_PROFILES); IPR001611 (PROSITE_PROFILES); IPR001611 (PROSITE_PROFILES); IPR001611 (PROSITE_PROFILES); IPR001611 (PROSITE_PROFILES); IPR001611 (PROSITE_PROFILES); IPR001611 (PROSITE_PROFILES); SSF52058 (SUPERFAMILY)
1945	298950_length_1670_cvg_10.0_tip_1_5	183	polycomb protein eed	556	0	85.10%	0.122	IPR001680 (SMART); IPR001680 (PFAM); IPR015943 (G3DSA:2.130.10.GENE3D); PTHR10253 (PANTHER); IPR019775 (PROSITE_PATTERNS); IPR001680 (PROSITE_PROFILES); IPR017986 (PROSITE_PROFILES); IPR001680 (PROSITE_PROFILES); IPR017986 (SUPERFAMILY)
1946	298976_length_1674_cvg_23.1_tip_1_1	606	alpha-aminoadipic semialdehyde dehydrogenase	558	0	83.80%	0.109	IPR016163 (G3DSA:3.40.309.GENE3D); IPR016162 (G3DSA:3.40.605.GENE3D); IPR015590 (PFAM); PTHR11699:SF144 (PANTHER); PTHR11699 (PANTHER); IPR029510 (PROSITE_PATTERNS); IPR016161 (SUPERFAMILY)
1947	298982_length_1674_cvg_56.0_tip_1_4	2959	carboxypeptidase b-like	558	2.53E-123	62.60%	0.141	IPR000834 (PRINTS); IPR000834 (SMART); IPR003146 (PFAM); IPR000834 (PFAM); IPR003146 (G3DSA:3.30.70.GENE3D); G3DSA:3.40.630.10 (GENE3D); PTHR11705 (PANTHER); IPR000834 (PROSITE_PATTERNS); NON_CYTOPLASMIC_DOMAIN (PHOBIUS); NON_CYTOPLASMIC_DOMAIN (PHOBIUS); CYTOPLASMIC_DOMAIN (PHOBIUS); TRANSMEMBRANE (PHOBIUS); TRANSMEMBRANE (PHOBIUS); IPR009020 (SUPERFAMILY); SSF53187 (SUPERFAMILY)
1948	298990_length_1676_cvg_3.7_tip_1_0	83	urokinase-type plasminogen activator	559	2.55E-59	54.00%	0.1	IPR001314 (PRINTS); IPR002172 (SMART); IPR001254 (SMART); IPR001254 (PFAM); G3DSA:2.40.10.10 (GENE3D); IPR002172 (PFAM); IPR002172 (G3DSA:4.10.400.GENE3D); G3DSA:2.40.10.10 (GENE3D); PTHR24265 (PANTHER); IPR023415 (PROSITE_PATTERNS); IPR018114 (PROSITE_PATTERNS); IPR018114 (PROSITE_PATTERNS); NON_CYTOPLASMIC_DOMAIN (PHOBIUS); TRANSMEMBRANE (PHOBIUS); CYTOPLASMIC_DOMAIN (PHOBIUS); IPR001254 (PROSITE_PROFILES); IPR002172 (PROSITE_PROFILES); IPR002172 (SUPERFAMILY); IPR009003 (SUPERFAMILY)
1949	299002_length_1677_cvg_16.4_tip_1_5	363	serine protease easter-like	559	1.13E-71	54.80%	0.206	IPR001314 (PRINTS); IPR001254 (SMART); IPR006604 (SMART); G3DSA:2.40.10.10 (GENE3D); G3DSA:2.40.10.10 (GENE3D); IPR001254 (PFAM); IPR022700 (PFAM); G3DSA:2.40.10.10 (GENE3D); PTHR24260:SF3 (PANTHER); PTHR24260 (PANTHER); IPR018114 (PROSITE_PATTERNS); IPR018114 (PROSITE_PATTERNS); CYTOPLASMIC_DOMAIN (PHOBIUS); NON_CYTOPLASMIC_DOMAIN (PHOBIUS); CYTOPLASMIC_DOMAIN (PHOBIUS); NON_CYTOPLASMIC_DOMAIN (PHOBIUS); TRANSMEMBRANE (PHOBIUS); TRANSMEMBRANE (PHOBIUS); TRANSMEMBRANE (PHOBIUS); IPR001254 (PROSITE_PROFILES); IPR009003 (SUPERFAMILY); TMhelix (TMHMM); TMhelix (TMHMM); TMhelix (TMHMM)
1950	299016_length_1679_cvg_31.4_tip_1_2	584	calumenin	559	1.64E-155	80.10%	0.142	IPR002048 (SMART); IPR002048 (PFAM); IPR011992 (G3DSA:1.10.238.GENE3D); IPR011992 (PFAM); PTHR10827 (PANTHER); IPR027239 (PTHR10827:PANTHER); IPR018247 (PROSITE_PATTERNS); IPR018247 (PROSITE_PATTERNS); IPR018247 (PROSITE_PATTERNS); IPR018247 (PROSITE_PATTERNS); IPR018247 (PROSITE_PATTERNS); CYTOPLASMIC_DOMAIN (PHOBIUS); NON_CYTOPLASMIC_DOMAIN (PHOBIUS); TRANSMEMBRANE (PHOBIUS); NON_CYTOPLASMIC_DOMAIN (PHOBIUS); CYTOPLASMIC_DOMAIN (PHOBIUS); TRANSMEMBRANE (PHOBIUS); TRANSMEMBRANE (PHOBIUS); IPR002048 (PROSITE_PROFILES); IPR002048 (PROSITE_PROFILES); IPR002048 (PROSITE_PROFILES); IPR002048 (PROSITE_PROFILES); IPR002048 (PROSITE_PROFILES); IPR002048 (PROSITE_PROFILES); SSF47473 (SUPERFAMILY); SSF47473 (SUPERFAMILY); TMhelix (TMHMM); TMhelix (TMHMM); TMhelix (TMHMM)
1951	299022_length_1681_cvg_9.2_tip_1_2	243	ras-related protein rab-8a isoform x2	560	9.75E-134	95.90%	0.147	IPR001806 (PRINTS); IPR003579 (SMART); IPR020849 (SMART); IPR024156 (SMART); IPR002041 (SMART); IPR003578 (SMART); IPR027417 (G3DSA:3.40.50.GENE3D); IPR001806 (PFAM); IPR005225 (TIGRFAM); PTHR24073:SF22 (PANTHER); PTHR24073 (PANTHER); PS51419 (PROSITE_PROFILES); IPR027417 (SUPERFAMILY)

1952	299028_length_1682_cvg_62.0_tip_1_0	22625	hemocytin isoform x1	561	1.01E-20	42.80%	0.101	CYTOPLASMIC_DOMAIN (PHOBIUS); TRANSMEMBRANE (PHOBIUS); NON_CYTOPLASMIC_DOMAIN (PHOBIUS); IPR006207 (PROSITE_PROFILES); SignalP-TM (SIGNALP_GRAM_POSITIVE); TMhelix (TMHMM)
1953	299042_length_1686_cvg_13.2_tip_1_0	219	angiotensin-converting enzyme-like	562	1.16E-113	78.90%	0.144	IPR001548 (PRINTS); IPR001548 (PFAM); PTHR10514:SF17 (PANTHER); IPR001548 (PANTHER); TRANSMEMBRANE (PHOBIUS); CYTOPLASMIC_DOMAIN (PHOBIUS); TRANSMEMBRANE (PHOBIUS); TRANSMEMBRANE (PHOBIUS); CYTOPLASMIC_DOMAIN (PHOBIUS); CYTOPLASMIC_DOMAIN (PHOBIUS); TRANSMEMBRANE (PHOBIUS); NON_CYTOPLASMIC_DOMAIN (PHOBIUS); NON_CYTOPLASMIC_DOMAIN (PHOBIUS); SSF55486 (SUPERFAMILY); TMhelix (TMHMM); TMhelix (TMHMM); TMhelix (TMHMM); TMhelix (TMHMM); TMhelix (TMHMM)
1954	299064_length_1689_cvg_7.9_tip_1_4	174	chorion peroxidase-like	563	8.28E-126	55.40%	0.136	IPR019791 (PRINTS); IPR019791 (PFAM); IPR019791 (G3DSA:1.10.640.GENE3D); PTHR11475 (PANTHER); IPR019791 (PROSITE_PROFILES); IPR010255 (SUPERFAMILY)
1955	299076_length_1692_cvg_22.1_tip_1_1	517	multiple inositol polyphosphate phosphatase 1	564	5.02E-145	61.10%	0.152	IPR029033 (G3DSA:3.40.50.GENE3D); IPR000560 (PFAM); PTHR20963 (PANTHER); PTHR20963:SF8 (PANTHER); IPR000560 (PROSITE_PATTERNS); NON_CYTOPLASMIC_DOMAIN (PHOBIUS); NON_CYTOPLASMIC_DOMAIN (PHOBIUS); TRANSMEMBRANE (PHOBIUS); TRANSMEMBRANE (PHOBIUS); CYTOPLASMIC_DOMAIN (PHOBIUS); TRANSMEMBRANE (PHOBIUS); CYTOPLASMIC_DOMAIN (PHOBIUS); IPR029033 (SUPERFAMILY)
1956	299084_length_1693_cvg_24.8_tip_1_5	473	neutral and basic amino acid transport protein rbat	564	6.30E-144	57.90%	0.187	IPR006589 (SMART); IPR013781 (G3DSA:3.20.20.GENE3D); G3DSA:3.90.400.10 (GENE3D); IPR006047 (PFAM); PTHR10357:SF77 (PANTHER); IPR015902 (PANTHER); TRANSMEMBRANE (PHOBIUS); CYTOPLASMIC_DOMAIN (PHOBIUS); NON_CYTOPLASMIC_DOMAIN (PHOBIUS); IPR017853 (SUPERFAMILY); TMhelix (TMHMM)
1957	299088_length_1693_cvg_6.0_tip_1_4	143	tyrosine-protein kinase csk	564	0	85.70%	0.322	IPR000980 (PRINTS); IPR001245 (PRINTS); IPR000980 (SMART); IPR020635 (SMART); G3DSA:1.10.510.10 (GENE3D); G3DSA:3.30.200.20 (GENE3D); IPR000980 (PFAM); IPR000980 (G3DSA:3.30.505.GENE3D); IPR001245 (PFAM); PTHR24418:SF192 (PANTHER); PTHR24418 (PANTHER); IPR017441 (PROSITE_PATTERNS); IPR008266 (PROSITE_PATTERNS); IPR000719 (PROSITE_PROFILES); IPR000980 (PROSITE_PROFILES); IPR011009 (SUPERFAMILY); IPR000980 (SUPERFAMILY); TMhelix (TMHMM)
1958	299096_length_1697_cvg_20.9_tip_1_3	443	pdgf- and vegf-related factor 1-like precursor	566	7.23E-54	55.50%	0.328	IPR000072 (SMART); IPR000072 (PFAM); IPR029034 (G3DSA:2.10.90.GENE3D); PTHR11633:SF1 (PANTHER); PTHR11633 (PANTHER); SIGNAL_PEPTIDE (PHOBIUS); SIGNAL_PEPTIDE_H_REGION (PHOBIUS); SIGNAL_PEPTIDE_N_REGION (PHOBIUS); SIGNAL_PEPTIDE_C_REGION (PHOBIUS); NON_CYTOPLASMIC_DOMAIN (PHOBIUS); IPR000072 (PROSITE_PROFILES); IPR029034 (SUPERFAMILY)
1959	299104_length_1698_cvg_7.2_tip_1_1	182	annexin a4	566	6.31E-94	67.60%	0.113	IPR001464 (PRINTS); IPR018502 (SMART); IPR018502 (PFAM); IPR018502 (G3DSA:1.10.220.GENE3D); IPR018502 (G3DSA:1.10.220.GENE3D); IPR018502 (G3DSA:1.10.220.GENE3D); IPR018502 (G3DSA:1.10.220.GENE3D); PTHR10502 (PANTHER); IPR018252 (PROSITE_PATTERNS); IPR018252 (PROSITE_PATTERNS); IPR018252 (PROSITE_PATTERNS); SSF47874 (SUPERFAMILY)
1960	299122_length_1701_cvg_22.7_tip_1_1	390	nucleolar protein 56	567	0	87.50%	0.25	Coil (COILS); IPR012976 (SMART); IPR002687 (PFAM); IPR012976 (PFAM); IPR012974 (PFAM); PTHR10894 (PANTHER); PTHR10894:SF0 (PANTHER); IPR002687 (PROSITE_PROFILES); SSF89124 (SUPERFAMILY)
1962	299150_length_1708_cvg_3.3_tip_1_5	80	collagen alpha-1 chain	569	1.43E-117	63.90%	0.099	IPR008160 (PFAM); PTHR24023 (PANTHER)
1961	299150_length_1708_cvg_3.3_tip_1_0	80	threonine-rich gpi-anchored glycoprotein	570	5.45E-60	43.60%	0.114	PTHR24023 (PANTHER); PTHR24023:SF405 (PANTHER)
1963	299168_length_1713_cvg_14.9_tip_1_1	365	wd40 repeat-containing protein smu1	571	0	93.80%	0.099	IPR020472 (PRINTS); IPR001680 (SMART); IPR006594 (SMART); IPR006595 (SMART); IPR001680 (PFAM); IPR015943 (G3DSA:2.130.10.GENE3D); PTHR22848 (PANTHER); IPR019775 (PROSITE_PATTERNS); IPR019775 (PROSITE_PATTERNS); IPR001680 (PROSITE_PROFILES); IPR001680 (PROSITE_PROFILES); IPR001680 (PROSITE_PROFILES); IPR006595 (PROSITE_PROFILES); IPR017986 (PROSITE_PROFILES); IPR001680 (PROSITE_PROFILES); IPR006594 (PROSITE_PROFILES); IPR001680 (PROSITE_PROFILES); IPR017986 (SUPERFAMILY)
1964	299172_length_1713_cvg_11.4_tip_1_0	282	estradiol 17-beta-dehydrogenase 2	571	1.09E-71	56.80%	0.123	IPR002198 (PRINTS); IPR002347 (PRINTS); IPR002198 (PFAM); IPR016040 (G3DSA:3.40.50.GENE3D); PTHR24316 (PANTHER); PTHR24316:SF215 (PANTHER); CYTOPLASMIC_DOMAIN (PHOBIUS); TRANSMEMBRANE (PHOBIUS); CYTOPLASMIC_DOMAIN (PHOBIUS); NON_CYTOPLASMIC_DOMAIN (PHOBIUS); NON_CYTOPLASMIC_DOMAIN (PHOBIUS); TRANSMEMBRANE (PHOBIUS); TRANSMEMBRANE (PHOBIUS); SSF51735 (SUPERFAMILY); TMhelix (TMHMM); TMhelix (TMHMM)

1965	299190_length_1716_cvg_8.7_tip_1_4	178	endothelin-converting enzyme 1	572	8.76E-165	64.60%	0.104	IPR018497 (PRINTS); IPR024079 (G3DSA:3.40.390.GENE3D); IPR018497 (PFAM); IPR008753 (PFAM); IPR000718 (PANTHER); PTHR11733:SF111 (PANTHER); SSF55486 (SUPERFAMILY)
1966	299198_length_1719_cvg_10.0_tip_1_3	234	peptidyl-prolyl cis-trans isomerase fkbp4-like	573	0	73.80%	0.128	Coil (COILS); IPR019734 (SMART); G3DSA:3.10.50.40 (GENE3D); IPR023114 (G3DSA:1.10.150.GENE3D); IPR001440 (PFAM); G3DSA:3.10.50.40 (GENE3D); IPR011990 (G3DSA:1.25.40.GENE3D); PF13414 (PFAM); IPR001179 (PFAM); IPR023566 (PANTHER); PTHR10516:SF274 (PANTHER); IPR001179 (PROSITE_PROFILES); IPR013026 (PROSITE_PROFILES); IPR019734 (PROSITE_PROFILES); IPR019734 (PROSITE_PROFILES); IPR013026 (PROSITE_PROFILES); IPR019734 (PROSITE_PROFILES); IPR001179 (PROSITE_PROFILES); SSF54534 (SUPERFAMILY); SSF48452 (SUPERFAMILY); SSF54534 (SUPERFAMILY)
1967	299226_length_1723_cvg_77.9_tip_1_4	7659	inactive pancreatic lipase-related protein 1	574	1.35E-88	56.80%	0.133	IPR002331 (PRINTS); IPR000734 (PRINTS); IPR001024 (G3DSA:2.60.60.GENE3D); IPR013818 (PFAM); IPR029058 (G3DSA:3.40.50.GENE3D); IPR000734 (PANTHER); TRANSMEMBRANE (PHOBIUS); NON_CYTOPLASMIC_DOMAIN (PHOBIUS); CYTOPLASMIC_DOMAIN (PHOBIUS); IPR029058 (SUPERFAMILY); IPR008976 (SUPERFAMILY)
1968	299278_length_1736_cvg_14.3_tip_1_2	330	hydroxysteroid dehydrogenase-like protein 2	578	0	77.00%	0.14	IPR002347 (PRINTS); IPR016040 (G3DSA:3.40.50.GENE3D); IPR003033 (PFAM); IPR003033 (G3DSA:3.30.1050.GENE3D); IPR002198 (PFAM); PTHR24322 (PANTHER); PTHR24322:SF273 (PANTHER); IPR003033 (SUPERFAMILY); SSF51735 (SUPERFAMILY)
1969	299280_length_1736_cvg_8.9_tip_1_4	209	cytochrome p450 2u1-like	579	6.67E-124	57.40%	0.107	IPR002401 (PRINTS); IPR001128 (PRINTS); IPR001128 (G3DSA:1.10.630.GENE3D); IPR001128 (PFAM); PTHR24300 (PANTHER); IPR017972 (PROSITE_PATTERNS); NON_CYTOPLASMIC_DOMAIN (PHOBIUS); CYTOPLASMIC_DOMAIN (PHOBIUS); TRANSMEMBRANE (PHOBIUS); NON_CYTOPLASMIC_DOMAIN (PHOBIUS); TRANSMEMBRANE (PHOBIUS); TRANSMEMBRANE (PHOBIUS); CYTOPLASMIC_DOMAIN (PHOBIUS); IPR001128 (SUPERFAMILY); TMhelix (TMHMM); TMhelix (TMHMM); TMhelix (TMHMM)
1970	299284_length_1736_cvg_56.0_tip_1_4	1575	leucine-rich repeat neuronal protein 3-like isoform x1	579	3.06E-107	54.90%	0.194	IPR003591 (SMART); IPR000483 (SMART); G3DSA:3.80.10.10 (GENE3D); G3DSA:3.80.10.10 (GENE3D); IPR001611 (PFAM); IPR001611 (PFAM); PTHR24373 (PANTHER); TRANSMEMBRANE (PHOBIUS); TRANSMEMBRANE (PHOBIUS); CYTOPLASMIC_DOMAIN (PHOBIUS); NON_CYTOPLASMIC_DOMAIN (PHOBIUS); CYTOPLASMIC_DOMAIN (PHOBIUS); IPR001611 (PROSITE_PROFILES); IPR001611 (PROSITE_PROFILES); IPR001611 (PROSITE_PROFILES); IPR001611 (PROSITE_PROFILES); IPR001611 (PROSITE_PROFILES); IPR001611 (PROSITE_PROFILES); IPR001611 (PROSITE_PROFILES); IPR001611 (PROSITE_PROFILES); SSF52058 (SUPERFAMILY); TMhelix (TMHMM); TMhelix (TMHMM); TMhelix (TMHMM)
1971	299310_length_1741_cvg_61.7_tip_1_5	1740	heat shock protein 70	580	0	96.30%	0.144	Coil (COILS); IPR013126 (PRINTS); IPR029047 (G3DSA:2.60.34.GENE3D); G3DSA:3.30.30.30 (GENE3D); IPR013126 (PFAM); G3DSA:3.90.640.10 (GENE3D); G3DSA:3.30.420.40 (GENE3D); G3DSA:3.30.420.40 (GENE3D); PTHR19375 (PANTHER); PTHR19375:SF142 (PANTHER); IPR018181 (PROSITE_PATTERNS); IPR018181 (PROSITE_PATTERNS); IPR018181 (PROSITE_PATTERNS); SSF53067 (SUPERFAMILY); IPR029047 (SUPERFAMILY); SSF53067 (SUPERFAMILY)
1972	299338_length_1749_cvg_6.8_tip_1_5	139	ribonuclease oy	583	1.94E-61	59.30%	0.285	IPR001568 (G3DSA:3.90.730.GENE3D); IPR001568 (PFAM); IPR001568 (PANTHER); IPR018188 (PROSITE_PATTERNS); CYTOPLASMIC_DOMAIN (PHOBIUS); TRANSMEMBRANE (PHOBIUS); NON_CYTOPLASMIC_DOMAIN (PHOBIUS); IPR001568 (SUPERFAMILY); TMhelix (TMHMM); TMhelix (TMHMM)
1973	299348_length_1752_cvg_54.9_tip_1_0	2174	polyketide synthase type i	584	6.23E-27	61.90%	0.115	IPR000436 (SMART); IPR000436 (PFAM); G3DSA:2.10.70.10 (GENE3D); G3DSA:2.10.70.10 (GENE3D); IPR000436 (PROSITE_PROFILES); IPR000436 (PROSITE_PROFILES); IPR000436 (SUPERFAMILY); IPR000436 (SUPERFAMILY)
1974	299348_length_1752_cvg_54.9_tip_1_1	2174	protein containing tetratricopeptide repeat	584	4.26E-07	76.00%	0.186	Coil (COILS); Coil (COILS); TRANSMEMBRANE (PHOBIUS); NON_CYTOPLASMIC_DOMAIN (PHOBIUS); CYTOPLASMIC_DOMAIN (PHOBIUS)

1975	299348_length_1752_cvg_54.9_tip_1_4	2174	mucin-1 precursor (muc-1) (polymorphic epithelial mucin) (tumor-associated mucin) (carcinoma-associated mucin) (tumor-associated epithelial membrane antigen)	584	1.17E-14	54.40%	0.186	G3DSA:2.160.10.10 (GENE3D); NON_CYTOPLASMIC_DOMAIN (PHOBIUS); TRANSMEMBRANE (PHOBIUS); TRANSMEMBRANE (PHOBIUS); TRANSMEMBRANE (PHOBIUS); TRANSMEMBRANE (PHOBIUS); CYTOPLASMIC_DOMAIN (PHOBIUS); CYTOPLASMIC_DOMAIN (PHOBIUS); TRANSMEMBRANE (PHOBIUS); CYTOPLASMIC_DOMAIN (PHOBIUS); TRANSMEMBRANE (PHOBIUS); CYTOPLASMIC_DOMAIN (PHOBIUS); NON_CYTOPLASMIC_DOMAIN (PHOBIUS); CYTOPLASMIC_DOMAIN (PHOBIUS); NON_CYTOPLASMIC_DOMAIN (PHOBIUS); TRANSMEMBRANE (PHOBIUS); TRANSMEMBRANE (PHOBIUS); TRANSMEMBRANE (PHOBIUS); CYTOPLASMIC_DOMAIN (PHOBIUS); NON_CYTOPLASMIC_DOMAIN (PHOBIUS); CYTOPLASMIC_DOMAIN (PHOBIUS); NON_CYTOPLASMIC_DOMAIN (PHOBIUS); TRANSMEMBRANE (PHOBIUS); NON_CYTOPLASMIC_DOMAIN (PHOBIUS); TRANSMEMBRANE (PHOBIUS); TRANSMEMBRANE (PHOBIUS); IPR011004 (SUPERFAMILY); TMhelix (TMHMM); TMhelix (TMHMM); TMhelix (TMHMM); TMhelix (TMHMM); TMhelix (TMHMM); TMhelix (TMHMM); TMhelix (TMHMM); TMhelix (TMHMM); TMhelix (TMHMM); TMhelix (TMHMM); TMhelix (TMHMM); TMhelix (TMHMM)
1977	299368_length_1757_cvg_77.7_tip_1_5	10089	vitelline membrane outer layer protein 1 homolog	585	1.27E-38	55.30%	0.099	IPR000742 (SMART); IPR020864 (SMART); IPR005515 (PFAM); IPR023413 (G3DSA:2.40.155.GENE3D); IPR005515 (G3DSA:2.100.10.GENE3D); IPR020864 (PFAM); IPR005515 (PANTHER); IPR000152 (PROSITE_PATTERNS); IPR013032 (PROSITE_PATTERNS); IPR020864 (PROSITE_PROFILES); IPR000742 (PROSITE_PROFILES); SSF57196 (SUPERFAMILY); IPR005515 (SUPERFAMILY)
1976	299366_length_1757_cvg_10.8_tip_1_0	249	delta-1-pyrroline-5-carboxylate mitochondrial	586	0	80.30%	0.344 Y	IPR016162 (G3DSA:3.40.605.GENE3D); IPR016163 (G3DSA:3.40.309.GENE3D); IPR005931 (TIGRFAM); IPR015590 (PFAM); PTHR11699 (PANTHER); PTHR11699:SF18 (PANTHER); IPR016160 (PROSITE_PATTERNS); NON_CYTOPLASMIC_DOMAIN (PHOBIUS); SIGNAL_PEPTIDE (PHOBIUS); SIGNAL_PEPTIDE_N_REGION (PHOBIUS); SIGNAL_PEPTIDE_H_REGION (PHOBIUS); SIGNAL_PEPTIDE_C_REGION (PHOBIUS); IPR016161 (SUPERFAMILY)
1978	299370_length_1758_cvg_54.9_tip_1_1	3803	cathepsin b	586	5.14E-164	78.80%	0.121	IPR000668 (PRINTS); IPR000668 (SMART); IPR012599 (PFAM); IPR000668 (PFAM); G3DSA:3.90.70.10 (GENE3D); IPR015643 (PTHR12411:PANTHER); IPR013128 (PANTHER); IPR000169 (PROSITE_PATTERNS); IPR025660 (PROSITE_PATTERNS); IPR025661 (PROSITE_PATTERNS); CYTOPLASMIC_DOMAIN (PHOBIUS); TRANSMEMBRANE (PHOBIUS); NON_CYTOPLASMIC_DOMAIN (PHOBIUS); TRANSMEMBRANE (PHOBIUS); CYTOPLASMIC_DOMAIN (PHOBIUS); SSF54001 (SUPERFAMILY); TMhelix (TMHMM)
1979	299374_length_1758_cvg_15.2_tip_1_5	278	serine threonine-protein kinase grp-like	586	0	74.50%	0.247	IPR002290 (SMART); G3DSA:3.30.200.20 (GENE3D); IPR000719 (PFAM); G3DSA:1.10.510.10 (GENE3D); PTHR24344 (PANTHER); IPR017441 (PROSITE_PATTERNS); IPR008271 (PROSITE_PATTERNS); IPR000719 (PROSITE_PROFILES); IPR011009 (SUPERFAMILY)
1980	299424_length_1767_cvg_9.6_tip_1_5	240	isoform b	589	7.71E-65	52.40%	0.11	IPR001079 (SMART); IPR001079 (SMART); IPR001079 (PFAM); IPR013320 (G3DSA:2.60.120.GENE3D); IPR013320 (G3DSA:2.60.120.GENE3D); PTHR11346:SF83 (PANTHER); PTHR11346 (PANTHER); TRANSMEMBRANE (PHOBIUS); NON_CYTOPLASMIC_DOMAIN (PHOBIUS); CYTOPLASMIC_DOMAIN (PHOBIUS); IPR001079 (PROSITE_PROFILES); IPR001079 (PROSITE_PROFILES); IPR013320 (SUPERFAMILY); IPR013320 (SUPERFAMILY); TMhelix (TMHMM)
1985	299496_length_1779_cvg_8.7_tip_1_3	190	cytochrome p450 partial	589	1.51E-113	55.40%	0.162	IPR002401 (PRINTS); IPR001128 (PRINTS); IPR001128 (G3DSA:1.10.630.GENE3D); IPR001128 (PFAM); PTHR24292 (PANTHER); PTHR24292:SF24 (PANTHER); IPR017972 (PROSITE_PATTERNS); TRANSMEMBRANE (PHOBIUS); NON_CYTOPLASMIC_DOMAIN (PHOBIUS); NON_CYTOPLASMIC_DOMAIN (PHOBIUS); TRANSMEMBRANE (PHOBIUS); CYTOPLASMIC_DOMAIN (PHOBIUS); IPR001128 (SUPERFAMILY); TMhelix (TMHMM); TMhelix (TMHMM)
1981	299476_length_1776_cvg_24.4_tip_1_5	612	chitinase 2	592	9.76E-132	57.00%	0.122	IPR002557 (SMART); IPR011583 (SMART); IPR001223 (PFAM); IPR002557 (PFAM); IPR013781 (G3DSA:3.20.20.GENE3D); IPR002557 (G3DSA:2.170.140.GENE3D); IPR029070 (G3DSA:3.10.50.GENE3D); PTHR11177 (PANTHER); PTHR11177:SF112 (PANTHER); IPR001579 (PROSITE_PATTERNS); TRANSMEMBRANE (PHOBIUS); CYTOPLASMIC_DOMAIN (PHOBIUS); NON_CYTOPLASMIC_DOMAIN (PHOBIUS); IPR002557 (PROSITE_PROFILES); IPR002557 (PROSITE_PROFILES); IPR002557 (SUPERFAMILY); IPR002557 (SUPERFAMILY); IPR017853 (SUPERFAMILY); IPR029070 (SUPERFAMILY); TMhelix (TMHMM)

1983	299492_length_1778_cvg_8.6_tip_1_5	220	calnexin	592	0	75.20%	0.216	Coil (COILS); IPR001580 (PRINTS); IPR013320 (G3DSA:2.60.120.GENE3D); IPR001580 (PFAM); IPR009033 (G3DSA:2.10.250.GENE3D); IPR009033 (G3DSA:2.10.250.GENE3D); PTHR11073:SF1 (PANTHER); IPR001580 (PANTHER); IPR018124 (PROSITE_PATTERNS); IPR018124 (PROSITE_PATTERNS); IPR018124 (PROSITE_PATTERNS); IPR018124 (PROSITE_PATTERNS); TRANSMEMBRANE (PHOBIUS); NON_CYTOPLASMIC_DOMAIN (PHOBIUS); CYTOPLASMIC_DOMAIN (PHOBIUS); IPR013320 (SUPERFAMILY); IPR009033 (SUPERFAMILY); TMhelix (TMHMM)
1982	299490_length_1778_cvg_6.1_tip_1_0	134	von willebrand factor a domain-containing protein 8	593	0	73.10%	0.169	IPR027417 (G3DSA:3.40.50.GENE3D); IPR011704 (PFAM); PTHR21610:SF9 (PANTHER); PTHR21610 (PANTHER); IPR027417 (SUPERFAMILY); IPR027417 (SUPERFAMILY)
1984	299494_length_1778_cvg_6.4_tip_1_4	201	laminin subunit alpha	593	2.29E-153	57.70%	0.137	IPR001791 (SMART); IPR013320 (G3DSA:2.60.120.GENE3D); IPR013320 (G3DSA:2.60.120.GENE3D); IPR001791 (PFAM); IPR013320 (G3DSA:2.60.120.GENE3D); PTHR10574:SF39 (PANTHER); PTHR10574 (PANTHER); IPR001791 (PROSITE_PROFILES); IPR001791 (PROSITE_PROFILES); IPR001791 (PROSITE_PROFILES); IPR013320 (SUPERFAMILY); IPR013320 (SUPERFAMILY); IPR013320 (SUPERFAMILY)
1986	299498_length_1779_cvg_11.0_tip_1_4	231	dnaj homolog subfamily c member 7	593	0	74.90%	0.104	IPR001623 (PRINTS); IPR001623 (SMART); IPR019734 (SMART); IPR001623 (PFAM); IPR013105 (PFAM); IPR001623 (G3DSA:1.10.287.GENE3D); IPR011990 (G3DSA:1.25.40.GENE3D); IPR011990 (G3DSA:1.25.40.GENE3D); PF13414 (PFAM); IPR019734 (PFAM); PTHR24078 (PANTHER); PTHR24078:SF140 (PANTHER); IPR018253 (PROSITE_PATTERNS); IPR013026 (PROSITE_PROFILES); IPR001623 (PROSITE_PROFILES); IPR019734 (PROSITE_PROFILES); IPR019734 (PROSITE_PROFILES); IPR019734 (PROSITE_PROFILES); IPR019734 (PROSITE_PROFILES); IPR019734 (PROSITE_PROFILES); IPR019734 (PROSITE_PROFILES); IPR019734 (PROSITE_PROFILES); SSF48452 (SUPERFAMILY); IPR001623 (SUPERFAMILY); SSF48452 (SUPERFAMILY); SSF48452 (SUPERFAMILY)
1987	299506_length_1781_cvg_5.8_tip_1_2	187	latrophilin cirl-like isoform x7	593	0	64.20%	0.165	IPR001879 (SMART); IPR000203 (SMART); IPR001879 (PFAM); IPR000922 (PFAM); IPR022624 (PFAM); IPR000832 (PFAM); IPR000203 (PFAM); PTHR12011:SF217 (PANTHER); PTHR12011 (PANTHER); CYTOPLASMIC_DOMAIN (PHOBIUS); NON_CYTOPLASMIC_DOMAIN (PHOBIUS); TRANSMEMBRANE (PHOBIUS); IPR000203 (PROSITE_PROFILES); IPR001879 (PROSITE_PROFILES); IPR000922 (PROSITE_PROFILES); SignalP-TM (SIGNALP_GRAM_POSITIVE); TMhelix (TMHMM)
1988	299508_length_1781_cvg_3.4_tip_1_2	86	#NAME?	593	0	82.30%	0.135	IPR006589 (SMART); IPR006407 (PIRSF); IPR006047 (PFAM); IPR013781 (G3DSA:3.20.20.GENE3D); IPR013780 (G3DSA:2.60.40.GENE3D); IPR006048 (PFAM); IPR015902 (PANTHER); PTHR10357:SF110 (PANTHER); IPR017853 (SUPERFAMILY); SSF51011 (SUPERFAMILY)
1989	299512_length_1781_cvg_14.2_tip_1_2	343	af425264_1lysozyme precursor	593	9.74E-37	65.30%	0.102	IPR001916 (PRINTS); IPR000974 (PRINTS); IPR001916 (SMART); G3DSA:1.10.530.10 (GENE3D); IPR001916 (PFAM); PTHR11407 (PANTHER); NON_CYTOPLASMIC_DOMAIN (PHOBIUS); TRANSMEMBRANE (PHOBIUS); TRANSMEMBRANE (PHOBIUS); NON_CYTOPLASMIC_DOMAIN (PHOBIUS); CYTOPLASMIC_DOMAIN (PHOBIUS); TRANSMEMBRANE (PHOBIUS); TRANSMEMBRANE (PHOBIUS); CYTOPLASMIC_DOMAIN (PHOBIUS); NON_CYTOPLASMIC_DOMAIN (PHOBIUS); IPR001916 (PROSITE_PROFILES); IPR023346 (SUPERFAMILY); TMhelix (TMHMM); TMhelix (TMHMM); TMhelix (TMHMM); TMhelix (TMHMM)
2009	299742_length_1828_cvg_72.7_tip_1_2	8741	retinal dehydrogenase 1-like	593	0	82.10%	0.121	IPR016163 (G3DSA:3.40.309.GENE3D); IPR016162 (G3DSA:3.40.605.GENE3D); IPR015590 (PFAM); PTHR11699 (PANTHER); PTHR11699:SF145 (PANTHER); IPR016160 (PROSITE_PATTERNS); IPR029510 (PROSITE_PATTERNS); IPR016161 (SUPERFAMILY)
1990	299518_length_1782_cvg_38.4_tip_1_5	964	5 -nucleotidase-like	594	8.53E-87	56.50%	0.133	IPR006179 (PRINTS); IPR029052 (G3DSA:3.60.21.GENE3D); IPR008334 (G3DSA:3.90.780.GENE3D); IPR008334 (PFAM); IPR006179 (PANTHER); PTHR11575:SF21 (PANTHER); IPR029052 (SUPERFAMILY); IPR008334 (SUPERFAMILY)
1991	299526_length_1784_cvg_36.1_tip_1_0	788	phosphatidylethanolamine-binding protein	595	4.63E-88	79.90%	0.168	IPR008914 (PFAM); IPR008914 (G3DSA:3.90.280.GENE3D); PTHR11362 (PANTHER); IPR008914 (SUPERFAMILY)

1992	299550_length_1788_cvg_6.8_tip_1_5	190	cadherin-like membrane protein	596	1.92E-51	45.70%	0.102	IPR002126 (PRINTS); IPR002126 (SMART); IPR002126 (PFAM); IPR002126 (G3DSA:2.60.40.GENE3D); IPR002126 (G3DSA:2.60.40.GENE3D); IPR002126 (G3DSA:2.60.40.GENE3D); PTHR24027 (PANTHER); IPR020894 (PROSITE_PATTERNS); IPR020894 (PROSITE_PATTERNS); IPR002126 (PROSITE_PROFILES); IPR002126 (PROSITE_PROFILES); IPR002126 (PROSITE_PROFILES); IPR002126 (PROSITE_PROFILES); IPR002126 (PROSITE_PROFILES); IPR015919 (SUPERFAMILY); IPR015919 (SUPERFAMILY); IPR015919 (SUPERFAMILY); IPR015919 (SUPERFAMILY); IPR015919 (SUPERFAMILY)
1993	299552_length_1788_cvg_35.8_tip_1_5	777	proclotting enzyme	596	6.25E-132	64.00%	0.205	IPR001314 (PRINTS); IPR006604 (SMART); IPR001254 (SMART); G3DSA:2.40.10.10 (GENE3D); IPR001254 (PFAM); G3DSA:2.40.10.10 (GENE3D); PTHR24268 (PANTHER); PTHR24268:SF81 (PANTHER); IPR018114 (PROSITE_PATTERNS); IPR018114 (PROSITE_PATTERNS); TRANSMEMBRANE (PHOBIUS); NON_CYTOPLASMIC_DOMAIN (PHOBIUS); CYTOPLASMIC_DOMAIN (PHOBIUS); IPR001254 (PROSITE_PROFILES); IPR009003 (SUPERFAMILY); TMhelix (TMHMM)
1994	299558_length_1789_cvg_6.8_tip_1_1	186	membrane metallo-endopeptidase-like 1-like	596	8.92E-64	47.70%	0.171	IPR018497 (PRINTS); IPR018497 (PFAM); IPR008753 (PFAM); IPR024079 (G3DSA:3.40.390.GENE3D); IPR000718 (PANTHER); SSF55486 (SUPERFAMILY)
2010	299780_length_1832_cvg_30.9_tip_1_1	870	microneme protein mic12	596	7.97E-20	38.80%	0.104	IPR000742 (SMART); IPR001881 (SMART); G3DSA:2.10.25.10 (GENE3D); G3DSA:2.10.25.10 (GENE3D); IPR001881 (PFAM); G3DSA:2.10.25.10 (GENE3D); G3DSA:2.10.25.10 (GENE3D); G3DSA:2.10.25.10 (GENE3D); PTHR24034 (PANTHER); PTHR24034:SF33 (PANTHER); IPR013032 (PROSITE_PATTERNS); IPR000152 (PROSITE_PATTERNS); IPR013032 (PROSITE_PATTERNS); IPR000152 (PROSITE_PATTERNS); IPR013032 (PROSITE_PATTERNS); IPR013032 (PROSITE_PATTERNS); IPR013032 (PROSITE_PATTERNS); IPR000152 (PROSITE_PATTERNS); CYTOPLASMIC_DOMAIN (PHOBIUS); TRANSMEMBRANE (PHOBIUS); NON_CYTOPLASMIC_DOMAIN (PHOBIUS); IPR000742 (PROSITE_PROFILES); IPR000742 (PROSITE_PROFILES); IPR000742 (PROSITE_PROFILES); IPR000742 (PROSITE_PROFILES); IPR009030 (SUPERFAMILY); IPR009030 (SUPERFAMILY); TMhelix (TMHMM)
1995	299566_length_1792_cvg_64.5_tip_1_3	2653	preamylase 1	598	0	76.60%	0.274	IPR006046 (PRINTS); IPR006048 (SMART); IPR006589 (SMART); IPR006048 (PFAM); IPR006047 (PFAM); IPR013781 (G3DSA:3.20.20.GENE3D); IPR013780 (G3DSA:2.60.40.GENE3D); PTHR10357:SF116 (PANTHER); IPR015902 (PANTHER); IPR017853 (SUPERFAMILY); SSF51011 (SUPERFAMILY); TMhelix (TMHMM)
1996	299568_length_1793_cvg_5.1_tip_1_3	119	low quality protein: cubilin	598	2.53E-38	47.10%	0.112	IPR000859 (SMART); IPR002035 (G3DSA:3.40.50.GENE3D); IPR000859 (G3DSA:2.60.120.GENE3D); IPR000859 (PFAM); IPR002035 (PFAM); IPR000859 (G3DSA:2.60.120.GENE3D); PTHR10127 (PANTHER); IPR000859 (PROSITE_PROFILES); IPR000859 (PROSITE_PROFILES); IPR000859 (PROSITE_PROFILES); IPR002035 (PROSITE_PROFILES); IPR000859 (SUPERFAMILY); IPR000859 (SUPERFAMILY); IPR002035 (SUPERFAMILY); IPR000859 (SUPERFAMILY)
1997	299572_length_1794_cvg_57.9_tip_1_3	3474	trypsinogen 2	598	1.54E-66	61.80%	0.117	IPR001314 (PRINTS); IPR001254 (SMART); G3DSA:2.40.10.10 (GENE3D); IPR001254 (PFAM); G3DSA:2.40.10.10 (GENE3D); G3DSA:2.40.10.10 (GENE3D); PTHR24265 (PANTHER); IPR018114 (PROSITE_PATTERNS); IPR018114 (PROSITE_PATTERNS); NON_CYTOPLASMIC_DOMAIN (PHOBIUS); TRANSMEMBRANE (PHOBIUS); CYTOPLASMIC_DOMAIN (PHOBIUS); IPR001254 (PROSITE_PROFILES); IPR009003 (SUPERFAMILY); TMhelix (TMHMM)
1998	299580_length_1795_cvg_19.6_tip_1_1	623	serpin 3	598	2.83E-82	58.90%	0.147	IPR023796 (SMART); G3DSA:3.30.497.10 (GENE3D); G3DSA:2.30.39.10 (GENE3D); IPR023796 (PFAM); IPR000215 (PANTHER); PTHR11461:SF52 (PANTHER); IPR023795 (PROSITE_PATTERNS); TRANSMEMBRANE (PHOBIUS); CYTOPLASMIC_DOMAIN (PHOBIUS); NON_CYTOPLASMIC_DOMAIN (PHOBIUS); IPR023796 (SUPERFAMILY); TMhelix (TMHMM)
1999	299604_length_1800_cvg_7.9_tip_1_3	172	letm1 and ef-hand domain-containing protein anon-mitochondrial-like	600	2.58E-105	58.30%	0.205	Coil (COILS); Coil (COILS); Coil (COILS); Coil (COILS); Coil (COILS); IPR011685 (PFAM); IPR011992 (G3DSA:1.10.238.GENE3D); PTHR14009:SF1 (PANTHER); PTHR14009 (PANTHER); IPR002048 (PROSITE_PROFILES); SSF47473 (SUPERFAMILY)
2000	299628_length_1806_cvg_9.5_tip_1_4	232	alkaline phosphatase 4-like	602	4.79E-160	64.00%	0.243	IPR001952 (PRINTS); IPR001952 (SMART); IPR001952 (PFAM); IPR017849 (G3DSA:3.40.720.GENE3D); PTHR11596 (PANTHER); IPR018299 (PROSITE_PATTERNS); NON_CYTOPLASMIC_DOMAIN (PHOBIUS); TRANSMEMBRANE (PHOBIUS); CYTOPLASMIC_DOMAIN (PHOBIUS); SignalP-TM (SIGNALP_GRAM_POSITIVE); IPR017850 (SUPERFAMILY); TMhelix (TMHMM)

2001	299636_length_1808_cvg_16.5_tip_0_2	429	fibrinogen-like protein 1	602	2.23E-23	63.00%	0.114	IPR002181 (SMART); IPR002181 (PFAM); IPR014715 (G3DSA:4.10.530.GENE3D); PTHR19143 (PANTHER); IPR020837 (PROSITE_PATTERNS); CYTOPLASMIC_DOMAIN (PHOBIUS); TRANSMEMBRANE (PHOBIUS); TRANSMEMBRANE (PHOBIUS); NON_CYTOPLASMIC_DOMAIN (PHOBIUS); TRANSMEMBRANE (PHOBIUS); CYTOPLASMIC_DOMAIN (PHOBIUS); NON_CYTOPLASMIC_DOMAIN (PHOBIUS); IPR002181 (PROSITE_PROFILES); IPR002181 (SUPERFAMILY)
2002	299640_length_1809_cvg_9.1_tip_1_2	220	leucine-rich repeat-containing protein 59	603	9.09E-63	65.50%	0.119	Coil (COILS); Coil (COILS); IPR003591 (SMART); IPR001611 (PFAM); G3DSA:3.80.10.10 (GENE3D); PTHR23155 (PANTHER); CYTOPLASMIC_DOMAIN (PHOBIUS); NON_CYTOPLASMIC_DOMAIN (PHOBIUS); TRANSMEMBRANE (PHOBIUS); CYTOPLASMIC_DOMAIN (PHOBIUS); NON_CYTOPLASMIC_DOMAIN (PHOBIUS); TRANSMEMBRANE (PHOBIUS); TRANSMEMBRANE (PHOBIUS); CYTOPLASMIC_DOMAIN (PHOBIUS); TRANSMEMBRANE (PHOBIUS); IPR001611 (PROSITE_PROFILES); IPR001611 (PROSITE_PROFILES); IPR001611 (PROSITE_PROFILES); IPR001611 (PROSITE_PROFILES); SSF52058 (SUPERFAMILY); TMhelix (TMHMM); TMhelix (TMHMM); TMhelix (TMHMM)
2003	299642_length_1810_cvg_8.1_tip_1_0	186	tyrosine-protein phosphatase lar-like isoform x6	604	0	93.00%	0.113	IPR000242 (PRINTS); IPR000242 (SMART); IPR003595 (SMART); IPR000242 (PFAM); IPR029021 (G3DSA:3.90.190.GENE3D); IPR029021 (G3DSA:3.90.190.GENE3D); PTHR19134 (PANTHER); PTHR19134:SF199 (PANTHER); IPR016130 (PROSITE_PATTERNS); IPR016130 (PROSITE_PATTERNS); IPR000387 (PROSITE_PROFILES); IPR000242 (PROSITE_PROFILES); IPR000387 (PROSITE_PROFILES); IPR000242 (PROSITE_PROFILES); IPR029021 (SUPERFAMILY); IPR029021 (SUPERFAMILY)
2004	299676_length_1815_cvg_38.1_tip_1_2	1177	peptidyl-prolyl cis-trans isomerase b	605	7.72E-108	86.30%	0.508 Y	IPR002130 (PRINTS); IPR002130 (PFAM); IPR029000 (G3DSA:2.40.100.GENE3D); IPR024936 (PANTHER); PTHR11071:SF63 (PANTHER); IPR020892 (PROSITE_PATTERNS); NON_CYTOPLASMIC_DOMAIN (PHOBIUS); TRANSMEMBRANE (PHOBIUS); NON_CYTOPLASMIC_DOMAIN (PHOBIUS); TRANSMEMBRANE (PHOBIUS); CYTOPLASMIC_DOMAIN (PHOBIUS); IPR002130 (PROSITE_PROFILES); SignalP-TM (SIGNALP_EUK); IPR029000 (SUPERFAMILY)
2005	299692_length_1818_cvg_15.5_tip_1_2	331	rna-binding protein 5-like	606	5.23E-153	64.30%	0.146	Coil (COILS); IPR000467 (SMART); IPR000467 (PFAM); PTHR13948 (PANTHER); NON_CYTOPLASMIC_DOMAIN (PHOBIUS); CYTOPLASMIC_DOMAIN (PHOBIUS); TRANSMEMBRANE (PHOBIUS); IPR000467 (PROSITE_PROFILES); IPR007087 (PROSITE_PROFILES)
2006	299710_length_1822_cvg_43.2_tip_1_2	834	universal minicircle sequence binding protein	607	8.97E-27	51.50%	0.183	IPR001878 (SMART); IPR001878 (G3DSA:4.10.60.GENE3D); IPR001878 (G3DSA:4.10.60.GENE3D); IPR001878 (PFAM); PTHR23002 (PANTHER); NON_CYTOPLASMIC_DOMAIN (PHOBIUS); CYTOPLASMIC_DOMAIN (PHOBIUS); NON_CYTOPLASMIC_DOMAIN (PHOBIUS); TRANSMEMBRANE (PHOBIUS); TRANSMEMBRANE (PHOBIUS); IPR001878 (PROSITE_PROFILES); IPR001878 (PROSITE_PROFILES); IPR001878 (PROSITE_PROFILES); IPR001878 (PROSITE_PROFILES); IPR001878 (PROSITE_PROFILES); IPR001878 (SUPERFAMILY); IPR001878 (SUPERFAMILY); IPR001878 (SUPERFAMILY); TMhelix (TMHMM); TMhelix (TMHMM)
2007	299718_length_1823_cvg_15.1_tip_1_1	432	allene oxide synthase-lipoxygenase	608	0	68.80%	0.109	Coil (COILS); IPR013819 (PRINTS); IPR013819 (PFAM); G3DSA:1.20.245.10 (GENE3D); PTHR11771:SF43 (PANTHER); IPR000907 (PANTHER); IPR020834 (PROSITE_PATTERNS); IPR013819 (PROSITE_PROFILES); IPR013819 (SUPERFAMILY)
2016	299824_length_1841_cvg_41.0_tip_1_3	2244	orcokinin precursor	609	2.79E-21	77.70%	0.241	no IPS match
2017	299824_length_1841_cvg_41.0_tip_1_4	2244	pedal peptide 2 precursor	609	5.82E-39	59.50%	0.25	no IPS match
2008	299740_length_1828_cvg_9.1_tip_1_0	245	cytochrome p450 3a24-like	610	6.96E-111	58.70%	0.141	IPR002401 (PRINTS); IPR001128 (PRINTS); IPR001128 (G3DSA:1.10.630.GENE3D); IPR001128 (PFAM); PTHR24302 (PANTHER); PTHR24302:SF7 (PANTHER); IPR017972 (PROSITE_PATTERNS); CYTOPLASMIC_DOMAIN (PHOBIUS); TRANSMEMBRANE (PHOBIUS); NON_CYTOPLASMIC_DOMAIN (PHOBIUS); CYTOPLASMIC_DOMAIN (PHOBIUS); TRANSMEMBRANE (PHOBIUS); TRANSMEMBRANE (PHOBIUS); NON_CYTOPLASMIC_DOMAIN (PHOBIUS); IPR001128 (SUPERFAMILY); TMhelix (TMHMM); TMhelix (TMHMM); TMhelix (TMHMM)

2011	299806_length_1839_cvg_6.9_tip_1_3	151	apical endosomal glyco	613	3.60E-14	39.00%	0.107		IPR000998 (SMART); IPR000859 (SMART); IPR002172 (SMART); IPR000998 (PFAM); IPR000859 (PFAM); IPR002172 (PFAM); IPR002172 (G3DSA:4.10.400.GENE3D); IPR000859 (G3DSA:2.60.120.GENE3D); PTHR22991 (PANTHER); PTHR22991:SF23 (PANTHER); IPR000998 (PROSITE_PROFILES); IPR002172 (PROSITE_PROFILES); IPR000998 (PROSITE_PROFILES); IPR000859 (PROSITE_PROFILES); IPR013320 (SUPERFAMILY); IPR013320 (SUPERFAMILY); IPR000859 (SUPERFAMILY); IPR002172 (SUPERFAMILY)
2012	299808_length_1839_cvg_9.2_tip_1_4	223	inter-alpha-trypsin inhibitor heavy chain h3-like	613	1.89E-121	62.70%	0.138		Coil (COILS); IPR002035 (SMART); IPR013694 (SMART); IPR002035 (PFAM); IPR002035 (G3DSA:3.40.50.GENE3D); IPR013694 (PFAM); PTHR10338 (PANTHER); PTHR10338:SF108 (PANTHER); NON_CYTOPLASMIC_DOMAIN (PHOBIUS); TRANSMEMBRANE (PHOBIUS); CYTOPLASMIC_DOMAIN (PHOBIUS); IPR002035 (PROSITE_PROFILES); IPR013694 (PROSITE_PROFILES); IPR002035 (SUPERFAMILY); TMhelix (TMHMM)
2013	299814_length_1840_cvg_5.4_tip_1_2	125	maguk p55 subfamily member 6	613	0	85.80%	0.104		IPR008145 (SMART); IPR027417 (G3DSA:3.40.50.GENE3D); G3DSA:3.30.63.10 (GENE3D); G3DSA:2.30.30.40 (GENE3D); IPR001452 (PFAM); IPR008145 (PFAM); PTHR23122 (PANTHER); IPR020590 (PROSITE_PATTERNS); TRANSMEMBRANE (PHOBIUS); NON_CYTOPLASMIC_DOMAIN (PHOBIUS); CYTOPLASMIC_DOMAIN (PHOBIUS); IPR001452 (PROSITE_PROFILES); IPR008144 (PROSITE_PROFILES); IPR001452 (SUPERFAMILY); IPR027417 (SUPERFAMILY)
2014	299818_length_1841_cvg_19.9_tip_1_1	402	chromodomain-helicase-dna-binding protein mi-2 homolog isoform x5	614	0	76.80%	0.14		Coil (COILS); Coil (COILS); IPR009462 (PFAM); IPR012957 (PFAM); IPR028727 (PTHR10799:PANTHER); PTHR10799 (PANTHER)
2015	299820_length_1841_cvg_9.0_tip_1_1	231	alpha-mannosidase 2	614	7.42E-110	58.20%	0.17		G3DSA:2.70.98.30 (GENE3D); IPR011682 (PFAM); IPR013780 (G3DSA:2.60.40.GENE3D); PTHR11607 (PANTHER); PTHR11607:SF4 (PANTHER); TRANSMEMBRANE (PHOBIUS); CYTOPLASMIC_DOMAIN (PHOBIUS); NON_CYTOPLASMIC_DOMAIN (PHOBIUS); IPR011013 (SUPERFAMILY); TMhelix (TMHMM)
2033	300028_length_1886_cvg_5.0_tip_1_4	129	n-sulphoglucosamine sulphohydrolase-like	614	0	79.40%	0.101		Coil (COILS); IPR017849 (G3DSA:3.40.720.GENE3D); G3DSA:3.30.1120.10 (GENE3D); IPR000917 (PFAM); PTHR10342 (PANTHER); PTHR10342:SF76 (PANTHER); TRANSMEMBRANE (PHOBIUS); TRANSMEMBRANE (PHOBIUS); CYTOPLASMIC_DOMAIN (PHOBIUS); CYTOPLASMIC_DOMAIN (PHOBIUS); NON_CYTOPLASMIC_DOMAIN (PHOBIUS); NON_CYTOPLASMIC_DOMAIN (PHOBIUS); TRANSMEMBRANE (PHOBIUS); IPR017850 (SUPERFAMILY)
2018	299840_length_1843_cvg_8.4_tip_1_0	267	sorbin and sh3 domain-containing protein partial	615	0	77.80%	0.106		Coil (COILS); PR00499 (PRINTS); IPR001452 (PRINTS); IPR001452 (SMART); G3DSA:2.30.30.40 (GENE3D); IPR001452 (PFAM); IPR001452 (PFAM); G3DSA:2.30.30.40 (GENE3D); G3DSA:2.30.30.40 (GENE3D); PTHR10661 (PANTHER); PTHR10661:SF66 (PANTHER); IPR001452 (PROSITE_PROFILES); IPR001452 (PROSITE_PROFILES); IPR001452 (PROSITE_PROFILES); IPR001452 (SUPERFAMILY); IPR001452 (SUPERFAMILY); IPR001452 (SUPERFAMILY)
2019	299856_length_1847_cvg_8.6_tip_1_0	185	ankyrin sam and basic leucine zipper domain-containing protein 1-like	616	1.49E-130	61.00%	0.099		IPR001660 (SMART); IPR002110 (SMART); IPR020683 (G3DSA:1.25.40.GENE3D); IPR020683 (PFAM); IPR021129 (PFAM); IPR013761 (G3DSA:1.10.150.GENE3D); PTHR24157 (PANTHER); IPR002110 (PROSITE_PROFILES); IPR020683 (PROSITE_PROFILES); IPR002110 (PROSITE_PROFILES); IPR002110 (PROSITE_PROFILES); IPR001660 (PROSITE_PROFILES); IPR013761 (SUPERFAMILY); IPR020683 (SUPERFAMILY)
2020	299868_length_1850_cvg_12.7_tip_1_2	306	pancreatic lipase-related protein 2-like	616	8.35E-157	64.60%	0.142		IPR002331 (PRINTS); IPR000734 (PRINTS); IPR029058 (G3DSA:3.40.50.GENE3D); IPR013818 (PFAM); IPR001024 (G3DSA:2.60.60.GENE3D); PTHR11610:SF83 (PANTHER); IPR000734 (PANTHER); IPR001024 (PROSITE_PROFILES); IPR029058 (SUPERFAMILY); IPR008976 (SUPERFAMILY)
2021	299874_length_1852_cvg_17.7_tip_1_2	538	cathepsin l-like	617	5.21E-166	79.60%	0.131		IPR000668 (PRINTS); IPR000668 (SMART); IPR013201 (SMART); G3DSA:3.90.70.10 (GENE3D); IPR013201 (PFAM); IPR000668 (PFAM); PTHR12411:SF57 (PANTHER); IPR013128 (PANTHER); IPR025660 (PROSITE_PATTERNS); IPR000169 (PROSITE_PATTERNS); IPR025661 (PROSITE_PATTERNS); NON_CYTOPLASMIC_DOMAIN (PHOBIUS); TRANSMEMBRANE (PHOBIUS); TRANSMEMBRANE (PHOBIUS); TRANSMEMBRANE (PHOBIUS); CYTOPLASMIC_DOMAIN (PHOBIUS); NON_CYTOPLASMIC_DOMAIN (PHOBIUS); CYTOPLASMIC_DOMAIN (PHOBIUS); SSF54001 (SUPERFAMILY); TMhelix (TMHMM); TMhelix (TMHMM); TMhelix (TMHMM)
2022	299898_length_1857_cvg_3.6_tip_1_4	102	mitochondrial import inner membrane translocase subunit tim8 a	619	3.34E-30	82.20%	0.449	Y	IPR004217 (G3DSA:1.10.287.GENE3D); IPR004217 (PFAM); PTHR21535 (PANTHER); IPR004217 (SUPERFAMILY)

2023	299902_length_1857_cvg_10.1_tip_1_1	196	ectonucleotide pyrophosphatase phosphodiesterase family member 6	619	3.86E-108	54.60%	0.106	IPR002591 (PFAM); IPR017849 (G3DSA:3.40.720.GENE3D); IPR024873 (PANTHER); TRANSMEMBRANE (PHOBIUS); CYTOPLASMIC_DOMAIN (PHOBIUS); NON_CYTOPLASMIC_DOMAIN (PHOBIUS); IPR017850 (SUPERFAMILY)
2024	299918_length_1862_cvg_5.9_tip_1_1	131	neurexin-4 isoform x3	621	0	64.10%	0.129	IPR001791 (SMART); IPR000742 (SMART); IPR013320 (G3DSA:2.60.120.GENE3D); G3DSA:2.10.25.10 (GENE3D); IPR013320 (G3DSA:2.60.120.GENE3D); IPR000742 (PFAM); IPR001791 (PFAM); PTHR10127:SF346 (PANTHER); PTHR10127 (PANTHER); IPR000742 (PROSITE_PROFILES); IPR001791 (PROSITE_PROFILES); IPR001791 (PROSITE_PROFILES); IPR000742 (PROSITE_PROFILES); IPR013320 (SUPERFAMILY); IPR013320 (SUPERFAMILY); IPR013320 (SUPERFAMILY)
2025	299924_length_1863_cvg_29.1_tip_1_1	862	iron zinc purple acid phosphatase-like protein	621	0	74.40%	0.238	IPR029052 (G3DSA:3.60.21.GENE3D); IPR025733 (PFAM); IPR015914 (G3DSA:2.60.40.GENE3D); IPR004843 (PFAM); PTHR22953 (PANTHER); PTHR22953:SF9 (PANTHER); TRANSMEMBRANE (PHOBIUS); CYTOPLASMIC_DOMAIN (PHOBIUS); NON_CYTOPLASMIC_DOMAIN (PHOBIUS); TRANSMEMBRANE (PHOBIUS); CYTOPLASMIC_DOMAIN (PHOBIUS); IPR029052 (SUPERFAMILY); IPR008963 (SUPERFAMILY); TMhelix (TMHMM)
2026	299934_length_1865_cvg_59.4_tip_0_4	3385	kielin chordin-like protein	622	9.00E-58	52.60%	0.117	IPR002223 (PRINTS); IPR002223 (SMART); IPR002223 (G3DSA:4.10.410.GENE3D); IPR002223 (PFAM); IPR008037 (PFAM); PTHR10083 (PANTHER); IPR020901 (PROSITE_PATTERNS); IPR002223 (PROSITE_PROFILES); IPR020862 (PROSITE_PROFILES); IPR020862 (PROSITE_PROFILES); IPR002223 (PROSITE_PROFILES); IPR008037 (SUPERFAMILY); IPR008037 (SUPERFAMILY); IPR002223 (SUPERFAMILY); IPR002223 (SUPERFAMILY)
2027	299948_length_1868_cvg_27.4_tip_1_2	655	mitochondrial-processing peptidase subunit beta	622	0	85.90%	0.111	IPR011765 (PFAM); IPR011237 (G3DSA:3.30.830.GENE3D); IPR007863 (PFAM); IPR011237 (G3DSA:3.30.830.GENE3D); PTHR11851:SF103 (PANTHER); PTHR11851 (PANTHER); IPR001431 (PROSITE_PATTERNS); IPR011249 (SUPERFAMILY); IPR011249 (SUPERFAMILY)
2028	299954_length_1868_cvg_53.6_tip_1_5	1840	ectonucleotide pyrophosphatase phosphodiesterase family member 6	622	4.96E-98	54.90%	0.245	IPR017849 (G3DSA:3.40.720.GENE3D); IPR002591 (PFAM); IPR024873 (PANTHER); SIGNAL_PEPTIDE (PHOBIUS); TRANSMEMBRANE (PHOBIUS); CYTOPLASMIC_DOMAIN (PHOBIUS); NON_CYTOPLASMIC_DOMAIN (PHOBIUS); NON_CYTOPLASMIC_DOMAIN (PHOBIUS); TRANSMEMBRANE (PHOBIUS); SIGNAL_PEPTIDE_H_REGION (PHOBIUS); TRANSMEMBRANE (PHOBIUS); CYTOPLASMIC_DOMAIN (PHOBIUS); SIGNAL_PEPTIDE_N_REGION (PHOBIUS); SIGNAL_PEPTIDE_C_REGION (PHOBIUS); IPR017850 (SUPERFAMILY); TMhelix (TMHMM); TMhelix (TMHMM); TMhelix (TMHMM); TMhelix (TMHMM)
2031	299994_length_1877_cvg_37.8_tip_1_5	1108	superoxide dismutase	625	1.62E-45	67.20%	0.149	IPR001424 (PRINTS); IPR001424 (G3DSA:2.60.40.GENE3D); IPR001424 (PFAM); PTHR10003 (PANTHER); IPR018152 (PROSITE_PATTERNS); IPR018152 (PROSITE_PATTERNS); TRANSMEMBRANE (PHOBIUS); TRANSMEMBRANE (PHOBIUS); TRANSMEMBRANE (PHOBIUS); CYTOPLASMIC_DOMAIN (PHOBIUS); NON_CYTOPLASMIC_DOMAIN (PHOBIUS); NON_CYTOPLASMIC_DOMAIN (PHOBIUS); CYTOPLASMIC_DOMAIN (PHOBIUS); IPR001424 (SUPERFAMILY)
2032	299996_length_1877_cvg_3.6_tip_1_5	123	homeotic protein spalt-major-like isoform x2	625	1.93E-118	63.60%	0.325	IPR015880 (SMART); PF13465 (PFAM); IPR013087 (G3DSA:3.30.160.GENE3D); IPR013087 (G3DSA:3.30.160.GENE3D); PTHR23233:SF51 (PANTHER); PTHR23233 (PANTHER); IPR007087 (PROSITE_PATTERNS); IPR007087 (PROSITE_PATTERNS); IPR007087 (PROSITE_PATTERNS); IPR007087 (PROSITE_PATTERNS); TRANSMEMBRANE (PHOBIUS); SIGNAL_PEPTIDE_N_REGION (PHOBIUS); SIGNAL_PEPTIDE (PHOBIUS); NON_CYTOPLASMIC_DOMAIN (PHOBIUS); CYTOPLASMIC_DOMAIN (PHOBIUS); SIGNAL_PEPTIDE_H_REGION (PHOBIUS); SIGNAL_PEPTIDE_C_REGION (PHOBIUS); IPR007087 (PROSITE_PROFILES); IPR007087 (PROSITE_PROFILES); IPR007087 (PROSITE_PROFILES); IPR007087 (PROSITE_PROFILES); SignalP-TM (SIGNALP_GRAM_POSITIVE); SignalP-noTM (SIGNALP_GRAM_NEGATIVE); SSF57667 (SUPERFAMILY); SSF57667 (SUPERFAMILY)
2029	299980_length_1876_cvg_21.2_tip_1_3	459	methylmalonate-semialdehyde dehydrogenase	626	0	87.60%	0.127	IPR010061 (TIGRFAM); IPR015590 (PFAM); IPR016163 (G3DSA:3.40.309.GENE3D); IPR016162 (G3DSA:3.40.605.GENE3D); IPR010061 (PTHR11699:PANTHER); PTHR11699 (PANTHER); IPR016160 (PROSITE_PATTERNS); TRANSMEMBRANE (PHOBIUS); NON_CYTOPLASMIC_DOMAIN (PHOBIUS); CYTOPLASMIC_DOMAIN (PHOBIUS); IPR016161 (SUPERFAMILY); TMhelix (TMHMM)

2030	299992_length_1877_cvg_54.7_tip_1_1	1707	ribosome-binding protein partial	626	2.10E-36	49.90%	0.098	Coil (COILS); Coil (COILS); Coil (COILS); Coil (COILS); Coil (COILS); Coil (COILS); Coil (COILS); Coil (COILS); Coil (COILS); PTHR18939:SF4 (PANTHER); PTHR18939 (PANTHER)
2034	300056_length_1896_cvg_6.6_tip_1_1	190	sphingomyelin phosphodiesterase	632	3.21E-176	62.50%	0.616 Y	IPR011160 (PIRSF); IPR004843 (PFAM); IPR011001 (G3DSA:1.10.225.GENE3D); IPR029052 (G3DSA:3.60.21.GENE3D); PTHR10340:SF13 (PANTHER); PTHR10340 (PANTHER); SIGNAL_PEPTIDE_N_REGION (PHOBIUS); SIGNAL_PEPTIDE (PHOBIUS); SIGNAL_PEPTIDE_C_REGION (PHOBIUS); SIGNAL_PEPTIDE_H_REGION (PHOBIUS); NON_CYTOPLASMIC_DOMAIN (PHOBIUS); IPR008139 (PROSITE_PROFILES); SignalP-TM (SIGNALP_EUK); SignalP-TM (SIGNALP_GRAM_POSITIVE); IPR029052 (SUPERFAMILY); IPR011001 (SUPERFAMILY); TMhelix (TMHMM)
2035	300108_length_1908_cvg_7.7_tip_1_2	198	katanin p60 atpase-containing subunit a-like 1	636	0	82.00%	0.14	IPR003593 (SMART); IPR027417 (G3DSA:3.40.50.GENE3D); G3DSA:1.10.8.60 (GENE3D); IPR003959 (PFAM); IPR015415 (PFAM); PTHR23074 (PANTHER); PTHR23074:SF65 (PANTHER); IPR003960 (PROSITE_PATTERNS); IPR028596 (HAMAP); IPR027417 (SUPERFAMILY)
2036	300128_length_1911_cvg_12.8_tip_1_3	255	wd repeat-containing protein 36	637	0	69.70%	0.111	IPR001680 (SMART); IPR007319 (PFAM); IPR015943 (G3DSA:2.130.10.GENE3D); IPR001680 (PFAM); IPR015943 (G3DSA:2.130.10.GENE3D); PTHR13889 (PANTHER); PTHR13889:SF2 (PANTHER); IPR020719 (PROSITE_PATTERNS); IPR001680 (PROSITE_PROFILES); IPR001680 (PROSITE_PROFILES); IPR001680 (PROSITE_PROFILES); IPR017986 (PROSITE_PROFILES); IPR017986 (PROSITE_PROFILES); IPR011047 (SUPERFAMILY)
2037	300136_length_1912_cvg_21.3_tip_1_5	595	dorsal-ventral patterning protein tolloid	637	0	79.50%	0.177	Coil (COILS); IPR001506 (PRINTS); IPR006026 (SMART); IPR000859 (SMART); IPR001506 (PFAM); IPR000859 (PFAM); IPR000859 (G3DSA:2.60.120.GENE3D); IPR024079 (G3DSA:3.40.390.GENE3D); PTHR10127:SF304 (PANTHER); PTHR10127 (PANTHER); IPR000859 (PROSITE_PROFILES); IPR000859 (PROSITE_PROFILES); SSF55486 (SUPERFAMILY); IPR000859 (SUPERFAMILY)
2038	300146_length_1915_cvg_10.9_tip_1_5	286	inter-alpha-trypsin inhibitor heavy chain h3-like	638	4.59E-122	62.80%	0.105	Coil (COILS); IPR002035 (SMART); IPR013694 (SMART); IPR013694 (PFAM); IPR002035 (PFAM); IPR002035 (G3DSA:3.40.50.GENE3D); PTHR10338 (PANTHER); PTHR10338:SF108 (PANTHER); NON_CYTOPLASMIC_DOMAIN (PHOBIUS); TRANSMEMBRANE (PHOBIUS); CYTOPLASMIC_DOMAIN (PHOBIUS); IPR013694 (PROSITE_PROFILES); IPR002035 (PROSITE_PROFILES); IPR002035 (SUPERFAMILY); TMhelix (TMHMM)
2039	300150_length_1915_cvg_11.4_tip_1_1	257	low-density lipoprotein receptor-related protein 2	638	1.12E-172	70.10%	0.147	IPR000033 (SMART); IPR000033 (PFAM); IPR011042 (G3DSA:2.120.10.GENE3D); G3DSA:2.10.25.10 (GENE3D); PTHR10529 (PANTHER); IPR013032 (PROSITE_PATTERNS); IPR013032 (PROSITE_PATTERNS); NON_CYTOPLASMIC_DOMAIN (PHOBIUS); TRANSMEMBRANE (PHOBIUS); CYTOPLASMIC_DOMAIN (PHOBIUS); NON_CYTOPLASMIC_DOMAIN (PHOBIUS); TRANSMEMBRANE (PHOBIUS); TRANSMEMBRANE (PHOBIUS); CYTOPLASMIC_DOMAIN (PHOBIUS); IPR000033 (PROSITE_PROFILES); IPR000033 (PROSITE_PROFILES); IPR000033 (PROSITE_PROFILES); IPR000033 (PROSITE_PROFILES); IPR000033 (PROSITE_PROFILES); IPR000742 (PROSITE_PROFILES); SSF57196 (SUPERFAMILY); SSF63825 (SUPERFAMILY); TMhelix (TMHMM); TMhelix (TMHMM); TMhelix (TMHMM)
2040	300166_length_1918_cvg_10.2_tip_1_2	308	stabilin-1 isoform x1	639	2.90E-127	65.80%	0.204	IPR000742 (SMART); G3DSA:2.10.25.10 (GENE3D); G3DSA:2.10.25.10 (GENE3D); G3DSA:2.10.25.10 (GENE3D); IPR013032 (PFAM); IPR013032 (PROSITE_PATTERNS); IPR013032 (PROSITE_PATTERNS); IPR013032 (PROSITE_PATTERNS); IPR013032 (PROSITE_PATTERNS); IPR013032 (PROSITE_PATTERNS); IPR013032 (PROSITE_PATTERNS); IPR013032 (PROSITE_PATTERNS); IPR013032 (PROSITE_PATTERNS); NON_CYTOPLASMIC_DOMAIN (PHOBIUS); CYTOPLASMIC_DOMAIN (PHOBIUS); TRANSMEMBRANE (PHOBIUS); IPR000742 (PROSITE_PROFILES); IPR000742 (PROSITE_PROFILES); IPR000742 (PROSITE_PROFILES); IPR000742 (PROSITE_PROFILES); IPR000742 (PROSITE_PROFILES); IPR000742 (PROSITE_PROFILES); IPR000742 (PROSITE_PROFILES); IPR000742 (PROSITE_PROFILES); TMhelix (TMHMM)
2041	300172_length_1919_cvg_5.4_tip_1_2	169	aael001570- partial	639	1.02E-168	55.20%	0.098	IPR005018 (SMART); IPR005018 (PFAM); PTHR22979:SF15 (PANTHER); PTHR22979 (PANTHER); IPR013032 (PROSITE_PATTERNS); IPR013032 (PROSITE_PATTERNS); IPR013032 (PROSITE_PATTERNS); IPR005018 (PROSITE_PROFILES); IPR005018 (PROSITE_PROFILES); IPR000742 (PROSITE_PROFILES); IPR000742 (PROSITE_PROFILES)

2042	300188_length_1924_cvg_61.8_tip_0_0	2791	twitchin isoform x4	642	0	87.40%	0.098	IPR002290 (SMART); IPR003598 (SMART); IPR003599 (SMART); G3DSA:3.30.200.20 (GENE3D); IPR013783 (G3DSA:2.60.40.GENE3D); IPR013783 (G3DSA:2.60.40.GENE3D); IPR013098 (PFAM); IPR000719 (PFAM); G3DSA:1.10.510.10 (GENE3D); PTHR19897 (PANTHER); PTHR19897:SF155 (PANTHER); IPR017441 (PROSITE_PATTERNS); IPR008271 (PROSITE_PATTERNS); IPR007110 (PROSITE_PROFILES); IPR007110 (PROSITE_PROFILES); IPR000719 (PROSITE_PROFILES); SSF48726 (SUPERFAMILY); IPR011009 (SUPERFAMILY); SSF48726 (SUPERFAMILY)
2043	300210_length_1931_cvg_17.2_tip_1_5	301	harmonin isoform x2	643	7.57E-52	55.80%	0.12	Coil (COILS); Coil (COILS); IPR001478 (SMART); IPR001478 (PFAM); IPR001478 (G3DSA:2.30.42.GENE3D); IPR001478 (G3DSA:2.30.42.GENE3D); PTHR23116 (PANTHER); IPR030237 (PTHR23116:PANTHER); IPR001478 (PROSITE_PROFILES); IPR001478 (PROSITE_PROFILES); IPR001478 (SUPERFAMILY); IPR001478 (SUPERFAMILY)
2044	300224_length_1935_cvg_6.0_tip_1_2	139	tolloid-like protein 2 isoform x2	645	0	82.60%	0.099	IPR000742 (SMART); IPR000859 (SMART); IPR001881 (SMART); G3DSA:2.10.25.10 (GENE3D); IPR001881 (PFAM); IPR000859 (G3DSA:2.60.120.GENE3D); PF14670 (PFAM); G3DSA:2.10.25.10 (GENE3D); IPR000859 (PFAM); IPR000859 (G3DSA:2.60.120.GENE3D); PTHR10127:SF304 (PANTHER); PTHR10127 (PANTHER); IPR000152 (PROSITE_PATTERNS); IPR000152 (PROSITE_PATTERNS); IPR013032 (PROSITE_PATTERNS); IPR018097 (PROSITE_PATTERNS); IPR013032 (PROSITE_PATTERNS); IPR018097 (PROSITE_PATTERNS); IPR000859 (PROSITE_PROFILES); IPR000742 (PROSITE_PROFILES); IPR000859 (PROSITE_PROFILES); IPR000742 (PROSITE_PROFILES); IPR000859 (PROSITE_PROFILES); IPR000859 (PROSITE_PROFILES); IPR000859 (SUPERFAMILY); IPR000859 (SUPERFAMILY); IPR000859 (SUPERFAMILY); IPR000859 (SUPERFAMILY); SSF57196 (SUPERFAMILY); SSF57196 (SUPERFAMILY)
2045	300230_length_1937_cvg_14.6_tip_1_3	438	leucine-rich repeat-containing protein partial	646	2.26E-51	47.30%	0.255	IPR000483 (SMART); IPR003591 (SMART); G3DSA:3.80.10.10 (GENE3D); IPR026906 (PFAM); G3DSA:3.80.10.10 (GENE3D); IPR001611 (PFAM); PTHR24373 (PANTHER); PTHR24373:SF100 (PANTHER); NON_CYTOPLASMIC_DOMAIN (PHOBIUS); NON_CYTOPLASMIC_DOMAIN (PHOBIUS); TRANSMEMBRANE (PHOBIUS); TRANSMEMBRANE (PHOBIUS); NON_CYTOPLASMIC_DOMAIN (PHOBIUS); CYTOPLASMIC_DOMAIN (PHOBIUS); TRANSMEMBRANE (PHOBIUS); CYTOPLASMIC_DOMAIN (PHOBIUS); TRANSMEMBRANE (PHOBIUS); IPR001611 (PROSITE_PROFILES); IPR001611 (PROSITE_PROFILES); IPR001611 (PROSITE_PROFILES); IPR001611 (PROSITE_PROFILES); IPR001611 (PROSITE_PROFILES); IPR001611 (PROSITE_PROFILES); IPR001611 (PROSITE_PROFILES); IPR001611 (PROSITE_PROFILES); SSF52058 (SUPERFAMILY); TMhelix (TMHMM); TMhelix (TMHMM); TMhelix (TMHMM); TMhelix (TMHMM)
2050	300352_length_1973_cvg_17.2_tip_1_5	422	transducin beta-like protein 2	648	1.16E-149	69.20%	0.161	Coil (COILS); IPR001680 (SMART); IPR015943 (G3DSA:2.130.10.GENE3D); IPR001680 (PFAM); PTHR22847:SF380 (PANTHER); PTHR22847 (PANTHER); IPR019775 (PROSITE_PATTERNS); TRANSMEMBRANE (PHOBIUS); NON_CYTOPLASMIC_DOMAIN (PHOBIUS); CYTOPLASMIC_DOMAIN (PHOBIUS); TRANSMEMBRANE (PHOBIUS); NON_CYTOPLASMIC_DOMAIN (PHOBIUS); IPR017986 (PROSITE_PROFILES); IPR001680 (PROSITE_PROFILES); IPR001680 (PROSITE_PROFILES); IPR017986 (SUPERFAMILY); TMhelix (TMHMM)
2046	300260_length_1949_cvg_21.6_tip_1_3	486	mucin- partial	650	2.14E-13	40.90%	0.148	Coil (COILS); Coil (COILS); G3DSA:2.10.70.10 (GENE3D); IPR001846 (PFAM); IPR014853 (PFAM); PTHR11339 (PANTHER); CYTOPLASMIC_DOMAIN (PHOBIUS); NON_CYTOPLASMIC_DOMAIN (PHOBIUS); NON_CYTOPLASMIC_DOMAIN (PHOBIUS); TRANSMEMBRANE (PHOBIUS); TRANSMEMBRANE (PHOBIUS); IPR000436 (PROSITE_PROFILES); IPR000436 (PROSITE_PROFILES); IPR001846 (PROSITE_PROFILES); IPR000436 (SUPERFAMILY); TMhelix (TMHMM)
2047	300284_length_1957_cvg_22.8_tip_1_4	486	methyltransferase-like protein 23	652	4.11E-93	76.80%	0.11	IPR000582 (PRINTS); IPR002110 (SMART); IPR000504 (SMART); IPR019410 (PFAM); IPR014352 (G3DSA:1.20.80.GENE3D); IPR020683 (G3DSA:1.25.40.GENE3D); IPR000582 (PFAM); IPR029063 (G3DSA:3.40.50.GENE3D); IPR000504 (PFAM); IPR012677 (G3DSA:3.30.70.GENE3D); IPR020683 (PFAM); PTHR24119 (PANTHER); PTHR24119:SF0 (PANTHER); IPR020683 (PROSITE_PROFILES); IPR002110 (PROSITE_PROFILES); IPR000504 (PROSITE_PROFILES); IPR002110 (PROSITE_PROFILES); IPR000582 (PROSITE_PROFILES); IPR029063 (SUPERFAMILY); IPR020683 (SUPERFAMILY); IPR000582 (SUPERFAMILY); SSF54928 (SUPERFAMILY)

2048	300298_length_1959_cvg_4.9_tip_1_0	147	membrane metallo-endopeptidase-like 1-like	653	0	78.40%	0.117	IPR018497 (PRINTS); IPR018497 (PFAM); IPR024079 (G3DSA:3.40.390.GENE3D); IPR008753 (PFAM); IPR000718 (PANTHER); PTHR11733:SF113 (PANTHER); SSF55486 (SUPERFAMILY)
2049	300318_length_1963_cvg_16.0_tip_1_4	381	coatomer subunit beta	654	0	89.00%	0.104	IPR001680 (SMART); IPR006692 (PFAM); IPR015943 (G3DSA:2.130.10.GENE3D); IPR001680 (PFAM); PTHR19876:SF2 (PANTHER); PTHR19876 (PANTHER); IPR001680 (PROSITE_PROFILES); IPR001680 (PROSITE_PROFILES); IPR017986 (PROSITE_PROFILES); IPR011044 (SUPERFAMILY); IPR017986 (SUPERFAMILY)
2051	300364_length_1975_cvg_13.0_tip_1_4	479	sphingomyelin phosphodiesterase	658	1.48E-177	62.80%	0.221	IPR008139 (SMART); IPR011160 (PIRSF); IPR004843 (PFAM); IPR007856 (PFAM); IPR029052 (G3DSA:3.60.21.GENE3D); IPR011001 (G3DSA:1.10.225.GENE3D); PTHR10340:SF13 (PANTHER); PTHR10340 (PANTHER); CYTOPLASMIC_DOMAIN (PHOBIUS); TRANSMEMBRANE (PHOBIUS); NON_CYTOPLASMIC_DOMAIN (PHOBIUS); IPR008139 (PROSITE_PROFILES); IPR011001 (SUPERFAMILY); IPR029052 (SUPERFAMILY); TMhelix (TMHMM)
2052	300386_length_1981_cvg_6.6_tip_1_3	136	protein phosphatase 1 regulatory subunit 7	661	9.45E-118	77.00%	0.112	SM00365 (SMART); IPR003591 (SMART); IPR003603 (SMART); IPR025875 (PFAM); PF14580 (PFAM); G3DSA:3.80.10.10 (GENE3D); PTHR10588 (PANTHER); IPR001611 (PROSITE_PROFILES); IPR001611 (PROSITE_PROFILES); IPR001611 (PROSITE_PROFILES); IPR001611 (PROSITE_PROFILES); IPR001611 (PROSITE_PROFILES); IPR001611 (PROSITE_PROFILES); IPR001611 (PROSITE_PROFILES); IPR001611 (PROSITE_PROFILES); IPR001611 (PROSITE_PROFILES); IPR001611 (PROSITE_PROFILES); IPR001611 (PROSITE_PROFILES); SSF52058 (SUPERFAMILY)
2053	300388_length_1981_cvg_11.0_tip_1_0	263	tyrosine--trna cytoplasmic	661	0	80.60%	0.134	IPR002307 (PRINTS); IPR002305 (PFAM); IPR014729 (G3DSA:3.40.50.GENE3D); G3DSA:1.10.240.10 (GENE3D); IPR002547 (PFAM); IPR002307 (TIGRFAM); IPR012340 (G3DSA:2.40.50.GENE3D); PTHR11946 (PANTHER); IPR002547 (PROSITE_PROFILES); SSF52374 (SUPERFAMILY); IPR010987 (SUPERFAMILY); IPR012340 (SUPERFAMILY)
2054	300442_length_1996_cvg_10.8_tip_1_4	267	chorion peroxidase-like	665	2.92E-152	68.20%	0.112	IPR019791 (PRINTS); IPR019791 (PFAM); IPR019791 (G3DSA:1.10.640.GENE3D); PTHR11475 (PANTHER); IPR019791 (PROSITE_PROFILES); IPR010255 (SUPERFAMILY)
2055	300452_length_2001_cvg_61.1_tip_1_4	3823	glycosyl-phosphatidylinositol-linked carbonic anhydrase	667	1.66E-67	57.90%	0.101	IPR001148 (SMART); IPR001148 (G3DSA:3.10.200.GENE3D); IPR001148 (PFAM); IPR023561 (PANTHER); IPR018338 (PROSITE_PATTERNS); TRANSMEMBRANE (PHOBIUS); CYTOPLASMIC_DOMAIN (PHOBIUS); CYTOPLASMIC_DOMAIN (PHOBIUS); TRANSMEMBRANE (PHOBIUS); NON_CYTOPLASMIC_DOMAIN (PHOBIUS); IPR001148 (PROSITE_PROFILES); IPR001148 (SUPERFAMILY)
2056	300454_length_2001_cvg_72.3_tip_0_2	914092	hemocyanin subunit type 1 precursor	667	0	76.40%	0.096	IPR013788 (PRINTS); IPR005204 (PFAM); IPR008922 (G3DSA:1.10.1280.GENE3D); IPR005204 (G3DSA:1.20.1370.GENE3D); IPR005203 (G3DSA:2.60.40.GENE3D); IPR000896 (PFAM); IPR005203 (PFAM); IPR013788 (PANTHER); IPR013788 (PROSITE_PATTERNS); IPR002227 (PROSITE_PATTERNS); IPR013788 (PROSITE_PATTERNS); IPR014756 (SUPERFAMILY); IPR008922 (SUPERFAMILY); IPR005204 (SUPERFAMILY)
2057	300464_length_2004_cvg_10.3_tip_1_2	265	scabrous protein	668	8.14E-119	55.30%	0.102	Coil (COILS); Coil (COILS); Coil (COILS); Coil (COILS); Coil (COILS); IPR002181 (SMART); IPR014715 (G3DSA:4.10.530.GENE3D); IPR014716 (G3DSA:3.90.215.GENE3D); IPR002181 (PFAM); PTHR19143 (PANTHER); PTHR19143:SF186 (PANTHER); IPR002181 (PROSITE_PROFILES); IPR002181 (SUPERFAMILY)
2058	300468_length_2004_cvg_9.9_tip_1_3	264	btb poz domain-containing protein 17	668	0	75.30%	0.138	IPR000210 (SMART); IPR011705 (SMART); IPR011705 (PFAM); IPR013069 (PFAM); G3DSA:3.30.710.10 (GENE3D); PTHR24410:SF9 (PANTHER); PTHR24410 (PANTHER); TRANSMEMBRANE (PHOBIUS); NON_CYTOPLASMIC_DOMAIN (PHOBIUS); CYTOPLASMIC_DOMAIN (PHOBIUS); IPR000210 (PROSITE_PROFILES); IPR011333 (SUPERFAMILY); TMhelix (TMHMM)
2059	300494_length_2010_cvg_8.8_tip_1_3	203	nucleolar protein 58-like	670	1.34E-171	83.50%	0.153	IPR012976 (SMART); IPR002687 (PFAM); IPR012976 (PFAM); PTHR10894:SF1 (PANTHER); PTHR10894 (PANTHER); IPR002687 (PROSITE_PROFILES); SSF89124 (SUPERFAMILY)
2060	300496_length_2010_cvg_5.0_tip_1_4	123	low quality protein: hemocytin-like	670	2.14E-66	43.30%	0.103	IPR001846 (SMART); G3DSA:2.10.25.10 (GENE3D); IPR001846 (PFAM); IPR014853 (PFAM); IPR002919 (PFAM); PTHR11339:SF25 (PANTHER); PTHR11339 (PANTHER); IPR001846 (PROSITE_PROFILES); IPR001846 (PROSITE_PROFILES); IPR002919 (SUPERFAMILY); IPR002919 (SUPERFAMILY)
2061	300502_length_2011_cvg_9.4_tip_1_3	290	serine threonine-protein phosphatase 2b catalytic subunit 2-like isoform x1	671	0	95.00%	0.108	IPR006186 (PRINTS); IPR006186 (SMART); IPR004843 (PFAM); IPR029052 (G3DSA:3.60.21.GENE3D); PTHR11668:SF186 (PANTHER); PTHR11668 (PANTHER); IPR006186 (PROSITE_PATTERNS); IPR029052 (SUPERFAMILY)

2062	300504_length_2011_cvg_5.2_tip_1_3	160	phosphatidylinositol -bisphosphate 5-phosphatase a-like	671	4.18E-19	55.50%	0.111	IPR029334 (PFAM)
2063	300506_length_2011_cvg_54.7_tip_1_3	2702	inactive pancreatic lipase-related protein 1	671	1.20E-96	57.30%	0.133	IPR002331 (PRINTS); IPR000734 (PRINTS); IPR001024 (G3DSA:2.60.60.GENE3D); IPR013818 (PFAM); IPR029058 (G3DSA:3.40.50.GENE3D); IPR000734 (PANTHER); IPR001024 (PROSITE_PROFILES); IPR029058 (SUPERFAMILY); IPR008976 (SUPERFAMILY)
2064	300522_length_2017_cvg_51.3_tip_1_0	2078	adp-ribosylation factor 1	673	3.10E-127	98.50%	0.253	IPR006689 (PRINTS); IPR006687 (SMART); IPR003579 (SMART); IPR024156 (SMART); IPR027417 (G3DSA:3.40.50.GENE3D); IPR005225 (TIGRFAM); IPR006689 (PFAM); PTHR11711 (PANTHER); PTHR11711:SF118 (PANTHER); NON_CYTOPLASMIC_DOMAIN (PHOBIUS); CYTOPLASMIC_DOMAIN (PHOBIUS); NON_CYTOPLASMIC_DOMAIN (PHOBIUS); TRANSMEMBRANE (PHOBIUS); TRANSMEMBRANE (PHOBIUS); NON_CYTOPLASMIC_DOMAIN (PHOBIUS); TRANSMEMBRANE (PHOBIUS); CYTOPLASMIC_DOMAIN (PHOBIUS); TRANSMEMBRANE (PHOBIUS); CYTOPLASMIC_DOMAIN (PHOBIUS); NON_CYTOPLASMIC_DOMAIN (PHOBIUS); TRANSMEMBRANE (PHOBIUS); TRANSMEMBRANE (PHOBIUS); TRANSMEMBRANE (PHOBIUS); CYTOPLASMIC_DOMAIN (PHOBIUS); IPR024156 (PROSITE_PROFILES); IPR027417 (SUPERFAMILY); TMhelix (TMHMM); TMhelix (TMHMM); TMhelix (TMHMM); TMhelix (TMHMM); TMhelix (TMHMM)
2065	300532_length_2020_cvg_4.2_tip_1_1	166	twitchin isoform x4	673	0	83.90%	0.113	PR00014 (PRINTS); IPR003961 (SMART); IPR003599 (SMART); IPR013783 (G3DSA:2.60.40.GENE3D); IPR013098 (PFAM); IPR013783 (G3DSA:2.60.40.GENE3D); IPR013783 (G3DSA:2.60.40.GENE3D); IPR013783 (G3DSA:2.60.40.GENE3D); IPR013783 (G3DSA:2.60.40.GENE3D); IPR003961 (PFAM); PTHR19897 (PANTHER); PTHR19897:SF155 (PANTHER); IPR003961 (PROSITE_PROFILES); IPR003961 (PROSITE_PROFILES); IPR003961 (PROSITE_PROFILES); IPR003961 (PROSITE_PROFILES); IPR003961 (PROSITE_PROFILES); IPR007110 (PROSITE_PROFILES); SSF48726 (SUPERFAMILY); IPR003961 (SUPERFAMILY); IPR003961 (SUPERFAMILY); IPR003961 (SUPERFAMILY); SSF48726 (SUPERFAMILY); SSF48726 (SUPERFAMILY)
2066	300538_length_2022_cvg_14.3_tip_1_2	313	low quality protein: low-density lipoprotein receptor-related protein 2-like	674	1.46E-25	50.00%	0.11	IPR002172 (PRINTS); IPR002172 (SMART); IPR002172 (G3DSA:4.10.400.GENE3D); IPR002172 (G3DSA:4.10.400.GENE3D); IPR002172 (PFAM); PTHR10529 (PANTHER); IPR002172 (PROSITE_PROFILES); IPR002172 (PROSITE_PROFILES); IPR002172 (PROSITE_PROFILES); IPR002172 (SUPERFAMILY); IPR002172 (SUPERFAMILY); IPR002172 (SUPERFAMILY)
2067	300542_length_2023_cvg_3.2_tip_1_5	97	heme utilization protein	674	1.67E-20	46.80%	0.123	no IPS match
2068	300560_length_2030_cvg_7.7_tip_1_1	231	uncharacterized threonine-rich gpi-anchored glyco isoform x5	677	0	90.60%	0.271	Coil (COILS); IPR002290 (SMART); IPR000719 (PFAM); G3DSA:1.10.510.10 (GENE3D); G3DSA:3.30.200.20 (GENE3D); PTHR13902 (PANTHER); IPR008271 (PROSITE_PATTERNS); IPR000719 (PROSITE_PROFILES); IPR011009 (SUPERFAMILY)
2069	300572_length_2032_cvg_26.3_tip_1_2	613	angiotensin-converting enzyme 2	677	2.92E-92	71.70%	0.128	IPR001548 (PRINTS); IPR001548 (PFAM); IPR001548 (PANTHER); NON_CYTOPLASMIC_DOMAIN (PHOBIUS); TRANSMEMBRANE (PHOBIUS); NON_CYTOPLASMIC_DOMAIN (PHOBIUS); CYTOPLASMIC_DOMAIN (PHOBIUS); NON_CYTOPLASMIC_DOMAIN (PHOBIUS); TRANSMEMBRANE (PHOBIUS); CYTOPLASMIC_DOMAIN (PHOBIUS); TRANSMEMBRANE (PHOBIUS); TRANSMEMBRANE (PHOBIUS); SSF55486 (SUPERFAMILY); TMhelix (TMHMM); TMhelix (TMHMM)
2070	300590_length_2039_cvg_35.4_tip_1_0	800	macrophage mannose receptor 1	680	2.37E-81	51.60%	0.105	IPR001304 (SMART); IPR016186 (G3DSA:3.10.100.GENE3D); IPR001304 (PFAM); IPR016186 (G3DSA:3.10.100.GENE3D); PTHR22803:SF16 (PANTHER); PTHR22803 (PANTHER); IPR018378 (PROSITE_PATTERNS); IPR018378 (PROSITE_PATTERNS); NON_CYTOPLASMIC_DOMAIN (PHOBIUS); CYTOPLASMIC_DOMAIN (PHOBIUS); TRANSMEMBRANE (PHOBIUS); IPR001304 (PROSITE_PROFILES); IPR001304 (PROSITE_PROFILES); IPR001304 (PROSITE_PROFILES); IPR016187 (SUPERFAMILY); IPR016187 (SUPERFAMILY); IPR016187 (SUPERFAMILY); TMhelix (TMHMM)

2071	300592_length_2040_cvg_20.6_tip_1_2	571	slit protein	680	6.92E-94	69.80%	0.112		IPR003591 (SMART); SM00365 (SMART); IPR001611 (PFAM); G3DSA:3.80.10.10 (GENE3D); PTHR24373 (PANTHER); PTHR24373:SF98 (PANTHER); NON_CYTOPLASMIC_DOMAIN (PHOBIUS); CYTOPLASMIC_DOMAIN (PHOBIUS); TRANSMEMBRANE (PHOBIUS); CYTOPLASMIC_DOMAIN (PHOBIUS); NON_CYTOPLASMIC_DOMAIN (PHOBIUS); NON_CYTOPLASMIC_DOMAIN (PHOBIUS); TRANSMEMBRANE (PHOBIUS); TRANSMEMBRANE (PHOBIUS); CYTOPLASMIC_DOMAIN (PHOBIUS); TRANSMEMBRANE (PHOBIUS); TRANSMEMBRANE (PHOBIUS); TRANSMEMBRANE (PHOBIUS); CYTOPLASMIC_DOMAIN (PHOBIUS); IPR001611 (PROSITE_PROFILES); IPR001611 (PROSITE_PROFILES); IPR001611 (PROSITE_PROFILES); IPR001611 (PROSITE_PROFILES); IPR001611 (PROSITE_PROFILES); IPR001611 (PROSITE_PROFILES); IPR001611 (PROSITE_PROFILES); IPR001611 (PROSITE_PROFILES); IPR001611 (PROSITE_PROFILES); SSF52058 (SUPERFAMILY); TMhelix (TMHMM); TMhelix (TMHMM); TMhelix (TMHMM); TMhelix (TMHMM); TMhelix (TMHMM)
2072	300608_length_2046_cvg_36.7_tip_1_2	943	n-acetylgalactosamine-6-sulfatase isoform x1	682	0	77.60%	0.097		G3DSA:3.30.1120.10 (GENE3D); IPR017849 (G3DSA:3.40.720.GENE3D); PF14707 (PFAM); IPR000917 (PFAM); PTHR10342:SF27 (PANTHER); PTHR10342 (PANTHER); IPR024607 (PROSITE_PATTERNS); IPR017850 (SUPERFAMILY)
2073	300620_length_2048_cvg_6.7_tip_1_0	257	peptidyl-prolyl cis-trans mitochondrial	683	1.94E-141	56.80%	0.175		IPR001841 (SMART); IPR013083 (G3DSA:3.30.40.GENE3D); IPR002130 (PFAM); IPR029000 (G3DSA:2.40.100.GENE3D); IPR024936 (PANTHER); IPR017907 (PROSITE_PATTERNS); NON_CYTOPLASMIC_DOMAIN (PHOBIUS); CYTOPLASMIC_DOMAIN (PHOBIUS); TRANSMEMBRANE (PHOBIUS); IPR002130 (PROSITE_PROFILES); IPR001841 (PROSITE_PROFILES); IPR000315 (PROSITE_PROFILES); SSF57850 (SUPERFAMILY); IPR029000 (SUPERFAMILY); SSF57845 (SUPERFAMILY)
2074	300626_length_2049_cvg_45.0_tip_1_0	1505	moxd1 homolog 2	683	6.02E-127	56.20%	0.241		IPR000945 (PRINTS); IPR005018 (SMART); IPR014784 (G3DSA:2.60.120.GENE3D); PF03712 (PFAM); IPR005018 (PFAM); IPR000323 (G3DSA:2.60.120.GENE3D); IPR015920 (G3DSA:2.60.40.GENE3D); IPR000323 (PFAM); IPR000945 (PANTHER); PTHR10157:SF23 (PANTHER); TRANSMEMBRANE (PHOBIUS); CYTOPLASMIC_DOMAIN (PHOBIUS); NON_CYTOPLASMIC_DOMAIN (PHOBIUS); IPR005018 (PROSITE_PROFILES); IPR008977 (SUPERFAMILY); SSF49344 (SUPERFAMILY); IPR008977 (SUPERFAMILY); TMhelix (TMHMM)
2075	300654_length_2056_cvg_6.2_tip_1_1	189	muscle m-line assembly protein unc-89	685	0	67.50%	0.106		IPR003599 (SMART); IPR003598 (SMART); IPR013783 (G3DSA:2.60.40.GENE3D); IPR013783 (G3DSA:2.60.40.GENE3D); IPR013783 (G3DSA:2.60.40.GENE3D); IPR013783 (G3DSA:2.60.40.GENE3D); IPR013783 (G3DSA:2.60.40.GENE3D); IPR013098 (PFAM); IPR020675 (PANTHER); PTHR22964:SF45 (PANTHER); IPR007110 (PROSITE_PROFILES); IPR007110 (PROSITE_PROFILES); IPR007110 (PROSITE_PROFILES); IPR007110 (PROSITE_PROFILES); IPR007110 (PROSITE_PROFILES); SSF48726 (SUPERFAMILY); SSF48726 (SUPERFAMILY); SSF48726 (SUPERFAMILY); SSF48726 (SUPERFAMILY); SSF48726 (SUPERFAMILY); SSF48726 (SUPERFAMILY)
2076	300676_length_2063_cvg_10.8_tip_1_2	236	counting factor associated protein d-like	687	0	76.80%	0.288		IPR000668 (PRINTS); IPR000668 (SMART); IPR013201 (SMART); IPR000668 (PFAM); IPR013201 (PFAM); G3DSA:3.90.70.10 (GENE3D); PTHR12411:SF284 (PANTHER); IPR013128 (PANTHER); IPR025660 (PROSITE_PATTERNS); IPR000169 (PROSITE_PATTERNS); IPR025661 (PROSITE_PATTERNS); CYTOPLASMIC_DOMAIN (PHOBIUS); TRANSMEMBRANE (PHOBIUS); NON_CYTOPLASMIC_DOMAIN (PHOBIUS); SSF54001 (SUPERFAMILY); TMhelix (TMHMM)
2077	300684_length_2065_cvg_50.9_tip_1_4	1596	spermine oxidase	688	5.41E-117	51.70%	0.531	Y	G3DSA:3.50.50.60 (GENE3D); IPR011032 (G3DSA:3.90.180.GENE3D); G3DSA:3.90.660.10 (GENE3D); IPR002937 (PFAM); PTHR10742:SF255 (PANTHER); PTHR10742 (PANTHER); TRANSMEMBRANE (PHOBIUS); CYTOPLASMIC_DOMAIN (PHOBIUS); TRANSMEMBRANE (PHOBIUS); NON_CYTOPLASMIC_DOMAIN (PHOBIUS); NON_CYTOPLASMIC_DOMAIN (PHOBIUS); SignalP-noTM (SIGNALP_EUK); SSF51905 (SUPERFAMILY); SSF54373 (SUPERFAMILY); TMhelix (TMHMM)
2079	300690_length_2066_cvg_23.9_tip_1_2	603	prostaglandin g h synthase 2-like isoform x3	688	0	70.20%	0.383	Y	IPR019791 (PRINTS); G3DSA:2.10.25.10 (GENE3D); IPR019791 (G3DSA:1.10.640.GENE3D); IPR019791 (PFAM); PTHR11903 (PANTHER); PTHR11903:SF10 (PANTHER); TRANSMEMBRANE (PHOBIUS); NON_CYTOPLASMIC_DOMAIN (PHOBIUS); CYTOPLASMIC_DOMAIN (PHOBIUS); IPR000742 (PROSITE_PROFILES); IPR019791 (PROSITE_PROFILES); SignalP-TM (SIGNALP_GRAM_POSITIVE); SSF57196 (SUPERFAMILY); IPR010255 (SUPERFAMILY); TMhelix (TMHMM)

2078	300688_length_2066_cvg_6.1_tip_1_1	175	carboxypeptidase d	689	0	63.70%	0.111	IPR000834 (PRINTS); IPR000834 (SMART); G3DSA:3.40.630.10 (GENE3D); G3DSA:3.40.630.10 (GENE3D); IPR000834 (PFAM); IPR014766 (G3DSA:2.60.40.GENE3D); PF13620 (PFAM); IPR015567 (PTHR11532:PANTHER); PTHR11532 (PANTHER); IPR000834 (PROSITE_PATTERNS); IPR000834 (PROSITE_PATTERNS); IPR008969 (SUPERFAMILY); SSF53187 (SUPERFAMILY); IPR008969 (SUPERFAMILY)
2080	300710_length_2071_cvg_5.0_tip_1_1	144	latrophilin- partial	690	2.13E-95	53.90%	0.116	IPR000832 (PRINTS); IPR000203 (SMART); IPR000203 (PFAM); IPR000832 (PFAM); IPR022624 (PFAM); PTHR12011 (PANTHER); CYTOPLASMIC_DOMAIN (PHOBIUS); TRANSMEMBRANE (PHOBIUS); TRANSMEMBRANE (PHOBIUS); NON_CYTOPLASMIC_DOMAIN (PHOBIUS); CYTOPLASMIC_DOMAIN (PHOBIUS); CYTOPLASMIC_DOMAIN (PHOBIUS); TRANSMEMBRANE (PHOBIUS); NON_CYTOPLASMIC_DOMAIN (PHOBIUS); TRANSMEMBRANE (PHOBIUS); TRANSMEMBRANE (PHOBIUS); NON_CYTOPLASMIC_DOMAIN (PHOBIUS); TRANSMEMBRANE (PHOBIUS); TRANSMEMBRANE (PHOBIUS); NON_CYTOPLASMIC_DOMAIN (PHOBIUS); NON_CYTOPLASMIC_DOMAIN (PHOBIUS); TRANSMEMBRANE (PHOBIUS); CYTOPLASMIC_DOMAIN (PHOBIUS); IPR000203 (PROSITE_PROFILES); IPR017981 (PROSITE_PROFILES); TMhelix (TMHMM); TMhelix (TMHMM); TMhelix (TMHMM); TMhelix (TMHMM); TMhelix (TMHMM); TMhelix (TMHMM); TMhelix (TMHMM)
2081	300714_length_2073_cvg_6.3_tip_1_3	179	extracellular serine threonine protein kinase fam20c isoform x1	691	0	71.70%	0.102	IPR009581 (PFAM); IPR024869 (PANTHER); PTHR12450:SF8 (PANTHER); TRANSMEMBRANE (PHOBIUS); CYTOPLASMIC_DOMAIN (PHOBIUS); NON_CYTOPLASMIC_DOMAIN (PHOBIUS); TRANSMEMBRANE (PHOBIUS); NON_CYTOPLASMIC_DOMAIN (PHOBIUS); TMhelix (TMHMM); TMhelix (TMHMM)
2082	300722_length_2077_cvg_34.8_tip_1_4	846	probable cytochrome p450 49a1	692	2.58E-143	62.70%	0.153	IPR002401 (PRINTS); IPR001128 (PRINTS); IPR001128 (G3DSA:1.10.630.GENE3D); IPR001128 (PFAM); PTHR24305:SF49 (PANTHER); PTHR24305 (PANTHER); IPR017972 (PROSITE_PATTERNS); IPR001128 (SUPERFAMILY)
2083	300736_length_2087_cvg_13.0_tip_1_1	481	probable chitinase 3	696	2.20E-133	56.70%	0.118	IPR002557 (SMART); IPR011583 (SMART); IPR029070 (G3DSA:3.10.50.GENE3D); IPR002557 (PFAM); IPR013781 (G3DSA:3.20.20.GENE3D); IPR002557 (G3DSA:2.170.140.GENE3D); IPR001223 (PFAM); PTHR11177 (PANTHER); PTHR11177:SF112 (PANTHER); IPR001579 (PROSITE_PATTERNS); TRANSMEMBRANE (PHOBIUS); CYTOPLASMIC_DOMAIN (PHOBIUS); TRANSMEMBRANE (PHOBIUS); CYTOPLASMIC_DOMAIN (PHOBIUS); NON_CYTOPLASMIC_DOMAIN (PHOBIUS); NON_CYTOPLASMIC_DOMAIN (PHOBIUS); TRANSMEMBRANE (PHOBIUS); IPR002557 (PROSITE_PROFILES); IPR029070 (SUPERFAMILY); IPR017853 (SUPERFAMILY); IPR002557 (SUPERFAMILY); TMhelix (TMHMM); TMhelix (TMHMM)
2084	300772_length_2099_cvg_6.0_tip_1_2	159	retinol dehydrogenase 13-like	699	5.97E-88	66.70%	0.315	IPR002198 (PRINTS); IPR002347 (PRINTS); IPR002198 (PFAM); IPR016040 (G3DSA:3.40.50.GENE3D); PTHR24320 (PANTHER); IPR020904 (PROSITE_PATTERNS); SIGNAL_PEPTIDE_H_REGION (PHOBIUS); NON_CYTOPLASMIC_DOMAIN (PHOBIUS); TRANSMEMBRANE (PHOBIUS); SIGNAL_PEPTIDE_C_REGION (PHOBIUS); SIGNAL_PEPTIDE_N_REGION (PHOBIUS); CYTOPLASMIC_DOMAIN (PHOBIUS); SIGNAL_PEPTIDE (PHOBIUS); SSF51735 (SUPERFAMILY); TMhelix (TMHMM)
2085	300776_length_2101_cvg_17.6_tip_1_3	621	vitellogenin 2	701	4.66E-106	49.50%	0.576 Y	IPR001747 (SMART); IPR015816 (G3DSA:2.30.230.GENE3D); IPR001747 (PFAM); IPR011030 (G3DSA:1.25.10.GENE3D); PTHR23345 (PANTHER); PTHR23345:SF1 (PANTHER); SIGNAL_PEPTIDE_C_REGION (PHOBIUS); SIGNAL_PEPTIDE (PHOBIUS); SIGNAL_PEPTIDE_H_REGION (PHOBIUS); SIGNAL_PEPTIDE_N_REGION (PHOBIUS); NON_CYTOPLASMIC_DOMAIN (PHOBIUS); IPR001747 (PROSITE_PROFILES); SignalP-TM (SIGNALP_EUK); IPR015819 (SUPERFAMILY); IPR011030 (SUPERFAMILY); TMhelix (TMHMM)
2086	300778_length_2101_cvg_63.1_tip_1_0	3426	aldehyde dehydrogenase 2 family	701	0	83.00%	0.228	IPR016163 (G3DSA:3.40.309.GENE3D); IPR016162 (G3DSA:3.40.605.GENE3D); IPR015590 (PFAM); PTHR11699:SF145 (PANTHER); PTHR11699 (PANTHER); IPR016160 (PROSITE_PATTERNS); IPR029510 (PROSITE_PATTERNS); IPR016161 (SUPERFAMILY)

2087	300802_length_2110_cvg_31.9_tip_1_4	1821	---NA---	703			0.477 Y	IPR012674 (G3DSA:2.40.128.GENE3D); PTHR10612 (PANTHER); PTHR10612:SF7 (PANTHER); SIGNAL_PEPTIDE (PHOBIUS); TRANSMEMBRANE (PHOBIUS); NON_CYTOPLASMIC_DOMAIN (PHOBIUS); CYTOPLASMIC_DOMAIN (PHOBIUS); TRANSMEMBRANE (PHOBIUS); NON_CYTOPLASMIC_DOMAIN (PHOBIUS); SIGNAL_PEPTIDE_C_REGION (PHOBIUS); CYTOPLASMIC_DOMAIN (PHOBIUS); SIGNAL_PEPTIDE_H_REGION (PHOBIUS); TRANSMEMBRANE (PHOBIUS); SIGNAL_PEPTIDE_N_REGION (PHOBIUS); IPR011038 (SUPERFAMILY); TMhelix (TMHMM); TMhelix (TMHMM); TMhelix (TMHMM); TMhelix (TMHMM)
2088	300806_length_2113_cvg_10.0_tip_1_5	298	lamin dm0-like isoform x1	704	0	75.60%	0.312	Coil (COILS); Coil (COILS); Coil (COILS); Coil (COILS); Coil (COILS); IPR001322 (G3DSA:2.60.40.GENE3D); G3DSA:1.20.5.170 (GENE3D); IPR001664 (PFAM); IPR001322 (PFAM); IPR001664 (PANTHER); IPR027696 (PTHR23239:PANTHER); IPR018039 (PROSITE_PATTERNS); SignalP-TM (SIGNALP_GRAM_POSITIVE); SSF64593 (SUPERFAMILY); SSF64593 (SUPERFAMILY); IPR001322 (SUPERFAMILY)
2089	300814_length_2115_cvg_10.6_tip_1_0	253	laminin subunit alpha-like	705	2.79E-83	61.10%	0.111	IPR001791 (SMART); IPR001791 (PFAM); IPR013320 (G3DSA:2.60.120.GENE3D); IPR013320 (G3DSA:2.60.120.GENE3D); PTHR10574:SF39 (PANTHER); PTHR10574 (PANTHER); TRANSMEMBRANE (PHOBIUS); NON_CYTOPLASMIC_DOMAIN (PHOBIUS); TRANSMEMBRANE (PHOBIUS); TRANSMEMBRANE (PHOBIUS); CYTOPLASMIC_DOMAIN (PHOBIUS); NON_CYTOPLASMIC_DOMAIN (PHOBIUS); CYTOPLASMIC_DOMAIN (PHOBIUS); TRANSMEMBRANE (PHOBIUS); NON_CYTOPLASMIC_DOMAIN (PHOBIUS); CYTOPLASMIC_DOMAIN (PHOBIUS); TRANSMEMBRANE (PHOBIUS); NON_CYTOPLASMIC_DOMAIN (PHOBIUS); TRANSMEMBRANE (PHOBIUS); TRANSMEMBRANE (PHOBIUS); CYTOPLASMIC_DOMAIN (PHOBIUS); IPR001791 (PROSITE_PROFILES); IPR001791 (PROSITE_PROFILES); IPR013320 (SUPERFAMILY); IPR013320 (SUPERFAMILY); TMhelix (TMHMM); TMhelix (TMHMM); TMhelix (TMHMM); TMhelix (TMHMM); TMhelix (TMHMM); TMhelix (TMHMM)
2090	300832_length_2117_cvg_29.3_tip_1_3	907	catalase	706	0	84.60%	0.278	IPR018028 (PRINTS); IPR011614 (SMART); IPR011614 (G3DSA:2.40.180.GENE3D); IPR011614 (PFAM); IPR010582 (PFAM); IPR018028 (PANTHER); IPR002226 (PROSITE_PATTERNS); IPR024708 (PROSITE_PATTERNS); IPR018028 (PROSITE_PROFILES); IPR020835 (SUPERFAMILY)
2091	300846_length_2122_cvg_47.4_tip_1_4	2745	von willebrand factor type egf and pentraxin domain-containing protein 1-like	707	9.85E-14	39.70%	0.707 Y	IPR000436 (SMART); IPR000436 (PFAM); G3DSA:2.10.70.10 (GENE3D); G3DSA:2.10.70.10 (GENE3D); G3DSA:2.10.70.10 (GENE3D); PTHR19325 (PANTHER); PTHR19325:SF321 (PANTHER); NON_CYTOPLASMIC_DOMAIN (PHOBIUS); TRANSMEMBRANE (PHOBIUS); CYTOPLASMIC_DOMAIN (PHOBIUS); IPR000436 (PROSITE_PROFILES); IPR000436 (PROSITE_PROFILES); IPR000436 (PROSITE_PROFILES); IPR000436 (PROSITE_PROFILES); SignalP-noTM (SIGNALP_EUK); IPR000436 (SUPERFAMILY); IPR000436 (SUPERFAMILY); TMhelix (TMHMM)
2092	300864_length_2132_cvg_8.7_tip_1_0	234	guanine nucleotide exchange	711	0	70.50%	0.102	Coil (COILS); IPR000219 (SMART); IPR001849 (SMART); IPR001452 (SMART); IPR000219 (PFAM); IPR001452 (PFAM); IPR000219 (G3DSA:1.20.900.GENE3D); G3DSA:2.30.30.40 (GENE3D); IPR011993 (G3DSA:2.30.29.GENE3D); PTHR12845 (PANTHER); PTHR12845:SF5 (PANTHER); IPR000219 (PROSITE_PROFILES); IPR001849 (PROSITE_PROFILES); IPR001452 (PROSITE_PROFILES); IPR001452 (SUPERFAMILY); IPR000219 (SUPERFAMILY); SSF50729 (SUPERFAMILY)
2093	300868_length_2134_cvg_14.0_tip_1_2	416	gels_homam ame: full= cytoplasmic ame: full=actin-depolymerizing factor short=adf	711	0	73.20%	0.138	IPR007122 (PRINTS); IPR007122 (SMART); IPR029006 (G3DSA:3.40.20.GENE3D); IPR029006 (G3DSA:3.40.20.GENE3D); IPR029006 (G3DSA:3.40.20.GENE3D); IPR007123 (PFAM); IPR007122 (PANTHER); SSF55753 (SUPERFAMILY); SSF55753 (SUPERFAMILY); SSF55753 (SUPERFAMILY); SSF55753 (SUPERFAMILY); SSF55753 (SUPERFAMILY)
2094	300870_length_2135_cvg_9.6_tip_1_2	302	angiotensin-converting enzyme-like	711	0	71.20%	0.507 Y	IPR001548 (PRINTS); IPR001548 (PFAM); IPR001548 (PANTHER); TRANSMEMBRANE (PHOBIUS); NON_CYTOPLASMIC_DOMAIN (PHOBIUS); CYTOPLASMIC_DOMAIN (PHOBIUS); SignalP-noTM (SIGNALP_EUK); SSF55486 (SUPERFAMILY)
2095	300878_length_2137_cvg_10.1_tip_1_4	291	cyclin-dependent kinase 12 isoform x1	712	0	82.00%	0.104	IPR002290 (SMART); IPR000719 (PFAM); G3DSA:1.10.510.10 (GENE3D); G3DSA:3.30.200.20 (GENE3D); PTHR24056:SF125 (PANTHER); PTHR24056 (PANTHER); IPR017441 (PROSITE_PATTERNS); IPR008271 (PROSITE_PATTERNS); IPR000719 (PROSITE_PROFILES); IPR011009 (SUPERFAMILY)
2096	300886_length_2142_cvg_9.5_tip_1_1	291	adenosine deaminase cecr1	714	2.68E-40	77.10%	0.114	G3DSA:3.20.20.140 (GENE3D); IPR001365 (PFAM); PTHR11409 (PANTHER); PTHR11409:SF39 (PANTHER); SSF51556 (SUPERFAMILY); TMhelix (TMHMM)

2097	300892_length_2143_cvg_9.7_tip_1_0	318	membrane metallo-endopeptidase-like 1-like	715	2.56E-163	62.10%	0.124	IPR018497 (PRINTS); IPR024079 (G3DSA:3.40.390.GENE3D); IPR008753 (PFAM); IPR018497 (PFAM); IPR000718 (PANTHER); SSF55486 (SUPERFAMILY)
2098	300896_length_2144_cvg_8.8_tip_1_0	259	early growth response	715	1.09E-120	54.80%	0.106	IPR015880 (SMART); IPR013087 (G3DSA:3.30.160.GENE3D); IPR013087 (G3DSA:3.30.160.GENE3D); IPR013087 (G3DSA:3.30.160.GENE3D); PF13465 (PFAM); PTHR10042:SF24 (PANTHER); PTHR10042 (PANTHER); IPR007087 (PROSITE_PATTERNS); IPR007087 (PROSITE_PATTERNS); IPR007087 (PROSITE_PATTERNS); CYTOPLASMIC_DOMAIN (PHOBIUS); NON_CYTOPLASMIC_DOMAIN (PHOBIUS); TRANSMEMBRANE (PHOBIUS); IPR007087 (PROSITE_PROFILES); IPR007087 (PROSITE_PROFILES); IPR007087 (PROSITE_PROFILES); SSF57667 (SUPERFAMILY); SSF57667 (SUPERFAMILY)
2099	300912_length_2149_cvg_12.4_tip_1_1	314	meprin a	716	5.34E-55	54.60%	0.112	IPR001506 (PRINTS); IPR006026 (SMART); IPR000859 (SMART); IPR024079 (G3DSA:3.40.390.GENE3D); IPR000859 (G3DSA:2.60.120.GENE3D); IPR000859 (G3DSA:2.60.120.GENE3D); IPR001506 (PFAM); IPR000859 (PFAM); PTHR10127 (PANTHER); IPR000859 (PROSITE_PROFILES); IPR000859 (SUPERFAMILY); SSF55486 (SUPERFAMILY); IPR000859 (SUPERFAMILY)
2100	300920_length_2152_cvg_9.0_tip_1_1	300	venom carboxylesterase-6 isoform x2	717	9.59E-138	58.20%	0.144	IPR002018 (PFAM); IPR029058 (G3DSA:3.40.50.GENE3D); PTHR11559 (PANTHER); IPR019819 (PROSITE_PATTERNS); IPR019826 (PROSITE_PATTERNS); CYTOPLASMIC_DOMAIN (PHOBIUS); TRANSMEMBRANE (PHOBIUS); TRANSMEMBRANE (PHOBIUS); NON_CYTOPLASMIC_DOMAIN (PHOBIUS); NON_CYTOPLASMIC_DOMAIN (PHOBIUS); IPR029058 (SUPERFAMILY)
2101	300926_length_2153_cvg_25.3_tip_1_5	690	chorion peroxidase-like	717	4.63E-166	59.50%	0.211	IPR019791 (PRINTS); IPR019791 (PFAM); IPR019791 (G3DSA:1.10.640.GENE3D); PTHR11475 (PANTHER); IPR019791 (PROSITE_PROFILES); IPR010255 (SUPERFAMILY)
2102	300936_length_2156_cvg_11.1_tip_1_5	231	mam and ldl-receptor class a domain-containing protein 1	718	2.40E-152	54.50%	0.152	IPR002172 (PRINTS); IPR000998 (SMART); IPR002172 (SMART); IPR002172 (G3DSA:4.10.400.GENE3D); IPR002172 (PFAM); IPR000998 (PFAM); IPR002172 (G3DSA:4.10.400.GENE3D); IPR002172 (G3DSA:4.10.400.GENE3D); PTHR23282 (PANTHER); IPR023415 (PROSITE_PATTERNS); IPR023415 (PROSITE_PATTERNS); IPR000998 (PROSITE_PROFILES); IPR000998 (PROSITE_PROFILES); IPR002172 (PROSITE_PROFILES); IPR002172 (PROSITE_PROFILES); IPR000998 (PROSITE_PROFILES); IPR002172 (PROSITE_PROFILES); IPR000998 (PROSITE_PROFILES); IPR002172 (SUPERFAMILY); IPR013320 (SUPERFAMILY); IPR002172 (SUPERFAMILY); IPR013320 (SUPERFAMILY); IPR013320 (SUPERFAMILY); IPR002172 (SUPERFAMILY); IPR013320 (SUPERFAMILY)
2103	300950_length_2165_cvg_27.8_tip_1_0	964	pancreatic lipase-related protein 1	722	4.88E-102	55.40%	0.146	IPR000734 (PRINTS); IPR029058 (G3DSA:3.40.50.GENE3D); IPR013818 (PFAM); PTHR11610:SF41 (PANTHER); IPR000734 (PANTHER); TRANSMEMBRANE (PHOBIUS); NON_CYTOPLASMIC_DOMAIN (PHOBIUS); CYTOPLASMIC_DOMAIN (PHOBIUS); IPR029058 (SUPERFAMILY)
2104	300952_length_2166_cvg_9.7_tip_1_1	259	glycoside catalytic core	722	5.33E-94	53.40%	0.282	Coil (COILS); IPR013781 (G3DSA:3.20.20.GENE3D); IPR001547 (PFAM); PTHR31308 (PANTHER); NON_CYTOPLASMIC_DOMAIN (PHOBIUS); TRANSMEMBRANE (PHOBIUS); TRANSMEMBRANE (PHOBIUS); CYTOPLASMIC_DOMAIN (PHOBIUS); NON_CYTOPLASMIC_DOMAIN (PHOBIUS); IPR017853 (SUPERFAMILY)
2105	300958_length_2172_cvg_14.7_tip_1_1	404	aspartate--trna cytoplasmic	724	0	85.30%	0.202	Coil (COILS); IPR002312 (PRINTS); IPR004364 (PFAM); IPR004523 (TIGRFAM); IPR004365 (PFAM); G3DSA:3.30.930.10 (GENE3D); IPR012340 (G3DSA:2.40.50.GENE3D); IPR018150 (PANTHER); IPR004523 (PTHR22594:PANTHER); TRANSMEMBRANE (PHOBIUS); TRANSMEMBRANE (PHOBIUS); CYTOPLASMIC_DOMAIN (PHOBIUS); CYTOPLASMIC_DOMAIN (PHOBIUS); TRANSMEMBRANE (PHOBIUS); NON_CYTOPLASMIC_DOMAIN (PHOBIUS); NON_CYTOPLASMIC_DOMAIN (PHOBIUS); IPR006195 (PROSITE_PROFILES); SSF55681 (SUPERFAMILY); IPR012340 (SUPERFAMILY)
2106	300970_length_2176_cvg_57.7_tip_1_2	3229	protein disulfide-isomerase a3	725	0	78.20%	0.361 Y	PR00421 (PRINTS); IPR005792 (TIGRFAM); IPR013766 (PFAM); IPR012336 (G3DSA:3.40.30.GENE3D); IPR012336 (G3DSA:3.40.30.GENE3D); IPR012336 (G3DSA:3.40.30.GENE3D); IPR005788 (TIGRFAM); PF13848 (PFAM); PTHR18929 (PANTHER); PTHR18929:SF60 (PANTHER); IPR017937 (PROSITE_PATTERNS); IPR017937 (PROSITE_PATTERNS); IPR012336 (PROSITE_PROFILES); IPR012336 (PROSITE_PROFILES); IPR012336 (SUPERFAMILY); IPR012336 (SUPERFAMILY); IPR012336 (SUPERFAMILY); IPR012336 (SUPERFAMILY)

2107	301014_length_2199_cvg_27.2_tip_1_4	725	latrophilin cirl-like isoform x1	733	6.32E-175	59.10%	0.232	Coil (COILS); IPR000832 (PRINTS); IPR000832 (PFAM); PTHR12011:SF258 (PANTHER); PTHR12011 (PANTHER); IPR017983 (PROSITE_PATTERNS); NON_CYTOPLASMIC_DOMAIN (PHOBIUS); TRANSMEMBRANE (PHOBIUS); CYTOPLASMIC_DOMAIN (PHOBIUS); TRANSMEMBRANE (PHOBIUS); TRANSMEMBRANE (PHOBIUS); TRANSMEMBRANE (PHOBIUS); NON_CYTOPLASMIC_DOMAIN (PHOBIUS); NON_CYTOPLASMIC_DOMAIN (PHOBIUS); CYTOPLASMIC_DOMAIN (PHOBIUS); TRANSMEMBRANE (PHOBIUS); CYTOPLASMIC_DOMAIN (PHOBIUS); IPR017981 (PROSITE_PROFILES); TMhelix (TMHMM); TMhelix (TMHMM); TMhelix (TMHMM); TMhelix (TMHMM); TMhelix (TMHMM)
2119	301180_length_2269_cvg_73.3_tip_1_2	18195	chitinase 1 precursor	735	0	69.50%	0.117	IPR011583 (SMART); IPR002557 (SMART); IPR002557 (G3DSA:2.170.140.GENE3D); IPR029070 (G3DSA:3.10.50.GENE3D); IPR001223 (PFAM); IPR013781 (G3DSA:3.20.20.GENE3D); IPR002557 (PFAM); PTHR11177:SF144 (PANTHER); PTHR11177 (PANTHER); NON_CYTOPLASMIC_DOMAIN (PHOBIUS); CYTOPLASMIC_DOMAIN (PHOBIUS); TRANSMEMBRANE (PHOBIUS); IPR002557 (PROSITE_PROFILES); IPR002557 (SUPERFAMILY); IPR017853 (SUPERFAMILY); IPR029070 (SUPERFAMILY)
2108	301068_length_2224_cvg_22.6_tip_1_2	680	lysine--trna ligase isoform x2	741	0	86.20%	0.108	Coil (COILS); IPR018149 (PRINTS); IPR004365 (PFAM); IPR002313 (TIGRFAM); IPR012340 (G3DSA:2.40.50.GENE3D); IPR004364 (PFAM); G3DSA:3.30.930.10 (GENE3D); PTHR22594:SF4 (PANTHER); IPR018150 (PANTHER); NON_CYTOPLASMIC_DOMAIN (PHOBIUS); TRANSMEMBRANE (PHOBIUS); TRANSMEMBRANE (PHOBIUS); NON_CYTOPLASMIC_DOMAIN (PHOBIUS); CYTOPLASMIC_DOMAIN (PHOBIUS); IPR006195 (PROSITE_PROFILES); IPR002313 (HAMAP); IPR012340 (SUPERFAMILY); SSF55681 (SUPERFAMILY); TMhelix (TMHMM); TMhelix (TMHMM)
2109	301072_length_2225_cvg_19.7_tip_1_2	631	delta -sterol reductase	741	0	78.60%	0.151	IPR006094 (PFAM); IPR016169 (G3DSA:3.30.465.GENE3D); PTHR10801 (PANTHER); CYTOPLASMIC_DOMAIN (PHOBIUS); NON_CYTOPLASMIC_DOMAIN (PHOBIUS); TRANSMEMBRANE (PHOBIUS); TRANSMEMBRANE (PHOBIUS); NON_CYTOPLASMIC_DOMAIN (PHOBIUS); CYTOPLASMIC_DOMAIN (PHOBIUS); NON_CYTOPLASMIC_DOMAIN (PHOBIUS); TRANSMEMBRANE (PHOBIUS); TRANSMEMBRANE (PHOBIUS); CYTOPLASMIC_DOMAIN (PHOBIUS); CYTOPLASMIC_DOMAIN (PHOBIUS); TRANSMEMBRANE (PHOBIUS); TRANSMEMBRANE (PHOBIUS); IPR016166 (PROSITE_PROFILES); IPR016164 (SUPERFAMILY); IPR016166 (SUPERFAMILY); TMhelix (TMHMM)
2110	301076_length_2228_cvg_47.9_tip_0_5	1944	plasma alpha-l-fucosidase	742	0	78.50%	0.102	IPR016286 (PRINTS); IPR000933 (SMART); IPR000933 (PFAM); IPR013780 (G3DSA:2.60.40.GENE3D); IPR013781 (G3DSA:3.20.20.GENE3D); PTHR10030:SF26 (PANTHER); IPR000933 (PANTHER); IPR018526 (PROSITE_PATTERNS); TRANSMEMBRANE (PHOBIUS); CYTOPLASMIC_DOMAIN (PHOBIUS); TRANSMEMBRANE (PHOBIUS); TRANSMEMBRANE (PHOBIUS); NON_CYTOPLASMIC_DOMAIN (PHOBIUS); CYTOPLASMIC_DOMAIN (PHOBIUS); NON_CYTOPLASMIC_DOMAIN (PHOBIUS); TRANSMEMBRANE (PHOBIUS); CYTOPLASMIC_DOMAIN (PHOBIUS); TRANSMEMBRANE (PHOBIUS); NON_CYTOPLASMIC_DOMAIN (PHOBIUS); IPR017853 (SUPERFAMILY)
2111	301078_length_2228_cvg_9.2_tip_1_2	368	PREDICTED: mucin-5AC-like	742	1.26E-07	52.25%	0.169	TRANSMEMBRANE (PHOBIUS); TRANSMEMBRANE (PHOBIUS); CYTOPLASMIC_DOMAIN (PHOBIUS); TRANSMEMBRANE (PHOBIUS); NON_CYTOPLASMIC_DOMAIN (PHOBIUS); NON_CYTOPLASMIC_DOMAIN (PHOBIUS); TRANSMEMBRANE (PHOBIUS); TRANSMEMBRANE (PHOBIUS); TRANSMEMBRANE (PHOBIUS); CYTOPLASMIC_DOMAIN (PHOBIUS); CYTOPLASMIC_DOMAIN (PHOBIUS); NON_CYTOPLASMIC_DOMAIN (PHOBIUS); CYTOPLASMIC_DOMAIN (PHOBIUS); TMhelix (TMHMM); TMhelix (TMHMM); TMhelix (TMHMM); TMhelix (TMHMM); TMhelix (TMHMM)
2112	301086_length_2233_cvg_3.8_tip_1_2	145	cd109 antigen	744	0	63.20%	0.187	IPR002172 (SMART); IPR002172 (G3DSA:4.10.400.GENE3D); IPR011626 (PFAM); IPR008930 (G3DSA:1.50.10.GENE3D); IPR001599 (PFAM); PTHR11412 (PANTHER); PTHR11412:SF85 (PANTHER); IPR002172 (PROSITE_PROFILES); IPR002172 (SUPERFAMILY); IPR008930 (SUPERFAMILY)
2113	301098_length_2237_cvg_9.9_tip_1_3	265	trehalase-like isoform x1	746	0	69.40%	0.128	IPR001661 (PRINTS); IPR001661 (PFAM); IPR001661 (PANTHER); PTHR23403:SF1 (PANTHER); IPR018232 (PROSITE_PATTERNS); IPR018232 (PROSITE_PATTERNS); CYTOPLASMIC_DOMAIN (PHOBIUS); TRANSMEMBRANE (PHOBIUS); NON_CYTOPLASMIC_DOMAIN (PHOBIUS); TRANSMEMBRANE (PHOBIUS); CYTOPLASMIC_DOMAIN (PHOBIUS); IPR008928 (SUPERFAMILY)

2114	301112_length_2240_cvg_35.3_tip_1_3	862	leucine rich repeat protein	747	6.95E-40	47.00%	0.119	SM00364 (SMART); SM00365 (SMART); IPR003591 (SMART); IPR001611 (PFAM); G3DSA:3.80.10.10 (GENE3D); G3DSA:3.80.10.10 (GENE3D); G3DSA:3.80.10.10 (GENE3D); IPR001611 (PFAM); PTHR24365 (PANTHER); IPR001611 (PROSITE_PROFILES); SSF52058 (SUPERFAMILY); SSF52058 (SUPERFAMILY)
2115	301134_length_2249_cvg_32.1_tip_1_2	1259	heat shock protein 70	749	0	90.60%	0.182	Coil (COILS); IPR013126 (PRINTS); G3DSA:3.30.420.40 (GENE3D); IPR029048 (G3DSA:1.20.1270.GENE3D); G3DSA:3.30.420.40 (GENE3D); G3DSA:3.30.30.30 (GENE3D); IPR013126 (PFAM); G3DSA:3.90.640.10 (GENE3D); IPR029047 (G3DSA:2.60.34.GENE3D); PTHR19375 (PANTHER); PTHR19375:SF142 (PANTHER); IPR018181 (PROSITE_PATTERNS); IPR018181 (PROSITE_PATTERNS); NON_CYTOPLASMIC_DOMAIN (PHOBIUS); CYTOPLASMIC_DOMAIN (PHOBIUS); TRANSMEMBRANE (PHOBIUS); IPR029047 (SUPERFAMILY); SSF53067 (SUPERFAMILY); SSF53067 (SUPERFAMILY); IPR029048 (SUPERFAMILY)
2116	301150_length_2253_cvg_12.5_tip_1_0	388	aquaporin 1	751	1.97E-103	72.70%	0.119	IPR000425 (PRINTS); IPR000425 (PFAM); IPR023271 (G3DSA:1.20.1080.GENE3D); IPR000425 (TIGRFAM); IPR000425 (PANTHER); CYTOPLASMIC_DOMAIN (PHOBIUS); CYTOPLASMIC_DOMAIN (PHOBIUS); TRANSMEMBRANE (PHOBIUS); TRANSMEMBRANE (PHOBIUS); NON_CYTOPLASMIC_DOMAIN (PHOBIUS); TRANSMEMBRANE (PHOBIUS); CYTOPLASMIC_DOMAIN (PHOBIUS); NON_CYTOPLASMIC_DOMAIN (PHOBIUS); TRANSMEMBRANE (PHOBIUS); NON_CYTOPLASMIC_DOMAIN (PHOBIUS); TRANSMEMBRANE (PHOBIUS); IPR023271 (SUPERFAMILY); TMhelix (TMHMM); TMhelix (TMHMM); TMhelix (TMHMM); TMhelix (TMHMM); TMhelix (TMHMM); TMhelix (TMHMM); TMhelix (TMHMM)
2117	301160_length_2261_cvg_6.5_tip_1_3	176	cytochrome p450 cyp44	754	0	65.70%	0.305	IPR002401 (PRINTS); IPR001128 (PRINTS); IPR001128 (PFAM); IPR001128 (G3DSA:1.10.630.GENE3D); PTHR24292:SF0 (PANTHER); PTHR24292 (PANTHER); IPR017972 (PROSITE_PATTERNS); IPR001128 (SUPERFAMILY)
2118	301176_length_2266_cvg_10.7_tip_1_5	217	exocyst complex component 3	755	0	75.40%	0.105	Coil (COILS); IPR010326 (PFAM); PTHR21292:SF1 (PANTHER); IPR010326 (PANTHER)
2120	301184_length_2270_cvg_60.8_tip_1_3	6611	calreticulin	757	0	84.20%	0.257	Coil (COILS); IPR001580 (PRINTS); IPR013320 (G3DSA:2.60.120.GENE3D); IPR001580 (PFAM); IPR001580 (PANTHER); PTHR11073:SF8 (PANTHER); IPR018124 (PROSITE_PATTERNS); IPR018124 (PROSITE_PATTERNS); IPR018124 (PROSITE_PATTERNS); IPR018124 (PROSITE_PATTERNS); IPR018124 (PROSITE_PATTERNS); TRANSMEMBRANE (PHOBIUS); NON_CYTOPLASMIC_DOMAIN (PHOBIUS); CYTOPLASMIC_DOMAIN (PHOBIUS); CYTOPLASMIC_DOMAIN (PHOBIUS); TRANSMEMBRANE (PHOBIUS); TRANSMEMBRANE (PHOBIUS); TRANSMEMBRANE (PHOBIUS); TRANSMEMBRANE (PHOBIUS); CYTOPLASMIC_DOMAIN (PHOBIUS); NON_CYTOPLASMIC_DOMAIN (PHOBIUS); NON_CYTOPLASMIC_DOMAIN (PHOBIUS); IPR013320 (SUPERFAMILY); IPR009033 (SUPERFAMILY); TMhelix (TMHMM); TMhelix (TMHMM); TMhelix (TMHMM); TMhelix (TMHMM)
2121	301192_length_2276_cvg_9.8_tip_1_2	258	mucosa-associated lymphoid tissue lymphoma translocation protein 1-like	758	5.53E-65	46.90%	0.118	IPR003599 (SMART); IPR015917 (SMART); IPR003598 (SMART); PF13895 (PFAM); IPR013098 (PFAM); IPR013783 (G3DSA:2.60.40.GENE3D); IPR013783 (G3DSA:2.60.40.GENE3D); IPR011600 (PFAM); IPR029030 (G3DSA:3.40.50.GENE3D); PTHR22576 (PANTHER); PTHR22576:SF20 (PANTHER); TRANSMEMBRANE (PHOBIUS); NON_CYTOPLASMIC_DOMAIN (PHOBIUS); CYTOPLASMIC_DOMAIN (PHOBIUS); IPR007110 (PROSITE_PROFILES); IPR001309 (PROSITE_PROFILES); IPR007110 (PROSITE_PROFILES); IPR007110 (PROSITE_PROFILES); SSF48726 (SUPERFAMILY); SSF48726 (SUPERFAMILY); SSF48726 (SUPERFAMILY); SSF52129 (SUPERFAMILY); TMhelix (TMHMM)

2122	301208_length_2285_cvg_27.5_tip_1_0	786	cytochrome p450	762	0	70.00%	0.33	Coil (COILS); IPR002401 (PRINTS); IPR001128 (PRINTS); IPR001128 (PFAM); IPR001128 (G3DSA:1.10.630.GENE3D); PTHR24290 (PANTHER); PTHR24290:SF0 (PANTHER); IPR017972 (PROSITE_PATTERNS); SIGNAL_PEPTIDE_N_REGION (PHOBIUS); TRANSMEMBRANE (PHOBIUS); NON_CYTOPLASMIC_DOMAIN (PHOBIUS); SIGNAL_PEPTIDE_C_REGION (PHOBIUS); SIGNAL_PEPTIDE (PHOBIUS); CYTOPLASMIC_DOMAIN (PHOBIUS); SIGNAL_PEPTIDE_H_REGION (PHOBIUS); IPR001128 (SUPERFAMILY)
2123	301226_length_2292_cvg_59.1_tip_1_0	4430	heat shock 70 kda protein cognate 4	764	0	94.40%	0.242	Coil (COILS); IPR013126 (PRINTS); IPR029048 (G3DSA:1.20.1270.GENE3D); IPR029047 (G3DSA:2.60.34.GENE3D); G3DSA:3.90.640.10 (GENE3D); G3DSA:3.30.420.40 (GENE3D); G3DSA:3.30.30.30 (GENE3D); IPR013126 (PFAM); G3DSA:3.30.420.40 (GENE3D); PTHR19375:SF142 (PANTHER); PTHR19375 (PANTHER); IPR018181 (PROSITE_PATTERNS); IPR018181 (PROSITE_PATTERNS); IPR018181 (PROSITE_PATTERNS); NON_CYTOPLASMIC_DOMAIN (PHOBIUS); TRANSMEMBRANE (PHOBIUS); CYTOPLASMIC_DOMAIN (PHOBIUS); NON_CYTOPLASMIC_DOMAIN (PHOBIUS); TRANSMEMBRANE (PHOBIUS); IPR029048 (SUPERFAMILY); SSF53067 (SUPERFAMILY); SSF53067 (SUPERFAMILY); IPR029047 (SUPERFAMILY); TMhelix (TMHMM); TMhelix (TMHMM)
2124	301230_length_2293_cvg_11.9_tip_1_3	377	arylsulfatase b	765	9.74E-173	64.80%	0.223	IPR017849 (G3DSA:3.40.720.GENE3D); G3DSA:3.30.1120.10 (GENE3D); IPR000917 (PFAM); PTHR10342:SF206 (PANTHER); PTHR10342 (PANTHER); IPR024607 (PROSITE_PATTERNS); IPR024607 (PROSITE_PATTERNS); NON_CYTOPLASMIC_DOMAIN (PHOBIUS); TRANSMEMBRANE (PHOBIUS); TRANSMEMBRANE (PHOBIUS); CYTOPLASMIC_DOMAIN (PHOBIUS); TRANSMEMBRANE (PHOBIUS); CYTOPLASMIC_DOMAIN (PHOBIUS); NON_CYTOPLASMIC_DOMAIN (PHOBIUS); IPR017850 (SUPERFAMILY); TMhelix (TMHMM)
2125	301238_length_2297_cvg_18.9_tip_1_3	536	utp--glucose-1-phosphate uridylyltransferase-like	766	0	84.40%	0.11	IPR002618 (PFAM); IPR029044 (G3DSA:3.90.550.GENE3D); IPR002618 (PANTHER); IPR016267 (PTHR11952:PANTHER); TRANSMEMBRANE (PHOBIUS); CYTOPLASMIC_DOMAIN (PHOBIUS); NON_CYTOPLASMIC_DOMAIN (PHOBIUS); TRANSMEMBRANE (PHOBIUS); NON_CYTOPLASMIC_DOMAIN (PHOBIUS); IPR029044 (SUPERFAMILY); TMhelix (TMHMM); TMhelix (TMHMM)
2126	301244_length_2298_cvg_6.9_tip_1_5	205	phospholipase a-2-activating protein	766	0	66.50%	0.119	IPR001680 (SMART); IPR001680 (PFAM); IPR015155 (PFAM); IPR013535 (PFAM); IPR015943 (G3DSA:2.130.10.GENE3D); PTHR19849 (PANTHER); PTHR19849:SF0 (PANTHER); IPR017986 (PROSITE_PROFILES); IPR001680 (PROSITE_PROFILES); IPR001680 (PROSITE_PROFILES); IPR001680 (PROSITE_PROFILES); IPR013535 (PROSITE_PROFILES); IPR015155 (PROSITE_PROFILES); IPR001680 (PROSITE_PROFILES); IPR017986 (SUPERFAMILY); IPR016024 (SUPERFAMILY)
2128	301254_length_2300_cvg_44.5_tip_1_5	1880	contactin associated protein 1	766	4.55E-61	50.00%	0.153	Coil (COILS); IPR001073 (SMART); IPR001073 (PFAM); IPR014716 (G3DSA:3.90.215.GENE3D); IPR008983 (G3DSA:2.60.120.GENE3D); PTHR10127 (PANTHER); PTHR10127:SF575 (PANTHER); TRANSMEMBRANE (PHOBIUS); CYTOPLASMIC_DOMAIN (PHOBIUS); CYTOPLASMIC_DOMAIN (PHOBIUS); TRANSMEMBRANE (PHOBIUS); NON_CYTOPLASMIC_DOMAIN (PHOBIUS); NON_CYTOPLASMIC_DOMAIN (PHOBIUS); TRANSMEMBRANE (PHOBIUS); NON_CYTOPLASMIC_DOMAIN (PHOBIUS); TRANSMEMBRANE (PHOBIUS); IPR001073 (PROSITE_PROFILES); IPR008983 (SUPERFAMILY); IPR002181 (SUPERFAMILY); TMhelix (TMHMM); TMhelix (TMHMM); TMhelix (TMHMM)
2127	301248_length_2299_cvg_64.0_tip_1_3	4342	meprin a	767	3.73E-38	48.80%	0.097	IPR000859 (SMART); IPR001506 (PFAM); IPR000859 (G3DSA:2.60.120.GENE3D); IPR024079 (G3DSA:3.40.390.GENE3D); IPR000859 (G3DSA:2.60.120.GENE3D); IPR000859 (PFAM); PTHR10127 (PANTHER); PTHR10127:SF580 (PANTHER); TRANSMEMBRANE (PHOBIUS); TRANSMEMBRANE (PHOBIUS); NON_CYTOPLASMIC_DOMAIN (PHOBIUS); CYTOPLASMIC_DOMAIN (PHOBIUS); CYTOPLASMIC_DOMAIN (PHOBIUS); NON_CYTOPLASMIC_DOMAIN (PHOBIUS); TRANSMEMBRANE (PHOBIUS); TRANSMEMBRANE (PHOBIUS); CYTOPLASMIC_DOMAIN (PHOBIUS); IPR000859 (PROSITE_PROFILES); IPR000859 (SUPERFAMILY); IPR000859 (SUPERFAMILY); SSF55486 (SUPERFAMILY); TMhelix (TMHMM); TMhelix (TMHMM); TMhelix (TMHMM); TMhelix (TMHMM)

2129	301268_length_2304_cvg_6.9_tip_1_2	191	low density lipoprotein	768	0	64.70%	0.102	IPR000742 (SMART); IPR002223 (SMART); IPR001881 (SMART); IPR006605 (SMART); IPR003886 (SMART); IPR023413 (G3DSA:2.40.155.GENE3D); IPR002223 (G3DSA:4.10.410.GENE3D); IPR002223 (PFAM); IPR001881 (PFAM); G3DSA:2.10.25.10 (GENE3D); G3DSA:2.10.25.10 (GENE3D); IPR006605 (PFAM); IPR003886 (PFAM); PTHR11339 (PANTHER); IPR013032 (PROSITE_PATTERNS); IPR000152 (PROSITE_PATTERNS); IPR018097 (PROSITE_PATTERNS); IPR013032 (PROSITE_PATTERNS); IPR000152 (PROSITE_PATTERNS); IPR013032 (PROSITE_PATTERNS); TRANSMEMBRANE (PHOBIUS); CYTOPLASMIC_DOMAIN (PHOBIUS); NON_CYTOPLASMIC_DOMAIN (PHOBIUS); IPR000742 (PROSITE_PROFILES); IPR000742 (PROSITE_PROFILES); IPR002223 (PROSITE_PROFILES); IPR000742 (PROSITE_PROFILES); IPR006605 (PROSITE_PROFILES); IPR003886 (PROSITE_PROFILES); IPR000742 (PROSITE_PROFILES); SSF57196 (SUPERFAMILY); SSF57196 (SUPERFAMILY); IPR002223 (SUPERFAMILY); IPR009030 (SUPERFAMILY); IPR009017 (SUPERFAMILY)
2130	301286_length_2313_cvg_16.0_tip_1_1	407	exocyst complex component 6b-like isoform 2	771	0	80.60%	0.098	Coil (COILS); Coil (COILS); Coil (COILS); IPR007225 (PIRSF); IPR007225 (PFAM); IPR007225 (PANTHER); CYTOPLASMIC_DOMAIN (PHOBIUS); TRANSMEMBRANE (PHOBIUS); NON_CYTOPLASMIC_DOMAIN (PHOBIUS); TMhelix (TMHMM)
2131	301300_length_2323_cvg_11.0_tip_1_5	359	phenoloxidase subunit a3-like	774	0	67.40%	0.19	IPR013788 (PRINTS); IPR008922 (G3DSA:1.10.1280.GENE3D); IPR005204 (G3DSA:1.20.1370.GENE3D); IPR005203 (G3DSA:2.60.40.GENE3D); IPR005204 (PFAM); IPR000896 (PFAM); IPR005203 (PFAM); IPR013788 (PANTHER); PTHR11511:SF24 (PANTHER); IPR002227 (PROSITE_PATTERNS); IPR013788 (PROSITE_PATTERNS); IPR013788 (PROSITE_PATTERNS); IPR005204 (SUPERFAMILY); IPR008922 (SUPERFAMILY); IPR014756 (SUPERFAMILY)
2132	301304_length_2324_cvg_38.4_tip_1_4	1036	lim domain-binding protein 3	775	1.25E-107	72.40%	0.327	IPR001781 (SMART); IPR001781 (G3DSA:2.10.110.GENE3D); IPR001781 (PFAM); IPR001781 (G3DSA:2.10.110.GENE3D); IPR001781 (G3DSA:2.10.110.GENE3D); PTHR24214:SF29 (PANTHER); PTHR24214 (PANTHER); IPR001781 (PROSITE_PATTERNS); IPR001781 (PROSITE_PATTERNS); TRANSMEMBRANE (PHOBIUS); TRANSMEMBRANE (PHOBIUS); NON_CYTOPLASMIC_DOMAIN (PHOBIUS); CYTOPLASMIC_DOMAIN (PHOBIUS); TRANSMEMBRANE (PHOBIUS); TRANSMEMBRANE (PHOBIUS); CYTOPLASMIC_DOMAIN (PHOBIUS); NON_CYTOPLASMIC_DOMAIN (PHOBIUS); NON_CYTOPLASMIC_DOMAIN (PHOBIUS); IPR001781 (PROSITE_PROFILES); IPR001781 (PROSITE_PROFILES); IPR001781 (PROSITE_PROFILES); SignalP-TM (SIGNALP_GRAM_POSITIVE); SignalP-noTM (SIGNALP_GRAM_NEGATIVE); SSF57716 (SUPERFAMILY); SSF57716 (SUPERFAMILY); SSF57716 (SUPERFAMILY); TMhelix (TMHMM); TMhelix (TMHMM); TMhelix (TMHMM); TMhelix (TMHMM)
2135	301410_length_2391_cvg_17.2_tip_1_2	530	14-3-3 protein epsilon	792	9.52E-164	95.40%	0.118	Coil (COILS); IPR000308 (PRINTS); IPR023410 (SMART); G3DSA:1.20.190.20 (GENE3D); IPR023410 (PFAM); IPR000308 (PANTHER); IPR023409 (PROSITE_PATTERNS); IPR023409 (PROSITE_PATTERNS); NON_CYTOPLASMIC_DOMAIN (PHOBIUS); NON_CYTOPLASMIC_DOMAIN (PHOBIUS); CYTOPLASMIC_DOMAIN (PHOBIUS); TRANSMEMBRANE (PHOBIUS); TRANSMEMBRANE (PHOBIUS); IPR023410 (SUPERFAMILY)
2133	301394_length_2381_cvg_45.2_tip_1_0	1629	superoxide dismutase soluble	794	3.91E-42	55.10%	0.109	IPR001424 (PRINTS); IPR001424 (G3DSA:2.60.40.GENE3D); IPR001424 (PFAM); IPR018152 (PROSITE_PATTERNS); IPR001424 (SUPERFAMILY)
2134	301396_length_2383_cvg_7.3_tip_1_0	276	n-acetylated-alpha-linked acidic dipeptidase	795	0	59.30%	0.21	G3DSA:3.50.30.30 (GENE3D); G3DSA:3.40.630.10 (GENE3D); IPR003137 (PFAM); IPR007365 (G3DSA:1.20.930.GENE3D); IPR007484 (PFAM); IPR007365 (PFAM); PTHR10404 (PANTHER); CYTOPLASMIC_DOMAIN (PHOBIUS); TRANSMEMBRANE (PHOBIUS); TRANSMEMBRANE (PHOBIUS); NON_CYTOPLASMIC_DOMAIN (PHOBIUS); NON_CYTOPLASMIC_DOMAIN (PHOBIUS); SSF52025 (SUPERFAMILY); IPR007365 (SUPERFAMILY); SSF53187 (SUPERFAMILY); TMhelix (TMHMM)
2136	301416_length_2394_cvg_44.8_tip_1_3	1520	chorion peroxidase-like	798	2.88E-133	55.30%	0.103	IPR019791 (PRINTS); IPR019791 (PFAM); IPR019791 (G3DSA:1.10.640.GENE3D); PTHR11475 (PANTHER); TRANSMEMBRANE (PHOBIUS); TRANSMEMBRANE (PHOBIUS); NON_CYTOPLASMIC_DOMAIN (PHOBIUS); CYTOPLASMIC_DOMAIN (PHOBIUS); NON_CYTOPLASMIC_DOMAIN (PHOBIUS); IPR019791 (PROSITE_PROFILES); IPR010255 (SUPERFAMILY); TMhelix (TMHMM); TMhelix (TMHMM)

2137	301422_length_2397_cvg_66.7_tip_1_3	123579	myosin heavy chain isoform 3	799	0	91.70%	0.101		Coil (COILS); Coil (COILS); Coil (COILS); Coil (COILS); Coil (COILS); Coil (COILS); Coil (COILS); Coil (COILS); Coil (COILS); IPR002928 (PFAM); PTHR13140:SF364 (PANTHER); PTHR13140 (PANTHER); NON_CYTOPLASMIC_DOMAIN (PHOBIUS); CYTOPLASMIC_DOMAIN (PHOBIUS); TRANSMEMBRANE (PHOBIUS); SSF90257 (SUPERFAMILY); SSF90257 (SUPERFAMILY); SSF90257 (SUPERFAMILY); TMhelix (TMHMM)
2138	301444_length_2425_cvg_52.0_tip_1_0	2386	neutral ceramidase	804	0	66.70%	0.145		IPR006823 (PFAM); IPR006823 (PANTHER); PTHR12670:SF1 (PANTHER); CYTOPLASMIC_DOMAIN (PHOBIUS); TRANSMEMBRANE (PHOBIUS); NON_CYTOPLASMIC_DOMAIN (PHOBIUS); TRANSMEMBRANE (PHOBIUS); NON_CYTOPLASMIC_DOMAIN (PHOBIUS)
2139	301468_length_2441_cvg_6.1_tip_1_0	200	sphingomyelin partial	814	0	76.30%	0.119		IPR008139 (SMART); IPR029052 (G3DSA:3.60.21.GENE3D); IPR004843 (PFAM); IPR011001 (G3DSA:1.10.225.GENE3D); PTHR10340:SF13 (PANTHER); PTHR10340 (PANTHER); CYTOPLASMIC_DOMAIN (PHOBIUS); TRANSMEMBRANE (PHOBIUS); TRANSMEMBRANE (PHOBIUS); TRANSMEMBRANE (PHOBIUS); NON_CYTOPLASMIC_DOMAIN (PHOBIUS); NON_CYTOPLASMIC_DOMAIN (PHOBIUS); CYTOPLASMIC_DOMAIN (PHOBIUS); IPR008139 (PROSITE_PROFILES); IPR029052 (SUPERFAMILY); IPR011001 (SUPERFAMILY); TMhelix (TMHMM)
2140	301494_length_2460_cvg_21.3_tip_1_5	650	low quality protein: mucin-5b-like	820	4.27E-08	56.33%	0.102		no IPS match
2141	301498_length_2461_cvg_43.9_tip_1_3	1459	heat shock 70 kda protein cognate 5	821	0	89.00%	0.158		Coil (COILS); IPR013126 (PRINTS); IPR012725 (TIGRFAM); G3DSA:3.30.420.40 (GENE3D); G3DSA:3.30.30.30 (GENE3D); IPR029048 (G3DSA:1.20.1270.GENE3D); G3DSA:3.30.420.40 (GENE3D); IPR013126 (PFAM); IPR029047 (G3DSA:2.60.34.GENE3D); G3DSA:3.90.640.10 (GENE3D); PTHR19375:SF173 (PANTHER); PTHR19375 (PANTHER); IPR018181 (PROSITE_PATTERNS); IPR018181 (PROSITE_PATTERNS); IPR018181 (PROSITE_PATTERNS); IPR012725 (HAMAP); SSF53067 (SUPERFAMILY); IPR029047 (SUPERFAMILY); SSF53067 (SUPERFAMILY)
2142	301512_length_2469_cvg_14.7_tip_1_1	424	sel1l protein	823	0	77.80%	0.298		Coil (COILS); IPR006597 (SMART); IPR011990 (G3DSA:1.25.40.GENE3D); IPR006597 (PFAM); IPR011990 (G3DSA:1.25.40.GENE3D); PTHR11102 (PANTHER); PTHR11102:SF55 (PANTHER); NON_CYTOPLASMIC_DOMAIN (PHOBIUS); TRANSMEMBRANE (PHOBIUS); CYTOPLASMIC_DOMAIN (PHOBIUS); CYTOPLASMIC_DOMAIN (PHOBIUS); TRANSMEMBRANE (PHOBIUS); SSF81901 (SUPERFAMILY); SSF81901 (SUPERFAMILY); SSF81901 (SUPERFAMILY); TMhelix (TMHMM); TMhelix (TMHMM)
2143	301528_length_2475_cvg_23.6_tip_1_2	609	superoxide dismutase	825	2.56E-61	67.40%	0.184		IPR001424 (PRINTS); IPR001424 (PFAM); IPR001424 (G3DSA:2.60.40.GENE3D); PTHR10003 (PANTHER); IPR018152 (PROSITE_PATTERNS); IPR018152 (PROSITE_PATTERNS); TRANSMEMBRANE (PHOBIUS); NON_CYTOPLASMIC_DOMAIN (PHOBIUS); CYTOPLASMIC_DOMAIN (PHOBIUS); IPR001424 (SUPERFAMILY); TMhelix (TMHMM)
2144	301546_length_2486_cvg_36.1_tip_1_4	1031	glucose dehydrogenase	829	0	65.90%	0.131		IPR000172 (PFAM); IPR007867 (PFAM); G3DSA:3.50.50.60 (GENE3D); PTHR11552:SF77 (PANTHER); PTHR11552 (PANTHER); IPR000172 (PROSITE_PATTERNS); IPR000172 (PROSITE_PATTERNS); CYTOPLASMIC_DOMAIN (PHOBIUS); TRANSMEMBRANE (PHOBIUS); NON_CYTOPLASMIC_DOMAIN (PHOBIUS); NON_CYTOPLASMIC_DOMAIN (PHOBIUS); TRANSMEMBRANE (PHOBIUS); SSF54373 (SUPERFAMILY); SSF51905 (SUPERFAMILY); TMhelix (TMHMM)
2145	301572_length_2497_cvg_29.2_tip_1_1	956	cytochrome p450 6k1-like	832	2.20E-129	59.20%	0.152		IPR001128 (PRINTS); IPR002401 (PRINTS); IPR001128 (G3DSA:1.10.630.GENE3D); IPR001128 (PFAM); PTHR24292 (PANTHER); PTHR24292:SF24 (PANTHER); IPR017972 (PROSITE_PATTERNS); TRANSMEMBRANE (PHOBIUS); TRANSMEMBRANE (PHOBIUS); NON_CYTOPLASMIC_DOMAIN (PHOBIUS); CYTOPLASMIC_DOMAIN (PHOBIUS); TRANSMEMBRANE (PHOBIUS); CYTOPLASMIC_DOMAIN (PHOBIUS); NON_CYTOPLASMIC_DOMAIN (PHOBIUS); IPR001128 (SUPERFAMILY); TMhelix (TMHMM); TMhelix (TMHMM)
2146	301582_length_2502_cvg_12.3_tip_1_2	561	hemocyte protein-glutamine gamma- partial	834	0	70.40%	0.461	Y	IPR002931 (SMART); IPR013783 (G3DSA:2.60.40.GENE3D); IPR013783 (G3DSA:2.60.40.GENE3D); IPR008958 (PFAM); IPR023608 (PIRSF); IPR013783 (G3DSA:2.60.40.GENE3D); IPR001102 (PFAM); IPR002931 (PFAM); IPR002931 (G3DSA:3.90.260.GENE3D); PTHR11590:SF40 (PANTHER); IPR023608 (PANTHER); SignalP-noTM (SIGNALP_EUK); IPR008958 (SUPERFAMILY); IPR008958 (SUPERFAMILY); SSF54001 (SUPERFAMILY); IPR014756 (SUPERFAMILY)

2147	301596_length_2515_cvg_6.2_tip_1_3	224	basement membrane-specific heparan sulfate proteoglycan core protein	839	0	59.70%	0.098	IPR003599 (SMART); IPR003598 (SMART); IPR013106 (SMART); IPR013783 (G3DSA:2.60.40.GENE3D); IPR013032 (PFAM); IPR002049 (PFAM); IPR013783 (G3DSA:2.60.40.GENE3D); G3DSA:2.170.300.10 (GENE3D); PF13895 (PFAM); IPR013783 (G3DSA:2.60.40.GENE3D); IPR013783 (G3DSA:2.60.40.GENE3D); IPR013098 (PFAM); IPR013151 (PFAM); IPR013783 (G3DSA:2.60.40.GENE3D); PTHR10489 (PANTHER); IPR013032 (PROSITE_PATTERNS); IPR002049 (PROSITE_PATTERNS); IPR013032 (PROSITE_PATTERNS); IPR013032 (PROSITE_PATTERNS); IPR013032 (PROSITE_PATTERNS); IPR000742 (PROSITE_PROFILES); IPR007110 (PROSITE_PROFILES); IPR007110 (PROSITE_PROFILES); IPR007110 (PROSITE_PROFILES); IPR007110 (PROSITE_PROFILES); IPR007110 (PROSITE_PROFILES); IPR007110 (PROSITE_PROFILES); IPR007110 (PROSITE_PROFILES); IPR000742 (PROSITE_PROFILES); SSF48726 (SUPERFAMILY); SSF48726 (SUPERFAMILY); SSF48726 (SUPERFAMILY); SSF48726 (SUPERFAMILY); SSF48726 (SUPERFAMILY); SSF48726 (SUPERFAMILY); SSF48726 (SUPERFAMILY); SSF48726 (SUPERFAMILY)
2148	301606_length_2520_cvg_9.9_tip_1_4	420	arylsulfatase b	840	0	72.00%	0.248	IPR000917 (PFAM); IPR017849 (G3DSA:3.40.720.GENE3D); G3DSA:3.30.1120.10 (GENE3D); PTHR10342 (PANTHER); PTHR10342:SF189 (PANTHER); IPR024607 (PROSITE_PATTERNS); IPR024607 (PROSITE_PATTERNS); NON_CYTOPLASMIC_DOMAIN (PHOBIUS); NON_CYTOPLASMIC_DOMAIN (PHOBIUS); TRANSMEMBRANE (PHOBIUS); CYTOPLASMIC_DOMAIN (PHOBIUS); CYTOPLASMIC_DOMAIN (PHOBIUS); TRANSMEMBRANE (PHOBIUS); CYTOPLASMIC_DOMAIN (PHOBIUS); TRANSMEMBRANE (PHOBIUS); TRANSMEMBRANE (PHOBIUS); TRANSMEMBRANE (PHOBIUS); NON_CYTOPLASMIC_DOMAIN (PHOBIUS); IPR017850 (SUPERFAMILY)
2149	301610_length_2521_cvg_31.4_tip_1_4	1290	krueppel-like factor 10	840	1.41E-71	63.10%	0.167	IPR015880 (SMART); IPR013087 (G3DSA:3.30.160.GENE3D); PF13465 (PFAM); IPR013087 (G3DSA:3.30.160.GENE3D); PTHR23223:SF21 (PANTHER); PTHR23223 (PANTHER); IPR007087 (PROSITE_PATTERNS); IPR007087 (PROSITE_PATTERNS); IPR007087 (PROSITE_PATTERNS); TRANSMEMBRANE (PHOBIUS); NON_CYTOPLASMIC_DOMAIN (PHOBIUS); TRANSMEMBRANE (PHOBIUS); TRANSMEMBRANE (PHOBIUS); CYTOPLASMIC_DOMAIN (PHOBIUS); NON_CYTOPLASMIC_DOMAIN (PHOBIUS); TRANSMEMBRANE (PHOBIUS); TRANSMEMBRANE (PHOBIUS); NON_CYTOPLASMIC_DOMAIN (PHOBIUS); TRANSMEMBRANE (PHOBIUS); NON_CYTOPLASMIC_DOMAIN (PHOBIUS); CYTOPLASMIC_DOMAIN (PHOBIUS); CYTOPLASMIC_DOMAIN (PHOBIUS); IPR007087 (PROSITE_PROFILES); IPR007087 (PROSITE_PROFILES); IPR007087 (PROSITE_PROFILES); SSF57667 (SUPERFAMILY); SSF57667 (SUPERFAMILY); TMhelix (TMHMM); TMhelix (TMHMM); TMhelix (TMHMM); TMhelix (TMHMM)
2150	301628_length_2531_cvg_7.6_tip_1_0	283	atp-dependent zinc metalloprotease yme1 homolog	844	0	75.30%	0.27	IPR003593 (SMART); IPR027417 (G3DSA:3.40.50.GENE3D); G3DSA:1.10.8.60 (GENE3D); IPR005936 (TIGRFAM); IPR003959 (PFAM); IPR000642 (PFAM); PTHR23076 (PANTHER); PTHR23076:SF37 (PANTHER); IPR003960 (PROSITE_PATTERNS); TRANSMEMBRANE (PHOBIUS); TRANSMEMBRANE (PHOBIUS); TRANSMEMBRANE (PHOBIUS); NON_CYTOPLASMIC_DOMAIN (PHOBIUS); CYTOPLASMIC_DOMAIN (PHOBIUS); CYTOPLASMIC_DOMAIN (PHOBIUS); CYTOPLASMIC_DOMAIN (PHOBIUS); NON_CYTOPLASMIC_DOMAIN (PHOBIUS); TRANSMEMBRANE (PHOBIUS); IPR005936 (HAMAP); SSF140990 (SUPERFAMILY); IPR027417 (SUPERFAMILY); TMhelix (TMHMM); TMhelix (TMHMM)
2151	301634_length_2535_cvg_44.7_tip_1_1	27594	enolase	845	0	89.50%	0.3	IPR000941 (PRINTS); IPR000941 (TIGRFAM); IPR020810 (PFAM); IPR029017 (G3DSA:3.30.390.GENE3D); IPR020811 (PFAM); IPR029065 (G3DSA:3.20.20.GENE3D); IPR000941 (PANTHER); IPR020809 (PROSITE_PATTERNS); CYTOPLASMIC_DOMAIN (PHOBIUS); CYTOPLASMIC_DOMAIN (PHOBIUS); TRANSMEMBRANE (PHOBIUS); TRANSMEMBRANE (PHOBIUS); NON_CYTOPLASMIC_DOMAIN (PHOBIUS); TRANSMEMBRANE (PHOBIUS); NON_CYTOPLASMIC_DOMAIN (PHOBIUS); TRANSMEMBRANE (PHOBIUS); CYTOPLASMIC_DOMAIN (PHOBIUS); TRANSMEMBRANE (PHOBIUS); NON_CYTOPLASMIC_DOMAIN (PHOBIUS); IPR000941 (HAMAP); IPR029065 (SUPERFAMILY); IPR029017 (SUPERFAMILY); TMhelix (TMHMM); TMhelix (TMHMM); TMhelix (TMHMM)

2152	301646_length_2550_cvg_6.2_tip_1_1	185	protein disulfide-isomerase a4-like	850	8.02E-48	70.40%	0.098	IPR012336 (G3DSA:3.40.30.GENE3D); IPR013766 (PFAM); PTHR18929 (PANTHER); PTHR18929:SF56 (PANTHER); IPR017937 (PROSITE_PATTERNS); NON_CYTOPLASMIC_DOMAIN (PHOBIUS); NON_CYTOPLASMIC_DOMAIN (PHOBIUS); CYTOPLASMIC_DOMAIN (PHOBIUS); TRANSMEMBRANE (PHOBIUS); NON_CYTOPLASMIC_DOMAIN (PHOBIUS); TRANSMEMBRANE (PHOBIUS); TRANSMEMBRANE (PHOBIUS); TRANSMEMBRANE (PHOBIUS); CYTOPLASMIC_DOMAIN (PHOBIUS); CYTOPLASMIC_DOMAIN (PHOBIUS); TRANSMEMBRANE (PHOBIUS); IPR012336 (PROSITE_PROFILES); IPR012336 (SUPERFAMILY); TMhelix (TMHMM)
2153	301650_length_2551_cvg_19.9_tip_1_2	648	growth differentiation factor 8-like	850	1.07E-109	64.30%	0.449 Y	IPR001839 (SMART); IPR029034 (G3DSA:2.10.90.GENE3D); IPR001111 (PFAM); IPR001839 (PFAM); PTHR11848:SF126 (PANTHER); IPR015615 (PANTHER); IPR017948 (PROSITE_PATTERNS); TRANSMEMBRANE (PHOBIUS); TRANSMEMBRANE (PHOBIUS); NON_CYTOPLASMIC_DOMAIN (PHOBIUS); CYTOPLASMIC_DOMAIN (PHOBIUS); TRANSMEMBRANE (PHOBIUS); NON_CYTOPLASMIC_DOMAIN (PHOBIUS); TRANSMEMBRANE (PHOBIUS); NON_CYTOPLASMIC_DOMAIN (PHOBIUS); NON_CYTOPLASMIC_DOMAIN (PHOBIUS); NON_CYTOPLASMIC_DOMAIN (PHOBIUS); CYTOPLASMIC_DOMAIN (PHOBIUS); CYTOPLASMIC_DOMAIN (PHOBIUS); TRANSMEMBRANE (PHOBIUS); CYTOPLASMIC_DOMAIN (PHOBIUS); TRANSMEMBRANE (PHOBIUS); TRANSMEMBRANE (PHOBIUS); TRANSMEMBRANE (PHOBIUS); IPR001839 (PROSITE_PROFILES); IPR029034 (SUPERFAMILY); TMhelix (TMHMM); TMhelix (TMHMM); TMhelix (TMHMM); TMhelix (TMHMM); TMhelix (TMHMM); TMhelix (TMHMM); TMhelix (TMHMM)
2154	301654_length_2554_cvg_58.0_tip_1_0	3376	heat shock 70 kda protein cognate 3	852	0	93.60%	0.182	Coil (COILS); Coil (COILS); IPR013126 (PRINTS); G3DSA:3.30.420.40 (GENE3D); IPR029048 (G3DSA:1.20.1270.GENE3D); G3DSA:3.90.640.10 (GENE3D); IPR013126 (PFAM); G3DSA:3.30.30.30 (GENE3D); IPR029047 (G3DSA:2.60.34.GENE3D); G3DSA:3.30.420.40 (GENE3D); PTHR19375 (PANTHER); PTHR19375:SF157 (PANTHER); IPR018181 (PROSITE_PATTERNS); IPR018181 (PROSITE_PATTERNS); IPR018181 (PROSITE_PATTERNS); CYTOPLASMIC_DOMAIN (PHOBIUS); TRANSMEMBRANE (PHOBIUS); NON_CYTOPLASMIC_DOMAIN (PHOBIUS); IPR029048 (SUPERFAMILY); SSF53067 (SUPERFAMILY); SSF53067 (SUPERFAMILY); IPR029047 (SUPERFAMILY); TMhelix (TMHMM); TMhelix (TMHMM)
2155	301662_length_2562_cvg_7.0_tip_1_3	288	tensin isoform x11	854	4.83E-143	60.90%	0.117	IPR029021 (G3DSA:3.90.190.GENE3D); G3DSA:2.60.40.1110 (GENE3D); IPR014020 (PFAM); PTHR12583 (PANTHER); PTHR12583:SF7 (PANTHER); IPR014020 (PROSITE_PROFILES); IPR029023 (PROSITE_PROFILES); IPR029021 (SUPERFAMILY); IPR000008 (SUPERFAMILY)
2156	301668_length_2567_cvg_58.3_tip_1_2	5939	ubiquitin- isoform a	855	0	99.00%	0.418 Y	IPR019956 (PRINTS); IPR000626 (SMART); IPR000626 (PFAM); G3DSA:3.10.20.90 (GENE3D); PTHR10666:SF94 (PANTHER); PTHR10666 (PANTHER); IPR019954 (PROSITE_PATTERNS); IPR019954 (PROSITE_PATTERNS); IPR019954 (PROSITE_PATTERNS); IPR019954 (PROSITE_PATTERNS); IPR019954 (PROSITE_PATTERNS); IPR019954 (PROSITE_PATTERNS); IPR019954 (PROSITE_PATTERNS); IPR019954 (PROSITE_PATTERNS); IPR019954 (PROSITE_PATTERNS); NON_CYTOPLASMIC_DOMAIN (PHOBIUS); CYTOPLASMIC_DOMAIN (PHOBIUS); TRANSMEMBRANE (PHOBIUS); IPR000626 (PROSITE_PROFILES); IPR000626 (PROSITE_PROFILES); IPR000626 (PROSITE_PROFILES); IPR000626 (PROSITE_PROFILES); IPR000626 (PROSITE_PROFILES); IPR000626 (PROSITE_PROFILES); IPR000626 (PROSITE_PROFILES); IPR000626 (PROSITE_PROFILES); IPR000626 (PROSITE_PROFILES); IPR029071 (SUPERFAMILY); IPR029071 (SUPERFAMILY); IPR029071 (SUPERFAMILY); IPR029071 (SUPERFAMILY); IPR029071 (SUPERFAMILY); IPR029071 (SUPERFAMILY); IPR029071 (SUPERFAMILY); IPR029071 (SUPERFAMILY); IPR029071 (SUPERFAMILY); TMhelix (TMHMM)
2157	301682_length_2589_cvg_4.9_tip_1_1	211	sodium calcium exchanger partial	863	0	75.00%	0.103	IPR004836 (PRINTS); IPR003644 (SMART); IPR004837 (PFAM); IPR003644 (PFAM); PTHR11878 (PANTHER); TRANSMEMBRANE (PHOBIUS); TRANSMEMBRANE (PHOBIUS); TRANSMEMBRANE (PHOBIUS); NON_CYTOPLASMIC_DOMAIN (PHOBIUS); CYTOPLASMIC_DOMAIN (PHOBIUS); TRANSMEMBRANE (PHOBIUS); TRANSMEMBRANE (PHOBIUS); NON_CYTOPLASMIC_DOMAIN (PHOBIUS); CYTOPLASMIC_DOMAIN (PHOBIUS); NON_CYTOPLASMIC_DOMAIN (PHOBIUS); TRANSMEMBRANE (PHOBIUS); NON_CYTOPLASMIC_DOMAIN (PHOBIUS); CYTOPLASMIC_DOMAIN (PHOBIUS); SSF141072 (SUPERFAMILY); SSF141072 (SUPERFAMILY); TMhelix (TMHMM); TMhelix (TMHMM); TMhelix (TMHMM); TMhelix (TMHMM); TMhelix (TMHMM)

2158	301684_length_2590_cvg_33.8_tip_1_2	1063	gastrula zinc finger	863	7.89E-52	51.80%	0.127		IPR001713 (PRINTS); IPR000010 (SMART); IPR015880 (SMART); IPR000010 (PFAM); IPR007087 (PFAM); IPR013087 (G3DSA:3.30.160.GENE3D); PF13894 (PFAM); G3DSA:3.10.450.10 (GENE3D); IPR013087 (G3DSA:3.30.160.GENE3D); IPR013087 (G3DSA:3.30.160.GENE3D); PF13465 (PFAM); PTHR24409:SF14 (PANTHER); PTHR24409 (PANTHER); IPR018073 (PROSITE_PATTERNS); IPR007087 (PROSITE_PATTERNS); IPR007087 (PROSITE_PATTERNS); IPR007087 (PROSITE_PATTERNS); IPR007087 (PROSITE_PATTERNS); IPR007087 (PROSITE_PATTERNS); IPR007087 (PROSITE_PATTERNS); IPR007087 (PROSITE_PATTERNS); IPR007087 (PROSITE_PATTERNS); IPR007087 (PROSITE_PATTERNS); IPR007087 (PROSITE_PATTERNS); IPR007087 (PROSITE_PROFILES); IPR007087 (PROSITE_PROFILES); IPR007087 (PROSITE_PROFILES); IPR007087 (PROSITE_PROFILES); IPR007087 (PROSITE_PROFILES); IPR007087 (PROSITE_PROFILES); IPR007087 (PROSITE_PROFILES); IPR007087 (PROSITE_PROFILES); IPR007087 (PROSITE_PROFILES); IPR007087 (PROSITE_PROFILES); IPR007087 (PROSITE_PROFILES); SSF54403 (SUPERFAMILY); SSF57667 (SUPERFAMILY); SSF57667 (SUPERFAMILY); SSF57667 (SUPERFAMILY); SSF57667 (SUPERFAMILY); SSF57667 (SUPERFAMILY); SSF57667 (SUPERFAMILY)
2159	301702_length_2608_cvg_34.2_tip_1_4	1033	annu_scham ame: full=annulin ame: full=protein-glutamine gamma-glutamyltransferase ame: full=transglutaminase	869	0	73.90%	0.11		IPR002931 (SMART); IPR013783 (G3DSA:2.60.40.GENE3D); IPR013783 (G3DSA:2.60.40.GENE3D); IPR002931 (G3DSA:3.90.260.GENE3D); IPR002931 (PFAM); IPR023608 (PIRSF); IPR008958 (PFAM); IPR001102 (PFAM); IPR013783 (G3DSA:2.60.40.GENE3D); PTHR11590:SF16 (PANTHER); IPR023608 (PANTHER); IPR008958 (SUPERFAMILY); IPR014756 (SUPERFAMILY); IPR008958 (SUPERFAMILY); SSF54001 (SUPERFAMILY)
2160	301732_length_2628_cvg_9.8_tip_1_0	318	nicotinamide phosphoribosyltransferase-like	876	0	81.60%	0.177		IPR007229 (PFAM); G3DSA:3.20.140.10 (GENE3D); IPR007229 (PANTHER); IPR016471 (PTHR11098:PANTHER); TRANSMEMBRANE (PHOBIUS); NON_CYTOPLASMIC_DOMAIN (PHOBIUS); TRANSMEMBRANE (PHOBIUS); CYTOPLASMIC_DOMAIN (PHOBIUS); NON_CYTOPLASMIC_DOMAIN (PHOBIUS); IPR002638 (SUPERFAMILY); TMhelix (TMHMM)
2161	301744_length_2636_cvg_12.7_tip_1_5	390	hypothetical protein RirG_019080	878	2.60E-06	50.00%	0.101		Coil (COILS); Coil (COILS); Coil (COILS); Coil (COILS); Coil (COILS); Coil (COILS); Coil (COILS); Coil (COILS); Coil (COILS); Coil (COILS); Coil (COILS); Coil (COILS); Coil (COILS); PTHR13140:SF342 (PANTHER); PTHR13140 (PANTHER)
2162	301748_length_2641_cvg_9.3_tip_1_5	389	tyrosine-protein phosphatase non-receptor type 13-like isoform x3	880	7.55E-119	51.60%	0.17		IPR001478 (SMART); IPR001478 (G3DSA:2.30.42.GENE3D); IPR001478 (G3DSA:2.30.42.GENE3D); IPR001478 (PFAM); PTHR19964 (PANTHER); PTHR19964:SF32 (PANTHER); IPR001478 (PROSITE_PROFILES); IPR001478 (PROSITE_PROFILES); IPR001478 (SUPERFAMILY); IPR001478 (SUPERFAMILY)
2163	301760_length_2652_cvg_17.7_tip_1_3	548	fasciclin- partial	884	0	58.30%	0.166		IPR009138 (PRINTS); IPR003961 (SMART); IPR003598 (SMART); IPR003599 (SMART); IPR013783 (G3DSA:2.60.40.GENE3D); IPR013783 (G3DSA:2.60.40.GENE3D); IPR013783 (G3DSA:2.60.40.GENE3D); IPR003961 (PFAM); PF13895 (PFAM); IPR013098 (PFAM); IPR013783 (G3DSA:2.60.40.GENE3D); PF13927 (PFAM); IPR013783 (G3DSA:2.60.40.GENE3D); IPR013783 (G3DSA:2.60.40.GENE3D); PTHR10489:SF556 (PANTHER); PTHR10489 (PANTHER); TRANSMEMBRANE (PHOBIUS); NON_CYTOPLASMIC_DOMAIN (PHOBIUS); CYTOPLASMIC_DOMAIN (PHOBIUS); IPR007110 (PROSITE_PROFILES); IPR007110 (PROSITE_PROFILES); IPR007110 (PROSITE_PROFILES); IPR007110 (PROSITE_PROFILES); IPR003961 (PROSITE_PROFILES); IPR007110 (PROSITE_PROFILES); IPR003961 (PROSITE_PROFILES); SSF48726 (SUPERFAMILY); SSF48726 (SUPERFAMILY); SSF48726 (SUPERFAMILY); SSF48726 (SUPERFAMILY); SSF48726 (SUPERFAMILY); IPR003961 (SUPERFAMILY); TMhelix (TMHMM)
2164	301764_length_2653_cvg_9.7_tip_1_0	471	matrix metalloproteinase-16 isoform x3	885	0	74.50%	0.547	Y	IPR021190 (PRINTS); IPR006026 (SMART); IPR018487 (SMART); IPR018487 (PFAM); IPR002477 (PFAM); IPR024079 (G3DSA:3.40.390.GENE3D); IPR000585 (G3DSA:2.110.10.GENE3D); IPR001818 (PFAM); PTHR10201 (PANTHER); PTHR10201:SF131 (PANTHER); IPR018486 (PROSITE_PATTERNS); TRANSMEMBRANE (PHOBIUS); CYTOPLASMIC_DOMAIN (PHOBIUS); NON_CYTOPLASMIC_DOMAIN (PHOBIUS); IPR018487 (PROSITE_PROFILES); IPR018487 (PROSITE_PROFILES); IPR018487 (PROSITE_PROFILES); IPR018487 (PROSITE_PROFILES); SignalP-noTM (SIGNALP_EUK); IPR002477 (SUPERFAMILY); SSF55486 (SUPERFAMILY); IPR000585 (SUPERFAMILY)

2165	301770_length_2656_cvg_18.7_tip_1_4	664	cd109 antigen-like isoform x2	885	0	73.70%	0.102	IPR009048 (G3DSA:2.60.40.GENE3D); IPR008930 (G3DSA:1.50.10.GENE3D); IPR009048 (PFAM); IPR019565 (PFAM); IPR011626 (PFAM); IPR001599 (PFAM); PTHR11412 (PANTHER); PTHR11412:SF84 (PANTHER); IPR019742 (PROSITE_PATTERNS); NON_CYTOPLASMIC_DOMAIN (PHOBIUS); CYTOPLASMIC_DOMAIN (PHOBIUS); TRANSMEMBRANE (PHOBIUS); IPR008930 (SUPERFAMILY); IPR014756 (SUPERFAMILY); IPR009048 (SUPERFAMILY); TMhelix (TMHMM)
2166	301778_length_2662_cvg_12.0_tip_1_2	430	retinoid-inducible serine carboxypeptidase	887	3.25E-143	67.30%	0.15	IPR001563 (PRINTS); IPR029058 (G3DSA:3.40.50.GENE3D); IPR001563 (PFAM); IPR001563 (PANTHER); PTHR11802:SF3 (PANTHER); IPR018202 (PROSITE_PATTERNS); CYTOPLASMIC_DOMAIN (PHOBIUS); TRANSMEMBRANE (PHOBIUS); CYTOPLASMIC_DOMAIN (PHOBIUS); TRANSMEMBRANE (PHOBIUS); NON_CYTOPLASMIC_DOMAIN (PHOBIUS); CYTOPLASMIC_DOMAIN (PHOBIUS); TRANSMEMBRANE (PHOBIUS); NON_CYTOPLASMIC_DOMAIN (PHOBIUS); TRANSMEMBRANE (PHOBIUS); TRANSMEMBRANE (PHOBIUS); NON_CYTOPLASMIC_DOMAIN (PHOBIUS); IPR029058 (SUPERFAMILY); TMhelix (TMHMM); TMhelix (TMHMM); TMhelix (TMHMM); TMhelix (TMHMM)
2167	301792_length_2672_cvg_12.8_tip_1_3	394	alpha-2-macroglobulin-like protein 1	891	6.73E-142	60.50%	0.203	IPR009048 (G3DSA:2.60.40.GENE3D); IPR009048 (PFAM); IPR011626 (PFAM); IPR008930 (G3DSA:1.50.10.GENE3D); PTHR11412 (PANTHER); NON_CYTOPLASMIC_DOMAIN (PHOBIUS); CYTOPLASMIC_DOMAIN (PHOBIUS); TRANSMEMBRANE (PHOBIUS); IPR008930 (SUPERFAMILY); IPR009048 (SUPERFAMILY); TMhelix (TMHMM)
2168	301806_length_2682_cvg_53.5_tip_1_4	2544	ankyrin-2 isoform x3	894	0	93.00%	0.107	IPR002110 (PRINTS); IPR002110 (SMART); IPR020683 (G3DSA:1.25.40.GENE3D); IPR020683 (G3DSA:1.25.40.GENE3D); IPR020683 (PFAM); IPR002110 (PFAM); IPR020683 (G3DSA:1.25.40.GENE3D); PTHR24123 (PANTHER); PTHR24123:SF0 (PANTHER); IPR002110 (PROSITE_PROFILES); IPR002110 (PROSITE_PROFILES); IPR002110 (PROSITE_PROFILES); IPR002110 (PROSITE_PROFILES); IPR002110 (PROSITE_PROFILES); IPR002110 (PROSITE_PROFILES); IPR002110 (PROSITE_PROFILES); IPR002110 (PROSITE_PROFILES); IPR002110 (PROSITE_PROFILES); IPR002110 (PROSITE_PROFILES); IPR002110 (PROSITE_PROFILES); IPR002110 (PROSITE_PROFILES); IPR002110 (PROSITE_PROFILES); IPR020683 (PROSITE_PROFILES); IPR002110 (PROSITE_PROFILES); IPR002110 (PROSITE_PROFILES); IPR002110 (PROSITE_PROFILES); IPR002110 (PROSITE_PROFILES); IPR002110 (PROSITE_PROFILES); IPR002110 (PROSITE_PROFILES); IPR002110 (PROSITE_PROFILES); IPR002110 (PROSITE_PROFILES); IPR020683 (SUPERFAMILY); IPR020683 (SUPERFAMILY)
2169	301812_length_2685_cvg_32.9_tip_1_2	1130	integral membrane protein 2c	895	1.02E-117	67.30%	0.177	IPR007084 (SMART); IPR007084 (PFAM); PTHR10962 (PANTHER); PTHR10962:SF1 (PANTHER); TRANSMEMBRANE (PHOBIUS); NON_CYTOPLASMIC_DOMAIN (PHOBIUS); NON_CYTOPLASMIC_DOMAIN (PHOBIUS); CYTOPLASMIC_DOMAIN (PHOBIUS); TRANSMEMBRANE (PHOBIUS); TRANSMEMBRANE (PHOBIUS); CYTOPLASMIC_DOMAIN (PHOBIUS); IPR007084 (PROSITE_PROFILES); TMhelix (TMHMM); TMhelix (TMHMM); TMhelix (TMHMM)
2170	301826_length_2696_cvg_11.0_tip_1_4	460	sortilin-related receptor-like	899	2.62E-132	55.70%	0.143	IPR003961 (SMART); IPR013783 (G3DSA:2.60.40.GENE3D); IPR003961 (PFAM); IPR013783 (G3DSA:2.60.40.GENE3D); IPR013783 (G3DSA:2.60.40.GENE3D); IPR013783 (G3DSA:2.60.40.GENE3D); PTHR10489 (PANTHER); TRANSMEMBRANE (PHOBIUS); TRANSMEMBRANE (PHOBIUS); CYTOPLASMIC_DOMAIN (PHOBIUS); NON_CYTOPLASMIC_DOMAIN (PHOBIUS); TRANSMEMBRANE (PHOBIUS); NON_CYTOPLASMIC_DOMAIN (PHOBIUS); CYTOPLASMIC_DOMAIN (PHOBIUS); IPR003961 (PROSITE_PROFILES); IPR003961 (PROSITE_PROFILES); IPR003961 (PROSITE_PROFILES); IPR003961 (PROSITE_PROFILES); IPR003961 (PROSITE_PROFILES); IPR003961 (PROSITE_PROFILES); IPR003961 (SUPERFAMILY); IPR003961 (SUPERFAMILY); IPR003961 (SUPERFAMILY); TMhelix (TMHMM)
2171	301828_length_2697_cvg_28.5_tip_1_5	818	calcium-activated chloride channel regulator 4-like	899	5.88E-120	53.90%	0.124	IPR003961 (SMART); IPR002035 (G3DSA:3.40.50.GENE3D); PF13519 (PFAM); IPR013783 (G3DSA:2.60.40.GENE3D); IPR015394 (PFAM); PTHR10579 (PANTHER); PTHR10579:SF8 (PANTHER); TRANSMEMBRANE (PHOBIUS); NON_CYTOPLASMIC_DOMAIN (PHOBIUS); CYTOPLASMIC_DOMAIN (PHOBIUS); IPR002035 (PROSITE_PROFILES); IPR003961 (SUPERFAMILY); IPR002035 (SUPERFAMILY); TMhelix (TMHMM)

2172	301842_length_2715_cvg_41.7_tip_1_0	2102	tissue factor pathway inhibitor 2	905	4.23E-33	62.80%	0.129	Coil (COILS); IPR002223 (PRINTS); IPR002223 (SMART); IPR002223 (PFAM); IPR002223 (G3DSA:4.10.410.GENE3D); IPR002223 (G3DSA:4.10.410.GENE3D); PTHR10083 (PANTHER); IPR020901 (PROSITE_PATTERNS); IPR020901 (PROSITE_PATTERNS); TRANSMEMBRANE (PHOBIUS); NON_CYTOPLASMIC_DOMAIN (PHOBIUS); NON_CYTOPLASMIC_DOMAIN (PHOBIUS); CYTOPLASMIC_DOMAIN (PHOBIUS); TRANSMEMBRANE (PHOBIUS); CYTOPLASMIC_DOMAIN (PHOBIUS); TRANSMEMBRANE (PHOBIUS); TRANSMEMBRANE (PHOBIUS); NON_CYTOPLASMIC_DOMAIN (PHOBIUS); CYTOPLASMIC_DOMAIN (PHOBIUS); TRANSMEMBRANE (PHOBIUS); IPR002223 (PROSITE_PROFILES); IPR002223 (PROSITE_PROFILES); IPR002223 (SUPERFAMILY); IPR002223 (SUPERFAMILY); TMhelix (TMHMM); TMhelix (TMHMM); TMhelix (TMHMM); TMhelix (TMHMM)
2173	301846_length_2721_cvg_13.3_tip_1_1	498	beta-galactosidase-like isoform x4	907	0	59.70%	0.425 Y	IPR001944 (PRINTS); IPR013781 (G3DSA:3.20.20.GENE3D); IPR008979 (G3DSA:2.60.120.GENE3D); IPR001944 (PFAM); PTHR23421:SF13 (PANTHER); IPR001944 (PANTHER); IPR019801 (PROSITE_PATTERNS); TRANSMEMBRANE (PHOBIUS); CYTOPLASMIC_DOMAIN (PHOBIUS); NON_CYTOPLASMIC_DOMAIN (PHOBIUS); IPR008979 (SUPERFAMILY); IPR017853 (SUPERFAMILY); TMhelix (TMHMM)
2174	301856_length_2732_cvg_6.5_tip_1_4	272	complement c1s subcomponent	911	3.70E-11	43.75%	0.106	IPR000859 (SMART); IPR000859 (G3DSA:2.60.120.GENE3D); IPR000859 (PFAM); CYTOPLASMIC_DOMAIN (PHOBIUS); TRANSMEMBRANE (PHOBIUS); NON_CYTOPLASMIC_DOMAIN (PHOBIUS); IPR000859 (PROSITE_PROFILES); IPR000859 (SUPERFAMILY); IPR000859 (SUPERFAMILY); TMhelix (TMHMM)
2175	301868_length_2755_cvg_2.8_tip_1_1	171	urokinase-type plasminogen activator	918	2.96E-61	48.00%	0.099	IPR002172 (PRINTS); IPR002172 (SMART); IPR001254 (SMART); IPR003609 (SMART); G3DSA:2.40.10.10 (GENE3D); IPR003014 (PFAM); G3DSA:2.40.10.10 (GENE3D); IPR001254 (PFAM); IPR002172 (PFAM); G3DSA:3.50.4.10 (GENE3D); IPR002172 (G3DSA:4.10.400.GENE3D); G3DSA:2.40.10.10 (GENE3D); PTHR24256 (PANTHER); IPR023415 (PROSITE_PATTERNS); IPR023415 (PROSITE_PATTERNS); IPR018114 (PROSITE_PATTERNS); IPR018114 (PROSITE_PATTERNS); IPR002172 (PROSITE_PROFILES); IPR003609 (PROSITE_PROFILES); IPR002172 (PROSITE_PROFILES); IPR003609 (PROSITE_PROFILES); IPR001254 (PROSITE_PROFILES); IPR002172 (PROSITE_PROFILES); IPR003609 (PROSITE_PROFILES); IPR003609 (PROSITE_PROFILES); IPR002172 (PROSITE_PROFILES); IPR009003 (SUPERFAMILY); IPR002172 (SUPERFAMILY); IPR002172 (SUPERFAMILY); IPR002172 (SUPERFAMILY); IPR002172 (SUPERFAMILY)
2176	301894_length_2783_cvg_7.9_tip_1_0	315	angiotensin-converting enzyme-like	928	1.04E-162	61.70%	0.276	Coil (COILS); IPR001548 (PRINTS); IPR001548 (PFAM); IPR001548 (PANTHER); TRANSMEMBRANE (PHOBIUS); CYTOPLASMIC_DOMAIN (PHOBIUS); NON_CYTOPLASMIC_DOMAIN (PHOBIUS); TRANSMEMBRANE (PHOBIUS); CYTOPLASMIC_DOMAIN (PHOBIUS); TRANSMEMBRANE (PHOBIUS); NON_CYTOPLASMIC_DOMAIN (PHOBIUS); CYTOPLASMIC_DOMAIN (PHOBIUS); TRANSMEMBRANE (PHOBIUS); SSF55486 (SUPERFAMILY); TMhelix (TMHMM); TMhelix (TMHMM); TMhelix (TMHMM); TMhelix (TMHMM)
2177	301900_length_2784_cvg_38.0_tip_1_2	1414	aael014548- partial	928	7.26E-130	88.20%	0.694 Y	IPR012336 (G3DSA:3.40.30.GENE3D); IPR019479 (PFAM); IPR000866 (PFAM); PTHR10681 (PANTHER); PTHR10681:SF101 (PANTHER); TRANSMEMBRANE (PHOBIUS); TRANSMEMBRANE (PHOBIUS); NON_CYTOPLASMIC_DOMAIN (PHOBIUS); SIGNAL_PEPTIDE (PHOBIUS); SIGNAL_PEPTIDE_H_REGION (PHOBIUS); SIGNAL_PEPTIDE_N_REGION (PHOBIUS); NON_CYTOPLASMIC_DOMAIN (PHOBIUS); TRANSMEMBRANE (PHOBIUS); CYTOPLASMIC_DOMAIN (PHOBIUS); CYTOPLASMIC_DOMAIN (PHOBIUS); SIGNAL_PEPTIDE_C_REGION (PHOBIUS); IPR012336 (PROSITE_PROFILES); SignalP-noTM (SIGNALP_GRAM_NEGATIVE); SignalP-noTM (SIGNALP_EUK); SignalP-TM (SIGNALP_GRAM_POSITIVE); IPR012336 (SUPERFAMILY); TMhelix (TMHMM); TMhelix (TMHMM); TMhelix (TMHMM)
2178	301902_length_2786_cvg_62.8_tip_1_1	2687	agap011476-pa-like protein	929	4.71E-74	44.80%	0.115	TRANSMEMBRANE (PHOBIUS); NON_CYTOPLASMIC_DOMAIN (PHOBIUS); CYTOPLASMIC_DOMAIN (PHOBIUS); TMhelix (TMHMM)
2179	301912_length_2792_cvg_40.1_tip_0_0	2385	map kinase-interacting serine threonine-protein kinase 1	931	0	81.40%	0.12	IPR002290 (SMART); G3DSA:3.30.200.20 (GENE3D); G3DSA:1.10.510.10 (GENE3D); IPR000719 (PFAM); PTHR24349 (PANTHER); IPR008271 (PROSITE_PATTERNS); CYTOPLASMIC_DOMAIN (PHOBIUS); TRANSMEMBRANE (PHOBIUS); NON_CYTOPLASMIC_DOMAIN (PHOBIUS); TRANSMEMBRANE (PHOBIUS); NON_CYTOPLASMIC_DOMAIN (PHOBIUS); IPR000719 (PROSITE_PROFILES); IPR011009 (SUPERFAMILY)

2180	301916_length_2796_cvg_54.7_tip_1_2	2335	pacifastin light chain	932	1.23E-51	42.30%	0.782	Y	IPR006552 (SMART); IPR008037 (PFAM); IPR020862 (PROSITE_PROFILES); IPR020862 (PROSITE_PROFILES); IPR020862 (PROSITE_PROFILES); IPR020862 (PROSITE_PROFILES); IPR020862 (PROSITE_PROFILES); IPR020862 (PROSITE_PROFILES); IPR020862 (PROSITE_PROFILES); IPR020862 (PROSITE_PROFILES); IPR001190 (PROSITE_PROFILES); IPR020862 (PROSITE_PROFILES); IPR020862 (PROSITE_PROFILES); IPR020862 (PROSITE_PROFILES); IPR020862 (PROSITE_PROFILES); IPR020862 (PROSITE_PROFILES); IPR020862 (PROSITE_PROFILES); IPR020862 (PROSITE_PROFILES); IPR020862 (PROSITE_PROFILES); IPR020862 (PROSITE_PROFILES); IPR020862 (PROSITE_PROFILES); IPR020862 (PROSITE_PROFILES); IPR020862 (PROSITE_PROFILES); IPR020862 (PROSITE_PROFILES); IPR020862 (PROSITE_PROFILES); SignalP-noTM (SIGNALP_EUK); IPR008037 (SUPERFAMILY)
2181	301920_length_2798_cvg_5.5_tip_1_2	228	protein canopy 4	932	9.21E-84	79.80%	0.219		IPR021852 (PFAM); PTHR15382 (PANTHER); CYTOPLASMIC_DOMAIN (PHOBIUS); TRANSMEMBRANE (PHOBIUS); NON_CYTOPLASMIC_DOMAIN (PHOBIUS); CYTOPLASMIC_DOMAIN (PHOBIUS); TRANSMEMBRANE (PHOBIUS); TRANSMEMBRANE (PHOBIUS); CYTOPLASMIC_DOMAIN (PHOBIUS); TRANSMEMBRANE (PHOBIUS); TRANSMEMBRANE (PHOBIUS); TRANSMEMBRANE (PHOBIUS); NON_CYTOPLASMIC_DOMAIN (PHOBIUS); NON_CYTOPLASMIC_DOMAIN (PHOBIUS); CYTOPLASMIC_DOMAIN (PHOBIUS); TRANSMEMBRANE (PHOBIUS); NON_CYTOPLASMIC_DOMAIN (PHOBIUS); TMhelix (TMHMM)
2182	301922_length_2798_cvg_25.5_tip_1_2	926	cathepsin l-associated protein	932	2.17E-77	61.10%	0.139		IPR000782 (SMART); IPR000782 (G3DSA:2.30.180.GENE3D); IPR000782 (G3DSA:2.30.180.GENE3D); IPR000782 (PFAM); PTHR10900 (PANTHER); CYTOPLASMIC_DOMAIN (PHOBIUS); TRANSMEMBRANE (PHOBIUS); NON_CYTOPLASMIC_DOMAIN (PHOBIUS); TRANSMEMBRANE (PHOBIUS); CYTOPLASMIC_DOMAIN (PHOBIUS); TRANSMEMBRANE (PHOBIUS); CYTOPLASMIC_DOMAIN (PHOBIUS); TRANSMEMBRANE (PHOBIUS); TRANSMEMBRANE (PHOBIUS); NON_CYTOPLASMIC_DOMAIN (PHOBIUS); TRANSMEMBRANE (PHOBIUS); CYTOPLASMIC_DOMAIN (PHOBIUS); NON_CYTOPLASMIC_DOMAIN (PHOBIUS); TRANSMEMBRANE (PHOBIUS); NON_CYTOPLASMIC_DOMAIN (PHOBIUS); IPR000782 (PROSITE_PROFILES); IPR000782 (PROSITE_PROFILES); IPR000782 (SUPERFAMILY); IPR000782 (SUPERFAMILY); TMhelix (TMHMM); TMhelix (TMHMM); TMhelix (TMHMM); TMhelix (TMHMM); TMhelix (TMHMM)
2183	301924_length_2801_cvg_16.3_tip_1_2	710	beta-mannosidase	933	0	63.30%	0.243		IPR013781 (G3DSA:3.20.20.GENE3D); IPR006104 (PFAM); IPR013812 (G3DSA:2.60.40.GENE3D); IPR006102 (PFAM); IPR006103 (PFAM); IPR008979 (G3DSA:2.60.120.GENE3D); PTHR10066 (PANTHER); IPR028369 (PTHR10066:PANTHER); IPR006102 (SUPERFAMILY); IPR008979 (SUPERFAMILY); IPR017853 (SUPERFAMILY); IPR006102 (SUPERFAMILY); IPR006102 (SUPERFAMILY)

2184	301934_length_2815_cvg_10.6_tip_1_5	387	zinc finger protein 239- partial	938	4.42E-72	55.60%	0.135	IPR015880 (SMART); IPR013087 (G3DSA:3.30.160.GENE3D); IPR013087 (G3DSA:3.30.160.GENE3D); PF13894 (PFAM); IPR013087 (G3DSA:3.30.160.GENE3D); IPR013087 (G3DSA:3.30.160.GENE3D); IPR013087 (G3DSA:3.30.160.GENE3D); PF13465 (PFAM); PTHR24409 (PANTHER); IPR007087 (PROSITE_PATTERNS); IPR007087 (PROSITE_PATTERNS); IPR007087 (PROSITE_PATTERNS); IPR007087 (PROSITE_PATTERNS); IPR007087 (PROSITE_PATTERNS); IPR007087 (PROSITE_PATTERNS); IPR007087 (PROSITE_PATTERNS); IPR007087 (PROSITE_PATTERNS); IPR007087 (PROSITE_PATTERNS); IPR007087 (PROSITE_PATTERNS); IPR007087 (PROSITE_PATTERNS); IPR007087 (PROSITE_PATTERNS); IPR007087 (PROSITE_PROFILES); IPR007087 (PROSITE_PROFILES); IPR007087 (PROSITE_PROFILES); IPR007087 (PROSITE_PROFILES); IPR007087 (PROSITE_PROFILES); IPR007087 (PROSITE_PROFILES); IPR007087 (PROSITE_PROFILES); IPR007087 (PROSITE_PROFILES); IPR007087 (PROSITE_PROFILES); IPR007087 (PROSITE_PROFILES); IPR007087 (PROSITE_PROFILES); IPR007087 (PROSITE_PROFILES); SSF57667 (SUPERFAMILY); SSF57667 (SUPERFAMILY); SSF57667 (SUPERFAMILY); SSF57667 (SUPERFAMILY); SSF57667 (SUPERFAMILY); SSF57667 (SUPERFAMILY)
2185	301938_length_2818_cvg_5.5_tip_1_3	246	dlp hdl-bgbp precursor	940	0	63.60%	0.727 Y	Coil (COILS); IPR001747 (SMART); IPR015816 (G3DSA:2.30.230.GENE3D); IPR001747 (PFAM); IPR015255 (PFAM); IPR015817 (G3DSA:2.20.50.GENE3D); IPR011030 (G3DSA:1.25.10.GENE3D); IPR015818 (G3DSA:2.20.80.GENE3D); PTHR23345:SF11 (PANTHER); PTHR23345 (PANTHER); SIGNAL_PEPTIDE_H_REGION (PHOBIUS); SIGNAL_PEPTIDE (PHOBIUS); SIGNAL_PEPTIDE_N_REGION (PHOBIUS); SIGNAL_PEPTIDE_C_REGION (PHOBIUS); NON_CYTOPLASMIC_DOMAIN (PHOBIUS); IPR001747 (PROSITE_PROFILES); SignalP-noTM (SIGNALP_EUK); IPR011030 (SUPERFAMILY); IPR015819 (SUPERFAMILY); IPR015819 (SUPERFAMILY); TMhelix (TMHMM)
2186	301942_length_2826_cvg_41.9_tip_1_1	3483	titin isoform x5	942	0	75.50%	0.099	IPR003598 (SMART); IPR003599 (SMART); IPR013098 (PFAM); IPR013783 (G3DSA:2.60.40.GENE3D); IPR013783 (G3DSA:2.60.40.GENE3D); IPR013783 (G3DSA:2.60.40.GENE3D); IPR013783 (G3DSA:2.60.40.GENE3D); PTHR19897 (PANTHER); PTHR19897:SF149 (PANTHER); CYTOPLASMIC_DOMAIN (PHOBIUS); CYTOPLASMIC_DOMAIN (PHOBIUS); TRANSMEMBRANE (PHOBIUS); NON_CYTOPLASMIC_DOMAIN (PHOBIUS); NON_CYTOPLASMIC_DOMAIN (PHOBIUS); TRANSMEMBRANE (PHOBIUS); NON_CYTOPLASMIC_DOMAIN (PHOBIUS); CYTOPLASMIC_DOMAIN (PHOBIUS); TRANSMEMBRANE (PHOBIUS); TRANSMEMBRANE (PHOBIUS); TRANSMEMBRANE (PHOBIUS); IPR007110 (PROSITE_PROFILES); IPR007110 (PROSITE_PROFILES); IPR007110 (PROSITE_PROFILES); IPR007110 (PROSITE_PROFILES); SSF48726 (SUPERFAMILY); SSF48726 (SUPERFAMILY); SSF48726 (SUPERFAMILY); SSF48726 (SUPERFAMILY); TMhelix (TMHMM); TMhelix (TMHMM); TMhelix (TMHMM)
2187	301948_length_2836_cvg_7.9_tip_1_1	326	angiotensin-converting enzyme	945	0	63.40%	0.155	IPR001548 (PRINTS); IPR001548 (PFAM); IPR001548 (PANTHER); SSF55486 (SUPERFAMILY); SSF55486 (SUPERFAMILY)
2188	301956_length_2849_cvg_9.3_tip_1_4	364	atp-dependent rna helicase ddx1	950	0	83.50%	0.332	IPR014001 (SMART); IPR001650 (SMART); IPR003877 (SMART); IPR011545 (PFAM); IPR001650 (PFAM); IPR003877 (PFAM); IPR027417 (G3DSA:3.40.50.GENE3D); IPR027417 (G3DSA:3.40.50.GENE3D); PTHR24031:SF211 (PANTHER); PTHR24031 (PANTHER); IPR014014 (PROSITE_PROFILES); IPR001870 (PROSITE_PROFILES); IPR014001 (PROSITE_PROFILES); IPR001650 (PROSITE_PROFILES); IPR027417 (SUPERFAMILY); IPR027417 (SUPERFAMILY); IPR013320 (SUPERFAMILY)
2189	301968_length_2860_cvg_30.4_tip_1_1	1296	carboxypeptidase q-like	953	0	75.80%	0.193	G3DSA:3.40.630.10 (GENE3D); IPR007484 (PFAM); PTHR12053 (PANTHER); NON_CYTOPLASMIC_DOMAIN (PHOBIUS); NON_CYTOPLASMIC_DOMAIN (PHOBIUS); TRANSMEMBRANE (PHOBIUS); TRANSMEMBRANE (PHOBIUS); NON_CYTOPLASMIC_DOMAIN (PHOBIUS); TRANSMEMBRANE (PHOBIUS); CYTOPLASMIC_DOMAIN (PHOBIUS); TRANSMEMBRANE (PHOBIUS); CYTOPLASMIC_DOMAIN (PHOBIUS); CYTOPLASMIC_DOMAIN (PHOBIUS); TRANSMEMBRANE (PHOBIUS); SSF53187 (SUPERFAMILY); TMhelix (TMHMM); TMhelix (TMHMM); TMhelix (TMHMM); TMhelix (TMHMM); TMhelix (TMHMM)
2190	301980_length_2882_cvg_33.8_tip_1_2	1149	muscle m-line assembly protein unc- partial	960	1.70E-45	49.40%	0.105	IPR003599 (SMART); IPR003598 (SMART); IPR013783 (G3DSA:2.60.40.GENE3D); IPR013098 (PFAM); IPR020675 (PANTHER); TRANSMEMBRANE (PHOBIUS); NON_CYTOPLASMIC_DOMAIN (PHOBIUS); CYTOPLASMIC_DOMAIN (PHOBIUS); NON_CYTOPLASMIC_DOMAIN (PHOBIUS); TRANSMEMBRANE (PHOBIUS); IPR007110 (PROSITE_PROFILES); SSF48726 (SUPERFAMILY)

2191	301998_length_2905_cvg_74.2_tip_1_0	10688	cathepsin d	969	0	80.50%	0.186		IPR001461 (PRINTS); IPR021109 (G3DSA:2.40.70.GENE3D); IPR021109 (G3DSA:2.40.70.GENE3D); IPR001461 (PFAM); IPR012848 (PFAM); PTHR13683:SF230 (PANTHER); IPR001461 (PANTHER); IPR001969 (PROSITE_PATTERNS); IPR001969 (PROSITE_PATTERNS); NON_CYTOPLASMIC_DOMAIN (PHOBIUS); TRANSMEMBRANE (PHOBIUS); CYTOPLASMIC_DOMAIN (PHOBIUS); TRANSMEMBRANE (PHOBIUS); NON_CYTOPLASMIC_DOMAIN (PHOBIUS); CYTOPLASMIC_DOMAIN (PHOBIUS); TRANSMEMBRANE (PHOBIUS); IPR021109 (SUPERFAMILY); TMhelix (TMHMM); TMhelix (TMHMM); TMhelix (TMHMM)
2192	302000_length_2906_cvg_49.4_tip_1_3	2066	low quality protein: mucin-19	969	1.35E-25	41.70%	0.162		TMhelix (TMHMM); TMhelix (TMHMM); TMhelix (TMHMM); TMhelix (TMHMM)
2193	302028_length_2934_cvg_21.5_tip_1_4	1071	villin-1-like isoform x1	978	0	66.30%	0.114		IPR007122 (PRINTS); IPR003128 (SMART); IPR007122 (SMART); IPR029006 (G3DSA:3.40.20.GENE3D); IPR003128 (G3DSA:1.10.950.GENE3D); IPR003128 (PFAM); IPR029006 (G3DSA:3.40.20.GENE3D); IPR029006 (G3DSA:3.40.20.GENE3D); IPR007123 (PFAM); IPR007122 (PANTHER); CYTOPLASMIC_DOMAIN (PHOBIUS); NON_CYTOPLASMIC_DOMAIN (PHOBIUS); TRANSMEMBRANE (PHOBIUS); IPR003128 (PROSITE_PROFILES); SSF55753 (SUPERFAMILY); SSF55753 (SUPERFAMILY); SSF55753 (SUPERFAMILY); IPR003128 (SUPERFAMILY); SSF55753 (SUPERFAMILY); SSF55753 (SUPERFAMILY); SSF55753 (SUPERFAMILY)
2194	302044_length_2946_cvg_27.1_tip_1_1	838	n-acetylated-alpha-linked acidic dipeptidase	982	0	60.00%	0.238		IPR003137 (PFAM); IPR007484 (PFAM); G3DSA:3.50.30.30 (GENE3D); IPR007365 (G3DSA:1.20.930.GENE3D); IPR007365 (PFAM); G3DSA:3.40.630.10 (GENE3D); PTHR10404 (PANTHER); CYTOPLASMIC_DOMAIN (PHOBIUS); CYTOPLASMIC_DOMAIN (PHOBIUS); TRANSMEMBRANE (PHOBIUS); NON_CYTOPLASMIC_DOMAIN (PHOBIUS); TRANSMEMBRANE (PHOBIUS); SSF53187 (SUPERFAMILY); SSF52025 (SUPERFAMILY); IPR007365 (SUPERFAMILY); TMhelix (TMHMM); TMhelix (TMHMM); TMhelix (TMHMM)
2195	302052_length_2954_cvg_8.0_tip_1_5	299	ferric-chelate reductase 1 homolog	984	2.10E-161	57.80%	0.751	Y	IPR006593 (SMART); IPR005018 (SMART); IPR005018 (PFAM); IPR004877 (PFAM); IPR002861 (PFAM); PTHR23130:SF70 (PANTHER); PTHR23130 (PANTHER); NON_CYTOPLASMIC_DOMAIN (PHOBIUS); TRANSMEMBRANE (PHOBIUS); SIGNAL_PEPTIDE_H_REGION (PHOBIUS); SIGNAL_PEPTIDE (PHOBIUS); SIGNAL_PEPTIDE_N_REGION (PHOBIUS); CYTOPLASMIC_DOMAIN (PHOBIUS); NON_CYTOPLASMIC_DOMAIN (PHOBIUS); NON_CYTOPLASMIC_DOMAIN (PHOBIUS); TRANSMEMBRANE (PHOBIUS); CYTOPLASMIC_DOMAIN (PHOBIUS); TRANSMEMBRANE (PHOBIUS); CYTOPLASMIC_DOMAIN (PHOBIUS); CYTOPLASMIC_DOMAIN (PHOBIUS); TRANSMEMBRANE (PHOBIUS); NON_CYTOPLASMIC_DOMAIN (PHOBIUS); TRANSMEMBRANE (PHOBIUS); TRANSMEMBRANE (PHOBIUS); SIGNAL_PEPTIDE_C_REGION (PHOBIUS); TRANSMEMBRANE (PHOBIUS); NON_CYTOPLASMIC_DOMAIN (PHOBIUS); TRANSMEMBRANE (PHOBIUS); IPR002861 (PROSITE_PROFILES); IPR005018 (PROSITE_PROFILES); IPR006593 (PROSITE_PROFILES); SignalP-TM (SIGNALP_EUK); TMhelix (TMHMM); TMhelix (TMHMM); TMhelix (TMHMM); TMhelix (TMHMM); TMhelix (TMHMM); TMhelix (TMHMM); TMhelix (TMHMM); TMhelix (TMHMM); TMhelix (TMHMM); TMhelix (TMHMM)
2196	302058_length_2961_cvg_25.6_tip_1_4	1175	beta-lactamase-like protein 2-like	987	1.86E-55	50.40%	0.381	Y	IPR012338 (G3DSA:3.40.710.GENE3D); IPR001466 (PFAM); PTHR22935 (PANTHER); PTHR22935:SF64 (PANTHER); NON_CYTOPLASMIC_DOMAIN (PHOBIUS); TRANSMEMBRANE (PHOBIUS); CYTOPLASMIC_DOMAIN (PHOBIUS); IPR012338 (SUPERFAMILY)
2197	302068_length_2971_cvg_43.0_tip_1_4	2060	hypothetical protein DAPPUDRAFT_200882	990	0	60.50%	0.116		IPR018497 (PRINTS); IPR008753 (PFAM); IPR018497 (PFAM); IPR024079 (G3DSA:3.40.390.GENE3D); PTHR11733:SF111 (PANTHER); IPR000718 (PANTHER); NON_CYTOPLASMIC_DOMAIN (PHOBIUS); CYTOPLASMIC_DOMAIN (PHOBIUS); TRANSMEMBRANE (PHOBIUS); SSF55486 (SUPERFAMILY); TMhelix (TMHMM)
2201	302086_length_3001_cvg_39.8_tip_1_1	2273	peroxidasin homolog	991	8.66E-119	54.50%	0.418	Y	Coil (COILS); IPR019791 (PRINTS); IPR019791 (G3DSA:1.10.640.GENE3D); IPR019791 (PFAM); PTHR11475 (PANTHER); CYTOPLASMIC_DOMAIN (PHOBIUS); TRANSMEMBRANE (PHOBIUS); NON_CYTOPLASMIC_DOMAIN (PHOBIUS); CYTOPLASMIC_DOMAIN (PHOBIUS); NON_CYTOPLASMIC_DOMAIN (PHOBIUS); TRANSMEMBRANE (PHOBIUS); TRANSMEMBRANE (PHOBIUS); IPR019791 (PROSITE_PROFILES); IPR010255 (SUPERFAMILY)

2198	302074_length_2976_cvg_37.4_tip_1_5	1404	proactivator polypeptide-like	992	0	54.80%	0.307	IPR008373 (PRINTS); IPR008139 (SMART); IPR003119 (SMART); IPR011001 (G3DSA:1.10.225.GENE3D); IPR011001 (G3DSA:1.10.225.GENE3D); IPR003119 (PFAM); IPR007856 (PFAM); IPR008138 (PFAM); IPR011001 (G3DSA:1.10.225.GENE3D); IPR011001 (G3DSA:1.10.225.GENE3D); PTHR11480:SF3 (PANTHER); PTHR11480 (PANTHER); TRANSMEMBRANE (PHOBIUS); CYTOPLASMIC_DOMAIN (PHOBIUS); TRANSMEMBRANE (PHOBIUS); NON_CYTOPLASMIC_DOMAIN (PHOBIUS); CYTOPLASMIC_DOMAIN (PHOBIUS); IPR003119 (PROSITE_PROFILES); IPR008139 (PROSITE_PROFILES); IPR008139 (PROSITE_PROFILES); IPR008139 (PROSITE_PROFILES); IPR008139 (PROSITE_PROFILES); IPR008139 (PROSITE_PROFILES); IPR008139 (PROSITE_PROFILES); IPR003119 (PROSITE_PROFILES); IPR008139 (PROSITE_PROFILES); IPR011001 (SUPERFAMILY); IPR011001 (SUPERFAMILY); IPR011001 (SUPERFAMILY); IPR011001 (SUPERFAMILY); IPR011001 (SUPERFAMILY); IPR011001 (SUPERFAMILY); IPR011001 (SUPERFAMILY); TMhelix (TMHMM)
2199	302076_length_2977_cvg_20.8_tip_1_5	673	sucrase- intestinal-like	992	0	61.70%	0.586 Y	IPR000322 (PFAM); PTHR22762 (PANTHER); PTHR22762:SF7 (PANTHER); IPR030458 (PROSITE_PATTERNS); IPR030459 (PROSITE_PATTERNS); SIGNAL_PEPTIDE_H_REGION (PHOBIUS); SIGNAL_PEPTIDE_C_REGION (PHOBIUS); NON_CYTOPLASMIC_DOMAIN (PHOBIUS); SIGNAL_PEPTIDE (PHOBIUS); SIGNAL_PEPTIDE_N_REGION (PHOBIUS); SignalP-noTM (SIGNALP_EUK); SSF51011 (SUPERFAMILY); IPR011013 (SUPERFAMILY); IPR017853 (SUPERFAMILY)
2200	302078_length_2981_cvg_44.7_tip_1_2	2064	pdz and lim domain protein zasp-like	993	6.42E-35	53.40%	0.103	IPR001478 (SMART); IPR001478 (G3DSA:2.30.42.GENE3D); IPR001478 (PFAM); PTHR24214:SF29 (PANTHER); PTHR24214 (PANTHER); TRANSMEMBRANE (PHOBIUS); CYTOPLASMIC_DOMAIN (PHOBIUS); NON_CYTOPLASMIC_DOMAIN (PHOBIUS); IPR001478 (PROSITE_PROFILES); IPR001478 (SUPERFAMILY)
2202	302092_length_3004_cvg_46.0_tip_1_4	1637	meprin a	1001	8.82E-72	50.10%	0.789 Y	IPR001506 (PRINTS); IPR006026 (SMART); IPR000859 (SMART); IPR001506 (PFAM); IPR000859 (PFAM); IPR000859 (G3DSA:2.60.120.GENE3D); IPR000859 (G3DSA:2.60.120.GENE3D); IPR024079 (G3DSA:3.40.390.GENE3D); PTHR10127 (PANTHER); PTHR10127:SF580 (PANTHER); TRANSMEMBRANE (PHOBIUS); NON_CYTOPLASMIC_DOMAIN (PHOBIUS); SIGNAL_PEPTIDE_H_REGION (PHOBIUS); SIGNAL_PEPTIDE_N_REGION (PHOBIUS); CYTOPLASMIC_DOMAIN (PHOBIUS); SIGNAL_PEPTIDE (PHOBIUS); TRANSMEMBRANE (PHOBIUS); SIGNAL_PEPTIDE_C_REGION (PHOBIUS); NON_CYTOPLASMIC_DOMAIN (PHOBIUS); IPR000859 (PROSITE_PROFILES); SignalP-noTM (SIGNALP_EUK); SSF55486 (SUPERFAMILY); IPR000859 (SUPERFAMILY)
2203	302114_length_3022_cvg_42.0_tip_1_5	1756	glucose dehydrogenase	1007	0	68.40%	0.141	G3DSA:3.50.50.60 (GENE3D); IPR007867 (PFAM); IPR000172 (PFAM); PTHR11552 (PANTHER); PTHR11552:SF77 (PANTHER); IPR000172 (PROSITE_PATTERNS); CYTOPLASMIC_DOMAIN (PHOBIUS); TRANSMEMBRANE (PHOBIUS); CYTOPLASMIC_DOMAIN (PHOBIUS); NON_CYTOPLASMIC_DOMAIN (PHOBIUS); TRANSMEMBRANE (PHOBIUS); SSF54373 (SUPERFAMILY); SSF51905 (SUPERFAMILY); TMhelix (TMHMM)
2204	302118_length_3030_cvg_15.1_tip_1_1	515	basement membrane-specific heparan sulfate proteoglycan core protein	1010	0	70.20%	0.109	IPR001791 (SMART); IPR001881 (SMART); IPR000742 (SMART); IPR003599 (SMART); IPR003598 (SMART); PF13895 (PFAM); IPR000742 (PFAM); IPR001791 (PFAM); IPR013320 (G3DSA:2.60.120.GENE3D); IPR013320 (G3DSA:2.60.120.GENE3D); IPR013320 (G3DSA:2.60.120.GENE3D); G3DSA:2.10.25.10 (GENE3D); IPR001791 (PFAM); G3DSA:2.10.25.10 (GENE3D); G3DSA:2.10.25.10 (GENE3D); IPR013783 (G3DSA:2.60.40.GENE3D); PTHR10574:SF247 (PANTHER); PTHR10574 (PANTHER); IPR013032 (PROSITE_PATTERNS); IPR013032 (PROSITE_PATTERNS); IPR000152 (PROSITE_PATTERNS); IPR013032 (PROSITE_PATTERNS); IPR013032 (PROSITE_PATTERNS); IPR013032 (PROSITE_PATTERNS); IPR013032 (PROSITE_PATTERNS); NON_CYTOPLASMIC_DOMAIN (PHOBIUS); TRANSMEMBRANE (PHOBIUS); CYTOPLASMIC_DOMAIN (PHOBIUS); IPR001791 (PROSITE_PROFILES); IPR000742 (PROSITE_PROFILES); IPR001791 (PROSITE_PROFILES); IPR000742 (PROSITE_PROFILES); IPR001791 (PROSITE_PROFILES); IPR000742 (PROSITE_PROFILES); IPR000742 (PROSITE_PROFILES); IPR007110 (PROSITE_PROFILES); SSF57196 (SUPERFAMILY); SSF48726 (SUPERFAMILY); IPR013320 (SUPERFAMILY); IPR013320 (SUPERFAMILY); IPR013320 (SUPERFAMILY); SSF57196 (SUPERFAMILY); TMhelix (TMHMM); TMhelix (TMHMM); TMhelix (TMHMM)

2205	302124_length_3038_cvg_34.3_tip_1_2	1853	protein ambp	1012	7.43E-27	55.30%	0.153	IPR002223 (PRINTS); IPR002223 (SMART); IPR002223 (G3DSA:4.10.410.GENE3D); IPR002223 (G3DSA:4.10.410.GENE3D); IPR002223 (PFAM); PTHR10083 (PANTHER); IPR020901 (PROSITE_PATTERNS); IPR020901 (PROSITE_PATTERNS); TRANSMEMBRANE (PHOBIUS); CYTOPLASMIC_DOMAIN (PHOBIUS); NON_CYTOPLASMIC_DOMAIN (PHOBIUS); IPR002223 (PROSITE_PROFILES); IPR002223 (PROSITE_PROFILES); IPR002223 (SUPERFAMILY); IPR002223 (SUPERFAMILY); TMhelix (TMHMM)
2206	302128_length_3046_cvg_22.3_tip_1_5	931	low quality protein: apolipophorins-like	1015	1.23E-38	40.50%	0.112	no IPS match
2207	302134_length_3047_cvg_12.6_tip_1_3	553	cadherin egf lag seven-pass g-type receptor partial	1016	3.59E-108	42.90%	0.16	IPR000203 (SMART); IPR000832 (PFAM); IPR000203 (PFAM); IPR022624 (PFAM); PTHR12011 (PANTHER); PTHR12011:SF74 (PANTHER); CYTOPLASMIC_DOMAIN (PHOBIUS); NON_CYTOPLASMIC_DOMAIN (PHOBIUS); NON_CYTOPLASMIC_DOMAIN (PHOBIUS); TRANSMEMBRANE (PHOBIUS); TRANSMEMBRANE (PHOBIUS); CYTOPLASMIC_DOMAIN (PHOBIUS); NON_CYTOPLASMIC_DOMAIN (PHOBIUS); TRANSMEMBRANE (PHOBIUS); NON_CYTOPLASMIC_DOMAIN (PHOBIUS); CYTOPLASMIC_DOMAIN (PHOBIUS); TRANSMEMBRANE (PHOBIUS); TRANSMEMBRANE (PHOBIUS); TRANSMEMBRANE (PHOBIUS); CYTOPLASMIC_DOMAIN (PHOBIUS); TRANSMEMBRANE (PHOBIUS); IPR000203 (PROSITE_PROFILES); IPR017981 (PROSITE_PROFILES); SSF81321 (SUPERFAMILY); IPR013320 (SUPERFAMILY); TMhelix (TMHMM); TMhelix (TMHMM); TMhelix (TMHMM); TMhelix (TMHMM); TMhelix (TMHMM); TMhelix (TMHMM); TMhelix (TMHMM)
2208	302136_length_3048_cvg_11.9_tip_1_4	352	agap000560-pa-like protein	1016	0	77.80%	0.174	IPR003593 (SMART); IPR005936 (TIGRFAM); IPR000642 (PFAM); IPR003959 (PFAM); G3DSA:1.10.8.60 (GENE3D); IPR027417 (G3DSA:3.40.50.GENE3D); PTHR23076:SF47 (PANTHER); PTHR23076 (PANTHER); NON_CYTOPLASMIC_DOMAIN (PHOBIUS); TRANSMEMBRANE (PHOBIUS); TRANSMEMBRANE (PHOBIUS); NON_CYTOPLASMIC_DOMAIN (PHOBIUS); CYTOPLASMIC_DOMAIN (PHOBIUS); CYTOPLASMIC_DOMAIN (PHOBIUS); TRANSMEMBRANE (PHOBIUS); IPR005936 (HAMAP); IPR027417 (SUPERFAMILY); SSF140990 (SUPERFAMILY); TMhelix (TMHMM); TMhelix (TMHMM)
2209	302138_length_3048_cvg_15.1_tip_1_2	542	neutral alpha-glucosidase ab-like	1016	0	71.60%	0.228	IPR000322 (PFAM); IPR025887 (PFAM); PTHR22762 (PANTHER); PTHR22762:SF54 (PANTHER); IPR011013 (SUPERFAMILY); IPR017853 (SUPERFAMILY); SSF51011 (SUPERFAMILY)
2210	302154_length_3070_cvg_29.0_tip_1_2	998	von willebrand domain-containing protein	1023	0	57.60%	0.127	Coil (COILS); IPR013694 (SMART); IPR002035 (SMART); IPR013694 (PFAM); IPR002035 (G3DSA:3.40.50.GENE3D); PF13768 (PFAM); PTHR10338 (PANTHER); PTHR10338:SF95 (PANTHER); IPR002035 (PROSITE_PROFILES); IPR013694 (PROSITE_PROFILES); IPR002035 (SUPERFAMILY)
2211	302156_length_3071_cvg_8.4_tip_1_2	368	wd repeat-containing protein 3	1023	0	74.00%	0.710 Y	Coil (COILS); IPR020472 (PRINTS); IPR001680 (SMART); IPR015943 (G3DSA:2.130.10.GENE3D); IPR007148 (PFAM); IPR015943 (G3DSA:2.130.10.GENE3D); IPR001680 (PFAM); PTHR19853 (PANTHER); PTHR19853:SF0 (PANTHER); IPR019775 (PROSITE_PATTERNS); IPR019775 (PROSITE_PATTERNS); IPR019775 (PROSITE_PATTERNS); IPR001680 (PROSITE_PROFILES); IPR001680 (PROSITE_PROFILES); IPR001680 (PROSITE_PROFILES); IPR001680 (PROSITE_PROFILES); IPR001680 (PROSITE_PROFILES); IPR001680 (PROSITE_PROFILES); IPR001680 (PROSITE_PROFILES); IPR001680 (PROSITE_PROFILES); IPR017986 (PROSITE_PROFILES); IPR001680 (PROSITE_PROFILES); SignalP-noTM (SIGNALP_EUK); IPR017986 (SUPERFAMILY); IPR011044 (SUPERFAMILY)
2212	302178_length_3109_cvg_21.8_tip_1_1	798	hypoxia up-regulated protein 1 isoform x1	1036	0	68.10%	0.112	Coil (COILS); Coil (COILS); Coil (COILS); IPR013126 (PRINTS); G3DSA:3.90.640.10 (GENE3D); IPR013126 (PFAM); IPR029047 (G3DSA:2.60.34.GENE3D); IPR029048 (G3DSA:1.20.1270.GENE3D); G3DSA:3.30.420.40 (GENE3D); PTHR19375 (PANTHER); PTHR19375:SF90 (PANTHER); SSF53067 (SUPERFAMILY); IPR029048 (SUPERFAMILY)
2213	302184_length_3120_cvg_30.8_tip_1_2	1603	low-density lipoprotein receptor-related protein 6	1040	5.09E-13	46.40%	0.142	IPR002172 (PRINTS); IPR000998 (SMART); IPR002172 (SMART); IPR002172 (G3DSA:4.10.400.GENE3D); IPR002172 (PFAM); IPR002172 (G3DSA:4.10.400.GENE3D); IPR000998 (PFAM); PTHR10529 (PANTHER); PTHR10529:SF208 (PANTHER); IPR002172 (PROSITE_PROFILES); IPR000998 (PROSITE_PROFILES); IPR000998 (PROSITE_PROFILES); IPR000998 (PROSITE_PROFILES); IPR000998 (PROSITE_PROFILES); IPR002172 (PROSITE_PROFILES); IPR013320 (SUPERFAMILY); IPR002172 (SUPERFAMILY); IPR013320 (SUPERFAMILY); IPR002172 (SUPERFAMILY); IPR013320 (SUPERFAMILY); IPR013320 (SUPERFAMILY)

2214	302186_length_3125_cvg_64.6_tip_0_3	36669	e3 ubiquitin-protein ligase partial	1042	0	53.20%	0.104	IPR001841 (SMART); IPR001478 (SMART); IPR013083 (G3DSA:3.30.40.GENE3D); PF02176 (PFAM); IPR001478 (PFAM); IPR001478 (G3DSA:2.30.42.GENE3D); PF13920 (PFAM); IPR013323 (G3DSA:3.90.890.GENE3D); IPR001478 (G3DSA:2.30.42.GENE3D); PTHR19964 (PANTHER); IPR017907 (PROSITE_PATTERNS); NON_CYTOPLASMIC_DOMAIN (PHOBIUS); TRANSMEMBRANE (PHOBIUS); TRANSMEMBRANE (PHOBIUS); NON_CYTOPLASMIC_DOMAIN (PHOBIUS); CYTOPLASMIC_DOMAIN (PHOBIUS); IPR001478 (PROSITE_PROFILES); IPR001478 (PROSITE_PROFILES); IPR001293 (PROSITE_PROFILES); IPR001841 (PROSITE_PROFILES); IPR001478 (PROSITE_PROFILES); IPR001478 (PROSITE_PROFILES); IPR001478 (SUPERFAMILY); IPR001478 (SUPERFAMILY); IPR008974 (SUPERFAMILY); SSF57850 (SUPERFAMILY); IPR001478 (SUPERFAMILY); IPR001478 (SUPERFAMILY); TMhelix (TMHMM); TMhelix (TMHMM); TMhelix (TMHMM)
2215	302196_length_3136_cvg_25.8_tip_1_0	942	a disintegrin and metalloproteinase with thrombospondin motifs 1 isoform x1	1046	4.28E-134	70.50%	0.107	IPR024079 (G3DSA:3.40.390.GENE3D); PF13583 (PFAM); PTHR13723 (PANTHER); PTHR13723:SF148 (PANTHER); NON_CYTOPLASMIC_DOMAIN (PHOBIUS); TRANSMEMBRANE (PHOBIUS); TRANSMEMBRANE (PHOBIUS); NON_CYTOPLASMIC_DOMAIN (PHOBIUS); CYTOPLASMIC_DOMAIN (PHOBIUS); IPR001590 (PROSITE_PROFILES); SSF55486 (SUPERFAMILY); TMhelix (TMHMM); TMhelix (TMHMM)
2220	302242_length_3182_cvg_22.2_tip_1_2	1103	transitional endoplasmic reticulum atpase ter94	1046	0	94.80%	0.127	IPR003593 (SMART); IPR004201 (SMART); IPR003338 (SMART); IPR005938 (TIGRFAM); IPR027417 (G3DSA:3.40.50.GENE3D); IPR029067 (G3DSA:3.10.330.GENE3D); IPR015415 (PFAM); G3DSA:1.10.8.60 (GENE3D); G3DSA:2.40.40.20 (GENE3D); IPR004201 (PFAM); IPR003338 (PFAM); IPR003959 (PFAM); IPR027417 (G3DSA:3.40.50.GENE3D); PTHR23077 (PANTHER); PTHR23077:SF69 (PANTHER); IPR003960 (PROSITE_PATTERNS); IPR003960 (PROSITE_PATTERNS); IPR029067 (SUPERFAMILY); IPR027417 (SUPERFAMILY); IPR027417 (SUPERFAMILY); IPR009010 (SUPERFAMILY)
2216	302204_length_3144_cvg_14.6_tip_1_4	575	a chain structural studies of protein tyrosine phosphatase beta catalytic domain in complex with inhibitors	1048	1.21E-75	57.90%	0.102	IPR000242 (PRINTS); IPR003595 (SMART); IPR000242 (SMART); IPR003961 (SMART); IPR029021 (G3DSA:3.90.190.GENE3D); IPR013783 (G3DSA:2.60.40.GENE3D); IPR013783 (G3DSA:2.60.40.GENE3D); IPR003961 (PFAM); IPR013783 (G3DSA:2.60.40.GENE3D); IPR000242 (PFAM); IPR013783 (G3DSA:2.60.40.GENE3D); PTHR19134 (PANTHER); IPR016130 (PROSITE_PATTERNS); TRANSMEMBRANE (PHOBIUS); NON_CYTOPLASMIC_DOMAIN (PHOBIUS); CYTOPLASMIC_DOMAIN (PHOBIUS); IPR003961 (PROSITE_PROFILES); IPR003961 (PROSITE_PROFILES); IPR003961 (PROSITE_PROFILES); IPR003961 (PROSITE_PROFILES); IPR000387 (PROSITE_PROFILES); IPR000242 (PROSITE_PROFILES); IPR003961 (SUPERFAMILY); IPR029021 (SUPERFAMILY); IPR003961 (SUPERFAMILY); IPR003961 (SUPERFAMILY)
2217	302216_length_3162_cvg_8.6_tip_1_1	352	serine threonine-protein kinase pim-3	1054	2.50E-128	76.90%	0.107	IPR002290 (SMART); IPR000719 (PFAM); G3DSA:1.10.510.10 (GENE3D); G3DSA:3.30.200.20 (GENE3D); PTHR22984:SF1 (PANTHER); PTHR22984 (PANTHER); IPR008271 (PROSITE_PATTERNS); IPR017441 (PROSITE_PATTERNS); CYTOPLASMIC_DOMAIN (PHOBIUS); CYTOPLASMIC_DOMAIN (PHOBIUS); TRANSMEMBRANE (PHOBIUS); NON_CYTOPLASMIC_DOMAIN (PHOBIUS); TRANSMEMBRANE (PHOBIUS); CYTOPLASMIC_DOMAIN (PHOBIUS); NON_CYTOPLASMIC_DOMAIN (PHOBIUS); TRANSMEMBRANE (PHOBIUS); TRANSMEMBRANE (PHOBIUS); NON_CYTOPLASMIC_DOMAIN (PHOBIUS); TRANSMEMBRANE (PHOBIUS); TRANSMEMBRANE (PHOBIUS); NON_CYTOPLASMIC_DOMAIN (PHOBIUS); IPR000719 (PROSITE_PROFILES); IPR011009 (SUPERFAMILY); TMhelix (TMHMM); TMhelix (TMHMM); TMhelix (TMHMM); TMhelix (TMHMM); TMhelix (TMHMM); TMhelix (TMHMM); TMhelix (TMHMM); TMhelix (TMHMM); TMhelix (TMHMM)
2218	302228_length_3169_cvg_55.9_tip_1_5	3632	cell surface protein	1056	1.08E-12	44.30%	0.179	TRANSMEMBRANE (PHOBIUS); TRANSMEMBRANE (PHOBIUS); NON_CYTOPLASMIC_DOMAIN (PHOBIUS); CYTOPLASMIC_DOMAIN (PHOBIUS); TRANSMEMBRANE (PHOBIUS); NON_CYTOPLASMIC_DOMAIN (PHOBIUS); CYTOPLASMIC_DOMAIN (PHOBIUS); TRANSMEMBRANE (PHOBIUS); CYTOPLASMIC_DOMAIN (PHOBIUS)
2219	302234_length_3171_cvg_6.1_tip_1_4	261	usher syndrome type-1g protein homolog	1057	5.35E-132	62.20%	0.11	Coil (COILS); IPR002110 (SMART); IPR001660 (SMART); IPR020683 (G3DSA:1.25.40.GENE3D); IPR013761 (G3DSA:1.10.150.GENE3D); IPR021129 (PFAM); IPR020683 (PFAM); PTHR24161:SF6 (PANTHER); PTHR24161 (PANTHER); IPR002110 (PROSITE_PROFILES); IPR002110 (PROSITE_PROFILES); IPR020683 (PROSITE_PROFILES); IPR013761 (SUPERFAMILY); IPR020683 (SUPERFAMILY)

2221	302244_length_3182_cvg_51.1_tip_1_0	2265	transforming growth factor-beta-induced protein ig-h3	1061	0	56.20%	0.11	Coil (COILS); IPR000782 (SMART); IPR000782 (G3DSA:2.30.180.GENE3D); IPR000782 (PFAM); PTHR10900 (PANTHER); PTHR10900:SF73 (PANTHER); NON_CYTOPLASMIC_DOMAIN (PHOBIUS); CYTOPLASMIC_DOMAIN (PHOBIUS); TRANSMEMBRANE (PHOBIUS); CYTOPLASMIC_DOMAIN (PHOBIUS); NON_CYTOPLASMIC_DOMAIN (PHOBIUS); TRANSMEMBRANE (PHOBIUS); TRANSMEMBRANE (PHOBIUS); IPR000782 (PROSITE_PROFILES); IPR000782 (PROSITE_PROFILES); IPR000782 (PROSITE_PROFILES); IPR000782 (PROSITE_PROFILES); IPR000782 (SUPERFAMILY); IPR000782 (SUPERFAMILY); IPR000782 (SUPERFAMILY); IPR000782 (SUPERFAMILY)
2222	302250_length_3191_cvg_34.7_tip_0_0	1777	leucine-rich repeat-containing protein ddb_g0290503 isoform x3	1064	1.87E-141	63.50%	0.1	Coil (COILS); Coil (COILS); Coil (COILS); Coil (COILS); Coil (COILS); Coil (COILS); Coil (COILS); Coil (COILS); Coil (COILS); Coil (COILS); Coil (COILS); IPR027881 (PFAM); PTHR15742 (PANTHER); PTHR15742:SF0 (PANTHER)
2223	302258_length_3208_cvg_33.3_tip_1_3	1686	l-aminoadipate-semialdehyde dehydrogenase-like	1070	0	66.30%	0.098	IPR002347 (PRINTS); IPR002198 (PRINTS); IPR013968 (SMART); G3DSA:3.30.300.30 (GENE3D); IPR016040 (G3DSA:3.40.50.GENE3D); IPR009081 (G3DSA:1.10.1200.GENE3D); IPR009081 (PFAM); IPR013120 (PFAM); IPR002198 (PFAM); IPR010080 (TIGRFAM); IPR016040 (G3DSA:3.40.50.GENE3D); PTHR24095:SF141 (PANTHER); PTHR24095 (PANTHER); IPR020904 (PROSITE_PATTERNS); CYTOPLASMIC_DOMAIN (PHOBIUS); NON_CYTOPLASMIC_DOMAIN (PHOBIUS); NON_CYTOPLASMIC_DOMAIN (PHOBIUS); TRANSMEMBRANE (PHOBIUS); TRANSMEMBRANE (PHOBIUS); TRANSMEMBRANE (PHOBIUS); CYTOPLASMIC_DOMAIN (PHOBIUS); IPR009081 (PROSITE_PROFILES); SSF51735 (SUPERFAMILY); IPR009081 (SUPERFAMILY); SSF51735 (SUPERFAMILY); TMhelix (TMHMM); TMhelix (TMHMM)
2224	302260_length_3210_cvg_65.0_tip_0_1	14644	von willebrand factor like partial	1070	6.05E-18	40.30%	0.108	no IPS match
2225	302270_length_3223_cvg_8.0_tip_1_1	296	zinc finger protein 850-like	1074	1.15E-176	48.20%	0.103	IPR015880 (SMART); IPR013087 (G3DSA:3.30.160.GENE3D); IPR013087 (G3DSA:3.30.160.GENE3D); IPR007087 (PFAM); PF13894 (PFAM); PF13465 (PFAM); IPR013087 (G3DSA:3.30.160.GENE3D); IPR013087 (G3DSA:3.30.160.GENE3D); IPR013087 (G3DSA:3.30.160.GENE3D); PTHR24409 (PANTHER); PTHR24409:SF14 (PANTHER); IPR007087 (PROSITE_PATTERNS); IPR007087 (PROSITE_PATTERNS); IPR007087 (PROSITE_PATTERNS); IPR007087 (PROSITE_PATTERNS); IPR007087 (PROSITE_PATTERNS); IPR007087 (PROSITE_PATTERNS); IPR007087 (PROSITE_PATTERNS); IPR007087 (PROSITE_PATTERNS); IPR007087 (PROSITE_PATTERNS); IPR007087 (PROSITE_PATTERNS); IPR007087 (PROSITE_PATTERNS); IPR007087 (PROSITE_PATTERNS); IPR007087 (PROSITE_PATTERNS); IPR007087 (PROSITE_PATTERNS); IPR007087 (PROSITE_PATTERNS); IPR007087 (PROSITE_PATTERNS); IPR007087 (PROSITE_PROFILES); IPR007087 (PROSITE_PROFILES); IPR007087 (PROSITE_PROFILES); IPR007087 (PROSITE_PROFILES); IPR007087 (PROSITE_PROFILES); IPR007087 (PROSITE_PROFILES); IPR007087 (PROSITE_PROFILES); IPR007087 (PROSITE_PROFILES); IPR007087 (PROSITE_PROFILES); IPR007087 (PROSITE_PROFILES); IPR007087 (PROSITE_PROFILES); IPR007087 (PROSITE_PROFILES); IPR007087 (PROSITE_PROFILES); IPR007087 (PROSITE_PROFILES); SSF57667 (SUPERFAMILY); SSF57667 (SUPERFAMILY); SSF57667 (SUPERFAMILY); SSF57667 (SUPERFAMILY); SSF57667 (SUPERFAMILY); SSF57667 (SUPERFAMILY); SSF57667 (SUPERFAMILY); SSF57667 (SUPERFAMILY)
2226	302288_length_3273_cvg_35.2_tip_1_2	1847	exosporium leader peptide domain protein	1091	1.32E-69	43.90%	0.156	NON_CYTOPLASMIC_DOMAIN (PHOBIUS); CYTOPLASMIC_DOMAIN (PHOBIUS); TRANSMEMBRANE (PHOBIUS); TMhelix (TMHMM)
2227	302288_length_3273_cvg_35.2_tip_1_5	1847	muscle m-line assembly protein unc-89	1091	3.74E-87	80.50%	0.114	Coil (COILS); IPR001849 (SMART); IPR011993 (G3DSA:2.30.29.GENE3D); IPR000219 (PFAM); IPR000219 (G3DSA:1.20.900.GENE3D); IPR000219 (PROSITE_PROFILES); IPR001849 (PROSITE_PROFILES); IPR000219 (SUPERFAMILY); SSF50729 (SUPERFAMILY)

2228	302296_length_3279_cvg_11.2_tip_1_3	380	low quality protein: titin-like	1093	0	82.00%	0.102	Coil (COILS); IPR003598 (SMART); IPR003599 (SMART); IPR013098 (PFAM); IPR013783 (G3DSA:2.60.40.GENE3D); IPR013783 (G3DSA:2.60.40.GENE3D); IPR013783 (G3DSA:2.60.40.GENE3D); IPR010939 (PFAM); IPR013783 (G3DSA:2.60.40.GENE3D); PTHR19897:SF149 (PANTHER); PTHR19897 (PANTHER); IPR007110 (PROSITE_PROFILES); IPR007110 (PROSITE_PROFILES); IPR007110 (PROSITE_PROFILES); IPR007110 (PROSITE_PROFILES); IPR007110 (PROSITE_PROFILES); IPR007110 (PROSITE_PROFILES); IPR007110 (PROSITE_PROFILES); IPR007110 (PROSITE_PROFILES); SSF48726 (SUPERFAMILY); SSF48726 (SUPERFAMILY); SSF48726 (SUPERFAMILY); SSF48726 (SUPERFAMILY); SSF48726 (SUPERFAMILY); SSF48726 (SUPERFAMILY); SSF48726 (SUPERFAMILY); SSF48726 (SUPERFAMILY)
2229	302298_length_3283_cvg_12.8_tip_1_0	576	Apolipophorin, partial	1095	0	57.90%	0.098	IPR001846 (SMART); IPR014853 (PFAM); IPR001846 (PFAM); PTHR23361:SF13 (PANTHER); PTHR23361 (PANTHER); CYTOPLASMIC_DOMAIN (PHOBIUS); TRANSMEMBRANE (PHOBIUS); NON_CYTOPLASMIC_DOMAIN (PHOBIUS); IPR001846 (PROSITE_PROFILES)
2230	302306_length_3296_cvg_16.9_tip_1_3	970	kielin chordin-like protein isoform x2	1099	1.86E-41	49.70%	0.136	IPR002223 (PRINTS); IPR002350 (SMART); IPR002223 (SMART); IPR000742 (SMART); IPR003645 (SMART); IPR002223 (PFAM); IPR002223 (G3DSA:4.10.410.GENE3D); IPR013783 (G3DSA:2.60.40.GENE3D); IPR002350 (PFAM); IPR002223 (G3DSA:4.10.410.GENE3D); IPR003961 (PFAM); G3DSA:2.10.25.10 (GENE3D); G3DSA:3.30.60.30 (GENE3D); G3DSA:2.170.300.10 (GENE3D); PTHR10083 (PANTHER); IPR013032 (PROSITE_PATTERNS); IPR013032 (PROSITE_PATTERNS); IPR013032 (PROSITE_PATTERNS); IPR020901 (PROSITE_PATTERNS); IPR020901 (PROSITE_PATTERNS); IPR020901 (PROSITE_PATTERNS); IPR020901 (PROSITE_PATTERNS); IPR003961 (PROSITE_PROFILES); IPR003961 (PROSITE_PROFILES); IPR003961 (PROSITE_PROFILES); IPR000742 (PROSITE_PROFILES); IPR002223 (PROSITE_PROFILES); IPR002350 (PROSITE_PROFILES); IPR002223 (PROSITE_PROFILES); IPR000742 (PROSITE_PROFILES); IPR002223 (PROSITE_PROFILES); IPR000742 (PROSITE_PROFILES); IPR002223 (PROSITE_PROFILES); SSF100895 (SUPERFAMILY); SSF57196 (SUPERFAMILY); IPR003961 (SUPERFAMILY); IPR002223 (SUPERFAMILY); IPR002223 (SUPERFAMILY); IPR002223 (SUPERFAMILY); IPR002223 (SUPERFAMILY); IPR003961 (SUPERFAMILY)
2231	302316_length_3322_cvg_16.6_tip_1_3	711	hemocytin isoform x4	1108	3.53E-86	59.30%	0.162	PR01217 (PRINTS); IPR000421 (SMART); IPR008979 (G3DSA:2.60.120.GENE3D); IPR000421 (PFAM); IPR000421 (PROSITE_PATTERNS); IPR000421 (PROSITE_PATTERNS); IPR000421 (PROSITE_PATTERNS); IPR000421 (PROSITE_PATTERNS); IPR000421 (PROSITE_PATTERNS); IPR000421 (PROSITE_PROFILES); IPR000421 (PROSITE_PROFILES); IPR008979 (SUPERFAMILY); IPR008979 (SUPERFAMILY)
2232	302332_length_3359_cvg_21.0_tip_1_3	1285	protein mesh isoform x2	1120	0	71.60%	0.255	IPR005533 (SMART); IPR003886 (SMART); IPR001846 (SMART); IPR000436 (SMART); IPR003886 (PFAM); IPR013783 (G3DSA:2.60.40.GENE3D); IPR001846 (PFAM); IPR005533 (PFAM); G3DSA:2.10.70.10 (GENE3D); IPR002909 (PFAM); IPR000436 (PFAM); PTHR13802:SF41 (PANTHER); PTHR13802 (PANTHER); TRANSMEMBRANE (PHOBIUS); CYTOPLASMIC_DOMAIN (PHOBIUS); NON_CYTOPLASMIC_DOMAIN (PHOBIUS); IPR001846 (PROSITE_PROFILES); IPR003886 (PROSITE_PROFILES); IPR005533 (PROSITE_PROFILES); IPR000436 (PROSITE_PROFILES); IPR000436 (SUPERFAMILY); IPR014756 (SUPERFAMILY)
2233	302334_length_3362_cvg_7.2_tip_1_5	422	low quality protein: twitchin-like	1120	0	82.40%	0.102	PR00014 (PRINTS); IPR003598 (SMART); IPR003599 (SMART); IPR003961 (SMART); IPR013098 (PFAM); IPR013783 (G3DSA:2.60.40.GENE3D); IPR013783 (G3DSA:2.60.40.GENE3D); IPR013783 (G3DSA:2.60.40.GENE3D); IPR013783 (G3DSA:2.60.40.GENE3D); IPR003961 (PFAM); IPR013783 (G3DSA:2.60.40.GENE3D); PTHR19897 (PANTHER); PTHR19897:SF155 (PANTHER); IPR007110 (PROSITE_PROFILES); IPR003961 (PROSITE_PROFILES); IPR003961 (PROSITE_PROFILES); IPR003961 (PROSITE_PROFILES); IPR003961 (PROSITE_PROFILES); IPR003961 (PROSITE_PROFILES); IPR007110 (PROSITE_PROFILES); IPR007110 (PROSITE_PROFILES); IPR003961 (PROSITE_PROFILES); IPR007110 (PROSITE_PROFILES); IPR003961 (PROSITE_PROFILES); IPR003961 (PROSITE_PROFILES); IPR003961 (SUPERFAMILY); IPR003961 (SUPERFAMILY); SSF48726 (SUPERFAMILY); IPR003961 (SUPERFAMILY); SSF48726 (SUPERFAMILY); IPR003961 (SUPERFAMILY); SSF48726 (SUPERFAMILY); IPR003961 (SUPERFAMILY); SSF48726 (SUPERFAMILY)

2234	302340_length_3374_cvg_18.8_tip_1_4	1079	staphylococcal nuclease domain-containing protein 1-like	1125	0	74.40%	0.147	Coil (COILS); IPR002999 (SMART); IPR016071 (SMART); IPR016071 (PFAM); IPR016071 (G3DSA:2.40.50.GENE3D); G3DSA:2.30.30.140 (GENE3D); IPR002999 (PFAM); IPR016071 (G3DSA:2.40.50.GENE3D); IPR030481 (PTHR12302:PANTHER); PTHR12302 (PANTHER); NON_CYTOPLASMIC_DOMAIN (PHOBIUS); TRANSMEMBRANE (PHOBIUS); CYTOPLASMIC_DOMAIN (PHOBIUS); IPR002999 (PROSITE_PROFILES); IPR016071 (PROSITE_PROFILES); IPR016071 (PROSITE_PROFILES); IPR016071 (PROSITE_PROFILES); IPR016071 (PROSITE_PROFILES); IPR016071 (SUPERFAMILY); SSF63748 (SUPERFAMILY); IPR016071 (SUPERFAMILY); IPR016071 (SUPERFAMILY); IPR016071 (SUPERFAMILY); IPR016071 (SUPERFAMILY); TMhelix (TMHMM); TMhelix (TMHMM)
2235	302352_length_3406_cvg_12.2_tip_1_2	505	uncharacterized family 31 glucosidase kiaa1161 isoform x5	1135	0	62.40%	0.137	IPR013785 (G3DSA:3.20.20.GENE3D); IPR000322 (PFAM); PTHR22762:SF73 (PANTHER); PTHR22762 (PANTHER); CYTOPLASMIC_DOMAIN (PHOBIUS); TRANSMEMBRANE (PHOBIUS); NON_CYTOPLASMIC_DOMAIN (PHOBIUS); SSF51011 (SUPERFAMILY); IPR017853 (SUPERFAMILY); TMhelix (TMHMM)
2236	302360_length_3422_cvg_14.6_tip_1_3	575	integrin alpha-8-like	1141	1.71E-153	48.70%	0.105	IPR000413 (PRINTS); IPR013519 (SMART); G3DSA:2.60.40.1530 (GENE3D); G3DSA:2.130.10.130 (GENE3D); IPR013649 (PFAM); PF13517 (PFAM); G3DSA:2.60.40.1510 (GENE3D); G3DSA:1.20.5.930 (GENE3D); G3DSA:2.60.40.1460 (GENE3D); PTHR23220 (PANTHER); PTHR23220:SF71 (PANTHER); TRANSMEMBRANE (PHOBIUS); NON_CYTOPLASMIC_DOMAIN (PHOBIUS); CYTOPLASMIC_DOMAIN (PHOBIUS); IPR013519 (PROSITE_PROFILES); IPR013519 (PROSITE_PROFILES); IPR013519 (PROSITE_PROFILES); IPR013519 (PROSITE_PROFILES); IPR013519 (PROSITE_PROFILES); IPR013519 (PROSITE_PROFILES); SSF69179 (SUPERFAMILY); SSF69179 (SUPERFAMILY); SSF69179 (SUPERFAMILY); SSF69318 (SUPERFAMILY); TMhelix (TMHMM)
2237	302362_length_3441_cvg_22.6_tip_1_3	1064	hemocyte protein-glutamine gamma-glutamyltransferase-like	1147	0	67.80%	0.101	Coil (COILS); IPR002931 (SMART); IPR013783 (G3DSA:2.60.40.GENE3D); IPR008958 (PFAM); IPR013783 (G3DSA:2.60.40.GENE3D); IPR001102 (PFAM); IPR002931 (G3DSA:3.90.260.GENE3D); IPR002931 (PFAM); IPR013783 (G3DSA:2.60.40.GENE3D); PTHR11590:SF40 (PANTHER); IPR023608 (PANTHER); IPR013808 (PROSITE_PATTERNS); TRANSMEMBRANE (PHOBIUS); NON_CYTOPLASMIC_DOMAIN (PHOBIUS); CYTOPLASMIC_DOMAIN (PHOBIUS); TRANSMEMBRANE (PHOBIUS); TRANSMEMBRANE (PHOBIUS); NON_CYTOPLASMIC_DOMAIN (PHOBIUS); CYTOPLASMIC_DOMAIN (PHOBIUS); IPR008958 (SUPERFAMILY); IPR008958 (SUPERFAMILY); IPR014756 (SUPERFAMILY); SSF54001 (SUPERFAMILY); TMhelix (TMHMM); TMhelix (TMHMM); TMhelix (TMHMM)
2238	302388_length_3517_cvg_20.7_tip_1_3	1041	cytosolic 10-formyltetrahydrofolate dehydrogenase isoform x1	1173	0	83.50%	0.123	Coil (COILS); Coil (COILS); IPR016163 (G3DSA:3.40.309.GENE3D); IPR005793 (PFAM); IPR015590 (PFAM); IPR009081 (G3DSA:1.10.1200.GENE3D); IPR016162 (G3DSA:3.40.605.GENE3D); IPR009081 (PFAM); IPR005793 (G3DSA:3.10.25.GENE3D); IPR002376 (PFAM); IPR002376 (G3DSA:3.40.50.GENE3D); PTHR11699 (PANTHER); PTHR11699:SF118 (PANTHER); IPR029510 (PROSITE_PATTERNS); IPR016160 (PROSITE_PATTERNS); IPR001555 (PROSITE_PATTERNS); IPR009081 (PROSITE_PROFILES); IPR009081 (SUPERFAMILY); IPR002376 (SUPERFAMILY); IPR016161 (SUPERFAMILY); IPR011034 (SUPERFAMILY)
2239	302394_length_3529_cvg_18.7_tip_1_4	929	glucose-6-phosphate isomerase	1176	0	87.70%	0.103	IPR001672 (PRINTS); IPR001672 (PFAM); G3DSA:3.40.50.10490 (GENE3D); IPR023096 (G3DSA:1.10.1390.GENE3D); IPR001672 (PANTHER); PTHR11469:SF4 (PANTHER); IPR018189 (PROSITE_PATTERNS); IPR018189 (PROSITE_PATTERNS); NON_CYTOPLASMIC_DOMAIN (PHOBIUS); NON_CYTOPLASMIC_DOMAIN (PHOBIUS); TRANSMEMBRANE (PHOBIUS); TRANSMEMBRANE (PHOBIUS); CYTOPLASMIC_DOMAIN (PHOBIUS); NON_CYTOPLASMIC_DOMAIN (PHOBIUS); TRANSMEMBRANE (PHOBIUS); NON_CYTOPLASMIC_DOMAIN (PHOBIUS); CYTOPLASMIC_DOMAIN (PHOBIUS); TRANSMEMBRANE (PHOBIUS); TRANSMEMBRANE (PHOBIUS); CYTOPLASMIC_DOMAIN (PHOBIUS); TRANSMEMBRANE (PHOBIUS); IPR001672 (PROSITE_PROFILES); IPR001672 (HAMAP); SSF53697 (SUPERFAMILY); TMhelix (TMHMM); TMhelix (TMHMM)
2240	302398_length_3539_cvg_9.3_tip_1_2	505	puromycin-sensitive aminopeptidase-like	1179	0	78.80%	0.218	Coil (COILS); IPR014782 (PRINTS); IPR024571 (PFAM); IPR014782 (PFAM); G3DSA:1.10.390.10 (GENE3D); IPR001930 (PANTHER); PTHR11533:SF163 (PANTHER); CYTOPLASMIC_DOMAIN (PHOBIUS); NON_CYTOPLASMIC_DOMAIN (PHOBIUS); TRANSMEMBRANE (PHOBIUS); SSF55486 (SUPERFAMILY); SSF63737 (SUPERFAMILY)

2241	302408_length_3568_cvg_32.4_tip_1_1	1548	agap005124-pc-like protein	1189	0	78.10%	0.217	Coil (COILS); IPR016162 (G3DSA:3.40.605.GENE3D); IPR016163 (G3DSA:3.40.309.GENE3D); IPR015590 (PFAM); PTHR11699 (PANTHER); PTHR11699:SF15 (PANTHER); IPR029510 (PROSITE_PATTERNS); CYTOPLASMIC_DOMAIN (PHOBIUS); NON_CYTOPLASMIC_DOMAIN (PHOBIUS); TRANSMEMBRANE (PHOBIUS); IPR016161 (SUPERFAMILY)
2242	302410_length_3569_cvg_27.6_tip_1_3	1524	lethal giant larvae protein homolog 1-like isoform x3	1190	0	67.10%	0.125	Coil (COILS); Coil (COILS); IPR000664 (PRINTS); IPR013905 (PFAM); IPR015943 (G3DSA:2.130.10.GENE3D); PTHR10241:SF28 (PANTHER); PTHR10241 (PANTHER); IPR019775 (PROSITE_PATTERNS); IPR001680 (PROSITE_PROFILES); IPR017986 (SUPERFAMILY)
2243	302412_length_3574_cvg_56.6_tip_1_4	4320	vitellogenin fused with superoxide dismutase	1191	4.11E-91	43.70%	0.207	IPR001846 (SMART); IPR015818 (G3DSA:2.20.80.GENE3D); IPR001846 (PFAM); IPR011030 (G3DSA:1.25.10.GENE3D); IPR015255 (PFAM); PTHR23345:SF1 (PANTHER); PTHR23345 (PANTHER); TRANSMEMBRANE (PHOBIUS); TRANSMEMBRANE (PHOBIUS); NON_CYTOPLASMIC_DOMAIN (PHOBIUS); CYTOPLASMIC_DOMAIN (PHOBIUS); NON_CYTOPLASMIC_DOMAIN (PHOBIUS); IPR001846 (PROSITE_PROFILES); IPR015819 (SUPERFAMILY); TMhelix (TMHMM); TMhelix (TMHMM)
2244	302422_length_3587_cvg_7.8_tip_1_3	455	sodium calcium exchanger 2 isoform x1	1196	0	77.30%	0.156	IPR004836 (PRINTS); IPR003644 (SMART); IPR004836 (TIGRFAM); IPR004837 (PFAM); IPR003644 (PFAM); PTHR11878 (PANTHER); CYTOPLASMIC_DOMAIN (PHOBIUS); NON_CYTOPLASMIC_DOMAIN (PHOBIUS); CYTOPLASMIC_DOMAIN (PHOBIUS); TRANSMEMBRANE (PHOBIUS); TRANSMEMBRANE (PHOBIUS); NON_CYTOPLASMIC_DOMAIN (PHOBIUS); TRANSMEMBRANE (PHOBIUS); TRANSMEMBRANE (PHOBIUS); TRANSMEMBRANE (PHOBIUS); NON_CYTOPLASMIC_DOMAIN (PHOBIUS); NON_CYTOPLASMIC_DOMAIN (PHOBIUS); CYTOPLASMIC_DOMAIN (PHOBIUS); TRANSMEMBRANE (PHOBIUS); CYTOPLASMIC_DOMAIN (PHOBIUS); NON_CYTOPLASMIC_DOMAIN (PHOBIUS); CYTOPLASMIC_DOMAIN (PHOBIUS); TRANSMEMBRANE (PHOBIUS); TRANSMEMBRANE (PHOBIUS); CYTOPLASMIC_DOMAIN (PHOBIUS); TRANSMEMBRANE (PHOBIUS); TRANSMEMBRANE (PHOBIUS); SSF141072 (SUPERFAMILY); SSF141072 (SUPERFAMILY); TMhelix (TMHMM); TMhelix (TMHMM); TMhelix (TMHMM); TMhelix (TMHMM); TMhelix (TMHMM); TMhelix (TMHMM); TMhelix (TMHMM); TMhelix (TMHMM); TMhelix (TMHMM); TMhelix (TMHMM)
2245	302434_length_3619_cvg_12.2_tip_1_5	506	insulin-degrading enzyme	1206	0	76.50%	0.26	Coil (COILS); IPR011237 (G3DSA:3.30.830.GENE3D); IPR007863 (PFAM); IPR011237 (G3DSA:3.30.830.GENE3D); IPR011765 (PFAM); IPR011237 (G3DSA:3.30.830.GENE3D); IPR011237 (G3DSA:3.30.830.GENE3D); PTHR11851 (PANTHER); PTHR11851:SF85 (PANTHER); IPR001431 (PROSITE_PATTERNS); IPR011249 (SUPERFAMILY); IPR011249 (SUPERFAMILY); IPR011249 (SUPERFAMILY); IPR011249 (SUPERFAMILY)
2246	302452_length_3674_cvg_32.4_tip_1_4	1795	titin	1225	0	82.60%	0.117	Coil (COILS); IPR003599 (SMART); IPR003598 (SMART); IPR013783 (G3DSA:2.60.40.GENE3D); IPR010939 (PFAM); IPR013783 (G3DSA:2.60.40.GENE3D); IPR013783 (G3DSA:2.60.40.GENE3D); IPR013783 (G3DSA:2.60.40.GENE3D); IPR013098 (PFAM); PTHR19897:SF149 (PANTHER); PTHR19897 (PANTHER); IPR007110 (PROSITE_PROFILES); IPR007110 (PROSITE_PROFILES); IPR007110 (PROSITE_PROFILES); IPR007110 (PROSITE_PROFILES); IPR007110 (PROSITE_PROFILES); IPR007110 (PROSITE_PROFILES); IPR007110 (PROSITE_PROFILES); IPR007110 (PROSITE_PROFILES); SSF48726 (SUPERFAMILY); SSF48726 (SUPERFAMILY); SSF48726 (SUPERFAMILY); SSF48726 (SUPERFAMILY); SSF48726 (SUPERFAMILY); SSF48726 (SUPERFAMILY); SSF48726 (SUPERFAMILY); SSF48726 (SUPERFAMILY)
2247	302456_length_3675_cvg_31.6_tip_1_5	1782	c-1-tetrahydrofolate cytoplasmic	1225	0	83.30%	0.179	Coil (COILS); IPR000672 (PRINTS); G3DSA:3.30.1510.10 (GENE3D); IPR027417 (G3DSA:3.40.50.GENE3D); IPR020630 (PFAM); G3DSA:3.40.192.10 (GENE3D); IPR000559 (PFAM); G3DSA:3.10.410.10 (GENE3D); IPR016040 (G3DSA:3.40.50.GENE3D); IPR020631 (PFAM); PTHR10025 (PANTHER); IPR020867 (PROSITE_PATTERNS); IPR020628 (PROSITE_PATTERNS); IPR020867 (PROSITE_PATTERNS); IPR020628 (PROSITE_PATTERNS); IPR000672 (HAMAP); IPR000559 (HAMAP); SSF51735 (SUPERFAMILY); IPR027417 (SUPERFAMILY); SSF53223 (SUPERFAMILY)
2248	302478_length_3743_cvg_50.9_tip_1_5	3901	sarcalumenin isoform x2	1247	0	79.80%	0.116	IPR027417 (G3DSA:3.40.50.GENE3D); IPR006073 (PFAM); PTHR11216:SF66 (PANTHER); PTHR11216 (PANTHER); NON_CYTOPLASMIC_DOMAIN (PHOBIUS); TRANSMEMBRANE (PHOBIUS); CYTOPLASMIC_DOMAIN (PHOBIUS); TRANSMEMBRANE (PHOBIUS); NON_CYTOPLASMIC_DOMAIN (PHOBIUS); IPR030381 (PROSITE_PROFILES); IPR027417 (SUPERFAMILY); TMhelix (TMHMM)

2249	302480_length_3745_cvg_28.5_tip_1_0	1976	low quality protein: twitchin-like	1249	0	79.30%	0.102	Coil (COILS); PR00014 (PRINTS); IPR003961 (SMART); IPR003599 (SMART); IPR013783 (G3DSA:2.60.40.GENE3D); IPR003961 (PFAM); IPR013783 (G3DSA:2.60.40.GENE3D); IPR013783 (G3DSA:2.60.40.GENE3D); IPR013783 (G3DSA:2.60.40.GENE3D); IPR013098 (PFAM); IPR013783 (G3DSA:2.60.40.GENE3D); IPR013783 (G3DSA:2.60.40.GENE3D); IPR013783 (G3DSA:2.60.40.GENE3D); PTHR19897:SF155 (PANTHER); PTHR19897 (PANTHER); IPR003961 (PROSITE_PROFILES); IPR003961 (PROSITE_PROFILES); IPR003961 (PROSITE_PROFILES); IPR007110 (PROSITE_PROFILES); IPR003961 (PROSITE_PROFILES); IPR003961 (PROSITE_PROFILES); IPR007110 (PROSITE_PROFILES); IPR003961 (PROSITE_PROFILES); IPR003961 (PROSITE_PROFILES); IPR003961 (SUPERFAMILY); IPR003961 (SUPERFAMILY); IPR003961 (SUPERFAMILY); SSF48726 (SUPERFAMILY); IPR003961 (SUPERFAMILY); SSF48726 (SUPERFAMILY); SSF48726 (SUPERFAMILY); SSF48726 (SUPERFAMILY); SSF48726 (SUPERFAMILY)
2250	302502_length_3805_cvg_27.0_tip_1_4	1697	nuclear anchorage protein 1-like	1268	0	73.30%	0.101	PR00014 (PRINTS); IPR003599 (SMART); IPR003961 (SMART); IPR002290 (SMART); IPR003598 (SMART); G3DSA:1.10.510.10 (GENE3D); IPR000719 (PFAM); IPR013098 (PFAM); IPR013783 (G3DSA:2.60.40.GENE3D); IPR013783 (G3DSA:2.60.40.GENE3D); IPR013783 (G3DSA:2.60.40.GENE3D); IPR003961 (PFAM); G3DSA:3.30.200.20 (GENE3D); IPR020675 (PANTHER); PTHR22964:SF57 (PANTHER); IPR008271 (PROSITE_PATTERNS); IPR017441 (PROSITE_PATTERNS); IPR000719 (PROSITE_PROFILES); IPR007110 (PROSITE_PROFILES); IPR003961 (PROSITE_PROFILES); IPR011009 (SUPERFAMILY); SSF48726 (SUPERFAMILY); IPR003961 (SUPERFAMILY); SSF48726 (SUPERFAMILY)
2251	302506_length_3819_cvg_30.7_tip_1_1	1432	scp-related protein	1273	2.92E-147	63.30%	0.107	IPR001283 (PRINTS); IPR014044 (SMART); IPR014044 (PFAM); IPR014044 (G3DSA:3.40.33.GENE3D); IPR001283 (PANTHER); PTHR10334:SF189 (PANTHER); IPR018244 (PROSITE_PATTERNS); IPR018244 (PROSITE_PATTERNS); TRANSMEMBRANE (PHOBIUS); TRANSMEMBRANE (PHOBIUS); TRANSMEMBRANE (PHOBIUS); TRANSMEMBRANE (PHOBIUS); NON_CYTOPLASMIC_DOMAIN (PHOBIUS); CYTOPLASMIC_DOMAIN (PHOBIUS); NON_CYTOPLASMIC_DOMAIN (PHOBIUS); NON_CYTOPLASMIC_DOMAIN (PHOBIUS); CYTOPLASMIC_DOMAIN (PHOBIUS); IPR014044 (SUPERFAMILY); IPR014044 (SUPERFAMILY); TMhelix (TMHMM); TMhelix (TMHMM); TMhelix (TMHMM); TMhelix (TMHMM)
2252	302512_length_3826_cvg_8.0_tip_1_0	454	low quality protein: twitchin-like	1276	0	81.90%	0.101	PR00014 (PRINTS); IPR003961 (SMART); IPR003598 (SMART); IPR003599 (SMART); IPR013783 (G3DSA:2.60.40.GENE3D); IPR013783 (G3DSA:2.60.40.GENE3D); IPR013783 (G3DSA:2.60.40.GENE3D); IPR003961 (PFAM); IPR013783 (G3DSA:2.60.40.GENE3D); IPR013783 (G3DSA:2.60.40.GENE3D); IPR013783 (G3DSA:2.60.40.GENE3D); IPR013783 (G3DSA:2.60.40.GENE3D); IPR013098 (PFAM); PTHR19897 (PANTHER); PTHR19897:SF155 (PANTHER); IPR007110 (PROSITE_PROFILES); IPR003961 (PROSITE_PROFILES); IPR007110 (PROSITE_PROFILES); IPR003961 (PROSITE_PROFILES); IPR003961 (PROSITE_PROFILES); IPR003961 (PROSITE_PROFILES); IPR003961 (PROSITE_PROFILES); IPR003961 (PROSITE_PROFILES); IPR003961 (PROSITE_PROFILES); IPR003961 (PROSITE_PROFILES); IPR003961 (PROSITE_PROFILES); IPR007110 (PROSITE_PROFILES); IPR003961 (PROSITE_PROFILES); SSF48726 (SUPERFAMILY); SSF48726 (SUPERFAMILY); IPR003961 (SUPERFAMILY); IPR003961 (SUPERFAMILY); SSF48726 (SUPERFAMILY); SSF48726 (SUPERFAMILY); IPR003961 (SUPERFAMILY); IPR003961 (SUPERFAMILY); IPR003961 (SUPERFAMILY)

2253	302526_length_3942_cvg_38.5_tip_1_4	1920	pacifastin light chain	1314	6.41E-50	54.10%	0.113	IPR002223 (PRINTS); IPR002223 (SMART); IPR006552 (SMART); IPR001007 (SMART); IPR002223 (G3DSA:4.10.410.GENE3D); IPR008037 (PFAM); IPR002223 (G3DSA:4.10.410.GENE3D); IPR002223 (PFAM); PTHR10083 (PANTHER); IPR020901 (PROSITE_PATTERNS); IPR020901 (PROSITE_PATTERNS); IPR020862 (PROSITE_PROFILES); IPR002223 (PROSITE_PROFILES); IPR020862 (PROSITE_PROFILES); IPR020862 (PROSITE_PROFILES); IPR002223 (PROSITE_PROFILES); IPR020862 (PROSITE_PROFILES); IPR020862 (PROSITE_PROFILES); IPR020862 (PROSITE_PROFILES); IPR001007 (PROSITE_PROFILES); IPR020862 (PROSITE_PROFILES); IPR020862 (PROSITE_PROFILES); IPR020862 (PROSITE_PROFILES); IPR020862 (PROSITE_PROFILES); IPR020862 (PROSITE_PROFILES); IPR001190 (PROSITE_PROFILES); IPR002223 (PROSITE_PROFILES); IPR001190 (PROSITE_PROFILES); IPR002223 (PROSITE_PROFILES); IPR008037 (SUPERFAMILY); IPR008037 (SUPERFAMILY); IPR002223 (SUPERFAMILY); IPR008037 (SUPERFAMILY); IPR002223 (SUPERFAMILY); IPR008037 (SUPERFAMILY); IPR002223 (SUPERFAMILY); IPR008037 (SUPERFAMILY); IPR008037 (SUPERFAMILY); IPR002223 (SUPERFAMILY); IPR008037 (SUPERFAMILY); IPR008037 (SUPERFAMILY); IPR008037 (SUPERFAMILY)
2254	302544_length_3998_cvg_7.2_tip_1_2	541	carboxypeptidase d-like	1332	8.30E-93	57.40%	0.106	IPR000834 (SMART); IPR000834 (PFAM); G3DSA:3.40.630.10 (GENE3D); PF13620 (PFAM); IPR014766 (G3DSA:2.60.40.GENE3D); PTHR11532 (PANTHER); IPR000834 (PROSITE_PATTERNS); NON_CYTOPLASMIC_DOMAIN (PHOBIUS); TRANSMEMBRANE (PHOBIUS); TRANSMEMBRANE (PHOBIUS); CYTOPLASMIC_DOMAIN (PHOBIUS); CYTOPLASMIC_DOMAIN (PHOBIUS); NON_CYTOPLASMIC_DOMAIN (PHOBIUS); TRANSMEMBRANE (PHOBIUS); CYTOPLASMIC_DOMAIN (PHOBIUS); TRANSMEMBRANE (PHOBIUS); TRANSMEMBRANE (PHOBIUS); CYTOPLASMIC_DOMAIN (PHOBIUS); NON_CYTOPLASMIC_DOMAIN (PHOBIUS); TRANSMEMBRANE (PHOBIUS); NON_CYTOPLASMIC_DOMAIN (PHOBIUS); TRANSMEMBRANE (PHOBIUS); TRANSMEMBRANE (PHOBIUS); NON_CYTOPLASMIC_DOMAIN (PHOBIUS); TRANSMEMBRANE (PHOBIUS); TRANSMEMBRANE (PHOBIUS); NON_CYTOPLASMIC_DOMAIN (PHOBIUS); CYTOPLASMIC_DOMAIN (PHOBIUS); CYTOPLASMIC_DOMAIN (PHOBIUS); TRANSMEMBRANE (PHOBIUS); SSF53187 (SUPERFAMILY); IPR008969 (SUPERFAMILY); TMhelix (TMHMM); TMhelix (TMHMM); TMhelix (TMHMM); TMhelix (TMHMM); TMhelix (TMHMM); TMhelix (TMHMM); TMhelix (TMHMM); TMhelix (TMHMM); TMhelix (TMHMM); TMhelix (TMHMM); TMhelix (TMHMM)
2255	302548_length_4015_cvg_32.7_tip_1_3	1274	coatomer subunit alpha isoform x2	1339	0	87.60%	0.128	IPR020472 (PRINTS); IPR001680 (SMART); IPR016391 (PIRSF); IPR010714 (PFAM); IPR006692 (PFAM); IPR001680 (PFAM); IPR015943 (G3DSA:2.130.10.GENE3D); PTHR19876 (PANTHER); IPR019775 (PROSITE_PATTERNS); IPR017986 (PROSITE_PROFILES); IPR001680 (PROSITE_PROFILES); IPR001680 (PROSITE_PROFILES); IPR001680 (PROSITE_PROFILES); IPR001680 (PROSITE_PROFILES); IPR001680 (PROSITE_PROFILES); SSF82171 (SUPERFAMILY); IPR017986 (SUPERFAMILY)
2256	302558_length_4055_cvg_66.1_tip_1_5	18192	long form-like	1351	0	86.30%	0.157	Coil (COILS); Coil (COILS); Coil (COILS); Coil (COILS); Coil (COILS); Coil (COILS); Coil (COILS); Coil (COILS); Coil (COILS); Coil (COILS); Coil (COILS); Coil (COILS); IPR002928 (PFAM); PTHR13140 (PANTHER); PTHR13140:SF164 (PANTHER); SSF90257 (SUPERFAMILY); SSF90257 (SUPERFAMILY); SSF90257 (SUPERFAMILY); SSF90257 (SUPERFAMILY)
2257	302578_length_4212_cvg_26.1_tip_1_5	1289	low quality protein: mucin-19	1404	4.27E-65	40.50%	0.099	TMhelix (TMHMM); TMhelix (TMHMM); TMhelix (TMHMM); TMhelix (TMHMM); TMhelix (TMHMM); TMhelix (TMHMM); TMhelix (TMHMM); TMhelix (TMHMM); TMhelix (TMHMM); TMhelix (TMHMM); TMhelix (TMHMM); TMhelix (TMHMM); TMhelix (TMHMM)
2258	302586_length_4276_cvg_12.5_tip_1_4	650	dystroglycan isoform x1	1425	0	61.20%	0.223	IPR006644 (SMART); IPR008009 (PFAM); IPR008465 (PFAM); IPR013783 (G3DSA:2.60.40.GENE3D); IPR027468 (G3DSA:3.30.70.GENE3D); PTHR21559:SF16 (PANTHER); PTHR21559 (PANTHER); NON_CYTOPLASMIC_DOMAIN (PHOBIUS); TRANSMEMBRANE (PHOBIUS); CYTOPLASMIC_DOMAIN (PHOBIUS); TRANSMEMBRANE (PHOBIUS); CYTOPLASMIC_DOMAIN (PHOBIUS); NON_CYTOPLASMIC_DOMAIN (PHOBIUS); TRANSMEMBRANE (PHOBIUS); IPR030398 (PROSITE_PROFILES); IPR030398 (PROSITE_PROFILES); IPR015919 (SUPERFAMILY); IPR015919 (SUPERFAMILY); IPR015919 (SUPERFAMILY); IPR027468 (SUPERFAMILY); TMhelix (TMHMM); TMhelix (TMHMM); TMhelix (TMHMM)

2259	302596_length_4334_cvg_8.3_tip_1_5	506	glutamyl aminopeptidase	1444	0	63.60%	0.111		IPR014782 (PRINTS); G3DSA:1.10.390.10 (GENE3D); IPR014782 (PFAM); IPR024571 (PFAM); IPR001930 (PANTHER); PTHR11533:SF165 (PANTHER); CYTOPLASMIC_DOMAIN (PHOBIUS); TRANSMEMBRANE (PHOBIUS); NON_CYTOPLASMIC_DOMAIN (PHOBIUS); SSF63737 (SUPERFAMILY); SSF55486 (SUPERFAMILY); TMhelix (TMHMM)
2260	302598_length_4353_cvg_17.5_tip_1_2	963	nad mitochondrial	1451	0	83.70%	0.146		IPR007886 (SMART); IPR007698 (SMART); IPR012136 (PFAM); IPR029035 (G3DSA:3.40.50.GENE3D); IPR007886 (PFAM); IPR024605 (PFAM); G3DSA:3.40.50.1770 (GENE3D); IPR026255 (TIGRFAM); IPR007698 (PFAM); G3DSA:3.40.50.1770 (GENE3D); PTHR10160 (PANTHER); PTHR10160:SF19 (PANTHER); NON_CYTOPLASMIC_DOMAIN (PHOBIUS); TRANSMEMBRANE (PHOBIUS); TRANSMEMBRANE (PHOBIUS); NON_CYTOPLASMIC_DOMAIN (PHOBIUS); CYTOPLASMIC_DOMAIN (PHOBIUS); TRANSMEMBRANE (PHOBIUS); NON_CYTOPLASMIC_DOMAIN (PHOBIUS); TRANSMEMBRANE (PHOBIUS); NON_CYTOPLASMIC_DOMAIN (PHOBIUS); CYTOPLASMIC_DOMAIN (PHOBIUS); CYTOPLASMIC_DOMAIN (PHOBIUS); TRANSMEMBRANE (PHOBIUS); TRANSMEMBRANE (PHOBIUS); TRANSMEMBRANE (PHOBIUS); TRANSMEMBRANE (PHOBIUS); CYTOPLASMIC_DOMAIN (PHOBIUS); TRANSMEMBRANE (PHOBIUS); TRANSMEMBRANE (PHOBIUS); CYTOPLASMIC_DOMAIN (PHOBIUS); CYTOPLASMIC_DOMAIN (PHOBIUS); NON_CYTOPLASMIC_DOMAIN (PHOBIUS); NON_CYTOPLASMIC_DOMAIN (PHOBIUS); CYTOPLASMIC_DOMAIN (PHOBIUS); TRANSMEMBRANE (PHOBIUS); TRANSMEMBRANE (PHOBIUS); CYTOPLASMIC_DOMAIN (PHOBIUS); TRANSMEMBRANE (PHOBIUS); TRANSMEMBRANE (PHOBIUS); CYTOPLASMIC_DOMAIN (PHOBIUS); NON_CYTOPLASMIC_DOMAIN (PHOBIUS); TRANSMEMBRANE (PHOBIUS); TRANSMEMBRANE (PHOBIUS); NON_CYTOPLASMIC_DOMAIN (PHOBIUS); SSF52283 (SUPERFAMILY); IPR029035 (SUPERFAMILY); SSF51735 (SUPERFAMILY); TMhelix (TMHMM); TMhelix (TMHMM); TMhelix (TMHMM); TMhelix (TMHMM); TMhelix (TMHMM); TMhelix (TMHMM); TMhelix (TMHMM); TMhelix (TMHMM); TMhelix (TMHMM); TMhelix (TMHMM); TMhelix (TMHMM); TMhelix (TMHMM); TMhelix (TMHMM); TMhelix (TMHMM); TMhelix (TMHMM)
2261	302600_length_4384_cvg_5.9_tip_1_1	419	urokinase-type plasminogen activator	1461	7.87E-59	56.00%	0.211		IPR002172 (PRINTS); IPR001254 (SMART); IPR002172 (SMART); IPR003609 (SMART); IPR003014 (PFAM); IPR002172 (G3DSA:4.10.400.GENE3D); G3DSA:2.40.10.10 (GENE3D); IPR002172 (G3DSA:4.10.400.GENE3D); IPR002172 (PFAM); G3DSA:2.40.10.10 (GENE3D); G3DSA:2.40.10.10 (GENE3D); G3DSA:3.50.4.10 (GENE3D); IPR001254 (PFAM); PTHR24265 (PANTHER); IPR018114 (PROSITE_PATTERNS); IPR018114 (PROSITE_PATTERNS); IPR023415 (PROSITE_PATTERNS); TRANSMEMBRANE (PHOBIUS); TRANSMEMBRANE (PHOBIUS); NON_CYTOPLASMIC_DOMAIN (PHOBIUS); TRANSMEMBRANE (PHOBIUS); CYTOPLASMIC_DOMAIN (PHOBIUS); CYTOPLASMIC_DOMAIN (PHOBIUS); CYTOPLASMIC_DOMAIN (PHOBIUS); TRANSMEMBRANE (PHOBIUS); NON_CYTOPLASMIC_DOMAIN (PHOBIUS); IPR002172 (PROSITE_PROFILES); IPR002172 (PROSITE_PROFILES); IPR003609 (PROSITE_PROFILES); IPR003609 (PROSITE_PROFILES); IPR002172 (PROSITE_PROFILES); IPR003609 (PROSITE_PROFILES); IPR002172 (PROSITE_PROFILES); IPR003609 (PROSITE_PROFILES); IPR001254 (PROSITE_PROFILES); IPR003609 (PROSITE_PROFILES); IPR002172 (SUPERFAMILY); IPR009003 (SUPERFAMILY); IPR002172 (SUPERFAMILY); IPR002172 (SUPERFAMILY); IPR002172 (SUPERFAMILY); TMhelix (TMHMM); TMhelix (TMHMM)
2262	302602_length_4388_cvg_13.7_tip_1_2	952	angiotensin-converting enzyme	1462	0	59.30%	0.438	Y	Coil (COILS); Coil (COILS); IPR001548 (PRINTS); IPR002000 (PFAM); IPR001548 (PFAM); IPR001548 (PANTHER); PTHR10514:SF17 (PANTHER); CYTOPLASMIC_DOMAIN (PHOBIUS); NON_CYTOPLASMIC_DOMAIN (PHOBIUS); TRANSMEMBRANE (PHOBIUS); NON_CYTOPLASMIC_DOMAIN (PHOBIUS); TRANSMEMBRANE (PHOBIUS); TRANSMEMBRANE (PHOBIUS); TRANSMEMBRANE (PHOBIUS); CYTOPLASMIC_DOMAIN (PHOBIUS); CYTOPLASMIC_DOMAIN (PHOBIUS); SignalP-TM (SIGNALP_GRAM_POSITIVE); SSF55486 (SUPERFAMILY); SSF55486 (SUPERFAMILY); TMhelix (TMHMM); TMhelix (TMHMM); TMhelix (TMHMM)
2263	302608_length_4402_cvg_40.4_tip_1_2	3048	inter-alpha-trypsin inhibitor heavy chain h4-like	1467	2.92E-162	53.20%	0.115		Coil (COILS); IPR013694 (SMART); IPR002035 (SMART); IPR006604 (SMART); IPR013694 (PFAM); IPR002035 (G3DSA:3.40.50.GENE3D); IPR002035 (PFAM); PTHR10338:SF108 (PANTHER); PTHR10338 (PANTHER); TRANSMEMBRANE (PHOBIUS); CYTOPLASMIC_DOMAIN (PHOBIUS); NON_CYTOPLASMIC_DOMAIN (PHOBIUS); IPR002035 (PROSITE_PROFILES); IPR013694 (PROSITE_PROFILES); IPR002035 (SUPERFAMILY)

2264	302616_length_4460_cvg_26.7_tip_1_2	1303	epithelial chloride channel partial	1486	0	52.00%	0.185	Coil (COILS); IPR002035 (SMART); IPR003961 (SMART); IPR002035 (G3DSA:3.40.50.GENE3D); IPR015394 (PFAM); IPR013642 (PFAM); IPR013783 (G3DSA:2.60.40.GENE3D); PF13519 (PFAM); PTHR10579:SF8 (PANTHER); PTHR10579 (PANTHER); TRANSMEMBRANE (PHOBIUS); NON_CYTOPLASMIC_DOMAIN (PHOBIUS); TRANSMEMBRANE (PHOBIUS); TRANSMEMBRANE (PHOBIUS); CYTOPLASMIC_DOMAIN (PHOBIUS); NON_CYTOPLASMIC_DOMAIN (PHOBIUS); CYTOPLASMIC_DOMAIN (PHOBIUS); TRANSMEMBRANE (PHOBIUS); TRANSMEMBRANE (PHOBIUS); CYTOPLASMIC_DOMAIN (PHOBIUS); NON_CYTOPLASMIC_DOMAIN (PHOBIUS); IPR002035 (PROSITE_PROFILES); IPR002035 (SUPERFAMILY); IPR003961 (SUPERFAMILY); TMhelix (TMHMM); TMhelix (TMHMM); TMhelix (TMHMM)
2265	302626_length_4503_cvg_28.2_tip_1_1	1987	myosin heavy non-muscle	1501	0	89.00%	0.115	Coil (COILS); Coil (COILS); Coil (COILS); Coil (COILS); Coil (COILS); Coil (COILS); Coil (COILS); Coil (COILS); Coil (COILS); Coil (COILS); Coil (COILS); IPR001609 (SMART); IPR001609 (PFAM); G3DSA:1.20.5.340 (GENE3D); IPR002928 (PFAM); IPR027401 (G3DSA:4.10.270.GENE3D); PTHR13140 (PANTHER); PTHR13140:SF301 (PANTHER); IPR000048 (PROSITE_PROFILES); IPR001609 (PROSITE_PROFILES); SSF90257 (SUPERFAMILY); SSF57997 (SUPERFAMILY); SSF90257 (SUPERFAMILY); IPR027417 (SUPERFAMILY); SSF90257 (SUPERFAMILY); SSF90257 (SUPERFAMILY)
2266	302628_length_4515_cvg_52.9_tip_0_0	5657	aplp_locmi ame: full=apolipophorins contains: ame: full=apolipophorin-2 ame: full=apolipophorin ii ame: full=apolp-2 contains: ame: full=apolipophorin-1 ame: full=apolipophorin i ame: full=apolp-1 flags: precursor	1505	1.25E-105	49.30%	0.099	Coil (COILS); IPR001846 (SMART); IPR001846 (PFAM); IPR014853 (PFAM); PTHR23361:SF13 (PANTHER); PTHR23361 (PANTHER); CYTOPLASMIC_DOMAIN (PHOBIUS); TRANSMEMBRANE (PHOBIUS); NON_CYTOPLASMIC_DOMAIN (PHOBIUS); IPR001846 (PROSITE_PROFILES); TMhelix (TMHMM)
2267	302632_length_4535_cvg_17.1_tip_1_1	968	Supervillin, partial	1512	0	64.10%	0.099	Coil (COILS); Coil (COILS); IPR007122 (PRINTS); IPR007122 (SMART); IPR003128 (SMART); IPR003128 (PFAM); IPR007123 (PFAM); IPR003128 (G3DSA:1.10.950.GENE3D); IPR029006 (G3DSA:3.40.20.GENE3D); IPR029006 (G3DSA:3.40.20.GENE3D); IPR015628 (PTHR11977:PANTHER); IPR007122 (PANTHER); IPR003128 (PROSITE_PROFILES); SSF55753 (SUPERFAMILY); SSF55753 (SUPERFAMILY); SSF55753 (SUPERFAMILY); SSF55753 (SUPERFAMILY); SSF55753 (SUPERFAMILY); IPR003128 (SUPERFAMILY)
2268	302644_length_4679_cvg_10.7_tip_1_0	547	neuroglian-like isoform 2	1560	0	77.50%	0.387 Y	IPR003599 (SMART); IPR003961 (SMART); IPR003598 (SMART); IPR013783 (G3DSA:2.60.40.GENE3D); PF13895 (PFAM); IPR013098 (PFAM); IPR013783 (G3DSA:2.60.40.GENE3D); IPR013783 (G3DSA:2.60.40.GENE3D); IPR013783 (G3DSA:2.60.40.GENE3D); IPR013783 (G3DSA:2.60.40.GENE3D); IPR003961 (PFAM); IPR013783 (G3DSA:2.60.40.GENE3D); IPR013783 (G3DSA:2.60.40.GENE3D); IPR013783 (G3DSA:2.60.40.GENE3D); IPR026966 (PFAM); PTHR10489 (PANTHER); PTHR10489:SF553 (PANTHER); TRANSMEMBRANE (PHOBIUS); CYTOPLASMIC_DOMAIN (PHOBIUS); NON_CYTOPLASMIC_DOMAIN (PHOBIUS); IPR003961 (PROSITE_PROFILES); IPR003961 (PROSITE_PROFILES); IPR007110 (PROSITE_PROFILES); IPR003961 (PROSITE_PROFILES); IPR007110 (PROSITE_PROFILES); IPR007110 (PROSITE_PROFILES); IPR007110 (PROSITE_PROFILES); IPR003961 (PROSITE_PROFILES); IPR007110 (PROSITE_PROFILES); IPR007110 (PROSITE_PROFILES); IPR003961 (PROSITE_PROFILES); IPR003961 (SUPERFAMILY); SSF48726 (SUPERFAMILY); SSF48726 (SUPERFAMILY); SSF48726 (SUPERFAMILY); SSF48726 (SUPERFAMILY); SSF48726 (SUPERFAMILY); IPR003961 (SUPERFAMILY); SSF48726 (SUPERFAMILY); IPR003961 (SUPERFAMILY); TMhelix (TMHMM); TMhelix (TMHMM)
2269	302646_length_4684_cvg_60.3_tip_1_1	8752	von willebrand factor a domain-containing protein 7	1561	9.06E-88	44.90%	0.121	PF13519 (PFAM); PTHR14905 (PANTHER); NON_CYTOPLASMIC_DOMAIN (PHOBIUS); TRANSMEMBRANE (PHOBIUS); CYTOPLASMIC_DOMAIN (PHOBIUS); TRANSMEMBRANE (PHOBIUS); TRANSMEMBRANE (PHOBIUS); CYTOPLASMIC_DOMAIN (PHOBIUS); NON_CYTOPLASMIC_DOMAIN (PHOBIUS); IPR002035 (SUPERFAMILY); TMhelix (TMHMM); TMhelix (TMHMM); TMhelix (TMHMM)
2270	302650_length_4698_cvg_57.4_tip_1_5	5375	low quality protein: hemocytin-like	1566	1.94E-67	39.50%	0.432 Y	IPR014853 (SMART); IPR001846 (SMART); PTHR11339 (PANTHER); PTHR11339:SF25 (PANTHER); IPR001007 (PROSITE_PATTERNS); CYTOPLASMIC_DOMAIN (PHOBIUS); TRANSMEMBRANE (PHOBIUS); NON_CYTOPLASMIC_DOMAIN (PHOBIUS); IPR001846 (PROSITE_PROFILES); IPR001846 (PROSITE_PROFILES); IPR002919 (SUPERFAMILY); IPR002919 (SUPERFAMILY)

2271	302668_length_4938_cvg_29.5_tip_1_4	2238	twitchin isoform x4	1646	0	81.20%	0.108	PR00014 (PRINTS); IPR003961 (SMART); IPR003599 (SMART); IPR003598 (SMART); IPR013783 (G3DSA:2.60.40.GENE3D); IPR013783 (G3DSA:2.60.40.GENE3D); IPR013783 (G3DSA:2.60.40.GENE3D); IPR003961 (PFAM); IPR013783 (G3DSA:2.60.40.GENE3D); IPR013783 (G3DSA:2.60.40.GENE3D); IPR013783 (G3DSA:2.60.40.GENE3D); IPR013783 (G3DSA:2.60.40.GENE3D); IPR013783 (G3DSA:2.60.40.GENE3D); IPR013098 (PFAM); PTHR19897:SF155 (PANTHER); PTHR19897 (PANTHER); IPR003961 (PROSITE_PROFILES); IPR003961 (PROSITE_PROFILES); IPR003961 (PROSITE_PROFILES); IPR007110 (PROSITE_PROFILES); IPR007110 (PROSITE_PROFILES); IPR003961 (PROSITE_PROFILES); IPR003961 (PROSITE_PROFILES); IPR007110 (PROSITE_PROFILES); IPR003961 (PROSITE_PROFILES); IPR003961 (PROSITE_PROFILES); IPR003961 (PROSITE_PROFILES); IPR003961 (PROSITE_PROFILES); IPR003961 (PROSITE_PROFILES); IPR003961 (PROSITE_PROFILES); IPR003961 (SUPERFAMILY); IPR003961 (SUPERFAMILY); IPR003961 (SUPERFAMILY); SSF48726 (SUPERFAMILY); IPR003961 (SUPERFAMILY); IPR003961 (SUPERFAMILY); IPR003961 (SUPERFAMILY); SSF48726 (SUPERFAMILY); SSF48726 (SUPERFAMILY); SSF48726 (SUPERFAMILY); IPR003961 (SUPERFAMILY); SSF48726 (SUPERFAMILY); SSF48726 (SUPERFAMILY)
2272	302670_length_4938_cvg_69.6_tip_1_5	1040057	vitellogenin 2	1646	2.09E-140	43.90%	0.108	IPR001747 (SMART); IPR001846 (SMART); IPR001846 (PFAM); IPR015255 (PFAM); IPR015816 (G3DSA:2.30.230.GENE3D); IPR015818 (G3DSA:2.20.80.GENE3D); IPR011030 (G3DSA:1.25.10.GENE3D); IPR001747 (PFAM); PTHR23345 (PANTHER); PTHR23345:SF1 (PANTHER); NON_CYTOPLASMIC_DOMAIN (PHOBIUS); CYTOPLASMIC_DOMAIN (PHOBIUS); TRANSMEMBRANE (PHOBIUS); IPR001747 (PROSITE_PROFILES); IPR001846 (PROSITE_PROFILES); IPR011030 (SUPERFAMILY); IPR015819 (SUPERFAMILY); IPR015819 (SUPERFAMILY)
2273	302672_length_4975_cvg_28.2_tip_1_3	2077	otogelin-like protein	1659	2.97E-17	43.80%	0.104	PD968187 (PRODOM)
2274	302680_length_5174_cvg_49.1_tip_1_0	5673	titin- partial	1725	0	70.30%	0.1	PR00014 (PRINTS); IPR003961 (SMART); IPR003598 (SMART); IPR003599 (SMART); IPR013783 (G3DSA:2.60.40.GENE3D); IPR013783 (G3DSA:2.60.40.GENE3D); IPR013783 (G3DSA:2.60.40.GENE3D); IPR003961 (PFAM); IPR013783 (G3DSA:2.60.40.GENE3D); IPR013098 (PFAM); IPR013783 (G3DSA:2.60.40.GENE3D); IPR013783 (G3DSA:2.60.40.GENE3D); PTHR19897:SF146 (PANTHER); PTHR19897 (PANTHER); CYTOPLASMIC_DOMAIN (PHOBIUS); CYTOPLASMIC_DOMAIN (PHOBIUS); NON_CYTOPLASMIC_DOMAIN (PHOBIUS); TRANSMEMBRANE (PHOBIUS); NON_CYTOPLASMIC_DOMAIN (PHOBIUS); TRANSMEMBRANE (PHOBIUS); TRANSMEMBRANE (PHOBIUS); IPR003961 (PROSITE_PROFILES); IPR003961 (PROSITE_PROFILES); IPR003961 (PROSITE_PROFILES); IPR007110 (PROSITE_PROFILES); IPR007110 (PROSITE_PROFILES); IPR007110 (PROSITE_PROFILES); IPR007110 (PROSITE_PROFILES); IPR003961 (PROSITE_PROFILES); IPR003961 (PROSITE_PROFILES); IPR007110 (PROSITE_PROFILES); IPR007110 (PROSITE_PROFILES); SSF48726 (SUPERFAMILY); SSF48726 (SUPERFAMILY); SSF48726 (SUPERFAMILY); IPR003961 (SUPERFAMILY); SSF48726 (SUPERFAMILY); SSF48726 (SUPERFAMILY); IPR003961 (SUPERFAMILY); SSF48726 (SUPERFAMILY); IPR003961 (SUPERFAMILY); TMhelix (TMHMM); TMhelix (TMHMM); TMhelix (TMHMM)
2275	302682_length_5191_cvg_45.3_tip_1_2	4677	hemocytin isoform x2	1730	0	52.30%	0.1	IPR014853 (SMART); IPR001846 (SMART); IPR001007 (SMART); IPR006207 (SMART); IPR014853 (PFAM); G3DSA:2.10.25.10 (GENE3D); IPR001846 (PFAM); IPR002919 (PFAM); PTHR11339 (PANTHER); PTHR11339:SF25 (PANTHER); IPR001007 (PROSITE_PATTERNS); TRANSMEMBRANE (PHOBIUS); NON_CYTOPLASMIC_DOMAIN (PHOBIUS); CYTOPLASMIC_DOMAIN (PHOBIUS); NON_CYTOPLASMIC_DOMAIN (PHOBIUS); TRANSMEMBRANE (PHOBIUS); IPR001846 (PROSITE_PROFILES); IPR001846 (PROSITE_PROFILES); IPR001007 (PROSITE_PROFILES); IPR006207 (PROSITE_PROFILES); IPR002919 (SUPERFAMILY); SSF57603 (SUPERFAMILY); IPR002919 (SUPERFAMILY)

2276	302692_length_5263_cvg_28.9_tip_1_4	1741	ring finger protein nhl-1	1754	0	74.10%	0.197	Coil (COILS); Coil (COILS); IPR001841 (SMART); IPR013083 (G3DSA:3.30.40.GENE3D); IPR018957 (PFAM); IPR011042 (G3DSA:2.120.10.GENE3D); IPR001258 (PFAM); PTHR24103:SF230 (PANTHER); PTHR24103 (PANTHER); IPR013017 (PROSITE_PROFILES); IPR001841 (PROSITE_PROFILES); IPR013017 (PROSITE_PROFILES); IPR013017 (PROSITE_PROFILES); IPR013017 (PROSITE_PROFILES); IPR013017 (PROSITE_PROFILES); IPR013017 (PROSITE_PROFILES); IPR000315 (PROSITE_PROFILES); IPR013017 (PROSITE_PROFILES); SSF57850 (SUPERFAMILY); SSF101898 (SUPERFAMILY)
2277	302702_length_5374_cvg_64.8_tip_1_2	259270	vitellogenin fused with superoxide dismutase	1791	0	45.70%	0.196	IPR001747 (SMART); IPR001846 (SMART); IPR015816 (G3DSA:2.30.230.GENE3D); IPR015255 (PFAM); IPR015818 (G3DSA:2.20.80.GENE3D); IPR001846 (PFAM); IPR011030 (G3DSA:1.25.10.GENE3D); IPR001747 (PFAM); PTHR23345:SF1 (PANTHER); PTHR23345 (PANTHER); TRANSMEMBRANE (PHOBIUS); TRANSMEMBRANE (PHOBIUS); NON_CYTOPLASMIC_DOMAIN (PHOBIUS); CYTOPLASMIC_DOMAIN (PHOBIUS); CYTOPLASMIC_DOMAIN (PHOBIUS); NON_CYTOPLASMIC_DOMAIN (PHOBIUS); TRANSMEMBRANE (PHOBIUS); IPR001846 (PROSITE_PROFILES); IPR001747 (PROSITE_PROFILES); IPR015819 (SUPERFAMILY); IPR015819 (SUPERFAMILY); IPR011030 (SUPERFAMILY); TMhelix (TMHMM)
2278	302706_length_5387_cvg_62.9_tip_1_0	101262	hypothetical protein DAPPUDRAFT_332703	1796	0	51.90%	0.107	Coil (COILS); IPR001846 (SMART); IPR014853 (SMART); IPR014853 (PFAM); IPR002035 (G3DSA:3.40.50.GENE3D); IPR001846 (PFAM); PTHR23361 (PANTHER); PTHR23361:SF13 (PANTHER); NON_CYTOPLASMIC_DOMAIN (PHOBIUS); NON_CYTOPLASMIC_DOMAIN (PHOBIUS); CYTOPLASMIC_DOMAIN (PHOBIUS); TRANSMEMBRANE (PHOBIUS); CYTOPLASMIC_DOMAIN (PHOBIUS); TRANSMEMBRANE (PHOBIUS); NON_CYTOPLASMIC_DOMAIN (PHOBIUS); TRANSMEMBRANE (PHOBIUS); TRANSMEMBRANE (PHOBIUS); IPR001846 (PROSITE_PROFILES); IPR002035 (SUPERFAMILY); TMhelix (TMHMM); TMhelix (TMHMM); TMhelix (TMHMM); TMhelix (TMHMM)
2279	302718_length_5567_cvg_34.7_tip_1_1	4412	myosin light chain smooth muscle	1856	0	59.20%	0.112	Coil (COILS); IPR003599 (SMART); IPR003598 (SMART); IPR003961 (SMART); IPR013106 (SMART); IPR013783 (G3DSA:2.60.40.GENE3D); IPR013783 (G3DSA:2.60.40.GENE3D); IPR003961 (PFAM); IPR013783 (G3DSA:2.60.40.GENE3D); IPR013783 (G3DSA:2.60.40.GENE3D); IPR013783 (G3DSA:2.60.40.GENE3D); IPR013098 (PFAM); IPR013783 (G3DSA:2.60.40.GENE3D); IPR013783 (G3DSA:2.60.40.GENE3D); IPR013783 (G3DSA:2.60.40.GENE3D); PTHR19897 (PANTHER); IPR007110 (PROSITE_PROFILES); IPR007110 (PROSITE_PROFILES); IPR007110 (PROSITE_PROFILES); IPR007110 (PROSITE_PROFILES); IPR007110 (PROSITE_PROFILES); IPR007110 (PROSITE_PROFILES); IPR007110 (PROSITE_PROFILES); IPR007110 (PROSITE_PROFILES); IPR007110 (PROSITE_PROFILES); IPR007110 (PROSITE_PROFILES); IPR003961 (PROSITE_PROFILES); IPR007110 (PROSITE_PROFILES); SSF48726 (SUPERFAMILY); SSF48726 (SUPERFAMILY); SSF48726 (SUPERFAMILY); SSF48726 (SUPERFAMILY); SSF48726 (SUPERFAMILY); SSF48726 (SUPERFAMILY); SSF48726 (SUPERFAMILY); SSF48726 (SUPERFAMILY); SSF48726 (SUPERFAMILY); IPR003961 (SUPERFAMILY); SSF48726 (SUPERFAMILY); SSF48726 (SUPERFAMILY)
2280	302722_length_5657_cvg_43.2_tip_1_5	3296	von willebrand factor	1885	1.64E-27	41.50%	0.11	IPR001007 (SMART); IPR001007 (PROSITE_PROFILES); IPR006207 (PROSITE_PROFILES)

2281	302740_length_6173_cvg_34.6_tip_1_5	3001	PREDICTED: acrosin-like	2057	8.21E-09	46.00%	0.146	CYTOPLASMIC_DOMAIN (PHOBIUS); TRANSMEMBRANE (PHOBIUS); CYTOPLASMIC_DOMAIN (PHOBIUS); CYTOPLASMIC_DOMAIN (PHOBIUS); TRANSMEMBRANE (PHOBIUS); NON_CYTOPLASMIC_DOMAIN (PHOBIUS); TRANSMEMBRANE (PHOBIUS); NON_CYTOPLASMIC_DOMAIN (PHOBIUS); NON_CYTOPLASMIC_DOMAIN (PHOBIUS); CYTOPLASMIC_DOMAIN (PHOBIUS); TRANSMEMBRANE (PHOBIUS); TRANSMEMBRANE (PHOBIUS); TRANSMEMBRANE (PHOBIUS); CYTOPLASMIC_DOMAIN (PHOBIUS); TRANSMEMBRANE (PHOBIUS); NON_CYTOPLASMIC_DOMAIN (PHOBIUS); NON_CYTOPLASMIC_DOMAIN (PHOBIUS); NON_CYTOPLASMIC_DOMAIN (PHOBIUS); CYTOPLASMIC_DOMAIN (PHOBIUS); TRANSMEMBRANE (PHOBIUS); CYTOPLASMIC_DOMAIN (PHOBIUS); NON_CYTOPLASMIC_DOMAIN (PHOBIUS); NON_CYTOPLASMIC_DOMAIN (PHOBIUS); NON_CYTOPLASMIC_DOMAIN (PHOBIUS); TRANSMEMBRANE (PHOBIUS); TRANSMEMBRANE (PHOBIUS); NON_CYTOPLASMIC_DOMAIN (PHOBIUS); TRANSMEMBRANE (PHOBIUS); CYTOPLASMIC_DOMAIN (PHOBIUS); NON_CYTOPLASMIC_DOMAIN (PHOBIUS); TRANSMEMBRANE (PHOBIUS); CYTOPLASMIC_DOMAIN (PHOBIUS); TRANSMEMBRANE (PHOBIUS); TRANSMEMBRANE (PHOBIUS); TRANSMEMBRANE (PHOBIUS); TRANSMEMBRANE (PHOBIUS); CYTOPLASMIC_DOMAIN (PHOBIUS); TRANSMEMBRANE (PHOBIUS); NON_CYTOPLASMIC_DOMAIN (PHOBIUS); NON_CYTOPLASMIC_DOMAIN (PHOBIUS); CYTOPLASMIC_DOMAIN (PHOBIUS); TRANSMEMBRANE (PHOBIUS); CYTOPLASMIC_DOMAIN (PHOBIUS); TRANSMEMBRANE (PHOBIUS); TRANSMEMBRANE (PHOBIUS); TRANSMEMBRANE (PHOBIUS); NON_CYTOPLASMIC_DOMAIN (PHOBIUS); TRANSMEMBRANE (PHOBIUS); NON_CYTOPLASMIC_DOMAIN (PHOBIUS); TRANSMEMBRANE (PHOBIUS); TRANSMEMBRANE (PHOBIUS); TRANSMEMBRANE (PHOBIUS); TRANSMEMBRANE (PHOBIUS); NON_CYTOPLASMIC_DOMAIN (PHOBIUS); TRANSMEMBRANE (PHOBIUS); TRANSMEMBRANE (PHOBIUS); CYTOPLASMIC_DOMAIN (PHOBIUS); TRANSMEMBRANE (PHOBIUS); CYTOPLASMIC_DOMAIN (PHOBIUS); CYTOPLASMIC_DOMAIN (PHOBIUS); NON_CYTOPLASMIC_DOMAIN (PHOBIUS); TRANSMEMBRANE (PHOBIUS); TRANSMEMBRANE (PHOBIUS); CYTOPLASMIC_DOMAIN (PHOBIUS); TRANSMEMBRANE (PHOBIUS); CYTOPLASMIC_DOMAIN (PHOBIUS); CYTOPLASMIC_DOMAIN (PHOBIUS); NON_CYTOPLASMIC_DOMAIN (PHOBIUS); NON_CYTOPLASMIC_DOMAIN
2282	302760_length_6729_cvg_39.9_tip_1_4	5049	titin- partial	2243	0	60.90%	0.101	Coil (COILS); Coil (COILS); IPR003598 (SMART); IPR001452 (SMART); IPR003599 (SMART); G3DSA:2.30.30.40 (GENE3D); IPR013783 (G3DSA:2.60.40.GENE3D); IPR013098 (PFAM); IPR013783 (G3DSA:2.60.40.GENE3D); IPR013783 (G3DSA:2.60.40.GENE3D); IPR013783 (G3DSA:2.60.40.GENE3D); IPR001452 (PFAM); PTHR19897 (PANTHER); IPR007110 (PROSITE_PROFILES); IPR007110 (PROSITE_PROFILES); IPR001452 (PROSITE_PROFILES); IPR007110 (PROSITE_PROFILES); IPR007110 (PROSITE_PROFILES); IPR007110 (PROSITE_PROFILES); IPR001452 (SUPERFAMILY); SSF48726 (SUPERFAMILY); SSF48726 (SUPERFAMILY); SSF48726 (SUPERFAMILY); SSF48726 (SUPERFAMILY); SSF48726 (SUPERFAMILY)
2283	302762_length_7189_cvg_30.7_tip_1_5	2638	hemocytin isoform x4	2396	2.89E-37	43.00%	0.137	IPR001846 (SMART); PTHR11339 (PANTHER); IPR001846 (PROSITE_PROFILES); IPR002919 (SUPERFAMILY)
2284	302768_length_7907_cvg_14.8_tip_1_2	1825	multiple ankyrin repeats single kh domain	2635	0	61.80%	0.101	Coil (COILS); Coil (COILS); Coil (COILS); Coil (COILS); Coil (COILS); IPR002110 (PRINTS); IPR002110 (SMART); IPR004087 (SMART); IPR004088 (PFAM); IPR020683 (G3DSA:1.25.40.GENE3D); IPR020683 (G3DSA:1.25.40.GENE3D); IPR004088 (G3DSA:3.30.1370.GENE3D); IPR020683 (PFAM); PTHR23206 (PANTHER); NON_CYTOPLASMIC_DOMAIN (PHOBIUS); NON_CYTOPLASMIC_DOMAIN (PHOBIUS); TRANSMEMBRANE (PHOBIUS); TRANSMEMBRANE (PHOBIUS); TRANSMEMBRANE (PHOBIUS); NON_CYTOPLASMIC_DOMAIN (PHOBIUS); CYTOPLASMIC_DOMAIN (PHOBIUS); CYTOPLASMIC_DOMAIN (PHOBIUS); TRANSMEMBRANE (PHOBIUS); CYTOPLASMIC_DOMAIN (PHOBIUS); TRANSMEMBRANE (PHOBIUS); IPR002110 (PROSITE_PROFILES); IPR002110 (PROSITE_PROFILES); IPR002110 (PROSITE_PROFILES); IPR002110 (PROSITE_PROFILES); IPR002110 (PROSITE_PROFILES); IPR020683 (PROSITE_PROFILES); IPR002110 (PROSITE_PROFILES); IPR002110 (PROSITE_PROFILES); IPR004088 (PROSITE_PROFILES); IPR002110 (PROSITE_PROFILES); IPR002110 (PROSITE_PROFILES); IPR004088 (SUPERFAMILY); IPR020683 (SUPERFAMILY); TMhelix (TMHMM); TMhelix (TMHMM)

2285	302774_length_8282_cvg_35.1_tip_1_1	6492	muscle m-line assembly protein unc-89	2761	0	68.70%	0.11	IPR003599 (SMART); IPR003961 (SMART); IPR003598 (SMART); IPR013098 (PFAM); G3DSA:1.10.510.10 (GENE3D); G3DSA:3.30.200.20 (GENE3D); IPR013783 (G3DSA:2.60.40.GENE3D); G3DSA:3.30.200.20 (GENE3D); IPR013783 (G3DSA:2.60.40.GENE3D); IPR013783 (G3DSA:2.60.40.GENE3D); IPR013783 (G3DSA:2.60.40.GENE3D); IPR013783 (G3DSA:2.60.40.GENE3D); IPR013783 (G3DSA:2.60.40.GENE3D); G3DSA:1.10.510.10 (GENE3D); IPR013783 (G3DSA:2.60.40.GENE3D); IPR013783 (G3DSA:2.60.40.GENE3D); IPR013783 (G3DSA:2.60.40.GENE3D); IPR013783 (G3DSA:2.60.40.GENE3D); IPR003961 (PFAM); IPR000719 (PFAM); IPR020675 (PANTHER); PTHR22964:SF45 (PANTHER); IPR017441 (PROSITE_PATTERNS); IPR007110 (PROSITE_PROFILES); IPR007110 (PROSITE_PROFILES); IPR007110 (PROSITE_PROFILES); IPR007110 (PROSITE_PROFILES); IPR007110 (PROSITE_PROFILES); IPR007110 (PROSITE_PROFILES); IPR003961 (PROSITE_PROFILES); IPR007110 (PROSITE_PROFILES); IPR007110 (PROSITE_PROFILES); IPR007110 (PROSITE_PROFILES); IPR007110 (PROSITE_PROFILES); IPR007110 (PROSITE_PROFILES); IPR000719 (PROSITE_PROFILES); IPR007110 (PROSITE_PROFILES); IPR003961 (PROSITE_PROFILES); IPR000719 (PROSITE_PROFILES); SSF48726 (SUPERFAMILY); SSF48726 (SUPERFAMILY); SSF48726 (SUPERFAMILY); SSF48726 (SUPERFAMILY); IPR003961 (SUPERFAMILY); SSF48726 (SUPERFAMILY); SSF48726 (SUPERFAMILY); SSF48726 (SUPERFAMILY); SSF48726 (SUPERFAMILY); IPR011009 (SUPERFAMILY); SSF48726 (SUPERFAMILY); SSF48726 (SUPERFAMILY); SSF48726 (SUPERFAMILY); SSF48726 (SUPERFAMILY); SSF48726 (SUPERFAMILY); IPR011009 (SUPERFAMILY)
2286	302784_length_11450_cvg_34.2_tip_1_0	14647	beta- -glucan-binding protein	3817	0	39.40%	0.096	no IPS match
2288	302790_length_15619_cvg_32.5_tip_1_2	16248	mucin- partial	5206	2.27E-11	42.29%	0.152	NON_CYTOPLASMIC_DOMAIN (PHOBIUS); TRANSMEMBRANE (PHOBIUS); TRANSMEMBRANE (PHOBIUS); NON_CYTOPLASMIC_DOMAIN (PHOBIUS); TRANSMEMBRANE (PHOBIUS); NON_CYTOPLASMIC_DOMAIN (PHOBIUS); CYTOPLASMIC_DOMAIN (PHOBIUS); NON_CYTOPLASMIC_DOMAIN (PHOBIUS); TRANSMEMBRANE (PHOBIUS); TRANSMEMBRANE (PHOBIUS); TRANSMEMBRANE (PHOBIUS); CYTOPLASMIC_DOMAIN (PHOBIUS); CYTOPLASMIC_DOMAIN (PHOBIUS); NON_CYTOPLASMIC_DOMAIN (PHOBIUS); TRANSMEMBRANE (PHOBIUS); NON_CYTOPLASMIC_DOMAIN (PHOBIUS); TRANSMEMBRANE (PHOBIUS); TRANSMEMBRANE (PHOBIUS); TRANSMEMBRANE (PHOBIUS); CYTOPLASMIC_DOMAIN (PHOBIUS); NON_CYTOPLASMIC_DOMAIN (PHOBIUS); TRANSMEMBRANE (PHOBIUS); TRANSMEMBRANE (PHOBIUS); TRANSMEMBRANE (PHOBIUS); TRANSMEMBRANE (PHOBIUS); CYTOPLASMIC_DOMAIN (PHOBIUS); TRANSMEMBRANE (PHOBIUS); TRANSMEMBRANE (PHOBIUS); TRANSMEMBRANE (PHOBIUS); TRANSMEMBRANE (PHOBIUS); CYTOPLASMIC_DOMAIN (PHOBIUS); TRANSMEMBRANE (PHOBIUS); CYTOPLASMIC_DOMAIN (PHOBIUS); TRANSMEMBRANE (PHOBIUS); CYTOPLASMIC_DOMAIN (PHOBIUS); TRANSMEMBRANE (PHOBIUS); TRANSMEMBRANE (PHOBIUS); CYTOPLASMIC_DOMAIN (PHOBIUS); CYTOPLASMIC_DOMAIN (PHOBIUS); TRANSMEMBRANE (PHOBIUS); TRANSMEMBRANE (PHOBIUS); TRANSMEMBRANE (PHOBIUS); TRANSMEMBRANE (PHOBIUS); NON_CYTOPLASMIC_DOMAIN (PHOBIUS); TRANSMEMBRANE (PHOBIUS); CYTOPLASMIC_DOMAIN (PHOBIUS); NON_CYTOPLASMIC_DOMAIN (PHOBIUS); NON_CYTOPLASMIC_DOMAIN (PHOBIUS); CYTOPLASMIC_DOMAIN (PHOBIUS); NON_CYTOPLASMIC_DOMAIN (PHOBIUS); TRANSMEMBRANE (PHOBIUS); TRANSMEMBRANE (PHOBIUS); CYTOPLASMIC_DOMAIN (PHOBIUS); NON_CYTOPLASMIC_DOMAIN (PHOBIUS); TRANSMEMBRANE (PHOBIUS); TRANSMEMBRANE (PHOBIUS); NON_CYTOPLASMIC_DOMAIN (PHOBIUS); CYTOPLASMIC_DOMAIN (PHOBIUS); TRANSMEMBRANE (PHOBIUS); TRANSMEMBRANE (PHOBIUS); CYTOPLASMIC_DOMAIN (PHOBIUS); CYTOPLASMIC_DOMAIN (PHOBIUS); CYTOPLASMIC_DOMAIN (PHOBIUS); NON_CYTOPLASMIC_DOMAIN (PHOBIUS); NON_CYTOPLASMIC_DOMAIN (PHOBIUS); NON_CYTOPLASMIC_DOMAIN (PHOBIUS); CYTOPLASMIC_DOMAIN (PHOBIUS); NON_CYTOPLASMIC_DOMAIN

2287	302790_length_15619_cvg_32.5_tip_1_0	16248	coagulation factor v	5207	1.93E-12	41.30%	0.292	TRANSMEMBRANE (PHOBIUS); NON_CYTOPLASMIC_DOMAIN (PHOBIUS); NON_CYTOPLASMIC_DOMAIN (PHOBIUS); SIGNAL_PEPTIDE_C_REGION (PHOBIUS); TRANSMEMBRANE (PHOBIUS); CYTOPLASMIC_DOMAIN (PHOBIUS); CYTOPLASMIC_DOMAIN (PHOBIUS); TRANSMEMBRANE (PHOBIUS); NON_CYTOPLASMIC_DOMAIN (PHOBIUS); NON_CYTOPLASMIC_DOMAIN (PHOBIUS); TRANSMEMBRANE (PHOBIUS); TRANSMEMBRANE (PHOBIUS); SIGNAL_PEPTIDE_H_REGION (PHOBIUS); CYTOPLASMIC_DOMAIN (PHOBIUS); TRANSMEMBRANE (PHOBIUS); TRANSMEMBRANE (PHOBIUS); NON_CYTOPLASMIC_DOMAIN (PHOBIUS); TRANSMEMBRANE (PHOBIUS); NON_CYTOPLASMIC_DOMAIN (PHOBIUS); TRANSMEMBRANE (PHOBIUS); TRANSMEMBRANE (PHOBIUS); TRANSMEMBRANE (PHOBIUS); NON_CYTOPLASMIC_DOMAIN (PHOBIUS); TRANSMEMBRANE (PHOBIUS); CYTOPLASMIC_DOMAIN (PHOBIUS); TRANSMEMBRANE (PHOBIUS); CYTOPLASMIC_DOMAIN (PHOBIUS); NON_CYTOPLASMIC_DOMAIN (PHOBIUS); CYTOPLASMIC_DOMAIN (PHOBIUS); NON_CYTOPLASMIC_DOMAIN (PHOBIUS); TRANSMEMBRANE (PHOBIUS); TRANSMEMBRANE (PHOBIUS); TRANSMEMBRANE (PHOBIUS); NON_CYTOPLASMIC_DOMAIN (PHOBIUS); TRANSMEMBRANE (PHOBIUS); TRANSMEMBRANE (PHOBIUS); NON_CYTOPLASMIC_DOMAIN (PHOBIUS); CYTOPLASMIC_DOMAIN (PHOBIUS); CYTOPLASMIC_DOMAIN (PHOBIUS); TRANSMEMBRANE (PHOBIUS); NON_CYTOPLASMIC_DOMAIN (PHOBIUS); CYTOPLASMIC_DOMAIN (PHOBIUS); TRANSMEMBRANE (PHOBIUS); TRANSMEMBRANE (PHOBIUS); CYTOPLASMIC_DOMAIN (PHOBIUS); CYTOPLASMIC_DOMAIN (PHOBIUS); NON_CYTOPLASMIC_DOMAIN (PHOBIUS); NON_CYTOPLASMIC_DOMAIN (PHOBIUS); TRANSMEMBRANE (PHOBIUS); NON_CYTOPLASMIC_DOMAIN (PHOBIUS); CYTOPLASMIC_DOMAIN (PHOBIUS); TRANSMEMBRANE (PHOBIUS); TRANSMEMBRANE (PHOBIUS); CYTOPLASMIC_DOMAIN (PHOBIUS); CYTOPLASMIC_DOMAIN (PHOBIUS); NON_CYTOPLASMIC_DOMAIN (PHOBIUS); TRANSMEMBRANE (PHOBIUS); TRANSMEMBRANE (PHOBIUS); NON_CYTOPLASMIC_DOMAIN (PHOBIUS); CYTOPLASMIC_DOMAIN (PHOBIUS); TRANSMEMBRANE (PHOBIUS); TRANSMEMBRANE (PHOBIUS); CYTOPLASMIC_DOMAIN (PHOBIUS); TRANSMEMBRANE (PHOBIUS); NON_CYTOPLASMIC_DOMAIN (PHOBIUS); CYTOPLASMIC_DOMAIN (PHOBIUS); NON_CYTOPLASMIC_DOMAIN (PHOBIUS); TRANSMEMBRANE
2289	302790_length_15619_cvg_32.5_tip_1_3	16248	mucin 2	5207	7.39E-66	47.80%	0.102	Coil (COILS); Coil (COILS); Coil (COILS); Coil (COILS); Coil (COILS); Coil (COILS); Coil (COILS); Coil (COILS)

Supplementary Table 7: Whole body tissue transcriptome (Trinity contigs that match against secreted proteins [UniProtSL0243)]

Nr.	Contig name	Read Numbers	Blast result (evalue=10^{-5}, BlastP, Nr database)	Seq. length	min. evalue	mean similarity	SigalP	InterProScan
1	c100101_g1_i1_len_662_path_1_0_661_1	16	tolloid-like protein 2 isoform x1	221	1.41E-75	64.60%	0.118	IPR002172 (SMART); IPR000859 (SMART); IPR000859 (PFAM); IPR000859 (G3DSA:2.60.120.GENE3D); IPR002172 (G3DSA:4.10.400.GENE3D); G3DSA:1.20.5.900 (GENE3D); PTHR10127:SF557 (PANTHER); PTHR10127 (PANTHER); NON_CYTOPLASMIC_DOMAIN (PHOBIUS); CYTOPLASMIC_DOMAIN (PHOBIUS); TRANSMEMBRANE (PHOBIUS); IPR000859 (PROSITE_PROFILES); IPR002172 (SUPERFAMILY); IPR000859 (SUPERFAMILY); TMhelix (TMHMM)
2	c100109_g1_i1_len_465_path_443_0_464_0	11	leucomyosuppressin precursor	155	6.19E-36	71.40%	0.262	NON_CYTOPLASMIC_DOMAIN (PHOBIUS); TRANSMEMBRANE (PHOBIUS); CYTOPLASMIC_DOMAIN (PHOBIUS); TMhelix (TMHMM)
3	c100138_g1_i1_len_390_path_1_0_389_0	10	isoform c	130	3.59E-50	79.90%	0.109	IPR000716 (SMART); IPR019577 (PFAM); IPR000716 (PFAM); IPR000716 (G3DSA:4.10.800.GENE3D); IPR011992 (G3DSA:1.10.238.GENE3D); PTHR12036 (PANTHER); IPR000716 (PROSITE_PATTERNS); IPR000716 (PROSITE_PROFILES); IPR000716 (SUPERFAMILY)
4	c100142_g1_i1_len_598_path_1_0_597_5	14	cadherin-related tumor suppressor	199	2.52E-72	74.40%	0.102	IPR002126 (SMART); IPR002126 (G3DSA:2.60.40.GENE3D); IPR002126 (PFAM); PTHR24026 (PANTHER); PTHR24026:SF41 (PANTHER); IPR002126 (PROSITE_PROFILES); IPR015919 (SUPERFAMILY); IPR015919 (SUPERFAMILY)
5	c100151_g1_i1_len_811_path_1_0_810_4	44	ecto-nox disulfide-thiol exchanger 2	270	1.73E-57	74.50%	0.1	Coil (COILS); PTHR16001 (PANTHER)
6	c100167_g1_i1_len_280_path_1_0_279_5	7	notch-regulated ankyrin repeat-containing protein	93	1.56E-32	74.40%	0.104	IPR002110 (SMART); PF13857 (PFAM); IPR020683 (G3DSA:1.25.40.GENE3D); PTHR24203 (PANTHER); IPR002110 (PROSITE_PROFILES); IPR020683 (PROSITE_PROFILES); IPR020683 (SUPERFAMILY)
7	c100323_g1_i1_len_255_path_1_0_254_2	4	torso-like protein isoform x2	85	3.62E-17	67.60%	0.118	no IPS match
8	c100372_g1_i1_len_234_path_212_0_233_3	2	lactose-binding lectin l-2-like	78	1.46E-08	59.10%	0.102	IPR016186 (G3DSA:3.10.100.GENE3D); IPR001304 (PFAM); PTHR22802 (PANTHER); IPR001304 (PROSITE_PROFILES); IPR016187 (SUPERFAMILY)
9	c100512_g1_i1_len_268_path_246_0_267_0	4	serine threonine-protein kinase pak 1	90	4.64E-49	93.00%	0.096	G3DSA:1.10.510.10 (GENE3D); IPR000719 (PFAM); PTHR24361 (PANTHER); PTHR24361:SF245 (PANTHER); IPR008271 (PROSITE_PATTERNS); IPR000719 (PROSITE_PROFILES); IPR011009 (SUPERFAMILY)
10	c100542_g1_i1_len_897_path_1_0_896_0	28	carbohydrate sulfotransferase 11	299	1.54E-70	65.40%	0.142	IPR005331 (PFAM); IPR018011 (PANTHER); PTHR12137:SF30 (PANTHER); IPR027417 (SUPERFAMILY)
11	c100584_g1_i1_len_303_path_281_0_302_3	6	oocyte zinc finger protein 20	101	4.73E-12	79.60%	0.099	IPR015880 (SMART); IPR013087 (G3DSA:3.30.160.GENE3D); IPR013087 (G3DSA:3.30.160.GENE3D); PF13465 (PFAM); PTHR24409 (PANTHER); IPR007087 (PROSITE_PATTERNS); IPR007087 (PROSITE_PROFILES); IPR007087 (PROSITE_PROFILES); SSF57667 (SUPERFAMILY)
12	c100807_g1_i1_len_324_path_1_0_323_3	11	aqp_aedae ame: full=aquaporin	108	8.34E-20	70.00%	0.21	IPR000425 (PRINTS); IPR023271 (G3DSA:1.20.1080.GENE3D); IPR000425 (PFAM); IPR000425 (PANTHER); PTHR19139:SF45 (PANTHER); IPR022357 (PROSITE_PATTERNS); TRANSMEMBRANE (PHOBIUS); NON_CYTOPLASMIC_DOMAIN (PHOBIUS); CYTOPLASMIC_DOMAIN (PHOBIUS); CYTOPLASMIC_DOMAIN (PHOBIUS); TRANSMEMBRANE (PHOBIUS); IPR023271 (SUPERFAMILY); TMhelix (TMHMM); TMhelix (TMHMM)
13	c100993_g1_i1_len_608_path_586_0_607_0	13	upf0364 protein c6orf211 homolog	203	6.77E-57	64.10%	0.153	IPR002791 (PFAM); PTHR12260:SF1 (PANTHER); PTHR12260 (PANTHER); IPR002791 (SUPERFAMILY)
14	c101109_g1_i1_len_1509_path_1_0_1508_2	64	neuropeptides capa receptor-like	503	3.90E-149	74.20%	0.209	Coil (COILS); IPR000276 (PRINTS); IPR005390 (PRINTS); G3DSA:1.20.1070.10 (GENE3D); IPR000276 (PFAM); PTHR24243 (PANTHER); PTHR24243:SF107 (PANTHER); IPR000276 (PROSITE_PATTERNS); NON_CYTOPLASMIC_DOMAIN (PHOBIUS); CYTOPLASMIC_DOMAIN (PHOBIUS); CYTOPLASMIC_DOMAIN (PHOBIUS); TRANSMEMBRANE (PHOBIUS); TRANSMEMBRANE (PHOBIUS); TRANSMEMBRANE (PHOBIUS); TRANSMEMBRANE (PHOBIUS); TRANSMEMBRANE (PHOBIUS); NON_CYTOPLASMIC_DOMAIN (PHOBIUS); CYTOPLASMIC_DOMAIN (PHOBIUS); TRANSMEMBRANE (PHOBIUS); CYTOPLASMIC_DOMAIN (PHOBIUS); NON_CYTOPLASMIC_DOMAIN (PHOBIUS); TRANSMEMBRANE (PHOBIUS); NON_CYTOPLASMIC_DOMAIN (PHOBIUS); IPR017452 (PROSITE_PROFILES); SSF81321 (SUPERFAMILY); TMhelix (TMHMM); TMhelix (TMHMM); TMhelix (TMHMM); TMhelix (TMHMM); TMhelix (TMHMM); TMhelix (TMHMM); TMhelix (TMHMM)
15	c101175_g1_i1_len_651_path_1_0_650_0	18	h aca ribonucleoprotein complex non-core subunit naf1	217	1.10E-52	76.90%	0.379Y	G3DSA:2.40.10.230 (GENE3D); IPR007504 (PFAM); PTHR31991 (PANTHER); IPR009000 (SUPERFAMILY)

16	c10122_g2_i1_len_364_path_596_0_363_2	6	limbic system-associated membrane protein	121	4.24E-26	57.60%	0.108	IPR013098 (PFAM); PF13895 (PFAM); IPR013783 (G3DSA:2.60.40.GENE3D); IPR013783 (G3DSA:2.60.40.GENE3D); PTHR19831 (PANTHER); PTHR19831:SF40 (PANTHER); IPR007110 (PROSITE_PROFILES); SSF48726 (SUPERFAMILY); SSF48726 (SUPERFAMILY)
17	c101367_g1_i1_len_905_path_1_0_904_2	292	flowering locus t	301	6.11E-24	58.30%	0.189	IPR008914 (G3DSA:3.90.280.GENE3D); IPR008914 (PFAM); PTHR11362 (PANTHER); IPR001858 (PROSITE_PATTERNS); CYTOPLASMIC_DOMAIN (PHOBIUS); NON_CYTOPLASMIC_DOMAIN (PHOBIUS); TRANSMEMBRANE (PHOBIUS); IPR008914 (SUPERFAMILY)
18	c101418_g1_i1_len_958_path_936_0_957_1	63	reticulocalbin-2	319	1.11E-97	70.20%	0.410Y	IPR002048 (SMART); IPR011992 (PFAM); IPR002048 (PFAM); IPR011992 (G3DSA:1.10.238.GENE3D); IPR011992 (G3DSA:1.10.238.GENE3D); PTHR10827:SF48 (PANTHER); PTHR10827 (PANTHER); IPR018247 (PROSITE_PATTERNS); IPR018247 (PROSITE_PATTERNS); IPR018247 (PROSITE_PATTERNS); IPR018247 (PROSITE_PATTERNS); IPR018247 (PROSITE_PATTERNS); IPR002048 (PROSITE_PROFILES); IPR002048 (PROSITE_PROFILES); IPR002048 (PROSITE_PROFILES); IPR002048 (PROSITE_PROFILES); IPR002048 (PROSITE_PROFILES); SSF47473 (SUPERFAMILY)
19	c101553_g1_i1_len_677_path_655_0_676_2	16	transient receptor potential channel pyrexia-like	225	1.79E-26	49.60%	0.168	IPR002110 (SMART); IPR020683 (PFAM); IPR020683 (G3DSA:1.25.40.GENE3D); PTHR24188 (PANTHER); TRANSMEMBRANE (PHOBIUS); CYTOPLASMIC_DOMAIN (PHOBIUS); NON_CYTOPLASMIC_DOMAIN (PHOBIUS); IPR020683 (PROSITE_PROFILES); IPR002110 (PROSITE_PROFILES); IPR002110 (PROSITE_PROFILES); IPR020683 (SUPERFAMILY)
20	c101573_g1_i1_len_495_path_473_0_494_0	6	scp-like extracellular domain containing protein 1	165	2.37E-23	53.70%	0.117	IPR001283 (PRINTS); IPR014044 (SMART); IPR014044 (PFAM); IPR014044 (G3DSA:3.40.33.GENE3D); IPR001283 (PANTHER); IPR014044 (SUPERFAMILY)
21	c10163_g1_i1_len_340_path_576_0_339_3	6	low quality protein: contactin-like	114	1.77E-27	61.20%	0.212	IPR003961 (SMART); IPR003961 (PFAM); IPR013783 (G3DSA:2.60.40.GENE3D); PTHR10489 (PANTHER); IPR003961 (PROSITE_PROFILES); IPR003961 (SUPERFAMILY)
22	c101875_g1_i1_len_341_path_1_0_340_2	4	kielin chordin-like protein	113	1.04E-18	54.50%	0.152	IPR013320 (SUPERFAMILY)
23	c101907_g1_i1_len_1228_path_1206_0_1227_0	22	aael001549- partial	410	0	95.10%	0.14	IPR000961 (SMART); IPR002290 (SMART); G3DSA:3.30.200.20 (GENE3D); G3DSA:1.10.510.10 (GENE3D); IPR000719 (PFAM); IPR017892 (PFAM); PTHR24357 (PANTHER); PTHR24357:SF80 (PANTHER); IPR008271 (PROSITE_PATTERNS); NON_CYTOPLASMIC_DOMAIN (PHOBIUS); TRANSMEMBRANE (PHOBIUS); CYTOPLASMIC_DOMAIN (PHOBIUS); IPR000961 (PROSITE_PROFILES); IPR000719 (PROSITE_PROFILES); IPR011009 (SUPERFAMILY)
24	c101978_g1_i1_len_685_path_663_0_684_0	20	succinate-semialdehyde mitochondrial	229	6.25E-89	79.70%	0.131	IPR016162 (G3DSA:3.40.605.GENE3D); IPR015590 (PFAM); PTHR11699:SF49 (PANTHER); PTHR11699 (PANTHER); IPR016161 (SUPERFAMILY)
25	c101985_g1_i1_len_405_path_1_0_404_0	8	mam and ldl-receptor class a domain-containing protein 2-like	135	1.02E-07	43.50%	0.099	IPR000998 (PFAM); PTHR23282 (PANTHER); PTHR23282:SF75 (PANTHER); IPR013320 (SUPERFAMILY)
26	c102102_g1_i1_len_431_path_409_0_430_5	7	domain and thioredoxin-containing	143	7.65E-49	70.00%	0.111	PR00421 (PRINTS); IPR013766 (PFAM); IPR012336 (G3DSA:3.40.30.GENE3D); PTHR24078 (PANTHER); PTHR24078:SF3 (PANTHER); IPR017937 (PROSITE_PATTERNS); IPR012336 (PROSITE_PROFILES); IPR012336 (SUPERFAMILY)
27	c102112_g1_i1_len_663_path_1_0_662_2	26	enoyl- delta isomerase mitochondrial-like	221	6.95E-58	68.00%	0.164	Coil (COILS); IPR000582 (PRINTS); IPR001753 (PFAM); IPR000582 (PFAM); IPR029045 (G3DSA:3.90.226.GENE3D); IPR014352 (G3DSA:1.20.80.GENE3D); PTHR23310 (PANTHER); PTHR23310:SF55 (PANTHER); IPR022408 (PROSITE_PATTERNS); IPR000582 (PROSITE_PROFILES); IPR000582 (SUPERFAMILY); IPR029045 (SUPERFAMILY)
28	c102211_g1_i1_len_657_path_635_0_656_4	9	leucine-rich repeat-containing protein 15	219	1.05E-44	61.90%	0.11	PR00019 (PRINTS); IPR003591 (SMART); SM00364 (SMART); G3DSA:3.80.10.10 (GENE3D); IPR001611 (PFAM); IPR001611 (PFAM); PTHR24365 (PANTHER); IPR001611 (PROSITE_PROFILES); IPR001611 (PROSITE_PROFILES); IPR001611 (PROSITE_PROFILES); IPR001611 (PROSITE_PROFILES); IPR001611 (PROSITE_PROFILES); IPR001611 (PROSITE_PROFILES); SSF52058 (SUPERFAMILY)
29	c102279_g1_i1_len_248_path_1_0_247_1	4	collagen alpha chain	83	6.52E-17	71.10%	0.11	IPR008160 (PFAM); PTHR24023 (PANTHER); PTHR24023:SF444 (PANTHER)
30	c102279_g1_i1_len_248_path_1_0_247_5	4	collagen-like protein	82	2.49E-09	50.70%	0.102	no IPS match

31	c102282_g1_i1_len_927_path_1_0_926_3	33	tumor necrosis factor receptor superfamily member 16	309	3.85E-53	50.50%	0.107	IPR001368 (SMART); IPR000488 (SMART); IPR000488 (PFAM); G3DSA:2.10.50.10 (GENE3D); IPR001368 (PFAM); G3DSA:2.10.50.10 (GENE3D); IPR011029 (G3DSA:1.10.533.GENE3D); PTHR23097 (PANTHER); PTHR23097:SF106 (PANTHER); IPR001368 (PROSITE_PATTERNS); IPR001368 (PROSITE_PATTERNS); TRANSMEMBRANE (PHOBIUS); NON_CYTOPLASMIC_DOMAIN (PHOBIUS); CYTOPLASMIC_DOMAIN (PHOBIUS); IPR001368 (PROSITE_PROFILES); IPR000488 (PROSITE_PROFILES); IPR001368 (PROSITE_PROFILES); SSF57586 (SUPERFAMILY); IPR011029 (SUPERFAMILY); TMhelix (TMHMM)
32	c102344_g1_i1_len_324_path_1_0_323_4	8	mam and ldl-receptor class a domain-containing protein 1	108	2.18E-20	62.30%	0.405Y	IPR002172 (G3DSA:4.10.400.GENE3D); IPR000998 (PFAM); IPR002172 (PFAM); PTHR23282 (PANTHER); IPR000998 (PROSITE_PROFILES); IPR002172 (PROSITE_PROFILES); IPR002172 (SUPERFAMILY); IPR013320 (SUPERFAMILY)
33	c102364_g1_i1_len_533_path_1_0_532_2	14	bcl-6 corepressor	177	3.57E-50	69.10%	0.147	IPR002110 (PRINTS); IPR002110 (SMART); IPR020683 (PFAM); IPR020683 (G3DSA:1.25.40.GENE3D); PTHR24117 (PANTHER); IPR002110 (PROSITE_PROFILES); IPR020683 (PROSITE_PROFILES); IPR002110 (PROSITE_PROFILES); IPR020683 (SUPERFAMILY)
34	c102593_g1_i1_len_364_path_342_0_363_3	14	zinc finger protein 569-like isoform x3	122	6.46E-24	59.60%	0.1	IPR015880 (SMART); IPR013087 (G3DSA:3.30.160.GENE3D); IPR013087 (G3DSA:3.30.160.GENE3D); PF13465 (PFAM); IPR013087 (G3DSA:3.30.160.GENE3D); PTHR24409 (PANTHER); IPR007087 (PROSITE_PATTERNS); IPR007087 (PROSITE_PATTERNS); IPR007087 (PROSITE_PATTERNS); IPR007087 (PROSITE_PROFILES); IPR007087 (PROSITE_PROFILES); IPR007087 (PROSITE_PROFILES); SSF57667 (SUPERFAMILY); SSF57667 (SUPERFAMILY)
35	c102640_g1_i1_len_536_path_1_0_535_1	13	thromboxane a synthase-like protein	179	4.02E-51	66.60%	0.12	IPR001128 (PRINTS); IPR002401 (PRINTS); IPR001128 (G3DSA:1.10.630.GENE3D); IPR001128 (PFAM); PTHR24292:SF24 (PANTHER); PTHR24292 (PANTHER); IPR001128 (SUPERFAMILY)
36	c102644_g1_i1_len_308_path_1_0_307_1	4	dorsal-ventral patterning protein sog isoform x2	103	4.59E-15	63.60%	0.148	PTHR11339 (PANTHER); PTHR11339:SF223 (PANTHER); IPR010895 (PROSITE_PROFILES); IPR010895 (PROSITE_PROFILES)
37	c102685_g1_i1_len_641_path_619_0_640_5	10	laminin subunit alpha	213	2.50E-68	66.20%	0.141	no IPS match
38	c102689_g1_i1_len_813_path_1_0_812_1	46	calbindin-32 isoform x2	271	2.43E-97	95.20%	0.106	IPR002048 (SMART); IPR011992 (G3DSA:1.10.238.GENE3D); IPR011992 (PFAM); IPR011992 (G3DSA:1.10.238.GENE3D); PTHR19972 (PANTHER); IPR018247 (PROSITE_PATTERNS); IPR018247 (PROSITE_PATTERNS); IPR018247 (PROSITE_PATTERNS); IPR018247 (PROSITE_PATTERNS); IPR002048 (PROSITE_PROFILES); IPR002048 (PROSITE_PROFILES); IPR002048 (PROSITE_PROFILES); IPR002048 (PROSITE_PROFILES); SSF47473 (SUPERFAMILY)
39	c102837_g1_i1_len_509_path_1_0_508_1	21	reelin-like isoform x4	170	4.31E-58	66.10%	0.114	PTHR11841 (PANTHER)
40	c10284_g1_i1_len_1091_path_1_0_249_251_ 250_273_2174_274_1090_3	21	gastrula zinc finger	364	5.63E-15	63.30%	0.107	IPR015880 (SMART); IPR013087 (G3DSA:3.30.160.GENE3D); IPR013087 (G3DSA:3.30.160.GENE3D); PF13465 (PFAM); PTHR24409 (PANTHER); PTHR24409:SF14 (PANTHER); IPR007087 (PROSITE_PATTERNS); IPR007087 (PROSITE_PATTERNS); IPR007087 (PROSITE_PATTERNS); TRANSMEMBRANE (PHOBIUS); NON_CYTOPLASMIC_DOMAIN (PHOBIUS); CYTOPLASMIC_DOMAIN (PHOBIUS); IPR007087 (PROSITE_PROFILES); IPR007087 (PROSITE_PROFILES); IPR007087 (PROSITE_PROFILES); SSF57667 (SUPERFAMILY); SSF57667 (SUPERFAMILY)
41	c102901_g1_i1_len_293_path_271_0_292_4	10	furin-like protease isoforms 1 1-x 2-like	98	5.46E-50	92.30%	0.288	IPR000209 (G3DSA:3.40.50.GENE3D); IPR000209 (PFAM); IPR015500 (PANTHER); PTHR10795:SF71 (PANTHER); IPR023828 (PROSITE_PATTERNS); IPR000209 (SUPERFAMILY)
42	c103281_g1_i1_len_574_path_1_0_573_3	14	zinc finger protein ozf-like	192	1.02E-32	56.30%	0.099	IPR015880 (SMART); IPR007087 (PFAM); IPR013087 (G3DSA:3.30.160.GENE3D); PF13465 (PFAM); IPR013087 (G3DSA:3.30.160.GENE3D); IPR013087 (G3DSA:3.30.160.GENE3D); IPR013087 (G3DSA:3.30.160.GENE3D); IPR013087 (G3DSA:3.30.160.GENE3D); PTHR24409 (PANTHER); PTHR24409:SF14 (PANTHER); IPR007087 (PROSITE_PATTERNS); IPR007087 (PROSITE_PATTERNS); IPR007087 (PROSITE_PATTERNS); IPR007087 (PROSITE_PROFILES); IPR007087 (PROSITE_PROFILES); IPR007087 (PROSITE_PROFILES); IPR007087 (PROSITE_PROFILES); IPR007087 (PROSITE_PROFILES); IPR007087 (PROSITE_PROFILES); SSF57667 (SUPERFAMILY); SSF57667 (SUPERFAMILY); SSF57667 (SUPERFAMILY); SSF57667 (SUPERFAMILY)

43	c103473_g1_i1_len_339_path_1_0_338_3	4	GH18519	113	5.38E-32	73.50%	0.145	PR00019 (PRINTS); IPR003591 (SMART); G3DSA:3.80.10.10 (GENE3D); IPR001611 (PFAM); IPR001611 (PFAM); PTHR24365 (PANTHER); IPR001611 (PROSITE_PROFILES); IPR001611 (PROSITE_PROFILES); IPR001611 (PROSITE_PROFILES); IPR001611 (PROSITE_PROFILES); SSF52058 (SUPERFAMILY)
44	c103517_g1_i1_len_768_path_1_0_767_2	23	protein lin-7 homolog b	256	1.36E-60	96.00%	0.098	IPR001478 (SMART); IPR001478 (G3DSA:2.30.42.GENE3D); IPR001478 (PFAM); PTHR14063 (PANTHER); TRANSMEMBRANE (PHOBIUS); NON_CYTOPLASMIC_DOMAIN (PHOBIUS); TRANSMEMBRANE (PHOBIUS); CYTOPLASMIC_DOMAIN (PHOBIUS); TRANSMEMBRANE (PHOBIUS); NON_CYTOPLASMIC_DOMAIN (PHOBIUS); CYTOPLASMIC_DOMAIN (PHOBIUS); IPR001478 (PROSITE_PROFILES); IPR001478 (SUPERFAMILY); TMhelix (TMHMM); TMhelix (TMHMM); TMhelix (TMHMM)
45	c103519_g1_i1_len_340_path_1_0_339_3	8	transferrin	114	2.97E-22	62.50%	0.1	IPR001156 (PRINTS); G3DSA:3.40.190.10 (GENE3D); IPR001156 (PFAM); G3DSA:3.40.190.10 (GENE3D); PTHR11485:SF18 (PANTHER); PTHR11485 (PANTHER); IPR001156 (PROSITE_PROFILES); SSF53850 (SUPERFAMILY)
46	c103717_g1_i1_len_342_path_320_0_341_4	9	proclotting enzyme	114	2.84E-47	77.50%	0.147	IPR001254 (SMART); G3DSA:2.40.10.10 (GENE3D); G3DSA:2.40.10.10 (GENE3D); IPR001254 (PFAM); PTHR24268 (PANTHER); IPR018114 (PROSITE_PATTERNS); IPR001254 (PROSITE_PROFILES); IPR009003 (SUPERFAMILY)
47	c103859_g1_i1_len_298_path_276_0_297_1	3	zinc finger protein 235	99	8.82E-18	59.40%	0.115	IPR015880 (SMART); IPR013087 (G3DSA:3.30.160.GENE3D); IPR013087 (G3DSA:3.30.160.GENE3D); PF13465 (PFAM); PTHR24409 (PANTHER); IPR007087 (PROSITE_PATTERNS); IPR007087 (PROSITE_PATTERNS); IPR007087 (PROSITE_PATTERNS); IPR007087 (PROSITE_PROFILES); IPR007087 (PROSITE_PROFILES); IPR007087 (PROSITE_PROFILES); SSF57667 (SUPERFAMILY); SSF57667 (SUPERFAMILY)
48	c103866_g1_i1_len_619_path_1_0_618_0	15	leucine-rich repeat-containing protein 70 isoform x1	207	8.92E-22	54.50%	0.115	IPR003591 (SMART); G3DSA:3.80.10.10 (GENE3D); G3DSA:3.80.10.10 (GENE3D); IPR001611 (PFAM); PTHR24367 (PANTHER); IPR001611 (PROSITE_PROFILES); IPR001611 (PROSITE_PROFILES); IPR001611 (PROSITE_PROFILES); IPR001611 (PROSITE_PROFILES); IPR001611 (PROSITE_PROFILES); SSF52058 (SUPERFAMILY)
49	c103881_g1_i1_len_481_path_459_0_480_1	15	dnaj homolog subfamily c member 25 homolog	160	8.98E-68	82.50%	0.105	Coil (COILS); Coil (COILS); IPR001623 (PRINTS); IPR001623 (SMART); IPR001623 (PFAM); IPR001623 (G3DSA:1.10.287.GENE3D); PTHR24078:SF125 (PANTHER); PTHR24078 (PANTHER); IPR018253 (PROSITE_PATTERNS); TRANSMEMBRANE (PHOBIUS); CYTOPLASMIC_DOMAIN (PHOBIUS); NON_CYTOPLASMIC_DOMAIN (PHOBIUS); IPR001623 (PROSITE_PROFILES); IPR001623 (SUPERFAMILY); TMhelix (TMHMM)
50	c103918_g1_i1_len_399_path_1_0_398_0	11	btd_takru ame: full=biotinidase short=biotinase flags: precursor	133	1.36E-13	56.70%	0.113	IPR003010 (G3DSA:3.60.110.GENE3D); IPR003010 (PFAM); IPR012101 (PANTHER); IPR003010 (PROSITE_PROFILES); IPR003010 (SUPERFAMILY)
51	c104026_g1_i1_len_336_path_1_0_335_3	8	chemosensory protein 1 partial	112	1.86E-38	79.20%	0.829Y	IPR005055 (G3DSA:1.10.2080.GENE3D); IPR005055 (PFAM); IPR005055 (PANTHER); PTHR11257:SF4 (PANTHER); SIGNAL_PEPTIDE_C_REGION (PHOBIUS); SIGNAL_PEPTIDE_H_REGION (PHOBIUS); SIGNAL_PEPTIDE_N_REGION (PHOBIUS); NON_CYTOPLASMIC_DOMAIN (PHOBIUS); SIGNAL_PEPTIDE (PHOBIUS); SignalP-noTM (SIGNALP_EUK); IPR005055 (SUPERFAMILY); TMhelix (TMHMM)
52	c104120_g1_i1_len_404_path_382_0_403_1	6	glucosylceramidase- partial	135	1.58E-52	72.40%	0.1	IPR001139 (PRINTS); IPR013781 (G3DSA:3.20.20.GENE3D); IPR001139 (PFAM); PTHR11069:SF7 (PANTHER); IPR001139 (PANTHER); IPR017853 (SUPERFAMILY)
53	c104363_g1_i1_len_344_path_1_0_343_2	4	vascular endothelial growth factor receptor partial	114	2.08E-19	57.70%	0.115	IPR013783 (G3DSA:2.60.40.GENE3D); PTHR15360 (PANTHER); SSF48726 (SUPERFAMILY)
54	c104536_g1_i1_len_560_path_4_0_559_3	12	zinc finger protein 271 (zinc finger protein 7) (zinc finger protein znfphex133) (epstein-barr virus-induced zinc finger protein) (znf-eb) (ct-zfp48) (zinc finger protein	187	2.02E-24	60.90%	0.112	IPR015880 (SMART); PF13894 (PFAM); IPR013087 (G3DSA:3.30.160.GENE3D); PF13465 (PFAM); IPR013087 (G3DSA:3.30.160.GENE3D); PTHR24409 (PANTHER); IPR007087 (PROSITE_PATTERNS); IPR007087 (PROSITE_PATTERNS); IPR007087 (PROSITE_PATTERNS); IPR007087 (PROSITE_PATTERNS); IPR007087 (PROSITE_PROFILES); IPR007087 (PROSITE_PROFILES); IPR007087 (PROSITE_PROFILES); IPR007087 (PROSITE_PROFILES); SSF57667 (SUPERFAMILY); SSF57667 (SUPERFAMILY)
55	c104577_g1_i1_len_308_path_1_0_307_1	5	protein kinase c-binding protein partial	103	1.78E-30	72.70%	0.11	Coil (COILS); PTHR24042:SF4 (PANTHER); PTHR24042 (PANTHER)
56	c104636_g1_i1_len_619_path_597_0_618_0	22	third variable lymphocyte receptor	207	3.29E-11	51.60%	0.125	IPR001611 (PFAM); G3DSA:3.80.10.10 (GENE3D); PTHR24365:SF241 (PANTHER); PTHR24365 (PANTHER); SSF52058 (SUPERFAMILY)

57	c104743_g1_i1_len_612_path_590_0_611_2	25	zinc finger protein 628 isoform x1	204	6.51E-16	56.10%	0.106	Coil (COILS); IPR015880 (SMART); PF13465 (PFAM); IPR013087 (G3DSA:3.30.160.GENE3D); IPR013087 (G3DSA:3.30.160.GENE3D); PTHR24409:SF14 (PANTHER); PTHR24409 (PANTHER); IPR007087 (PROSITE_PATTERNS); IPR007087 (PROSITE_PATTERNS); IPR007087 (PROSITE_PROFILES); IPR007087 (PROSITE_PROFILES); IPR007087 (PROSITE_PROFILES); SSF57667 (SUPERFAMILY); SSF57667 (SUPERFAMILY)
58	c104990_g1_i1_len_363_path_341_0_362_0	7	wd repeat-containing protein 47 isoform x5	121	6.42E-75	94.30%	0.13	IPR001680 (SMART); IPR001680 (PFAM); IPR015943 (G3DSA:2.130.10.GENE3D); PTHR19863 (PANTHER); IPR017986 (PROSITE_PROFILES); IPR001680 (PROSITE_PROFILES); IPR001680 (PROSITE_PROFILES); IPR017986 (SUPERFAMILY)
59	c105120_g1_i1_len_347_path_1_0_346_5	7	serine threonine-protein kinase osr1 isoform x1	115	1.05E-67	94.60%	0.199	IPR000719 (PFAM); G3DSA:3.30.200.20 (GENE3D); G3DSA:1.10.510.10 (GENE3D); PTHR24361:SF201 (PANTHER); PTHR24361 (PANTHER); TRANSMEMBRANE (PHOBIUS); NON_CYTOPLASMIC_DOMAIN (PHOBIUS); CYTOPLASMIC_DOMAIN (PHOBIUS); IPR000719 (PROSITE_PROFILES); IPR011009 (SUPERFAMILY)
60	c105183_g1_i1_len_948_path_907_0_884_1792_885_905_1792_906_926_1792_927_947_0	49	ves g 5 allergen	316	7.46E-49	53.00%	0.382Y	IPR001283 (PRINTS); IPR002413 (PRINTS); IPR014044 (SMART); IPR014044 (G3DSA:3.40.33.GENE3D); IPR014044 (PFAM); IPR001283 (PANTHER); CYTOPLASMIC_DOMAIN (PHOBIUS); TRANSMEMBRANE (PHOBIUS); NON_CYTOPLASMIC_DOMAIN (PHOBIUS); IPR014044 (SUPERFAMILY); TMhelix (TMHMM)
62	c10534_g1_i1_len_346_path_324_0_345_1	1	zinc finger protein 709-like	115	1.57E-21	63.20%	0.109	IPR015880 (SMART); IPR007087 (PFAM); IPR013087 (G3DSA:3.30.160.GENE3D); PF13465 (PFAM); IPR013087 (G3DSA:3.30.160.GENE3D); PTHR24409 (PANTHER); PTHR24409:SF14 (PANTHER); IPR007087 (PROSITE_PATTERNS); IPR007087 (PROSITE_PATTERNS); IPR007087 (PROSITE_PATTERNS); IPR007087 (PROSITE_PROFILES); IPR007087 (PROSITE_PROFILES); IPR007087 (PROSITE_PROFILES); SSF57667 (SUPERFAMILY); SSF57667 (SUPERFAMILY)
63	c10534_g2_i1_len_499_path_1123_0_498_2	6	zinc finger protein 850-like isoform x1	166	1.11E-23	55.10%	0.116	IPR015880 (SMART); IPR007087 (PFAM); IPR013087 (G3DSA:3.30.160.GENE3D); IPR013087 (G3DSA:3.30.160.GENE3D); PF13894 (PFAM); PF13912 (PFAM); IPR013087 (G3DSA:3.30.160.GENE3D); PF13465 (PFAM); PTHR24409 (PANTHER); PTHR24409:SF13 (PANTHER); IPR007087 (PROSITE_PATTERNS); IPR007087 (PROSITE_PATTERNS); IPR007087 (PROSITE_PATTERNS); IPR007087 (PROSITE_PROFILES); IPR007087 (PROSITE_PROFILES); IPR007087 (PROSITE_PROFILES); IPR007087 (PROSITE_PROFILES); SSF57667 (SUPERFAMILY); SSF57667 (SUPERFAMILY)
61	c105340_g1_i1_len_407_path_1_0_406_1	6	upf0454 protein c12orf49 homolog	136	6.78E-34	87.60%	0.132	IPR019352 (PFAM); IPR019352 (PANTHER)
64	c105558_g1_i1_len_279_path_1_0_278_4	4	mam and ldl-receptor class a domain-containing protein 2-like	93	1.96E-10	52.90%	0.103	IPR000998 (PFAM); PTHR23282:SF75 (PANTHER); PTHR23282 (PANTHER); IPR000998 (PROSITE_PROFILES); IPR013320 (SUPERFAMILY)
65	c105706_g1_i1_len_327_path_305_0_326_5	4	metallophosphoesterase domain-containing protein 1	109	5.53E-57	92.20%	0.134	IPR004843 (PFAM); IPR029052 (G3DSA:3.60.21.GENE3D); PTHR12905 (PANTHER); IPR029052 (SUPERFAMILY)
66	c105858_g1_i1_len_413_path_1_0_412_1	7	non-structural protein ns5	138	5.38E-07	52.43%	0.101	IPR029063 (G3DSA:3.40.50.GENE3D)
67	c105886_g1_i1_len_317_path_295_0_316_2	6	cysteine-rich motor neuron 1 protein	105	9.73E-26	59.40%	0.11	G3DSA:2.10.70.10 (GENE3D); SSF57603 (SUPERFAMILY)
68	c10607_g1_i1_len_240_path_218_0_239_2	3	protein fam20c	80	1.01E-44	90.70%	0.131	IPR009581 (PFAM); PTHR12450:SF8 (PANTHER); IPR024869 (PANTHER)
69	c10607_g2_i1_len_179_path_435_0_178_4	2	extracellular serine threonine protein kinase fam20c-like	60	5.64E-26	94.20%	0.098	IPR029207 (PFAM); IPR024869 (PANTHER); PTHR12450:SF8 (PANTHER)
70	c106203_g1_i1_len_378_path_356_0_377_2	6	cyclophilin a	126	7.74E-64	91.90%	0.205	IPR002130 (PRINTS); IPR002130 (PFAM); IPR024936 (PIRSF); IPR029000 (G3DSA:2.40.100.GENE3D); IPR024936 (PANTHER); IPR020892 (PROSITE_PATTERNS); IPR002130 (PROSITE_PROFILES); IPR029000 (SUPERFAMILY)
71	c106240_g1_i1_len_655_path_1_0_654_5	19	ryanodine receptor 44f	218	4.07E-102	83.20%	0.105	IPR003032 (PFAM); IPR003877 (PFAM); IPR015925 (PANTHER); PTHR13715:SF74 (PANTHER); IPR001870 (PROSITE_PROFILES); IPR013320 (SUPERFAMILY)
72	c106268_g1_i1_len_465_path_1_0_464_1	14	chymotrypsin 1	155	1.34E-14	60.20%	0.203	G3DSA:2.40.10.10 (GENE3D); G3DSA:2.40.10.10 (GENE3D); IPR001254 (PFAM); PTHR24276 (PANTHER); IPR018114 (PROSITE_PATTERNS); IPR001254 (PROSITE_PROFILES); IPR009003 (SUPERFAMILY)
73	c106414_g1_i1_len_626_path_604_0_625_3	10	receptor-type tyrosine-protein phosphatase kappa	209	1.96E-120	89.90%	0.103	IPR000242 (SMART); IPR029021 (G3DSA:3.90.190.GENE3D); IPR000242 (PFAM); PTHR19134:SF227 (PANTHER); PTHR19134 (PANTHER); IPR000242 (PROSITE_PROFILES); IPR029021 (SUPERFAMILY)
74	c106462_g1_i1_len_421_path_1_0_420_4	5	glutaminyl-peptide cyclotransferase-like isoform x2	140	4.79E-16	58.50%	0.192	G3DSA:3.40.630.10 (GENE3D); PTHR12283 (PANTHER); CYTOPLASMIC_DOMAIN (PHOBIUS); TRANSMEMBRANE (PHOBIUS); NON_CYTOPLASMIC_DOMAIN (PHOBIUS); SSF53187 (SUPERFAMILY); TMhelix (TMHMM); TMhelix (TMHMM)

75	c10675_g1_i1_len_632_path_610_0_631_2	25	cytochrome p450 3a31	210	9.34E-51	70.00%	0.151	IPR002401 (PRINTS); IPR001128 (PRINTS); IPR001128 (PFAM); IPR001128 (G3DSA:1.10.630.GENE3D); PTHR24292:SF24 (PANTHER); PTHR24292 (PANTHER); IPR001128 (SUPERFAMILY)
76	c106831_g1_i1_len_472_path_450_0_471_0	8	lipoprotein receptor	158	1.34E-21	54.00%	0.113	IPR000203 (SMART); IPR000203 (PFAM); PTHR12011:SF260 (PANTHER); PTHR12011 (PANTHER); IPR000203 (PROSITE_PROFILES)
77	c107555_g1_i1_len_203_path_1_0_202_0	0	carboxypeptidase b-like	68	6.90E-18	69.30%	0.343Y	IPR000834 (PFAM); G3DSA:3.40.630.10 (GENE3D); PTHR11705 (PANTHER); SSF53187 (SUPERFAMILY)
78	c107967_g1_i1_len_278_path_256_0_277_5	4	basement membrane-specific heparan sulfate proteoglycan core protein	92	5.21E-35	78.70%	0.118	IPR002172 (PRINTS); IPR002172 (SMART); IPR002172 (G3DSA:4.10.400.GENE3D); IPR002172 (G3DSA:4.10.400.GENE3D); IPR002172 (PFAM); PTHR10529 (PANTHER); IPR023415 (PROSITE_PATTERNS); IPR023415 (PROSITE_PATTERNS); IPR002172 (PROSITE_PROFILES); IPR002172 (PROSITE_PROFILES); IPR002172 (SUPERFAMILY); IPR002172 (SUPERFAMILY)
79	c108345_g1_i1_len_370_path_1_0_369_1	5	lipoprotein receptor	123	4.94E-08	42.33%	0.118	IPR002172 (G3DSA:4.10.400.GENE3D); IPR002172 (PROSITE_PROFILES); IPR002172 (SUPERFAMILY); IPR002172 (SUPERFAMILY)
80	c10860_g1_i1_len_771_path_749_0_770_1	53	zinc finger protein 234-like isoform x2	257	2.23E-31	54.60%	0.101	IPR015880 (SMART); IPR013087 (G3DSA:3.30.160.GENE3D); PF13465 (PFAM); IPR013087 (G3DSA:3.30.160.GENE3D); PTHR24409:SF13 (PANTHER); PTHR24409 (PANTHER); IPR007087 (PROSITE_PATTERNS); IPR007087 (PROSITE_PATTERNS); IPR007087 (PROSITE_PATTERNS); IPR007087 (PROSITE_PROFILES); IPR007087 (PROSITE_PROFILES); IPR007087 (PROSITE_PROFILES); SSF57667 (SUPERFAMILY); SSF57667 (SUPERFAMILY); SSF57667 (SUPERFAMILY)
81	c108721_g1_i1_len_290_path_268_0_289_0	4	neural-cadherin isoform x10	97	3.48E-43	83.40%	0.101	IPR013320 (G3DSA:2.60.120.GENE3D); IPR001791 (PFAM); PTHR24027 (PANTHER); PTHR24027:SF285 (PANTHER); IPR013032 (PROSITE_PATTERNS); IPR013320 (SUPERFAMILY)
82	c108786_g1_i1_len_258_path_1_0_257_4	4	thiol peroxiredoxin	86	1.87E-44	97.60%	0.101	IPR012336 (G3DSA:3.40.30.GENE3D); G3DSA:3.30.1020.10 (GENE3D); IPR019479 (PFAM); PTHR10681:SF67 (PANTHER); PTHR10681 (PANTHER); IPR012336 (SUPERFAMILY)
83	c108852_g1_i1_len_694_path_1_0_693_4	17	protein fam3c-like	231	5.92E-59	62.70%	0.455 Y	PTHR14592:SF7 (PANTHER); IPR026101 (PANTHER); SignalP-noTM (SIGNALP_EUK)
84	c108888_g1_i1_len_458_path_436_0_457_0	7	follistatin-related protein 5-like	153	1.06E-83	86.20%	0.103	IPR013783 (G3DSA:2.60.40.GENE3D); IPR011992 (G3DSA:1.10.238.GENE3D); IPR013098 (PFAM); IPR011992 (PFAM); PTHR10913:SF14 (PANTHER); PTHR10913 (PANTHER); IPR018247 (PROSITE_PATTERNS); IPR018247 (PROSITE_PATTERNS); IPR002048 (PROSITE_PROFILES); IPR002048 (PROSITE_PROFILES); SSF48726 (SUPERFAMILY); SSF47473 (SUPERFAMILY)
85	c10894_g1_i1_len_915_path_1_0_914_5	56	estradiol 17-beta-dehydrogenase 12	305	8.87E-56	67.40%	0.097	IPR002347 (PRINTS); IPR002198 (PRINTS); IPR002198 (PFAM); IPR016040 (G3DSA:3.40.50.GENE3D); PTHR24316:SF68 (PANTHER); PTHR24316 (PANTHER); CYTOPLASMIC_DOMAIN (PHOBIUS); TRANSMEMBRANE (PHOBIUS); CYTOPLASMIC_DOMAIN (PHOBIUS); NON_CYTOPLASMIC_DOMAIN (PHOBIUS); TRANSMEMBRANE (PHOBIUS); SSF51735 (SUPERFAMILY); TMhelix (TMHMM); TMhelix (TMHMM)
86	c108980_g1_i1_len_422_path_1_0_421_1	6	protein turtle-like isoform x1	141	1.11E-20	54.80%	0.1	IPR003599 (SMART); IPR003598 (SMART); IPR013783 (G3DSA:2.60.40.GENE3D); IPR013098 (PFAM); PTHR10489 (PANTHER); IPR007110 (PROSITE_PROFILES); SSF48726 (SUPERFAMILY)
87	c109017_g1_i1_len_679_path_657_0_678_2	10	epithelial chloride channel	226	1.84E-68	60.80%	0.821 Y	IPR013642 (PFAM); PTHR10579 (PANTHER); PTHR10579:SF8 (PANTHER); SIGNAL_PEPTIDE_H_REGION (PHOBIUS); SIGNAL_PEPTIDE_C_REGION (PHOBIUS); NON_CYTOPLASMIC_DOMAIN (PHOBIUS); SIGNAL_PEPTIDE (PHOBIUS); SIGNAL_PEPTIDE_N_REGION (PHOBIUS); SignalP-noTM (SIGNALP_EUK)
88	c109030_g1_i1_len_797_path_775_0_796_1	16	zinc finger protein 57-like	266	3.98E-115	76.50%	0.102	IPR015880 (SMART); PF13894 (PFAM); IPR013087 (G3DSA:3.30.160.GENE3D); PF13465 (PFAM); IPR013087 (G3DSA:3.30.160.GENE3D); IPR013087 (G3DSA:3.30.160.GENE3D); PTHR24409 (PANTHER); IPR007087 (PROSITE_PATTERNS); IPR007087 (PROSITE_PATTERNS); IPR007087 (PROSITE_PATTERNS); IPR007087 (PROSITE_PATTERNS); IPR007087 (PROSITE_PROFILES); IPR007087 (PROSITE_PROFILES); IPR007087 (PROSITE_PROFILES); IPR007087 (PROSITE_PROFILES); IPR007087 (PROSITE_PROFILES); IPR007087 (PROSITE_PROFILES); SSF57667 (SUPERFAMILY); SSF57667 (SUPERFAMILY); SSF57667 (SUPERFAMILY); SSF57667 (SUPERFAMILY); SSF57667 (SUPERFAMILY)
89	c109113_g1_i1_len_267_path_1_0_266_5	7	aldehyde mitochondrial precursor	89	9.55E-40	86.00%	0.1	IPR016163 (G3DSA:3.40.309.GENE3D); IPR015590 (PFAM); PTHR11699 (PANTHER); IPR015657 (PTHR11699:PANTHER); IPR016161 (SUPERFAMILY)

90	c109136_g1_i1_len_347_path_1_0_346_4	8	zinc finger protein 37-like	116	5.24E-19	58.40%	0.102	IPR015880 (SMART); PF13465 (PFAM); IPR013087 (G3DSA:3.30.160.GENE3D); PF13894 (PFAM); IPR007087 (PFAM); IPR013087 (G3DSA:3.30.160.GENE3D); IPR013087 (G3DSA:3.30.160.GENE3D); PTHR24409:SF14 (PANTHER); PTHR24409 (PANTHER); IPR007087 (PROSITE_PATTERNS); IPR007087 (PROSITE_PATTERNS); IPR007087 (PROSITE_PATTERNS); IPR007087 (PROSITE_PATTERNS); IPR007087 (PROSITE_PROFILES); IPR007087 (PROSITE_PROFILES); IPR007087 (PROSITE_PROFILES); IPR007087 (PROSITE_PROFILES); SSF57667 (SUPERFAMILY); SSF57667 (SUPERFAMILY)
91	c109170_g1_i1_len_142_path_1_0_141_3	1	peptidylglycine alpha-hydroxylating monooxygenase	48	6.90E-14	76.90%	0.119	IPR014784 (G3DSA:2.60.120.GENE3D); PF03712 (PFAM); PTHR10680 (PANTHER); IPR008977 (SUPERFAMILY)
92	c109283_g1_i1_len_365_path_343_0_364_5	6	mam receptor domain-containing partial	121	5.10E-24	56.90%	0.107	IPR000998 (PFAM); G3DSA:4.10.1220.10 (GENE3D); PTHR23282:SF75 (PANTHER); PTHR23282 (PANTHER); IPR000998 (PROSITE_PROFILES); IPR013320 (SUPERFAMILY)
93	c10953_g1_i1_len_643_path_1_0_642_4	24	reelin- partial	214	6.75E-54	57.90%	0.255	PTHR11841 (PANTHER)
94	c10953_g2_i1_len_1143_path_1241_0_1142_5	44	reelin-like isoform x1	381	3.32E-82	54.10%	0.132	G3DSA:2.10.25.10 (GENE3D); PTHR11841 (PANTHER); IPR013032 (PROSITE_PATTERNS); IPR013032 (PROSITE_PATTERNS)
95	c109571_g1_i1_len_253_path_1_0_252_1	4	---NA---	84			0.481 Y	CYTOPLASMIC_DOMAIN (PHOBIUS); TRANSMEMBRANE (PHOBIUS); NON_CYTOPLASMIC_DOMAIN (PHOBIUS); TMhelix (TMHMM)
96	c10972_g2_i1_len_457_path_435_0_456_1	11	hemicentin- partial	152	7.08E-17	51.60%	0.107	IPR013783 (G3DSA:2.60.40.GENE3D); PF13895 (PFAM); IPR013783 (G3DSA:2.60.40.GENE3D); PTHR23279 (PANTHER); IPR007110 (PROSITE_PROFILES); SSF48726 (SUPERFAMILY); SSF48726 (SUPERFAMILY)
97	c109790_g1_i1_len_221_path_199_0_220_5	1	histone-lysine n-methyltransferase prdm9-like	73	3.80E-15	62.20%	0.108	IPR015880 (SMART); PF13894 (PFAM); IPR013087 (G3DSA:3.30.160.GENE3D); IPR013087 (G3DSA:3.30.160.GENE3D); PF13465 (PFAM); PTHR24409:SF14 (PANTHER); PTHR24409 (PANTHER); IPR007087 (PROSITE_PATTERNS); IPR007087 (PROSITE_PATTERNS); IPR007087 (PROSITE_PROFILES); IPR007087 (PROSITE_PROFILES); SSF57667 (SUPERFAMILY); SSF57667 (SUPERFAMILY)
98	c110322_g1_i1_len_336_path_314_0_335_3	4	latrophilin and seven transmembrane domain-containing protein 1	112	4.12E-08	55.60%	0.287	IPR000832 (PFAM); PTHR12011 (PANTHER); PTHR12011:SF61 (PANTHER); TRANSMEMBRANE (PHOBIUS); TRANSMEMBRANE (PHOBIUS); CYTOPLASMIC_DOMAIN (PHOBIUS); CYTOPLASMIC_DOMAIN (PHOBIUS); NON_CYTOPLASMIC_DOMAIN (PHOBIUS); IPR017981 (PROSITE_PROFILES); TMhelix (TMHMM); TMhelix (TMHMM)
99	c110452_g1_i1_len_476_path_267_0_55_323_56_265_323_266_475_0	2	repetitive proline-rich cell wall protein 2-like isoform x2	159	7.74E-19	53.70%	0.143	no IPS match
100	c11060_g2_i1_len_637_path_1383_0_636_4	19	homeotic protein spalt-major-like	212	5.58E-97	88.40%	0.108	Coil (COILS); IPR015880 (SMART); IPR013087 (G3DSA:3.30.160.GENE3D); IPR013087 (G3DSA:3.30.160.GENE3D); PF13465 (PFAM); IPR013087 (G3DSA:3.30.160.GENE3D); PF13894 (PFAM); PTHR23233:SF49 (PANTHER); PTHR23233 (PANTHER); IPR007087 (PROSITE_PATTERNS); IPR007087 (PROSITE_PATTERNS); IPR007087 (PROSITE_PATTERNS); IPR007087 (PROSITE_PROFILES); IPR007087 (PROSITE_PROFILES); IPR007087 (PROSITE_PROFILES); SSF57667 (SUPERFAMILY); SSF57667 (SUPERFAMILY)
101	c110796_g1_i1_len_292_path_1_0_291_4	4	tetratricopeptide-like helical domain-containing protein	97	1.57E-16	74.50%	0.157	IPR002130 (PRINTS); IPR002130 (PFAM); IPR029000 (G3DSA:2.40.100.GENE3D); IPR024936 (PANTHER); IPR002130 (PROSITE_PROFILES); IPR029000 (SUPERFAMILY)
102	c11088_g1_i1_len_445_path_1_0_444_1	6	endothelin-converting enzyme 1-like	148	5.86E-47	60.10%	0.106	IPR008753 (PFAM); G3DSA:1.10.1380.10 (GENE3D); IPR000718 (PANTHER); PTHR11733:SF124 (PANTHER); SSF55486 (SUPERFAMILY)
103	c111081_g1_i1_len_477_path_455_0_476_1	7	down syndrome cell adhesion molecule-like protein dscam2	159	1.97E-81	89.60%	0.108	IPR003599 (SMART); IPR003598 (SMART); IPR013783 (G3DSA:2.60.40.GENE3D); IPR013783 (G3DSA:2.60.40.GENE3D); IPR013098 (PFAM); PTHR10489 (PANTHER); PTHR10489:SF567 (PANTHER); IPR007110 (PROSITE_PROFILES); IPR007110 (PROSITE_PROFILES); SSF48726 (SUPERFAMILY); SSF48726 (SUPERFAMILY)
104	c111275_g1_i1_len_332_path_1_0_331_4	4	cul o 2 allergen	111	6.56E-21	58.40%	0.105	IPR018155 (PFAM); IPR013785 (G3DSA:3.20.20.GENE3D); IPR018155 (PANTHER); PTHR11769:SF8 (PANTHER); IPR017853 (SUPERFAMILY)
105	c111295_g1_i1_len_267_path_245_0_266_3	5	zinc finger protein 431 isoform x2	89	1.23E-18	67.20%	0.109	IPR015880 (SMART); PF13465 (PFAM); IPR013087 (G3DSA:3.30.160.GENE3D); IPR013087 (G3DSA:3.30.160.GENE3D); PTHR24409 (PANTHER); IPR007087 (PROSITE_PATTERNS); IPR007087 (PROSITE_PATTERNS); IPR007087 (PROSITE_PATTERNS); IPR007087 (PROSITE_PROFILES); IPR007087 (PROSITE_PROFILES); IPR007087 (PROSITE_PROFILES); SSF57667 (SUPERFAMILY); SSF57667 (SUPERFAMILY)

106	c111625_g1_i1_len_385_path_1_0_384_3	8	zinc finger partial	129	9.95E-20	56.20%	0.101	IPR015880 (SMART); IPR013087 (G3DSA:3.30.160.GENE3D); IPR013087 (G3DSA:3.30.160.GENE3D); IPR007087 (PFAM); PF13465 (PFAM); PTHR24409 (PANTHER); PTHR24409:SF14 (PANTHER); IPR007087 (PROSITE_PATTERNS); IPR007087 (PROSITE_PATTERNS); IPR007087 (PROSITE_PATTERNS); IPR007087 (PROSITE_PROFILES); IPR007087 (PROSITE_PROFILES); IPR007087 (PROSITE_PROFILES); IPR007087 (PROSITE_PROFILES); SSF57667 (SUPERFAMILY); SSF57667 (SUPERFAMILY)
107	c11163_g2_i1_len_486_path_1042_0_485_3	12	leucine-rich repeat-containing protein 15-like	162	8.65E-39	65.70%	0.1	PR00019 (PRINTS); IPR003591 (SMART); SM00365 (SMART); IPR001611 (PFAM); PF13516 (PFAM); IPR001611 (PFAM); G3DSA:3.80.10.10 (GENE3D); PTHR24365 (PANTHER); IPR001611 (PROSITE_PROFILES); IPR001611 (PROSITE_PROFILES); IPR001611 (PROSITE_PROFILES); IPR001611 (PROSITE_PROFILES); SSF52058 (SUPERFAMILY)
108	c111716_g1_i1_len_523_path_501_0_522_4	8	probable isocitrate dehydrogenase	174	1.57E-103	93.40%	0.102	IPR024084 (PFAM); IPR024084 (G3DSA:3.40.718.GENE3D); PTHR11835:SF34 (PANTHER); IPR001804 (PANTHER); SSF53659 (SUPERFAMILY)
109	c112031_g1_i1_len_316_path_26_0_315_3	7	pr domain zinc finger protein 1 isoform x2	106	1.61E-20	65.20%	0.099	IPR015880 (SMART); PF13465 (PFAM); IPR013087 (G3DSA:3.30.160.GENE3D); IPR007087 (PFAM); PTHR24409 (PANTHER); IPR007087 (PROSITE_PATTERNS); IPR007087 (PROSITE_PATTERNS); IPR007087 (PROSITE_PATTERNS); IPR007087 (PROSITE_PROFILES); IPR007087 (PROSITE_PROFILES); IPR007087 (PROSITE_PROFILES); SSF57667 (SUPERFAMILY); SSF57667 (SUPERFAMILY)
110	c112136_g1_i1_len_341_path_1_0_340_1	7	transcription factor e4f1-like	114	1.10E-30	68.00%	0.107	IPR015880 (SMART); IPR013087 (G3DSA:3.30.160.GENE3D); IPR013087 (G3DSA:3.30.160.GENE3D); PF13894 (PFAM); PF13912 (PFAM); PF13465 (PFAM); IPR007087 (PFAM); IPR013087 (G3DSA:3.30.160.GENE3D); IPR013087 (G3DSA:3.30.160.GENE3D); PTHR24409 (PANTHER); IPR007087 (PROSITE_PATTERNS); IPR007087 (PROSITE_PATTERNS); IPR007087 (PROSITE_PATTERNS); IPR007087 (PROSITE_PATTERNS); IPR007087 (PROSITE_PROFILES); IPR007087 (PROSITE_PROFILES); IPR007087 (PROSITE_PROFILES); IPR007087 (PROSITE_PROFILES); SSF57667 (SUPERFAMILY); SSF57667 (SUPERFAMILY)
111	c112223_g1_i1_len_158_path_1_0_157_1	1	paralytic	53	2.09E-23	98.30%	0.288	Coil (COILS); IPR005821 (PFAM); IPR028814 (PTHR10037:PANTHER); PTHR10037 (PANTHER); CYTOPLASMIC_DOMAIN (PHOBIUS); TRANSMEMBRANE (PHOBIUS); NON_CYTOPLASMIC_DOMAIN (PHOBIUS); TMhelix (TMHMM)
112	c112669_g1_i1_len_283_path_261_0_282_3	13	krueppel-like factor 13	95	7.12E-46	85.20%	0.113	IPR015880 (SMART); IPR013087 (G3DSA:3.30.160.GENE3D); IPR013087 (G3DSA:3.30.160.GENE3D); PF13465 (PFAM); PTHR23223 (PANTHER); IPR007087 (PROSITE_PATTERNS); IPR007087 (PROSITE_PATTERNS); IPR007087 (PROSITE_PROFILES); IPR007087 (PROSITE_PROFILES); IPR007087 (PROSITE_PROFILES); SSF57667 (SUPERFAMILY)
113	c112937_g1_i1_len_259_path_1_0_258_2	4	fibrillin- partial	86	3.72E-32	81.50%	0.141	IPR017878 (G3DSA:3.90.290.GENE3D); IPR017878 (PFAM); IPR011398 (PANTHER); IPR017878 (PROSITE_PROFILES); IPR017878 (SUPERFAMILY)
114	c113217_g1_i1_len_363_path_1_0_362_3	4	chaperone protein	121	4.91E-34	81.40%	0.118	Coil (COILS); IPR029047 (G3DSA:2.60.34.GENE3D); IPR013126 (PFAM); PTHR19375 (PANTHER); IPR029047 (SUPERFAMILY)
115	c113327_g1_i1_len_195_path_1_0_194_1	3	ferritin hch	65	3.51E-30	87.30%	0.104	IPR008331 (PFAM); IPR012347 (G3DSA:1.20.1260.GENE3D); IPR001519 (PANTHER); PTHR11431:SF29 (PANTHER); IPR009040 (PROSITE_PROFILES); IPR009078 (SUPERFAMILY)
116	c113929_g1_i1_len_518_path_1_0_517_0	9	prolow-density lipoprotein receptor-related protein 1	173	2.73E-52	65.80%	0.114	IPR002172 (PRINTS); IPR002172 (SMART); IPR002172 (G3DSA:4.10.400.GENE3D); G3DSA:4.10.1220.10 (GENE3D); IPR002172 (PFAM); IPR002172 (G3DSA:4.10.400.GENE3D); IPR002172 (G3DSA:4.10.400.GENE3D); PTHR10529 (PANTHER); IPR023415 (PROSITE_PATTERNS); IPR023415 (PROSITE_PATTERNS); IPR023415 (PROSITE_PATTERNS); IPR023415 (PROSITE_PATTERNS); IPR002172 (PROSITE_PROFILES); IPR002172 (PROSITE_PROFILES); IPR002172 (PROSITE_PROFILES); IPR002172 (PROSITE_PROFILES); IPR002172 (SUPERFAMILY); IPR002172 (SUPERFAMILY); IPR002172 (SUPERFAMILY); IPR002172 (SUPERFAMILY)
117	c114135_g1_i1_len_302_path_1_0_301_0	4	laminin subunit alpha-2- partial	101	4.75E-36	73.80%	0.112	IPR013320 (G3DSA:2.60.120.GENE3D); IPR001791 (PFAM); PTHR10574 (PANTHER); PTHR10574:SF148 (PANTHER); IPR001791 (PROSITE_PROFILES); IPR013320 (SUPERFAMILY)
118	c114170_g1_i1_len_268_path_1_0_267_2	3	partial	89	4.21E-55	99.70%	0.119	IPR003959 (PFAM); IPR027417 (G3DSA:3.40.50.GENE3D); PTHR23078 (PANTHER); IPR027417 (SUPERFAMILY)

119	c114694_g1_i1_len_366_path_344_0_365_2	6	peroxisomal multifunctional enzyme type 2-like	122	3.57E-63	91.40%	0.099	IPR002347 (PRINTS); IPR002198 (PRINTS); IPR016040 (G3DSA:3.40.50.GENE3D); IPR002198 (PFAM); PTHR24316:SF2 (PANTHER); PTHR24316 (PANTHER); IPR020904 (PROSITE_PATTERNS); SSF51735 (SUPERFAMILY)
120	c11473_g1_i1_len_325_path_303_0_324_1	8	serine protease nudel	108	9.65E-59	90.10%	0.113	IPR001254 (SMART); IPR001254 (PFAM); G3DSA:2.40.10.10 (GENE3D); PTHR24256 (PANTHER); IPR018114 (PROSITE_PATTERNS); IPR001254 (PROSITE_PROFILES); IPR009003 (SUPERFAMILY)
121	c114781_g1_i1_len_351_path_329_0_350_0	6	perlucin-like protein	117	1.69E-11	50.90%	0.117	IPR001304 (PFAM); IPR016186 (G3DSA:3.10.100.GENE3D); PTHR22802:SF219 (PANTHER); PTHR22802 (PANTHER); IPR001304 (PROSITE_PROFILES); IPR016187 (SUPERFAMILY)
122	c114810_g1_i1_len_287_path_1_0_286_0	4	vascular endothelial growth factor receptor 1 isoform x4	96	1.55E-34	84.00%	0.128	IPR001245 (PFAM); G3DSA:3.30.200.20 (GENE3D); PTHR24416 (PANTHER); PTHR24416:SF298 (PANTHER); IPR001824 (PROSITE_PATTERNS); IPR000719 (PROSITE_PROFILES); IPR011009 (SUPERFAMILY)
123	c114822_g1_i1_len_259_path_237_0_258_2	4	female neotenic-specific protein 3	86	8.44E-08	59.33%	0.106	no IPS match
124	c11513_g1_i1_len_1990_path_2066_0_1223_3289_1224_1248_50_1249_1989_1	263	tyrosine--trna cytoplasmic	663	0	80.30%	0.134	IPR002307 (PRINTS); IPR002305 (PFAM); G3DSA:1.10.240.10 (GENE3D); IPR002307 (TIGRFAM); IPR002547 (PFAM); IPR012340 (G3DSA:2.40.50.GENE3D); IPR014729 (G3DSA:3.40.50.GENE3D); PTHR11946 (PANTHER); IPR002547 (PROSITE_PROFILES); SSF52374 (SUPERFAMILY); IPR010987 (SUPERFAMILY); IPR012340 (SUPERFAMILY)
125	c115373_g1_i1_len_259_path_1_0_258_1	4	enolase	86	6.04E-32	80.80%	0.136	IPR020810 (PFAM); IPR029065 (G3DSA:3.20.20.GENE3D); IPR000941 (PANTHER); IPR029065 (SUPERFAMILY)
126	c115399_g1_i1_len_315_path_293_0_314_2	5	para sodium channel	105	1.68E-51	90.10%	0.263	IPR001696 (PRINTS); IPR005821 (PFAM); IPR028814 (PTHR10037:PANTHER); PTHR10037 (PANTHER); CYTOPLASMIC_DOMAIN (PHOBIUS); TRANSMEMBRANE (PHOBIUS); NON_CYTOPLASMIC_DOMAIN (PHOBIUS); TMhelix (TMHMM)
127	c11588_g1_i1_len_780_path_239_0_779_3	22	xanthine dehydrogenase	260	1.43E-96	71.30%	0.128	IPR002888 (PFAM); IPR012675 (G3DSA:3.10.20.GENE3D); IPR016167 (G3DSA:3.30.43.GENE3D); IPR002888 (G3DSA:1.10.150.GENE3D); IPR002346 (PFAM); PTHR11908:SF32 (PANTHER); PTHR11908 (PANTHER); IPR016166 (SUPERFAMILY); IPR001041 (SUPERFAMILY); IPR002888 (SUPERFAMILY)
128	c116953_g1_i1_len_364_path_1_0_363_3	6	protein toll	122	6.02E-30	63.30%	0.113	G3DSA:3.80.10.10 (GENE3D); IPR001611 (PFAM); PTHR24369 (PANTHER); IPR001611 (PROSITE_PROFILES); IPR001611 (PROSITE_PROFILES); IPR001611 (PROSITE_PROFILES); SSF52058 (SUPERFAMILY)
129	c117073_g1_i1_len_345_path_1_0_344_1	4	trehalase 1	115	9.79E-52	82.00%	0.124	IPR001661 (PFAM); IPR001661 (PANTHER); PTHR23403:SF1 (PANTHER); IPR008928 (SUPERFAMILY)
130	c11767_g2_i1_len_336_path_314_0_335_3	4	down syndrome cell adhesion	112	8.86E-57	90.40%	0.119	IPR003961 (PFAM); IPR013783 (G3DSA:2.60.40.GENE3D); IPR013783 (G3DSA:2.60.40.GENE3D); PTHR10489 (PANTHER); PTHR10489:SF567 (PANTHER); IPR003961 (PROSITE_PROFILES); IPR003961 (PROSITE_PROFILES); IPR003961 (SUPERFAMILY)
131	c118792_g1_i1_len_257_path_1_0_256_1	4	serine threonine-protein kinase nim1	86	3.79E-33	81.20%	0.103	IPR000719 (PFAM); G3DSA:1.10.510.10 (GENE3D); PTHR24343 (PANTHER); PTHR24343:SF106 (PANTHER); IPR008271 (PROSITE_PATTERNS); IPR000719 (PROSITE_PROFILES); IPR011009 (SUPERFAMILY)
132	c11892_g1_i1_len_449_path_697_0_430_73_431_447_1161_448_448_3	10	zinc finger protein 271-like	150	4.19E-39	68.60%	0.114	IPR015880 (SMART); PF13465 (PFAM); PF13912 (PFAM); IPR013087 (G3DSA:3.30.160.GENE3D); IPR007087 (PFAM); IPR013087 (G3DSA:3.30.160.GENE3D); IPR013087 (G3DSA:3.30.160.GENE3D); IPR013087 (G3DSA:3.30.160.GENE3D); PTHR24409 (PANTHER); IPR007087 (PROSITE_PATTERNS); IPR007087 (PROSITE_PATTERNS); IPR007087 (PROSITE_PATTERNS); IPR007087 (PROSITE_PATTERNS); IPR007087 (PROSITE_PROFILES); IPR007087 (PROSITE_PROFILES); IPR007087 (PROSITE_PROFILES); IPR007087 (PROSITE_PROFILES); SSF57667 (SUPERFAMILY); SSF57667 (SUPERFAMILY); SSF57667 (SUPERFAMILY)
133	c119952_g1_i1_len_2075_path_2053_0_2074_1	151	protein disulfide-isomerase a6	692	0	77.20%	0.239	PR00421 (PRINTS); IPR005788 (TIGRFAM); IPR012336 (G3DSA:3.40.30.GENE3D); IPR013766 (PFAM); PTHR18929:SF38 (PANTHER); PTHR18929 (PANTHER); IPR017937 (PROSITE_PATTERNS); IPR017937 (PROSITE_PATTERNS); TRANSMEMBRANE (PHOBIUS); NON_CYTOPLASMIC_DOMAIN (PHOBIUS); CYTOPLASMIC_DOMAIN (PHOBIUS); IPR012336 (PROSITE_PROFILES); IPR012336 (PROSITE_PROFILES); IPR012336 (SUPERFAMILY); IPR012336 (SUPERFAMILY); IPR012336 (SUPERFAMILY); TMhelix (TMHMM)

134	c119979_g1_i1_len_3138_path_1_0_3137_2	379	atp-dependent rna helicase ddx1	1046	0	83.50%	0.332	IPR014001 (SMART); IPR001650 (SMART); IPR003877 (SMART); IPR027417 (G3DSA:3.40.50.GENE3D); IPR003877 (PFAM); IPR027417 (G3DSA:3.40.50.GENE3D); IPR011545 (PFAM); IPR001650 (PFAM); PTHR24031 (PANTHER); PTHR24031:SF211 (PANTHER); NON_CYTOPLASMIC_DOMAIN (PHOBIUS); CYTOPLASMIC_DOMAIN (PHOBIUS); TRANSMEMBRANE (PHOBIUS); IPR001650 (PROSITE_PROFILES); IPR014001 (PROSITE_PROFILES); IPR014014 (PROSITE_PROFILES); IPR001870 (PROSITE_PROFILES); IPR027417 (SUPERFAMILY); IPR027417 (SUPERFAMILY); IPR013320 (SUPERFAMILY); TMhelix (TMHMM)
136	c11999_g1_i1_len_816_path_794_0_815_4	44	adp-ribosylation factor-like protein 4a	272	2.70E-95	86.70%	0.119	IPR006689 (PRINTS); IPR006687 (SMART); IPR003579 (SMART); IPR024156 (SMART); IPR005225 (TIGRFAM); IPR006689 (PFAM); IPR027417 (G3DSA:3.40.50.GENE3D); PTHR11711 (PANTHER); PTHR11711:SF121 (PANTHER); IPR024156 (PROSITE_PROFILES); IPR027417 (SUPERFAMILY)
135	c119990_g1_i1_len_375_path_353_0_374_2	6	cysteine peptidase c	125	2.08E-33	75.80%	0.098	G3DSA:3.90.70.10 (GENE3D); IPR000668 (PFAM); IPR013128 (PANTHER); SSF54001 (SUPERFAMILY)
137	c120014_g1_i1_len_900_path_878_0_899_3	36	heat shock 70 kda protein cognate 4	300	1.24E-80	81.70%	0.138	Coil (COILS); IPR013126 (PRINTS); G3DSA:3.30.30.30 (GENE3D); IPR013126 (PFAM); G3DSA:3.30.420.40 (GENE3D); PTHR19375 (PANTHER); IPR018181 (PROSITE_PATTERNS); SSF53067 (SUPERFAMILY)
138	c120054_g1_i1_len_658_path_1_0_657_3	11	group xv phospholipase a2-like	220	3.84E-74	84.10%	0.361Y	IPR003386 (PFAM); PTHR11440 (PANTHER); PTHR11440:SF47 (PANTHER); TMhelix (TMHMM)
139	c120123_g1_i1_len_344_path_322_0_343_3	5	moxd1 homolog 2	115	6.60E-19	57.10%	0.104	IPR000323 (G3DSA:2.60.120.GENE3D); IPR000323 (PFAM); PTHR10157:SF23 (PANTHER); IPR000945 (PANTHER); IPR008977 (SUPERFAMILY)
140	c120323_g1_i1_len_1235_path_1213_0_1234_4	42	cartilage oligomeric matrix protein	412	2.02E-83	53.80%	0.109	Coil (COILS); IPR024665 (PFAM); CYTOPLASMIC_DOMAIN (PHOBIUS); NON_CYTOPLASMIC_DOMAIN (PHOBIUS); TRANSMEMBRANE (PHOBIUS); TRANSMEMBRANE (PHOBIUS); NON_CYTOPLASMIC_DOMAIN (PHOBIUS)
141	c120340_g1_i1_len_1355_path_1333_0_1354_0	54	low quality protein: prolow-density lipoprotein receptor-related protein 1	452	2.13E-120	59.80%	0.104	IPR000742 (SMART); IPR000033 (SMART); G3DSA:2.10.25.10 (GENE3D); G3DSA:2.10.25.10 (GENE3D); IPR011042 (G3DSA:2.120.10.GENE3D); G3DSA:2.10.25.10 (GENE3D); IPR000033 (PFAM); PTHR10529 (PANTHER); PTHR10529:SF204 (PANTHER); IPR013032 (PROSITE_PATTERNS); IPR013032 (PROSITE_PATTERNS); IPR000742 (PROSITE_PROFILES); IPR000742 (PROSITE_PROFILES); IPR000742 (PROSITE_PROFILES); IPR000033 (PROSITE_PROFILES); IPR000033 (PROSITE_PROFILES); IPR000033 (PROSITE_PROFILES); IPR000033 (PROSITE_PROFILES); SSF57196 (SUPERFAMILY); SSF63825 (SUPERFAMILY)
142	c12041_g1_i1_len_918_path_1_0_917_4	19	low affinity immunoglobulin epsilon fc receptor isoform x2	306	2.89E-19	54.60%	0.125	IPR001304 (SMART); IPR001304 (PFAM); IPR016186 (G3DSA:3.10.100.GENE3D); PTHR22802 (PANTHER); IPR001304 (PROSITE_PROFILES); IPR016187 (SUPERFAMILY)
143	c12041_g1_i1_len_918_path_1_0_917_5	19	macrophage mannose receptor 1-like	306	4.44E-28	55.60%	0.257	IPR001304 (SMART); IPR001304 (PFAM); IPR016186 (G3DSA:3.10.100.GENE3D); PTHR22803 (PANTHER); PTHR22803:SF16 (PANTHER); IPR001304 (PROSITE_PROFILES); IPR001304 (PROSITE_PROFILES); IPR016187 (SUPERFAMILY); IPR016187 (SUPERFAMILY)
144	c120425_g1_i1_len_788_path_1_0_787_5	12	c4b-binding protein beta chain	262	8.43E-49	60.50%	0.166	IPR000436 (SMART); IPR008197 (G3DSA:4.10.75.GENE3D); IPR000436 (PFAM); G3DSA:2.10.70.10 (GENE3D); IPR008197 (PFAM); PTHR19325 (PANTHER); TRANSMEMBRANE (PHOBIUS); CYTOPLASMIC_DOMAIN (PHOBIUS); NON_CYTOPLASMIC_DOMAIN (PHOBIUS); IPR000436 (PROSITE_PROFILES); IPR008197 (PROSITE_PROFILES); IPR000436 (PROSITE_PROFILES); IPR000436 (SUPERFAMILY); IPR000436 (SUPERFAMILY)
145	c120450_g1_i1_len_626_path_1_0_625_5	16	cyclin-t isoform x6	208	1.53E-128	94.20%	0.103	IPR013763 (SMART); PIRSF028758 (PIRSF); IPR006671 (PFAM); IPR013763 (G3DSA:1.10.472.GENE3D); IPR015430 (PTHR10026:PANTHER); IPR015429 (PANTHER); IPR013763 (SUPERFAMILY); IPR013763 (SUPERFAMILY)
146	c120473_g1_i1_len_449_path_1_0_448_5	8	voltage-gated sodium partial	149	1.92E-79	92.10%	0.106	IPR027359 (G3DSA:1.20.120.GENE3D); IPR005821 (PFAM); PTHR10037 (PANTHER); IPR028814 (PTHR10037:PANTHER); CYTOPLASMIC_DOMAIN (PHOBIUS); NON_CYTOPLASMIC_DOMAIN (PHOBIUS); TRANSMEMBRANE (PHOBIUS); TRANSMEMBRANE (PHOBIUS); CYTOPLASMIC_DOMAIN (PHOBIUS); NON_CYTOPLASMIC_DOMAIN (PHOBIUS); TRANSMEMBRANE (PHOBIUS); SSF81324 (SUPERFAMILY); TMhelix (TMHMM); TMhelix (TMHMM); TMhelix (TMHMM)

147	c120490_g1_i1_len_750_path_728_0_749_3	60	fat-like cadherin-related tumor suppressor homolog isoform x5	250	3.61E-88	70.00%	0.101	IPR001881 (SMART); IPR000742 (SMART); G3DSA:2.10.25.10 (GENE3D); G3DSA:2.10.25.10 (GENE3D); IPR001881 (PFAM); PTHR24026:SF39 (PANTHER); PTHR24026 (PANTHER); IPR018097 (PROSITE_PATTERNS); IPR000152 (PROSITE_PATTERNS); IPR013032 (PROSITE_PATTERNS); NON_CYTOPLASMIC_DOMAIN (PHOBIUS); CYTOPLASMIC_DOMAIN (PHOBIUS); TRANSMEMBRANE (PHOBIUS); IPR000742 (PROSITE_PROFILES); IPR000742 (PROSITE_PROFILES); IPR000742 (PROSITE_PROFILES); SSF57196 (SUPERFAMILY); SSF57196 (SUPERFAMILY); TMhelix (TMHMM)
148	c120533_g1_i1_len_388_path_1_0_387_1	8	cadherin- partial	129	1.53E-38	66.70%	0.099	IPR002126 (SMART); IPR002126 (PFAM); IPR002126 (G3DSA:2.60.40.GENE3D); IPR002126 (G3DSA:2.60.40.GENE3D); PTHR24027 (PANTHER); PTHR24027:SF248 (PANTHER); IPR002126 (PROSITE_PROFILES); IPR015919 (SUPERFAMILY); IPR015919 (SUPERFAMILY)
149	c120537_g1_i1_len_1403_path_1_0_1402_0	69	neural cell adhesion molecule 1 isoform x1	468	3.99E-52	52.10%	0.115	IPR003599 (SMART); IPR003598 (SMART); IPR013783 (G3DSA:2.60.40.GENE3D); IPR013098 (PFAM); IPR013783 (G3DSA:2.60.40.GENE3D); PTHR19831:SF40 (PANTHER); PTHR19831 (PANTHER); CYTOPLASMIC_DOMAIN (PHOBIUS); TRANSMEMBRANE (PHOBIUS); NON_CYTOPLASMIC_DOMAIN (PHOBIUS); IPR007110 (PROSITE_PROFILES); IPR003961 (PROSITE_PROFILES); IPR007110 (PROSITE_PROFILES); IPR003961 (SUPERFAMILY); SSF48726 (SUPERFAMILY); SSF48726 (SUPERFAMILY); TMhelix (TMHMM)
150	c120579_g1_i1_len_674_path_1_0_673_4	20	venom allergen 3-like	225	1.23E-15	53.20%	0.288	IPR014044 (SMART); IPR014044 (PFAM); IPR014044 (G3DSA:3.40.33.GENE3D); IPR001283 (PANTHER); IPR014044 (SUPERFAMILY)
151	c120742_g1_i1_len_1347_path_1_0_1346_3	82	camp-dependent protein kinase catalytic	449	4.84E-121	86.30%	0.101	IPR000961 (SMART); IPR002290 (SMART); IPR017892 (PFAM); G3DSA:1.10.510.10 (GENE3D); IPR000719 (PFAM); PTHR24357:SF70 (PANTHER); PTHR24357 (PANTHER); NON_CYTOPLASMIC_DOMAIN (PHOBIUS); CYTOPLASMIC_DOMAIN (PHOBIUS); TRANSMEMBRANE (PHOBIUS); IPR000719 (PROSITE_PROFILES); IPR000961 (PROSITE_PROFILES); IPR011009 (SUPERFAMILY)
152	c120753_g1_i1_len_1340_path_1_0_1339_0	76	ras-responsive element-binding protein 1	447	1.34E-109	61.10%	0.109	IPR015880 (SMART); IPR013087 (G3DSA:3.30.160.GENE3D); IPR013087 (G3DSA:3.30.160.GENE3D); IPR013087 (G3DSA:3.30.160.GENE3D); PF13894 (PFAM); IPR013087 (G3DSA:3.30.160.GENE3D); IPR007087 (PFAM); PF13465 (PFAM); PTHR23233 (PANTHER); PTHR23233:SF42 (PANTHER); IPR007087 (PROSITE_PATTERNS); IPR007087 (PROSITE_PATTERNS); IPR007087 (PROSITE_PATTERNS); IPR007087 (PROSITE_PATTERNS); IPR007087 (PROSITE_PATTERNS); IPR007087 (PROSITE_PATTERNS); IPR007087 (PROSITE_PROFILES); IPR007087 (PROSITE_PROFILES); IPR007087 (PROSITE_PROFILES); IPR007087 (PROSITE_PROFILES); IPR007087 (PROSITE_PROFILES); SSF57667 (SUPERFAMILY); SSF57667 (SUPERFAMILY); SSF57667 (SUPERFAMILY)
153	c120768_g1_i1_len_1117_path_1_0_1116_3	49	u4 u6 small nuclear ribonucleoprotein prp31	373	0	89.10%	0.099	IPR012976 (SMART); IPR002687 (PFAM); IPR012976 (PFAM); IPR019175 (PFAM); IPR027105 (PANTHER); IPR002687 (PROSITE_PROFILES); SSF89124 (SUPERFAMILY)
154	c120779_g1_i1_len_709_path_687_0_708_4	18	endothelin-converting enzyme 1-like	236	1.42E-45	50.60%	0.102	IPR018497 (PFAM); IPR024079 (G3DSA:3.40.390.GENE3D); IPR008753 (PFAM); IPR000718 (PANTHER); SSF55486 (SUPERFAMILY)
155	c120869_g1_i1_len_989_path_1_0_988_3	45	epidermal growth factor receptor isoform x1	330	1.23E-100	65.90%	0.257	IPR001245 (PRINTS); IPR020635 (SMART); G3DSA:1.10.510.10 (GENE3D); IPR001245 (PFAM); PTHR24416:SF90 (PANTHER); PTHR24416 (PANTHER); IPR000719 (PROSITE_PROFILES); IPR011009 (SUPERFAMILY)
156	c120886_g1_i1_len_1683_path_1_0_1682_2	363	serine protease easter-like	561	1.03E-71	54.80%	0.2	IPR001314 (PRINTS); IPR001254 (SMART); IPR006604 (SMART); G3DSA:2.40.10.10 (GENE3D); G3DSA:2.40.10.10 (GENE3D); IPR001254 (PFAM); G3DSA:2.40.10.10 (GENE3D); IPR022700 (PFAM); PTHR24260:SF3 (PANTHER); PTHR24260 (PANTHER); IPR018114 (PROSITE_PATTERNS); IPR018114 (PROSITE_PATTERNS); NON_CYTOPLASMIC_DOMAIN (PHOBIUS); TRANSMEMBRANE (PHOBIUS); CYTOPLASMIC_DOMAIN (PHOBIUS); NON_CYTOPLASMIC_DOMAIN (PHOBIUS); TRANSMEMBRANE (PHOBIUS); CYTOPLASMIC_DOMAIN (PHOBIUS); TRANSMEMBRANE (PHOBIUS); IPR001254 (PROSITE_PROFILES); IPR009003 (SUPERFAMILY); TMhelix (TMHMM); TMhelix (TMHMM); TMhelix (TMHMM)
157	c120898_g1_i1_len_465_path_1_0_464_3	14	retinol dehydrogenase 13	155	4.62E-46	72.60%	0.166	IPR002347 (PRINTS); IPR002198 (PFAM); IPR016040 (G3DSA:3.40.50.GENE3D); PTHR24320:SF38 (PANTHER); PTHR24320 (PANTHER); TRANSMEMBRANE (PHOBIUS); CYTOPLASMIC_DOMAIN (PHOBIUS); NON_CYTOPLASMIC_DOMAIN (PHOBIUS); SSF51735 (SUPERFAMILY); TMhelix (TMHMM)

158	c120950_g1_i1_len_432_path_1_0_431_5	10	zinc finger protein 45-like	144	2.98E-16	62.70%	0.15	IPR015880 (SMART); IPR007087 (PFAM); IPR013087 (G3DSA:3.30.160.GENE3D); IPR013087 (G3DSA:3.30.160.GENE3D); IPR013087 (G3DSA:3.30.160.GENE3D); PF13465 (PFAM); PTHR24409 (PANTHER); IPR007087 (PROSITE_PATTERNS); IPR007087 (PROSITE_PATTERNS); IPR007087 (PROSITE_PATTERNS); IPR007087 (PROSITE_PROFILES); IPR007087 (PROSITE_PROFILES); IPR007087 (PROSITE_PROFILES); SSF57667 (SUPERFAMILY); SSF57667 (SUPERFAMILY)
159	c120969_g1_i1_len_459_path_1_0_458_5	12	l-asparaginase	153	1.01E-17	66.70%	0.116	IPR002110 (SMART); IPR020683 (G3DSA:1.25.40.GENE3D); IPR020683 (PFAM); PTHR11707 (PANTHER); PTHR11707:SF3 (PANTHER); IPR002110 (PROSITE_PROFILES); IPR020683 (PROSITE_PROFILES); IPR020683 (SUPERFAMILY)
160	c120970_g1_i1_len_732_path_710_0_731_0	17	GL24937	244	2.47E-30	69.80%	0.1	IPR019149 (PFAM); PTHR13617 (PANTHER)
161	c120994_g1_i1_len_1190_path_1168_0_1189_0	208	gpi-anchor transamidase	397	0	85.60%	0.804 Y	IPR001096 (PRINTS); IPR001096 (PFAM); IPR001096 (PIRSF); IPR028361 (PIRSF); IPR028361 (PTHR12000:PANTHER); IPR001096 (PANTHER); NON_CYTOPLASMIC_DOMAIN (PHOBIUS); SIGNAL_PEPTIDE_C_REGION (PHOBIUS); SIGNAL_PEPTIDE_N_REGION (PHOBIUS); SIGNAL_PEPTIDE (PHOBIUS); SIGNAL_PEPTIDE_H_REGION (PHOBIUS); SignalP-noTM (SIGNALP_EUK); SignalP-TM (SIGNALP_GRAM_POSITIVE); SignalP-noTM (SIGNALP_GRAM_NEGATIVE)
162	c12100_g1_i1_len_265_path_243_0_264_2	8	er degradation-enhancing alpha-mannosidase-like 1	88	6.93E-33	81.80%	0.095	IPR001382 (G3DSA:1.50.10.GENE3D); IPR001382 (PFAM); PTHR11742:SF25 (PANTHER); IPR001382 (PANTHER); IPR001382 (SUPERFAMILY)
163	c121015_g1_i1_len_1910_path_1_0_1909_0	402	wd repeat-containing protein 86-like	637	7.08E-128	65.60%	0.16	IPR020472 (PRINTS); IPR001680 (SMART); IPR015943 (G3DSA:2.130.10.GENE3D); IPR001680 (PFAM); IPR015943 (G3DSA:2.130.10.GENE3D); PTHR22844 (PANTHER); PTHR22844:SF166 (PANTHER); IPR019775 (PROSITE_PATTERNS); IPR019775 (PROSITE_PATTERNS); NON_CYTOPLASMIC_DOMAIN (PHOBIUS); TRANSMEMBRANE (PHOBIUS); CYTOPLASMIC_DOMAIN (PHOBIUS); IPR001680 (PROSITE_PROFILES); IPR001680 (PROSITE_PROFILES); IPR001680 (PROSITE_PROFILES); IPR017986 (PROSITE_PROFILES); IPR001680 (PROSITE_PROFILES); IPR017986 (SUPERFAMILY); IPR011047 (SUPERFAMILY); TMhelix (TMHMM)
164	c121019_g1_i1_len_1976_path_1_0_1975_0	163	zinc finger protein 135-like isoform x2	659	4.99E-72	56.50%	0.228	IPR015880 (SMART); PF13465 (PFAM); IPR013087 (G3DSA:3.30.160.GENE3D); IPR013087 (G3DSA:3.30.160.GENE3D); PF13912 (PFAM); PTHR24409 (PANTHER); IPR007087 (PROSITE_PATTERNS); IPR007087 (PROSITE_PATTERNS); IPR007087 (PROSITE_PATTERNS); IPR007087 (PROSITE_PATTERNS); IPR007087 (PROSITE_PATTERNS); IPR007087 (PROSITE_PATTERNS); IPR007087 (PROSITE_PATTERNS); IPR007087 (PROSITE_PATTERNS); IPR007087 (PROSITE_PROFILES); IPR007087 (PROSITE_PROFILES); IPR007087 (PROSITE_PROFILES); IPR007087 (PROSITE_PROFILES); IPR007087 (PROSITE_PROFILES); IPR007087 (PROSITE_PROFILES); IPR007087 (PROSITE_PROFILES); IPR007087 (PROSITE_PROFILES); IPR007087 (PROSITE_PROFILES); SSF57667 (SUPERFAMILY); SSF57667 (SUPERFAMILY); SSF57667 (SUPERFAMILY); SSF57667 (SUPERFAMILY); SSF57667 (SUPERFAMILY); SSF57667 (SUPERFAMILY)
165	c121045_g1_i1_len_1500_path_1478_0_1499_4	3329	peptidyl-prolyl cis-trans isomerase	500	4.96E-99	92.50%	0.327	IPR002130 (PRINTS); IPR029000 (G3DSA:2.40.100.GENE3D); IPR002130 (PFAM); IPR024936 (PANTHER); PTHR11071:SF116 (PANTHER); IPR020892 (PROSITE_PATTERNS); TRANSMEMBRANE (PHOBIUS); CYTOPLASMIC_DOMAIN (PHOBIUS); NON_CYTOPLASMIC_DOMAIN (PHOBIUS); IPR002130 (PROSITE_PROFILES); IPR029000 (SUPERFAMILY)
166	c121054_g1_i1_len_544_path_522_0_543_0	13	ccr4-not transcription complex subunit 6-like isoform x7	182	1.87E-61	80.90%	0.413Y	IPR003591 (SMART); G3DSA:3.80.10.10 (GENE3D); IPR025875 (PFAM); PTHR12121 (PANTHER); PTHR12121:SF35 (PANTHER); IPR001611 (PROSITE_PROFILES); IPR001611 (PROSITE_PROFILES); IPR001611 (PROSITE_PROFILES); SSF52058 (SUPERFAMILY)
167	c121078_g1_i1_len_1420_path_1398_0_1419_3	92	27 kda hemolymph	474	1.14E-44	56.60%	0.174	IPR009832 (PFAM); PTHR20997 (PANTHER); CYTOPLASMIC_DOMAIN (PHOBIUS); TRANSMEMBRANE (PHOBIUS); NON_CYTOPLASMIC_DOMAIN (PHOBIUS); TRANSMEMBRANE (PHOBIUS); CYTOPLASMIC_DOMAIN (PHOBIUS)
168	c121183_g1_i1_len_1093_path_1_0_1092_1	30	cd109 partial	346	5.32E-67	63.10%	0.284	IPR002890 (PFAM); PTHR11412:SF85 (PANTHER); PTHR11412 (PANTHER); TRANSMEMBRANE (PHOBIUS); NON_CYTOPLASMIC_DOMAIN (PHOBIUS); CYTOPLASMIC_DOMAIN (PHOBIUS)

169	c121189_g1_i1_len_1270_path_1_0_1269_4	50	histone-lysine n-methyltransferase prdm9	423	7.56E-65	57.60%	0.106	Coil (COILS); IPR015880 (SMART); PF13465 (PFAM); IPR013087 (G3DSA:3.30.160.GENE3D); PF13894 (PFAM); IPR013087 (G3DSA:3.30.160.GENE3D); IPR013087 (G3DSA:3.30.160.GENE3D); IPR007087 (PFAM); IPR013087 (G3DSA:3.30.160.GENE3D); PTHR24409 (PANTHER); IPR007087 (PROSITE_PATTERNS); IPR007087 (PROSITE_PATTERNS); IPR007087 (PROSITE_PATTERNS); IPR007087 (PROSITE_PATTERNS); IPR007087 (PROSITE_PATTERNS); IPR007087 (PROSITE_PATTERNS); IPR007087 (PROSITE_PATTERNS); IPR007087 (PROSITE_PATTERNS); IPR007087 (PROSITE_PATTERNS); IPR007087 (PROSITE_PROFILES); IPR007087 (PROSITE_PROFILES); IPR007087 (PROSITE_PROFILES); IPR007087 (PROSITE_PROFILES); IPR007087 (PROSITE_PROFILES); IPR007087 (PROSITE_PROFILES); IPR007087 (PROSITE_PROFILES); IPR007087 (PROSITE_PROFILES); IPR007087 (PROSITE_PROFILES); SSF57667 (SUPERFAMILY); SSF57667 (SUPERFAMILY); SSF57667 (SUPERFAMILY); SSF57667 (SUPERFAMILY); SSF57667 (SUPERFAMILY)
170	c121193_g1_i1_len_1113_path_1_0_1112_3	42	gastrula zinc finger protein - partial	371	8.60E-63	52.50%	0.134	IPR015880 (SMART); PF13465 (PFAM); IPR007087 (PFAM); IPR013087 (G3DSA:3.30.160.GENE3D); IPR013087 (G3DSA:3.30.160.GENE3D); IPR013087 (G3DSA:3.30.160.GENE3D); IPR013087 (G3DSA:3.30.160.GENE3D); PF13894 (PFAM); IPR013087 (G3DSA:3.30.160.GENE3D); PTHR24409:SF14 (PANTHER); PTHR24409 (PANTHER); IPR007087 (PROSITE_PATTERNS); IPR007087 (PROSITE_PATTERNS); IPR007087 (PROSITE_PATTERNS); IPR007087 (PROSITE_PATTERNS); IPR007087 (PROSITE_PATTERNS); IPR007087 (PROSITE_PATTERNS); IPR007087 (PROSITE_PATTERNS); IPR007087 (PROSITE_PATTERNS); IPR007087 (PROSITE_PATTERNS); IPR007087 (PROSITE_PROFILES); IPR007087 (PROSITE_PROFILES); IPR007087 (PROSITE_PROFILES); IPR007087 (PROSITE_PROFILES); IPR007087 (PROSITE_PROFILES); IPR007087 (PROSITE_PROFILES); IPR007087 (PROSITE_PROFILES); IPR007087 (PROSITE_PROFILES); IPR007087 (PROSITE_PROFILES); IPR007087 (PROSITE_PROFILES); SSF57667 (SUPERFAMILY); SSF57667 (SUPERFAMILY); SSF57667 (SUPERFAMILY); SSF57667 (SUPERFAMILY); SSF57667 (SUPERFAMILY)
171	c121246_g1_i1_len_2521_path_1_0_2520_0	317	angiotensin-converting enzyme-like	841	0	74.50%	0.344Y	IPR001548 (PRINTS); IPR001548 (PFAM); IPR001548 (PANTHER); PTHR10514:SF17 (PANTHER); TRANSMEMBRANE (PHOBIUS); CYTOPLASMIC_DOMAIN (PHOBIUS); NON_CYTOPLASMIC_DOMAIN (PHOBIUS); TRANSMEMBRANE (PHOBIUS); TRANSMEMBRANE (PHOBIUS); CYTOPLASMIC_DOMAIN (PHOBIUS); NON_CYTOPLASMIC_DOMAIN (PHOBIUS); SSF55486 (SUPERFAMILY); TMhelix (TMHMM); TMhelix (TMHMM)
172	c121256_g1_i1_len_654_path_1_0_653_5	268	peptidase s8	218	3.28E-25	69.80%	0.342Y	IPR007280 (PFAM); SIGNAL_PEPTIDE_H_REGION (PHOBIUS); NON_CYTOPLASMIC_DOMAIN (PHOBIUS); SIGNAL_PEPTIDE_N_REGION (PHOBIUS); SIGNAL_PEPTIDE_C_REGION (PHOBIUS); SIGNAL_PEPTIDE (PHOBIUS); TMhelix (TMHMM); TMhelix (TMHMM)
173	c121263_g1_i1_len_814_path_1_0_813_1	54	glypican-5 isoform x5	271	1.50E-33	57.00%	0.182	IPR001863 (PFAM); IPR001863 (PANTHER); TRANSMEMBRANE (PHOBIUS); TRANSMEMBRANE (PHOBIUS); CYTOPLASMIC_DOMAIN (PHOBIUS); NON_CYTOPLASMIC_DOMAIN (PHOBIUS); NON_CYTOPLASMIC_DOMAIN (PHOBIUS); TMhelix (TMHMM)
174	c121332_g1_i1_len_643_path_621_0_642_2	16	membrane metallo-endopeptidase-like 1-like	214	6.15E-87	73.40%	0.163	IPR018497 (PRINTS); IPR024079 (G3DSA:3.40.390.GENE3D); IPR018497 (PFAM); IPR000718 (PANTHER); PTHR11733:SF113 (PANTHER); TRANSMEMBRANE (PHOBIUS); CYTOPLASMIC_DOMAIN (PHOBIUS); NON_CYTOPLASMIC_DOMAIN (PHOBIUS); SSF55486 (SUPERFAMILY); TMhelix (TMHMM)
175	c121378_g1_i1_len_245_path_223_0_244_3	4	14/03/2003	82	3.75E-15	88.10%	0.335	G3DSA:1.20.190.20 (GENE3D); IPR023410 (PFAM); IPR000308 (PANTHER); IPR023410 (SUPERFAMILY)
176	c121397_g1_i1_len_1474_path_1_0_1473_4	770	perlucin-like protein	491	2.44E-19	53.70%	0.864 Y	Coil (COILS); IPR001304 (SMART); IPR001304 (PFAM); IPR016186 (G3DSA:3.10.100.GENE3D); PTHR22802 (PANTHER); IPR018378 (PROSITE_PATTERNS); NON_CYTOPLASMIC_DOMAIN (PHOBIUS); TRANSMEMBRANE (PHOBIUS); TRANSMEMBRANE (PHOBIUS); NON_CYTOPLASMIC_DOMAIN (PHOBIUS); CYTOPLASMIC_DOMAIN (PHOBIUS); IPR001304 (PROSITE_PROFILES); SignalP-noTM (SIGNALP_EUK); IPR016187 (SUPERFAMILY); TMhelix (TMHMM)
177	c121427_g1_i1_len_1327_path_3_0_1326_3	48	low quality protein: sialate o-acetylesterase	443	4.34E-118	62.00%	0.195	IPR005181 (PFAM); PTHR22901 (PANTHER); IPR013830 (SUPERFAMILY)
178	c121461_g1_i1_len_345_path_323_0_344_0	10	partial	115	8.28E-14	50.80%	0.104	IPR000372 (SMART); IPR000372 (PFAM); G3DSA:3.80.10.10 (GENE3D)

179	c121467_g1_i1_len_467_path_445_0_466_4	9	wd sam and u-box domain-containing protein 1-like	156	6.81E-41	69.30%	0.15	IPR020472 (PRINTS); IPR001680 (SMART); IPR001680 (PFAM); IPR015943 (G3DSA:2.130.10.GENE3D); PTHR22847 (PANTHER); IPR019775 (PROSITE_PATTERNS); IPR019775 (PROSITE_PATTERNS); IPR001680 (PROSITE_PROFILES); IPR001680 (PROSITE_PROFILES); IPR017986 (PROSITE_PROFILES); IPR001680 (PROSITE_PROFILES); IPR011047 (SUPERFAMILY)
180	c121607_g1_i1_len_1338_path_1_0_1337_2	95	allatostatin precursor protein	446	1.16E-33	53.40%	0.104	IPR010276 (PFAM); TRANSMEMBRANE (PHOBIUS); NON_CYTOPLASMIC_DOMAIN (PHOBIUS); TRANSMEMBRANE (PHOBIUS); CYTOPLASMIC_DOMAIN (PHOBIUS); CYTOPLASMIC_DOMAIN (PHOBIUS)
181	c121625_g1_i1_len_387_path_365_0_386_1	10	scavenger partial	129	3.61E-58	82.50%	0.113	IPR001695 (PFAM); PTHR19331:SF77 (PANTHER); PTHR19331 (PANTHER); IPR001190 (PROSITE_PROFILES); IPR017448 (SUPERFAMILY)
182	c121685_g1_i1_len_358_path_336_0_357_2	6	ldl receptor protein isoform g	119	2.57E-25	63.40%	0.106	IPR002172 (PRINTS); IPR002172 (SMART); IPR002172 (G3DSA:4.10.400.GENE3D); G3DSA:4.10.1220.10 (GENE3D); IPR002172 (PFAM); PTHR10529 (PANTHER); IPR023415 (PROSITE_PATTERNS); IPR023415 (PROSITE_PROFILES); IPR002172 (PROSITE_PROFILES); IPR002172 (PROSITE_PROFILES); IPR002172 (SUPERFAMILY); IPR002172 (SUPERFAMILY)
183	c121716_g1_i1_len_715_path_693_0_714_1	13	macrophage mannose receptor 1-like	238	1.35E-22	43.00%	0.113	IPR001304 (SMART); IPR016186 (G3DSA:3.10.100.GENE3D); IPR016186 (G3DSA:3.10.100.GENE3D); IPR016186 (G3DSA:3.10.100.GENE3D); IPR001304 (PFAM); PTHR22803 (PANTHER); IPR001304 (PROSITE_PROFILES); IPR001304 (PROSITE_PROFILES); IPR016187 (SUPERFAMILY); IPR016187 (SUPERFAMILY); IPR016187 (SUPERFAMILY)
184	c121773_g1_i1_len_436_path_414_0_435_5	14	fras1-related extracellular matrix protein 1	145	8.27E-22	54.80%	0.113	IPR001304 (SMART); IPR001304 (PFAM); IPR016186 (G3DSA:3.10.100.GENE3D); PTHR22801 (PANTHER); IPR018378 (PROSITE_PATTERNS); IPR001304 (PROSITE_PROFILES); IPR016187 (SUPERFAMILY)
185	c121787_g1_i1_len_267_path_245_0_266_3	5	dopamine beta- partial	89	1.96E-15	61.70%	0.118	IPR000945 (PRINTS); IPR000323 (PFAM); IPR000323 (G3DSA:2.60.120.GENE3D); IPR000945 (PANTHER); PTHR10157:SF22 (PANTHER); IPR008977 (SUPERFAMILY)
186	c121827_g1_i1_len_1397_path_1375_0_1396_0	36	retinol dehydrogenase 13-like	466	1.07E-52	64.70%	0.126	IPR002347 (PRINTS); IPR016040 (G3DSA:3.40.50.GENE3D); PTHR24320:SF55 (PANTHER); PTHR24320 (PANTHER); SSF51735 (SUPERFAMILY)
187	c121851_g1_i1_len_1327_path_1305_0_1326_4	49	probable serine carboxypeptidase cpvl isoform x2	442	3.01E-118	62.80%	0.13	IPR001563 (PRINTS); IPR029058 (G3DSA:3.40.50.GENE3D); IPR001563 (PFAM); PTHR11802:SF30 (PANTHER); IPR001563 (PANTHER); IPR018202 (PROSITE_PATTERNS); IPR029058 (SUPERFAMILY)
188	c121856_g1_i1_len_1194_path_1172_0_1193_0	61	26s protease regulatory subunit 6b	398	0	95.10%	0.099	Coil (COILS); IPR001270 (PRINTS); IPR003593 (SMART); IPR027417 (G3DSA:3.40.50.GENE3D); G3DSA:1.10.8.60 (GENE3D); IPR003959 (PFAM); IPR005937 (TIGRFAM); PTHR23073 (PANTHER); PTHR23073:SF8 (PANTHER); IPR003960 (PROSITE_PATTERNS); IPR027417 (SUPERFAMILY)
219	c122_g1_i1_len_1057_path_1085_0_1056_4	46	plexin- partial	352	1.78E-118	78.40%	0.269	IPR002909 (SMART); G3DSA:3.30.1680.10 (GENE3D); IPR002909 (PFAM); IPR013783 (G3DSA:2.60.40.GENE3D); IPR013783 (G3DSA:2.60.40.GENE3D); PTHR22625 (PANTHER); PTHR22625:SF16 (PANTHER); IPR014756 (SUPERFAMILY); IPR014756 (SUPERFAMILY)
189	c122010_g1_i1_len_798_path_1_0_797_5	14	low quality protein: sco-spondin	266	3.63E-23	44.50%	0.114	IPR002172 (PRINTS); IPR002172 (SMART); IPR002172 (G3DSA:4.10.400.GENE3D); G3DSA:4.10.1220.10 (GENE3D); IPR002172 (G3DSA:4.10.400.GENE3D); IPR002172 (PFAM); IPR002172 (G3DSA:4.10.400.GENE3D); PTHR10529:SF208 (PANTHER); PTHR10529 (PANTHER); IPR002172 (PROSITE_PROFILES); IPR002172 (PROSITE_PROFILES); IPR002172 (PROSITE_PROFILES); IPR002172 (PROSITE_PROFILES); IPR002172 (PROSITE_PROFILES); IPR002172 (SUPERFAMILY); IPR002172 (SUPERFAMILY); IPR002172 (SUPERFAMILY); IPR002172 (SUPERFAMILY); IPR002172 (SUPERFAMILY)
190	c122024_g1_i1_len_307_path_1_0_306_3	10	heat shock protein	103	2.21E-48	89.90%	0.11	IPR029047 (G3DSA:2.60.34.GENE3D); IPR029048 (G3DSA:1.20.1270.GENE3D); IPR013126 (PFAM); PTHR19375:SF142 (PANTHER); PTHR19375 (PANTHER); IPR029047 (SUPERFAMILY); IPR029048 (SUPERFAMILY)
191	c122041_g1_i1_len_330_path_1_0_329_2	21	peptidyl-prolyl cis-trans isomerase-like	110	2.72E-43	84.90%	0.599Y	IPR002130 (PRINTS); IPR002130 (PFAM); IPR029000 (G3DSA:2.40.100.GENE3D); IPR024936 (PANTHER); IPR020892 (PROSITE_PATTERNS); IPR002130 (PROSITE_PROFILES); SignalP-noTM (SIGNALP_EUK); IPR029000 (SUPERFAMILY)
192	c122114_g1_i1_len_888_path_1_0_887_2	15205	translationally controlled tumor protein	296	4.17E-71	79.20%	0.269	IPR018105 (PRINTS); IPR018105 (PFAM); IPR011323 (G3DSA:2.170.150.GENE3D); IPR018105 (PANTHER); IPR011057 (SUPERFAMILY)

193	c122187_g1_i1_len_1330_path_1_0_1329_3	61	acid sphingomyelinase-like phosphodiesterase 3b	444	1.88E-66	55.00%	0.108	IPR029052 (G3DSA:3.60.21.GENE3D); PTHR10340 (PANTHER); NON_CYTOPLASMIC_DOMAIN (PHOBIUS); TRANSMEMBRANE (PHOBIUS); CYTOPLASMIC_DOMAIN (PHOBIUS); IPR029052 (SUPERFAMILY); TMhelix (TMHMM)
194	c122188_g1_i1_len_1175_path_1153_0_1174_2	75	Ectodysplasin-A	391	2.08E-57	58.30%	0.106	IPR008983 (G3DSA:2.60.120.GENE3D); IPR006052 (PFAM); PTHR15151:SF4 (PANTHER); PTHR15151 (PANTHER); IPR021184 (PROSITE_PATTERNS); IPR006052 (PROSITE_PROFILES); IPR008983 (SUPERFAMILY)
195	c12224_g1_i1_len_159_path_137_0_158_2	1	thrombospondin type-1 domain-containing protein 4-like	53	1.16E-17	69.20%	0.127	IPR000884 (PFAM); G3DSA:2.20.100.10 (GENE3D); PTHR13723 (PANTHER); IPR000884 (PROSITE_PROFILES); IPR000884 (SUPERFAMILY)
196	c122335_g1_i1_len_1622_path_1650_0_1621_4	146	agap005784-pa-like protein	541	1.70E-47	61.70%	0.114	IPR001258 (PFAM); IPR011042 (G3DSA:2.120.10.GENE3D); PTHR10680 (PANTHER); IPR013017 (PROSITE_PROFILES); IPR013017 (PROSITE_PROFILES); IPR013017 (PROSITE_PROFILES); SSF101898 (SUPERFAMILY)
197	c122368_g1_i1_len_952_path_930_0_951_4	31	sh3 and multiple ankyrin repeat domains protein partial	317	4.35E-167	86.60%	0.098	IPR002110 (SMART); IPR020683 (PFAM); IPR020683 (G3DSA:1.25.40.GENE3D); PTHR24135 (PANTHER); IPR002110 (PROSITE_PROFILES); IPR020683 (PROSITE_PROFILES); IPR002110 (PROSITE_PROFILES); IPR020683 (SUPERFAMILY)
198	c122370_g1_i1_len_389_path_367_0_388_4	5	long form	130	3.66E-74	96.20%	0.109	Coil (COILS); Coil (COILS); Coil (COILS); IPR002928 (PFAM); PTHR13140:SF164 (PANTHER); PTHR13140 (PANTHER)
199	c122372_g1_i1_len_2532_path_1_0_2531_3	283	atp-dependent zinc metalloprotease yme1 homolog	844	0	75.30%	0.27	IPR003593 (SMART); G3DSA:1.10.8.60 (GENE3D); IPR027417 (G3DSA:3.40.50.GENE3D); IPR005936 (TIGRFAM); IPR000642 (PFAM); IPR003959 (PFAM); PTHR23076:SF37 (PANTHER); PTHR23076 (PANTHER); IPR003960 (PROSITE_PATTERNS); CYTOPLASMIC_DOMAIN (PHOBIUS); CYTOPLASMIC_DOMAIN (PHOBIUS); NON_CYTOPLASMIC_DOMAIN (PHOBIUS); TRANSMEMBRANE (PHOBIUS); TRANSMEMBRANE (PHOBIUS); NON_CYTOPLASMIC_DOMAIN (PHOBIUS); TRANSMEMBRANE (PHOBIUS); TRANSMEMBRANE (PHOBIUS); CYTOPLASMIC_DOMAIN (PHOBIUS); IPR005936 (HAMAP); SSF140990 (SUPERFAMILY); IPR027417 (SUPERFAMILY); TMhelix (TMHMM); TMhelix (TMHMM)
200	c122374_g1_i1_len_606_path_1_0_605_2	13	protein cbfa2t1-like isoform x5	202	4.66E-70	77.50%	0.134	Coil (COILS); Coil (COILS); IPR013289 (PRINTS); IPR002893 (PFAM); PTHR10379 (PANTHER); IPR002893 (PROSITE_PATTERNS); IPR002893 (PROSITE_PROFILES); SSF144232 (SUPERFAMILY)
201	c122383_g1_i1_len_583_path_561_0_582_1	24	low quality protein: myosin heavy muscle	194	1.42E-114	98.20%	0.112	Coil (COILS); Coil (COILS); IPR002928 (PFAM); PTHR13140 (PANTHER); PTHR13140:SF364 (PANTHER); SSF57997 (SUPERFAMILY)
202	c122423_g1_i1_len_1203_path_1181_0_1202_2	176	isoform d	401	5.52E-48	60.00%	0.223	IPR003585 (SMART); IPR027789 (PFAM); PTHR10915:SF1 (PANTHER); IPR001050 (PANTHER); IPR030479 (PROSITE_PATTERNS); TRANSMEMBRANE (PHOBIUS); CYTOPLASMIC_DOMAIN (PHOBIUS); CYTOPLASMIC_DOMAIN (PHOBIUS); NON_CYTOPLASMIC_DOMAIN (PHOBIUS); TRANSMEMBRANE (PHOBIUS); TMhelix (TMHMM)
203	c122429_g1_i1_len_1230_path_1_0_1229_2	112	heat shock protein 40	410	0	82.80%	0.143	IPR001623 (PRINTS); IPR001623 (SMART); IPR002939 (PFAM); G3DSA:2.60.260.20 (GENE3D); IPR001623 (PFAM); IPR001623 (G3DSA:1.10.287.GENE3D); G3DSA:2.60.260.20 (GENE3D); PTHR24077:SF211 (PANTHER); PTHR24077 (PANTHER); IPR018253 (PROSITE_PATTERNS); IPR001623 (PROSITE_PROFILES); IPR008971 (SUPERFAMILY); IPR001623 (SUPERFAMILY); IPR008971 (SUPERFAMILY)
204	c122486_g1_i1_len_750_path_728_0_749_1	13	transferrin	250	1.18E-58	57.90%	0.106	Coil (COILS); IPR001156 (PRINTS); IPR001156 (SMART); G3DSA:3.40.190.10 (GENE3D); G3DSA:3.40.190.10 (GENE3D); IPR001156 (PFAM); PTHR11485 (PANTHER); PTHR11485:SF19 (PANTHER); IPR001156 (PROSITE_PROFILES); IPR001156 (PROSITE_PROFILES); SSF53850 (SUPERFAMILY); SSF53850 (SUPERFAMILY)
205	c122547_g1_i1_len_842_path_1_0_841_2	17	peroxisomal sarcosine oxidase	280	7.24E-74	59.70%	0.153	IPR006076 (PFAM); G3DSA:3.50.50.60 (GENE3D); G3DSA:3.30.9.10 (GENE3D); PTHR10961 (PANTHER); PTHR10961:SF7 (PANTHER); SSF51905 (SUPERFAMILY)
206	c122611_g1_i1_len_606_path_584_0_605_2	17	vascular endothelial growth factor receptor 1 isoform x2	202	5.86E-26	47.60%	0.157	IPR003599 (SMART); IPR003598 (SMART); IPR013783 (G3DSA:2.60.40.GENE3D); IPR013106 (PFAM); IPR007110 (PROSITE_PROFILES); SSF48726 (SUPERFAMILY)
207	c122699_g1_i1_len_765_path_743_0_764_0	31	hornerin isoform x2	255	3.48E-29	50.50%	0.104	IPR001314 (PRINTS); IPR001254 (SMART); G3DSA:2.40.10.10 (GENE3D); IPR001254 (PFAM); G3DSA:2.40.10.10 (GENE3D); G3DSA:2.40.10.10 (GENE3D); PTHR24268 (PANTHER); IPR001254 (PROSITE_PROFILES); IPR009003 (SUPERFAMILY)

208	c122748_g1_i1_len_910_path_1_0_909_1	63	l-xylulose reductase	303	1.96E-114	78.00%	0.12	IPR002347 (PRINTS); IPR002198 (PRINTS); IPR013968 (SMART); PF13561 (PFAM); IPR016040 (G3DSA:3.40.50.GENE3D); PTHR24311:SF6 (PANTHER); PTHR24311 (PANTHER); IPR020904 (PROSITE_PATTERNS); SSF51735 (SUPERFAMILY)
209	c122754_g1_i1_len_1082_path_1060_0_1081_1	65	probable serine carboxypeptidase cpvl isoform x1	361	7.98E-93	65.30%	0.252	IPR001563 (PRINTS); IPR029058 (G3DSA:3.40.50.GENE3D); IPR001563 (PFAM); PTHR11802:SF30 (PANTHER); IPR001563 (PANTHER); IPR029058 (SUPERFAMILY)
210	c122755_g1_i1_len_1450_path_1_0_1449_0	280	26s protease regulatory subunit 7	484	0	96.10%	0.186	IPR003593 (SMART); G3DSA:1.10.8.60 (GENE3D); IPR027417 (G3DSA:3.40.50.GENE3D); IPR005937 (TIGRFAM); IPR003959 (PFAM); PTHR23073:SF13 (PANTHER); PTHR23073 (PANTHER); IPR003960 (PROSITE_PATTERNS); TRANSMEMBRANE (PHOBIUS); CYTOPLASMIC_DOMAIN (PHOBIUS); NON_CYTOPLASMIC_DOMAIN (PHOBIUS); IPR027417 (SUPERFAMILY); TMhelix (TMHMM)
211	c122781_g1_i1_len_866_path_1_0_865_0	22	chorion peroxidase	289	2.05E-51	61.00%	0.163	IPR019791 (PFAM); IPR019791 (G3DSA:1.10.640.GENE3D); PTHR11475 (PANTHER); IPR019791 (PROSITE_PROFILES); IPR010255 (SUPERFAMILY)
212	c12279_g1_i1_len_409_path_387_0_408_5	14	venom serine carboxypeptidase-like	136	2.75E-29	66.00%	0.117	IPR001563 (PFAM); IPR029058 (G3DSA:3.40.50.GENE3D); IPR001563 (PANTHER); IPR018202 (PROSITE_PATTERNS); IPR029058 (SUPERFAMILY)
213	c122866_g1_i1_len_719_path_697_0_718_0	21	titin- partial	240	4.26E-115	81.90%	0.098	IPR003599 (SMART); IPR003598 (SMART); IPR013098 (PFAM); IPR013783 (G3DSA:2.60.40.GENE3D); IPR013783 (G3DSA:2.60.40.GENE3D); PTHR19897:SF149 (PANTHER); PTHR19897 (PANTHER); IPR007110 (PROSITE_PROFILES); IPR007110 (PROSITE_PROFILES); SSF48726 (SUPERFAMILY); SSF48726 (SUPERFAMILY)
217	c1229_g1_i1_len_197_path_675_0_63_202_64_196_5	2	cadherin-related tumor suppressor- partial	65	3.17E-08	64.00%	0.19	IPR000742 (SMART); IPR000742 (PFAM); G3DSA:2.10.25.10 (GENE3D); IPR013032 (PROSITE_PATTERNS); IPR000742 (PROSITE_PROFILES); SSF57196 (SUPERFAMILY)
218	c1229_g1_i2_len_334_path_1_0_200_202_201_333_5	3	agrin- partial	111	5.00E-37	71.50%	0.155	IPR000742 (SMART); IPR013320 (G3DSA:2.60.120.GENE3D); G3DSA:2.10.25.10 (GENE3D); IPR000742 (PFAM); PTHR10574:SF227 (PANTHER); PTHR10574 (PANTHER); IPR013032 (PROSITE_PATTERNS); IPR000742 (PROSITE_PROFILES); IPR013320 (SUPERFAMILY)
214	c122900_g1_i1_len_636_path_614_0_635_3	13	meteorin-like protein	212	3.46E-14	62.20%	0.101	no IPS match
215	c122940_g1_i1_len_600_path_578_0_599_3	13	neutral endopeptidase	200	2.98E-59	70.40%	0.132	IPR008753 (PFAM); G3DSA:1.10.1380.10 (GENE3D); PTHR11733:SF113 (PANTHER); IPR000718 (PANTHER); SSF55486 (SUPERFAMILY)
216	c122990_g1_i1_len_764_path_1_0_763_1	14	adam 17-like protease	255	2.85E-83	82.90%	0.1	PTHR11905 (PANTHER); PTHR11905:SF121 (PANTHER); NON_CYTOPLASMIC_DOMAIN (PHOBIUS); CYTOPLASMIC_DOMAIN (PHOBIUS); TRANSMEMBRANE (PHOBIUS); TRANSMEMBRANE (PHOBIUS); NON_CYTOPLASMIC_DOMAIN (PHOBIUS); TMhelix (TMHMM); TMhelix (TMHMM)
220	c123004_g1_i1_len_252_path_230_0_251_0	4	neurexin-3- partial	84	1.32E-13	74.20%	0.23	IPR027789 (PFAM); CYTOPLASMIC_DOMAIN (PHOBIUS); NON_CYTOPLASMIC_DOMAIN (PHOBIUS); TRANSMEMBRANE (PHOBIUS); TMhelix (TMHMM)
221	c123034_g1_i1_len_1781_path_1_0_1780_1	97	dual specificity mitogen-activated protein kinase kinase 4-like	594	0	89.60%	0.169	IPR002290 (SMART); IPR000719 (PFAM); G3DSA:3.30.200.20 (GENE3D); G3DSA:1.10.510.10 (GENE3D); PTHR24360:SF46 (PANTHER); PTHR24360 (PANTHER); IPR017441 (PROSITE_PATTERNS); IPR008271 (PROSITE_PATTERNS); IPR000719 (PROSITE_PROFILES); IPR011009 (SUPERFAMILY)
222	c123063_g1_i1_len_341_path_1_0_340_2	12	xanthine dehydrogenase	113	3.79E-40	80.20%	0.104	IPR000674 (PFAM); IPR000674 (G3DSA:3.90.1170.GENE3D); IPR008274 (PFAM); IPR008274 (G3DSA:3.30.365.GENE3D); PTHR11908 (PANTHER); PTHR11908:SF32 (PANTHER); IPR008274 (SUPERFAMILY); IPR000674 (SUPERFAMILY)
223	c123088_g1_i1_len_1114_path_1092_0_1113_2	144	aminoacyl trna synthase complex-interacting multifunctional protein 1	371	8.53E-100	68.30%	0.119	Coil (COILS); IPR002547 (PFAM); IPR012340 (G3DSA:2.40.50.GENE3D); PTHR11586 (PANTHER); PTHR11586:SF1 (PANTHER); IPR002547 (PROSITE_PROFILES); IPR012340 (SUPERFAMILY)
224	c123130_g1_i1_len_1322_path_1_0_1321_3	345	adp-ribosylation factor-like protein 8b-a	441	6.97E-124	97.70%	0.118	IPR006689 (PRINTS); IPR003579 (SMART); IPR006687 (SMART); IPR024156 (SMART); IPR005225 (TIGRFAM); IPR027417 (G3DSA:3.40.50.GENE3D); IPR006689 (PFAM); PTHR11711 (PANTHER); PTHR11711:SF122 (PANTHER); NON_CYTOPLASMIC_DOMAIN (PHOBIUS); CYTOPLASMIC_DOMAIN (PHOBIUS); NON_CYTOPLASMIC_DOMAIN (PHOBIUS); TRANSMEMBRANE (PHOBIUS); TRANSMEMBRANE (PHOBIUS); IPR024156 (PROSITE_PROFILES); IPR027417 (SUPERFAMILY)

225	c123208_g1_i1_len_1203_path_1181_0_1202_4	32	c4b-binding protein beta	401	0	75.50%	0.102	IPR000436 (SMART); G3DSA:2.10.70.10 (GENE3D); G3DSA:2.10.70.10 (GENE3D); IPR000436 (PFAM); G3DSA:2.10.70.10 (GENE3D); IPR013783 (G3DSA:2.60.40.GENE3D); PTHR19325 (PANTHER); PTHR19325:SF230 (PANTHER); IPR000436 (PROSITE_PROFILES); IPR000436 (PROSITE_PROFILES); IPR000436 (PROSITE_PROFILES); IPR000436 (PROSITE_PROFILES); SSF48726 (SUPERFAMILY); IPR000436 (SUPERFAMILY); IPR000436 (SUPERFAMILY); IPR000436 (SUPERFAMILY)
226	c123384_g1_i1_len_409_path_1_0_408_1	6	prolow-density lipoprotein receptor-related protein 1-like isoform x6	136	2.57E-15	52.20%	0.105	IPR002172 (PRINTS); IPR002172 (SMART); IPR002172 (G3DSA:4.10.400.GENE3D); IPR002172 (G3DSA:4.10.400.GENE3D); G3DSA:4.10.1220.10 (GENE3D); IPR002172 (PFAM); PTHR10529:SF204 (PANTHER); PTHR10529 (PANTHER); IPR002172 (PROSITE_PROFILES); IPR002172 (PROSITE_PROFILES); IPR002172 (SUPERFAMILY); IPR002172 (SUPERFAMILY); IPR002172 (SUPERFAMILY)
227	c123444_g1_i1_len_392_path_1_0_391_4	10	pr domain zinc finger protein 1	131	2.38E-71	98.00%	0.097	IPR015880 (SMART); IPR013087 (G3DSA:3.30.160.GENE3D); IPR013087 (G3DSA:3.30.160.GENE3D); IPR013087 (G3DSA:3.30.160.GENE3D); PF13465 (PFAM); IPR016608 (PTHR11389:PANTHER); PTHR11389 (PANTHER); IPR007087 (PROSITE_PATTERNS); IPR007087 (PROSITE_PATTERNS); IPR007087 (PROSITE_PATTERNS); IPR007087 (PROSITE_PROFILES); IPR007087 (PROSITE_PROFILES); IPR007087 (PROSITE_PROFILES); SSF57667 (SUPERFAMILY); SSF57667 (SUPERFAMILY)
228	c12348_g1_i1_len_1269_path_1830_0_1195_99_1196_1268_3	114	camp-dependent protein kinase catalytic subunit	423	7.64E-151	94.20%	0.124	Coil (COILS); IPR002290 (SMART); IPR000719 (PFAM); G3DSA:1.10.510.10 (GENE3D); G3DSA:3.30.200.20 (GENE3D); PTHR24353 (PANTHER); IPR017441 (PROSITE_PATTERNS); IPR008271 (PROSITE_PATTERNS); IPR000719 (PROSITE_PROFILES); IPR011009 (SUPERFAMILY)
229	c12348_g1_i2_len_1597_path_1830_0_1195_3025_1196_1596_4	143	camp-dependent protein kinase catalytic subunit	532	1.58E-147	94.20%	0.368Y	Coil (COILS); IPR002290 (SMART); IPR000719 (PFAM); G3DSA:3.30.200.20 (GENE3D); G3DSA:1.10.510.10 (GENE3D); PTHR24353 (PANTHER); IPR008271 (PROSITE_PATTERNS); IPR017441 (PROSITE_PATTERNS); IPR000719 (PROSITE_PROFILES); IPR011009 (SUPERFAMILY)
230	c123527_g1_i1_len_411_path_389_0_410_1	15	hemagglutinin amebocyte aggregation factor-like	137	4.57E-13	56.50%	0.16	PF14704 (PFAM); IPR026645 (PANTHER); PTHR15040:SF1 (PANTHER); TRANSMEMBRANE (PHOBIUS); CYTOPLASMIC_DOMAIN (PHOBIUS); NON_CYTOPLASMIC_DOMAIN (PHOBIUS); TMhelix (TMHMM)
231	c123625_g1_i1_len_1122_path_1_0_1121_2	36	low quality protein: de-cadherin	374	4.27E-61	57.30%	0.155	IPR001791 (SMART); IPR027397 (G3DSA:4.10.900.GENE3D); IPR013320 (G3DSA:2.60.120.GENE3D); IPR001791 (PFAM); IPR000233 (PFAM); PTHR24027:SF276 (PANTHER); PTHR24027 (PANTHER); CYTOPLASMIC_DOMAIN (PHOBIUS); NON_CYTOPLASMIC_DOMAIN (PHOBIUS); TRANSMEMBRANE (PHOBIUS); IPR001791 (PROSITE_PROFILES); IPR013320 (SUPERFAMILY); TMhelix (TMHMM)
232	c123759_g1_i1_len_287_path_265_0_286_5	5	matrix metalloproteinase-17-like	95	1.22E-47	86.40%	0.129	IPR018487 (SMART); IPR000585 (G3DSA:2.110.10.GENE3D); IPR018487 (PFAM); PTHR10201:SF135 (PANTHER); PTHR10201 (PANTHER); IPR018487 (PROSITE_PROFILES); IPR018487 (PROSITE_PROFILES); IPR000585 (SUPERFAMILY)
233	c123824_g1_i1_len_552_path_1_0_551_1	18	cd166 antigen	184	2.08E-35	60.50%	0.104	IPR013783 (G3DSA:2.60.40.GENE3D); PTHR11640:SF52 (PANTHER); PTHR11640 (PANTHER); TRANSMEMBRANE (PHOBIUS); NON_CYTOPLASMIC_DOMAIN (PHOBIUS); CYTOPLASMIC_DOMAIN (PHOBIUS); SSF48726 (SUPERFAMILY); TMhelix (TMHMM)
234	c123838_g1_i1_len_646_path_624_0_645_2	30	fibronectin type 3 and ankyrin repeat domains protein 1	215	4.25E-42	69.50%	0.455 Y	IPR002110 (PRINTS); IPR002110 (SMART); IPR020683 (G3DSA:1.25.40.GENE3D); IPR020683 (PFAM); IPR002110 (PFAM); PTHR24183:SF1 (PANTHER); PTHR24183 (PANTHER); NON_CYTOPLASMIC_DOMAIN (PHOBIUS); SIGNAL_PEPTIDE_N_REGION (PHOBIUS); SIGNAL_PEPTIDE_C_REGION (PHOBIUS); SIGNAL_PEPTIDE (PHOBIUS); SIGNAL_PEPTIDE_H_REGION (PHOBIUS); IPR020683 (PROSITE_PROFILES); IPR002110 (PROSITE_PROFILES); IPR002110 (PROSITE_PROFILES); IPR002110 (PROSITE_PROFILES); IPR002110 (PROSITE_PROFILES); SignalP-noTM (SIGNALP_EUK); IPR020683 (SUPERFAMILY)
235	c123895_g1_i1_len_372_path_1_0_371_0	5	ovochymase- partial	124	6.16E-23	55.50%	0.362 Y	IPR001254 (PFAM); G3DSA:2.40.10.10 (GENE3D); PTHR24265 (PANTHER); IPR018114 (PROSITE_PATTERNS); IPR001254 (PROSITE_PROFILES); IPR009003 (SUPERFAMILY)
236	c123902_g1_i1_len_808_path_786_0_807_0	36	hemagglutinin	270	1.42E-26	55.50%	0.175	G3DSA:1.10.390.10 (GENE3D); IPR001570 (PFAM); IPR007280 (PFAM); TRANSMEMBRANE (PHOBIUS); CYTOPLASMIC_DOMAIN (PHOBIUS); CYTOPLASMIC_DOMAIN (PHOBIUS); NON_CYTOPLASMIC_DOMAIN (PHOBIUS); TRANSMEMBRANE (PHOBIUS); SSF55486 (SUPERFAMILY); TMhelix (TMHMM); TMhelix (TMHMM)

237	c123945_g1_i1_len_743_path_1_0_742_1	14	trehalase-like isoform x1	248	1.77E-68	69.20%	0.129	IPR001661 (PRINTS); IPR001661 (PFAM); IPR001661 (PANTHER); PTHR23403:SF1 (PANTHER); IPR018232 (PROSITE_PATTERNS); IPR008928 (SUPERFAMILY)
238	c123971_g1_i1_len_478_path_1_0_477_4	12	dual specificity testis-specific protein kinase 2	159	2.07E-78	84.90%	0.103	IPR001245 (PRINTS); G3DSA:1.10.510.10 (GENE3D); IPR001245 (PFAM); PTHR23257:SF324 (PANTHER); PTHR23257 (PANTHER); IPR008266 (PROSITE_PATTERNS); IPR000719 (PROSITE_PROFILES); IPR011009 (SUPERFAMILY)
239	c123979_g1_i1_len_1227_path_1205_0_1226_1	63	protein twisted gastrulation	409	1.27E-108	76.40%	0.151	IPR006761 (PFAM); IPR006761 (PANTHER); PTHR12312:SF15 (PANTHER); CYTOPLASMIC_DOMAIN (PHOBIUS); TRANSMEMBRANE (PHOBIUS); NON_CYTOPLASMIC_DOMAIN (PHOBIUS)
240	c124050_g1_i1_len_1382_path_1_0_1381_5	132	aael014742- partial	460	3.09E-34	47.20%	0.224	IPR002110 (PRINTS); IPR002110 (SMART); IPR020683 (PFAM); IPR020683 (G3DSA:1.25.40.GENE3D); PTHR24198 (PANTHER); IPR002110 (PROSITE_PROFILES); IPR002110 (PROSITE_PROFILES); IPR002110 (PROSITE_PROFILES); IPR002110 (PROSITE_PROFILES); IPR002110 (PROSITE_PROFILES); IPR002110 (PROSITE_PROFILES); IPR020683 (PROSITE_PROFILES); IPR002110 (PROSITE_PROFILES); IPR020683 (SUPERFAMILY)
241	c12408_g1_i1_len_529_path_1_0_528_1	9	---NA---	176			0.145	IPR000421 (PFAM); IPR008979 (G3DSA:2.60.120.GENE3D); IPR000421 (PROSITE_PATTERNS); IPR000421 (PROSITE_PROFILES); IPR008979 (SUPERFAMILY)
242	c124175_g1_i1_len_674_path_1_0_673_4	26	heat shock 70 kda protein 14-like	225	1.90E-78	68.40%	0.104	G3DSA:3.30.420.40 (GENE3D); G3DSA:3.30.420.40 (GENE3D); IPR013126 (PFAM); G3DSA:3.90.640.10 (GENE3D); PTHR19375:SF158 (PANTHER); PTHR19375 (PANTHER); SSF53067 (SUPERFAMILY); SSF53067 (SUPERFAMILY)
243	c124181_g1_i1_len_454_path_1_0_453_0	5	protease inhibitor-like	152	1.29E-15	67.70%	0.585Y	IPR002223 (PRINTS); IPR002223 (SMART); IPR002223 (PFAM); IPR002223 (G3DSA:4.10.410.GENE3D); PTHR10083 (PANTHER); IPR020901 (PROSITE_PATTERNS); NON_CYTOPLASMIC_DOMAIN (PHOBIUS); SIGNAL_PEPTIDE_C_REGION (PHOBIUS); SIGNAL_PEPTIDE_N_REGION (PHOBIUS); SIGNAL_PEPTIDE (PHOBIUS); SIGNAL_PEPTIDE_H_REGION (PHOBIUS); IPR002223 (PROSITE_PROFILES); SignalP-TM (SIGNALP_EUK); IPR002223 (SUPERFAMILY); TMhelix (TMHMM)
244	c124343_g1_i1_len_1887_path_1_0_1886_2	179	leukotriene a-4 hydrolase	629	0	73.70%	0.125	IPR014782 (PRINTS); IPR014782 (PFAM); G3DSA:1.10.390.10 (GENE3D); IPR012777 (TIGRFAM); IPR015211 (PFAM); PTHR11533:SF4 (PANTHER); IPR001930 (PANTHER); IPR016024 (SUPERFAMILY); SSF55486 (SUPERFAMILY); SSF63737 (SUPERFAMILY)
245	c124358_g1_i1_len_757_path_1_0_756_1	53	upf0669 protein c6orf120 homolog	252	2.77E-37	64.00%	0.107	PTHR31703 (PANTHER)
246	c124495_g1_i1_len_297_path_1_0_296_3	5	aael002876- partial	99	7.59E-40	79.40%	0.101	IPR000859 (G3DSA:2.60.120.GENE3D); IPR000859 (PFAM); PTHR10127:SF557 (PANTHER); PTHR10127 (PANTHER); IPR000859 (PROSITE_PROFILES); IPR000859 (SUPERFAMILY)
247	c124561_g1_i1_len_402_path_1_0_200_1_201_401_1	4	ankyrin domain partial	134	1.31E-22	54.70%	0.106	IPR002110 (SMART); IPR020683 (G3DSA:1.25.40.GENE3D); IPR020683 (G3DSA:1.25.40.GENE3D); PF13637 (PFAM); IPR020683 (PFAM); PTHR24198 (PANTHER); IPR020683 (PROSITE_PROFILES); IPR002110 (PROSITE_PROFILES); IPR002110 (PROSITE_PROFILES); IPR002110 (PROSITE_PROFILES); IPR020683 (SUPERFAMILY)
248	c124577_g1_i1_len_1118_path_1096_0_1117_1	35	secreted frizzled-related protein 3	373	6.98E-61	57.90%	0.172	Coil (COILS); IPR018933 (SMART); IPR020067 (SMART); G3DSA:2.40.50.120 (GENE3D); IPR020067 (PFAM); IPR018933 (PFAM); IPR020067 (G3DSA:1.10.2000.GENE3D); IPR015526 (PANTHER); PTHR11309:SF83 (PANTHER); IPR020067 (PROSITE_PROFILES); IPR001134 (PROSITE_PROFILES); IPR020067 (SUPERFAMILY); IPR008993 (SUPERFAMILY)
249	c124595_g1_i1_len_598_path_1_0_597_4	18	sco- partial	199	1.41E-08	52.25%	0.105	IPR025155 (PFAM)
250	c12482_g1_i1_len_674_path_652_0_94_747_95_103_756_104_673_2	18	protein unc-45 homolog b	224	1.10E-59	73.10%	0.119	IPR019734 (SMART); IPR013105 (PFAM); PF13414 (PFAM); IPR011990 (G3DSA:1.25.40.GENE3D); PTHR22904:SF302 (PANTHER); PTHR22904 (PANTHER); IPR019734 (PROSITE_PROFILES); IPR019734 (PROSITE_PROFILES); IPR019734 (PROSITE_PROFILES); IPR013026 (PROSITE_PROFILES); SSF48452 (SUPERFAMILY)
251	c12482_g1_i2_len_770_path_652_0_94_1454_95_199_756_200_769_2	14	protein unc-45 homolog b	256	1.88E-59	73.10%	0.113	IPR019734 (SMART); PF13414 (PFAM); IPR013105 (PFAM); IPR011990 (G3DSA:1.25.40.GENE3D); PTHR22904:SF302 (PANTHER); PTHR22904 (PANTHER); CYTOPLASMIC_DOMAIN (PHOBIUS); NON_CYTOPLASMIC_DOMAIN (PHOBIUS); TRANSMEMBRANE (PHOBIUS); IPR019734 (PROSITE_PROFILES); IPR019734 (PROSITE_PROFILES); IPR013026 (PROSITE_PROFILES); IPR019734 (PROSITE_PROFILES); SSF48452 (SUPERFAMILY); TMhelix (TMHMM)

252	c124960_g1_i1_len_1131_path_1_0_1130_5	33	matrix metalloproteinase 9	377	7.83E-14	49.10%	0.106		IPR000562 (SMART); IPR000562 (PFAM); IPR000562 (G3DSA:2.10.10.GENE3D); IPR000562 (G3DSA:2.10.10.GENE3D); IPR000562 (PROSITE_PROFILES); IPR000562 (PROSITE_PROFILES); IPR013806 (SUPERFAMILY); IPR013806 (SUPERFAMILY)
253	c124966_g1_i1_len_879_path_857_0_878_0	62	e3 ubiquitin-protein ligase mib2 isoform x1	293	5.65E-141	77.70%	0.1		IPR002110 (PRINTS); IPR002110 (SMART); IPR020683 (G3DSA:1.25.40.GENE3D); IPR020683 (G3DSA:1.25.40.GENE3D); IPR020683 (PFAM); PTHR24202 (PANTHER); PTHR24202:SF4 (PANTHER); IPR002110 (PROSITE_PROFILES); IPR002110 (PROSITE_PROFILES); IPR002110 (PROSITE_PROFILES); IPR002110 (PROSITE_PROFILES); IPR020683 (PROSITE_PROFILES); IPR020683 (SUPERFAMILY)
254	c125282_g1_i1_len_357_path_335_0_356_4	8	egf-like module-containing mucin-like hormone receptor-like partial	119	2.09E-22	60.20%	0.242		IPR000832 (PFAM); PTHR12011:SF235 (PANTHER); PTHR12011 (PANTHER); TRANSMEMBRANE (PHOBIUS); CYTOPLASMIC_DOMAIN (PHOBIUS); NON_CYTOPLASMIC_DOMAIN (PHOBIUS); TRANSMEMBRANE (PHOBIUS); NON_CYTOPLASMIC_DOMAIN (PHOBIUS); TRANSMEMBRANE (PHOBIUS); CYTOPLASMIC_DOMAIN (PHOBIUS); IPR017981 (PROSITE_PROFILES); TMhelix (TMHMM); TMhelix (TMHMM); TMhelix (TMHMM)
255	c125283_g1_i1_len_271_path_249_0_270_1	4	macrophage mannose receptor 1-like	90	1.68E-10	55.00%	0.115		IPR016186 (G3DSA:3.10.100.GENE3D); IPR001304 (PFAM); PTHR22803 (PANTHER); IPR001304 (PROSITE_PROFILES); IPR016187 (SUPERFAMILY)
256	c125450_g1_i1_len_1093_path_1_0_1092_5	26	attractin-like protein 1 isoform x1	364	3.35E-140	73.60%	0.106		IPR006652 (SMART); IPR000742 (SMART); IPR006652 (PFAM); IPR000859 (G3DSA:2.60.120.GENE3D); PF13418 (PFAM); IPR015916 (G3DSA:2.130.10.GENE3D); G3DSA:2.170.300.10 (GENE3D); IPR013111 (PFAM); PTHR10574 (PANTHER); PTHR10574:SF3 (PANTHER); IPR013032 (PROSITE_PATTERNS); IPR013032 (PROSITE_PATTERNS); IPR013032 (PROSITE_PATTERNS); IPR000742 (PROSITE_PROFILES); SSF117281 (SUPERFAMILY); IPR000859 (SUPERFAMILY)
257	c125508_g1_i1_len_1258_path_1236_0_1257_4	69	carboxypeptidase b-like	419	3.21E-127	62.40%	0.642	Y	IPR000834 (PRINTS); IPR000834 (SMART); IPR003146 (G3DSA:3.30.70.GENE3D); G3DSA:3.40.630.10 (GENE3D); IPR003146 (PFAM); IPR000834 (PFAM); PTHR11705 (PANTHER); IPR000834 (PROSITE_PATTERNS); NON_CYTOPLASMIC_DOMAIN (PHOBIUS); SIGNAL_PEPTIDE_N_REGION (PHOBIUS); SIGNAL_PEPTIDE (PHOBIUS); SIGNAL_PEPTIDE_H_REGION (PHOBIUS); SIGNAL_PEPTIDE_C_REGION (PHOBIUS); SignalP-noTM (SIGNALP_EUK); IPR009020 (SUPERFAMILY); SSF53187 (SUPERFAMILY)
258	c125515_g1_i1_len_415_path_1_0_414_0	6	phosphoglycerate kinase	139	1.81E-38	66.30%	0.11		IPR001576 (PRINTS); IPR015901 (G3DSA:3.40.50.GENE3D); IPR001576 (PFAM); IPR001576 (PANTHER); PTHR11406:SF6 (PANTHER); IPR001576 (SUPERFAMILY)
259	c125609_g1_i1_len_364_path_342_0_363_1	12	polypeptide n-acetylgalactosaminyltransferase 2-like	121	8.46E-30	58.70%	0.337		PTHR11675:SF24 (PANTHER); PTHR11675 (PANTHER); NON_CYTOPLASMIC_DOMAIN (PHOBIUS); CYTOPLASMIC_DOMAIN (PHOBIUS); TRANSMEMBRANE (PHOBIUS); TMhelix (TMHMM)
260	c12563_g1_i1_len_572_path_825_0_322_1148_323_433_50_434_571_2	22	calponin homology domain containing	190	9.70E-59	66.20%	0.146		Coil (COILS); IPR025875 (PFAM); G3DSA:3.80.10.10 (GENE3D); PTHR23155 (PANTHER); PTHR23155:SF423 (PANTHER); IPR001611 (PROSITE_PROFILES); IPR001611 (PROSITE_PROFILES); IPR001611 (PROSITE_PROFILES); SSF52075 (SUPERFAMILY)
261	c12563_g1_i2_len_776_path_825_0_322_1148_323_433_1258_434_637_50_638_775_2	36	leucine-rich repeat and calponin homology domain-containing protein 1	258	1.30E-67	58.80%	0.146		Coil (COILS); IPR001611 (PFAM); G3DSA:3.80.10.10 (GENE3D); PTHR23155 (PANTHER); PTHR23155:SF423 (PANTHER); IPR001611 (PROSITE_PROFILES); IPR001611 (PROSITE_PROFILES); IPR001611 (PROSITE_PROFILES); SSF52075 (SUPERFAMILY)
262	c125789_g1_i1_len_567_path_1_0_566_2	12	growth differentiation factor 8	189	5.14E-31	65.60%	0.124		IPR001111 (PFAM); IPR015616 (PTHR11848:PANTHER); IPR015615 (PANTHER)
263	c126170_g1_i1_len_378_path_383_0_377_2	4553	60s acidic ribosomal protein p1	126	3.65E-31	93.40%	0.131		PF00428 (PFAM); PTHR21141 (PANTHER); IPR027534 (HAMAP)
264	c126295_g1_i1_len_372_path_1_0_371_2	6	polyprotein precursor	124	6.54E-19	54.80%	0.102		IPR000208 (PFAM); SSF56672 (SUPERFAMILY)
265	c126328_g1_i1_len_680_path_1_0_679_4	25	neuroendocrine convertase 2-like	227	6.97E-135	87.70%	0.126		IPR002884 (PFAM); IPR008979 (G3DSA:2.60.120.GENE3D); IPR000209 (G3DSA:3.40.50.GENE3D); PTHR10795:SF11 (PANTHER); IPR015500 (PANTHER); IPR000209 (SUPERFAMILY); IPR008979 (SUPERFAMILY)
266	c126361_g1_i1_len_343_path_321_0_342_0	10	transmembrane protease serine partial	115	1.06E-19	68.40%	0.147		G3DSA:2.40.10.10 (GENE3D); IPR001254 (PFAM); PTHR24268:SF81 (PANTHER); PTHR24268 (PANTHER); IPR018114 (PROSITE_PATTERNS); IPR001254 (PROSITE_PROFILES); IPR009003 (SUPERFAMILY)
267	c126565_g1_i1_len_262_path_1_0_261_0	7	matrix metalloproteinase-16 isoform x3	88	1.12E-30	78.30%	0.111		IPR018487 (SMART); IPR018487 (PFAM); IPR000585 (G3DSA:2.110.10.GENE3D); PTHR10201 (PANTHER); PTHR10201:SF131 (PANTHER); IPR018487 (PROSITE_PROFILES); IPR018487 (PROSITE_PROFILES); IPR000585 (SUPERFAMILY)

268	c126700_g1_i1_len_696_path_1_0_695_5	15	rna polymerase ii largest subunit	232	8.43E-55	85.90%	0.096	PR01217 (PRINTS); IPR000684 (PFAM); IPR000684 (PROSITE_PATTERNS); IPR000684 (PROSITE_PATTERNS); IPR000684 (PROSITE_PATTERNS); IPR000684 (PROSITE_PATTERNS); IPR000684 (PROSITE_PATTERNS); IPR000684 (PROSITE_PATTERNS); IPR000684 (PROSITE_PATTERNS); IPR000684 (PROSITE_PATTERNS); IPR000684 (PROSITE_PATTERNS); IPR000684 (PROSITE_PATTERNS); IPR000684 (PROSITE_PATTERNS); IPR000684 (PROSITE_PATTERNS); IPR000684 (PROSITE_PATTERNS)
269	c126806_g1_i1_len_455_path_433_0_454_5	14	exocyst complex component 7	151	4.54E-37	77.70%	0.147	PTHR12542:SF7 (PANTHER); IPR004140 (PANTHER)
270	c126822_g1_i1_len_647_path_1_0_646_0	18	chondroitin sulfate synthase 1-like	216	1.46E-69	68.50%	0.406Y	IPR008428 (PFAM); IPR029044 (G3DSA:3.90.550.GENE3D); PTHR12369:SF11 (PANTHER); PTHR12369 (PANTHER); IPR029044 (SUPERFAMILY)
271	c127048_g1_i1_len_289_path_1_0_288_0	8	collagen alpha-1 chain	97	3.08E-10	61.70%	0.097	IPR008160 (PFAM); PTHR24023 (PANTHER)
272	c127048_g1_i1_len_289_path_1_0_288_5	8	hypothetical protein, partial	96	1.86E-10	48.50%	0.126	no IPS match
273	c127220_g1_i1_len_459_path_437_0_458_4	12	vanin-like protein 1-like	153	2.09E-10	49.80%	0.103	PTHR10609:SF14 (PANTHER); IPR012101 (PANTHER)
274	c127260_g1_i1_len_169_path_157_0_168_2	0	dehydrogenase reductase sdr family member 11	56	4.52E-14	77.60%	0.148	IPR016040 (G3DSA:3.40.50.GENE3D); PTHR24322 (PANTHER); PTHR24322:SF310 (PANTHER); SSF51735 (SUPERFAMILY)
275	c127377_g1_i1_len_1071_path_1_0_1070_0	38	leucine-rich repeat-containing protein 58 isoform x2	357	2.38E-93	75.20%	0.157	IPR003591 (SMART); SM00364 (SMART); IPR001611 (PFAM); G3DSA:3.80.10.10 (GENE3D); PTHR23155:SF408 (PANTHER); PTHR23155 (PANTHER); IPR001611 (PROSITE_PROFILES); IPR001611 (PROSITE_PROFILES); IPR001611 (PROSITE_PROFILES); IPR001611 (PROSITE_PROFILES); SSF52058 (SUPERFAMILY)
276	c127400_g1_i1_len_776_path_1_0_775_2	15	leucine-rich repeat protein soc-2 isoform x1	258	1.20E-138	88.10%	0.11	IPR001611 (PFAM); G3DSA:3.80.10.10 (GENE3D); IPR001611 (PFAM); PTHR16083:SF2 (PANTHER); PTHR16083 (PANTHER); SSF52075 (SUPERFAMILY)
277	c12743_g1_i1_len_207_path_1_0_206_2	2	low quality protein: von willebrand factor type egf and pentraxin domain-containing protein 1-like	69	1.04E-16	70.70%	0.108	IPR011641 (PFAM); PTHR24049:SF6 (PANTHER); PTHR24049 (PANTHER); IPR009030 (SUPERFAMILY)
278	c12743_g2_i1_len_150_path_369_0_149_0	2	von willebrand factor type egf and pentraxin domain-containing protein 1	50	4.72E-13	80.00%	0.107	IPR001881 (SMART); IPR000742 (SMART); G3DSA:2.10.25.10 (GENE3D); IPR000742 (PFAM); PTHR24049 (PANTHER); PTHR24049:SF6 (PANTHER); IPR013032 (PROSITE_PATTERNS); IPR013032 (PROSITE_PATTERNS); IPR000742 (PROSITE_PROFILES); SSF57196 (SUPERFAMILY)
279	c127534_g1_i1_len_1409_path_1_0_1408_0	50	chondroitin sulfate synthase 1	470	0	75.90%	0.231	IPR008428 (PFAM); PTHR12369 (PANTHER); PTHR12369:SF11 (PANTHER)
280	c127601_g1_i1_len_257_path_1_0_256_0	4	iron zinc purple acid phosphatase-like protein isoform x1	86	4.02E-25	79.30%	0.108	IPR025733 (PFAM); IPR029052 (G3DSA:3.60.21.GENE3D); PTHR22953 (PANTHER); IPR029052 (SUPERFAMILY)
281	c127665_g1_i1_len_275_path_1_0_274_4	4	zinc finger protein 543	92	2.08E-12	57.30%	0.099	IPR015880 (SMART); PF13894 (PFAM); PF13465 (PFAM); IPR013087 (G3DSA:3.30.160.GENE3D); IPR013087 (G3DSA:3.30.160.GENE3D); PTHR24409:SF14 (PANTHER); PTHR24409 (PANTHER); IPR007087 (PROSITE_PATTERNS); IPR007087 (PROSITE_PATTERNS); IPR007087 (PROSITE_PROFILES); IPR007087 (PROSITE_PROFILES); SSF57667 (SUPERFAMILY)
282	c127709_g1_i1_len_417_path_395_0_416_5	10	zinc finger protein 665-like	139	1.88E-53	70.80%	0.101	IPR015880 (SMART); IPR013087 (G3DSA:3.30.160.GENE3D); IPR013087 (G3DSA:3.30.160.GENE3D); PF13465 (PFAM); IPR013087 (G3DSA:3.30.160.GENE3D); IPR013087 (G3DSA:3.30.160.GENE3D); PTHR24409 (PANTHER); IPR007087 (PROSITE_PATTERNS); IPR007087 (PROSITE_PATTERNS); IPR007087 (PROSITE_PATTERNS); IPR007087 (PROSITE_PATTERNS); IPR007087 (PROSITE_PROFILES); IPR007087 (PROSITE_PROFILES); IPR007087 (PROSITE_PROFILES); IPR007087 (PROSITE_PROFILES); SSF57667 (SUPERFAMILY); SSF57667 (SUPERFAMILY); SSF57667 (SUPERFAMILY)
283	c127858_g1_i1_len_299_path_277_0_298_0	8	imaginal disc growth factor	100	1.93E-55	88.70%	0.14	IPR001223 (PFAM); IPR013781 (G3DSA:3.20.20.GENE3D); IPR029070 (G3DSA:3.10.50.GENE3D); IPR015520 (PTHR11177:PANTHER); PTHR11177 (PANTHER); IPR029070 (SUPERFAMILY); IPR017853 (SUPERFAMILY)
284	c127859_g1_i1_len_643_path_1_0_642_0	14	blastula protease 10-like	215	2.22E-17	52.10%	0.293	IPR001506 (PFAM); IPR024079 (G3DSA:3.40.390.GENE3D); PTHR10127:SF580 (PANTHER); PTHR10127 (PANTHER); SSF55486 (SUPERFAMILY)
285	c127981_g1_i1_len_310_path_1_0_309_0	7	er-type hsp70	104	1.53E-26	77.00%	0.104	IPR013126 (PFAM); IPR029047 (G3DSA:2.60.34.GENE3D); IPR029048 (G3DSA:1.20.1270.GENE3D); PTHR19375 (PANTHER); PTHR19375:SF144 (PANTHER); IPR029047 (SUPERFAMILY)
286	c127984_g1_i1_len_314_path_292_0_313_2	27	low-density lipoprotein receptor class a domain-containing protein 3	104	8.84E-11	53.00%	0.117	IPR002172 (SMART); IPR002172 (PFAM); IPR002172 (G3DSA:4.10.400.GENE3D); PTHR10529 (PANTHER); IPR002172 (PROSITE_PROFILES); IPR002172 (SUPERFAMILY); IPR002172 (SUPERFAMILY)
287	c128048_g1_i1_len_244_path_222_0_243_4	4	#NAME?	81	5.31E-14	61.00%	0.105	IPR002110 (SMART); IPR020683 (PFAM); IPR020683 (G3DSA:1.25.40.GENE3D); PTHR24188 (PANTHER); IPR020683 (PROSITE_PROFILES); IPR002110 (PROSITE_PROFILES); IPR002110 (PROSITE_PROFILES); IPR020683 (SUPERFAMILY)

288	c128120_g1_i1_len_305_path_1_0_304_1	10	isoform e	102	2.37E-07	57.00%	0.238	IPR002172 (SMART); IPR002172 (G3DSA:4.10.400.GENE3D); IPR002172 (PFAM); IPR002172 (PROSITE_PROFILES); IPR002172 (SUPERFAMILY)
289	c128203_g1_i1_len_281_path_259_0_280_0	4	caskin-1 isoform x9	94	1.94E-22	73.00%	0.104	IPR020683 (PFAM); IPR020683 (G3DSA:1.25.40.GENE3D); IPR027013 (PTHR24177:PANTHER); IPR027001 (PANTHER); IPR020683 (PROSITE_PROFILES); IPR002110 (PROSITE_PROFILES); IPR020683 (SUPERFAMILY)
291	c12823_g1_i1_len_1062_path_1337_0_213_298_214_1061_2	101	n-acetylglucosamine-1-phosphotransferase subunit gamma-like	354	4.35E-72	60.30%	0.340Y	Coil (COILS); PF13015 (PFAM); IPR009011 (G3DSA:2.70.130.GENE3D); PTHR12630 (PANTHER); PTHR12630:SF6 (PANTHER); IPR009011 (SUPERFAMILY)
292	c12823_g1_i2_len_996_path_150_0_147_298_148_995_2	74	n-acetylglucosamine-1-phosphotransferase subunit partial	332	1.03E-68	61.80%	0.137	Coil (COILS); PF13015 (PFAM); IPR009011 (G3DSA:2.70.130.GENE3D); PTHR12630:SF6 (PANTHER); PTHR12630 (PANTHER); SignalP-TM (SIGNALP_GRAM_POSITIVE); IPR009011 (SUPERFAMILY)
290	c128233_g1_i1_len_336_path_314_0_335_1	10	transient receptor potential cation channel subfamily a member 1 homolog	112	6.12E-20	60.20%	0.128	IPR002110 (PRINTS); IPR002110 (SMART); IPR020683 (G3DSA:1.25.40.GENE3D); IPR020683 (PFAM); IPR002110 (PFAM); PTHR24188 (PANTHER); IPR020683 (PROSITE_PROFILES); IPR002110 (PROSITE_PROFILES); IPR002110 (PROSITE_PROFILES); IPR020683 (SUPERFAMILY)
293	c128407_g1_i1_len_516_path_494_0_515_1	11	zinc finger protein 271 (zinc finger protein 7) (zinc finger protein znfphex133) (epstein-barr virus-induced zinc finger protein) (znf-eb) (ct-zfp48) (zinc finger protein	172	1.43E-24	59.10%	0.099	IPR015880 (SMART); IPR013087 (G3DSA:3.30.160.GENE3D); PF13465 (PFAM); IPR013087 (G3DSA:3.30.160.GENE3D); PTHR24409 (PANTHER); IPR007087 (PROSITE_PATTERNS); IPR007087 (PROSITE_PATTERNS); IPR007087 (PROSITE_PATTERNS); IPR007087 (PROSITE_PATTERNS); IPR007087 (PROSITE_PROFILES); IPR007087 (PROSITE_PROFILES); IPR007087 (PROSITE_PROFILES); IPR007087 (PROSITE_PROFILES); SSF57667 (SUPERFAMILY); SSF57667 (SUPERFAMILY)
294	c128413_g1_i1_len_381_path_1_0_380_2	8	collagen alpha chain cg42342 isoform x1	127	5.78E-22	80.00%	0.114	IPR008160 (PFAM); PTHR24023 (PANTHER); PTHR24023:SF379 (PANTHER)
295	c128413_g1_i1_len_381_path_1_0_380_5	8	collagen-like partial	127	2.82E-17	54.30%	0.136	no IPS match
296	c128425_g1_i1_len_308_path_286_0_307_0	6	PREDICTED: uncharacterized protein C4orf29 homolog isoform X5	103	5.43E-40	81.20%	0.147	IPR019149 (PFAM); PTHR13617 (PANTHER)
297	c128496_g1_i1_len_392_path_370_0_391_1	8	hemicentin- partial	131	5.54E-11	56.50%	0.191	IPR000884 (SMART); G3DSA:2.20.100.10 (GENE3D); IPR000884 (PFAM); PTHR19897 (PANTHER); NON_CYTOPLASMIC_DOMAIN (PHOBIUS); TRANSMEMBRANE (PHOBIUS); CYTOPLASMIC_DOMAIN (PHOBIUS); IPR000884 (PROSITE_PROFILES); IPR000884 (SUPERFAMILY); TMhelix (TMHMM)
298	c128508_g1_i1_len_1004_path_1_0_1003_5	42	hypothetical protein LOTGIDRAFT_71188, partial	334	5.02E-85	58.50%	0.243	IPR000859 (SMART); IPR000859 (G3DSA:2.60.120.GENE3D); IPR000859 (G3DSA:2.60.120.GENE3D); IPR000859 (PFAM); PTHR10127 (PANTHER); IPR028876 (PTHR10127:PANTHER); IPR000859 (PROSITE_PROFILES); IPR000859 (PROSITE_PROFILES); IPR000859 (PROSITE_PROFILES); IPR000859 (SUPERFAMILY); IPR000859 (SUPERFAMILY); IPR000859 (SUPERFAMILY)
299	c128528_g1_i1_len_323_path_301_0_322_2	4	von willebrand factor type egf and pentraxin domain-containing protein 1	107	1.06E-10	61.50%	0.105	IPR000436 (SMART); G3DSA:2.10.70.10 (GENE3D); IPR000436 (PFAM); PTHR19325 (PANTHER); IPR000436 (PROSITE_PROFILES); IPR000436 (SUPERFAMILY)
300	c128649_g1_i1_len_274_path_252_0_273_2	4	very low-density lipoprotein receptor	91	2.01E-21	63.20%	0.106	IPR002172 (PRINTS); IPR002172 (SMART); IPR002172 (G3DSA:4.10.400.GENE3D); G3DSA:4.10.1220.10 (GENE3D); IPR002172 (PFAM); PTHR10529 (PANTHER); PTHR10529:SF204 (PANTHER); IPR023415 (PROSITE_PATTERNS); IPR002172 (PROSITE_PROFILES); IPR002172 (SUPERFAMILY)
301	c12871_g1_i1_len_143_path_1_0_142_2	1	rap guanine nucleotide exchange factor 2	47	3.07E-21	95.20%	0.108	IPR001478 (PFAM); IPR001478 (G3DSA:2.30.42.GENE3D); PTHR23113 (PANTHER); PTHR23113:SF198 (PANTHER); IPR001478 (PROSITE_PROFILES); IPR001478 (SUPERFAMILY)
302	c129003_g1_i1_len_479_path_457_0_478_1	7	tectonic-like protein	160	2.04E-38	63.70%	0.174	PTHR14611 (PANTHER); PTHR14611:SF0 (PANTHER)
303	c129146_g1_i1_len_819_path_1_0_818_5	34	zinc finger protein 676-like	273	3.85E-39	67.60%	0.11	IPR015880 (SMART); PF13465 (PFAM); IPR007087 (PFAM); IPR013087 (G3DSA:3.30.160.GENE3D); IPR013087 (G3DSA:3.30.160.GENE3D); PTHR24409 (PANTHER); IPR007087 (PROSITE_PATTERNS); IPR007087 (PROSITE_PATTERNS); IPR007087 (PROSITE_PROFILES); IPR007087 (PROSITE_PROFILES); SSF57667 (SUPERFAMILY)
304	c129363_g1_i1_len_404_path_382_0_403_1	6	very low-density lipoprotein receptor	135	3.35E-09	66.90%	0.112	IPR002172 (SMART); IPR002172 (G3DSA:4.10.400.GENE3D); IPR002172 (PFAM); IPR023415 (PROSITE_PATTERNS); IPR002172 (PROSITE_PROFILES); IPR002172 (SUPERFAMILY)
305	c129448_g1_i1_len_312_path_1_0_311_5	6	core histone macro-	104	1.04E-37	85.90%	0.25	IPR002119 (PRINTS); IPR002119 (SMART); IPR009072 (G3DSA:1.10.20.GENE3D); IPR007125 (PFAM); PTHR23430:SF33 (PANTHER); PTHR23430 (PANTHER); IPR009072 (SUPERFAMILY)

306	c129612_g1_i1_len_392_path_1_0_391_5	8	3-ketodihydrosphingosine reductase	130	8.10E-13	70.60%	0.242	IPR016040 (G3DSA:3.40.50.GENE3D); IPR002198 (PFAM); PTHR24320 (PANTHER); CYTOPLASMIC_DOMAIN (PHOBIUS); TRANSMEMBRANE (PHOBIUS); NON_CYTOPLASMIC_DOMAIN (PHOBIUS); TRANSMEMBRANE (PHOBIUS); NON_CYTOPLASMIC_DOMAIN (PHOBIUS); SSF51735 (SUPERFAMILY); TMhelix (TMHMM)
307	c129673_g1_i1_len_385_path_1_0_384_5	5	gamma-glutamyltranspeptidase 1	128	3.68E-38	65.00%	0.101	IPR000101 (PRINTS); IPR000101 (PFAM); IPR000101 (PANTHER); PTHR11686:SF15 (PANTHER); IPR029055 (SUPERFAMILY)
308	c129682_g1_i1_len_482_path_460_0_481_1	7	lysosomal pro-x carboxypeptidase	161	2.69E-66	72.60%	0.132	IPR008758 (PFAM); IPR008758 (PANTHER); PTHR11010:SF11 (PANTHER)
309	c129762_g1_i1_len_377_path_1_0_376_1	14	basement membrane-specific heparan sulfate proteoglycan core protein	126	1.22E-33	66.00%	0.122	IPR018031 (SMART); IPR000034 (PFAM); PTHR10574 (PANTHER); PTHR10574:SF252 (PANTHER); IPR000034 (PROSITE_PROFILES)
310	c13003_g1_i1_len_396_path_584_0_395_2	9	serine protease easter	132	5.30E-23	60.20%	0.119	IPR001254 (SMART); IPR001254 (PFAM); G3DSA:2.40.10.10 (GENE3D); PTHR24260 (PANTHER); IPR018114 (PROSITE_PATTERNS); IPR001254 (PROSITE_PROFILES); IPR009003 (SUPERFAMILY)
311	c130255_g1_i1_len_1089_path_1067_0_1088_1	142	prohormone-1-like	363	7.69E-30	77.80%	0.151	CYTOPLASMIC_DOMAIN (PHOBIUS); NON_CYTOPLASMIC_DOMAIN (PHOBIUS); TRANSMEMBRANE (PHOBIUS); TMhelix (TMHMM)
312	c130283_g1_i1_len_301_path_279_0_300_0	7	protein flightless-1	101	4.46E-46	88.00%	0.165	IPR029006 (G3DSA:3.40.20.GENE3D); IPR007123 (PFAM); IPR007122 (PANTHER); IPR029919 (PTHR11977:PANTHER); SSF55753 (SUPERFAMILY)
313	c130460_g1_i1_len_436_path_1_0_435_5	10	trehalase isoform x1	145	7.41E-28	68.40%	0.196	IPR001661 (PFAM); IPR001661 (PANTHER); PTHR23403:SF1 (PANTHER); IPR008928 (SUPERFAMILY)
314	c130728_g1_i1_len_659_path_637_0_658_5	19	leucine-rich repeat protein soc-2 homolog	219	4.40E-130	93.70%	0.111	IPR003591 (SMART); IPR025875 (PFAM); G3DSA:3.80.10.10 (GENE3D); G3DSA:3.80.10.10 (GENE3D); IPR001611 (PFAM); IPR027036 (PTHR23155:PANTHER); PTHR23155 (PANTHER); IPR001611 (PROSITE_PROFILES); IPR001611 (PROSITE_PROFILES); IPR001611 (PROSITE_PROFILES); IPR001611 (PROSITE_PROFILES); IPR001611 (PROSITE_PROFILES); IPR001611 (PROSITE_PROFILES); IPR001611 (PROSITE_PROFILES); SSF52058 (SUPERFAMILY)
315	c130729_g1_i1_len_468_path_446_0_467_0	10	kunitz and ntr domain-containing protein 2	156	1.42E-49	89.00%	0.13	IPR003598 (SMART); IPR013098 (PFAM); IPR013783 (G3DSA:2.60.40.GENE3D); NON_CYTOPLASMIC_DOMAIN (PHOBIUS); CYTOPLASMIC_DOMAIN (PHOBIUS); TRANSMEMBRANE (PHOBIUS); IPR007110 (PROSITE_PROFILES); SSF48726 (SUPERFAMILY); TMhelix (TMHMM)
316	c130789_g1_i1_len_265_path_1_0_264_4	4	neurotactin	88	4.73E-28	76.20%	0.147	IPR029058 (G3DSA:3.40.50.GENE3D); IPR002018 (PFAM); PTHR11559 (PANTHER); PTHR11559:SF140 (PANTHER); IPR029058 (SUPERFAMILY)
317	c130830_g1_i1_len_310_path_1_0_309_5	7	#NAME?	103	9.23E-49	91.60%	0.126	G3DSA:3.40.50.2000 (GENE3D); IPR001830 (PFAM); PTHR10788 (PANTHER); PTHR10788:SF6 (PANTHER); SSF53756 (SUPERFAMILY)
318	c13094_g2_i1_len_146_path_354_0_145_2	1	multiple epidermal growth factor-like domains protein 8	48	3.11E-14	92.30%	0.151	TRANSMEMBRANE (PHOBIUS); NON_CYTOPLASMIC_DOMAIN (PHOBIUS); CYTOPLASMIC_DOMAIN (PHOBIUS); TMhelix (TMHMM)
319	c130993_g1_i1_len_483_path_1_0_482_3	13	mlrp1_acrmi ame: full=mam and ldl-receptor class a domain-containing protein 1 ame: full=skeletal organic matrix mam and ldl-receptor 1 short=som mam and ldl-receptor partial	161	3.04E-16	64.20%	0.31	IPR000998 (PFAM); PTHR23282:SF75 (PANTHER); PTHR23282 (PANTHER); CYTOPLASMIC_DOMAIN (PHOBIUS); TRANSMEMBRANE (PHOBIUS); CYTOPLASMIC_DOMAIN (PHOBIUS); NON_CYTOPLASMIC_DOMAIN (PHOBIUS); TRANSMEMBRANE (PHOBIUS); IPR000998 (PROSITE_PROFILES); IPR000998 (PROSITE_PROFILES); IPR013320 (SUPERFAMILY); TMhelix (TMHMM); TMhelix (TMHMM)
320	c131137_g1_i1_len_359_path_337_0_358_5	7	angiopoietin-related protein 1	119	1.46E-26	71.10%	0.125	IPR014715 (G3DSA:4.10.530.GENE3D); IPR002181 (PFAM); PTHR19143 (PANTHER); IPR020837 (PROSITE_PATTERNS); IPR002181 (PROSITE_PROFILES); IPR002181 (SUPERFAMILY)
321	c131300_g1_i1_len_357_path_1_0_356_1	6	insulin-like growth factor-binding protein complex acid labile chain	119	6.83E-30	63.10%	0.102	IPR003591 (SMART); G3DSA:3.80.10.10 (GENE3D); IPR001611 (PFAM); PTHR24369 (PANTHER); SSF52058 (SUPERFAMILY)
322	c131397_g1_i1_len_311_path_1_0_310_4	7	receptor tyrosine phosphatase type r2a	104	1.51E-38	80.10%	0.115	IPR003599 (SMART); IPR003598 (SMART); IPR013098 (PFAM); IPR013783 (G3DSA:2.60.40.GENE3D); PTHR19134 (PANTHER); PTHR19134:SF199 (PANTHER); IPR007110 (PROSITE_PROFILES); SSF48726 (SUPERFAMILY)
325	c1314_g1_i1_len_151_path_343_0_150_5	3	isoform b	50	5.36E-21	92.20%	0.097	IPR001245 (PFAM); G3DSA:3.30.200.20 (GENE3D); PTHR24418:SF0 (PANTHER); PTHR24418 (PANTHER); IPR000719 (PROSITE_PROFILES); IPR011009 (SUPERFAMILY)
323	c131460_g1_i1_len_336_path_1_0_335_2	3	low quality protein: de-cadherin	112	1.42E-22	61.20%	0.205	Coil (COILS); IPR000233 (PFAM); PTHR24027:SF251 (PANTHER); PTHR24027 (PANTHER); NON_CYTOPLASMIC_DOMAIN (PHOBIUS); CYTOPLASMIC_DOMAIN (PHOBIUS); TRANSMEMBRANE (PHOBIUS); TMhelix (TMHMM)
324	c131483_g1_i1_len_582_path_560_0_581_1	9	limbic system-associated membrane protein	194	3.65E-81	81.40%	0.099	IPR003598 (SMART); IPR003599 (SMART); IPR013783 (G3DSA:2.60.40.GENE3D); IPR013098 (PFAM); PF13895 (PFAM); PTHR23279 (PANTHER); IPR007110 (PROSITE_PROFILES); SSF48726 (SUPERFAMILY); SSF48726 (SUPERFAMILY); TMhelix (TMHMM)

326	c13168_g1_i1_len_638_path_616_0_637_0	49	alpha-mannosidase partial	213	1.55E-110	81.30%	0.102	IPR000602 (PFAM); IPR027291 (G3DSA:3.20.110.GENE3D); IPR015341 (G3DSA:1.20.1270.GENE3D); PTHR11607 (PANTHER); PTHR11607:SF4 (PANTHER); IPR011330 (SUPERFAMILY)
327	c131734_g1_i1_len_781_path_1_0_780_3	18	fasciclin- partial	261	3.56E-30	47.40%	0.102	IPR003599 (SMART); IPR003598 (SMART); IPR013783 (G3DSA:2.60.40.GENE3D); IPR013783 (G3DSA:2.60.40.GENE3D); IPR013098 (PFAM); IPR013783 (G3DSA:2.60.40.GENE3D); PTHR10489 (PANTHER); IPR007110 (PROSITE_PROFILES); IPR007110 (PROSITE_PROFILES); IPR007110 (PROSITE_PROFILES); SSF48726 (SUPERFAMILY); SSF48726 (SUPERFAMILY); SSF48726 (SUPERFAMILY)
328	c131753_g1_i1_len_343_path_321_0_342_1	3	low quality protein: basement membrane-specific heparan sulfate proteoglycan core	114	2.17E-16	64.90%	0.420Y	IPR002172 (PRINTS); IPR002172 (SMART); IPR002172 (G3DSA:4.10.400.GENE3D); IPR002172 (G3DSA:4.10.400.GENE3D); IPR002172 (PFAM); PTHR10529 (PANTHER); IPR023415 (PROSITE_PATTERNS); IPR023415 (PROSITE_PATTERNS); NON_CYTOPLASMIC_DOMAIN (PHOBIUS); SIGNAL_PEPTIDE_H_REGION (PHOBIUS); SIGNAL_PEPTIDE (PHOBIUS); SIGNAL_PEPTIDE_C_REGION (PHOBIUS); SIGNAL_PEPTIDE_N_REGION (PHOBIUS); IPR002172 (PROSITE_PROFILES); IPR002172 (PROSITE_PROFILES); IPR002172 (SUPERFAMILY); IPR002172 (SUPERFAMILY)
329	c131783_g1_i1_len_293_path_271_0_292_1	2	lysosomal pro-x carboxypeptidase	98	1.73E-42	79.40%	0.152	IPR008758 (PFAM); IPR029058 (G3DSA:3.40.50.GENE3D); PTHR11010:SF11 (PANTHER); IPR008758 (PANTHER); IPR029058 (SUPERFAMILY)
330	c131936_g1_i1_len_265_path_1_0_264_5	4	sortilin-related receptor	88	3.48E-08	49.90%	0.122	IPR002172 (PRINTS); IPR002172 (SMART); IPR002172 (G3DSA:4.10.400.GENE3D); PTHR10529 (PANTHER); IPR002172 (PROSITE_PROFILES); IPR002172 (SUPERFAMILY); IPR002172 (SUPERFAMILY)
331	c131982_g1_i1_len_750_path_728_0_749_4	35	aldehyde dehydrogenase family 9 member a1-b-like	250	1.72E-111	78.80%	0.112	IPR016163 (G3DSA:3.40.309.GENE3D); IPR016162 (G3DSA:3.40.605.GENE3D); IPR015590 (PFAM); PTHR11699 (PANTHER); PTHR11699:SF114 (PANTHER); IPR016160 (PROSITE_PATTERNS); IPR029510 (PROSITE_PATTERNS); IPR016161 (SUPERFAMILY)
332	c132012_g1_i1_len_346_path_1_0_345_1	10	discoidin domain-containing receptor partial	115	1.07E-47	79.40%	0.107	IPR020635 (SMART); IPR001245 (PFAM); G3DSA:1.10.510.10 (GENE3D); PTHR24416 (PANTHER); PTHR24416:SF210 (PANTHER); IPR000719 (PROSITE_PROFILES); IPR011009 (SUPERFAMILY)
333	c132014_g1_i1_len_389_path_1_0_388_5	5	four-jointed box protein 1-like	129	4.58E-59	79.40%	0.101	IPR024868 (PRINTS); IPR024868 (PANTHER); PTHR13147:SF5 (PANTHER)
334	c132073_g1_i1_len_285_path_1_0_284_1	4	mucin-19 isoform x2	95	5.04E-08	60.00%	0.146	IPR001846 (PFAM); IPR001846 (PROSITE_PROFILES)
335	c132540_g1_i1_len_353_path_331_0_352_2	4	gastrula zinc finger protein	117	4.30E-15	58.50%	0.105	IPR015880 (SMART); PF13465 (PFAM); IPR013087 (G3DSA:3.30.160.GENE3D); IPR013087 (G3DSA:3.30.160.GENE3D); PTHR24409 (PANTHER); PTHR24409:SF14 (PANTHER); IPR007087 (PROSITE_PATTERNS); IPR007087 (PROSITE_PATTERNS); IPR007087 (PROSITE_PROFILES); IPR007087 (PROSITE_PROFILES); SSF57667 (SUPERFAMILY); SSF57667 (SUPERFAMILY)
336	c132579_g1_i1_len_295_path_1_0_294_3	2	beta- -galactosyltransferase 1-like	99	8.40E-33	77.00%	0.112	IPR029044 (G3DSA:3.90.550.GENE3D); IPR027995 (PFAM); IPR003859 (PANTHER); IPR029044 (SUPERFAMILY)
338	c1326_g1_i1_len_881_path_2725_0_880_3	35	peroxidasin homolog	294	2.62E-29	47.40%	0.125	IPR019791 (PRINTS); IPR019791 (PFAM); IPR019791 (G3DSA:1.10.640.GENE3D); PTHR11475 (PANTHER); IPR019791 (PROSITE_PROFILES); IPR010255 (SUPERFAMILY)
337	c132639_g1_i1_len_606_path_584_0_605_3	10	dlp hdl-bgbp precursor	202	9.12E-64	71.80%	0.098	IPR015817 (G3DSA:2.20.50.GENE3D); IPR001747 (PFAM); IPR015255 (PFAM); IPR011030 (G3DSA:1.25.10.GENE3D); PTHR23345:SF11 (PANTHER); PTHR23345 (PANTHER); IPR015819 (SUPERFAMILY); IPR011030 (SUPERFAMILY)
339	c132822_g1_i1_len_355_path_1_0_354_4	7	Agrin, partial	118	2.69E-19	62.10%	0.143	IPR013320 (G3DSA:2.60.120.GENE3D); IPR001791 (PFAM); PTHR10574 (PANTHER); IPR001791 (PROSITE_PROFILES); IPR013320 (SUPERFAMILY)
340	c132862_g1_i1_len_388_path_366_0_387_0	6	neural- partial	130	1.73E-37	61.00%	0.106	G3DSA:2.170.300.10 (GENE3D); IPR000742 (PFAM); IPR013320 (G3DSA:2.60.120.GENE3D); IPR013032 (PROSITE_PATTERNS); IPR013032 (PROSITE_PATTERNS); IPR000742 (PROSITE_PROFILES); IPR013320 (SUPERFAMILY); SSF57196 (SUPERFAMILY)
341	c133015_g1_i1_len_251_path_229_0_250_5	3	low quality protein: coagulation factor vii	83	5.44E-06	54.00%	0.121	IPR001254 (PFAM); G3DSA:2.40.10.10 (GENE3D); IPR018114 (PROSITE_PATTERNS); IPR009003 (SUPERFAMILY)
342	c133040_g1_i1_len_259_path_1_0_258_4	4	glucose dehydrogenase	86	7.34E-22	68.40%	0.112	G3DSA:3.50.50.60 (GENE3D); IPR000172 (PFAM); PTHR11552:SF77 (PANTHER); PTHR11552 (PANTHER); SSF51905 (SUPERFAMILY)
343	c133072_g1_i1_len_372_path_350_0_371_1	8	hypothetical protein L798_10748	124	2.82E-23	60.40%	0.165	IPR007074 (PFAM); PTHR13627:SF24 (PANTHER); PTHR13627 (PANTHER)
344	c133269_g1_i1_len_382_path_360_0_381_1	13	oocyte zinc finger protein 26-like isoform x2	127	1.61E-10	67.40%	0.112	IPR015880 (SMART); IPR013087 (G3DSA:3.30.160.GENE3D); IPR013087 (G3DSA:3.30.160.GENE3D); PF13465 (PFAM); PTHR24409 (PANTHER); IPR007087 (PROSITE_PATTERNS); IPR007087 (PROSITE_PROFILES); SSF57667 (SUPERFAMILY)
345	c133478_g1_i1_len_382_path_1_0_381_5	6	mucin-19 isoform x1	127	4.22E-09	46.20%	0.125	IPR001846 (PFAM); PTHR11339 (PANTHER); IPR001846 (PROSITE_PROFILES)

346	c133538_g1_i1_len_293_path_1_0_292_0	4	fmrfamide-related neuropeptides	98	2.01E-08	70.80%	0.143	TRANSMEMBRANE (PHOBIUS); NON_CYTOPLASMIC_DOMAIN (PHOBIUS); CYTOPLASMIC_DOMAIN (PHOBIUS); TMhelix (TMHMM)
347	c133563_g1_i1_len_234_path_212_0_233_4	3	glucose dehydrogenase	78	1.92E-18	88.00%	0.127	G3DSA:3.50.50.60 (GENE3D); IPR007867 (PFAM); PTHR11552:SF90 (PANTHER); PTHR11552 (PANTHER); SSF51905 (SUPERFAMILY)
348	c133596_g1_i1_len_257_path_1_0_256_4	4	transforming growth factor-beta-induced protein ig-h3	86	6.64E-28	63.80%	0.104	IPR000782 (G3DSA:2.30.180.GENE3D); IPR000782 (PFAM); PTHR10900 (PANTHER); IPR000782 (PROSITE_PROFILES); IPR000782 (SUPERFAMILY)
349	c133652_g1_i1_len_286_path_264_0_285_2	9	alpha- -mannosyl-glycoprotein 4-beta-n-acetylglucosaminyltransferase partial	95	4.38E-24	63.10%	0.149	IPR006759 (PFAM); IPR006759 (PANTHER); PTHR12062:SF0 (PANTHER)
350	c133657_g1_i1_len_744_path_722_0_743_3	11	polypeptide n-acetylgalactosaminyltransferase 14	248	1.47E-97	76.80%	0.105	IPR029044 (G3DSA:3.90.550.GENE3D); IPR001173 (PFAM); PTHR11675 (PANTHER); IPR029044 (SUPERFAMILY)
351	c133671_g1_i1_len_393_path_1_0_392_5	6	serine proteinase stubble-like	131	2.63E-07	71.40%	0.115	G3DSA:2.40.10.10 (GENE3D); IPR001254 (PFAM); PTHR24268:SF94 (PANTHER); PTHR24268 (PANTHER); IPR009003 (SUPERFAMILY)
352	c133697_g1_i1_len_333_path_311_0_332_1	4	signal cub and egf-like domain-containing protein 1 isoform x1	111	4.26E-27	81.00%	0.112	PTHR24046:SF4 (PANTHER); PTHR24046 (PANTHER)
353	c134148_g1_i1_len_562_path_540_0_561_0	25	isoform b	188	1.21E-11	62.60%	0.115	G3DSA:2.40.10.10 (GENE3D); IPR001254 (PFAM); PTHR24268:SF94 (PANTHER); PTHR24268 (PANTHER); IPR018114 (PROSITE_PATTERNS); IPR009003 (SUPERFAMILY)
354	c134556_g1_i1_len_345_path_323_0_344_1	4	laminin subunit beta-1 isoform x1	115	2.03E-32	71.30%	0.102	Coil (COILS); Coil (COILS); G3DSA:1.20.58.60 (GENE3D); SSF46966 (SUPERFAMILY)
355	c134572_g1_i1_len_479_path_1_0_478_0	8	a disintegrin and metalloproteinase with thrombospondin motifs 14	160	6.56E-15	53.40%	0.096	IPR002870 (PFAM); PTHR13723 (PANTHER); PTHR13723:SF153 (PANTHER)
356	c13471_g2_i1_len_535_path_255_0_534_0	38	cyclin-g-associated kinase	179	2.70E-69	78.80%	0.105	IPR029021 (G3DSA:3.90.190.GENE3D); IPR000340 (PFAM); PTHR23172:SF19 (PANTHER); PTHR23172 (PANTHER); IPR014020 (PROSITE_PROFILES); IPR029023 (PROSITE_PROFILES); IPR029021 (SUPERFAMILY)
357	c134735_g1_i1_len_383_path_1_0_382_0	10	chloride calcium family member 3	128	1.86E-12	63.60%	0.322	IPR013642 (PFAM); PTHR10579:SF8 (PANTHER); PTHR10579 (PANTHER); NON_CYTOPLASMIC_DOMAIN (PHOBIUS); CYTOPLASMIC_DOMAIN (PHOBIUS); TRANSMEMBRANE (PHOBIUS); TMhelix (TMHMM)
358	c13487_g1_i1_len_2145_path_101_0_2144_5	6588	calreticulin	715	0	84.20%	0.18	Coil (COILS); IPR001580 (PRINTS); IPR001580 (PFAM); IPR013320 (G3DSA:2.60.120.GENE3D); PTHR11073:SF8 (PANTHER); IPR001580 (PANTHER); IPR018124 (PROSITE_PATTERNS); IPR018124 (PROSITE_PATTERNS); IPR018124 (PROSITE_PATTERNS); IPR018124 (PROSITE_PATTERNS); IPR018124 (PROSITE_PATTERNS); NON_CYTOPLASMIC_DOMAIN (PHOBIUS); CYTOPLASMIC_DOMAIN (PHOBIUS); NON_CYTOPLASMIC_DOMAIN (PHOBIUS); CYTOPLASMIC_DOMAIN (PHOBIUS); TRANSMEMBRANE (PHOBIUS); CYTOPLASMIC_DOMAIN (PHOBIUS); TRANSMEMBRANE (PHOBIUS); NON_CYTOPLASMIC_DOMAIN (PHOBIUS); TRANSMEMBRANE (PHOBIUS); TRANSMEMBRANE (PHOBIUS); TRANSMEMBRANE (PHOBIUS); IPR009033 (SUPERFAMILY); IPR013320 (SUPERFAMILY); TMhelix (TMHMM); TMhelix (TMHMM); TMhelix (TMHMM); TMhelix (TMHMM)
359	c134888_g1_i1_len_290_path_1_0_289_1	10	zinc finger protein ozf-like	97	1.78E-10	59.50%	0.116	IPR015880 (SMART); PF13465 (PFAM); IPR013087 (G3DSA:3.30.160.GENE3D); IPR013087 (G3DSA:3.30.160.GENE3D); PTHR24409:SF14 (PANTHER); PTHR24409 (PANTHER); IPR007087 (PROSITE_PATTERNS); IPR007087 (PROSITE_PROFILES); IPR007087 (PROSITE_PROFILES); SSF57667 (SUPERFAMILY)
360	c134941_g1_i1_len_260_path_238_0_259_1	4	zinc finger protein 649 isoform x1	87	1.71E-09	66.50%	0.151	IPR015880 (SMART); IPR007087 (PFAM); IPR013087 (G3DSA:3.30.160.GENE3D); IPR013087 (G3DSA:3.30.160.GENE3D); PTHR24409 (PANTHER); IPR007087 (PROSITE_PATTERNS); IPR007087 (PROSITE_PROFILES); SSF57667 (SUPERFAMILY)
361	c135229_g1_i1_len_401_path_379_0_400_1	17	latrophilin cirl-like isoform x1	134	3.28E-12	54.00%	0.126	IPR000203 (PFAM); PTHR12011 (PANTHER); IPR000203 (PROSITE_PROFILES)
362	c135283_g1_i1_len_261_path_1_0_260_4	5	zinc finger protein 337-like	87	4.56E-19	62.20%	0.098	IPR015880 (SMART); IPR013087 (G3DSA:3.30.160.GENE3D); IPR013087 (G3DSA:3.30.160.GENE3D); IPR013087 (G3DSA:3.30.160.GENE3D); PF13465 (PFAM); IPR007087 (PFAM); PTHR24409 (PANTHER); IPR007087 (PROSITE_PATTERNS); IPR007087 (PROSITE_PATTERNS); IPR007087 (PROSITE_PATTERNS); IPR007087 (PROSITE_PROFILES); IPR007087 (PROSITE_PROFILES); IPR007087 (PROSITE_PROFILES); SSF57667 (SUPERFAMILY); SSF57667 (SUPERFAMILY)

363	c13540_g2_i1_len_997_path_1173_0_996_5	39	cytoglobin-2-like isoform 1	332	5.92E-46	61.80%	0.137	IPR002336 (PRINTS); IPR000971 (PFAM); IPR012292 (G3DSA:1.10.490.GENE3D); PTHR11442:SF36 (PANTHER); PTHR11442 (PANTHER); NON_CYTOPLASMIC_DOMAIN (PHOBIUS); CYTOPLASMIC_DOMAIN (PHOBIUS); TRANSMEMBRANE (PHOBIUS); TRANSMEMBRANE (PHOBIUS); CYTOPLASMIC_DOMAIN (PHOBIUS); NON_CYTOPLASMIC_DOMAIN (PHOBIUS); TRANSMEMBRANE (PHOBIUS); IPR000971 (PROSITE_PROFILES); IPR009050 (SUPERFAMILY); TMhelix (TMHMM); TMhelix (TMHMM); TMhelix (TMHMM)
364	c135472_g1_i1_len_601_path_1_0_600_1	24	low-density lipoprotein receptor-related protein 2	200	3.63E-96	83.90%	0.1	IPR000742 (SMART); IPR001881 (SMART); IPR002172 (SMART); IPR002172 (PFAM); IPR002172 (G3DSA:4.10.400.GENE3D); IPR026823 (PFAM); G3DSA:2.10.25.10 (GENE3D); IPR011042 (G3DSA:2.120.10.GENE3D); G3DSA:2.10.25.10 (GENE3D); PTHR10529 (PANTHER); IPR000152 (PROSITE_PATTERNS); IPR018097 (PROSITE_PATTERNS); IPR018097 (PROSITE_PATTERNS); IPR023415 (PROSITE_PATTERNS); IPR013032 (PROSITE_PATTERNS); IPR000152 (PROSITE_PATTERNS); IPR000742 (PROSITE_PROFILES); IPR000742 (PROSITE_PROFILES); IPR002172 (PROSITE_PROFILES); SSF63825 (SUPERFAMILY); SSF57196 (SUPERFAMILY); SSF57196 (SUPERFAMILY); IPR002172 (SUPERFAMILY)
365	c136024_g1_i1_len_247_path_225_0_246_3	4	insulin-like growth factor-binding protein complex acid labile subunit	83	1.08E-28	77.60%	0.106	SM00365 (SMART); IPR003591 (SMART); PF13516 (PFAM); IPR001611 (PFAM); G3DSA:3.80.10.10 (GENE3D); PTHR24365 (PANTHER); IPR001611 (PROSITE_PROFILES); IPR001611 (PROSITE_PROFILES); IPR001611 (PROSITE_PROFILES); SSF52058 (SUPERFAMILY)
366	c136260_g1_i1_len_456_path_434_0_455_3	8	laminin subunit alpha-like	152	1.34E-65	78.20%	0.102	PR00011 (PRINTS); IPR002049 (SMART); G3DSA:2.10.25.10 (GENE3D); IPR002049 (PFAM); PTHR10574:SF39 (PANTHER); PTHR10574 (PANTHER); SSF57196 (SUPERFAMILY); SSF57196 (SUPERFAMILY)
367	c136411_g1_i1_len_655_path_1_0_654_4	26	ribosomal protein s6 kinase beta-1-like	218	9.75E-98	82.70%	0.366Y	IPR000961 (SMART); G3DSA:1.10.510.10 (GENE3D); IPR017892 (PFAM); IPR000719 (PFAM); PTHR24351 (PANTHER); PTHR24351:SF64 (PANTHER); IPR000719 (PROSITE_PROFILES); IPR000961 (PROSITE_PROFILES); IPR011009 (SUPERFAMILY)
368	c136560_g1_i1_len_302_path_1_0_301_2	7	er degradation-enhancing alpha-mannosidase-like 1	100	5.52E-46	86.20%	0.255	IPR001382 (G3DSA:1.50.10.GENE3D); IPR001382 (PFAM); IPR001382 (PANTHER); PTHR11742:SF25 (PANTHER); IPR001382 (SUPERFAMILY)
369	c136798_g1_i1_len_247_path_225_0_246_0	2	sortilin-related receptor-like	83	4.53E-12	55.30%	0.102	IPR002172 (SMART); G3DSA:4.10.1220.10 (GENE3D); IPR002172 (PFAM); IPR002172 (G3DSA:4.10.400.GENE3D); PTHR10529 (PANTHER); IPR023415 (PROSITE_PATTERNS); IPR002172 (PROSITE_PROFILES); IPR002172 (SUPERFAMILY); IPR002172 (SUPERFAMILY)
370	c136840_g1_i1_len_273_path_251_0_272_5	6	von willebrand factor type egf and pentraxin domain-containing protein 1	91	3.04E-15	57.00%	0.104	IPR000436 (SMART); G3DSA:2.10.70.10 (GENE3D); IPR000436 (PFAM); G3DSA:2.10.70.10 (GENE3D); PTHR19325:SF343 (PANTHER); PTHR19325 (PANTHER); IPR000436 (PROSITE_PROFILES); IPR000436 (PROSITE_PROFILES); IPR000436 (SUPERFAMILY); IPR000436 (SUPERFAMILY)
371	c136897_g1_i1_len_313_path_1_0_312_1	9	alpha beta hydrolase domain-containing protein 17c-like	104	1.96E-23	85.50%	0.108	PTHR12277:SF52 (PANTHER); PTHR12277 (PANTHER)
372	c136963_g1_i1_len_301_path_279_0_300_2	6	stress response element binding protein	100	2.15E-14	71.40%	0.101	IPR015880 (SMART); IPR013087 (G3DSA:3.30.160.GENE3D); IPR013087 (G3DSA:3.30.160.GENE3D); PF13465 (PFAM); PTHR24409 (PANTHER); IPR007087 (PROSITE_PATTERNS); IPR007087 (PROSITE_PATTERNS); IPR007087 (PROSITE_PROFILES); IPR007087 (PROSITE_PROFILES); SSF57667 (SUPERFAMILY)
373	c137000_g1_i1_len_312_path_290_0_311_5	4	threonine synthase-like 2	104	3.01E-39	76.30%	0.114	G3DSA:3.40.50.1100 (GENE3D); PTHR10314 (PANTHER); IPR027457 (PTHR10314:PANTHER); IPR001926 (SUPERFAMILY)
374	c13708_g1_i1_len_1305_path_533_0_1039_291_1040_1304_3	549	serine protease1 2	435	1.05E-54	56.90%	0.335	IPR001314 (PRINTS); IPR001254 (SMART); IPR001254 (PFAM); G3DSA:2.40.10.10 (GENE3D); G3DSA:2.40.10.10 (GENE3D); PTHR24260 (PANTHER); IPR018114 (PROSITE_PATTERNS); IPR018114 (PROSITE_PATTERNS); CYTOPLASMIC_DOMAIN (PHOBIUS); NON_CYTOPLASMIC_DOMAIN (PHOBIUS); TRANSMEMBRANE (PHOBIUS); IPR001254 (PROSITE_PROFILES); IPR009003 (SUPERFAMILY); TMhelix (TMHMM)
375	c13708_g1_i2_len_1180_path_533_0_1039_1572_1040_1179_4	678	serine protease1 2	393	3.16E-55	56.90%	0.755Y	IPR001314 (PRINTS); IPR001254 (SMART); G3DSA:2.40.10.10 (GENE3D); IPR001254 (PFAM); G3DSA:2.40.10.10 (GENE3D); PTHR24260 (PANTHER); IPR018114 (PROSITE_PATTERNS); IPR018114 (PROSITE_PATTERNS); IPR001254 (PROSITE_PROFILES); SignalP-noTM (SIGNALP_EUK); IPR009003 (SUPERFAMILY)
376	c137305_g1_i1_len_272_path_1_0_271_5	4	neprilysin-2-like isoform x3	90	4.27E-12	63.30%	0.11	IPR008753 (PFAM); IPR024079 (G3DSA:3.40.390.GENE3D); IPR000718 (PANTHER); SSF55486 (SUPERFAMILY)

377	c137420_g1_i1_len_294_path_1_0_293_3	4	zinc finger protein 502	98	4.07E-21	65.20%	0.108	IPR015880 (SMART); IPR013087 (G3DSA:3.30.160.GENE3D); PF13465 (PFAM); PTHR24409 (PANTHER); IPR007087 (PROSITE_PATTERNS); IPR007087 (PROSITE_PATTERNS); IPR007087 (PROSITE_PROFILES); IPR007087 (PROSITE_PROFILES); SSF57667 (SUPERFAMILY)
378	c13753_g1_i1_len_1724_path_1_0_1723_5	1973	leucine rich repeat protein	574	2.54E-29	50.60%	0.593Y	IPR003591 (SMART); SM00364 (SMART); IPR001611 (PFAM); G3DSA:3.80.10.10 (GENE3D); IPR001611 (PFAM); G3DSA:3.80.10.10 (GENE3D); G3DSA:3.80.10.10 (GENE3D); PTHR24365 (PANTHER); SIGNAL_PEPTIDE_N_REGION (PHOBIUS); SIGNAL_PEPTIDE_H_REGION (PHOBIUS); SIGNAL_PEPTIDE (PHOBIUS); SIGNAL_PEPTIDE_C_REGION (PHOBIUS); NON_CYTOPLASMIC_DOMAIN (PHOBIUS); TRANSMEMBRANE (PHOBIUS); CYTOPLASMIC_DOMAIN (PHOBIUS); IPR001611 (PROSITE_PROFILES); IPR001611 (PROSITE_PROFILES); IPR001611 (PROSITE_PROFILES); IPR001611 (PROSITE_PROFILES); IPR001611 (PROSITE_PROFILES); IPR001611 (PROSITE_PROFILES); IPR001611 (PROSITE_PROFILES); IPR001611 (PROSITE_PROFILES); IPR001611 (PROSITE_PROFILES); IPR001611 (PROSITE_PROFILES); IPR001611 (PROSITE_PROFILES); IPR001611 (PROSITE_PROFILES); IPR001611 (PROSITE_PROFILES); IPR001611 (PROSITE_PROFILES); SignalP-noTM (SIGNALP_EUK); SSF52058 (SUPERFAMILY)
379	c137823_g1_i1_len_286_path_1_0_285_1	4	xanthine dehydrogenase	95	1.54E-21	69.50%	0.103	IPR000674 (SMART); IPR000674 (PFAM); IPR000674 (G3DSA:3.90.1170.GENE3D); PTHR11908:SF32 (PANTHER); PTHR11908 (PANTHER); IPR000674 (SUPERFAMILY)
380	c13812_g2_i1_len_678_path_656_0_677_3	15	low quality protein: von willebrand factor type egf and pentraxin domain-containing protein 1	226	3.17E-41	51.00%	0.104	IPR000436 (SMART); G3DSA:2.10.70.10 (GENE3D); G3DSA:2.10.70.10 (GENE3D); G3DSA:2.10.70.10 (GENE3D); G3DSA:2.10.70.10 (GENE3D); IPR000436 (PFAM); PTHR19325 (PANTHER); IPR000436 (PROSITE_PROFILES); IPR000436 (PROSITE_PROFILES); IPR000436 (PROSITE_PROFILES); IPR000436 (PROSITE_PROFILES); IPR000436 (SUPERFAMILY); IPR000436 (SUPERFAMILY); IPR000436 (SUPERFAMILY); IPR000436 (SUPERFAMILY)
381	c138240_g1_i1_len_348_path_1_0_347_1	6	glucose dehydrogenase	116	3.40E-43	75.80%	0.1	G3DSA:3.50.50.60 (GENE3D); IPR000172 (PFAM); PTHR11552 (PANTHER); PTHR11552:SF90 (PANTHER); IPR000172 (PROSITE_PATTERNS); SSF51905 (SUPERFAMILY)
382	c13827_g1_i1_len_692_path_841_0_598_86_599_691_2	27	lysosomal pro-x carboxypeptidase	230	3.43E-89	70.40%	0.244	IPR008758 (PFAM); PTHR11010:SF11 (PANTHER); IPR008758 (PANTHER)
383	c13827_g1_i2_len_178_path_1_0_84_86_85_177_1	1	lysosomal pro-x carboxypeptidase-like	59	3.05E-12	79.60%	0.107	PTHR11010:SF11 (PANTHER); IPR008758 (PANTHER)
384	c138410_g1_i1_len_385_path_363_0_384_2	6	prenylcysteine oxidase-like	128	1.15E-35	70.10%	0.122	G3DSA:3.50.50.60 (GENE3D); PF13450 (PFAM); IPR017046 (PANTHER); SSF51905 (SUPERFAMILY)
385	c138415_g1_i1_len_406_path_1_0_405_1	9	low-density lipoprotein	135	5.39E-52	69.00%	0.104	IPR002172 (PRINTS); IPR002172 (SMART); IPR002172 (PFAM); IPR002172 (G3DSA:4.10.400.GENE3D); IPR002172 (G3DSA:4.10.400.GENE3D); IPR002172 (G3DSA:4.10.400.GENE3D); IPR002172 (G3DSA:4.10.400.GENE3D); PTHR10529 (PANTHER); IPR023415 (PROSITE_PATTERNS); IPR023415 (PROSITE_PATTERNS); IPR023415 (PROSITE_PATTERNS); IPR002172 (PROSITE_PROFILES); IPR002172 (PROSITE_PROFILES); IPR002172 (SUPERFAMILY); IPR002172 (SUPERFAMILY); IPR002172 (SUPERFAMILY)
386	c138485_g1_i1_len_427_path_1_0_426_3	6	chaperone protein	143	6.22E-41	82.60%	0.102	IPR013126 (PRINTS); G3DSA:3.30.30.30 (GENE3D); G3DSA:3.30.420.40 (GENE3D); IPR013126 (PFAM); PTHR19375 (PANTHER); PTHR19375:SF173 (PANTHER); IPR018181 (PROSITE_PATTERNS); SSF53067 (SUPERFAMILY)
387	c13849_g1_i1_len_905_path_883_0_904_0	90	gamma-interferon-inducible lysosomal thiol reductase	302	1.60E-43	51.30%	0.242	IPR004911 (PFAM); IPR004911 (PANTHER); PTHR13234:SF8 (PANTHER)
388	c13858_g1_i1_len_630_path_1_0_237_1042_238_629_3	9	metabotropic gaba-b receptor subtype isoform b	210	1.33E-26	83.40%	0.108	G3DSA:3.40.50.2300 (GENE3D); IPR002455 (PANTHER); PTHR10519:SF3 (PANTHER); IPR028082 (SUPERFAMILY)
389	c13858_g1_i1_len_630_path_1_0_237_1042_238_629_4	9	gamma-aminobutyric acid type b receptor subunit 1 isoform x3	210	3.24E-74	90.40%	0.11	PR01176 (PRINTS); IPR001828 (PFAM); G3DSA:3.40.50.2300 (GENE3D); IPR002455 (PANTHER); PTHR10519:SF3 (PANTHER); IPR028082 (SUPERFAMILY)
390	c13858_g1_i2_len_336_path_1_0_237_239_238_335_3	12	gamma-aminobutyric acid type b receptor subunit 1-like	112	2.98E-44	81.70%	0.117	IPR002456 (PRINTS); IPR001828 (PFAM); G3DSA:3.40.50.2300 (GENE3D); IPR002455 (PANTHER); PTHR10519:SF3 (PANTHER); IPR028082 (SUPERFAMILY)
391	c13881_g1_i1_len_494_path_472_0_276_749_277_493_5	22	peptidyl-prolyl cis-trans isomerase h	164	9.07E-57	96.10%	0.1	IPR002130 (PRINTS); IPR029000 (G3DSA:2.40.100.GENE3D); IPR002130 (PFAM); IPR024936 (PANTHER); PTHR11071:SF58 (PANTHER); TRANSMEMBRANE (PHOBIUS); NON_CYTOPLASMIC_DOMAIN (PHOBIUS); CYTOPLASMIC_DOMAIN (PHOBIUS); IPR002130 (PROSITE_PROFILES); IPR029000 (SUPERFAMILY); TMhelix (TMHMM)

392	c13885_g1_i1_len_2216_path_2194_0_2215_1	145	afg3-like protein 2	739	0	92.90%	0.107	IPR003593 (SMART); IPR003959 (PFAM); IPR005936 (TIGRFAM); IPR027417 (G3DSA:3.40.50.GENE3D); IPR000642 (PFAM); G3DSA:1.10.8.60 (GENE3D); PTHR23076:SF38 (PANTHER); PTHR23076 (PANTHER); IPR003960 (PROSITE_PATTERNS); NON_CYTOPLASMIC_DOMAIN (PHOBIUS); CYTOPLASMIC_DOMAIN (PHOBIUS); TRANSMEMBRANE (PHOBIUS); NON_CYTOPLASMIC_DOMAIN (PHOBIUS); TRANSMEMBRANE (PHOBIUS); IPR005936 (HAMAP); SSF140990 (SUPERFAMILY); IPR027417 (SUPERFAMILY); TMhelix (TMHMM)
393	c138880_g1_i1_len_346_path_1_0_345_4	16	---NA---	115			0.104	no IPS match
394	c139001_g1_i1_len_267_path_245_0_266_0	4	---NA---	89			0.106	IPR001190 (PFAM); IPR017448 (G3DSA:3.10.250.GENE3D); IPR001190 (PROSITE_PROFILES); IPR017448 (SUPERFAMILY)
395	c13927_g2_i1_len_612_path_590_0_611_5	14	keratin-associated protein 5-	204	2.88E-56	56.10%	0.118	IPR000742 (SMART); G3DSA:2.170.300.10 (GENE3D); IPR013032 (PFAM); IPR000742 (PFAM); G3DSA:2.10.25.10 (GENE3D); PTHR24838 (PANTHER); IPR013032 (PROSITE_PATTERNS); IPR013032 (PROSITE_PATTERNS); IPR013032 (PROSITE_PATTERNS); IPR013032 (PROSITE_PATTERNS); IPR013032 (PROSITE_PATTERNS); IPR013032 (PROSITE_PATTERNS); IPR013032 (PROSITE_PATTERNS); IPR013032 (PROSITE_PATTERNS); IPR000742 (PROSITE_PROFILES); IPR000742 (PROSITE_PROFILES); IPR000742 (PROSITE_PROFILES); SSF57196 (SUPERFAMILY); SSF57196 (SUPERFAMILY)
396	c139353_g1_i1_len_269_path_1_0_268_2	4	#NAME?	89	4.95E-47	91.50%	0.141	IPR001830 (PFAM); G3DSA:3.40.50.2000 (GENE3D); PTHR10788 (PANTHER); PTHR10788:SF6 (PANTHER); SSF53756 (SUPERFAMILY)
397	c13937_g1_i1_len_879_path_1_0_878_3	52	probable isocitrate dehydrogenase	293	2.95E-162	87.50%	0.104	IPR024084 (G3DSA:3.40.718.GENE3D); IPR024084 (PFAM); IPR004434 (TIGRFAM); PTHR11835:SF39 (PANTHER); IPR001804 (PANTHER); IPR019818 (PROSITE_PATTERNS); SSF53659 (SUPERFAMILY)
398	c139468_g1_i1_len_554_path_532_0_553_5	7	wd repeat domain-containing protein 83	184	9.24E-40	84.60%	0.33	IPR001680 (SMART); IPR001680 (PFAM); IPR015943 (G3DSA:2.130.10.GENE3D); PTHR22842 (PANTHER); IPR019775 (PROSITE_PATTERNS); IPR001680 (PROSITE_PROFILES); IPR017986 (PROSITE_PROFILES); IPR001680 (PROSITE_PROFILES); IPR017986 (SUPERFAMILY)
399	c139535_g1_i1_len_314_path_1_0_313_3	4	protease inhibitors-like isoform x2	105	2.29E-11	53.80%	0.104	IPR008037 (PFAM); IPR020862 (PROSITE_PROFILES); IPR020862 (PROSITE_PROFILES); IPR008037 (SUPERFAMILY); IPR008037 (SUPERFAMILY)
400	c13967_g1_i1_len_408_path_1_0_407_5	6	fibrillin-2-like	136	2.41E-61	78.90%	0.101	IPR000742 (SMART); IPR001881 (SMART); IPR017878 (G3DSA:3.90.290.GENE3D); G3DSA:2.10.25.10 (GENE3D); IPR017878 (PFAM); G3DSA:2.10.25.10 (GENE3D); IPR001881 (PFAM); PTHR24039:SF20 (PANTHER); IPR011398 (PANTHER); IPR018097 (PROSITE_PATTERNS); IPR013032 (PROSITE_PATTERNS); IPR000152 (PROSITE_PATTERNS); IPR013032 (PROSITE_PATTERNS); IPR017878 (PROSITE_PROFILES); IPR000742 (PROSITE_PROFILES); IPR017878 (SUPERFAMILY); SSF57196 (SUPERFAMILY)
401	c13967_g2_i1_len_1580_path_2328_0_1579_0	83	fibrillin-2-like	527	1.02E-163	64.40%	0.117	IPR000742 (SMART); IPR001881 (SMART); G3DSA:2.10.25.10 (GENE3D); G3DSA:2.10.25.10 (GENE3D); G3DSA:2.10.25.10 (GENE3D); G3DSA:2.10.25.10 (GENE3D); IPR001881 (PFAM); PTHR24039:SF20 (PANTHER); IPR011398 (PANTHER); IPR013032 (PROSITE_PATTERNS); IPR000152 (PROSITE_PATTERNS); IPR013032 (PROSITE_PATTERNS); IPR018097 (PROSITE_PATTERNS); IPR013032 (PROSITE_PATTERNS); IPR013032 (PROSITE_PATTERNS); IPR000152 (PROSITE_PATTERNS); IPR000152 (PROSITE_PATTERNS); IPR013032 (PROSITE_PATTERNS); IPR018097 (PROSITE_PATTERNS); IPR013032 (PROSITE_PATTERNS); IPR000152 (PROSITE_PATTERNS); IPR013032 (PROSITE_PATTERNS); IPR000152 (PROSITE_PATTERNS); IPR018097 (PROSITE_PATTERNS); IPR018097 (PROSITE_PATTERNS); IPR018097 (PROSITE_PATTERNS); IPR000152 (PROSITE_PATTERNS); IPR000742 (PROSITE_PROFILES); IPR000742 (PROSITE_PROFILES); IPR000742 (PROSITE_PROFILES); IPR000742 (PROSITE_PROFILES); IPR000742 (PROSITE_PROFILES); IPR000742 (PROSITE_PROFILES); SSF57196 (SUPERFAMILY); IPR009030 (SUPERFAMILY); IPR009030 (SUPERFAMILY)
402	c13970_g1_i1_len_150_path_1_0_149_3	2	b-cell lymphoma leukemia 11a-like	50	8.11E-14	78.60%	0.105	IPR013087 (G3DSA:3.30.160.GENE3D); IPR013087 (G3DSA:3.30.160.GENE3D); PF13465 (PFAM); PTHR24409 (PANTHER); IPR007087 (PROSITE_PATTERNS); IPR007087 (PROSITE_PROFILES); SSF57667 (SUPERFAMILY)

403	c13972_g1_i1_len_1112_path_1_0_431_433_432_1111_5	39	zinc finger protein 501-like	370	1.82E-21	47.70%	0.1	IPR015880 (SMART); PF13894 (PFAM); IPR007087 (PFAM); IPR013087 (G3DSA:3.30.160.GENE3D); IPR013087 (G3DSA:3.30.160.GENE3D); IPR013087 (G3DSA:3.30.160.GENE3D); PTHR24409 (PANTHER); IPR007087 (PROSITE_PATTERNS); IPR007087 (PROSITE_PATTERNS); IPR007087 (PROSITE_PATTERNS); IPR007087 (PROSITE_PROFILES); IPR007087 (PROSITE_PROFILES); IPR007087 (PROSITE_PROFILES); IPR007087 (PROSITE_PROFILES); SSF57667 (SUPERFAMILY); SSF57667 (SUPERFAMILY); SSF57667 (SUPERFAMILY)
404	c140065_g1_i1_len_464_path_1_0_463_3	7	membrane metallo-endopeptidase-like 1-like	155	3.39E-42	68.20%	0.114	IPR008753 (PFAM); G3DSA:1.10.1380.10 (GENE3D); PTHR11733:SF113 (PANTHER); IPR000718 (PANTHER); SSF55486 (SUPERFAMILY)
405	c14022_g1_i1_len_1074_path_1_0_92_94_93_1073_0	56	aael003561- partial	358	1.95E-25	52.80%	0.178	IPR001211 (PRINTS); IPR016090 (SMART); IPR016090 (G3DSA:1.20.90.GENE3D); IPR016090 (PFAM); IPR001211 (PANTHER); PTHR11716:SF45 (PANTHER); IPR013090 (PROSITE_PATTERNS); IPR013090 (PROSITE_PATTERNS); TRANSMEMBRANE (PHOBIUS); CYTOPLASMIC_DOMAIN (PHOBIUS); TRANSMEMBRANE (PHOBIUS); NON_CYTOPLASMIC_DOMAIN (PHOBIUS); CYTOPLASMIC_DOMAIN (PHOBIUS); NON_CYTOPLASMIC_DOMAIN (PHOBIUS); TRANSMEMBRANE (PHOBIUS); IPR016090 (SUPERFAMILY); TMhelix (TMHMM)
406	c14022_g1_i2_len_1059_path_2103_0_77_94_78_1058_0	42	aael003561- partial	353	1.83E-25	52.60%	0.206	IPR001211 (PRINTS); IPR016090 (SMART); IPR016090 (PFAM); IPR016090 (G3DSA:1.20.90.GENE3D); IPR001211 (PANTHER); IPR013090 (PROSITE_PATTERNS); IPR013090 (PROSITE_PATTERNS); TRANSMEMBRANE (PHOBIUS); NON_CYTOPLASMIC_DOMAIN (PHOBIUS); CYTOPLASMIC_DOMAIN (PHOBIUS); CYTOPLASMIC_DOMAIN (PHOBIUS); TRANSMEMBRANE (PHOBIUS); TRANSMEMBRANE (PHOBIUS); NON_CYTOPLASMIC_DOMAIN (PHOBIUS); IPR016090 (SUPERFAMILY); TMhelix (TMHMM)
407	c140284_g1_i1_len_141_path_1_0_140_1	0	myosin heavy chain partial	47	2.98E-16	97.40%	0.105	Coil (COILS); IPR002928 (PFAM); PTHR13140:SF364 (PANTHER); PTHR13140 (PANTHER)
408	c140392_g1_i1_len_289_path_1_0_288_1	6	serine proteinase stubble-like	96	7.99E-21	71.20%	0.13	G3DSA:2.40.10.10 (GENE3D); IPR001254 (PFAM); PTHR24275 (PANTHER); IPR018114 (PROSITE_PATTERNS); IPR001254 (PROSITE_PROFILES); IPR009003 (SUPERFAMILY)
409	c14080_g2_i1_len_1186_path_1305_0_1185_5	61	ankyrin repeat domain-containing protein partial	395	1.16E-48	75.60%	0.112	IPR002110 (SMART); IPR020683 (G3DSA:1.25.40.GENE3D); IPR002110 (PFAM); IPR020683 (PFAM); PTHR24149 (PANTHER); IPR002110 (PROSITE_PROFILES); IPR020683 (PROSITE_PROFILES); IPR002110 (PROSITE_PROFILES); IPR002110 (PROSITE_PROFILES); IPR020683 (SUPERFAMILY)
410	c140921_g1_i1_len_270_path_248_0_269_4	4	cathepsin b	90	2.02E-20	72.20%	0.136	IPR000668 (PFAM); G3DSA:3.90.70.10 (GENE3D); PTHR12411:SF15 (PANTHER); IPR013128 (PANTHER); SSF54001 (SUPERFAMILY)
411	c141015_g1_i1_len_394_path_1_0_393_2	5	low-density lipoprotein receptor-related protein 4	131	2.06E-06	62.80%	0.109	IPR002172 (SMART); IPR002172 (PFAM); IPR002172 (G3DSA:4.10.400.GENE3D); IPR002172 (PROSITE_PROFILES); IPR002172 (SUPERFAMILY)
412	c14123_g1_i1_len_374_path_470_0_310_962_311_373_4	15	urokinase-type plasminogen activator	125	1.88E-16	72.70%	0.132	IPR001254 (PFAM); G3DSA:2.40.10.10 (GENE3D); PTHR24265 (PANTHER); IPR018114 (PROSITE_PATTERNS); IPR001254 (PROSITE_PROFILES); IPR009003 (SUPERFAMILY)
413	c14123_g1_i2_len_492_path_470_0_310_781_311_491_5	19	urokinase-type plasminogen activator	164	1.84E-12	78.00%	0.167	IPR001254 (PFAM); G3DSA:2.40.10.10 (GENE3D); PTHR24265 (PANTHER); IPR001254 (PROSITE_PROFILES); IPR009003 (SUPERFAMILY)
414	c141445_g1_i1_len_180_path_1_0_179_5	3	plexin-b-like isoform x5	60	5.99E-27	91.30%	0.111	IPR002909 (PFAM); IPR013783 (G3DSA:2.60.40.GENE3D); PTHR22625:SF31 (PANTHER); PTHR22625 (PANTHER); IPR014756 (SUPERFAMILY)
415	c141926_g1_i1_len_320_path_298_0_319_0	4	von willebrand factor type egf and pentraxin domain-containing protein 1	107	9.12E-10	56.00%	0.113	G3DSA:2.10.70.10 (GENE3D); IPR000436 (PFAM); IPR000436 (PROSITE_PROFILES); IPR000436 (SUPERFAMILY)
416	c142041_g1_i1_len_387_path_365_0_386_2	6	protein dachsous-like	129	1.05E-42	72.20%	0.119	IPR002126 (G3DSA:2.60.40.GENE3D); IPR002126 (PFAM); IPR002126 (G3DSA:2.60.40.GENE3D); PTHR24027 (PANTHER); PTHR24027:SF256 (PANTHER); IPR002126 (PROSITE_PROFILES); IPR002126 (PROSITE_PROFILES); IPR015919 (SUPERFAMILY); IPR015919 (SUPERFAMILY)
417	c142423_g1_i1_len_336_path_1_0_335_3	5	acid sphingomyelinase-like phosphodiesterase 3a	112	7.63E-20	60.00%	0.257	IPR029052 (G3DSA:3.60.21.GENE3D); PTHR10340 (PANTHER); PTHR10340:SF14 (PANTHER); SIGNAL_PEPTIDE_N_REGION (PHOBIUS); NON_CYTOPLASMIC_DOMAIN (PHOBIUS); SIGNAL_PEPTIDE_C_REGION (PHOBIUS); SIGNAL_PEPTIDE_H_REGION (PHOBIUS); SIGNAL_PEPTIDE (PHOBIUS); TMhelix (TMHMM)

418	c14323_g1_i1_len_926_path_1_0_925_4	44	tropinone reductase 2-like	309	8.21E-137	83.50%	0.247	IPR002198 (PRINTS); IPR002347 (PRINTS); IPR002198 (PFAM); IPR016040 (G3DSA:3.40.50.GENE3D); PTHR24322 (PANTHER); PTHR24322:SF111 (PANTHER); IPR020904 (PROSITE_PATTERNS); NON_CYTOPLASMIC_DOMAIN (PHOBIUS); TRANSMEMBRANE (PHOBIUS); CYTOPLASMIC_DOMAIN (PHOBIUS); NON_CYTOPLASMIC_DOMAIN (PHOBIUS); TRANSMEMBRANE (PHOBIUS); SSF51735 (SUPERFAMILY); TMhelix (TMHMM); TMhelix (TMHMM)
419	c14329_g1_i1_len_1588_path_1556_0_171_3134_172_181_1728_182_1587_1	60	ww domain-containing oxidoreductase	529	3.16E-167	74.70%	0.131	Coil (COILS); IPR002347 (PRINTS); IPR001202 (SMART); IPR002198 (PFAM); IPR016040 (G3DSA:3.40.50.GENE3D); G3DSA:2.20.70.10 (GENE3D); PTHR24316:SF115 (PANTHER); PTHR24316 (PANTHER); IPR001202 (PROSITE_PROFILES); IPR001202 (SUPERFAMILY); SSF51735 (SUPERFAMILY)
420	c143471_g1_i1_len_215_path_1_0_214_0	3	blastula protease 10-like	72	6.98E-25	74.40%	0.102	IPR001506 (PRINTS); IPR001506 (PFAM); IPR024079 (G3DSA:3.40.390.GENE3D); PTHR10127 (PANTHER); SSF55486 (SUPERFAMILY)
421	c14408_g1_i1_len_1064_path_1042_0_268_1311_269_1063_2	40	arylsulfatase a-like	354	1.43E-114	71.10%	0.294	IPR017849 (G3DSA:3.40.720.GENE3D); IPR000917 (PFAM); PTHR10342 (PANTHER); PTHR10342:SF22 (PANTHER); IPR024607 (PROSITE_PATTERNS); NON_CYTOPLASMIC_DOMAIN (PHOBIUS); CYTOPLASMIC_DOMAIN (PHOBIUS); TRANSMEMBRANE (PHOBIUS); IPR017850 (SUPERFAMILY); TMhelix (TMHMM)
422	c14408_g1_i2_len_487_path_1042_0_268_2106_269_486_2	17	arylsulfatase a-like	162	8.42E-11	74.80%	0.294	IPR000917 (PFAM); IPR017849 (G3DSA:3.40.720.GENE3D); PTHR10342 (PANTHER); PTHR10342:SF27 (PANTHER); CYTOPLASMIC_DOMAIN (PHOBIUS); NON_CYTOPLASMIC_DOMAIN (PHOBIUS); TRANSMEMBRANE (PHOBIUS); CYTOPLASMIC_DOMAIN (PHOBIUS); TRANSMEMBRANE (PHOBIUS); TRANSMEMBRANE (PHOBIUS); NON_CYTOPLASMIC_DOMAIN (PHOBIUS); IPR017850 (SUPERFAMILY); TMhelix (TMHMM); TMhelix (TMHMM); TMhelix (TMHMM)
423	c144328_g1_i1_len_147_path_125_0_146_0	2	achain structural basis for dscam isoform specificity	49	5.07E-24	97.00%	0.099	PTHR10489:SF567 (PANTHER); PTHR10489 (PANTHER)
424	c144399_g1_i1_len_165_path_1_0_164_4	1	low-density lipoprotein	55	3.06E-14	73.20%	0.126	IPR001881 (SMART); IPR001881 (PFAM); G3DSA:2.10.25.10 (GENE3D); PTHR10529 (PANTHER); PTHR10529:SF210 (PANTHER); IPR013032 (PROSITE_PATTERNS); IPR018097 (PROSITE_PATTERNS); IPR000152 (PROSITE_PATTERNS); SSF57196 (SUPERFAMILY)
425	c14479_g1_i1_len_1311_path_1_0_208_210_209_504_506_505_1310_2	210	protein spaetzle	437	2.21E-28	54.20%	0.245	PTHR23199 (PANTHER); IPR029034 (SUPERFAMILY)
426	c14479_g1_i2_len_1015_path_1_0_208_506_209_1014_0	122	spaetzle 1-2 isoform x1	339	1.61E-21	54.40%	0.471Y	PTHR23199 (PANTHER); SignalP-noTM (SIGNALP_EUK); IPR029034 (SUPERFAMILY)
427	c14493_g1_i1_len_837_path_1_0_836_4	43	alpha-mannosidase 2	279	9.21E-139	78.50%	0.102	IPR015341 (SMART); IPR015341 (PFAM); IPR000602 (PFAM); IPR027291 (G3DSA:3.20.110.GENE3D); IPR015341 (G3DSA:1.20.1270.GENE3D); PTHR11607:SF22 (PANTHER); PTHR11607 (PANTHER); IPR011330 (SUPERFAMILY); IPR028995 (SUPERFAMILY)
428	c14493_g2_i1_len_1087_path_1629_0_1086_1	40	alpha-mannosidase 2	362	7.74E-167	73.80%	0.370Y	IPR000602 (PFAM); IPR027291 (G3DSA:3.20.110.GENE3D); PTHR11607 (PANTHER); PTHR11607:SF22 (PANTHER); CYTOPLASMIC_DOMAIN (PHOBIUS); TRANSMEMBRANE (PHOBIUS); NON_CYTOPLASMIC_DOMAIN (PHOBIUS); IPR011330 (SUPERFAMILY); TMhelix (TMHMM)
429	c145096_g1_i1_len_363_path_341_0_362_1	6	zinc finger protein 678-like	121	1.09E-39	69.30%	0.105	IPR015880 (SMART); IPR013087 (G3DSA:3.30.160.GENE3D); IPR013087 (G3DSA:3.30.160.GENE3D); IPR013087 (G3DSA:3.30.160.GENE3D); PF13465 (PFAM); PTHR24409 (PANTHER); IPR007087 (PROSITE_PATTERNS); IPR007087 (PROSITE_PATTERNS); IPR007087 (PROSITE_PATTERNS); IPR007087 (PROSITE_PROFILES); IPR007087 (PROSITE_PROFILES); IPR007087 (PROSITE_PROFILES); IPR007087 (PROSITE_PROFILES); SSF57667 (SUPERFAMILY); SSF57667 (SUPERFAMILY); SSF57667 (SUPERFAMILY)
430	c145103_g1_i1_len_1155_path_1133_0_1154_0	29	85 88 kda calcium-independent phospholipase a2 isoform x1	385	4.92E-137	68.80%	0.178	IPR002110 (SMART); IPR020683 (G3DSA:1.25.40.GENE3D); IPR002641 (PFAM); IPR020683 (PFAM); G3DSA:3.40.1090.10 (GENE3D); PTHR24139 (PANTHER); PTHR24139:SF33 (PANTHER); IPR002110 (PROSITE_PROFILES); IPR002110 (PROSITE_PROFILES); IPR020683 (PROSITE_PROFILES); IPR020683 (SUPERFAMILY); IPR016035 (SUPERFAMILY)
431	c145113_g1_i1_len_2339_path_1_0_2338_5	284	scabrous protein	779	6.61E-118	51.80%	0.096	Coil (COILS); Coil (COILS); Coil (COILS); Coil (COILS); Coil (COILS); Coil (COILS); Coil (COILS); IPR002181 (SMART); IPR014716 (G3DSA:3.90.215.GENE3D); IPR002181 (PFAM); IPR014715 (G3DSA:4.10.530.GENE3D); PTHR19143 (PANTHER); IPR002181 (PROSITE_PROFILES); IPR002181 (SUPERFAMILY)

432	c145223_g1_i1_len_975_path_953_0_974_2	39	cgmp-dependent protein isozyme 2 forms cd4 t1 t3a t3b-like isoform x3	325	0	91.50%	0.1	IPR002290 (SMART); IPR000961 (SMART); G3DSA:1.10.510.10 (GENE3D); G3DSA:3.30.200.20 (GENE3D); IPR000719 (PFAM); PTHR24353:SF65 (PANTHER); PTHR24353 (PANTHER); IPR008271 (PROSITE_PATTERNS); IPR000719 (PROSITE_PROFILES); IPR000961 (PROSITE_PROFILES); IPR011009 (SUPERFAMILY)
433	c145247_g1_i1_len_1424_path_1_0_1423_4	63	limulus clotting factor partial	475	1.50E-68	56.90%	0.115	IPR001314 (PRINTS); IPR001881 (SMART); IPR001254 (SMART); G3DSA:2.40.10.10 (GENE3D); G3DSA:2.10.25.10 (GENE3D); IPR001254 (PFAM); G3DSA:2.40.10.10 (GENE3D); PTHR24260 (PANTHER); IPR018114 (PROSITE_PATTERNS); IPR018114 (PROSITE_PATTERNS); IPR013032 (PROSITE_PATTERNS); IPR001254 (PROSITE_PROFILES); IPR000436 (PROSITE_PROFILES); SSF57196 (SUPERFAMILY); IPR009003 (SUPERFAMILY)
434	c145250_g1_i1_len_1684_path_1662_0_1683_0	584	calumenin	562	1.24E-155	79.90%	0.137	IPR002048 (SMART); IPR011992 (PFAM); IPR002048 (PFAM); IPR011992 (G3DSA:1.10.238.GENE3D); PTHR10827 (PANTHER); IPR027239 (PTHR10827:PANTHER); IPR018247 (PROSITE_PATTERNS); IPR018247 (PROSITE_PATTERNS); IPR018247 (PROSITE_PATTERNS); IPR018247 (PROSITE_PATTERNS); IPR018247 (PROSITE_PATTERNS); CYTOPLASMIC_DOMAIN (PHOBIUS); NON_CYTOPLASMIC_DOMAIN (PHOBIUS); TRANSMEMBRANE (PHOBIUS); TRANSMEMBRANE (PHOBIUS); TRANSMEMBRANE (PHOBIUS); NON_CYTOPLASMIC_DOMAIN (PHOBIUS); CYTOPLASMIC_DOMAIN (PHOBIUS); IPR002048 (PROSITE_PROFILES); IPR002048 (PROSITE_PROFILES); IPR002048 (PROSITE_PROFILES); IPR002048 (PROSITE_PROFILES); IPR002048 (PROSITE_PROFILES); IPR002048 (PROSITE_PROFILES); SSF47473 (SUPERFAMILY); SSF47473 (SUPERFAMILY); TMhelix (TMHMM); TMhelix (TMHMM); TMhelix (TMHMM)
435	c14529_g1_i1_len_604_path_582_0_125_708_126_603_5	19	lysosomal pro-x carboxypeptidase-like	201	1.02E-18	77.90%	0.208	IPR008758 (PFAM); IPR008758 (PANTHER); PTHR11010:SF11 (PANTHER); CYTOPLASMIC_DOMAIN (PHOBIUS); NON_CYTOPLASMIC_DOMAIN (PHOBIUS); TRANSMEMBRANE (PHOBIUS); TMhelix (TMHMM)
436	c145309_g1_i1_len_1107_path_1085_0_1106_1	62	adp-ribosylation factor-related protein 1	369	6.82E-125	94.60%	0.103	IPR001806 (PRINTS); IPR003579 (SMART); IPR006687 (SMART); IPR024156 (SMART); IPR006689 (PFAM); IPR005225 (TIGRFAM); IPR027417 (G3DSA:3.40.50.GENE3D); PTHR11711 (PANTHER); PTHR11711:SF19 (PANTHER); IPR024156 (PROSITE_PROFILES); IPR027417 (SUPERFAMILY); TMhelix (TMHMM)
437	c145327_g1_i1_len_2269_path_2247_0_2268_0	405	aspartate--trna cytoplasmic	757	0	85.30%	0.231	Coil (COILS); IPR002312 (PRINTS); G3DSA:3.30.930.10 (GENE3D); IPR004523 (TIGRFAM); IPR004364 (PFAM); IPR012340 (G3DSA:2.40.50.GENE3D); IPR004365 (PFAM); IPR018150 (PANTHER); IPR004523 (PTHR22594:PANTHER); NON_CYTOPLASMIC_DOMAIN (PHOBIUS); NON_CYTOPLASMIC_DOMAIN (PHOBIUS); CYTOPLASMIC_DOMAIN (PHOBIUS); CYTOPLASMIC_DOMAIN (PHOBIUS); TRANSMEMBRANE (PHOBIUS); TRANSMEMBRANE (PHOBIUS); TRANSMEMBRANE (PHOBIUS); IPR006195 (PROSITE_PROFILES); SSF55681 (SUPERFAMILY); IPR012340 (SUPERFAMILY)
439	c14541_g2_i1_len_671_path_899_0_670_1	20	26s protease regulatory subunit 6a-b	224	5.31E-155	98.20%	0.16	IPR003593 (SMART); IPR027417 (G3DSA:3.40.50.GENE3D); IPR003959 (PFAM); PTHR23073 (PANTHER); PTHR23073:SF7 (PANTHER); IPR027417 (SUPERFAMILY)
438	c145419_g1_i1_len_582_path_560_0_581_0	22	achain structural determinants for improved thermal stability of designed ankyrin repeat proteins with a redesigned c- capping	194	2.49E-22	53.70%	0.11	IPR002110 (SMART); IPR020683 (PFAM); IPR020683 (G3DSA:1.25.40.GENE3D); PTHR24198 (PANTHER); IPR002110 (PROSITE_PROFILES); IPR020683 (PROSITE_PROFILES); IPR002110 (PROSITE_PROFILES); IPR002110 (PROSITE_PROFILES); IPR002110 (PROSITE_PROFILES); IPR020683 (SUPERFAMILY)
440	c14555_g1_i1_len_642_path_2469_0_15_1866_16_641_4	20	leucine-rich repeat-containing protein 70 isoform x1	214	1.68E-15	48.20%	0.111	IPR003591 (SMART); IPR001611 (PFAM); G3DSA:3.80.10.10 (GENE3D); IPR001611 (PFAM); PTHR24369 (PANTHER); SSF52058 (SUPERFAMILY)
441	c14555_g1_i2_len_1257_path_1235_0_630_1866_631_1256_4	70	leucine-rich repeat-containing protein 70 isoform x1	419	5.82E-15	50.30%	0.111	IPR003591 (SMART); IPR001611 (PFAM); G3DSA:3.80.10.10 (GENE3D); IPR001611 (PFAM); PTHR24369 (PANTHER); NON_CYTOPLASMIC_DOMAIN (PHOBIUS); TRANSMEMBRANE (PHOBIUS); CYTOPLASMIC_DOMAIN (PHOBIUS); NON_CYTOPLASMIC_DOMAIN (PHOBIUS); CYTOPLASMIC_DOMAIN (PHOBIUS); TRANSMEMBRANE (PHOBIUS); TRANSMEMBRANE (PHOBIUS); SSF52058 (SUPERFAMILY); TMhelix (TMHMM); TMhelix (TMHMM); TMhelix (TMHMM)

443	c14560_g1_i1_len_892_path_870_0_751_1622_752_771_1642_772_891_1	465	pacifastin light chain	297	1.23E-27	44.60%	0.375Y	IPR006552 (SMART); IPR008037 (PFAM); IPR020862 (PROSITE_PROFILES); IPR020862 (PROSITE_PROFILES); IPR020862 (PROSITE_PROFILES); IPR020862 (PROSITE_PROFILES); IPR020862 (PROSITE_PROFILES); IPR008037 (SUPERFAMILY); IPR008037 (SUPERFAMILY); IPR008037 (SUPERFAMILY); IPR008037 (SUPERFAMILY); IPR008037 (SUPERFAMILY)
442	c145602_g1_i1_len_283_path_1_0_282_5	12	endo-beta- - partial	94	3.24E-28	71.50%	0.115	IPR012341 (G3DSA:1.50.10.GENE3D); IPR001701 (PFAM); PTHR22298 (PANTHER); PTHR22298:SF17 (PANTHER); IPR008928 (SUPERFAMILY)
444	c145625_g1_i1_len_853_path_1_0_852_4	34	pleiotropic regulator 1	284	2.53E-118	73.00%	0.101	IPR020472 (PRINTS); IPR001680 (SMART); IPR001680 (PFAM); IPR015943 (G3DSA:2.130.10.GENE3D); PTHR19923:SF0 (PANTHER); PTHR19923 (PANTHER); IPR019775 (PROSITE_PATTERNS); IPR001680 (PROSITE_PROFILES); IPR017986 (PROSITE_PROFILES); IPR001680 (PROSITE_PROFILES); IPR001680 (PROSITE_PROFILES); IPR017986 (SUPERFAMILY)
445	c145638_g1_i1_len_472_path_1_0_471_1	10	laminin subunit alpha	157	6.13E-66	78.50%	0.117	IPR002049 (SMART); G3DSA:2.10.25.10 (GENE3D); IPR002049 (PFAM); G3DSA:2.10.25.10 (GENE3D); PTHR10574 (PANTHER); PTHR10574:SF39 (PANTHER); IPR002049 (PROSITE_PATTERNS); IPR002049 (PROSITE_PATTERNS); IPR002049 (PROSITE_PROFILES); IPR002049 (PROSITE_PROFILES); IPR002049 (PROSITE_PROFILES); IPR002049 (PROSITE_PROFILES); SSF57196 (SUPERFAMILY); SSF57196 (SUPERFAMILY)
446	c145641_g1_i1_len_298_path_276_0_297_2	5	furin-like protease isoform 1-crr isoform x3	99	2.22E-56	96.90%	0.106	IPR000209 (PFAM); IPR000209 (G3DSA:3.40.50.GENE3D); IPR015500 (PANTHER); PTHR10795:SF71 (PANTHER); IPR000209 (SUPERFAMILY)
447	c145728_g1_i1_len_607_path_585_0_606_2	14	mam and ldl-receptor class a domain-containing protein 2-like	202	7.45E-64	66.10%	0.124	IPR000998 (SMART); IPR000436 (SMART); G3DSA:2.10.70.10 (GENE3D); IPR000436 (PFAM); IPR000998 (PFAM); PTHR23282 (PANTHER); IPR000436 (PROSITE_PROFILES); IPR000436 (PROSITE_PROFILES); IPR000998 (PROSITE_PROFILES); IPR013320 (SUPERFAMILY); IPR000436 (SUPERFAMILY)
448	c145755_g1_i1_len_2626_path_2604_0_2625_3	197	membrane skeletal	876	1.04E-37	63.80%	0.162	IPR022086 (PFAM); TRANSMEMBRANE (PHOBIUS); NON_CYTOPLASMIC_DOMAIN (PHOBIUS); CYTOPLASMIC_DOMAIN (PHOBIUS); NON_CYTOPLASMIC_DOMAIN (PHOBIUS); CYTOPLASMIC_DOMAIN (PHOBIUS); CYTOPLASMIC_DOMAIN (PHOBIUS); TRANSMEMBRANE (PHOBIUS); TRANSMEMBRANE (PHOBIUS); TRANSMEMBRANE (PHOBIUS); TMhelix (TMHMM); TMhelix (TMHMM); TMhelix (TMHMM)
449	c145775_g1_i1_len_949_path_927_0_948_3	56	structural maintenance of chromosomes protein 2	317	6.69E-87	71.70%	0.1	Coil (COILS); Coil (COILS); Coil (COILS); Coil (COILS); PTHR18937 (PANTHER); IPR027120 (PTHR18937:PANTHER)
450	c145782_g1_i1_len_494_path_472_0_493_0	18	receptor-type tyrosine-protein phosphatase kappa	165	3.50E-42	66.60%	0.106	IPR000242 (PRINTS); IPR000242 (SMART); IPR029021 (G3DSA:3.90.190.GENE3D); IPR000242 (PFAM); PTHR19134 (PANTHER); IPR000242 (PROSITE_PROFILES); IPR029021 (SUPERFAMILY)
451	c145793_g1_i1_len_1492_path_1470_0_1491_1	130	alpha-mannosidase 2	497	0	70.50%	0.125	IPR011682 (PFAM); IPR013780 (G3DSA:2.60.40.GENE3D); G3DSA:2.70.98.30 (GENE3D); PTHR11607 (PANTHER); PTHR11607:SF22 (PANTHER); IPR011013 (SUPERFAMILY)
452	c145872_g1_i1_len_1655_path_1633_0_1654_1	104	pre-mrna-processing factor 19	552	0	90.70%	0.126	IPR020472 (PRINTS); IPR001680 (SMART); IPR003613 (SMART); IPR003613 (PFAM); IPR013915 (PFAM); IPR015943 (G3DSA:2.130.10.GENE3D); IPR001680 (PFAM); IPR013083 (G3DSA:3.30.40.GENE3D); PTHR13889 (PANTHER); IPR019775 (PROSITE_PATTERNS); IPR019775 (PROSITE_PATTERNS); IPR001680 (PROSITE_PROFILES); IPR001680 (PROSITE_PROFILES); IPR017986 (PROSITE_PROFILES); IPR001680 (PROSITE_PROFILES); IPR003613 (PROSITE_PROFILES); IPR017986 (SUPERFAMILY); SSF57850 (SUPERFAMILY)
453	c145962_g1_i1_len_400_path_378_0_399_4	8	mlrp1_acrmi ame: full=mam and ldl-receptor class a domain-containing protein 1 ame: full=skeletal organic matrix mam and ldl-receptor 1 short=som mam and ldl-receptor partial	133	1.77E-24	59.20%	0.106	IPR000998 (PRINTS); IPR000998 (SMART); IPR000998 (PFAM); PTHR23282 (PANTHER); IPR000998 (PROSITE_PROFILES); IPR013320 (SUPERFAMILY)
454	c145986_g1_i1_len_431_path_1_0_430_4	7	serine threonine-protein kinase mst4 isoform x3	144	2.39E-96	98.80%	0.22	IPR002290 (SMART); IPR000719 (PFAM); G3DSA:1.10.510.10 (GENE3D); PTHR24361:SF208 (PANTHER); PTHR24361 (PANTHER); IPR000719 (PROSITE_PROFILES); IPR011009 (SUPERFAMILY)
455	c146014_g1_i1_len_1556_path_1_0_1555_0	157	microfibrillar-associated protein 1-like	519	3.24E-177	77.90%	0.1	Coil (COILS); Coil (COILS); Coil (COILS); Coil (COILS); IPR009730 (PFAM); PTHR15327:SF0 (PANTHER); IPR009730 (PANTHER)

456	c146019_g1_i1_len_3081_path_1_0_3080_0	368	wd repeat-containing protein 3	1027	0	74.00%	0.704 Y	Coil (COILS); IPR020472 (PRINTS); IPR001680 (SMART); IPR015943 (G3DSA:2.130.10.GENE3D); IPR007148 (PFAM); IPR001680 (PFAM); IPR015943 (G3DSA:2.130.10.GENE3D); PTHR19853 (PANTHER); PTHR19853:SF0 (PANTHER); IPR019775 (PROSITE_PATTERNS); IPR019775 (PROSITE_PATTERNS); IPR019775 (PROSITE_PATTERNS); CYTOPLASMIC_DOMAIN (PHOBIUS); TRANSMEMBRANE (PHOBIUS); NON_CYTOPLASMIC_DOMAIN (PHOBIUS); IPR001680 (PROSITE_PROFILES); IPR001680 (PROSITE_PROFILES); IPR001680 (PROSITE_PROFILES); IPR001680 (PROSITE_PROFILES); IPR001680 (PROSITE_PROFILES); IPR001680 (PROSITE_PROFILES); IPR001680 (PROSITE_PROFILES); IPR001680 (PROSITE_PROFILES); IPR017986 (PROSITE_PROFILES); IPR001680 (PROSITE_PROFILES); SignalP-noTM (SIGNALP_EUK); IPR017986 (SUPERFAMILY); IPR017986 (SUPERFAMILY)
457	c146028_g1_i1_len_652_path_1_0_651_4	9	zinc finger protein ozf-like isoform x2	217	7.21E-53	58.50%	0.146	IPR015880 (SMART); IPR013087 (G3DSA:3.30.160.GENE3D); PF13894 (PFAM); IPR013087 (G3DSA:3.30.160.GENE3D); PF13465 (PFAM); PTHR24409:SF13 (PANTHER); PTHR24409 (PANTHER); IPR007087 (PROSITE_PATTERNS); IPR007087 (PROSITE_PATTERNS); IPR007087 (PROSITE_PATTERNS); IPR007087 (PROSITE_PATTERNS); IPR007087 (PROSITE_PATTERNS); IPR007087 (PROSITE_PATTERNS); IPR007087 (PROSITE_PROFILES); IPR007087 (PROSITE_PROFILES); IPR007087 (PROSITE_PROFILES); IPR007087 (PROSITE_PROFILES); IPR007087 (PROSITE_PROFILES); IPR007087 (PROSITE_PROFILES); IPR007087 (PROSITE_PROFILES); IPR007087 (PROSITE_PROFILES); SSF57667 (SUPERFAMILY); SSF57667 (SUPERFAMILY); SSF57667 (SUPERFAMILY); SSF57667 (SUPERFAMILY); SSF57667 (SUPERFAMILY)
458	c146030_g1_i1_len_504_path_482_0_503_0	10	tyrosine-protein kinase wsck	168	3.25E-57	63.60%	0.122	IPR001245 (PRINTS); IPR020635 (SMART); G3DSA:1.10.510.10 (GENE3D); IPR001245 (PFAM); PTHR24416:SF220 (PANTHER); PTHR24416 (PANTHER); IPR000719 (PROSITE_PROFILES); IPR011009 (SUPERFAMILY)
459	c146039_g1_i1_len_1111_path_1089_0_1110_1	110	c-type mannose receptor 2	370	6.40E-16	47.30%	0.178	IPR001304 (SMART); IPR016186 (G3DSA:3.10.100.GENE3D); IPR001304 (PFAM); PTHR22802:SF149 (PANTHER); PTHR22802 (PANTHER); IPR001304 (PROSITE_PROFILES); IPR016187 (SUPERFAMILY); TMhelix (TMHMM)
460	c146040_g1_i1_len_245_path_1_0_244_2	4	serine threonine protein phosphatase ppt1	81	4.95E-31	79.30%	0.138	IPR006186 (PRINTS); IPR029052 (G3DSA:3.60.21.GENE3D); IPR011236 (PTHR11668:PANTHER); PTHR11668 (PANTHER); IPR029052 (SUPERFAMILY)
461	c146113_g1_i1_len_349_path_1_0_348_4	13	gastrula zinc finger partial	116	8.00E-20	56.80%	0.107	IPR015880 (SMART); IPR013087 (G3DSA:3.30.160.GENE3D); IPR013087 (G3DSA:3.30.160.GENE3D); IPR013087 (G3DSA:3.30.160.GENE3D); IPR013087 (G3DSA:3.30.160.GENE3D); IPR007087 (PFAM); PF13465 (PFAM); PTHR24409 (PANTHER); IPR007087 (PROSITE_PATTERNS); IPR007087 (PROSITE_PATTERNS); IPR007087 (PROSITE_PROFILES); IPR007087 (PROSITE_PROFILES); IPR007087 (PROSITE_PROFILES); IPR007087 (PROSITE_PROFILES); SSF57667 (SUPERFAMILY); SSF57667 (SUPERFAMILY); SSF57667 (SUPERFAMILY)
462	c146186_g1_i1_len_959_path_1_0_958_0	34	maltase- intestinal-like	320	2.28E-68	57.50%	0.125	IPR000322 (PFAM); PTHR22762:SF76 (PANTHER); PTHR22762 (PANTHER); SSF51011 (SUPERFAMILY); IPR017853 (SUPERFAMILY)
463	c14619_g1_i1_len_975_path_953_0_974_5	28	glutamate synthase	325	1.07E-171	83.60%	0.1	PR00419 (PRINTS); IPR016040 (G3DSA:3.40.50.GENE3D); G3DSA:1.10.1060.10 (GENE3D); IPR028261 (PFAM); PF13450 (PFAM); PTHR11938:SF61 (PANTHER); PTHR11938 (PANTHER); IPR009051 (SUPERFAMILY); SSF51971 (SUPERFAMILY)
464	c146230_g1_i1_len_138_path_1_0_137_2	0	protein 5nuc- partial	46	9.17E-10	74.90%	0.111	IPR029052 (G3DSA:3.60.21.GENE3D); IPR029052 (SUPERFAMILY)
465	c146278_g1_i1_len_1280_path_1_0_1279_4	45	vitellogenin fused with superoxide dismutase	427	3.08E-46	47.90%	0.148	IPR011030 (G3DSA:1.25.10.GENE3D); IPR001747 (PFAM); PTHR23345 (PANTHER); PTHR23345:SF1 (PANTHER); IPR001747 (PROSITE_PROFILES); IPR011030 (SUPERFAMILY)
466	c146284_g1_i1_len_1623_path_1601_0_1622_2	83	collagen alpha-1 chain	541	3.15E-85	54.20%	0.103	IPR008160 (PFAM); PTHR24023 (PANTHER); PTHR24023:SF397 (PANTHER)
467	c146284_g1_i1_len_1623_path_1601_0_1622_5	83	mucin-2-like isoform x2	541	2.93E-56	44.80%	0.103	PTHR24023 (PANTHER); PTHR24023:SF405 (PANTHER)
468	c146336_g1_i1_len_602_path_580_0_601_5	11	leucine-rich repeats and immunoglobulin-like domains protein partial	200	4.07E-71	72.60%	0.119	IPR000483 (SMART); IPR003599 (SMART); IPR003598 (SMART); IPR003591 (SMART); PF13895 (PFAM); IPR013783 (G3DSA:2.60.40.GENE3D); IPR000483 (PFAM); IPR001611 (PFAM); G3DSA:3.80.10.10 (GENE3D); PTHR24367:SF242 (PANTHER); PTHR24367 (PANTHER); IPR007110 (PROSITE_PROFILES); IPR001611 (PROSITE_PROFILES); SSF48726 (SUPERFAMILY); SSF52058 (SUPERFAMILY)

470	c14636_g2_i1_len_332_path_514_0_331_3	6	serine threonine-protein kinase 10 isoform x1	111	2.33E-64	96.00%	0.102	IPR002290 (SMART); IPR000719 (PFAM); G3DSA:1.10.510.10 (GENE3D); PTHR24361:SF249 (PANTHER); PTHR24361 (PANTHER); IPR008271 (PROSITE_PATTERNS); IPR000719 (PROSITE_PROFILES); IPR011009 (SUPERFAMILY)
469	c146364_g1_i1_len_1278_path_1243_0_1223_2467_1224_1241_2467_1242_1259_2467_1260_1277_2	58	cyclin-g-associated kinase-like	426	1.04E-153	77.00%	0.159	IPR002290 (SMART); G3DSA:3.30.200.20 (GENE3D); G3DSA:1.10.510.10 (GENE3D); IPR000719 (PFAM); PTHR23172:SF19 (PANTHER); PTHR23172 (PANTHER); IPR008271 (PROSITE_PATTERNS); IPR000719 (PROSITE_PROFILES); IPR011009 (SUPERFAMILY)
471	c146396_g1_i1_len_806_path_784_0_805_5	30	omega-amidase nit2-like	268	5.87E-145	83.30%	0.107	IPR003010 (G3DSA:3.60.110.GENE3D); IPR003010 (PFAM); PTHR23088:SF20 (PANTHER); PTHR23088 (PANTHER); IPR003010 (PROSITE_PROFILES); IPR003010 (SUPERFAMILY)
472	c146430_g1_i1_len_233_path_211_0_232_2	5	prolyl 4- beta subunit	77	6.18E-18	70.20%	0.113	IPR012336 (G3DSA:3.40.30.GENE3D); PTHR18929:SF74 (PANTHER); PTHR18929 (PANTHER); IPR012336 (SUPERFAMILY)
473	c146452_g1_i1_len_278_path_1_0_277_0	4	transient receptor potential channel pyrexia	93	8.82E-18	68.80%	0.139	IPR002110 (SMART); IPR020683 (G3DSA:1.25.40.GENE3D); IPR020683 (PFAM); PTHR24193 (PANTHER); IPR002110 (PROSITE_PROFILES); IPR002110 (PROSITE_PROFILES); IPR020683 (PROSITE_PROFILES); IPR020683 (SUPERFAMILY)
474	c146457_g1_i1_len_2142_path_2120_0_2141_4	168	alpha-2-macroglobulin-like protein 1 isoform x1	714	5.40E-144	55.20%	0.197	IPR011625 (PFAM); IPR002890 (PFAM); PTHR11412 (PANTHER)
475	c146458_g1_i1_len_303_path_1_0_302_1	5	isoform c	101	8.27E-25	67.60%	0.123	IPR014782 (PRINTS); G3DSA:1.10.390.10 (GENE3D); IPR014782 (PFAM); PTHR11533:SF165 (PANTHER); IPR001930 (PANTHER); SSF55486 (SUPERFAMILY)
477	c14648_g1_i1_len_2505_path_1_0_2504_4	278	n-acetylated-alpha-linked acidic dipeptidase	835	0	59.30%	0.175	IPR007365 (G3DSA:1.20.930.GENE3D); G3DSA:3.50.30.30 (GENE3D); IPR007365 (PFAM); IPR007484 (PFAM); IPR003137 (PFAM); G3DSA:3.40.630.10 (GENE3D); PTHR10404 (PANTHER); CYTOPLASMIC_DOMAIN (PHOBIUS); TRANSMEMBRANE (PHOBIUS); TRANSMEMBRANE (PHOBIUS); TRANSMEMBRANE (PHOBIUS); CYTOPLASMIC_DOMAIN (PHOBIUS); NON_CYTOPLASMIC_DOMAIN (PHOBIUS); NON_CYTOPLASMIC_DOMAIN (PHOBIUS); SSF53187 (SUPERFAMILY); IPR007365 (SUPERFAMILY); SSF52025 (SUPERFAMILY); TMhelix (TMHMM); TMhelix (TMHMM); TMhelix (TMHMM)
476	c146489_g1_i1_len_416_path_394_0_415_3	12	phosphatidylinositol -trisphosphate 3-phosphatase and dual-specificity protein phosphatase pten	139	2.98E-49	86.10%	0.126	IPR029021 (G3DSA:3.90.190.GENE3D); PTHR12305 (PANTHER); CYTOPLASMIC_DOMAIN (PHOBIUS); TRANSMEMBRANE (PHOBIUS); NON_CYTOPLASMIC_DOMAIN (PHOBIUS); IPR029023 (PROSITE_PROFILES); IPR029021 (SUPERFAMILY)
478	c146509_g1_i1_len_852_path_1_0_851_3	4885	hemagglutinin amebocyte aggregation factor-like	284	9.35E-38	58.00%	0.147	PF14704 (PFAM); IPR026645 (PANTHER); NON_CYTOPLASMIC_DOMAIN (PHOBIUS); CYTOPLASMIC_DOMAIN (PHOBIUS); TRANSMEMBRANE (PHOBIUS); TMhelix (TMHMM)
479	c146512_g1_i1_len_531_path_1_0_530_1	16	ectonucleoside triphosphate diphosphohydrolase 7	177	5.87E-29	75.20%	0.108	IPR000407 (PFAM); IPR000407 (PANTHER); PTHR11782:SF37 (PANTHER); CYTOPLASMIC_DOMAIN (PHOBIUS); NON_CYTOPLASMIC_DOMAIN (PHOBIUS); TRANSMEMBRANE (PHOBIUS); TMhelix (TMHMM)
480	c14678_g1_i1_len_2191_path_2169_0_1289_3459_1290_1383_3553_1384_2190_4	3230	protein disulfide-isomerase a3	730	0	78.20%	0.294	PR00421 (PRINTS); IPR012336 (G3DSA:3.40.30.GENE3D); PF13848 (PFAM); IPR012336 (G3DSA:3.40.30.GENE3D); IPR013766 (PFAM); IPR005792 (TIGRFAM); IPR012336 (G3DSA:3.40.30.GENE3D); IPR005788 (TIGRFAM); PTHR18929:SF60 (PANTHER); PTHR18929 (PANTHER); IPR017937 (PROSITE_PATTERNS); IPR017937 (PROSITE_PATTERNS); IPR012336 (PROSITE_PROFILES); IPR012336 (PROSITE_PROFILES); IPR012336 (SUPERFAMILY); IPR012336 (SUPERFAMILY); IPR012336 (SUPERFAMILY); IPR012336 (SUPERFAMILY)
481	c146790_g1_i1_len_582_path_560_0_581_0	20	macrophage mannose receptor 1-like	194	7.04E-30	52.00%	0.370Y	IPR001304 (SMART); IPR016186 (G3DSA:3.10.100.GENE3D); IPR016186 (G3DSA:3.10.100.GENE3D); IPR001304 (PFAM); PTHR22803 (PANTHER); PTHR22803:SF16 (PANTHER); IPR018378 (PROSITE_PATTERNS); IPR001304 (PROSITE_PROFILES); IPR001304 (PROSITE_PROFILES); IPR016187 (SUPERFAMILY); IPR016187 (SUPERFAMILY)
482	c146818_g1_i1_len_672_path_1_0_671_5	17	cyclin-dependent kinase 2 isoform x2	224	3.05E-89	75.60%	0.139	IPR002290 (SMART); G3DSA:3.30.200.20 (GENE3D); G3DSA:1.10.510.10 (GENE3D); IPR000719 (PFAM); PTHR24056 (PANTHER); PTHR24056:SF168 (PANTHER); IPR017441 (PROSITE_PATTERNS); IPR008271 (PROSITE_PATTERNS); NON_CYTOPLASMIC_DOMAIN (PHOBIUS); CYTOPLASMIC_DOMAIN (PHOBIUS); TRANSMEMBRANE (PHOBIUS); IPR000719 (PROSITE_PROFILES); IPR011009 (SUPERFAMILY)

483	c146826_g1_i1_len_375_path_353_0_374_3	6	cysteine-rich motor neuron 1 partial	125	8.02E-38	67.40%	0.128	IPR001007 (SMART); IPR001007 (PFAM); G3DSA:2.10.70.10 (GENE3D); PTHR11339 (PANTHER); IPR001007 (PROSITE_PATTERNS); IPR001007 (PROSITE_PATTERNS); IPR001007 (PROSITE_PROFILES); IPR001007 (PROSITE_PROFILES); SSF57603 (SUPERFAMILY); SSF57603 (SUPERFAMILY)
484	c146839_g1_i1_len_370_path_348_0_369_2	8	slit homolog 2 protein	123	5.13E-57	81.50%	0.123	G3DSA:3.80.10.10 (GENE3D); PTHR24365:SF270 (PANTHER); PTHR24365 (PANTHER); SSF52058 (SUPERFAMILY)
485	c146855_g1_i1_len_267_path_245_0_266_2	4	prolyl 3-hydroxylase 2-like isoform x1	89	7.39E-29	73.50%	0.186	PTHR14049 (PANTHER)
486	c146859_g1_i1_len_1128_path_1_0_1127_1	51	insulin-like growth factor-binding protein complex acid labile chain	376	3.44E-30	54.80%	0.093	G3DSA:3.80.10.10 (GENE3D); PTHR24369 (PANTHER); SSF52058 (SUPERFAMILY)
487	c146925_g1_i1_len_744_path_1_0_743_4	27	adenylate cyclase type 9	248	1.04E-80	86.20%	0.1	IPR001054 (SMART); IPR001054 (PFAM); IPR001054 (G3DSA:3.30.70.GENE3D); PTHR11920:SF272 (PANTHER); PTHR11920 (PANTHER); IPR018297 (PROSITE_PATTERNS); CYTOPLASMIC_DOMAIN (PHOBIUS); NON_CYTOPLASMIC_DOMAIN (PHOBIUS); TRANSMEMBRANE (PHOBIUS); IPR001054 (PROSITE_PROFILES); IPR029787 (SUPERFAMILY); TMhelix (TMHMM)
488	c146957_g1_i1_len_645_path_39_0_644_3	54	low quality protein: tenascin	208	2.68E-28	63.50%	0.12	IPR002181 (SMART); IPR014715 (G3DSA:4.10.530.GENE3D); IPR014716 (G3DSA:3.90.215.GENE3D); IPR002181 (PFAM); PTHR19143 (PANTHER); IPR020837 (PROSITE_PATTERNS); IPR002181 (PROSITE_PROFILES); IPR002181 (SUPERFAMILY)
489	c14700_g1_i1_len_2244_path_1_0_2243_0	271	wd repeat-containing protein 36	748	0	70.10%	0.192	IPR001680 (SMART); IPR015943 (G3DSA:2.130.10.GENE3D); IPR001680 (PFAM); IPR007319 (PFAM); IPR015943 (G3DSA:2.130.10.GENE3D); PTHR13889:SF2 (PANTHER); PTHR13889 (PANTHER); IPR001680 (PROSITE_PROFILES); IPR001680 (PROSITE_PROFILES); IPR017986 (PROSITE_PROFILES); IPR001680 (PROSITE_PROFILES); IPR017986 (PROSITE_PROFILES); IPR001680 (PROSITE_PROFILES); IPR017986 (SUPERFAMILY)
490	c147019_g1_i1_len_392_path_370_0_391_4	10	protein yellow-like	131	4.80E-22	58.60%	0.11	IPR017996 (PFAM); IPR011042 (G3DSA:2.120.10.GENE3D)
491	c147039_g1_i1_len_1585_path_1_0_1584_2	1586	d-aspartate oxidase	528	1.45E-98	63.60%	0.164	IPR016040 (G3DSA:3.40.50.GENE3D); IPR006076 (PFAM); PTHR11530:SF0 (PANTHER); IPR023209 (PANTHER); IPR006181 (PROSITE_PATTERNS); SSF54373 (SUPERFAMILY); SSF51971 (SUPERFAMILY); TMhelix (TMHMM)
492	c147044_g1_i1_len_721_path_1_0_720_5	20	gamma-glutamyltranspeptidase 1-like isoform x2	240	4.18E-75	69.70%	0.103	IPR000101 (PRINTS); IPR000101 (PFAM); IPR000101 (PANTHER); PTHR11686:SF15 (PANTHER); IPR029055 (SUPERFAMILY)
493	c14706_g1_i1_len_1999_path_1977_0_1998_5	169	myosin light chain smooth muscle	666	0	79.20%	0.101	Coil (COILS); IPR003599 (SMART); IPR003598 (SMART); IPR013098 (PFAM); IPR013783 (G3DSA:2.60.40.GENE3D); IPR013783 (G3DSA:2.60.40.GENE3D); IPR013783 (G3DSA:2.60.40.GENE3D); PF13895 (PFAM); IPR013783 (G3DSA:2.60.40.GENE3D); IPR013783 (G3DSA:2.60.40.GENE3D); PTHR19897 (PANTHER); IPR007110 (PROSITE_PROFILES); IPR007110 (PROSITE_PROFILES); IPR007110 (PROSITE_PROFILES); IPR007110 (PROSITE_PROFILES); IPR007110 (PROSITE_PROFILES); IPR007110 (PROSITE_PROFILES); SSF48726 (SUPERFAMILY); SSF48726 (SUPERFAMILY); SSF48726 (SUPERFAMILY); SSF48726 (SUPERFAMILY); SSF48726 (SUPERFAMILY); SSF48726 (SUPERFAMILY)
494	c147164_g1_i1_len_742_path_720_0_741_0	12	zinc finger protein 120-like isoform x1	248	5.36E-70	67.20%	0.1	IPR015880 (SMART); IPR013087 (G3DSA:3.30.160.GENE3D); PF13465 (PFAM); IPR013087 (G3DSA:3.30.160.GENE3D); IPR013087 (G3DSA:3.30.160.GENE3D); IPR013087 (G3DSA:3.30.160.GENE3D); IPR007087 (PFAM); PTHR24409 (PANTHER); IPR007087 (PROSITE_PATTERNS); IPR007087 (PROSITE_PATTERNS); IPR007087 (PROSITE_PATTERNS); IPR007087 (PROSITE_PATTERNS); IPR007087 (PROSITE_PATTERNS); IPR007087 (PROSITE_PATTERNS); IPR007087 (PROSITE_PATTERNS); IPR007087 (PROSITE_PROFILES); IPR007087 (PROSITE_PROFILES); IPR007087 (PROSITE_PROFILES); IPR007087 (PROSITE_PROFILES); IPR007087 (PROSITE_PROFILES); IPR007087 (PROSITE_PROFILES); IPR007087 (PROSITE_PROFILES); IPR007087 (PROSITE_PROFILES); SSF57667 (SUPERFAMILY); SSF57667 (SUPERFAMILY); SSF57667 (SUPERFAMILY); SSF57667 (SUPERFAMILY)
495	c147180_g1_i1_len_310_path_288_0_309_4	4	membrane metallo-endopeptidase-like 1	103	9.85E-38	83.30%	0.108	IPR024079 (G3DSA:3.40.390.GENE3D); IPR018497 (PFAM); PTHR11733:SF111 (PANTHER); IPR000718 (PANTHER); SSF55486 (SUPERFAMILY)

496	c14719_g1_i1_len_1710_path_1_0_1644_3583_1645_1709_4	3589	cathepsin b	570	3.64E-164	78.60%	0.174	IPR000668 (PRINTS); IPR000668 (SMART); G3DSA:3.90.70.10 (GENE3D); IPR000668 (PFAM); IPR012599 (PFAM); IPR015643 (PTHR12411:PANTHER); IPR013128 (PANTHER); IPR025660 (PROSITE_PATTERNS); IPR000169 (PROSITE_PATTERNS); IPR025661 (PROSITE_PATTERNS); CYTOPLASMIC_DOMAIN (PHOBIUS); TRANSMEMBRANE (PHOBIUS); TRANSMEMBRANE (PHOBIUS); CYTOPLASMIC_DOMAIN (PHOBIUS); NON_CYTOPLASMIC_DOMAIN (PHOBIUS); TRANSMEMBRANE (PHOBIUS); NON_CYTOPLASMIC_DOMAIN (PHOBIUS); TRANSMEMBRANE (PHOBIUS); CYTOPLASMIC_DOMAIN (PHOBIUS); NON_CYTOPLASMIC_DOMAIN (PHOBIUS); TRANSMEMBRANE (PHOBIUS); SSF54001 (SUPERFAMILY); TMhelix (TMHMM); TMhelix (TMHMM)
497	c14719_g1_i2_len_1770_path_1_0_1644_1646_1645_1769_4	3689	cathepsin b	590	6.07E-164	78.60%	0.117	IPR000668 (PRINTS); IPR000668 (SMART); G3DSA:3.90.70.10 (GENE3D); IPR012599 (PFAM); IPR000668 (PFAM); IPR013128 (PANTHER); IPR015643 (PTHR12411:PANTHER); IPR000169 (PROSITE_PATTERNS); IPR025660 (PROSITE_PATTERNS); IPR025661 (PROSITE_PATTERNS); TRANSMEMBRANE (PHOBIUS); TRANSMEMBRANE (PHOBIUS); NON_CYTOPLASMIC_DOMAIN (PHOBIUS); CYTOPLASMIC_DOMAIN (PHOBIUS); CYTOPLASMIC_DOMAIN (PHOBIUS); SSF54001 (SUPERFAMILY); TMhelix (TMHMM)
498	c147217_g1_i1_len_401_path_379_0_400_2	12	wd repeat and fyve domain-containing protein 2	133	1.90E-69	82.60%	0.143	IPR001680 (SMART); IPR015943 (G3DSA:2.130.10.GENE3D); IPR001680 (PFAM); PTHR13856:SF7 (PANTHER); PTHR13856 (PANTHER); IPR019775 (PROSITE_PATTERNS); IPR019775 (PROSITE_PATTERNS); IPR001680 (PROSITE_PROFILES); IPR017986 (PROSITE_PROFILES); IPR001680 (PROSITE_PROFILES); IPR017986 (SUPERFAMILY)
499	c14722_g1_i1_len_3082_path_3733_0_2522_25_2523_3081_4	447	exocyst complex component 6b-like isoform 2	1027	0	80.70%	0.141	Coil (COILS); Coil (COILS); Coil (COILS); IPR007225 (PFAM); IPR007225 (PANTHER); CYTOPLASMIC_DOMAIN (PHOBIUS); TRANSMEMBRANE (PHOBIUS); CYTOPLASMIC_DOMAIN (PHOBIUS); TRANSMEMBRANE (PHOBIUS); NON_CYTOPLASMIC_DOMAIN (PHOBIUS); TRANSMEMBRANE (PHOBIUS); NON_CYTOPLASMIC_DOMAIN (PHOBIUS); TMhelix (TMHMM); TMhelix (TMHMM)
500	c147253_g1_i1_len_667_path_645_0_666_5	24	protein disulfide isomerase family member 4	222	2.59E-69	63.40%	0.126	IPR012336 (G3DSA:3.40.30.GENE3D); IPR013766 (PFAM); PF13848 (PFAM); IPR012336 (G3DSA:3.40.30.GENE3D); PTHR18929:SF56 (PANTHER); PTHR18929 (PANTHER); IPR012336 (PROSITE_PROFILES); IPR012336 (SUPERFAMILY); IPR012336 (SUPERFAMILY)
501	c147263_g1_i1_len_285_path_1_0_284_2	4	uncharacterized aarf domain-containing protein kinase 1-like	95	3.18E-35	77.50%	0.162	PTHR10566:SF63 (PANTHER); PTHR10566 (PANTHER)
502	c14731_g1_i1_len_875_path_1_0_874_5	57	oocyte zinc finger protein partial	291	1.55E-08	59.50%	0.1	IPR007087 (PROSITE_PATTERNS); IPR007087 (PROSITE_PROFILES); SSF57667 (SUPERFAMILY)
503	c14739_g1_i1_len_365_path_343_0_364_5	15	vesicle-fusing atpase 1-like	121	3.51E-40	76.90%	0.12	IPR003338 (SMART); G3DSA:2.40.40.20 (GENE3D); IPR003338 (PFAM); PTHR23078 (PANTHER); SignalP-TM (SIGNALP_GRAM_POSITIVE); IPR009010 (SUPERFAMILY)
504	c147416_g1_i1_len_573_path_551_0_572_4	12	group 3 secretory phospholipase a2-like	191	8.09E-43	71.30%	0.277	IPR016090 (G3DSA:1.20.90.GENE3D); IPR001211 (PFAM); PTHR12253 (PANTHER); PTHR12253:SF8 (PANTHER); IPR013090 (PROSITE_PATTERNS); IPR016090 (SUPERFAMILY)
505	c147494_g1_i1_len_955_path_933_0_954_2	46	dehydrogenase reductase sdr family protein 7-like	318	2.87E-85	83.20%	0.104	IPR002347 (PRINTS); IPR016040 (G3DSA:3.40.50.GENE3D); IPR002198 (PFAM); PTHR24322 (PANTHER); PTHR24322:SF325 (PANTHER); IPR020904 (PROSITE_PATTERNS); CYTOPLASMIC_DOMAIN (PHOBIUS); NON_CYTOPLASMIC_DOMAIN (PHOBIUS); TRANSMEMBRANE (PHOBIUS); SSF51735 (SUPERFAMILY)
506	c147558_g1_i1_len_834_path_1_0_833_3	15	serine threonine-protein kinase dclk2-like isoform x3	278	4.08E-42	75.80%	0.304	G3DSA:1.10.510.10 (GENE3D); IPR000719 (PFAM); PTHR24347:SF49 (PANTHER); IPR020636 (PANTHER); TRANSMEMBRANE (PHOBIUS); CYTOPLASMIC_DOMAIN (PHOBIUS); NON_CYTOPLASMIC_DOMAIN (PHOBIUS); IPR000719 (PROSITE_PROFILES); IPR011009 (SUPERFAMILY); TMhelix (TMHMM)
507	c147561_g1_i1_len_249_path_227_0_248_0	3	matrix metalloproteinase	83	1.58E-17	67.90%	0.491Y	IPR021190 (PRINTS); IPR024079 (G3DSA:3.40.390.GENE3D); IPR001818 (PFAM); PTHR10201 (PANTHER); SignalP-noTM (SIGNALP_EUK); SSF55486 (SUPERFAMILY)
508	c147606_g1_i1_len_1008_path_1_0_1007_4	21	low affinity immunoglobulin epsilon fc receptor-like	336	1.05E-18	48.80%	0.11	IPR001304 (SMART); IPR001304 (PFAM); IPR016186 (G3DSA:3.10.100.GENE3D); PTHR22802 (PANTHER); IPR018378 (PROSITE_PATTERNS); IPR001304 (PROSITE_PROFILES); IPR016187 (SUPERFAMILY)
509	c147773_g1_i1_len_242_path_220_0_241_1	6	esterase e4-like	81	9.76E-29	77.20%	0.201	IPR002018 (PFAM); IPR029058 (G3DSA:3.40.50.GENE3D); PTHR11559 (PANTHER); IPR029058 (SUPERFAMILY)

510	c14789_g1_i1_len_818_path_1_0_817_1	96	glutathione peroxidase 7	273	7.88E-76	70.60%	0.531Y	IPR000889 (PRINTS); IPR012336 (G3DSA:3.40.30.GENE3D); IPR000889 (PFAM); IPR000889 (PANTHER); PTHR11592:SF10 (PANTHER); IPR029759 (PROSITE_PATTERNS); IPR029760 (PROSITE_PATTERNS); TRANSMEMBRANE (PHOBIUS); NON_CYTOPLASMIC_DOMAIN (PHOBIUS); CYTOPLASMIC_DOMAIN (PHOBIUS); IPR000889 (PROSITE_PROFILES); SignalP-noTM (SIGNALP_EUK); IPR012336 (SUPERFAMILY); TMhelix (TMHMM)
511	c147928_g1_i1_len_693_path_671_0_692_2	14	integrin alpha-ps1 isoform x2	231	1.04E-96	74.30%	0.111	IPR000413 (PRINTS); IPR013519 (SMART); G3DSA:2.130.10.130 (GENE3D); PF13517 (PFAM); IPR013517 (PFAM); G3DSA:2.60.40.1460 (GENE3D); PTHR23220 (PANTHER); PTHR23220:SF77 (PANTHER); IPR013519 (PROSITE_PROFILES); IPR013519 (PROSITE_PROFILES); IPR013519 (PROSITE_PROFILES); SSF69318 (SUPERFAMILY)
512	c147969_g1_i1_len_276_path_254_0_275_3	12	titin isoform x1	92	2.37E-33	77.80%	0.111	IPR003599 (SMART); IPR013098 (PFAM); IPR013783 (G3DSA:2.60.40.GENE3D); PTHR19897 (PANTHER); PTHR19897:SF149 (PANTHER); IPR007110 (PROSITE_PROFILES); SSF48726 (SUPERFAMILY)
513	c148104_g1_i1_len_353_path_1_0_352_0	5	low-density lipoprotein receptor-related protein 4	118	1.98E-15	57.90%	0.106	IPR002172 (PRINTS); IPR002172 (SMART); IPR002172 (G3DSA:4.10.400.GENE3D); IPR002172 (G3DSA:4.10.400.GENE3D); IPR002172 (PFAM); PTHR10529 (PANTHER); IPR023415 (PROSITE_PATTERNS); IPR023415 (PROSITE_PATTERNS); IPR002172 (PROSITE_PROFILES); IPR002172 (PROSITE_PROFILES); IPR002172 (SUPERFAMILY); IPR002172 (SUPERFAMILY)
514	c148132_g1_i1_len_507_path_1_0_278_280_279_392_280_393_506_2	13	sortilin-related receptor-like	169	6.74E-10	47.40%	0.099	IPR002172 (SMART); IPR002172 (G3DSA:4.10.400.GENE3D); IPR002172 (PFAM); G3DSA:4.10.1220.10 (GENE3D); PTHR10529 (PANTHER); IPR023415 (PROSITE_PATTERNS); IPR002172 (PROSITE_PROFILES); IPR002172 (SUPERFAMILY)
515	c148179_g1_i1_len_247_path_1_0_246_1	4	c-type lectin domain family 4 member d	82	3.70E-09	55.90%	0.112	IPR016186 (G3DSA:3.10.100.GENE3D); IPR001304 (PFAM); IPR001304 (PROSITE_PROFILES); IPR016187 (SUPERFAMILY)
516	c148194_g1_i1_len_310_path_288_0_309_4	6	hormone-sensitive lipase	103	1.08E-42	76.10%	0.182	IPR013094 (PFAM); IPR029058 (G3DSA:3.40.50.GENE3D); PTHR23025 (PANTHER); PTHR23025:SF3 (PANTHER); IPR029058 (SUPERFAMILY)
521	c1482_g1_i1_len_375_path_125_0_374_4	8	down syndrome cell adhesion molecule isoform x101	125	1.38E-65	93.00%	0.121	PR00014 (PRINTS); IPR003961 (SMART); IPR003961 (PFAM); IPR013783 (G3DSA:2.60.40.GENE3D); IPR013783 (G3DSA:2.60.40.GENE3D); PTHR10489 (PANTHER); PTHR10489:SF567 (PANTHER); IPR003961 (PROSITE_PROFILES); IPR003961 (SUPERFAMILY); SSF48726 (SUPERFAMILY)
517	c148222_g1_i1_len_1616_path_1_0_1615_2	150	thioredoxin-related transmembrane protein 1-like	538	1.08E-97	71.30%	0.684Y	IPR012336 (G3DSA:3.40.30.GENE3D); IPR013766 (PFAM); PTHR18929 (PANTHER); PTHR18929:SF81 (PANTHER); IPR017937 (PROSITE_PATTERNS); SIGNAL_PEPTIDE_H_REGION (PHOBIUS); NON_CYTOPLASMIC_DOMAIN (PHOBIUS); SIGNAL_PEPTIDE_N_REGION (PHOBIUS); SIGNAL_PEPTIDE (PHOBIUS); TRANSMEMBRANE (PHOBIUS); SIGNAL_PEPTIDE_C_REGION (PHOBIUS); CYTOPLASMIC_DOMAIN (PHOBIUS); IPR012336 (PROSITE_PROFILES); SignalP-noTM (SIGNALP_EUK); IPR012336 (SUPERFAMILY); TMhelix (TMHMM)
518	c148260_g1_i1_len_594_path_1_0_296_1_297_593_0	15	titin-like isoform x26	198	9.38E-12	45.40%	0.107	IPR003961 (SMART); IPR013783 (G3DSA:2.60.40.GENE3D); IPR003961 (PFAM); IPR013783 (G3DSA:2.60.40.GENE3D); PTHR10489 (PANTHER); IPR003961 (PROSITE_PROFILES); IPR003961 (PROSITE_PROFILES); IPR003961 (SUPERFAMILY)
519	c148265_g1_i1_len_639_path_1_0_638_2	42	aldehyde dehydrogenase family 9 member a1-a-like	213	1.95E-54	81.40%	0.112	IPR016163 (G3DSA:3.40.309.GENE3D); IPR015590 (PFAM); PTHR11699:SF114 (PANTHER); PTHR11699 (PANTHER); IPR016161 (SUPERFAMILY)
520	c148274_g1_i1_len_705_path_1_0_704_0	14	acyl- delta desaturase	235	5.33E-83	73.70%	0.1	IPR015876 (PRINTS); IPR005804 (PFAM); PTHR11351 (PANTHER); PTHR11351:SF28 (PANTHER); CYTOPLASMIC_DOMAIN (PHOBIUS); NON_CYTOPLASMIC_DOMAIN (PHOBIUS); TRANSMEMBRANE (PHOBIUS); CYTOPLASMIC_DOMAIN (PHOBIUS); TRANSMEMBRANE (PHOBIUS); NON_CYTOPLASMIC_DOMAIN (PHOBIUS); TRANSMEMBRANE (PHOBIUS); TMhelix (TMHMM); TMhelix (TMHMM); TMhelix (TMHMM)
522	c148374_g1_i1_len_363_path_341_0_362_4	6	glucosylceramidase- partial	121	3.60E-37	68.20%	0.144	IPR013780 (G3DSA:2.60.40.GENE3D); IPR013781 (G3DSA:3.20.20.GENE3D); IPR001139 (PFAM); PTHR11069:SF7 (PANTHER); IPR001139 (PANTHER); IPR017853 (SUPERFAMILY)
523	c148388_g1_i1_len_1220_path_1198_0_1219_3	51	peptidylprolyl isomerase domain and wd repeat-containing protein 1	407	0	88.40%	0.105	IPR002130 (PRINTS); IPR015943 (G3DSA:2.130.10.GENE3D); IPR029000 (G3DSA:2.40.100.GENE3D); IPR002130 (PFAM); IPR024936 (PANTHER); PTHR11071:SF228 (PANTHER); IPR002130 (PROSITE_PROFILES); SSF69322 (SUPERFAMILY); IPR029000 (SUPERFAMILY)

524	c148496_g1_i1_len_738_path_716_0_737_1	34	contactin associated protein 1	246	6.40E-31	50.30%	0.104	IPR001073 (PRINTS); IPR001073 (SMART); IPR008983 (G3DSA:2.60.120.GENE3D); IPR014716 (G3DSA:3.90.215.GENE3D); IPR001073 (PFAM); PTHR10127 (PANTHER); PTHR10127:SF575 (PANTHER); IPR001073 (PROSITE_PROFILES); IPR008983 (SUPERFAMILY); IPR002181 (SUPERFAMILY)
525	c148521_g1_i1_len_1110_path_1_0_1109_4	57	iduronate 2-sulfatase-like	370	1.20E-88	59.70%	0.115	IPR017849 (G3DSA:3.40.720.GENE3D); G3DSA:3.30.1120.10 (GENE3D); IPR000917 (PFAM); PTHR10342 (PANTHER); PTHR10342:SF185 (PANTHER); TRANSMEMBRANE (PHOBIUS); TRANSMEMBRANE (PHOBIUS); NON_CYTOPLASMIC_DOMAIN (PHOBIUS); NON_CYTOPLASMIC_DOMAIN (PHOBIUS); CYTOPLASMIC_DOMAIN (PHOBIUS); IPR017850 (SUPERFAMILY)
526	c148556_g1_i1_len_260_path_1_0_259_2	8	dna damage checkpoint protein	86	1.12E-15	93.50%	0.109	G3DSA:1.20.190.20 (GENE3D); IPR023410 (PFAM); IPR000308 (PANTHER); IPR023409 (PROSITE_PATTERNS); IPR023410 (SUPERFAMILY)
527	c14856_g1_i1_len_645_path_463_0_644_0	28	dnaj homolog dnj-5	215	2.99E-95	84.00%	0.11	IPR001623 (PRINTS); IPR001623 (SMART); G3DSA:3.90.10.10 (GENE3D); PF14901 (PFAM); IPR001623 (G3DSA:1.10.287.GENE3D); IPR001623 (PFAM); PTHR24078 (PANTHER); PTHR24078:SF141 (PANTHER); IPR001623 (PROSITE_PROFILES); IPR001623 (SUPERFAMILY)
528	c148607_g1_i1_len_738_path_716_0_737_0	59	peptidyl-prolyl cis-trans isomerase-like 1	234	1.90E-86	92.30%	0.115	IPR002130 (PRINTS); IPR029000 (G3DSA:2.40.100.GENE3D); IPR002130 (PFAM); IPR024936 (PANTHER); IPR020892 (PROSITE_PATTERNS); IPR002130 (PROSITE_PROFILES); IPR029000 (SUPERFAMILY)
529	c148690_g1_i1_len_963_path_1_0_962_5	86	protein tanc2 isoform x2	321	1.52E-121	78.40%	0.133	IPR002110 (SMART); IPR002110 (PFAM); IPR020683 (PFAM); IPR020683 (G3DSA:1.25.40.GENE3D); IPR020683 (G3DSA:1.25.40.GENE3D); PTHR24166 (PANTHER); PTHR24166:SF8 (PANTHER); IPR002110 (PROSITE_PROFILES); IPR002110 (PROSITE_PROFILES); IPR002110 (PROSITE_PROFILES); IPR020683 (PROSITE_PROFILES); IPR002110 (PROSITE_PROFILES); IPR020683 (SUPERFAMILY)
530	c148730_g1_i1_len_310_path_1_0_309_0	10	ankyrin repeat	104	2.21E-22	60.30%	0.113	IPR002110 (PRINTS); IPR002110 (SMART); IPR020683 (PFAM); IPR002110 (PFAM); IPR020683 (G3DSA:1.25.40.GENE3D); IPR020683 (G3DSA:1.25.40.GENE3D); PTHR24198 (PANTHER); IPR020683 (PROSITE_PROFILES); IPR002110 (PROSITE_PROFILES); IPR002110 (PROSITE_PROFILES); IPR002110 (PROSITE_PROFILES); IPR020683 (SUPERFAMILY)
531	c148787_g1_i1_len_381_path_359_0_380_2	6	d chain coupling of remote alternating-access transport mechanisms for protons and substrates in the multidrug efflux pump acrb	127	6.01E-18	68.60%	0.104	IPR002110 (PRINTS); IPR002110 (SMART); IPR020683 (PFAM); IPR020683 (G3DSA:1.25.40.GENE3D); PTHR24182 (PANTHER); IPR020683 (PROSITE_PROFILES); IPR002110 (PROSITE_PROFILES); IPR002110 (PROSITE_PROFILES); IPR020683 (SUPERFAMILY)
532	c148867_g1_i1_len_323_path_1_0_322_3	4	peptidylprolyl isomerase	108	2.22E-39	68.70%	0.11	IPR001179 (PFAM); G3DSA:3.10.50.40 (GENE3D); IPR023566 (PANTHER); IPR001179 (PROSITE_PROFILES); SSF54534 (SUPERFAMILY)
533	c148874_g1_i1_len_1820_path_1_0_1819_3	38	protein jagged-1-like	607	4.13E-14	38.10%	0.107	no IPS match
534	c148924_g1_i1_len_318_path_296_0_317_1	12	proclotting enzyme	106	2.55E-19	59.50%	0.133	G3DSA:2.40.10.10 (GENE3D); IPR001254 (PFAM); G3DSA:2.40.10.10 (GENE3D); PTHR24260:SF3 (PANTHER); PTHR24260 (PANTHER); IPR018114 (PROSITE_PATTERNS); IPR001254 (PROSITE_PROFILES); IPR009003 (SUPERFAMILY)
535	c148990_g1_i1_len_423_path_401_0_422_0	6	protogenin a-like	141	6.19E-26	65.00%	0.111	IPR013098 (PFAM); IPR013783 (G3DSA:2.60.40.GENE3D); IPR013783 (G3DSA:2.60.40.GENE3D); PTHR10489 (PANTHER); PTHR10489:SF20 (PANTHER); IPR007110 (PROSITE_PROFILES); IPR007110 (PROSITE_PROFILES); SSF48726 (SUPERFAMILY); SSF48726 (SUPERFAMILY)
536	c149035_g1_i1_len_396_path_374_0_395_1	22	ankyrin repeat domain-containing protein 16-like	132	7.57E-30	63.50%	0.241	IPR002110 (SMART); IPR002110 (PFAM); IPR020683 (PFAM); IPR020683 (G3DSA:1.25.40.GENE3D); PTHR24181:SF20 (PANTHER); PTHR24181 (PANTHER); IPR020683 (PROSITE_PROFILES); IPR002110 (PROSITE_PROFILES); IPR002110 (PROSITE_PROFILES); IPR020683 (SUPERFAMILY)
537	c149055_g1_i1_len_728_path_706_0_727_5	11	endothelin-converting enzyme 1 isoform x2	242	1.73E-41	73.60%	0.146	IPR000718 (PANTHER); IPR029734 (PTHR11733:PANTHER); CYTOPLASMIC_DOMAIN (PHOBIUS); NON_CYTOPLASMIC_DOMAIN (PHOBIUS); TRANSMEMBRANE (PHOBIUS); TRANSMEMBRANE (PHOBIUS); NON_CYTOPLASMIC_DOMAIN (PHOBIUS); SSF55486 (SUPERFAMILY); TMhelix (TMHMM); TMhelix (TMHMM)

538	c149086_g1_i1_len_382_path_360_0_381_4	4	zinc finger protein 214 isoform x3	127	1.09E-31	68.00%	0.108	IPR015880 (SMART); IPR013087 (G3DSA:3.30.160.GENE3D); IPR013087 (G3DSA:3.30.160.GENE3D); PF13465 (PFAM); IPR013087 (G3DSA:3.30.160.GENE3D); PTHR24409 (PANTHER); IPR007087 (PROSITE_PATTERNS); IPR007087 (PROSITE_PATTERNS); IPR007087 (PROSITE_PATTERNS); IPR007087 (PROSITE_PATTERNS); IPR007087 (PROSITE_PROFILES); IPR007087 (PROSITE_PROFILES); IPR007087 (PROSITE_PROFILES); IPR007087 (PROSITE_PROFILES); SSF57667 (SUPERFAMILY); SSF57667 (SUPERFAMILY)
539	c149118_g1_i1_len_1347_path_1_0_1346_5	71	low quality protein: hemocytin	449	4.38E-37	48.40%	0.1	IPR025155 (PFAM); IPR008979 (G3DSA:2.60.120.GENE3D); IPR000421 (PFAM); PTHR24543 (PANTHER); IPR000421 (PROSITE_PROFILES); IPR008979 (SUPERFAMILY)
540	c149154_g1_i1_len_944_path_945_0_747_1693_748_943_2	52	zinc finger protein 275	314	1.03E-20	57.50%	0.112	IPR015880 (SMART); IPR007087 (PFAM); PF13894 (PFAM); IPR013087 (G3DSA:3.30.160.GENE3D); IPR013087 (G3DSA:3.30.160.GENE3D); PF13913 (PFAM); PTHR24409 (PANTHER); IPR007087 (PROSITE_PATTERNS); IPR007087 (PROSITE_PATTERNS); IPR007087 (PROSITE_PATTERNS); IPR007087 (PROSITE_PATTERNS); IPR007087 (PROSITE_PROFILES); IPR007087 (PROSITE_PROFILES); IPR007087 (PROSITE_PROFILES); IPR007087 (PROSITE_PROFILES); SSF57667 (SUPERFAMILY); SSF57667 (SUPERFAMILY)
541	c149176_g1_i1_len_1191_path_1169_0_1190_5	35	amyloid beta a4 protein	397	8.92E-145	70.00%	0.1	IPR008197 (PRINTS); IPR002223 (PRINTS); IPR002223 (SMART); IPR006150 (SMART); IPR008197 (SMART); IPR000716 (SMART); IPR000716 (G3DSA:4.10.800.GENE3D); IPR004094 (PFAM); IPR000716 (PFAM); IPR028150 (PFAM); IPR008197 (G3DSA:4.10.75.GENE3D); IPR002223 (G3DSA:4.10.410.GENE3D); IPR002223 (PFAM); IPR004094 (G3DSA:2.10.22.GENE3D); IPR008197 (PFAM); PTHR19441:SF10 (PANTHER); PTHR19441 (PANTHER); IPR000716 (PROSITE_PATTERNS); IPR000716 (PROSITE_PATTERNS); IPR020901 (PROSITE_PATTERNS); IPR000716 (PROSITE_PROFILES); IPR000716 (PROSITE_PROFILES); IPR004094 (PROSITE_PROFILES); IPR008197 (PROSITE_PROFILES); IPR002223 (PROSITE_PROFILES); IPR011061 (SUPERFAMILY); IPR000716 (SUPERFAMILY); IPR000716 (SUPERFAMILY); IPR008197 (SUPERFAMILY); IPR002223 (SUPERFAMILY)
542	c149210_g1_i1_len_455_path_433_0_454_4	8	leucine-rich repeat and calponin homology domain-containing protein 1 isoform x2	152	1.67E-59	79.50%	0.112	IPR003591 (SMART); IPR001611 (PFAM); G3DSA:3.80.10.10 (GENE3D); IPR001611 (PFAM); PTHR23155 (PANTHER); PTHR23155:SF423 (PANTHER); IPR001611 (PROSITE_PROFILES); IPR001611 (PROSITE_PROFILES); IPR001611 (PROSITE_PROFILES); IPR001611 (PROSITE_PROFILES); SSF52058 (SUPERFAMILY)
543	c149380_g1_i1_len_647_path_625_0_646_5	22	leukocyte-antigen-related- isoform b	215	3.56E-83	78.30%	0.188	PR00014 (PRINTS); IPR003961 (SMART); IPR003961 (PFAM); IPR013783 (G3DSA:2.60.40.GENE3D); PTHR19134:SF199 (PANTHER); PTHR19134 (PANTHER); IPR003961 (PROSITE_PROFILES); IPR003961 (PROSITE_PROFILES); IPR003961 (PROSITE_PROFILES); IPR003961 (SUPERFAMILY); IPR003961 (SUPERFAMILY)
544	c14940_g2_i1_len_509_path_527_0_508_1	15	alpha-mannosidase 2x	170	3.86E-53	74.40%	0.103	IPR027291 (G3DSA:3.20.110.GENE3D); IPR000602 (PFAM); PTHR11607:SF4 (PANTHER); PTHR11607 (PANTHER); IPR011330 (SUPERFAMILY)
545	c149430_g1_i1_len_1191_path_1169_0_1190_1	78	low quality protein: thioredoxin domain-containing protein 11-like	397	1.84E-69	54.50%	0.105	IPR012336 (G3DSA:3.40.30.GENE3D); IPR013766 (PFAM); PTHR18929:SF61 (PANTHER); PTHR18929 (PANTHER); IPR012336 (PROSITE_PROFILES); IPR012336 (SUPERFAMILY); TMhelix (TMHMM)
546	c149551_g1_i1_len_749_path_727_0_748_5	12	receptor-type tyrosine-protein phosphatase f-like isoform x2	249	8.45E-19	54.70%	0.12	IPR000242 (PFAM); G3DSA:2.170.300.10 (GENE3D); IPR029021 (G3DSA:3.90.190.GENE3D); PTHR19134 (PANTHER); IPR013032 (PROSITE_PATTERNS); TRANSMEMBRANE (PHOBIUS); NON_CYTOPLASMIC_DOMAIN (PHOBIUS); CYTOPLASMIC_DOMAIN (PHOBIUS); IPR000242 (PROSITE_PROFILES); IPR009030 (SUPERFAMILY); IPR029021 (SUPERFAMILY); TMhelix (TMHMM)
547	c149674_g1_i1_len_356_path_1_0_355_3	7	low quality protein: myosin heavy muscle	119	1.71E-64	92.70%	0.103	Coil (COILS); Coil (COILS); IPR002928 (PFAM); PTHR13140 (PANTHER); PTHR13140:SF332 (PANTHER); SSF90257 (SUPERFAMILY)
548	c149677_g1_i1_len_373_path_1_0_372_1	4	reelin- partial	124	6.55E-26	59.70%	0.105	PTHR11841 (PANTHER)
549	c149808_g1_i1_len_1530_path_1508_0_1529_0	60	ga-binding protein subunit beta-2	510	8.15E-99	56.70%	0.538Y	Coil (COILS); IPR002110 (PRINTS); IPR002110 (SMART); IPR020683 (PFAM); IPR020683 (G3DSA:1.25.40.GENE3D); IPR020683 (G3DSA:1.25.40.GENE3D); PTHR24193:SF73 (PANTHER); PTHR24193 (PANTHER); IPR002110 (PROSITE_PROFILES); IPR002110 (PROSITE_PROFILES); IPR002110 (PROSITE_PROFILES); IPR020683 (PROSITE_PROFILES); SignalP-noTM (SIGNALP_EUK); IPR020683 (SUPERFAMILY)

550	c14985_g1_i1_len_1031_path_1009_0_629_1 639_630_1030_5	49	n-alpha-acetyltransferase auxiliary subunit	343	6.94E-123	68.10%	0.112	IPR011990 (G3DSA:1.25.40.GENE3D); IPR011990 (G3DSA:1.25.40.GENE3D); PTHR22767:SF3 (PANTHER); PTHR22767 (PANTHER); IPR013026 (PROSITE_PROFILES); SSF48452 (SUPERFAMILY)
551	c149861_g1_i1_len_373_path_1_0_372_4	39	alpha-carbonic anhydrase	124	5.21E-35	65.20%	0.143	IPR001148 (SMART); IPR001148 (PFAM); IPR001148 (G3DSA:3.10.200.GENE3D); IPR023561 (PANTHER); PTHR18952:SF92 (PANTHER); IPR001148 (PROSITE_PROFILES); IPR001148 (SUPERFAMILY)
552	c149863_g1_i1_len_591_path_32_0_590_5	30	homeotic protein spalt-major-like isoform x2	197	1.32E-59	60.10%	0.325	IPR015880 (SMART); IPR013087 (G3DSA:3.30.160.GENE3D); IPR013087 (G3DSA:3.30.160.GENE3D); PF13465 (PFAM); IPR013087 (G3DSA:3.30.160.GENE3D); IPR007087 (PFAM); PTHR23233:SF51 (PANTHER); PTHR23233 (PANTHER); IPR007087 (PROSITE_PATTERNS); IPR007087 (PROSITE_PATTERNS); SIGNAL_PEPTIDE_C_REGION (PHOBIUS); SIGNAL_PEPTIDE (PHOBIUS); SIGNAL_PEPTIDE_N_REGION (PHOBIUS); SIGNAL_PEPTIDE_H_REGION (PHOBIUS); NON_CYTOPLASMIC_DOMAIN (PHOBIUS); IPR007087 (PROSITE_PROFILES); IPR007087 (PROSITE_PROFILES); IPR007087 (PROSITE_PROFILES); SignalP-noTM (SIGNALP_GRAM_NEGATIVE); SignalP-TM (SIGNALP_GRAM_POSITIVE); SSF57667 (SUPERFAMILY); SSF57667 (SUPERFAMILY)
553	c149870_g1_i1_len_869_path_1_0_868_1	17	gastrula zinc finger	290	4.23E-148	66.00%	0.109	IPR015880 (SMART); IPR013087 (G3DSA:3.30.160.GENE3D); IPR007087 (PFAM); IPR013087 (G3DSA:3.30.160.GENE3D); IPR013087 (G3DSA:3.30.160.GENE3D); PF13465 (PFAM); PTHR24409 (PANTHER); IPR007087 (PROSITE_PATTERNS); IPR007087 (PROSITE_PATTERNS); IPR007087 (PROSITE_PATTERNS); IPR007087 (PROSITE_PATTERNS); IPR007087 (PROSITE_PATTERNS); IPR007087 (PROSITE_PATTERNS); IPR007087 (PROSITE_PATTERNS); IPR007087 (PROSITE_PATTERNS); IPR007087 (PROSITE_PROFILES); IPR007087 (PROSITE_PROFILES); IPR007087 (PROSITE_PROFILES); IPR007087 (PROSITE_PROFILES); IPR007087 (PROSITE_PROFILES); IPR007087 (PROSITE_PROFILES); IPR007087 (PROSITE_PROFILES); IPR007087 (PROSITE_PROFILES); IPR007087 (PROSITE_PROFILES); SSF57667 (SUPERFAMILY); SSF57667 (SUPERFAMILY); SSF57667 (SUPERFAMILY); SSF57667 (SUPERFAMILY); SSF57667 (SUPERFAMILY)
554	c149954_g1_i1_len_543_path_521_0_542_2	22	arylsulfatase b	181	6.30E-39	56.90%	0.098	IPR000917 (PFAM); IPR017849 (G3DSA:3.40.720.GENE3D); G3DSA:3.30.1120.10 (GENE3D); PTHR10342:SF196 (PANTHER); PTHR10342 (PANTHER); IPR017850 (SUPERFAMILY)
555	c150145_g1_i1_len_471_path_1_0_470_1	12	probable flavin-containing monoamine oxidase a	157	5.16E-51	71.60%	0.17	G3DSA:1.10.405.10 (GENE3D); G3DSA:3.50.50.60 (GENE3D); PF13450 (PFAM); PTHR10742 (PANTHER); PTHR10742:SF217 (PANTHER); TRANSMEMBRANE (PHOBIUS); CYTOPLASMIC_DOMAIN (PHOBIUS); NON_CYTOPLASMIC_DOMAIN (PHOBIUS); SSF51905 (SUPERFAMILY); TMhelix (TMHMM)
556	c15023_g1_i1_len_169_path_1_0_168_0	2	type-2 ice-structuring isoform x2	57	4.19E-07	57.50%	0.103	IPR001304 (PFAM); IPR016186 (G3DSA:3.10.100.GENE3D); IPR001304 (PROSITE_PROFILES); IPR016187 (SUPERFAMILY)
557	c150262_g1_i1_len_308_path_1_0_307_3	4	upf0454 protein c12orf49 homolog	103	6.09E-34	68.80%	0.517Y	IPR019352 (PFAM); IPR019352 (PANTHER); SIGNAL_PEPTIDE_H_REGION (PHOBIUS); NON_CYTOPLASMIC_DOMAIN (PHOBIUS); SIGNAL_PEPTIDE (PHOBIUS); SIGNAL_PEPTIDE_N_REGION (PHOBIUS); SIGNAL_PEPTIDE_C_REGION (PHOBIUS); SignalP-noTM (SIGNALP_GRAM_NEGATIVE); SignalP-TM (SIGNALP_GRAM_POSITIVE); SignalP-TM (SIGNALP_EUK); TMhelix (TMHMM)
558	c150386_g1_i1_len_385_path_363_0_384_5	6	zinc finger protein 225	128	3.27E-31	61.20%	0.111	IPR015880 (SMART); PF13465 (PFAM); PF13894 (PFAM); IPR013087 (G3DSA:3.30.160.GENE3D); PTHR24409 (PANTHER); IPR007087 (PROSITE_PATTERNS); IPR007087 (PROSITE_PATTERNS); IPR007087 (PROSITE_PROFILES); IPR007087 (PROSITE_PROFILES); IPR007087 (PROSITE_PROFILES); SSF57667 (SUPERFAMILY); SSF57667 (SUPERFAMILY)
559	c150418_g1_i1_len_361_path_1_0_360_3	7	von willebrand factor type egf and pentraxin domain-containing protein 1 isoform x1	121	4.59E-40	72.00%	0.116	IPR000436 (SMART); G3DSA:2.10.70.10 (GENE3D); IPR000436 (PFAM); G3DSA:2.10.70.10 (GENE3D); PTHR19325 (PANTHER); PTHR19325:SF340 (PANTHER); IPR000436 (PROSITE_PROFILES); IPR000436 (PROSITE_PROFILES); IPR000436 (SUPERFAMILY); IPR000436 (SUPERFAMILY); IPR000436 (SUPERFAMILY)
560	c150530_g1_i1_len_462_path_1_0_461_1	10	lysosomal pro-x carboxypeptidase	154	2.07E-69	78.00%	0.101	IPR029058 (G3DSA:3.40.50.GENE3D); IPR008758 (PFAM); IPR008758 (PANTHER); PTHR11010:SF11 (PANTHER); IPR029058 (SUPERFAMILY)
561	c150632_g1_i1_len_534_path_1_0_77_79_78 _305_79_306_533_4	10	ubiquitin (ribosomal protein l40)	178	5.88E-100	90.60%	0.128	IPR000626 (SMART); G3DSA:3.10.20.90 (GENE3D); G3DSA:3.10.20.90 (GENE3D); IPR000626 (PFAM); PTHR10666 (PANTHER); IPR000626 (PROSITE_PROFILES); IPR000626 (PROSITE_PROFILES); IPR029071 (SUPERFAMILY); IPR029071 (SUPERFAMILY)

562	c15064_g1_i1_len_497_path_607_0_496_4	15	aael017480- partial	166	2.02E-27	56.40%	0.27	IPR002110 (PRINTS); IPR002110 (SMART); IPR020683 (G3DSA:1.25.40.GENE3D); IPR002110 (PFAM); IPR020683 (PFAM); PTHR24198 (PANTHER); IPR002110 (PROSITE_PROFILES); IPR002110 (PROSITE_PROFILES); IPR020683 (PROSITE_PROFILES); IPR002110 (PROSITE_PROFILES); IPR020683 (SUPERFAMILY)
563	c150793_g1_i1_len_776_path_754_0_775_1	18	glycoprotein-n-acetylgalactosamine 3-beta-galactosyltransferase 1	259	8.70E-93	75.10%	0.1	IPR003378 (PFAM); PTHR23033 (PANTHER)
566	c1508_g1_i1_len_913_path_891_0_912_2	48	sortilin-related receptor-like	304	2.55E-115	71.30%	0.121	IPR006581 (SMART); G3DSA:2.130.10.140 (GENE3D); IPR002860 (PFAM); PTHR12106:SF7 (PANTHER); PTHR12106 (PANTHER); SSF110296 (SUPERFAMILY)
564	c150868_g1_i1_len_492_path_470_0_491_4	11	receptor-type tyrosine-protein phosphatase v-like	164	1.32E-09	64.70%	0.203	IPR029021 (G3DSA:3.90.190.GENE3D); PTHR19134 (PANTHER); CYTOPLASMIC_DOMAIN (PHOBIUS); NON_CYTOPLASMIC_DOMAIN (PHOBIUS); TRANSMEMBRANE (PHOBIUS); IPR000242 (PROSITE_PROFILES); IPR029021 (SUPERFAMILY); TMhelix (TMHMM)
565	c150892_g1_i1_len_850_path_864_0_849_1	24	xanthine dehydrogenase isoform x1	283	1.54E-119	76.30%	0.106	IPR008274 (G3DSA:3.30.365.GENE3D); IPR008274 (G3DSA:3.30.365.GENE3D); IPR008274 (G3DSA:3.30.365.GENE3D); IPR008274 (PFAM); PTHR11908 (PANTHER); PTHR11908:SF32 (PANTHER); IPR008274 (SUPERFAMILY)
567	c150943_g1_i1_len_584_path_562_0_583_3	10	gamma-glutamyltransferase 7	195	5.69E-32	67.00%	0.201	IPR000101 (PRINTS); IPR000101 (PFAM); IPR000101 (PANTHER); PTHR11686:SF18 (PANTHER); CYTOPLASMIC_DOMAIN (PHOBIUS); TRANSMEMBRANE (PHOBIUS); NON_CYTOPLASMIC_DOMAIN (PHOBIUS); TRANSMEMBRANE (PHOBIUS); NON_CYTOPLASMIC_DOMAIN (PHOBIUS); IPR029055 (SUPERFAMILY); TMhelix (TMHMM); TMhelix (TMHMM); TMhelix (TMHMM)
568	c150946_g1_i1_len_914_path_892_0_913_1	18	vascular endothelial growth factor receptor 1	305	4.51E-33	46.40%	0.113	IPR003599 (SMART); IPR003598 (SMART); IPR013098 (PFAM); IPR013783 (G3DSA:2.60.40.GENE3D); IPR013783 (G3DSA:2.60.40.GENE3D); IPR013783 (G3DSA:2.60.40.GENE3D); PF13895 (PFAM); PTHR10489:SF518 (PANTHER); PTHR10489 (PANTHER); IPR007110 (PROSITE_PROFILES); IPR007110 (PROSITE_PROFILES); SSF48726 (SUPERFAMILY); SSF48726 (SUPERFAMILY); SSF48726 (SUPERFAMILY)
569	c15106_g2_i1_len_580_path_558_0_579_0	9	zinc finger protein 729- partial	194	2.58E-24	57.20%	0.098	IPR015880 (SMART); PF13912 (PFAM); IPR007087 (PFAM); PF13465 (PFAM); IPR013087 (G3DSA:3.30.160.GENE3D); IPR013087 (G3DSA:3.30.160.GENE3D); IPR013087 (G3DSA:3.30.160.GENE3D); PTHR24409 (PANTHER); IPR007087 (PROSITE_PATTERNS); IPR007087 (PROSITE_PATTERNS); IPR007087 (PROSITE_PATTERNS); IPR007087 (PROSITE_PATTERNS); IPR007087 (PROSITE_PROFILES); IPR007087 (PROSITE_PROFILES); IPR007087 (PROSITE_PROFILES); SSF57667 (SUPERFAMILY); SSF57667 (SUPERFAMILY); SSF57667 (SUPERFAMILY)
570	c151081_g1_i1_len_826_path_804_0_825_2	66	insulin-like growth factor-binding protein 7 precursor	275	4.97E-74	58.90%	0.544Y	IPR000867 (SMART); IPR003598 (SMART); IPR002350 (SMART); G3DSA:3.30.60.30 (GENE3D); IPR013098 (PFAM); IPR000867 (PFAM); IPR002350 (PFAM); IPR013783 (G3DSA:2.60.40.GENE3D); IPR011390 (PANTHER); PTHR14186:SF9 (PANTHER); NON_CYTOPLASMIC_DOMAIN (PHOBIUS); SIGNAL_PEPTIDE_N_REGION (PHOBIUS); SIGNAL_PEPTIDE_H_REGION (PHOBIUS); SIGNAL_PEPTIDE_C_REGION (PHOBIUS); SIGNAL_PEPTIDE (PHOBIUS); IPR002350 (PROSITE_PROFILES); IPR000867 (PROSITE_PROFILES); IPR007110 (PROSITE_PROFILES); SignalP-TM (SIGNALP_EUK); SSF100895 (SUPERFAMILY); SSF48726 (SUPERFAMILY); IPR009030 (SUPERFAMILY)
571	c15117_g3_i1_len_639_path_617_0_638_4	25	---NA---	213			0.149	TRANSMEMBRANE (PHOBIUS); TRANSMEMBRANE (PHOBIUS); NON_CYTOPLASMIC_DOMAIN (PHOBIUS); CYTOPLASMIC_DOMAIN (PHOBIUS); NON_CYTOPLASMIC_DOMAIN (PHOBIUS); TMhelix (TMHMM)
572	c151198_g1_i1_len_945_path_1_0_944_1	38	lachesin	315	6.82E-139	81.00%	0.374Y	IPR013106 (SMART); IPR003599 (SMART); IPR003598 (SMART); IPR013098 (PFAM); IPR016243 (PIRSF); IPR013783 (G3DSA:2.60.40.GENE3D); IPR013783 (G3DSA:2.60.40.GENE3D); PF13895 (PFAM); PTHR19831:SF43 (PANTHER); PTHR19831 (PANTHER); SIGNAL_PEPTIDE_C_REGION (PHOBIUS); SIGNAL_PEPTIDE_H_REGION (PHOBIUS); SIGNAL_PEPTIDE (PHOBIUS); NON_CYTOPLASMIC_DOMAIN (PHOBIUS); SIGNAL_PEPTIDE_N_REGION (PHOBIUS); IPR007110 (PROSITE_PROFILES); IPR007110 (PROSITE_PROFILES); IPR007110 (PROSITE_PROFILES); SSF48726 (SUPERFAMILY); SSF48726 (SUPERFAMILY); SSF48726 (SUPERFAMILY); TMhelix (TMHMM)
573	c151330_g1_i1_len_385_path_1_0_384_1	9	collagen alpha-5 chain	128	9.60E-18	58.20%	0.099	IPR008160 (PFAM); PTHR24023 (PANTHER); PTHR24023:SF379 (PANTHER)
574	c151391_g1_i1_len_806_path_1_0_805_1	33	follistatin-related protein 5-like	269	1.32E-160	88.30%	0.121	PTHR10913 (PANTHER); PTHR10913:SF14 (PANTHER)

575	c151443_g1_i1_len_477_path_455_0_476_0	24	heat shock 70 kda protein 14	159	1.95E-31	54.90%	0.125	IPR029047 (G3DSA:2.60.34.GENE3D); IPR013126 (PFAM); G3DSA:3.30.420.40 (GENE3D); PTHR19375:SF158 (PANTHER); PTHR19375 (PANTHER); IPR018181 (PROSITE_PATTERNS); IPR029047 (SUPERFAMILY); SSF53067 (SUPERFAMILY)
576	c151594_g1_i1_len_510_path_1_0_509_3	12	zinc finger protein 777- partial	170	1.25E-13	73.90%	0.122	IPR015880 (SMART); PF13465 (PFAM); IPR013087 (G3DSA:3.30.160.GENE3D); IPR013087 (G3DSA:3.30.160.GENE3D); PTHR24409 (PANTHER); IPR007087 (PROSITE_PATTERNS); IPR007087 (PROSITE_PATTERNS); IPR007087 (PROSITE_PROFILES); IPR007087 (PROSITE_PROFILES); SSF57667 (SUPERFAMILY)
577	c151693_g1_i1_len_1188_path_1166_0_1187_1	22	protein flightless-1	396	0	86.20%	0.102	Coil (COILS); IPR007122 (PRINTS); IPR007122 (SMART); IPR001611 (PFAM); IPR029006 (G3DSA:3.40.20.GENE3D); IPR007123 (PFAM); G3DSA:3.80.10.10 (GENE3D); IPR029006 (G3DSA:3.40.20.GENE3D); IPR029919 (PTHR11977:PANTHER); IPR007122 (PANTHER); SSF55753 (SUPERFAMILY); SSF55753 (SUPERFAMILY); SSF52058 (SUPERFAMILY)
578	c151706_g1_i1_len_260_path_1_0_259_2	7	carboxypeptidase b-like	86	8.08E-28	76.20%	0.125	IPR000834 (PFAM); G3DSA:3.40.630.10 (GENE3D); PTHR11705 (PANTHER); SSF53187 (SUPERFAMILY)
579	c151866_g1_i1_len_761_path_12_0_760_0	19	apolipophorin precursor	254	5.73E-41	51.60%	0.111	IPR014853 (SMART); IPR001846 (PFAM); IPR014853 (PFAM); PTHR23361 (PANTHER); PTHR23361:SF13 (PANTHER); IPR001846 (PROSITE_PROFILES)
580	c151873_g1_i1_len_261_path_1_0_260_4	6	adamts-like protein 4 isoform x1	87	4.05E-31	57.20%	0.119	IPR000884 (SMART); G3DSA:2.20.100.10 (GENE3D); IPR000884 (PFAM); PTHR13723 (PANTHER); IPR000884 (PROSITE_PROFILES); IPR000884 (SUPERFAMILY); IPR000884 (SUPERFAMILY)
581	c15193_g1_i1_len_393_path_371_0_392_5	9	serine threonine-protein kinase 17b-like	131	2.78E-39	82.20%	0.411Y	IPR000719 (PFAM); G3DSA:1.10.510.10 (GENE3D); PTHR24342 (PANTHER); IPR000719 (PROSITE_PROFILES); IPR011009 (SUPERFAMILY)
582	c15193_g2_i1_len_177_path_895_0_176_4	2	serine threonine-protein kinase 17a-like	59	2.18E-28	94.00%	0.101	IPR000719 (PFAM); G3DSA:1.10.510.10 (GENE3D); PTHR24342 (PANTHER); IPR008271 (PROSITE_PATTERNS); IPR000719 (PROSITE_PROFILES); IPR011009 (SUPERFAMILY)
583	c151946_g1_i1_len_572_path_1_0_571_4	9	low quality protein: zinc finger protein 233	191	5.90E-47	69.20%	0.165	IPR015880 (SMART); IPR013087 (G3DSA:3.30.160.GENE3D); IPR013087 (G3DSA:3.30.160.GENE3D); PF13465 (PFAM); IPR013087 (G3DSA:3.30.160.GENE3D); PTHR24409 (PANTHER); PTHR24409:SF13 (PANTHER); IPR007087 (PROSITE_PATTERNS); IPR007087 (PROSITE_PATTERNS); IPR007087 (PROSITE_PATTERNS); IPR007087 (PROSITE_PATTERNS); IPR007087 (PROSITE_PATTERNS); IPR007087 (PROSITE_PROFILES); IPR007087 (PROSITE_PROFILES); IPR007087 (PROSITE_PROFILES); IPR007087 (PROSITE_PROFILES); IPR007087 (PROSITE_PROFILES); SSF57667 (SUPERFAMILY); SSF57667 (SUPERFAMILY); SSF57667 (SUPERFAMILY)
584	c151970_g1_i1_len_449_path_1_0_448_3	8	cytokine receptor	150	7.49E-39	69.60%	0.113	IPR003961 (SMART); IPR003961 (PFAM); IPR013783 (G3DSA:2.60.40.GENE3D); PTHR10489 (PANTHER); IPR003961 (PROSITE_PROFILES); IPR003961 (PROSITE_PROFILES); IPR003961 (SUPERFAMILY)
585	c151998_g1_i1_len_442_path_1_0_441_1	12	crustacean cardioactive peptide	147	1.67E-14	64.80%	0.102	IPR024276 (PFAM); NON_CYTOPLASMIC_DOMAIN (PHOBIUS); CYTOPLASMIC_DOMAIN (PHOBIUS); TRANSMEMBRANE (PHOBIUS); IPR024276 (PRODOM); TMhelix (TMHMM)
586	c152186_g1_i1_len_603_path_581_0_602_5	27	gastrula zinc finger isoform x1	201	2.05E-64	68.20%	0.105	IPR015880 (SMART); PF13465 (PFAM); IPR013087 (G3DSA:3.30.160.GENE3D); IPR013087 (G3DSA:3.30.160.GENE3D); IPR013087 (G3DSA:3.30.160.GENE3D); IPR013087 (G3DSA:3.30.160.GENE3D); PTHR24409 (PANTHER); IPR007087 (PROSITE_PATTERNS); IPR007087 (PROSITE_PATTERNS); IPR007087 (PROSITE_PATTERNS); IPR007087 (PROSITE_PATTERNS); IPR007087 (PROSITE_PATTERNS); IPR007087 (PROSITE_PATTERNS); IPR007087 (PROSITE_PROFILES); IPR007087 (PROSITE_PROFILES); IPR007087 (PROSITE_PROFILES); IPR007087 (PROSITE_PROFILES); IPR007087 (PROSITE_PROFILES); IPR007087 (PROSITE_PROFILES); IPR007087 (PROSITE_PROFILES); SSF57667 (SUPERFAMILY); SSF57667 (SUPERFAMILY); SSF57667 (SUPERFAMILY); SSF57667 (SUPERFAMILY)
587	c15222_g1_i1_len_3156_path_3184_0_3155_2	356	agap000560-pa-like protein	1052	0	77.80%	0.166	IPR003593 (SMART); G3DSA:1.10.8.60 (GENE3D); IPR027417 (G3DSA:3.40.50.GENE3D); IPR003959 (PFAM); IPR005936 (TIGRFAM); IPR000642 (PFAM); PTHR23076 (PANTHER); PTHR23076:SF47 (PANTHER); NON_CYTOPLASMIC_DOMAIN (PHOBIUS); NON_CYTOPLASMIC_DOMAIN (PHOBIUS); TRANSMEMBRANE (PHOBIUS); CYTOPLASMIC_DOMAIN (PHOBIUS); TRANSMEMBRANE (PHOBIUS); TRANSMEMBRANE (PHOBIUS); CYTOPLASMIC_DOMAIN (PHOBIUS); IPR005936 (HAMAP); IPR027417 (SUPERFAMILY); SSF140990 (SUPERFAMILY); TMhelix (TMHMM); TMhelix (TMHMM)

588	c152413_g1_i1_len_643_path_621_0_642_1	23	beta- -n-acetylgalactosaminyltransferase bre-4	214	1.78E-17	63.70%	0.099	Coil (COILS); IPR029044 (G3DSA:3.90.550.GENE3D); IPR027995 (PFAM); IPR003859 (PANTHER); IPR029044 (SUPERFAMILY)
589	c152413_g1_i1_len_643_path_621_0_642_2	23	beta- -n-acetylgalactosaminyltransferase bre- partial	214	5.41E-16	62.00%	0.14	IPR003859 (PRINTS); IPR029044 (G3DSA:3.90.550.GENE3D); IPR027791 (PFAM); IPR003859 (PANTHER); IPR029044 (SUPERFAMILY)
590	c15251_g2_i1_len_439_path_915_0_438_1	6	basement membrane-specific heparan sulfate proteoglycan core protein	146	4.33E-12	64.40%	0.201	IPR002172 (SMART); IPR002172 (PFAM); IPR002172 (G3DSA:4.10.400.GENE3D); PTHR10529:SF3 (PANTHER); PTHR10529 (PANTHER); IPR023415 (PROSITE_PATTERNS); IPR002172 (PROSITE_PROFILES); IPR002172 (SUPERFAMILY)
591	c152604_g1_i1_len_371_path_1_0_370_4	6	transforming growth factor-beta-induced protein ig-h3-like	124	1.07E-10	60.40%	0.581Y	IPR000782 (G3DSA:2.30.180.GENE3D); IPR000782 (PFAM); PTHR10900 (PANTHER); IPR000782 (PROSITE_PROFILES); SignalP-noTM (SIGNALP_EUK); IPR000782 (SUPERFAMILY)
592	c15261_g1_i1_len_157_path_135_0_156_3	3	laminin subunit alpha	53	1.58E-16	78.40%	0.102	IPR002049 (SMART); IPR002049 (PFAM); G3DSA:2.10.25.10 (GENE3D); PTHR10574 (PANTHER); PTHR10574:SF39 (PANTHER); IPR002049 (PROSITE_PROFILES); SSF57196 (SUPERFAMILY)
593	c152630_g1_i1_len_378_path_1_0_377_0	34	blastula protease 10-like	126	3.03E-16	50.10%	0.104	IPR000859 (PFAM); IPR000859 (G3DSA:2.60.120.GENE3D); IPR000859 (PROSITE_PROFILES); IPR000859 (SUPERFAMILY)
594	c152663_g1_i1_len_248_path_1_0_247_1	2	oocyte zinc finger protein xlcof22	83	6.48E-17	60.90%	0.097	IPR015880 (SMART); IPR013087 (G3DSA:3.30.160.GENE3D); IPR013087 (G3DSA:3.30.160.GENE3D); IPR007087 (PFAM); PTHR24409:SF14 (PANTHER); PTHR24409 (PANTHER); IPR007087 (PROSITE_PATTERNS); IPR007087 (PROSITE_PATTERNS); IPR007087 (PROSITE_PROFILES); IPR007087 (PROSITE_PROFILES); SSF57667 (SUPERFAMILY)
595	c152721_g1_i1_len_302_path_280_0_301_0	13	xanthine dehydrogenase isoform x1	101	2.28E-26	74.30%	0.098	IPR000674 (PFAM); IPR000674 (G3DSA:3.90.1170.GENE3D); PTHR11908:SF32 (PANTHER); PTHR11908 (PANTHER); IPR000674 (SUPERFAMILY)
596	c152800_g1_i1_len_630_path_608_0_629_0	12	ring finger and spry domain-containing protein 1-like	210	2.61E-108	82.30%	0.127	PTHR13363 (PANTHER); IPR016024 (SUPERFAMILY); IPR013320 (SUPERFAMILY)
597	c152840_g1_i1_len_327_path_1_0_326_5	6	xdh protein	109	6.80E-21	61.60%	0.103	IPR000674 (SMART); IPR000674 (PFAM); IPR000674 (G3DSA:3.90.1170.GENE3D); IPR008274 (G3DSA:3.30.365.GENE3D); PTHR11908 (PANTHER); PTHR11908:SF65 (PANTHER); IPR000674 (SUPERFAMILY)
598	c152885_g1_i1_len_468_path_446_0_467_5	11	#NAME?	156	3.61E-74	83.90%	0.131	G3DSA:3.40.50.2000 (GENE3D); IPR001830 (PFAM); PTHR10788 (PANTHER); PTHR10788:SF6 (PANTHER); SSF53756 (SUPERFAMILY)
599	c152888_g1_i1_len_366_path_344_0_365_1	6	zinc finger protein 850-like	122	2.41E-18	55.00%	0.099	IPR015880 (SMART); PF13465 (PFAM); IPR013087 (G3DSA:3.30.160.GENE3D); IPR013087 (G3DSA:3.30.160.GENE3D); IPR013087 (G3DSA:3.30.160.GENE3D); IPR007087 (PFAM); PTHR24409 (PANTHER); PTHR24409:SF14 (PANTHER); IPR007087 (PROSITE_PATTERNS); IPR007087 (PROSITE_PATTERNS); IPR007087 (PROSITE_PATTERNS); IPR007087 (PROSITE_PATTERNS); IPR007087 (PROSITE_PROFILES); IPR007087 (PROSITE_PROFILES); IPR007087 (PROSITE_PROFILES); SSF57667 (SUPERFAMILY); SSF57667 (SUPERFAMILY)
600	c15291_g1_i1_len_794_path_628_0_627_155 1_628_793_4	24	sortilin-related l(dlr class) a repeats-containing-like isoform x2	265	2.16E-17	55.40%	0.278	Coil (COILS); Coil (COILS); IPR002172 (PRINTS); IPR002172 (SMART); IPR002172 (G3DSA:4.10.400.GENE3D); IPR002172 (PFAM); IPR002172 (G3DSA:4.10.400.GENE3D); PTHR10529 (PANTHER); IPR023415 (PROSITE_PATTERNS); IPR023415 (PROSITE_PATTERNS); IPR002172 (PROSITE_PROFILES); IPR002172 (PROSITE_PROFILES); IPR002172 (SUPERFAMILY); IPR002172 (SUPERFAMILY)
601	c152964_g1_i1_len_429_path_1_0_428_3	9	alpha platelet-derived growth factor receptor	143	2.84E-19	46.60%	0.111	PF13895 (PFAM); IPR013783 (G3DSA:2.60.40.GENE3D); SSF48726 (SUPERFAMILY)
602	c153145_g1_i1_len_648_path_626_0_647_3	11	glyceraldehyde-3-phosphate dehydrogenase	216	2.97E-137	92.60%	0.102	IPR020831 (PRINTS); IPR020828 (SMART); IPR020828 (PFAM); IPR016040 (G3DSA:3.40.50.GENE3D); IPR020829 (PFAM); G3DSA:3.30.360.10 (GENE3D); IPR020831 (PANTHER); IPR020830 (PROSITE_PATTERNS); SSF51735 (SUPERFAMILY); SSF55347 (SUPERFAMILY)
603	c153149_g1_i1_len_276_path_1_0_275_3	6	---NA---	92			0.576Y	IPR012674 (G3DSA:2.40.128.GENE3D); SIGNAL_PEPTIDE_H_REGION (PHOBIUS); SIGNAL_PEPTIDE (PHOBIUS); NON_CYTOPLASMIC_DOMAIN (PHOBIUS); SIGNAL_PEPTIDE_N_REGION (PHOBIUS); SIGNAL_PEPTIDE_C_REGION (PHOBIUS); SignalP-TM (SIGNALP_EUK); IPR011038 (SUPERFAMILY); TMhelix (TMHMM)

604	c153194_g1_i1_len_461_path_51_0_460_4	8	zinc finger protein 420-like	154	3.02E-30	61.20%	0.101	IPR015880 (SMART); IPR013087 (G3DSA:3.30.160.GENE3D); IPR007087 (PFAM); PF13894 (PFAM); IPR013087 (G3DSA:3.30.160.GENE3D); PF13465 (PFAM); PTHR24409 (PANTHER); PTHR24409:SF13 (PANTHER); IPR007087 (PROSITE_PATTERNS); IPR007087 (PROSITE_PATTERNS); IPR007087 (PROSITE_PATTERNS); IPR007087 (PROSITE_PATTERNS); IPR007087 (PROSITE_PATTERNS); IPR007087 (PROSITE_PROFILES); IPR007087 (PROSITE_PROFILES); IPR007087 (PROSITE_PROFILES); IPR007087 (PROSITE_PROFILES); IPR007087 (PROSITE_PROFILES); SSF57667 (SUPERFAMILY); SSF57667 (SUPERFAMILY); SSF57667 (SUPERFAMILY); SSF57667 (SUPERFAMILY)
605	c153235_g1_i1_len_470_path_1_0_469_4	7	a disintegrin and metalloproteinase with thrombospondin motifs 3-like	157	6.49E-65	69.00%	0.126	PR01705 (PRINTS); IPR000884 (SMART); G3DSA:2.20.100.10 (GENE3D); IPR000884 (PFAM); PTHR13723 (PANTHER); IPR000884 (PROSITE_PROFILES); IPR000884 (SUPERFAMILY)
606	c153266_g1_i1_len_397_path_375_0_396_1	5	cysteine-rich motor neuron 1 protein	132	1.76E-09	54.90%	0.157	SIGNAL_PEPTIDE (PHOBIUS); SIGNAL_PEPTIDE_N_REGION (PHOBIUS); NON_CYTOPLASMIC_DOMAIN (PHOBIUS); SIGNAL_PEPTIDE_C_REGION (PHOBIUS); SIGNAL_PEPTIDE_H_REGION (PHOBIUS); TMhelix (TMHMM)
607	c153609_g1_i1_len_629_path_607_0_628_4	22	basement membrane-specific heparan sulfate proteoglycan core isoform x2	210	3.30E-93	78.50%	0.102	IPR013106 (SMART); IPR003598 (SMART); IPR003599 (SMART); IPR013783 (G3DSA:2.60.40.GENE3D); IPR013106 (PFAM); PF13895 (PFAM); PTHR23279 (PANTHER); PTHR23279:SF6 (PANTHER); IPR007110 (PROSITE_PROFILES); IPR007110 (PROSITE_PROFILES); SSF48726 (SUPERFAMILY); SSF48726 (SUPERFAMILY)
608	c153785_g1_i1_len_492_path_470_0_491_2	12	group xv phospholipase a2-like	164	2.20E-59	70.20%	0.119	IPR003386 (PFAM); PTHR11440 (PANTHER); PTHR11440:SF47 (PANTHER); IPR029058 (SUPERFAMILY)
609	c153924_g1_i1_len_552_path_600_0_551_4	10	zinc finger protein 271 (zinc finger protein 7) (zinc finger protein znfphex133) (epstein-barr virus-induced zinc finger protein) (znf-eb) (ct-zfp48) (zinc finger protein	184	5.00E-23	55.60%	0.121	IPR015880 (SMART); IPR007087 (PFAM); IPR013087 (G3DSA:3.30.160.GENE3D); PF13894 (PFAM); IPR013087 (G3DSA:3.30.160.GENE3D); IPR013087 (G3DSA:3.30.160.GENE3D); PTHR24409 (PANTHER); PTHR24409:SF14 (PANTHER); IPR007087 (PROSITE_PATTERNS); IPR007087 (PROSITE_PATTERNS); IPR007087 (PROSITE_PATTERNS); IPR007087 (PROSITE_PATTERNS); IPR007087 (PROSITE_PROFILES); IPR007087 (PROSITE_PROFILES); IPR007087 (PROSITE_PROFILES); IPR007087 (PROSITE_PROFILES); SSF57667 (SUPERFAMILY); SSF57667 (SUPERFAMILY); SSF57667 (SUPERFAMILY)
611	c15396_g1_i1_len_991_path_1_0_990_1	262	sulfakinin-like peptide	330	8.48E-16	65.70%	0.205	IPR013152 (PROSITE_PATTERNS); IPR013152 (PROSITE_PATTERNS); TRANSMEMBRANE (PHOBIUS); CYTOPLASMIC_DOMAIN (PHOBIUS); TRANSMEMBRANE (PHOBIUS); NON_CYTOPLASMIC_DOMAIN (PHOBIUS); CYTOPLASMIC_DOMAIN (PHOBIUS); TMhelix (TMHMM)
610	c153960_g1_i1_len_509_path_1_0_508_2	19	serine beta-lactamase-like protein mitochondrial isoform x2	169	1.01E-57	74.40%	0.107	Coil (COILS); IPR001466 (PFAM); IPR012338 (G3DSA:3.40.710.GENE3D); PTHR22935 (PANTHER); PTHR22935:SF64 (PANTHER); IPR012338 (SUPERFAMILY)
612	c154126_g1_i1_len_400_path_378_0_399_1	19	collagen alpha-5 chain	133	9.03E-12	66.10%	0.131	IPR008160 (PFAM); PTHR24023:SF381 (PANTHER); PTHR24023 (PANTHER); TRANSMEMBRANE (PHOBIUS); CYTOPLASMIC_DOMAIN (PHOBIUS); NON_CYTOPLASMIC_DOMAIN (PHOBIUS); TMhelix (TMHMM)
613	c154126_g1_i1_len_400_path_378_0_399_4	19	signal recognition protein	133	1.19E-07	54.00%	0.122	no IPS match
615	c1543_g1_i1_len_923_path_45_0_278_323_279_548_44_549_922_4	17	protein partial	308	8.80E-68	77.10%	0.106	IPR001478 (SMART); IPR001478 (G3DSA:2.30.42.GENE3D); IPR001478 (PFAM); IPR016313 (PANTHER); IPR001478 (PROSITE_PROFILES); IPR001478 (SUPERFAMILY)
616	c1543_g1_i2_len_653_path_45_0_278_44_279_652_4	11	low quality protein: protein lap4-like	218	5.86E-39	79.80%	0.106	IPR001478 (SMART); IPR001478 (PFAM); IPR001478 (G3DSA:2.30.42.GENE3D); IPR016313 (PANTHER); PTHR23119:SF28 (PANTHER); IPR001478 (PROSITE_PROFILES); IPR001478 (SUPERFAMILY)
614	c154383_g1_i1_len_347_path_1_0_346_2	6	collagen alpha-1 chain	115	4.32E-07	52.90%	0.103	IPR008160 (PFAM); PTHR24023 (PANTHER)
617	c154455_g1_i1_len_410_path_1_0_409_5	12	granzyme c	136	1.26E-23	61.10%	0.105	IPR001314 (PRINTS); IPR001254 (SMART); IPR001254 (PFAM); G3DSA:2.40.10.10 (GENE3D); PTHR24268 (PANTHER); IPR018114 (PROSITE_PATTERNS); IPR001254 (PROSITE_PROFILES); IPR009003 (SUPERFAMILY)
618	c154766_g1_i1_len_289_path_1_0_288_4	4	beta- -n-acetylgalactosaminyltransferase bre-4	96	3.66E-40	78.60%	0.104	IPR003859 (PRINTS); IPR029044 (G3DSA:3.90.550.GENE3D); IPR027791 (PFAM); IPR003859 (PANTHER); IPR029044 (SUPERFAMILY)
619	c154852_g1_i1_len_421_path_399_0_420_3	7	zinc finger protein 271 (zinc finger protein 7) (zinc finger protein znfphex133) (epstein-barr virus-induced zinc finger protein) (znf-eb) (ct-zfp48) (zinc finger protein	141	5.34E-18	63.20%	0.1	IPR015880 (SMART); IPR013087 (G3DSA:3.30.160.GENE3D); PF13465 (PFAM); IPR013087 (G3DSA:3.30.160.GENE3D); IPR013087 (G3DSA:3.30.160.GENE3D); PTHR24409 (PANTHER); IPR007087 (PROSITE_PATTERNS); IPR007087 (PROSITE_PATTERNS); IPR007087 (PROSITE_PATTERNS); IPR007087 (PROSITE_PROFILES); IPR007087 (PROSITE_PROFILES); IPR007087 (PROSITE_PROFILES); SSF57667 (SUPERFAMILY); SSF57667 (SUPERFAMILY)

620	c155425_g1_i1_len_352_path_330_0_351_2	5	zinc finger protein 431 isoform x2	117	4.88E-13	64.50%	0.126		IPR015880 (SMART); IPR007087 (PFAM); IPR013087 (G3DSA:3.30.160.GENE3D); IPR013087 (G3DSA:3.30.160.GENE3D); PF13465 (PFAM); PTHR24409 (PANTHER); PTHR24409:SF14 (PANTHER); IPR007087 (PROSITE_PATTERNS); IPR007087 (PROSITE_PATTERNS); IPR007087 (PROSITE_PROFILES); IPR007087 (PROSITE_PROFILES); IPR007087 (PROSITE_PROFILES); SSF57667 (SUPERFAMILY); SSF57667 (SUPERFAMILY)
621	c155528_g1_i1_len_591_path_569_0_590_1	9	epidermal growth factor receptor isoform x1	197	9.42E-105	87.40%	0.121		IPR006212 (SMART); IPR006211 (PFAM); G3DSA:2.10.220.10 (GENE3D); G3DSA:2.10.220.10 (GENE3D); IPR000494 (G3DSA:3.80.20.GENE3D); IPR000494 (PFAM); PTHR24416:SF95 (PANTHER); PTHR24416 (PANTHER); IPR009030 (SUPERFAMILY); SSF52058 (SUPERFAMILY)
622	c155529_g1_i1_len_587_path_565_0_586_1	10	serine protease snake	196	7.01E-26	52.50%	0.459	Y	IPR001314 (PRINTS); IPR001254 (SMART); IPR001254 (PFAM); G3DSA:2.40.10.10 (GENE3D); G3DSA:2.40.10.10 (GENE3D); PTHR24260 (PANTHER); IPR018114 (PROSITE_PATTERNS); IPR001254 (PROSITE_PROFILES); SignalP-noTM (SIGNALP_EUK); IPR009003 (SUPERFAMILY)
623	c155596_g1_i1_len_568_path_1_0_567_4	7	zinc finger protein 629- partial	189	2.33E-13	62.40%	0.096		IPR015880 (SMART); PF13912 (PFAM); PF13894 (PFAM); IPR013087 (G3DSA:3.30.160.GENE3D); IPR013087 (G3DSA:3.30.160.GENE3D); PTHR24409 (PANTHER); IPR007087 (PROSITE_PATTERNS); IPR007087 (PROSITE_PATTERNS); IPR007087 (PROSITE_PROFILES); IPR007087 (PROSITE_PROFILES); SSF57667 (SUPERFAMILY)
624	c155823_g1_i1_len_542_path_1_0_541_1	12	zinc finger protein 699-like	181	2.73E-31	56.70%	0.103		IPR015880 (SMART); IPR007087 (PFAM); IPR013087 (G3DSA:3.30.160.GENE3D); IPR013087 (G3DSA:3.30.160.GENE3D); IPR013087 (G3DSA:3.30.160.GENE3D); PTHR24409 (PANTHER); PTHR24409:SF14 (PANTHER); IPR007087 (PROSITE_PATTERNS); IPR007087 (PROSITE_PATTERNS); IPR007087 (PROSITE_PATTERNS); IPR007087 (PROSITE_PATTERNS); IPR007087 (PROSITE_PATTERNS); IPR007087 (PROSITE_PATTERNS); IPR007087 (PROSITE_PROFILES); IPR007087 (PROSITE_PROFILES); IPR007087 (PROSITE_PROFILES); IPR007087 (PROSITE_PROFILES); IPR007087 (PROSITE_PROFILES); IPR007087 (PROSITE_PROFILES); SSF57667 (SUPERFAMILY); SSF57667 (SUPERFAMILY); SSF57667 (SUPERFAMILY)
625	c155929_g1_i1_len_510_path_488_0_509_2	17	carboxypeptidase pm20d1	170	1.19E-11	80.30%	0.108		G3DSA:3.40.630.10 (GENE3D); NON_CYTOPLASMIC_DOMAIN (PHOBIUS); TRANSMEMBRANE (PHOBIUS); CYTOPLASMIC_DOMAIN (PHOBIUS); TMhelix (TMHMM)
626	c155979_g1_i1_len_481_path_459_0_480_0	9	tyramine beta-hydroxylase	161	2.64E-66	75.30%	0.164		IPR014784 (G3DSA:2.60.120.GENE3D); PF03712 (PFAM); IPR000945 (PANTHER); IPR028460 (PTHR10157:PANTHER); IPR008977 (SUPERFAMILY)
627	c155999_g1_i1_len_263_path_241_0_262_1	10	5 -nucleotidase-like	88	4.26E-21	70.20%	0.162		IPR029052 (G3DSA:3.60.21.GENE3D); IPR006179 (PANTHER); PTHR11575:SF21 (PANTHER); IPR006146 (PROSITE_PATTERNS); IPR029052 (SUPERFAMILY)
628	c156237_g1_i1_len_434_path_1_0_433_1	7	a disintegrin and metalloproteinase with thrombospondin motifs partial	145	3.04E-22	59.40%	0.137		IPR024079 (G3DSA:3.40.390.GENE3D); PTHR13723:SF160 (PANTHER); PTHR13723 (PANTHER); IPR001590 (PROSITE_PROFILES); SSF55486 (SUPERFAMILY)
629	c156465_g1_i1_len_324_path_302_0_323_4	7	arylsulfatase d	108	1.72E-20	58.60%	0.124		PF14707 (PFAM); G3DSA:3.30.1120.10 (GENE3D); PTHR10342 (PANTHER); PTHR10342:SF200 (PANTHER); IPR017850 (SUPERFAMILY)
630	c1565_g1_i1_len_1380_path_1_0_46_48_47_1379_2	88	zinc finger protein zpr1	460	2.35E-177	73.70%	0.185		IPR004457 (SMART); IPR004457 (TIGRFAM); IPR004457 (PFAM); PTHR10876:SF0 (PANTHER); PTHR10876 (PANTHER)
631	c1565_g1_i2_len_1372_path_1445_0_38_48_39_1371_0	91	zinc finger protein zpr1	458	1.25E-177	73.80%	0.126		IPR004457 (SMART); IPR004457 (TIGRFAM); IPR004457 (PFAM); PTHR10876 (PANTHER); PTHR10876:SF0 (PANTHER)
632	c1566_g1_i1_len_2494_path_3057_0_532_3589_533_2493_0	171	von willebrand factor a domain-containing protein partial	832	0	65.50%	0.117		PTHR21610 (PANTHER); PTHR21610:SF9 (PANTHER)
633	c1566_g1_i2_len_825_path_3057_0_532_25_533_824_0	47	von willebrand factor a domain-containing protein 8-like	275	2.71E-84	86.00%	0.117		PTHR21610:SF9 (PANTHER); PTHR21610 (PANTHER); TRANSMEMBRANE (PHOBIUS); CYTOPLASMIC_DOMAIN (PHOBIUS); NON_CYTOPLASMIC_DOMAIN (PHOBIUS); TMhelix (TMHMM)
634	c156720_g1_i1_len_641_path_1_0_640_3	10	fibroblast growth factor receptor-like 1	214	1.34E-40	59.70%	0.098		IPR003599 (SMART); IPR013098 (PFAM); IPR013783 (G3DSA:2.60.40.GENE3D); PTHR19890 (PANTHER); PTHR19890:SF5 (PANTHER); TRANSMEMBRANE (PHOBIUS); CYTOPLASMIC_DOMAIN (PHOBIUS); NON_CYTOPLASMIC_DOMAIN (PHOBIUS); IPR007110 (PROSITE_PROFILES); SSF48726 (SUPERFAMILY); TMhelix (TMHMM)
635	c156724_g1_i1_len_280_path_258_0_279_2	4	hemicentin-1-like	93	3.32E-14	59.10%	0.128		IPR013098 (PFAM); IPR013783 (G3DSA:2.60.40.GENE3D); IPR013783 (G3DSA:2.60.40.GENE3D); PTHR10489 (PANTHER); IPR007110 (PROSITE_PROFILES); SSF48726 (SUPERFAMILY)

636	c156732_g1_i1_len_537_path_515_0_536_2	16	complement c1q tumor necrosis factor-related protein 2	179	1.44E-27	61.50%	0.624Y	IPR001073 (PRINTS); IPR001073 (SMART); IPR001073 (PFAM); IPR008983 (G3DSA:2.60.120.GENE3D); PTHR22923 (PANTHER); PTHR22923:SF51 (PANTHER); SIGNAL_PEPTIDE (PHOBIUS); SIGNAL_PEPTIDE_C_REGION (PHOBIUS); SIGNAL_PEPTIDE_N_REGION (PHOBIUS); NON_CYTOPLASMIC_DOMAIN (PHOBIUS); SIGNAL_PEPTIDE_H_REGION (PHOBIUS); IPR001073 (PROSITE_PROFILES); SignalP-noTM (SIGNALP_EUK); SignalP-TM (SIGNALP_GRAM_POSITIVE); IPR008983 (SUPERFAMILY); TMhelix (TMHMM); TMhelix (TMHMM)
637	c156886_g1_i1_len_321_path_1_0_320_3	7	guanine nucleotide-binding protein subunit beta-5	107	1.56E-62	93.70%	0.123	IPR020472 (PRINTS); IPR001680 (SMART); IPR015943 (G3DSA:2.130.10.GENE3D); IPR001680 (PFAM); PTHR19850:SF12 (PANTHER); IPR016346 (PANTHER); IPR019775 (PROSITE_PATTERNS); IPR001680 (PROSITE_PROFILES); IPR001680 (PROSITE_PROFILES); IPR001680 (PROSITE_PROFILES); IPR017986 (PROSITE_PROFILES); IPR017986 (SUPERFAMILY)
638	c156990_g1_i1_len_249_path_1_0_248_4	4	tsa family protein	83	4.89E-27	78.10%	0.137	IPR000866 (PFAM); IPR012336 (G3DSA:3.40.30.GENE3D); PTHR10681:SF101 (PANTHER); PTHR10681 (PANTHER); IPR012336 (SUPERFAMILY)
639	c157041_g1_i1_len_371_path_349_0_370_1	27	14-3-3 protein	124	1.39E-58	85.90%	0.103	IPR000308 (PRINTS); IPR023410 (SMART); IPR023410 (PFAM); G3DSA:1.20.190.20 (GENE3D); IPR000308 (PANTHER); IPR023410 (SUPERFAMILY)
640	c157058_g1_i1_len_474_path_1_0_473_3	6	protein disulfide-isomerase a6-like	158	9.49E-22	52.70%	0.101	IPR012336 (G3DSA:3.40.30.GENE3D); IPR013766 (PFAM); IPR012336 (G3DSA:3.40.30.GENE3D); PTHR18929 (PANTHER); IPR017937 (PROSITE_PATTERNS); IPR012336 (PROSITE_PROFILES); IPR012336 (SUPERFAMILY); IPR012336 (SUPERFAMILY)
641	c15706_g1_i1_len_2510_path_2587_0_2509_0	543265	hemocyanin subunit type 2 precursor	837	0	73.10%	0.332	IPR013788 (PRINTS); IPR000896 (PFAM); IPR005203 (PFAM); IPR005204 (G3DSA:1.20.1370.GENE3D); IPR008922 (G3DSA:1.10.1280.GENE3D); IPR005204 (PFAM); IPR005203 (G3DSA:2.60.40.GENE3D); IPR013788 (PANTHER); IPR013788 (PROSITE_PATTERNS); IPR002227 (PROSITE_PATTERNS); IPR013788 (PROSITE_PATTERNS); CYTOPLASMIC_DOMAIN (PHOBIUS); TRANSMEMBRANE (PHOBIUS); NON_CYTOPLASMIC_DOMAIN (PHOBIUS); IPR005204 (SUPERFAMILY); IPR008922 (SUPERFAMILY); IPR014756 (SUPERFAMILY)
642	c157101_g1_i1_len_322_path_1_0_321_0	8	enolase 2-like	108	4.26E-43	81.20%	0.108	IPR000941 (PRINTS); IPR020810 (PFAM); IPR029065 (G3DSA:3.20.20.GENE3D); IPR000941 (PANTHER); IPR029065 (SUPERFAMILY)
643	c157129_g1_i1_len_717_path_695_0_716_3	46	low-density lipoprotein receptor-related protein 2	239	1.87E-39	57.10%	0.104	IPR002172 (PRINTS); IPR002172 (SMART); IPR002172 (PFAM); IPR002172 (G3DSA:4.10.400.GENE3D); G3DSA:4.10.1220.10 (GENE3D); IPR002172 (G3DSA:4.10.400.GENE3D); PTHR10529 (PANTHER); IPR023415 (PROSITE_PATTERNS); IPR023415 (PROSITE_PATTERNS); IPR023415 (PROSITE_PATTERNS); IPR023415 (PROSITE_PATTERNS); IPR002172 (PROSITE_PROFILES); IPR002172 (PROSITE_PROFILES); IPR002172 (PROSITE_PROFILES); IPR002172 (PROSITE_PROFILES); IPR002172 (SUPERFAMILY); IPR002172 (SUPERFAMILY); IPR002172 (SUPERFAMILY); IPR002172 (SUPERFAMILY)
644	c157258_g1_i1_len_440_path_418_0_439_4	12	gastrula zinc finger	147	3.61E-52	69.10%	0.101	IPR015880 (SMART); IPR013087 (G3DSA:3.30.160.GENE3D); PF13465 (PFAM); IPR013087 (G3DSA:3.30.160.GENE3D); IPR013087 (G3DSA:3.30.160.GENE3D); PTHR24409 (PANTHER); IPR007087 (PROSITE_PATTERNS); IPR007087 (PROSITE_PATTERNS); IPR007087 (PROSITE_PATTERNS); IPR007087 (PROSITE_PATTERNS); IPR007087 (PROSITE_PATTERNS); IPR007087 (PROSITE_PROFILES); IPR007087 (PROSITE_PROFILES); IPR007087 (PROSITE_PROFILES); IPR007087 (PROSITE_PROFILES); IPR007087 (PROSITE_PROFILES); IPR007087 (PROSITE_PROFILES); SSF57667 (SUPERFAMILY); SSF57667 (SUPERFAMILY); SSF57667 (SUPERFAMILY)
645	c157272_g1_i1_len_413_path_1_0_412_1	6	zinc finger protein 705a-like isoform x1	138	1.03E-17	51.20%	0.099	IPR015880 (SMART); G3DSA:3.30.60.20 (GENE3D); IPR013087 (G3DSA:3.30.160.GENE3D); PF13465 (PFAM); PTHR24409:SF13 (PANTHER); PTHR24409 (PANTHER); IPR007087 (PROSITE_PATTERNS); IPR007087 (PROSITE_PATTERNS); IPR007087 (PROSITE_PROFILES); IPR007087 (PROSITE_PROFILES); SSF57667 (SUPERFAMILY); SSF57667 (SUPERFAMILY)
646	c157401_g1_i1_len_269_path_1_0_268_3	4	semaphorin-1a	90	3.17E-41	87.00%	0.111	IPR001627 (PFAM); IPR015943 (G3DSA:2.130.10.GENE3D); PTHR11036:SF66 (PANTHER); IPR027231 (PANTHER); IPR001627 (PROSITE_PROFILES); IPR001627 (SUPERFAMILY)
647	c157484_g1_i1_len_260_path_1_0_259_0	5	iron zinc purple acid phosphatase-like isoform x1	87	1.21E-32	75.80%	0.099	IPR015914 (G3DSA:2.60.40.GENE3D); PTHR22953:SF9 (PANTHER); PTHR22953 (PANTHER); IPR008963 (SUPERFAMILY)

648	c157751_g1_i1_len_511_path_489_0_510_1	8	cell division protein kinase 5	170	9.75E-119	99.10%	0.101	IPR002290 (SMART); IPR000719 (PFAM); G3DSA:3.30.200.20 (GENE3D); G3DSA:1.10.510.10 (GENE3D); PTHR24056 (PANTHER); IPR008271 (PROSITE_PATTERNS); IPR000719 (PROSITE_PROFILES); IPR011009 (SUPERFAMILY)
649	c157968_g1_i1_len_511_path_489_0_510_1	12	tyrosine-protein phosphatase 69d	170	5.52E-82	84.80%	0.102	IPR000242 (PRINTS); IPR000242 (SMART); IPR029021 (G3DSA:3.90.190.GENE3D); IPR000242 (PFAM); PTHR19134:SF277 (PANTHER); PTHR19134 (PANTHER); IPR000242 (PROSITE_PROFILES); IPR029021 (SUPERFAMILY)
650	c158058_g1_i1_len_262_path_240_0_261_1	6	wd repeat-containing protein 33	87	8.03E-47	92.10%	0.102	IPR001680 (SMART); IPR001680 (PFAM); IPR015943 (G3DSA:2.130.10.GENE3D); PTHR22836 (PANTHER); IPR001680 (PROSITE_PROFILES); IPR017986 (PROSITE_PROFILES); IPR001680 (PROSITE_PROFILES); IPR017986 (SUPERFAMILY)
651	c158154_g1_i1_len_587_path_569_0_586_2	20	myostatin	195	2.54E-76	84.70%	0.464Y	IPR001839 (SMART); IPR001839 (PFAM); IPR029034 (G3DSA:2.10.90.GENE3D); IPR015615 (PANTHER); IPR015616 (PTHR11848:PANTHER); IPR017948 (PROSITE_PATTERNS); IPR001839 (PROSITE_PROFILES); SignalP-noTM (SIGNALP_EUK); IPR029034 (SUPERFAMILY)
652	c158168_g1_i1_len_260_path_238_0_259_0	4	low quality protein: matrilin-4	87	1.28E-06	61.33%	0.104	IPR001881 (SMART); PF14670 (PFAM); G3DSA:2.10.25.10 (GENE3D); IPR013032 (PROSITE_PATTERNS); SSF57196 (SUPERFAMILY)
653	c158233_g1_i1_len_201_path_179_0_200_1	1	---NA---	67			0.411Y	G3DSA:2.40.10.10 (GENE3D); IPR001254 (PFAM); IPR009003 (SUPERFAMILY)
654	c158245_g1_i1_len_386_path_1_0_385_5	3	cyclin-dependent kinase-like partial	128	5.63E-40	90.20%	0.125	IPR000719 (PFAM); G3DSA:3.30.200.20 (GENE3D); G3DSA:3.30.200.20 (GENE3D); PTHR24056:SF153 (PANTHER); PTHR24056 (PANTHER); IPR000719 (PROSITE_PROFILES); IPR011009 (SUPERFAMILY)
655	c158358_g1_i1_len_688_path_666_0_687_0	18	adam 17-like protease	230	2.34E-67	70.20%	0.214	IPR002870 (PFAM); PTHR11905:SF121 (PANTHER); PTHR11905 (PANTHER); CYTOPLASMIC_DOMAIN (PHOBIUS); TRANSMEMBRANE (PHOBIUS); NON_CYTOPLASMIC_DOMAIN (PHOBIUS)
656	c158504_g1_i1_len_402_path_1_0_401_0	5	proclotting enzyme	134	2.16E-08	77.30%	0.184	IPR001254 (PFAM); G3DSA:2.40.10.10 (GENE3D); PTHR24268 (PANTHER); IPR009003 (SUPERFAMILY)
657	c158730_g1_i1_len_638_path_616_0_637_2	21	venom protease-like	212	3.12E-54	61.40%	0.147	IPR001314 (PRINTS); IPR001254 (SMART); G3DSA:2.40.10.10 (GENE3D); G3DSA:2.40.10.10 (GENE3D); IPR001254 (PFAM); G3DSA:2.40.10.10 (GENE3D); PTHR24268 (PANTHER); IPR018114 (PROSITE_PATTERNS); IPR018114 (PROSITE_PATTERNS); IPR001254 (PROSITE_PROFILES); IPR009003 (SUPERFAMILY)
658	c158838_g1_i1_len_373_path_1_0_372_1	9	microtubule-associated serine threonine-protein kinase 3 isoform x1	124	4.66E-78	95.50%	0.126	IPR002290 (SMART); IPR000719 (PFAM); G3DSA:1.10.510.10 (GENE3D); PTHR24356 (PANTHER); PTHR24356:SF36 (PANTHER); NON_CYTOPLASMIC_DOMAIN (PHOBIUS); TRANSMEMBRANE (PHOBIUS); CYTOPLASMIC_DOMAIN (PHOBIUS); IPR000719 (PROSITE_PROFILES); IPR011009 (SUPERFAMILY)
659	c15885_g1_i1_len_1404_path_333_0_859_1192_860_964_332_965_1403_4	70	serine proteinase	468	2.79E-71	56.70%	0.278	IPR001314 (PRINTS); IPR001254 (SMART); G3DSA:2.40.10.10 (GENE3D); G3DSA:2.40.10.10 (GENE3D); IPR001254 (PFAM); G3DSA:2.40.10.10 (GENE3D); PTHR24268 (PANTHER); PTHR24268:SF81 (PANTHER); IPR018114 (PROSITE_PATTERNS); TRANSMEMBRANE (PHOBIUS); NON_CYTOPLASMIC_DOMAIN (PHOBIUS); NON_CYTOPLASMIC_DOMAIN (PHOBIUS); CYTOPLASMIC_DOMAIN (PHOBIUS); TRANSMEMBRANE (PHOBIUS); IPR001254 (PROSITE_PROFILES); IPR009003 (SUPERFAMILY)
660	c15885_g1_i2_len_1440_path_333_0_859_191_860_1000_332_1001_1439_4	76	venom protease-like	480	7.01E-71	55.40%	0.278	IPR001314 (PRINTS); IPR001254 (SMART); G3DSA:2.40.10.10 (GENE3D); G3DSA:2.40.10.10 (GENE3D); IPR001254 (PFAM); G3DSA:2.40.10.10 (GENE3D); PTHR24265 (PANTHER); IPR018114 (PROSITE_PATTERNS); NON_CYTOPLASMIC_DOMAIN (PHOBIUS); TRANSMEMBRANE (PHOBIUS); TRANSMEMBRANE (PHOBIUS); NON_CYTOPLASMIC_DOMAIN (PHOBIUS); CYTOPLASMIC_DOMAIN (PHOBIUS); IPR001254 (PROSITE_PROFILES); IPR009003 (SUPERFAMILY)
661	c158907_g1_i1_len_313_path_1_0_312_0	4	endonuclease mitochondrial	105	6.06E-52	84.10%	0.1	IPR020821 (SMART); IPR020821 (G3DSA:3.40.570.GENE3D); IPR001604 (PFAM); PTHR13966 (PANTHER); SSF54060 (SUPERFAMILY)
662	c158913_g1_i1_len_278_path_1_0_277_4	8	hemicentin-1-like protein	93	1.12E-25	66.00%	0.11	G3DSA:2.10.25.10 (GENE3D); G3DSA:2.10.25.10 (GENE3D); IPR026823 (PFAM); PTHR24034:SF33 (PANTHER); PTHR24034 (PANTHER); IPR013032 (PROSITE_PATTERNS); IPR018097 (PROSITE_PATTERNS); SSF57196 (SUPERFAMILY)
663	c158919_g1_i1_len_332_path_1_0_331_4	4	fat-like cadherin-related tumor suppressor-like protein	111	1.90E-44	82.50%	0.142	IPR002126 (PRINTS); IPR002126 (SMART); IPR002126 (PFAM); IPR002126 (G3DSA:2.60.40.GENE3D); PTHR24026:SF39 (PANTHER); PTHR24026 (PANTHER); IPR002126 (PROSITE_PROFILES); IPR015919 (SUPERFAMILY)
664	c15893_g2_i1_len_138_path_312_0_137_1	0	t-cell immunomodulatory protein	46	5.99E-06	67.00%	0.105	no IPS match

665	c158946_g1_i1_len_243_path_221_0_242_3	7	neutral endopeptidase	81	1.02E-23	72.00%	0.113	IPR018497 (PRINTS); IPR024079 (G3DSA:3.40.390.GENE3D); IPR018497 (PFAM); PTHR11733:SF113 (PANTHER); IPR000718 (PANTHER); NON_CYTOPLASMIC_DOMAIN (PHOBIUS); CYTOPLASMIC_DOMAIN (PHOBIUS); TRANSMEMBRANE (PHOBIUS); SSF55486 (SUPERFAMILY); TMhelix (TMHMM)
666	c159006_g1_i1_len_523_path_1_0_522_2	8	serine threonine-protein kinase partial	174	5.49E-81	83.10%	0.113	IPR001245 (PFAM); G3DSA:3.30.200.20 (GENE3D); G3DSA:1.10.510.10 (GENE3D); PTHR23257:SF63 (PANTHER); PTHR23257 (PANTHER); IPR017441 (PROSITE_PATTERNS); IPR000719 (PROSITE_PROFILES); IPR011009 (SUPERFAMILY)
667	c159461_g1_i1_len_519_path_1_0_518_2	11	gastrula zinc finger	173	1.71E-35	59.50%	0.099	IPR015880 (SMART); IPR007087 (PFAM); IPR013087 (G3DSA:3.30.160.GENE3D); IPR013087 (G3DSA:3.30.160.GENE3D); IPR013087 (G3DSA:3.30.160.GENE3D); PF13465 (PFAM); PTHR24409:SF13 (PANTHER); PTHR24409 (PANTHER); IPR007087 (PROSITE_PATTERNS); IPR007087 (PROSITE_PATTERNS); IPR007087 (PROSITE_PATTERNS); IPR007087 (PROSITE_PATTERNS); IPR007087 (PROSITE_PROFILES); IPR007087 (PROSITE_PROFILES); IPR007087 (PROSITE_PROFILES); IPR007087 (PROSITE_PROFILES); IPR007087 (PROSITE_PROFILES); IPR007087 (PROSITE_PROFILES); SSF57667 (SUPERFAMILY); SSF57667 (SUPERFAMILY); SSF57667 (SUPERFAMILY)
668	c159700_g1_i1_len_150_path_128_0_149_3	3	blastula protease 10-like	50	2.99E-11	70.80%	0.106	IPR024079 (G3DSA:3.40.390.GENE3D); IPR001506 (PFAM); PTHR10127 (PANTHER); PTHR10127:SF580 (PANTHER); SSF55486 (SUPERFAMILY)
669	c159989_g1_i1_len_422_path_1_0_421_2	8	fibrillin-2 isoform x3	140	1.82E-49	73.90%	0.099	IPR000742 (SMART); IPR001881 (SMART); G3DSA:2.10.25.10 (GENE3D); IPR026823 (PFAM); G3DSA:2.10.25.10 (GENE3D); G3DSA:2.10.25.10 (GENE3D); IPR001881 (PFAM); G3DSA:2.10.25.10 (GENE3D); PF14670 (PFAM); PTHR24034 (PANTHER); IPR018097 (PROSITE_PATTERNS); IPR000152 (PROSITE_PATTERNS); IPR000152 (PROSITE_PATTERNS); IPR013032 (PROSITE_PATTERNS); IPR018097 (PROSITE_PATTERNS); IPR000742 (PROSITE_PROFILES); IPR000742 (PROSITE_PROFILES); IPR009030 (SUPERFAMILY)
670	c160259_g1_i1_len_746_path_1_0_745_3	15	ras-responsive element-binding protein 1	249	1.79E-55	57.60%	0.1	IPR015880 (SMART); PF13465 (PFAM); IPR013087 (G3DSA:3.30.160.GENE3D); IPR013087 (G3DSA:3.30.160.GENE3D); PF13894 (PFAM); PTHR23233:SF42 (PANTHER); PTHR23233 (PANTHER); IPR007087 (PROSITE_PATTERNS); IPR007087 (PROSITE_PATTERNS); IPR007087 (PROSITE_PATTERNS); IPR007087 (PROSITE_PATTERNS); IPR007087 (PROSITE_PROFILES); IPR007087 (PROSITE_PROFILES); IPR007087 (PROSITE_PROFILES); SSF57667 (SUPERFAMILY); SSF57667 (SUPERFAMILY)
671	c160268_g1_i1_len_482_path_1_0_481_3	14	wd sam and u-box domain-containing protein 1-like isoform x2	161	5.25E-35	63.60%	0.119	IPR020472 (PRINTS); IPR001680 (SMART); IPR001680 (PFAM); IPR015943 (G3DSA:2.130.10.GENE3D); PTHR22847 (PANTHER); IPR019775 (PROSITE_PATTERNS); IPR019775 (PROSITE_PATTERNS); IPR001680 (PROSITE_PROFILES); IPR017986 (PROSITE_PROFILES); IPR001680 (PROSITE_PROFILES); IPR017986 (SUPERFAMILY)
672	c160415_g1_i1_len_357_path_1_0_356_3	8	zinc finger protein 160-like	119	1.09E-24	64.60%	0.106	IPR015880 (SMART); IPR013087 (G3DSA:3.30.160.GENE3D); IPR013087 (G3DSA:3.30.160.GENE3D); IPR007087 (PFAM); PF13465 (PFAM); IPR013087 (G3DSA:3.30.160.GENE3D); IPR013087 (G3DSA:3.30.160.GENE3D); PTHR24409 (PANTHER); PTHR24409:SF14 (PANTHER); IPR007087 (PROSITE_PATTERNS); IPR007087 (PROSITE_PATTERNS); IPR007087 (PROSITE_PROFILES); IPR007087 (PROSITE_PROFILES); IPR007087 (PROSITE_PROFILES); SSF57667 (SUPERFAMILY); SSF57667 (SUPERFAMILY)
673	c160718_g1_i1_len_152_path_1_0_151_1	20	cathepsin l	51	1.99E-20	92.20%	0.099	IPR000668 (PFAM); G3DSA:3.90.70.10 (GENE3D); PTHR12411:SF57 (PANTHER); IPR013128 (PANTHER); SSF54001 (SUPERFAMILY)
674	c160855_g1_i1_len_139_path_1_0_138_4	0	pdz domain-containing protein 2	46	7.72E-17	88.20%	0.136	IPR001478 (PFAM); IPR001478 (G3DSA:2.30.42.GENE3D); PTHR19964 (PANTHER); PTHR19964:SF22 (PANTHER); IPR001478 (PROSITE_PROFILES); IPR001478 (SUPERFAMILY)
675	c161216_g1_i1_len_276_path_1_0_275_4	4	carbonyl reductase	92	7.46E-33	79.60%	0.1	IPR002347 (PRINTS); IPR016040 (G3DSA:3.40.50.GENE3D); IPR002198 (PFAM); PTHR24322 (PANTHER); PTHR24322:SF58 (PANTHER); SSF51735 (SUPERFAMILY)
676	c161385_g1_i1_len_542_path_1_0_541_2	21	gilt-like protein	180	1.40E-30	63.50%	0.133	IPR004911 (PFAM); PTHR13234:SF8 (PANTHER); IPR004911 (PANTHER); TRANSMEMBRANE (PHOBIUS); CYTOPLASMIC_DOMAIN (PHOBIUS); NON_CYTOPLASMIC_DOMAIN (PHOBIUS); TMhelix (TMHMM)
677	c161429_g1_i1_len_341_path_319_0_340_0	4	transposase	114	2.11E-12	53.00%	0.108	no IPS match

678	c1616_g1_i1_len_1652_path_1_0_1651_3	224	di-n-acetylchitobiase	551	2.82E-114	63.00%	0.881Y	IPR011583 (SMART); IPR001223 (PFAM); IPR013781 (G3DSA:3.20.20.GENE3D); PTHR11177:SF146 (PANTHER); PTHR11177 (PANTHER); NON_CYTOPLASMIC_DOMAIN (PHOBIUS); SIGNAL_PEPTIDE (PHOBIUS); SIGNAL_PEPTIDE_C_REGION (PHOBIUS); SIGNAL_PEPTIDE_N_REGION (PHOBIUS); SIGNAL_PEPTIDE_H_REGION (PHOBIUS); SignalP-noTM (SIGNALP_EUK); IPR017853 (SUPERFAMILY); TMhelix (TMHMM)
679	c1618_g1_i1_len_1780_path_1_0_1779_4	187	secreted frizzled-related protein 5	593	2.47E-116	63.80%	0.105	Coil (COILS); IPR020067 (SMART); IPR018933 (PFAM); IPR020067 (G3DSA:1.10.2000.GENE3D); IPR020067 (PFAM); IPR015526 (PANTHER); PTHR11309:SF80 (PANTHER); NON_CYTOPLASMIC_DOMAIN (PHOBIUS); CYTOPLASMIC_DOMAIN (PHOBIUS); TRANSMEMBRANE (PHOBIUS); IPR020067 (PROSITE_PROFILES); IPR001134 (PROSITE_PROFILES); IPR008993 (SUPERFAMILY); IPR020067 (SUPERFAMILY); TMhelix (TMHMM)
680	c162416_g1_i1_len_283_path_261_0_282_1	4	nadph--cytochrome p450 reductase	94	7.36E-56	96.50%	0.103	IPR002401 (PRINTS); IPR001128 (G3DSA:1.10.630.GENE3D); IPR001128 (PFAM); PTHR24305:SF21 (PANTHER); PTHR24305 (PANTHER); IPR001128 (SUPERFAMILY)
681	c162748_g1_i1_len_472_path_1_0_471_3	7	thrombospondin type-1 domain-containing protein 4-like	158	7.49E-16	60.40%	0.112	IPR000884 (SUPERFAMILY)
682	c162826_g1_i1_len_277_path_1_0_276_0	2	collagen alpha-1 chain	93	3.79E-12	61.50%	0.1	IPR008160 (PFAM); PTHR24023 (PANTHER); PTHR24023:SF372 (PANTHER)
683	c162898_g1_i1_len_280_path_258_0_279_0	4	nad h-hydrate epimerase isoform x1	94	1.00E-41	82.20%	0.164	IPR004443 (G3DSA:3.40.50.GENE3D); IPR004443 (PFAM); PTHR13232:SF11 (PANTHER); PTHR13232 (PANTHER); IPR004443 (PROSITE_PROFILES); IPR004443 (SUPERFAMILY)
684	c163161_g1_i1_len_257_path_235_0_256_2	4	moxd1 homolog 1-like	85	1.97E-06	68.00%	0.155	PF03712 (PFAM); IPR014784 (G3DSA:2.60.120.GENE3D); IPR008977 (SUPERFAMILY)
685	c1636_g1_i1_len_540_path_1_0_539_3	10	tyrosine-protein phosphatase non-receptor type 9 isoform x6	180	3.87E-77	81.70%	0.106	IPR000242 (PRINTS); IPR000242 (SMART); IPR029021 (G3DSA:3.90.190.GENE3D); IPR000242 (PFAM); PTHR19134:SF232 (PANTHER); PTHR19134 (PANTHER); IPR000242 (PROSITE_PROFILES); IPR029021 (SUPERFAMILY)
686	c164763_g1_i1_len_392_path_1_0_391_0	17	maguk p55 subfamily member 5	131	4.98E-72	92.00%	0.107	Coil (COILS); Coil (COILS); IPR008145 (SMART); IPR027417 (G3DSA:3.40.50.GENE3D); IPR008145 (PFAM); PTHR23122 (PANTHER); PTHR23122:SF32 (PANTHER); IPR008144 (PROSITE_PROFILES); IPR027417 (SUPERFAMILY)
687	c164825_g1_i1_len_436_path_414_0_435_2	6	von willebrand factor type egf and pentraxin domain-containing protein 1- partial	145	3.07E-69	83.80%	0.119	IPR003410 (PFAM); G3DSA:2.10.70.10 (GENE3D); PTHR24049:SF6 (PANTHER); PTHR24049 (PANTHER); IPR003410 (PROSITE_PROFILES)
688	c165078_g1_i1_len_370_path_1_0_369_2	10	#NAME?	123	6.61E-25	83.40%	0.177	Coil (COILS); IPR001623 (PRINTS); IPR001623 (SMART); IPR001623 (PFAM); IPR001623 (G3DSA:1.10.287.GENE3D); PTHR24077 (PANTHER); NON_CYTOPLASMIC_DOMAIN (PHOBIUS); TRANSMEMBRANE (PHOBIUS); CYTOPLASMIC_DOMAIN (PHOBIUS); IPR001623 (PROSITE_PROFILES); IPR001623 (SUPERFAMILY)
689	c165124_g1_i1_len_268_path_246_0_267_4	4	zinc finger protein 567 isoform x2	89	1.44E-46	87.50%	0.107	IPR015880 (SMART); IPR013087 (G3DSA:3.30.160.GENE3D); PF13465 (PFAM); PTHR23228 (PANTHER); PTHR23228:SF14 (PANTHER); IPR007087 (PROSITE_PATTERNS); IPR007087 (PROSITE_PATTERNS); IPR007087 (PROSITE_PROFILES); IPR007087 (PROSITE_PROFILES); IPR007087 (PROSITE_PROFILES); SSF57667 (SUPERFAMILY); SSF57667 (SUPERFAMILY)
690	c16540_g1_i1_len_2213_path_43_0_173_21_174_2212_2	183	extracellular serine threonine protein kinase fam20c isoform x1	737	0	71.70%	0.15	IPR009581 (PFAM); PTHR12450:SF8 (PANTHER); IPR024869 (PANTHER); TRANSMEMBRANE (PHOBIUS); NON_CYTOPLASMIC_DOMAIN (PHOBIUS); CYTOPLASMIC_DOMAIN (PHOBIUS); CYTOPLASMIC_DOMAIN (PHOBIUS); TRANSMEMBRANE (PHOBIUS); TRANSMEMBRANE (PHOBIUS); NON_CYTOPLASMIC_DOMAIN (PHOBIUS); TMhelix (TMHMM); TMhelix (TMHMM)
691	c16589_g1_i1_len_1362_path_1390_0_1361_5	58	isoform b	454	2.12E-107	64.90%	0.101	IPR015812 (PRINTS); G3DSA:2.10.25.10 (GENE3D); G3DSA:2.10.25.10 (GENE3D); IPR014836 (G3DSA:1.20.5.GENE3D); IPR014836 (PFAM); G3DSA:1.20.5.100 (GENE3D); IPR015812 (PANTHER); PTHR10082:SF16 (PANTHER); IPR015812 (PROSITE_PATTERNS); IPR015812 (PROSITE_PATTERNS); IPR015812 (PROSITE_PATTERNS); CYTOPLASMIC_DOMAIN (PHOBIUS); NON_CYTOPLASMIC_DOMAIN (PHOBIUS); TRANSMEMBRANE (PHOBIUS); SSF57196 (SUPERFAMILY); IPR012896 (SUPERFAMILY); TMhelix (TMHMM)
692	c166729_g1_i1_len_248_path_1_0_247_0	6	carboxypeptidase n subunit 2	83	1.19E-11	62.50%	0.106	IPR001611 (PFAM); G3DSA:3.80.10.10 (GENE3D); PTHR24365 (PANTHER); SSF52058 (SUPERFAMILY)
693	c166983_g1_i1_len_253_path_231_0_252_5	4	chaperone protein	84	2.88E-25	86.10%	0.154	IPR013126 (PRINTS); IPR013126 (PFAM); G3DSA:3.30.420.40 (GENE3D); PTHR19375 (PANTHER); PTHR19375:SF173 (PANTHER); IPR018181 (PROSITE_PATTERNS); SSF53067 (SUPERFAMILY)

694	c16699_g1_i1_len_2273_path_2301_0_2272_2	297	serine threonine-protein kinase grp-like	757	0	74.50%	0.173	IPR002290 (SMART); G3DSA:3.30.200.20 (GENE3D); G3DSA:1.10.510.10 (GENE3D); IPR000719 (PFAM); PTHR24344 (PANTHER); IPR008271 (PROSITE_PATTERNS); IPR017441 (PROSITE_PATTERNS); TRANSMEMBRANE (PHOBIUS); CYTOPLASMIC_DOMAIN (PHOBIUS); NON_CYTOPLASMIC_DOMAIN (PHOBIUS); NON_CYTOPLASMIC_DOMAIN (PHOBIUS); TRANSMEMBRANE (PHOBIUS); TRANSMEMBRANE (PHOBIUS); CYTOPLASMIC_DOMAIN (PHOBIUS); IPR000719 (PROSITE_PROFILES); IPR011009 (SUPERFAMILY); TMhelix (TMHMM); TMhelix (TMHMM); TMhelix (TMHMM)
695	c167229_g1_i1_len_380_path_358_0_379_4	5	kinase d-interacting substrate of 220 kda isoform x7	127	1.34E-63	87.70%	0.105	IPR002110 (SMART); IPR020683 (G3DSA:1.25.40.GENE3D); IPR020683 (PFAM); PTHR24139 (PANTHER); PTHR24139:SF10 (PANTHER); IPR002110 (PROSITE_PROFILES); IPR002110 (PROSITE_PROFILES); IPR002110 (PROSITE_PROFILES); IPR020683 (PROSITE_PROFILES); IPR020683 (SUPERFAMILY)
696	c16754_g1_i1_len_2135_path_2163_0_2134_4	146	leucine rich repeat protein	712	5.73E-36	53.10%	0.505 Y	PR00019 (PRINTS); IPR003591 (SMART); SM00364 (SMART); SM00365 (SMART); G3DSA:3.80.10.10 (GENE3D); G3DSA:3.80.10.10 (GENE3D); IPR001611 (PFAM); IPR001611 (PFAM); G3DSA:3.80.10.10 (GENE3D); PTHR24365 (PANTHER); CYTOPLASMIC_DOMAIN (PHOBIUS); TRANSMEMBRANE (PHOBIUS); NON_CYTOPLASMIC_DOMAIN (PHOBIUS); IPR001611 (PROSITE_PROFILES); IPR001611 (PROSITE_PROFILES); IPR001611 (PROSITE_PROFILES); IPR001611 (PROSITE_PROFILES); IPR001611 (PROSITE_PROFILES); IPR001611 (PROSITE_PROFILES); IPR001611 (PROSITE_PROFILES); IPR001611 (PROSITE_PROFILES); IPR001611 (PROSITE_PROFILES); IPR001611 (PROSITE_PROFILES); IPR001611 (PROSITE_PROFILES); IPR001611 (PROSITE_PROFILES); IPR001611 (PROSITE_PROFILES); IPR001611 (PROSITE_PROFILES); SignalP-noTM (SIGNALP_EUK); SSF52058 (SUPERFAMILY); SSF52058 (SUPERFAMILY); TMhelix (TMHMM); TMhelix (TMHMM)
697	c167603_g1_i1_len_189_path_1_0_188_3	0	zinc finger protein 717-like	63	8.92E-11	66.20%	0.099	IPR015880 (SMART); IPR013087 (G3DSA:3.30.160.GENE3D); PF13465 (PFAM); PTHR24409 (PANTHER); PTHR24409:SF13 (PANTHER); IPR007087 (PROSITE_PATTERNS); IPR007087 (PROSITE_PROFILES); SSF57667 (SUPERFAMILY)
698	c169380_g1_i1_len_278_path_256_0_277_1	2	vitellogenin-1-like isoform x2	93	1.10E-09	56.75%	0.311	IPR001747 (PFAM); IPR015816 (G3DSA:2.30.230.GENE3D); IPR015819 (SUPERFAMILY)
699	c170238_g1_i1_len_890_path_1_0_889_2	22	variable lymphocyte receptor a	296	1.01E-09	51.00%	0.114	SM00364 (SMART); IPR003591 (SMART); IPR001611 (PFAM); G3DSA:3.80.10.10 (GENE3D); IPR001611 (PFAM); G3DSA:3.80.10.10 (GENE3D); PTHR24365 (PANTHER); TRANSMEMBRANE (PHOBIUS); CYTOPLASMIC_DOMAIN (PHOBIUS); NON_CYTOPLASMIC_DOMAIN (PHOBIUS); IPR001611 (PROSITE_PROFILES); IPR001611 (PROSITE_PROFILES); IPR001611 (PROSITE_PROFILES); IPR001611 (PROSITE_PROFILES); IPR001611 (PROSITE_PROFILES); IPR001611 (PROSITE_PROFILES); IPR001611 (PROSITE_PROFILES); SSF52058 (SUPERFAMILY); TMhelix (TMHMM)
700	c170248_g1_i1_len_1471_path_1_0_1470_4	84	transducin beta-like protein 3	490	6.78E-107	76.10%	0.209	Coil (COILS); Coil (COILS); IPR001680 (SMART); IPR001680 (PFAM); IPR015943 (G3DSA:2.130.10.GENE3D); IPR013934 (PFAM); PTHR19854 (PANTHER); PTHR19854:SF15 (PANTHER); IPR001680 (PROSITE_PROFILES); IPR001680 (PROSITE_PROFILES); IPR017986 (PROSITE_PROFILES); IPR001680 (PROSITE_PROFILES); IPR017986 (SUPERFAMILY)
701	c170250_g1_i1_len_1648_path_1_0_1647_2	941	ras-related protein rab-10	549	3.99E-116	92.90%	0.173	IPR001806 (PRINTS); IPR003579 (SMART); IPR003578 (SMART); IPR002041 (SMART); IPR024156 (SMART); IPR020849 (SMART); IPR001806 (PFAM); IPR005225 (TIGRFAM); IPR027417 (G3DSA:3.40.50.GENE3D); PTHR24073:SF456 (PANTHER); PTHR24073 (PANTHER); NON_CYTOPLASMIC_DOMAIN (PHOBIUS); TRANSMEMBRANE (PHOBIUS); CYTOPLASMIC_DOMAIN (PHOBIUS); PS51419 (PROSITE_PROFILES); IPR027417 (SUPERFAMILY)
702	c170269_g1_i1_len_693_path_671_0_692_5	36	vitellogenic-like precursor	231	6.80E-67	67.00%	0.16	IPR001563 (PRINTS); IPR001563 (PFAM); IPR029058 (G3DSA:3.40.50.GENE3D); IPR001563 (PANTHER); IPR029058 (SUPERFAMILY)
703	c170278_g1_i1_len_1317_path_1_0_1316_1	56	dual specificity mitogen-activated protein kinase kinase dsor1	439	0	85.60%	0.104	IPR002290 (SMART); G3DSA:1.10.510.10 (GENE3D); G3DSA:3.30.200.20 (GENE3D); IPR000719 (PFAM); PTHR24360:SF15 (PANTHER); PTHR24360 (PANTHER); IPR008271 (PROSITE_PATTERNS); IPR017441 (PROSITE_PATTERNS); IPR000719 (PROSITE_PROFILES); IPR011009 (SUPERFAMILY)
704	c170303_g1_i1_len_467_path_1_0_466_3	7	fras1-related extracellular matrix protein 1-like	156	2.15E-07	57.00%	0.126	SSF141072 (SUPERFAMILY)

705	c170330_g1_i1_len_554_path_532_0_553_2	10	plexin-b	184	9.51E-90	90.70%	0.123	IPR013548 (PFAM); PTHR22625:SF31 (PANTHER); PTHR22625 (PANTHER); IPR008936 (SUPERFAMILY)
706	c170438_g1_i1_len_950_path_928_0_949_2	922	mitochondrial manganese superoxide dismutase	316	1.02E-121	86.10%	0.127	Coil (COILS); IPR001189 (PRINTS); IPR019831 (PFAM); G3DSA:1.10.287.990 (GENE3D); IPR019832 (PFAM); PTHR11404:SF6 (PANTHER); IPR001189 (PANTHER); IPR019833 (PROSITE_PATTERNS); NON_CYTOPLASMIC_DOMAIN (PHOBIUS); TRANSMEMBRANE (PHOBIUS); CYTOPLASMIC_DOMAIN (PHOBIUS); IPR019831 (SUPERFAMILY); IPR019832 (SUPERFAMILY); TMhelix (TMHMM)
707	c170490_g1_i1_len_574_path_1_0_573_2	10	fascilin 2-like protein	191	8.12E-25	53.10%	0.105	IPR003598 (SMART); IPR003599 (SMART); IPR013783 (G3DSA:2.60.40.GENE3D); IPR013098 (PFAM); IPR013783 (G3DSA:2.60.40.GENE3D); PF13895 (PFAM); PTHR10489 (PANTHER); IPR007110 (PROSITE_PROFILES); IPR007110 (PROSITE_PROFILES); IPR007110 (PROSITE_PROFILES); SSF48726 (SUPERFAMILY); SSF48726 (SUPERFAMILY); SSF48726 (SUPERFAMILY)
708	c170523_g1_i1_len_969_path_1_0_968_0	88	ion transport peptide isoform x2	323	7.37E-06	86.00%	0.203	IPR001166 (PFAM); IPR001166 (G3DSA:1.10.2010.GENE3D); IPR001166 (SUPERFAMILY)
709	c170523_g1_i1_len_969_path_1_0_968_1	88	ion transport peptide-like	323	1.97E-26	68.50%	0.133	IPR001166 (PRINTS); IPR001166 (G3DSA:1.10.2010.GENE3D); IPR001166 (PFAM); NON_CYTOPLASMIC_DOMAIN (PHOBIUS); TRANSMEMBRANE (PHOBIUS); CYTOPLASMIC_DOMAIN (PHOBIUS); IPR001166 (SUPERFAMILY)
710	c17056_g1_i1_len_1172_path_1524_0_1171_1	34	centrosomal protein of 290 kda	391	5.48E-63	58.90%	0.102	Coil (COILS); Coil (COILS); Coil (COILS); Coil (COILS); Coil (COILS); Coil (COILS); Coil (COILS); IPR026201 (PANTHER)
711	c170573_g1_i1_len_2096_path_1_0_2095_2	124	mitogen-activated protein kinase kinase kinase 4	698	2.83E-176	66.30%	0.161	IPR002290 (SMART); G3DSA:3.30.200.20 (GENE3D); G3DSA:1.10.510.10 (GENE3D); IPR000719 (PFAM); PTHR24360 (PANTHER); PTHR24360:SF47 (PANTHER); IPR008271 (PROSITE_PATTERNS); IPR017441 (PROSITE_PATTERNS); IPR000719 (PROSITE_PROFILES); IPR011009 (SUPERFAMILY)
712	c170606_g1_i1_len_2190_path_1_0_2189_5	142	latrophilin-2-like isoform x4	730	4.29E-83	48.00%	0.118	IPR000832 (PRINTS); IPR000203 (SMART); IPR000203 (PFAM); IPR000832 (PFAM); IPR022624 (PFAM); PTHR12011 (PANTHER); TRANSMEMBRANE (PHOBIUS); NON_CYTOPLASMIC_DOMAIN (PHOBIUS); TRANSMEMBRANE (PHOBIUS); NON_CYTOPLASMIC_DOMAIN (PHOBIUS); TRANSMEMBRANE (PHOBIUS); NON_CYTOPLASMIC_DOMAIN (PHOBIUS); CYTOPLASMIC_DOMAIN (PHOBIUS); CYTOPLASMIC_DOMAIN (PHOBIUS); CYTOPLASMIC_DOMAIN (PHOBIUS); TRANSMEMBRANE (PHOBIUS); NON_CYTOPLASMIC_DOMAIN (PHOBIUS); CYTOPLASMIC_DOMAIN (PHOBIUS); TRANSMEMBRANE (PHOBIUS); TRANSMEMBRANE (PHOBIUS); TRANSMEMBRANE (PHOBIUS); IPR017981 (PROSITE_PROFILES); IPR000203 (PROSITE_PROFILES); SSF81321 (SUPERFAMILY); TMhelix (TMHMM); TMhelix (TMHMM); TMhelix (TMHMM); TMhelix (TMHMM); TMhelix (TMHMM); TMhelix (TMHMM); TMhelix (TMHMM)
713	c170617_g1_i1_len_1207_path_1185_0_1206_1	64	zinc finger protein 699-like	402	7.34E-58	56.90%	0.105	IPR015880 (SMART); PF13894 (PFAM); IPR013087 (G3DSA:3.30.160.GENE3D); PF13465 (PFAM); IPR007087 (PFAM); IPR013087 (G3DSA:3.30.160.GENE3D); IPR013087 (G3DSA:3.30.160.GENE3D); PTHR24409:SF13 (PANTHER); PTHR24409 (PANTHER); IPR007087 (PROSITE_PATTERNS); IPR007087 (PROSITE_PATTERNS); IPR007087 (PROSITE_PATTERNS); IPR007087 (PROSITE_PATTERNS); IPR007087 (PROSITE_PATTERNS); IPR007087 (PROSITE_PATTERNS); IPR007087 (PROSITE_PATTERNS); IPR007087 (PROSITE_PATTERNS); IPR007087 (PROSITE_PATTERNS); IPR007087 (PROSITE_PATTERNS); IPR007087 (PROSITE_PROFILES); IPR007087 (PROSITE_PROFILES); IPR007087 (PROSITE_PROFILES); IPR007087 (PROSITE_PROFILES); IPR007087 (PROSITE_PROFILES); IPR007087 (PROSITE_PROFILES); IPR007087 (PROSITE_PROFILES); IPR007087 (PROSITE_PROFILES); IPR007087 (PROSITE_PROFILES); IPR007087 (PROSITE_PROFILES); SSF57667 (SUPERFAMILY); SSF57667 (SUPERFAMILY); SSF57667 (SUPERFAMILY); SSF57667 (SUPERFAMILY); SSF57667 (SUPERFAMILY)
714	c170733_g1_i1_len_317_path_295_0_316_0	6	nad h-hydrate epimerase-like	106	3.36E-47	79.90%	0.115	IPR004443 (G3DSA:3.40.50.GENE3D); IPR004443 (PFAM); PTHR13232 (PANTHER); IPR004443 (PROSITE_PROFILES); IPR004443 (SUPERFAMILY)
715	c170789_g1_i1_len_1683_path_987_0_288_1276_289_985_1276_986_1682_3	128	protein phosphatase 1 regulatory subunit 16a isoform x3	561	1.26E-54	82.50%	0.223	IPR002110 (SMART); IPR020683 (G3DSA:1.25.40.GENE3D); IPR020683 (PFAM); PTHR24186 (PANTHER); IPR020683 (PROSITE_PROFILES); IPR002110 (PROSITE_PROFILES); IPR002110 (PROSITE_PROFILES); IPR020683 (SUPERFAMILY)

716	c170789_g1_i1_len_1683_path_987_0_288_1276_289_985_1276_986_1682_4	128	protein phosphatase 1 regulatory subunit 16a	561	8.47E-61	60.30%	0.196	Coil (COILS); IPR002110 (SMART); IPR020683 (G3DSA:1.25.40.GENE3D); IPR020683 (PFAM); PTHR24186 (PANTHER); CYTOPLASMIC_DOMAIN (PHOBIUS); NON_CYTOPLASMIC_DOMAIN (PHOBIUS); TRANSMEMBRANE (PHOBIUS); IPR002110 (PROSITE_PROFILES); IPR002110 (PROSITE_PROFILES); IPR020683 (PROSITE_PROFILES); IPR020683 (SUPERFAMILY)
717	c170826_g1_i1_len_522_path_500_0_521_4	10	fat-like cadherin-related tumor suppressor homolog	174	1.87E-66	73.30%	0.128	IPR002126 (PRINTS); IPR002126 (SMART); IPR002126 (PFAM); IPR002126 (G3DSA:2.60.40.GENE3D); PTHR24027 (PANTHER); PTHR24027:SF260 (PANTHER); IPR020894 (PROSITE_PATTERNS); IPR020894 (PROSITE_PATTERNS); IPR002126 (PROSITE_PROFILES); IPR002126 (PROSITE_PROFILES); IPR015919 (SUPERFAMILY); IPR015919 (SUPERFAMILY)
718	c170831_g1_i1_len_455_path_1_0_454_5	14	glutamyl aminopeptidase	151	5.99E-14	53.40%	0.164	IPR024571 (PFAM); PTHR11533:SF165 (PANTHER); IPR001930 (PANTHER)
719	c170849_g1_i1_len_931_path_1_0_930_1	23	zinc finger protein 850-like	310	2.27E-38	57.90%	0.1	IPR015880 (SMART); IPR013087 (G3DSA:3.30.160.GENE3D); IPR007087 (PFAM); PF13912 (PFAM); IPR013087 (G3DSA:3.30.160.GENE3D); PF13894 (PFAM); IPR013087 (G3DSA:3.30.160.GENE3D); PF13465 (PFAM); PTHR24409:SF14 (PANTHER); PTHR24409 (PANTHER); IPR007087 (PROSITE_PATTERNS); IPR007087 (PROSITE_PATTERNS); IPR007087 (PROSITE_PATTERNS); IPR007087 (PROSITE_PATTERNS); IPR007087 (PROSITE_PATTERNS); IPR007087 (PROSITE_PATTERNS); IPR007087 (PROSITE_PATTERNS); IPR007087 (PROSITE_PROFILES); IPR007087 (PROSITE_PROFILES); IPR007087 (PROSITE_PROFILES); IPR007087 (PROSITE_PROFILES); IPR007087 (PROSITE_PROFILES); IPR007087 (PROSITE_PROFILES); IPR007087 (PROSITE_PROFILES); SSF57667 (SUPERFAMILY); SSF57667 (SUPERFAMILY); SSF57667 (SUPERFAMILY)
720	c170862_g1_i1_len_471_path_1_0_470_2	8	---NA---	157			0.104	NON_CYTOPLASMIC_DOMAIN (PHOBIUS); TRANSMEMBRANE (PHOBIUS); TRANSMEMBRANE (PHOBIUS); NON_CYTOPLASMIC_DOMAIN (PHOBIUS); CYTOPLASMIC_DOMAIN (PHOBIUS); TMhelix (TMHMM); TMhelix (TMHMM)
721	c170906_g1_i1_len_1364_path_1342_0_1363_5	59	mam and ldl-receptor class a domain-containing protein 1-like	454	4.03E-11	42.70%	0.466 Y	IPR000998 (SMART); IPR000859 (SMART); IPR000859 (PFAM); IPR000859 (G3DSA:2.60.120.GENE3D); IPR000859 (G3DSA:2.60.120.GENE3D); IPR000998 (PFAM); PTHR22991 (PANTHER); IPR000859 (PROSITE_PROFILES); IPR000998 (PROSITE_PROFILES); IPR000998 (PROSITE_PROFILES); IPR013320 (SUPERFAMILY); IPR000859 (SUPERFAMILY); TMhelix (TMHMM)
722	c170908_g1_i1_len_1168_path_1_0_1167_3	126	acyl- delta desaturase	390	2.03E-163	76.30%	0.500Y	IPR015876 (PRINTS); IPR005804 (PFAM); PTHR11351:SF28 (PANTHER); PTHR11351 (PANTHER); CYTOPLASMIC_DOMAIN (PHOBIUS); NON_CYTOPLASMIC_DOMAIN (PHOBIUS); CYTOPLASMIC_DOMAIN (PHOBIUS); NON_CYTOPLASMIC_DOMAIN (PHOBIUS); CYTOPLASMIC_DOMAIN (PHOBIUS); TRANSMEMBRANE (PHOBIUS); TRANSMEMBRANE (PHOBIUS); TRANSMEMBRANE (PHOBIUS); TRANSMEMBRANE (PHOBIUS); SignalP-noTM (SIGNALP_EUK); TMhelix (TMHMM); TMhelix (TMHMM); TMhelix (TMHMM); TMhelix (TMHMM)
723	c170910_g1_i1_len_292_path_270_0_291_1	4	angiotensin-converting enzyme-like	97	2.18E-39	81.30%	0.131	IPR001548 (PRINTS); IPR001548 (PFAM); PTHR10514:SF17 (PANTHER); IPR001548 (PANTHER); SSF55486 (SUPERFAMILY)
724	c170929_g1_i1_len_1119_path_1_0_1118_1	24	connectin isoform x1	373	4.19E-45	65.70%	0.127	IPR003591 (SMART); IPR001611 (PFAM); G3DSA:3.80.10.10 (GENE3D); PTHR24369 (PANTHER); SSF52058 (SUPERFAMILY)
725	c170934_g1_i1_len_293_path_271_0_292_4	4	kielin chordin-like protein	98	7.87E-16	55.70%	0.111	G3DSA:2.10.70.10 (GENE3D); IPR001007 (PFAM); SSF57603 (SUPERFAMILY); SSF57603 (SUPERFAMILY)
726	c171102_g1_i1_len_1075_path_1_0_1074_4	103	serine threonine-protein kinase pim-3	358	1.08E-139	77.80%	0.132	IPR002290 (SMART); IPR000719 (PFAM); G3DSA:3.30.200.20 (GENE3D); G3DSA:1.10.510.10 (GENE3D); IPR017348 (PIRSF); PTHR22984 (PANTHER); PTHR22984:SF1 (PANTHER); IPR008271 (PROSITE_PATTERNS); IPR017441 (PROSITE_PATTERNS); IPR000719 (PROSITE_PROFILES); IPR011009 (SUPERFAMILY)
727	c171176_g1_i1_len_431_path_1_0_430_5	12	mam and ldl-receptor class a domain-containing protein 2-like	143	8.73E-30	58.90%	0.108	IPR002172 (SMART); IPR002172 (PFAM); IPR000998 (PFAM); IPR002172 (G3DSA:4.10.400.GENE3D); PTHR23282:SF75 (PANTHER); PTHR23282 (PANTHER); IPR023415 (PROSITE_PATTERNS); IPR002172 (PROSITE_PROFILES); IPR000998 (PROSITE_PROFILES); IPR000998 (PROSITE_PROFILES); IPR002172 (SUPERFAMILY)

728	c171277_g1_i1_len_557_path_1_0_556_3	9	slit protein	186	4.08E-92	85.60%	0.111	PR00019 (PRINTS); IPR000742 (SMART); IPR000483 (SMART); IPR003591 (SMART); IPR001611 (PFAM); IPR000742 (PFAM); G3DSA:2.10.25.10 (GENE3D); G3DSA:3.80.10.10 (GENE3D); PTHR24373 (PANTHER); PTHR24373:SF110 (PANTHER); IPR013032 (PROSITE_PATTERNS); IPR013032 (PROSITE_PATTERNS); IPR001611 (PROSITE_PROFILES); IPR000742 (PROSITE_PROFILES); IPR001611 (PROSITE_PROFILES); IPR001611 (PROSITE_PROFILES); SSF57196 (SUPERFAMILY); SSF52058 (SUPERFAMILY)
729	c171288_g1_i1_len_1299_path_1_0_1298_5	44	cytochrome p450 monooxygenase	433	3.36E-116	59.90%	0.099	IPR002401 (PRINTS); IPR001128 (PRINTS); IPR001128 (PFAM); IPR001128 (G3DSA:1.10.630.GENE3D); PTHR24300 (PANTHER); IPR001128 (SUPERFAMILY)
730	c171314_g1_i1_len_1872_path_1_0_1871_2	114	serine threonine-protein phosphatase 6 regulatory ankyrin repeat subunit c-like	624	4.63E-96	55.00%	0.102	IPR002110 (SMART); IPR020683 (G3DSA:1.25.40.GENE3D); IPR002110 (PFAM); IPR020683 (G3DSA:1.25.40.GENE3D); IPR020683 (PFAM); IPR020683 (G3DSA:1.25.40.GENE3D); PTHR24198 (PANTHER); CYTOPLASMIC_DOMAIN (PHOBIUS); TRANSMEMBRANE (PHOBIUS); NON_CYTOPLASMIC_DOMAIN (PHOBIUS); IPR002110 (PROSITE_PROFILES); IPR002110 (PROSITE_PROFILES); IPR020683 (PROSITE_PROFILES); IPR002110 (PROSITE_PROFILES); IPR002110 (PROSITE_PROFILES); IPR002110 (PROSITE_PROFILES); IPR002110 (PROSITE_PROFILES); IPR002110 (PROSITE_PROFILES); IPR020683 (SUPERFAMILY); TMhelix (TMHMM)
731	c171386_g1_i1_len_1445_path_1_0_1444_3	548	26s protease regulatory subunit 4	482	0	97.10%	0.105	IPR003593 (SMART); IPR003959 (PFAM); IPR005937 (TIGRFAM); G3DSA:1.10.8.60 (GENE3D); IPR027417 (G3DSA:3.40.50.GENE3D); PTHR23073:SF9 (PANTHER); PTHR23073 (PANTHER); IPR003960 (PROSITE_PATTERNS); IPR027417 (SUPERFAMILY)
732	c171397_g1_i1_len_693_path_671_0_692_3	25	hemagglutinin amebocyte aggregation factor-like	231	3.32E-42	61.10%	0.302	PF14704 (PFAM); IPR026645 (PANTHER); CYTOPLASMIC_DOMAIN (PHOBIUS); NON_CYTOPLASMIC_DOMAIN (PHOBIUS); TRANSMEMBRANE (PHOBIUS); TMhelix (TMHMM)
733	c171420_g1_i1_len_698_path_1_0_697_0	28	fat-like cadherin-related tumor suppressor homolog isoform x5	233	6.52E-97	77.60%	0.104	IPR001791 (SMART); IPR000742 (SMART); G3DSA:2.10.25.10 (GENE3D); IPR001791 (PFAM); IPR013320 (G3DSA:2.60.120.GENE3D); G3DSA:2.10.25.10 (GENE3D); PTHR24026 (PANTHER); PTHR24026:SF39 (PANTHER); IPR013032 (PROSITE_PATTERNS); IPR013032 (PROSITE_PATTERNS); IPR013032 (PROSITE_PATTERNS); IPR013032 (PROSITE_PATTERNS); IPR001791 (PROSITE_PROFILES); IPR000742 (PROSITE_PROFILES); IPR013320 (SUPERFAMILY)
734	c17148_g1_i1_len_822_path_1064_0_821_0	33	calcium-activated chloride channel regulator 2	274	2.77E-29	47.00%	0.118	IPR002035 (G3DSA:3.40.50.GENE3D); IPR013642 (PFAM); PTHR10579 (PANTHER); PTHR10579:SF8 (PANTHER); IPR002035 (PROSITE_PROFILES); IPR002035 (SUPERFAMILY)
735	c171613_g1_i1_len_1823_path_1_0_1822_2	68	partial	607	3.26E-116	76.80%	0.105	IPR002290 (SMART); G3DSA:3.30.200.20 (GENE3D); G3DSA:1.10.510.10 (GENE3D); IPR000719 (PFAM); PTHR24419:SF19 (PANTHER); PTHR24419 (PANTHER); IPR008271 (PROSITE_PATTERNS); IPR000719 (PROSITE_PROFILES); IPR011009 (SUPERFAMILY)
736	c171626_g1_i1_len_858_path_836_0_857_1	21	von willebrand factor type egf and pentraxin domain-containing protein 1	286	1.96E-95	69.90%	0.106	IPR000436 (SMART); G3DSA:2.10.70.10 (GENE3D); IPR000436 (PFAM); G3DSA:2.10.70.10 (GENE3D); PTHR19325:SF340 (PANTHER); PTHR19325 (PANTHER); IPR000436 (PROSITE_PROFILES); IPR000436 (PROSITE_PROFILES); IPR000436 (PROSITE_PROFILES); IPR000436 (PROSITE_PROFILES); IPR000436 (SUPERFAMILY); IPR000436 (SUPERFAMILY); IPR000436 (SUPERFAMILY); IPR000436 (SUPERFAMILY)
737	c171713_g1_i1_len_1068_path_1046_0_1067_2	38	gamma-aminobutyric acid type b receptor subunit 1	356	1.11E-164	81.60%	0.165	Coil (COILS); IPR002456 (PRINTS); PR01176 (PRINTS); IPR017978 (PFAM); IPR002455 (PANTHER); PTHR10519:SF3 (PANTHER); NON_CYTOPLASMIC_DOMAIN (PHOBIUS); TRANSMEMBRANE (PHOBIUS); NON_CYTOPLASMIC_DOMAIN (PHOBIUS); CYTOPLASMIC_DOMAIN (PHOBIUS); TRANSMEMBRANE (PHOBIUS); CYTOPLASMIC_DOMAIN (PHOBIUS); TRANSMEMBRANE (PHOBIUS); TRANSMEMBRANE (PHOBIUS); CYTOPLASMIC_DOMAIN (PHOBIUS); CYTOPLASMIC_DOMAIN (PHOBIUS); NON_CYTOPLASMIC_DOMAIN (PHOBIUS); TRANSMEMBRANE (PHOBIUS); TRANSMEMBRANE (PHOBIUS); IPR017978 (PROSITE_PROFILES); TMhelix (TMHMM); TMhelix (TMHMM); TMhelix (TMHMM); TMhelix (TMHMM); TMhelix (TMHMM); TMhelix (TMHMM)
738	c171731_g1_i1_len_1330_path_1308_0_1329_1	3867	low quality protein: glutathione peroxidase 3	443	3.99E-59	63.90%	0.21	IPR000889 (PFAM); IPR012336 (G3DSA:3.40.30.GENE3D); IPR000889 (PANTHER); IPR029760 (PROSITE_PATTERNS); TRANSMEMBRANE (PHOBIUS); CYTOPLASMIC_DOMAIN (PHOBIUS); NON_CYTOPLASMIC_DOMAIN (PHOBIUS); IPR000889 (PROSITE_PROFILES); IPR012336 (SUPERFAMILY); TMhelix (TMHMM)
739	c17175_g2_i1_len_238_path_466_0_237_1	2	preprotachykinin b	79	1.15E-19	62.50%	0.107	no IPS match

740	c171806_g1_i1_len_979_path_1_0_978_1	415	protein them6	326	2.64E-36	58.40%	0.129	Coil (COILS); PF13279 (PFAM); IPR029069 (G3DSA:3.10.129.GENE3D); PTHR12475 (PANTHER); NON_CYTOPLASMIC_DOMAIN (PHOBIUS); TRANSMEMBRANE (PHOBIUS); CYTOPLASMIC_DOMAIN (PHOBIUS); IPR029069 (SUPERFAMILY); TMhelix (TMHMM)
741	c171832_g1_i1_len_559_path_537_0_558_5	23	short neuropeptide f	186	2.17E-22	68.60%	0.175	no IPS match
742	c171932_g1_i1_len_471_path_449_0_470_0	13	protocadherin fat 2	157	2.84E-74	83.50%	0.098	IPR002126 (PRINTS); IPR002126 (SMART); IPR002126 (G3DSA:2.60.40.GENE3D); IPR002126 (PFAM); PTHR24026 (PANTHER); PTHR24026:SF39 (PANTHER); IPR020894 (PROSITE_PATTERNS); IPR002126 (PROSITE_PROFILES); IPR002126 (PROSITE_PROFILES); IPR015919 (SUPERFAMILY); IPR015919 (SUPERFAMILY)
743	c171933_g1_i1_len_350_path_328_0_349_3	6	cubilin isoform x1	117	3.26E-40	68.20%	0.11	IPR000859 (SMART); IPR000859 (PFAM); IPR000859 (G3DSA:2.60.120.GENE3D); PTHR10127 (PANTHER); IPR000859 (PROSITE_PROFILES); IPR000859 (SUPERFAMILY)
744	c172043_g1_i1_len_396_path_374_0_395_0	10	low-density lipoprotein receptor-related protein 2	132	1.37E-15	57.40%	0.115	IPR002172 (PRINTS); IPR002172 (SMART); IPR002172 (G3DSA:4.10.400.GENE3D); IPR002172 (PFAM); IPR002172 (G3DSA:4.10.400.GENE3D); IPR002172 (G3DSA:4.10.400.GENE3D); PTHR10529 (PANTHER); IPR002172 (PROSITE_PROFILES); IPR002172 (SUPERFAMILY); IPR002172 (SUPERFAMILY); IPR002172 (SUPERFAMILY)
745	c172057_g1_i1_len_310_path_1_0_309_4	23	heat shock cognate 71 kda	103	1.43E-49	98.00%	0.155	IPR013126 (PRINTS); G3DSA:3.30.30.30 (GENE3D); IPR013126 (PFAM); G3DSA:3.30.420.40 (GENE3D); PTHR19375:SF142 (PANTHER); PTHR19375 (PANTHER); IPR018181 (PROSITE_PATTERNS); SSF53067 (SUPERFAMILY)
746	c172141_g1_i1_len_289_path_267_0_288_0	20	iron zinc purple acid phosphatase-like protein isoform x2	97	2.69E-47	89.30%	0.1	IPR004843 (PFAM); IPR029052 (G3DSA:3.60.21.GENE3D); PTHR22953:SF9 (PANTHER); PTHR22953 (PANTHER); IPR029052 (SUPERFAMILY)
747	c172300_g1_i1_len_569_path_534_0_520_10 55_521_532_1055_533_544_1055_545_556_ 1055_557_568_4	49	protoporphyrin ix magnesium chelatase	190	5.70E-11	67.50%	0.12	TRANSMEMBRANE (PHOBIUS); TRANSMEMBRANE (PHOBIUS); CYTOPLASMIC_DOMAIN (PHOBIUS); NON_CYTOPLASMIC_DOMAIN (PHOBIUS); CYTOPLASMIC_DOMAIN (PHOBIUS)
748	c172320_g1_i1_len_700_path_1_0_699_1	183	diuretic hormone class 2-like	233	1.36E-29	73.30%	0.155	NON_CYTOPLASMIC_DOMAIN (PHOBIUS); CYTOPLASMIC_DOMAIN (PHOBIUS); TRANSMEMBRANE (PHOBIUS); TMhelix (TMHMM)
749	c172410_g1_i1_len_430_path_1_0_429_4	22	adp-ribosylation factor-like protein 16	143	1.62E-27	73.10%	0.26	IPR006689 (PFAM); IPR027417 (G3DSA:3.40.50.GENE3D); PTHR11711:SF42 (PANTHER); PTHR11711 (PANTHER); IPR027417 (SUPERFAMILY)
750	c172437_g1_i1_len_1333_path_1_0_1332_2	48	eukaryotic translation initiation factor 2-alpha kinase	444	1.95E-88	67.00%	0.101	IPR002290 (SMART); IPR000719 (PFAM); G3DSA:1.10.510.10 (GENE3D); PTHR11042 (PANTHER); PTHR11042:SF86 (PANTHER); IPR008271 (PROSITE_PATTERNS); IPR000719 (PROSITE_PROFILES); IPR011009 (SUPERFAMILY)
751	c172496_g1_i1_len_701_path_1_0_700_1	25	zinc finger protein ozf-like	234	1.56E-27	56.50%	0.107	IPR015880 (SMART); IPR013087 (G3DSA:3.30.160.GENE3D); PF13465 (PFAM); IPR013087 (G3DSA:3.30.160.GENE3D); IPR013087 (G3DSA:3.30.160.GENE3D); PTHR24409:SF13 (PANTHER); PTHR24409 (PANTHER); IPR007087 (PROSITE_PATTERNS); IPR007087 (PROSITE_PATTERNS); IPR007087 (PROSITE_PATTERNS); IPR007087 (PROSITE_PROFILES); IPR007087 (PROSITE_PROFILES); IPR007087 (PROSITE_PROFILES); SSF57667 (SUPERFAMILY); SSF57667 (SUPERFAMILY)
755	c1725_g2_i1_len_1000_path_978_0_999_4	36	laccase- partial	333	1.99E-75	67.50%	0.116	IPR008972 (G3DSA:2.60.40.GENE3D); IPR008972 (G3DSA:2.60.40.GENE3D); IPR011707 (PFAM); PTHR11709:SF33 (PANTHER); PTHR11709 (PANTHER); IPR008972 (SUPERFAMILY)
752	c172505_g1_i1_len_2031_path_2009_0_2030 _3	74	big brain	677	8.98E-136	61.60%	0.236	IPR000425 (PRINTS); IPR000425 (TIGRFAM); IPR023271 (G3DSA:1.20.1080.GENE3D); IPR000425 (PFAM); PTHR19139:SF85 (PANTHER); IPR000425 (PANTHER); IPR022357 (PROSITE_PATTERNS); CYTOPLASMIC_DOMAIN (PHOBIUS); TRANSMEMBRANE (PHOBIUS); CYTOPLASMIC_DOMAIN (PHOBIUS); CYTOPLASMIC_DOMAIN (PHOBIUS); CYTOPLASMIC_DOMAIN (PHOBIUS); TRANSMEMBRANE (PHOBIUS); TRANSMEMBRANE (PHOBIUS); NON_CYTOPLASMIC_DOMAIN (PHOBIUS); NON_CYTOPLASMIC_DOMAIN (PHOBIUS); TRANSMEMBRANE (PHOBIUS); TRANSMEMBRANE (PHOBIUS); NON_CYTOPLASMIC_DOMAIN (PHOBIUS); TRANSMEMBRANE (PHOBIUS); IPR023271 (SUPERFAMILY); TMhelix (TMHMM); TMhelix (TMHMM); TMhelix (TMHMM); TMhelix (TMHMM); TMhelix (TMHMM); TMhelix (TMHMM)

753	c172531_g1_i1_len_424_path_1_0_423_3	11	low-density lipoprotein	142	2.35E-48	72.20%	0.134	IPR000742 (SMART); IPR002172 (SMART); IPR001881 (SMART); IPR001881 (PFAM); G3DSA:2.10.25.10 (GENE3D); IPR002172 (G3DSA:4.10.400.GENE3D); G3DSA:2.10.25.10 (GENE3D); IPR002172 (PFAM); PTHR10529 (PANTHER); PTHR10529:SF3 (PANTHER); IPR018097 (PROSITE_PATTERNS); IPR000152 (PROSITE_PATTERNS); IPR018097 (PROSITE_PATTERNS); IPR023415 (PROSITE_PATTERNS); IPR000152 (PROSITE_PATTERNS); IPR002172 (PROSITE_PROFILES); SSF57196 (SUPERFAMILY); SSF57196 (SUPERFAMILY); IPR002172 (SUPERFAMILY)
754	c172596_g1_i1_len_757_path_735_0_756_4	18	zinc finger protein 345 isoform x3	252	3.19E-65	60.50%	0.098	IPR015880 (SMART); IPR013087 (G3DSA:3.30.160.GENE3D); IPR013087 (G3DSA:3.30.160.GENE3D); IPR013087 (G3DSA:3.30.160.GENE3D); IPR013087 (G3DSA:3.30.160.GENE3D); IPR007087 (PFAM); PF13465 (PFAM); PTHR24409 (PANTHER); IPR007087 (PROSITE_PATTERNS); IPR007087 (PROSITE_PATTERNS); IPR007087 (PROSITE_PATTERNS); IPR007087 (PROSITE_PATTERNS); IPR007087 (PROSITE_PATTERNS); IPR007087 (PROSITE_PATTERNS); IPR007087 (PROSITE_PATTERNS); IPR007087 (PROSITE_PATTERNS); IPR007087 (PROSITE_PROFILES); IPR007087 (PROSITE_PROFILES); IPR007087 (PROSITE_PROFILES); IPR007087 (PROSITE_PROFILES); IPR007087 (PROSITE_PROFILES); IPR007087 (PROSITE_PROFILES); IPR007087 (PROSITE_PROFILES); IPR007087 (PROSITE_PROFILES); SSF57667 (SUPERFAMILY); SSF57667 (SUPERFAMILY); SSF57667 (SUPERFAMILY); SSF57667 (SUPERFAMILY); SSF57667 (SUPERFAMILY)
756	c172660_g1_i1_len_921_path_899_0_920_2	28	---NA---	307			0.158	IPR002172 (SMART); IPR002172 (G3DSA:4.10.400.GENE3D); IPR002172 (PFAM); IPR023415 (PROSITE_PATTERNS); TRANSMEMBRANE (PHOBIUS); NON_CYTOPLASMIC_DOMAIN (PHOBIUS); CYTOPLASMIC_DOMAIN (PHOBIUS); IPR002172 (PROSITE_PROFILES); IPR002172 (SUPERFAMILY)
757	c172664_g1_i1_len_519_path_1_0_518_3	14	endonuclease mitochondrial	173	4.93E-72	73.60%	0.195	IPR020821 (SMART); IPR020821 (G3DSA:3.40.570.GENE3D); IPR001604 (PFAM); PTHR13966 (PANTHER); IPR018524 (PROSITE_PATTERNS); SSF54060 (SUPERFAMILY)
758	c172692_g1_i1_len_2005_path_1_0_2004_1	45	irregular chiasm c-roughest protein isoform x1	668	1.67E-122	58.90%	0.111	IPR003599 (SMART); IPR003598 (SMART); IPR013098 (PFAM); IPR013783 (G3DSA:2.60.40.GENE3D); PTHR11640 (PANTHER); PTHR11640:SF54 (PANTHER); NON_CYTOPLASMIC_DOMAIN (PHOBIUS); CYTOPLASMIC_DOMAIN (PHOBIUS); TRANSMEMBRANE (PHOBIUS); IPR007110 (PROSITE_PROFILES); SSF48726 (SUPERFAMILY); TMhelix (TMHMM)
760	c1727_g1_i1_len_1253_path_1_0_1252_2	40	zinc finger protein 568-like	417	1.88E-66	52.10%	0.103	IPR015880 (SMART); IPR013087 (G3DSA:3.30.160.GENE3D); PF13465 (PFAM); PF13894 (PFAM); IPR013087 (G3DSA:3.30.160.GENE3D); IPR013087 (G3DSA:3.30.160.GENE3D); IPR013087 (G3DSA:3.30.160.GENE3D); IPR007087 (PFAM); PTHR24409 (PANTHER); IPR007087 (PROSITE_PATTERNS); IPR007087 (PROSITE_PATTERNS); IPR007087 (PROSITE_PATTERNS); IPR007087 (PROSITE_PATTERNS); IPR007087 (PROSITE_PATTERNS); IPR007087 (PROSITE_PATTERNS); IPR007087 (PROSITE_PATTERNS); IPR007087 (PROSITE_PATTERNS); IPR007087 (PROSITE_PROFILES); IPR007087 (PROSITE_PROFILES); IPR007087 (PROSITE_PROFILES); IPR007087 (PROSITE_PROFILES); IPR007087 (PROSITE_PROFILES); IPR007087 (PROSITE_PROFILES); IPR007087 (PROSITE_PROFILES); IPR007087 (PROSITE_PROFILES); SSF57667 (SUPERFAMILY); SSF57667 (SUPERFAMILY); SSF57667 (SUPERFAMILY); SSF57667 (SUPERFAMILY); SSF57667 (SUPERFAMILY); SSF57667 (SUPERFAMILY)
759	c172788_g1_i1_len_941_path_919_0_940_5	28	thrombospondin type-1 domain-containing protein 4-like	313	2.66E-56	59.00%	0.11	IPR000884 (SMART); IPR010909 (PFAM); G3DSA:2.20.100.10 (GENE3D); IPR000884 (PFAM); PTHR13723 (PANTHER); IPR000884 (PROSITE_PROFILES); IPR010909 (PROSITE_PROFILES); IPR000884 (PROSITE_PROFILES); IPR000884 (SUPERFAMILY); IPR000884 (SUPERFAMILY)
761	c172827_g1_i1_len_2399_path_1_0_2398_1	209	phospholipase a-2-activating protein	800	0	66.00%	0.11	IPR001680 (SMART); IPR015943 (G3DSA:2.130.10.GENE3D); IPR013535 (PFAM); IPR015155 (PFAM); IPR001680 (PFAM); PTHR19849 (PANTHER); PTHR19849:SF0 (PANTHER); IPR001680 (PROSITE_PROFILES); IPR001680 (PROSITE_PROFILES); IPR001680 (PROSITE_PROFILES); IPR015155 (PROSITE_PROFILES); IPR013535 (PROSITE_PROFILES); IPR017986 (PROSITE_PROFILES); IPR001680 (PROSITE_PROFILES); IPR017986 (SUPERFAMILY); IPR016024 (SUPERFAMILY)
762	c172847_g1_i1_len_236_path_1_0_235_4	3	---NA---	79			0.133	IPR029058 (G3DSA:3.40.50.GENE3D)

763	c172890_g1_i1_len_161_path_139_0_160_0	2	stromal cell-derived factor 2	54	3.04E-21	83.50%	0.099	G3DSA:2.80.10.50 (GENE3D); IPR027005 (PANTHER); IPR016093 (SUPERFAMILY)
764	c172981_g1_i1_len_1019_path_997_0_1018_2	37	nedd4 family-interacting protein 1-like	339	2.46E-82	62.20%	0.214	IPR019325 (PFAM); PTHR13396 (PANTHER); TRANSMEMBRANE (PHOBIUS); TRANSMEMBRANE (PHOBIUS); NON_CYTOPLASMIC_DOMAIN (PHOBIUS); NON_CYTOPLASMIC_DOMAIN (PHOBIUS); TRANSMEMBRANE (PHOBIUS); TRANSMEMBRANE (PHOBIUS); CYTOPLASMIC_DOMAIN (PHOBIUS); NON_CYTOPLASMIC_DOMAIN (PHOBIUS); CYTOPLASMIC_DOMAIN (PHOBIUS); TMhelix (TMHMM); TMhelix (TMHMM); TMhelix (TMHMM)
765	c172999_g1_i1_len_763_path_741_0_762_2	17	spermatogenesis-associated protein partial	254	2.12E-06	72.00%	0.101	no IPS match
766	c173056_g1_i1_len_515_path_493_0_514_0	6	ankyrin repeat	172	6.46E-23	59.30%	0.231	IPR002110 (PRINTS); IPR002110 (SMART); IPR020683 (G3DSA:1.25.40.GENE3D); IPR020683 (PFAM); PTHR24198 (PANTHER); IPR002110 (PROSITE_PROFILES); IPR002110 (PROSITE_PROFILES); IPR020683 (PROSITE_PROFILES); IPR020683 (SUPERFAMILY)
767	c173088_g1_i1_len_593_path_571_0_592_1	13	ankyrin-2-like isoform x2	198	6.23E-17	54.80%	0.142	IPR002110 (PRINTS); IPR002110 (SMART); IPR020683 (G3DSA:1.25.40.GENE3D); IPR002110 (PFAM); IPR020683 (PFAM); PTHR24198 (PANTHER); IPR002110 (PROSITE_PROFILES); IPR002110 (PROSITE_PROFILES); IPR020683 (PROSITE_PROFILES); IPR002110 (PROSITE_PROFILES); IPR002110 (PROSITE_PROFILES); IPR020683 (SUPERFAMILY)
768	c173092_g1_i1_len_381_path_359_0_380_5	5	adenosine monophosphate-protein transferase ficd homolog	127	6.97E-61	85.80%	0.107	IPR003812 (G3DSA:1.10.3290.GENE3D); IPR003812 (PFAM); PTHR13504:SF12 (PANTHER); PTHR13504 (PANTHER); IPR003812 (PROSITE_PROFILES); IPR003812 (SUPERFAMILY)
769	c173102_g1_i1_len_694_path_672_0_693_0	18	chorion peroxidase	232	2.63E-45	74.60%	0.157	IPR019791 (PFAM); IPR019791 (G3DSA:1.10.640.GENE3D); PTHR11475 (PANTHER); IPR019791 (PROSITE_PROFILES); IPR010255 (SUPERFAMILY)
770	c173186_g1_i1_len_327_path_1_0_326_0	6	serine threonine-protein kinase 17b-like	109	1.44E-52	86.10%	0.109	IPR000719 (PFAM); G3DSA:1.10.510.10 (GENE3D); PTHR24342 (PANTHER); IPR000719 (PROSITE_PROFILES); IPR011009 (SUPERFAMILY)
771	c173195_g1_i1_len_351_path_329_0_350_2	10	prostaglandin f synthase-like	117	3.31E-38	71.20%	0.108	IPR020471 (PRINTS); IPR023210 (PFAM); IPR023210 (G3DSA:3.20.20.GENE3D); IPR001395 (PANTHER); PTHR11732:SF133 (PANTHER); IPR018170 (PROSITE_PATTERNS); IPR023210 (SUPERFAMILY)
772	c173214_g1_i1_len_1052_path_1_0_1051_2	40	hemicentin-1 isoform x2	350	3.92E-86	57.20%	0.105	PR01832 (PRINTS); IPR013162 (PFAM); IPR013783 (G3DSA:2.60.40.GENE3D); IPR013783 (G3DSA:2.60.40.GENE3D); PF13895 (PFAM); PTHR11640:SF6 (PANTHER); PTHR11640 (PANTHER); IPR007110 (PROSITE_PROFILES); IPR007110 (PROSITE_PROFILES); IPR007110 (PROSITE_PROFILES); SSF48726 (SUPERFAMILY); SSF48726 (SUPERFAMILY); SSF48726 (SUPERFAMILY)
773	c173227_g1_i1_len_432_path_1_0_431_0	6	deleted in malignant brain tumors 1	144	5.48E-13	44.80%	0.103	IPR017448 (G3DSA:3.10.250.GENE3D); IPR001190 (PFAM); PTHR19331:SF253 (PANTHER); PTHR19331 (PANTHER); IPR001190 (PROSITE_PROFILES); IPR001190 (PROSITE_PROFILES); IPR017448 (SUPERFAMILY); IPR017448 (SUPERFAMILY)
774	c173280_g1_i1_len_682_path_1_0_681_1	16	hexamerin 1	227	1.47E-70	81.20%	0.101	IPR005203 (PFAM); IPR005203 (G3DSA:2.60.40.GENE3D); IPR013788 (PANTHER); PTHR11511:SF5 (PANTHER); IPR014756 (SUPERFAMILY)
775	c173288_g1_i1_len_1049_path_1_0_1048_0	36	latrophilin-3 isoform x2	350	1.86E-61	67.20%	0.411 Y	IPR000832 (PRINTS); IPR000832 (PFAM); PTHR12011 (PANTHER); TRANSMEMBRANE (PHOBIUS); TRANSMEMBRANE (PHOBIUS); NON_CYTOPLASMIC_DOMAIN (PHOBIUS); TRANSMEMBRANE (PHOBIUS); TRANSMEMBRANE (PHOBIUS); CYTOPLASMIC_DOMAIN (PHOBIUS); CYTOPLASMIC_DOMAIN (PHOBIUS); NON_CYTOPLASMIC_DOMAIN (PHOBIUS); TRANSMEMBRANE (PHOBIUS); CYTOPLASMIC_DOMAIN (PHOBIUS); TRANSMEMBRANE (PHOBIUS); NON_CYTOPLASMIC_DOMAIN (PHOBIUS); CYTOPLASMIC_DOMAIN (PHOBIUS); IPR017981 (PROSITE_PROFILES); TMhelix (TMHMM); TMhelix (TMHMM); TMhelix (TMHMM); TMhelix (TMHMM); TMhelix (TMHMM)
776	c173362_g1_i1_len_1136_path_1114_0_1135_0	39	dbh-like monooxygenase protein partial	379	4.74E-14	50.10%	0.107	IPR014784 (G3DSA:2.60.120.GENE3D); PF03712 (PFAM); IPR000945 (PANTHER); IPR008977 (SUPERFAMILY)
779	c1735_g1_i1_len_1231_path_1209_0_1230_1	41	very low-density lipoprotein partial	410	1.94E-23	48.60%	0.205	IPR002172 (PRINTS); IPR002172 (SMART); IPR002172 (PFAM); IPR002172 (G3DSA:4.10.400.GENE3D); IPR002172 (G3DSA:4.10.400.GENE3D); IPR002172 (G3DSA:4.10.400.GENE3D); PTHR10529 (PANTHER); IPR023415 (PROSITE_PATTERNS); TRANSMEMBRANE (PHOBIUS); CYTOPLASMIC_DOMAIN (PHOBIUS); NON_CYTOPLASMIC_DOMAIN (PHOBIUS); IPR002172 (PROSITE_PROFILES); IPR002172 (PROSITE_PROFILES); IPR002172 (PROSITE_PROFILES); IPR002172 (PROSITE_PROFILES); IPR002172 (PROSITE_PROFILES); IPR002172 (SUPERFAMILY); IPR002172 (SUPERFAMILY); IPR002172 (SUPERFAMILY); IPR002172 (SUPERFAMILY)

780	c1735_g1_i1_len_1231_path_1209_0_1230_2	41	very low-density lipoprotein receptor-like isoform x2	410	1.13E-21	43.60%	0.102	IPR002172 (PRINTS); IPR002172 (SMART); IPR002172 (G3DSA:4.10.400.GENE3D); IPR002172 (PFAM); IPR002172 (G3DSA:4.10.400.GENE3D); G3DSA:4.10.1220.10 (GENE3D); PTHR10529 (PANTHER); IPR002172 (PROSITE_PROFILES); IPR002172 (PROSITE_PROFILES); IPR002172 (PROSITE_PROFILES); IPR002172 (SUPERFAMILY); IPR002172 (SUPERFAMILY); IPR002172 (SUPERFAMILY); IPR002172 (SUPERFAMILY)
777	c17356_g1_i1_len_1327_path_1355_0_1326_3	80	lipophorin receptor	443	2.28E-98	56.60%	0.304	IPR002172 (PRINTS); IPR000742 (SMART); IPR000033 (SMART); IPR002172 (SMART); IPR001881 (SMART); IPR002172 (G3DSA:4.10.400.GENE3D); IPR001881 (PFAM); G3DSA:2.10.25.10 (GENE3D); IPR002172 (G3DSA:4.10.400.GENE3D); IPR000033 (PFAM); IPR011042 (G3DSA:2.120.10.GENE3D); G3DSA:2.10.25.10 (GENE3D); IPR002172 (PFAM); PTHR10529 (PANTHER); IPR018097 (PROSITE_PATTERNS); IPR000152 (PROSITE_PATTERNS); IPR023415 (PROSITE_PATTERNS); IPR023415 (PROSITE_PATTERNS); IPR023415 (PROSITE_PATTERNS); IPR023415 (PROSITE_PATTERNS); CYTOPLASMIC_DOMAIN (PHOBIUS); NON_CYTOPLASMIC_DOMAIN (PHOBIUS); TRANSMEMBRANE (PHOBIUS); IPR000033 (PROSITE_PROFILES); IPR002172 (PROSITE_PROFILES); IPR002172 (PROSITE_PROFILES); IPR000033 (PROSITE_PROFILES); IPR002172 (PROSITE_PROFILES); IPR000033 (PROSITE_PROFILES); IPR002172 (PROSITE_PROFILES); SSF63825 (SUPERFAMILY); IPR002172 (SUPERFAMILY); IPR002172 (SUPERFAMILY); IPR009030 (SUPERFAMILY); IPR002172 (SUPERFAMILY); IPR002172 (SUPERFAMILY); TMhelix (TMHMM)
778	c173595_g1_i1_len_3140_path_1_0_3139_4	202	macrophage mannose receptor 1	1047	9.20E-20	38.50%	0.14	IPR001304 (SMART); IPR016186 (G3DSA:3.10.100.GENE3D); IPR001304 (PFAM); IPR016186 (G3DSA:3.10.100.GENE3D); PTHR22803:SF55 (PANTHER); PTHR22803 (PANTHER); IPR001304 (PROSITE_PROFILES); IPR001304 (PROSITE_PROFILES); IPR001304 (PROSITE_PROFILES); IPR016187 (SUPERFAMILY); IPR016187 (SUPERFAMILY); IPR016187 (SUPERFAMILY)
782	c17366_g1_i1_len_251_path_121_0_70_30_71_130_30_131_190_30_191_250_3	5	hypothetical protein KLMA_20728	84	7.59E-06	50.50%	0.101	no IPS match
781	c173662_g1_i1_len_270_path_248_0_269_2	4	disease resistance family protein lrr family	90	2.76E-06	60.50%	0.096	IPR025875 (PFAM); G3DSA:3.80.10.10 (GENE3D); SSF52058 (SUPERFAMILY)
783	c173689_g1_i1_len_300_path_1_0_299_4	6	beta- -n-acetylgalactosaminyltransferase bre-4	100	8.10E-56	88.40%	0.105	IPR003859 (PRINTS); IPR027791 (PFAM); IPR029044 (G3DSA:3.90.550.GENE3D); PTHR19300:SF30 (PANTHER); IPR003859 (PANTHER); IPR029044 (SUPERFAMILY)
784	c173820_g1_i1_len_577_path_555_0_576_0	7	achain crystal structure of engineered northeast structural genomics consortium target	193	6.31E-17	55.10%	0.121	IPR002110 (SMART); IPR020683 (G3DSA:1.25.40.GENE3D); IPR020683 (PFAM); PTHR24193 (PANTHER); IPR002110 (PROSITE_PROFILES); IPR020683 (PROSITE_PROFILES); IPR002110 (PROSITE_PROFILES); IPR020683 (SUPERFAMILY)
785	c17401_g2_i1_len_1411_path_835_0_1410_4	61	neuropilin and tolloid-like protein 2-like	470	1.11E-126	61.00%	0.109	IPR000859 (SMART); IPR002172 (SMART); IPR002172 (G3DSA:4.10.400.GENE3D); IPR000859 (G3DSA:2.60.120.GENE3D); IPR000859 (PFAM); IPR000859 (G3DSA:2.60.120.GENE3D); PTHR10127 (PANTHER); PTHR10127:SF572 (PANTHER); NON_CYTOPLASMIC_DOMAIN (PHOBIUS); TRANSMEMBRANE (PHOBIUS); CYTOPLASMIC_DOMAIN (PHOBIUS); IPR000859 (PROSITE_PROFILES); IPR002172 (PROSITE_PROFILES); IPR000859 (PROSITE_PROFILES); IPR002172 (SUPERFAMILY); IPR000859 (SUPERFAMILY); IPR000859 (SUPERFAMILY); TMhelix (TMHMM)
786	c174047_g1_i1_len_279_path_257_0_278_0	4	transient receptor potential cation channel subfamily a member 1 homolog	93	1.57E-28	66.80%	0.103	IPR002110 (SMART); IPR020683 (G3DSA:1.25.40.GENE3D); IPR002110 (PFAM); IPR020683 (PFAM); PTHR24198 (PANTHER); IPR002110 (PROSITE_PROFILES); IPR020683 (PROSITE_PROFILES); IPR002110 (PROSITE_PROFILES); IPR020683 (SUPERFAMILY)
787	c174091_g1_i1_len_372_path_1_0_371_2	8	c-type mbl-2 protein	124	2.08E-11	54.00%	0.137	IPR001304 (SMART); IPR016186 (G3DSA:3.10.100.GENE3D); IPR001304 (PFAM); PTHR22802 (PANTHER); IPR001304 (PROSITE_PROFILES); IPR016187 (SUPERFAMILY)
788	c174119_g1_i1_len_271_path_1_0_270_5	5	tyrosine-protein phosphatase lar isoform x1	90	7.03E-34	80.70%	0.105	IPR013783 (G3DSA:2.60.40.GENE3D); IPR013098 (PFAM); PTHR19134 (PANTHER); PTHR19134:SF199 (PANTHER); IPR007110 (PROSITE_PROFILES); SSF48726 (SUPERFAMILY)
789	c174164_g1_i1_len_922_path_900_0_921_2	31	ankyrin repeat domain-containing protein 39	307	2.15E-53	66.80%	0.292	IPR002110 (SMART); IPR020683 (PFAM); IPR020683 (G3DSA:1.25.40.GENE3D); PTHR24148 (PANTHER); IPR002110 (PROSITE_PROFILES); IPR002110 (PROSITE_PROFILES); IPR020683 (PROSITE_PROFILES); IPR002110 (PROSITE_PROFILES); IPR020683 (SUPERFAMILY)

790	c174184_g1_i1_len_764_path_742_0_763_0	24	cytokine receptor	255	4.03E-40	57.50%	0.134	IPR003961 (SMART); IPR013783 (G3DSA:2.60.40.GENE3D); IPR003961 (PFAM); IPR013783 (G3DSA:2.60.40.GENE3D); PTHR10489 (PANTHER); IPR003961 (PROSITE_PROFILES); IPR003961 (PROSITE_PROFILES); IPR003961 (PROSITE_PROFILES); IPR003961 (SUPERFAMILY); IPR003961 (SUPERFAMILY)
791	c174196_g1_i1_len_412_path_1_0_411_3	9	papain family cysteine protease	138	6.61E-45	69.10%	0.245	IPR000668 (SMART); IPR000668 (PFAM); G3DSA:3.90.70.10 (GENE3D); PTHR12411:SF15 (PANTHER); IPR013128 (PANTHER); SSF54001 (SUPERFAMILY)
792	c174268_g1_i1_len_665_path_1_0_664_2	18	centaurin-gamma 1a	221	3.76E-77	71.20%	0.164	IPR002110 (SMART); IPR020683 (G3DSA:1.25.40.GENE3D); IPR001164 (PFAM); IPR020683 (PFAM); PTHR23180 (PANTHER); PTHR23180:SF229 (PANTHER); IPR020683 (PROSITE_PROFILES); IPR002110 (PROSITE_PROFILES); IPR020683 (SUPERFAMILY); SSF57863 (SUPERFAMILY)
793	c174298_g1_i1_len_1112_path_1090_0_1111_1	505	histone -like	371	2.20E-81	99.20%	0.105	IPR002119 (PRINTS); IPR002119 (SMART); IPR007125 (PFAM); IPR009072 (G3DSA:1.10.20.GENE3D); PTHR23430:SF34 (PANTHER); PTHR23430 (PANTHER); IPR002119 (PROSITE_PATTERNS); NON_CYTOPLASMIC_DOMAIN (PHOBIUS); TRANSMEMBRANE (PHOBIUS); CYTOPLASMIC_DOMAIN (PHOBIUS); TRANSMEMBRANE (PHOBIUS); CYTOPLASMIC_DOMAIN (PHOBIUS); IPR009072 (SUPERFAMILY)
794	c174348_g1_i1_len_826_path_1_0_825_0	12	prophenoloxidase activating factor	276	1.05E-60	62.30%	0.099	IPR001314 (PRINTS); G3DSA:2.40.10.10 (GENE3D); G3DSA:2.40.10.10 (GENE3D); IPR001254 (PFAM); PTHR24268 (PANTHER); PTHR24268:SF95 (PANTHER); IPR018114 (PROSITE_PATTERNS); IPR001254 (PROSITE_PROFILES); IPR009003 (SUPERFAMILY)
795	c174371_g1_i1_len_790_path_1_0_789_5	12	gastrula zinc finger	263	1.96E-43	57.00%	0.098	IPR015880 (SMART); PF13465 (PFAM); IPR013087 (G3DSA:3.30.160.GENE3D); IPR007087 (PFAM); IPR013087 (G3DSA:3.30.160.GENE3D); PTHR24409 (PANTHER); IPR007087 (PROSITE_PATTERNS); IPR007087 (PROSITE_PATTERNS); IPR007087 (PROSITE_PATTERNS); IPR007087 (PROSITE_PATTERNS); IPR007087 (PROSITE_PATTERNS); IPR007087 (PROSITE_PATTERNS); IPR007087 (PROSITE_PROFILES); IPR007087 (PROSITE_PROFILES); IPR007087 (PROSITE_PROFILES); IPR007087 (PROSITE_PROFILES); IPR007087 (PROSITE_PROFILES); IPR007087 (PROSITE_PROFILES); SSF57667 (SUPERFAMILY); SSF57667 (SUPERFAMILY); SSF57667 (SUPERFAMILY)
796	c174385_g1_i1_len_482_path_460_0_481_1	12	dnaj homolog subfamily c member 17	161	9.87E-57	77.50%	0.105	Coil (COILS); Coil (COILS); IPR001623 (PRINTS); IPR001623 (SMART); IPR001623 (PFAM); IPR001623 (G3DSA:1.10.287.GENE3D); PTHR24078:SF153 (PANTHER); PTHR24078 (PANTHER); IPR001623 (PROSITE_PROFILES); IPR001623 (SUPERFAMILY)
797	c174551_g1_i1_len_322_path_1_0_321_3	5	pdz domain protein	108	2.57E-41	83.10%	0.138	IPR001478 (SMART); IPR001478 (G3DSA:2.30.42.GENE3D); IPR001478 (PFAM); PTHR19964 (PANTHER); PTHR19964:SF22 (PANTHER); IPR001478 (PROSITE_PROFILES); IPR001478 (SUPERFAMILY)
798	c174674_g1_i1_len_1282_path_1260_0_1281_5	39	endo-beta- -glucanase	427	3.88E-51	49.10%	0.911Y	IPR012341 (G3DSA:1.50.10.GENE3D); IPR001701 (PFAM); PTHR22298 (PANTHER); NON_CYTOPLASMIC_DOMAIN (PHOBIUS); SIGNAL_PEPTIDE_C_REGION (PHOBIUS); SIGNAL_PEPTIDE_N_REGION (PHOBIUS); SIGNAL_PEPTIDE_H_REGION (PHOBIUS); SIGNAL_PEPTIDE (PHOBIUS); SignalP-TM (SIGNALP_GRAM_POSITIVE); SignalP-noTM (SIGNALP_GRAM_NEGATIVE); SignalP-noTM (SIGNALP_EUK); IPR008928 (SUPERFAMILY); IPR008965 (SUPERFAMILY)
800	c17468_g1_i1_len_611_path_339_0_442_25_443_610_2	11	tequila, partial	203	3.49E-70	70.30%	0.111	IPR001190 (PRINTS); IPR017448 (SMART); IPR002172 (SMART); IPR001190 (PFAM); IPR002172 (G3DSA:4.10.400.GENE3D); IPR002172 (PFAM); IPR017448 (G3DSA:3.10.250.GENE3D); PTHR19331 (PANTHER); IPR023415 (PROSITE_PATTERNS); IPR001190 (PROSITE_PROFILES); IPR002172 (PROSITE_PROFILES); IPR001190 (PROSITE_PROFILES); IPR017448 (SUPERFAMILY); IPR017448 (SUPERFAMILY); IPR002172 (SUPERFAMILY)
799	c174686_g1_i1_len_646_path_7_0_645_3	1663	protein bv8	216	2.74E-20	59.10%	0.177	G3DSA:2.10.80.10 (GENE3D)
801	c174770_g1_i1_len_3316_path_3294_0_3315_4	948	14-3-3 protein epsilon	1105	2.31E-161	95.20%	0.165	Coil (COILS); IPR000308 (PRINTS); IPR023410 (SMART); G3DSA:1.20.190.20 (GENE3D); IPR023410 (PFAM); IPR000308 (PANTHER); IPR023409 (PROSITE_PATTERNS); IPR023409 (PROSITE_PATTERNS); NON_CYTOPLASMIC_DOMAIN (PHOBIUS); TRANSMEMBRANE (PHOBIUS); CYTOPLASMIC_DOMAIN (PHOBIUS); TRANSMEMBRANE (PHOBIUS); NON_CYTOPLASMIC_DOMAIN (PHOBIUS); CYTOPLASMIC_DOMAIN (PHOBIUS); TRANSMEMBRANE (PHOBIUS); IPR023410 (SUPERFAMILY); TMhelix (TMHMM)
802	c174808_g1_i1_len_312_path_290_0_311_0	9	cytochrome p450 mitochondrial-like	104	1.79E-30	63.30%	0.133	IPR002397 (PRINTS); IPR001128 (G3DSA:1.10.630.GENE3D); IPR001128 (PFAM); PTHR24291 (PANTHER); IPR001128 (SUPERFAMILY)

803	c174811_g1_i1_len_971_path_949_0_970_5	30	cysteine-rich with egf-like domain protein 2	323	1.91E-106	67.40%	0.289	IPR021852 (PFAM); G3DSA:2.10.25.10 (GENE3D); IPR001881 (PFAM); PTHR24838:SF13 (PANTHER); PTHR24838 (PANTHER); IPR002049 (PROSITE_PATTERNS); IPR013032 (PROSITE_PATTERNS); IPR018097 (PROSITE_PATTERNS); TRANSMEMBRANE (PHOBIUS); NON_CYTOPLASMIC_DOMAIN (PHOBIUS); CYTOPLASMIC_DOMAIN (PHOBIUS); IPR000742 (PROSITE_PROFILES); TMhelix (TMHMM)
804	c174858_g1_i1_len_560_path_538_0_559_3	16	thrombospondin type-1 domain-containing protein 4-like	187	6.53E-40	77.10%	0.104	IPR010294 (PFAM); PTHR13723 (PANTHER)
805	c174881_g1_i1_len_155_path_133_0_154_3	1	hemicentin- partial	52	1.55E-14	73.10%	0.126	IPR023413 (G3DSA:2.40.155.GENE3D); PTHR19897:SF158 (PANTHER); PTHR19897 (PANTHER); IPR006605 (PROSITE_PROFILES); IPR009017 (SUPERFAMILY)
806	c175170_g1_i1_len_906_path_884_0_905_0	27	low quality protein: papilin-like	302	3.99E-144	81.80%	0.1	IPR002223 (PRINTS); IPR002223 (SMART); IPR002223 (G3DSA:4.10.410.GENE3D); IPR002223 (PFAM); IPR002223 (G3DSA:4.10.410.GENE3D); PTHR10083 (PANTHER); IPR020901 (PROSITE_PATTERNS); IPR020901 (PROSITE_PATTERNS); IPR002223 (PROSITE_PROFILES); IPR002223 (PROSITE_PROFILES); IPR002223 (SUPERFAMILY); IPR002223 (SUPERFAMILY)
808	c1753_g1_i1_len_3051_path_53_0_3050_0	326	otopetrin-2-like isoform x3	1017	0	70.20%	0.139	IPR004878 (PFAM); PTHR21522:SF41 (PANTHER); IPR004878 (PANTHER); TRANSMEMBRANE (PHOBIUS); TRANSMEMBRANE (PHOBIUS); NON_CYTOPLASMIC_DOMAIN (PHOBIUS); NON_CYTOPLASMIC_DOMAIN (PHOBIUS); TRANSMEMBRANE (PHOBIUS); CYTOPLASMIC_DOMAIN (PHOBIUS); NON_CYTOPLASMIC_DOMAIN (PHOBIUS); CYTOPLASMIC_DOMAIN (PHOBIUS); TRANSMEMBRANE (PHOBIUS); CYTOPLASMIC_DOMAIN (PHOBIUS); CYTOPLASMIC_DOMAIN (PHOBIUS); CYTOPLASMIC_DOMAIN (PHOBIUS); NON_CYTOPLASMIC_DOMAIN (PHOBIUS); NON_CYTOPLASMIC_DOMAIN (PHOBIUS); CYTOPLASMIC_DOMAIN (PHOBIUS); TRANSMEMBRANE (PHOBIUS); TRANSMEMBRANE (PHOBIUS); TRANSMEMBRANE (PHOBIUS); TRANSMEMBRANE (PHOBIUS); TRANSMEMBRANE (PHOBIUS); TRANSMEMBRANE (PHOBIUS); TRANSMEMBRANE (PHOBIUS); CYTOPLASMIC_DOMAIN (PHOBIUS); TRANSMEMBRANE (PHOBIUS); NON_CYTOPLASMIC_DOMAIN (PHOBIUS); TMhelix (TMHMM); TMhelix (TMHMM); TMhelix (TMHMM); TMhelix (TMHMM); TMhelix (TMHMM); TMhelix (TMHMM); TMhelix (TMHMM); TMhelix (TMHMM); TMhelix (TMHMM); TMhelix (TMHMM); TMhelix (TMHMM); TMhelix (TMHMM)
807	c175323_g1_i1_len_414_path_392_0_413_2	8	zinc finger protein	138	2.93E-27	56.00%	0.108	IPR015880 (SMART); IPR013087 (G3DSA:3.30.160.GENE3D); IPR013087 (G3DSA:3.30.160.GENE3D); PF13894 (PFAM); IPR007087 (PFAM); IPR013087 (G3DSA:3.30.160.GENE3D); PTHR24409 (PANTHER); PTHR24409:SF14 (PANTHER); IPR007087 (PROSITE_PATTERNS); IPR007087 (PROSITE_PATTERNS); IPR007087 (PROSITE_PATTERNS); IPR007087 (PROSITE_PATTERNS); IPR007087 (PROSITE_PATTERNS); IPR007087 (PROSITE_PROFILES); IPR007087 (PROSITE_PROFILES); IPR007087 (PROSITE_PROFILES); IPR007087 (PROSITE_PROFILES); IPR007087 (PROSITE_PROFILES); SSF57667 (SUPERFAMILY); SSF57667 (SUPERFAMILY)
809	c175454_g1_i1_len_844_path_1_0_843_0	41	adp-ribosylation factor-like protein 1	272	2.63E-90	95.60%	0.104	IPR006689 (PRINTS); IPR024156 (SMART); IPR006687 (SMART); IPR005225 (TIGRFAM); IPR006689 (PFAM); IPR027417 (G3DSA:3.40.50.GENE3D); PTHR11711:SF41 (PANTHER); PTHR11711 (PANTHER); IPR024156 (PROSITE_PROFILES); IPR027417 (SUPERFAMILY)
810	c175481_g1_i1_len_355_path_1_0_354_0	8	fat-like cadherin-related tumor suppressor-like protein	119	6.45E-50	86.40%	0.12	IPR002126 (PRINTS); IPR002126 (SMART); IPR002126 (PFAM); IPR002126 (G3DSA:2.60.40.GENE3D); PTHR24026:SF39 (PANTHER); PTHR24026 (PANTHER); IPR020894 (PROSITE_PATTERNS); IPR002126 (PROSITE_PROFILES); IPR002126 (PROSITE_PROFILES); IPR015919 (SUPERFAMILY)
811	c175494_g1_i1_len_1087_path_1_0_1086_0	34	phosphodiesterase i	363	7.65E-17	61.20%	0.171	IPR017849 (G3DSA:3.40.720.GENE3D); IPR002591 (PFAM); IPR024873 (PANTHER); TRANSMEMBRANE (PHOBIUS); TRANSMEMBRANE (PHOBIUS); TRANSMEMBRANE (PHOBIUS); TRANSMEMBRANE (PHOBIUS); NON_CYTOPLASMIC_DOMAIN (PHOBIUS); CYTOPLASMIC_DOMAIN (PHOBIUS); CYTOPLASMIC_DOMAIN (PHOBIUS); NON_CYTOPLASMIC_DOMAIN (PHOBIUS); NON_CYTOPLASMIC_DOMAIN (PHOBIUS); IPR017850 (SUPERFAMILY)
812	c175494_g1_i1_len_1087_path_1_0_1086_2	34	ectonucleotide pyrophosphatase phosphodiesterase family member 6	362	1.64E-60	57.00%	0.169	IPR017849 (G3DSA:3.40.720.GENE3D); IPR002591 (PFAM); IPR024873 (PANTHER); NON_CYTOPLASMIC_DOMAIN (PHOBIUS); TRANSMEMBRANE (PHOBIUS); CYTOPLASMIC_DOMAIN (PHOBIUS); IPR017850 (SUPERFAMILY)

813	c175585_g1_i1_len_534_path_1_0_533_3	10	phenoloxidase subunit a3	178	1.64E-52	66.50%	0.102	IPR013788 (PRINTS); IPR008922 (G3DSA:1.10.1280.GENE3D); IPR000896 (PFAM); IPR013788 (PANTHER); PTHR11511:SF24 (PANTHER); IPR013788 (PROSITE_PATTERNS); IPR008922 (SUPERFAMILY)
814	c175748_g1_i1_len_862_path_840_0_861_3	29	tripartite motif-containing protein 2-like isoform x5	288	2.35E-148	84.80%	0.114	IPR001258 (PFAM); IPR011042 (G3DSA:2.120.10.GENE3D); PTHR24103:SF1 (PANTHER); PTHR24103 (PANTHER); IPR013017 (PROSITE_PROFILES); IPR013017 (PROSITE_PROFILES); IPR013017 (PROSITE_PROFILES); IPR013017 (PROSITE_PROFILES); IPR013017 (PROSITE_PROFILES); IPR013017 (PROSITE_PROFILES); SSF101898 (SUPERFAMILY)
815	c17588_g1_i1_len_2247_path_53_0_2246_0	120	conserved hypothetical protein	749	9.96E-139	53.70%	0.493 Y	Coil (COILS); IPR002223 (PRINTS); IPR001791 (SMART); IPR002223 (SMART); IPR002223 (PFAM); IPR013320 (G3DSA:2.60.120.GENE3D); IPR001791 (PFAM); IPR002223 (G3DSA:4.10.410.GENE3D); IPR013320 (G3DSA:2.60.120.GENE3D); PTHR10574 (PANTHER); PTHR10574:SF39 (PANTHER); IPR020901 (PROSITE_PATTERNS); NON_CYTOPLASMIC_DOMAIN (PHOBIUS); TRANSMEMBRANE (PHOBIUS); CYTOPLASMIC_DOMAIN (PHOBIUS); NON_CYTOPLASMIC_DOMAIN (PHOBIUS); TRANSMEMBRANE (PHOBIUS); IPR001791 (PROSITE_PROFILES); IPR001791 (PROSITE_PROFILES); IPR002223 (PROSITE_PROFILES); SignalP-noTM (SIGNALP_EUK); IPR013320 (SUPERFAMILY); IPR002223 (SUPERFAMILY); IPR013320 (SUPERFAMILY)
816	c175929_g1_i1_len_664_path_1_0_663_4	14	protein vein	221	1.71E-57	61.40%	0.139	IPR003598 (SMART); IPR003599 (SMART); IPR000742 (SMART); IPR013783 (G3DSA:2.60.40.GENE3D); IPR000742 (PFAM); G3DSA:2.10.25.10 (GENE3D); IPR013098 (PFAM); PTHR11100 (PANTHER); IPR013032 (PROSITE_PATTERNS); IPR013032 (PROSITE_PATTERNS); IPR000742 (PROSITE_PROFILES); IPR007110 (PROSITE_PROFILES); SSF48726 (SUPERFAMILY); SSF57196 (SUPERFAMILY)
817	c175993_g1_i1_len_902_path_880_0_901_4	32	5 -nucleotidase-like	301	9.47E-86	64.40%	0.098	IPR006179 (PRINTS); IPR008334 (PFAM); IPR029052 (G3DSA:3.60.21.GENE3D); IPR008334 (G3DSA:3.90.780.GENE3D); PTHR11575:SF21 (PANTHER); IPR006179 (PANTHER); IPR008334 (SUPERFAMILY); IPR029052 (SUPERFAMILY)
818	c176038_g1_i1_len_670_path_1_0_669_2	10	zinc finger protein 121 isoform x2	223	4.42E-21	73.50%	0.2	IPR015880 (SMART); IPR013087 (G3DSA:3.30.160.GENE3D); IPR013087 (G3DSA:3.30.160.GENE3D); PF13465 (PFAM); PTHR24409 (PANTHER); IPR007087 (PROSITE_PATTERNS); IPR007087 (PROSITE_PATTERNS); IPR007087 (PROSITE_PROFILES); IPR007087 (PROSITE_PROFILES); SSF57667 (SUPERFAMILY)
819	c176069_g1_i1_len_1324_path_1_0_1323_0	71	trans- -dihydrobenzene- -diol dehydrogenase-like	442	4.87E-139	70.80%	0.206	IPR016040 (G3DSA:3.40.50.GENE3D); IPR000683 (PFAM); G3DSA:3.30.360.10 (GENE3D); IPR004104 (PFAM); PTHR22604:SF96 (PANTHER); PTHR22604 (PANTHER); NON_CYTOPLASMIC_DOMAIN (PHOBIUS); TRANSMEMBRANE (PHOBIUS); CYTOPLASMIC_DOMAIN (PHOBIUS); SSF55347 (SUPERFAMILY); SSF51735 (SUPERFAMILY)
820	c176089_g1_i1_len_485_path_463_0_484_1	25	zinc finger protein 64	162	2.65E-59	77.80%	0.102	IPR015880 (SMART); IPR013087 (G3DSA:3.30.160.GENE3D); PF13465 (PFAM); IPR013087 (G3DSA:3.30.160.GENE3D); IPR013087 (G3DSA:3.30.160.GENE3D); PTHR24409 (PANTHER); IPR007087 (PROSITE_PATTERNS); IPR007087 (PROSITE_PROFILES); IPR007087 (PROSITE_PROFILES); IPR007087 (PROSITE_PROFILES); IPR007087 (PROSITE_PROFILES); SSF57667 (SUPERFAMILY); SSF57667 (SUPERFAMILY)
821	c176113_g1_i1_len_322_path_300_0_321_0	5	low-density lipoprotein receptor-related protein 1b-like	108	1.40E-06	67.10%	0.105	IPR002172 (SMART); IPR002172 (G3DSA:4.10.400.GENE3D); IPR002172 (PFAM); IPR023415 (PROSITE_PATTERNS); IPR002172 (PROSITE_PROFILES); IPR002172 (SUPERFAMILY)
822	c176610_g1_i1_len_416_path_394_0_415_4	12	dappu_318553-like protein	139	8.22E-32	69.30%	0.145	IPR019791 (PRINTS); IPR019791 (G3DSA:1.10.640.GENE3D); IPR019791 (PFAM); PTHR11475 (PANTHER); IPR019791 (PROSITE_PROFILES); IPR010255 (SUPERFAMILY)
823	c176746_g1_i1_len_549_path_527_0_548_4	11	---NA---	183			0.136	G3DSA:2.40.10.10 (GENE3D); IPR001254 (PFAM); PTHR24264 (PANTHER); PTHR24264:SF9 (PANTHER); IPR009003 (SUPERFAMILY)
824	c176794_g1_i1_len_430_path_1_0_15_17_16_222_17_223_429_1	4	peptidyl-alpha-hydroxyglycine alpha-amidating lyase 1	143	3.36E-14	57.30%	0.129	IPR011042 (G3DSA:2.120.10.GENE3D); IPR001258 (PFAM); PTHR10680 (PANTHER); SSF63825 (SUPERFAMILY)
825	c177022_g1_i1_len_182_path_160_0_181_1	3	protein tanc2	61	3.92E-09	62.90%	0.098	IPR002110 (SMART); IPR020683 (PFAM); IPR020683 (G3DSA:1.25.40.GENE3D); PTHR24181 (PANTHER); IPR020683 (PROSITE_PROFILES); IPR002110 (PROSITE_PROFILES); IPR020683 (SUPERFAMILY)
826	c177338_g1_i1_len_413_path_391_0_412_2	19	calbindin isoform a	137	4.69E-87	93.20%	0.107	IPR002048 (SMART); IPR011992 (PFAM); IPR011992 (G3DSA:1.10.238.GENE3D); IPR011992 (G3DSA:1.10.238.GENE3D); PTHR19972 (PANTHER); IPR018247 (PROSITE_PATTERNS); IPR018247 (PROSITE_PATTERNS); IPR002048 (PROSITE_PROFILES); IPR002048 (PROSITE_PROFILES); IPR002048 (PROSITE_PROFILES); SSF47473 (SUPERFAMILY)

827	c177440_g1_i1_len_1489_path_1467_0_1488_2	81	aael005189- partial	496	7.00E-118	69.90%	0.104	IPR003112 (SMART); IPR003112 (PFAM); PTHR23192 (PANTHER); PTHR23192:SF33 (PANTHER); IPR003112 (PROSITE_PROFILES)
828	c177455_g1_i1_len_258_path_236_0_257_0	4	fibroblast growth factor receptor-like protein 1	86	1.72E-42	89.40%	0.107	IPR020635 (SMART); IPR001245 (PFAM); G3DSA:1.10.510.10 (GENE3D); PTHR24416:SF291 (PANTHER); PTHR24416 (PANTHER); IPR008266 (PROSITE_PATTERNS); IPR000719 (PROSITE_PROFILES); IPR011009 (SUPERFAMILY)
829	c17751_g1_i1_len_498_path_143_0_140_284_141_497_4	14	lachesin isoform x2	166	7.57E-66	75.70%	0.121	IPR003598 (SMART); IPR003599 (SMART); IPR013106 (PFAM); IPR013098 (PFAM); IPR013783 (G3DSA:2.60.40.GENE3D); PTHR19831:SF43 (PANTHER); PTHR19831 (PANTHER); IPR007110 (PROSITE_PROFILES); SSF48726 (SUPERFAMILY); SSF48726 (SUPERFAMILY)
830	c177703_g1_i1_len_596_path_336_0_73_410_74_334_410_335_595_3	8	zinc finger protein 271 (zinc finger protein 7) (zinc finger protein znfphex133) (epstein-barr virus-induced zinc finger protein) (znf-eb) (ct-zfp48) (zinc finger protein	199	8.14E-38	57.40%	0.1	IPR015880 (SMART); IPR013087 (G3DSA:3.30.160.GENE3D); PF13465 (PFAM); IPR013087 (G3DSA:3.30.160.GENE3D); IPR013087 (G3DSA:3.30.160.GENE3D); PTHR24409:SF14 (PANTHER); PTHR24409 (PANTHER); IPR007087 (PROSITE_PATTERNS); IPR007087 (PROSITE_PATTERNS); IPR007087 (PROSITE_PATTERNS); IPR007087 (PROSITE_PATTERNS); IPR007087 (PROSITE_PATTERNS); IPR007087 (PROSITE_PROFILES); IPR007087 (PROSITE_PROFILES); IPR007087 (PROSITE_PROFILES); IPR007087 (PROSITE_PROFILES); IPR007087 (PROSITE_PROFILES); IPR007087 (PROSITE_PROFILES); SSF57667 (SUPERFAMILY); SSF57667 (SUPERFAMILY); SSF57667 (SUPERFAMILY); SSF57667 (SUPERFAMILY)
831	c177712_g1_i1_len_350_path_1_0_349_4	8	st14a protein	117	2.12E-10	54.60%	0.163	IPR001254 (PFAM); G3DSA:2.40.10.10 (GENE3D); IPR009003 (SUPERFAMILY)
832	c177815_g1_i1_len_490_path_468_0_489_1	6	cadherin- partial	163	3.79E-55	69.50%	0.132	IPR002126 (PRINTS); IPR002126 (SMART); IPR002126 (G3DSA:2.60.40.GENE3D); IPR002126 (PFAM); IPR002126 (G3DSA:2.60.40.GENE3D); PTHR24027 (PANTHER); PTHR24027:SF45 (PANTHER); IPR002126 (PROSITE_PROFILES); IPR002126 (PROSITE_PROFILES); IPR015919 (SUPERFAMILY); IPR015919 (SUPERFAMILY)
833	c178059_g1_i1_len_1423_path_1_0_1422_2	116	alpha-2-macroglobulin-like protein 1	474	2.33E-131	59.10%	0.164	IPR001599 (PFAM); IPR011625 (PFAM); IPR019565 (PFAM); IPR008930 (G3DSA:1.50.10.GENE3D); PTHR11412 (PANTHER); IPR019742 (PROSITE_PATTERNS); IPR014756 (SUPERFAMILY); IPR008930 (SUPERFAMILY)
834	c178076_g1_i1_len_313_path_291_0_312_2	4	poc1 centriolar protein homolog partial	104	1.76E-45	81.00%	0.096	IPR001680 (SMART); IPR001680 (PFAM); IPR015943 (G3DSA:2.130.10.GENE3D); PTHR22847 (PANTHER); PTHR22847:SF319 (PANTHER); IPR017986 (PROSITE_PROFILES); IPR001680 (PROSITE_PROFILES); IPR017986 (SUPERFAMILY)
835	c178154_g1_i1_len_532_path_510_0_531_3	18	beta-glucuronidase- partial	178	1.02E-74	75.60%	0.127	IPR006101 (PRINTS); IPR013781 (G3DSA:3.20.20.GENE3D); IPR006103 (PFAM); PTHR10066:SF51 (PANTHER); PTHR10066 (PANTHER); IPR023232 (PROSITE_PATTERNS); IPR023230 (PROSITE_PATTERNS); IPR017853 (SUPERFAMILY)
836	c178180_g1_i1_len_344_path_322_0_343_0	5	serine protease partial	115	2.00E-06	74.00%	0.196	G3DSA:2.40.10.10 (GENE3D); IPR001254 (PFAM); PTHR24256 (PANTHER); IPR009003 (SUPERFAMILY)
837	c178339_g1_i1_len_405_path_383_0_404_3	10	low quality protein: sco-spondin	135	6.09E-12	54.90%	0.208	IPR002172 (PRINTS); IPR002172 (SMART); IPR002172 (G3DSA:4.10.400.GENE3D); IPR002172 (G3DSA:4.10.400.GENE3D); IPR002172 (PFAM); PTHR10529:SF204 (PANTHER); PTHR10529 (PANTHER); CYTOPLASMIC_DOMAIN (PHOBIUS); NON_CYTOPLASMIC_DOMAIN (PHOBIUS); TRANSMEMBRANE (PHOBIUS); IPR002172 (PROSITE_PROFILES); IPR002172 (PROSITE_PROFILES); IPR002172 (SUPERFAMILY); IPR002172 (SUPERFAMILY)
838	c178424_g1_i1_len_721_path_1_0_720_1	37	protein phosphatase 1 regulatory subunit 27	240	2.58E-61	70.60%	0.191	IPR002110 (SMART); IPR020683 (G3DSA:1.25.40.GENE3D); IPR020683 (PFAM); PTHR24179 (PANTHER); PTHR24179:SF23 (PANTHER); IPR002110 (PROSITE_PROFILES); IPR020683 (PROSITE_PROFILES); IPR002110 (PROSITE_PROFILES); IPR020683 (SUPERFAMILY)
839	c17854_g1_i1_len_163_path_1_0_162_3	1	PREDICTED: uncharacterized protein CG3556	55	1.82E-17	77.40%	0.314	no IPS match
840	c178604_g1_i1_len_527_path_1_0_526_2	10	hypothetical protein DAPPUDRAFT_192774	175	1.79E-49	63.50%	0.191	IPR001424 (G3DSA:2.60.40.GENE3D); IPR001424 (PFAM); PTHR20910 (PANTHER); IPR001424 (SUPERFAMILY)
841	c178966_g1_i1_len_257_path_1_0_256_2	4	cubilin- partial	85	4.58E-16	65.80%	0.121	IPR000859 (SMART); IPR000859 (G3DSA:2.60.120.GENE3D); IPR000859 (PFAM); PTHR10127 (PANTHER); IPR000859 (PROSITE_PROFILES); IPR000859 (SUPERFAMILY)
842	c179017_g1_i1_len_263_path_1_0_262_0	2	protein kinase c-binding protein nell1-like	88	2.09E-17	61.00%	0.115	IPR001007 (PFAM); G3DSA:2.10.70.10 (GENE3D); PTHR24042:SF4 (PANTHER); PTHR24042 (PANTHER); IPR001007 (PROSITE_PROFILES); SSF57603 (SUPERFAMILY)

843	c179089_g1_i1_len_464_path_471_0_463_5	28	interstitial collagenase	154	2.17E-54	70.00%	0.134	IPR021190 (PRINTS); IPR018487 (SMART); IPR024079 (G3DSA:3.40.390.GENE3D); IPR018487 (PFAM); IPR000585 (G3DSA:2.110.10.GENE3D); IPR001818 (PFAM); PTHR10201 (PANTHER); IPR018486 (PROSITE_PATTERNS); IPR018487 (PROSITE_PROFILES); SSF55486 (SUPERFAMILY); IPR000585 (SUPERFAMILY)
844	c179206_g1_i1_len_635_path_1_0_634_1	10	upf0672 protein c3orf58 homolog	212	3.81E-30	55.90%	0.106	IPR022049 (PFAM); G3DSA:1.10.510.10 (GENE3D); PTHR32073 (PANTHER); PTHR32073:SF7 (PANTHER); IPR011009 (SUPERFAMILY)
845	c179369_g1_i1_len_392_path_1_0_391_5	6	glypican- partial	130	4.42E-66	81.80%	0.101	IPR001863 (PFAM); IPR001863 (PANTHER); IPR019803 (PROSITE_PATTERNS)
846	c179430_g1_i1_len_286_path_1_0_285_5	8	allergen 5	95	2.91E-12	59.80%	0.099	IPR002413 (PRINTS); IPR014044 (G3DSA:3.40.33.GENE3D); IPR014044 (SUPERFAMILY)
847	c179803_g1_i1_len_398_path_20_0_397_5	8	low-density lipoprotein receptor-related protein partial	132	1.74E-08	46.60%	0.102	IPR002172 (SMART); IPR002172 (PFAM); IPR002172 (G3DSA:4.10.400.GENE3D); PTHR10529 (PANTHER); IPR023415 (PROSITE_PATTERNS); IPR002172 (PROSITE_PROFILES); IPR002172 (SUPERFAMILY)
848	c179814_g1_i1_len_603_path_1_0_602_4	8	tyrosine-protein kinase transmembrane receptor ror	201	7.94E-122	90.50%	0.107	IPR001245 (PRINTS); IPR020635 (SMART); G3DSA:3.30.200.20 (GENE3D); IPR001245 (PFAM); G3DSA:1.10.510.10 (GENE3D); PTHR24416:SF309 (PANTHER); PTHR24416 (PANTHER); IPR008266 (PROSITE_PATTERNS); IPR002011 (PROSITE_PATTERNS); IPR000719 (PROSITE_PROFILES); IPR011009 (SUPERFAMILY)
849	c180065_g1_i1_len_550_path_528_0_549_5	15	polypeptide n-acetylgalactosaminyltransferase 2	183	1.21E-21	52.30%	0.102	IPR000772 (SMART); G3DSA:2.80.10.50 (GENE3D); IPR000772 (PFAM); PTHR11675:SF24 (PANTHER); PTHR11675 (PANTHER); IPR000772 (PROSITE_PROFILES); IPR000772 (SUPERFAMILY)
850	c18027_g2_i1_len_145_path_203_0_144_0	4	notch protein	49	1.97E-16	87.00%	0.11	IPR001881 (SMART); IPR000742 (SMART); G3DSA:2.10.25.10 (GENE3D); IPR000742 (PFAM); PTHR24033:SF36 (PANTHER); PTHR24033 (PANTHER); IPR013032 (PROSITE_PATTERNS); IPR000152 (PROSITE_PATTERNS); IPR018097 (PROSITE_PATTERNS); IPR000742 (PROSITE_PROFILES); SSF57196 (SUPERFAMILY)
851	c180292_g1_i1_len_256_path_1_0_255_1	6	achain 3ank: a designed ankyrin repeat protein with three identical consensus repeats	85	7.92E-13	65.60%	0.112	IPR002110 (SMART); IPR020683 (PFAM); IPR020683 (G3DSA:1.25.40.GENE3D); PTHR24193 (PANTHER); IPR020683 (PROSITE_PROFILES); IPR002110 (PROSITE_PROFILES); IPR002110 (PROSITE_PROFILES); IPR020683 (SUPERFAMILY)
[truncated: 2,004,258 more chars]
